# Supplementary figures and images for: Classification of current density vector map using transformer hybrid residual network (part 2 of 6)
Source: PLoS One. 2025 Dec 16;20(12):e0338189. doi: 10.1371/journal.pone.0338189 (PMC12707687; doi:10.1371/journal.pone.0338189)

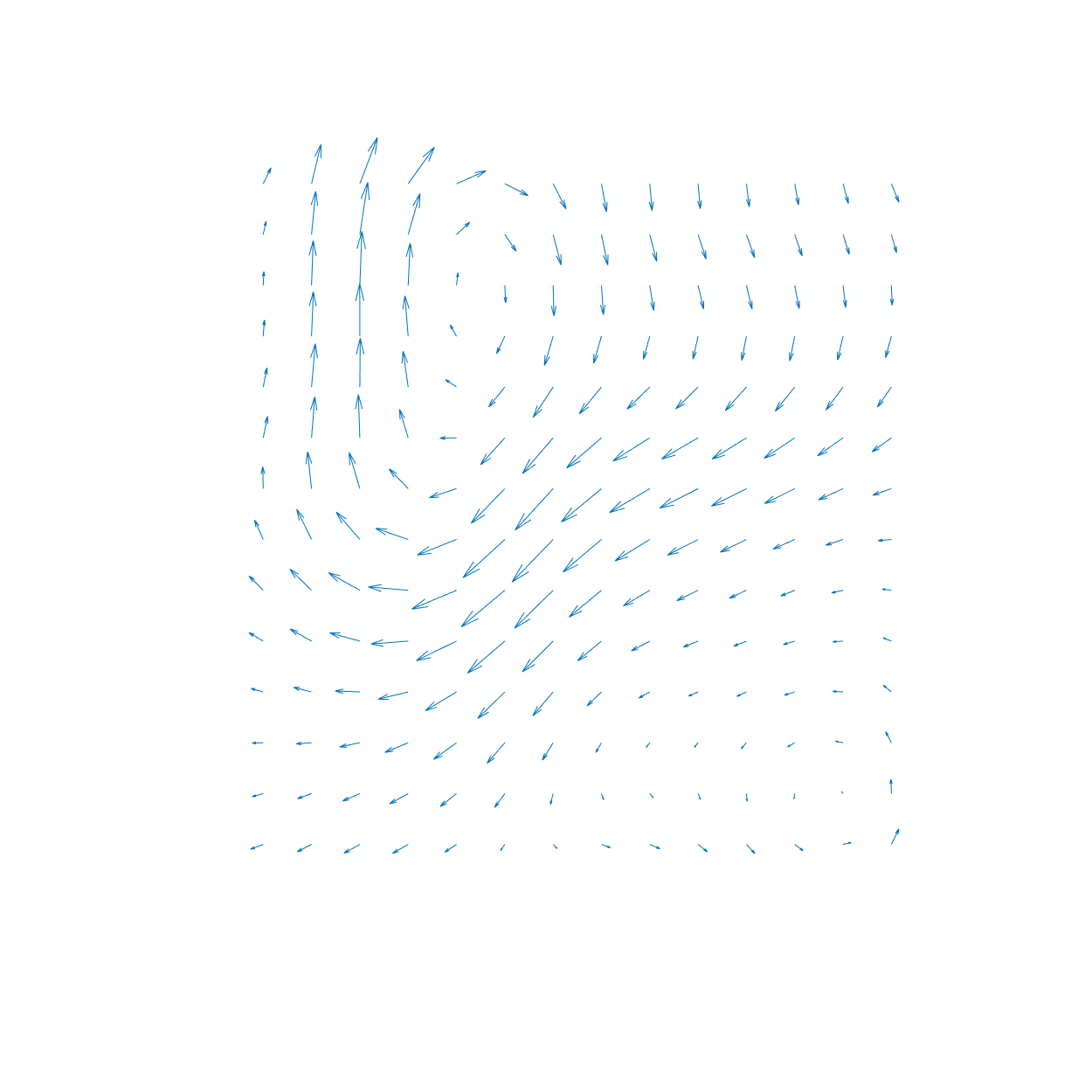

Supplement: S1 MCG raw data 1 — The raw MCG dataset includes categories 0-4 for testing. (ZIP) [file pone.0338189.s001.zip › test/1/p2_500_4.png]

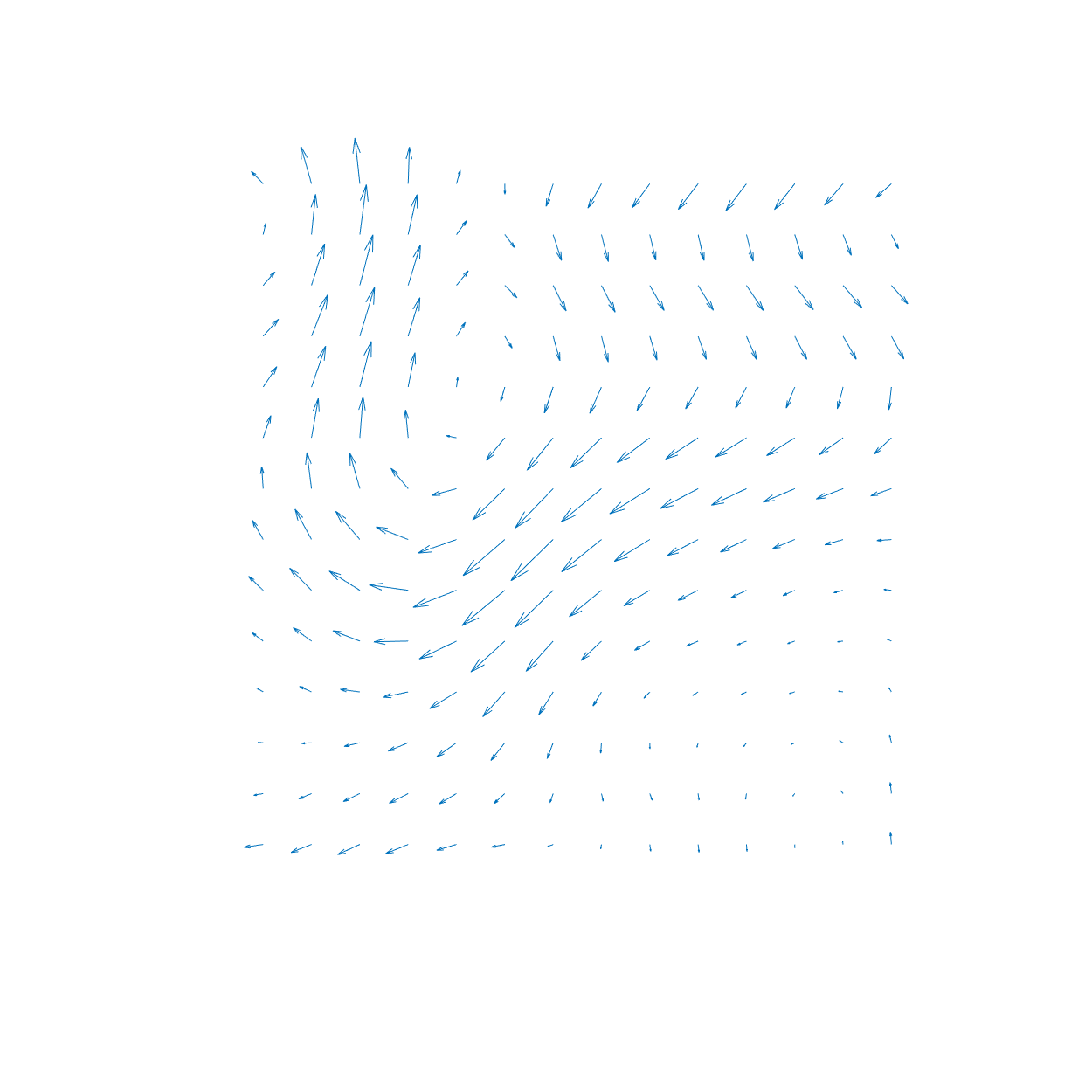

Supplement: S1 MCG raw data 1 — The raw MCG dataset includes categories 0-4 for testing. (ZIP) [file pone.0338189.s001.zip › test/1/p2_505_4.png]

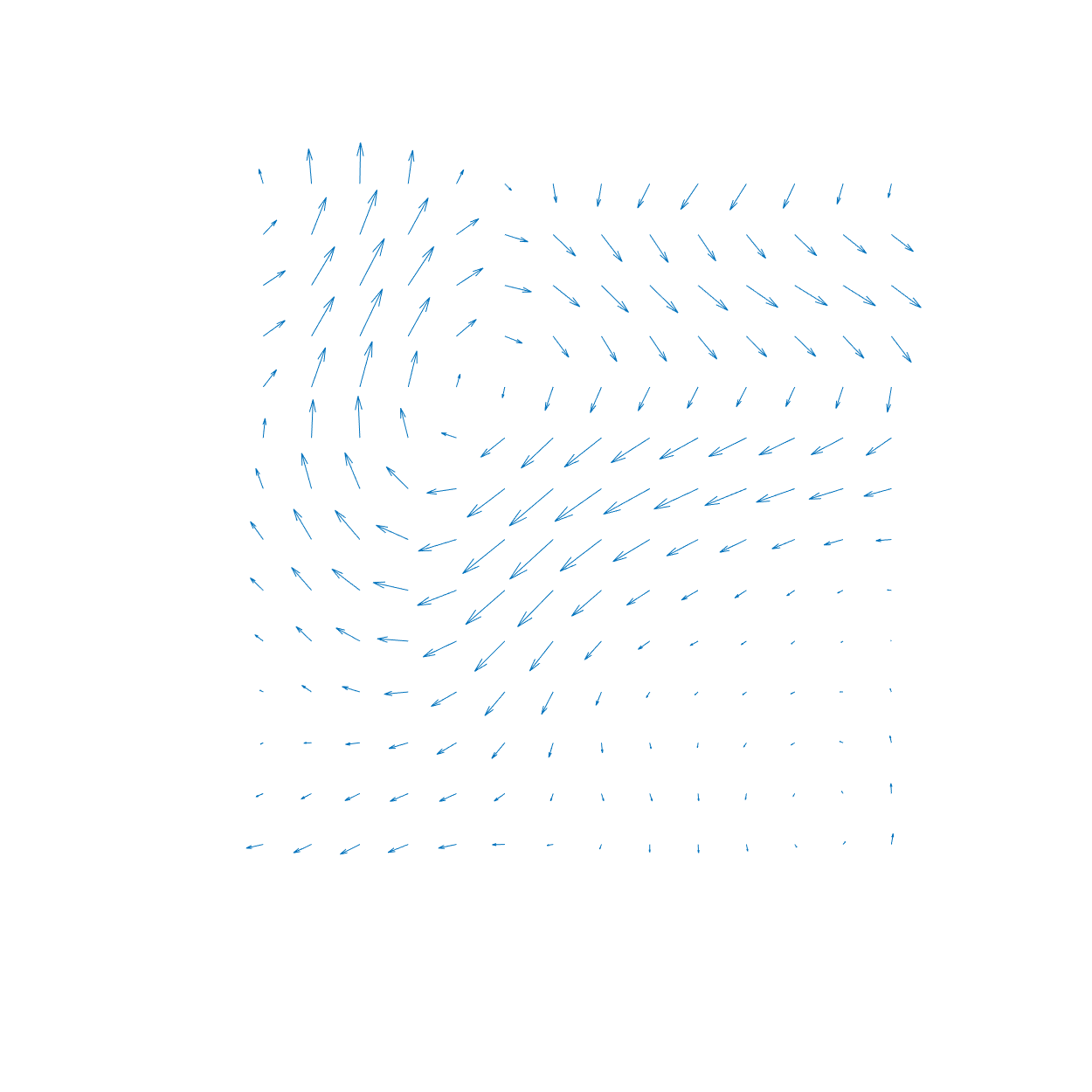

Supplement: S1 MCG raw data 1 — The raw MCG dataset includes categories 0-4 for testing. (ZIP) [file pone.0338189.s001.zip › test/1/p2_510_4.png]

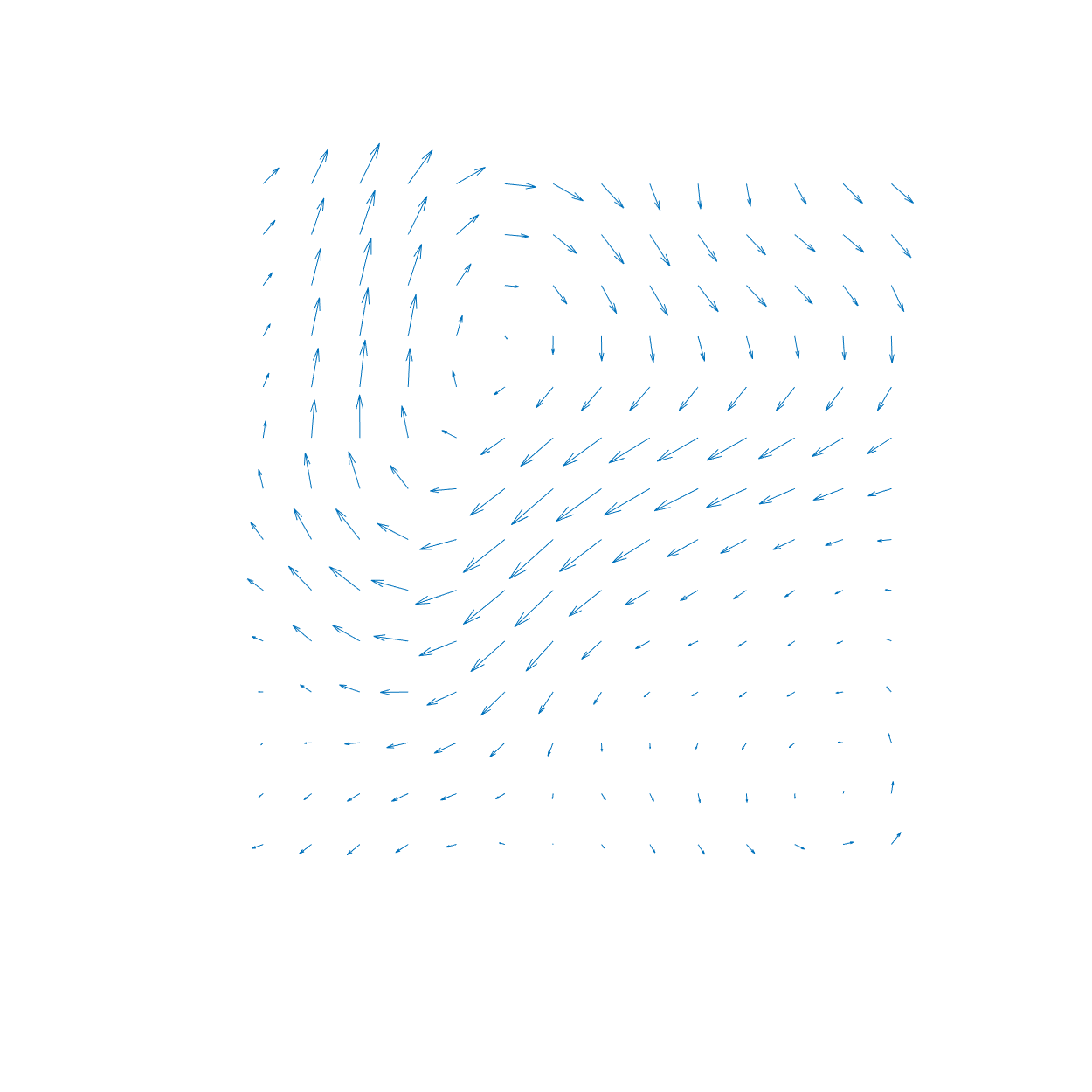

Supplement: S1 MCG raw data 1 — The raw MCG dataset includes categories 0-4 for testing. (ZIP) [file pone.0338189.s001.zip › test/1/p2_515_4.png]

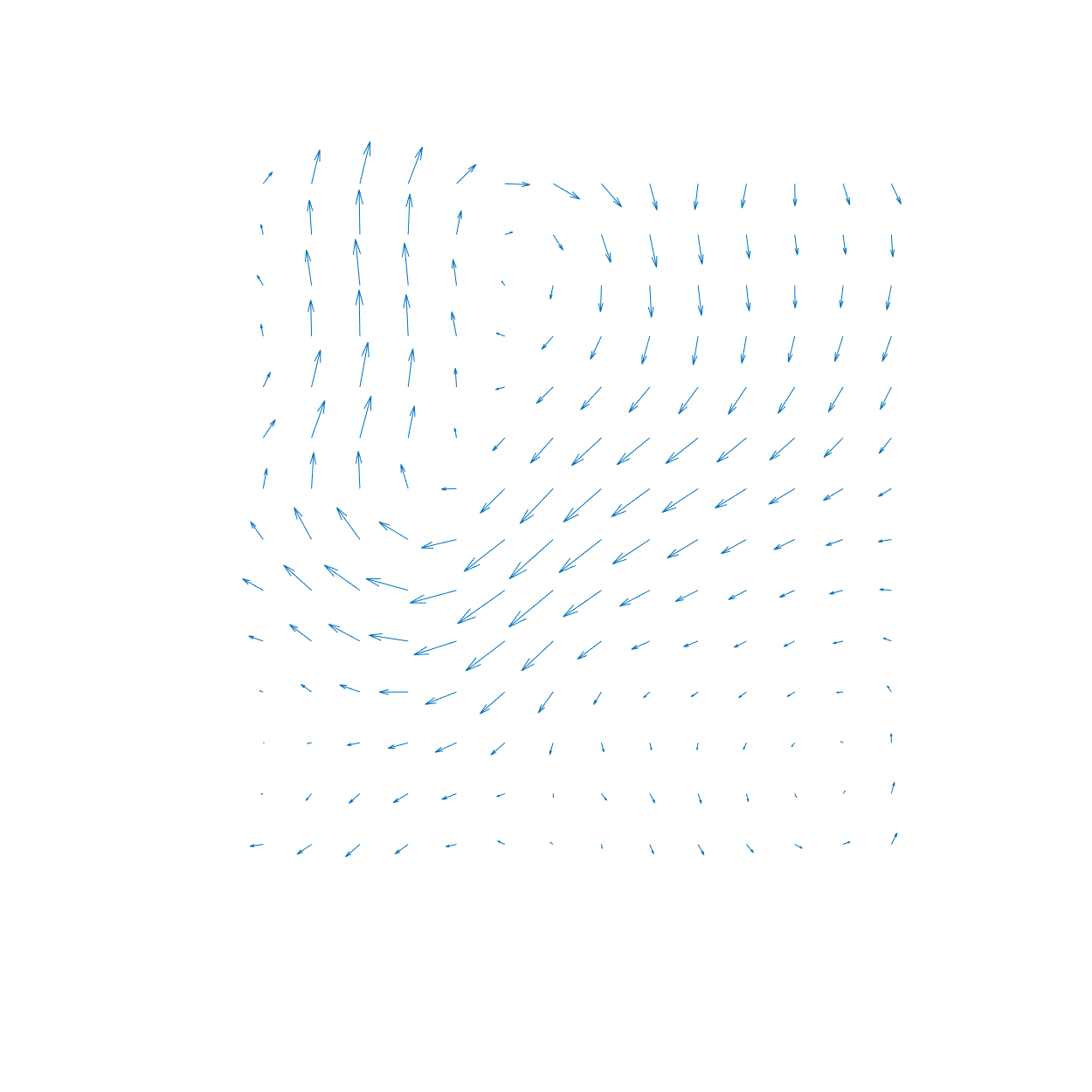

Supplement: S1 MCG raw data 1 — The raw MCG dataset includes categories 0-4 for testing. (ZIP) [file pone.0338189.s001.zip › test/1/p2_520_4.png]

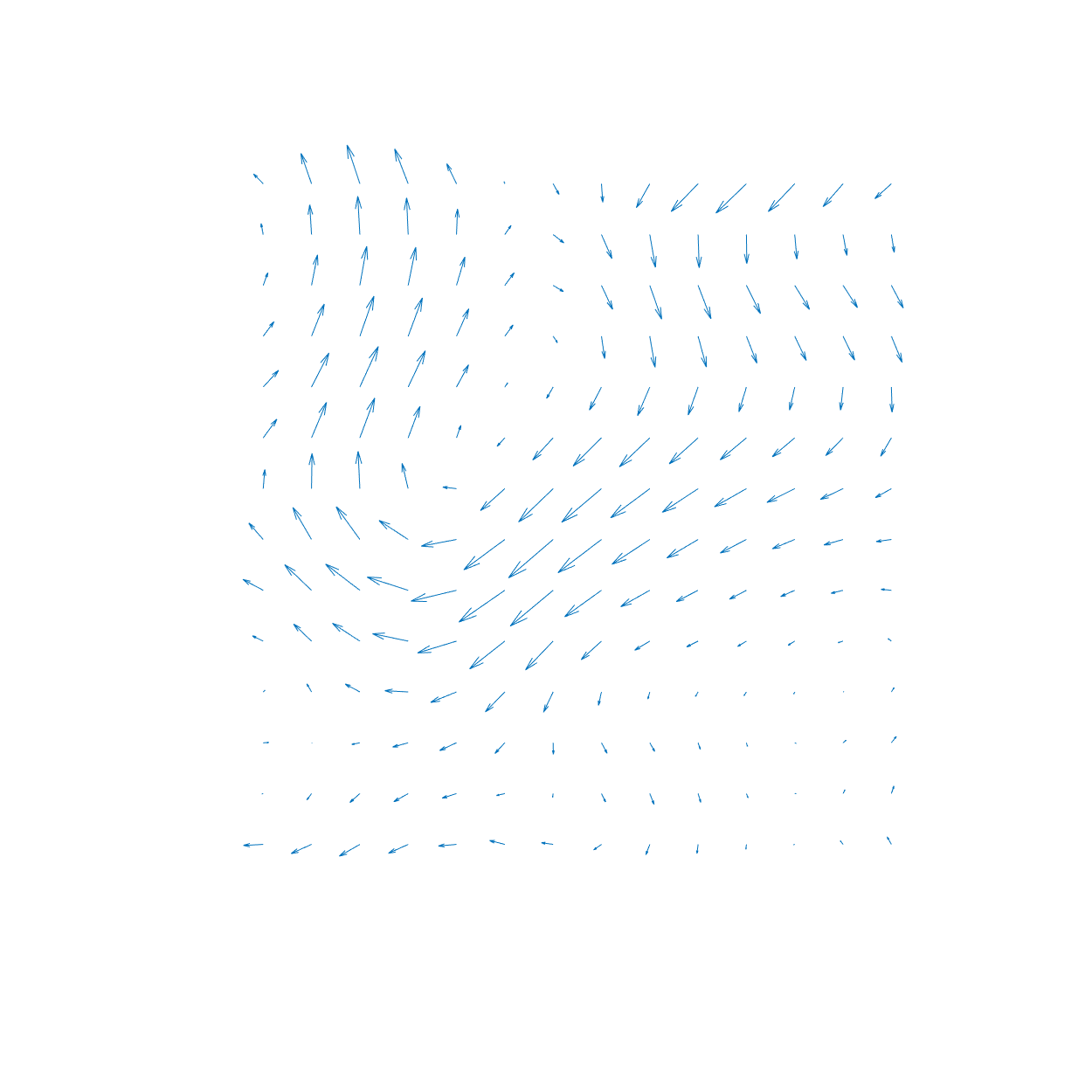

Supplement: S1 MCG raw data 1 — The raw MCG dataset includes categories 0-4 for testing. (ZIP) [file pone.0338189.s001.zip › test/1/p2_525_4.png]

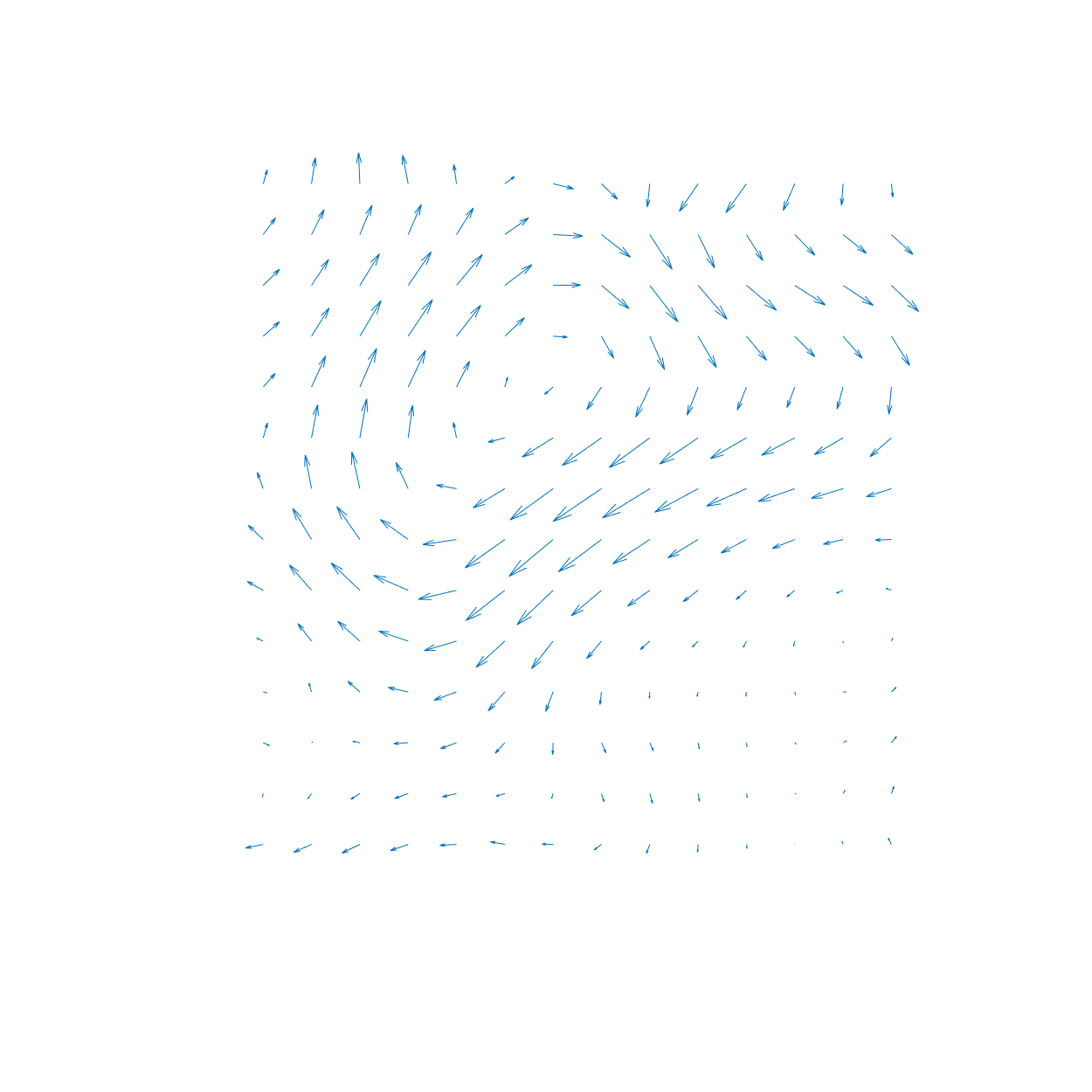

Supplement: S1 MCG raw data 1 — The raw MCG dataset includes categories 0-4 for testing. (ZIP) [file pone.0338189.s001.zip › test/1/p2_530_4.png]

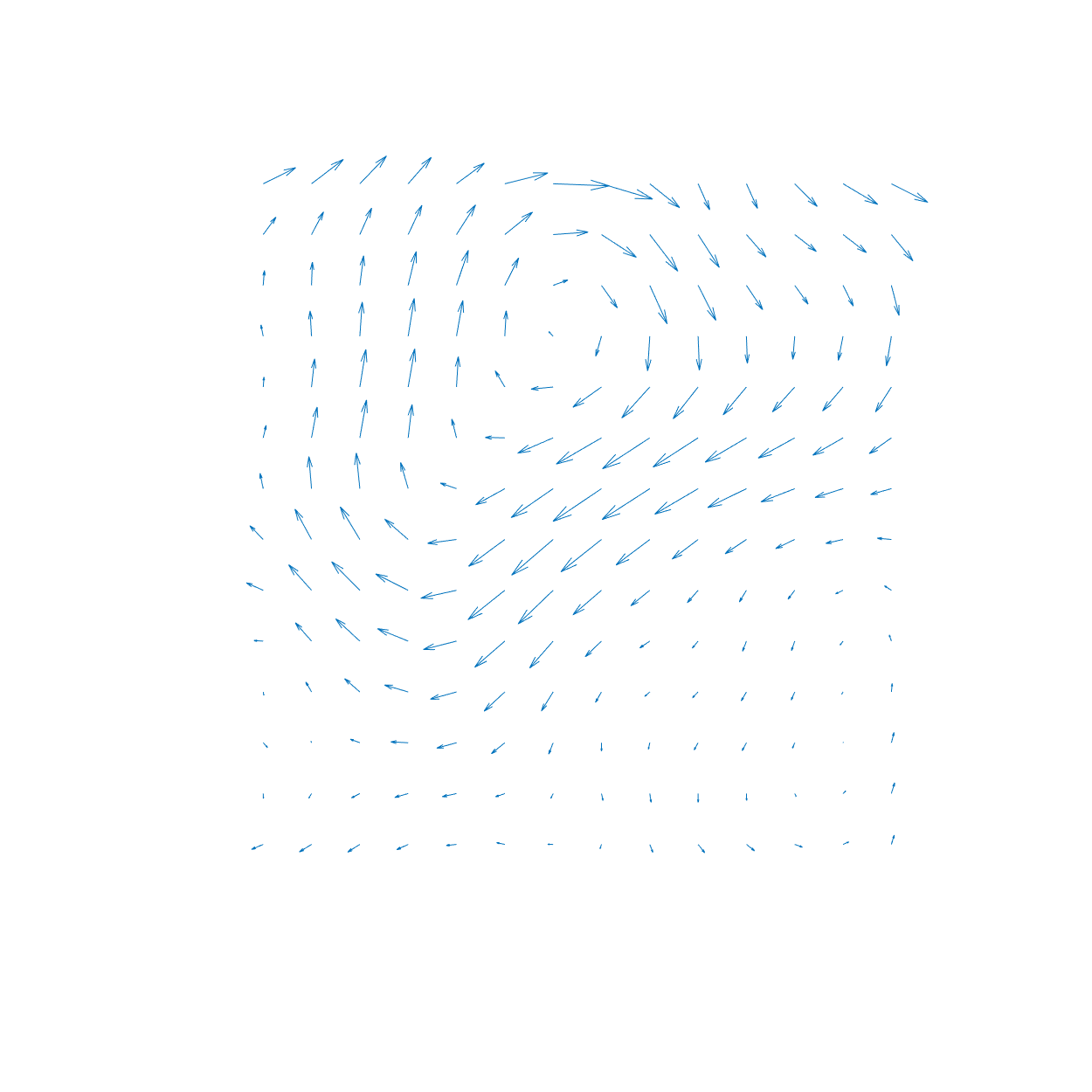

Supplement: S1 MCG raw data 1 — The raw MCG dataset includes categories 0-4 for testing. (ZIP) [file pone.0338189.s001.zip › test/1/p2_535_4.png]

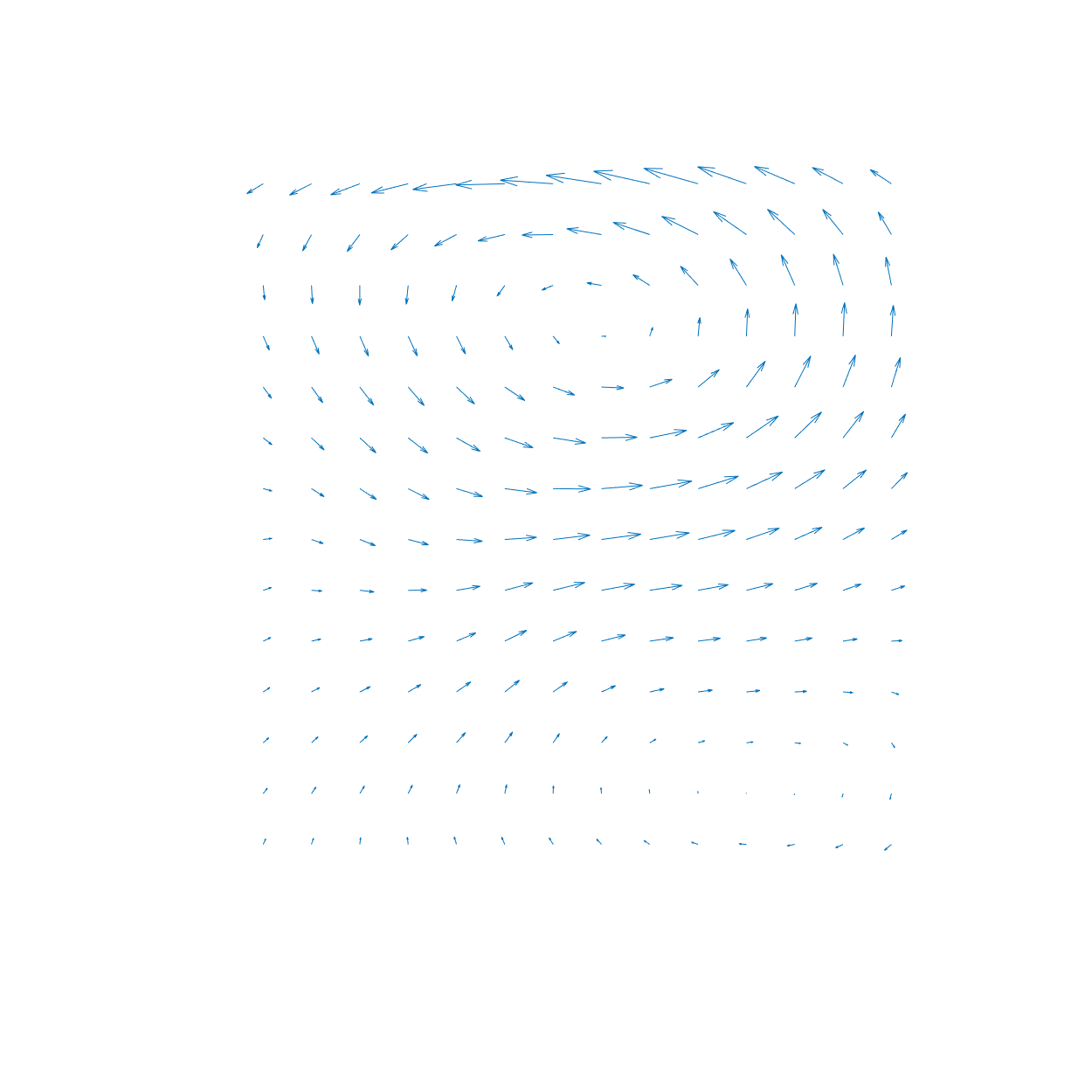

Supplement: S1 MCG raw data 1 — The raw MCG dataset includes categories 0-4 for testing. (ZIP) [file pone.0338189.s001.zip › test/1/p3_205_4.png]

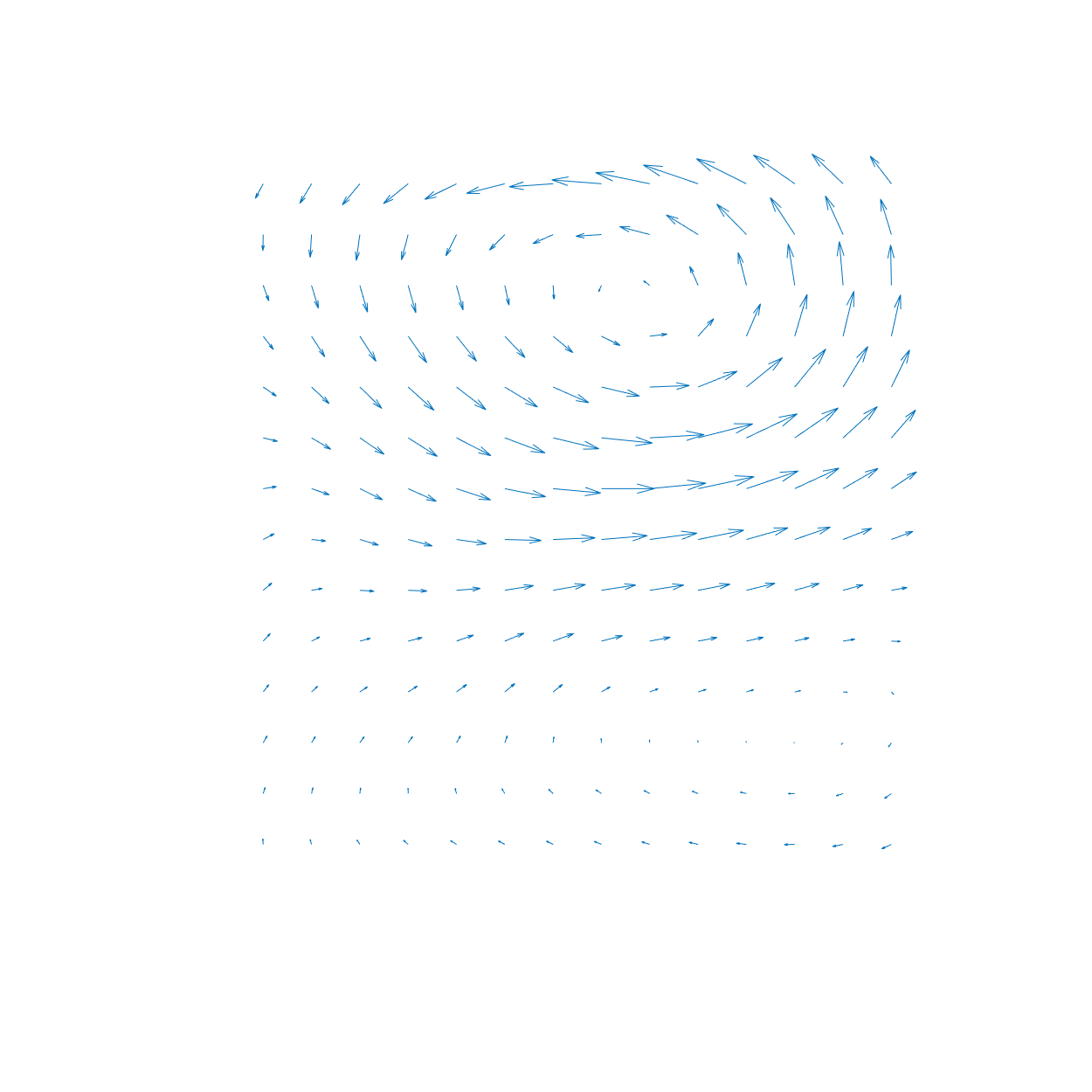

Supplement: S1 MCG raw data 1 — The raw MCG dataset includes categories 0-4 for testing. (ZIP) [file pone.0338189.s001.zip › test/1/p3_210_4.png]

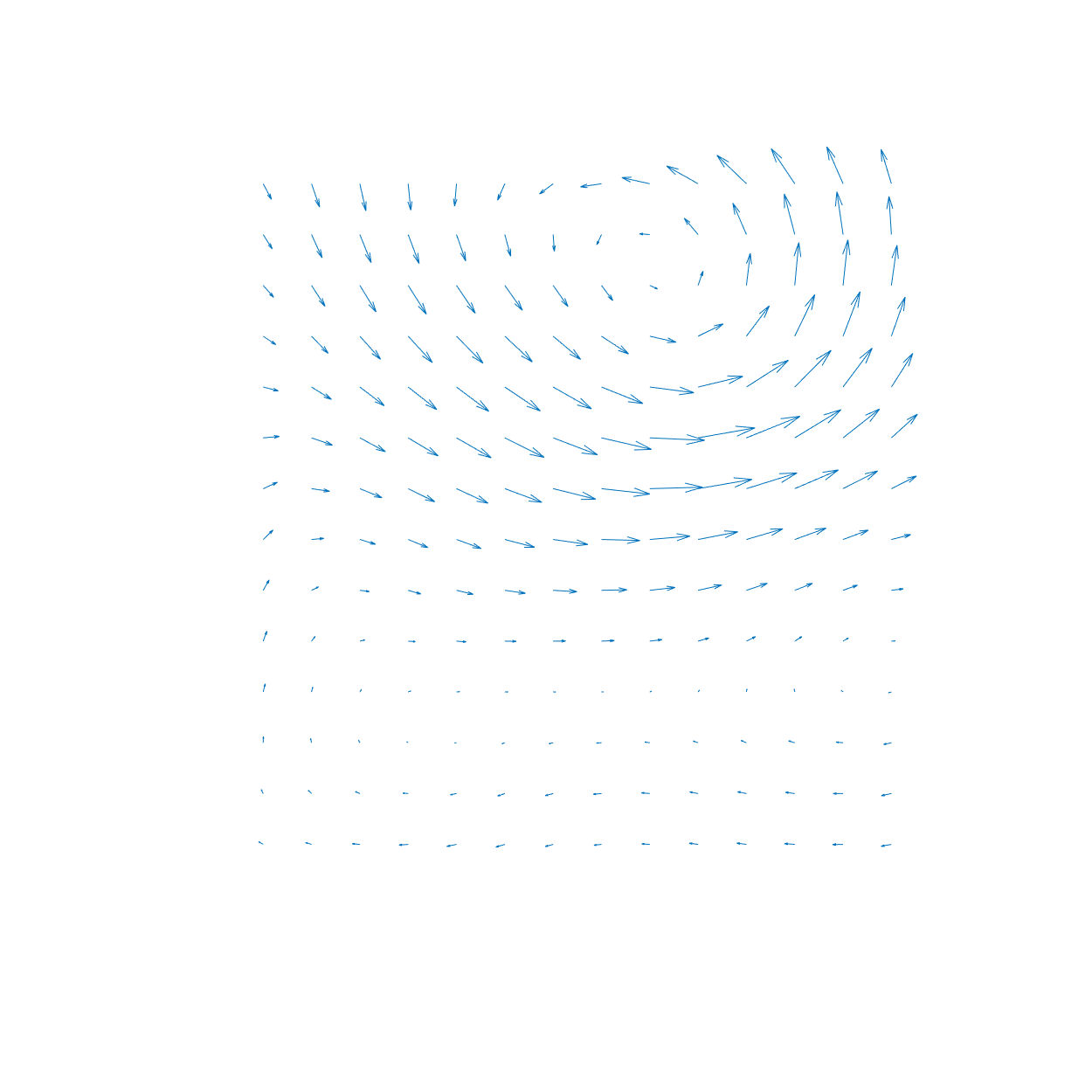

Supplement: S1 MCG raw data 1 — The raw MCG dataset includes categories 0-4 for testing. (ZIP) [file pone.0338189.s001.zip › test/1/p3_215_4.png]

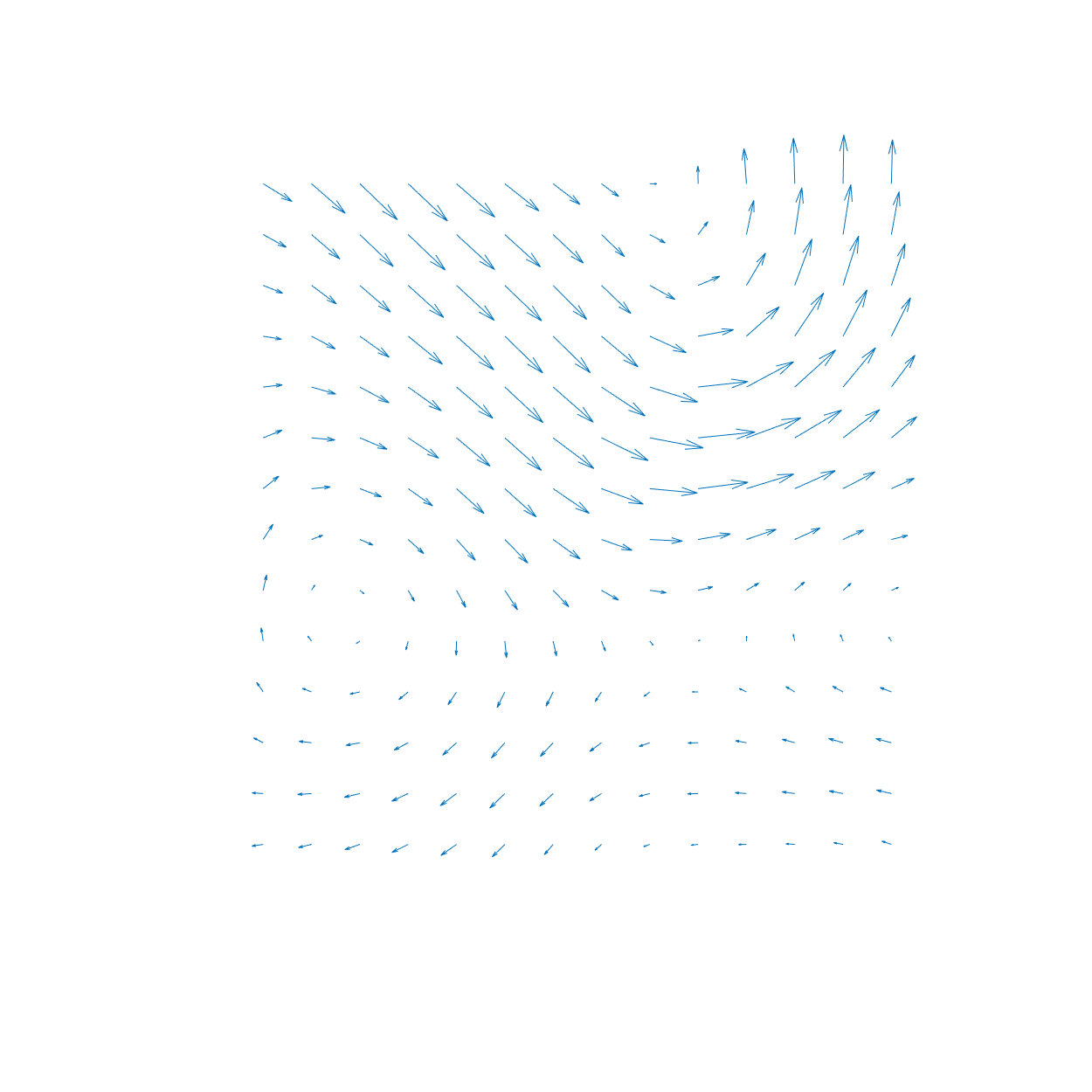

Supplement: S1 MCG raw data 1 — The raw MCG dataset includes categories 0-4 for testing. (ZIP) [file pone.0338189.s001.zip › test/1/p3_220_4.png]

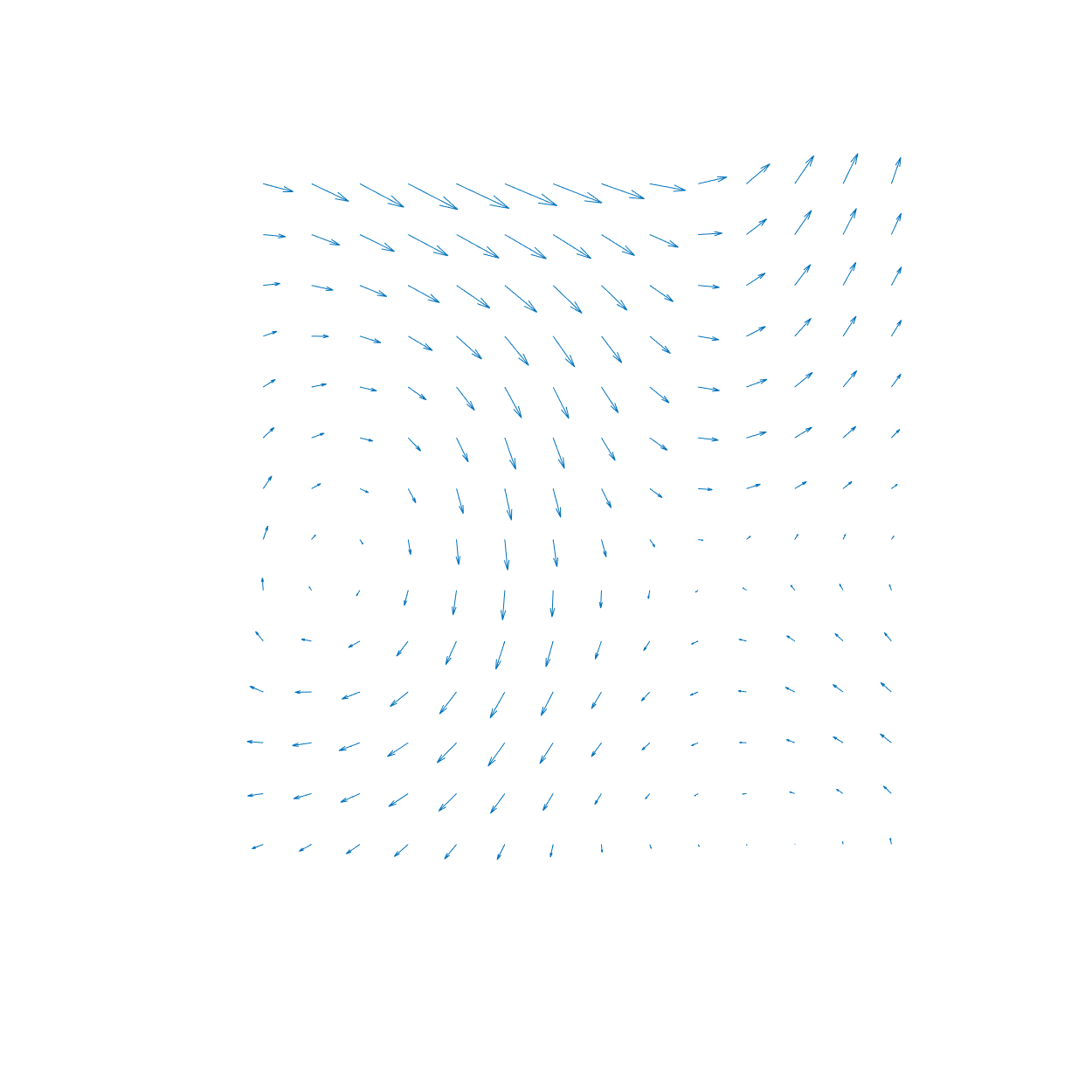

Supplement: S1 MCG raw data 1 — The raw MCG dataset includes categories 0-4 for testing. (ZIP) [file pone.0338189.s001.zip › test/1/p3_225_4.png]

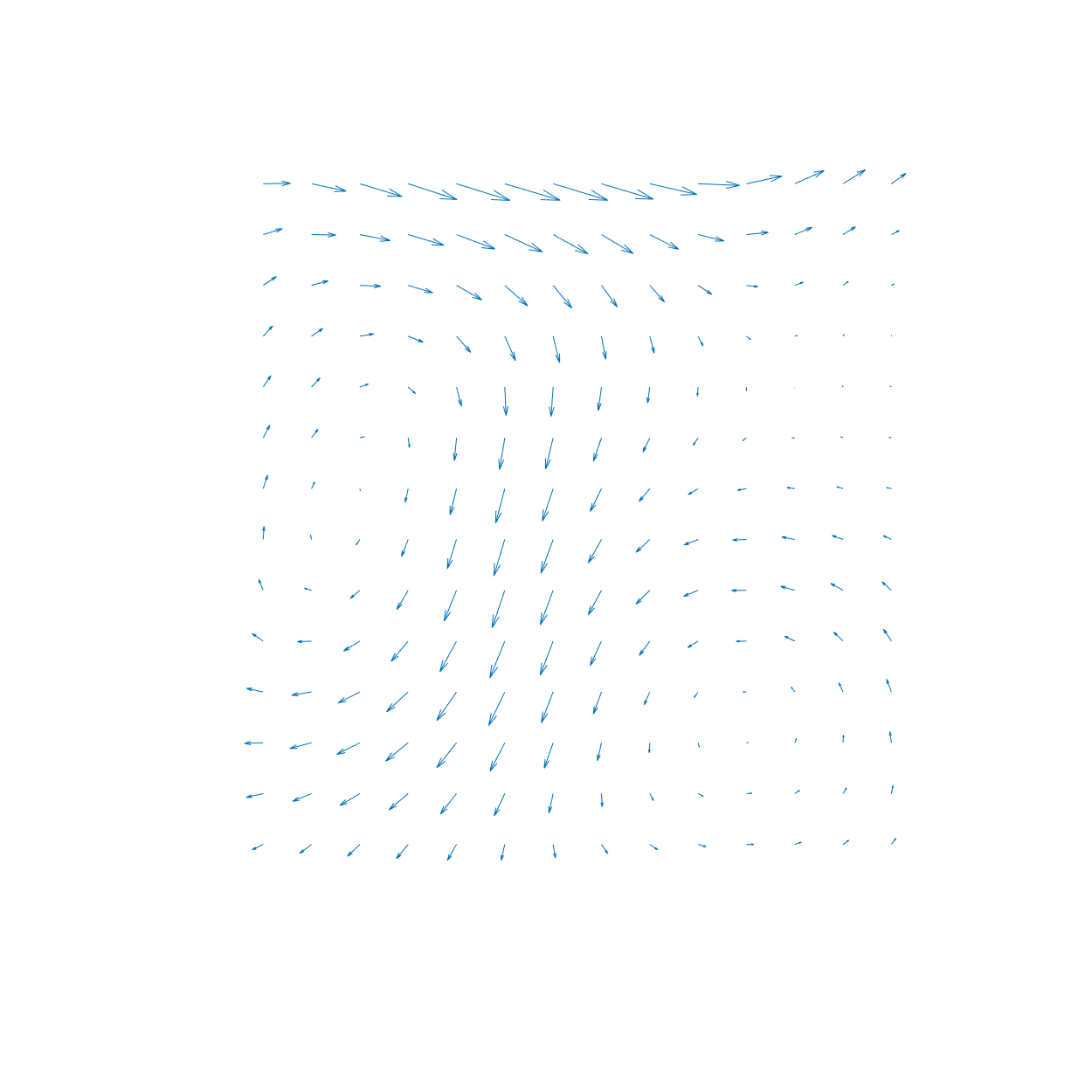

Supplement: S1 MCG raw data 1 — The raw MCG dataset includes categories 0-4 for testing. (ZIP) [file pone.0338189.s001.zip › test/1/p3_230_4.png]

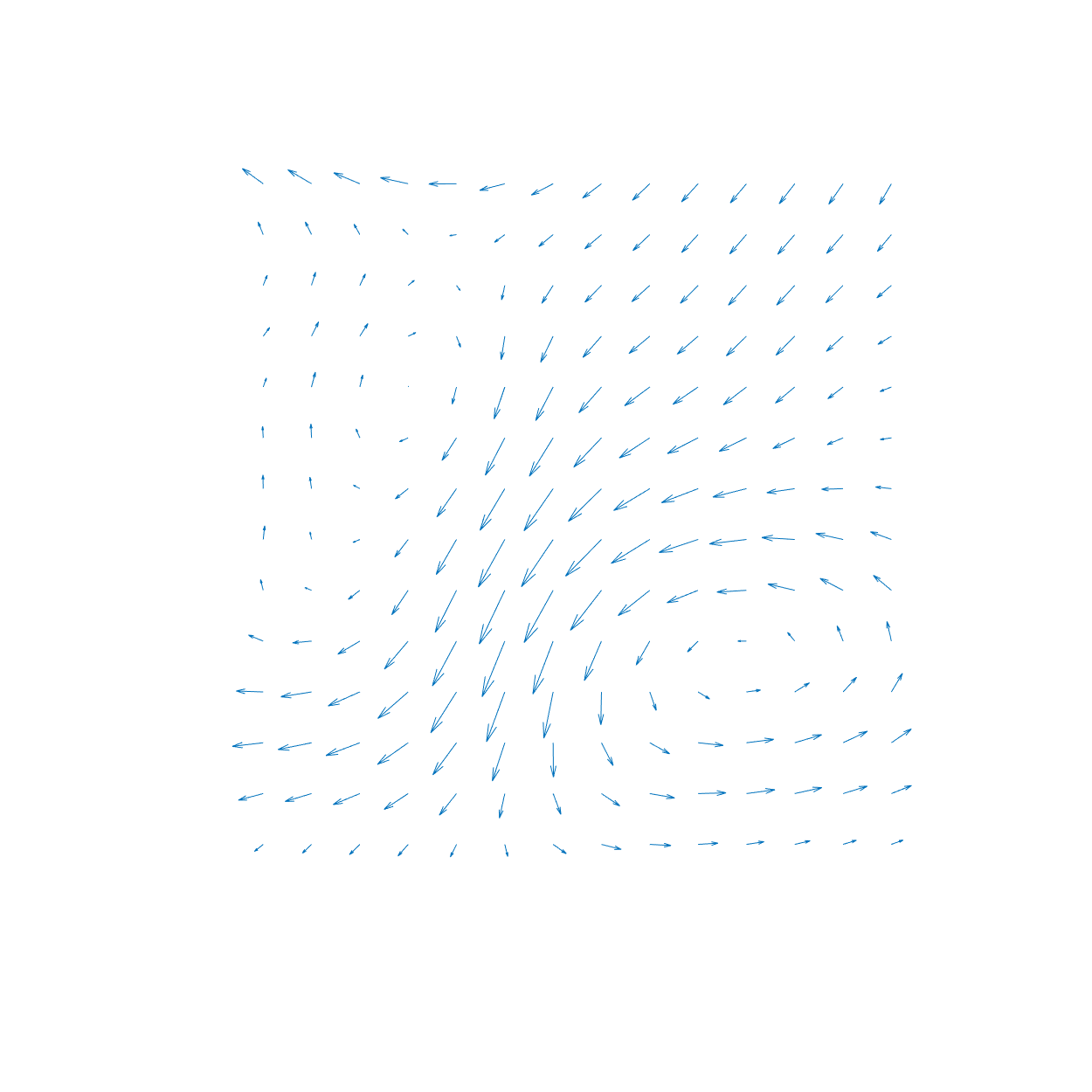

Supplement: S1 MCG raw data 1 — The raw MCG dataset includes categories 0-4 for testing. (ZIP) [file pone.0338189.s001.zip › test/1/p3_245_4.png]

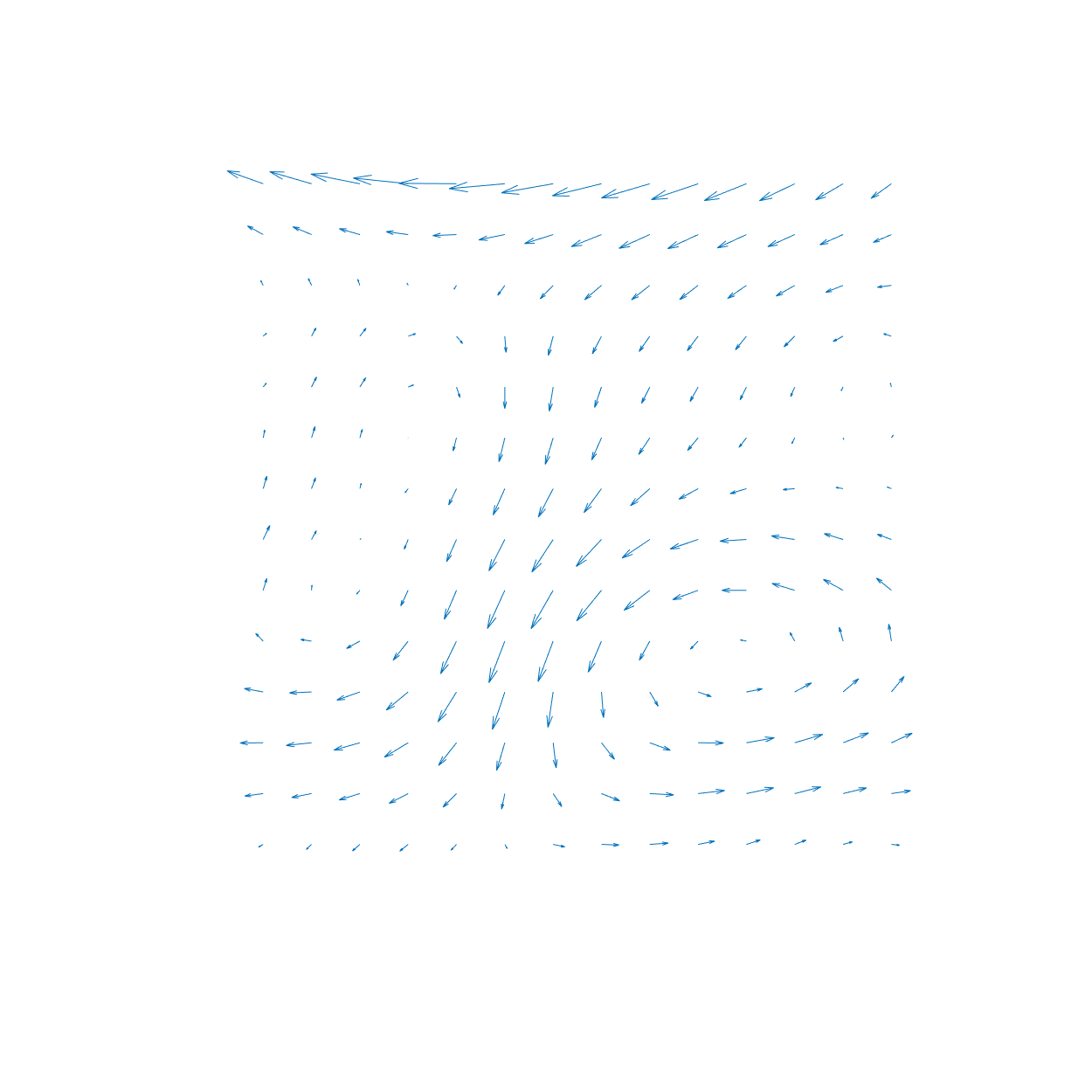

Supplement: S1 MCG raw data 1 — The raw MCG dataset includes categories 0-4 for testing. (ZIP) [file pone.0338189.s001.zip › test/1/p3_250_4.png]

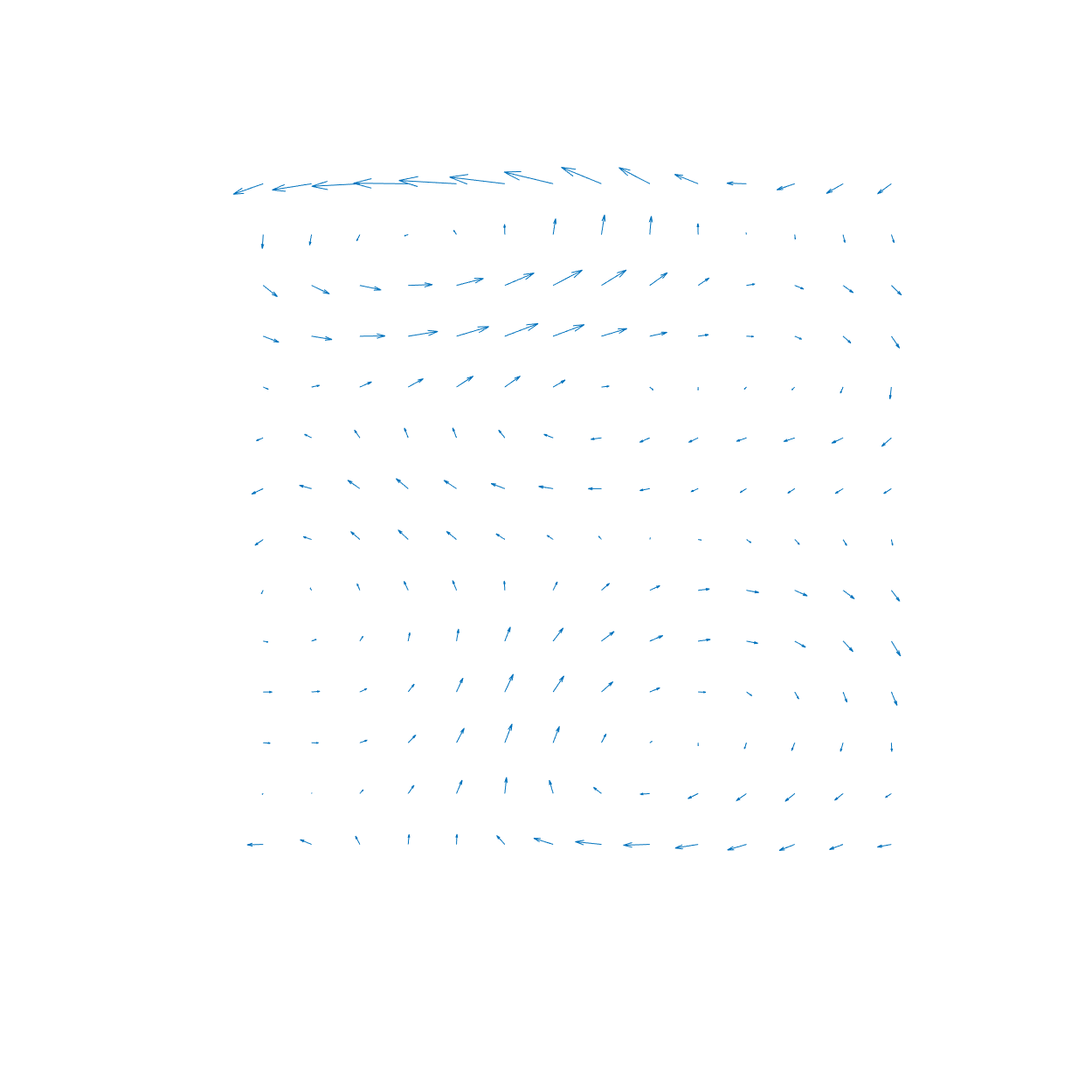

Supplement: S1 MCG raw data 1 — The raw MCG dataset includes categories 0-4 for testing. (ZIP) [file pone.0338189.s001.zip › test/1/p3_400_4.png]

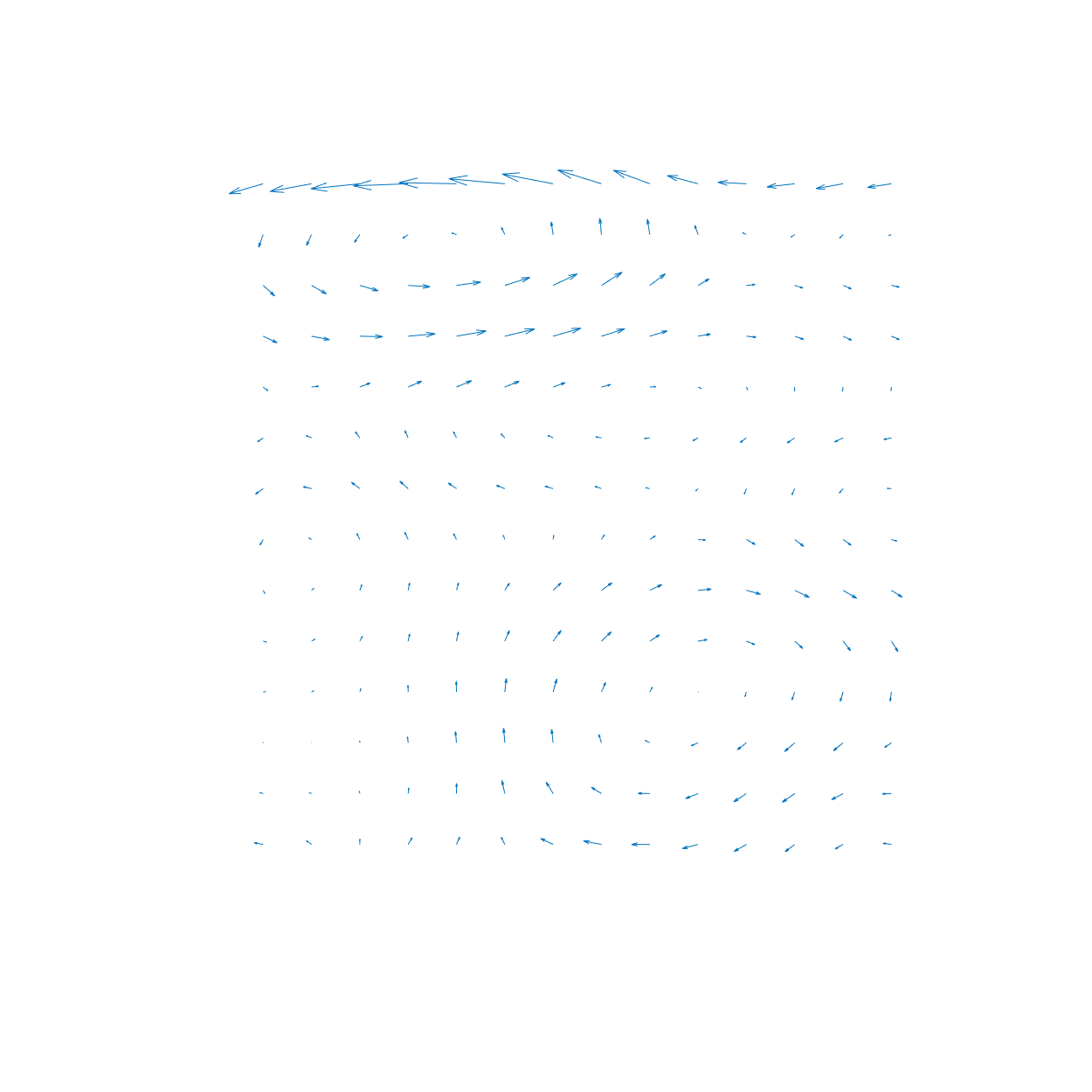

Supplement: S1 MCG raw data 1 — The raw MCG dataset includes categories 0-4 for testing. (ZIP) [file pone.0338189.s001.zip › test/1/p3_405_4.png]

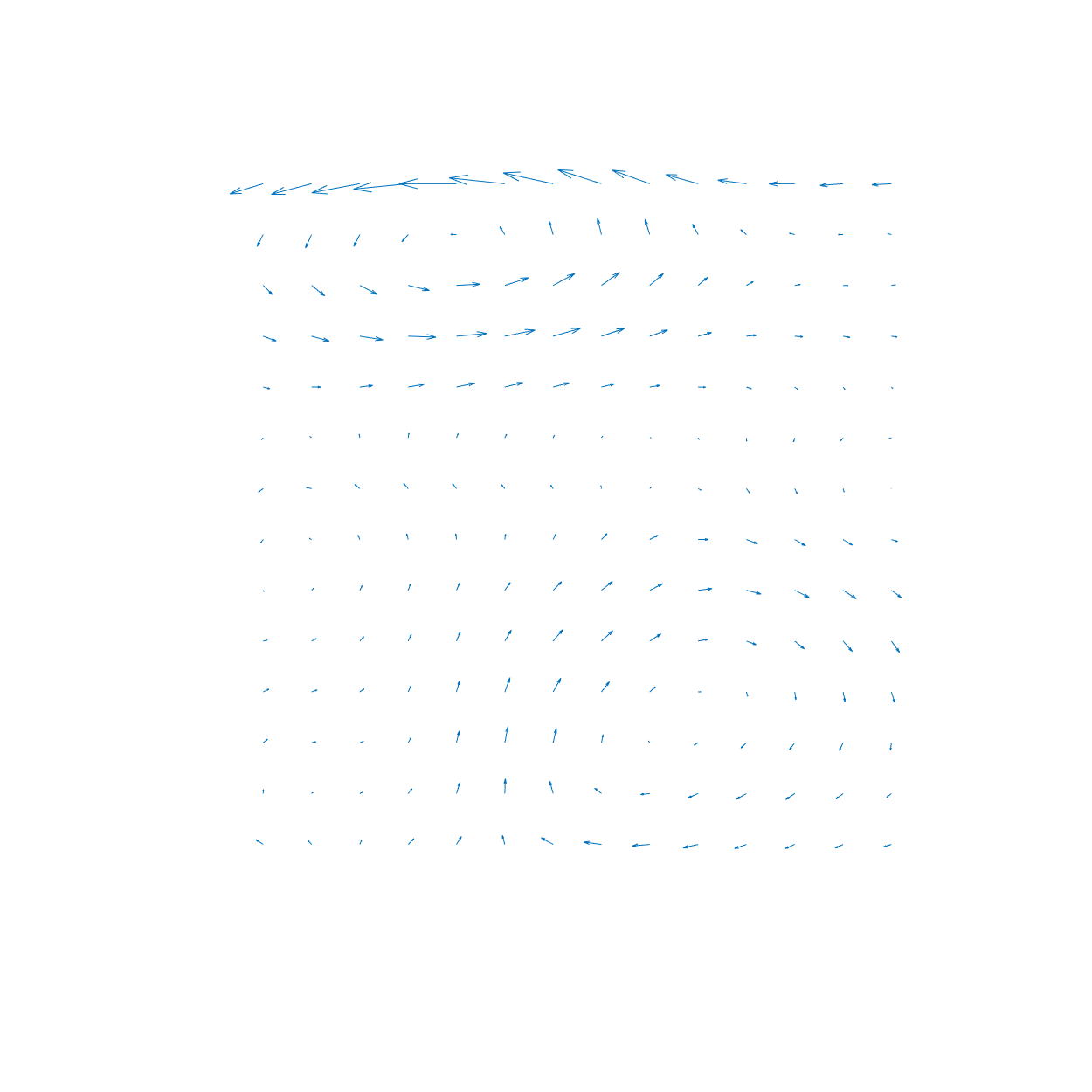

Supplement: S1 MCG raw data 1 — The raw MCG dataset includes categories 0-4 for testing. (ZIP) [file pone.0338189.s001.zip › test/1/p3_410_4.png]

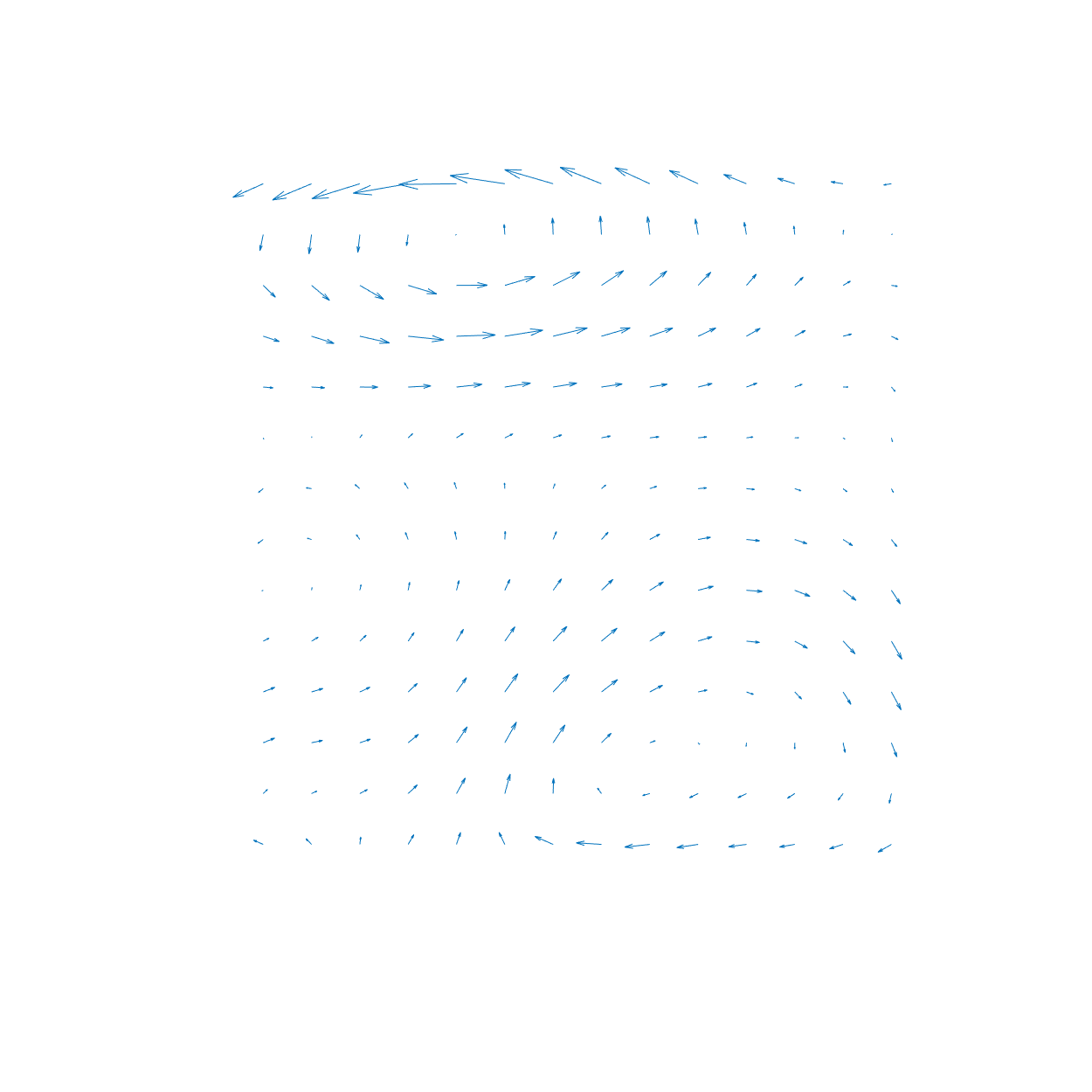

Supplement: S1 MCG raw data 1 — The raw MCG dataset includes categories 0-4 for testing. (ZIP) [file pone.0338189.s001.zip › test/1/p3_415_4.png]

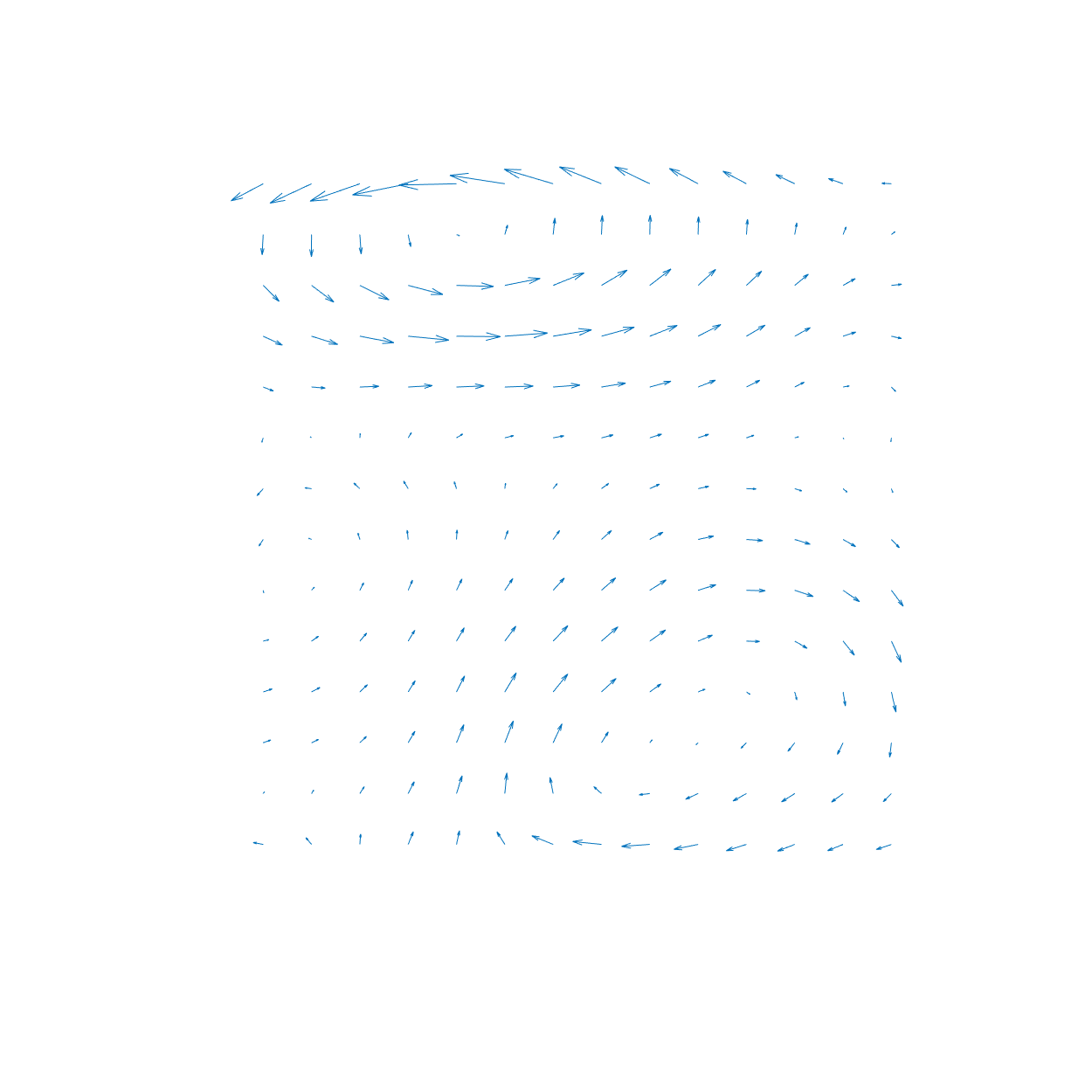

Supplement: S1 MCG raw data 1 — The raw MCG dataset includes categories 0-4 for testing. (ZIP) [file pone.0338189.s001.zip › test/1/p3_420_4.png]

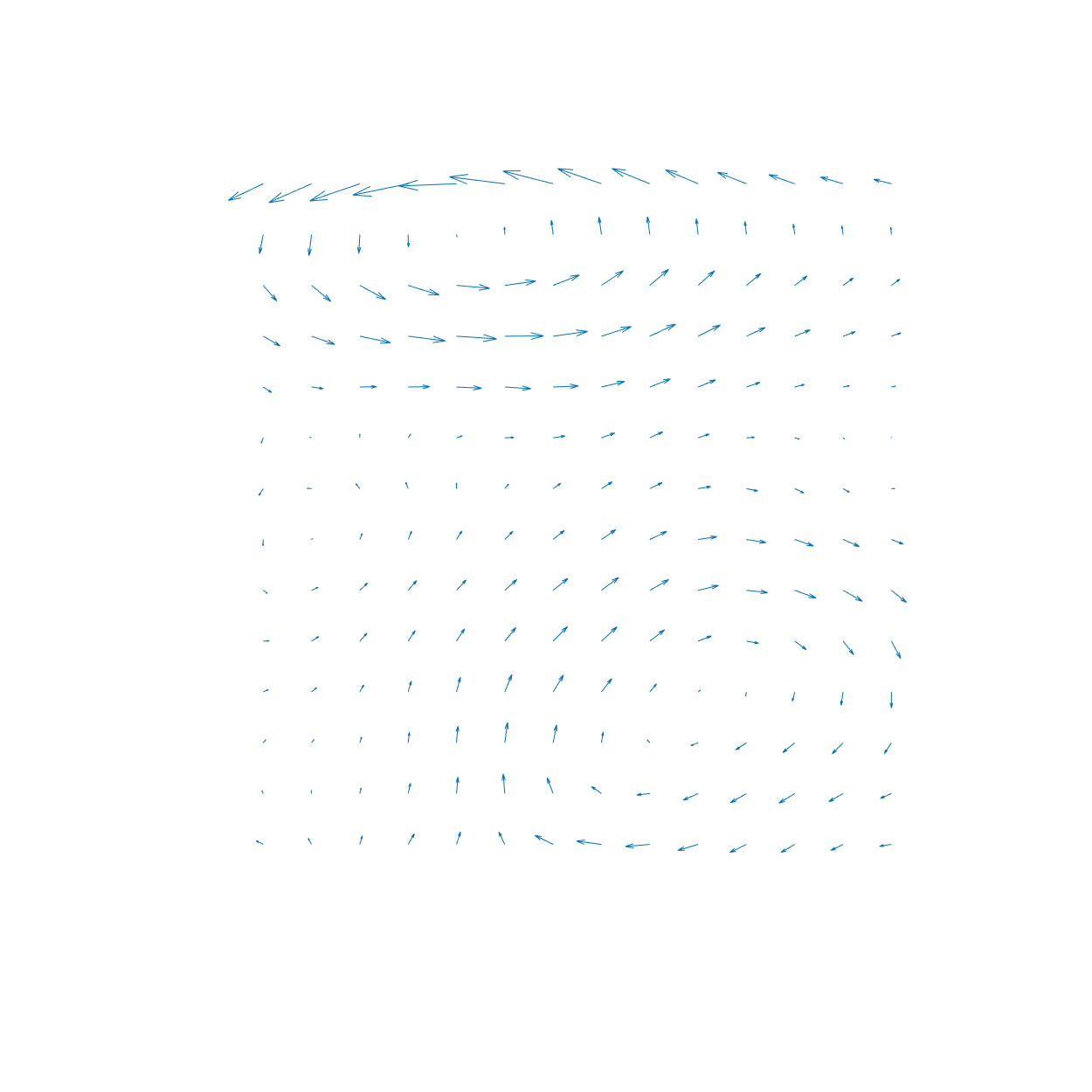

Supplement: S1 MCG raw data 1 — The raw MCG dataset includes categories 0-4 for testing. (ZIP) [file pone.0338189.s001.zip › test/1/p3_425_4.png]

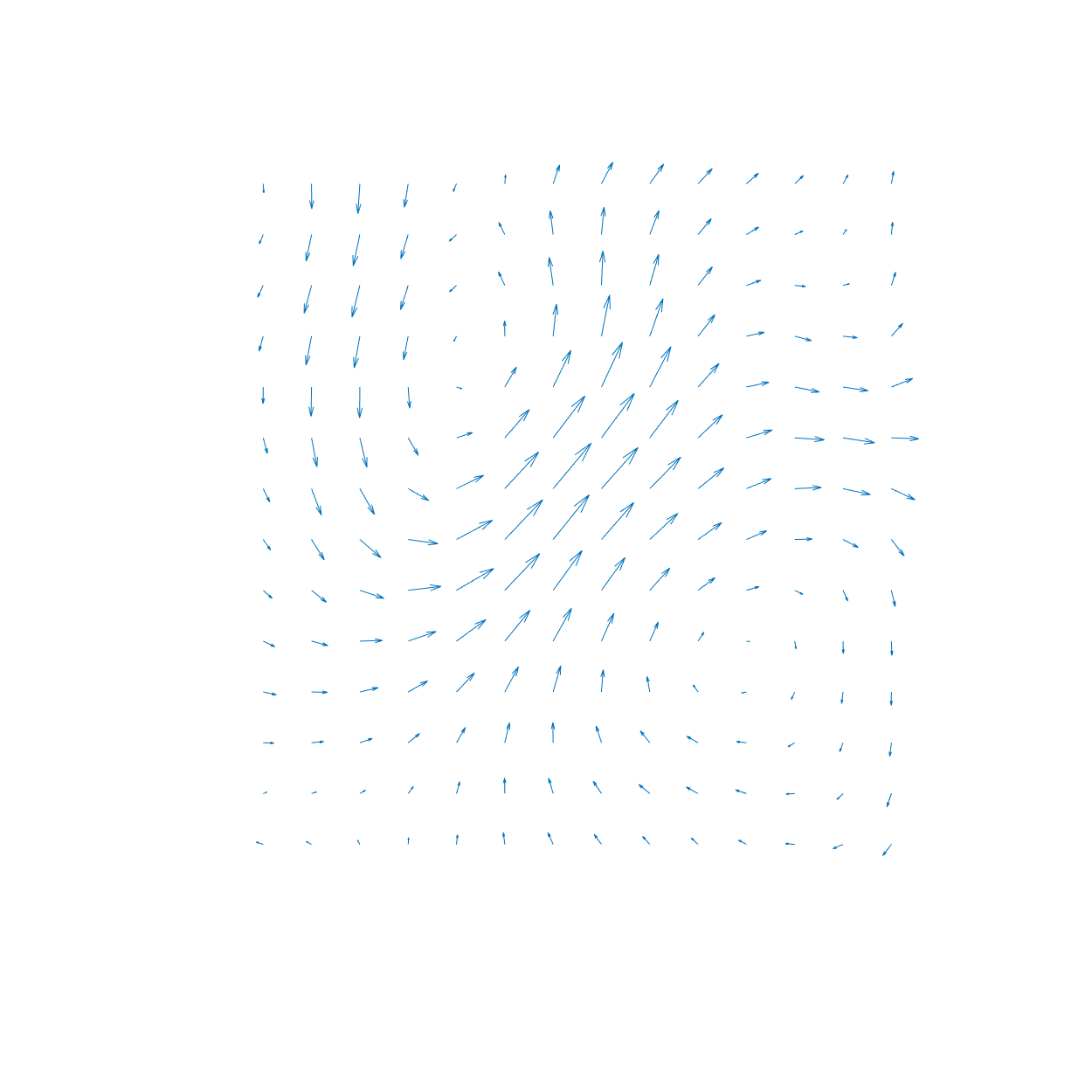

Supplement: S1 MCG raw data 1 — The raw MCG dataset includes categories 0-4 for testing. (ZIP) [file pone.0338189.s001.zip › test/1/p4_395_4.png]

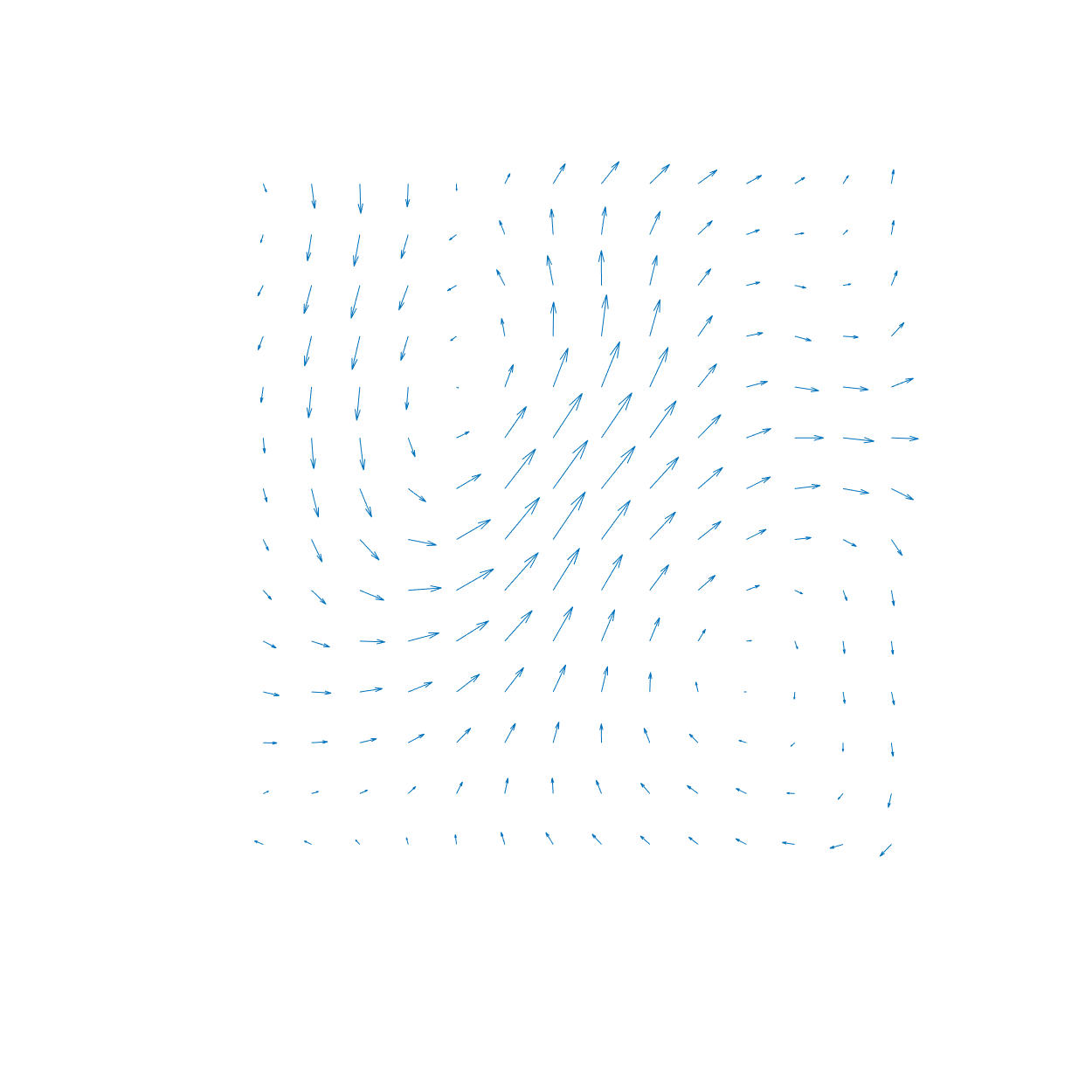

Supplement: S1 MCG raw data 1 — The raw MCG dataset includes categories 0-4 for testing. (ZIP) [file pone.0338189.s001.zip › test/1/p4_400_4.png]

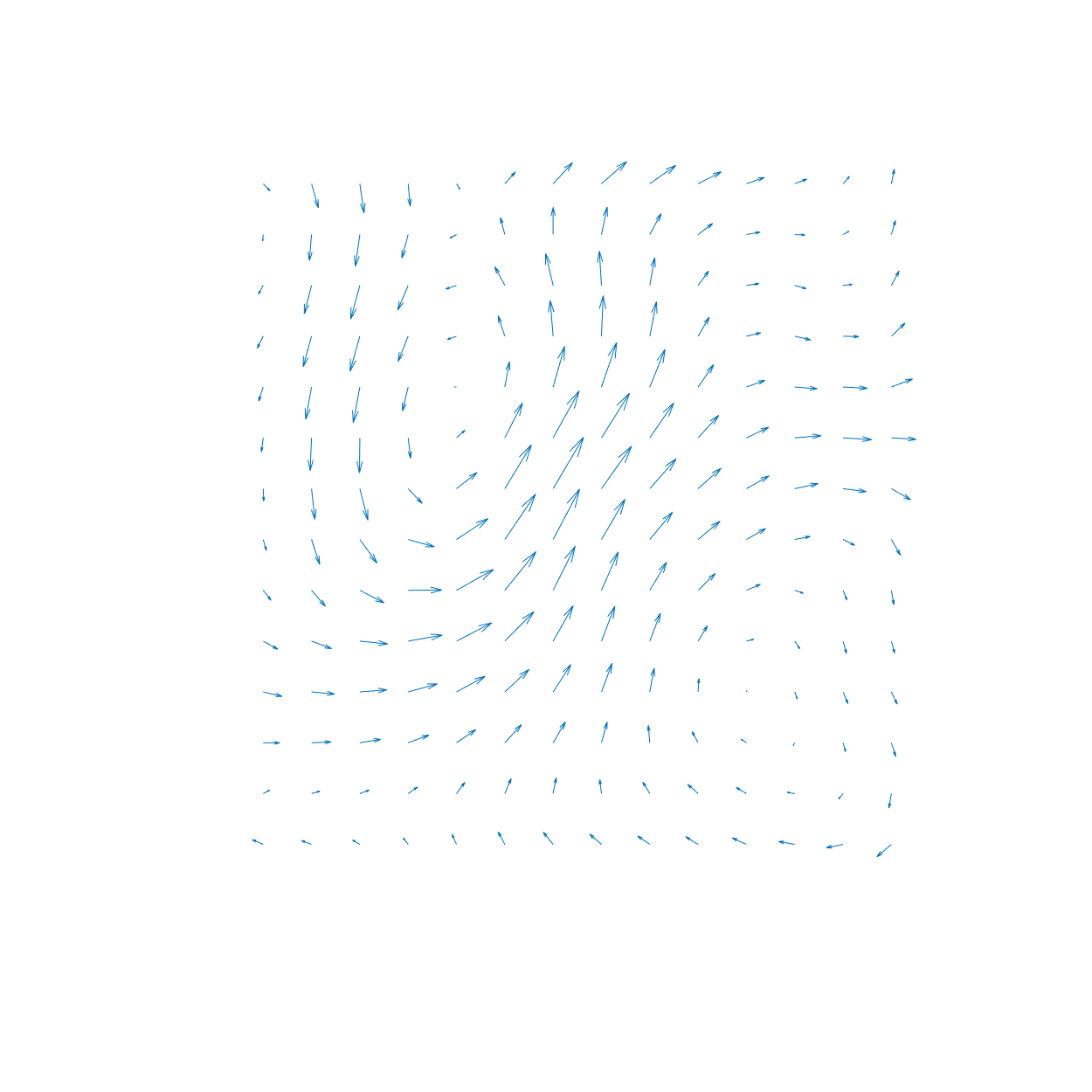

Supplement: S1 MCG raw data 1 — The raw MCG dataset includes categories 0-4 for testing. (ZIP) [file pone.0338189.s001.zip › test/1/p4_405_4.png]

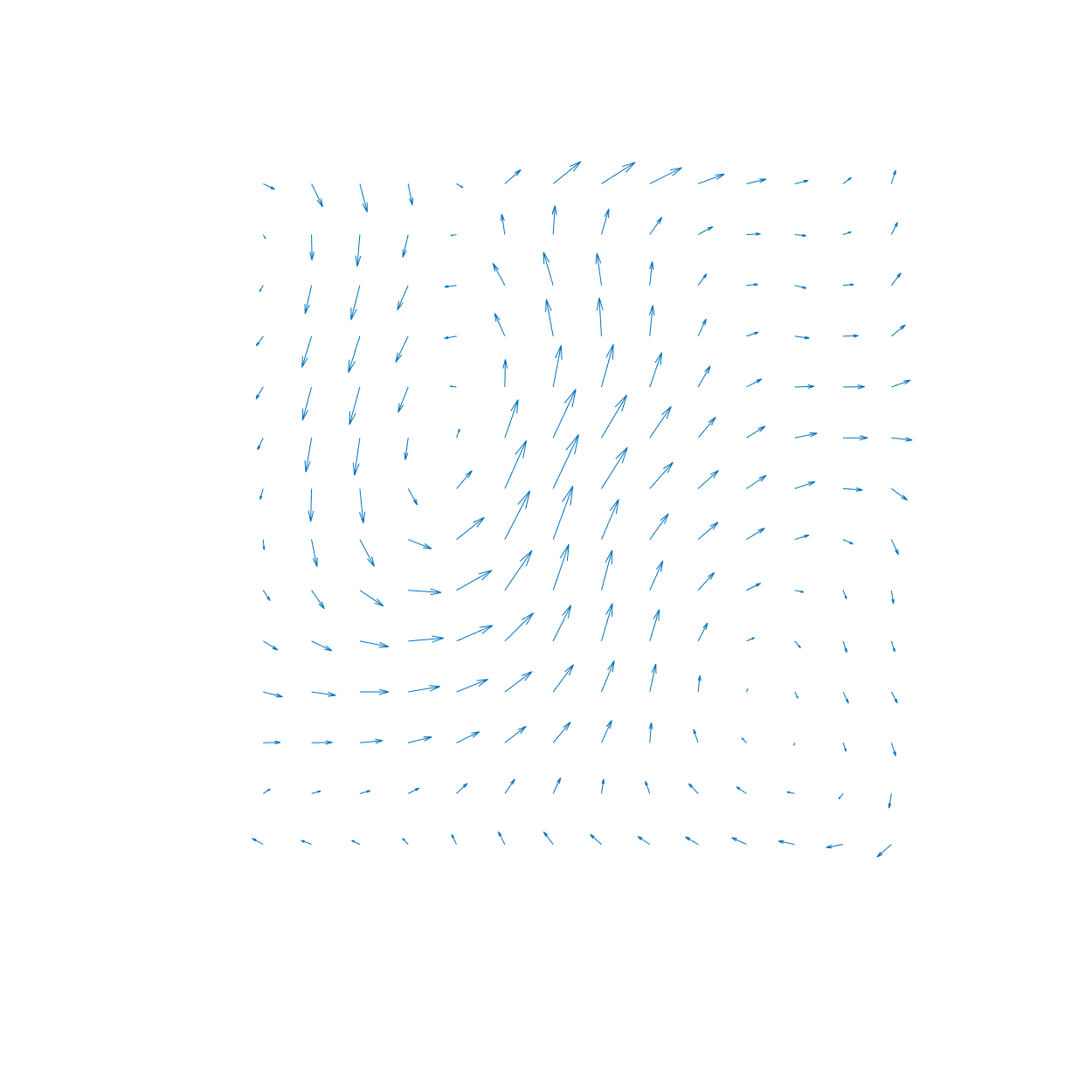

Supplement: S1 MCG raw data 1 — The raw MCG dataset includes categories 0-4 for testing. (ZIP) [file pone.0338189.s001.zip › test/1/p4_410_4.png]

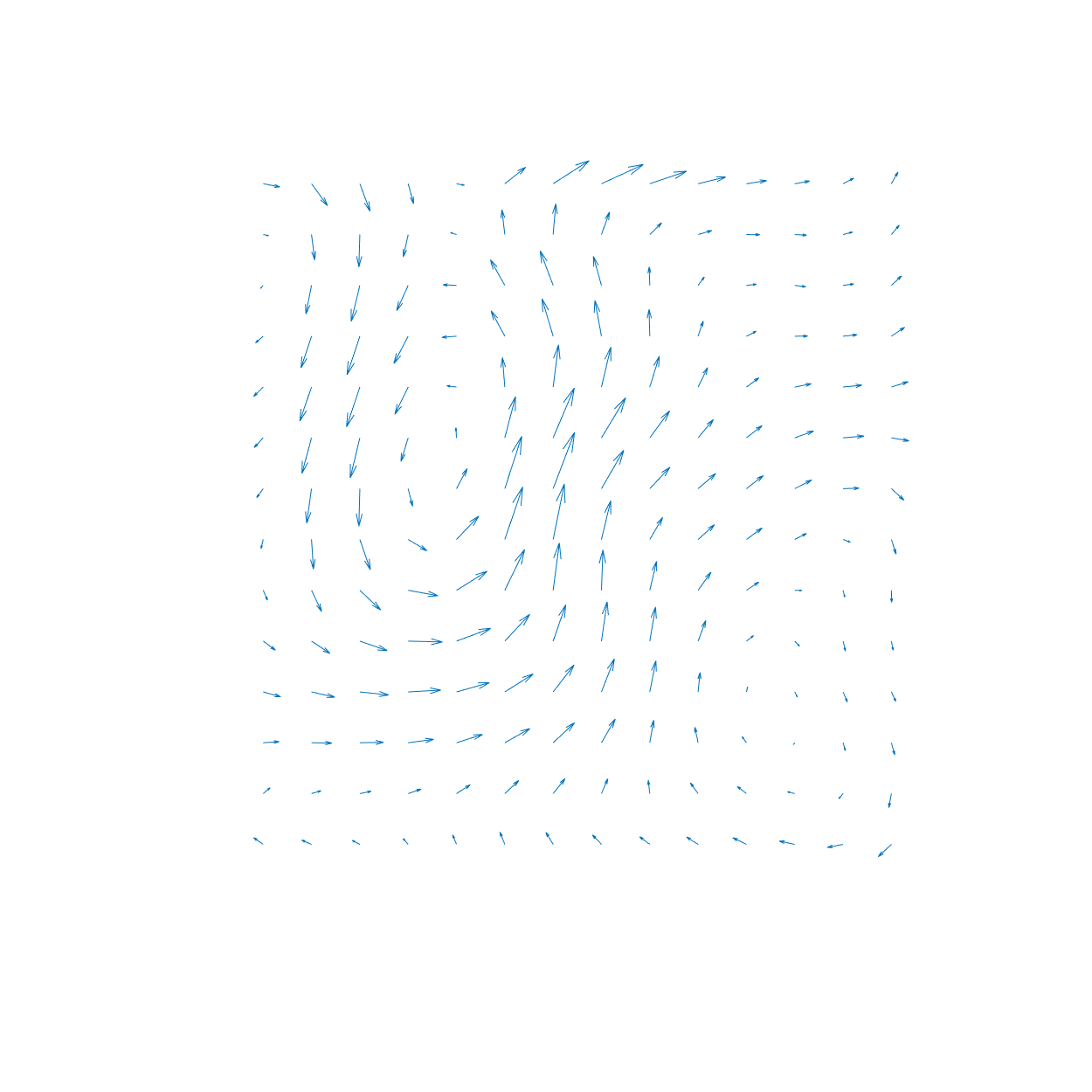

Supplement: S1 MCG raw data 1 — The raw MCG dataset includes categories 0-4 for testing. (ZIP) [file pone.0338189.s001.zip › test/1/p4_415_4.png]

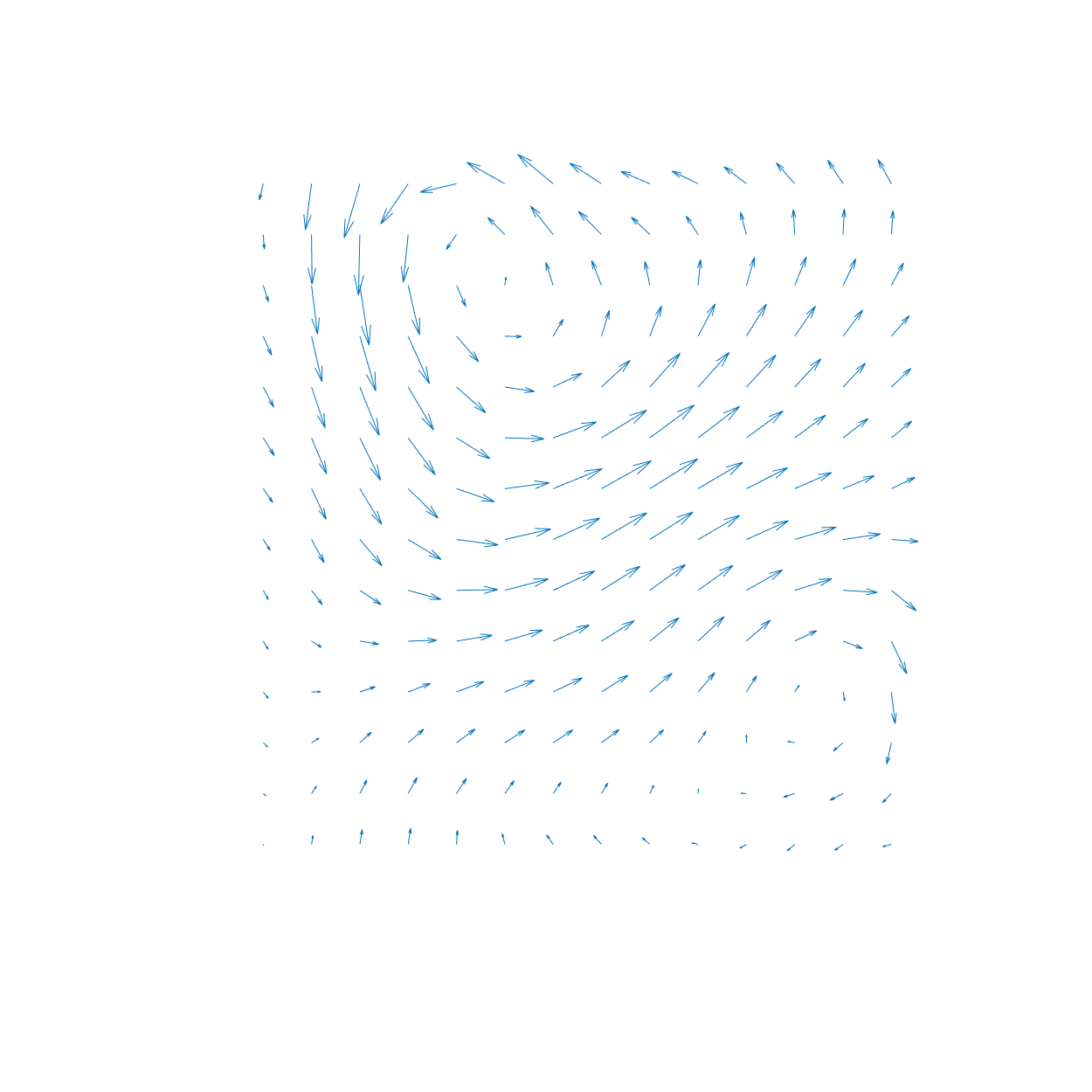

Supplement: S1 MCG raw data 1 — The raw MCG dataset includes categories 0-4 for testing. (ZIP) [file pone.0338189.s001.zip › test/1/p6_400_4.png]

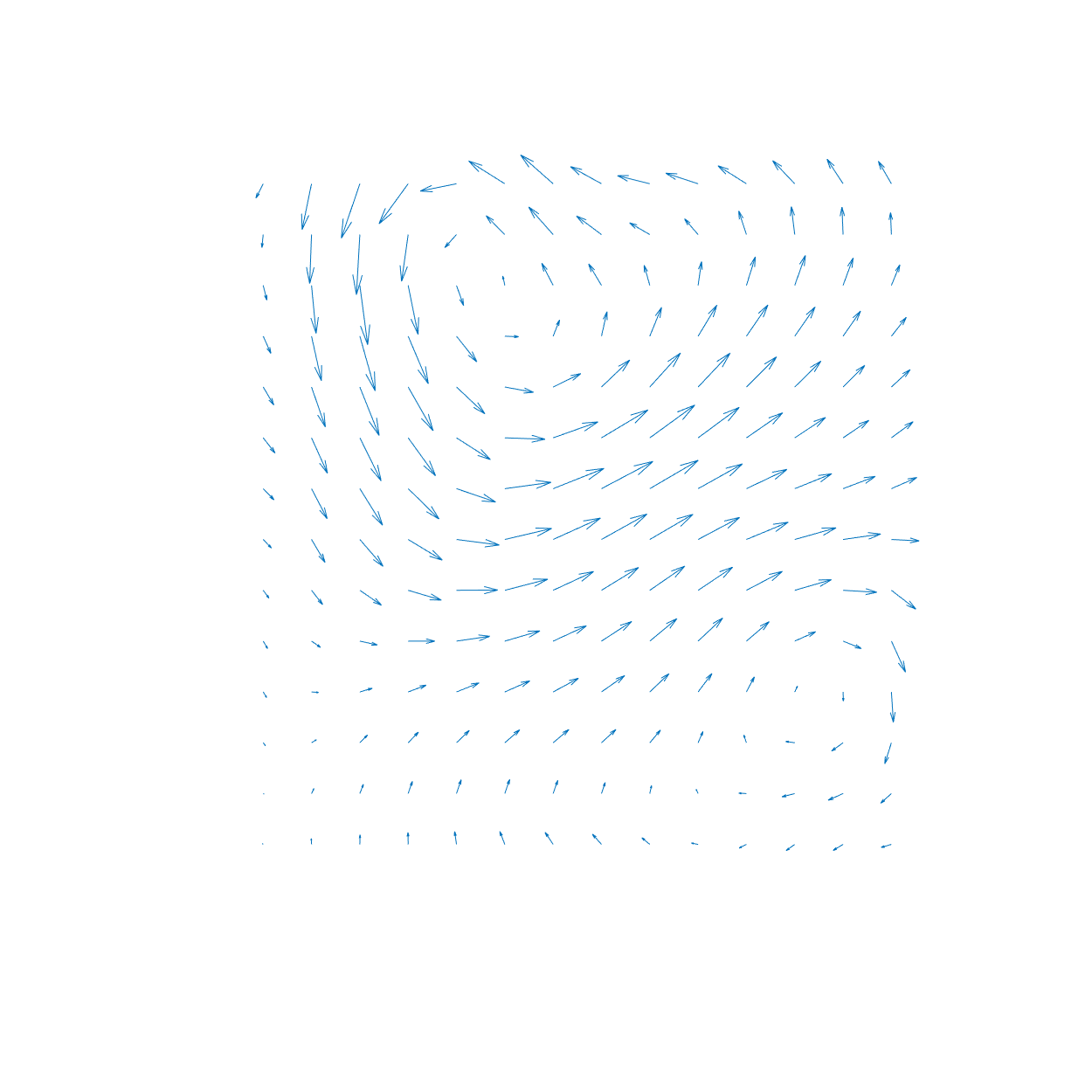

Supplement: S1 MCG raw data 1 — The raw MCG dataset includes categories 0-4 for testing. (ZIP) [file pone.0338189.s001.zip › test/1/p6_405_4.png]

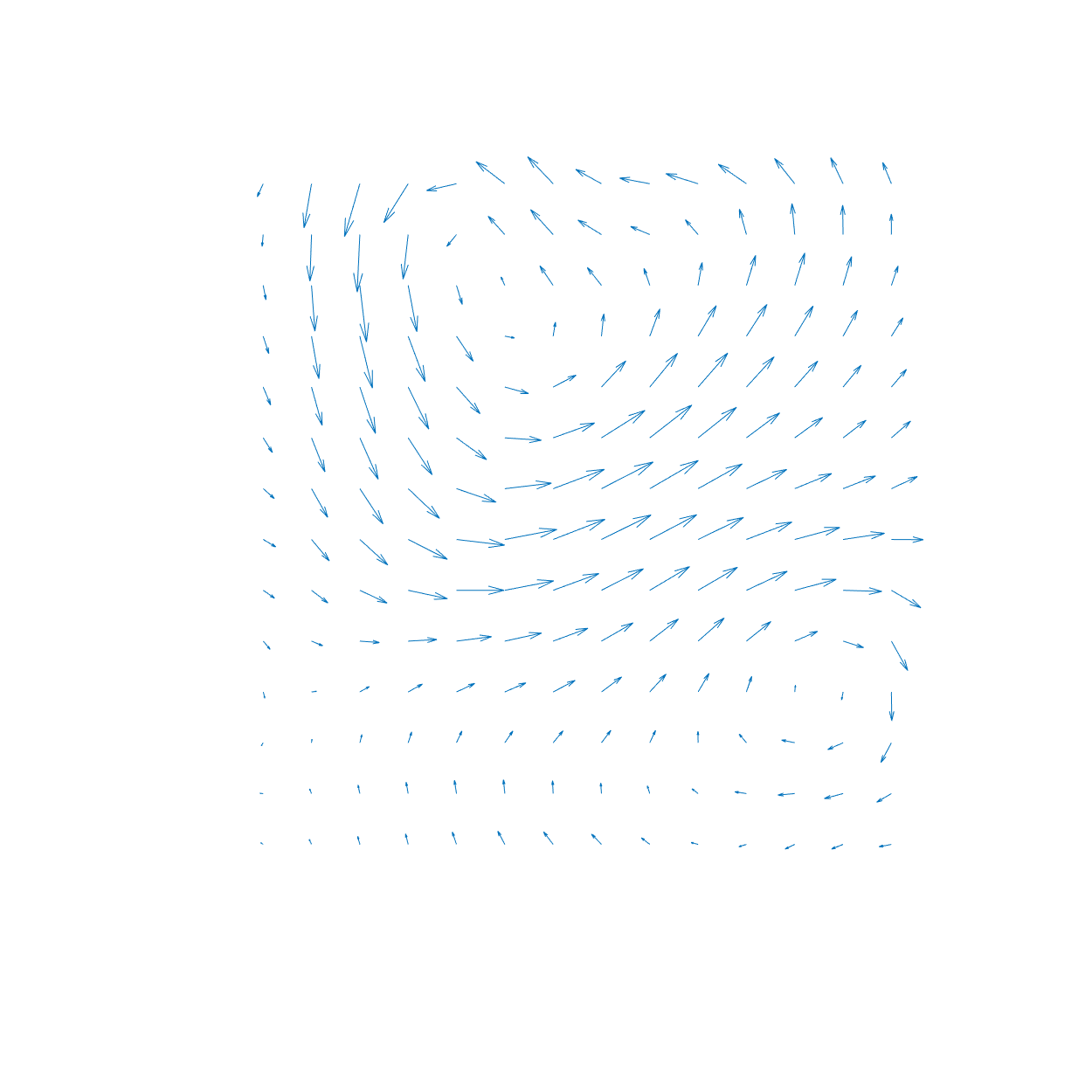

Supplement: S1 MCG raw data 1 — The raw MCG dataset includes categories 0-4 for testing. (ZIP) [file pone.0338189.s001.zip › test/1/p6_410_4.png]

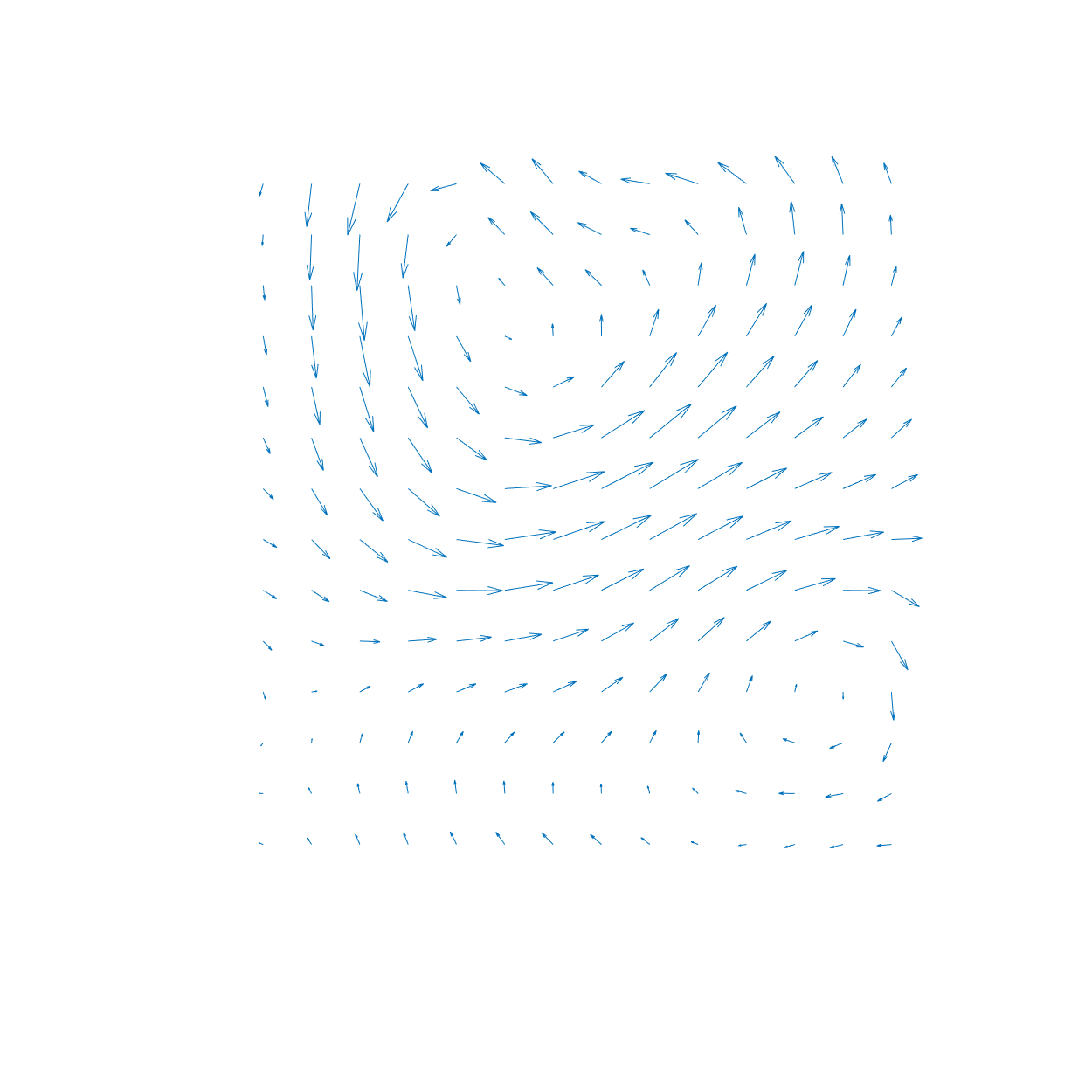

Supplement: S1 MCG raw data 1 — The raw MCG dataset includes categories 0-4 for testing. (ZIP) [file pone.0338189.s001.zip › test/1/p6_415_4.png]

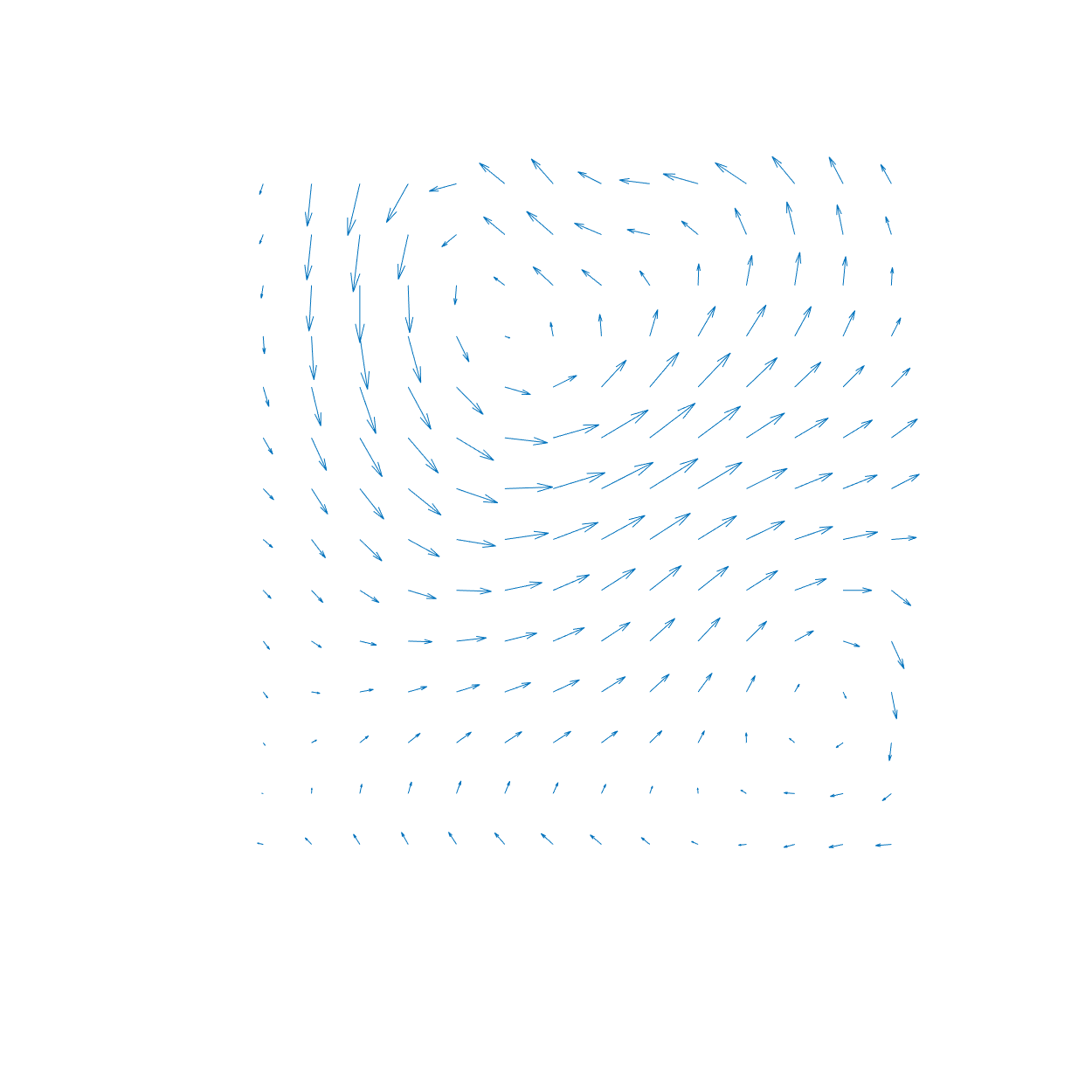

Supplement: S1 MCG raw data 1 — The raw MCG dataset includes categories 0-4 for testing. (ZIP) [file pone.0338189.s001.zip › test/1/p6_420_4.png]

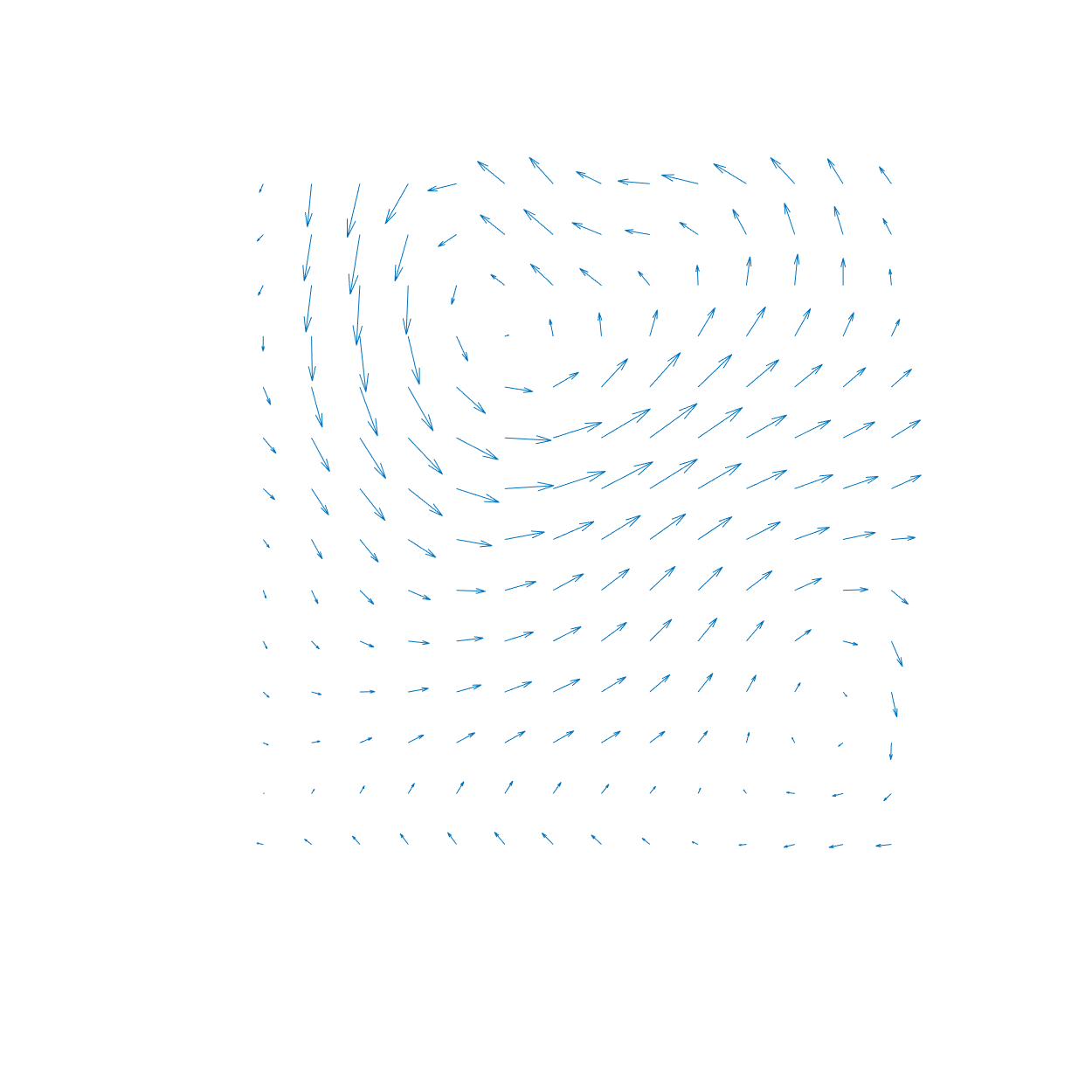

Supplement: S1 MCG raw data 1 — The raw MCG dataset includes categories 0-4 for testing. (ZIP) [file pone.0338189.s001.zip › test/1/p6_425_4.png]

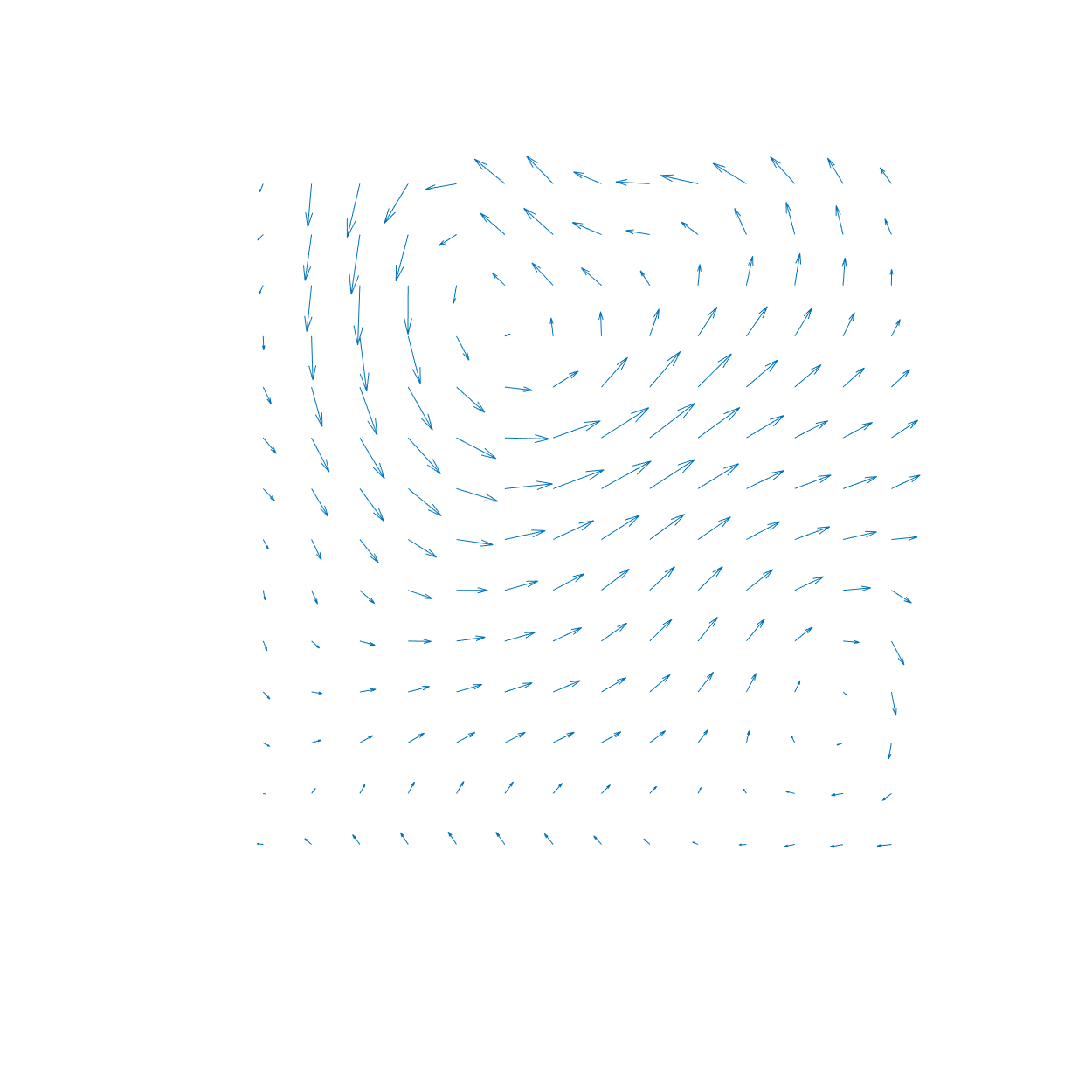

Supplement: S1 MCG raw data 1 — The raw MCG dataset includes categories 0-4 for testing. (ZIP) [file pone.0338189.s001.zip › test/1/p6_430_4.png]

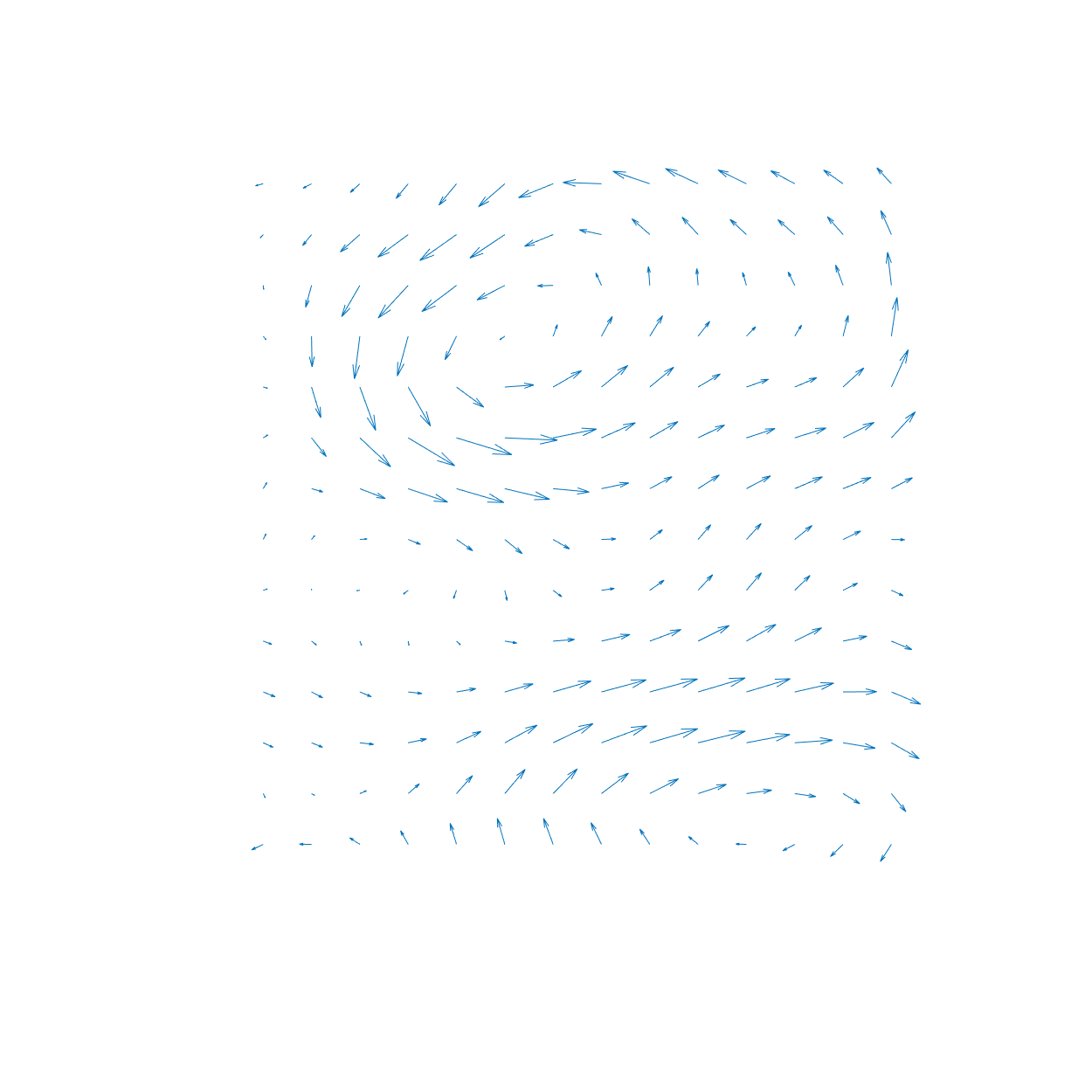

Supplement: S1 MCG raw data 1 — The raw MCG dataset includes categories 0-4 for testing. (ZIP) [file pone.0338189.s001.zip › test/2/p10_490_4.png]

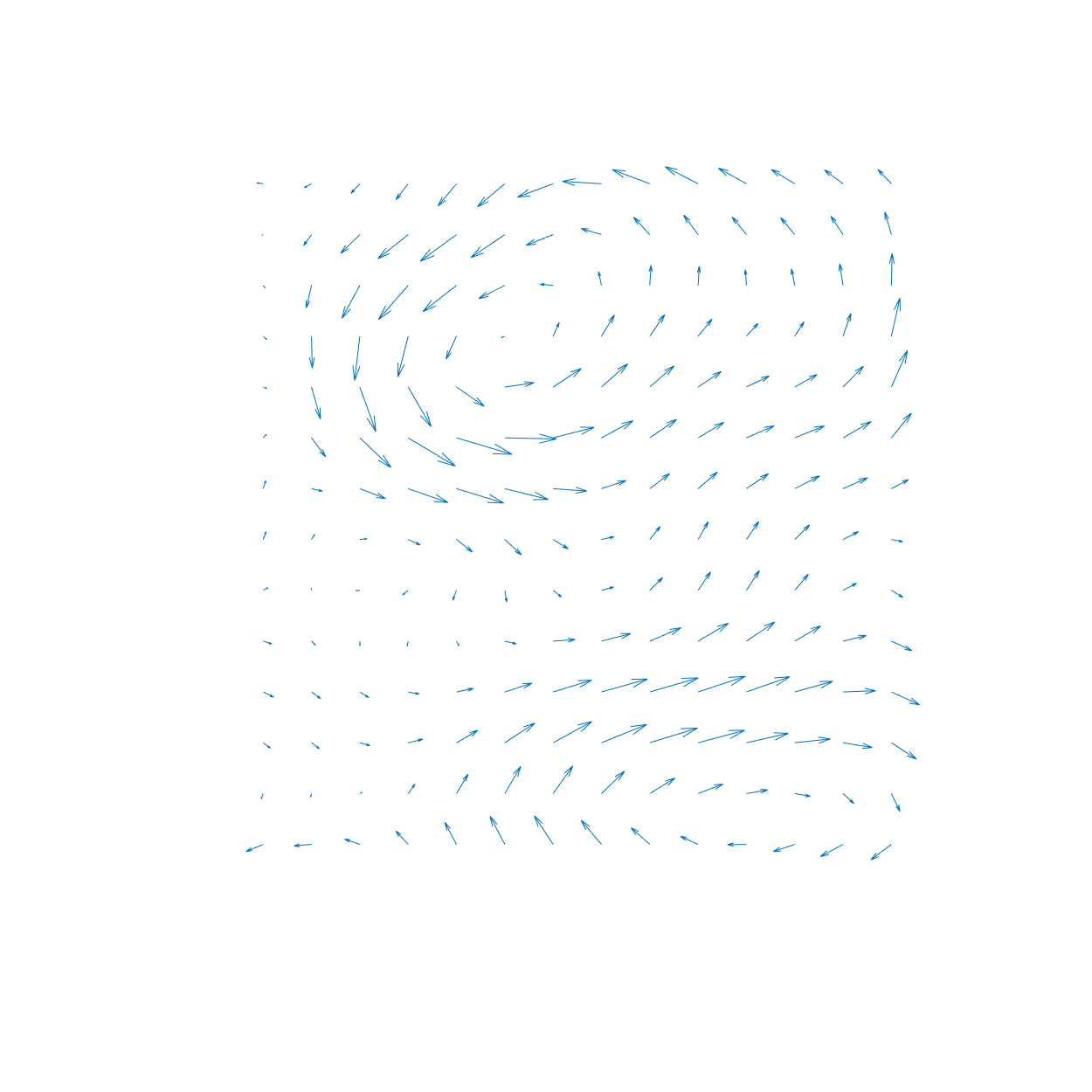

Supplement: S1 MCG raw data 1 — The raw MCG dataset includes categories 0-4 for testing. (ZIP) [file pone.0338189.s001.zip › test/2/p10_495_4.png]

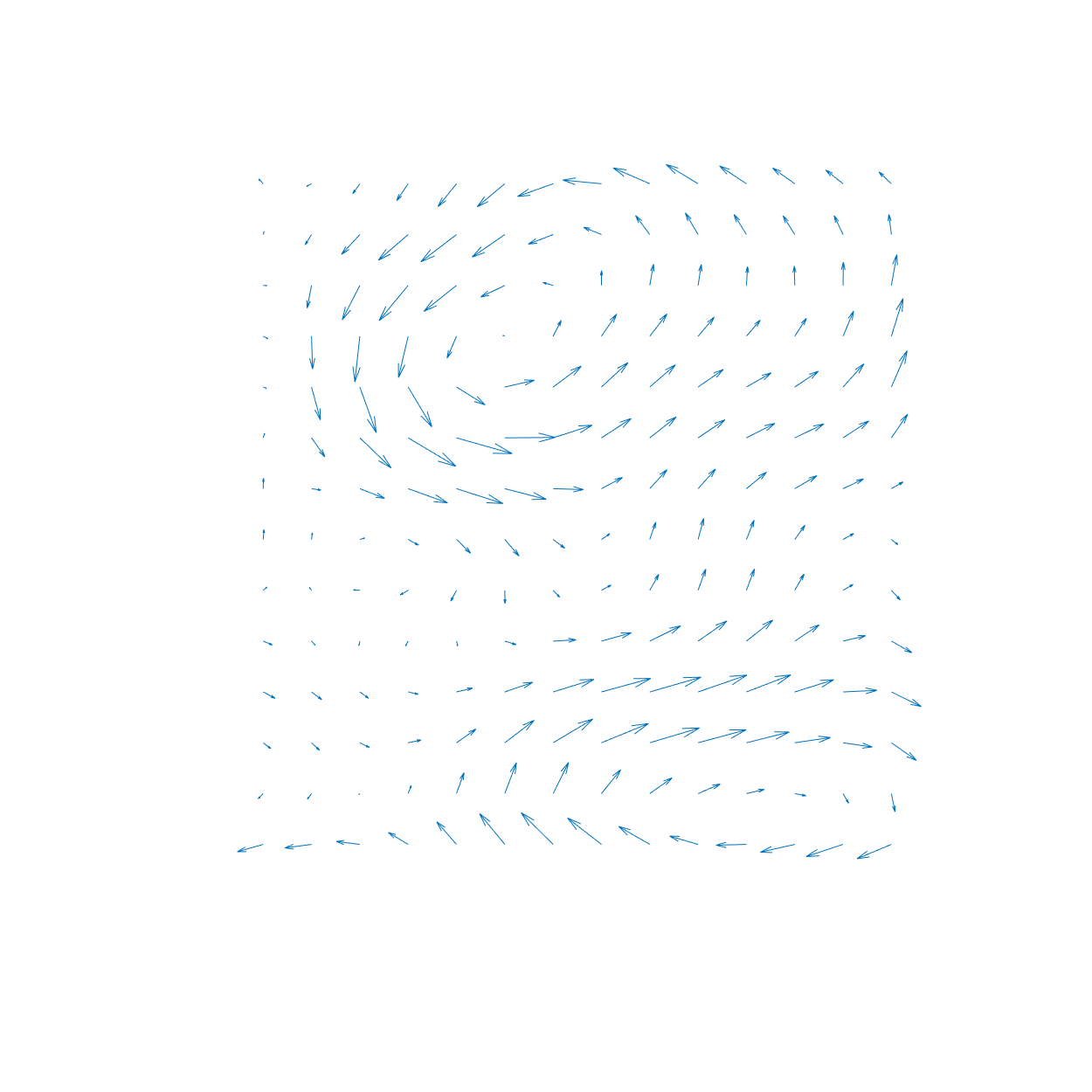

Supplement: S1 MCG raw data 1 — The raw MCG dataset includes categories 0-4 for testing. (ZIP) [file pone.0338189.s001.zip › test/2/p10_500_4.png]

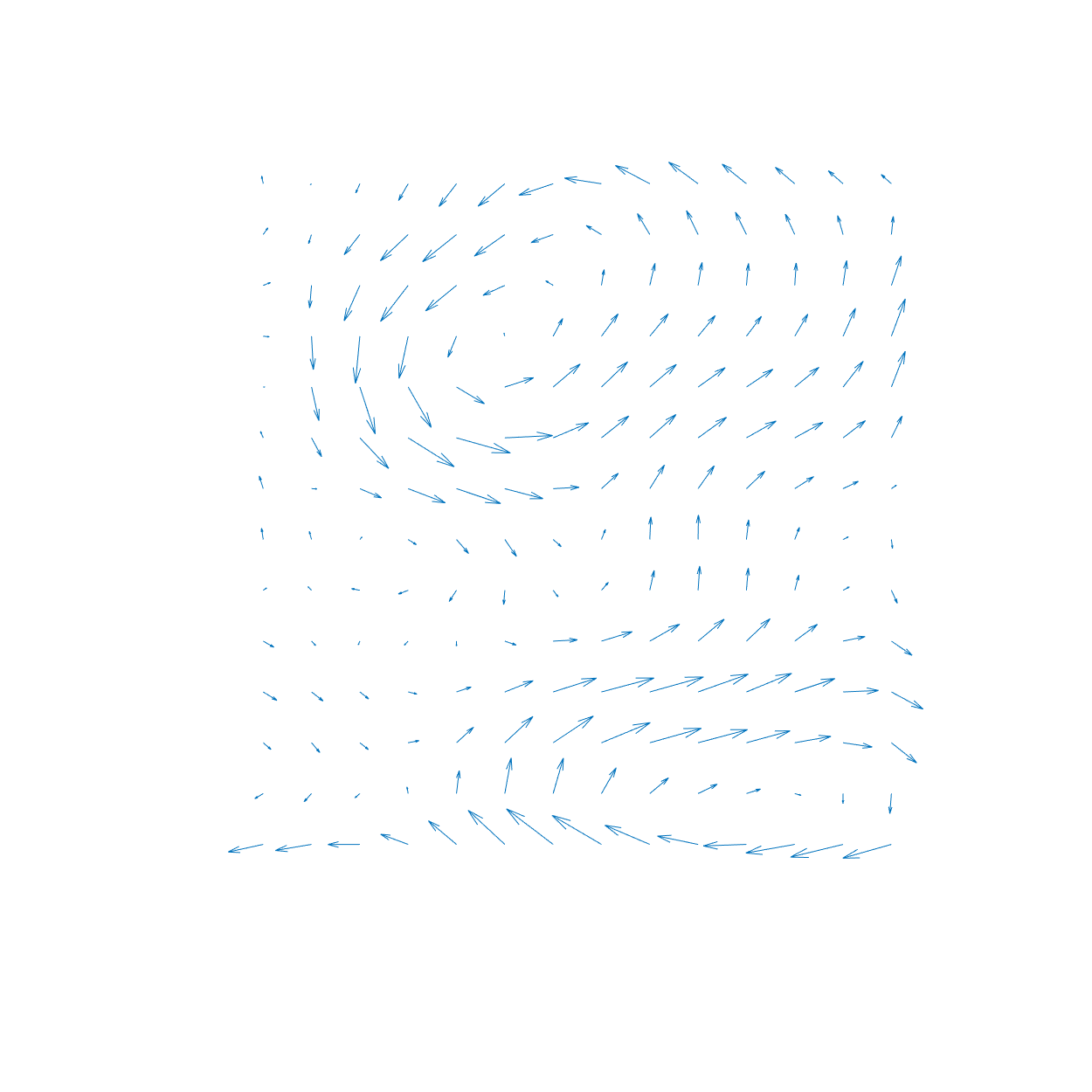

Supplement: S1 MCG raw data 1 — The raw MCG dataset includes categories 0-4 for testing. (ZIP) [file pone.0338189.s001.zip › test/2/p10_505_4.png]

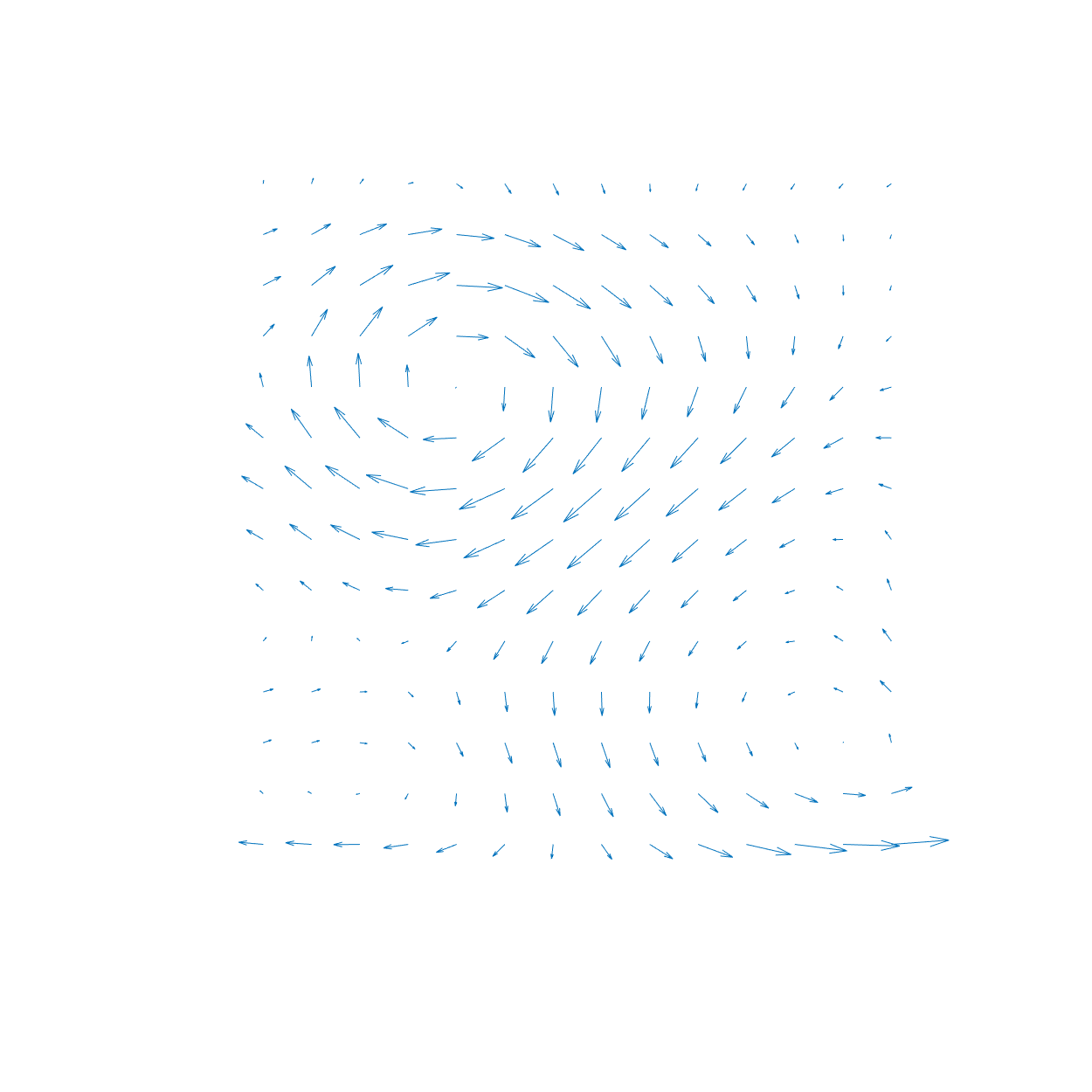

Supplement: S1 MCG raw data 1 — The raw MCG dataset includes categories 0-4 for testing. (ZIP) [file pone.0338189.s001.zip › test/2/p12_220_4.png]

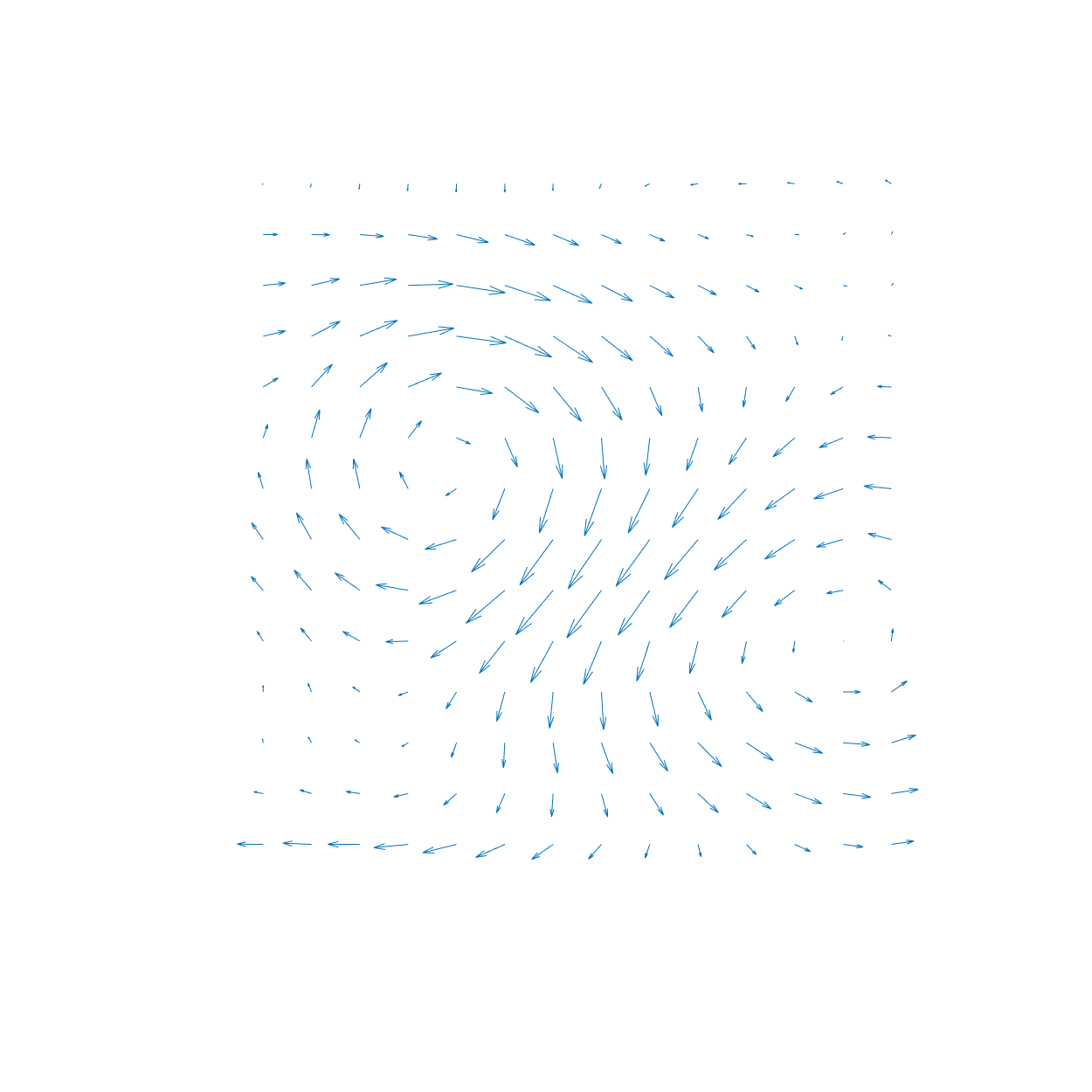

Supplement: S1 MCG raw data 1 — The raw MCG dataset includes categories 0-4 for testing. (ZIP) [file pone.0338189.s001.zip › test/2/p12_225_4.png]

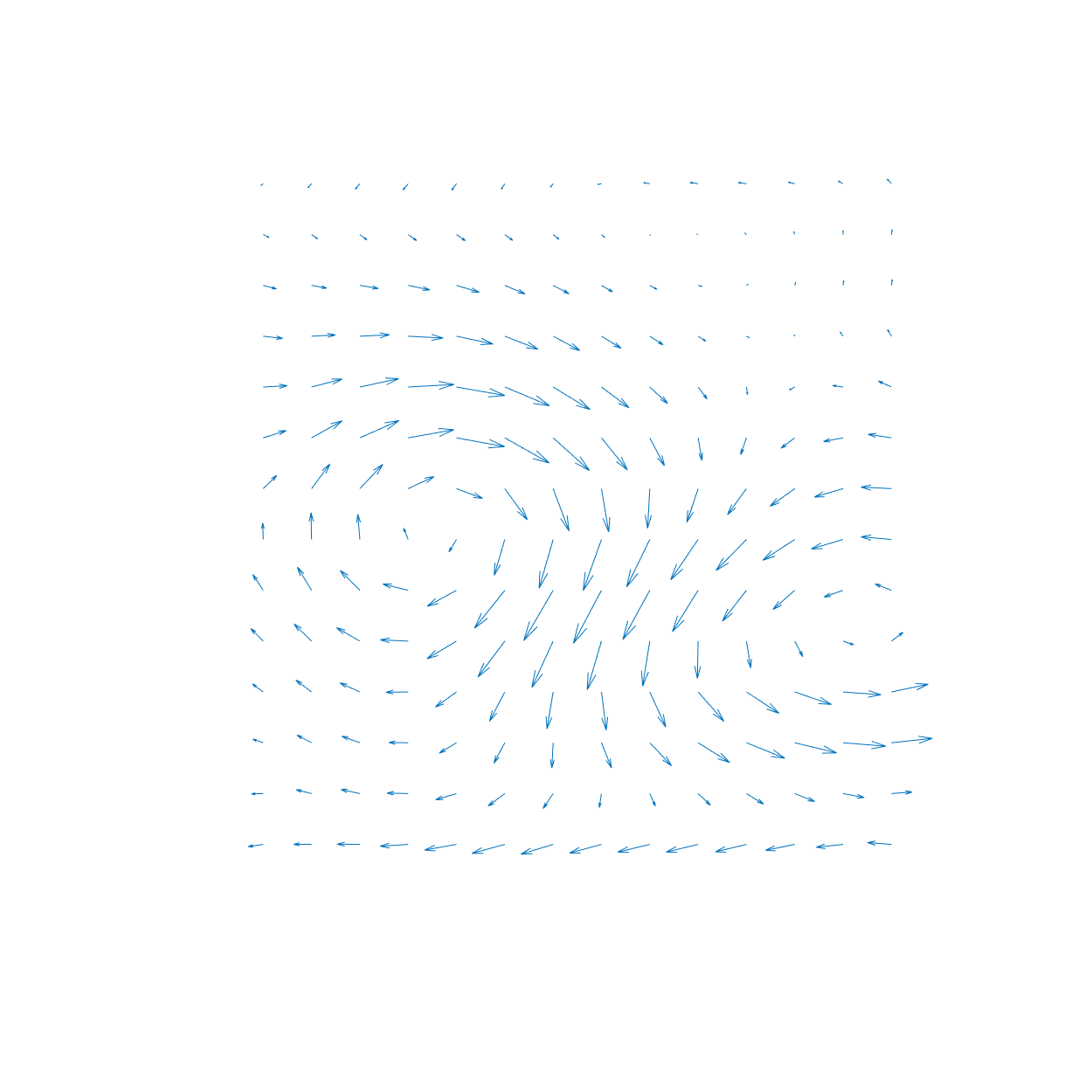

Supplement: S1 MCG raw data 1 — The raw MCG dataset includes categories 0-4 for testing. (ZIP) [file pone.0338189.s001.zip › test/2/p12_230_4.png]

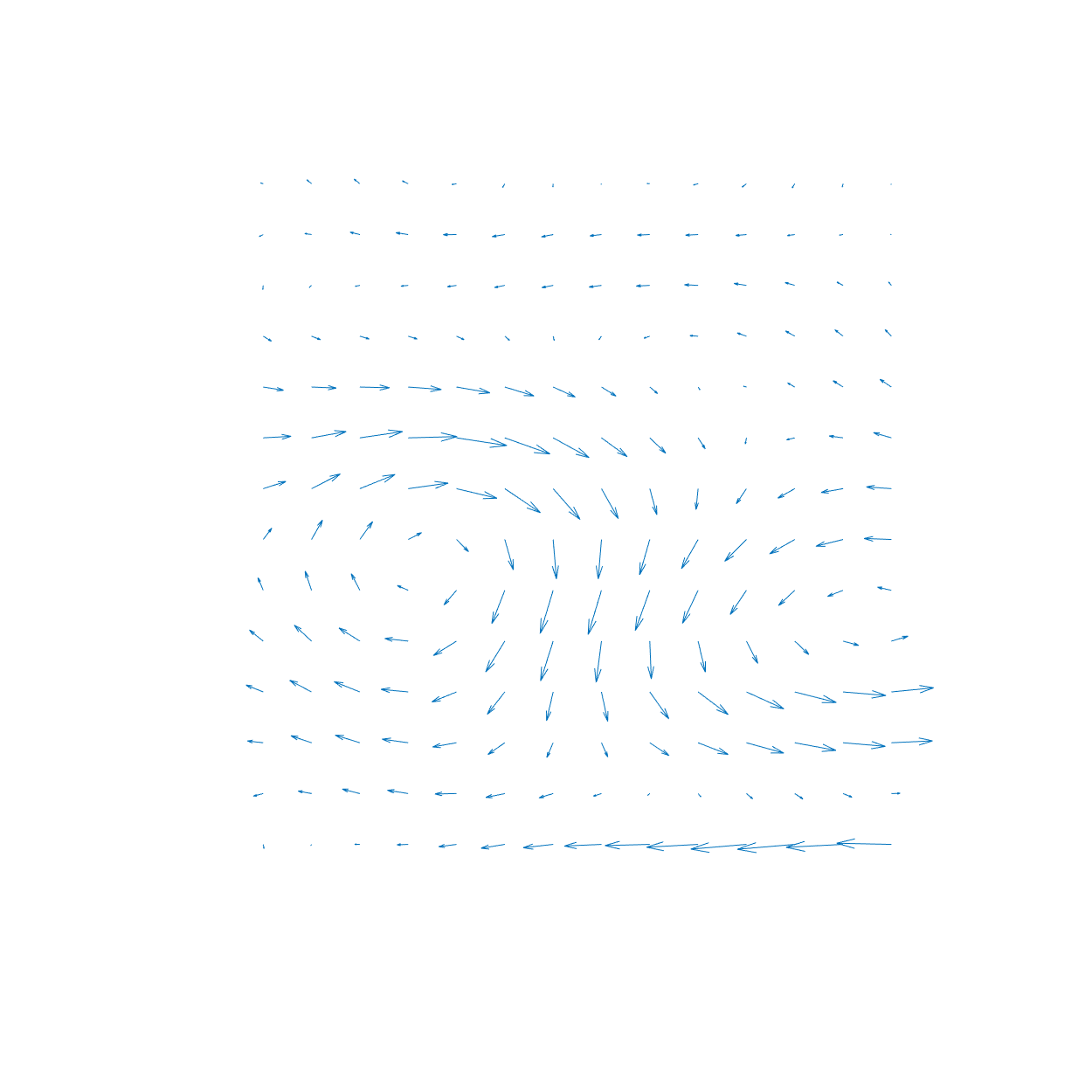

Supplement: S1 MCG raw data 1 — The raw MCG dataset includes categories 0-4 for testing. (ZIP) [file pone.0338189.s001.zip › test/2/p12_235_4.png]

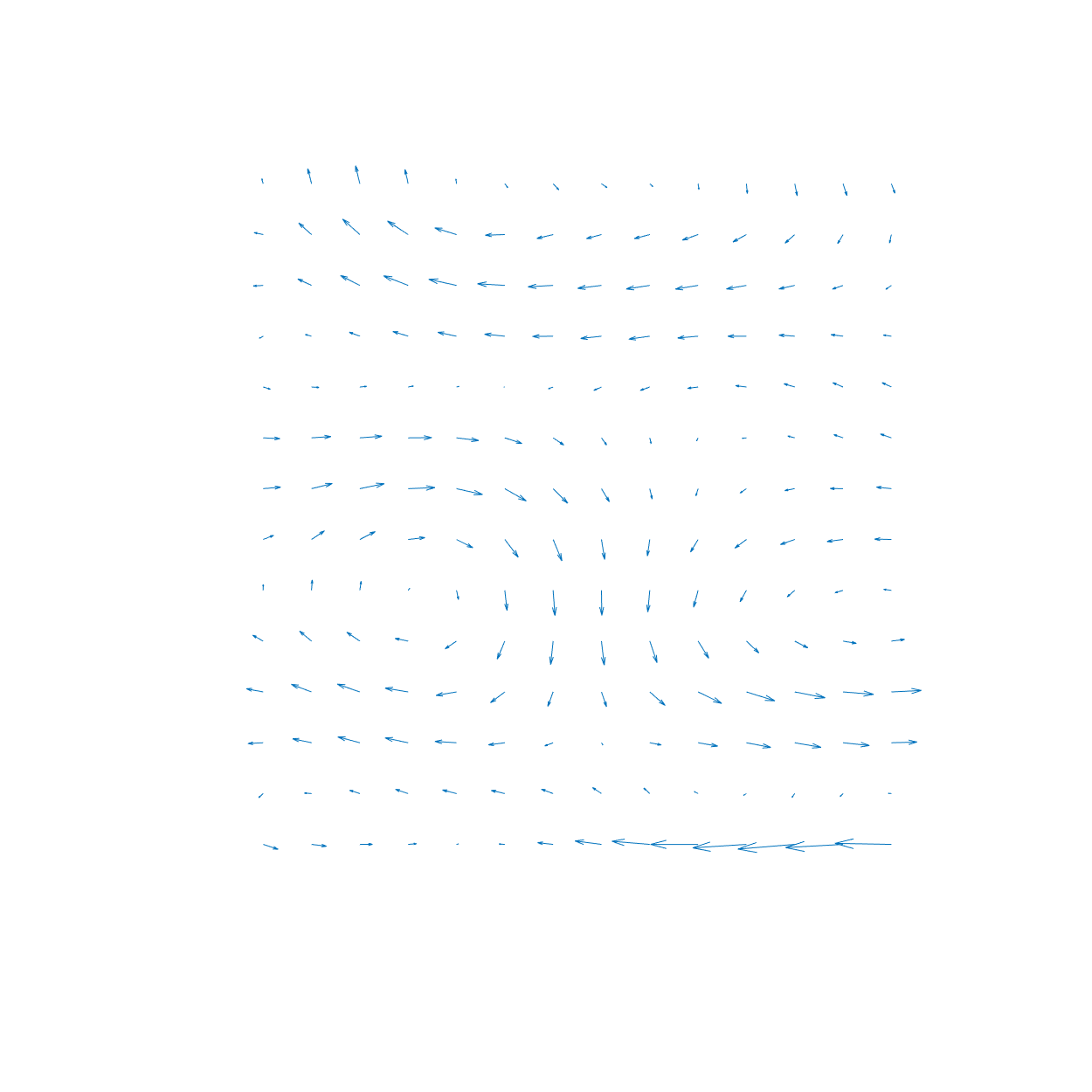

Supplement: S1 MCG raw data 1 — The raw MCG dataset includes categories 0-4 for testing. (ZIP) [file pone.0338189.s001.zip › test/2/p12_240_4.png]

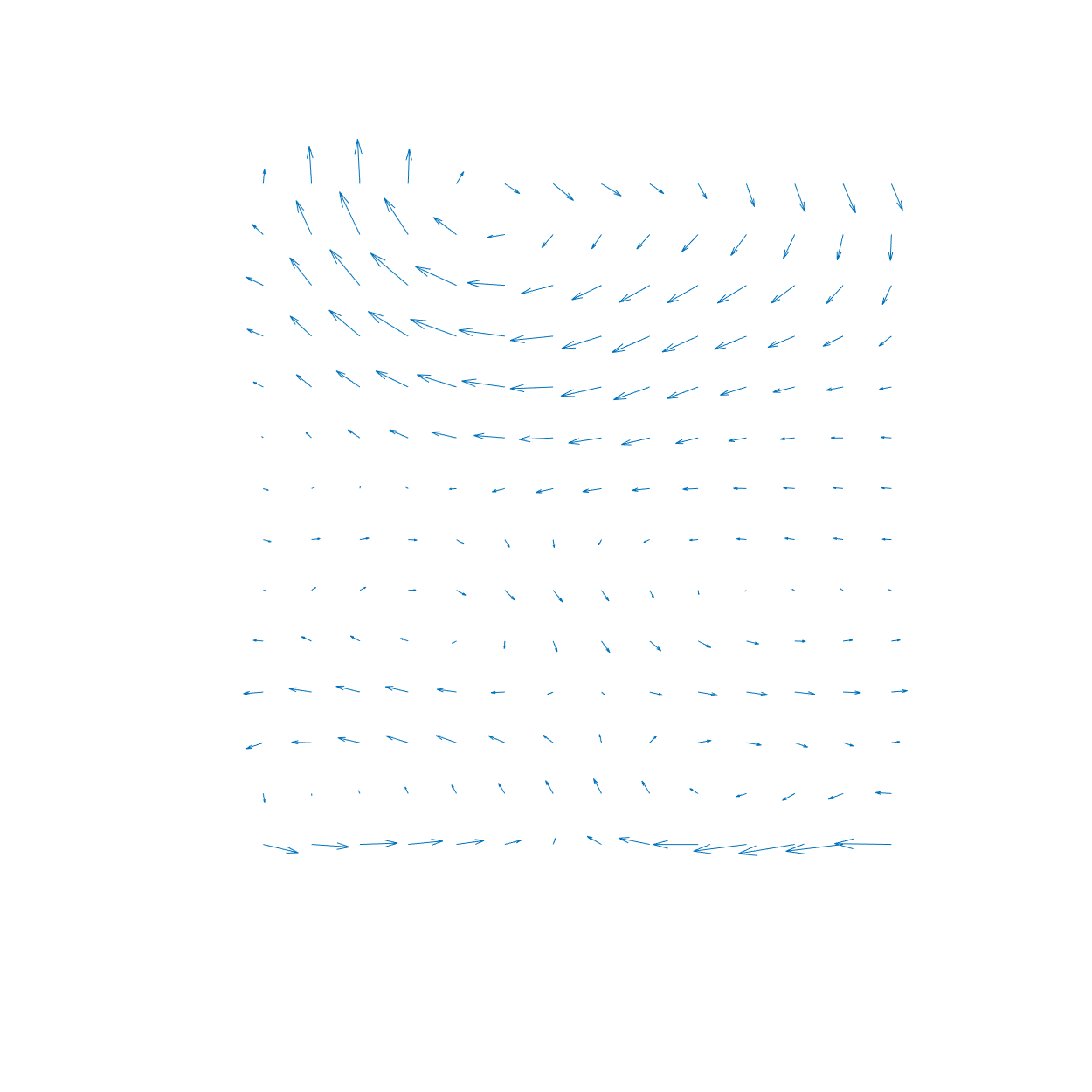

Supplement: S1 MCG raw data 1 — The raw MCG dataset includes categories 0-4 for testing. (ZIP) [file pone.0338189.s001.zip › test/2/p12_245_4.png]

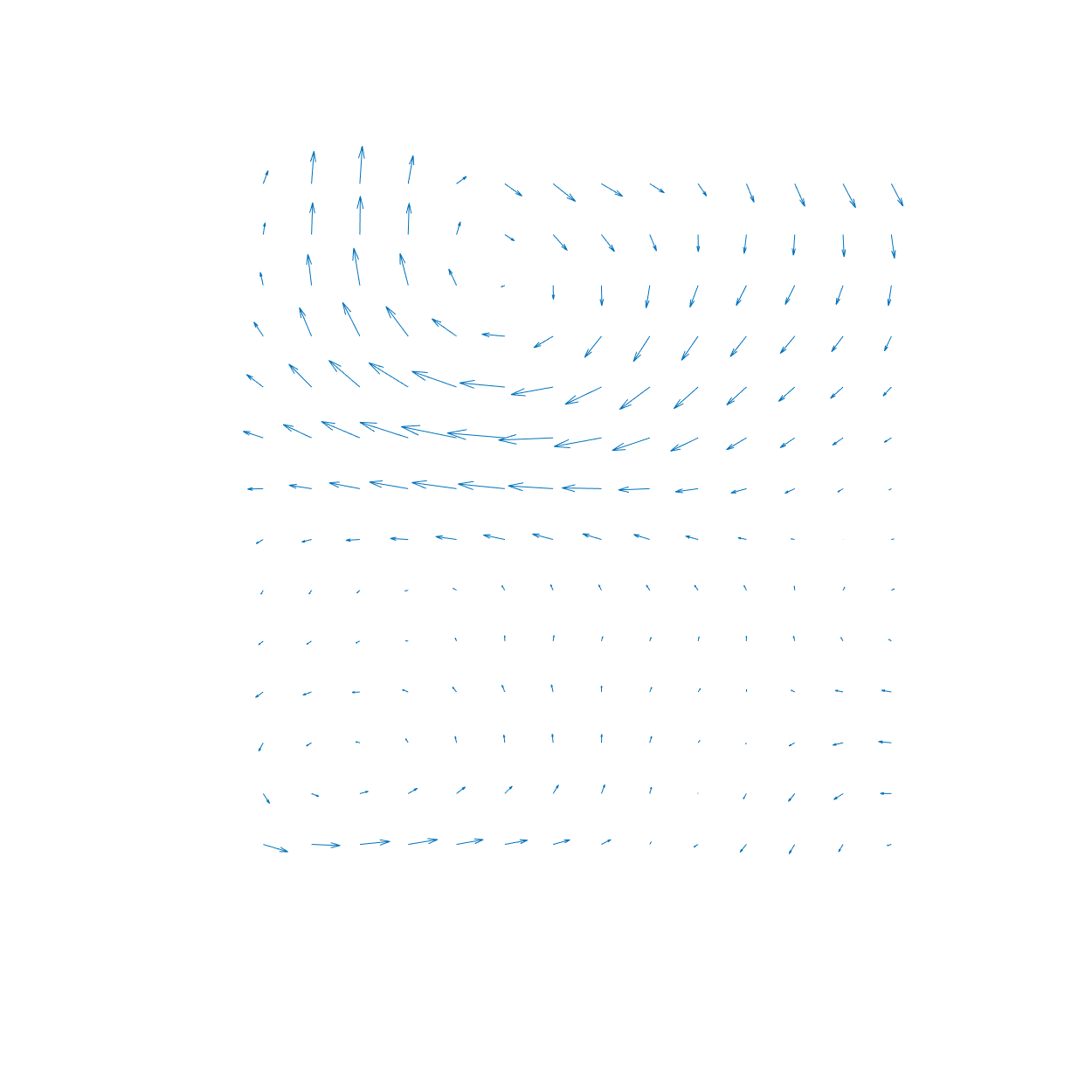

Supplement: S1 MCG raw data 1 — The raw MCG dataset includes categories 0-4 for testing. (ZIP) [file pone.0338189.s001.zip › test/2/p12_250_4.png]

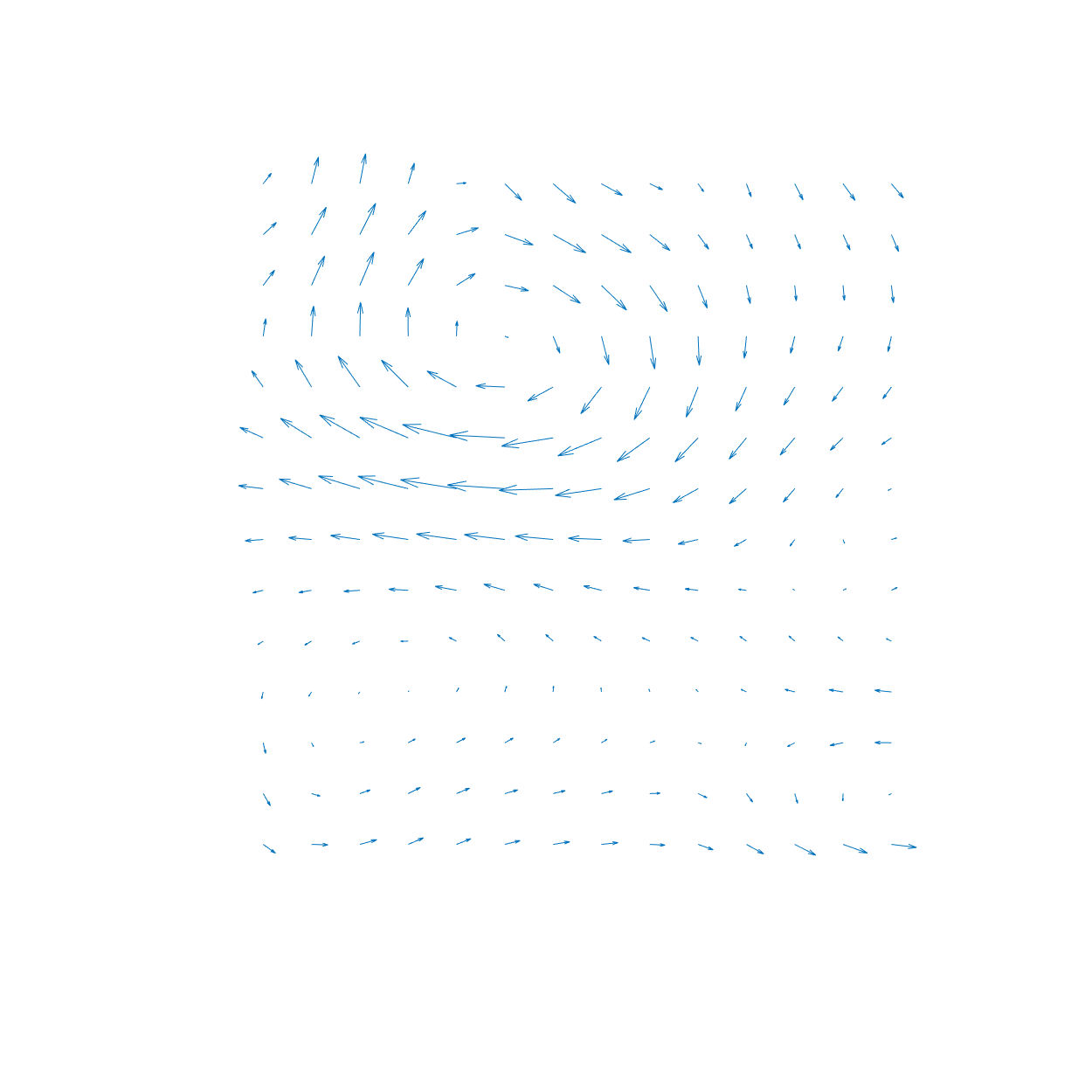

Supplement: S1 MCG raw data 1 — The raw MCG dataset includes categories 0-4 for testing. (ZIP) [file pone.0338189.s001.zip › test/2/p12_255_4.png]

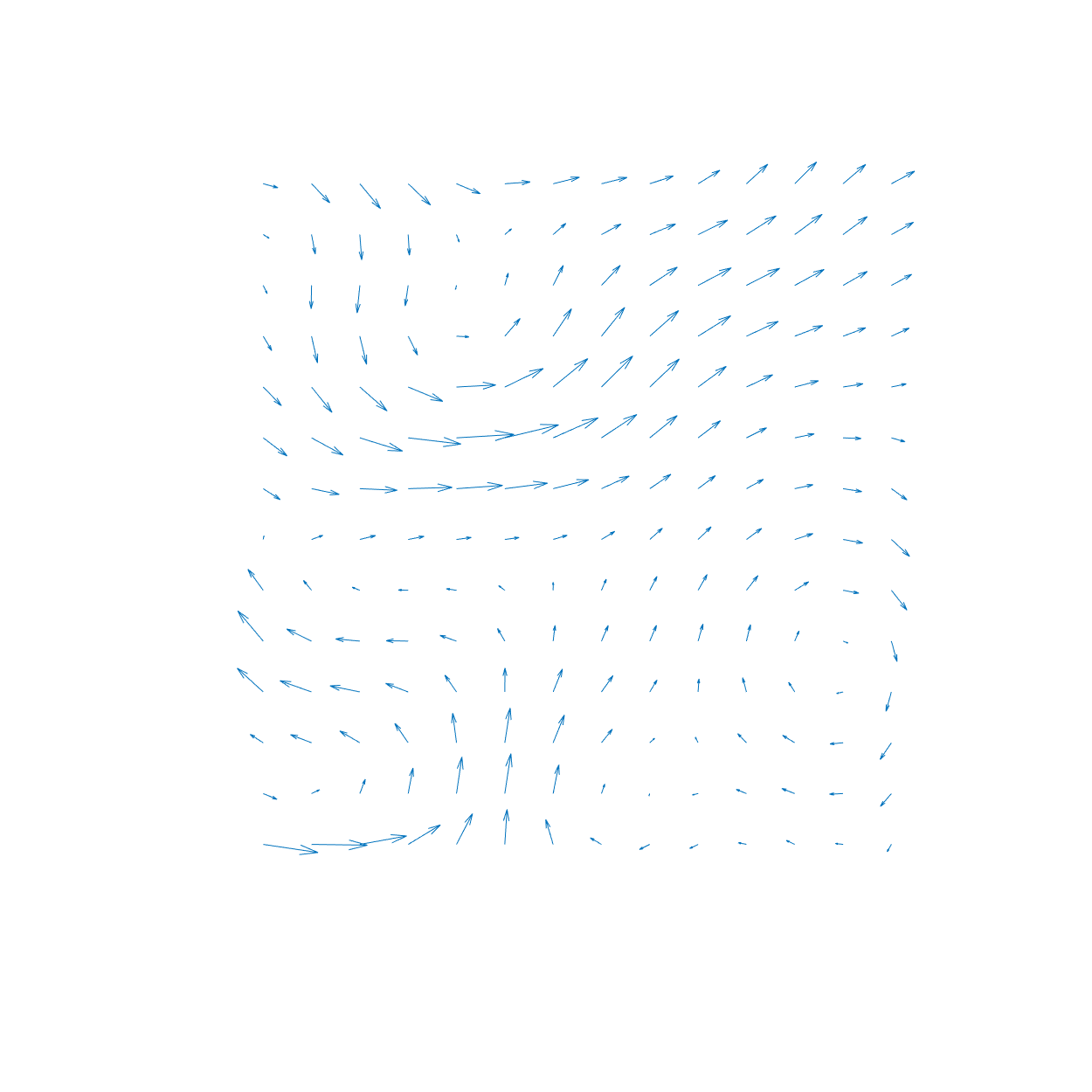

Supplement: S1 MCG raw data 1 — The raw MCG dataset includes categories 0-4 for testing. (ZIP) [file pone.0338189.s001.zip › test/2/p15_385_4.png]

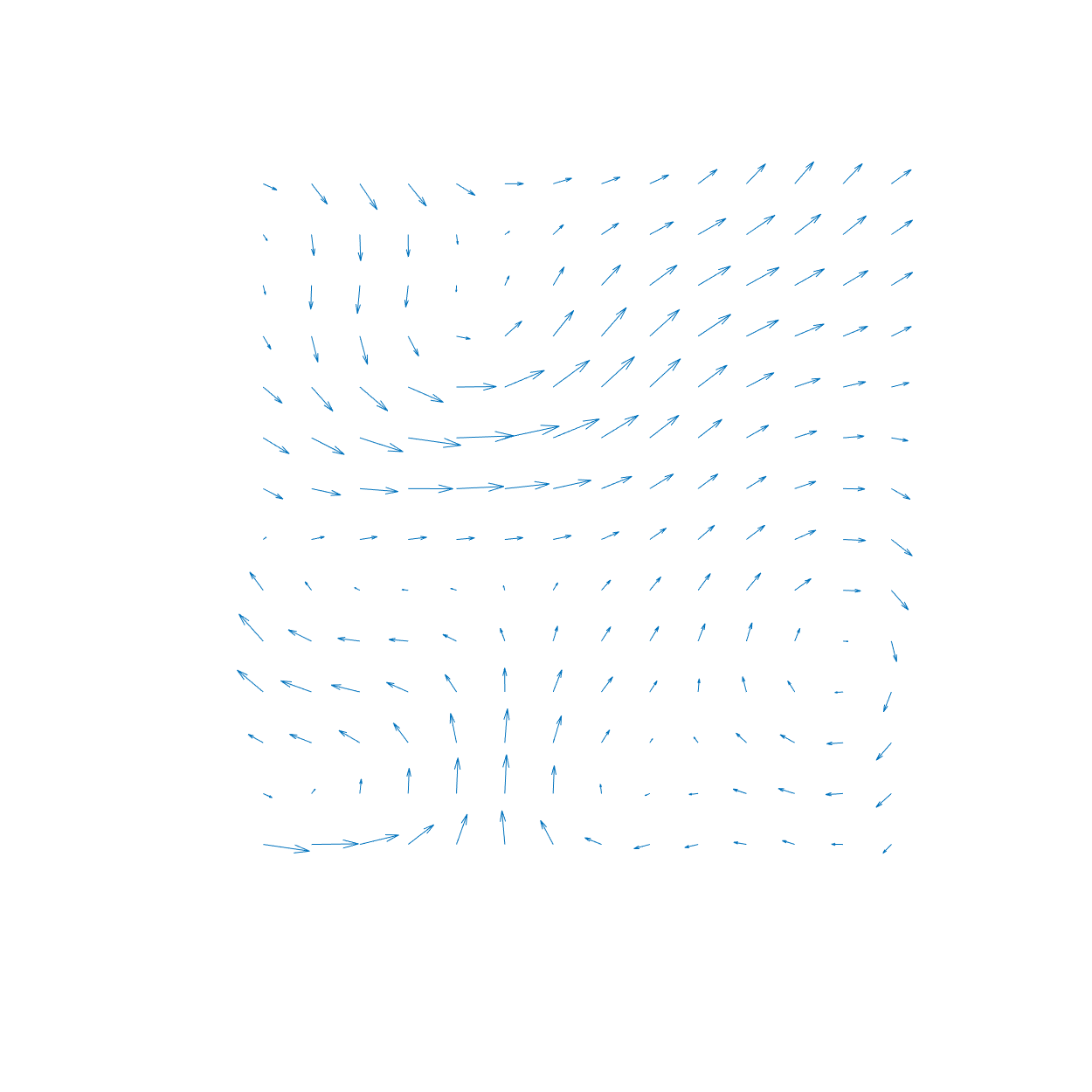

Supplement: S1 MCG raw data 1 — The raw MCG dataset includes categories 0-4 for testing. (ZIP) [file pone.0338189.s001.zip › test/2/p15_390_4.png]

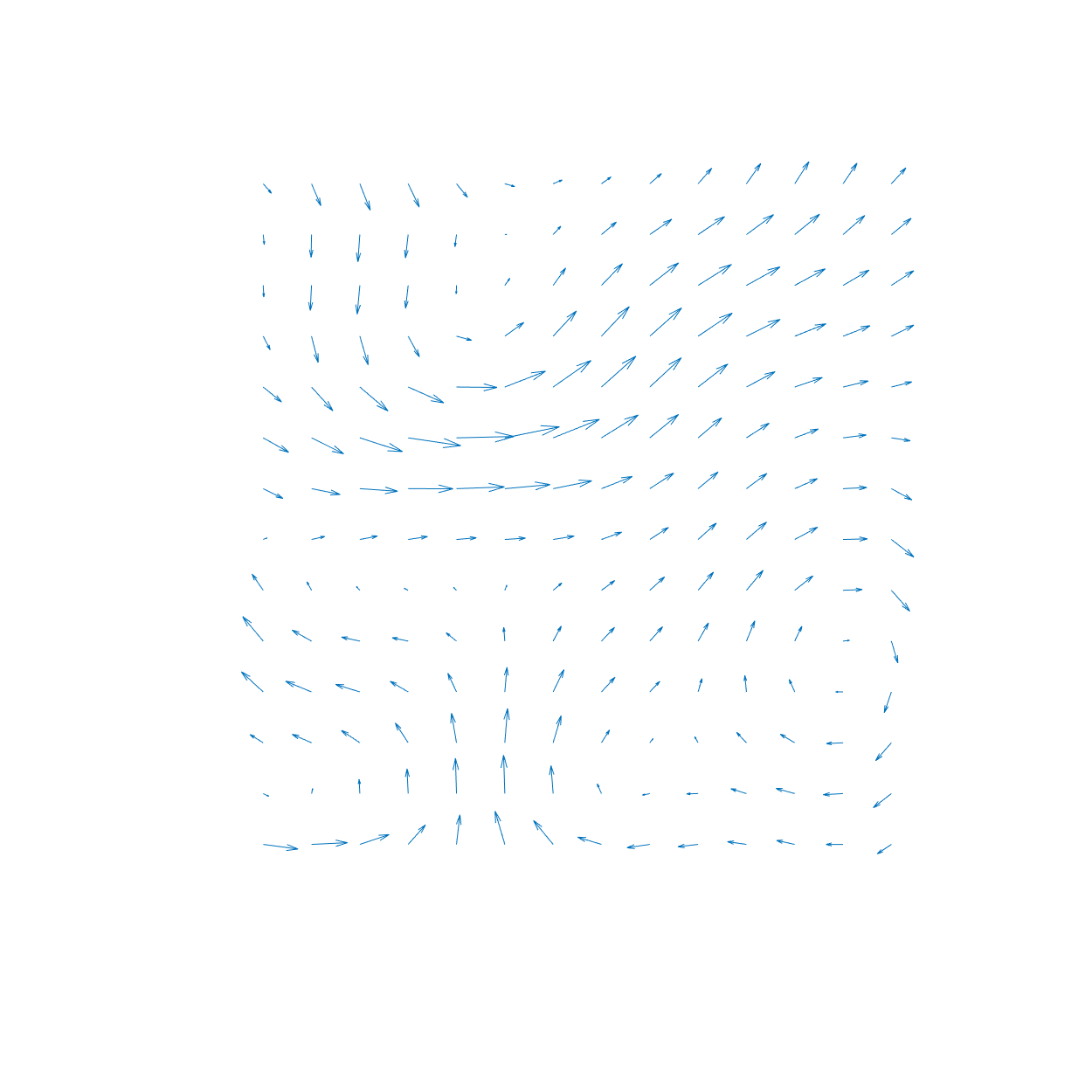

Supplement: S1 MCG raw data 1 — The raw MCG dataset includes categories 0-4 for testing. (ZIP) [file pone.0338189.s001.zip › test/2/p15_395_4.png]

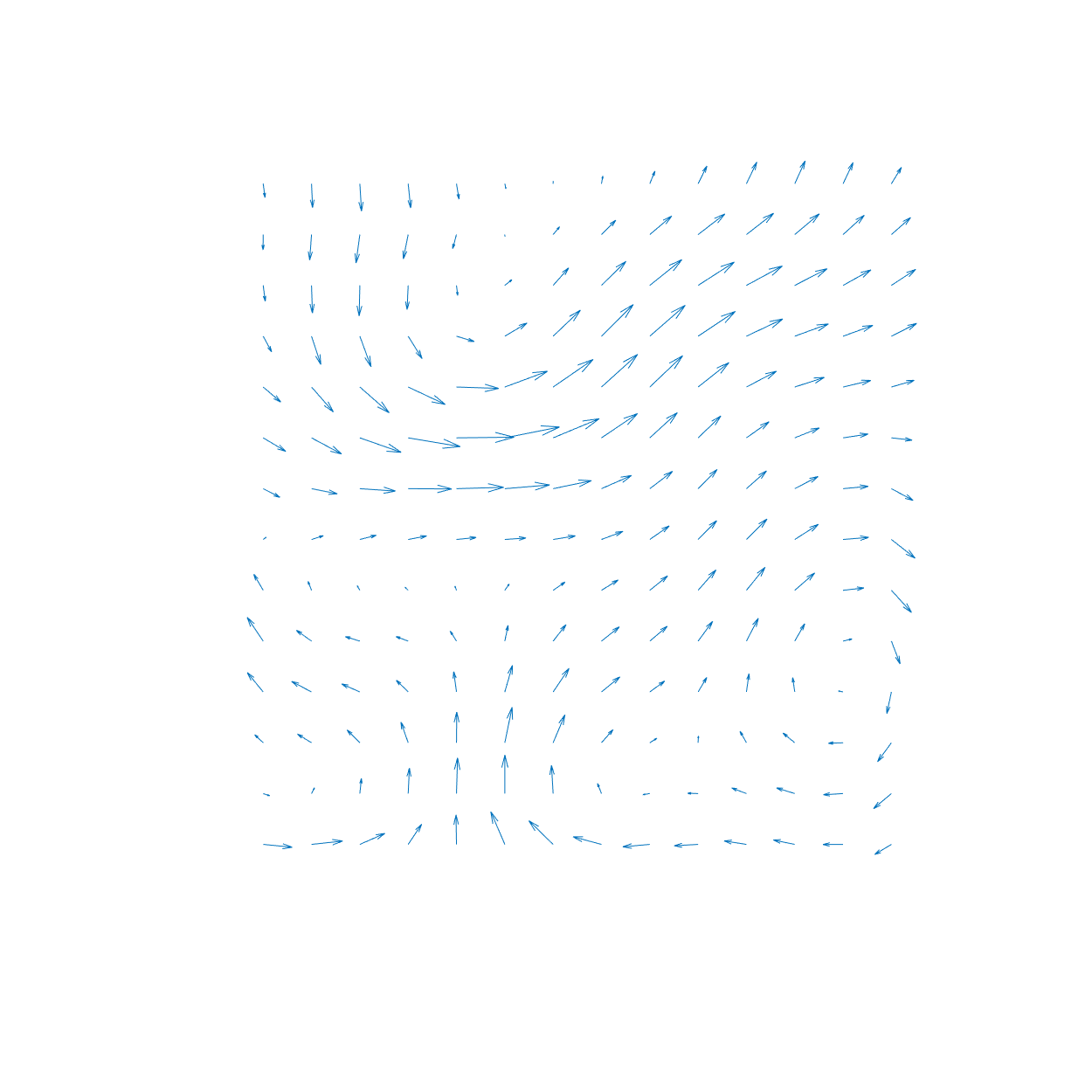

Supplement: S1 MCG raw data 1 — The raw MCG dataset includes categories 0-4 for testing. (ZIP) [file pone.0338189.s001.zip › test/2/p15_400_4.png]

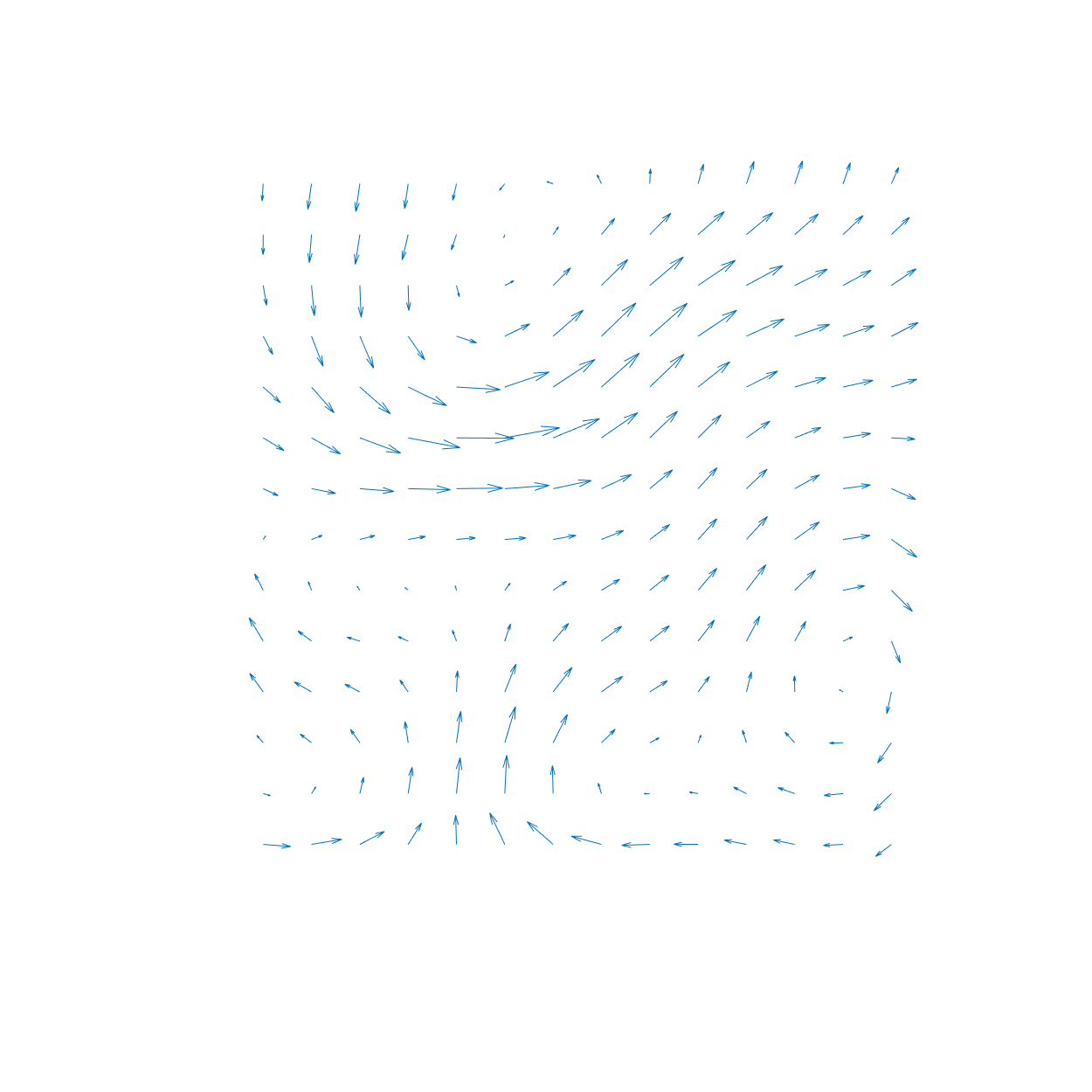

Supplement: S1 MCG raw data 1 — The raw MCG dataset includes categories 0-4 for testing. (ZIP) [file pone.0338189.s001.zip › test/2/p15_405_4.png]

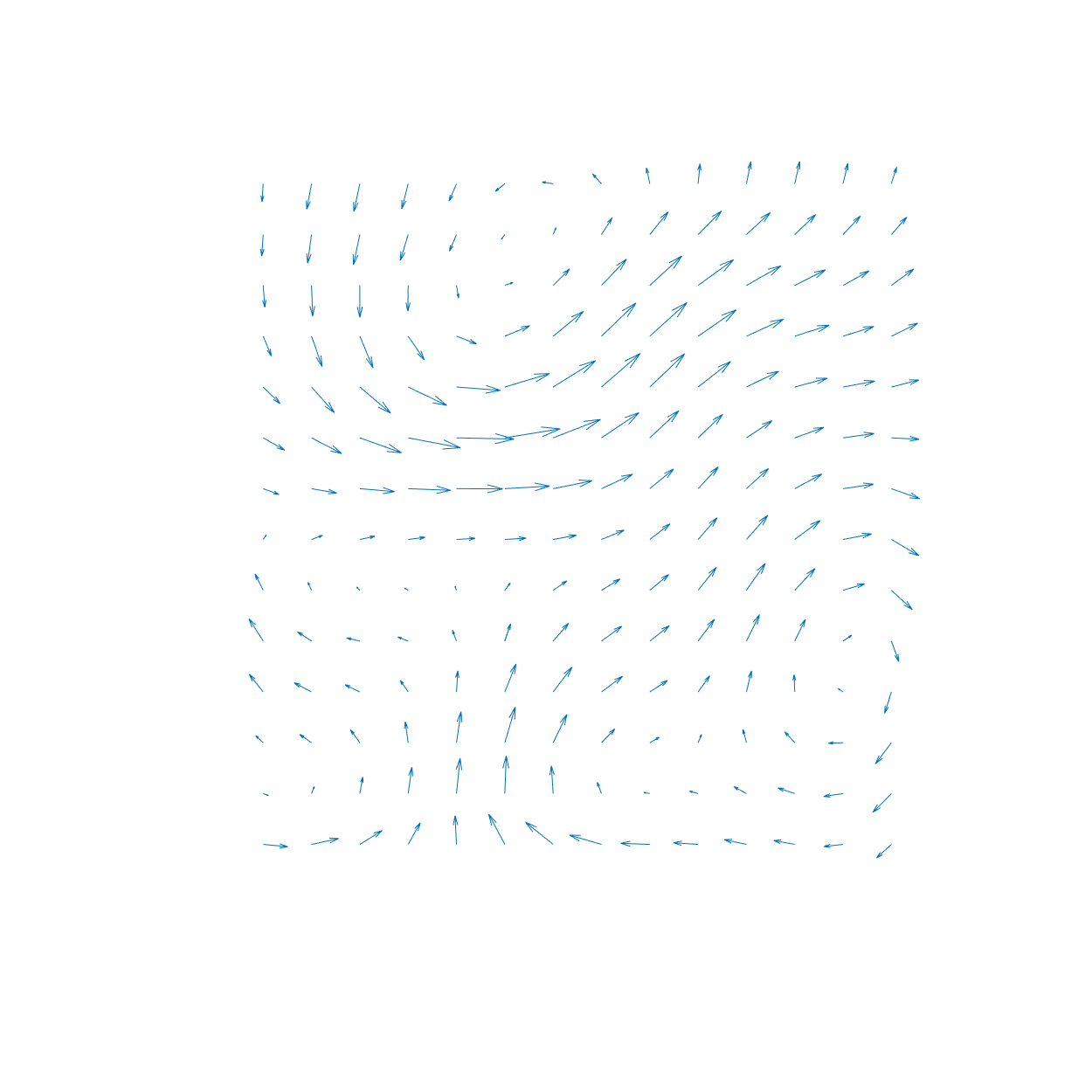

Supplement: S1 MCG raw data 1 — The raw MCG dataset includes categories 0-4 for testing. (ZIP) [file pone.0338189.s001.zip › test/2/p15_410_4.png]

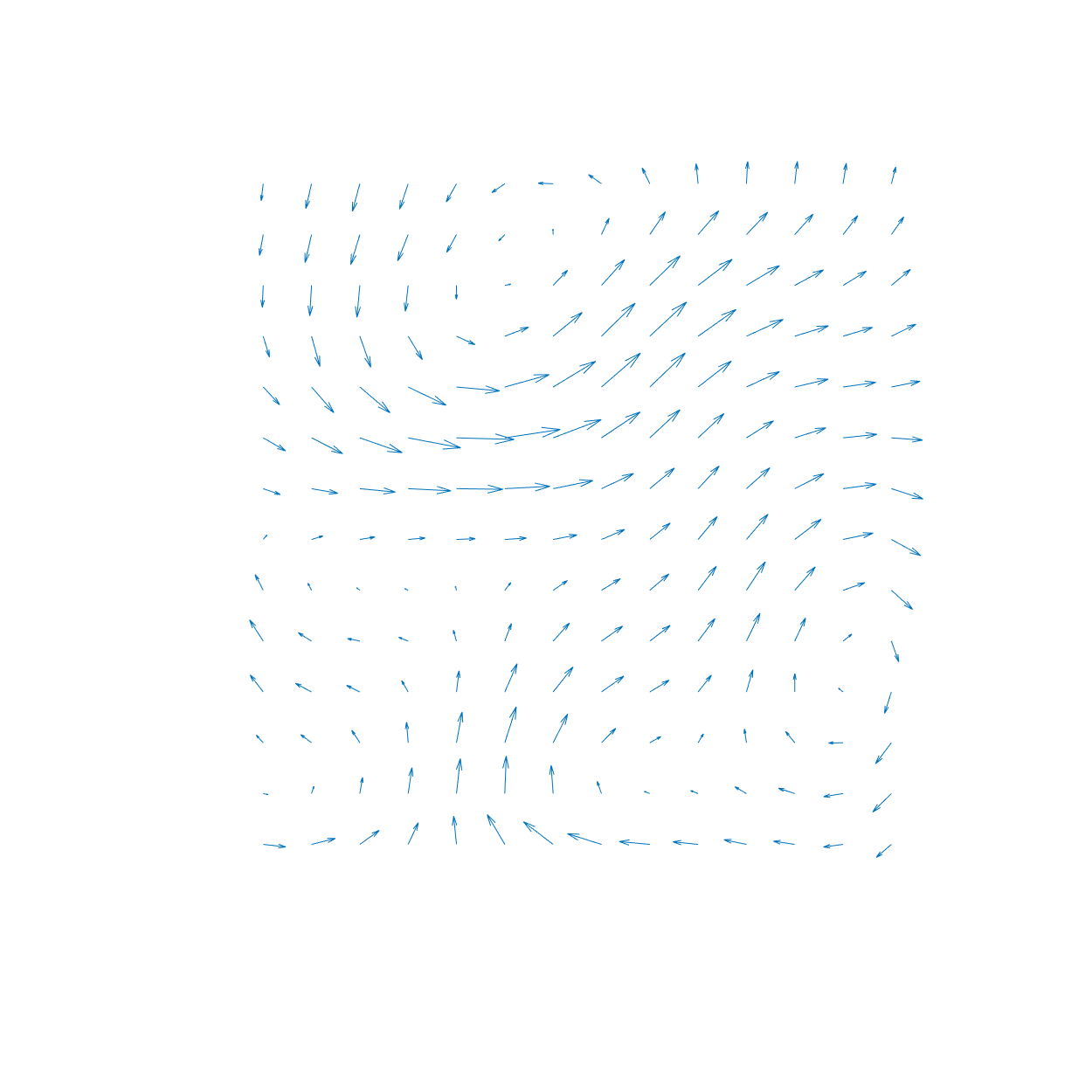

Supplement: S1 MCG raw data 1 — The raw MCG dataset includes categories 0-4 for testing. (ZIP) [file pone.0338189.s001.zip › test/2/p15_415_4.png]

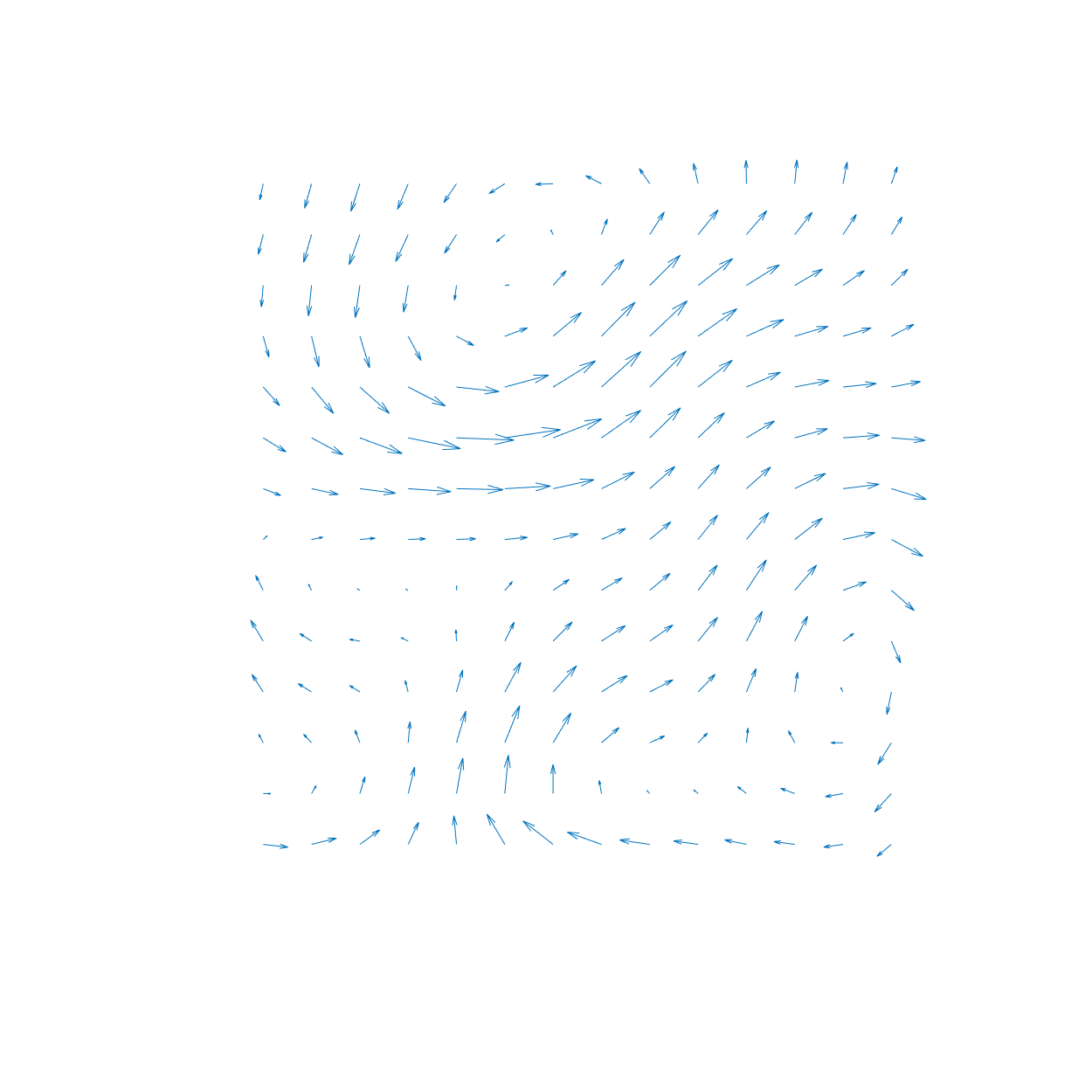

Supplement: S1 MCG raw data 1 — The raw MCG dataset includes categories 0-4 for testing. (ZIP) [file pone.0338189.s001.zip › test/2/p15_420_4.png]

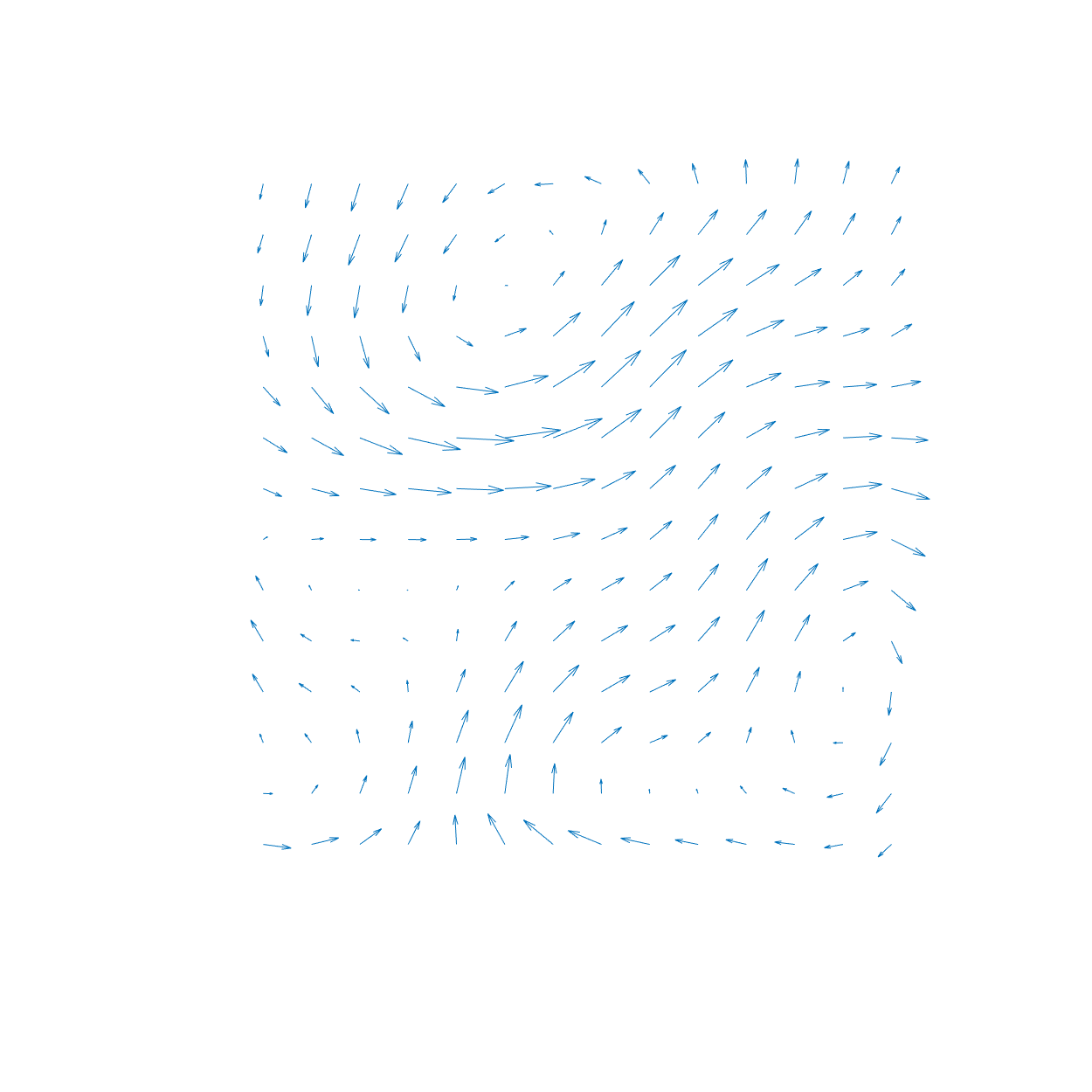

Supplement: S1 MCG raw data 1 — The raw MCG dataset includes categories 0-4 for testing. (ZIP) [file pone.0338189.s001.zip › test/2/p15_425_4.png]

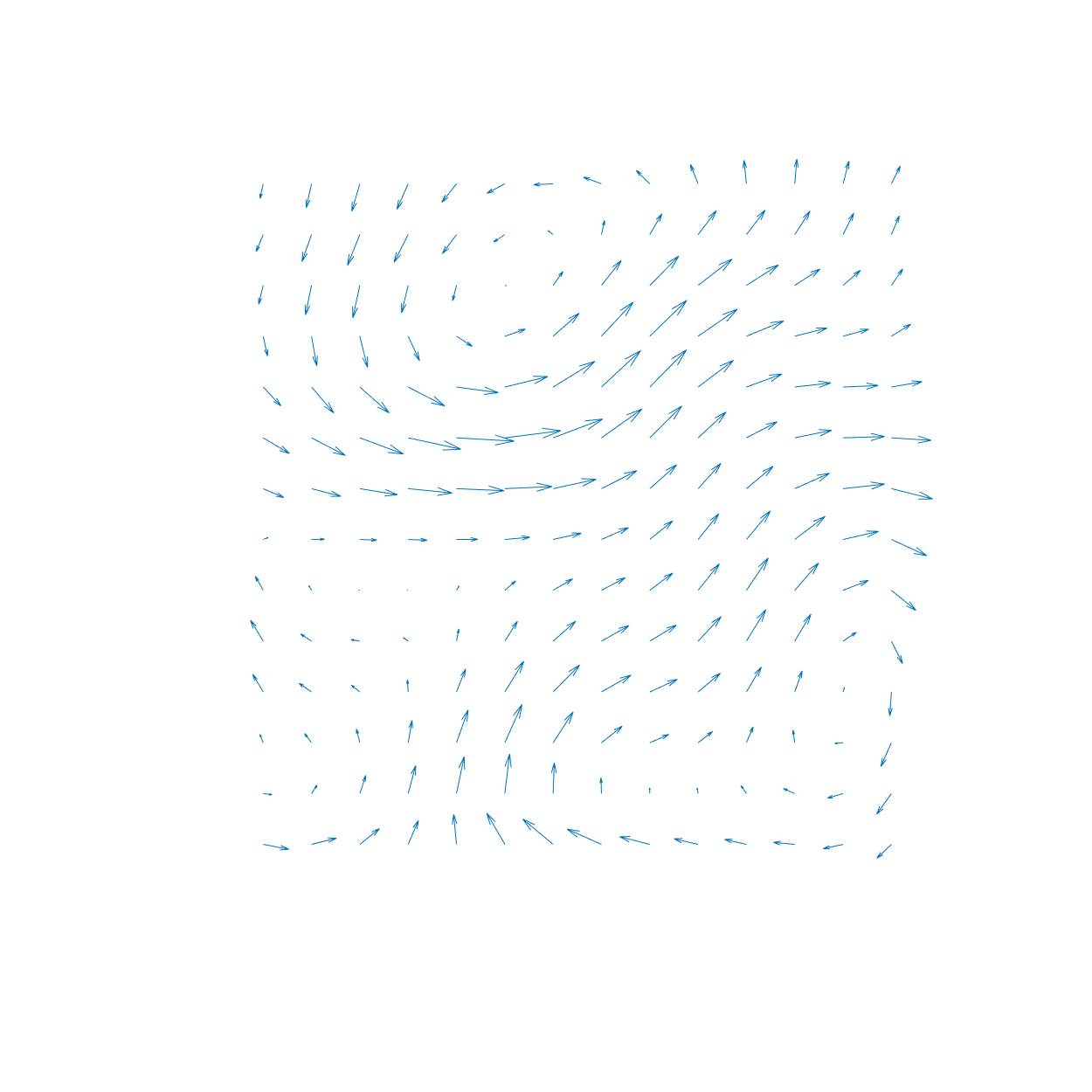

Supplement: S1 MCG raw data 1 — The raw MCG dataset includes categories 0-4 for testing. (ZIP) [file pone.0338189.s001.zip › test/2/p15_430_4.png]

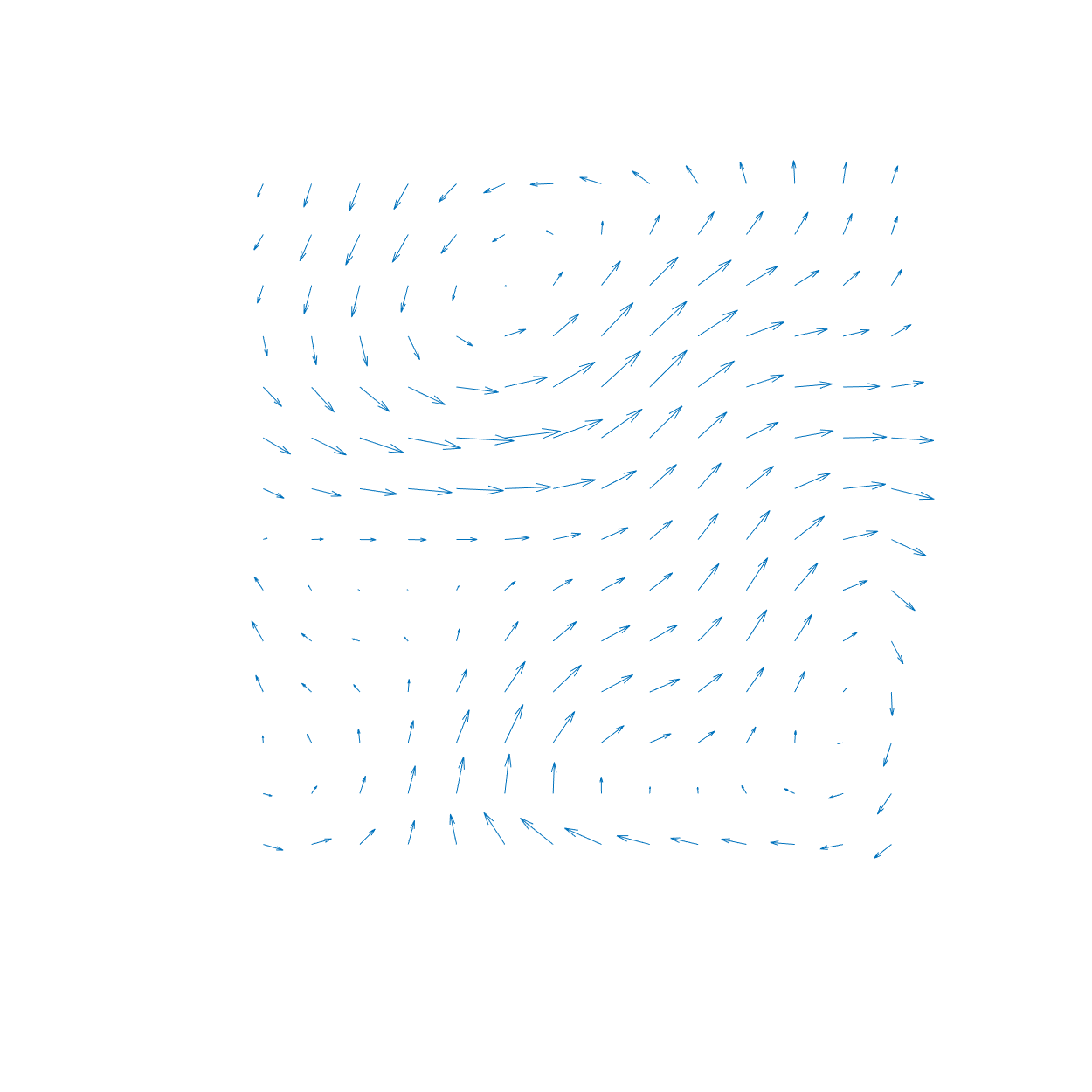

Supplement: S1 MCG raw data 1 — The raw MCG dataset includes categories 0-4 for testing. (ZIP) [file pone.0338189.s001.zip › test/2/p15_435_4.png]

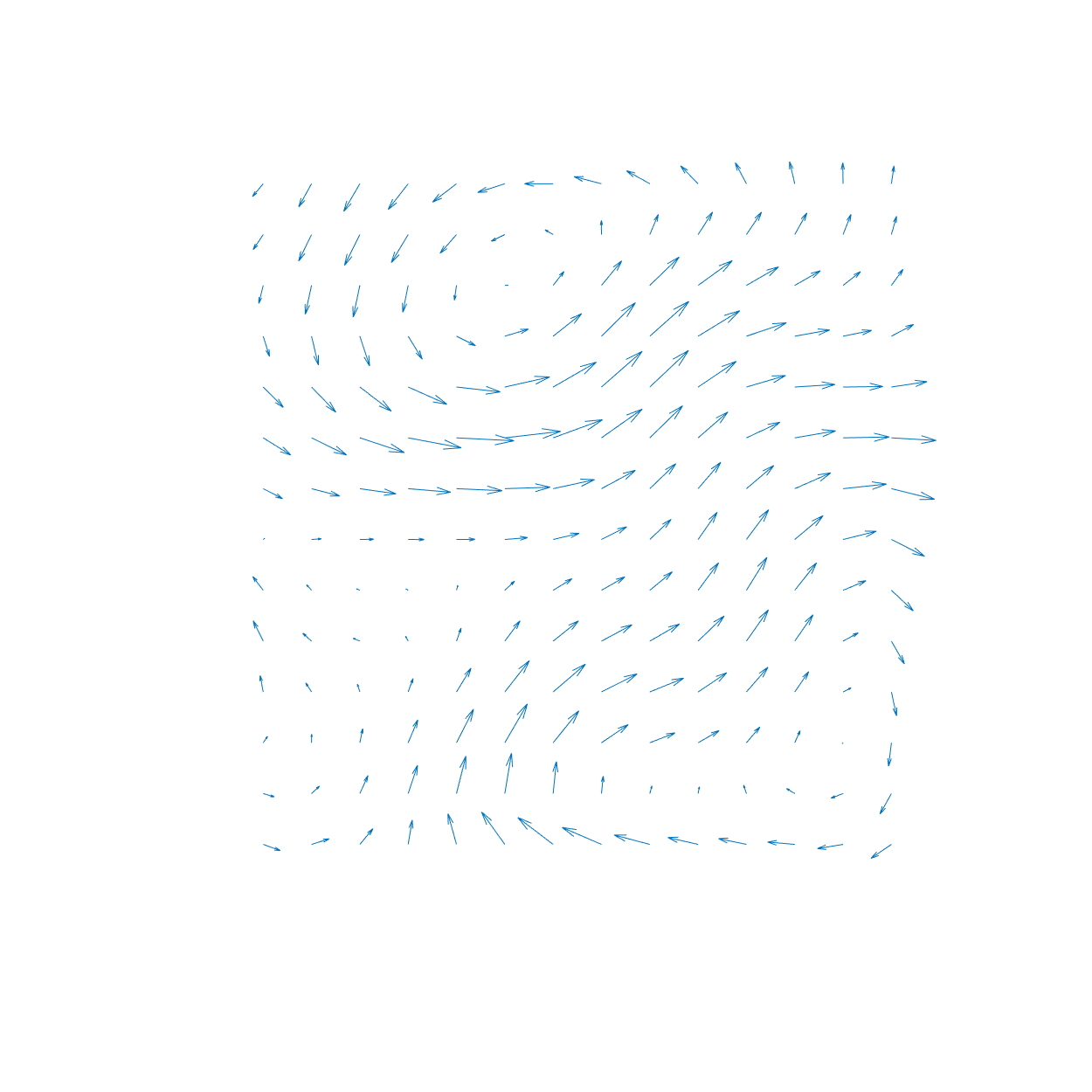

Supplement: S1 MCG raw data 1 — The raw MCG dataset includes categories 0-4 for testing. (ZIP) [file pone.0338189.s001.zip › test/2/p15_440_4.png]

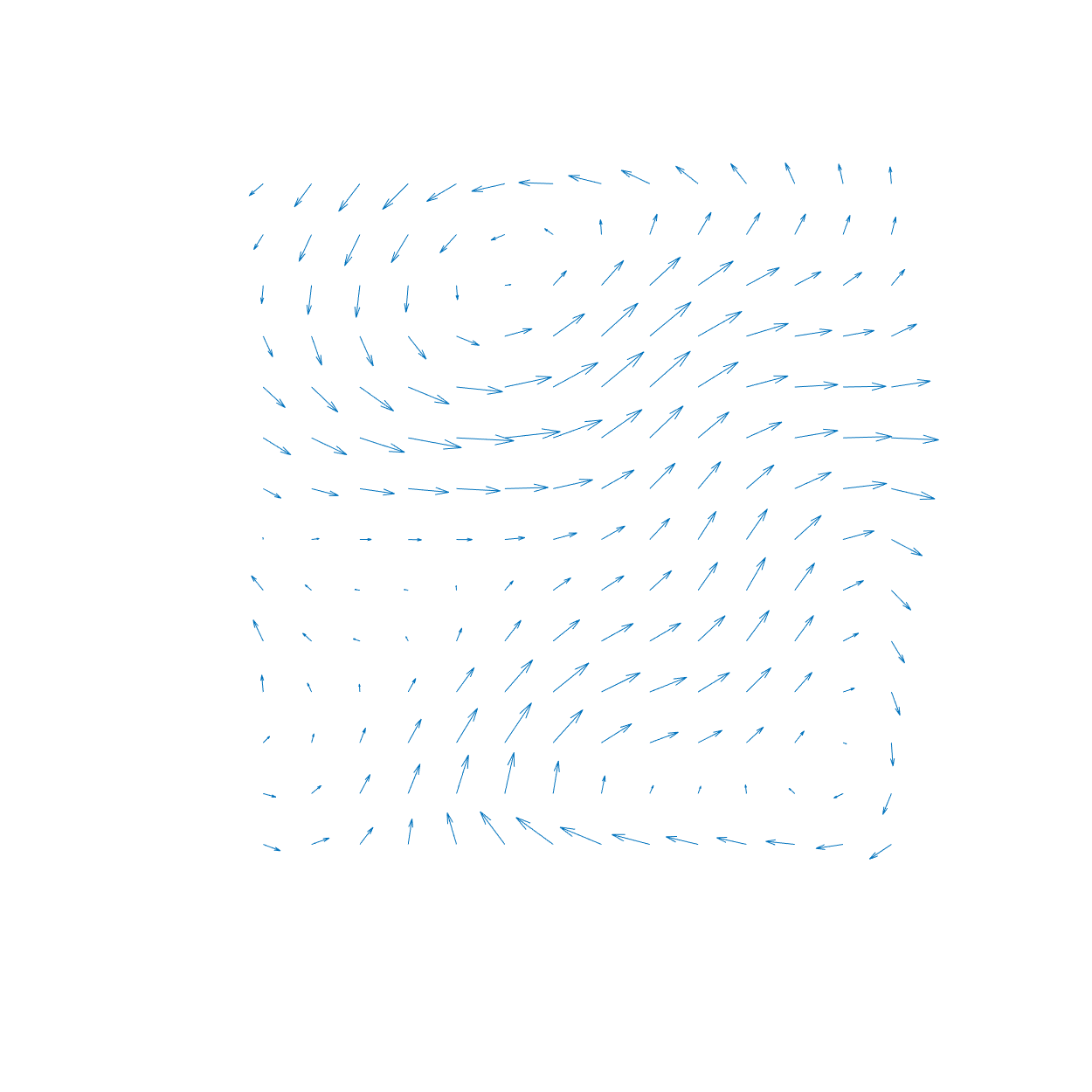

Supplement: S1 MCG raw data 1 — The raw MCG dataset includes categories 0-4 for testing. (ZIP) [file pone.0338189.s001.zip › test/2/p15_445_4.png]

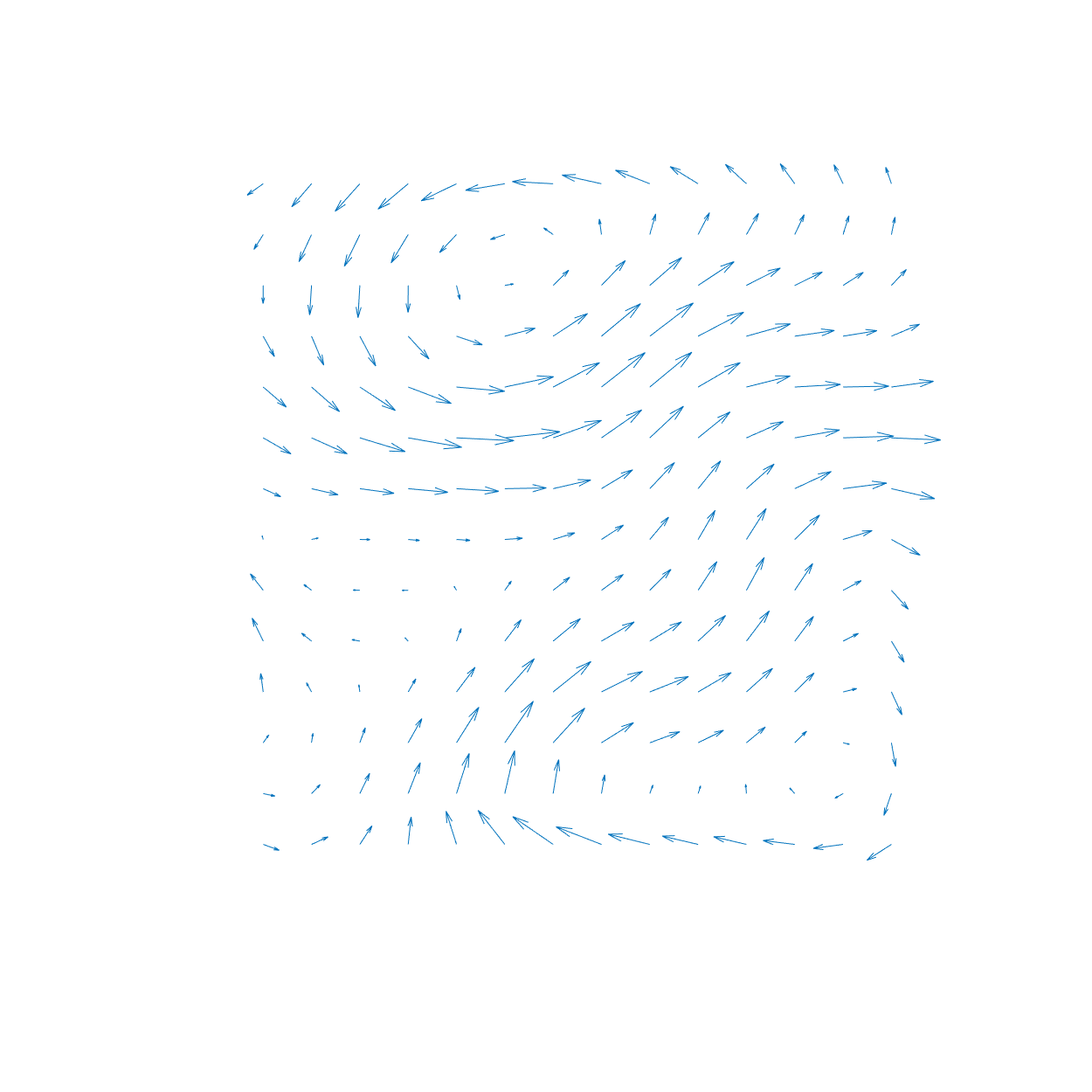

Supplement: S1 MCG raw data 1 — The raw MCG dataset includes categories 0-4 for testing. (ZIP) [file pone.0338189.s001.zip › test/2/p15_450_4.png]

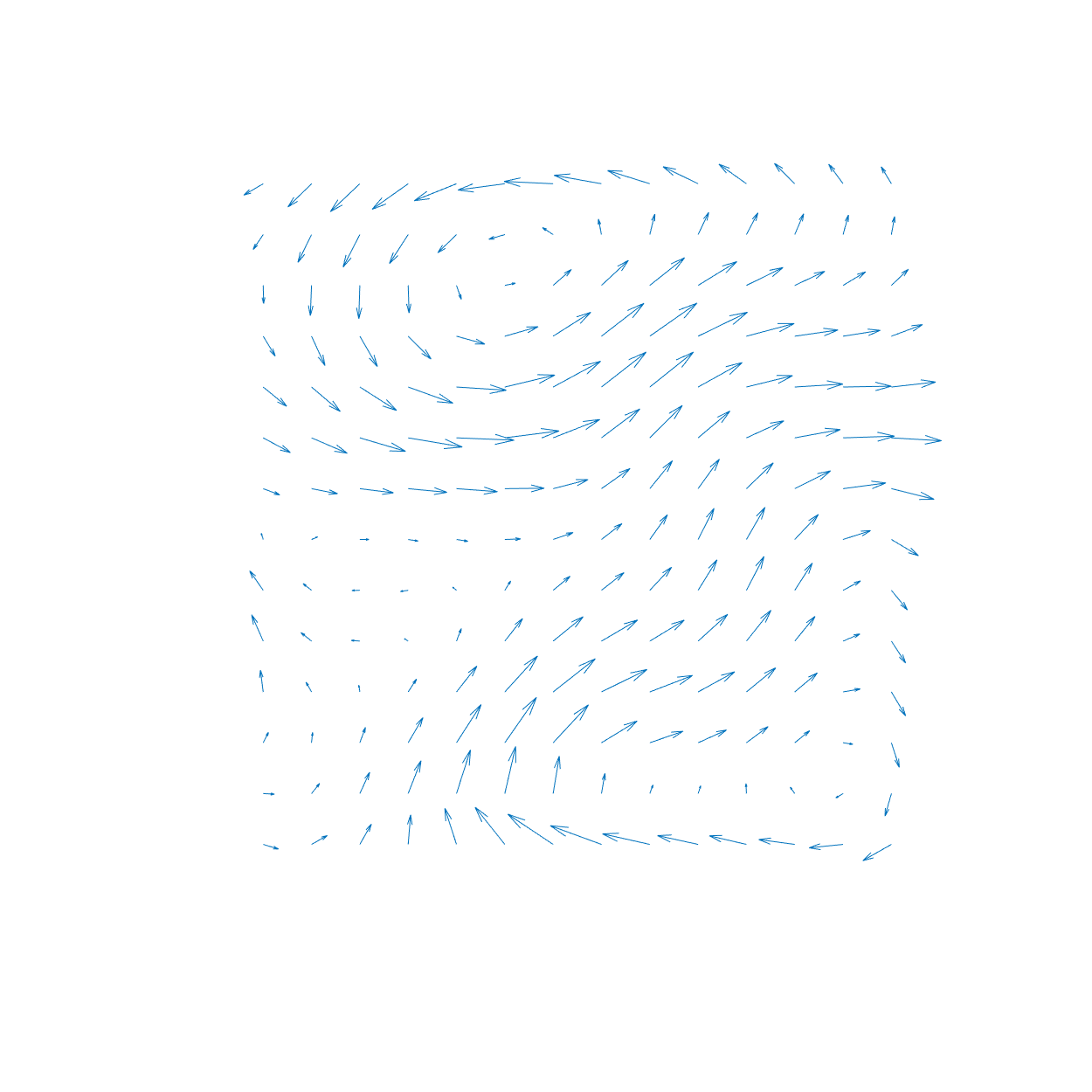

Supplement: S1 MCG raw data 1 — The raw MCG dataset includes categories 0-4 for testing. (ZIP) [file pone.0338189.s001.zip › test/2/p15_455_4.png]

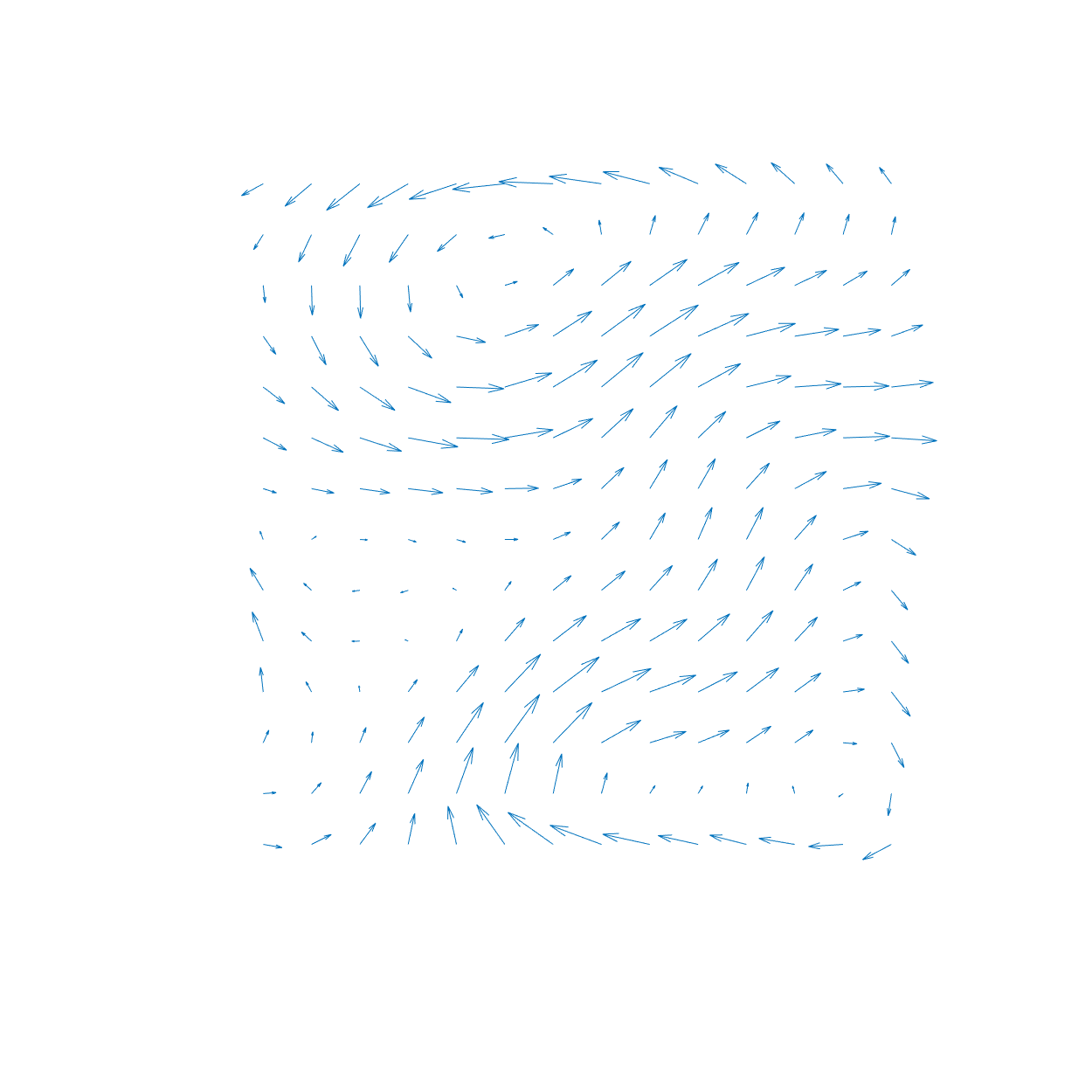

Supplement: S1 MCG raw data 1 — The raw MCG dataset includes categories 0-4 for testing. (ZIP) [file pone.0338189.s001.zip › test/2/p15_460_4.png]

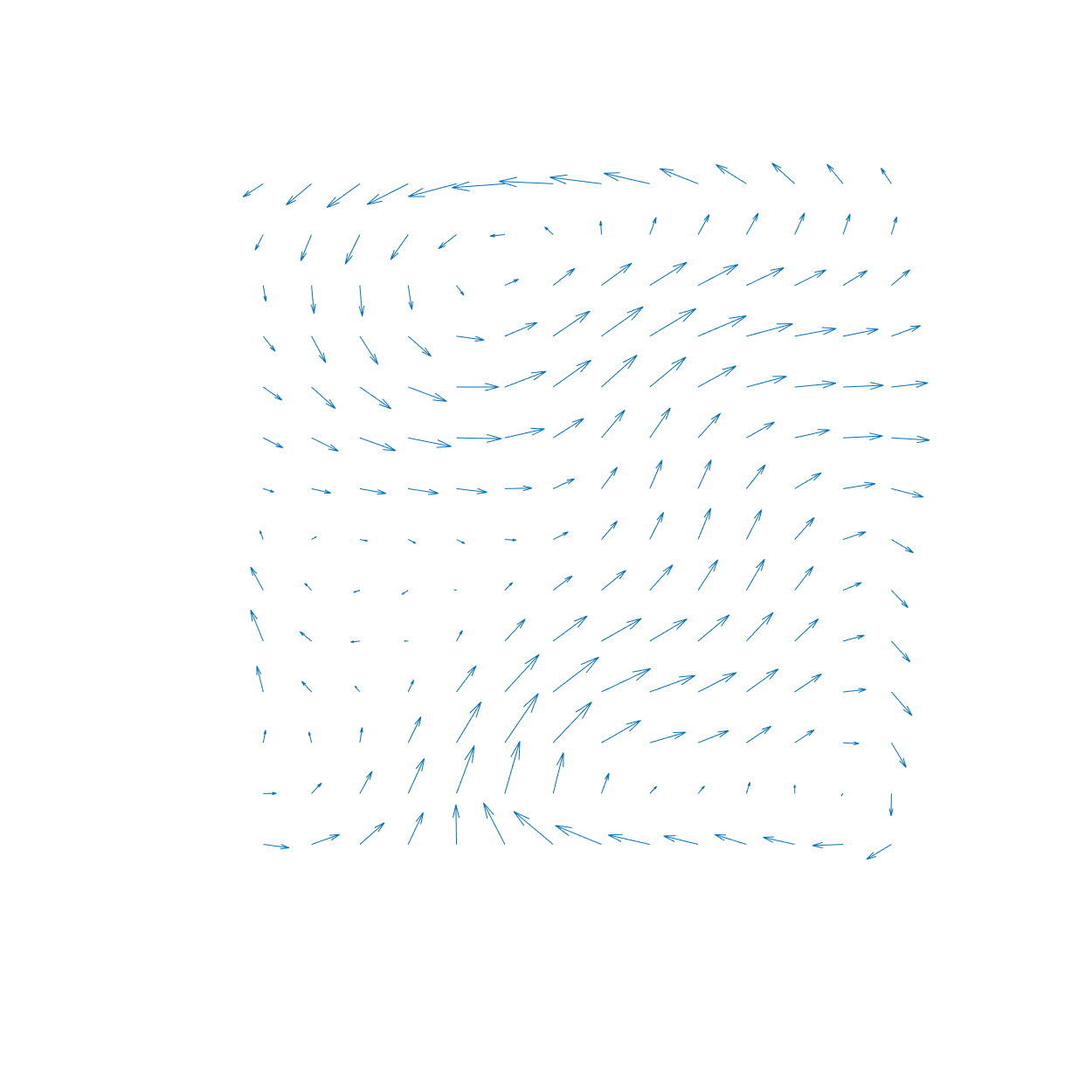

Supplement: S1 MCG raw data 1 — The raw MCG dataset includes categories 0-4 for testing. (ZIP) [file pone.0338189.s001.zip › test/2/p15_465_4.png]

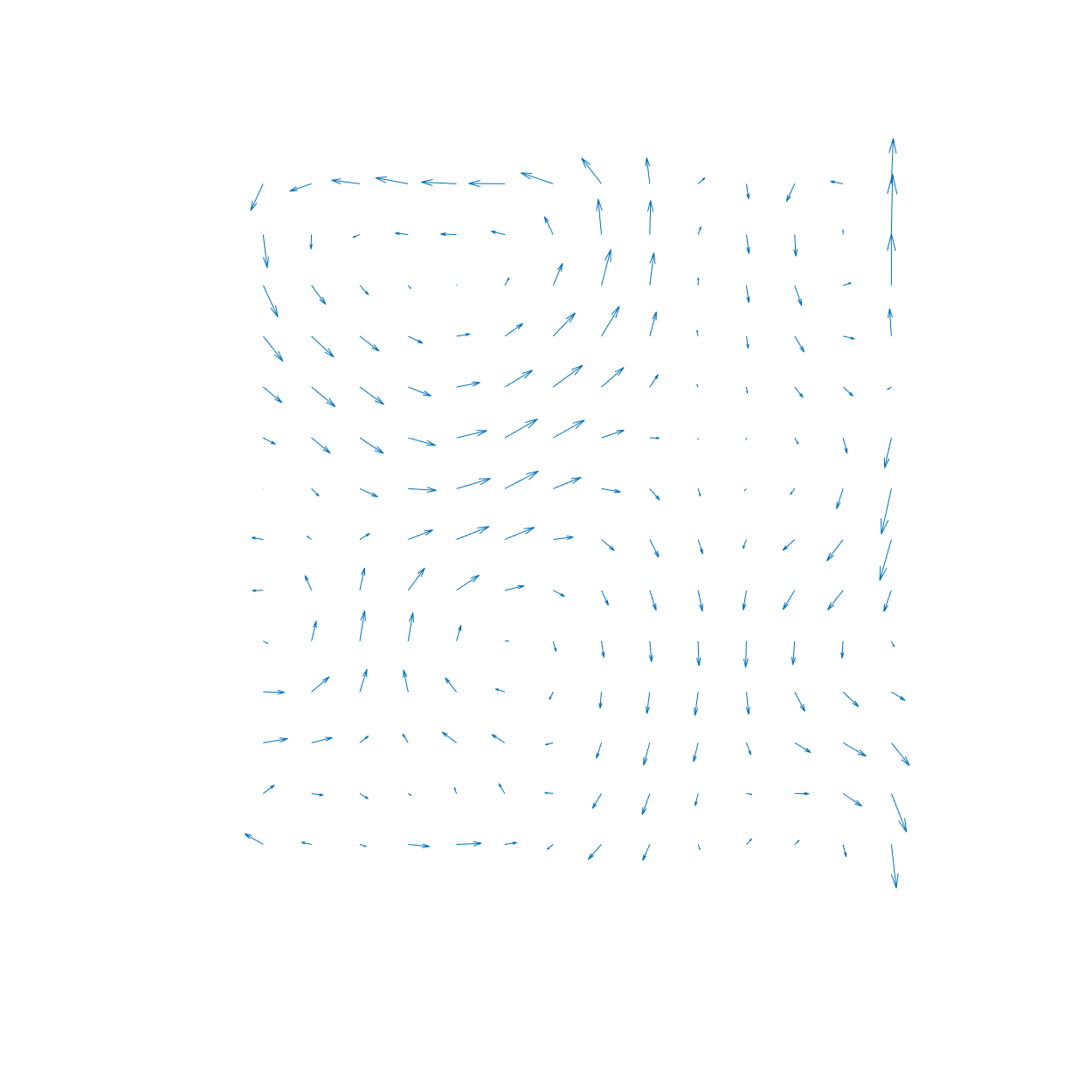

Supplement: S1 MCG raw data 1 — The raw MCG dataset includes categories 0-4 for testing. (ZIP) [file pone.0338189.s001.zip › test/2/p1_400_4.png]

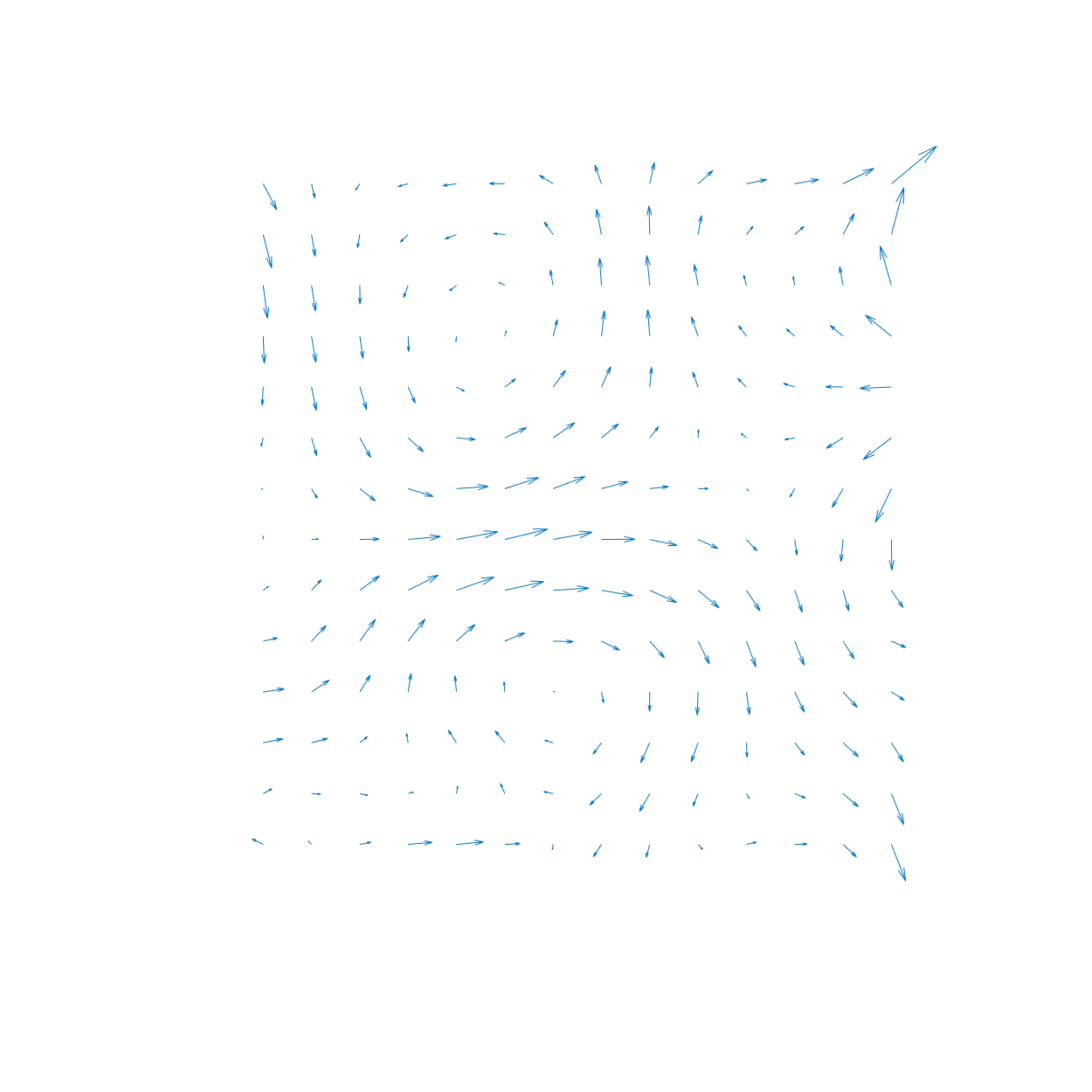

Supplement: S1 MCG raw data 1 — The raw MCG dataset includes categories 0-4 for testing. (ZIP) [file pone.0338189.s001.zip › test/2/p1_405_4.png]

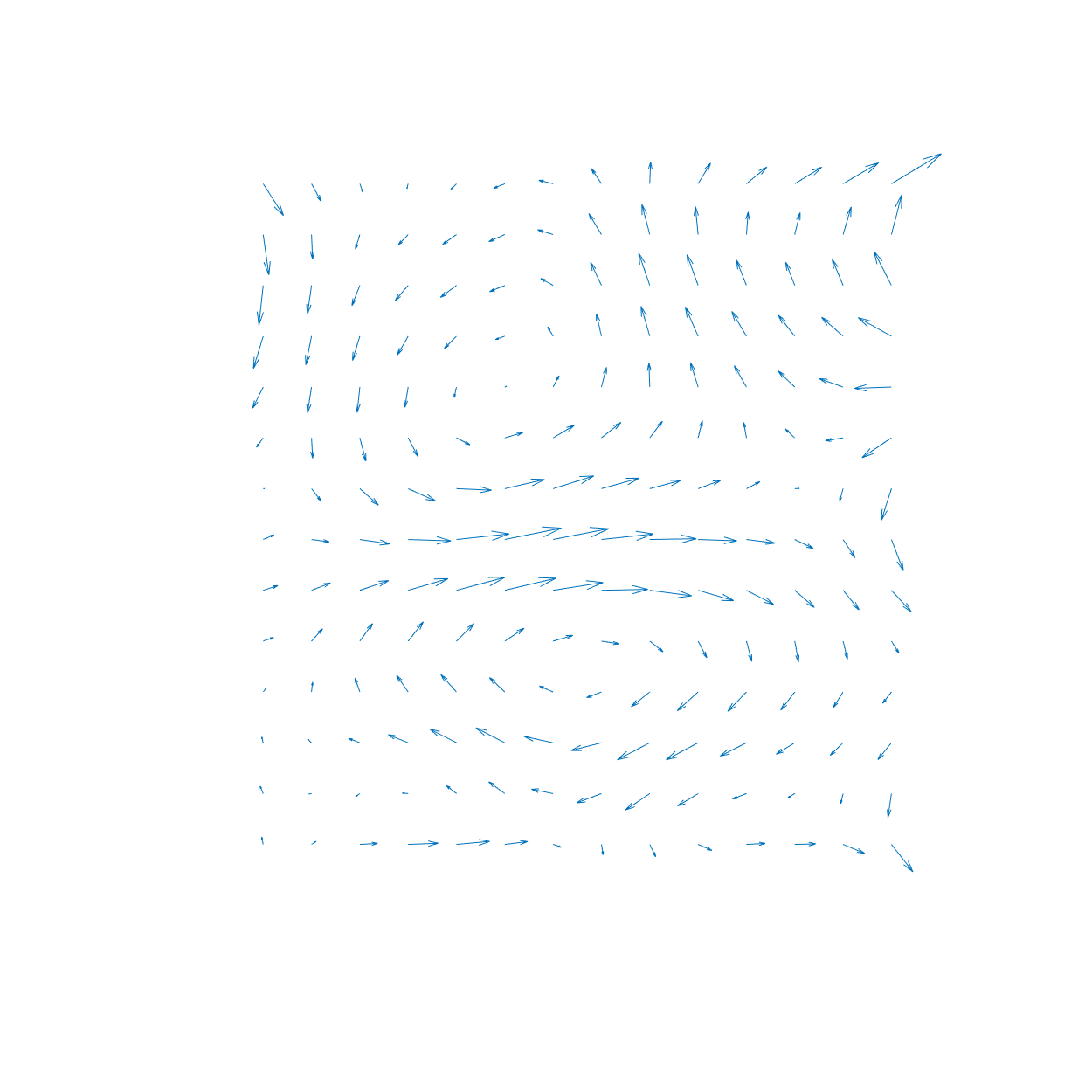

Supplement: S1 MCG raw data 1 — The raw MCG dataset includes categories 0-4 for testing. (ZIP) [file pone.0338189.s001.zip › test/2/p1_410_4.png]

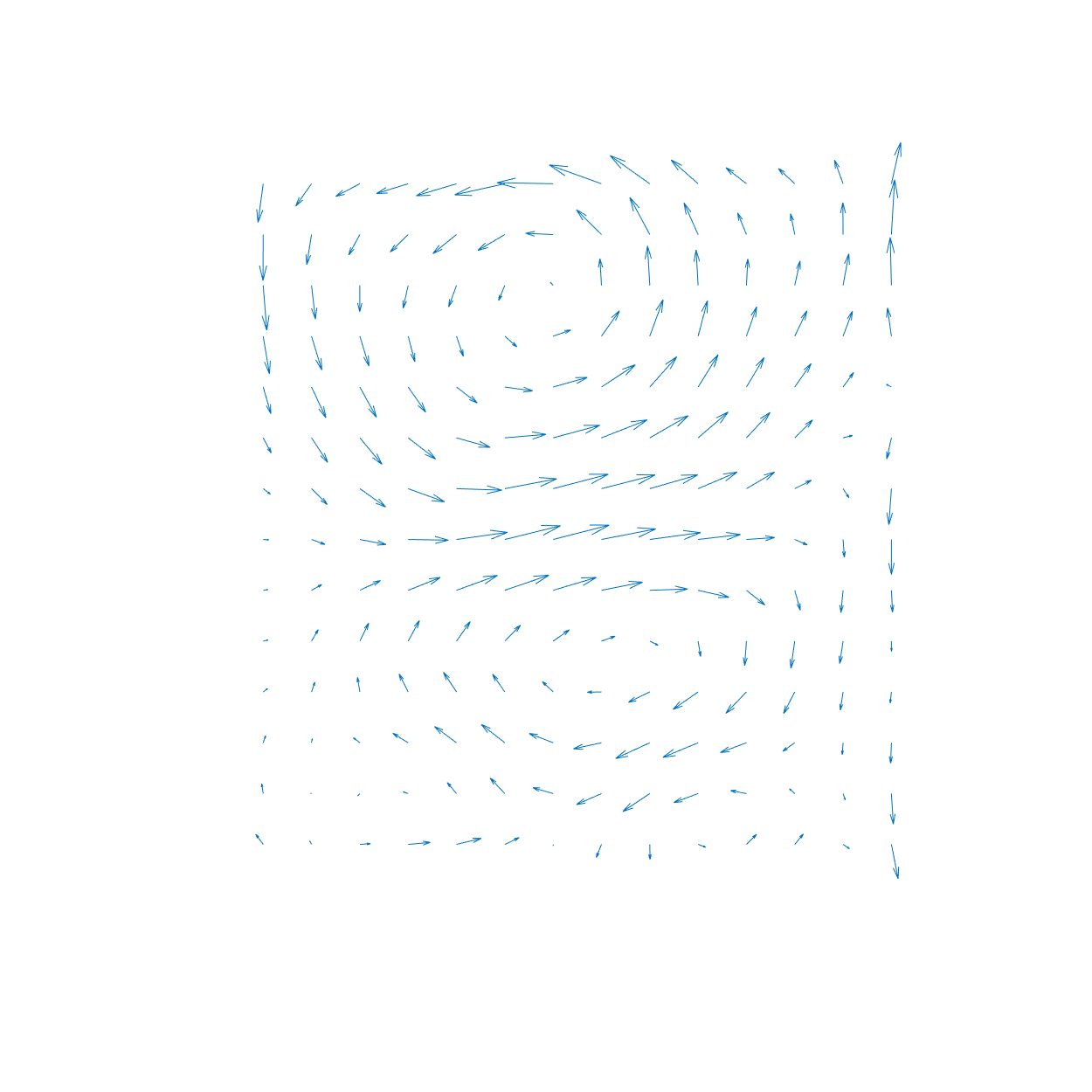

Supplement: S1 MCG raw data 1 — The raw MCG dataset includes categories 0-4 for testing. (ZIP) [file pone.0338189.s001.zip › test/2/p1_415_4.png]

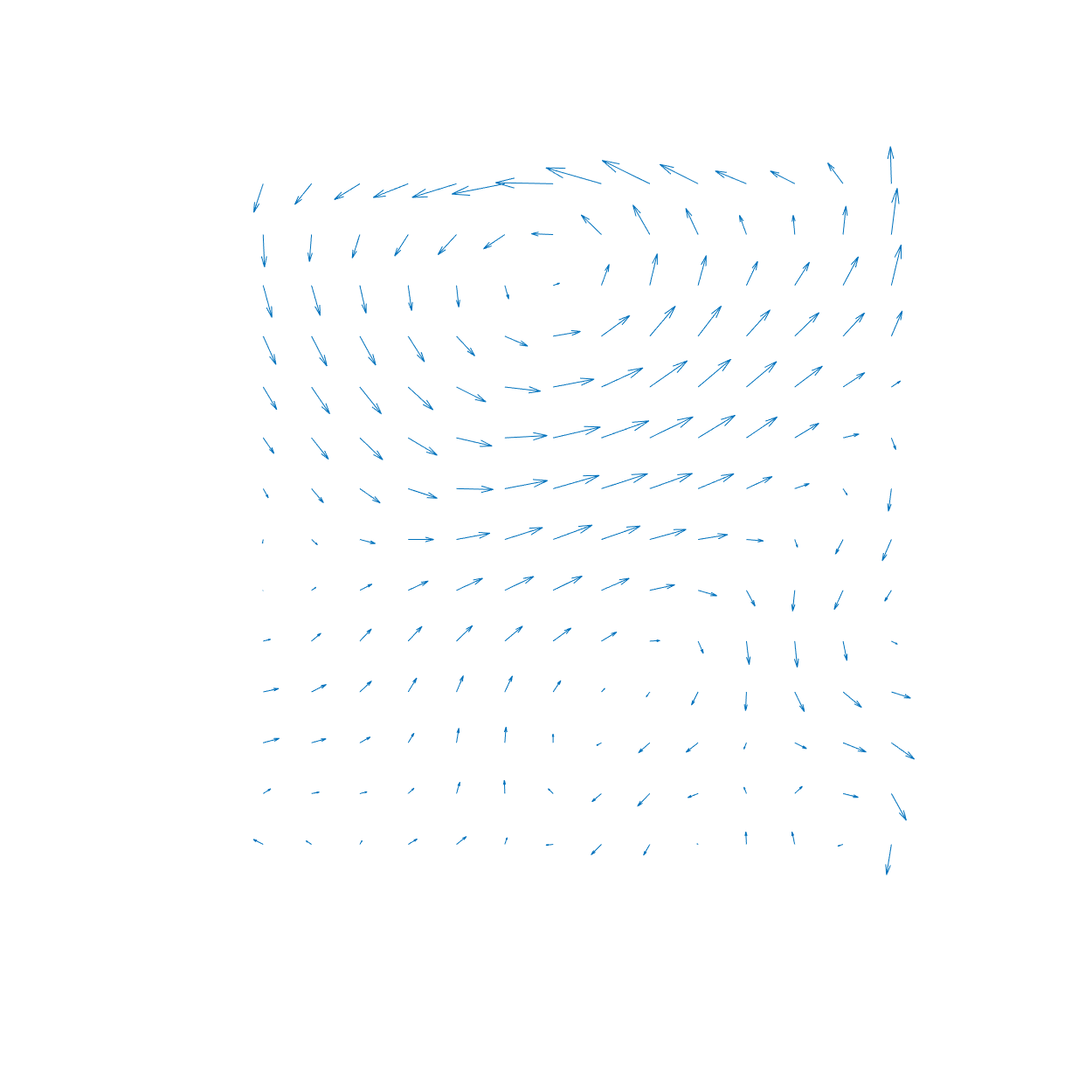

Supplement: S1 MCG raw data 1 — The raw MCG dataset includes categories 0-4 for testing. (ZIP) [file pone.0338189.s001.zip › test/2/p1_420_4.png]

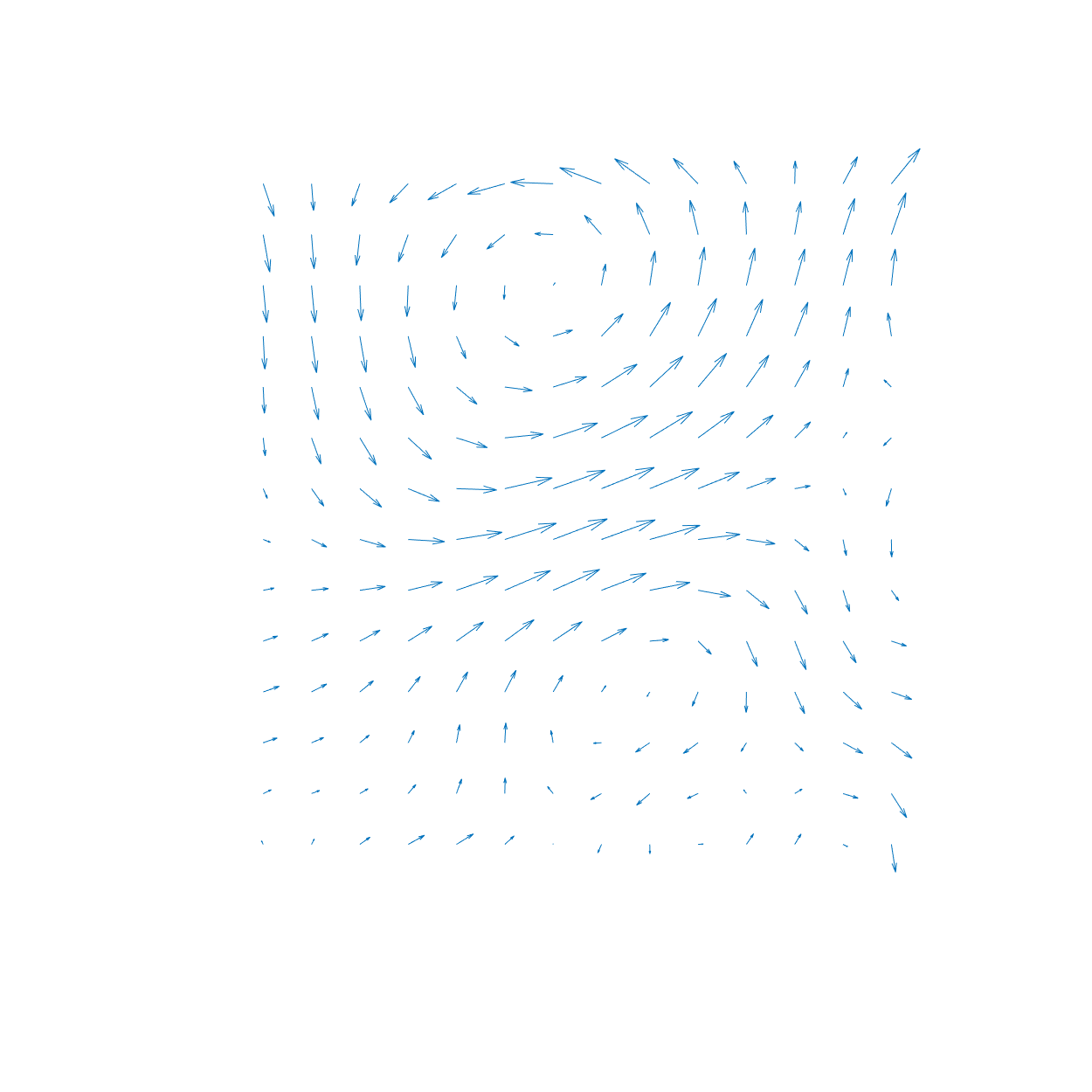

Supplement: S1 MCG raw data 1 — The raw MCG dataset includes categories 0-4 for testing. (ZIP) [file pone.0338189.s001.zip › test/2/p1_425_4.png]

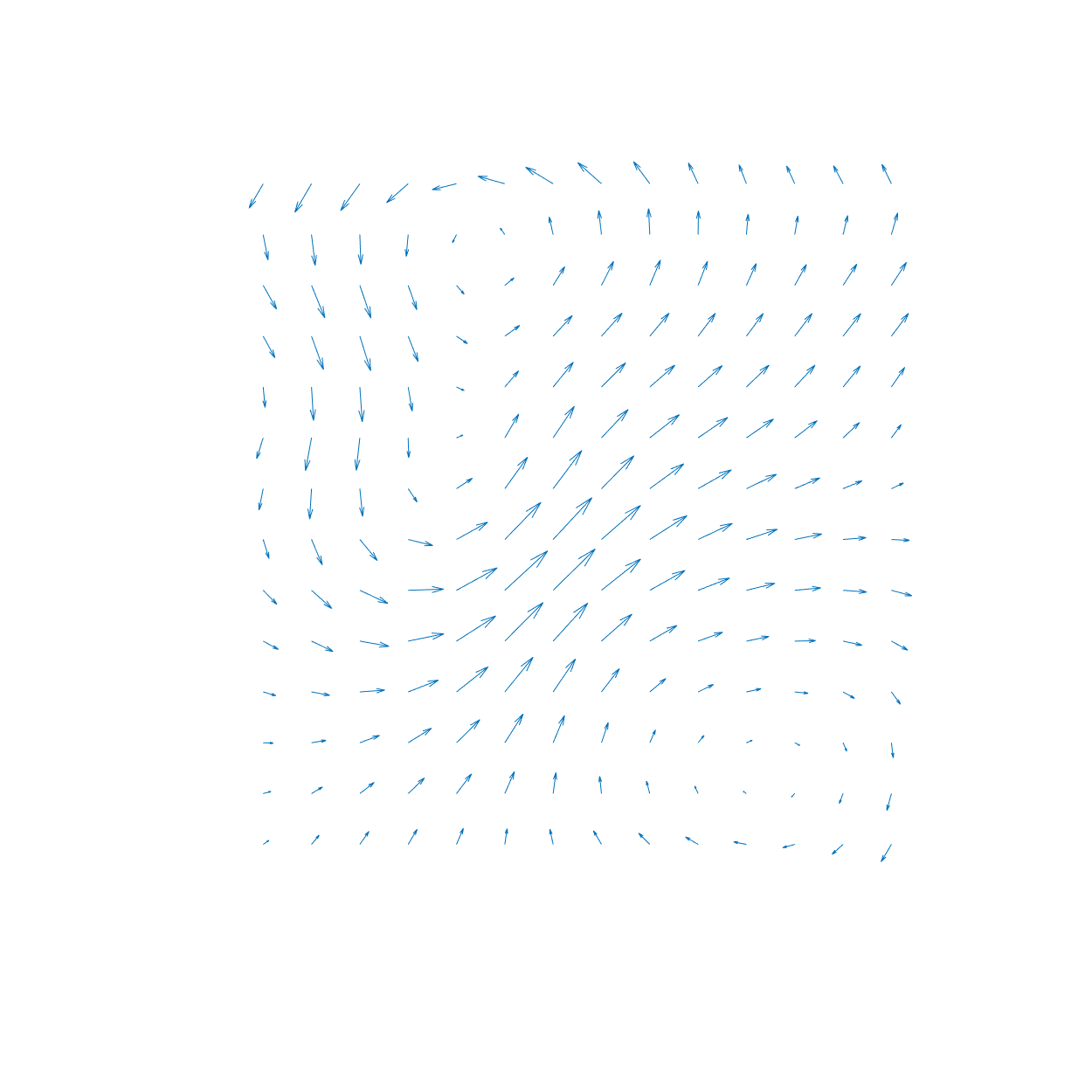

Supplement: S1 MCG raw data 1 — The raw MCG dataset includes categories 0-4 for testing. (ZIP) [file pone.0338189.s001.zip › test/2/p2_410_4.png]

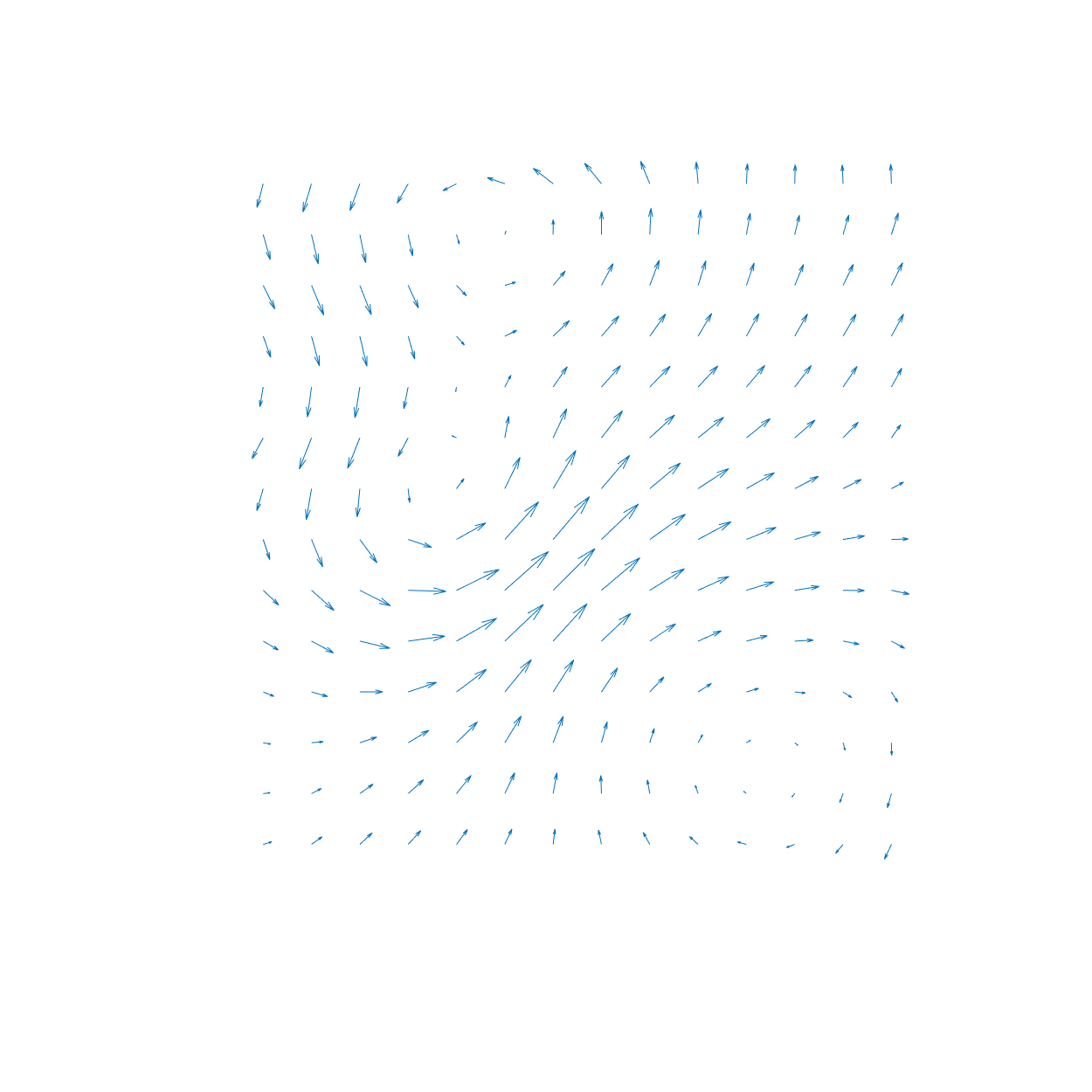

Supplement: S1 MCG raw data 1 — The raw MCG dataset includes categories 0-4 for testing. (ZIP) [file pone.0338189.s001.zip › test/2/p2_415_4.png]

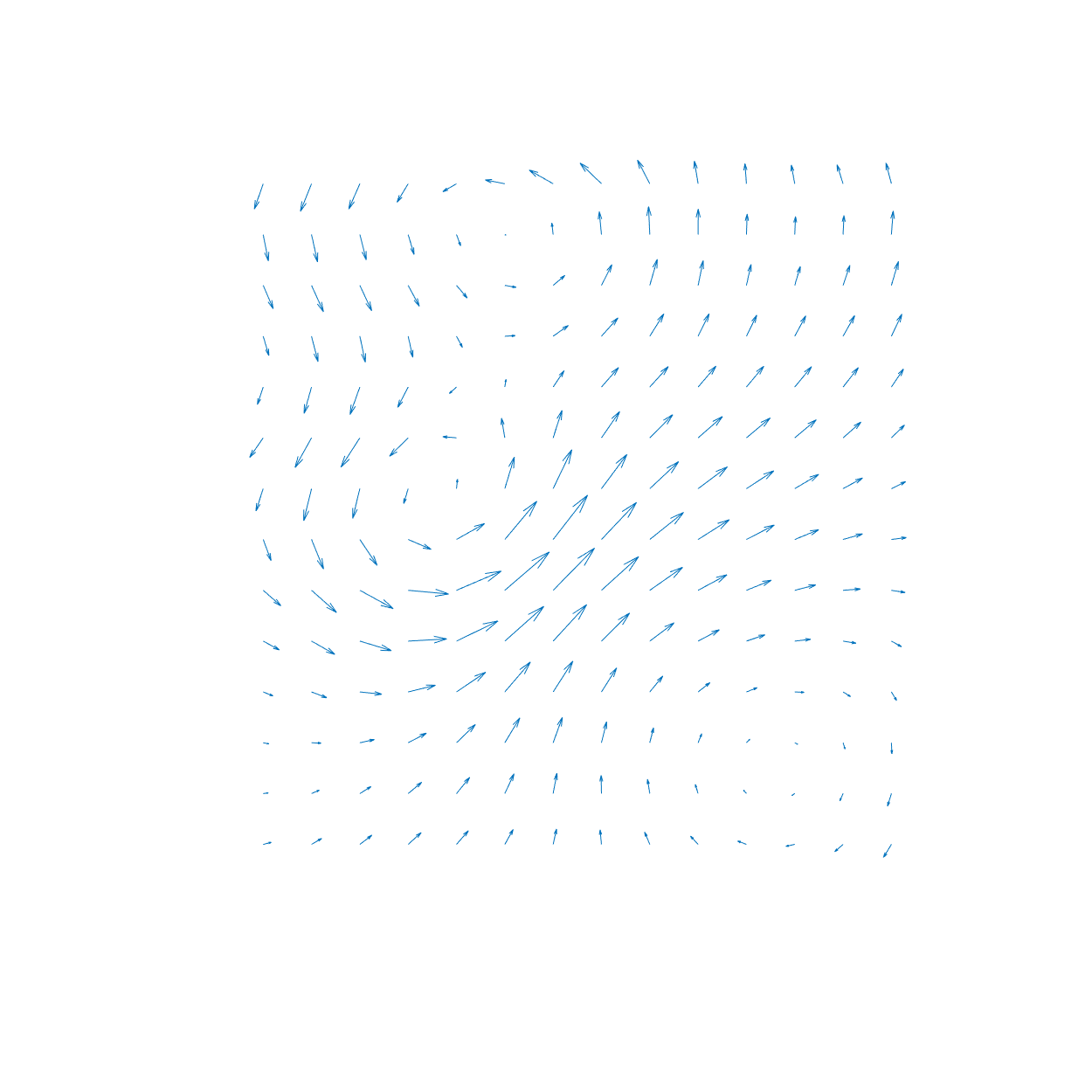

Supplement: S1 MCG raw data 1 — The raw MCG dataset includes categories 0-4 for testing. (ZIP) [file pone.0338189.s001.zip › test/2/p2_420_4.png]

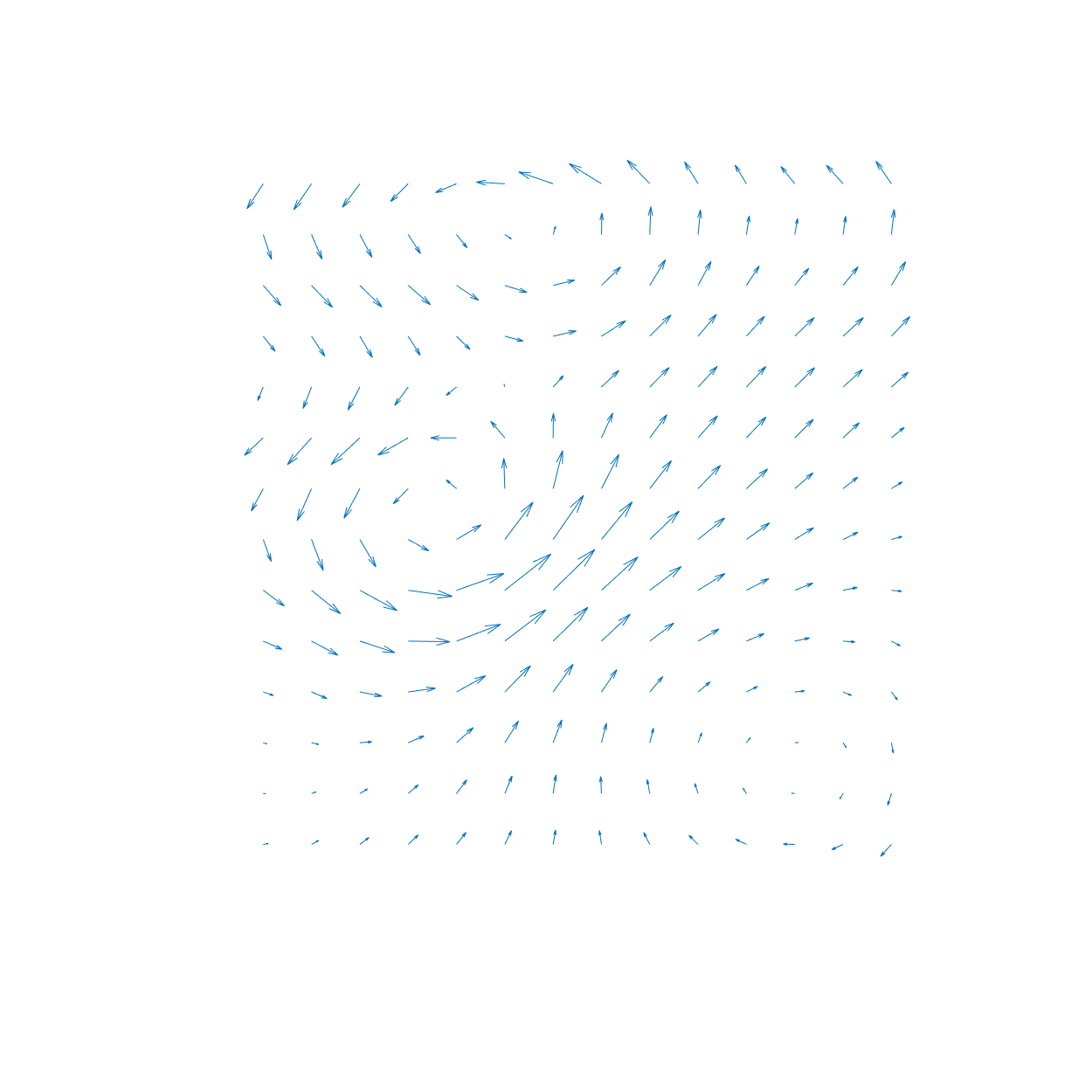

Supplement: S1 MCG raw data 1 — The raw MCG dataset includes categories 0-4 for testing. (ZIP) [file pone.0338189.s001.zip › test/2/p2_425_4.png]

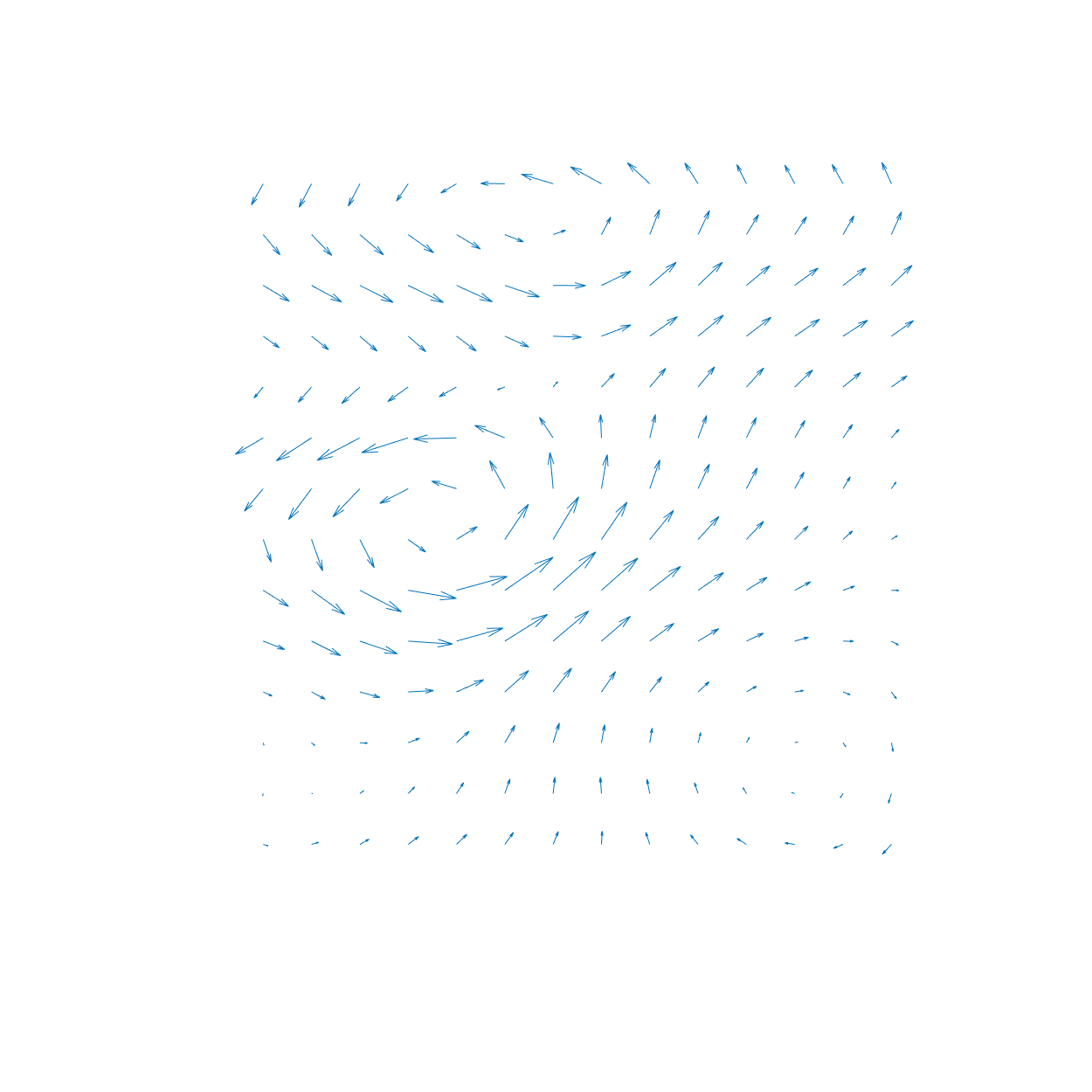

Supplement: S1 MCG raw data 1 — The raw MCG dataset includes categories 0-4 for testing. (ZIP) [file pone.0338189.s001.zip › test/2/p2_430_4.png]

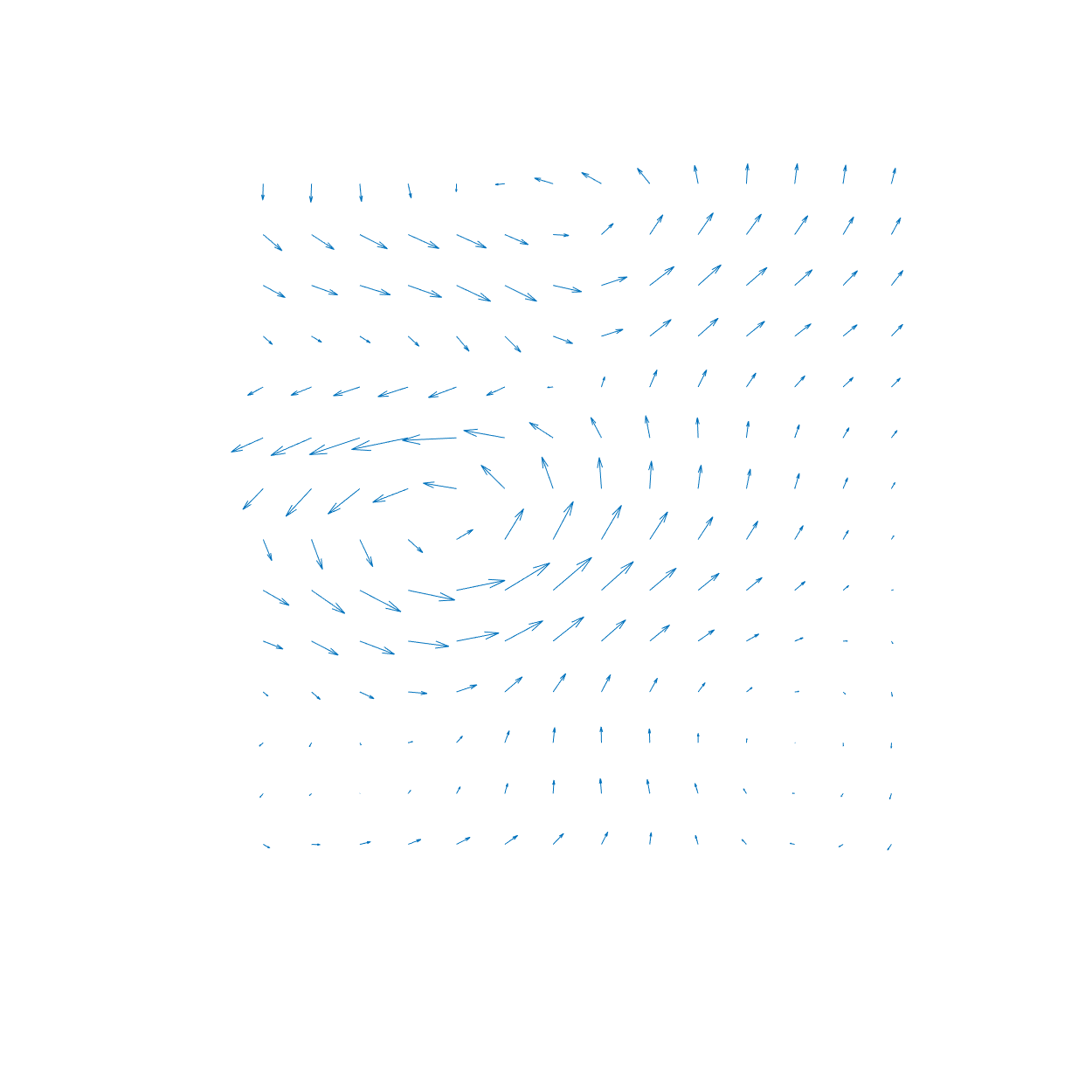

Supplement: S1 MCG raw data 1 — The raw MCG dataset includes categories 0-4 for testing. (ZIP) [file pone.0338189.s001.zip › test/2/p2_435_4.png]

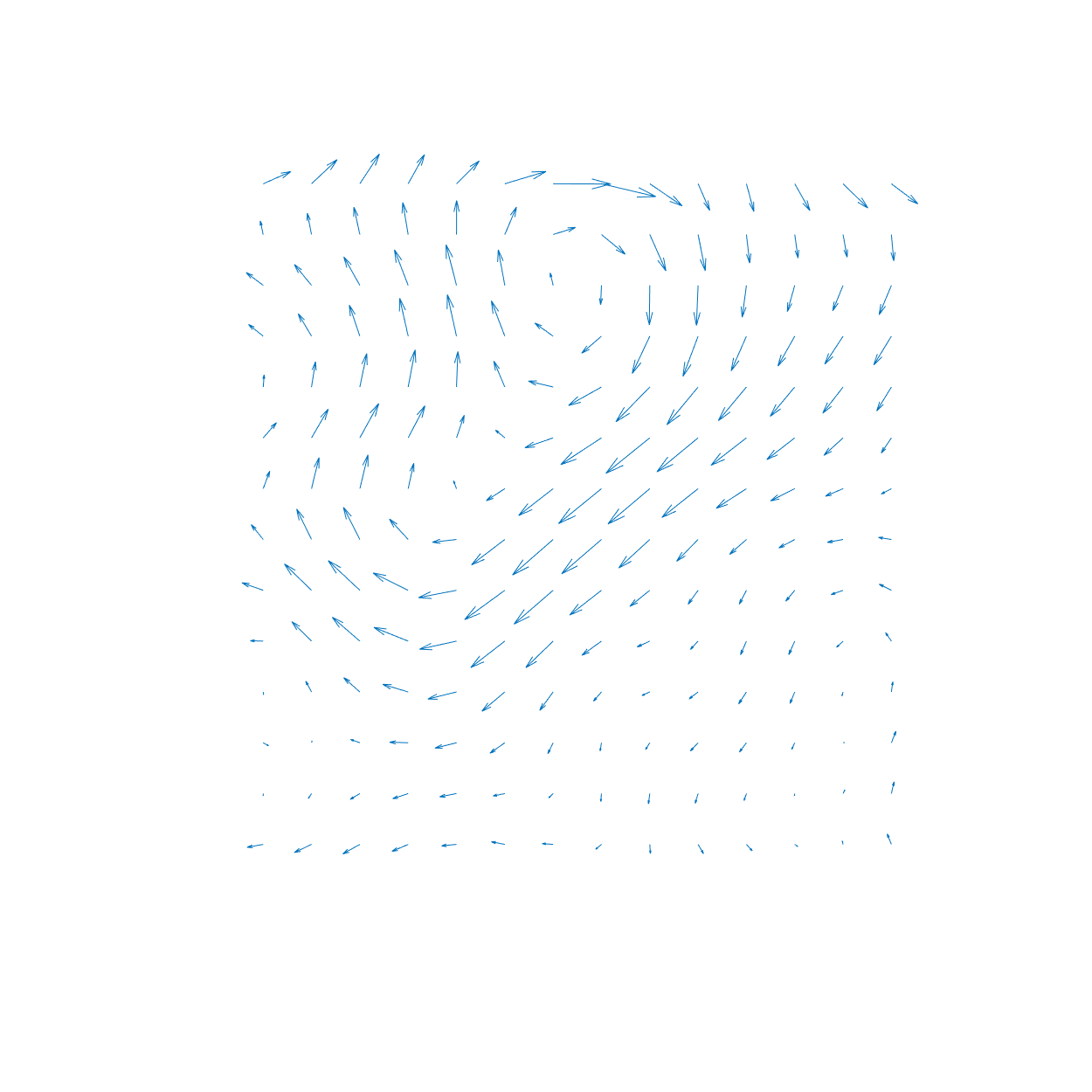

Supplement: S1 MCG raw data 1 — The raw MCG dataset includes categories 0-4 for testing. (ZIP) [file pone.0338189.s001.zip › test/2/p2_540_4.png]

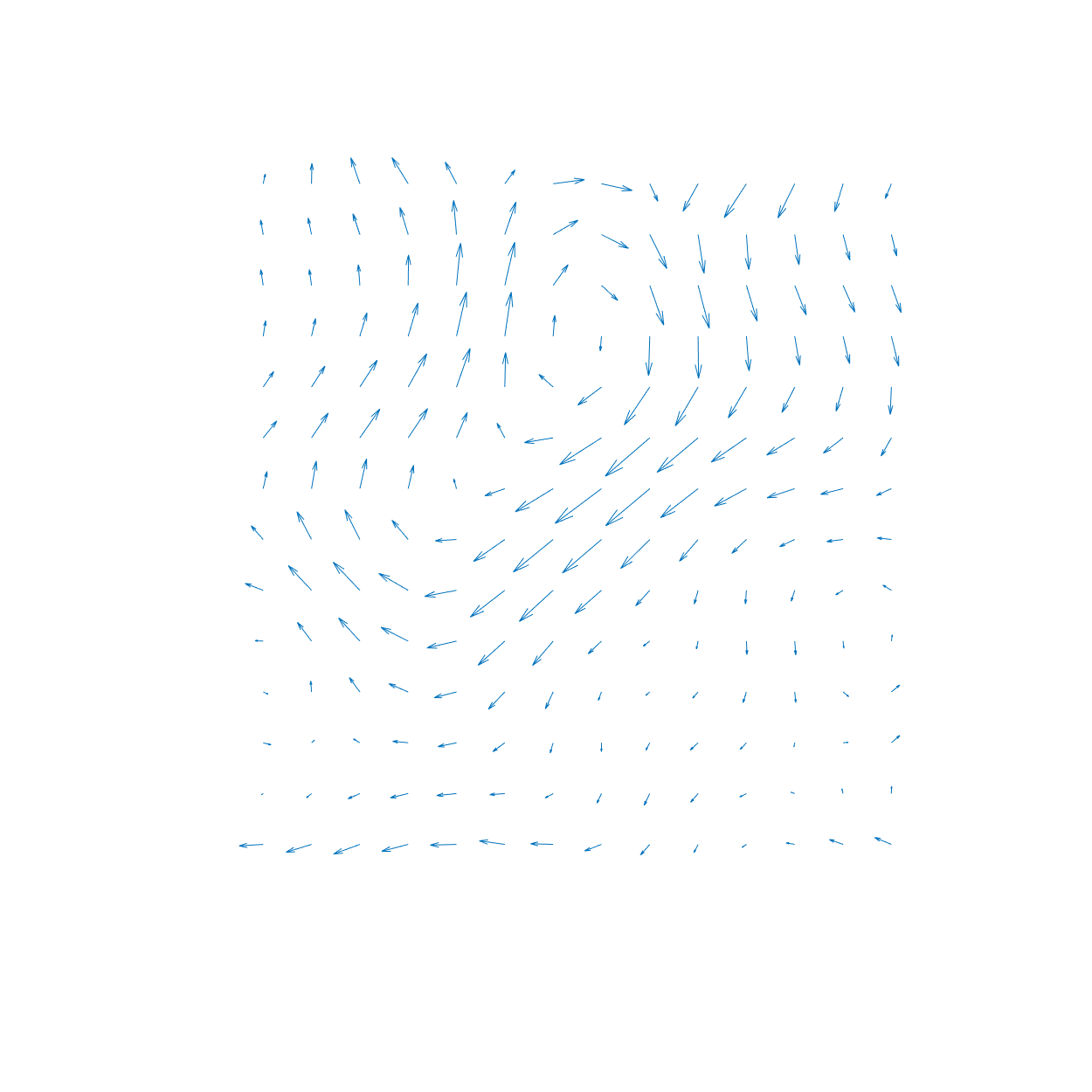

Supplement: S1 MCG raw data 1 — The raw MCG dataset includes categories 0-4 for testing. (ZIP) [file pone.0338189.s001.zip › test/2/p2_545_4.png]

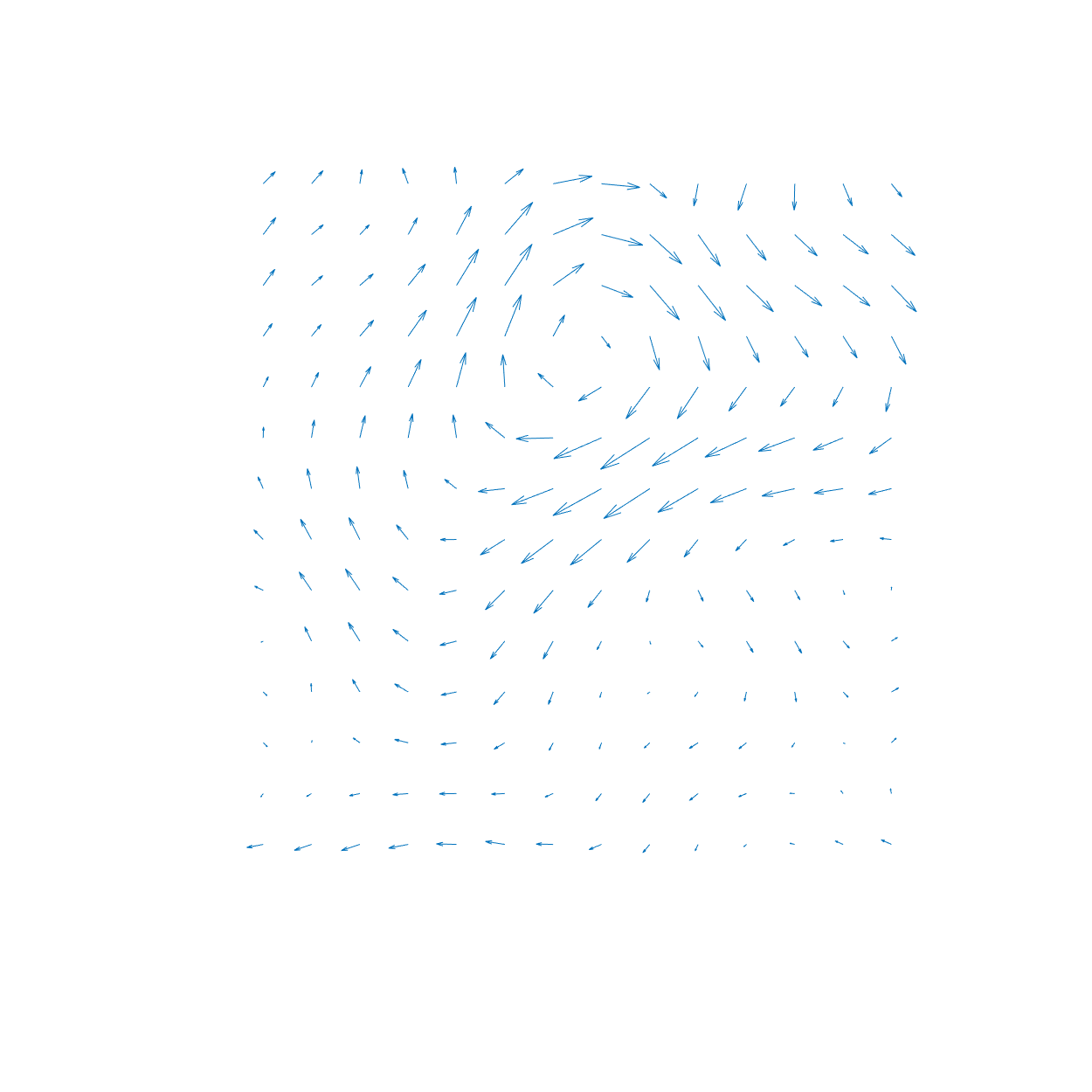

Supplement: S1 MCG raw data 1 — The raw MCG dataset includes categories 0-4 for testing. (ZIP) [file pone.0338189.s001.zip › test/2/p2_550_4.png]

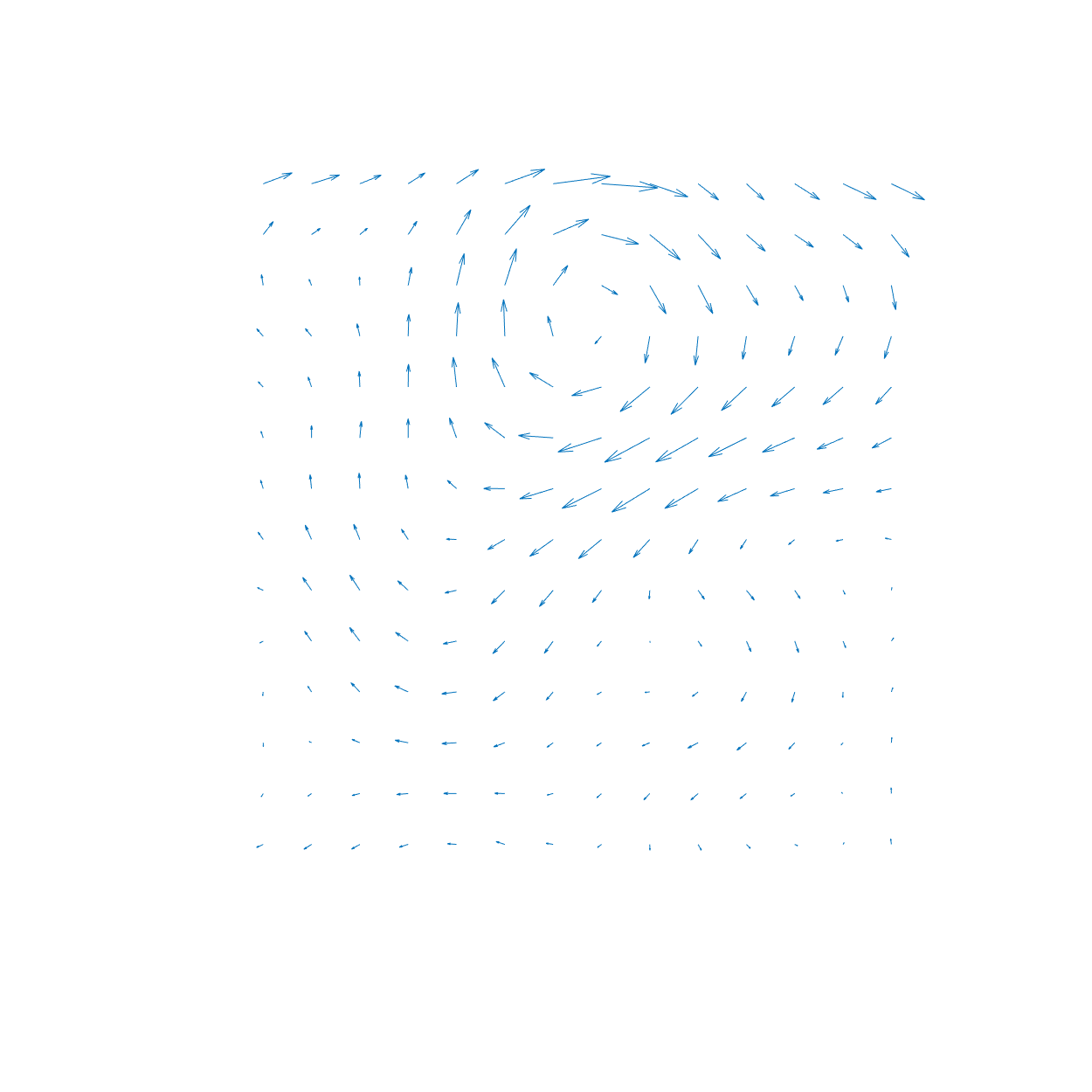

Supplement: S1 MCG raw data 1 — The raw MCG dataset includes categories 0-4 for testing. (ZIP) [file pone.0338189.s001.zip › test/2/p2_555_4.png]

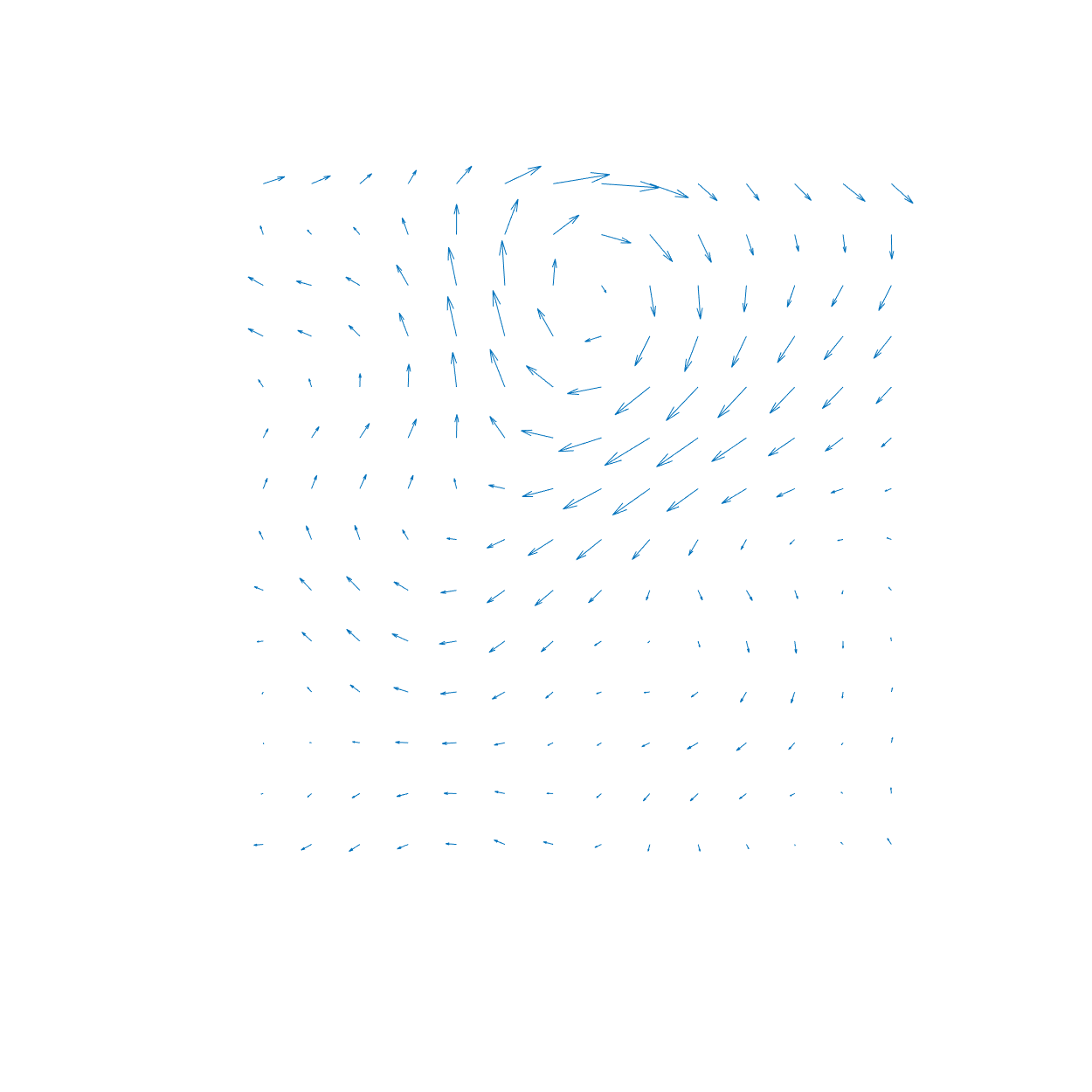

Supplement: S1 MCG raw data 1 — The raw MCG dataset includes categories 0-4 for testing. (ZIP) [file pone.0338189.s001.zip › test/2/p2_560_4.png]

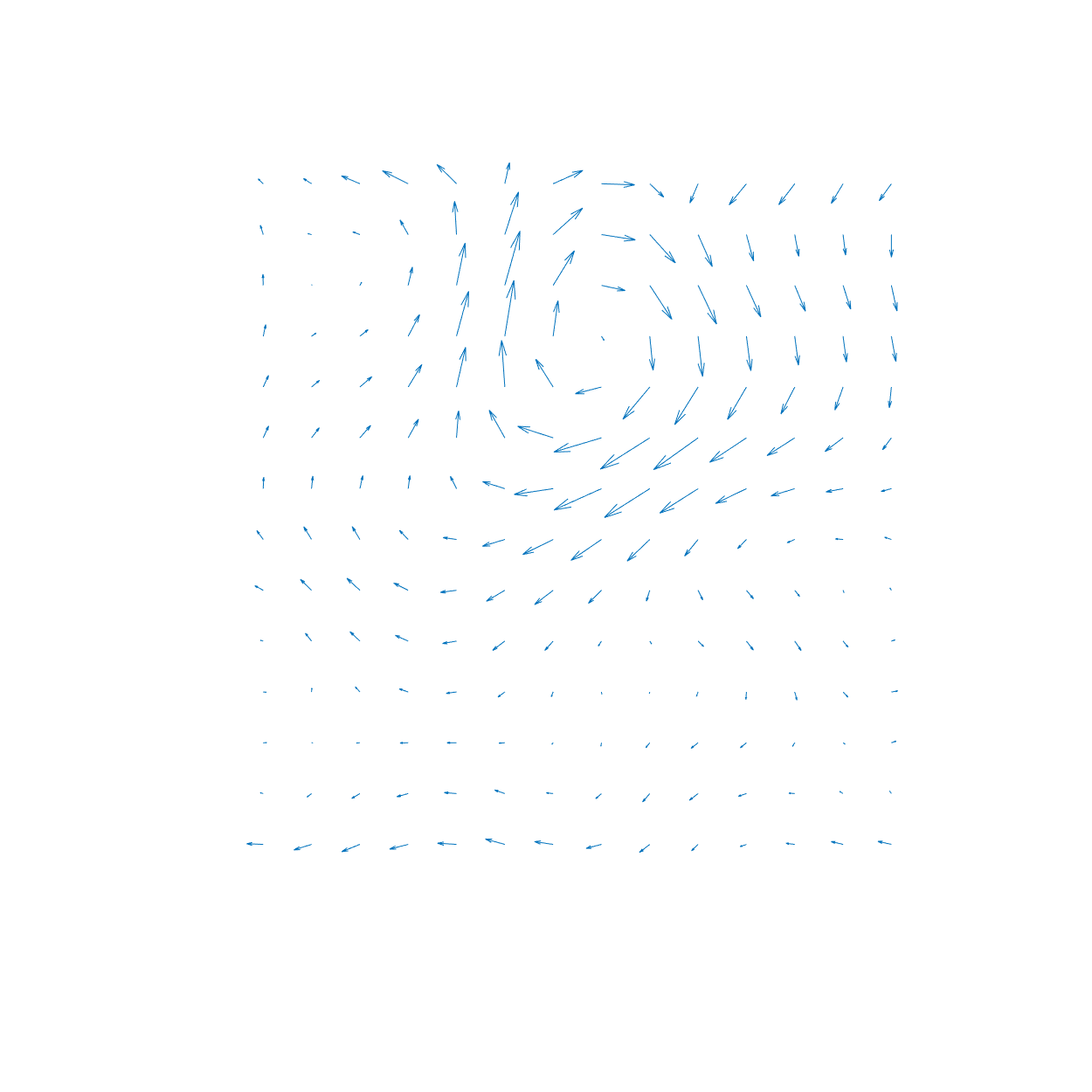

Supplement: S1 MCG raw data 1 — The raw MCG dataset includes categories 0-4 for testing. (ZIP) [file pone.0338189.s001.zip › test/2/p2_565_4.png]

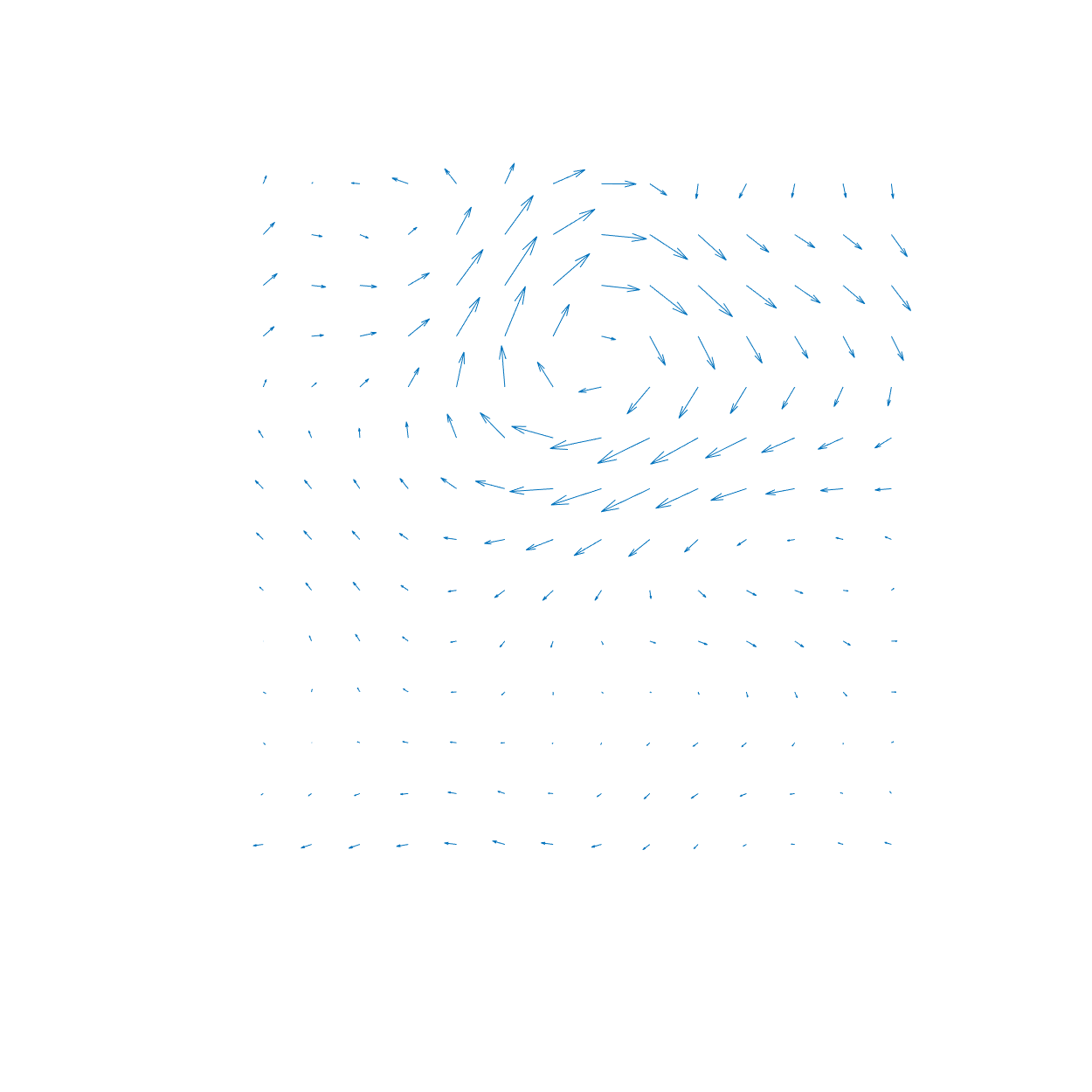

Supplement: S1 MCG raw data 1 — The raw MCG dataset includes categories 0-4 for testing. (ZIP) [file pone.0338189.s001.zip › test/2/p2_570_4.png]

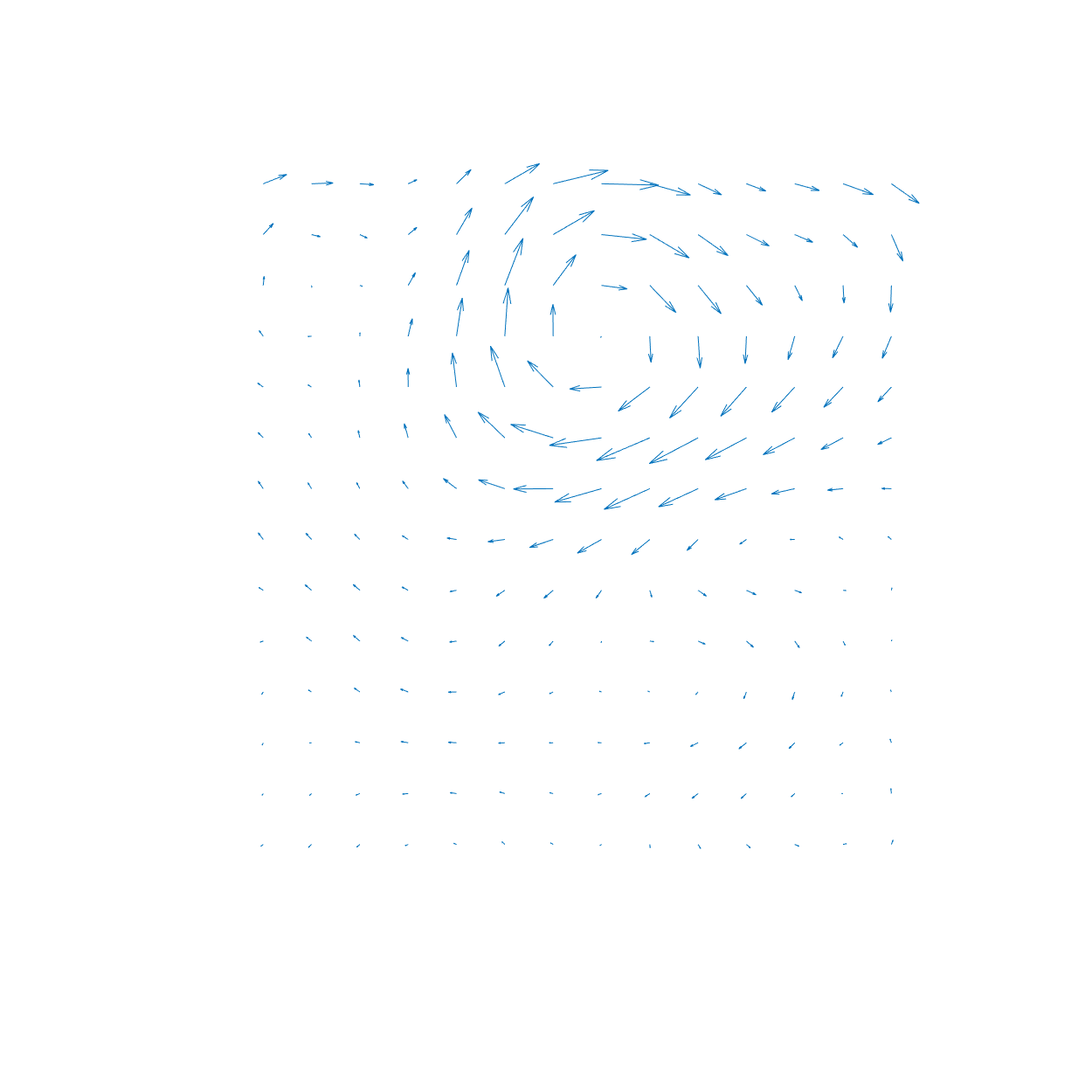

Supplement: S1 MCG raw data 1 — The raw MCG dataset includes categories 0-4 for testing. (ZIP) [file pone.0338189.s001.zip › test/2/p2_575_4.png]

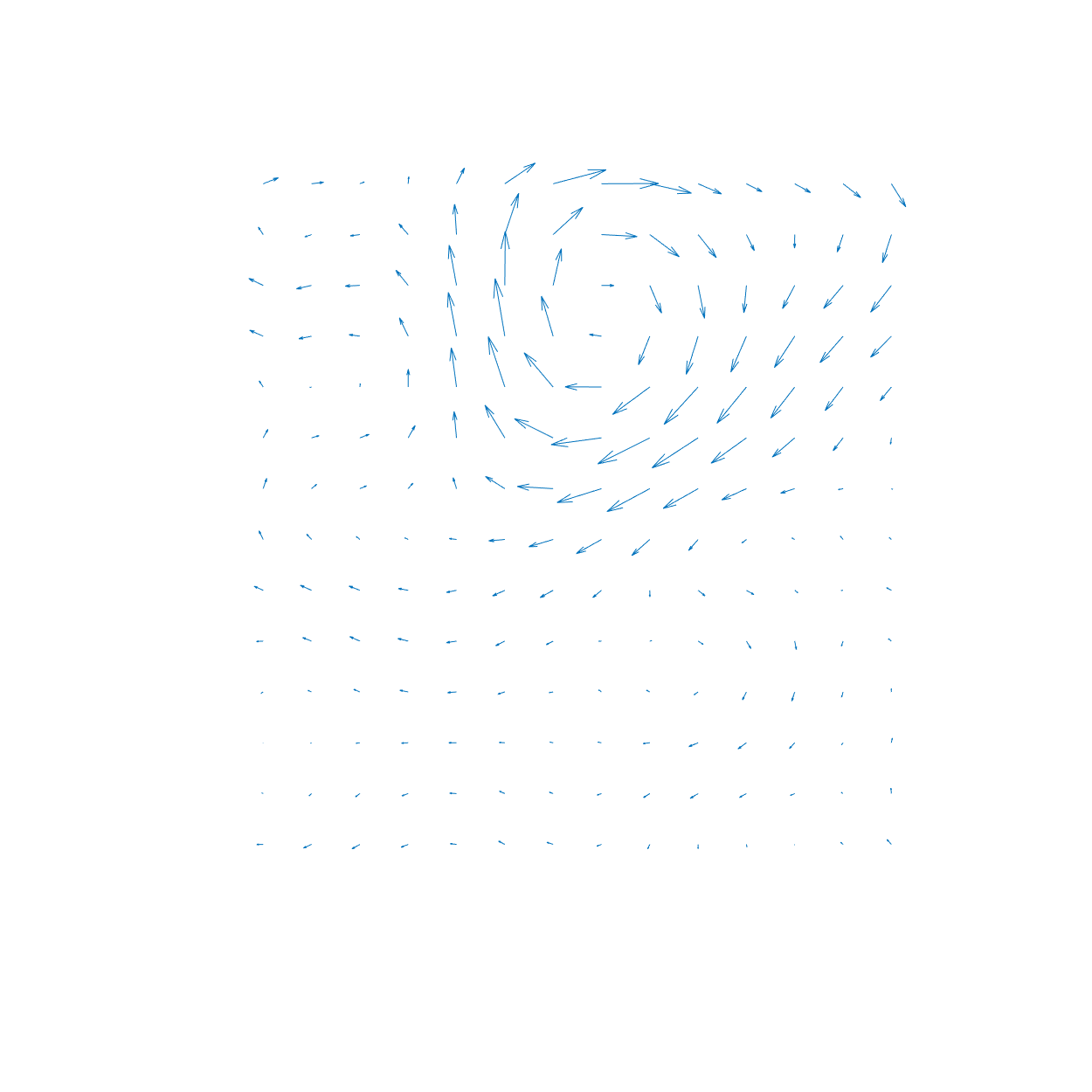

Supplement: S1 MCG raw data 1 — The raw MCG dataset includes categories 0-4 for testing. (ZIP) [file pone.0338189.s001.zip › test/2/p2_580_4.png]

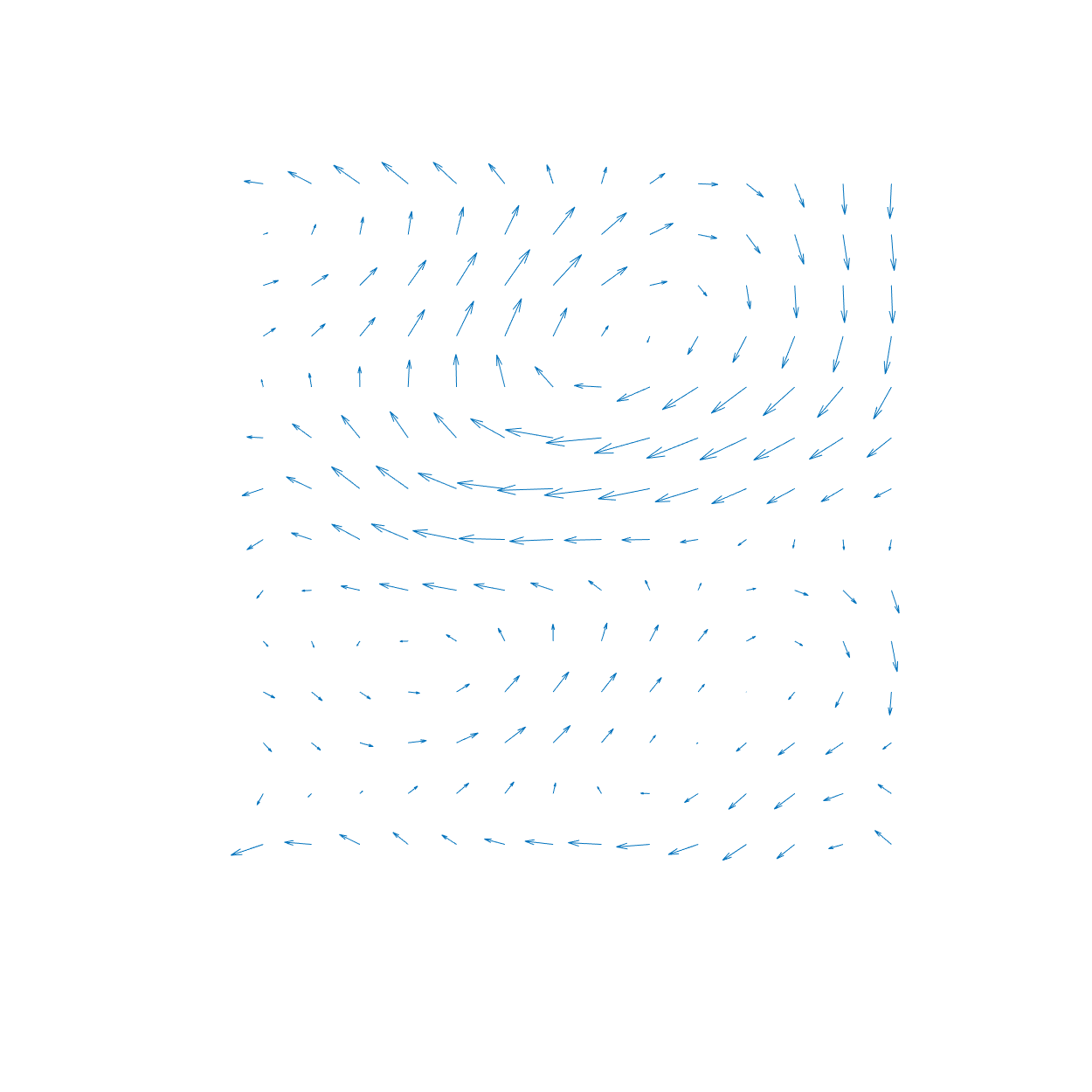

Supplement: S1 MCG raw data 1 — The raw MCG dataset includes categories 0-4 for testing. (ZIP) [file pone.0338189.s001.zip › test/2/p3_540_4.png]

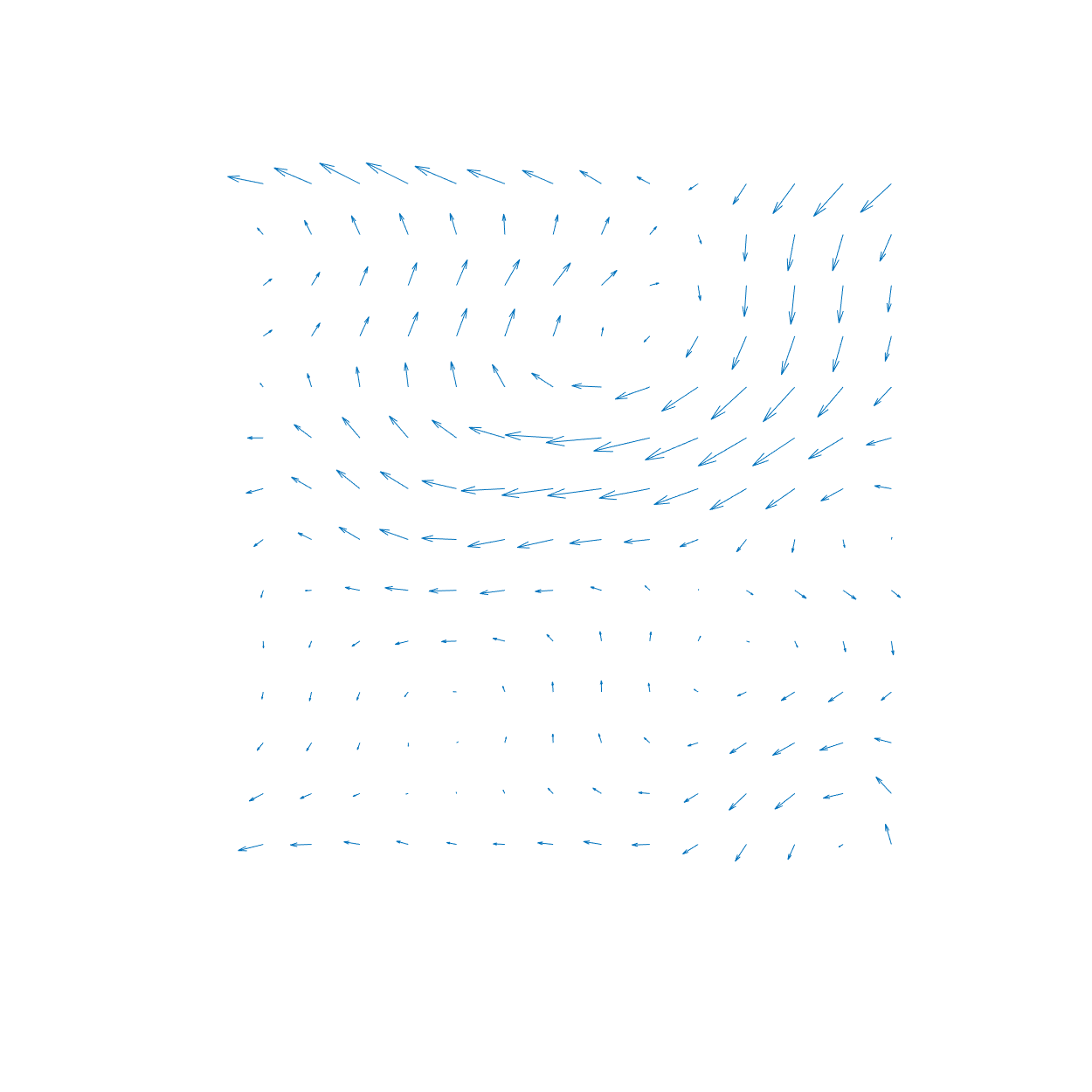

Supplement: S1 MCG raw data 1 — The raw MCG dataset includes categories 0-4 for testing. (ZIP) [file pone.0338189.s001.zip › test/2/p3_545_4.png]

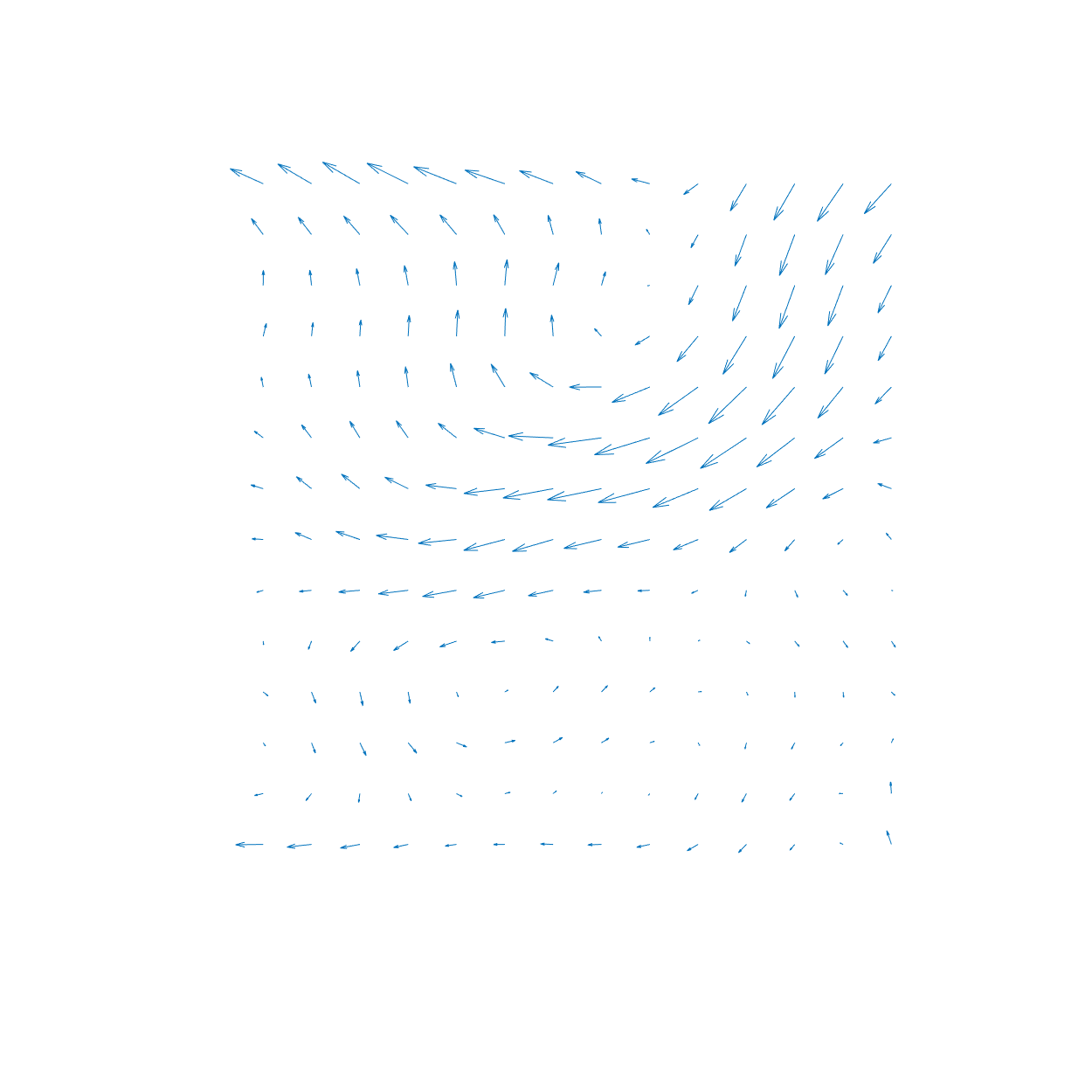

Supplement: S1 MCG raw data 1 — The raw MCG dataset includes categories 0-4 for testing. (ZIP) [file pone.0338189.s001.zip › test/2/p3_550_4.png]

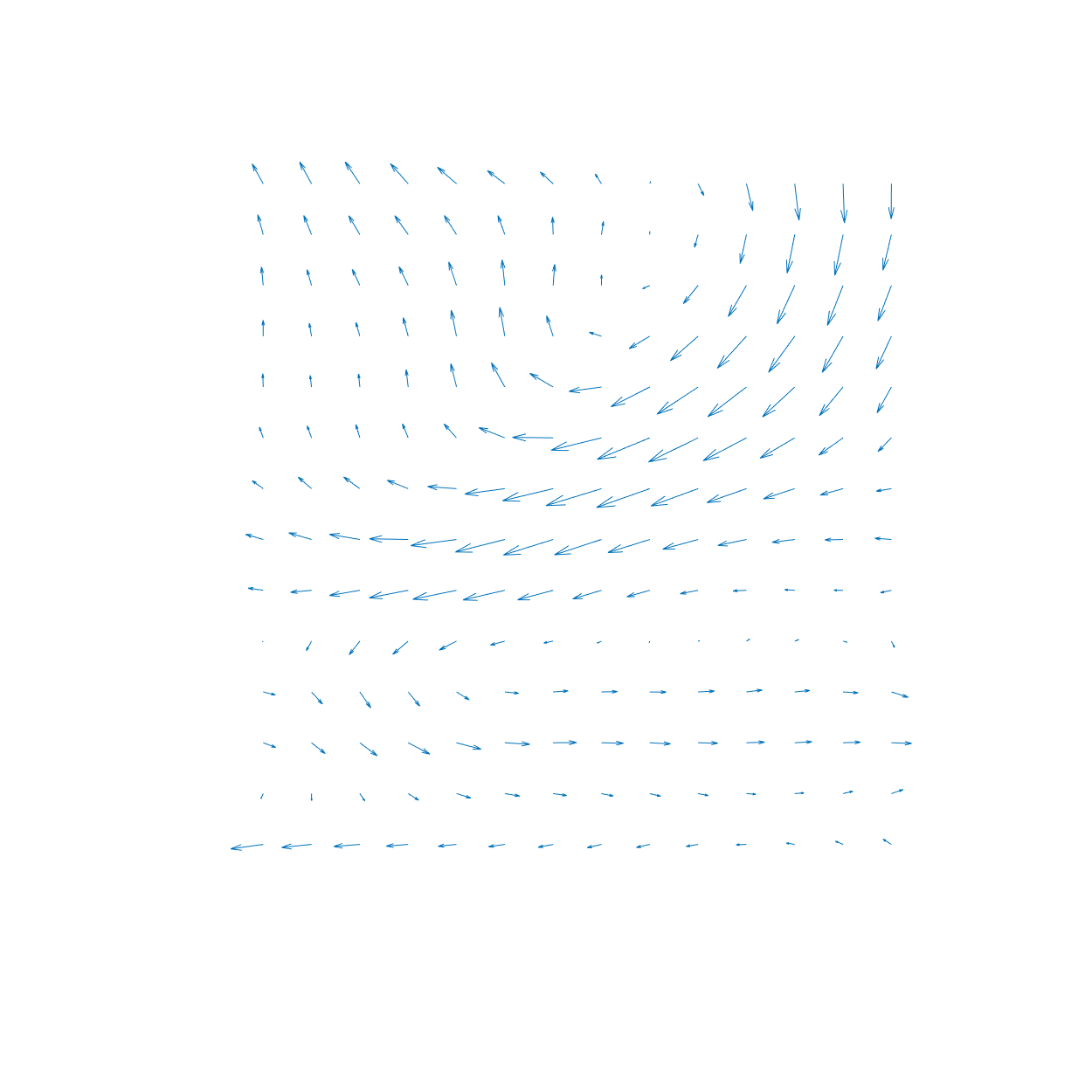

Supplement: S1 MCG raw data 1 — The raw MCG dataset includes categories 0-4 for testing. (ZIP) [file pone.0338189.s001.zip › test/2/p3_555_4.png]

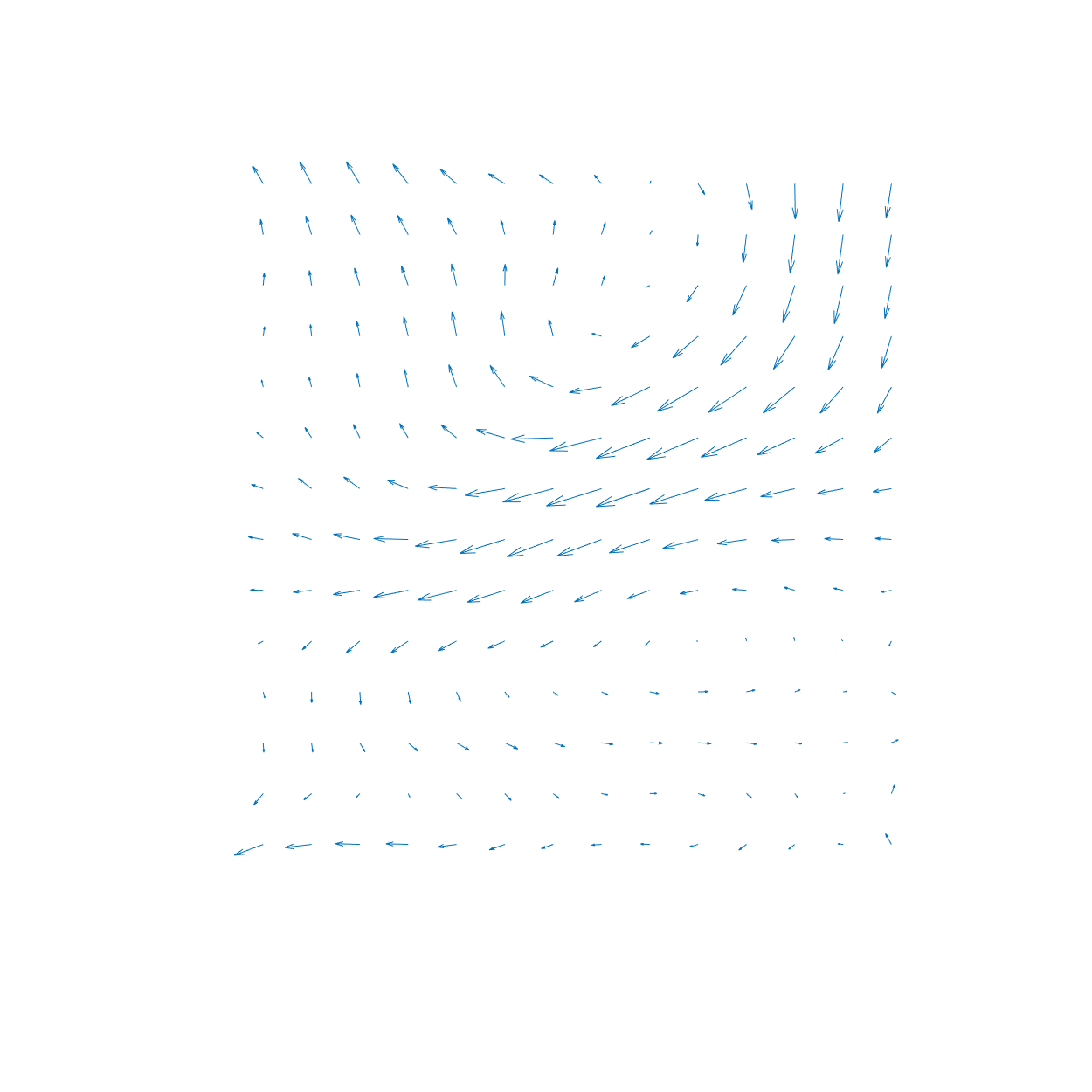

Supplement: S1 MCG raw data 1 — The raw MCG dataset includes categories 0-4 for testing. (ZIP) [file pone.0338189.s001.zip › test/2/p3_560_4.png]

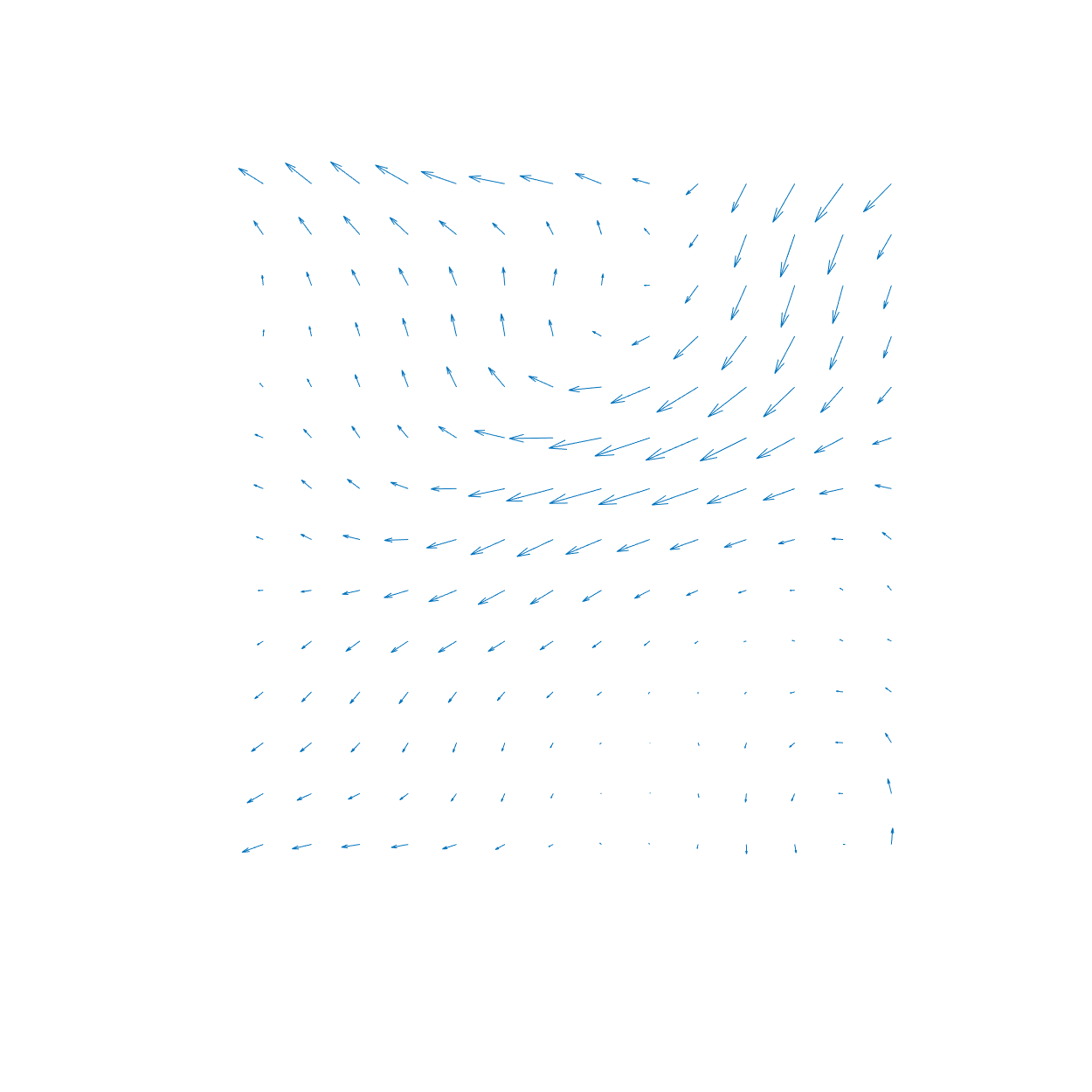

Supplement: S1 MCG raw data 1 — The raw MCG dataset includes categories 0-4 for testing. (ZIP) [file pone.0338189.s001.zip › test/2/p3_565_4.png]

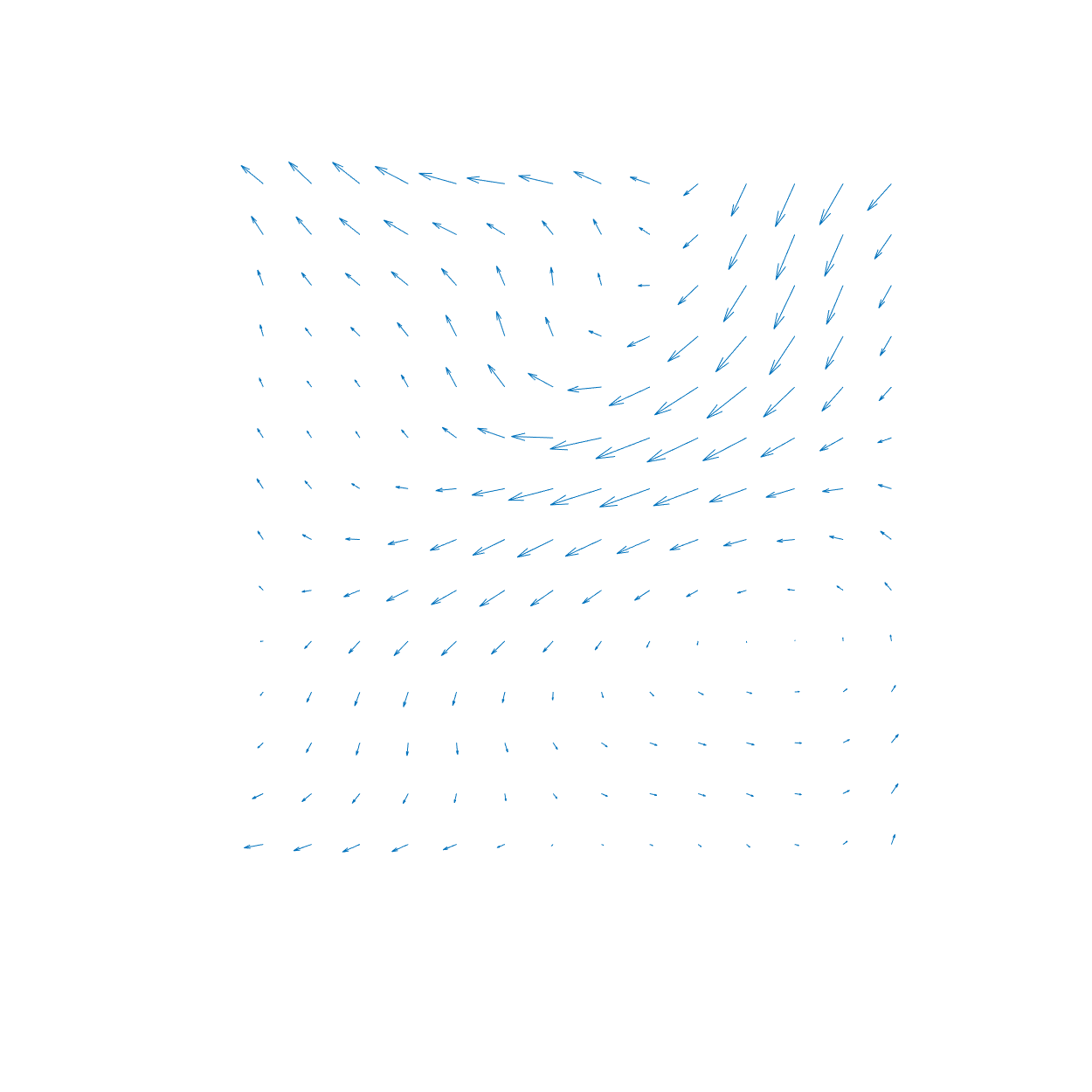

Supplement: S1 MCG raw data 1 — The raw MCG dataset includes categories 0-4 for testing. (ZIP) [file pone.0338189.s001.zip › test/2/p3_570_4.png]

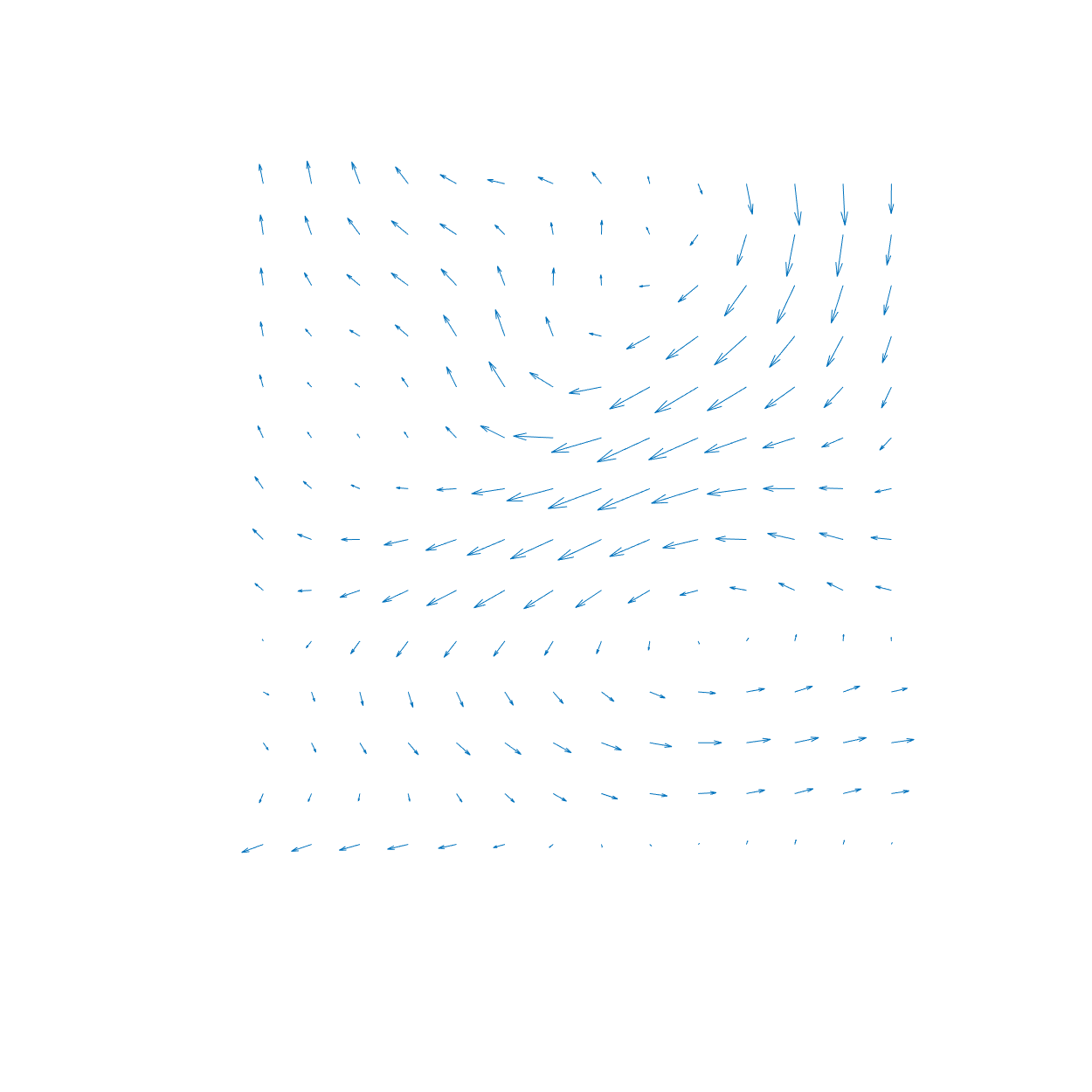

Supplement: S1 MCG raw data 1 — The raw MCG dataset includes categories 0-4 for testing. (ZIP) [file pone.0338189.s001.zip › test/2/p3_575_4.png]

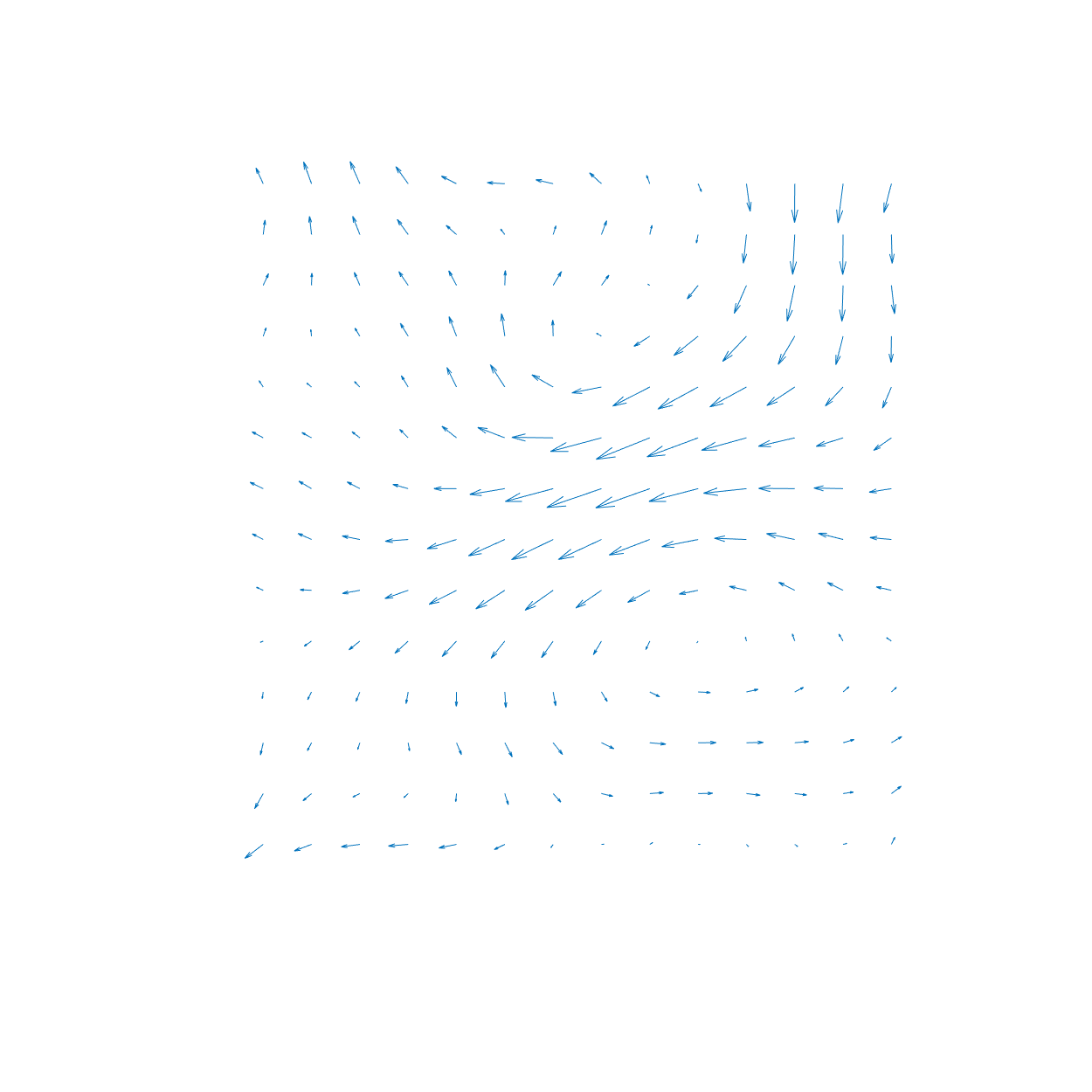

Supplement: S1 MCG raw data 1 — The raw MCG dataset includes categories 0-4 for testing. (ZIP) [file pone.0338189.s001.zip › test/2/p3_580_4.png]

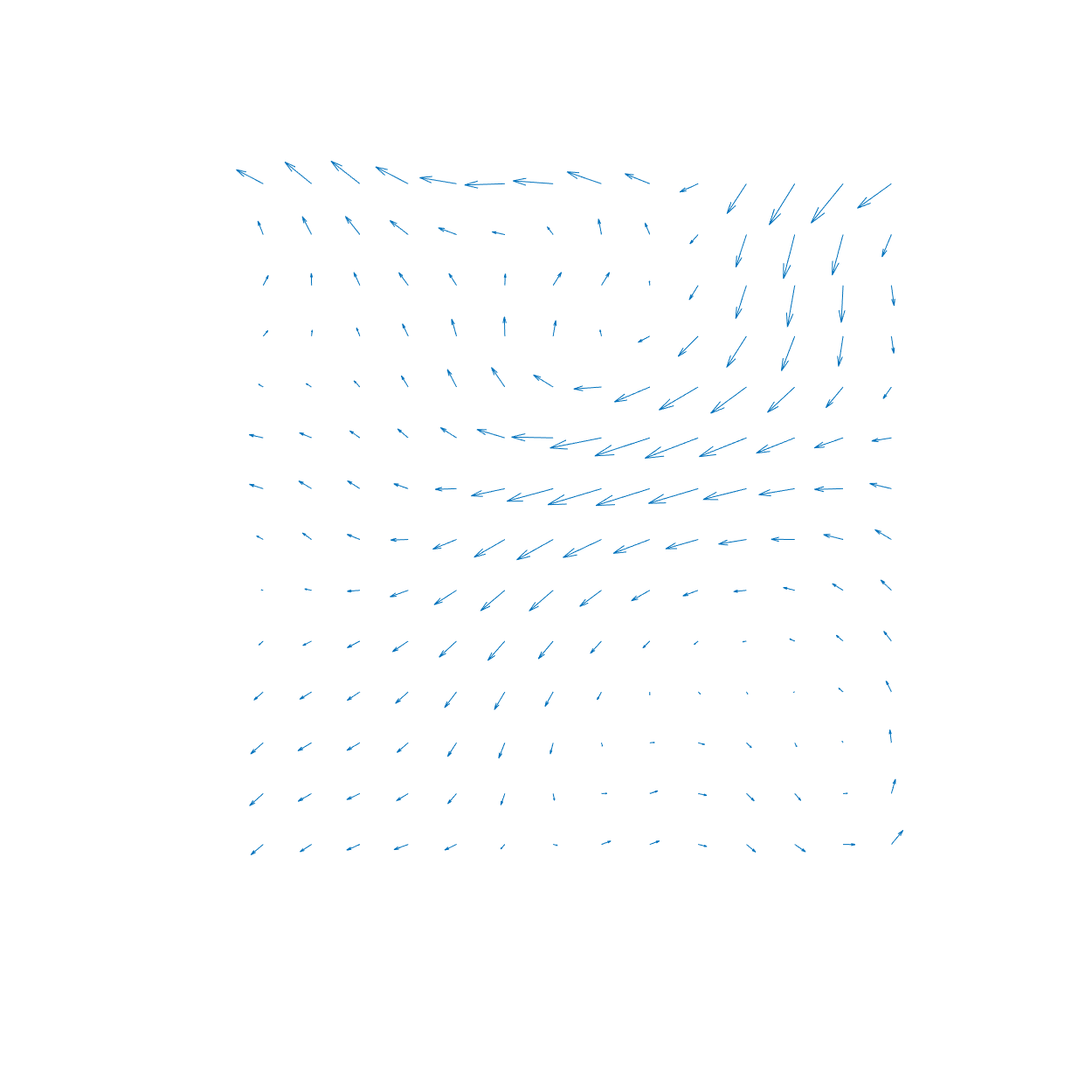

Supplement: S1 MCG raw data 1 — The raw MCG dataset includes categories 0-4 for testing. (ZIP) [file pone.0338189.s001.zip › test/2/p3_585_4.png]

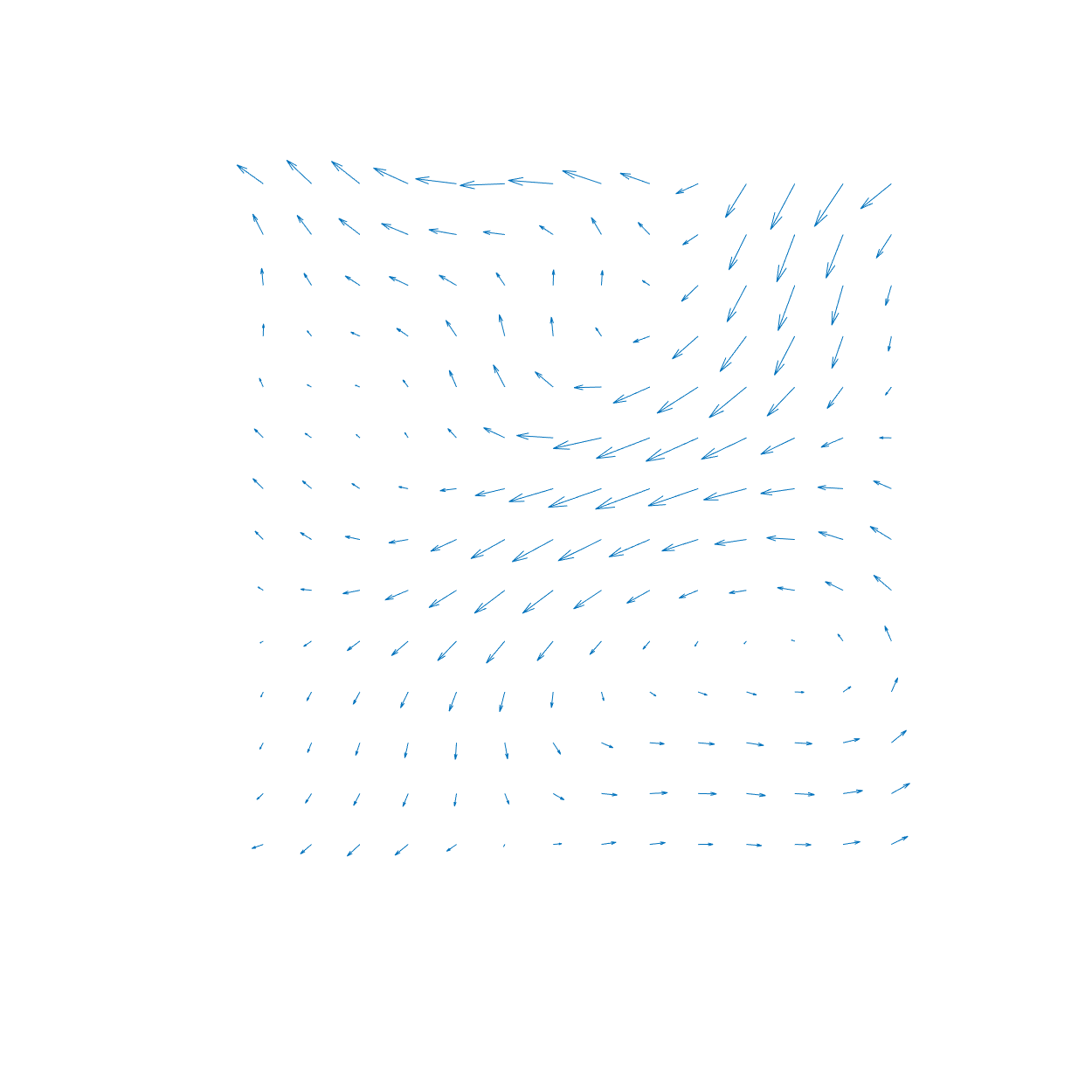

Supplement: S1 MCG raw data 1 — The raw MCG dataset includes categories 0-4 for testing. (ZIP) [file pone.0338189.s001.zip › test/2/p3_590_4.png]

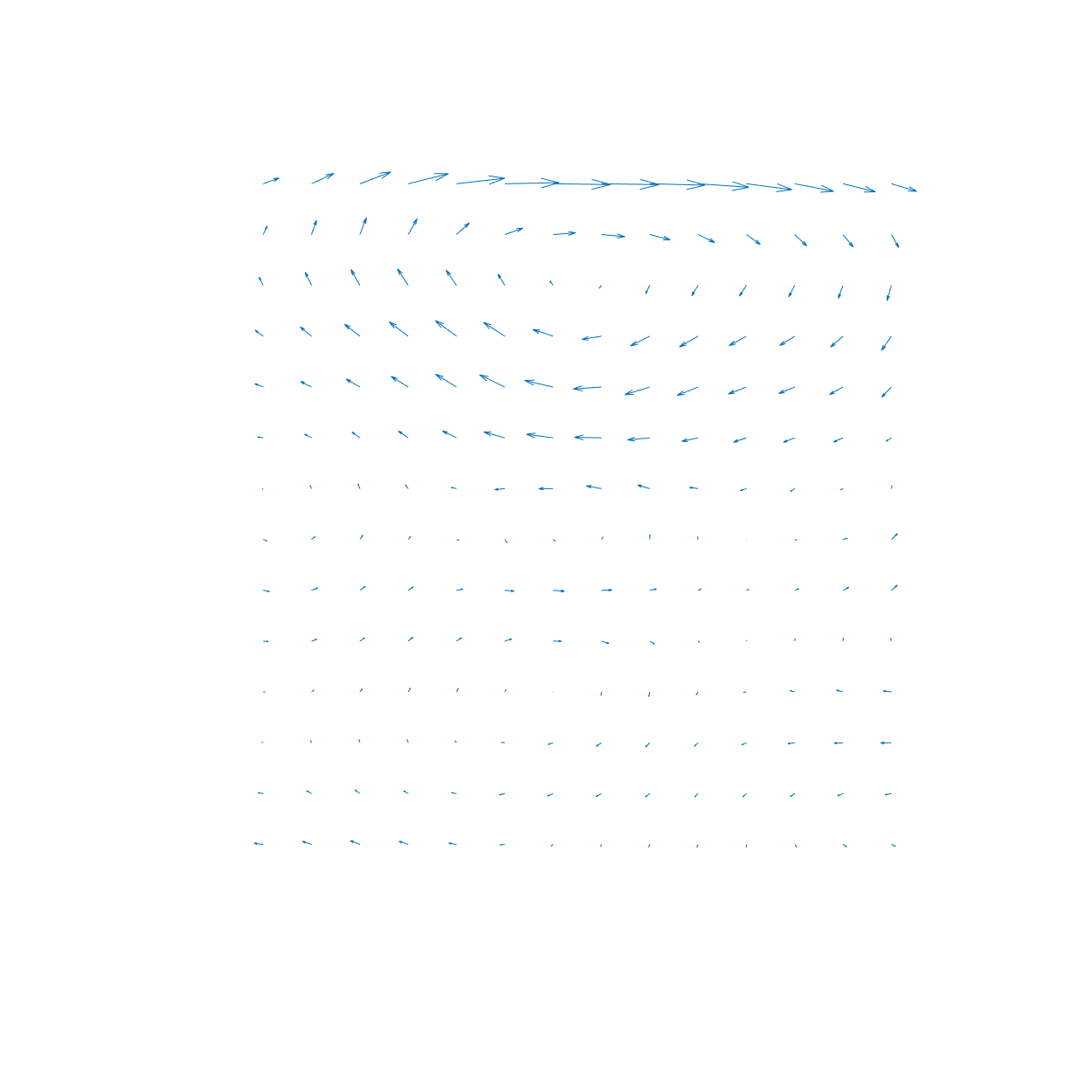

Supplement: S1 MCG raw data 1 — The raw MCG dataset includes categories 0-4 for testing. (ZIP) [file pone.0338189.s001.zip › test/2/p4_230_4.png]

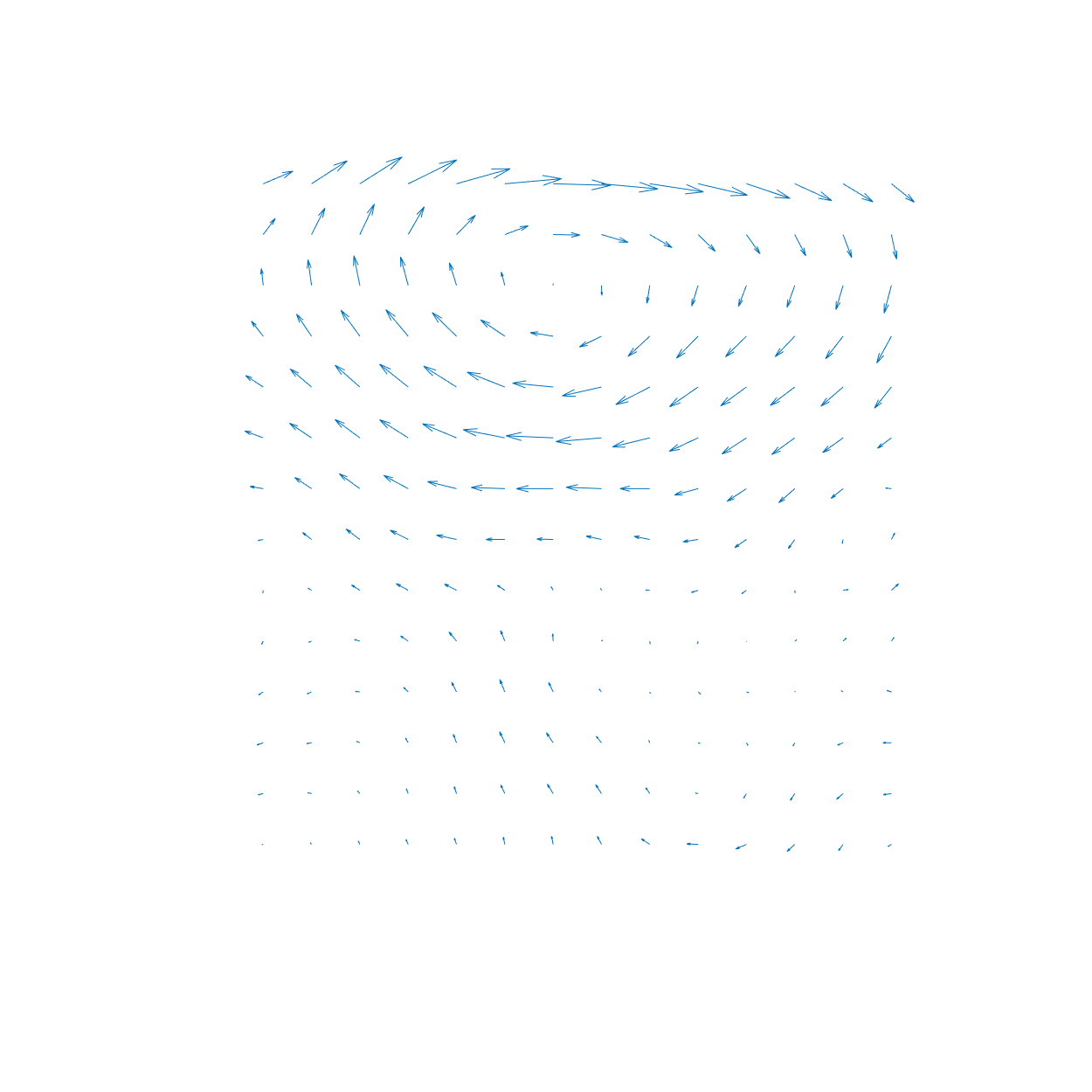

Supplement: S1 MCG raw data 1 — The raw MCG dataset includes categories 0-4 for testing. (ZIP) [file pone.0338189.s001.zip › test/2/p4_235_4.png]

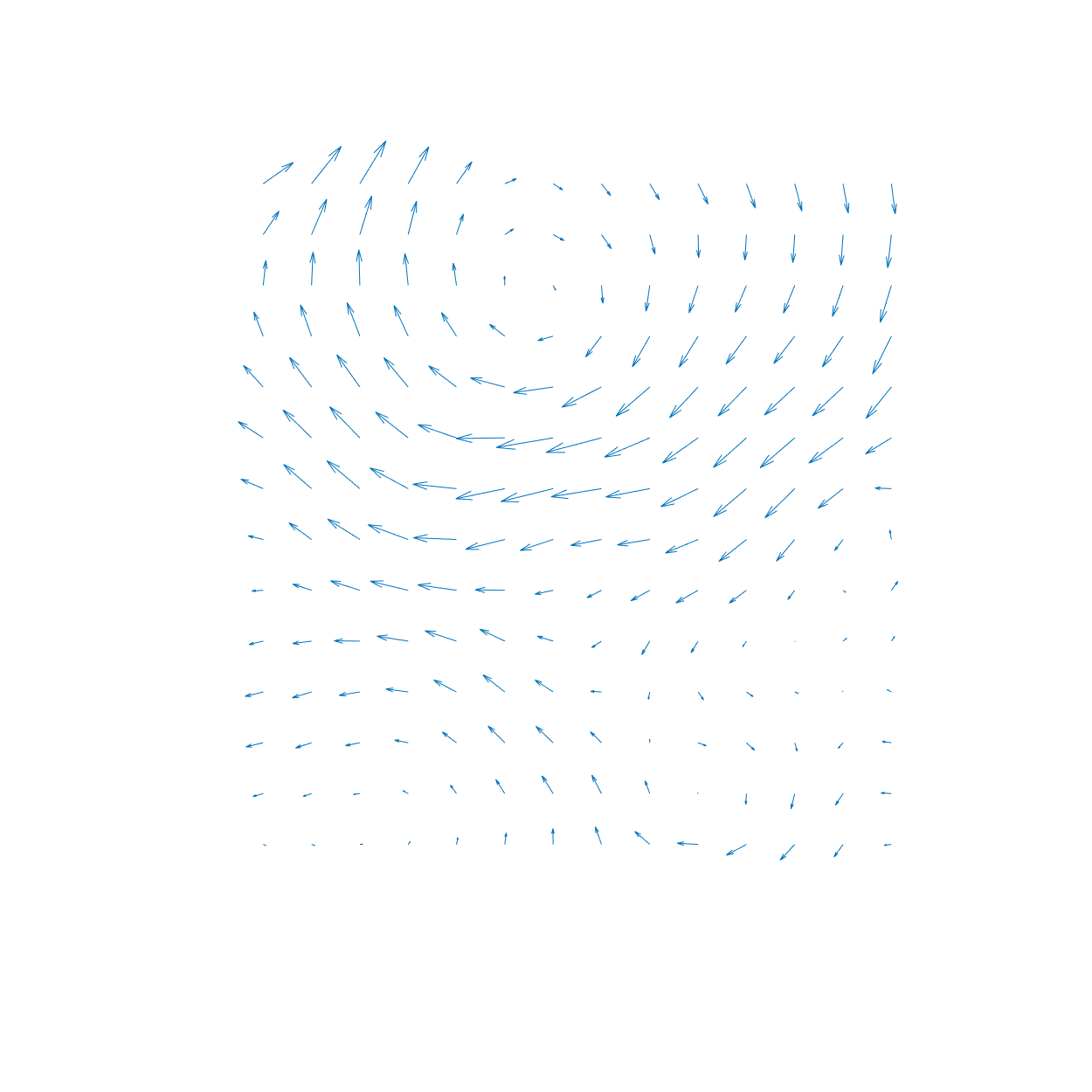

Supplement: S1 MCG raw data 1 — The raw MCG dataset includes categories 0-4 for testing. (ZIP) [file pone.0338189.s001.zip › test/2/p4_240_4.png]

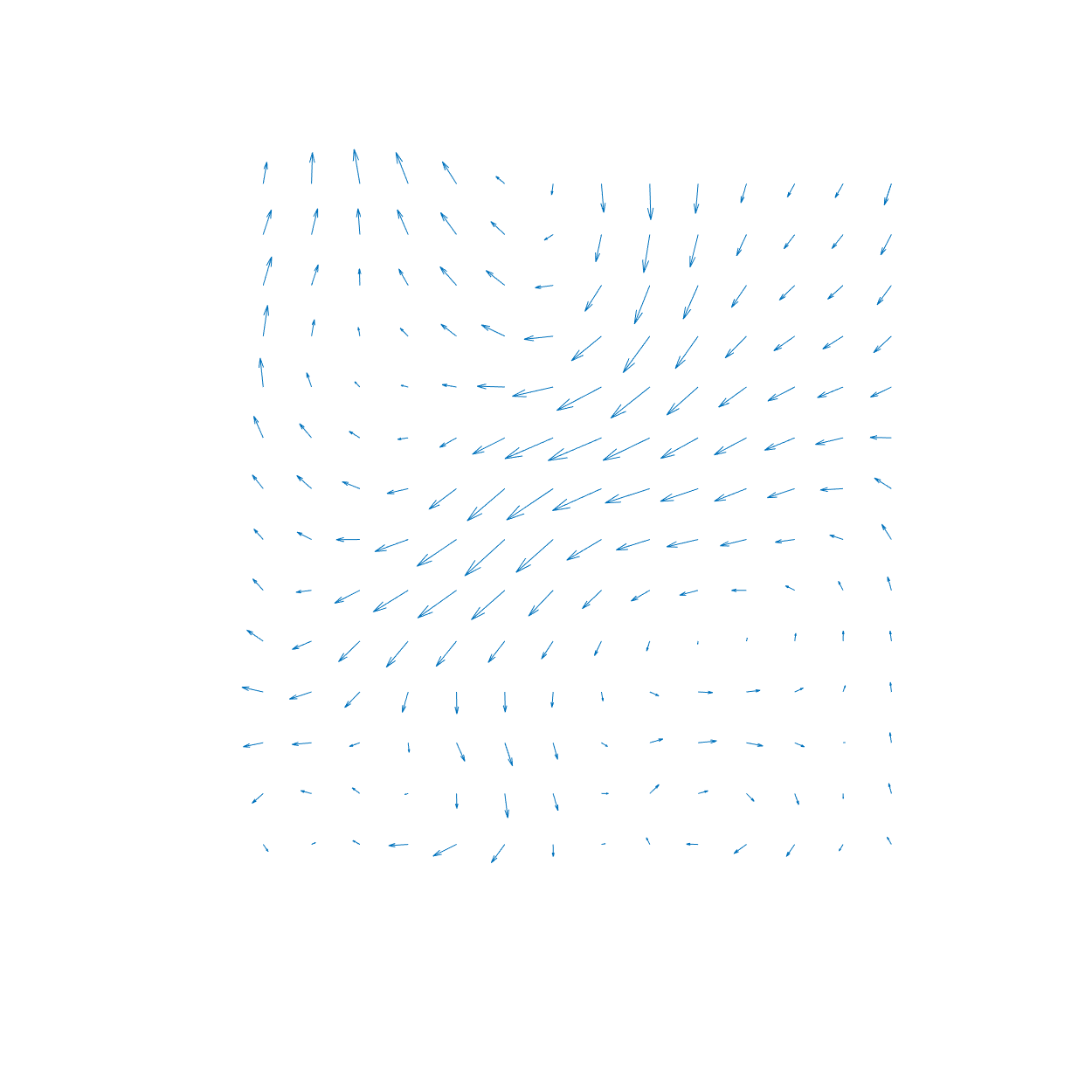

Supplement: S1 MCG raw data 1 — The raw MCG dataset includes categories 0-4 for testing. (ZIP) [file pone.0338189.s001.zip › test/2/p4_465_4.png]

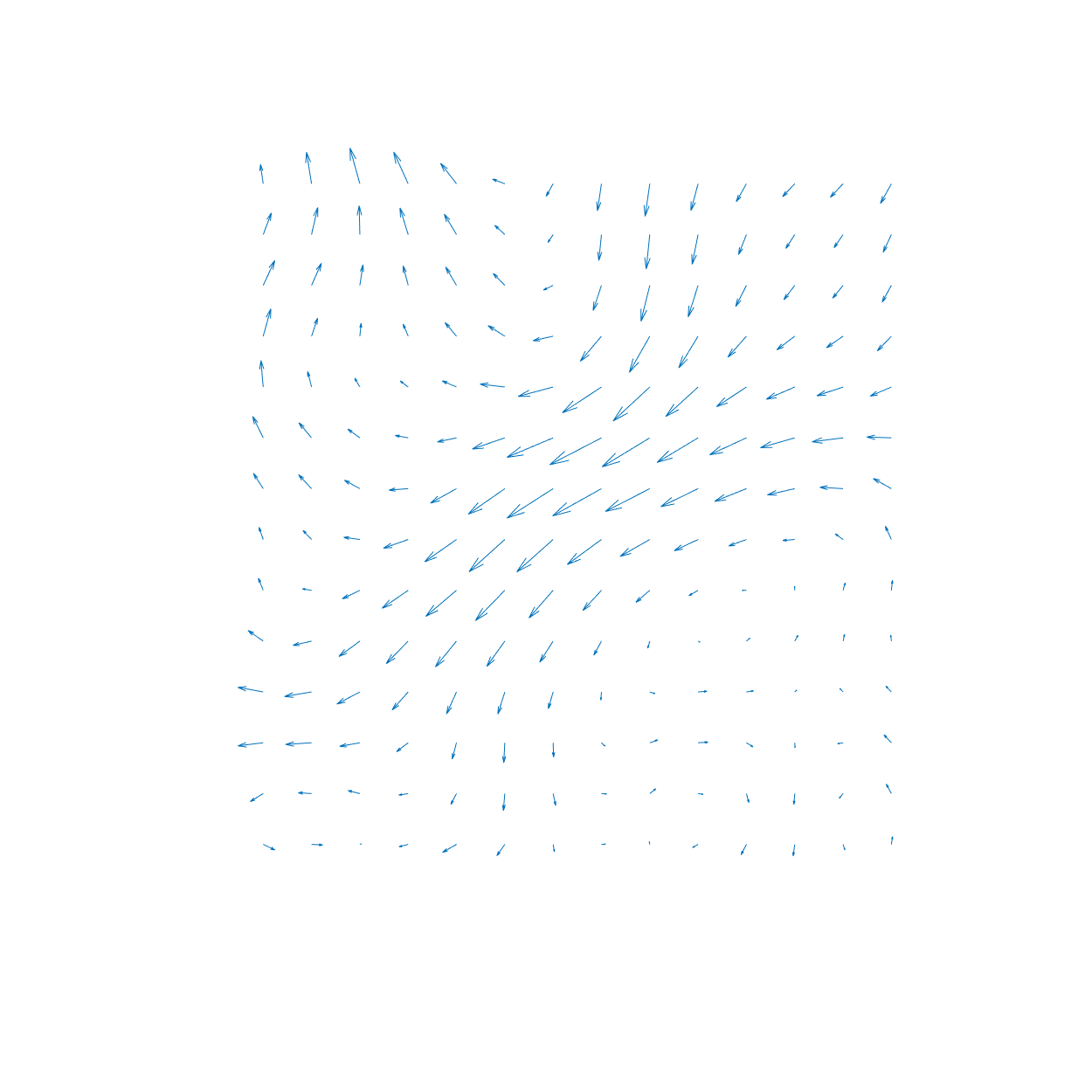

Supplement: S1 MCG raw data 1 — The raw MCG dataset includes categories 0-4 for testing. (ZIP) [file pone.0338189.s001.zip › test/2/p4_470_4.png]
