# Supplementary figures and images for: Classification of current density vector map using transformer hybrid residual network (part 1 of 6)
Source: PLoS One. 2025 Dec 16;20(12):e0338189. doi: 10.1371/journal.pone.0338189 (PMC12707687; doi:10.1371/journal.pone.0338189)

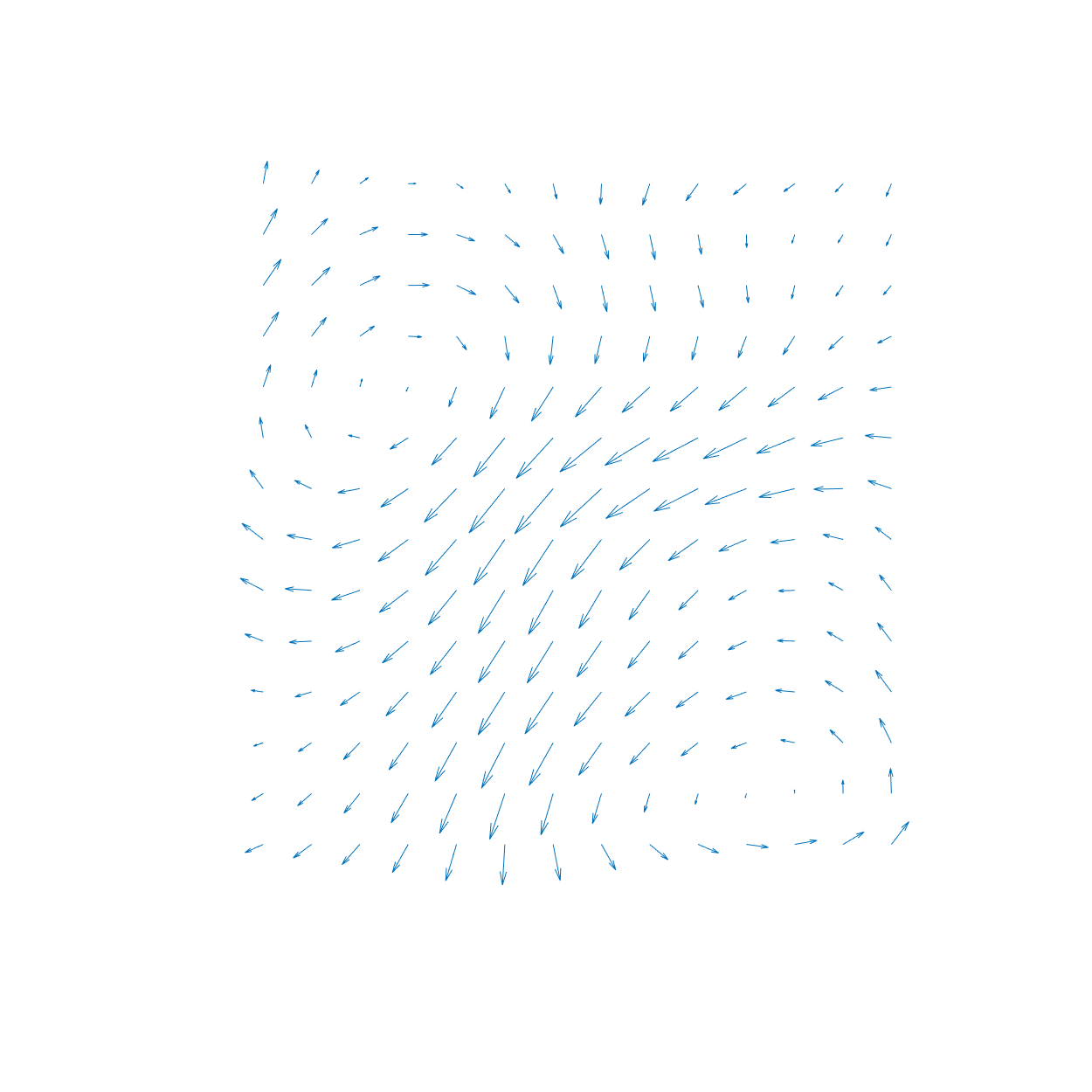

Supplement: S1 MCG raw data 1 — The raw MCG dataset includes categories 0-4 for testing. (ZIP) [file pone.0338189.s001.zip › test/0/p11_245_4.png]

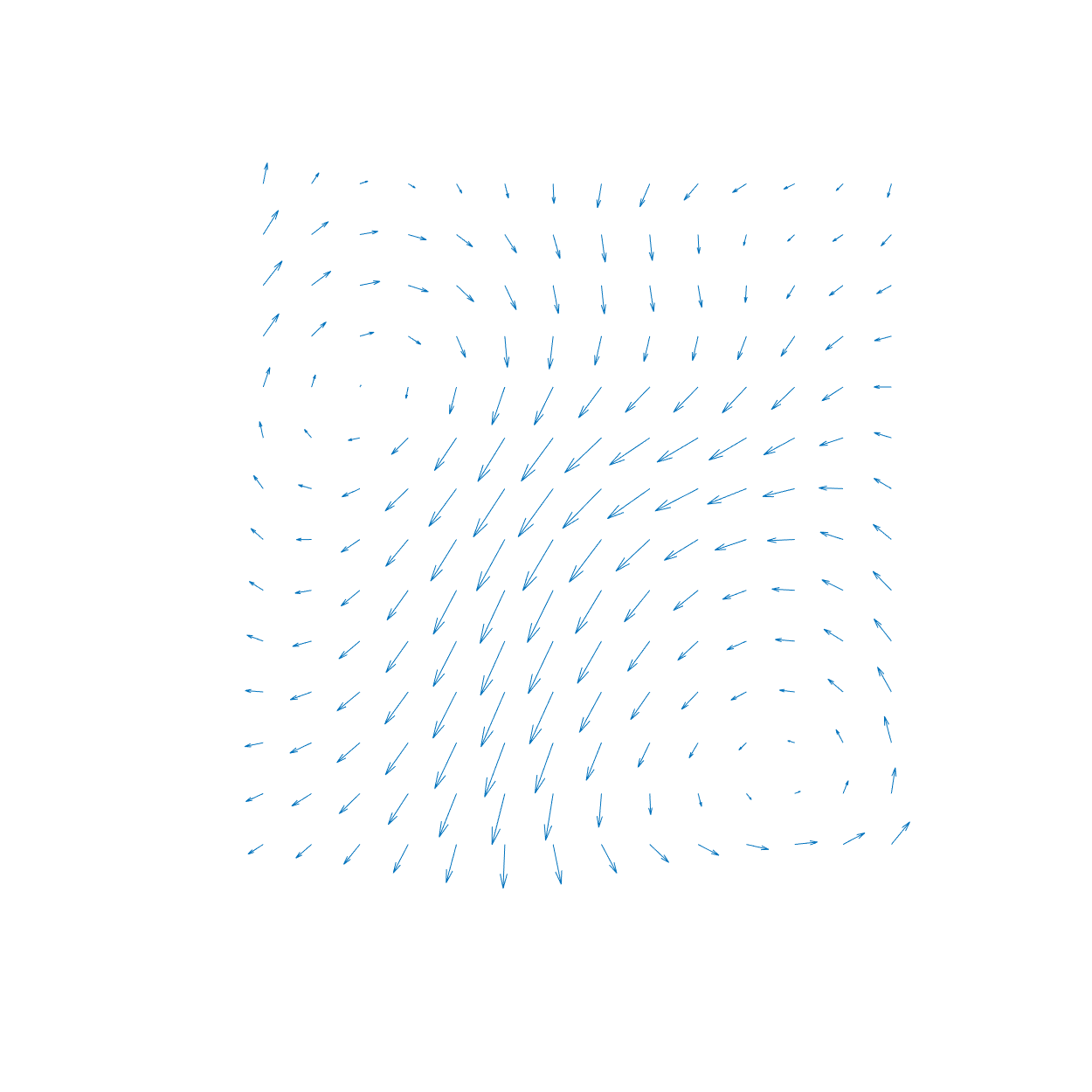

Supplement: S1 MCG raw data 1 — The raw MCG dataset includes categories 0-4 for testing. (ZIP) [file pone.0338189.s001.zip › test/0/p11_250_4.png]

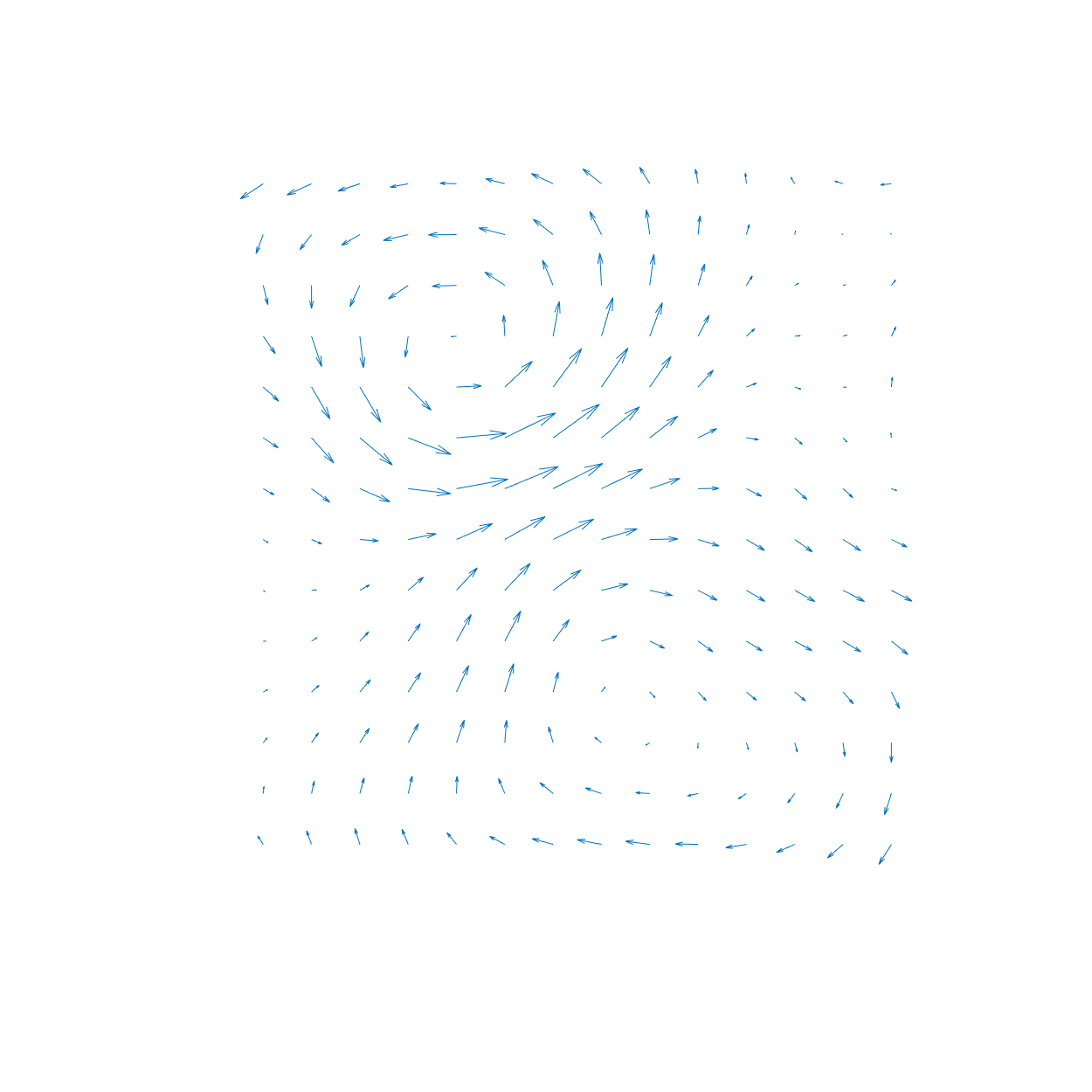

Supplement: S1 MCG raw data 1 — The raw MCG dataset includes categories 0-4 for testing. (ZIP) [file pone.0338189.s001.zip › test/0/p2_365_4.png]

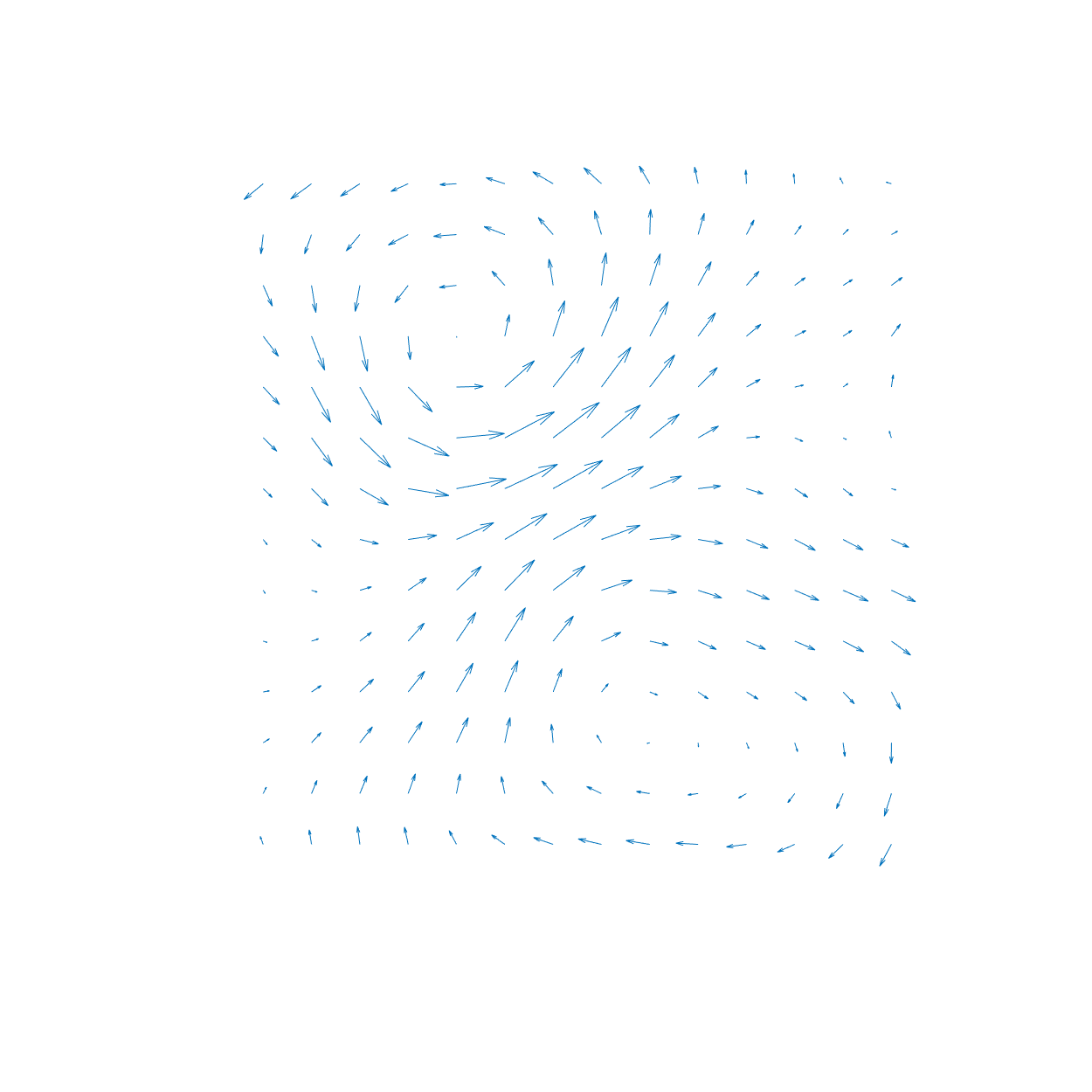

Supplement: S1 MCG raw data 1 — The raw MCG dataset includes categories 0-4 for testing. (ZIP) [file pone.0338189.s001.zip › test/0/p2_370_4.png]

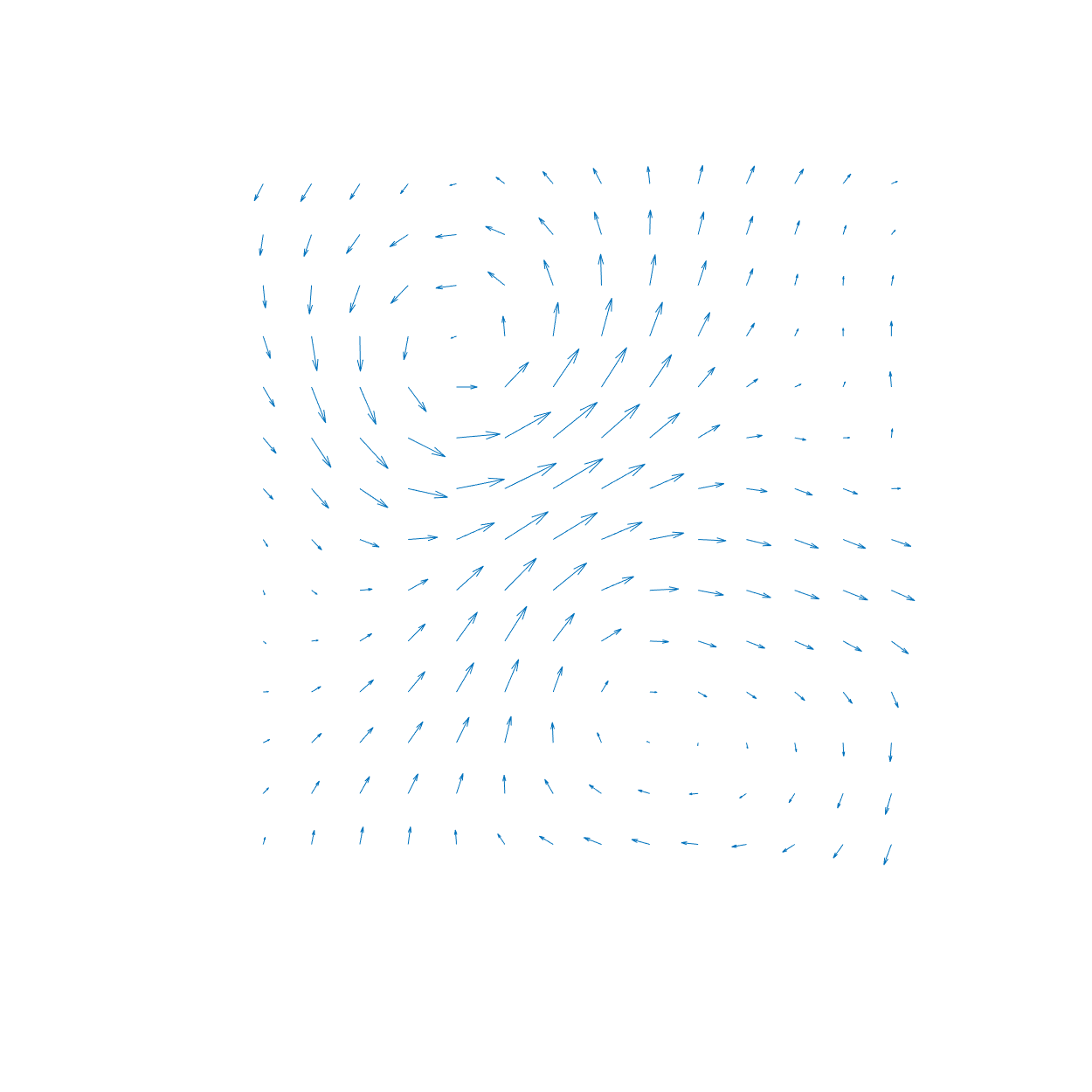

Supplement: S1 MCG raw data 1 — The raw MCG dataset includes categories 0-4 for testing. (ZIP) [file pone.0338189.s001.zip › test/0/p2_375_4.png]

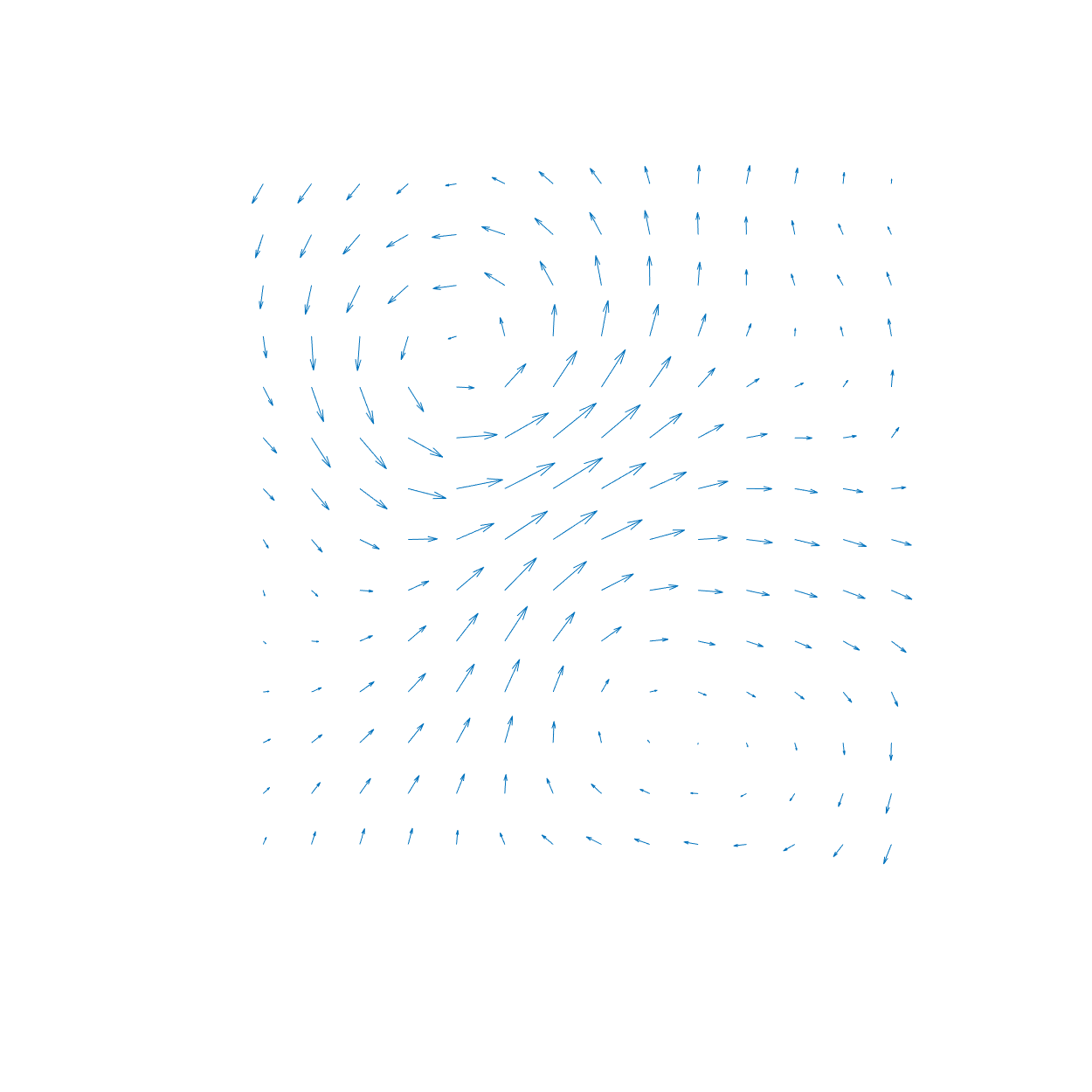

Supplement: S1 MCG raw data 1 — The raw MCG dataset includes categories 0-4 for testing. (ZIP) [file pone.0338189.s001.zip › test/0/p2_380_4.png]

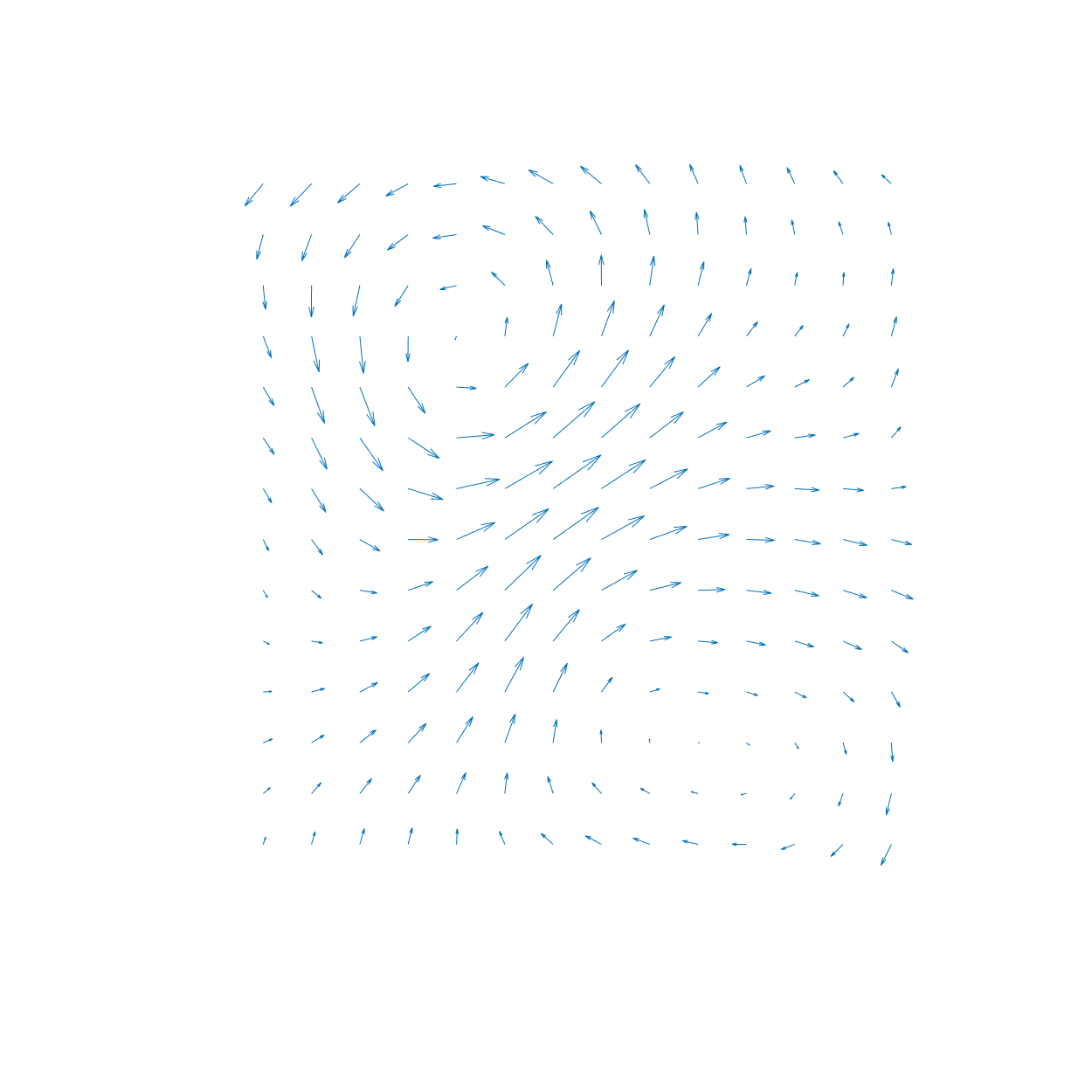

Supplement: S1 MCG raw data 1 — The raw MCG dataset includes categories 0-4 for testing. (ZIP) [file pone.0338189.s001.zip › test/0/p2_385_4.png]

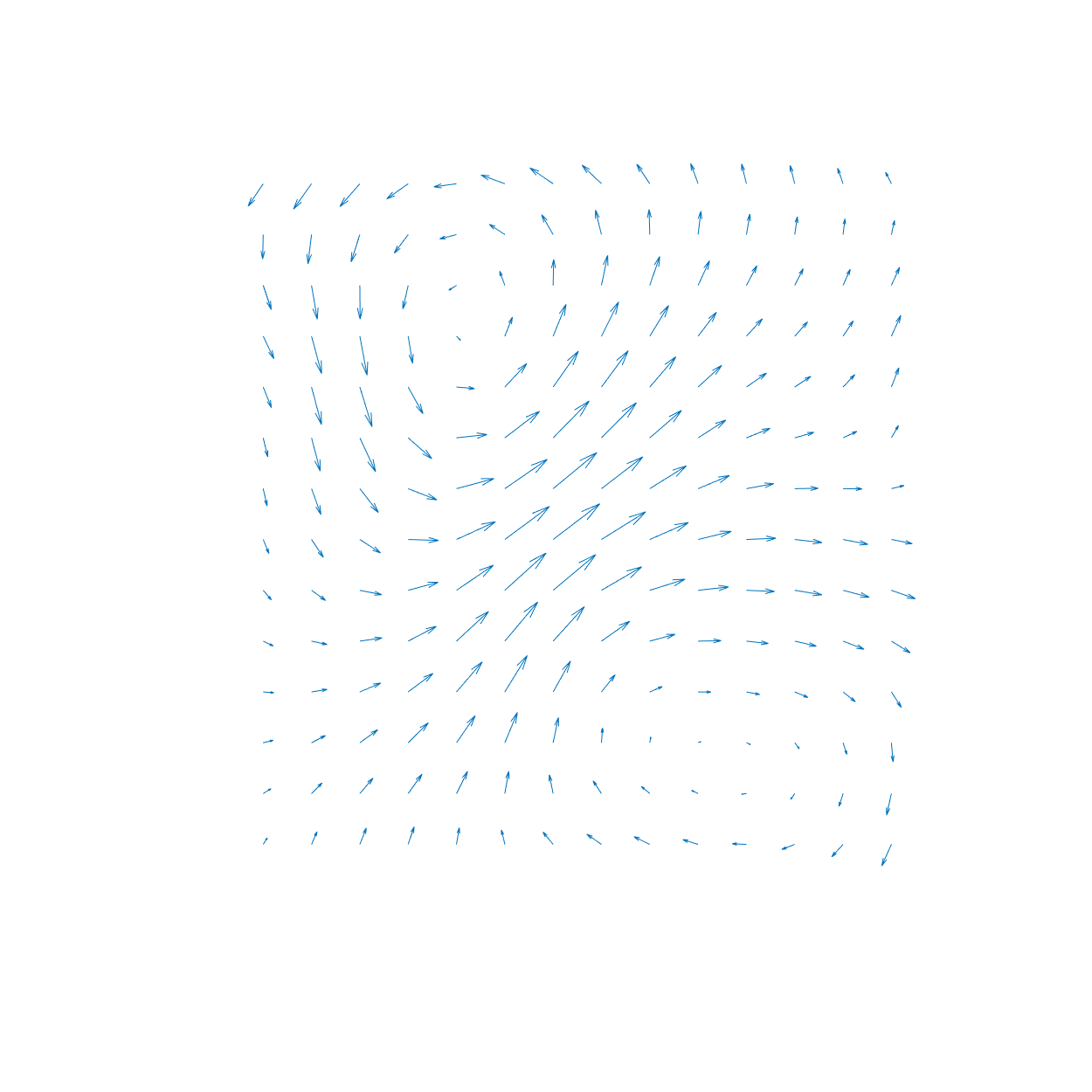

Supplement: S1 MCG raw data 1 — The raw MCG dataset includes categories 0-4 for testing. (ZIP) [file pone.0338189.s001.zip › test/0/p2_390_4.png]

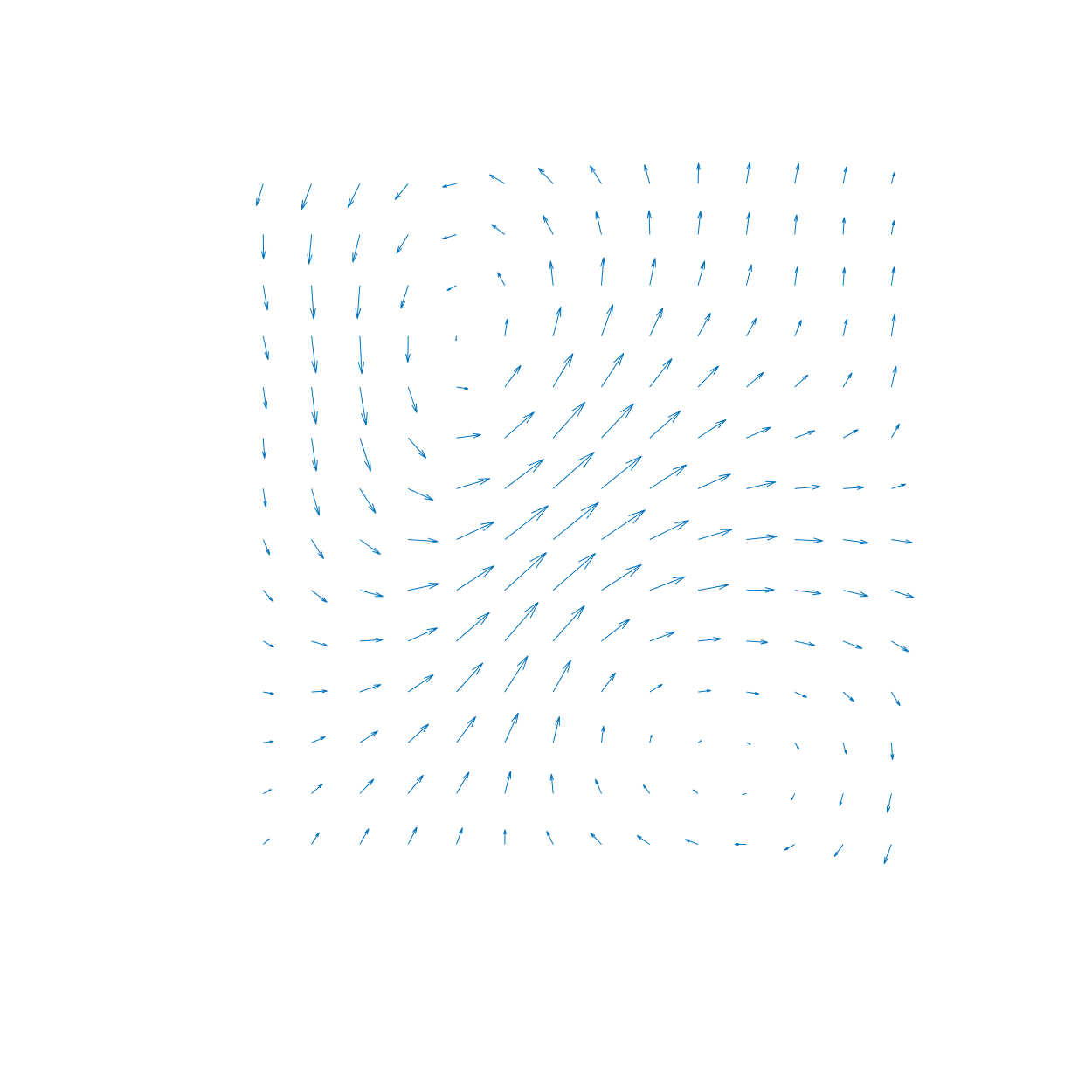

Supplement: S1 MCG raw data 1 — The raw MCG dataset includes categories 0-4 for testing. (ZIP) [file pone.0338189.s001.zip › test/0/p2_395_4.png]

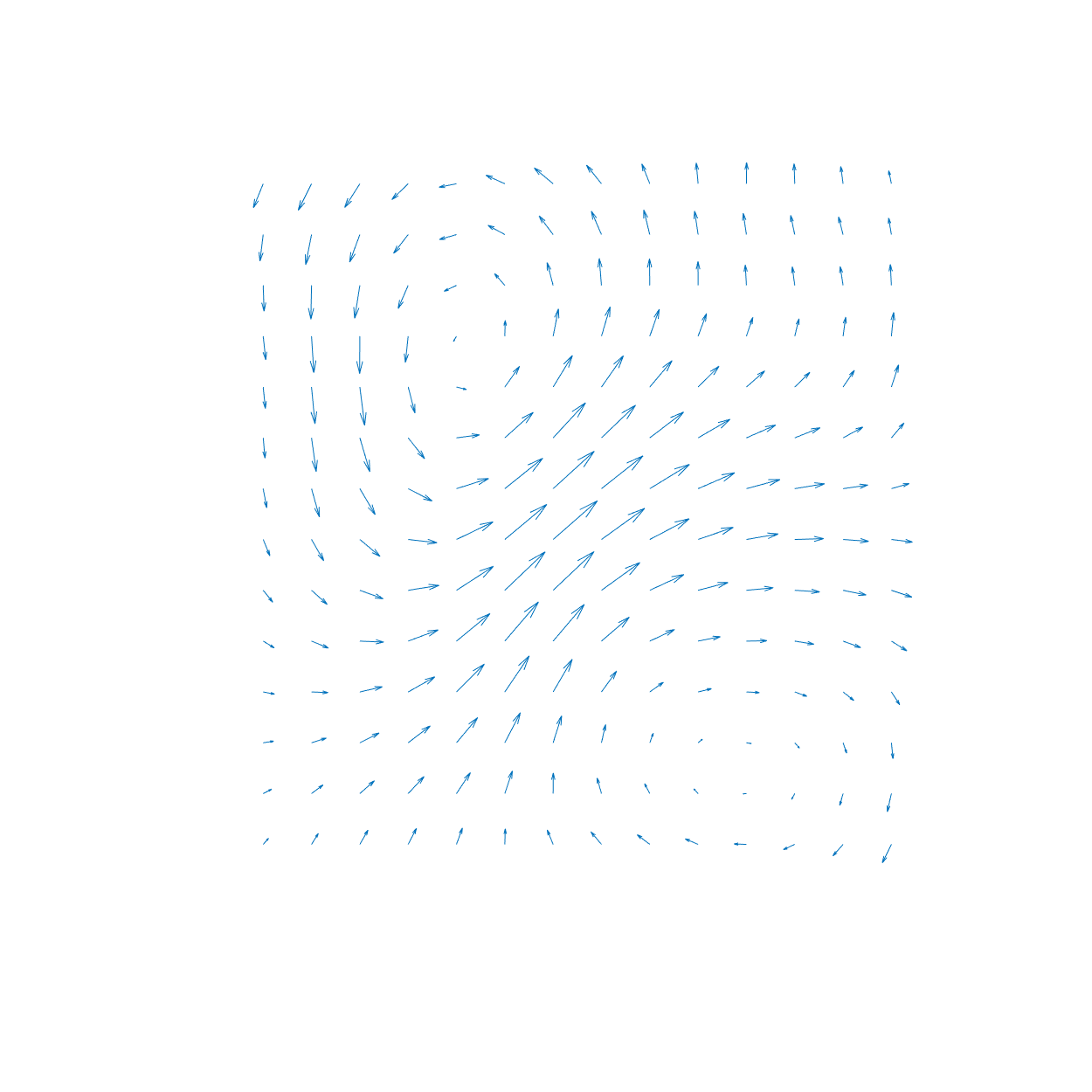

Supplement: S1 MCG raw data 1 — The raw MCG dataset includes categories 0-4 for testing. (ZIP) [file pone.0338189.s001.zip › test/0/p2_400_4.png]

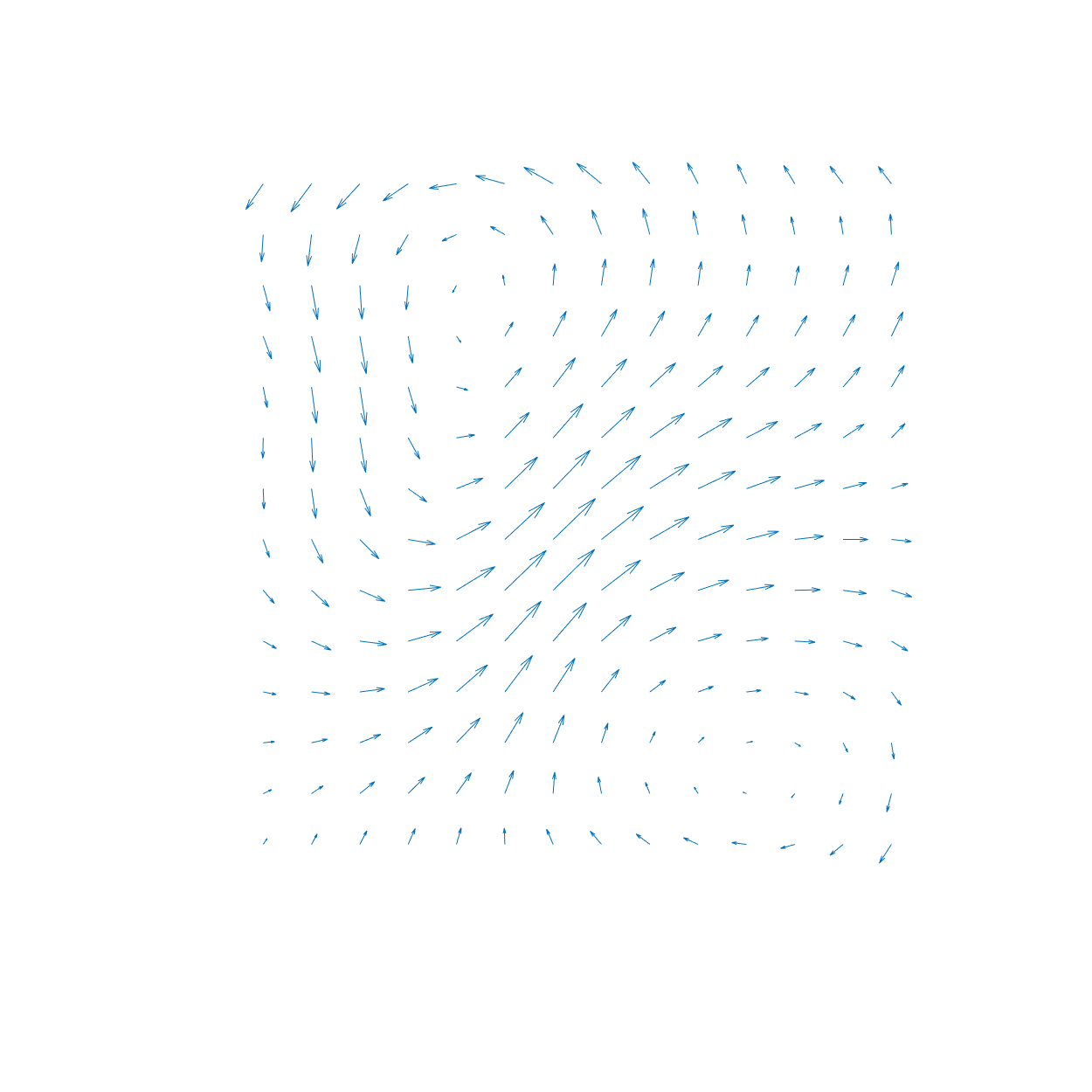

Supplement: S1 MCG raw data 1 — The raw MCG dataset includes categories 0-4 for testing. (ZIP) [file pone.0338189.s001.zip › test/0/p2_405_4.png]

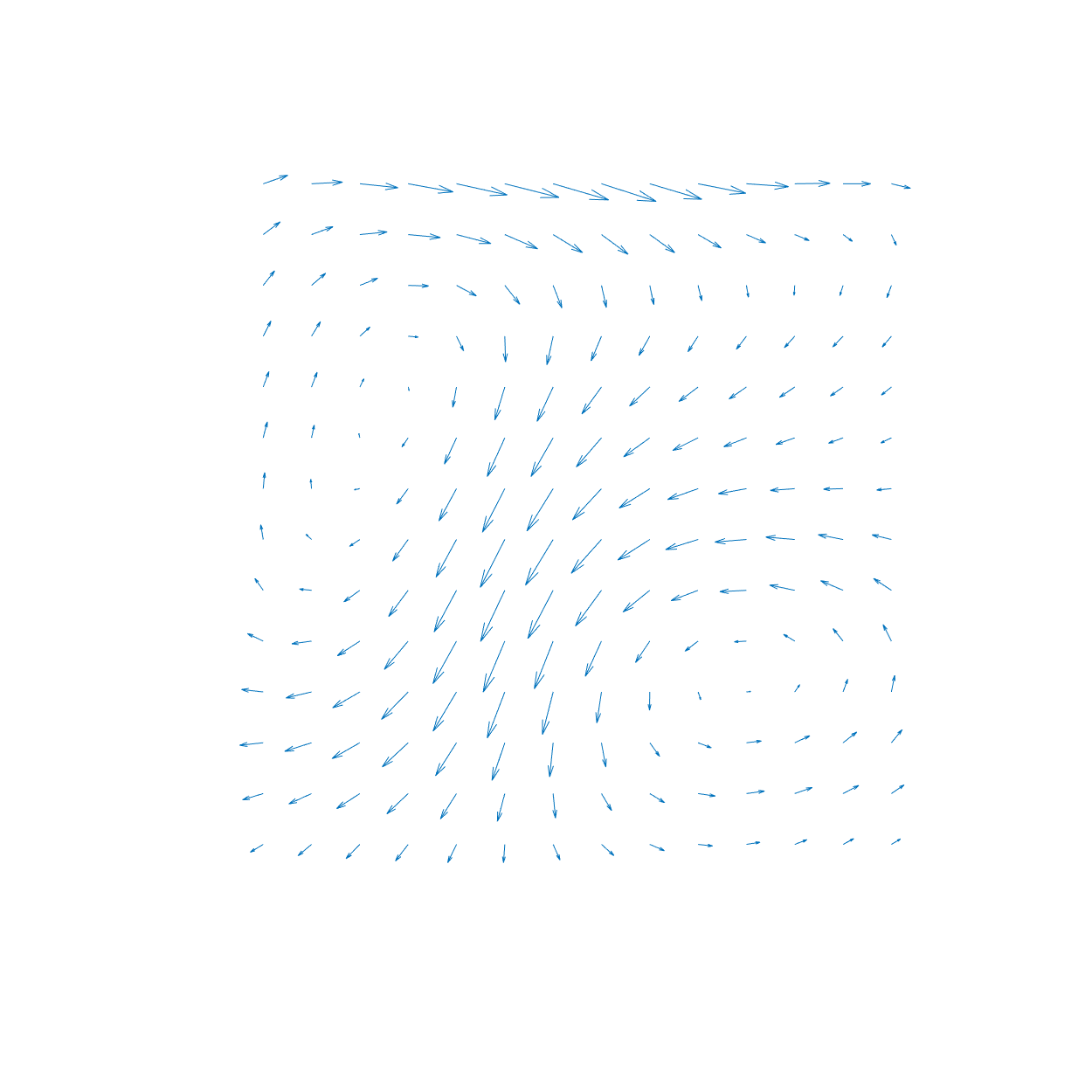

Supplement: S1 MCG raw data 1 — The raw MCG dataset includes categories 0-4 for testing. (ZIP) [file pone.0338189.s001.zip › test/0/p3_235_4.png]

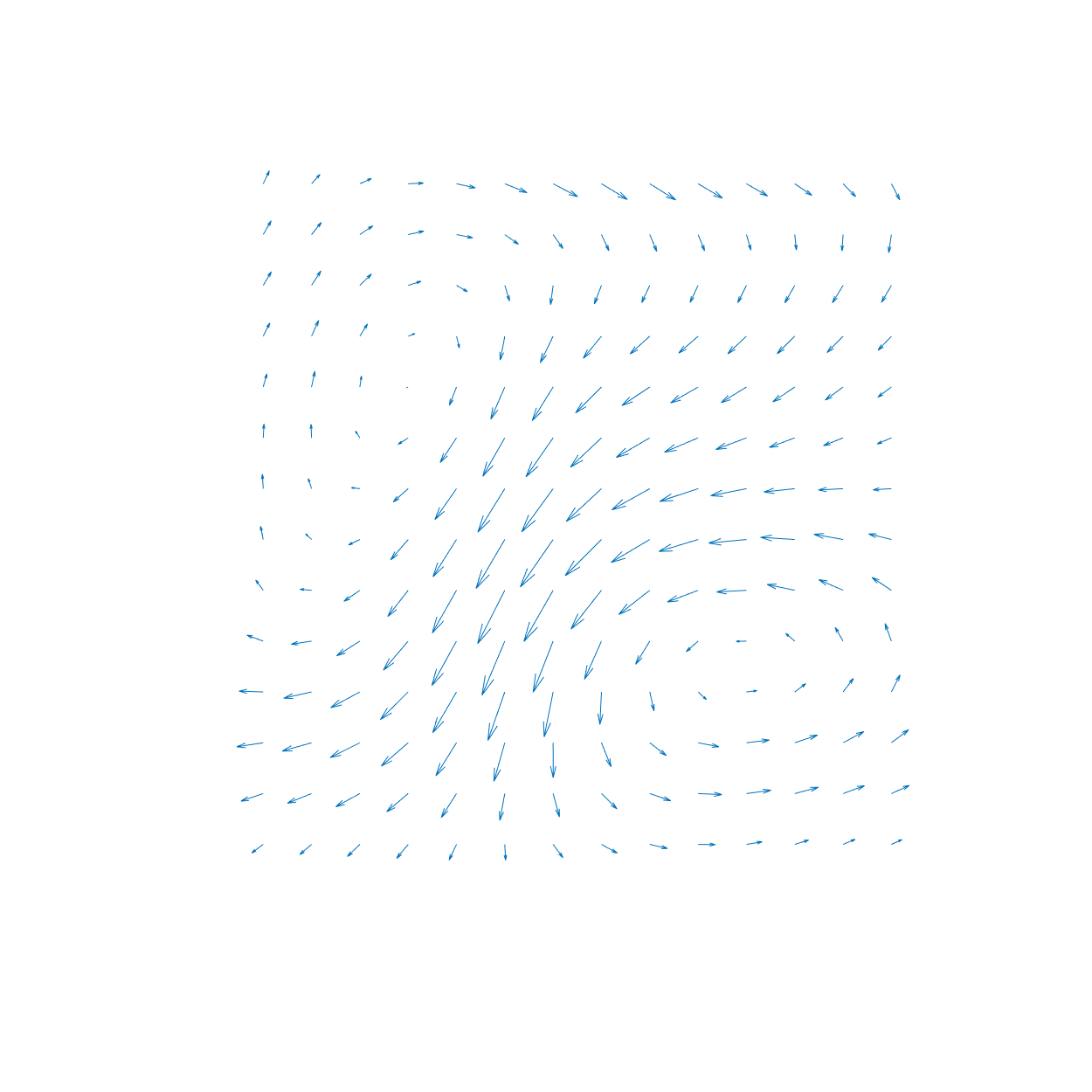

Supplement: S1 MCG raw data 1 — The raw MCG dataset includes categories 0-4 for testing. (ZIP) [file pone.0338189.s001.zip › test/0/p3_240_4.png]

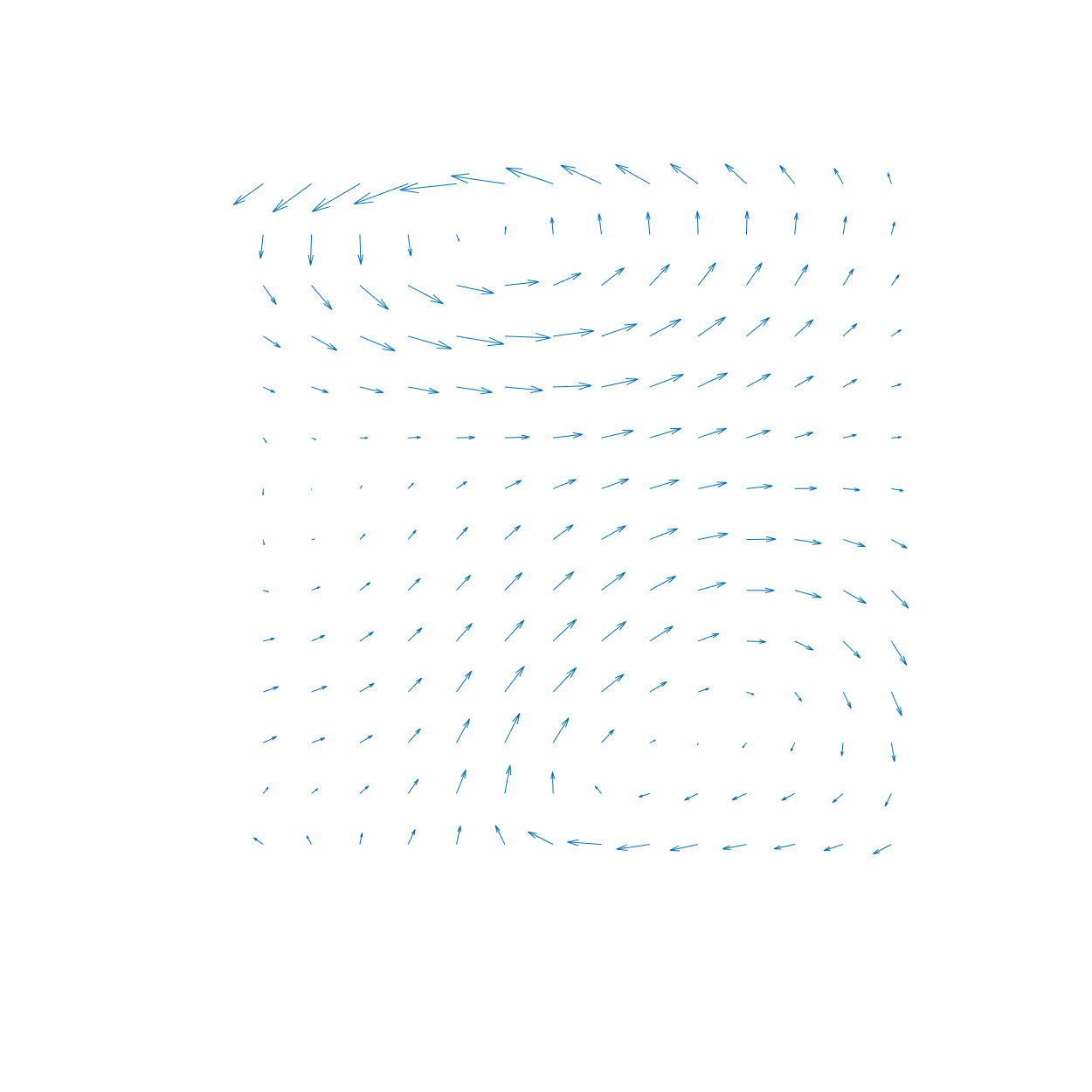

Supplement: S1 MCG raw data 1 — The raw MCG dataset includes categories 0-4 for testing. (ZIP) [file pone.0338189.s001.zip › test/0/p3_435_4.png]

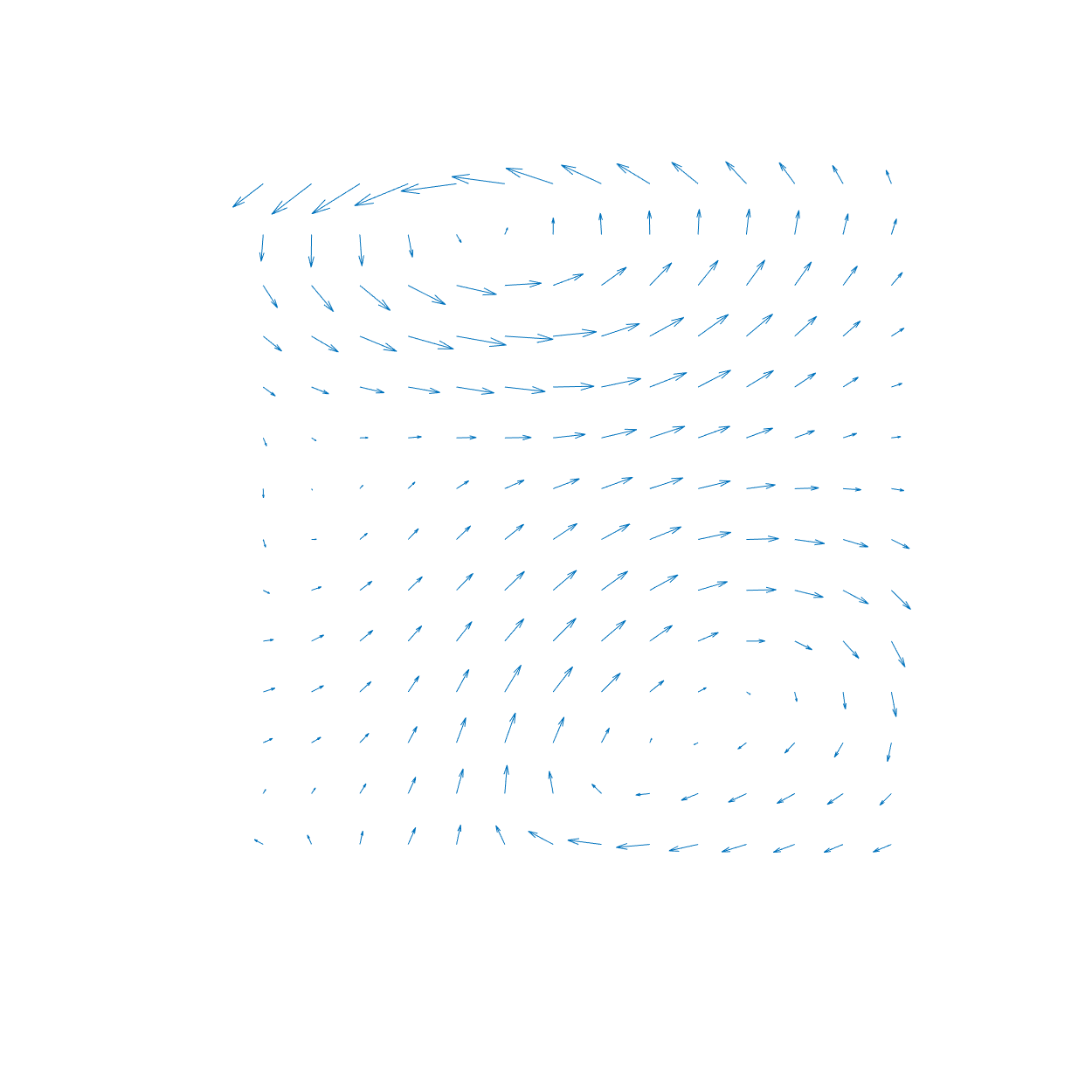

Supplement: S1 MCG raw data 1 — The raw MCG dataset includes categories 0-4 for testing. (ZIP) [file pone.0338189.s001.zip › test/0/p3_440_4.png]

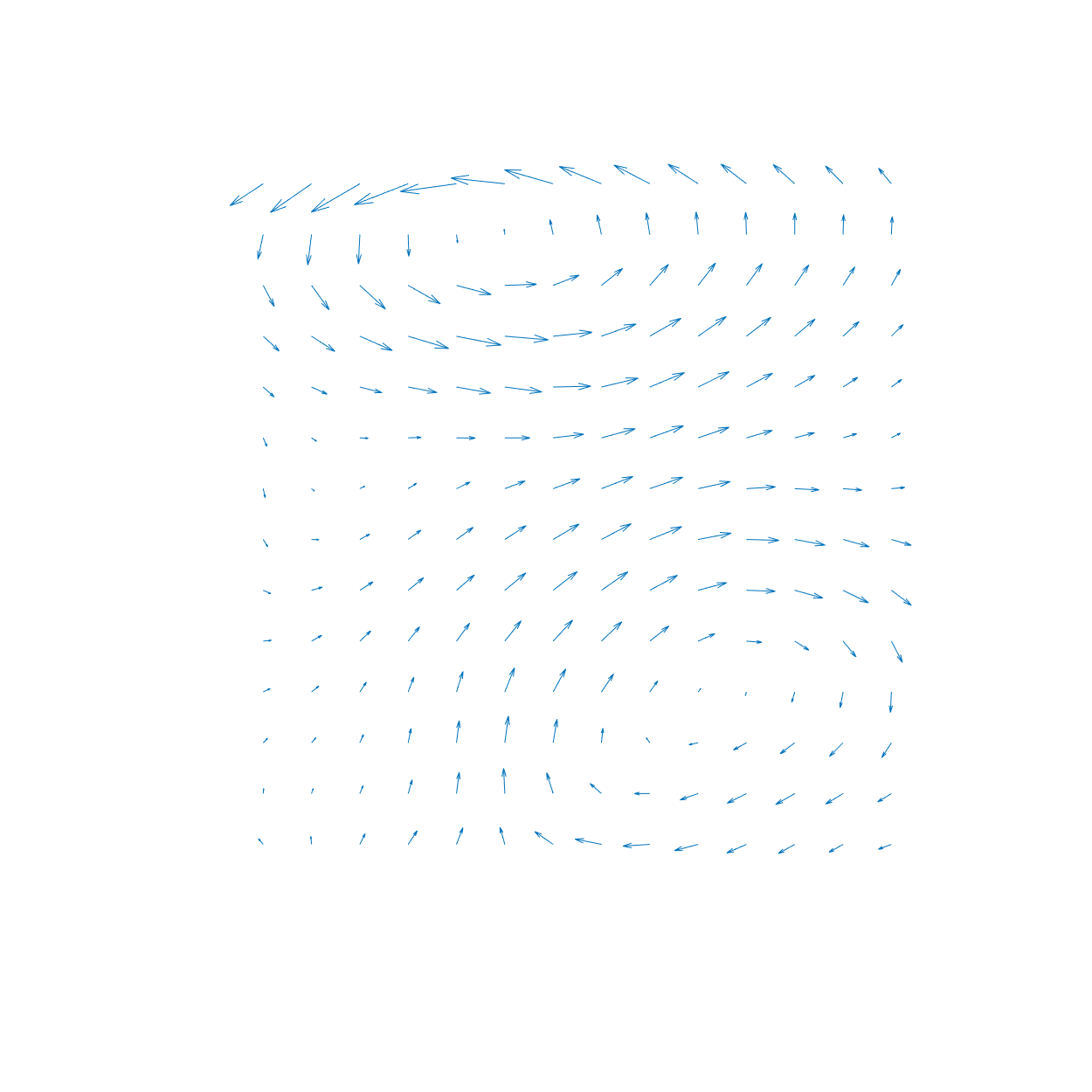

Supplement: S1 MCG raw data 1 — The raw MCG dataset includes categories 0-4 for testing. (ZIP) [file pone.0338189.s001.zip › test/0/p3_445_4.png]

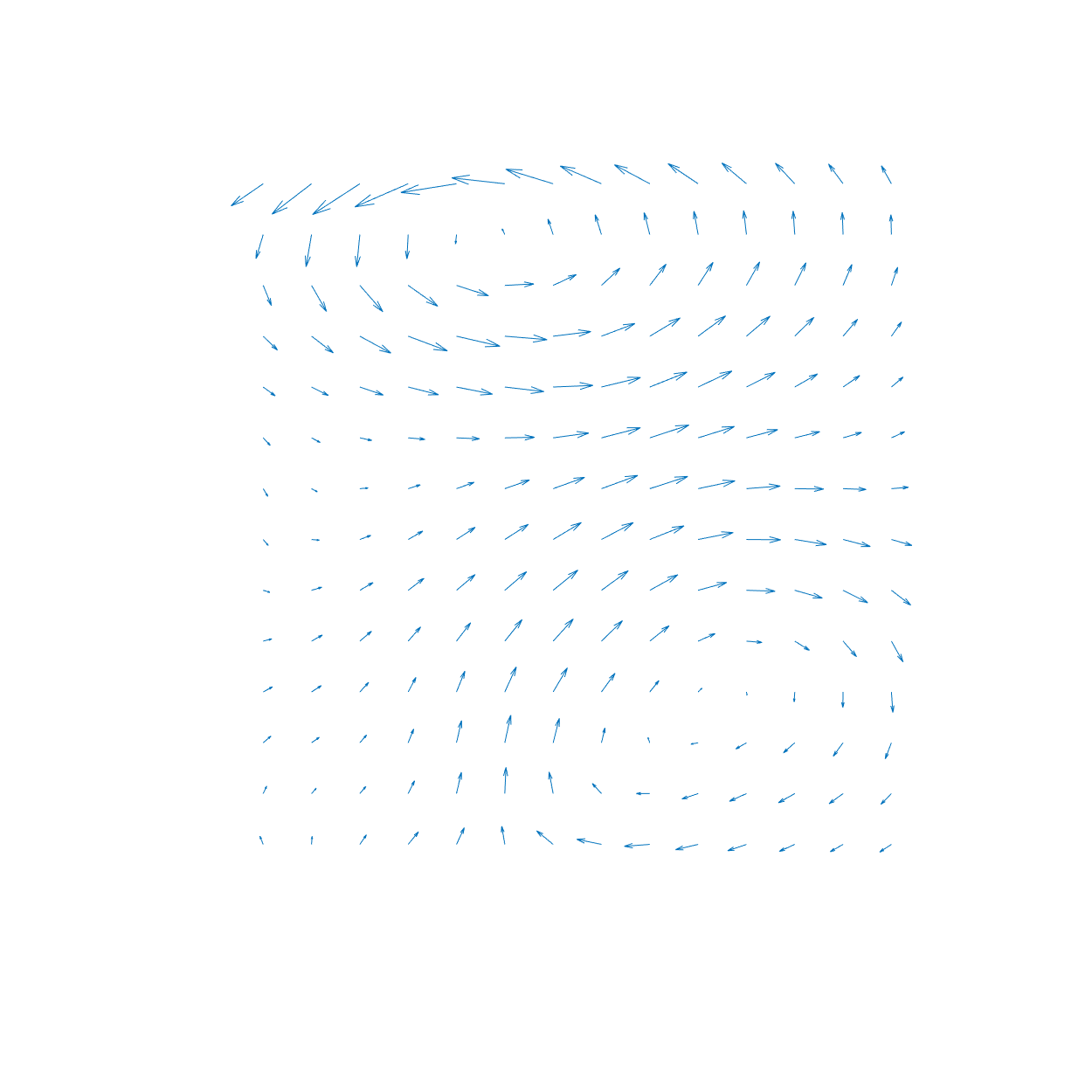

Supplement: S1 MCG raw data 1 — The raw MCG dataset includes categories 0-4 for testing. (ZIP) [file pone.0338189.s001.zip › test/0/p3_450_4.png]

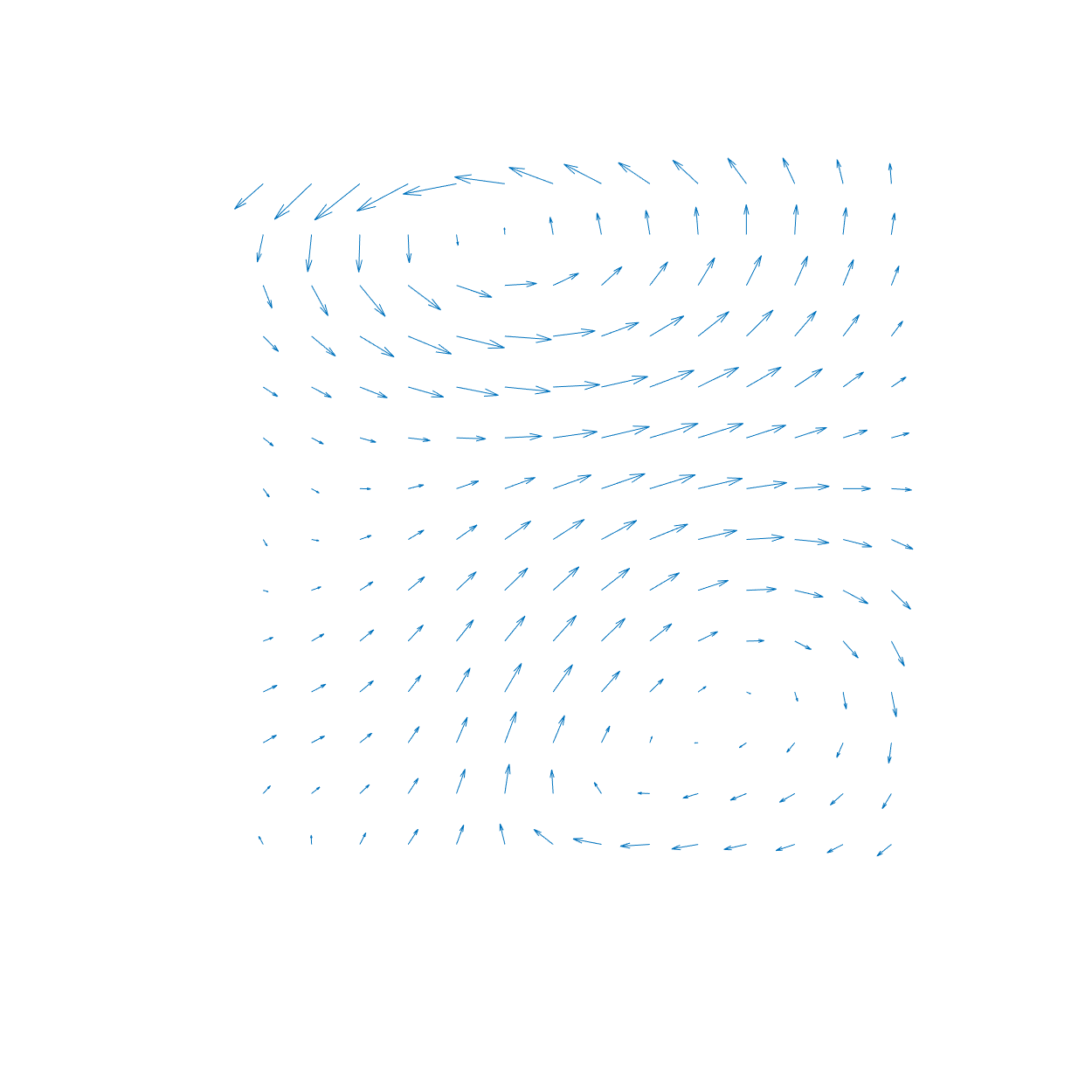

Supplement: S1 MCG raw data 1 — The raw MCG dataset includes categories 0-4 for testing. (ZIP) [file pone.0338189.s001.zip › test/0/p3_455_4.png]

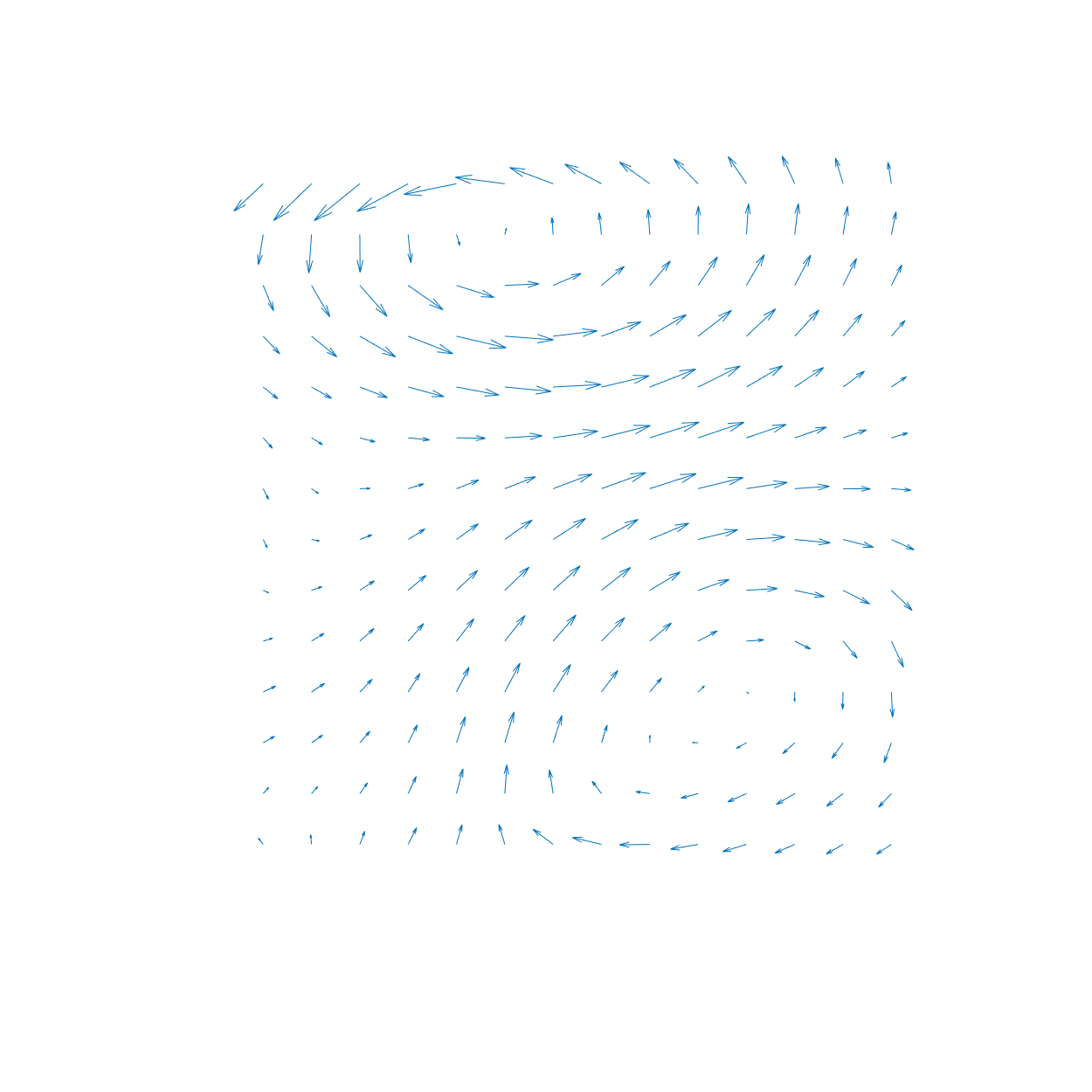

Supplement: S1 MCG raw data 1 — The raw MCG dataset includes categories 0-4 for testing. (ZIP) [file pone.0338189.s001.zip › test/0/p3_460_4.png]

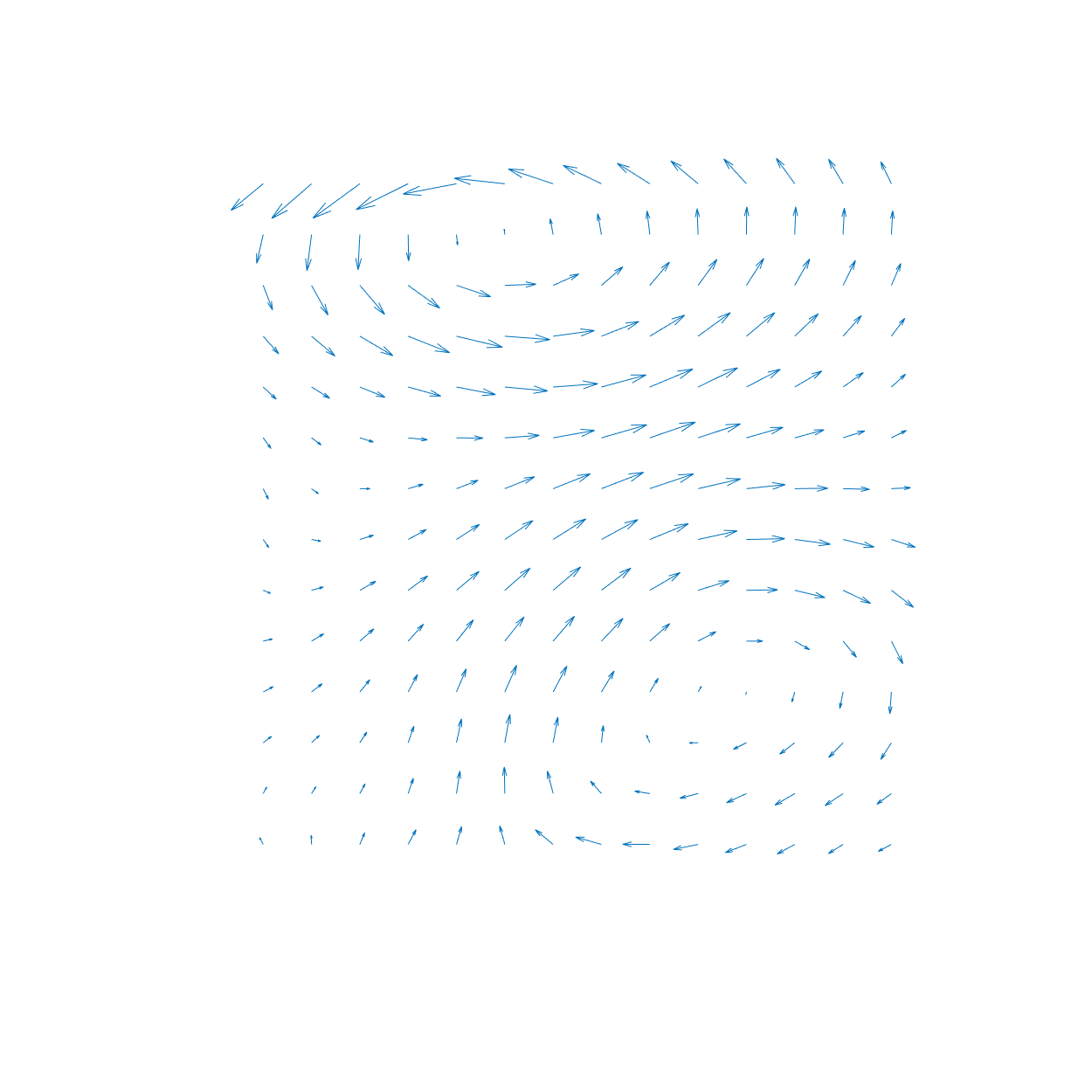

Supplement: S1 MCG raw data 1 — The raw MCG dataset includes categories 0-4 for testing. (ZIP) [file pone.0338189.s001.zip › test/0/p3_465_4.png]

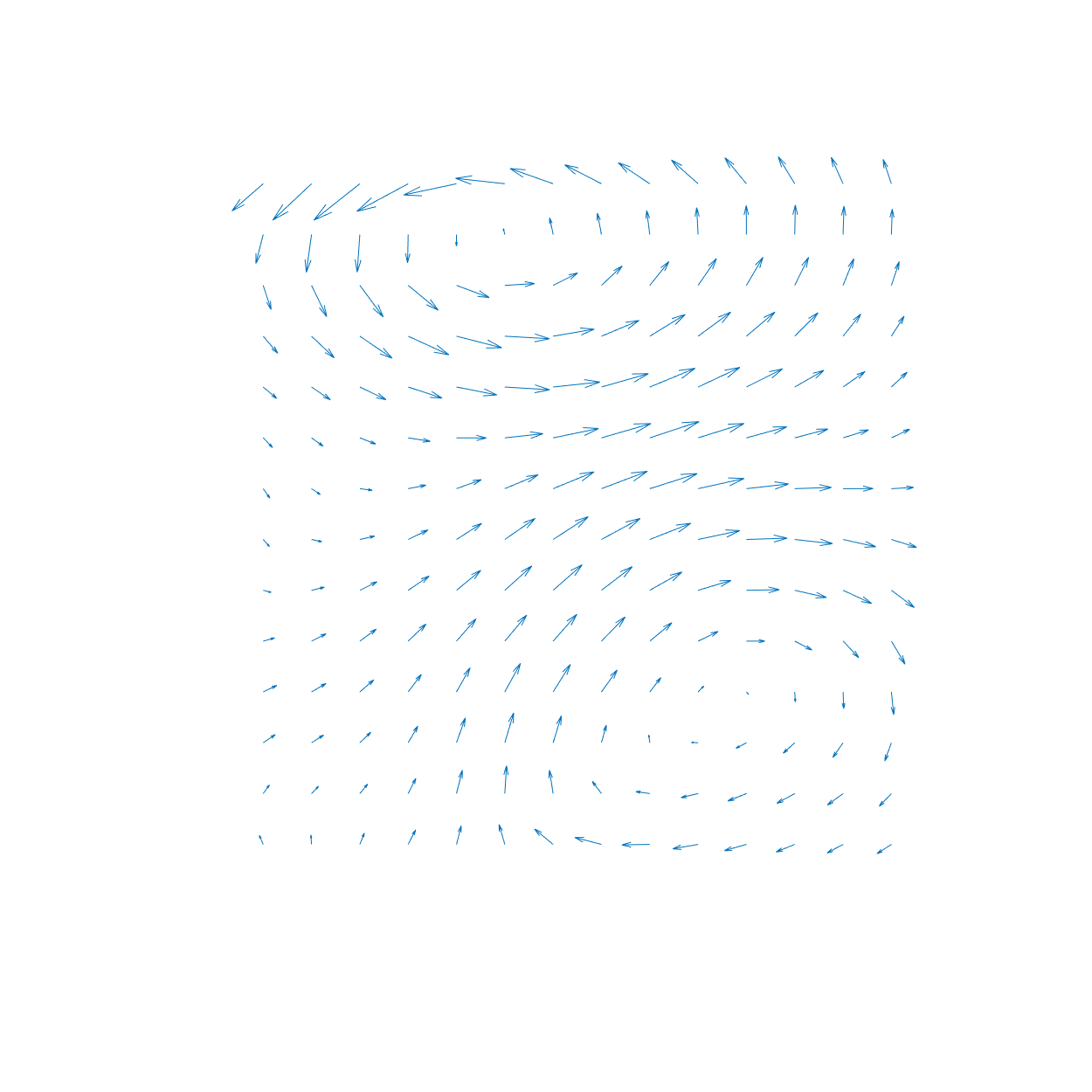

Supplement: S1 MCG raw data 1 — The raw MCG dataset includes categories 0-4 for testing. (ZIP) [file pone.0338189.s001.zip › test/0/p3_470_4.png]

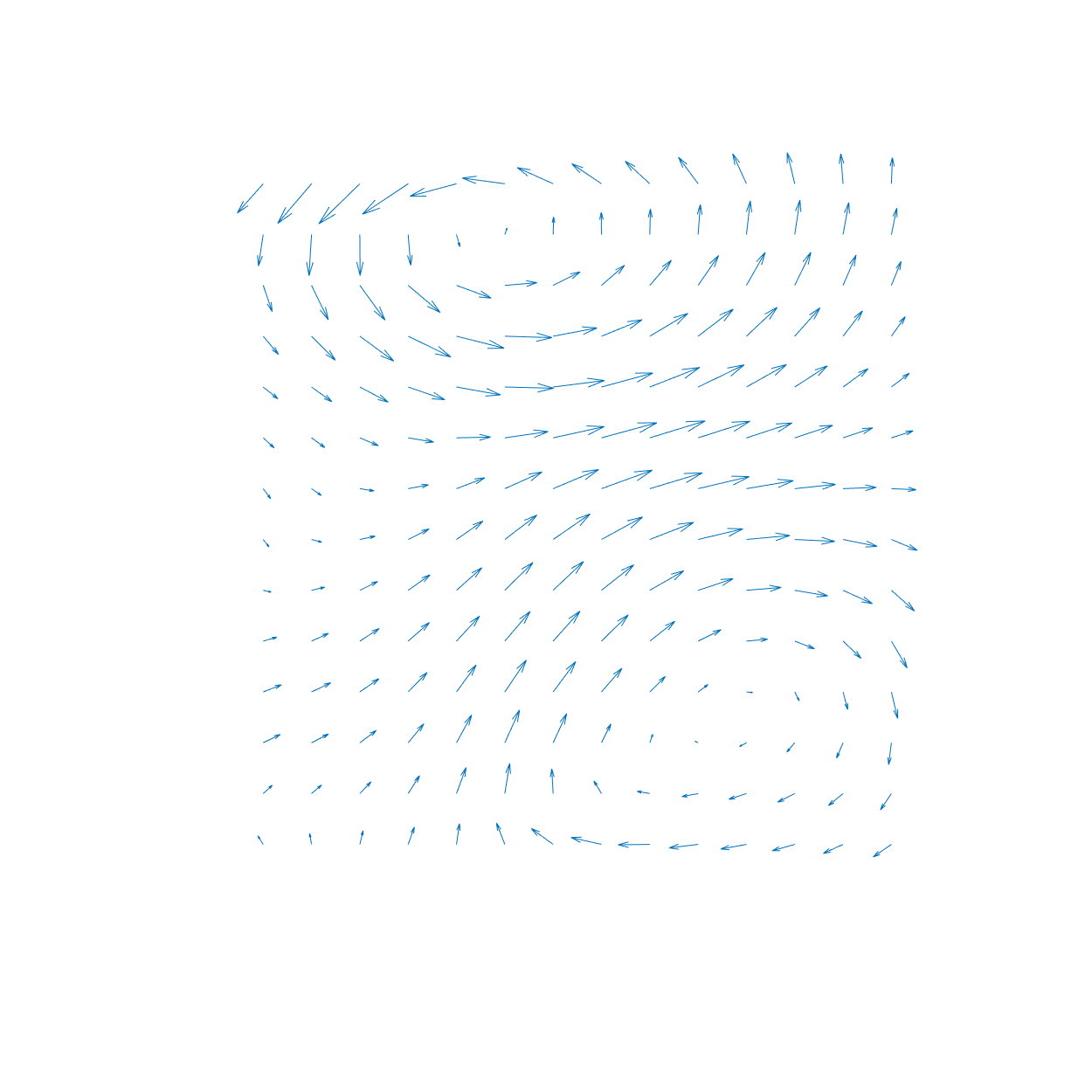

Supplement: S1 MCG raw data 1 — The raw MCG dataset includes categories 0-4 for testing. (ZIP) [file pone.0338189.s001.zip › test/0/p3_475_4.png]

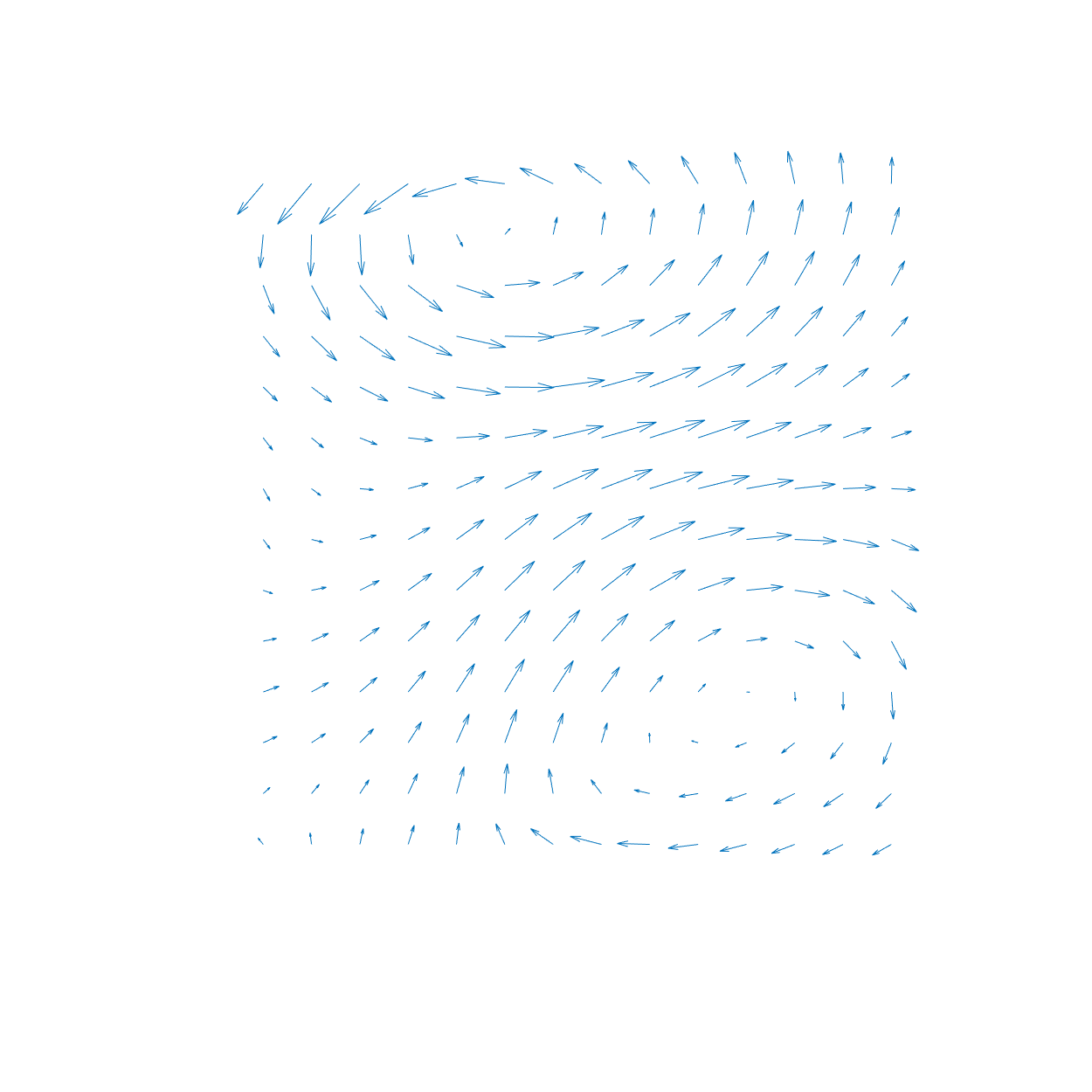

Supplement: S1 MCG raw data 1 — The raw MCG dataset includes categories 0-4 for testing. (ZIP) [file pone.0338189.s001.zip › test/0/p3_480_4.png]

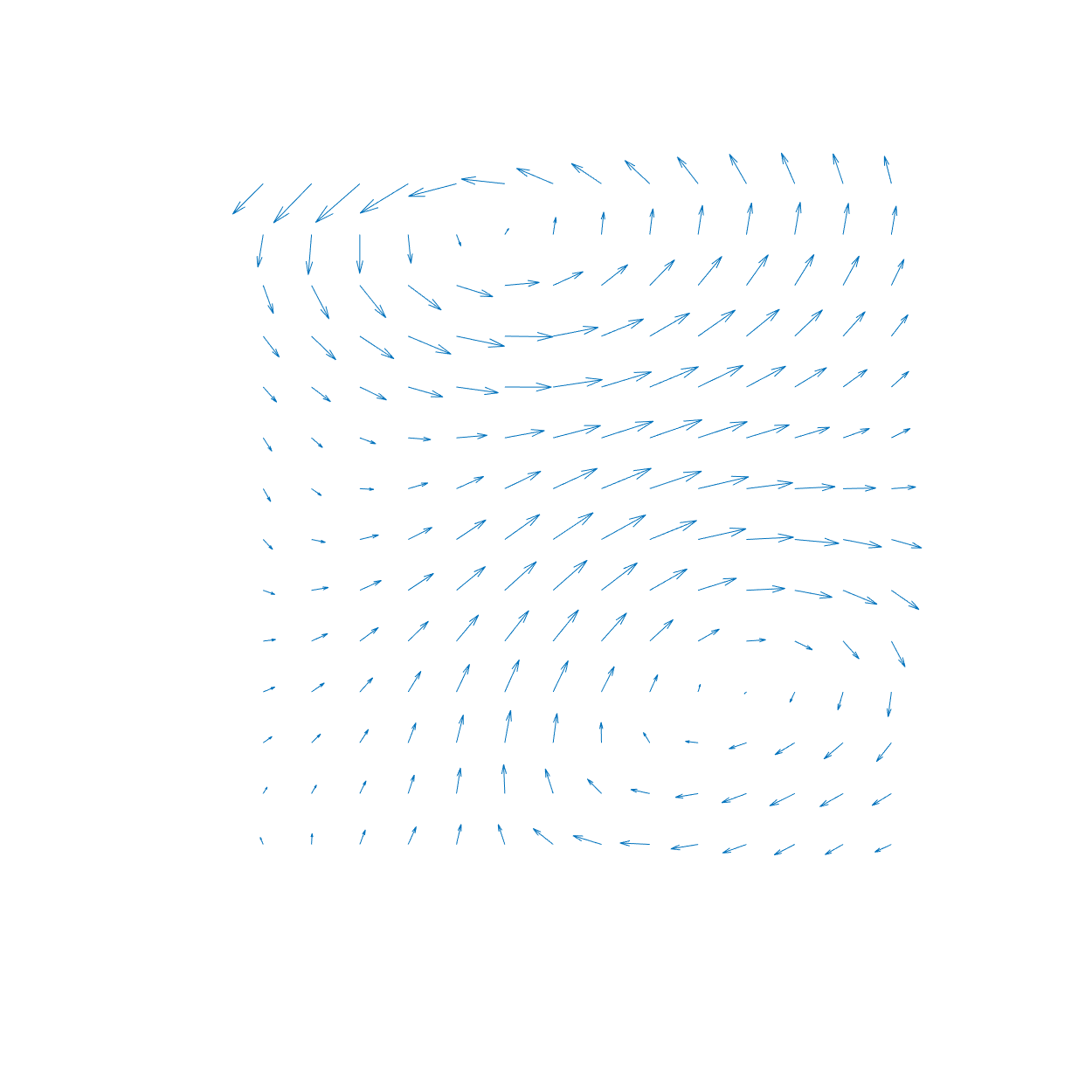

Supplement: S1 MCG raw data 1 — The raw MCG dataset includes categories 0-4 for testing. (ZIP) [file pone.0338189.s001.zip › test/0/p3_485_4.png]

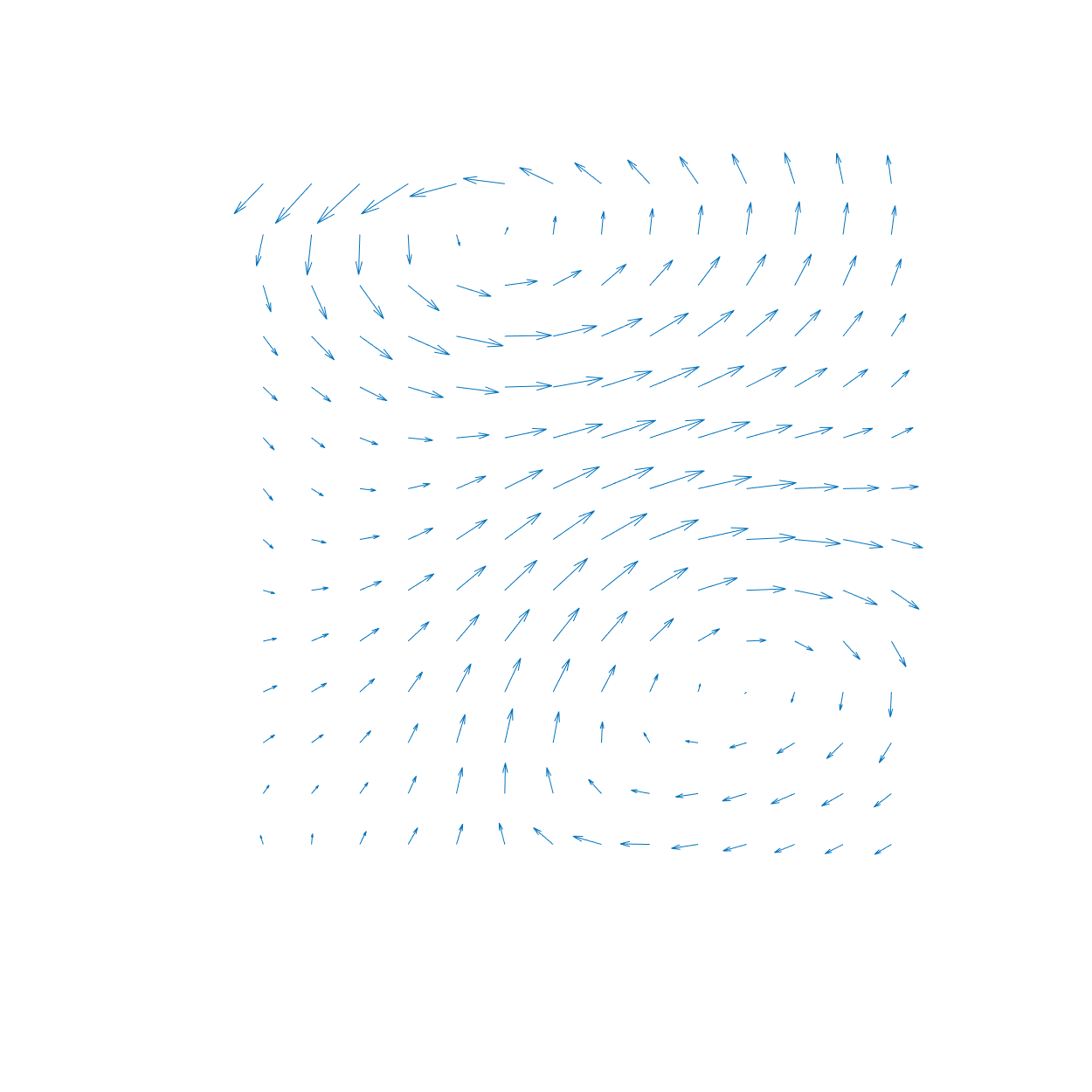

Supplement: S1 MCG raw data 1 — The raw MCG dataset includes categories 0-4 for testing. (ZIP) [file pone.0338189.s001.zip › test/0/p3_490_4.png]

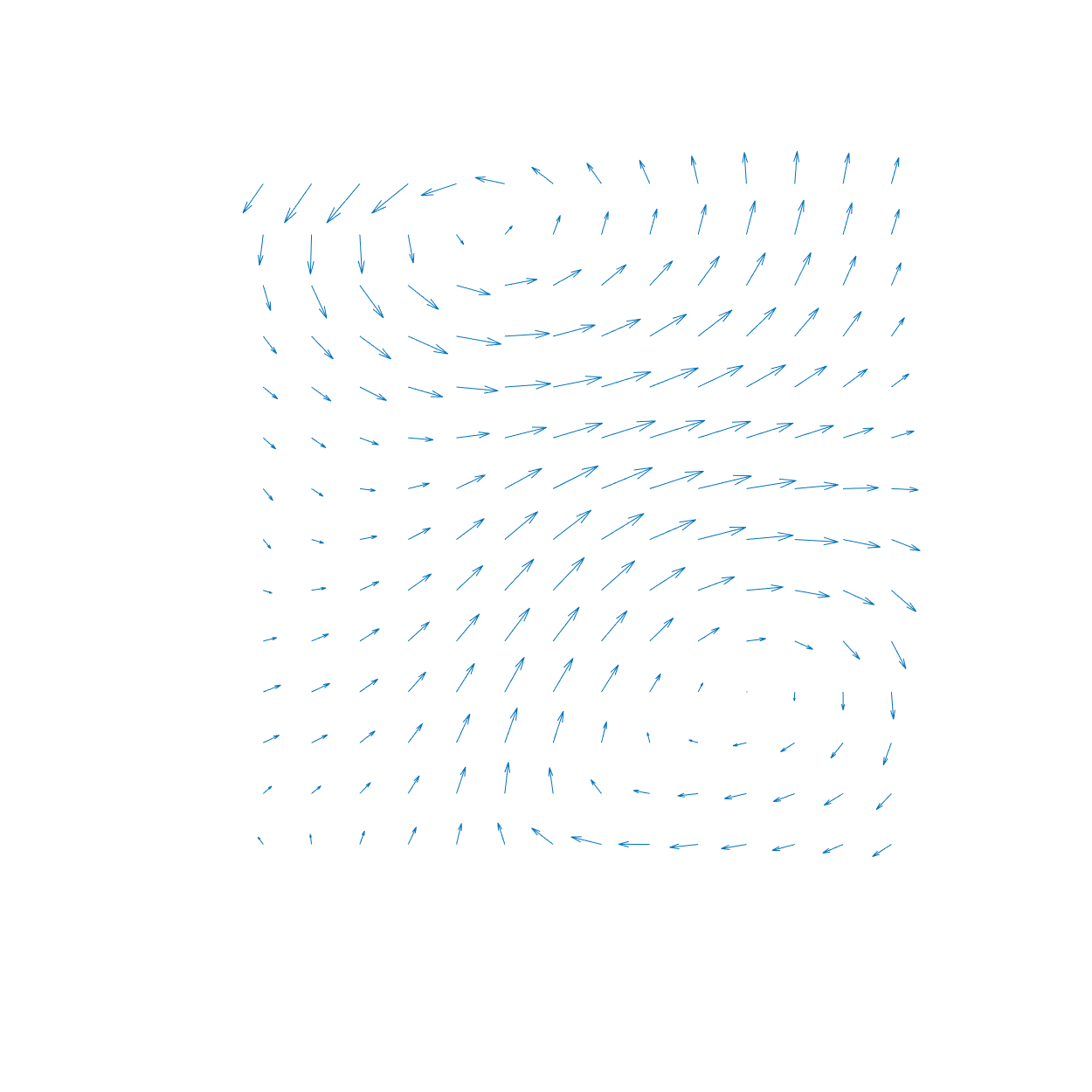

Supplement: S1 MCG raw data 1 — The raw MCG dataset includes categories 0-4 for testing. (ZIP) [file pone.0338189.s001.zip › test/0/p3_495_4.png]

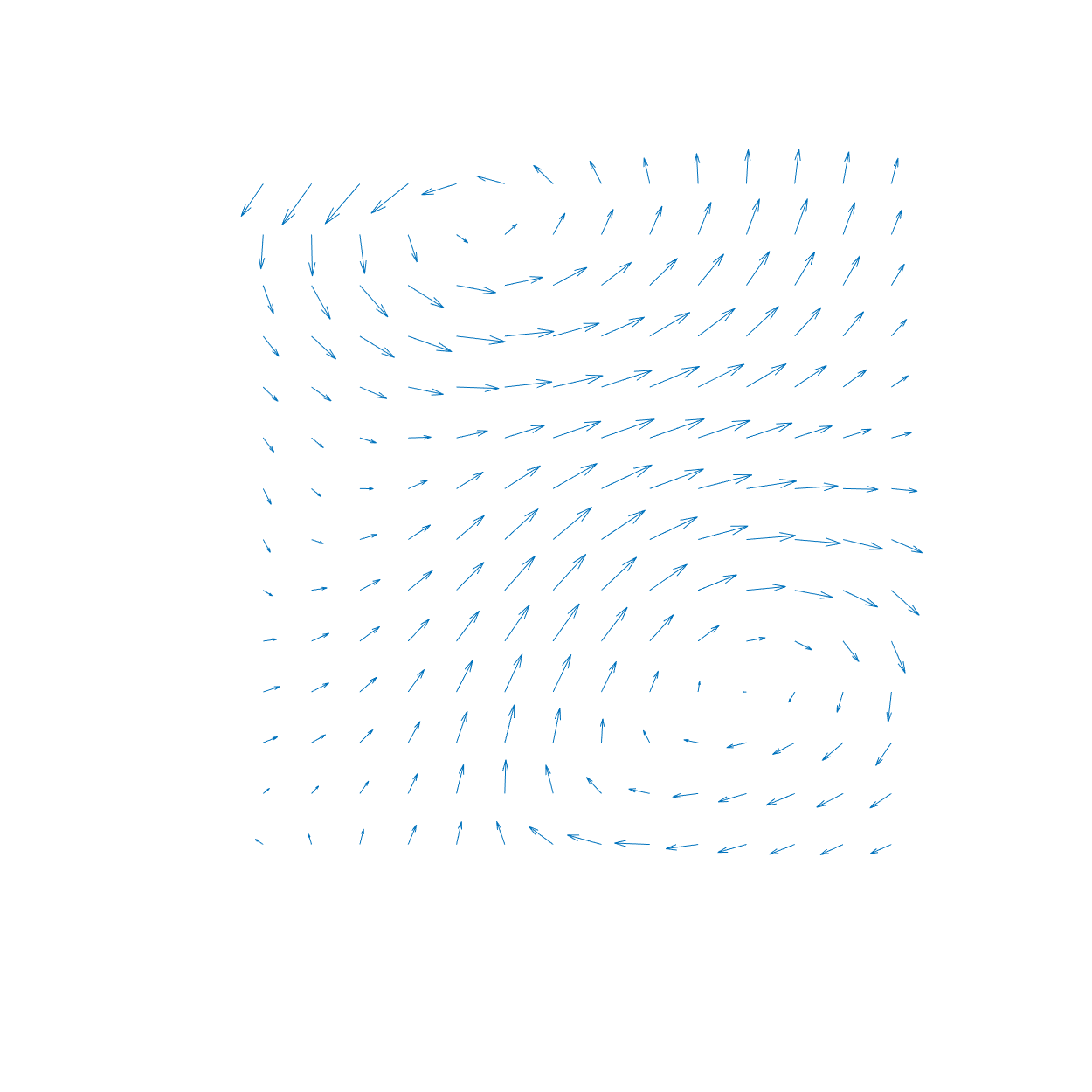

Supplement: S1 MCG raw data 1 — The raw MCG dataset includes categories 0-4 for testing. (ZIP) [file pone.0338189.s001.zip › test/0/p3_500_4.png]

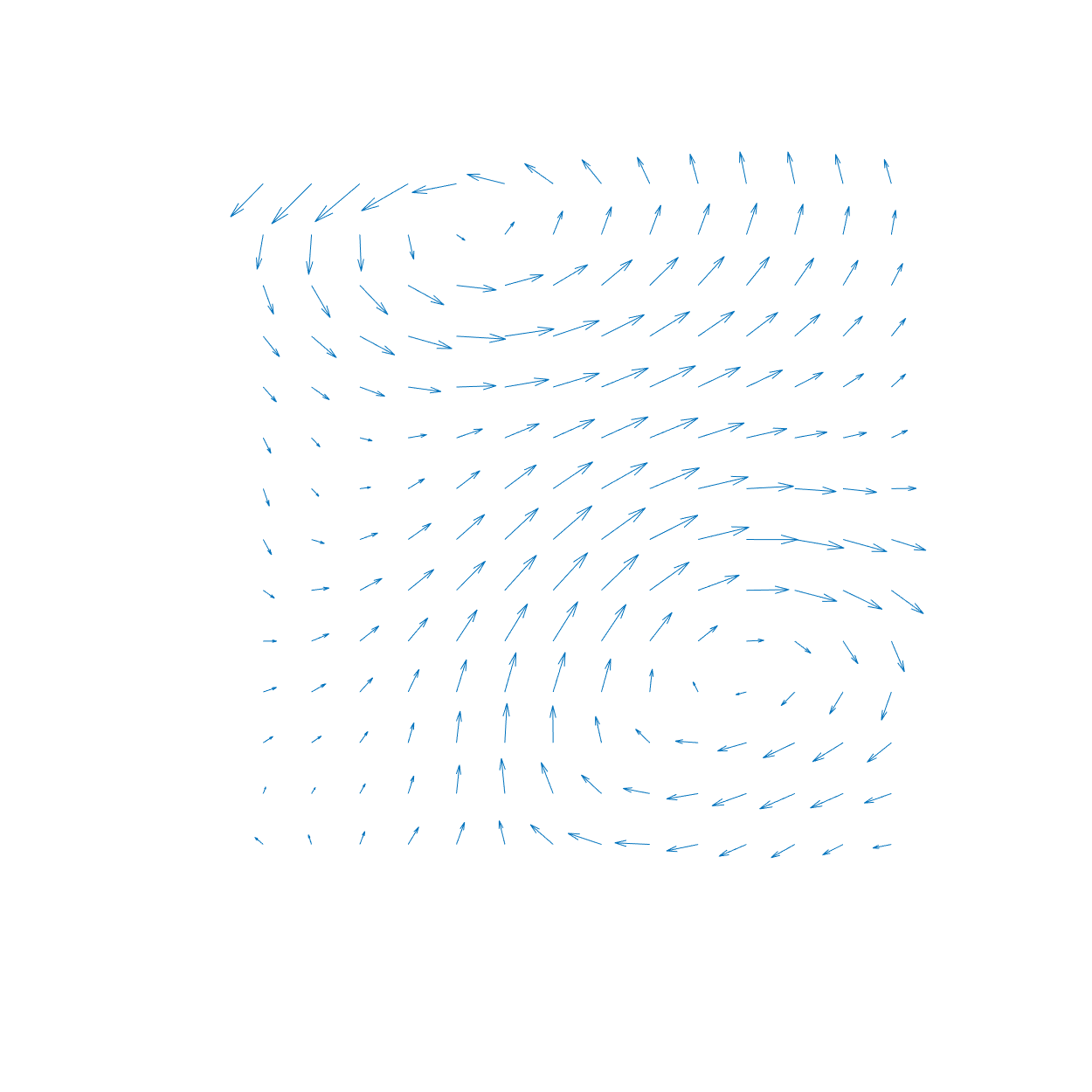

Supplement: S1 MCG raw data 1 — The raw MCG dataset includes categories 0-4 for testing. (ZIP) [file pone.0338189.s001.zip › test/0/p3_505_4.png]

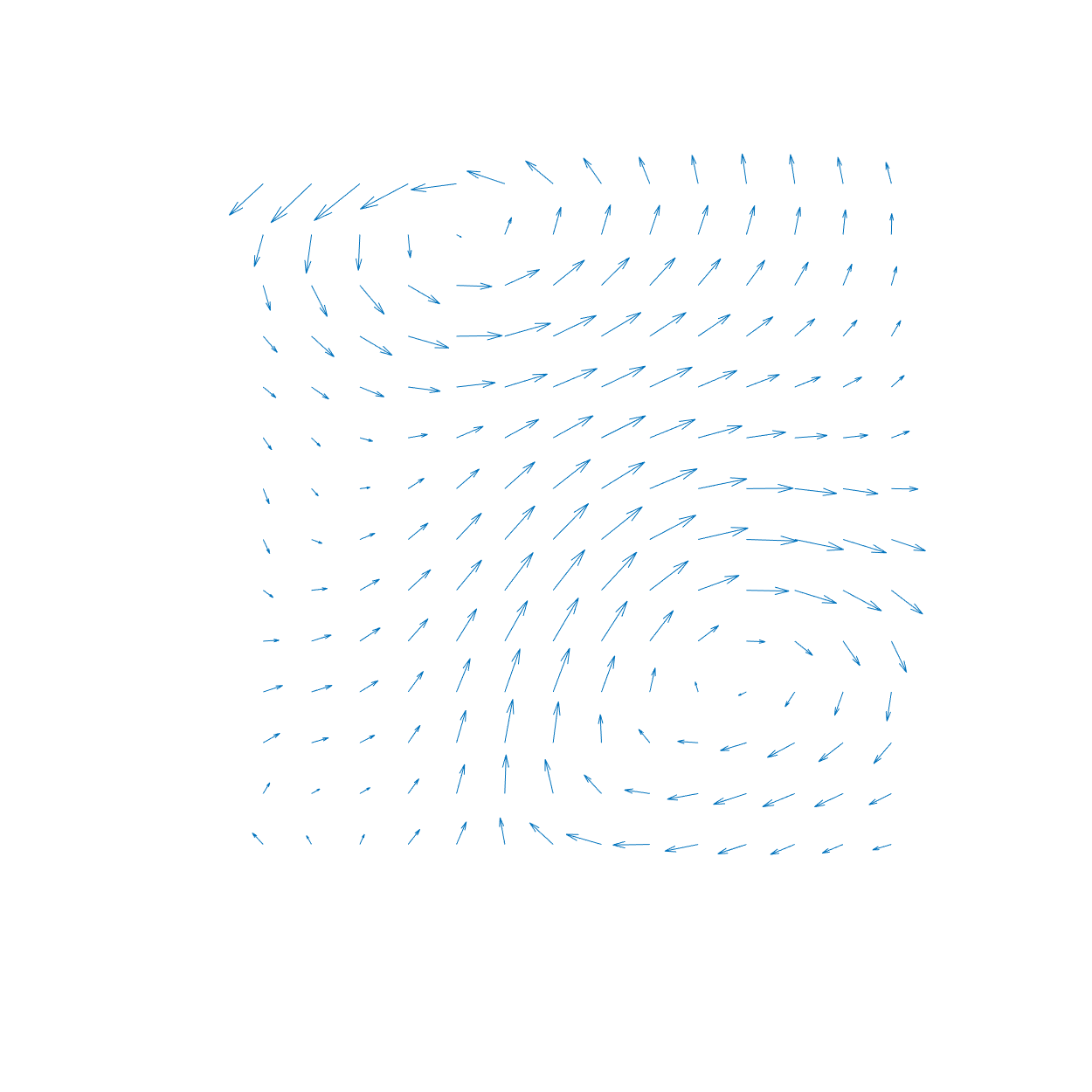

Supplement: S1 MCG raw data 1 — The raw MCG dataset includes categories 0-4 for testing. (ZIP) [file pone.0338189.s001.zip › test/0/p3_510_4.png]

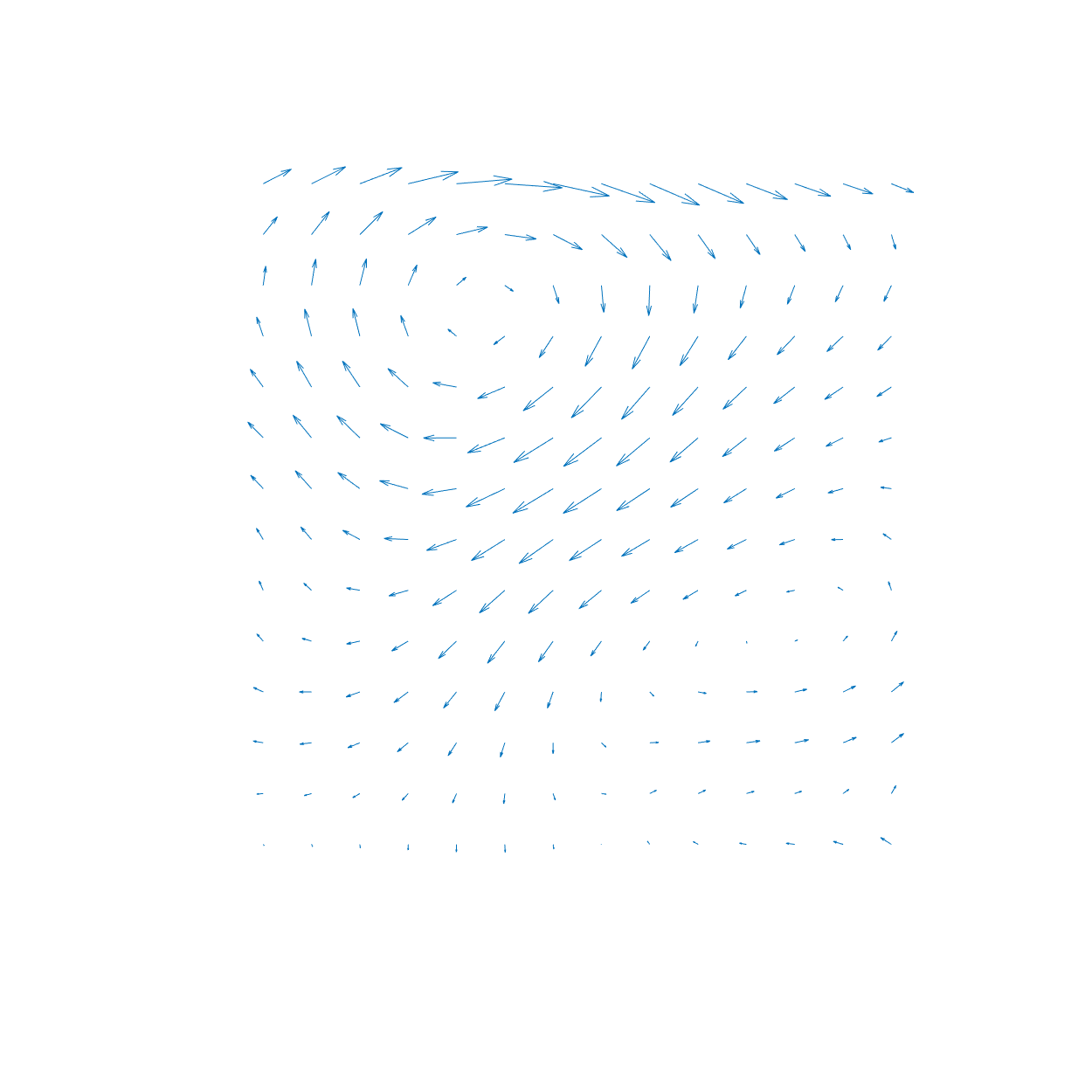

Supplement: S1 MCG raw data 1 — The raw MCG dataset includes categories 0-4 for testing. (ZIP) [file pone.0338189.s001.zip › test/0/p4_200_4.png]

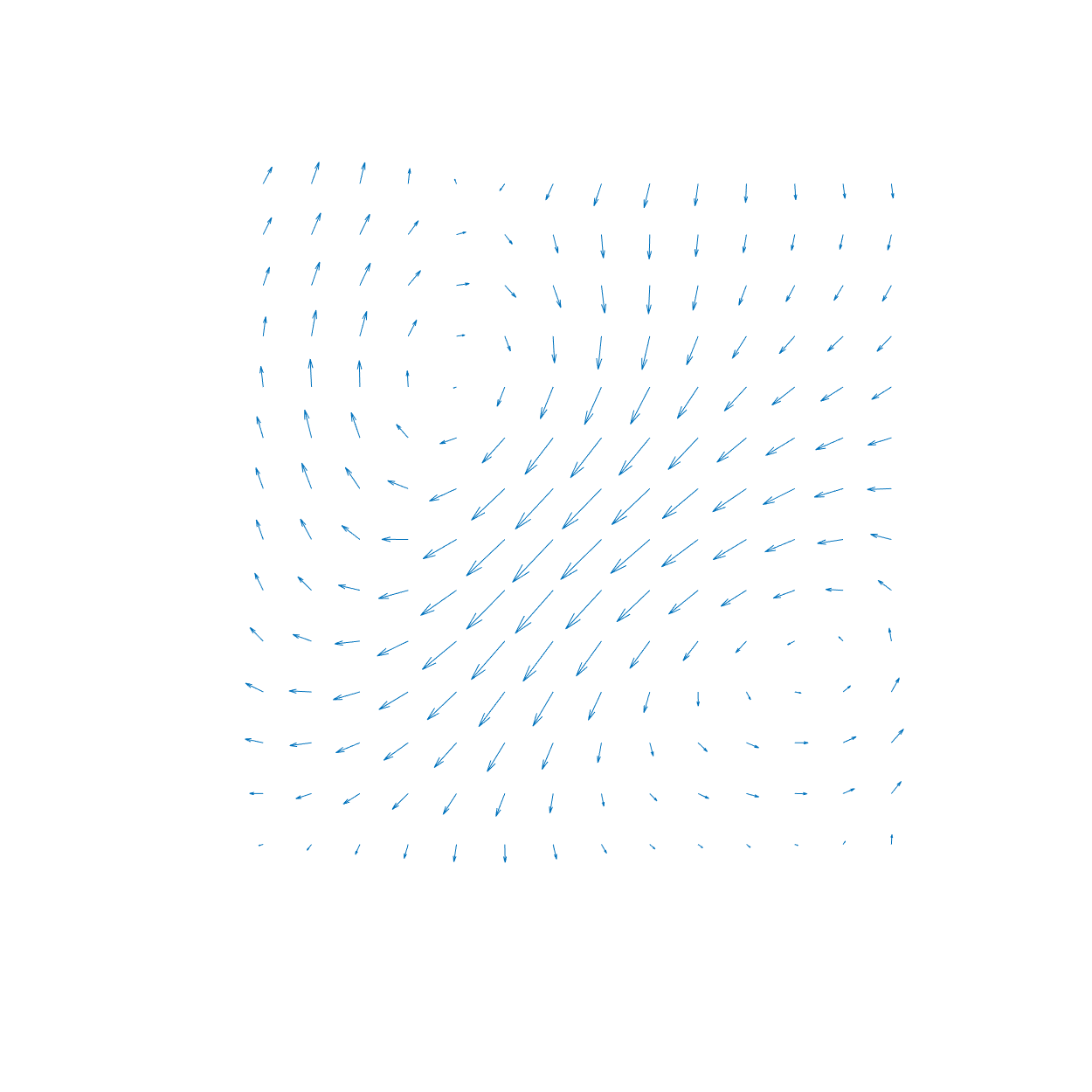

Supplement: S1 MCG raw data 1 — The raw MCG dataset includes categories 0-4 for testing. (ZIP) [file pone.0338189.s001.zip › test/0/p4_205_4.png]

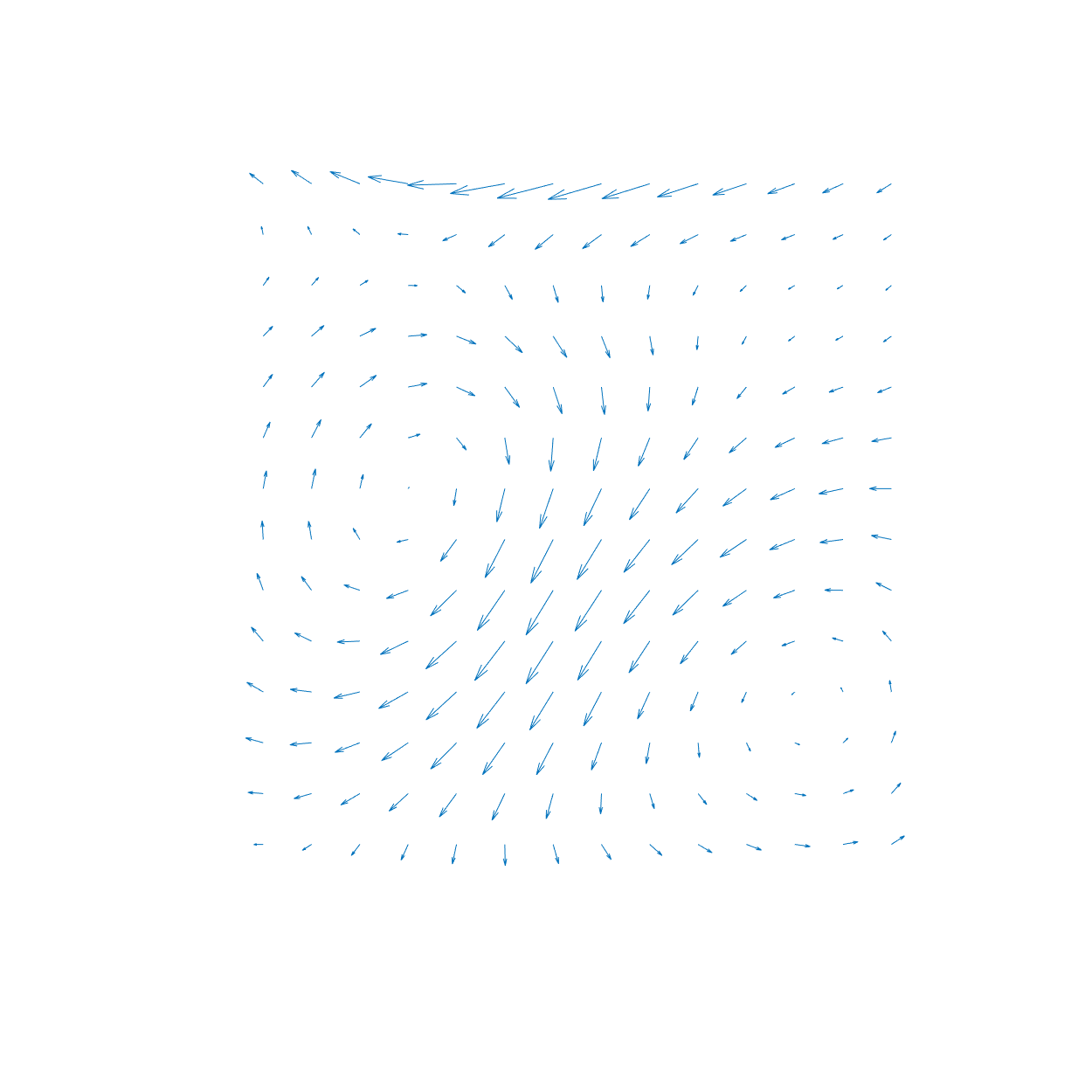

Supplement: S1 MCG raw data 1 — The raw MCG dataset includes categories 0-4 for testing. (ZIP) [file pone.0338189.s001.zip › test/0/p4_210_4.png]

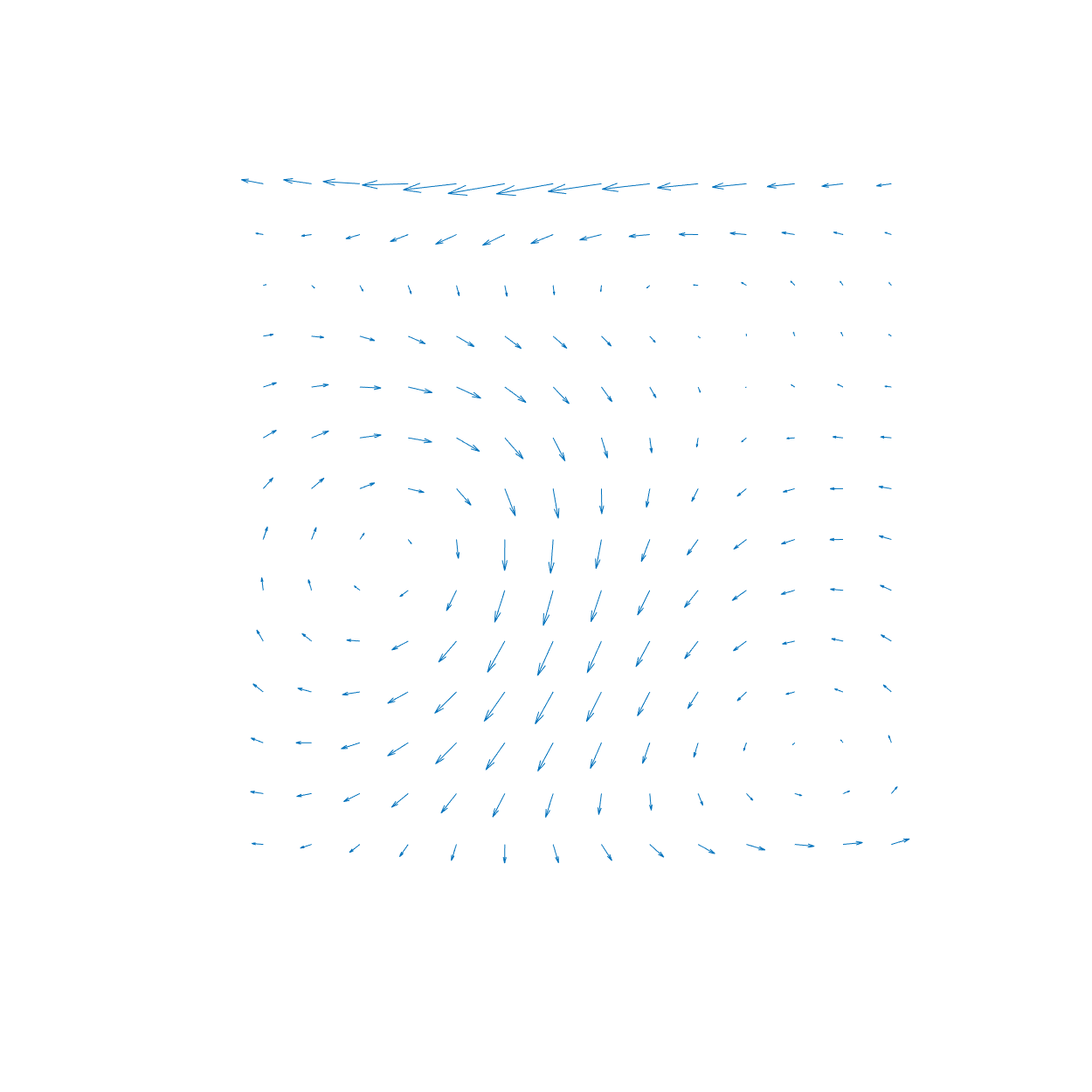

Supplement: S1 MCG raw data 1 — The raw MCG dataset includes categories 0-4 for testing. (ZIP) [file pone.0338189.s001.zip › test/0/p4_215_4.png]

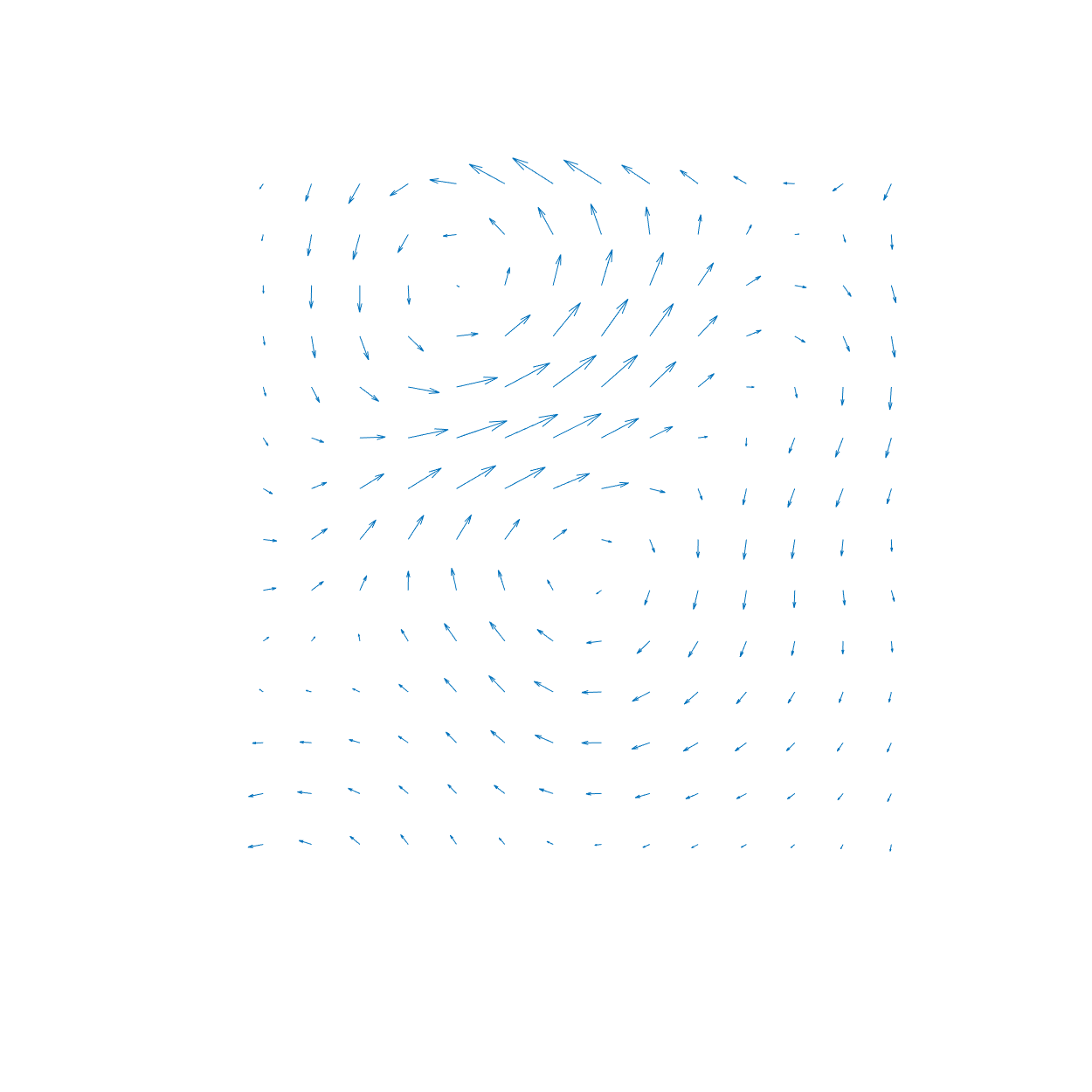

Supplement: S1 MCG raw data 1 — The raw MCG dataset includes categories 0-4 for testing. (ZIP) [file pone.0338189.s001.zip › test/0/p4_330_4.png]

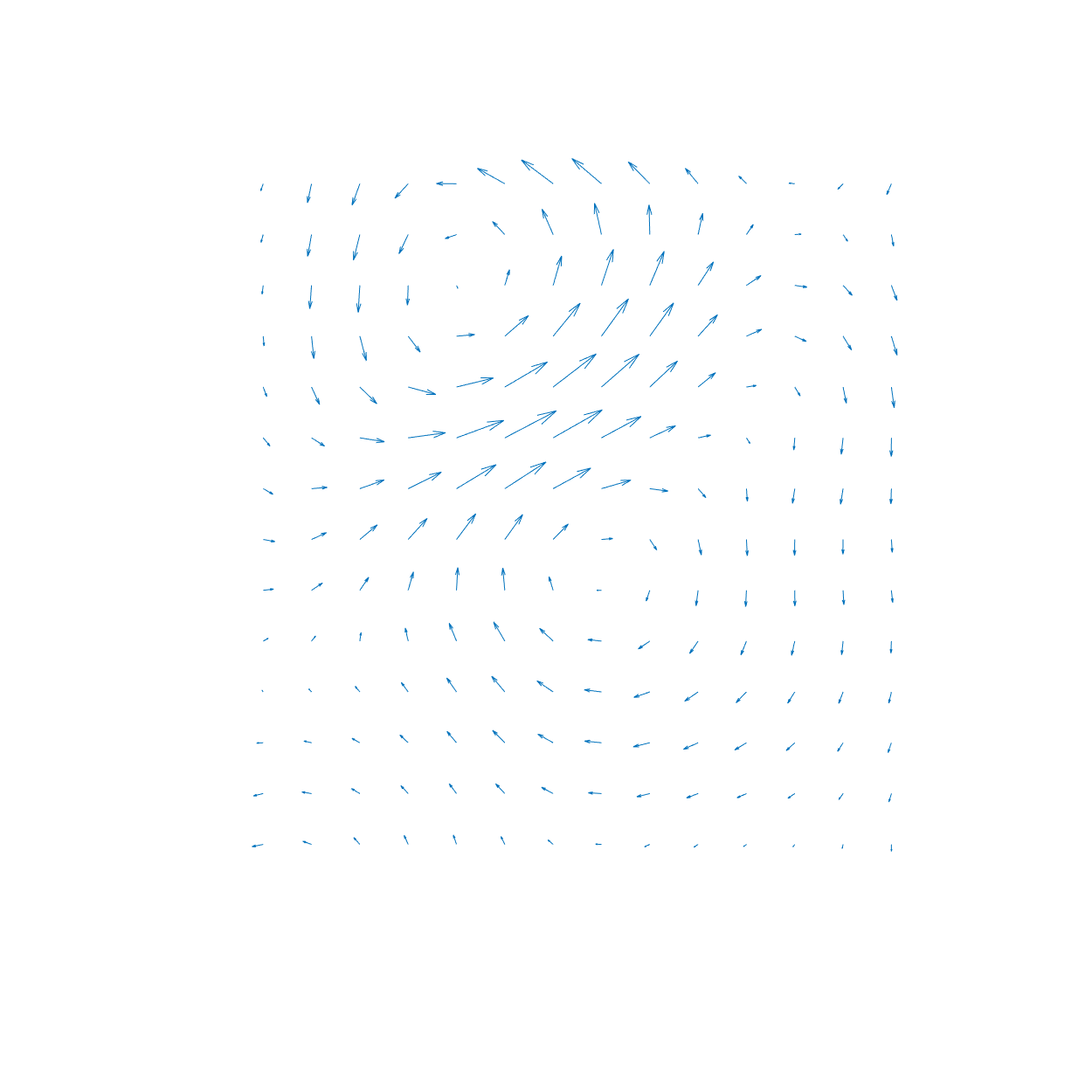

Supplement: S1 MCG raw data 1 — The raw MCG dataset includes categories 0-4 for testing. (ZIP) [file pone.0338189.s001.zip › test/0/p4_335_4.png]

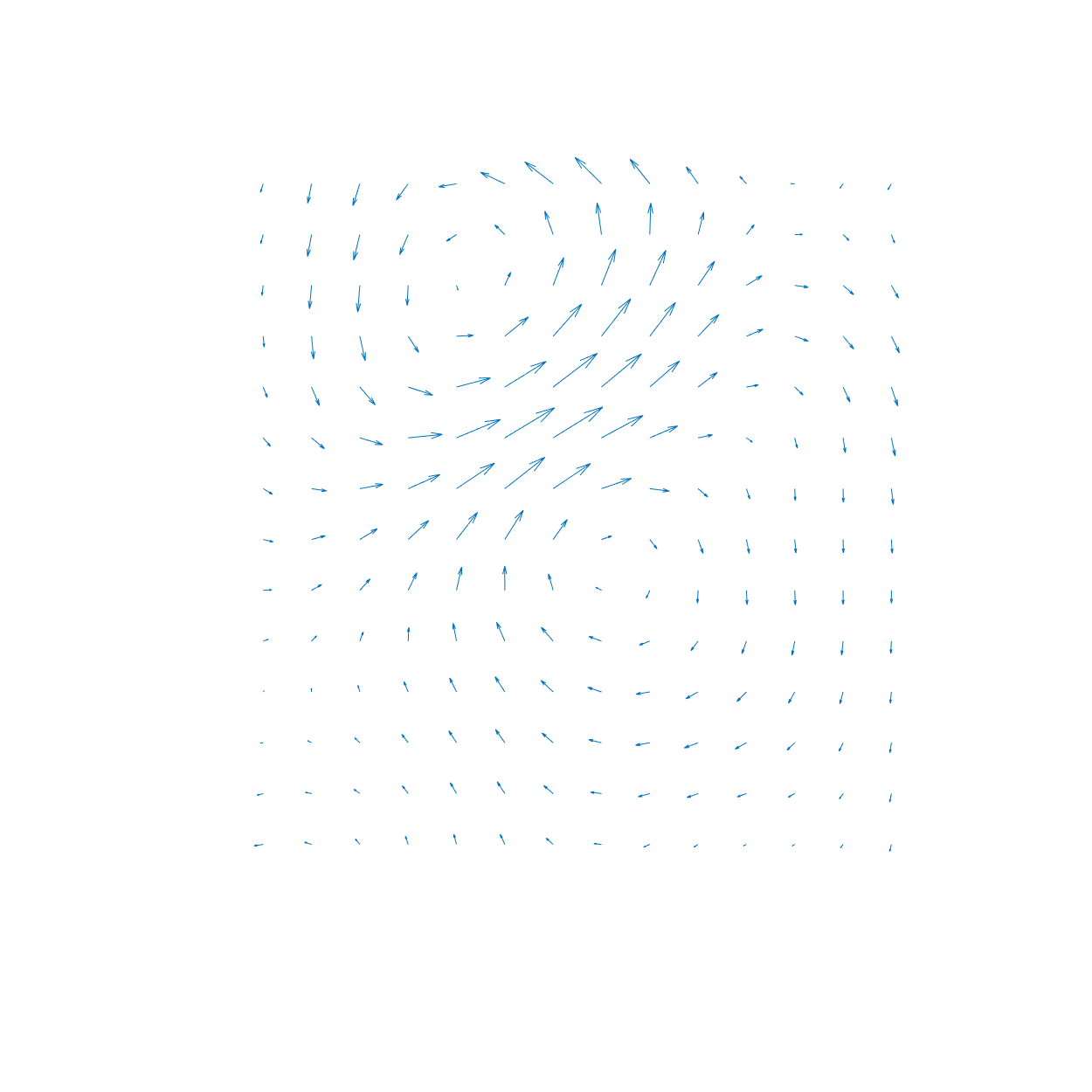

Supplement: S1 MCG raw data 1 — The raw MCG dataset includes categories 0-4 for testing. (ZIP) [file pone.0338189.s001.zip › test/0/p4_340_4.png]

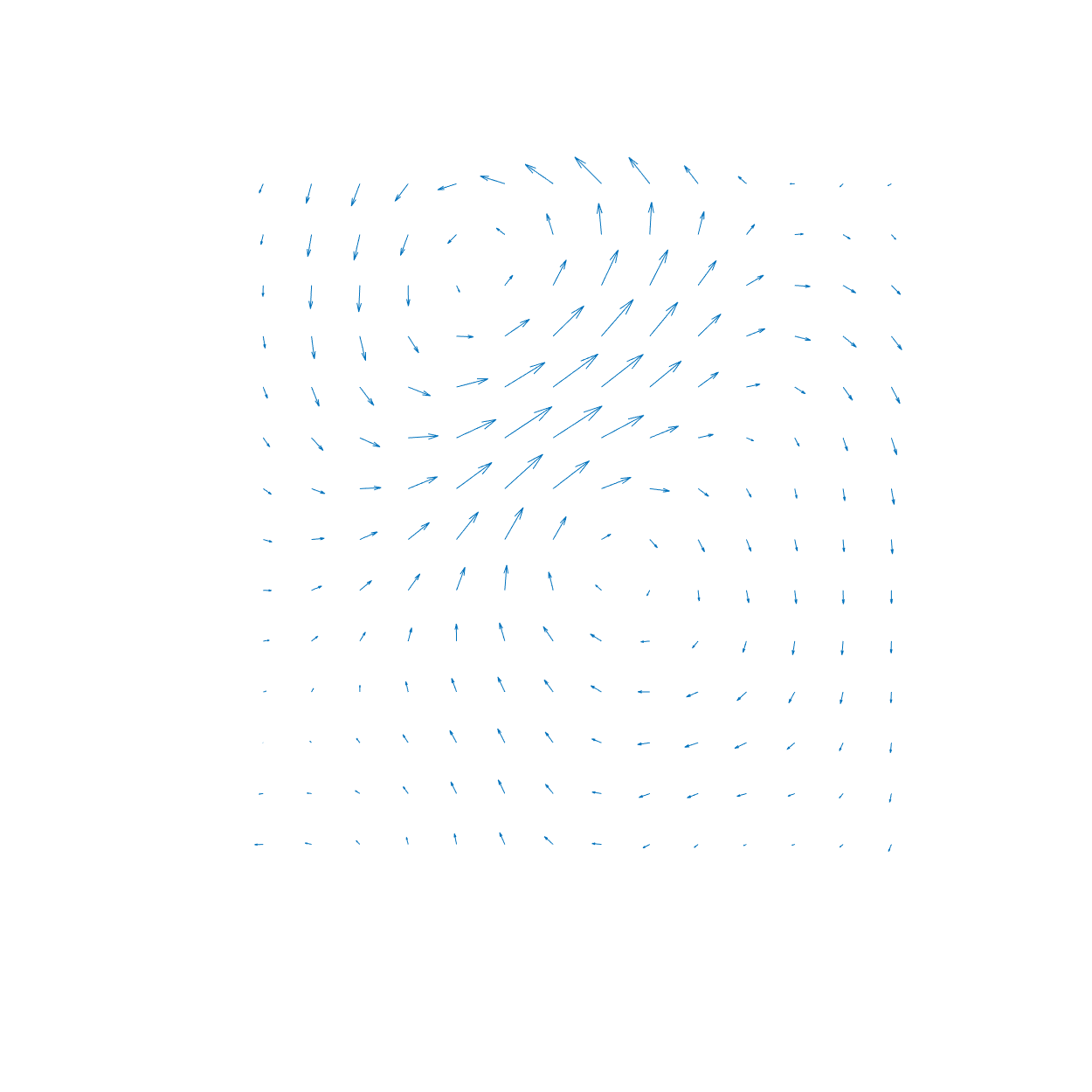

Supplement: S1 MCG raw data 1 — The raw MCG dataset includes categories 0-4 for testing. (ZIP) [file pone.0338189.s001.zip › test/0/p4_345_4.png]

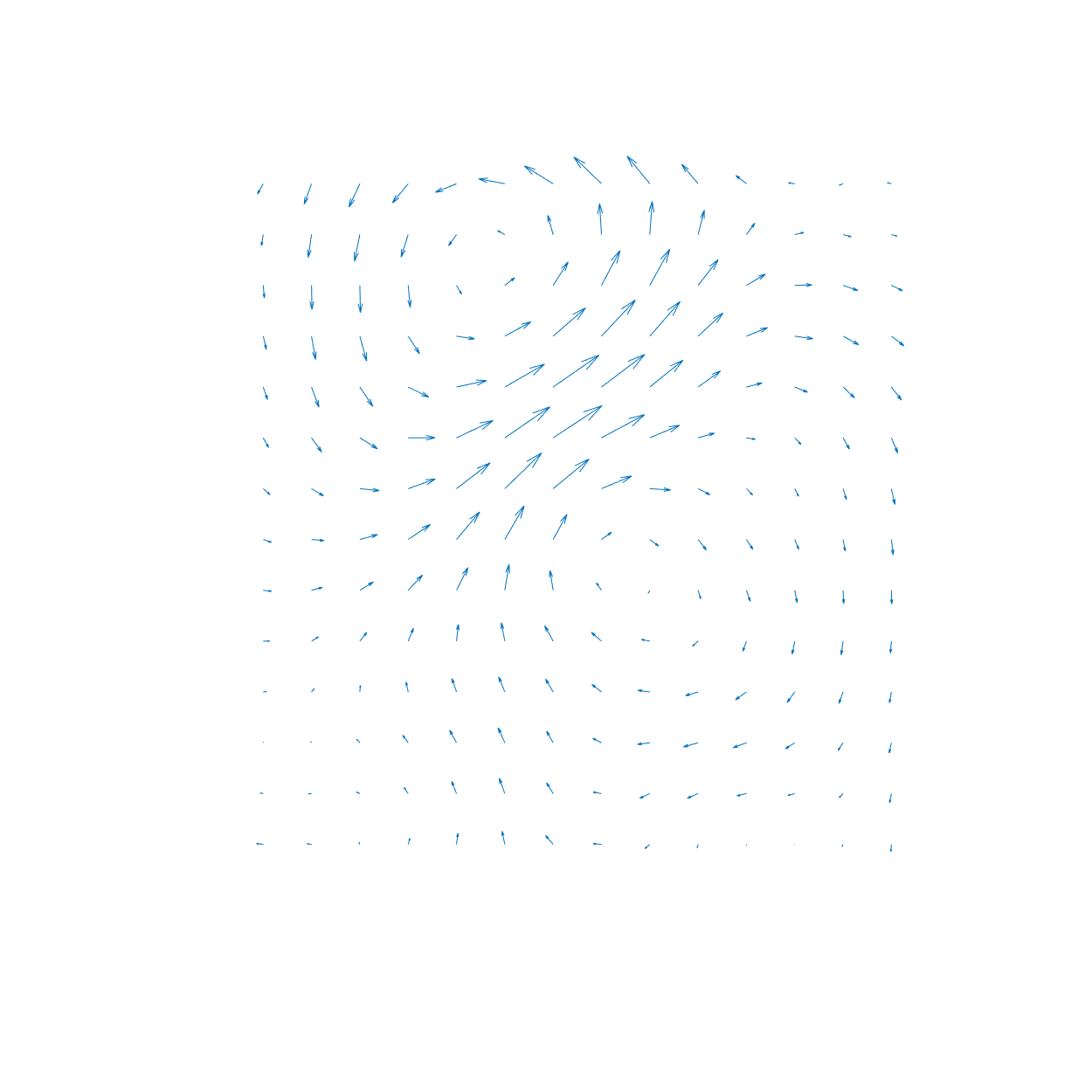

Supplement: S1 MCG raw data 1 — The raw MCG dataset includes categories 0-4 for testing. (ZIP) [file pone.0338189.s001.zip › test/0/p4_350_4.png]

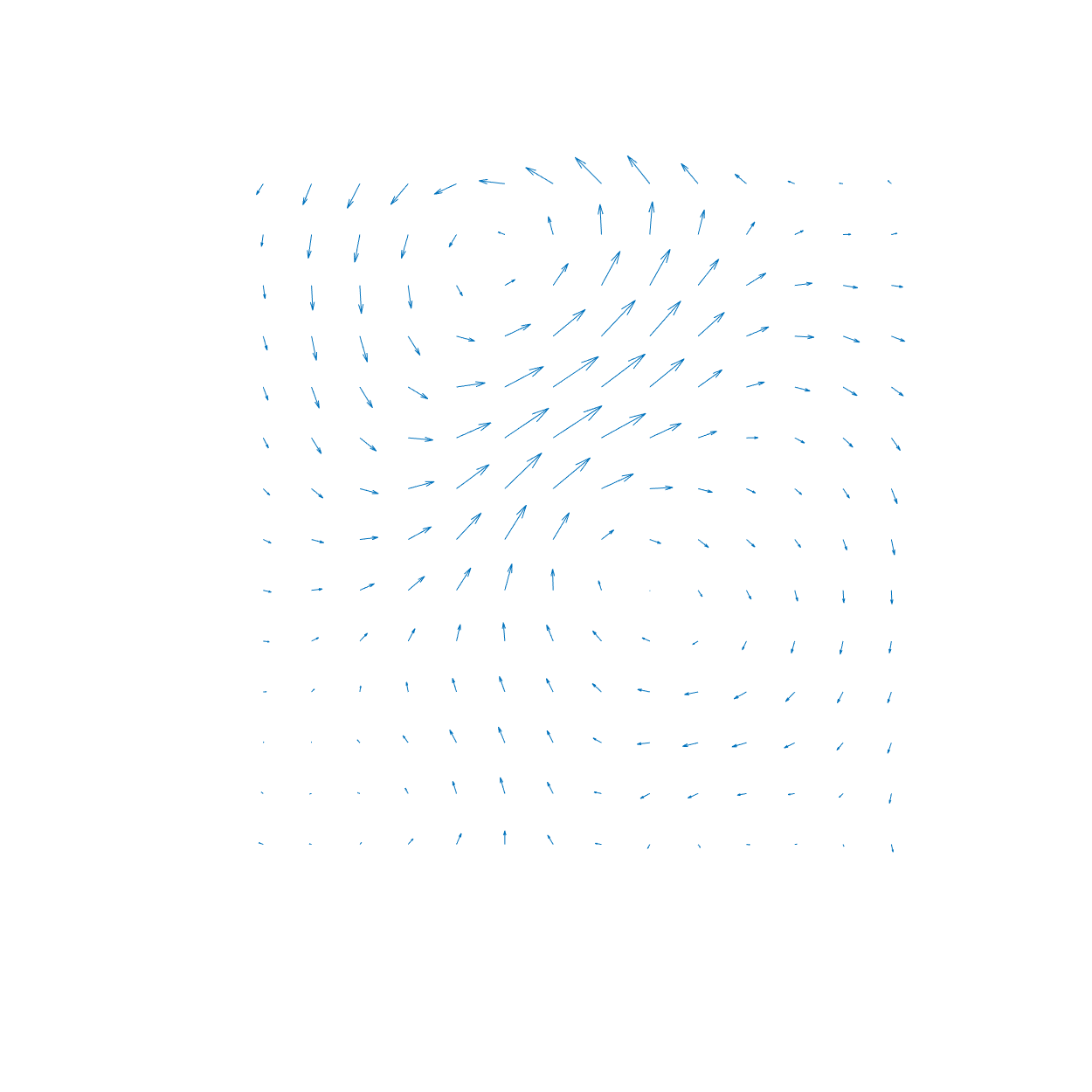

Supplement: S1 MCG raw data 1 — The raw MCG dataset includes categories 0-4 for testing. (ZIP) [file pone.0338189.s001.zip › test/0/p4_355_4.png]

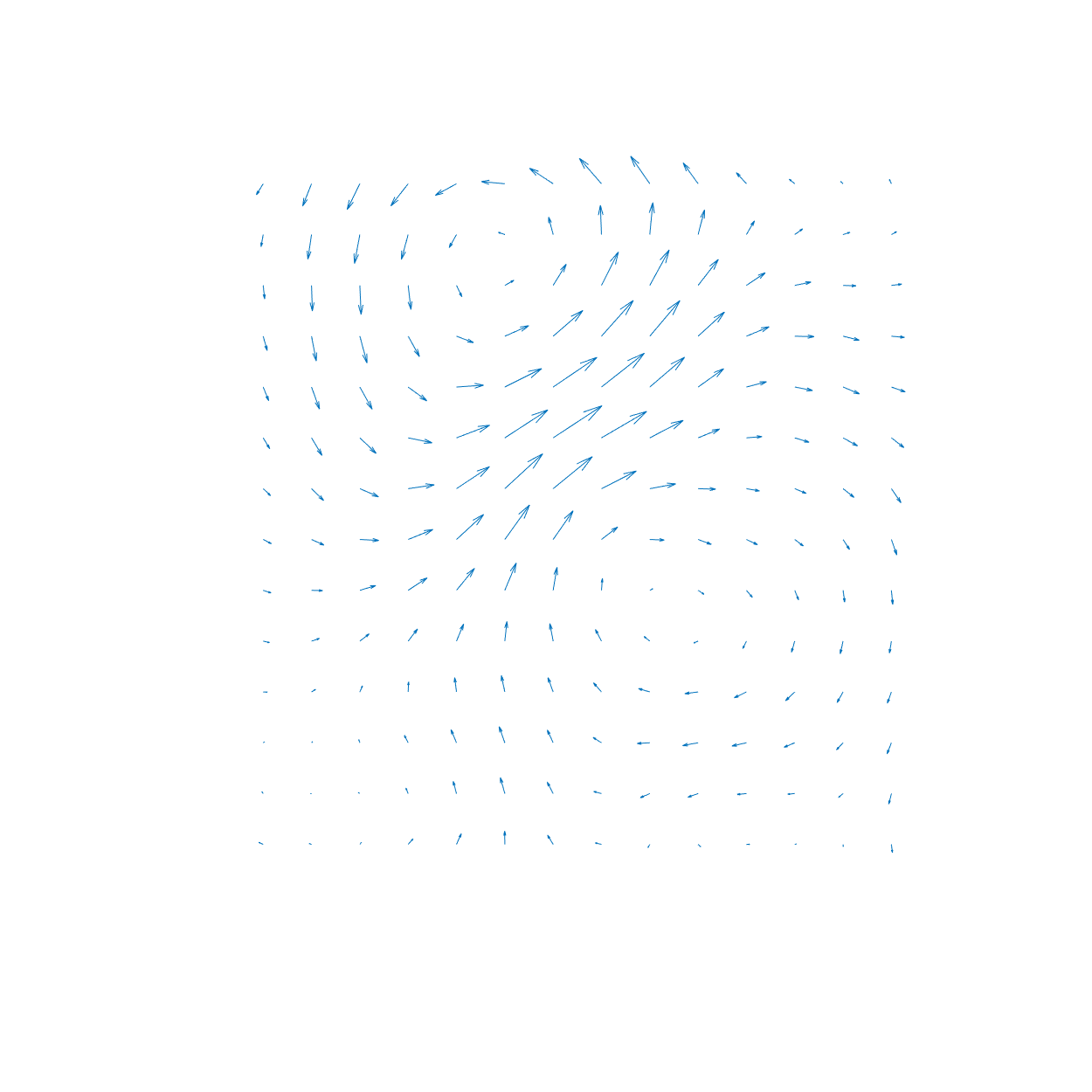

Supplement: S1 MCG raw data 1 — The raw MCG dataset includes categories 0-4 for testing. (ZIP) [file pone.0338189.s001.zip › test/0/p4_360_4.png]

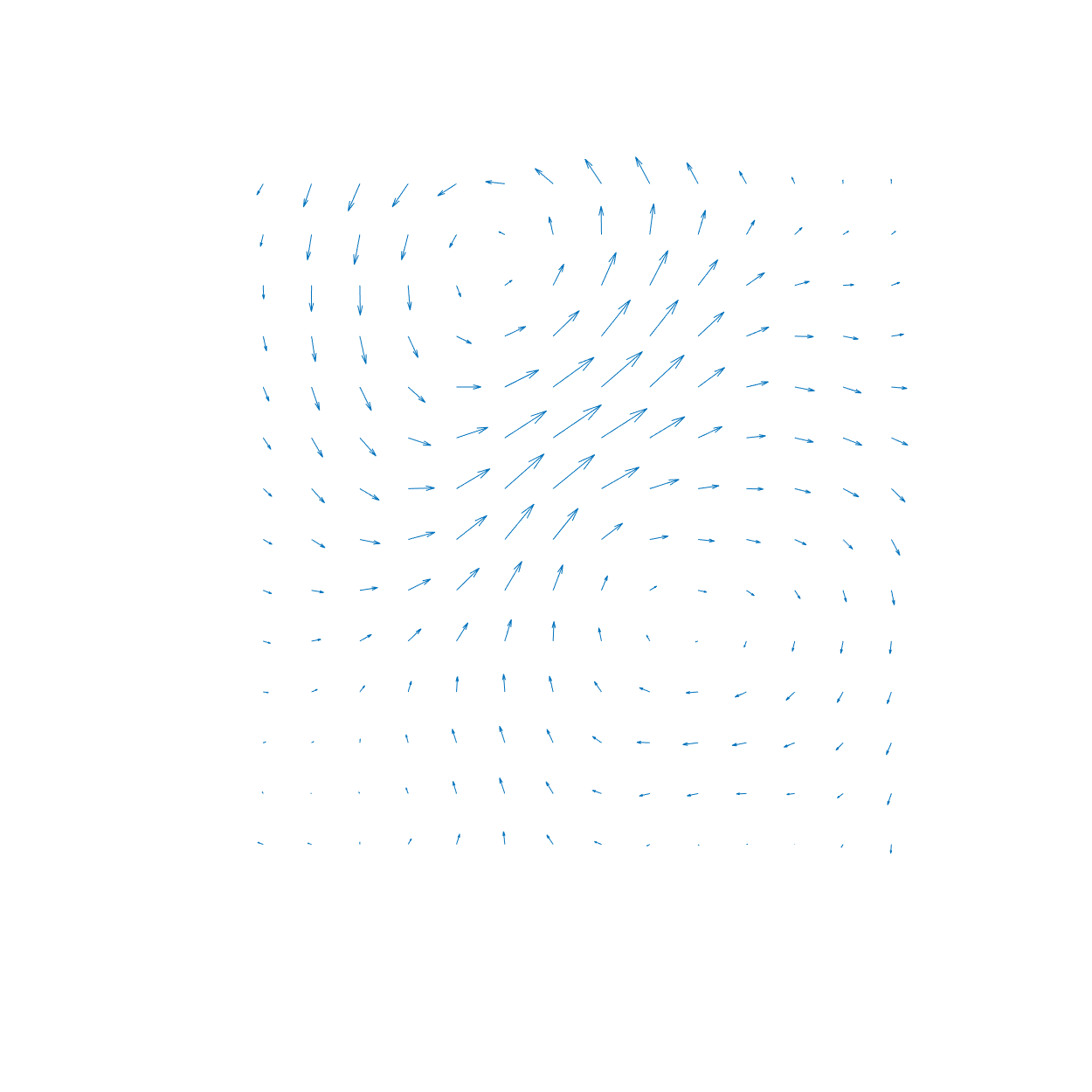

Supplement: S1 MCG raw data 1 — The raw MCG dataset includes categories 0-4 for testing. (ZIP) [file pone.0338189.s001.zip › test/0/p4_365_4.png]

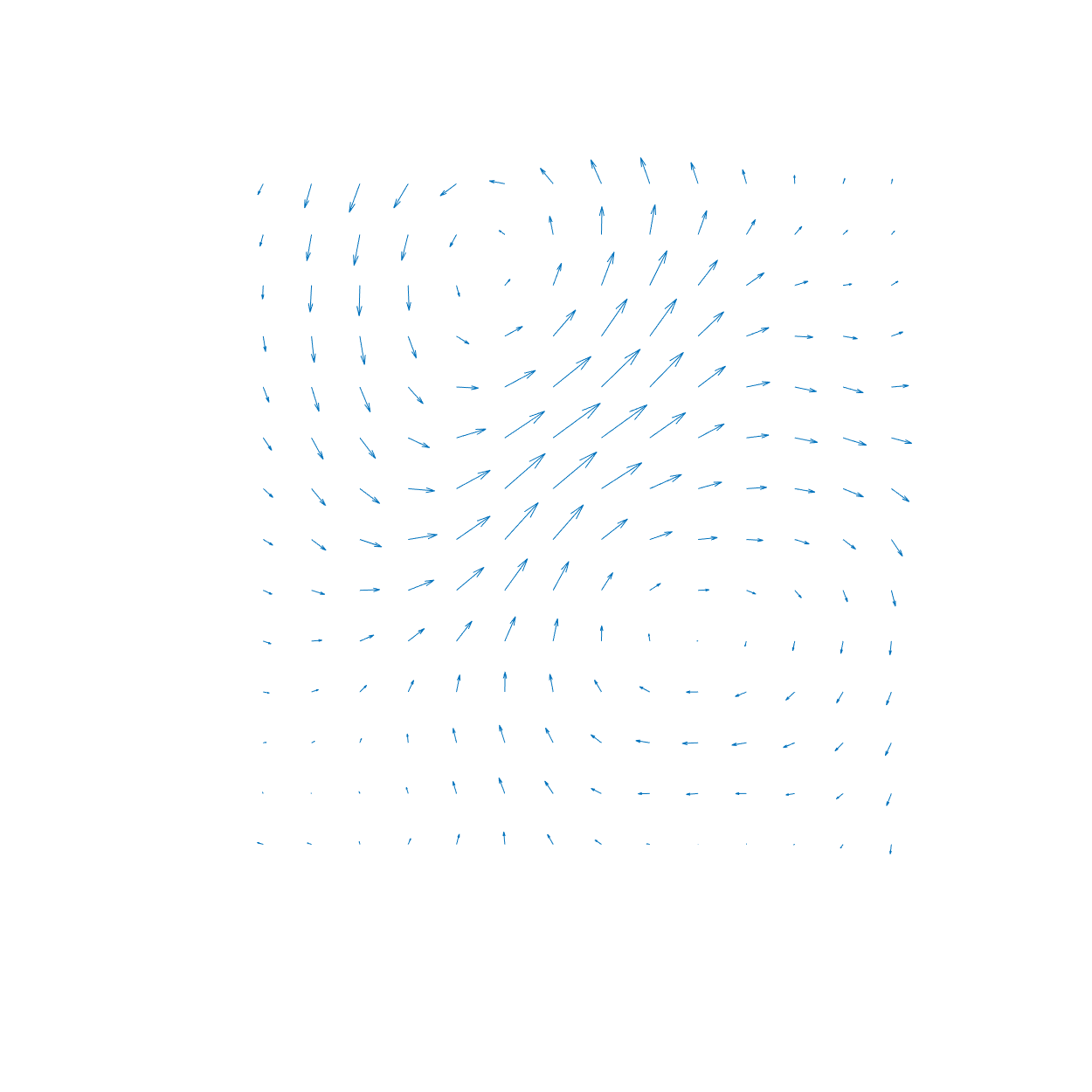

Supplement: S1 MCG raw data 1 — The raw MCG dataset includes categories 0-4 for testing. (ZIP) [file pone.0338189.s001.zip › test/0/p4_370_4.png]

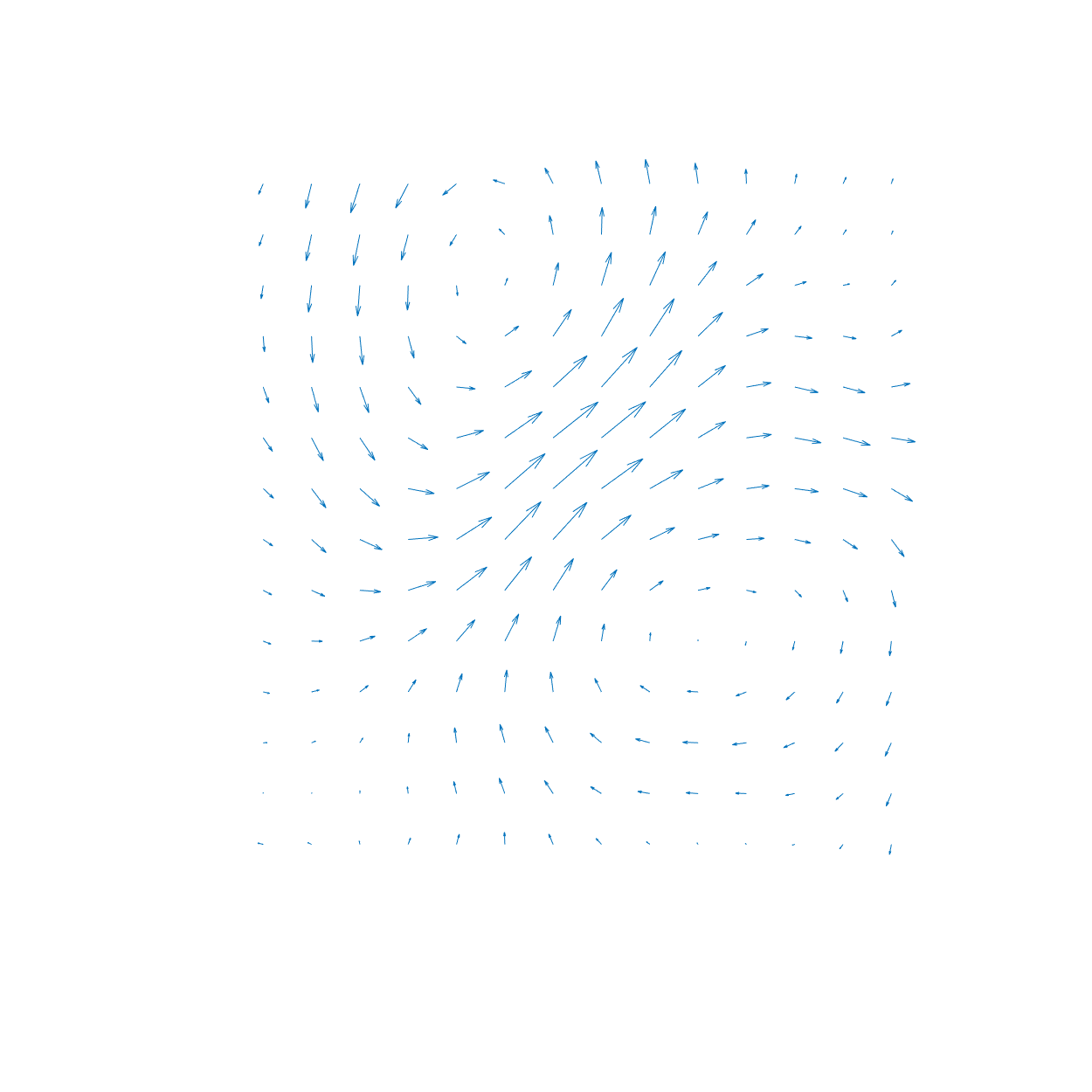

Supplement: S1 MCG raw data 1 — The raw MCG dataset includes categories 0-4 for testing. (ZIP) [file pone.0338189.s001.zip › test/0/p4_375_4.png]

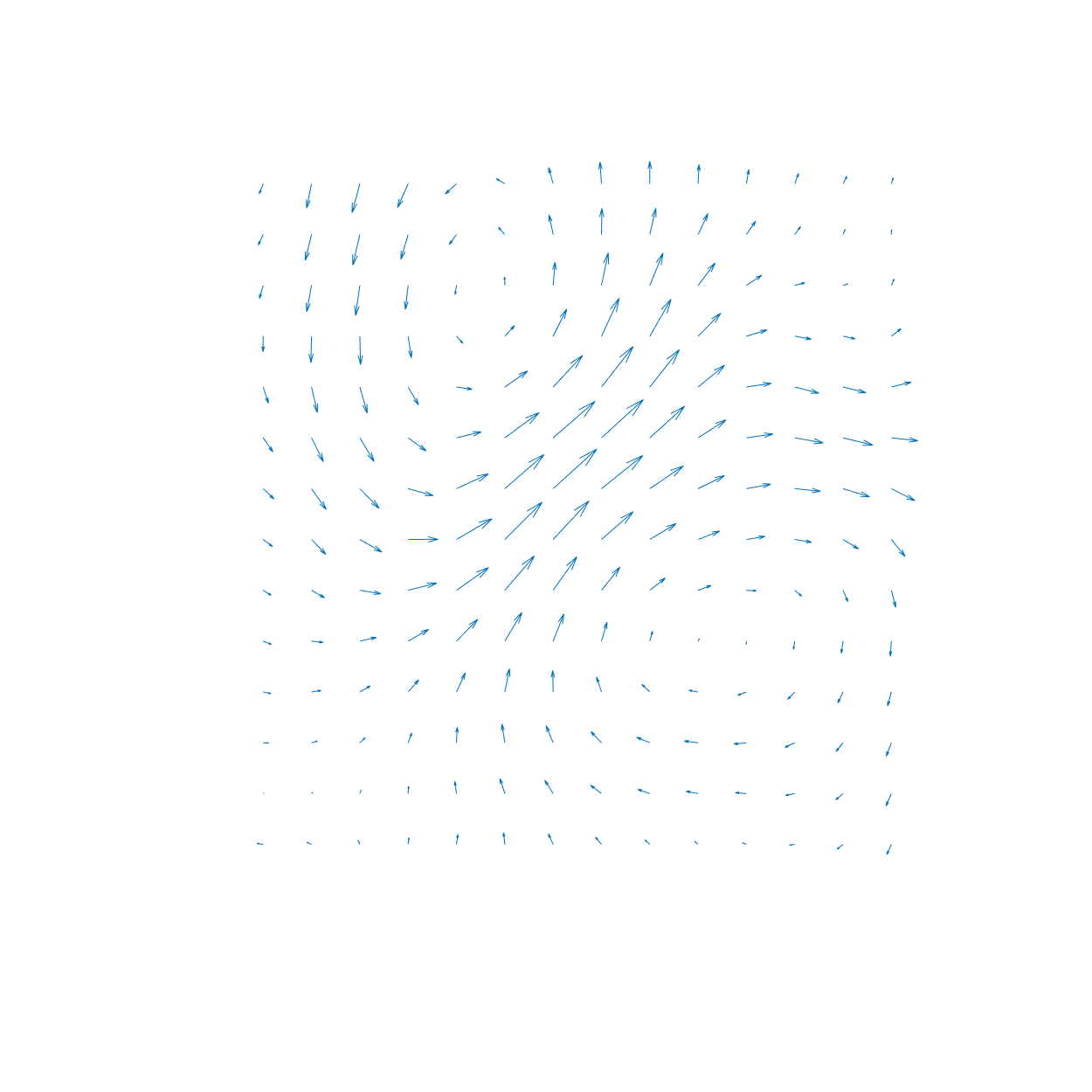

Supplement: S1 MCG raw data 1 — The raw MCG dataset includes categories 0-4 for testing. (ZIP) [file pone.0338189.s001.zip › test/0/p4_380_4.png]

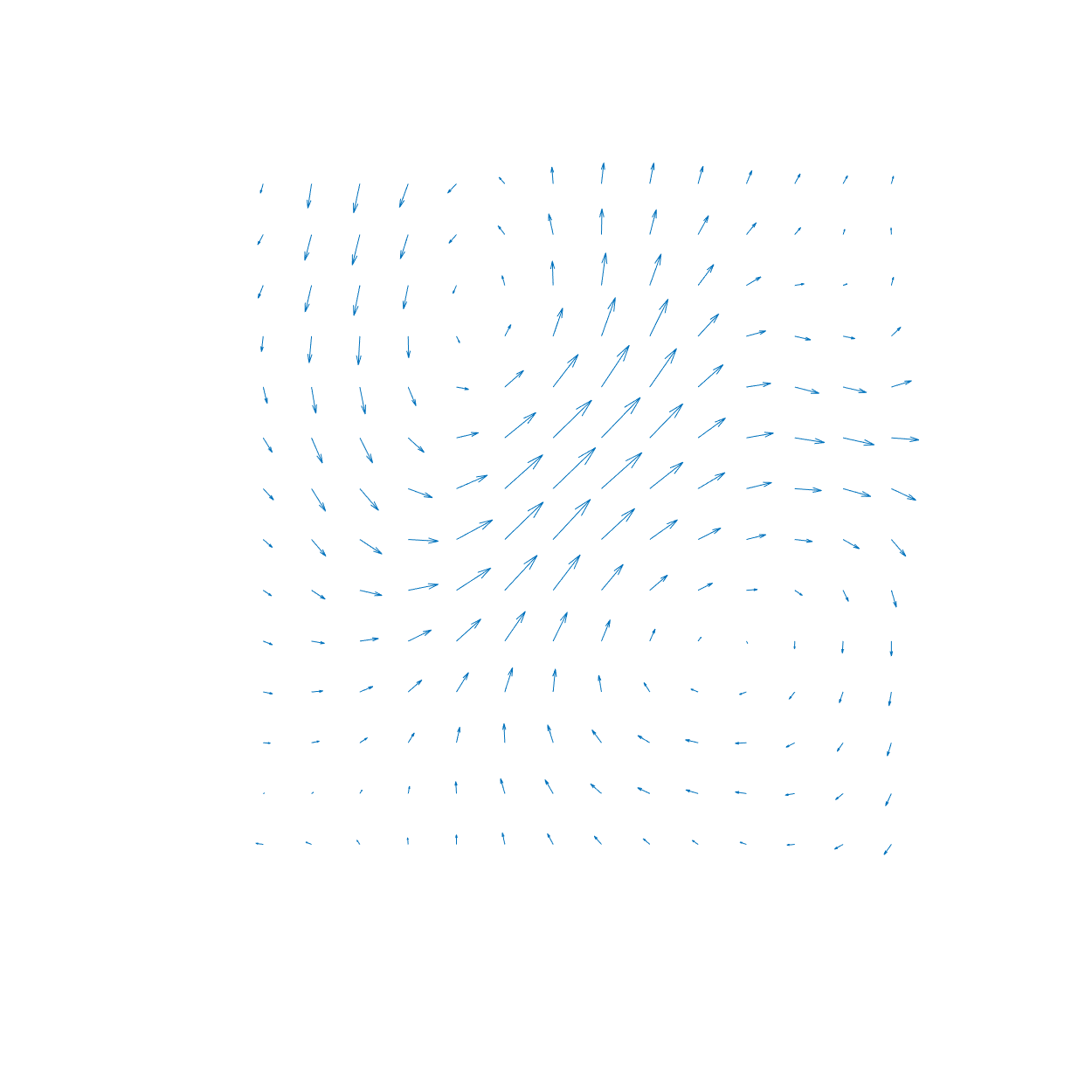

Supplement: S1 MCG raw data 1 — The raw MCG dataset includes categories 0-4 for testing. (ZIP) [file pone.0338189.s001.zip › test/0/p4_385_4.png]

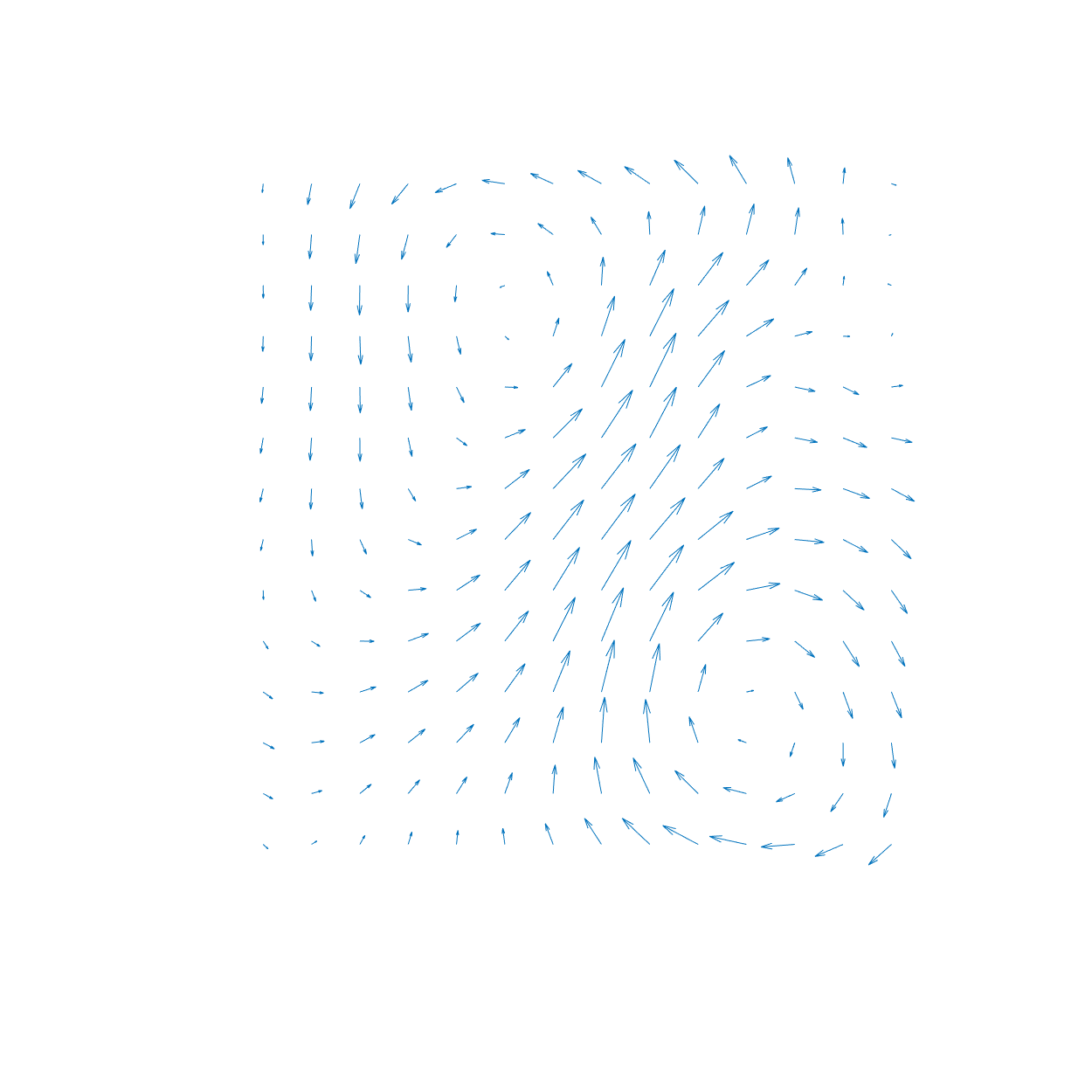

Supplement: S1 MCG raw data 1 — The raw MCG dataset includes categories 0-4 for testing. (ZIP) [file pone.0338189.s001.zip › test/0/p5_385_4.png]

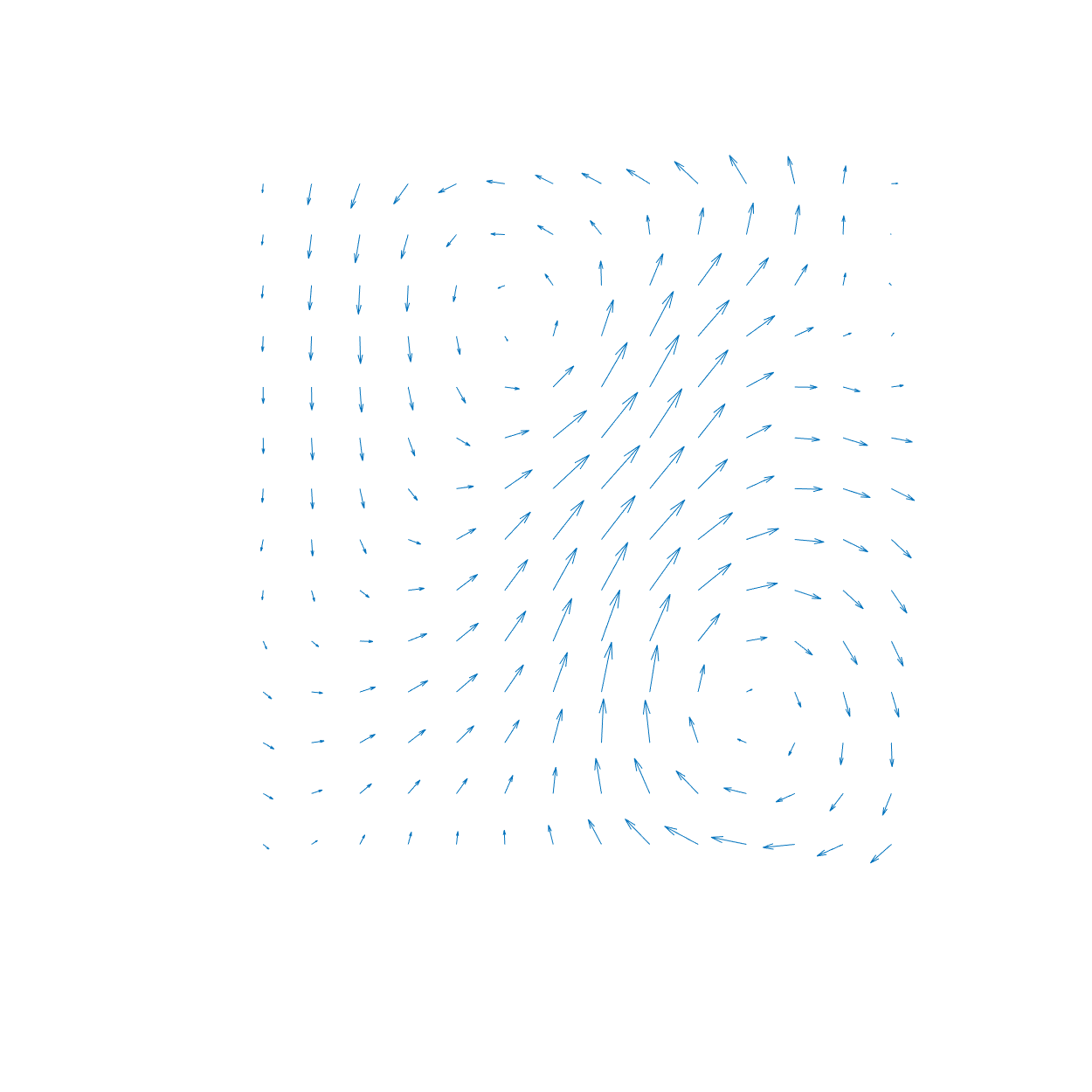

Supplement: S1 MCG raw data 1 — The raw MCG dataset includes categories 0-4 for testing. (ZIP) [file pone.0338189.s001.zip › test/0/p5_390_4.png]

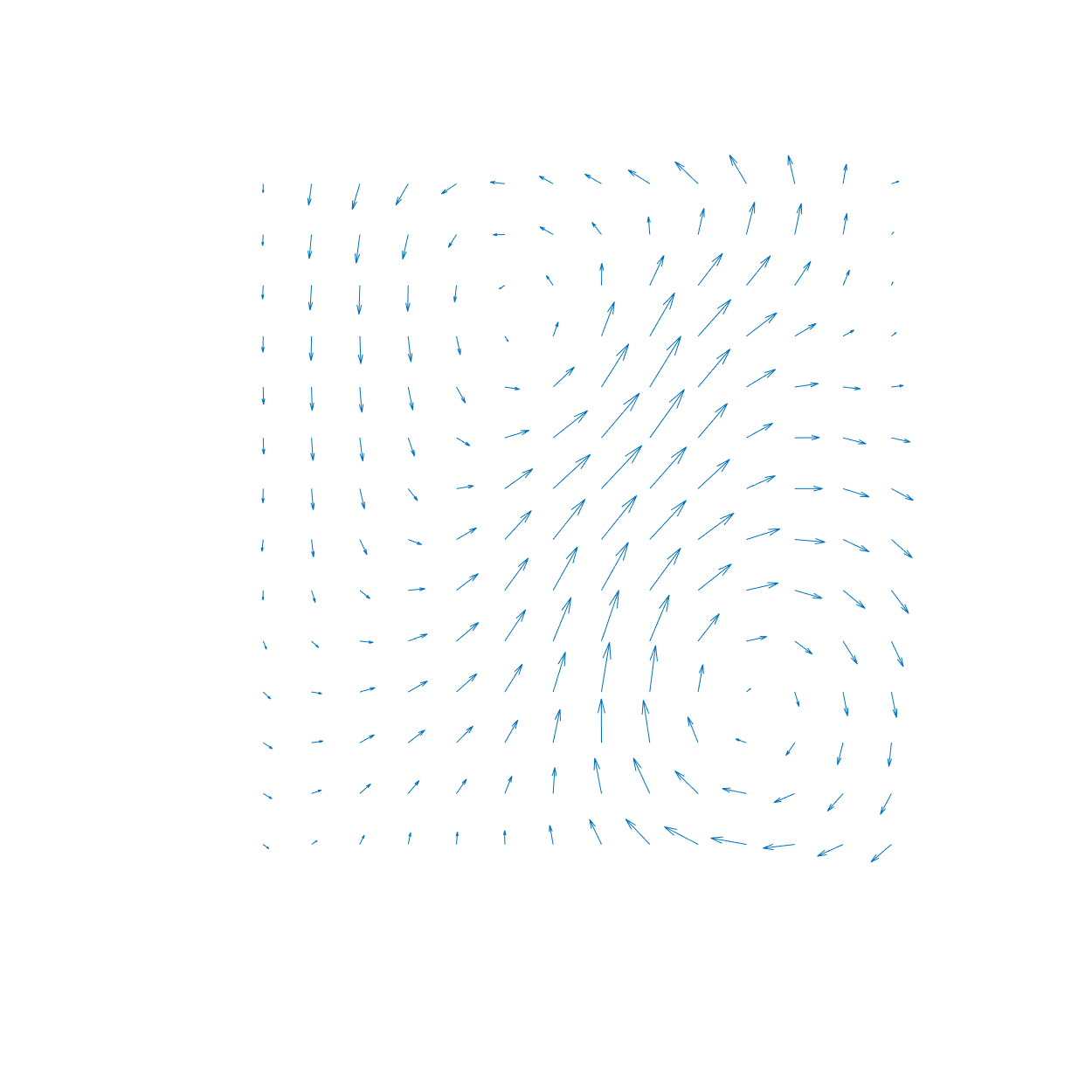

Supplement: S1 MCG raw data 1 — The raw MCG dataset includes categories 0-4 for testing. (ZIP) [file pone.0338189.s001.zip › test/0/p5_395_4.png]

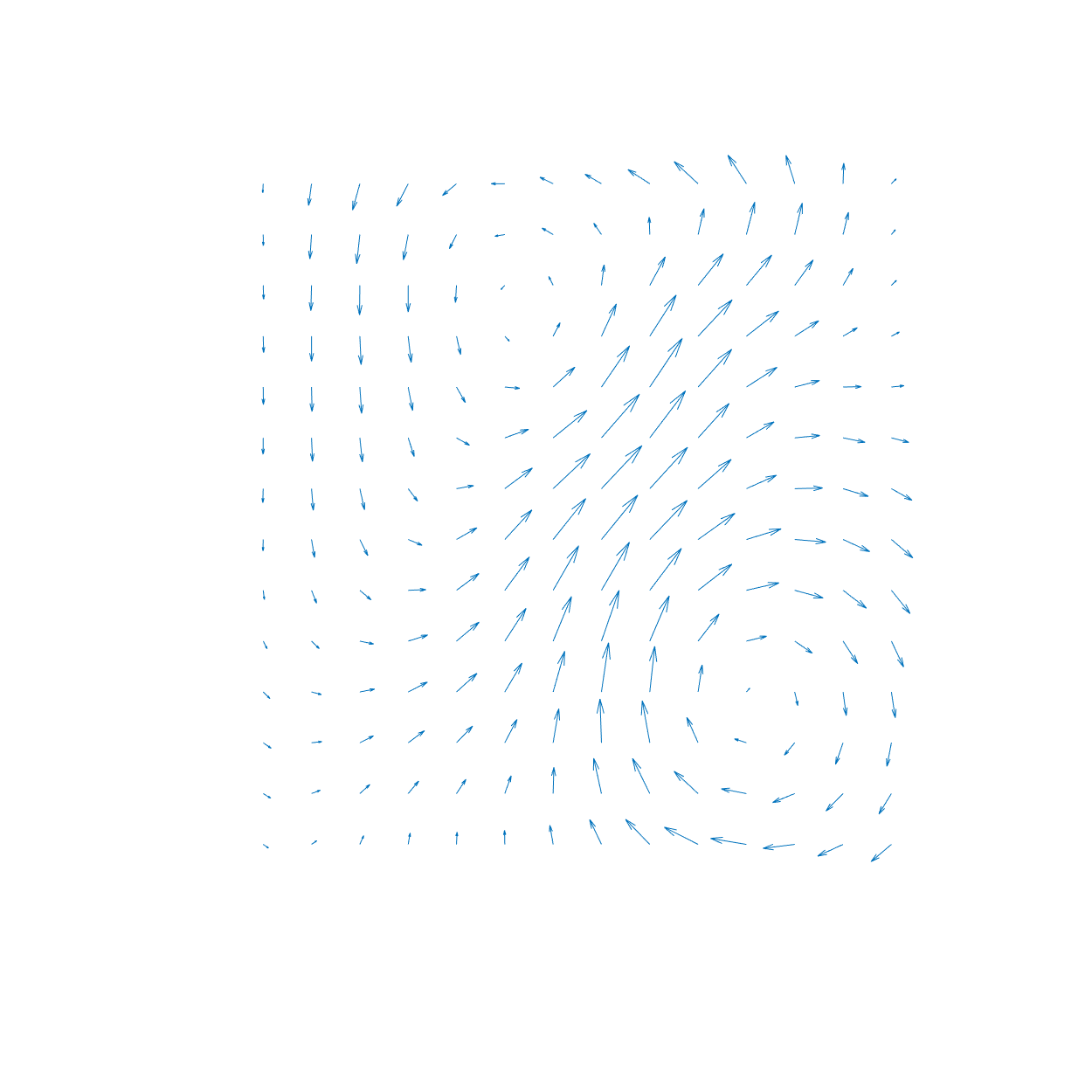

Supplement: S1 MCG raw data 1 — The raw MCG dataset includes categories 0-4 for testing. (ZIP) [file pone.0338189.s001.zip › test/0/p5_400_4.png]

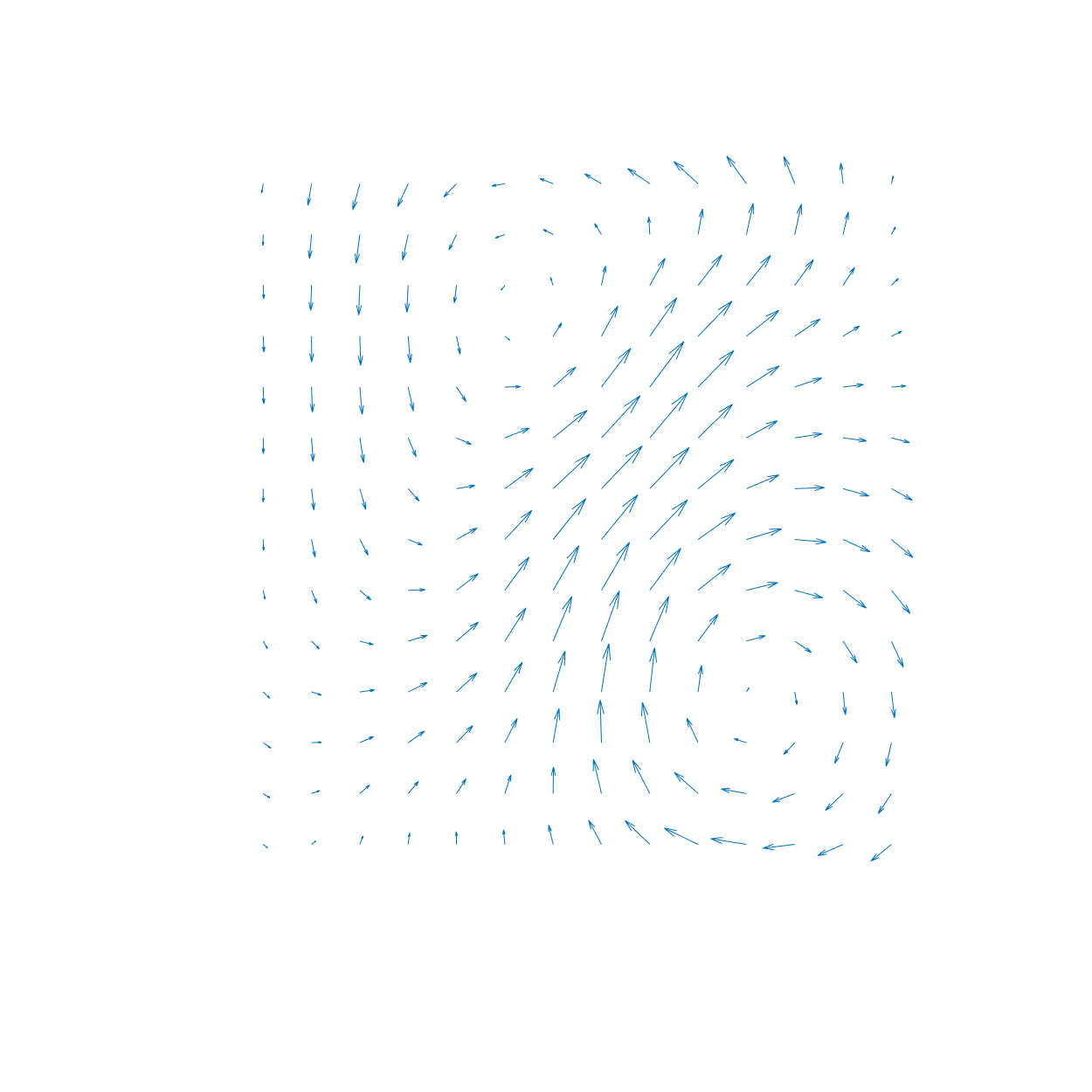

Supplement: S1 MCG raw data 1 — The raw MCG dataset includes categories 0-4 for testing. (ZIP) [file pone.0338189.s001.zip › test/0/p5_405_4.png]

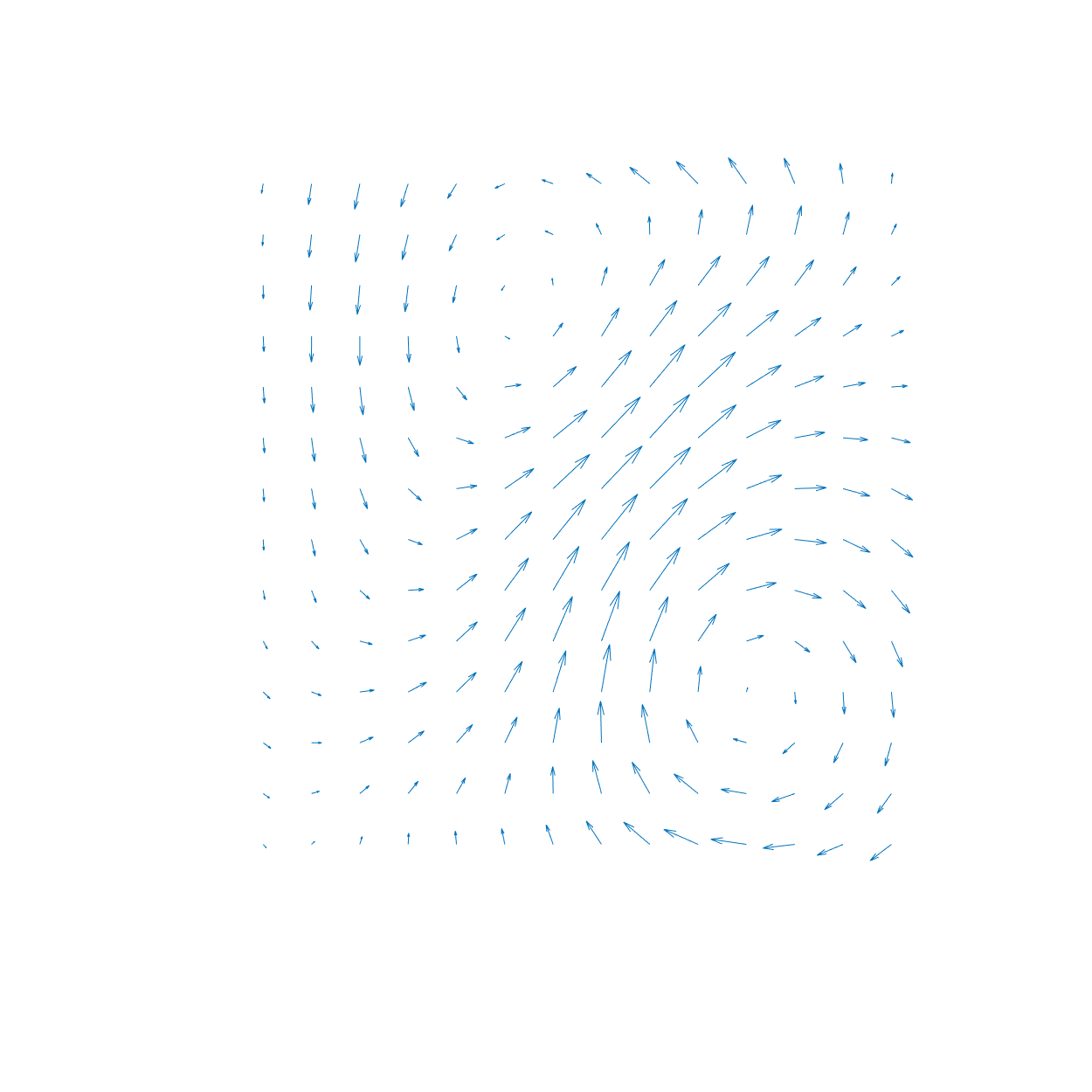

Supplement: S1 MCG raw data 1 — The raw MCG dataset includes categories 0-4 for testing. (ZIP) [file pone.0338189.s001.zip › test/0/p5_410_4.png]

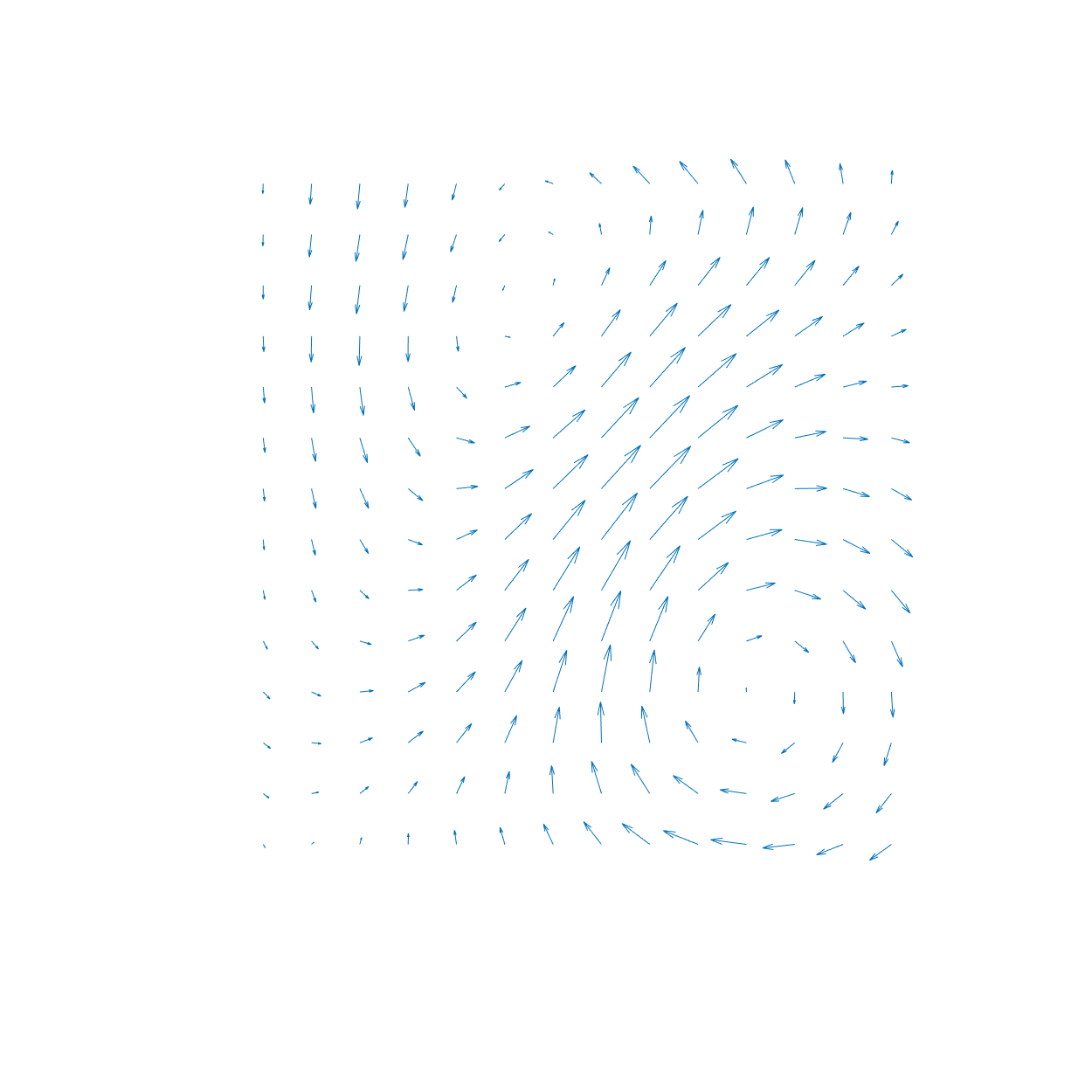

Supplement: S1 MCG raw data 1 — The raw MCG dataset includes categories 0-4 for testing. (ZIP) [file pone.0338189.s001.zip › test/0/p5_415_4.png]

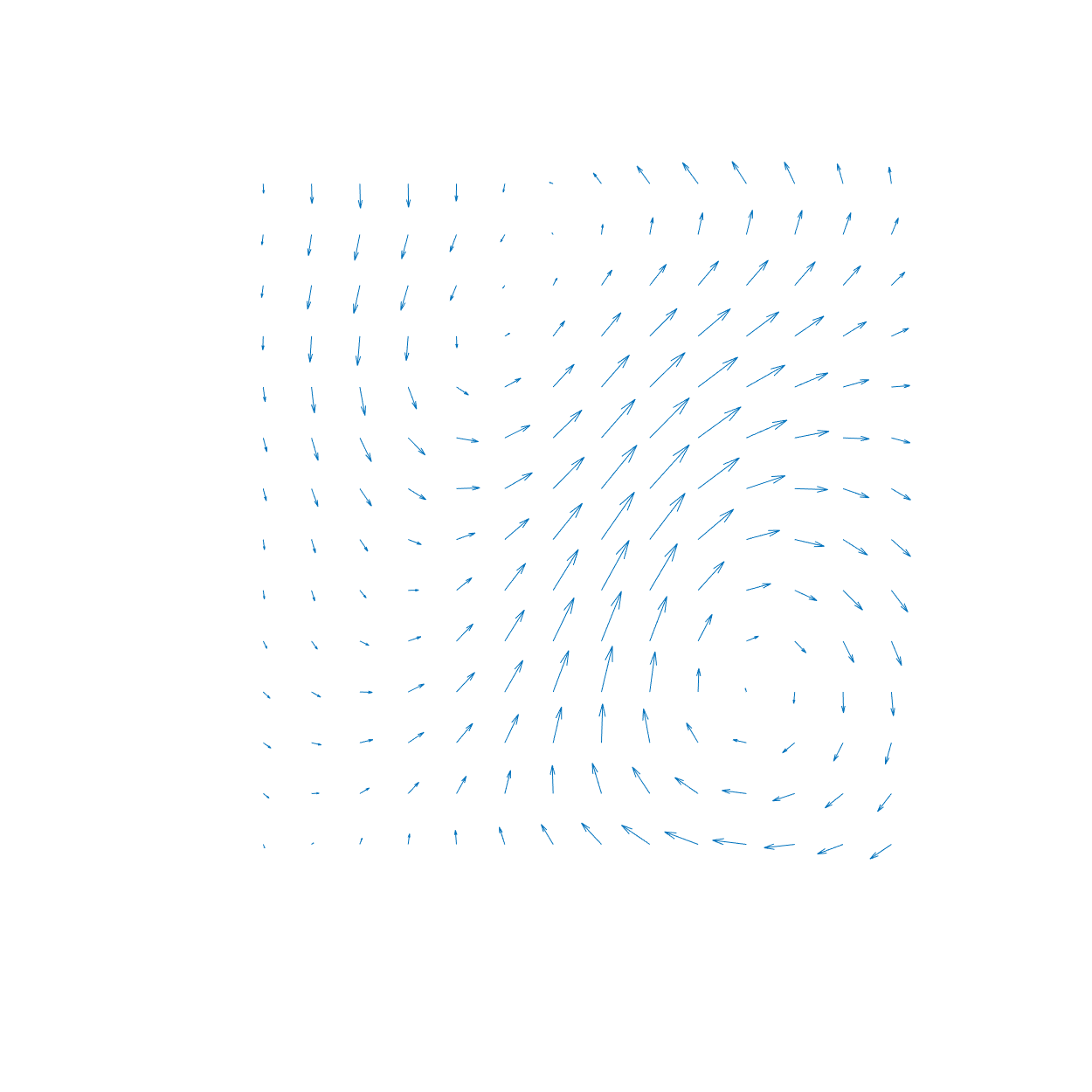

Supplement: S1 MCG raw data 1 — The raw MCG dataset includes categories 0-4 for testing. (ZIP) [file pone.0338189.s001.zip › test/0/p5_425_4.png]

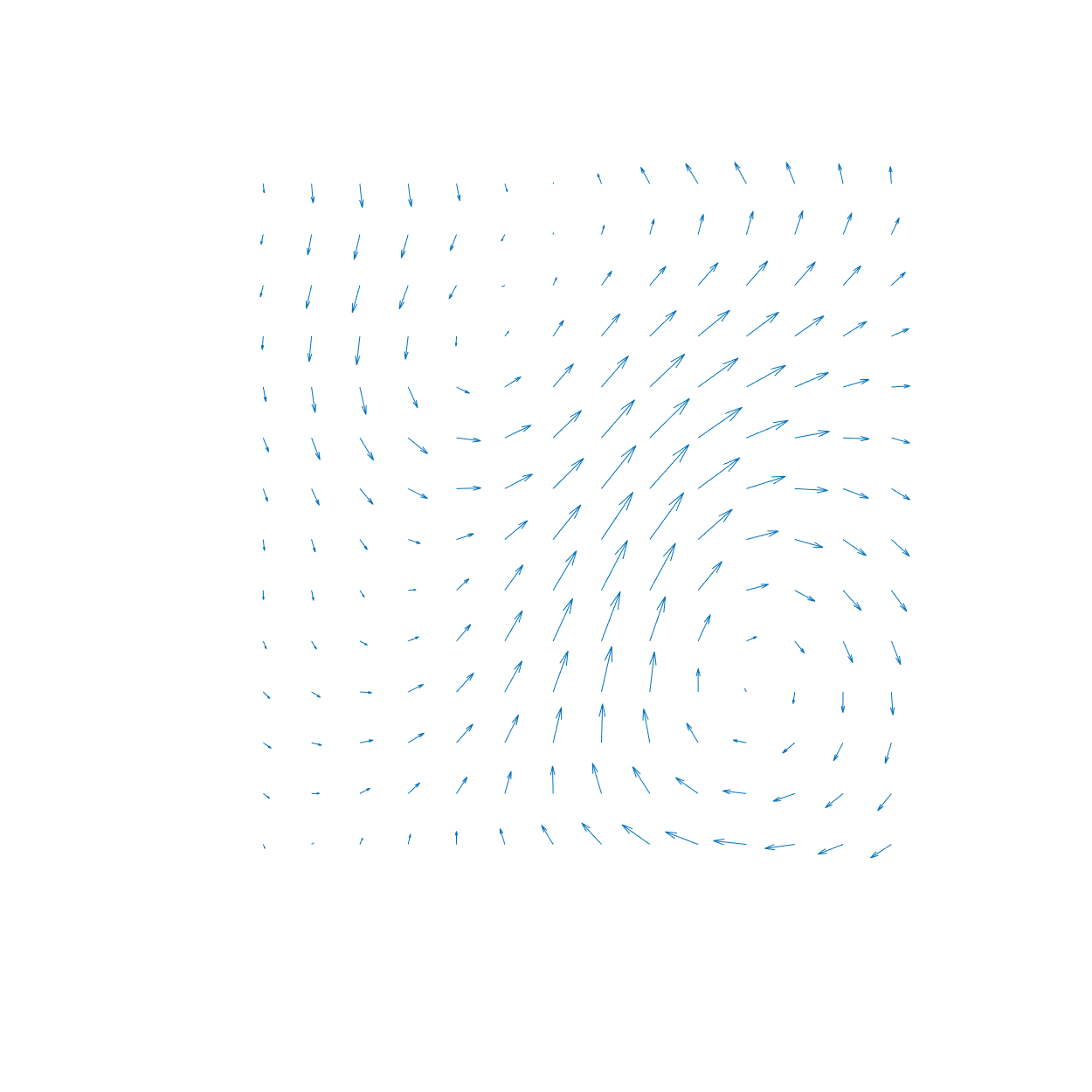

Supplement: S1 MCG raw data 1 — The raw MCG dataset includes categories 0-4 for testing. (ZIP) [file pone.0338189.s001.zip › test/0/p5_430_4.png]

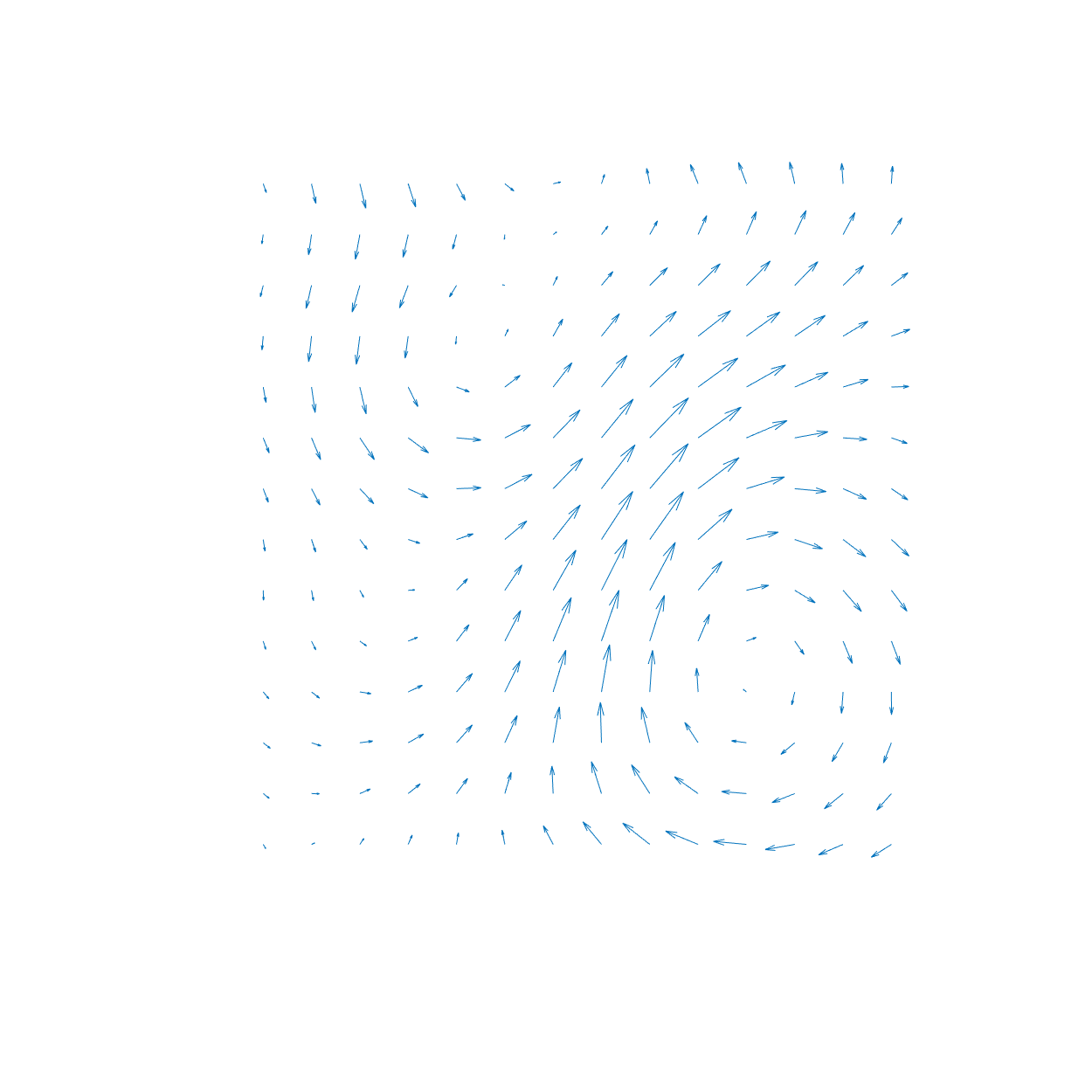

Supplement: S1 MCG raw data 1 — The raw MCG dataset includes categories 0-4 for testing. (ZIP) [file pone.0338189.s001.zip › test/0/p5_435_4.png]

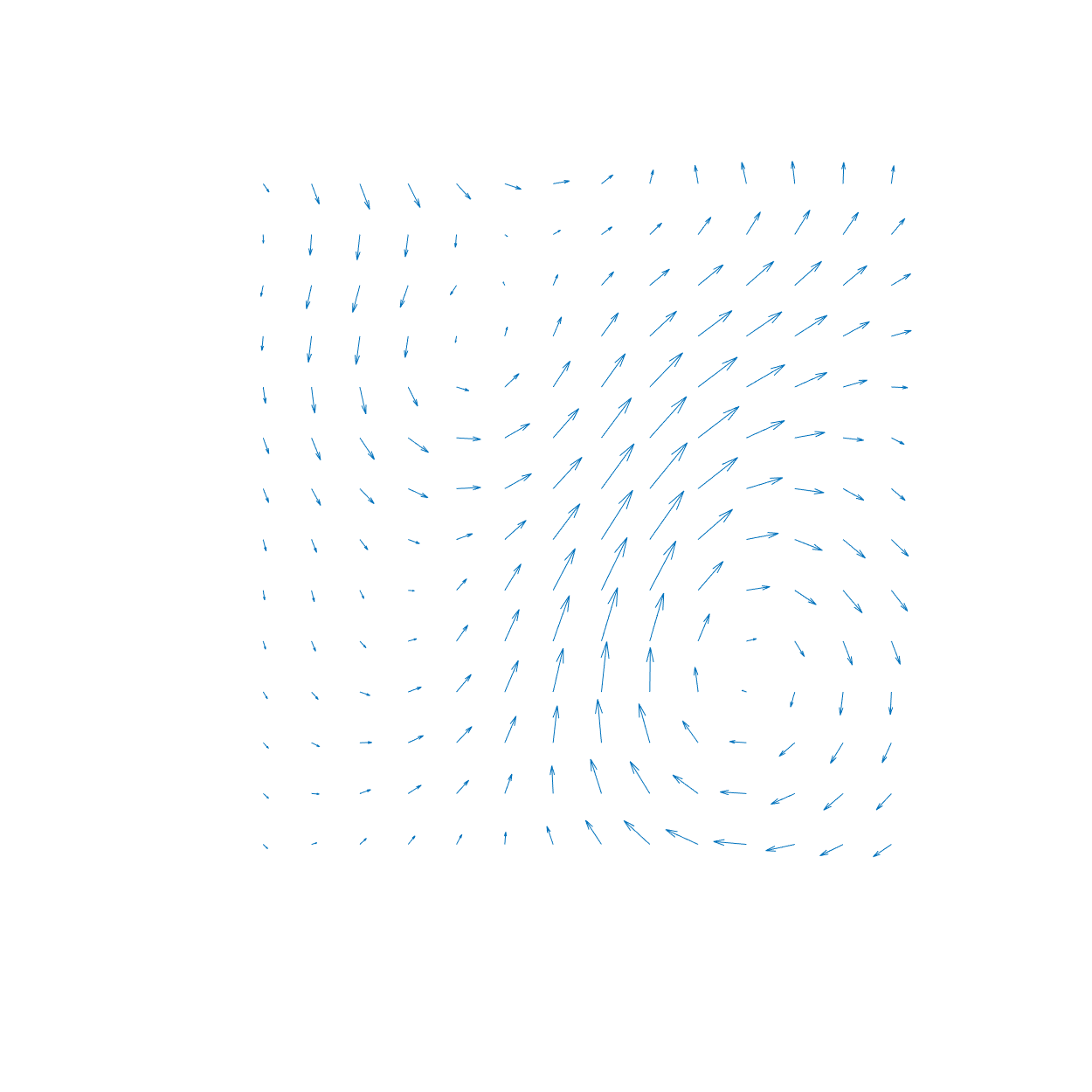

Supplement: S1 MCG raw data 1 — The raw MCG dataset includes categories 0-4 for testing. (ZIP) [file pone.0338189.s001.zip › test/0/p5_440_4.png]

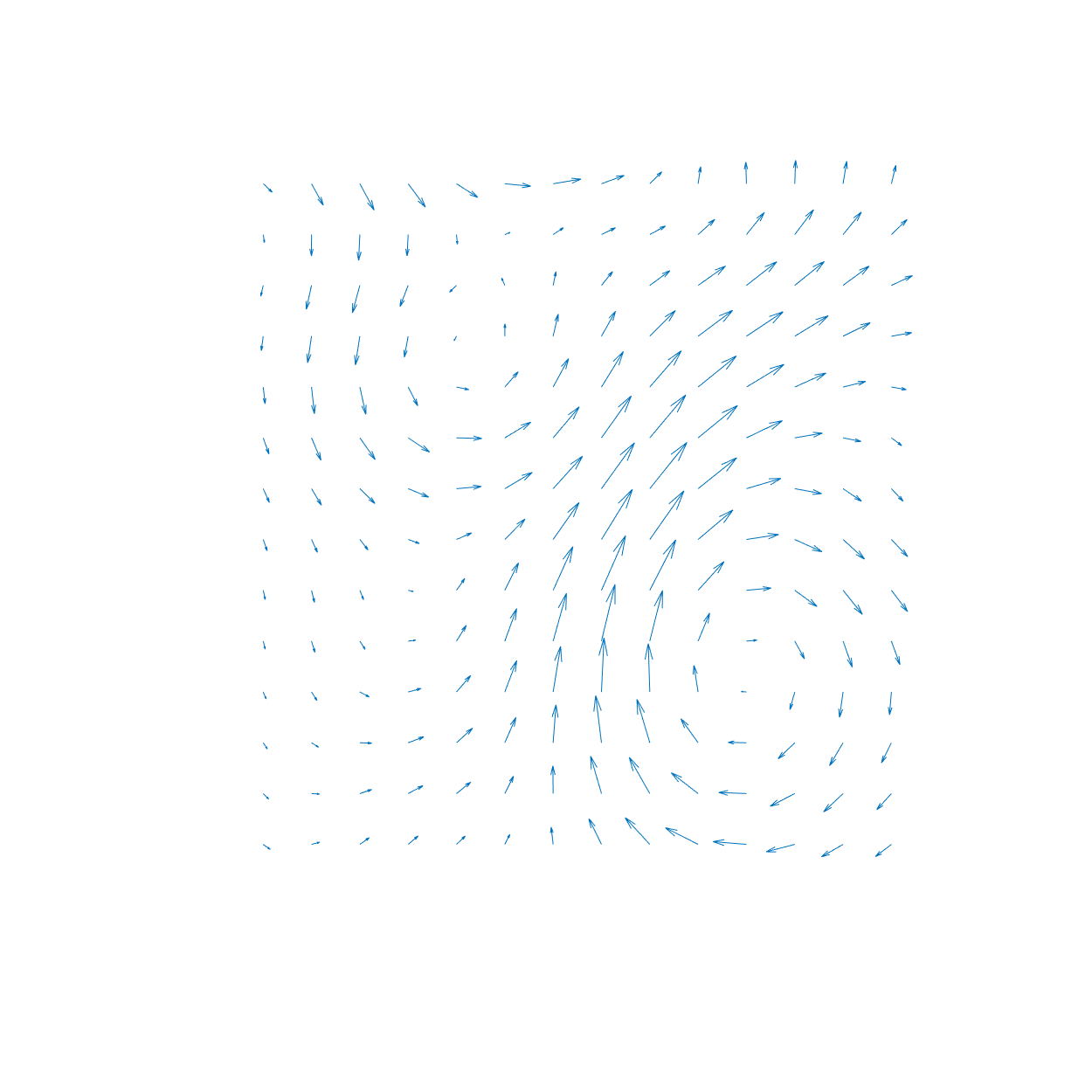

Supplement: S1 MCG raw data 1 — The raw MCG dataset includes categories 0-4 for testing. (ZIP) [file pone.0338189.s001.zip › test/0/p5_445_4.png]

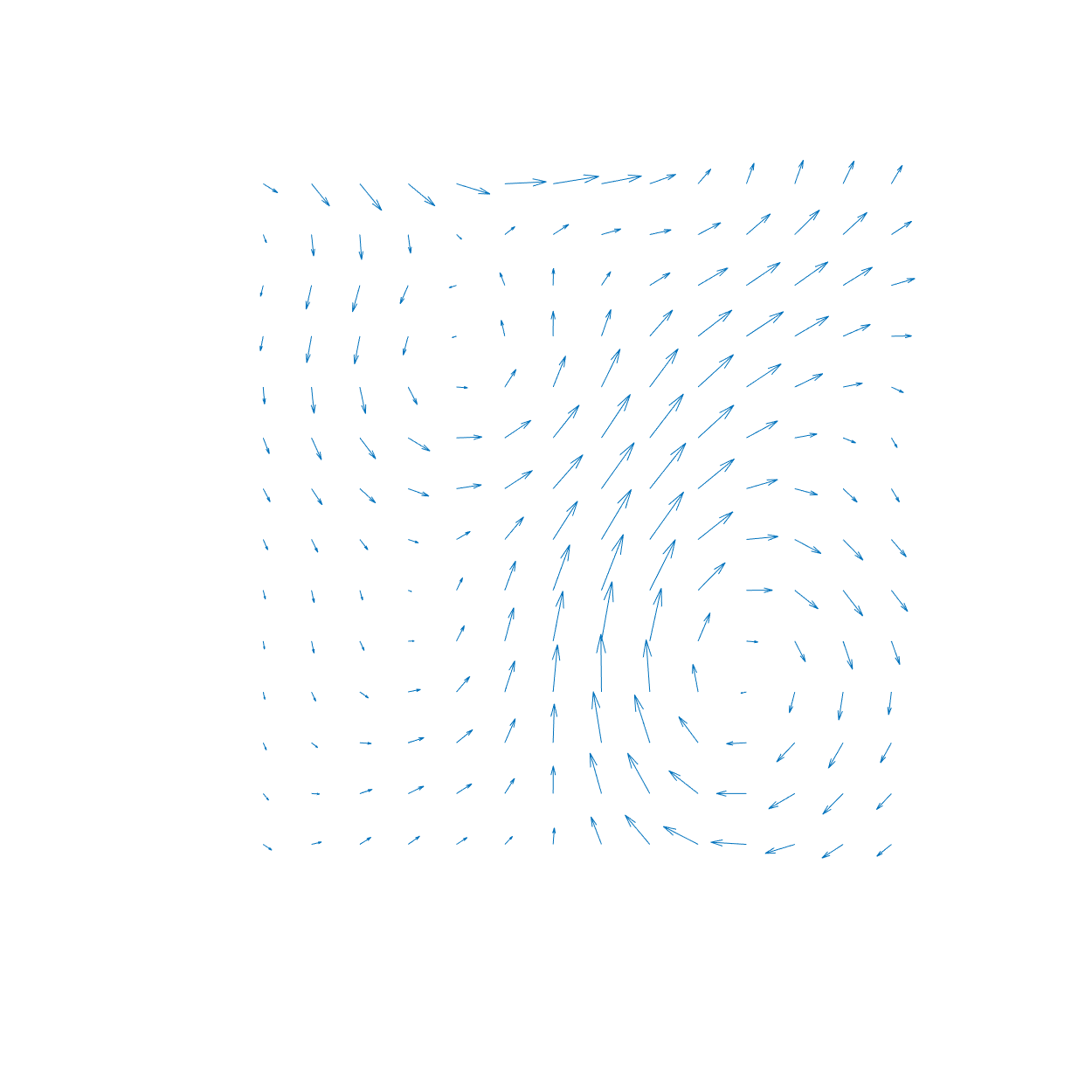

Supplement: S1 MCG raw data 1 — The raw MCG dataset includes categories 0-4 for testing. (ZIP) [file pone.0338189.s001.zip › test/0/p5_450_4.png]

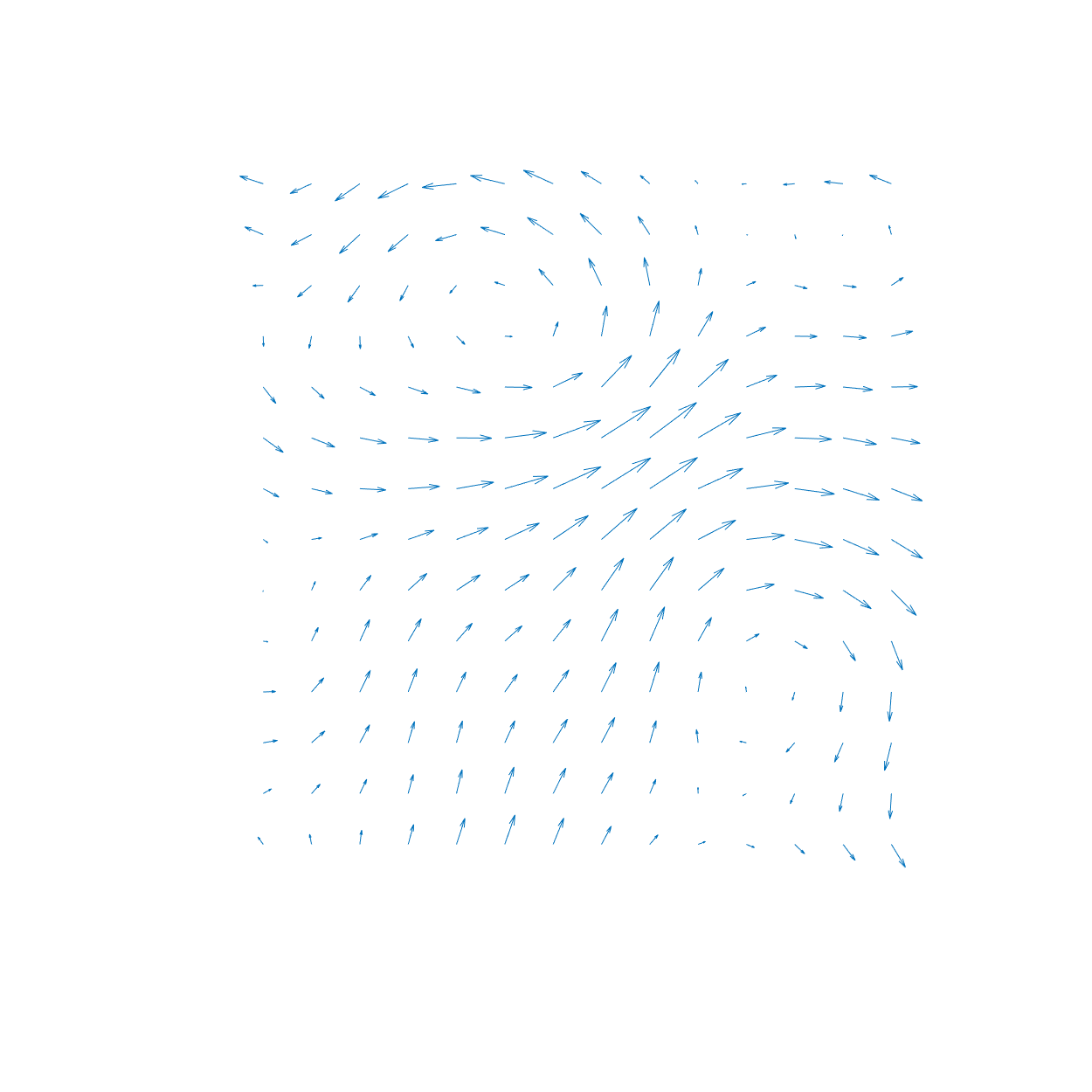

Supplement: S1 MCG raw data 1 — The raw MCG dataset includes categories 0-4 for testing. (ZIP) [file pone.0338189.s001.zip › test/1/p11_470_4.png]

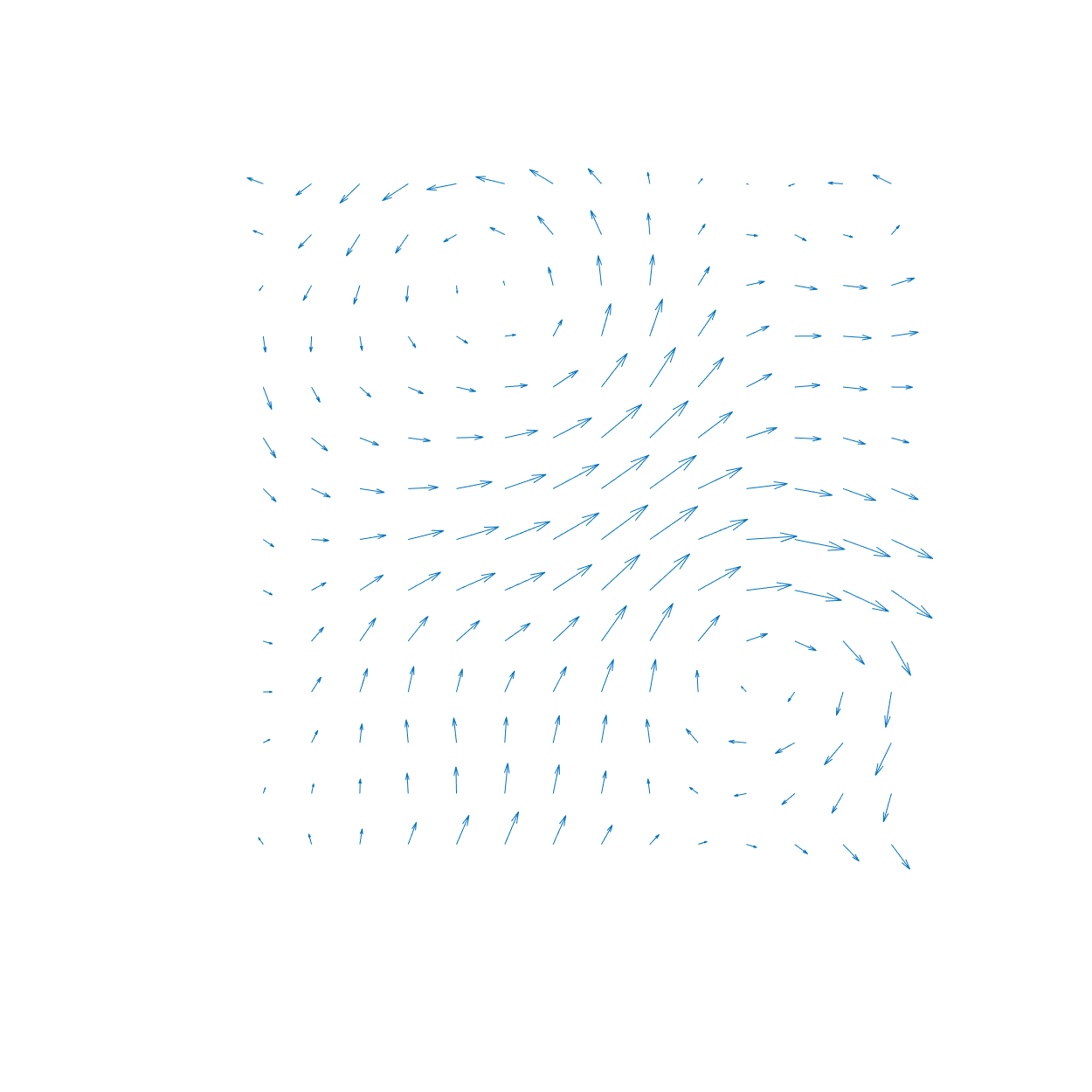

Supplement: S1 MCG raw data 1 — The raw MCG dataset includes categories 0-4 for testing. (ZIP) [file pone.0338189.s001.zip › test/1/p11_475_4.png]

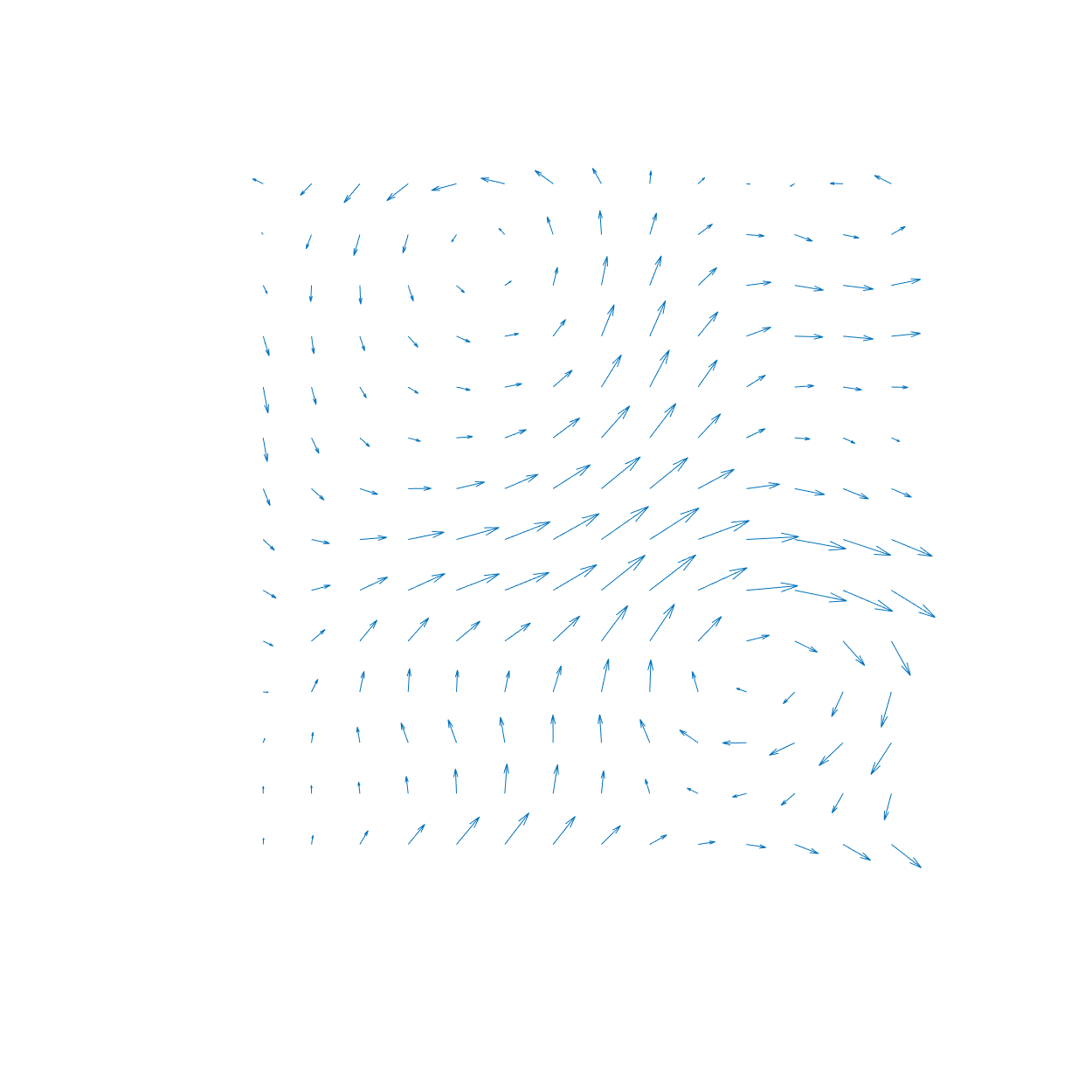

Supplement: S1 MCG raw data 1 — The raw MCG dataset includes categories 0-4 for testing. (ZIP) [file pone.0338189.s001.zip › test/1/p11_480_4.png]

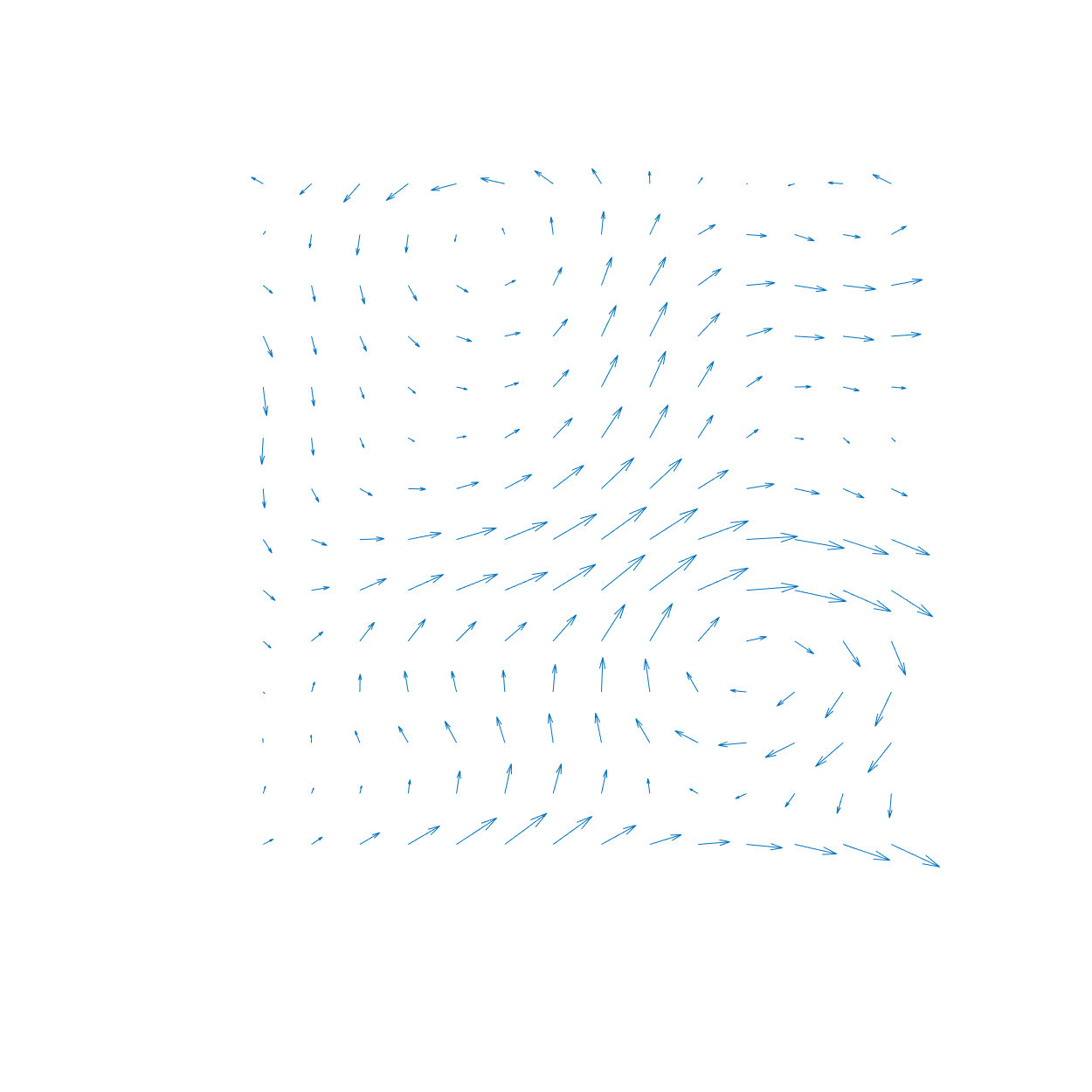

Supplement: S1 MCG raw data 1 — The raw MCG dataset includes categories 0-4 for testing. (ZIP) [file pone.0338189.s001.zip › test/1/p11_485_4.png]

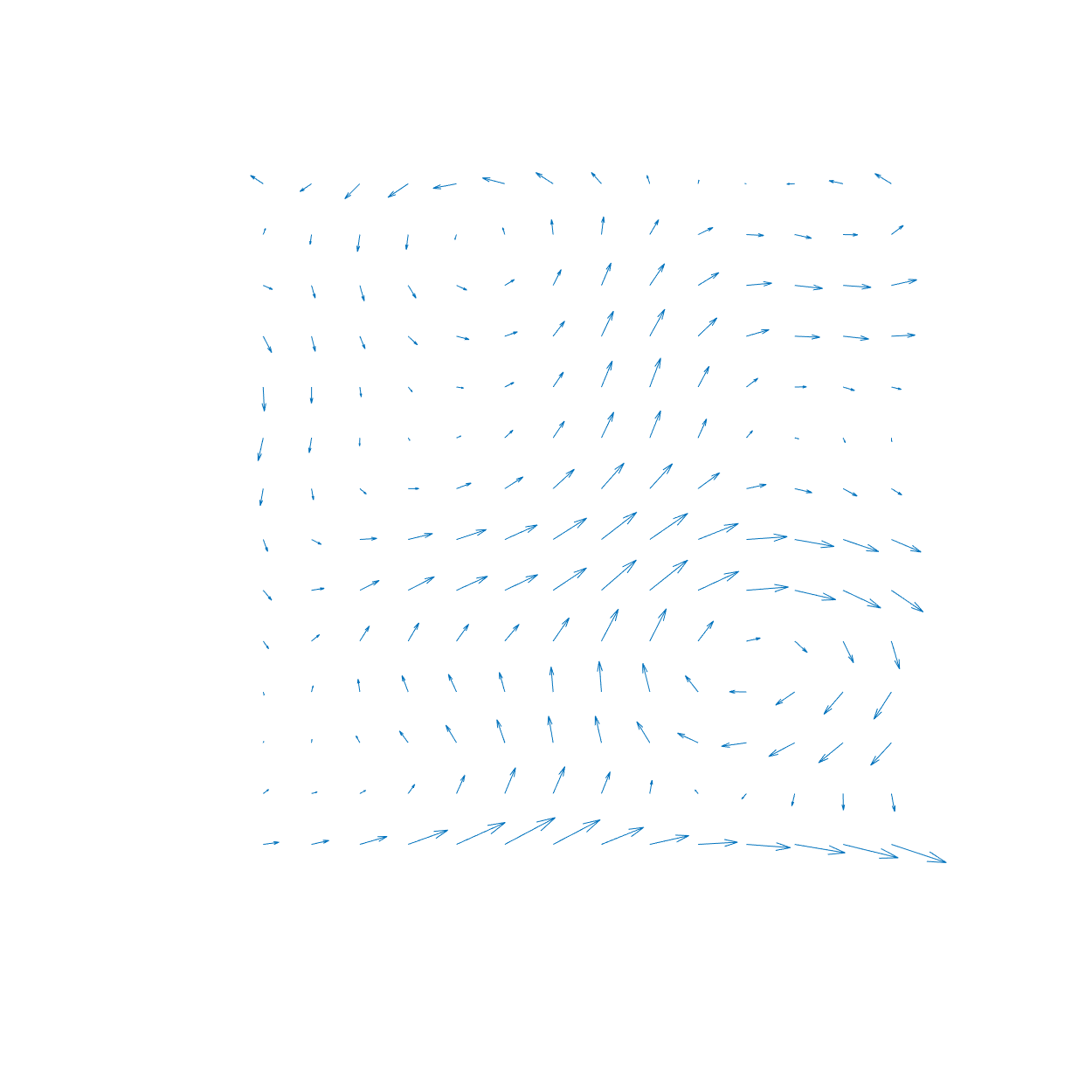

Supplement: S1 MCG raw data 1 — The raw MCG dataset includes categories 0-4 for testing. (ZIP) [file pone.0338189.s001.zip › test/1/p11_490_4.png]

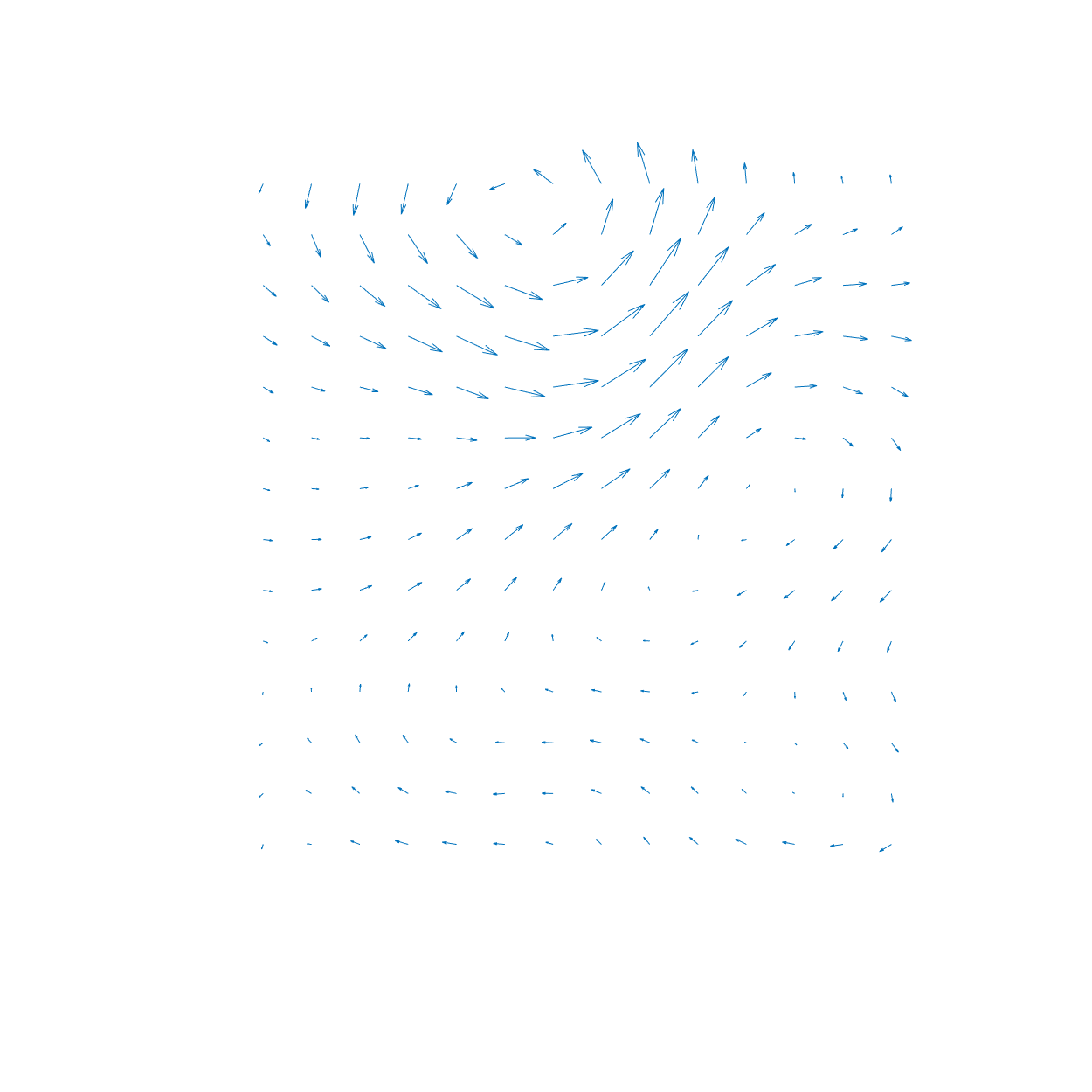

Supplement: S1 MCG raw data 1 — The raw MCG dataset includes categories 0-4 for testing. (ZIP) [file pone.0338189.s001.zip › test/1/p12_380_4.png]

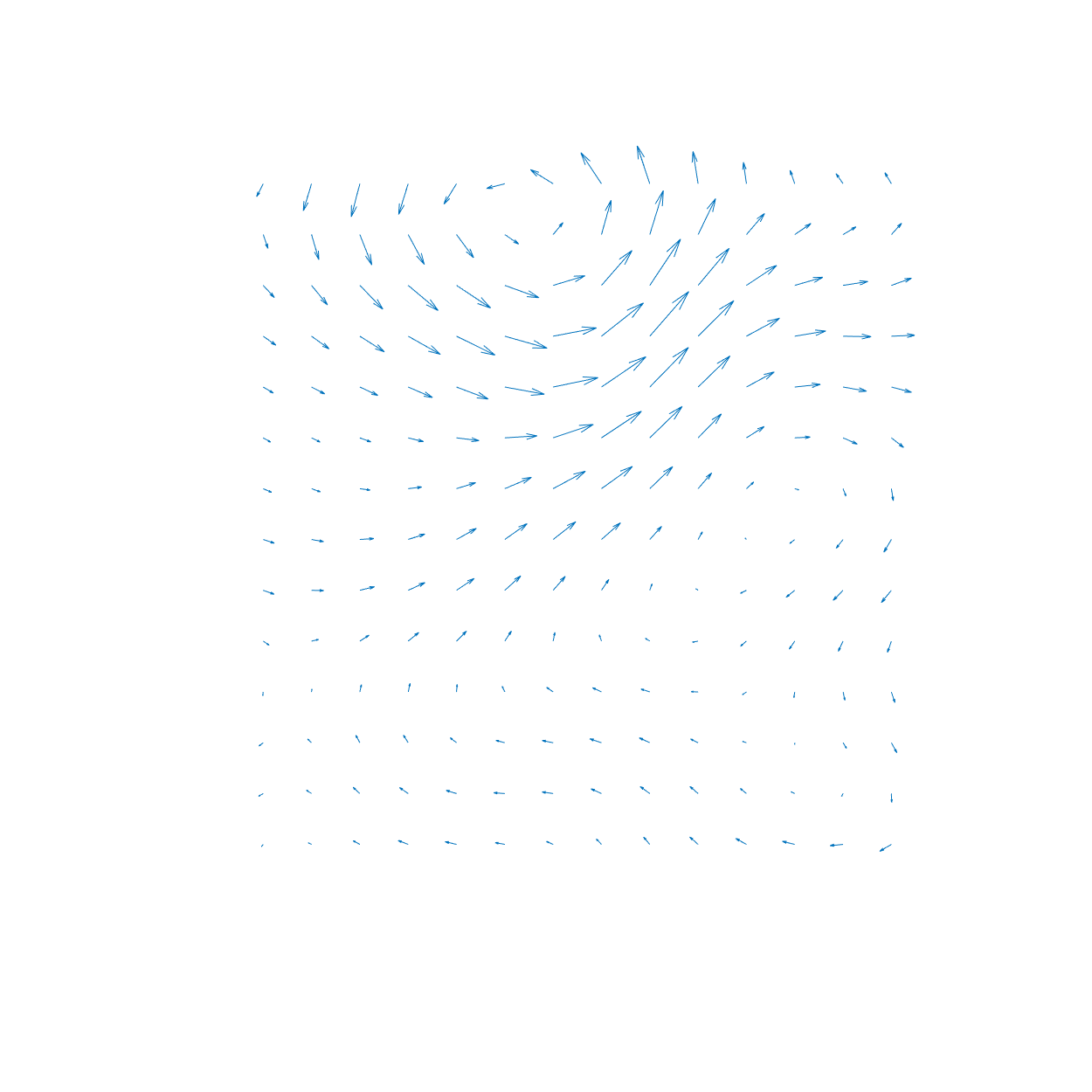

Supplement: S1 MCG raw data 1 — The raw MCG dataset includes categories 0-4 for testing. (ZIP) [file pone.0338189.s001.zip › test/1/p12_385_4.png]

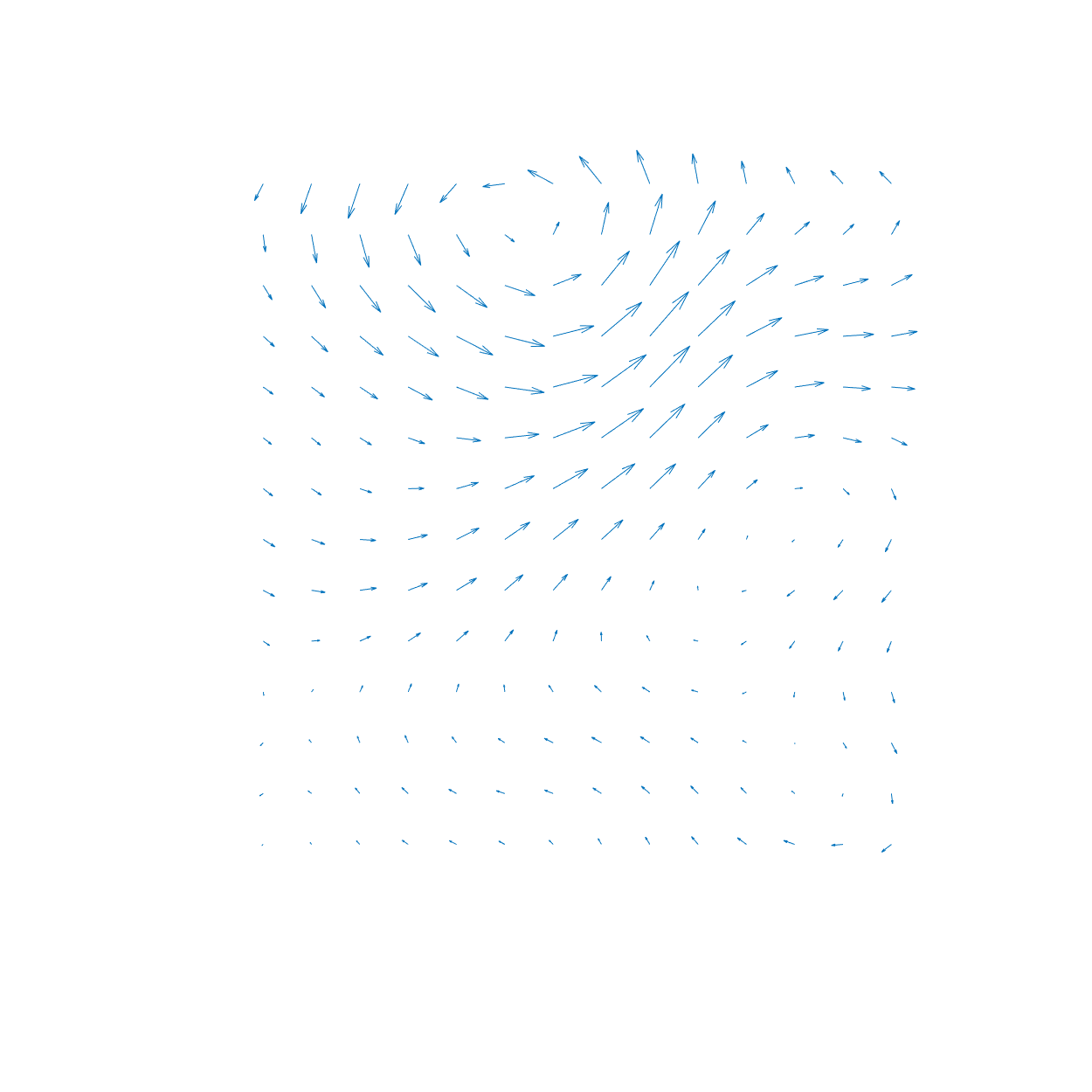

Supplement: S1 MCG raw data 1 — The raw MCG dataset includes categories 0-4 for testing. (ZIP) [file pone.0338189.s001.zip › test/1/p12_390_4.png]

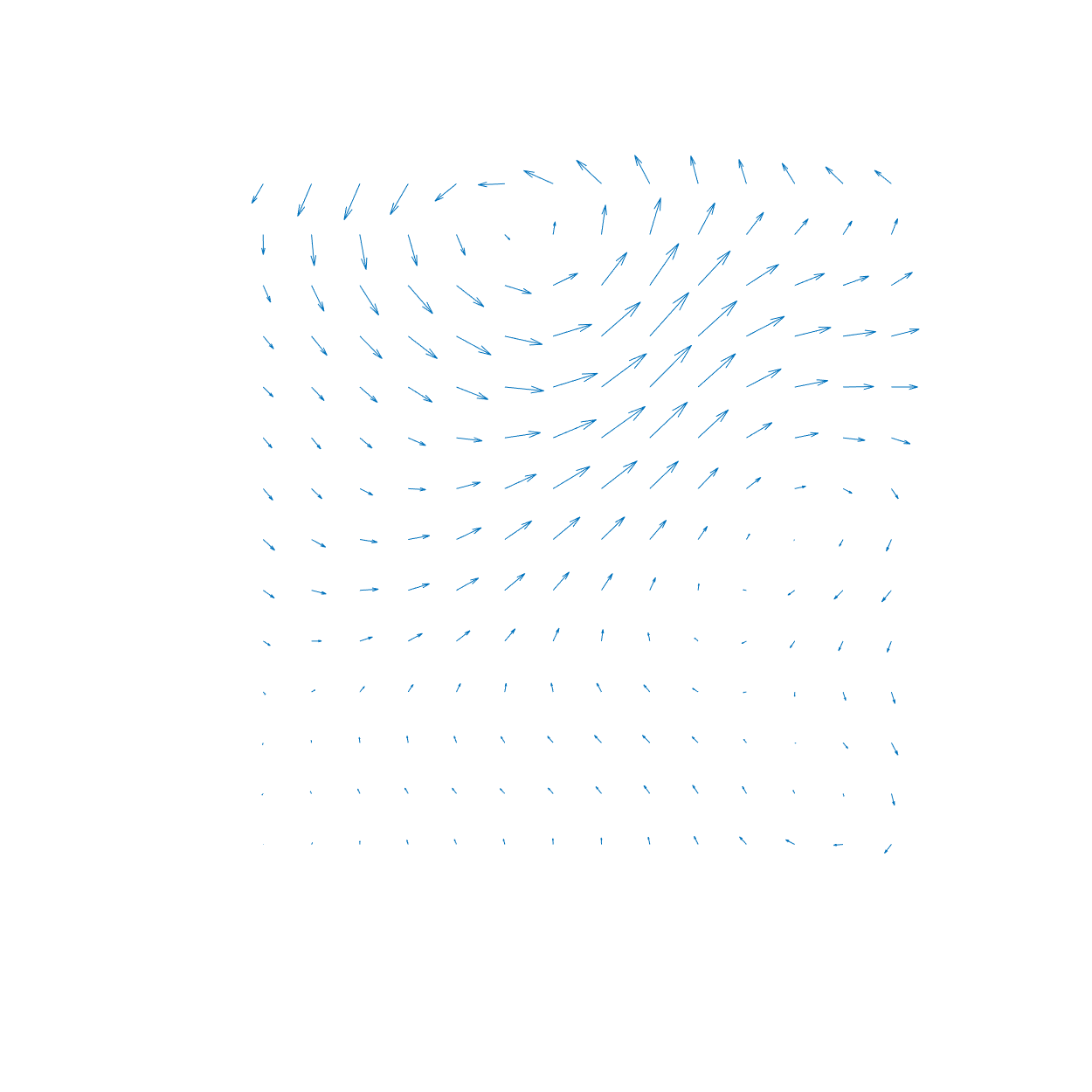

Supplement: S1 MCG raw data 1 — The raw MCG dataset includes categories 0-4 for testing. (ZIP) [file pone.0338189.s001.zip › test/1/p12_395_4.png]

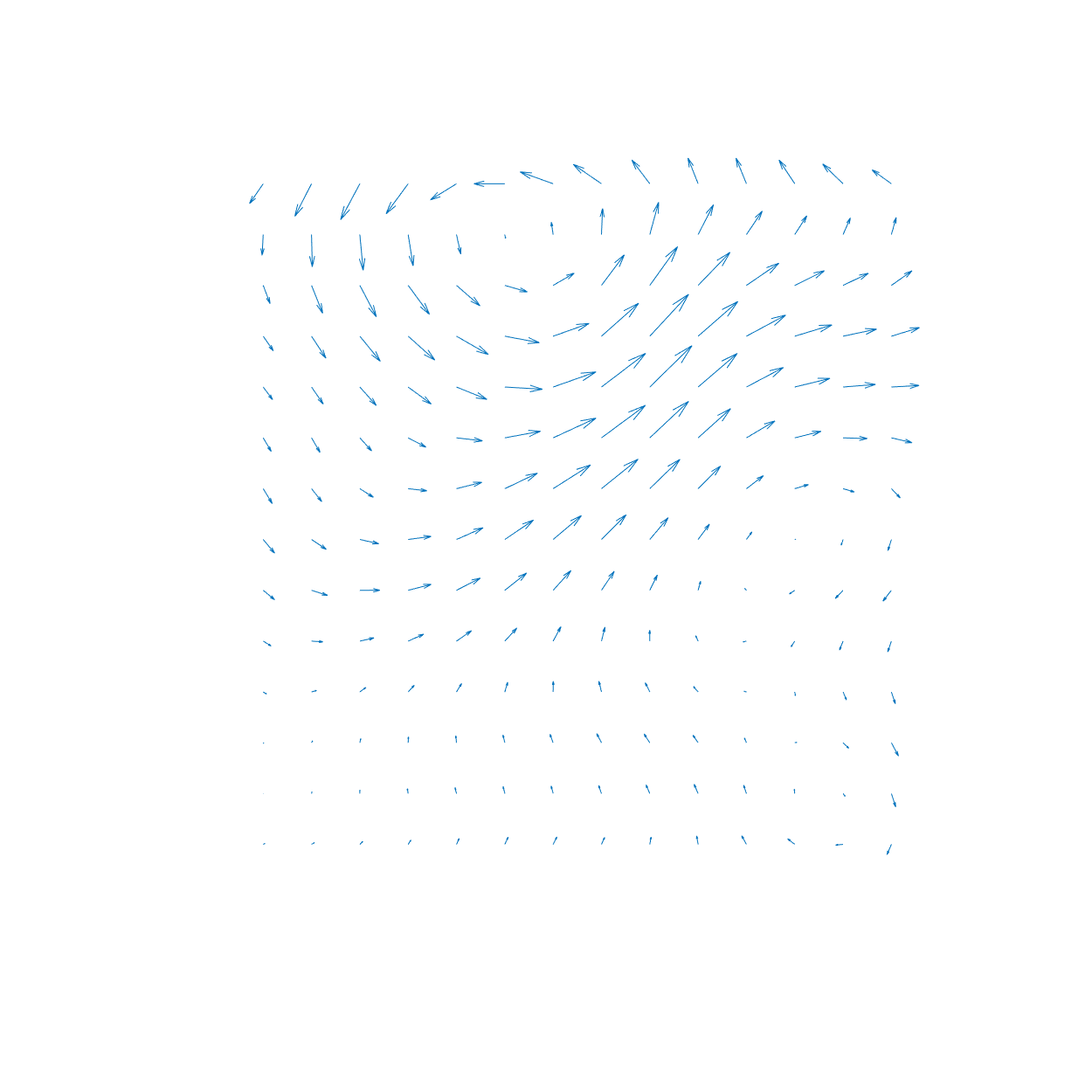

Supplement: S1 MCG raw data 1 — The raw MCG dataset includes categories 0-4 for testing. (ZIP) [file pone.0338189.s001.zip › test/1/p12_400_4.png]

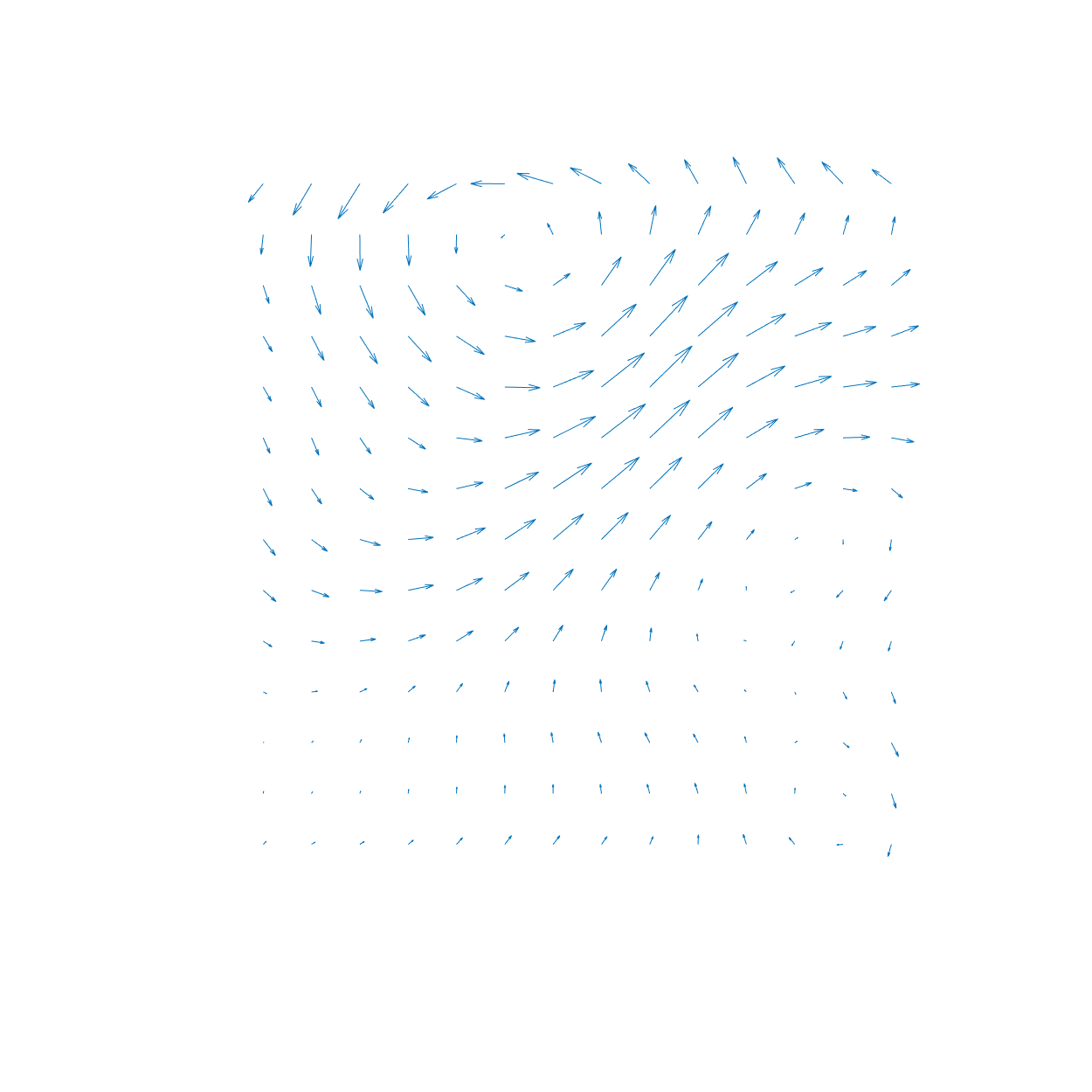

Supplement: S1 MCG raw data 1 — The raw MCG dataset includes categories 0-4 for testing. (ZIP) [file pone.0338189.s001.zip › test/1/p12_405_4.png]

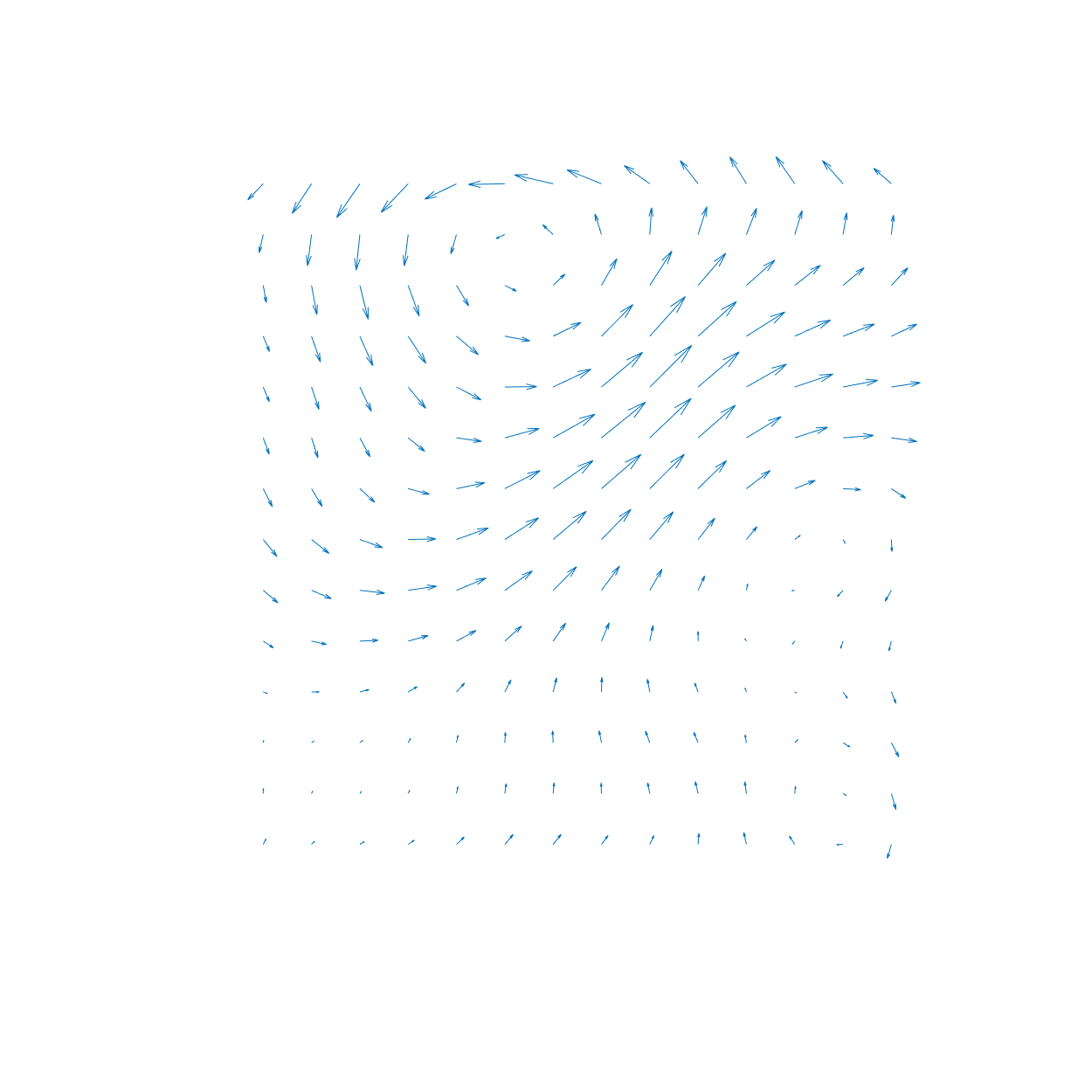

Supplement: S1 MCG raw data 1 — The raw MCG dataset includes categories 0-4 for testing. (ZIP) [file pone.0338189.s001.zip › test/1/p12_410_4.png]

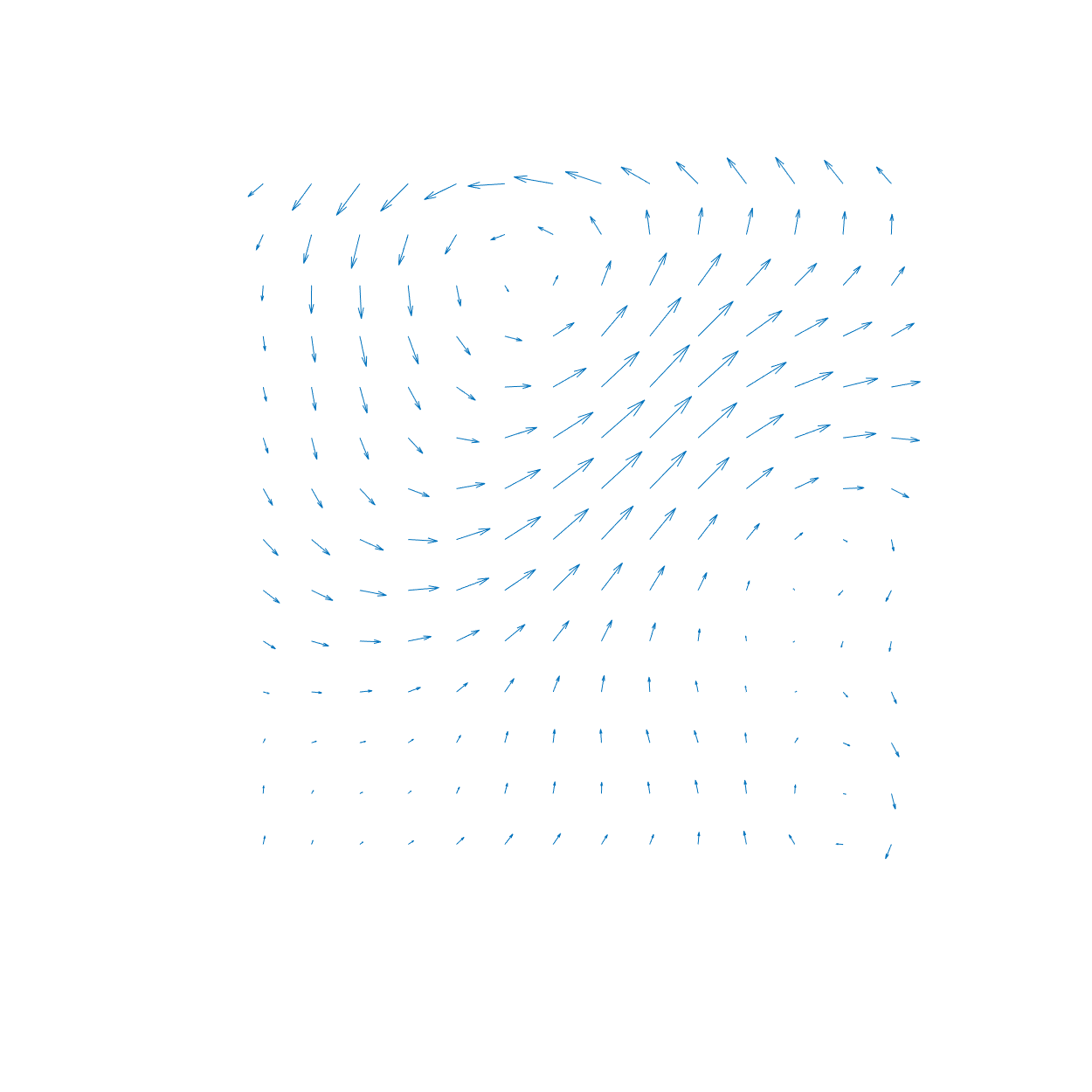

Supplement: S1 MCG raw data 1 — The raw MCG dataset includes categories 0-4 for testing. (ZIP) [file pone.0338189.s001.zip › test/1/p12_415_4.png]

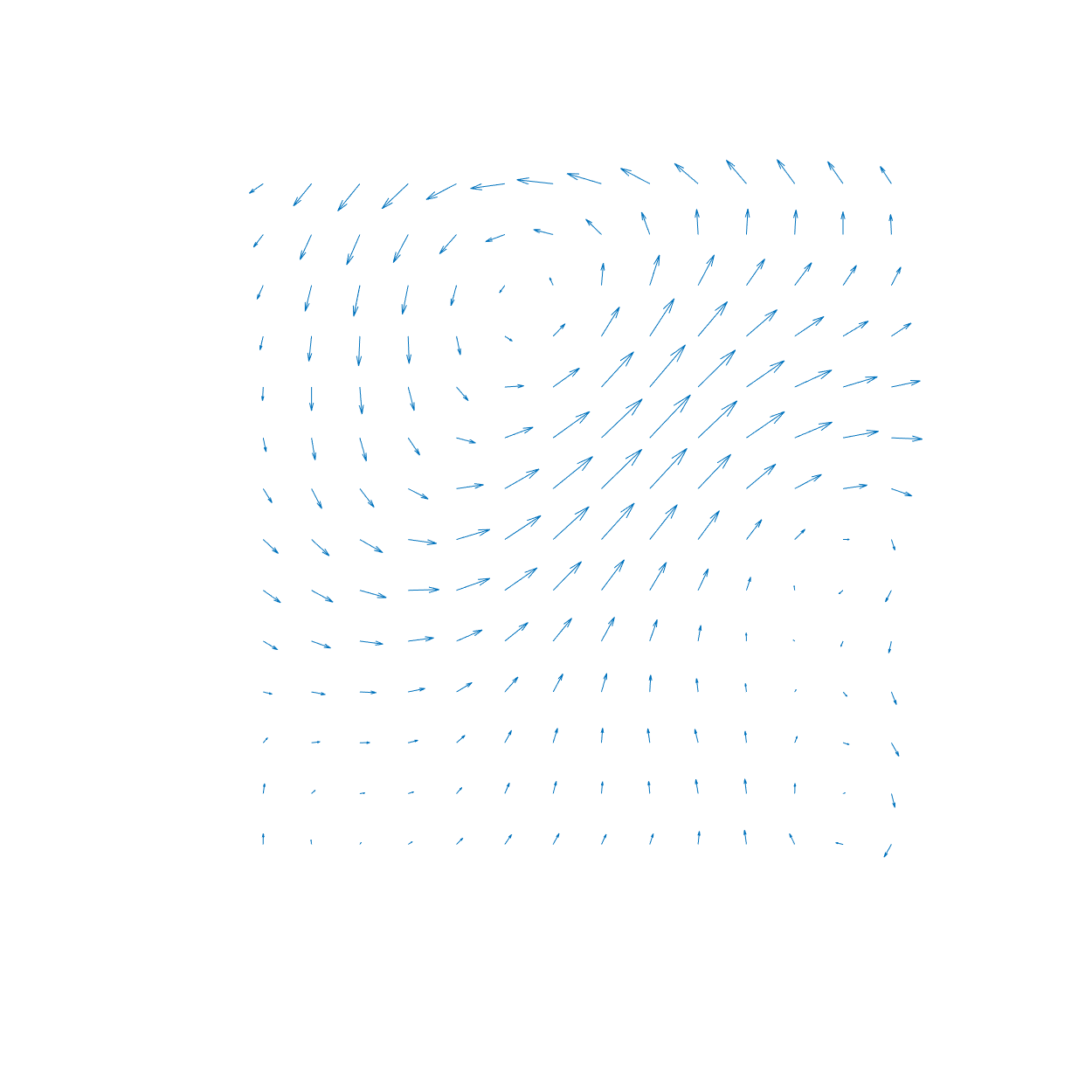

Supplement: S1 MCG raw data 1 — The raw MCG dataset includes categories 0-4 for testing. (ZIP) [file pone.0338189.s001.zip › test/1/p12_420_4.png]

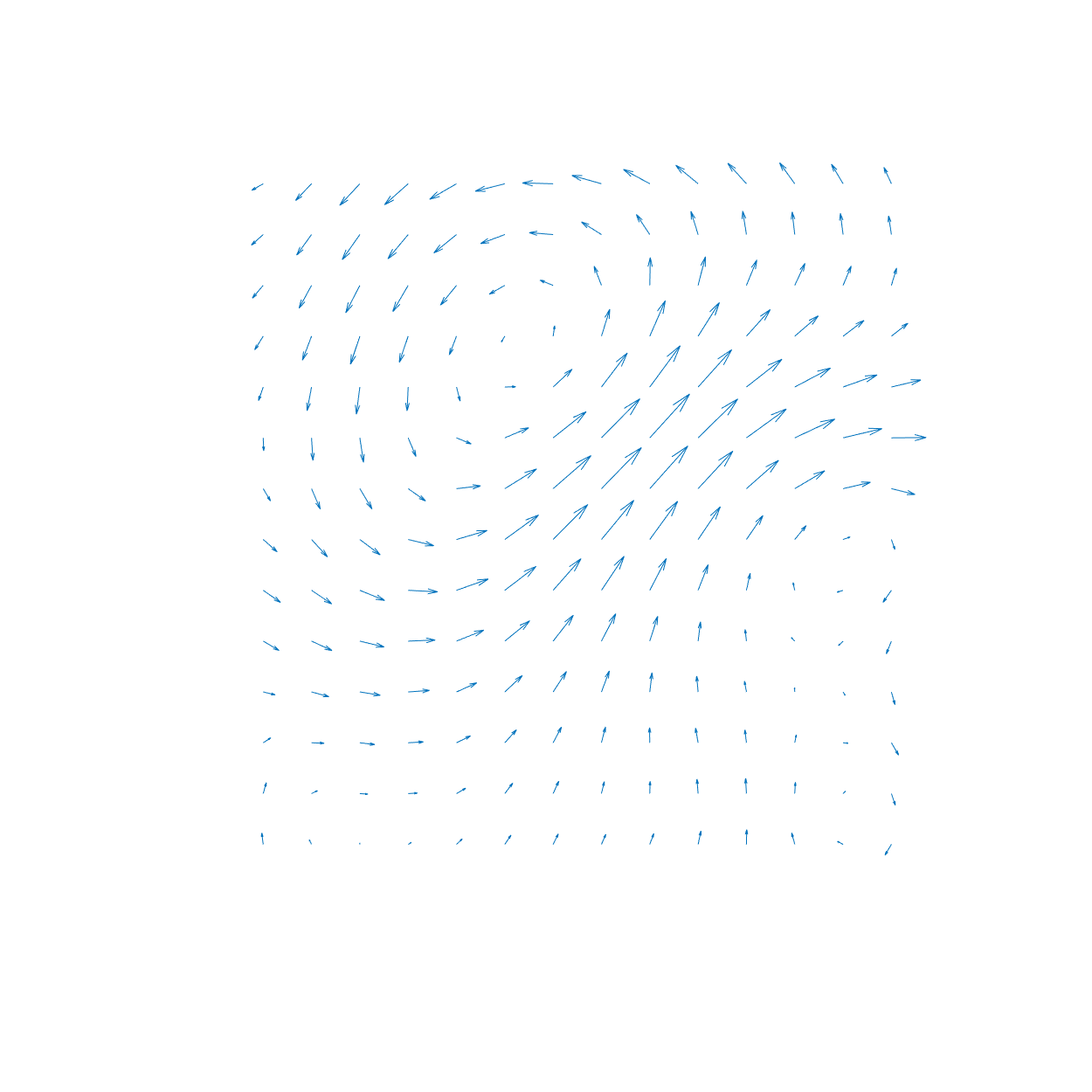

Supplement: S1 MCG raw data 1 — The raw MCG dataset includes categories 0-4 for testing. (ZIP) [file pone.0338189.s001.zip › test/1/p12_425_4.png]

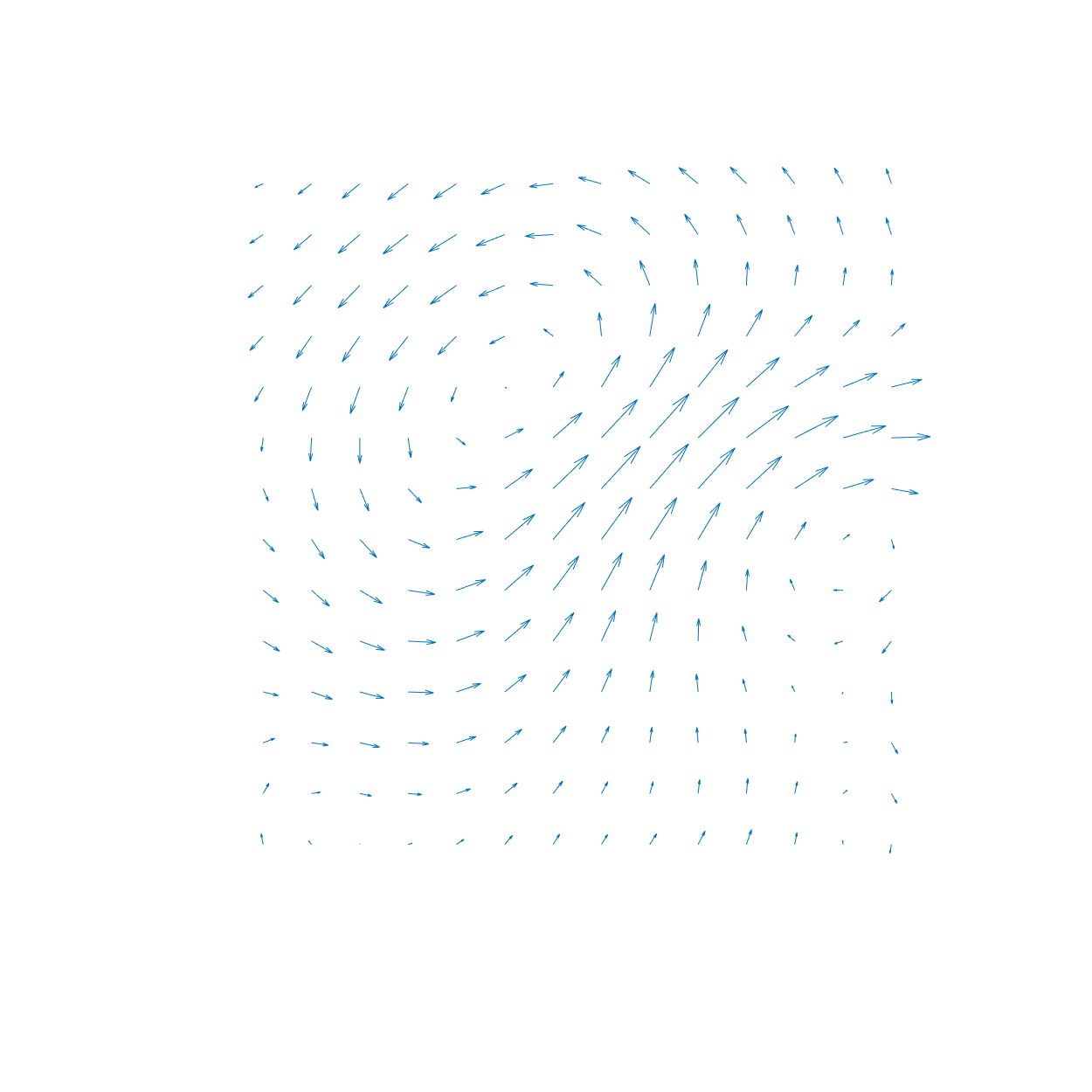

Supplement: S1 MCG raw data 1 — The raw MCG dataset includes categories 0-4 for testing. (ZIP) [file pone.0338189.s001.zip › test/1/p12_430_4.png]

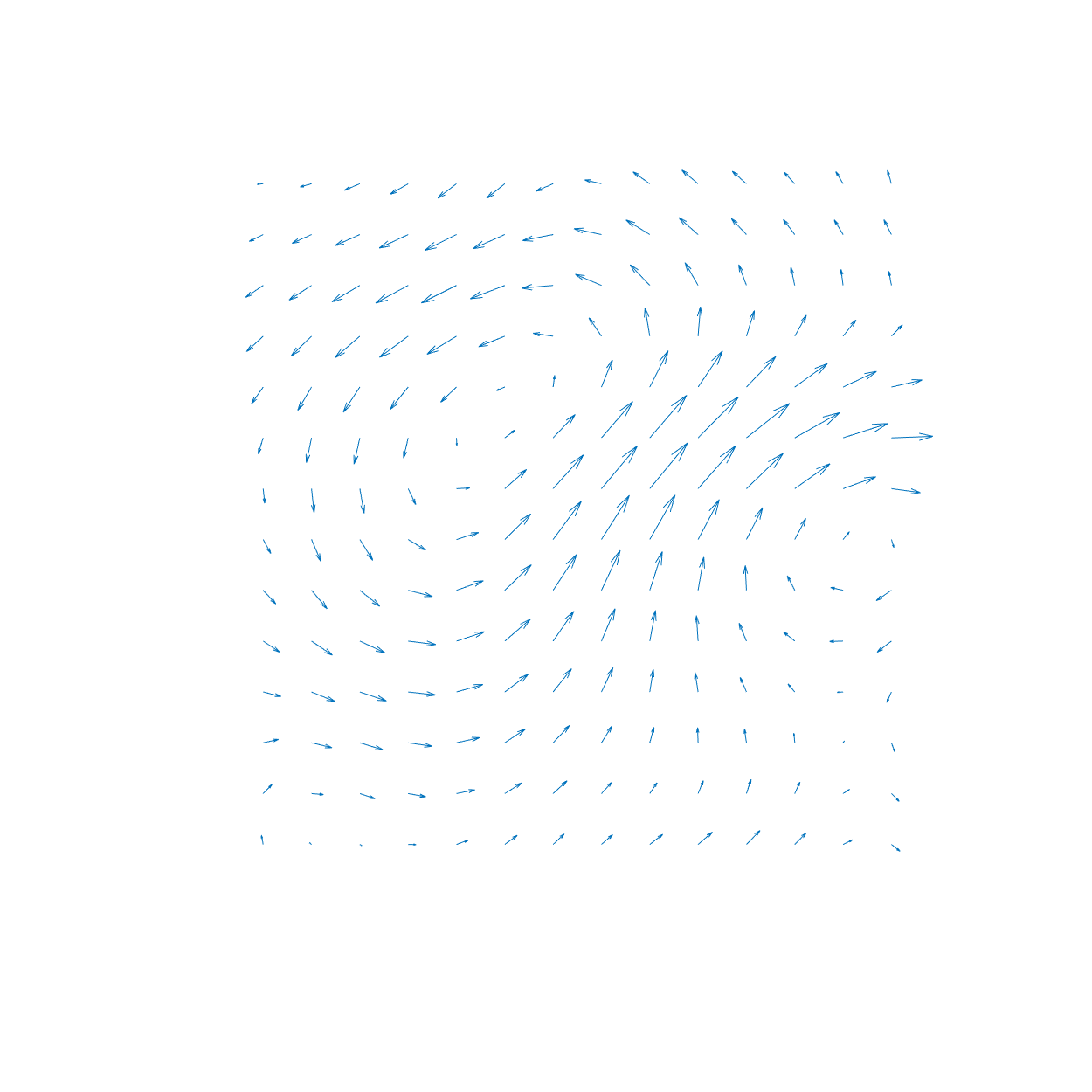

Supplement: S1 MCG raw data 1 — The raw MCG dataset includes categories 0-4 for testing. (ZIP) [file pone.0338189.s001.zip › test/1/p12_435_4.png]

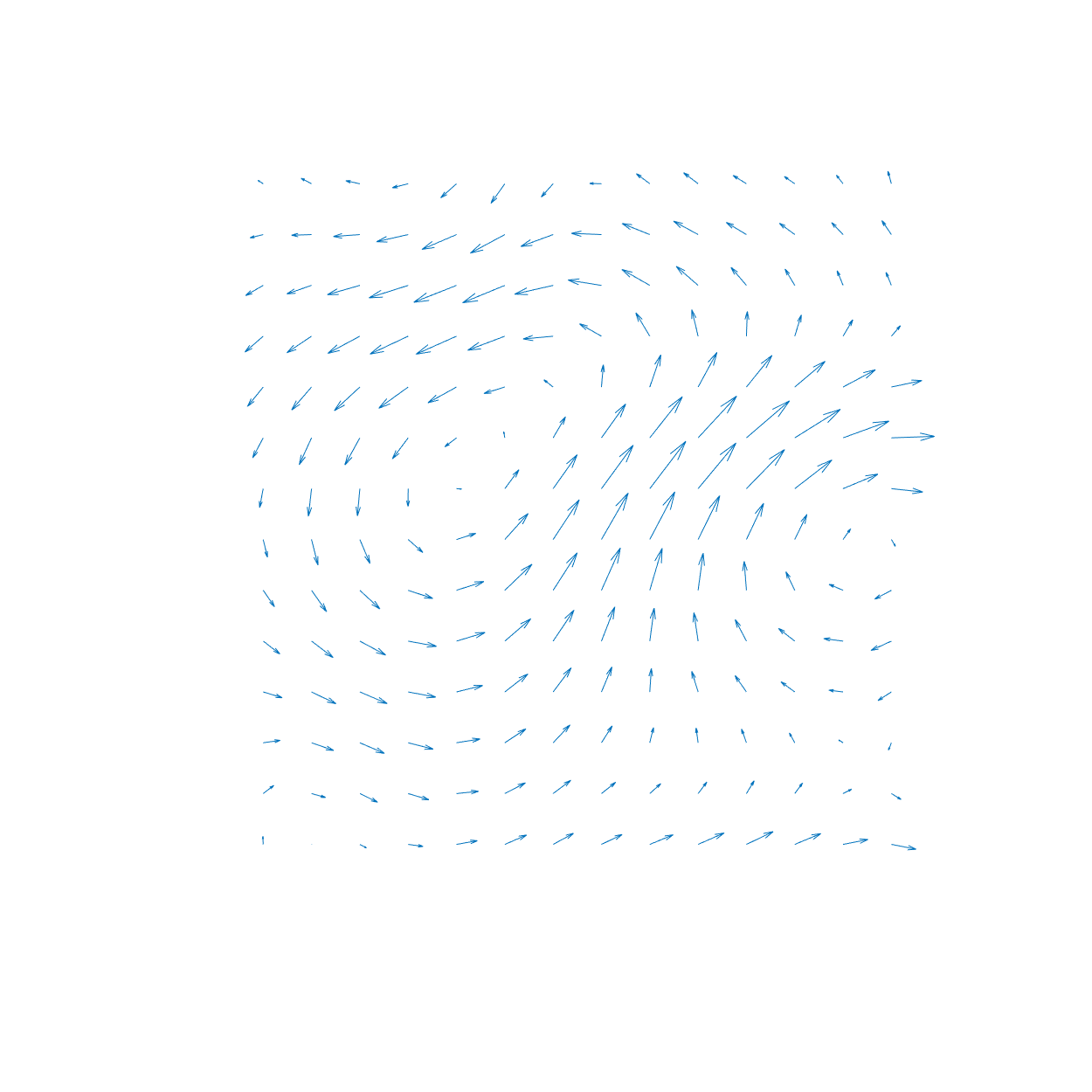

Supplement: S1 MCG raw data 1 — The raw MCG dataset includes categories 0-4 for testing. (ZIP) [file pone.0338189.s001.zip › test/1/p12_440_4.png]

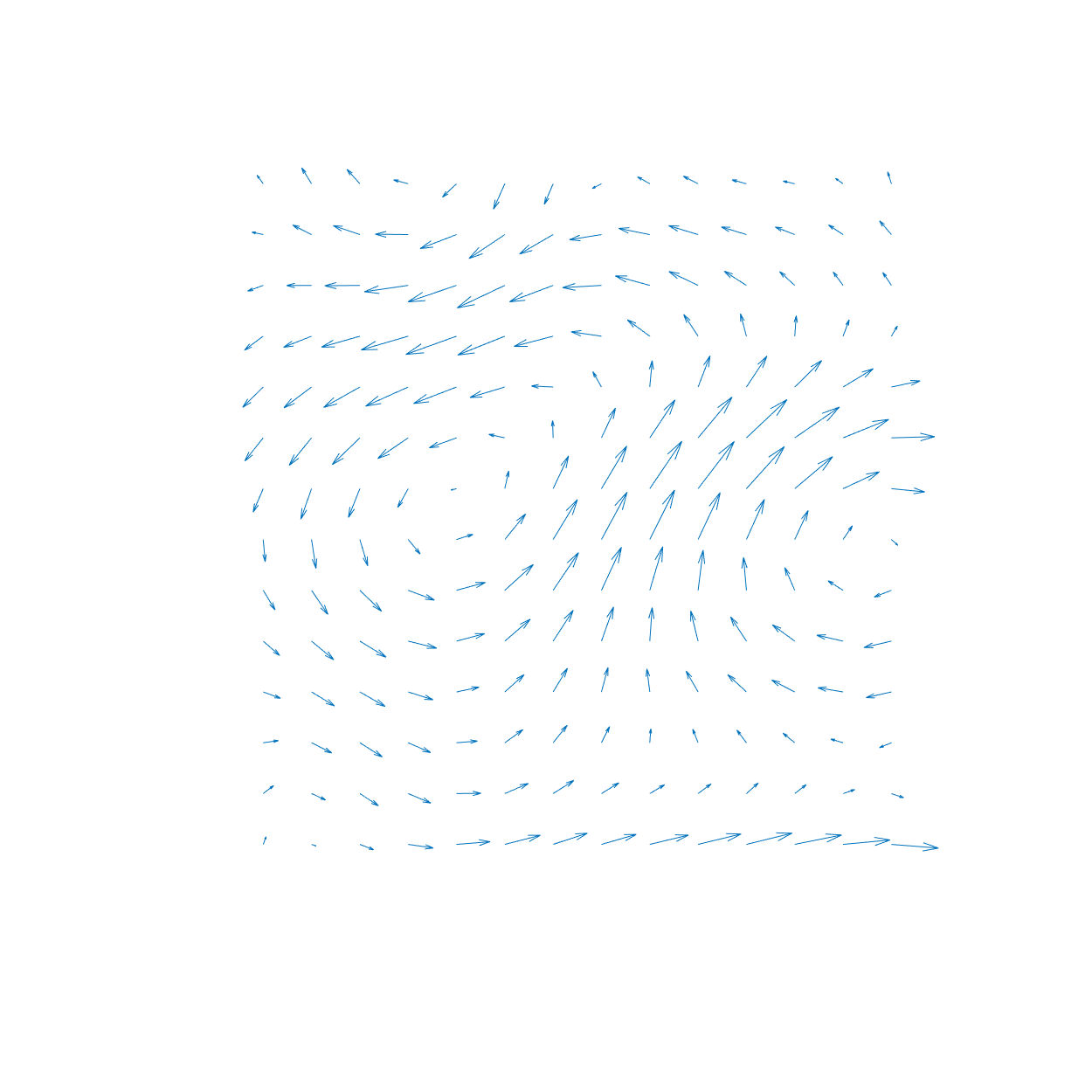

Supplement: S1 MCG raw data 1 — The raw MCG dataset includes categories 0-4 for testing. (ZIP) [file pone.0338189.s001.zip › test/1/p12_445_4.png]

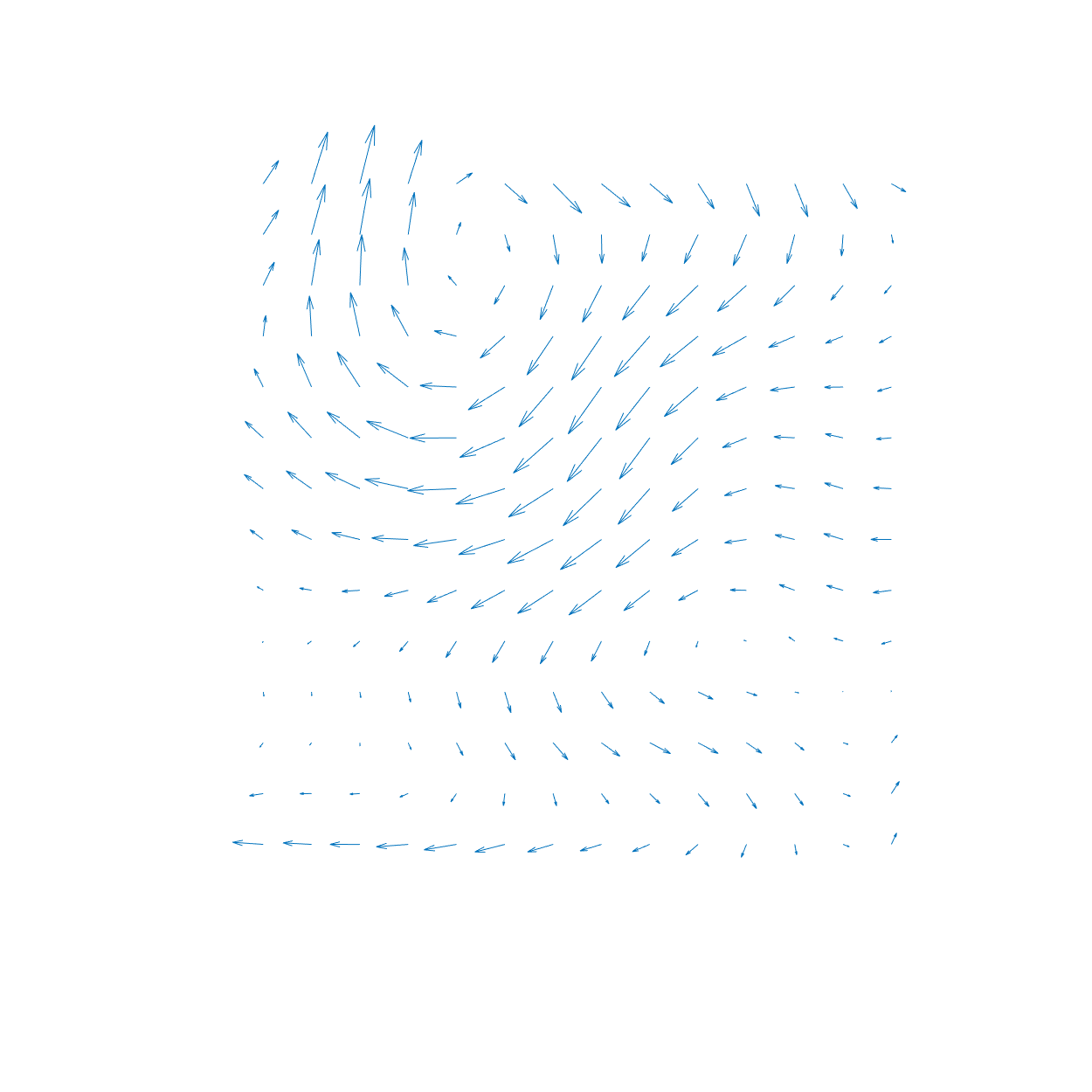

Supplement: S1 MCG raw data 1 — The raw MCG dataset includes categories 0-4 for testing. (ZIP) [file pone.0338189.s001.zip › test/1/p12_485_4.png]

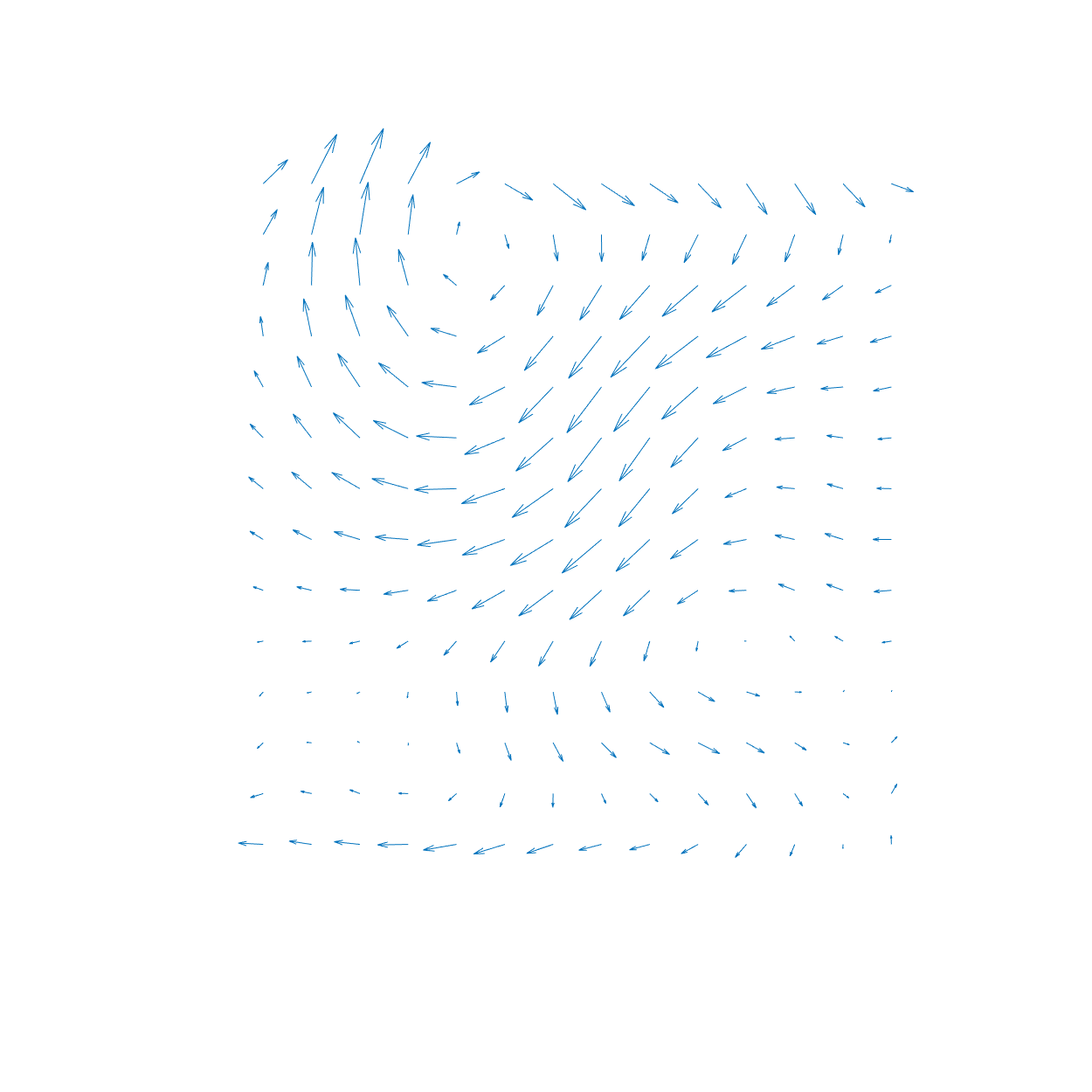

Supplement: S1 MCG raw data 1 — The raw MCG dataset includes categories 0-4 for testing. (ZIP) [file pone.0338189.s001.zip › test/1/p12_490_4.png]

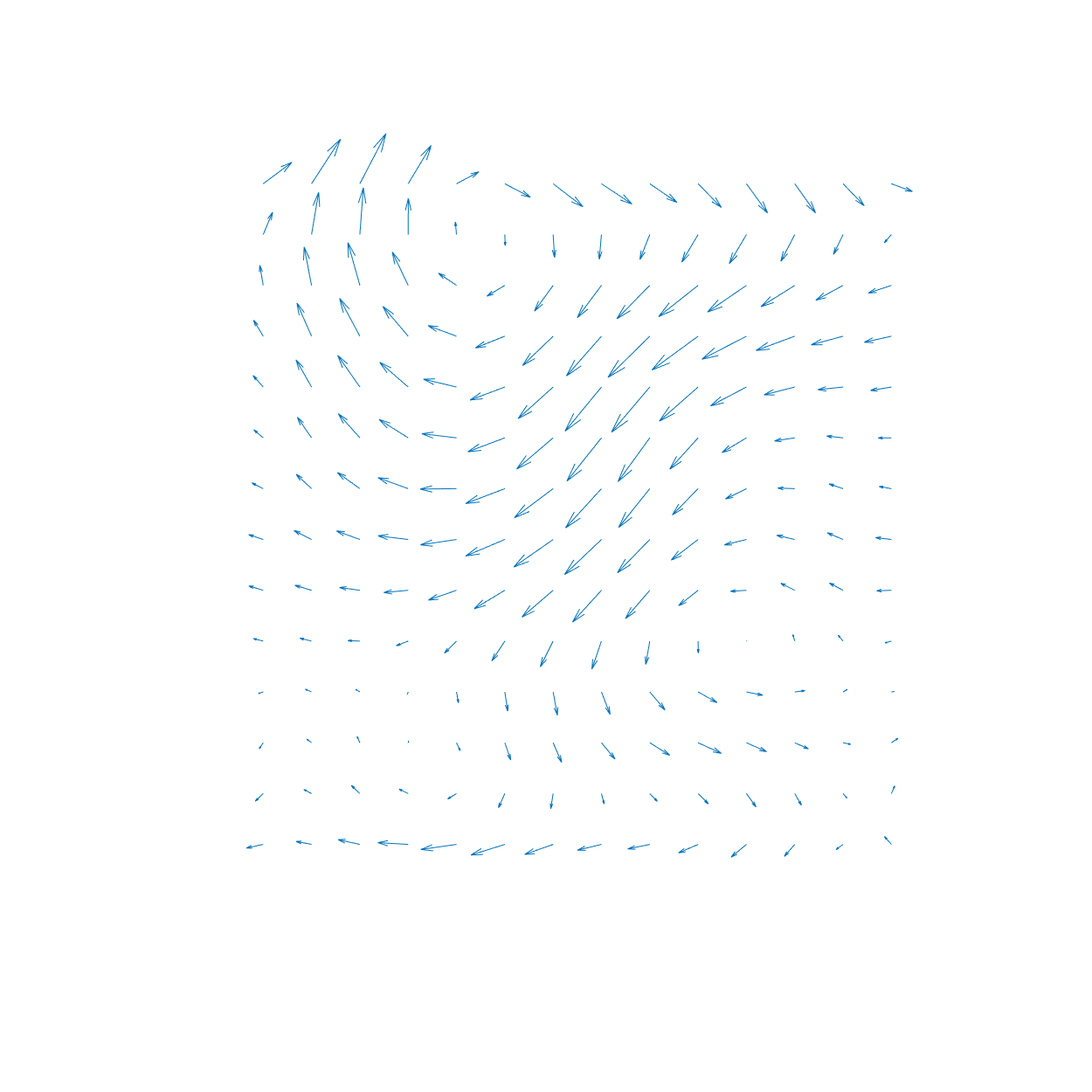

Supplement: S1 MCG raw data 1 — The raw MCG dataset includes categories 0-4 for testing. (ZIP) [file pone.0338189.s001.zip › test/1/p12_495_4.png]

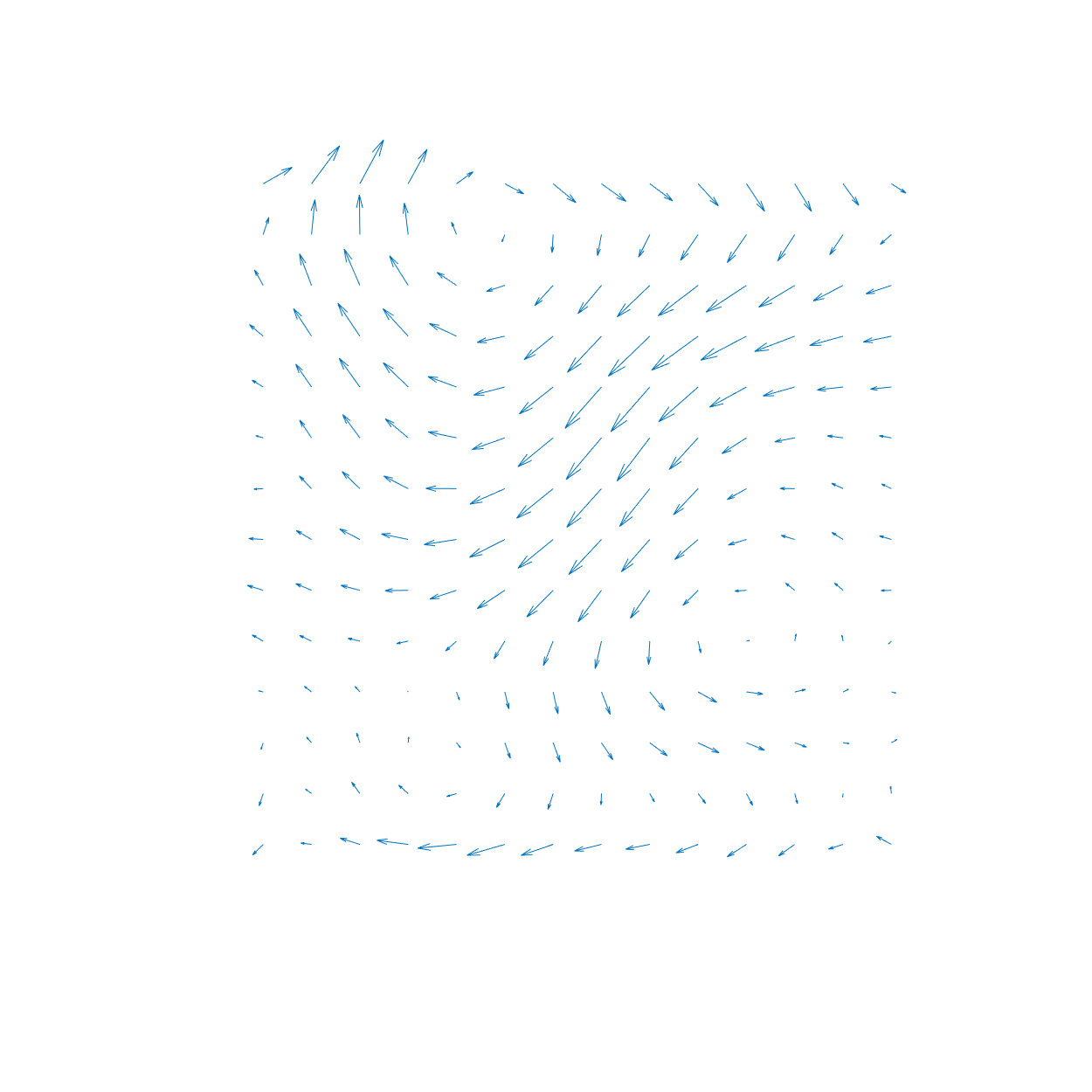

Supplement: S1 MCG raw data 1 — The raw MCG dataset includes categories 0-4 for testing. (ZIP) [file pone.0338189.s001.zip › test/1/p12_500_4.png]

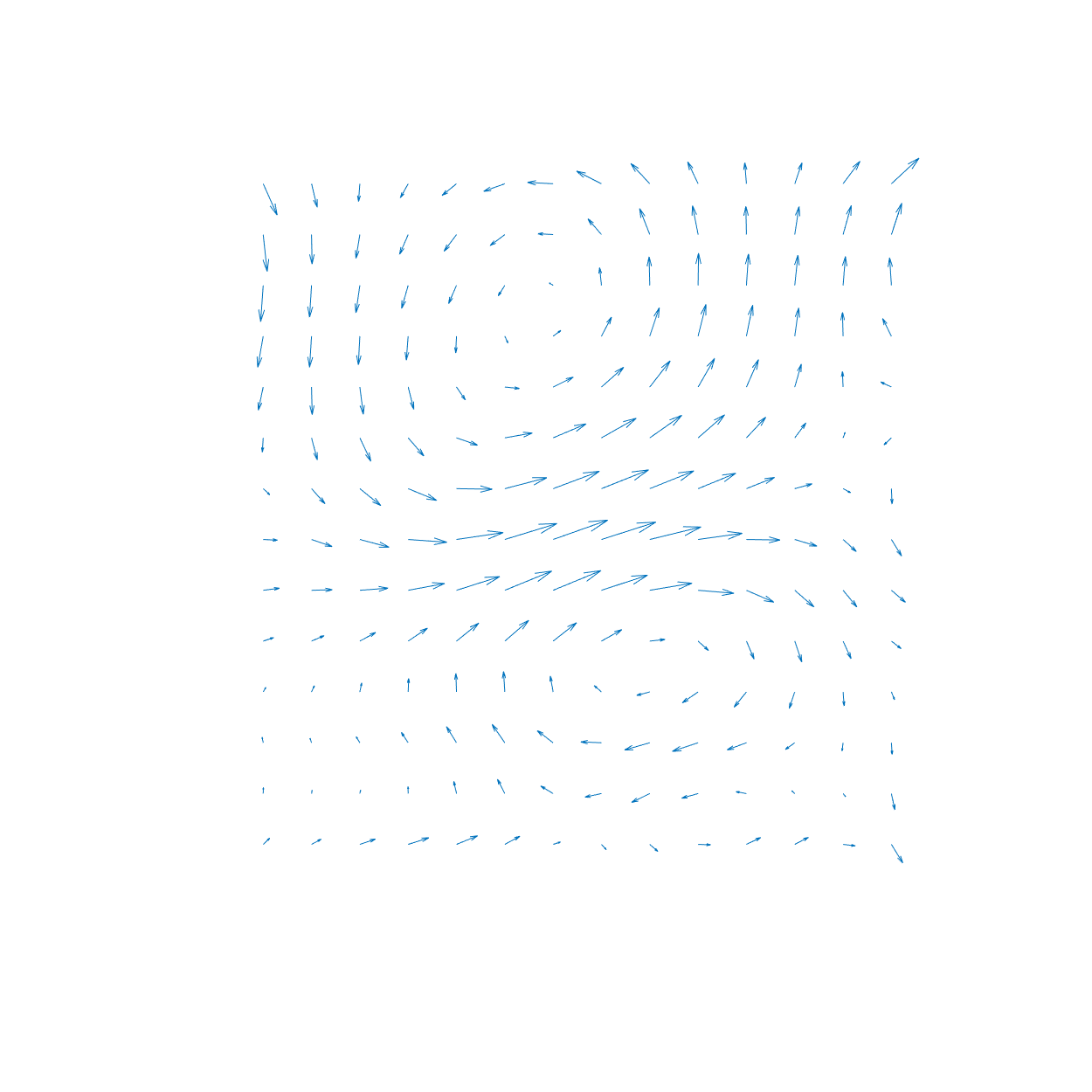

Supplement: S1 MCG raw data 1 — The raw MCG dataset includes categories 0-4 for testing. (ZIP) [file pone.0338189.s001.zip › test/1/p1_430_4.png]

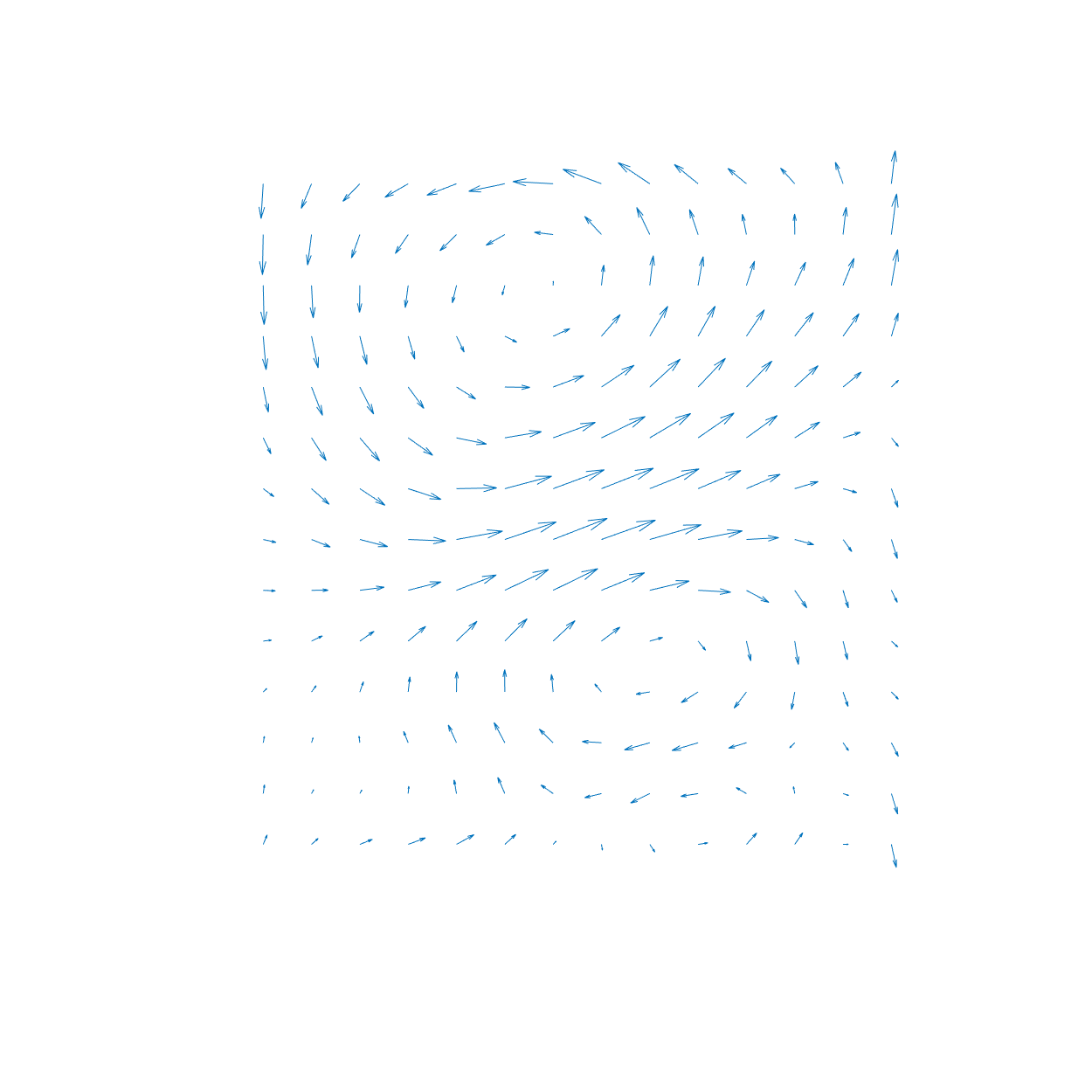

Supplement: S1 MCG raw data 1 — The raw MCG dataset includes categories 0-4 for testing. (ZIP) [file pone.0338189.s001.zip › test/1/p1_435_4.png]

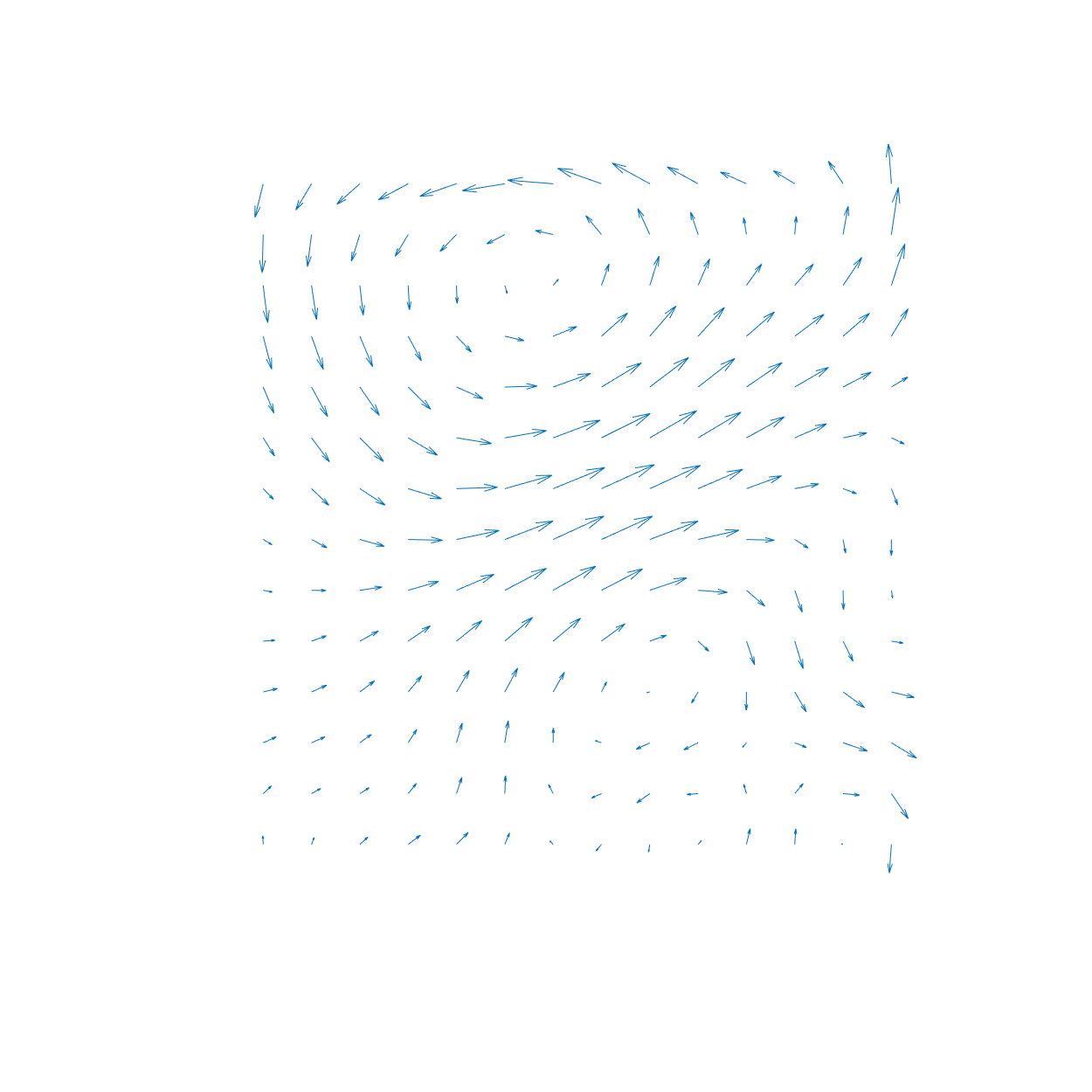

Supplement: S1 MCG raw data 1 — The raw MCG dataset includes categories 0-4 for testing. (ZIP) [file pone.0338189.s001.zip › test/1/p1_440_4.png]

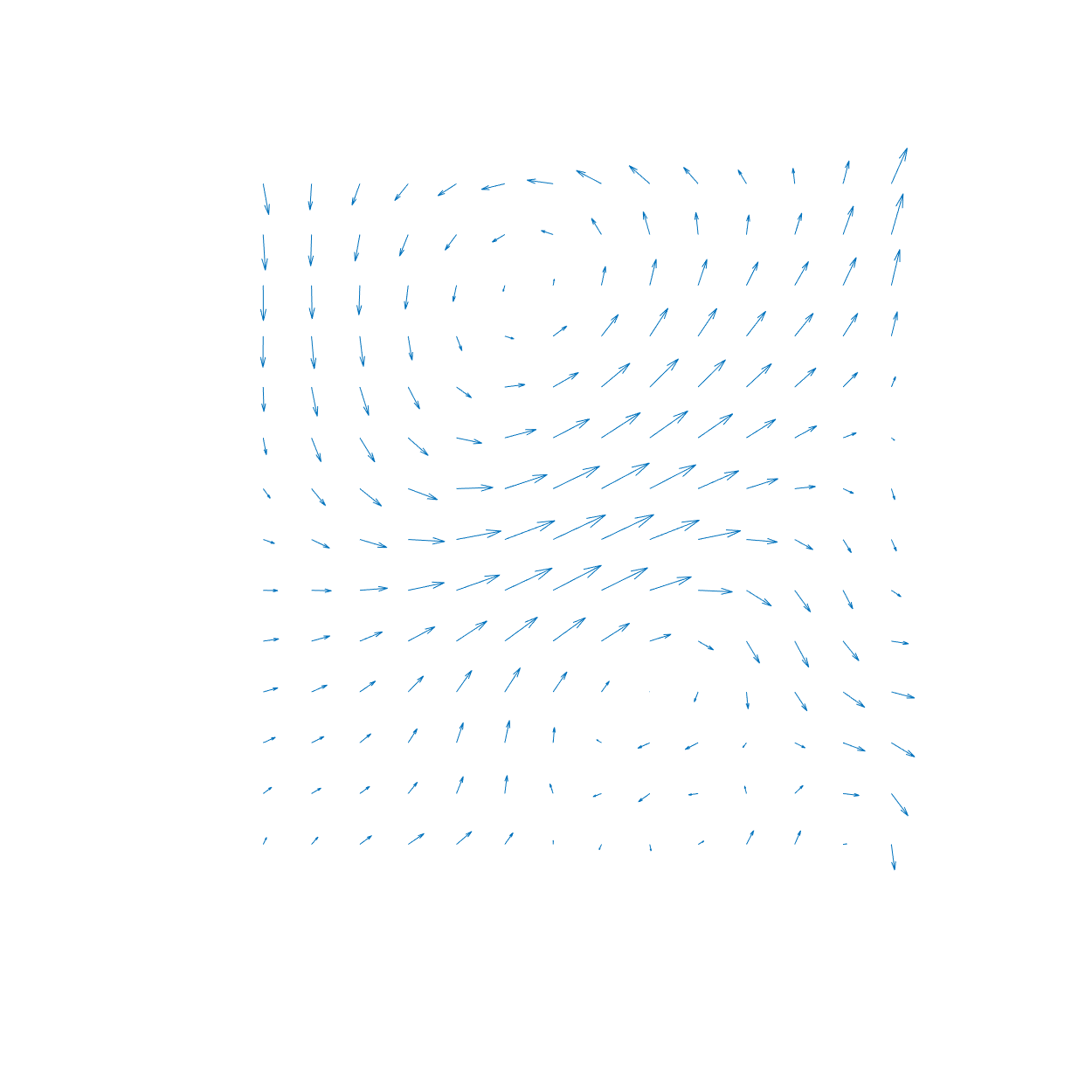

Supplement: S1 MCG raw data 1 — The raw MCG dataset includes categories 0-4 for testing. (ZIP) [file pone.0338189.s001.zip › test/1/p1_445_4.png]

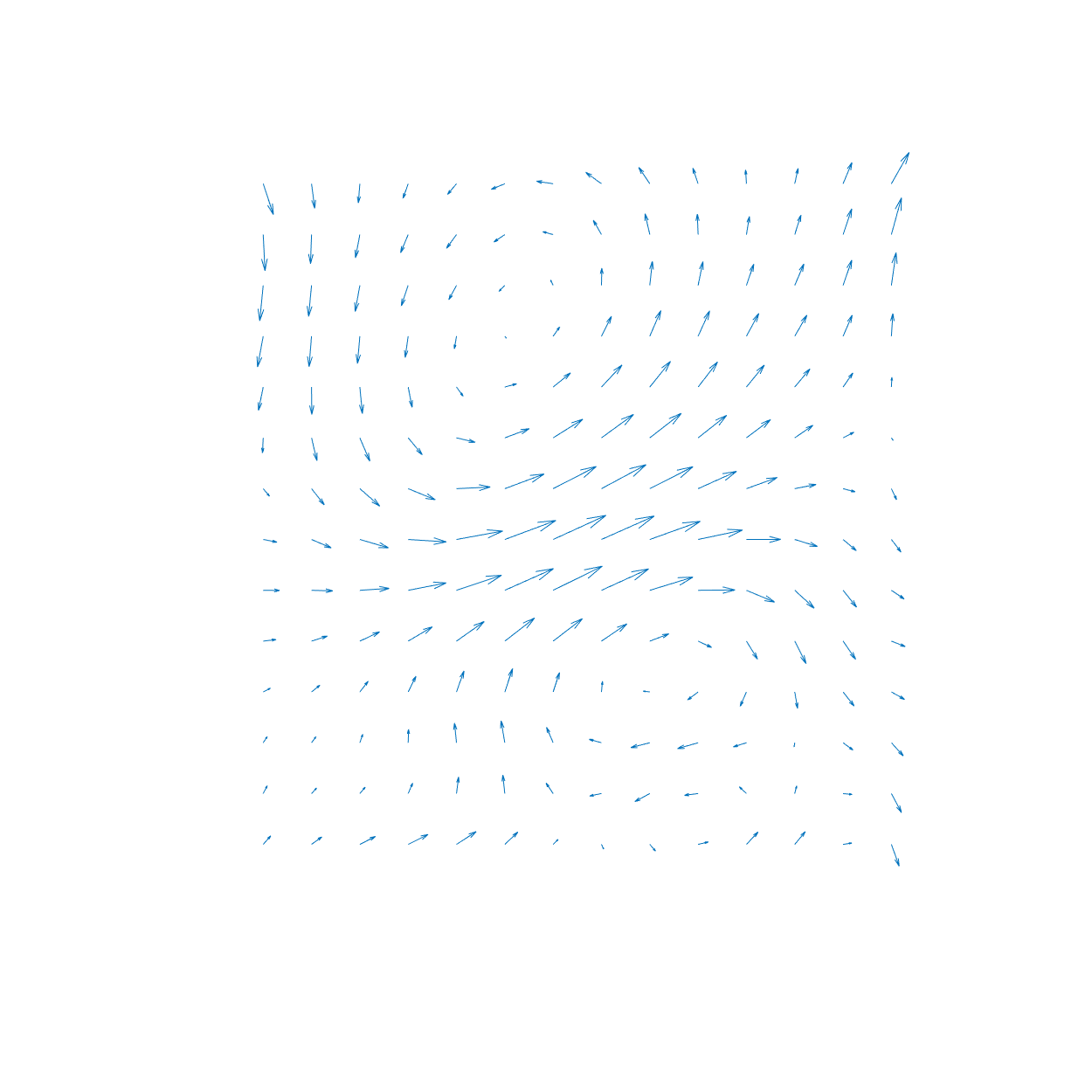

Supplement: S1 MCG raw data 1 — The raw MCG dataset includes categories 0-4 for testing. (ZIP) [file pone.0338189.s001.zip › test/1/p1_450_4.png]

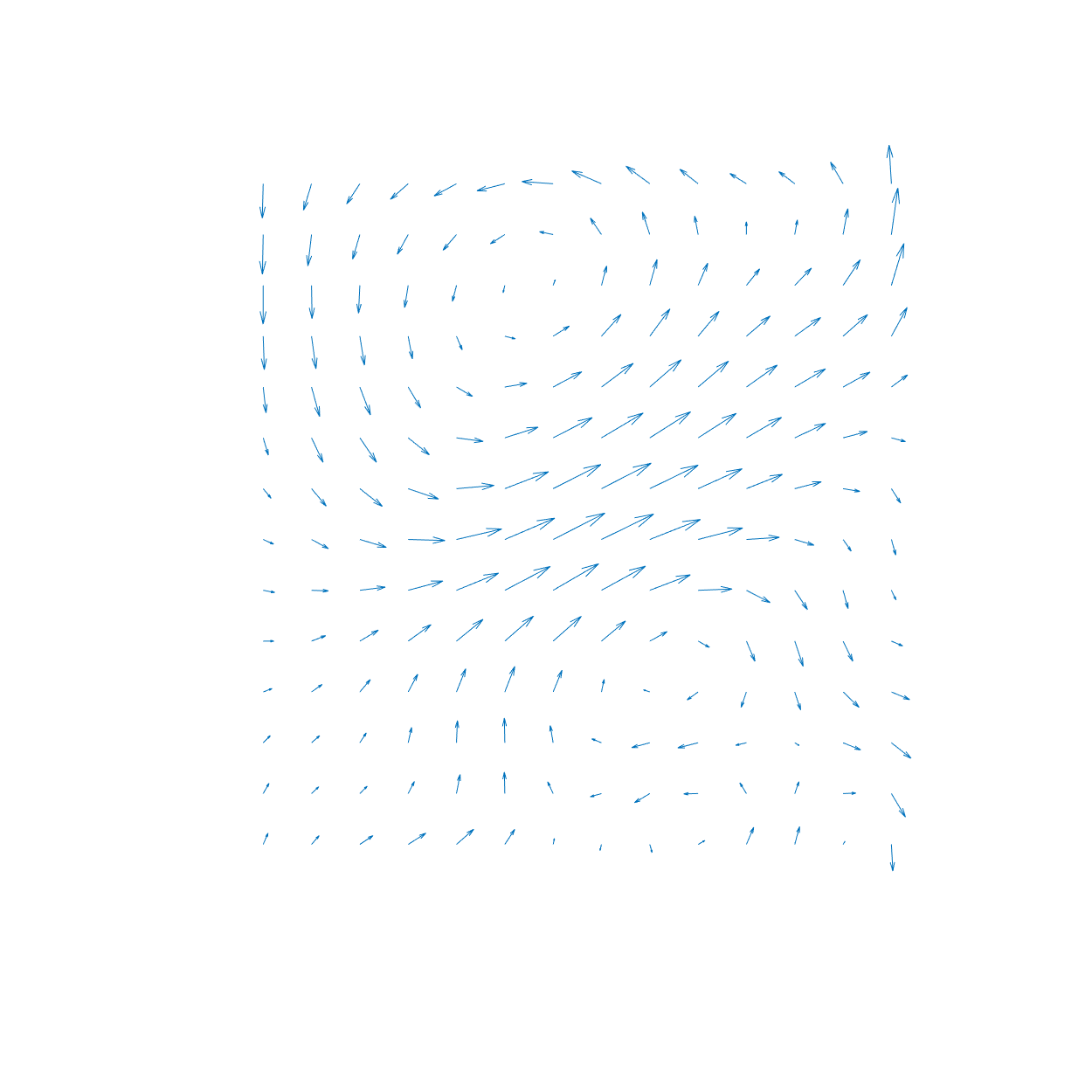

Supplement: S1 MCG raw data 1 — The raw MCG dataset includes categories 0-4 for testing. (ZIP) [file pone.0338189.s001.zip › test/1/p1_455_4.png]

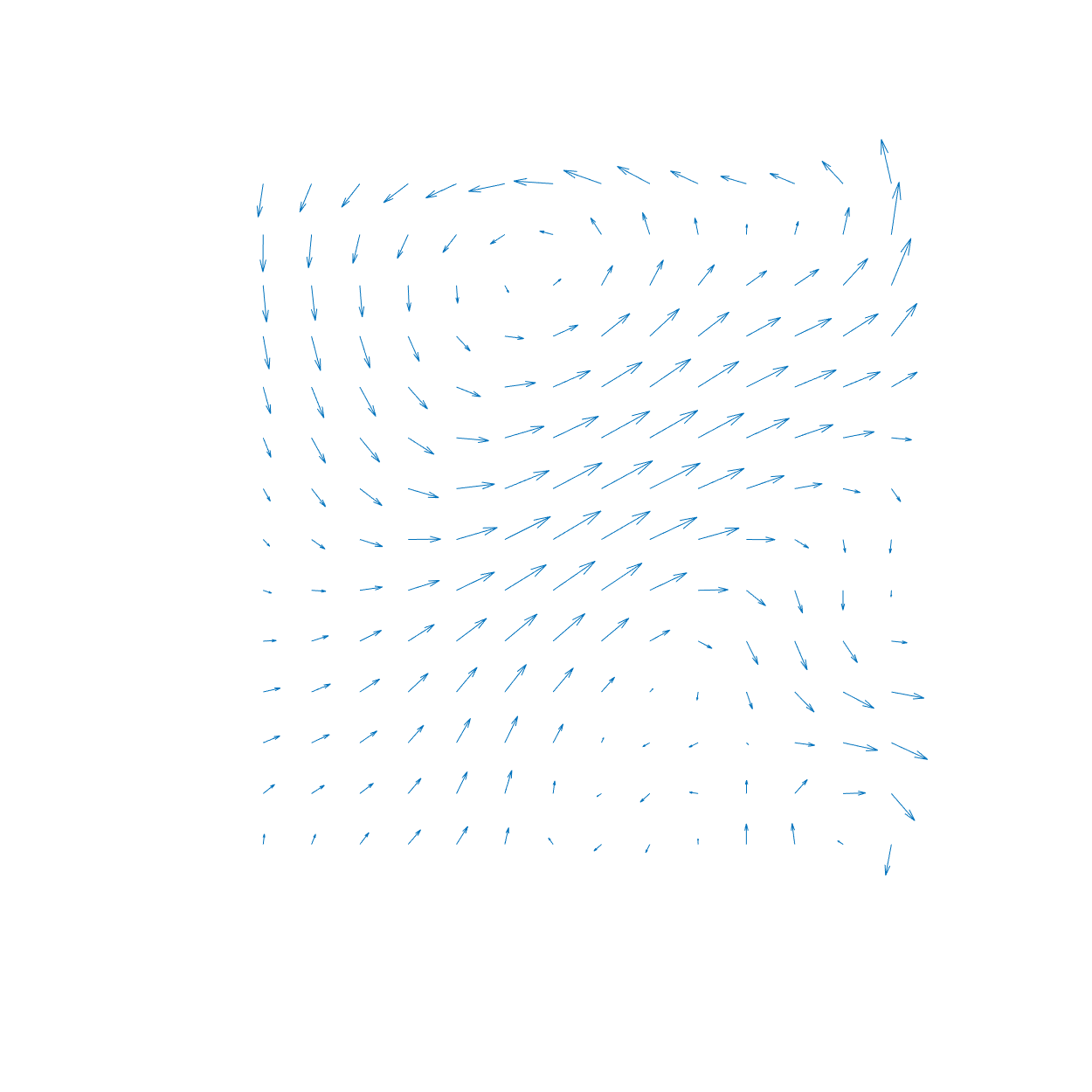

Supplement: S1 MCG raw data 1 — The raw MCG dataset includes categories 0-4 for testing. (ZIP) [file pone.0338189.s001.zip › test/1/p1_460_4.png]

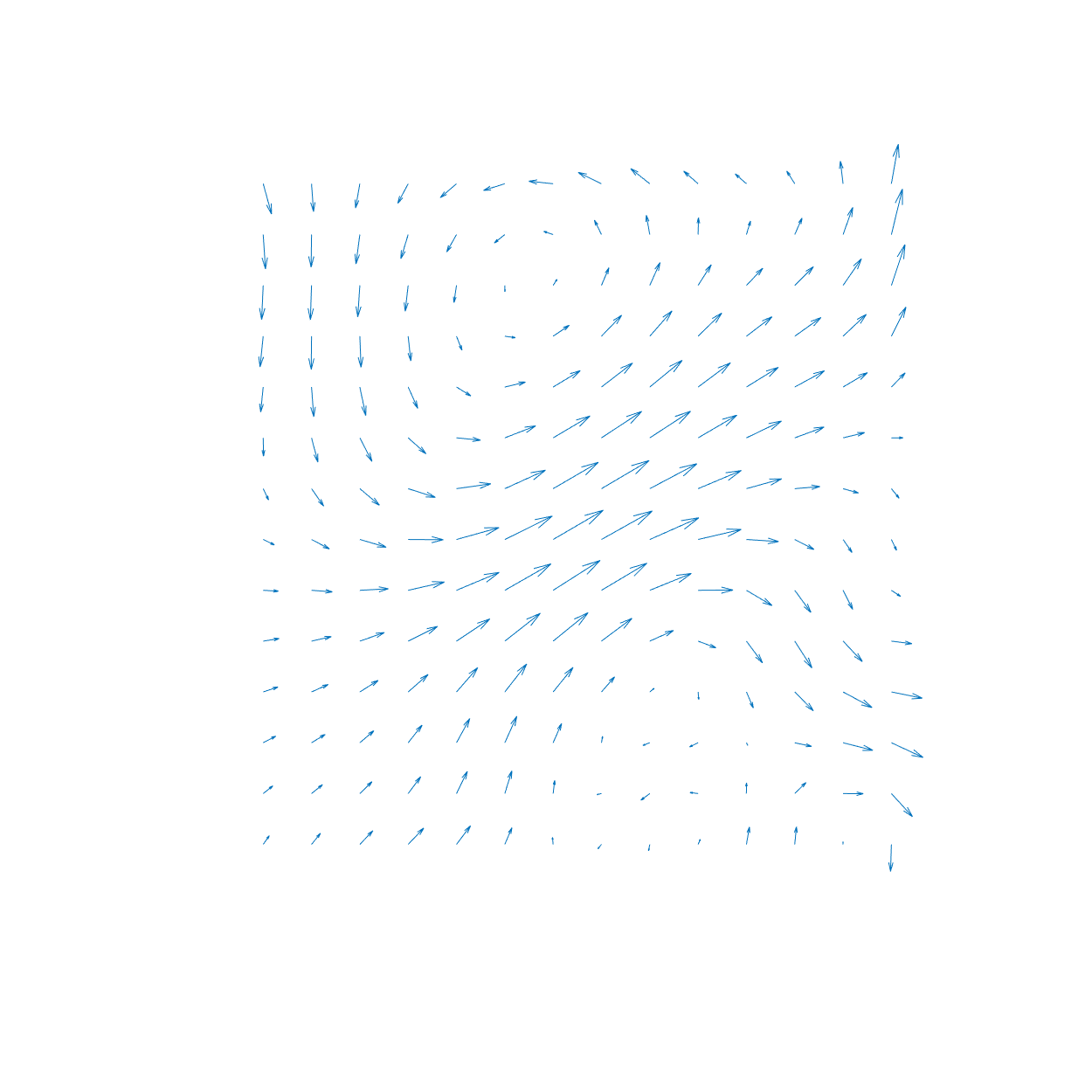

Supplement: S1 MCG raw data 1 — The raw MCG dataset includes categories 0-4 for testing. (ZIP) [file pone.0338189.s001.zip › test/1/p1_465_4.png]

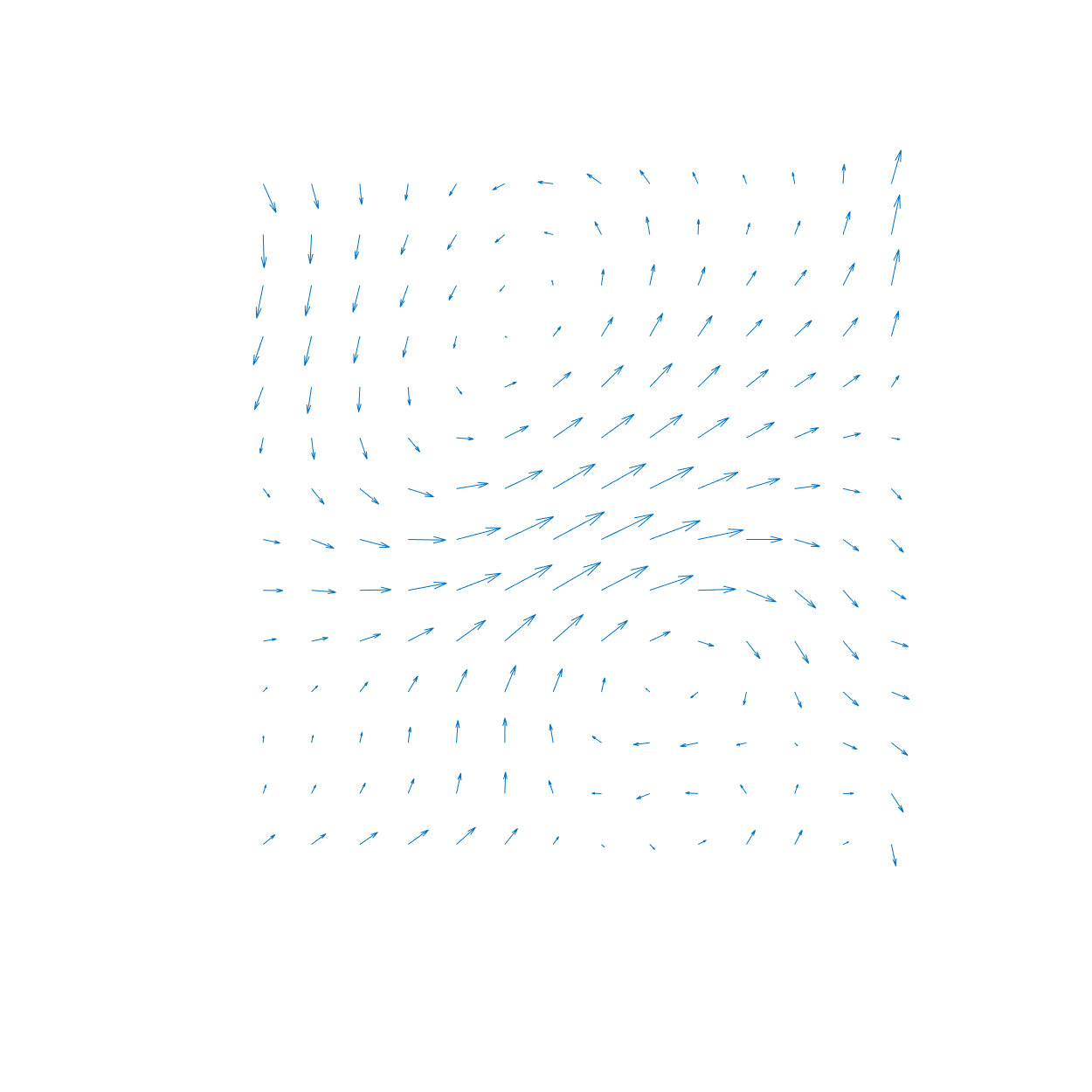

Supplement: S1 MCG raw data 1 — The raw MCG dataset includes categories 0-4 for testing. (ZIP) [file pone.0338189.s001.zip › test/1/p1_470_4.png]

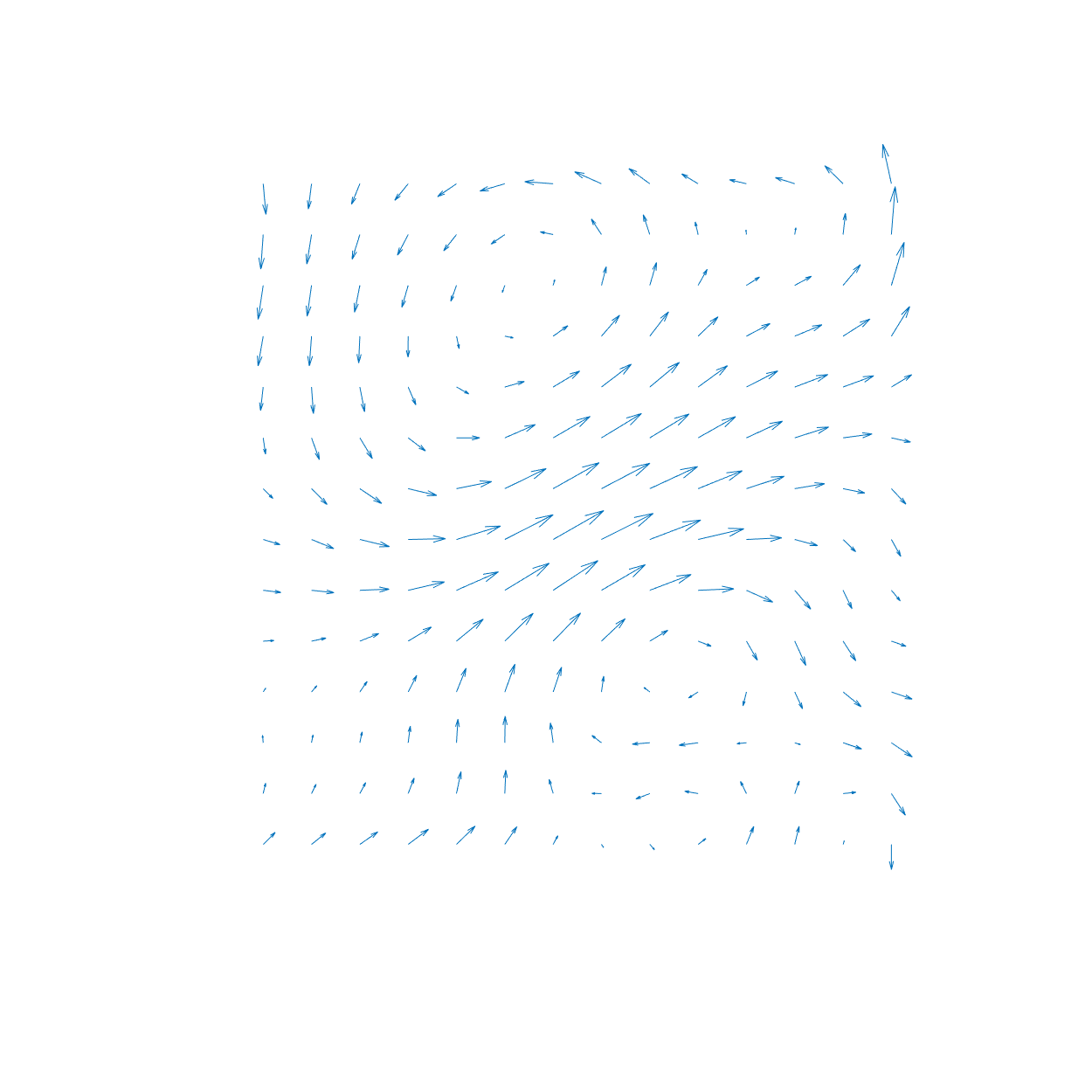

Supplement: S1 MCG raw data 1 — The raw MCG dataset includes categories 0-4 for testing. (ZIP) [file pone.0338189.s001.zip › test/1/p1_475_4.png]

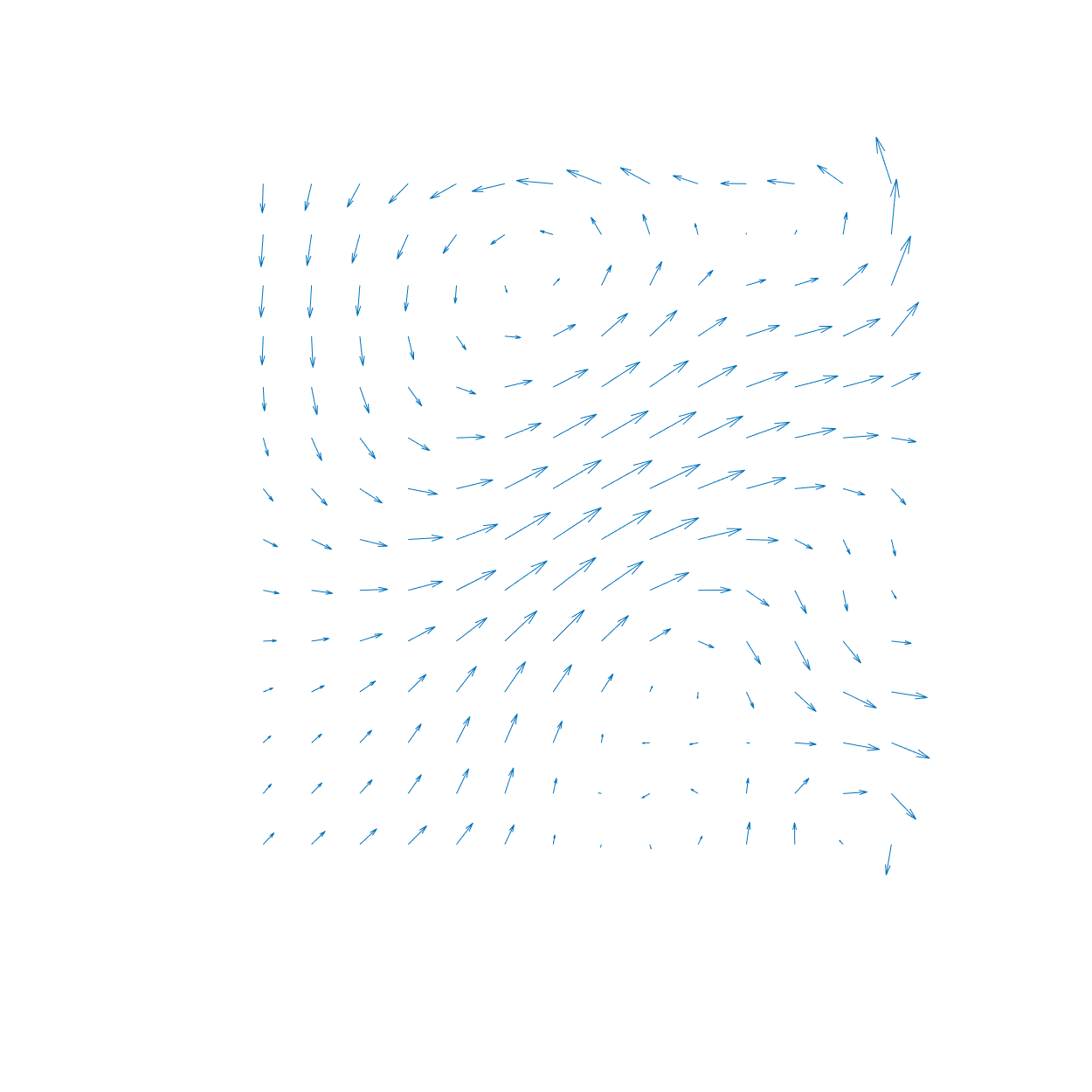

Supplement: S1 MCG raw data 1 — The raw MCG dataset includes categories 0-4 for testing. (ZIP) [file pone.0338189.s001.zip › test/1/p1_480_4.png]

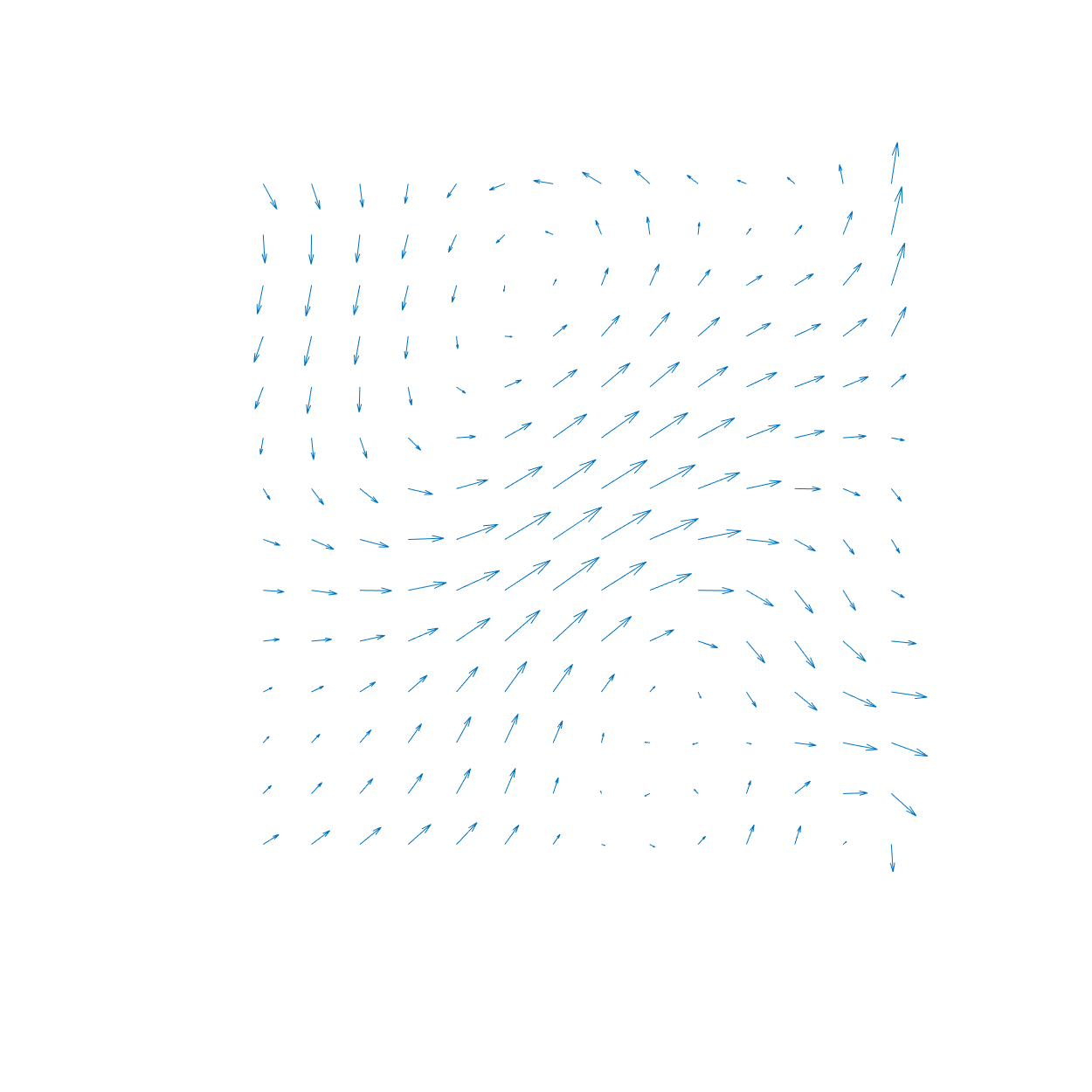

Supplement: S1 MCG raw data 1 — The raw MCG dataset includes categories 0-4 for testing. (ZIP) [file pone.0338189.s001.zip › test/1/p1_485_4.png]

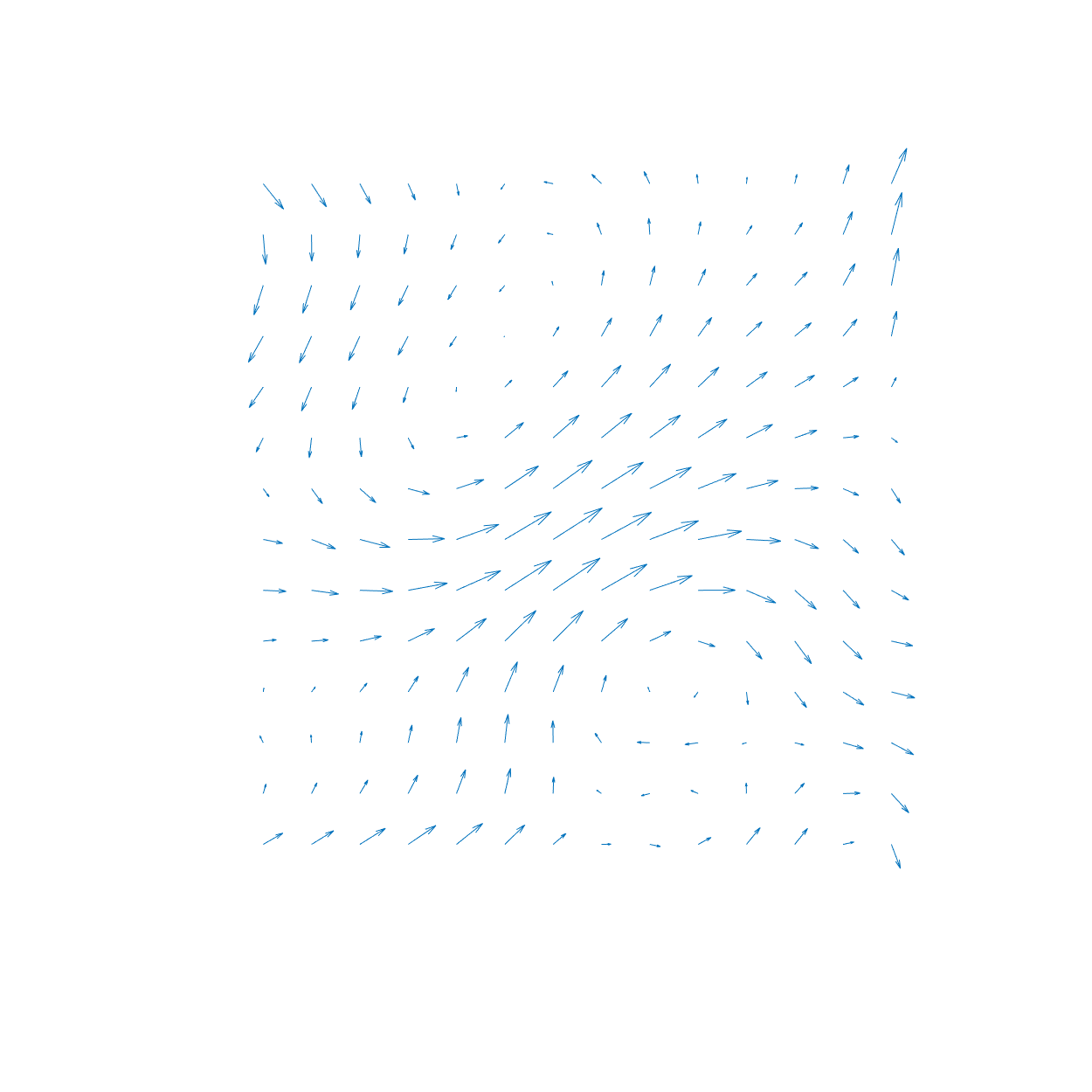

Supplement: S1 MCG raw data 1 — The raw MCG dataset includes categories 0-4 for testing. (ZIP) [file pone.0338189.s001.zip › test/1/p1_490_4.png]

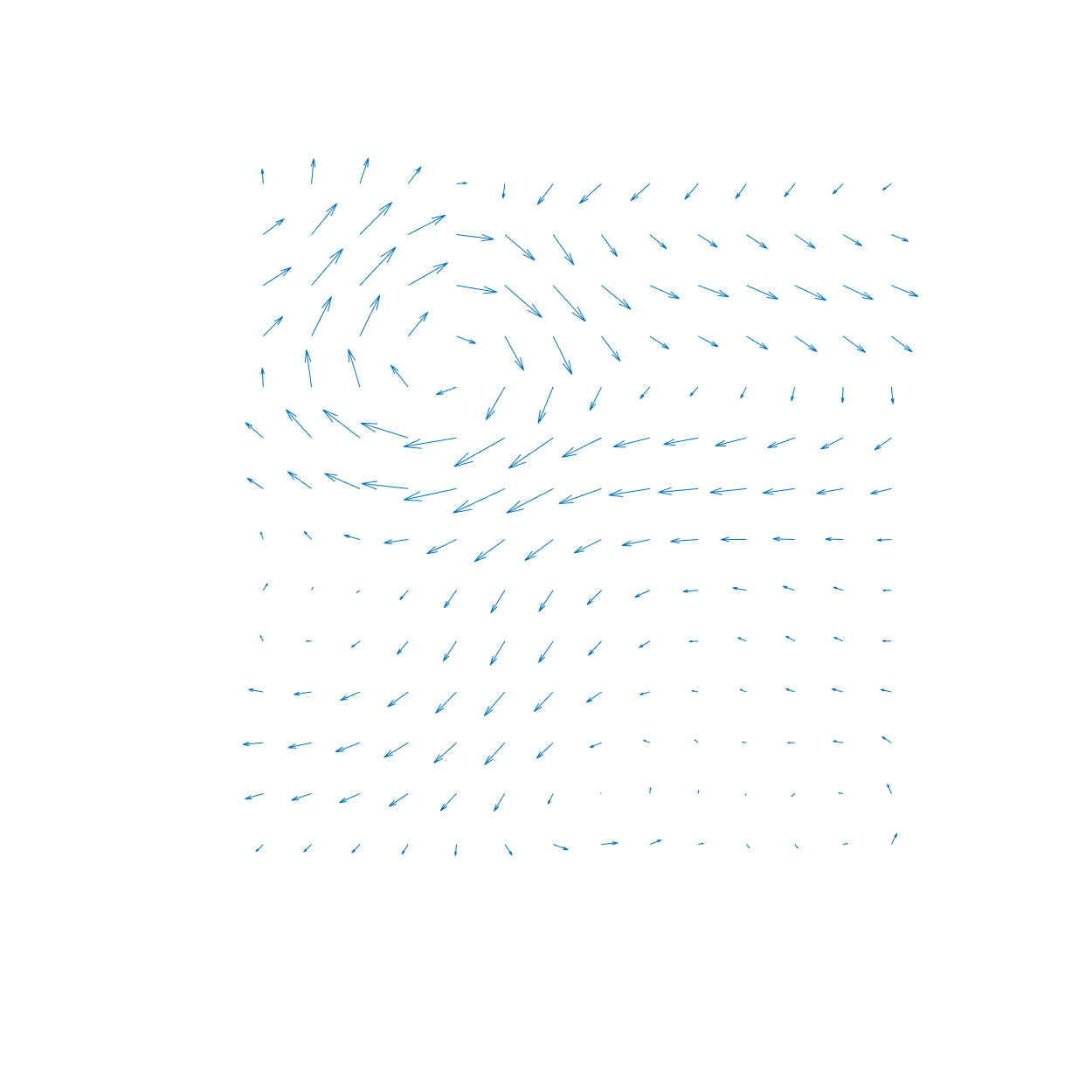

Supplement: S1 MCG raw data 1 — The raw MCG dataset includes categories 0-4 for testing. (ZIP) [file pone.0338189.s001.zip › test/1/p2_470_4.png]

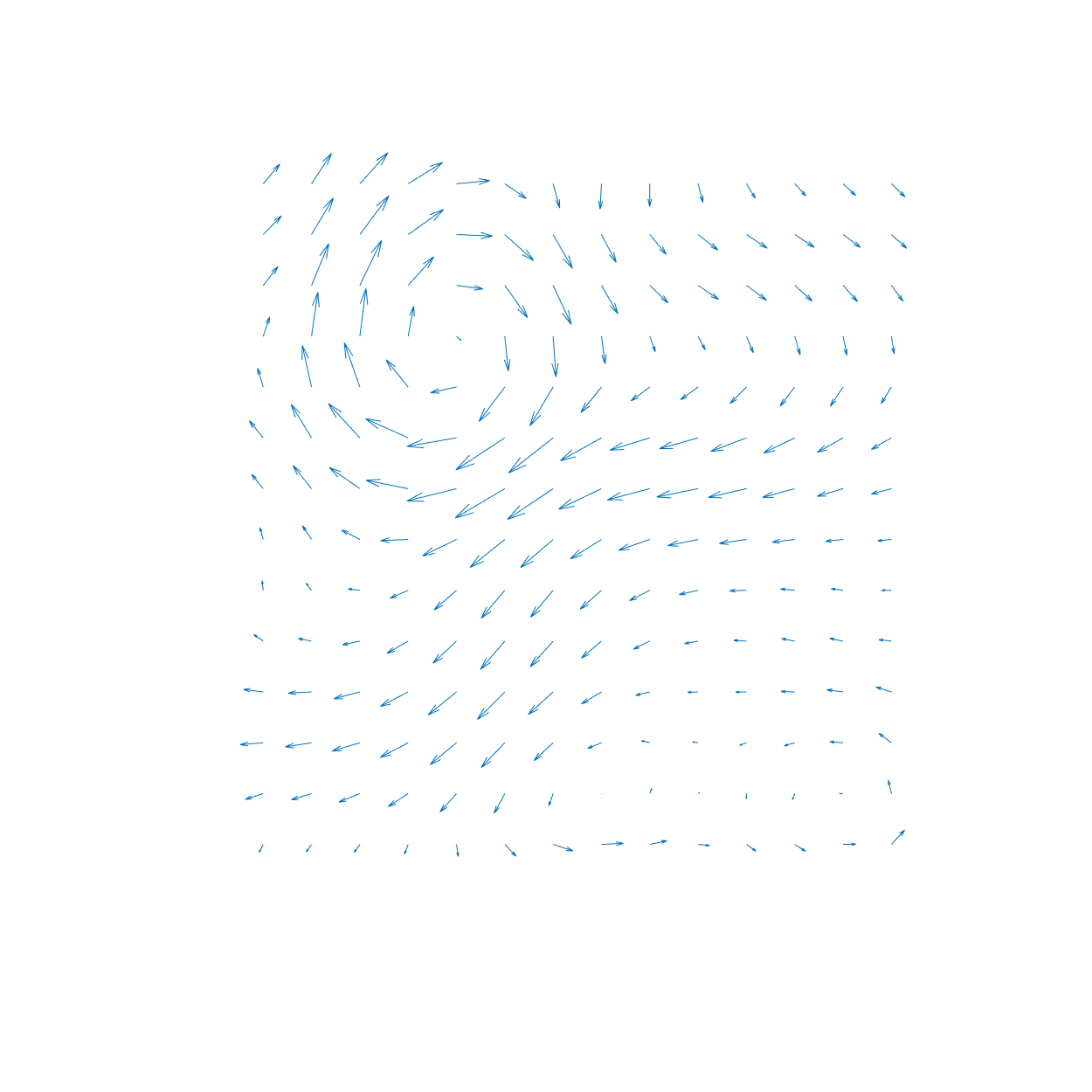

Supplement: S1 MCG raw data 1 — The raw MCG dataset includes categories 0-4 for testing. (ZIP) [file pone.0338189.s001.zip › test/1/p2_475_4.png]

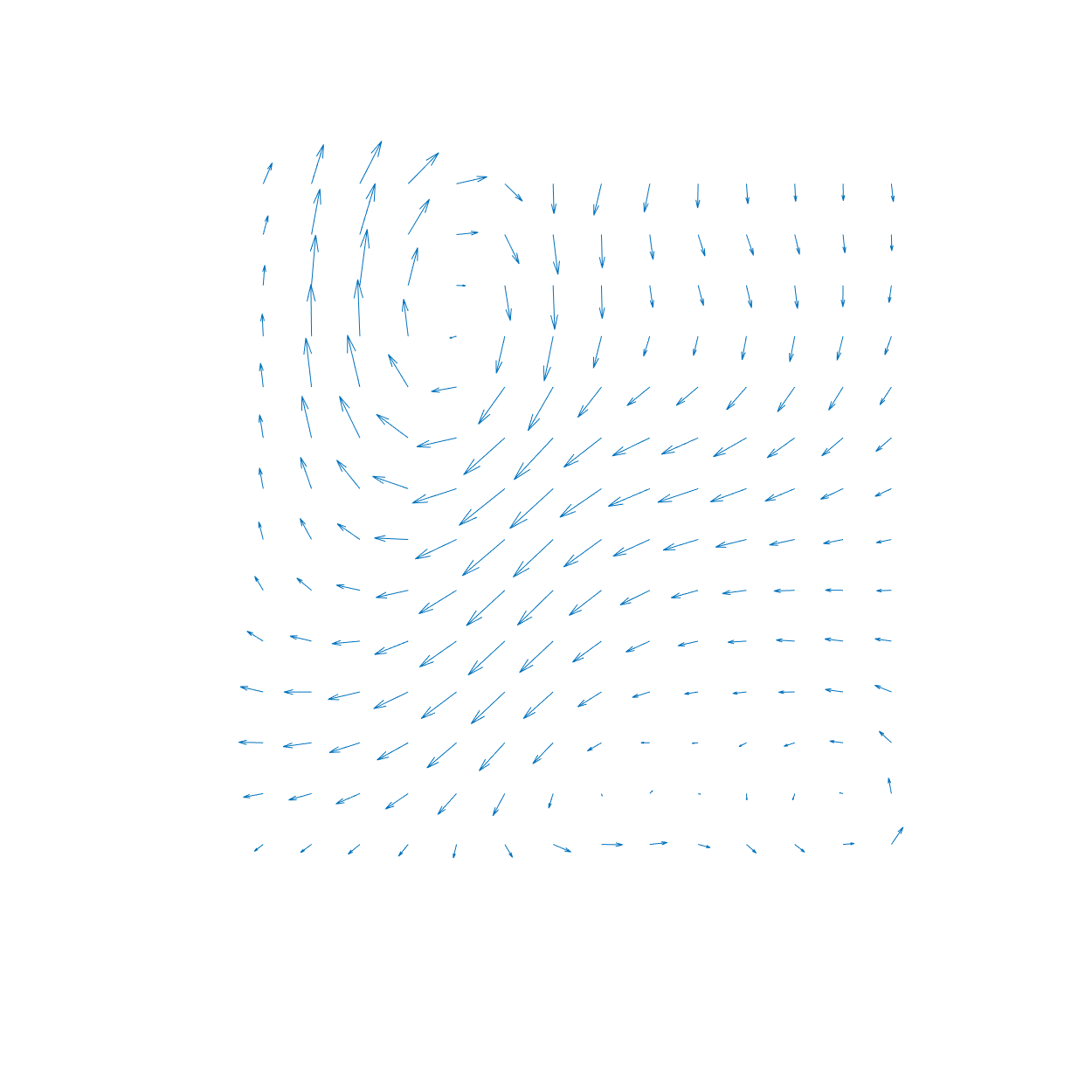

Supplement: S1 MCG raw data 1 — The raw MCG dataset includes categories 0-4 for testing. (ZIP) [file pone.0338189.s001.zip › test/1/p2_480_4.png]

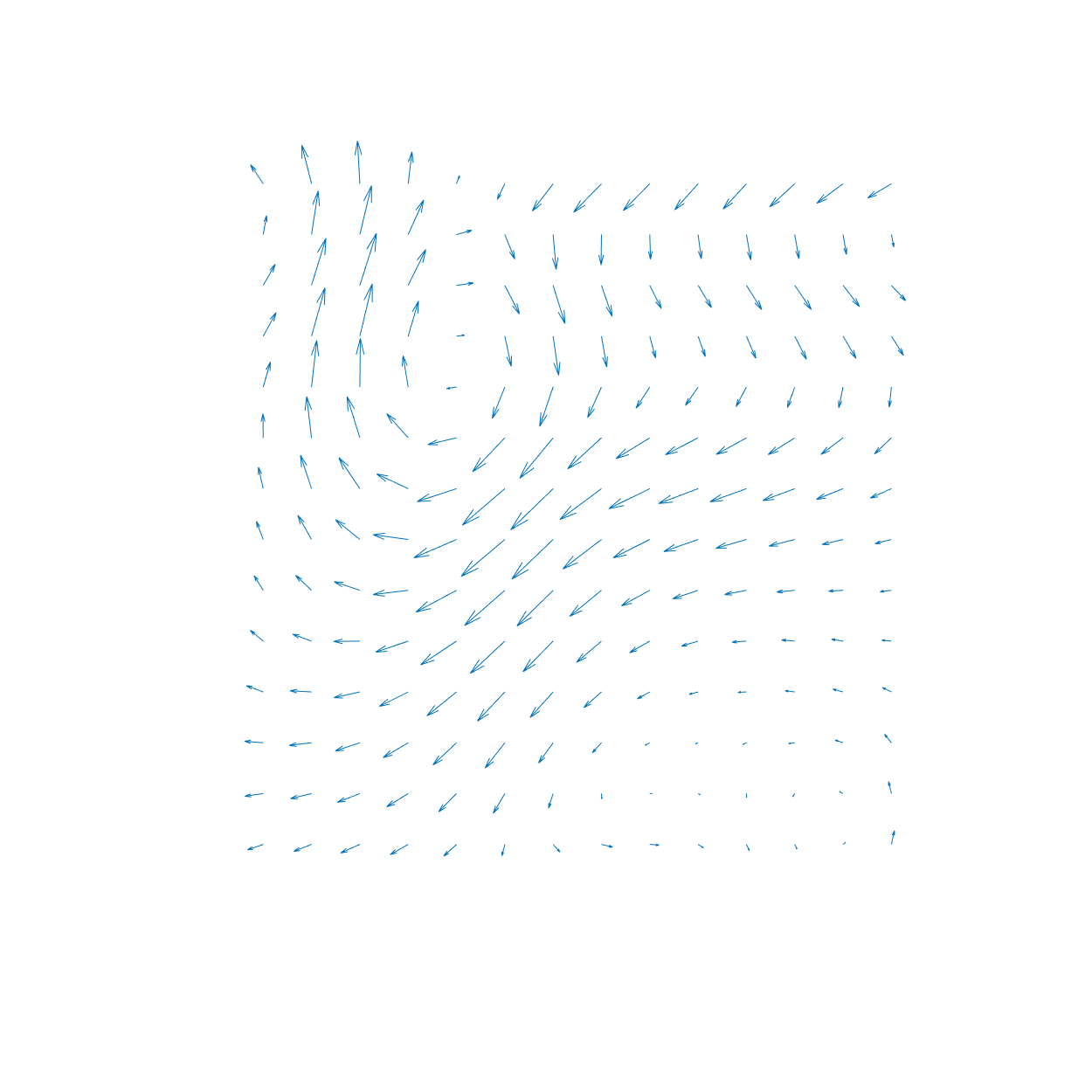

Supplement: S1 MCG raw data 1 — The raw MCG dataset includes categories 0-4 for testing. (ZIP) [file pone.0338189.s001.zip › test/1/p2_485_4.png]

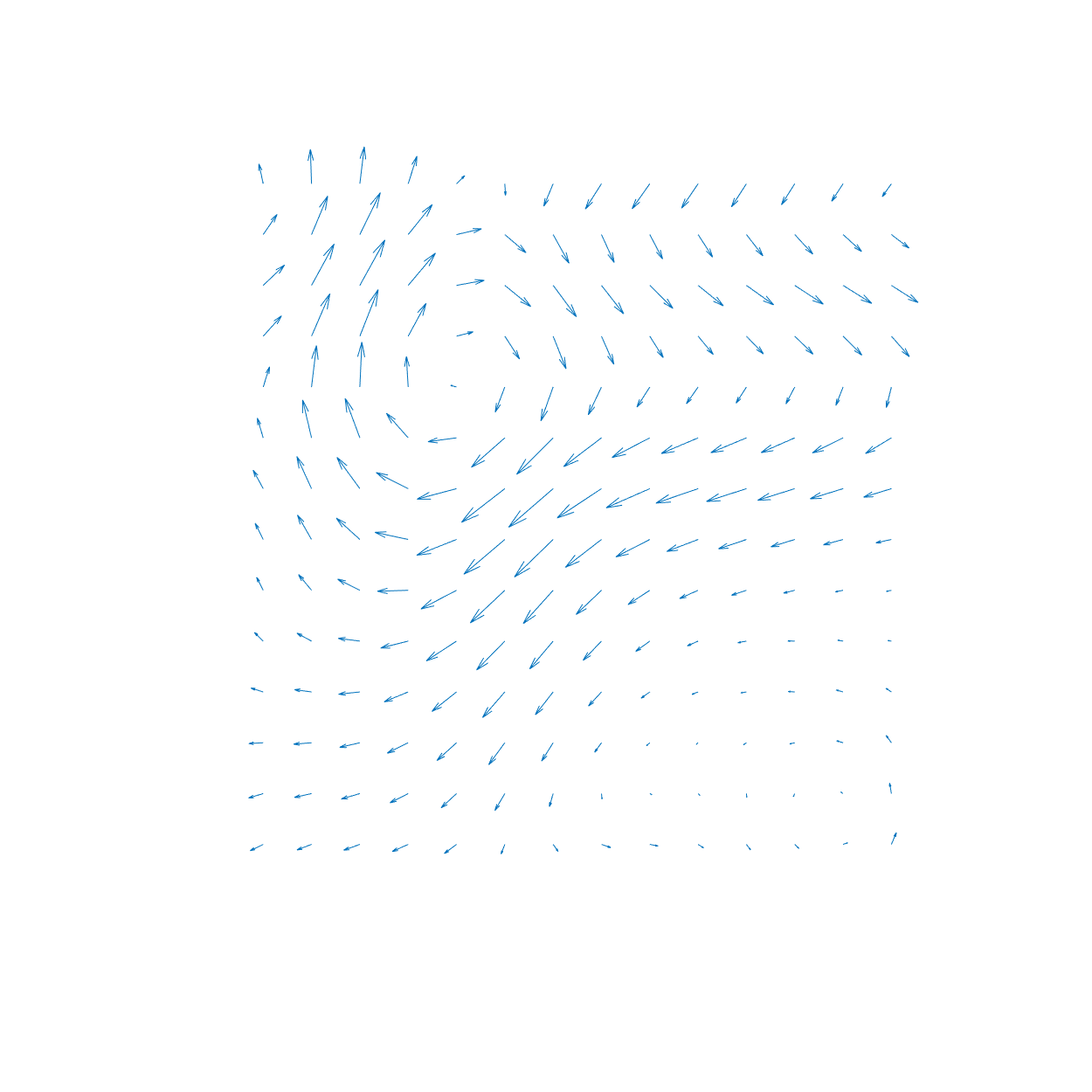

Supplement: S1 MCG raw data 1 — The raw MCG dataset includes categories 0-4 for testing. (ZIP) [file pone.0338189.s001.zip › test/1/p2_490_4.png]

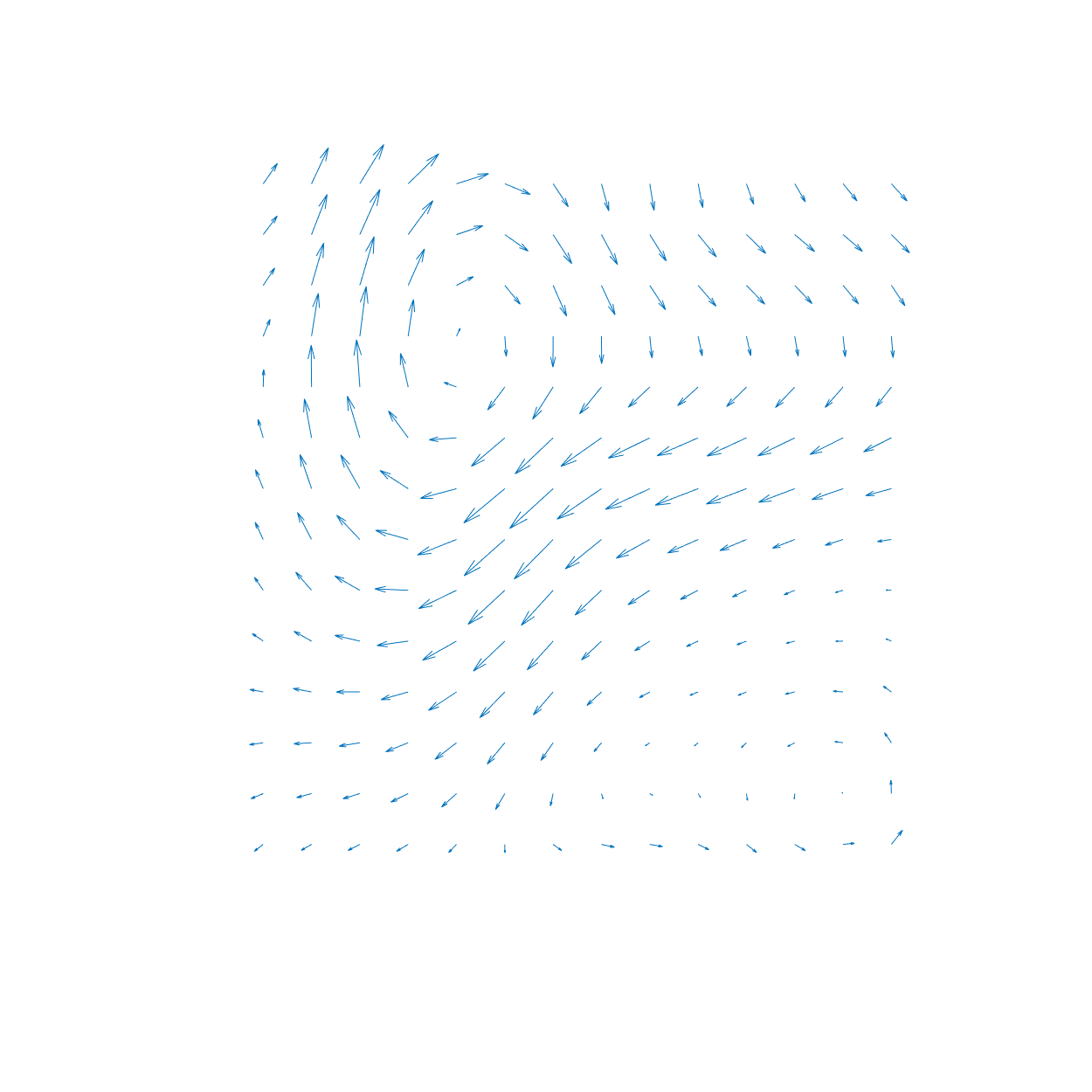

Supplement: S1 MCG raw data 1 — The raw MCG dataset includes categories 0-4 for testing. (ZIP) [file pone.0338189.s001.zip › test/1/p2_495_4.png]
