# Supplementary figures and images for: Classification of current density vector map using transformer hybrid residual network (part 4 of 6)
Source: PLoS One. 2025 Dec 16;20(12):e0338189. doi: 10.1371/journal.pone.0338189 (PMC12707687; doi:10.1371/journal.pone.0338189)

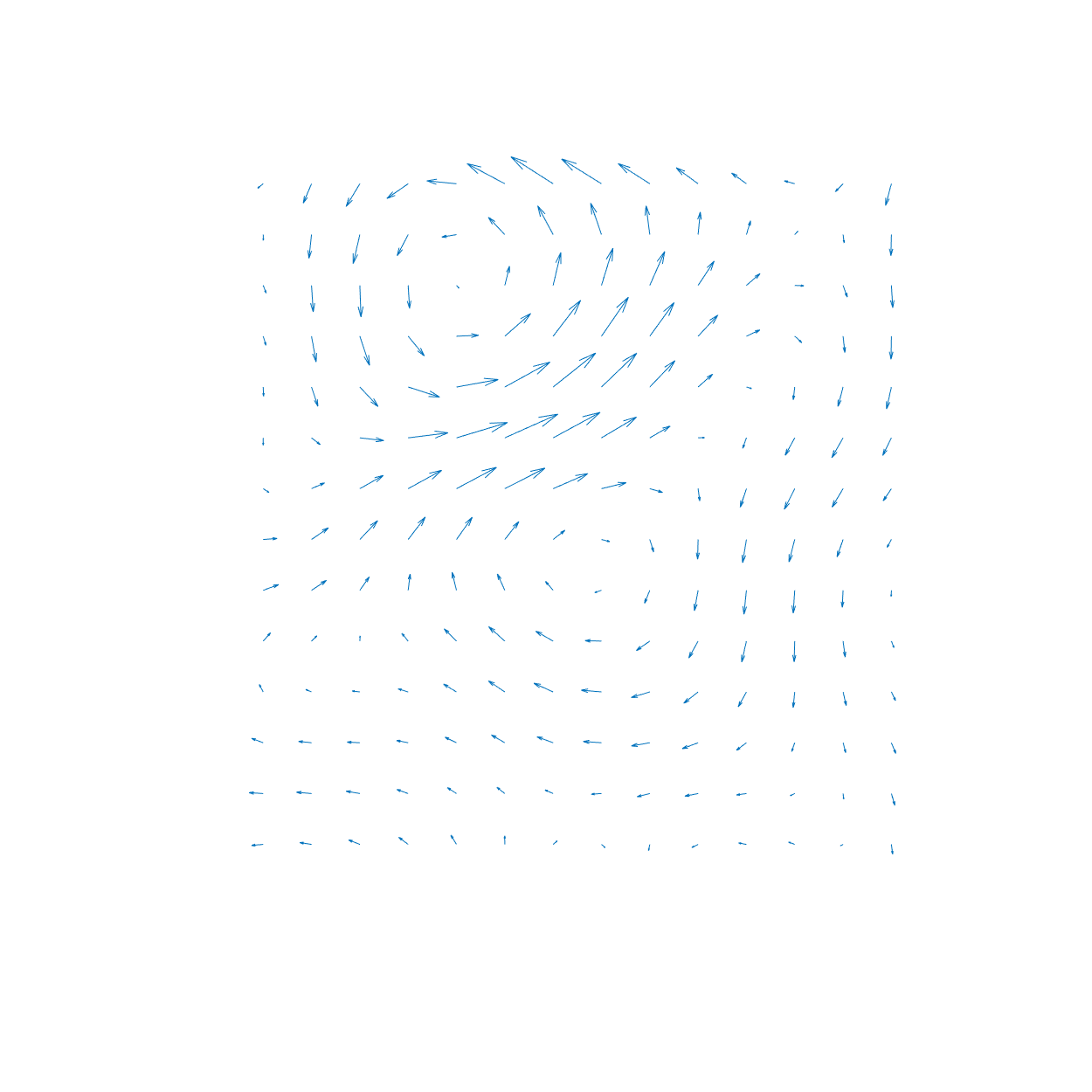

Supplement: S2 MCG raw data 2 — The raw MCG dataset includes categories 0-3 for training and validation. (ZIP) [file pone.0338189.s002.zip › train/0/p4_330_2.png]

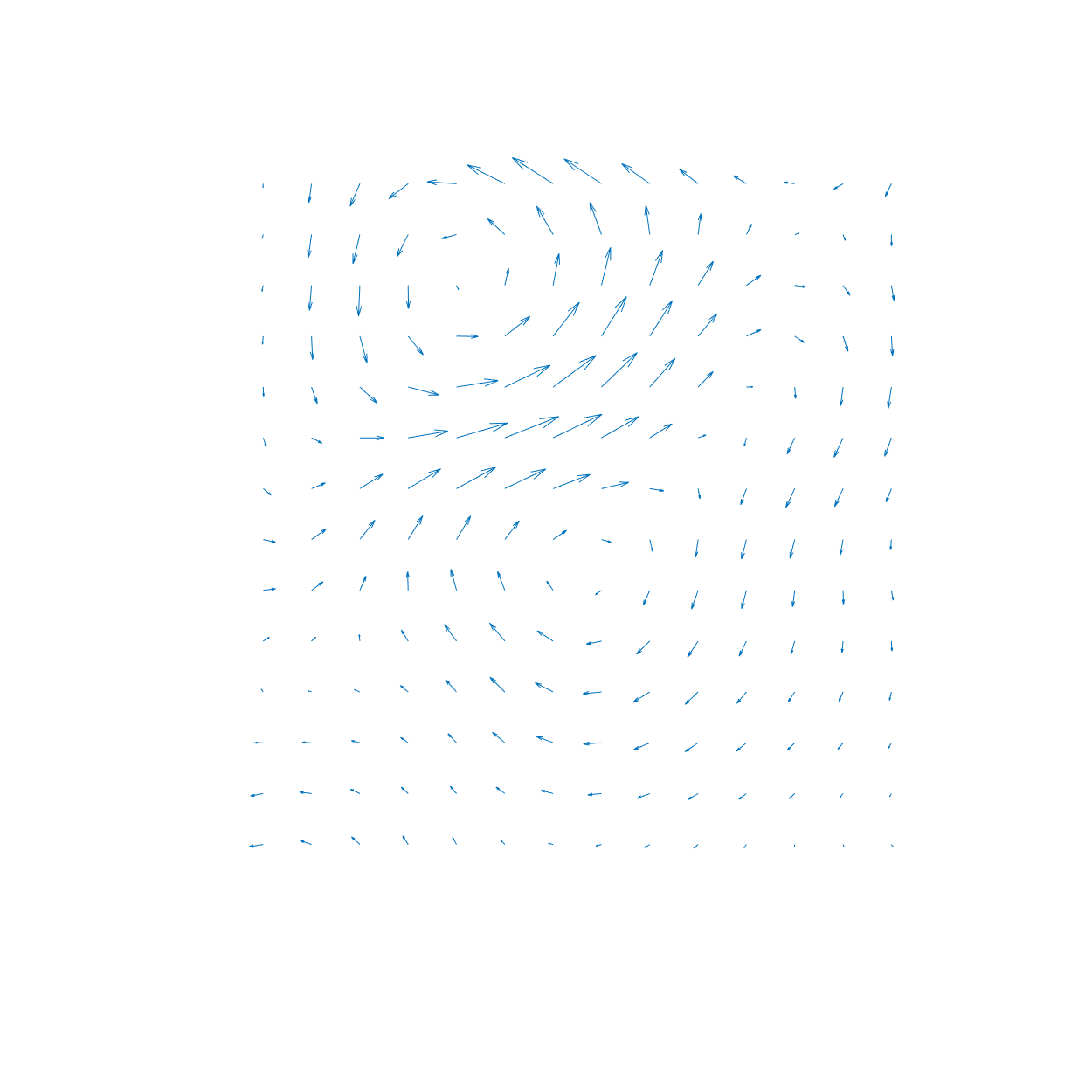

Supplement: S2 MCG raw data 2 — The raw MCG dataset includes categories 0-3 for training and validation. (ZIP) [file pone.0338189.s002.zip › train/0/p4_330_3.png]

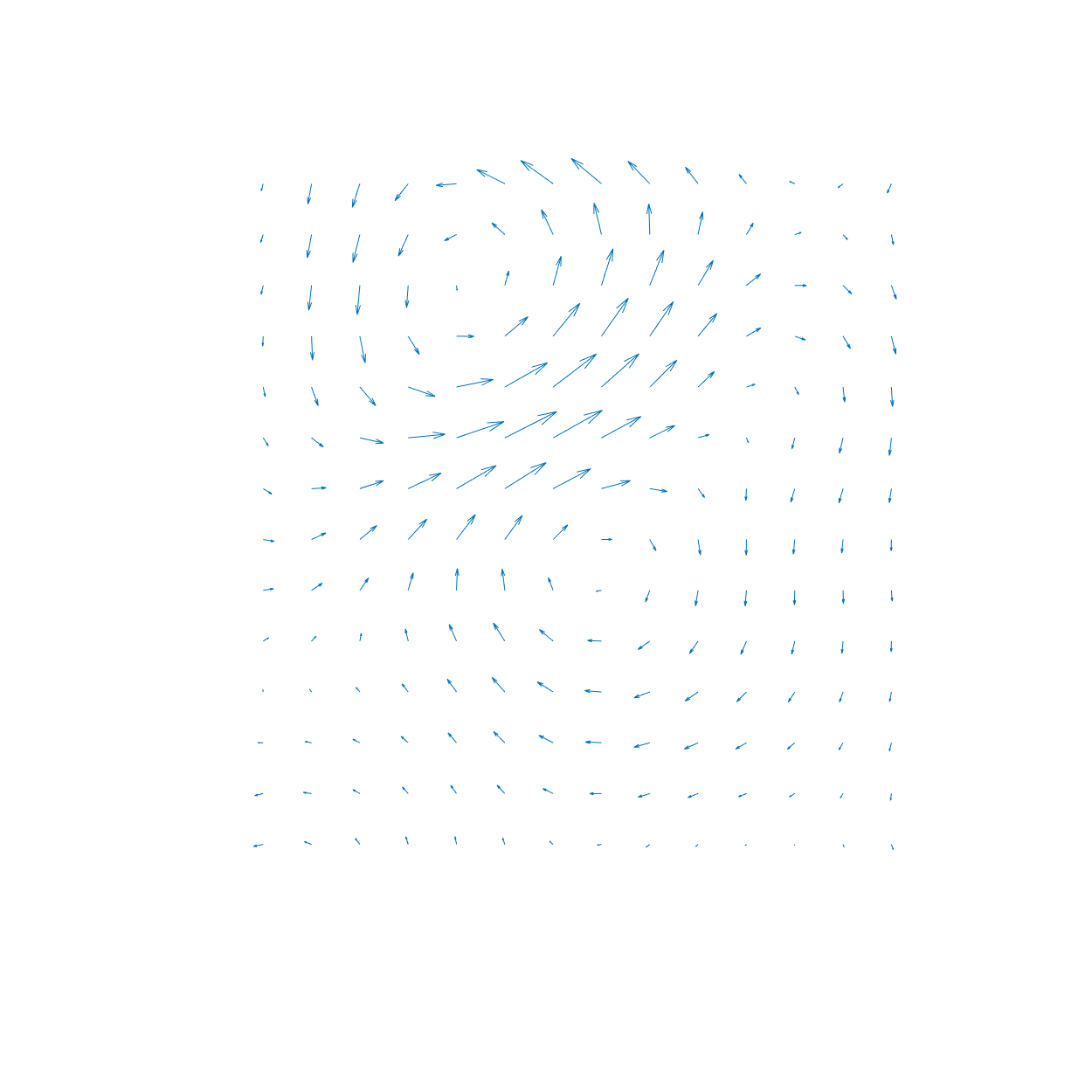

Supplement: S2 MCG raw data 2 — The raw MCG dataset includes categories 0-3 for training and validation. (ZIP) [file pone.0338189.s002.zip › train/0/p4_335_1.png]

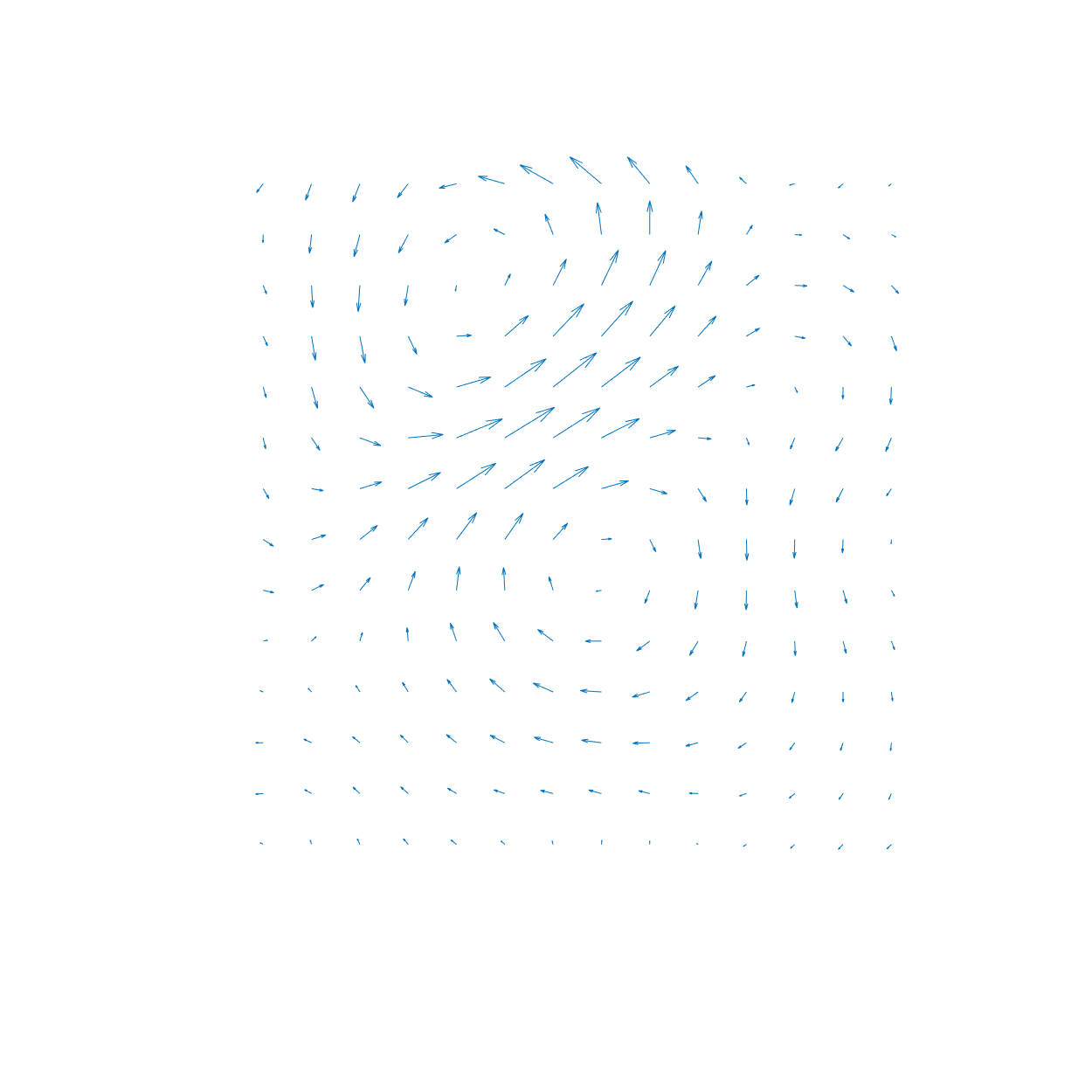

Supplement: S2 MCG raw data 2 — The raw MCG dataset includes categories 0-3 for training and validation. (ZIP) [file pone.0338189.s002.zip › train/0/p4_335_2.png]

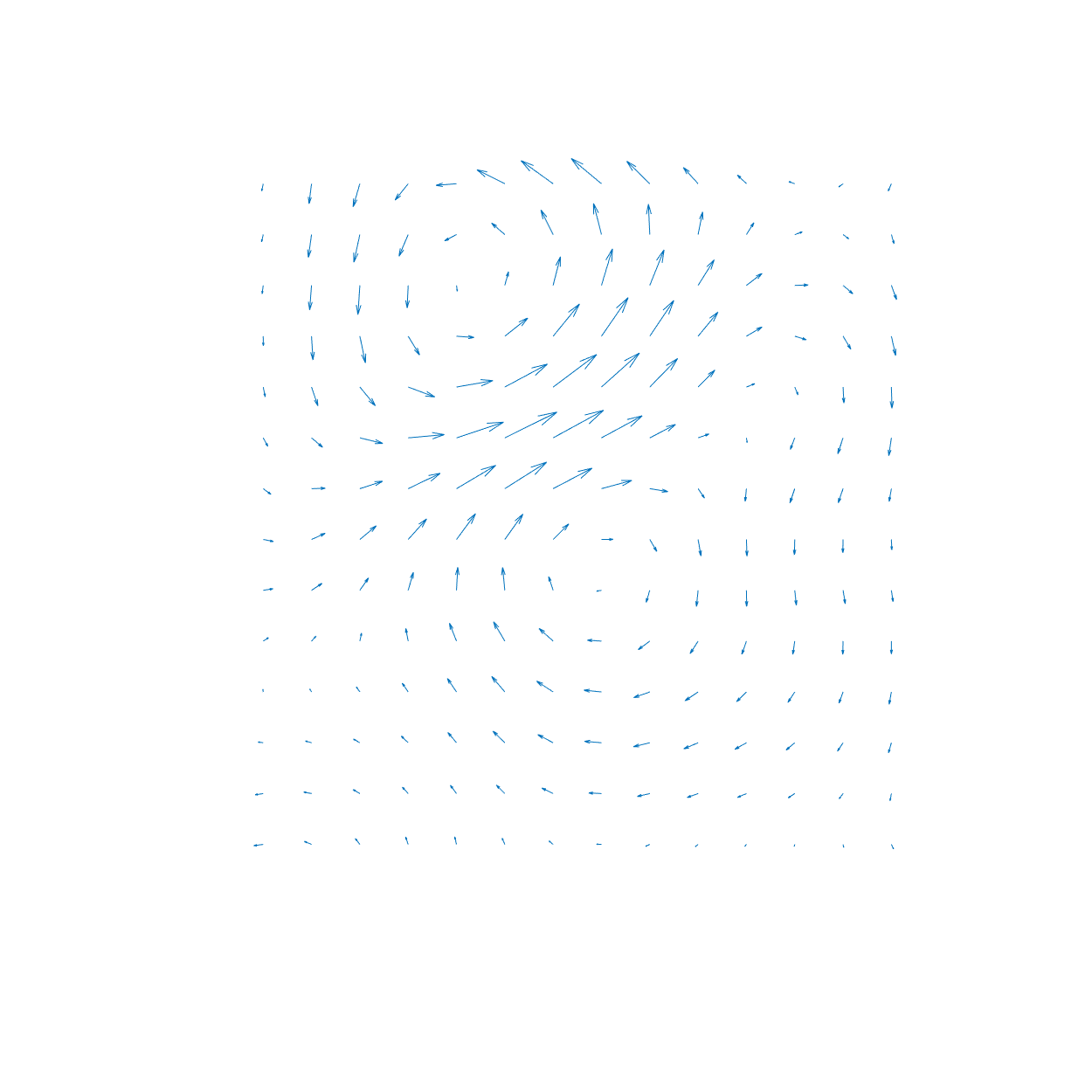

Supplement: S2 MCG raw data 2 — The raw MCG dataset includes categories 0-3 for training and validation. (ZIP) [file pone.0338189.s002.zip › train/0/p4_335_3.png]

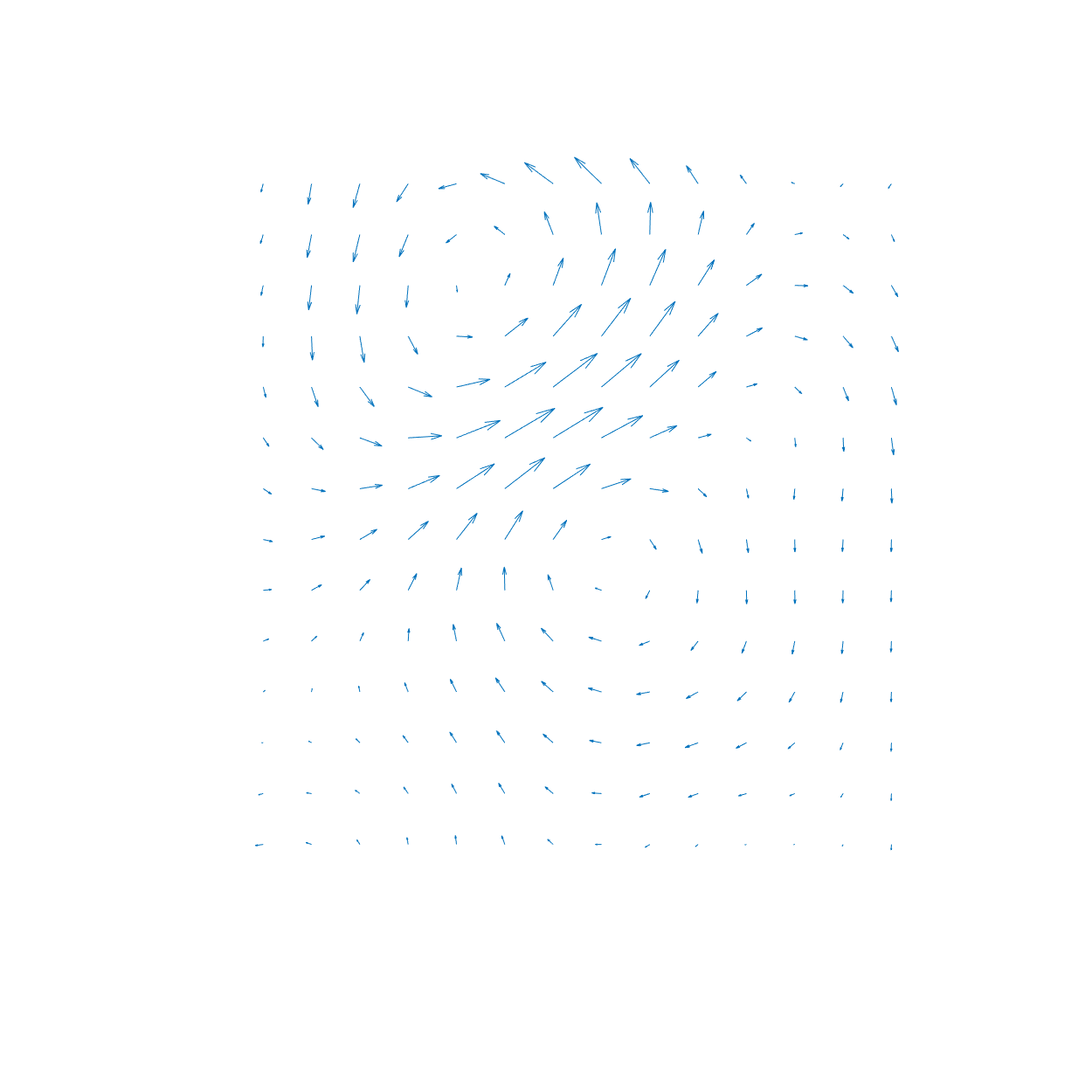

Supplement: S2 MCG raw data 2 — The raw MCG dataset includes categories 0-3 for training and validation. (ZIP) [file pone.0338189.s002.zip › train/0/p4_340_1.png]

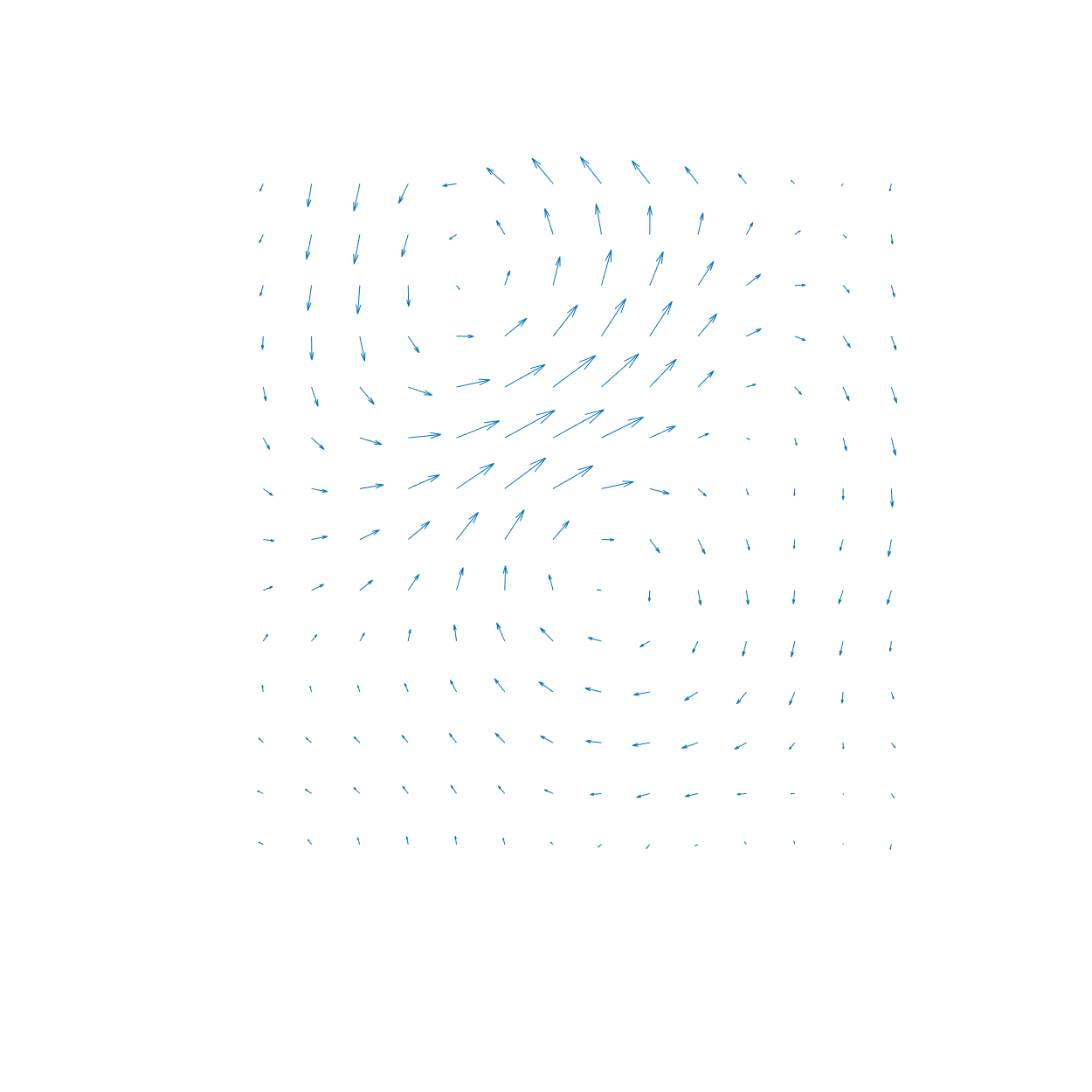

Supplement: S2 MCG raw data 2 — The raw MCG dataset includes categories 0-3 for training and validation. (ZIP) [file pone.0338189.s002.zip › train/0/p4_340_2.png]

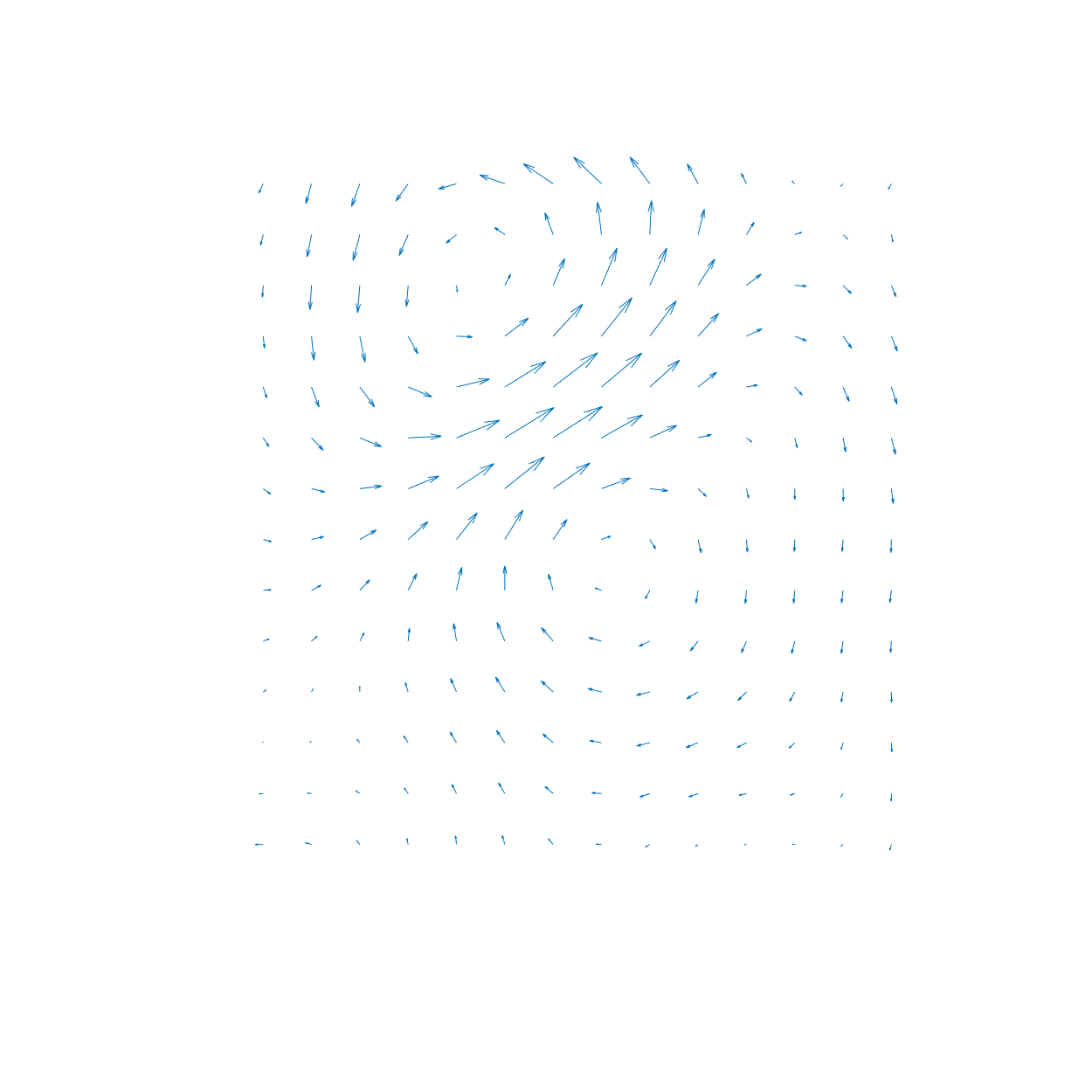

Supplement: S2 MCG raw data 2 — The raw MCG dataset includes categories 0-3 for training and validation. (ZIP) [file pone.0338189.s002.zip › train/0/p4_340_3.png]

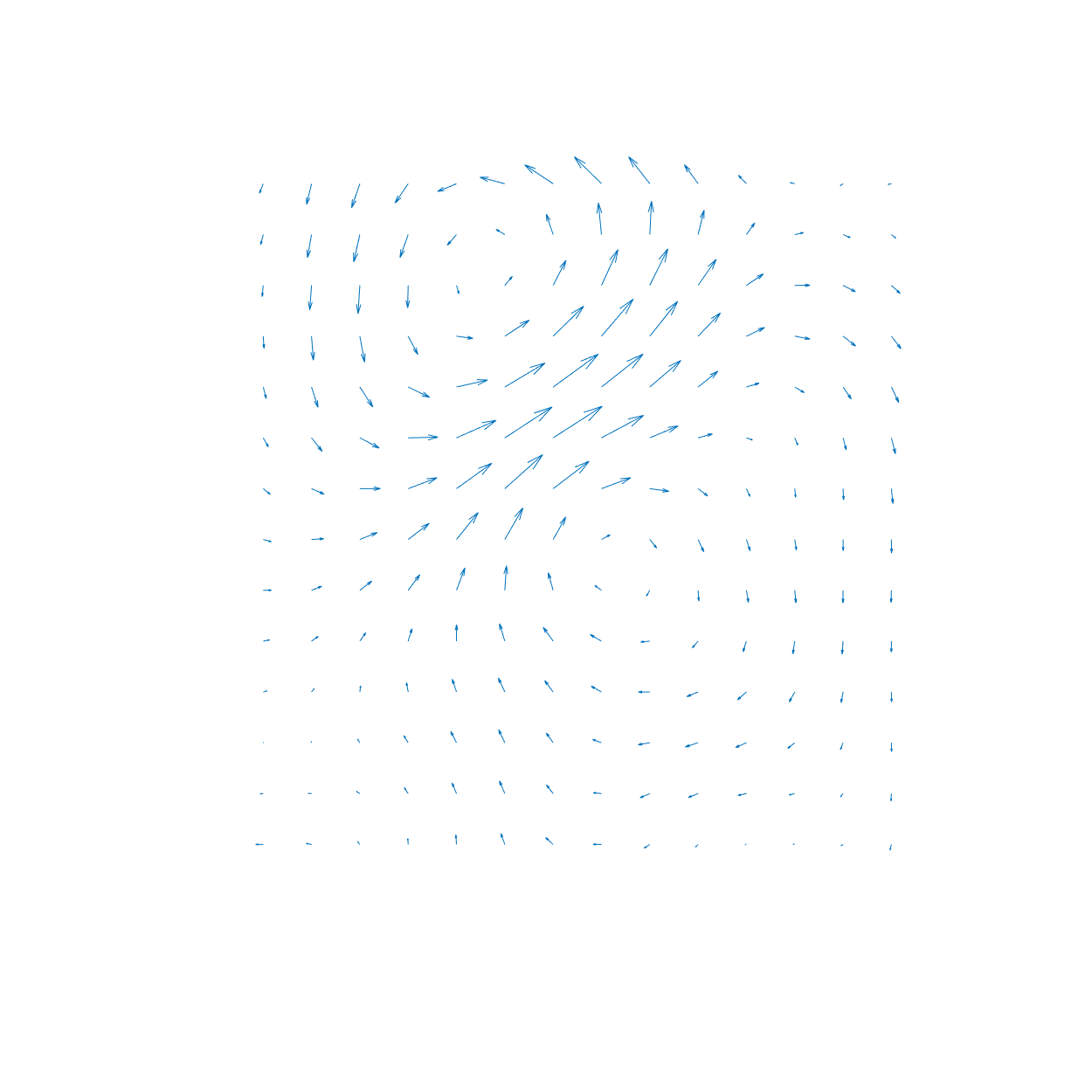

Supplement: S2 MCG raw data 2 — The raw MCG dataset includes categories 0-3 for training and validation. (ZIP) [file pone.0338189.s002.zip › train/0/p4_345_1.png]

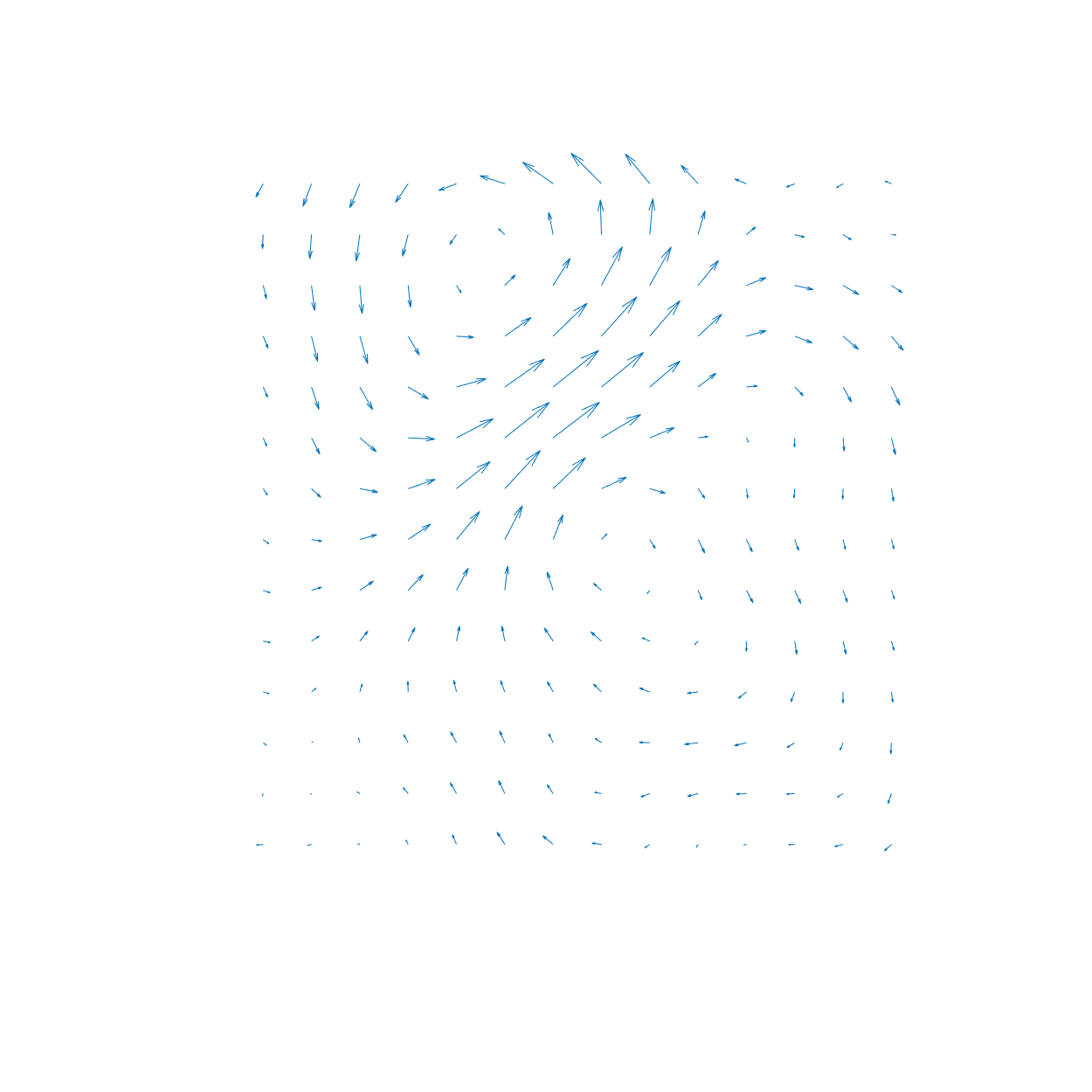

Supplement: S2 MCG raw data 2 — The raw MCG dataset includes categories 0-3 for training and validation. (ZIP) [file pone.0338189.s002.zip › train/0/p4_345_2.png]

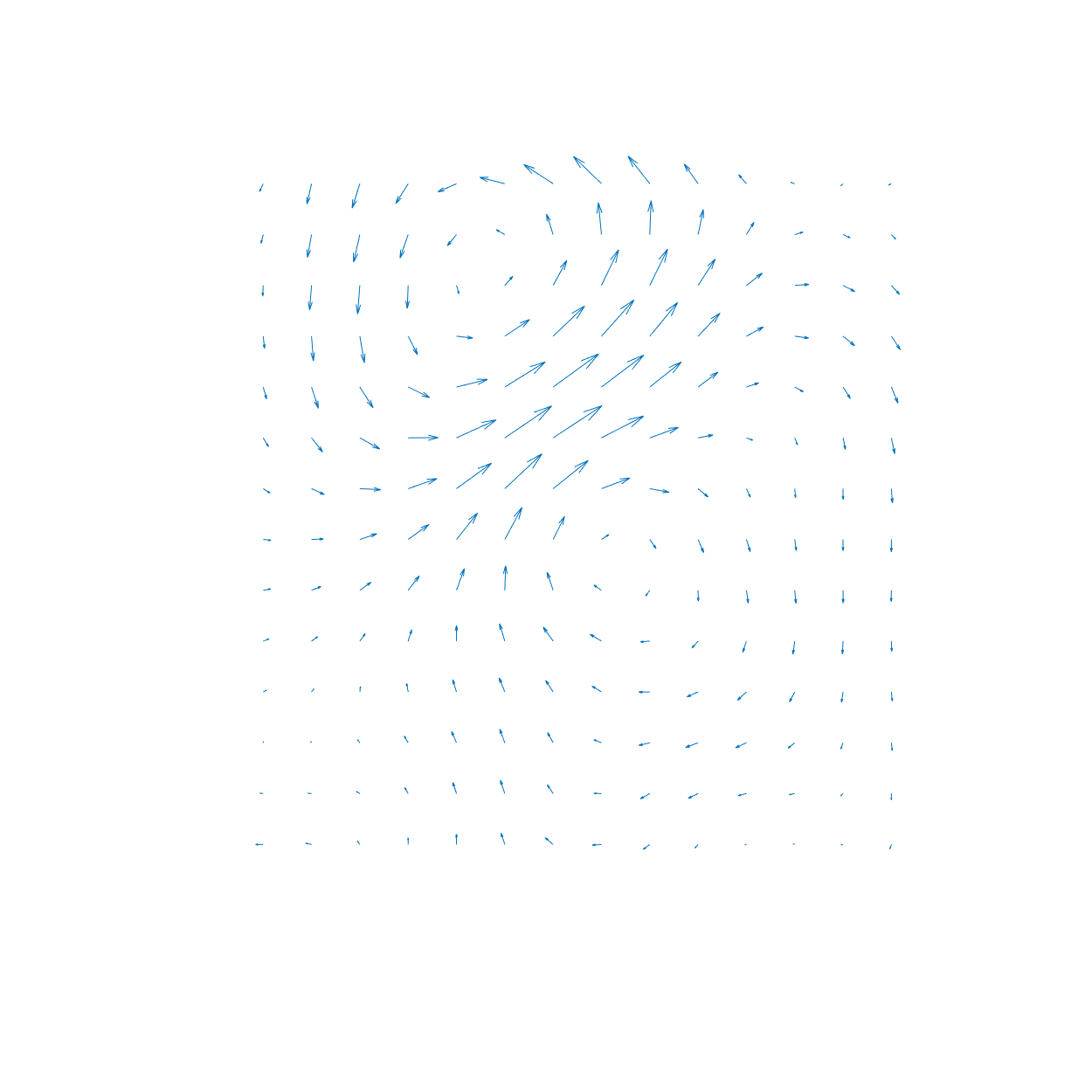

Supplement: S2 MCG raw data 2 — The raw MCG dataset includes categories 0-3 for training and validation. (ZIP) [file pone.0338189.s002.zip › train/0/p4_345_3.png]

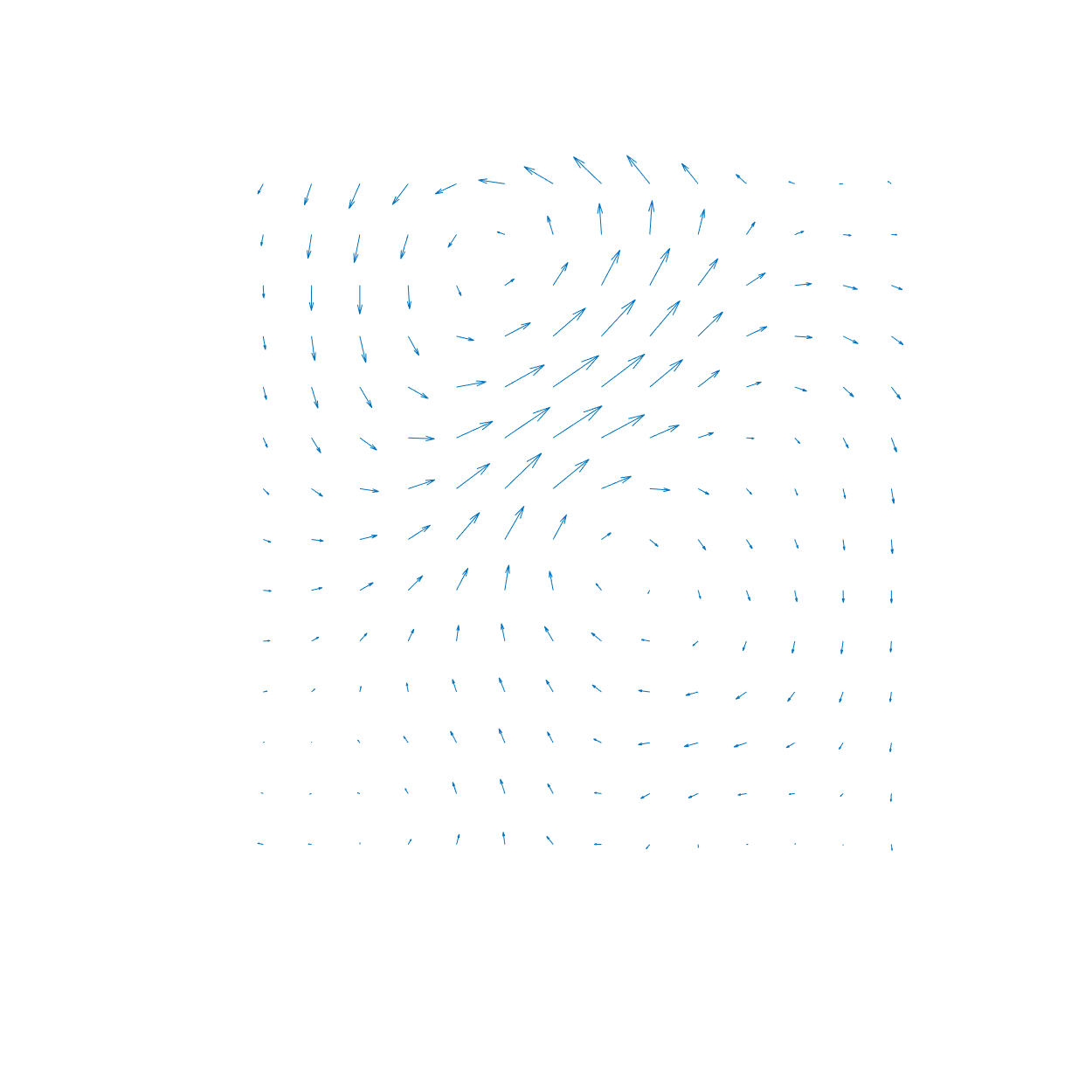

Supplement: S2 MCG raw data 2 — The raw MCG dataset includes categories 0-3 for training and validation. (ZIP) [file pone.0338189.s002.zip › train/0/p4_350_1.png]

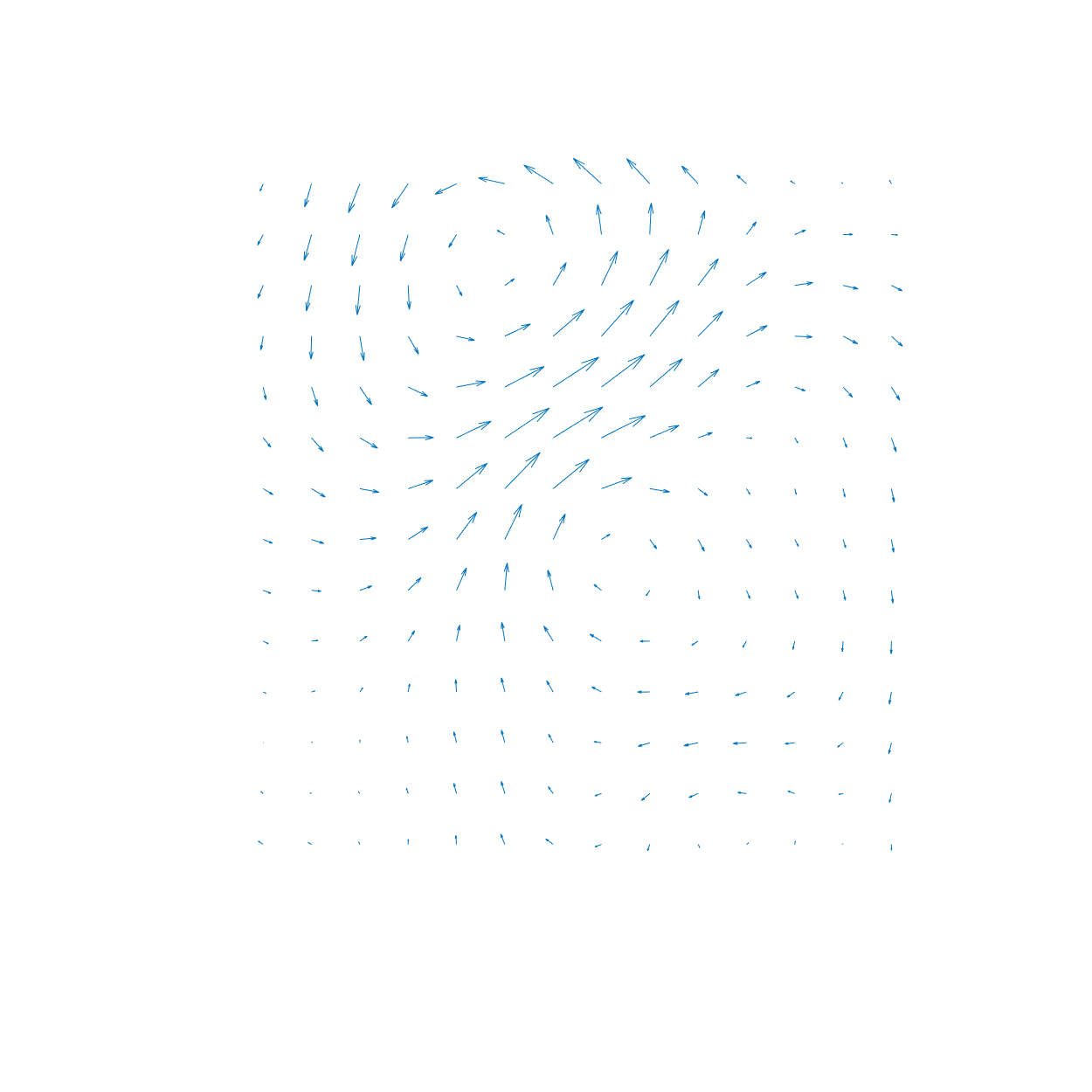

Supplement: S2 MCG raw data 2 — The raw MCG dataset includes categories 0-3 for training and validation. (ZIP) [file pone.0338189.s002.zip › train/0/p4_350_2.png]

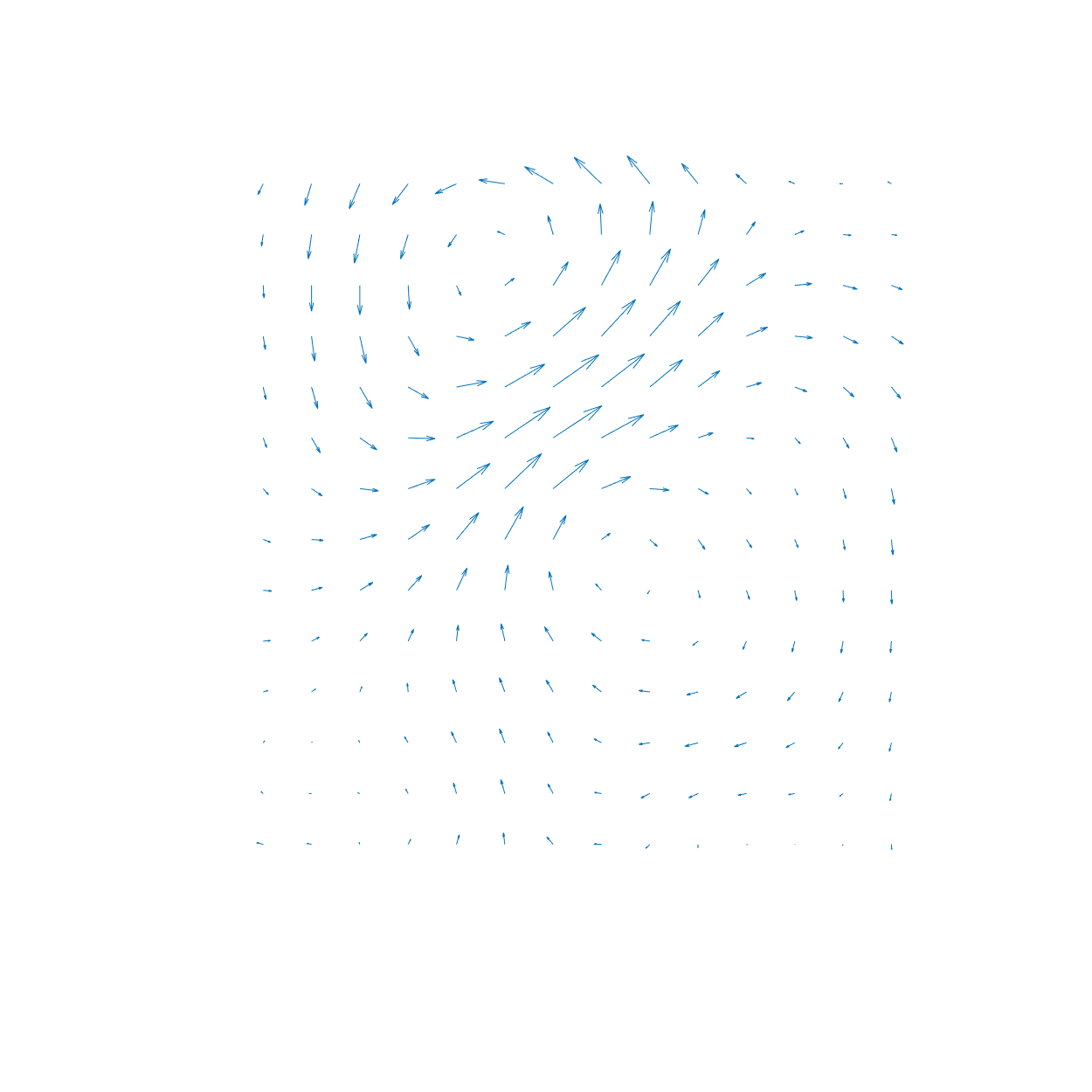

Supplement: S2 MCG raw data 2 — The raw MCG dataset includes categories 0-3 for training and validation. (ZIP) [file pone.0338189.s002.zip › train/0/p4_350_3.png]

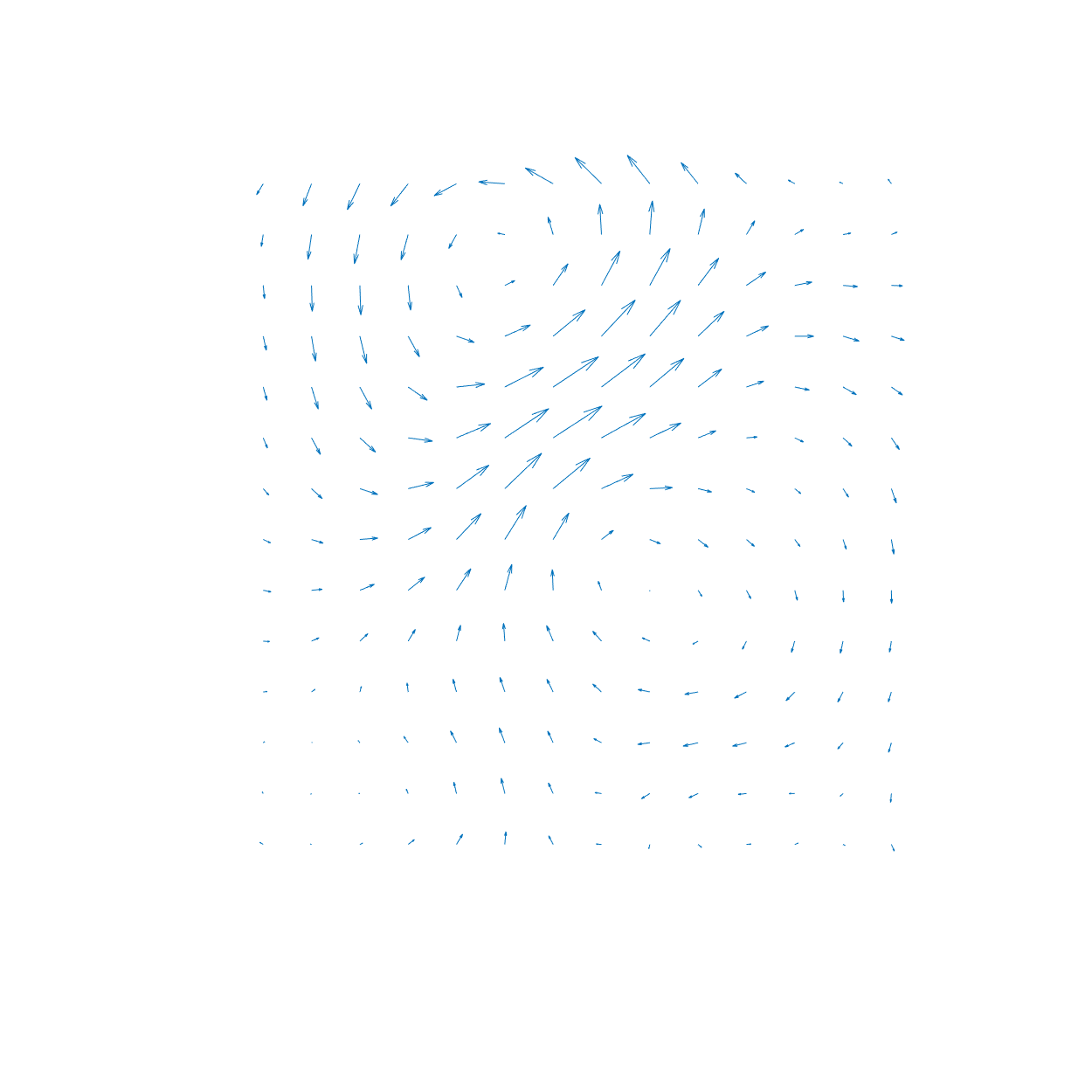

Supplement: S2 MCG raw data 2 — The raw MCG dataset includes categories 0-3 for training and validation. (ZIP) [file pone.0338189.s002.zip › train/0/p4_355_1.png]

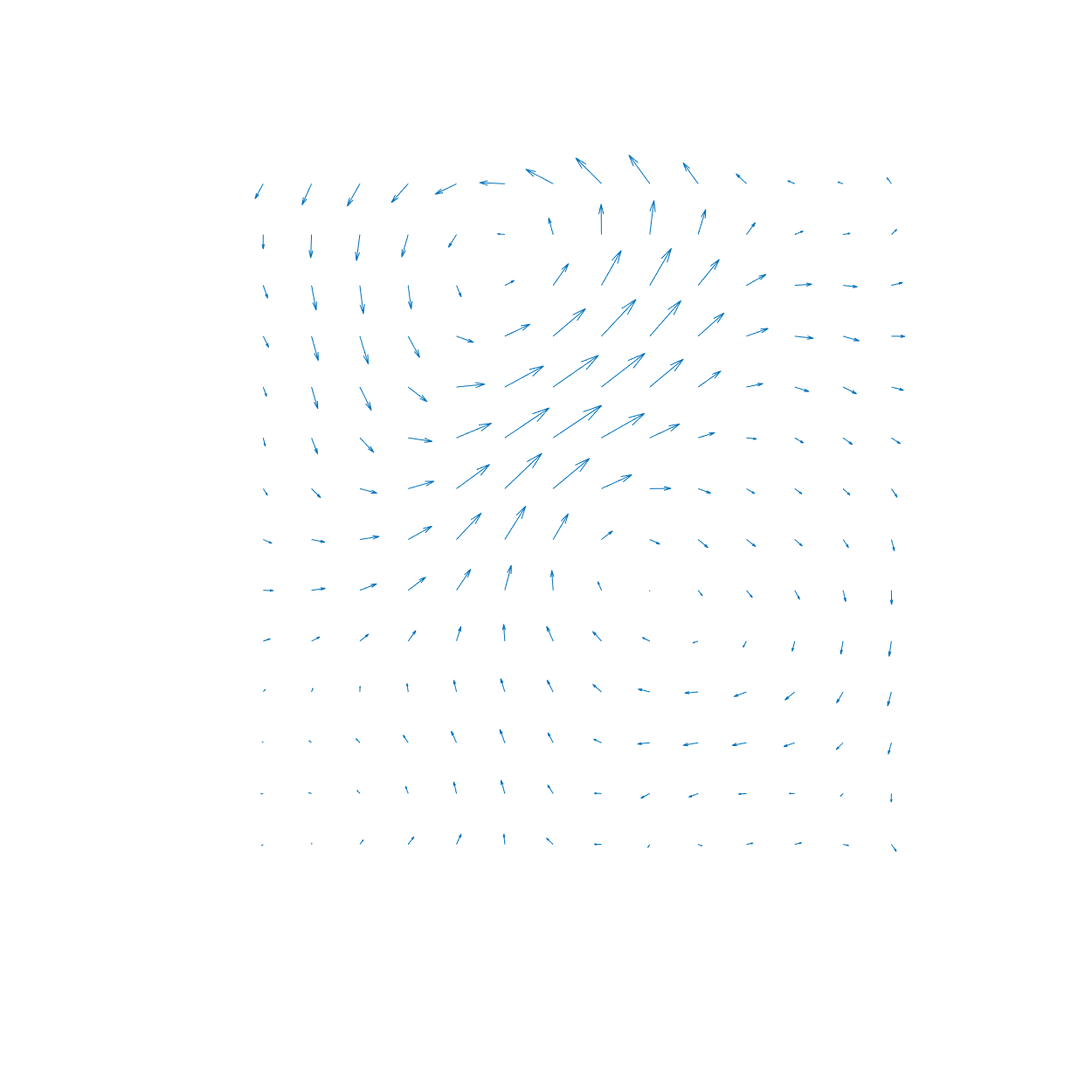

Supplement: S2 MCG raw data 2 — The raw MCG dataset includes categories 0-3 for training and validation. (ZIP) [file pone.0338189.s002.zip › train/0/p4_355_2.png]

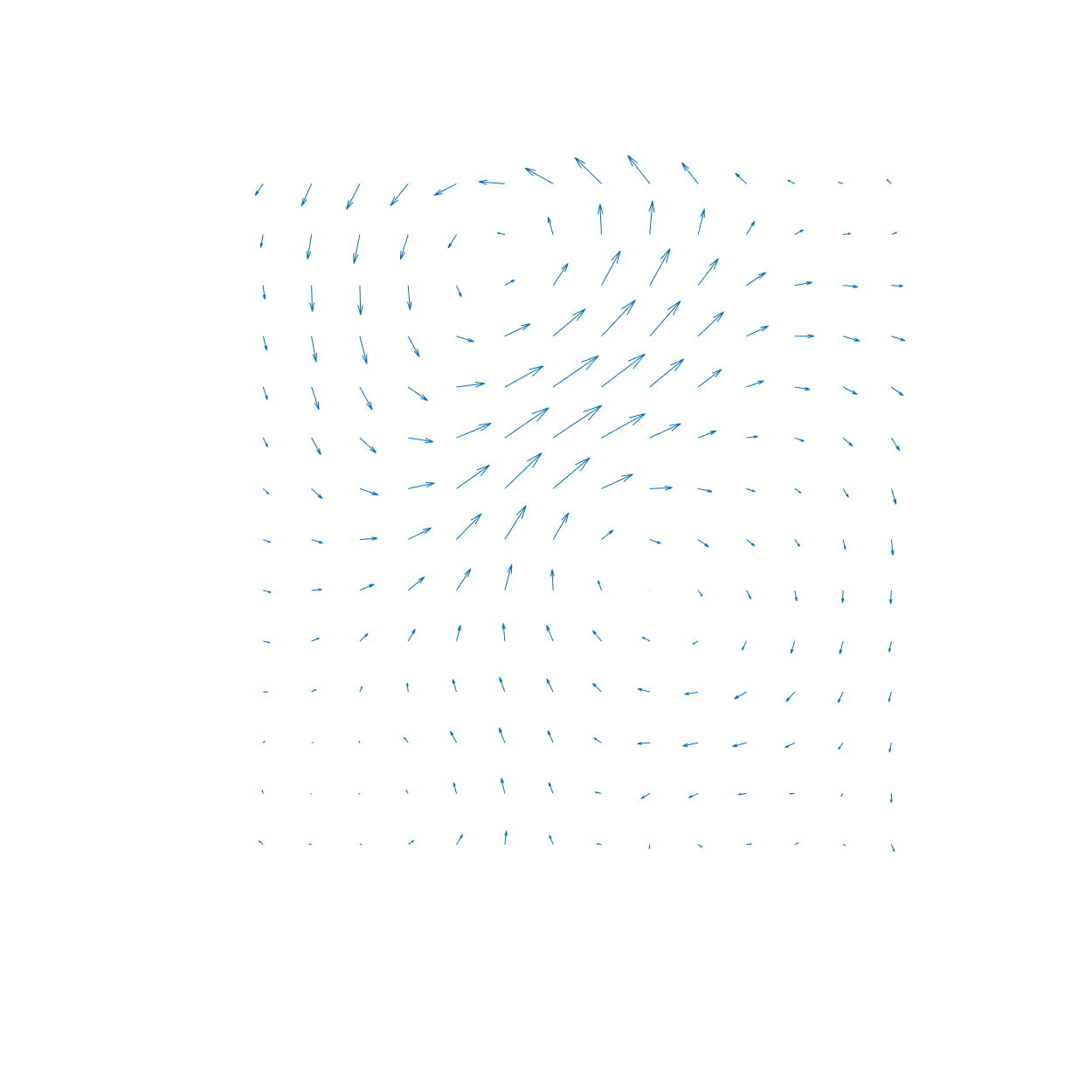

Supplement: S2 MCG raw data 2 — The raw MCG dataset includes categories 0-3 for training and validation. (ZIP) [file pone.0338189.s002.zip › train/0/p4_355_3.png]

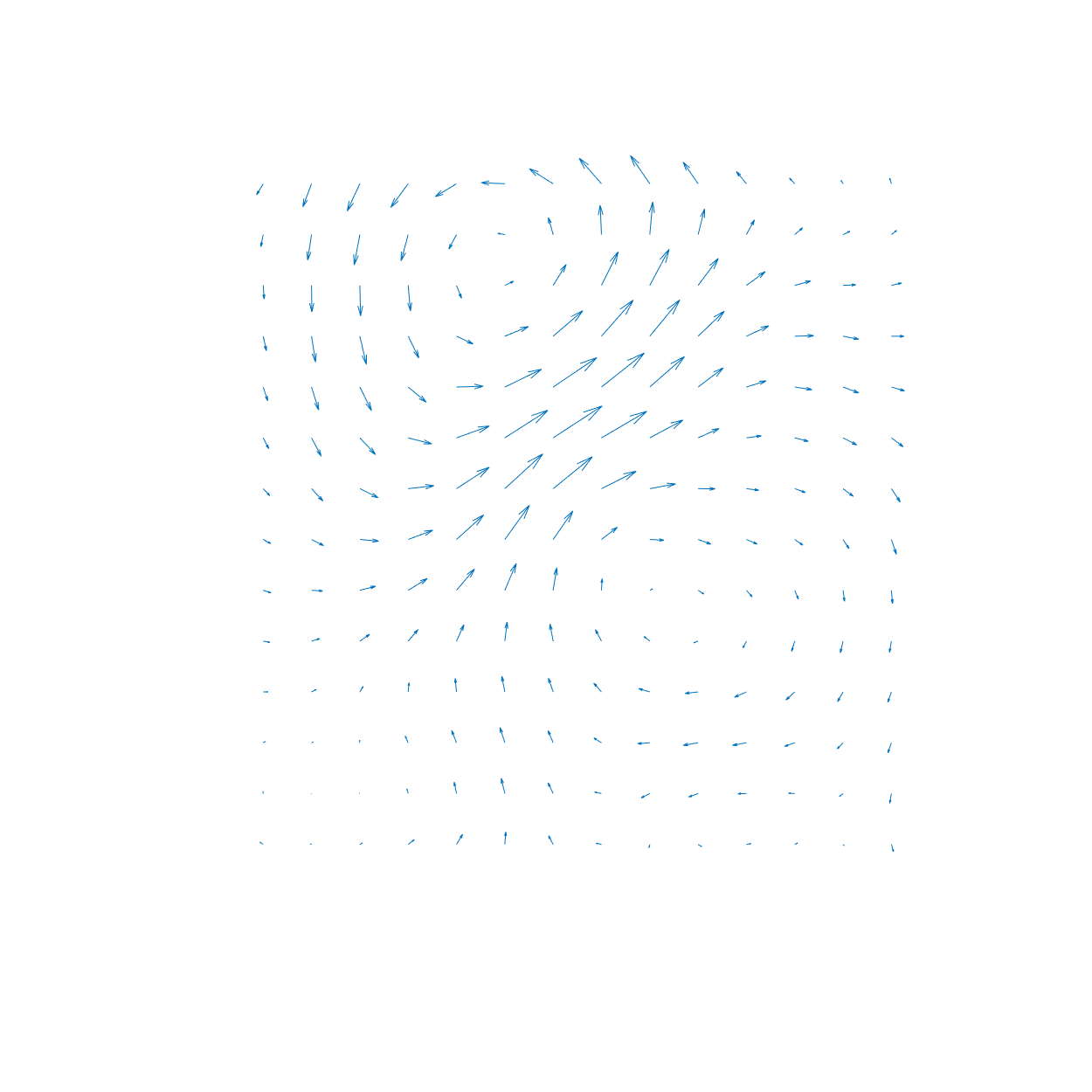

Supplement: S2 MCG raw data 2 — The raw MCG dataset includes categories 0-3 for training and validation. (ZIP) [file pone.0338189.s002.zip › train/0/p4_360_1.png]

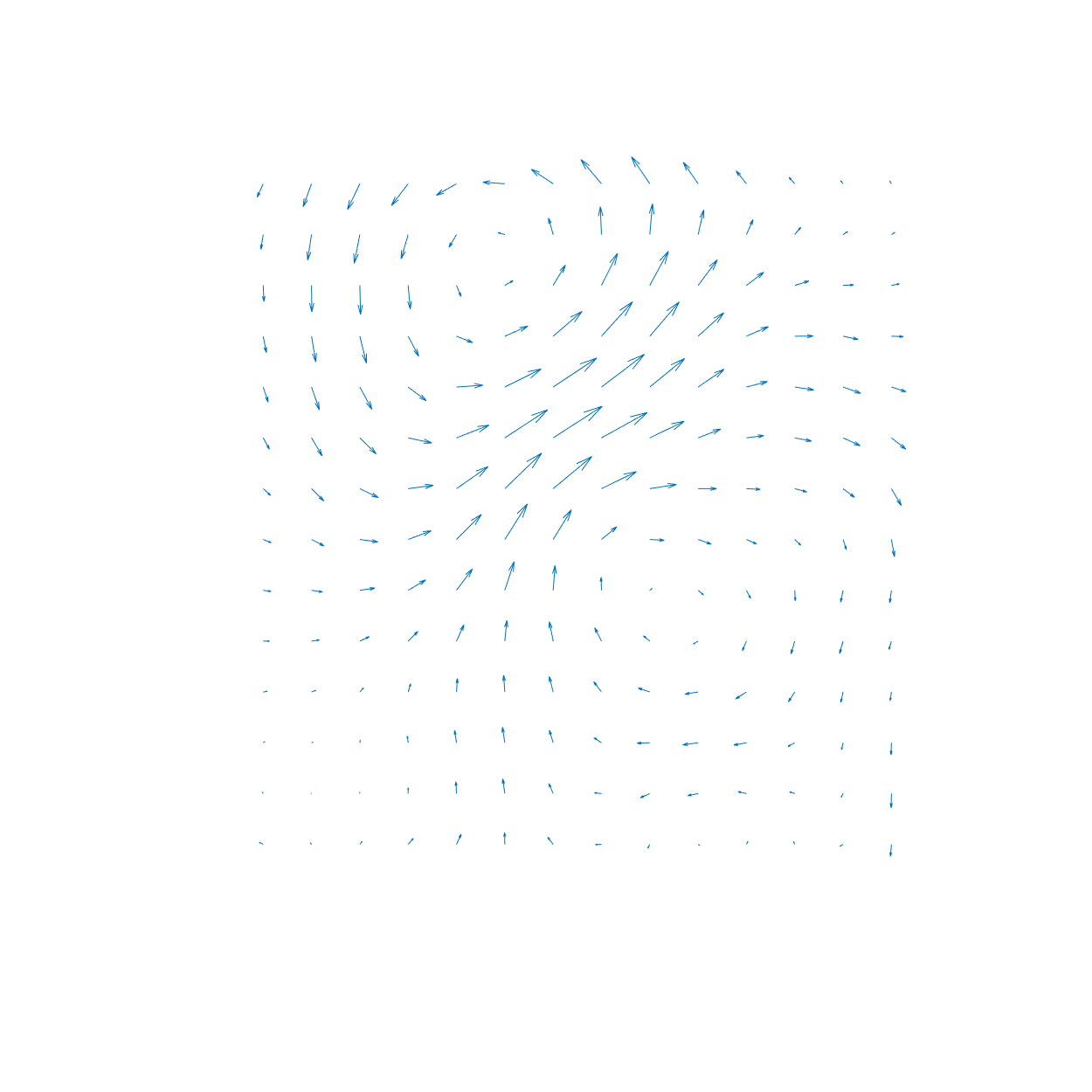

Supplement: S2 MCG raw data 2 — The raw MCG dataset includes categories 0-3 for training and validation. (ZIP) [file pone.0338189.s002.zip › train/0/p4_360_2.png]

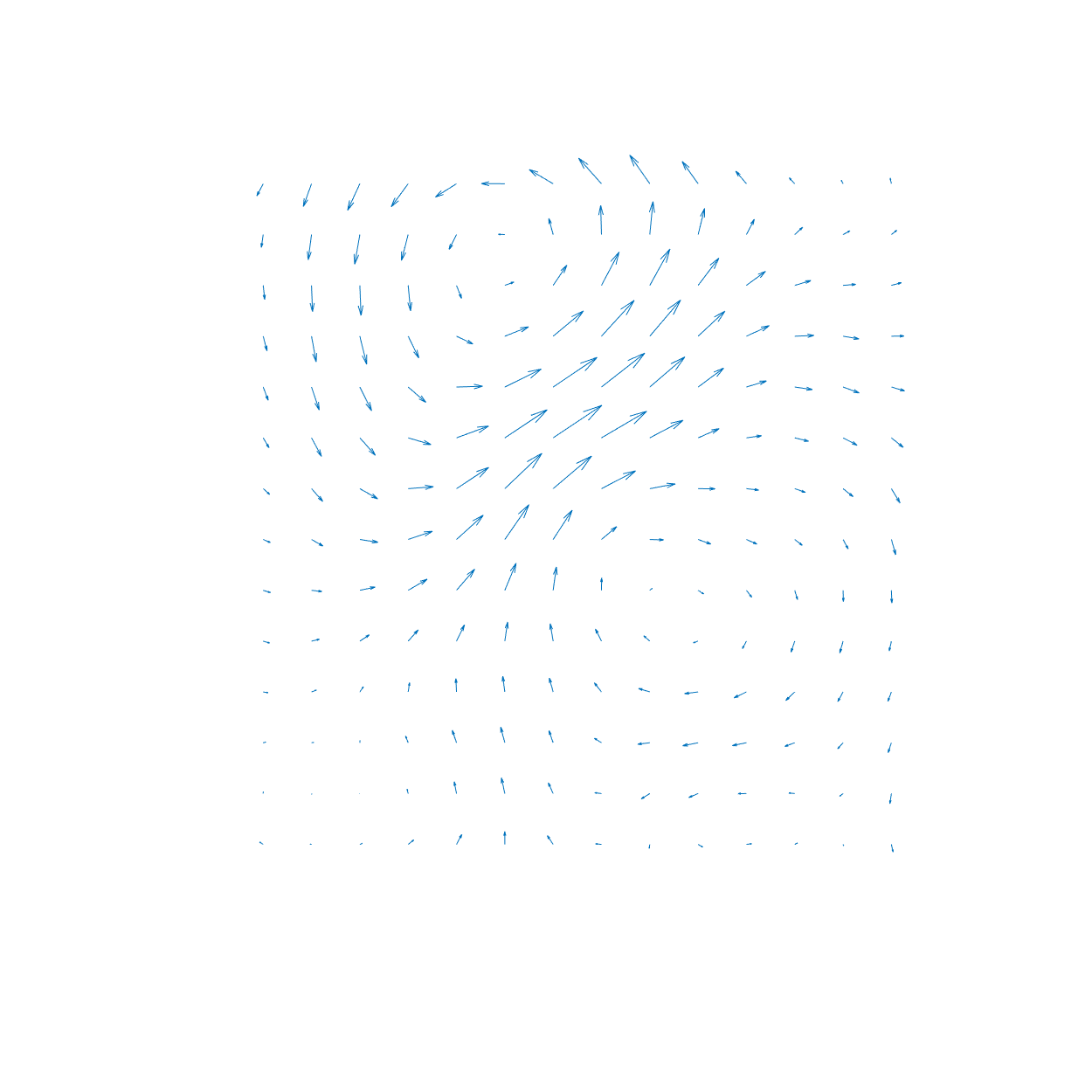

Supplement: S2 MCG raw data 2 — The raw MCG dataset includes categories 0-3 for training and validation. (ZIP) [file pone.0338189.s002.zip › train/0/p4_360_3.png]

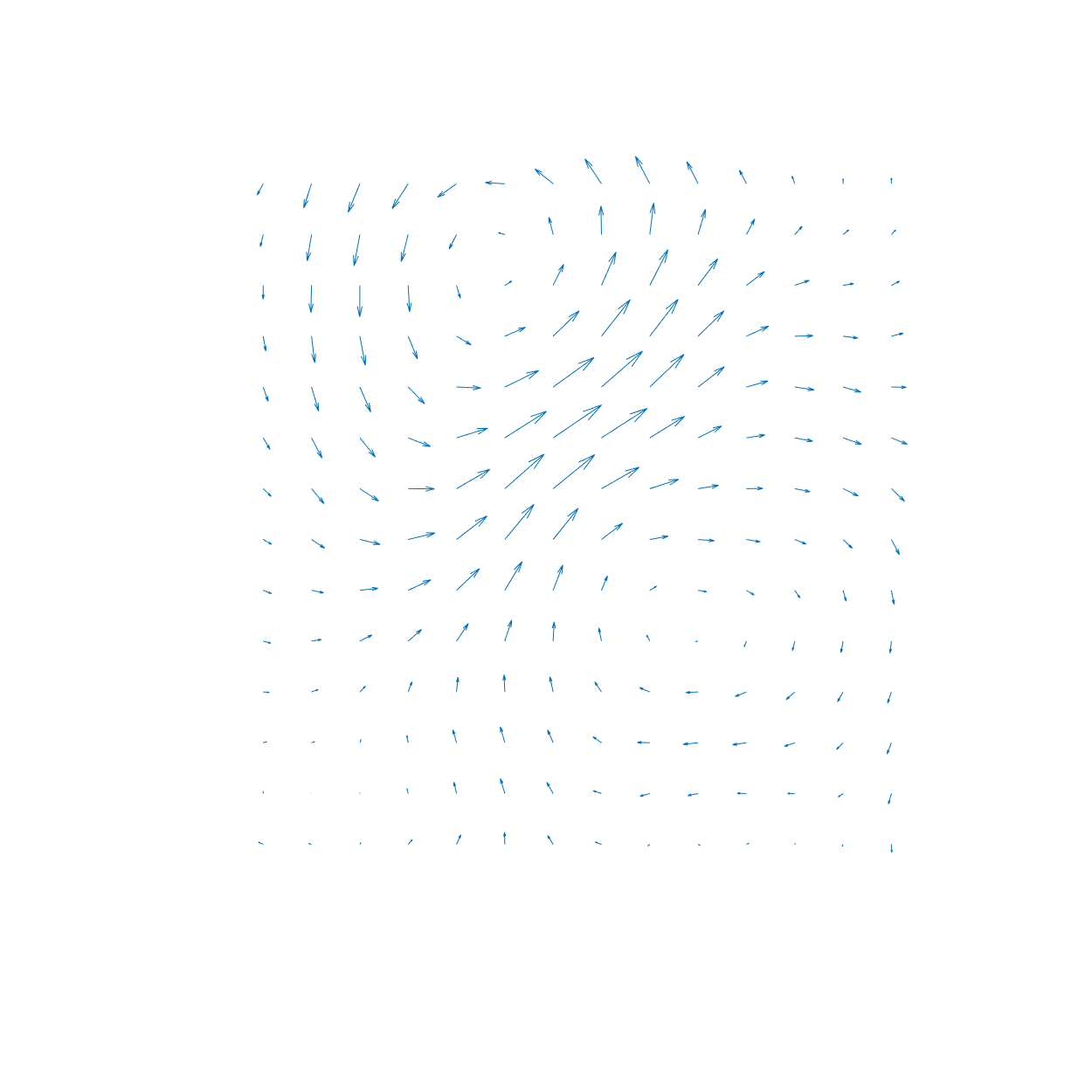

Supplement: S2 MCG raw data 2 — The raw MCG dataset includes categories 0-3 for training and validation. (ZIP) [file pone.0338189.s002.zip › train/0/p4_365_1.png]

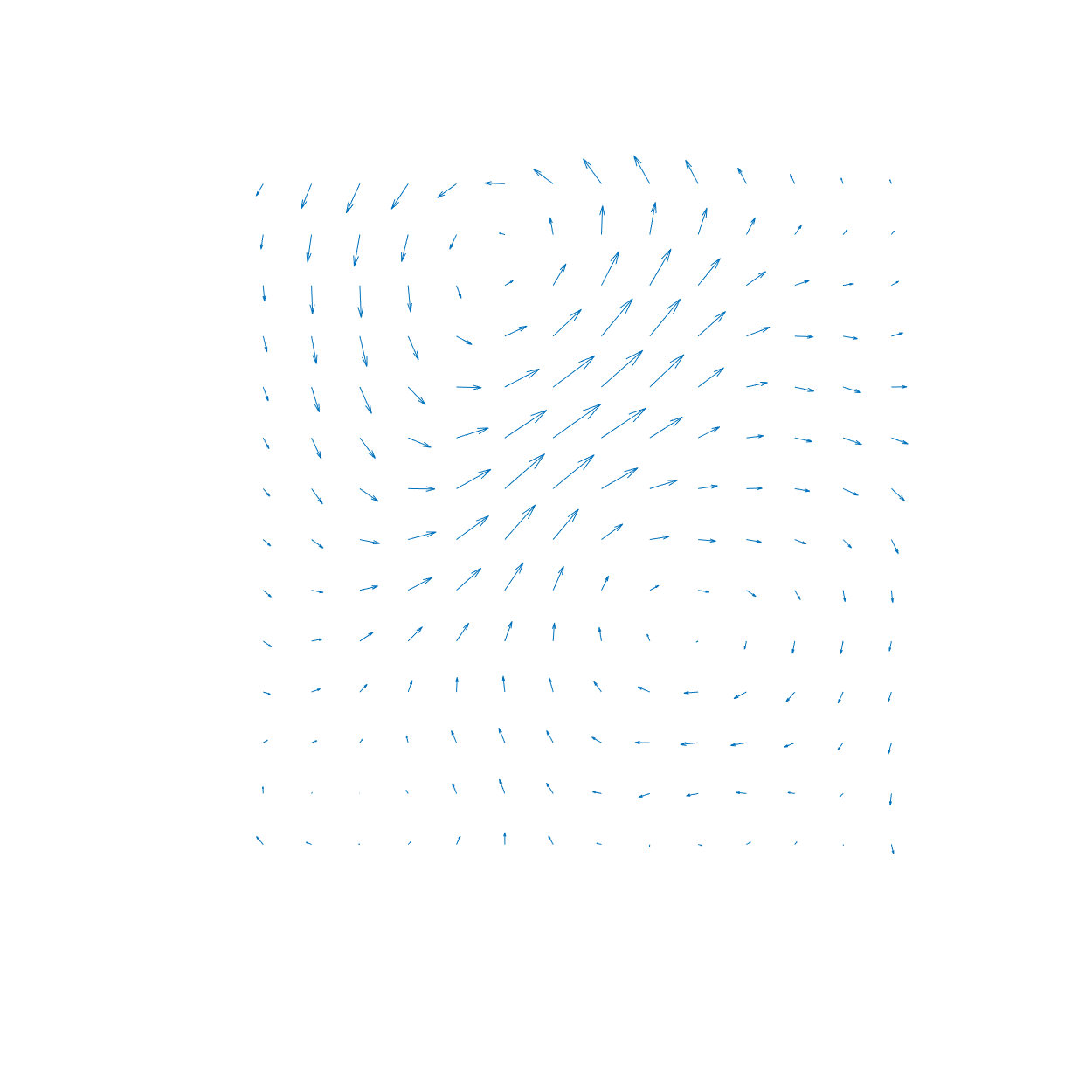

Supplement: S2 MCG raw data 2 — The raw MCG dataset includes categories 0-3 for training and validation. (ZIP) [file pone.0338189.s002.zip › train/0/p4_365_2.png]

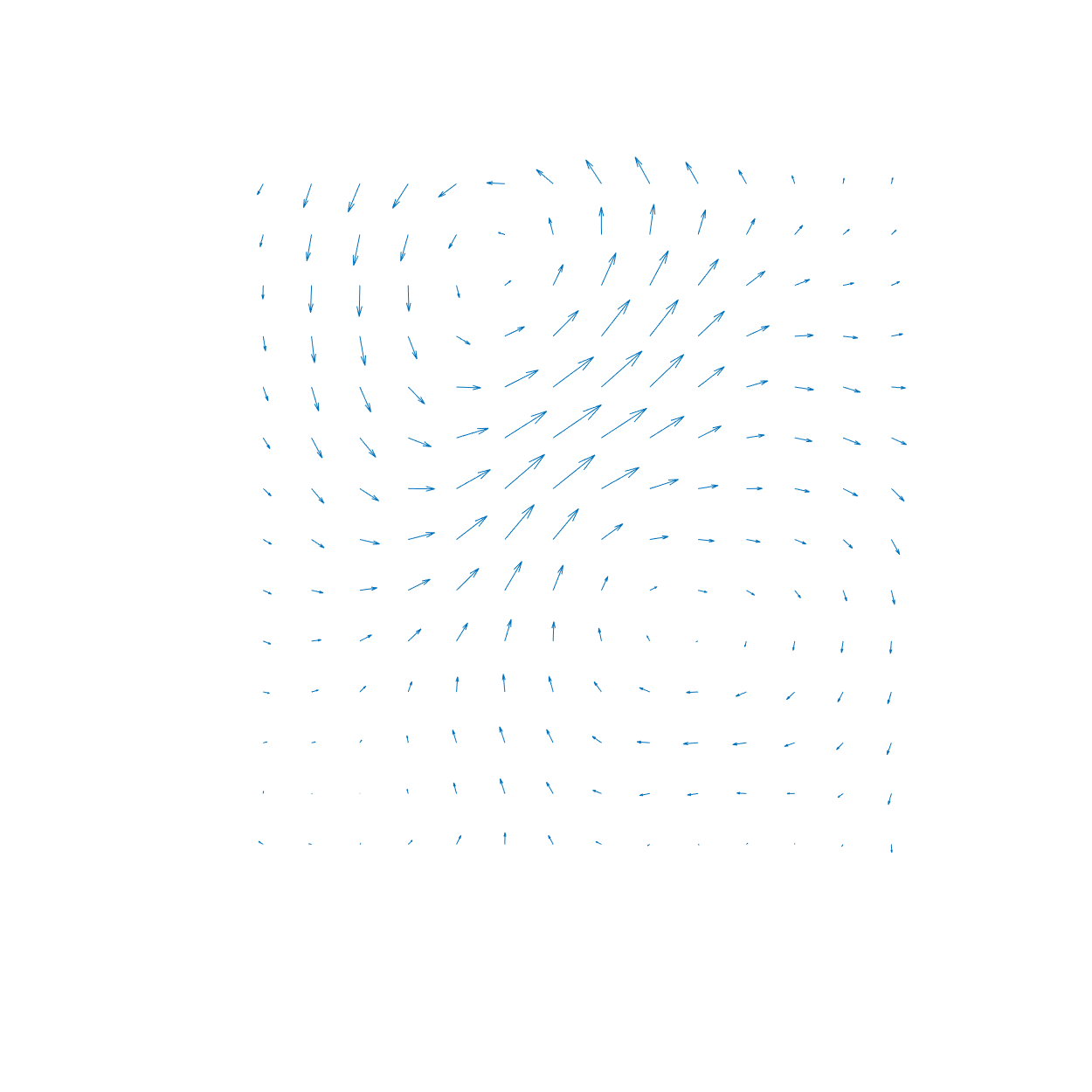

Supplement: S2 MCG raw data 2 — The raw MCG dataset includes categories 0-3 for training and validation. (ZIP) [file pone.0338189.s002.zip › train/0/p4_365_3.png]

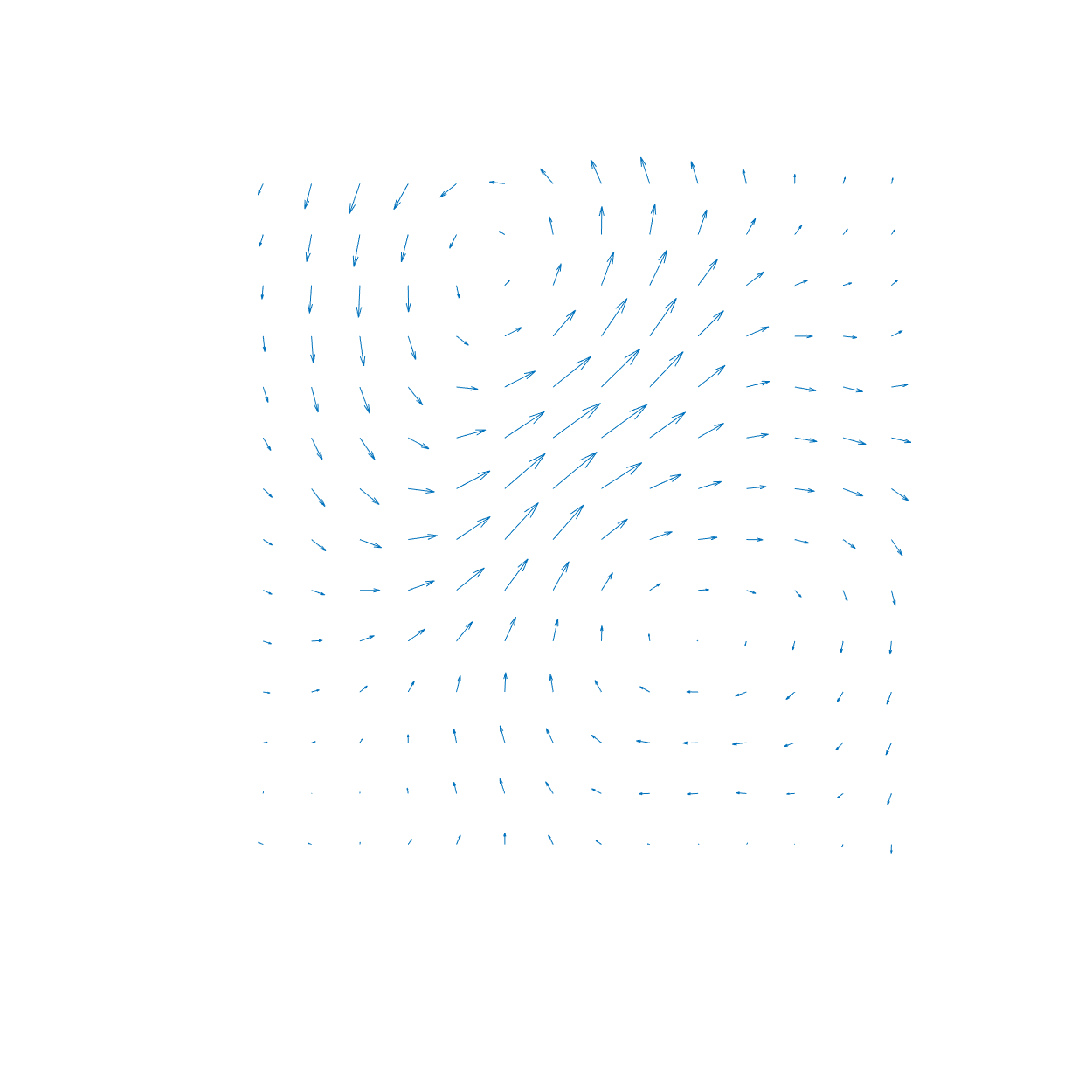

Supplement: S2 MCG raw data 2 — The raw MCG dataset includes categories 0-3 for training and validation. (ZIP) [file pone.0338189.s002.zip › train/0/p4_370_1.png]

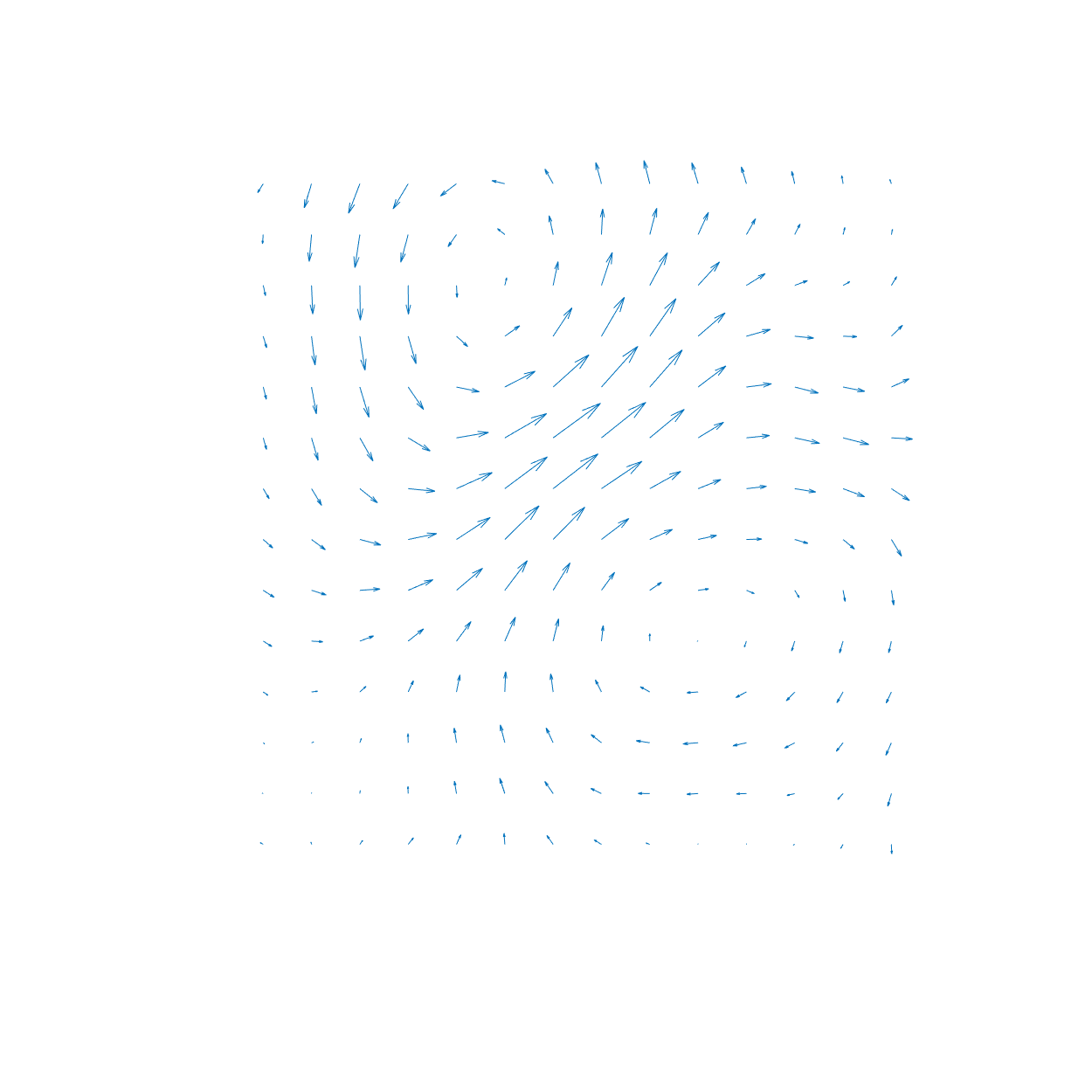

Supplement: S2 MCG raw data 2 — The raw MCG dataset includes categories 0-3 for training and validation. (ZIP) [file pone.0338189.s002.zip › train/0/p4_370_2.png]

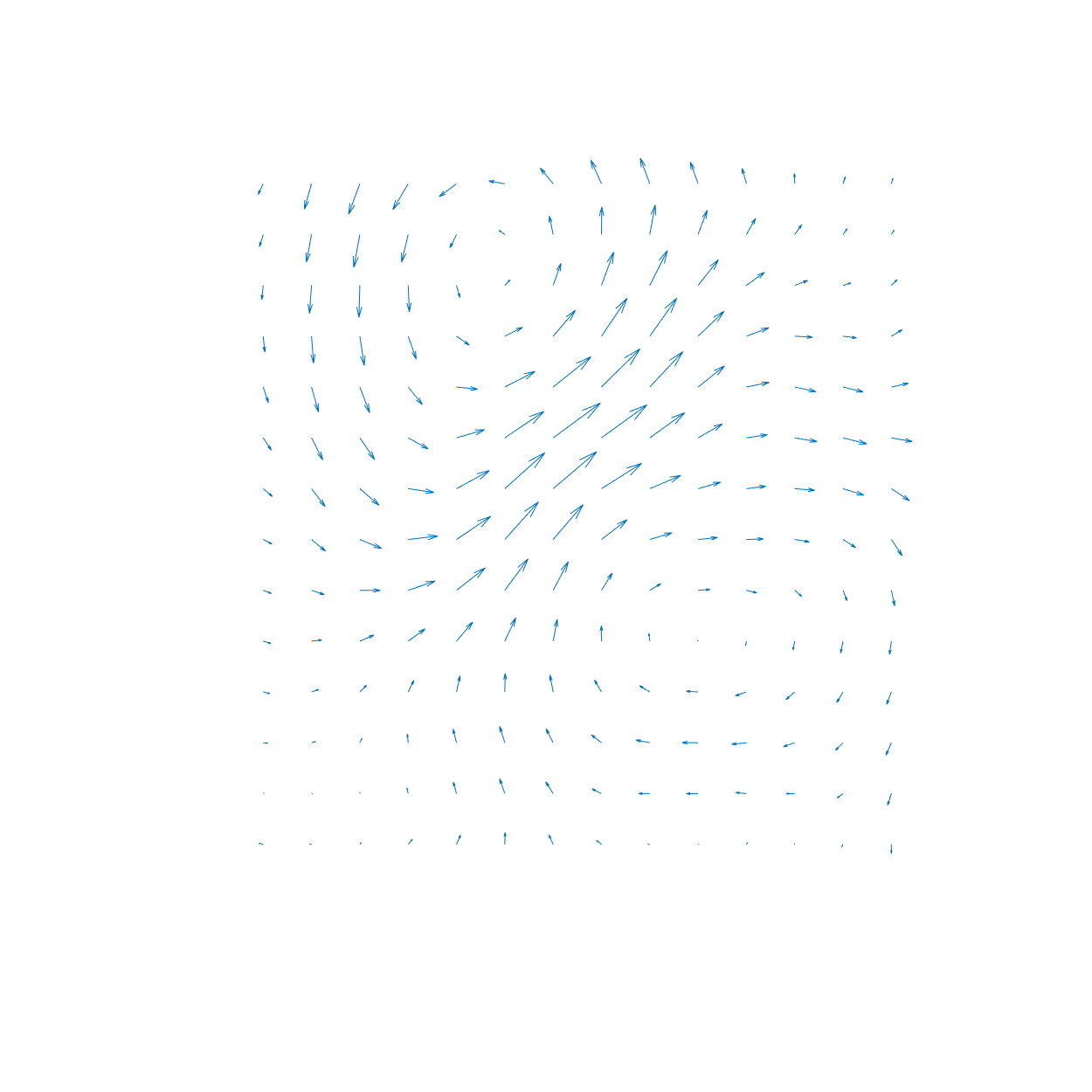

Supplement: S2 MCG raw data 2 — The raw MCG dataset includes categories 0-3 for training and validation. (ZIP) [file pone.0338189.s002.zip › train/0/p4_370_3.png]

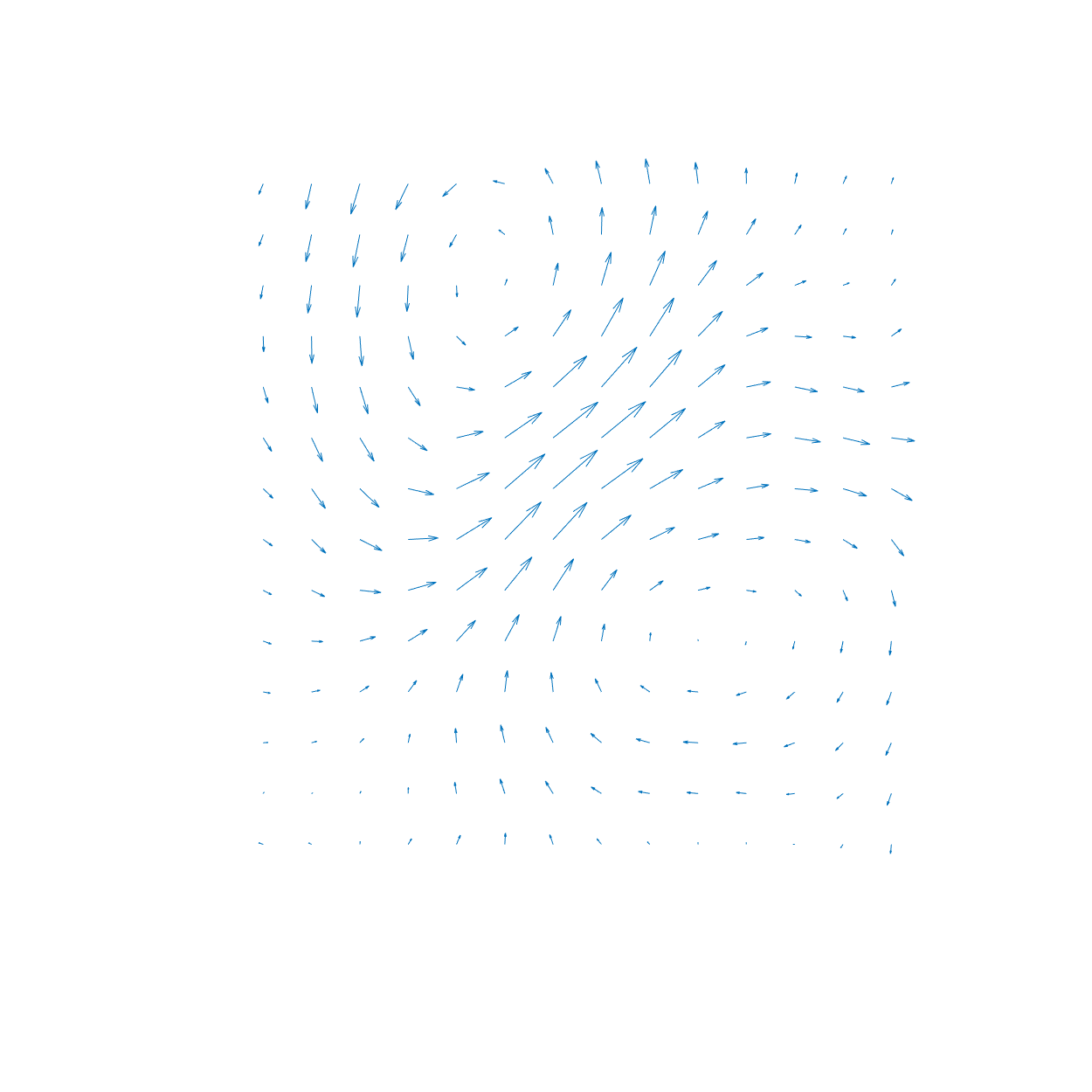

Supplement: S2 MCG raw data 2 — The raw MCG dataset includes categories 0-3 for training and validation. (ZIP) [file pone.0338189.s002.zip › train/0/p4_375_1.png]

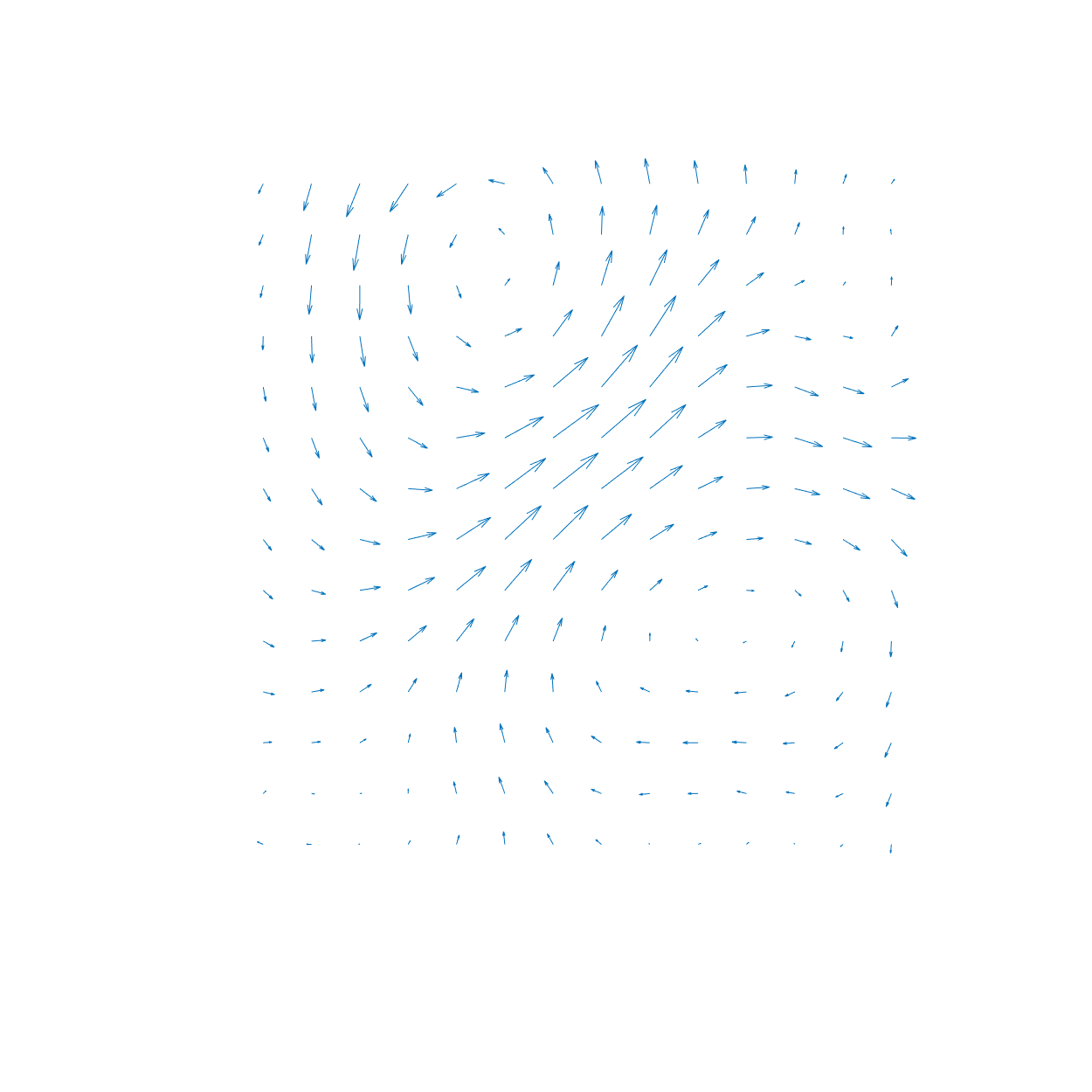

Supplement: S2 MCG raw data 2 — The raw MCG dataset includes categories 0-3 for training and validation. (ZIP) [file pone.0338189.s002.zip › train/0/p4_375_2.png]

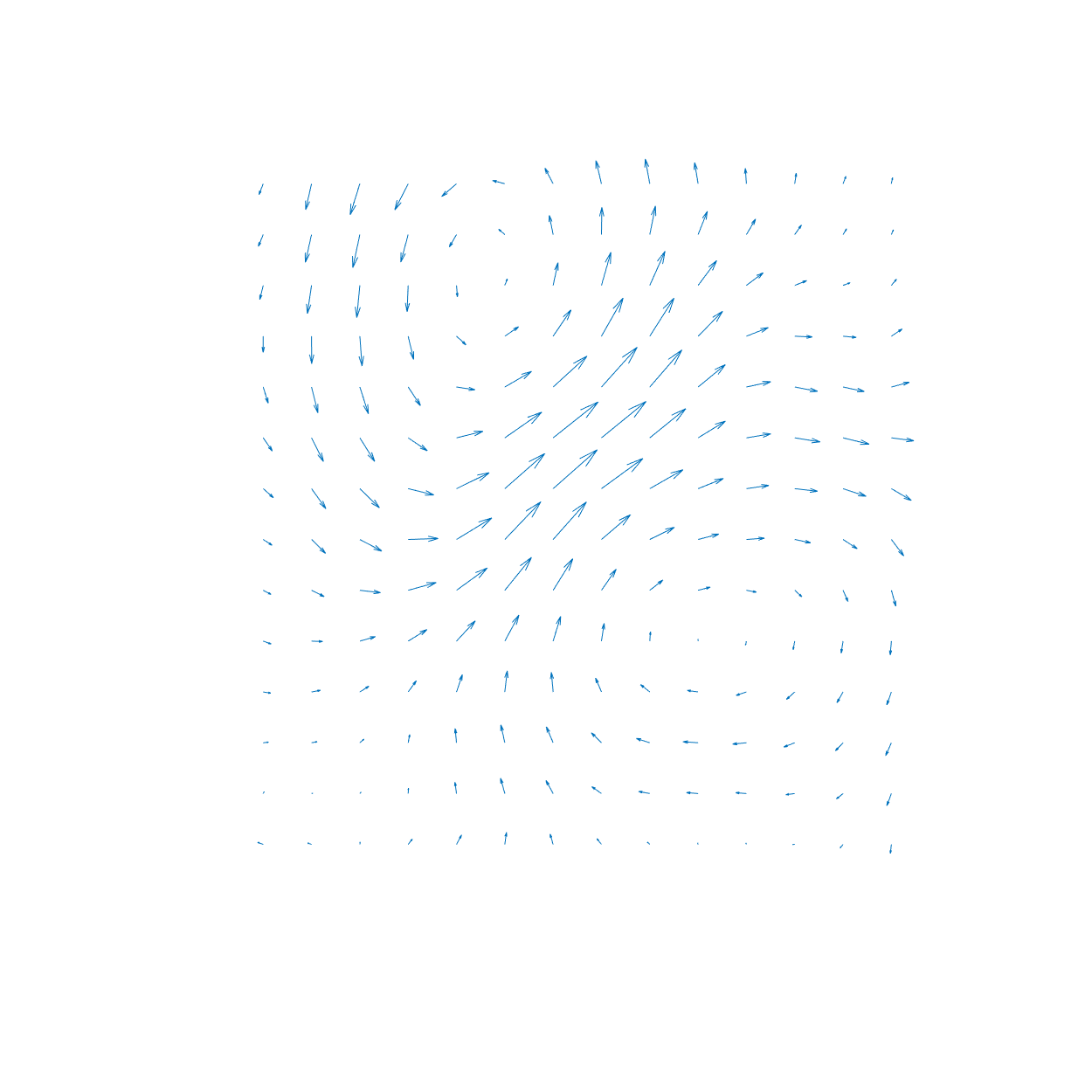

Supplement: S2 MCG raw data 2 — The raw MCG dataset includes categories 0-3 for training and validation. (ZIP) [file pone.0338189.s002.zip › train/0/p4_375_3.png]

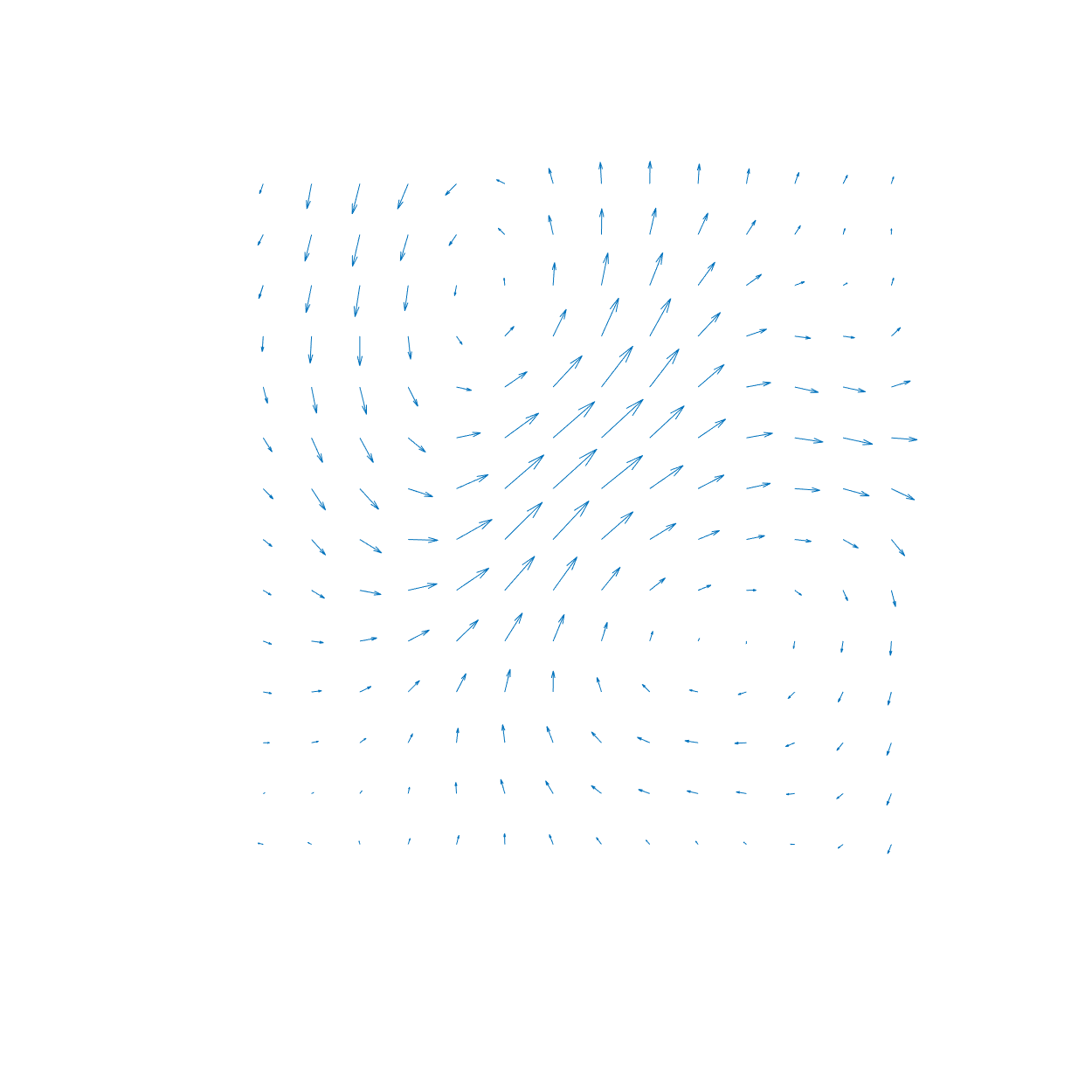

Supplement: S2 MCG raw data 2 — The raw MCG dataset includes categories 0-3 for training and validation. (ZIP) [file pone.0338189.s002.zip › train/0/p4_380_1.png]

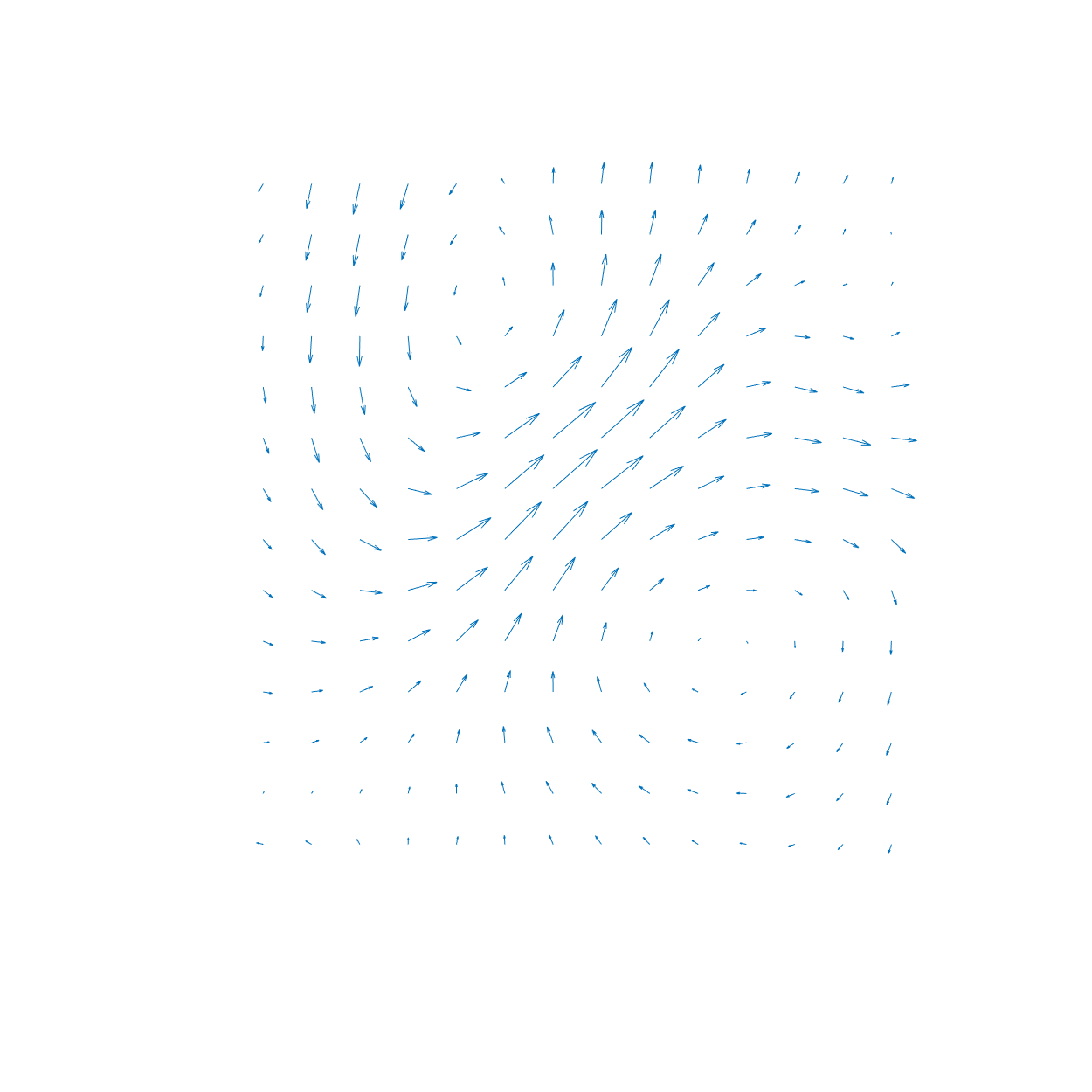

Supplement: S2 MCG raw data 2 — The raw MCG dataset includes categories 0-3 for training and validation. (ZIP) [file pone.0338189.s002.zip › train/0/p4_380_2.png]

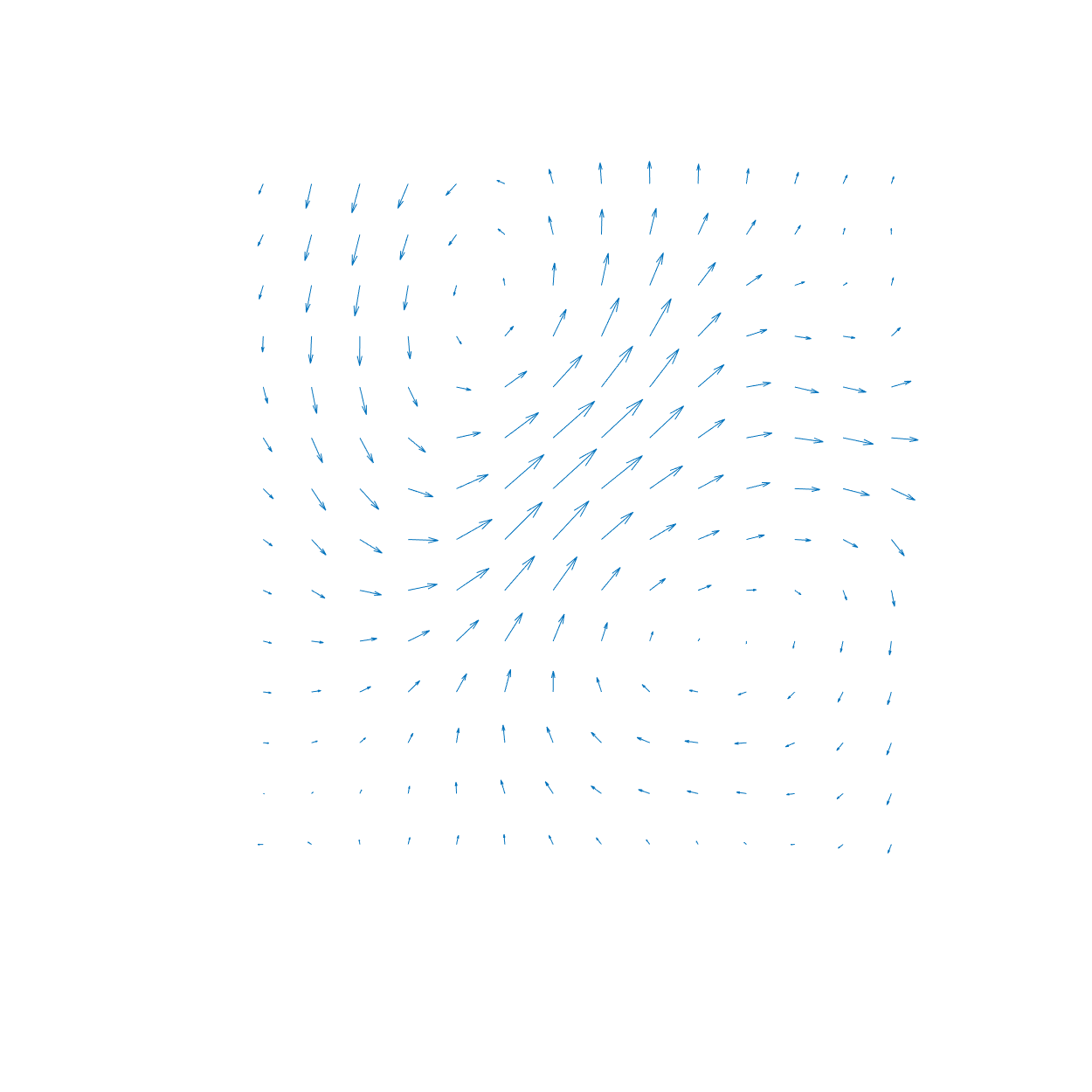

Supplement: S2 MCG raw data 2 — The raw MCG dataset includes categories 0-3 for training and validation. (ZIP) [file pone.0338189.s002.zip › train/0/p4_380_3.png]

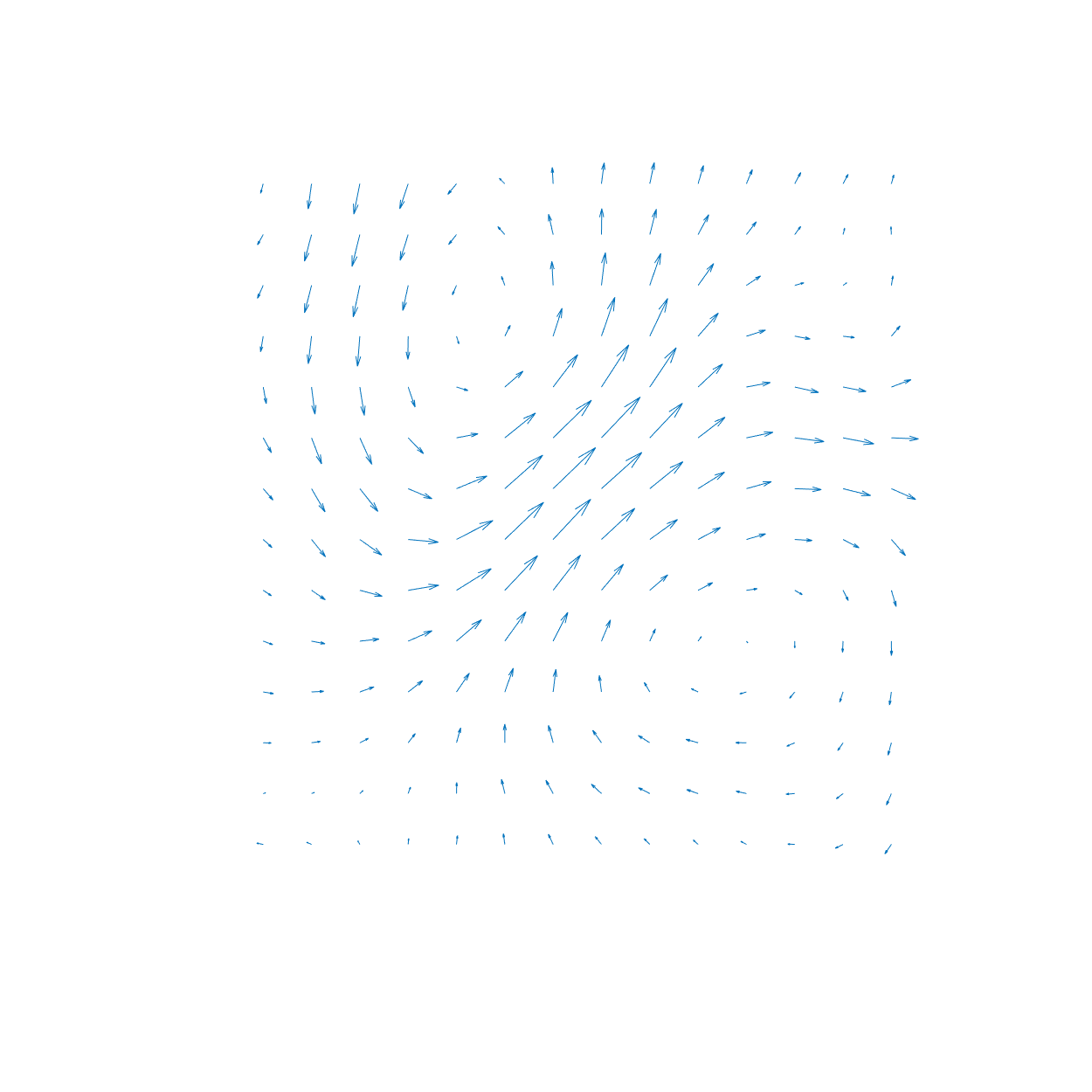

Supplement: S2 MCG raw data 2 — The raw MCG dataset includes categories 0-3 for training and validation. (ZIP) [file pone.0338189.s002.zip › train/0/p4_385_1.png]

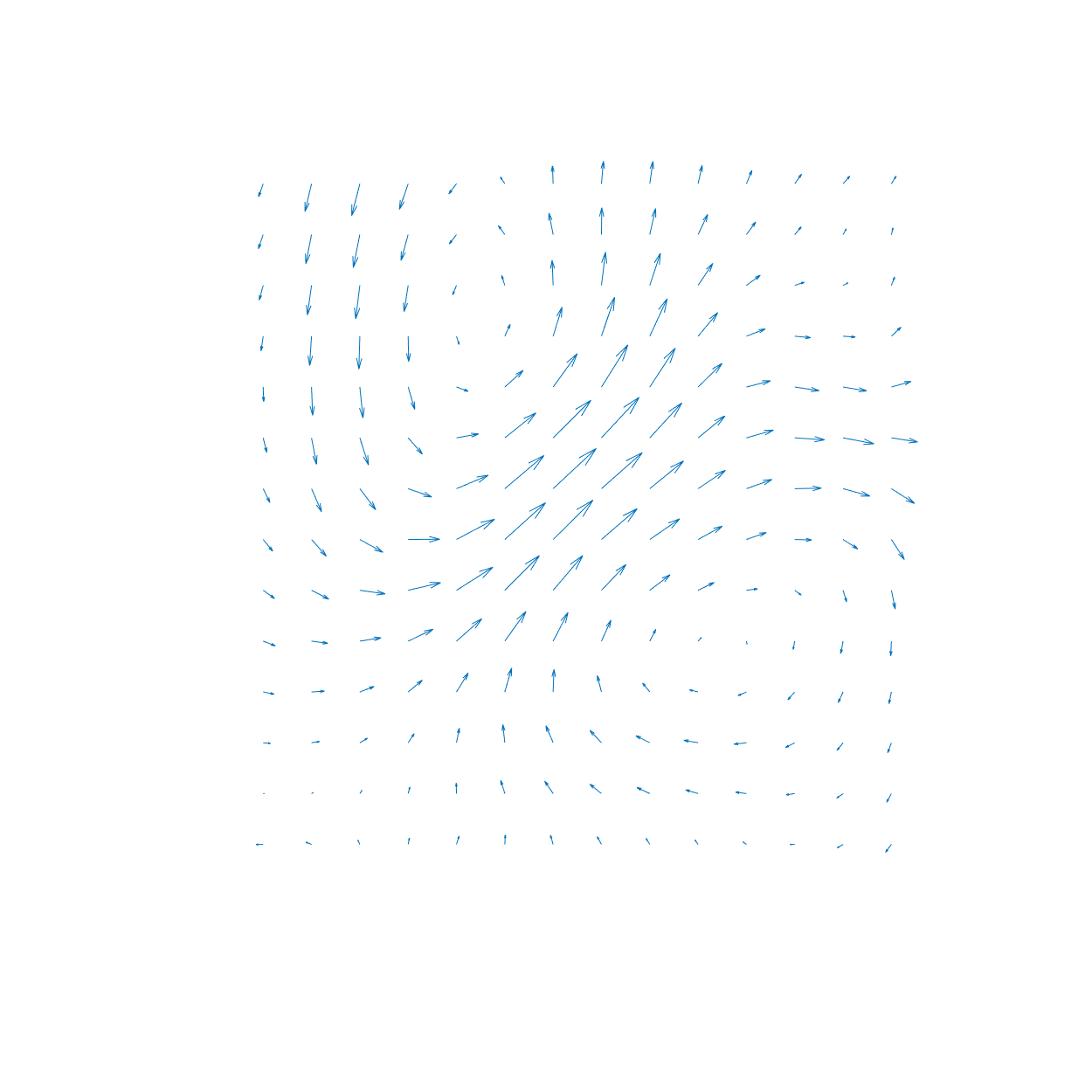

Supplement: S2 MCG raw data 2 — The raw MCG dataset includes categories 0-3 for training and validation. (ZIP) [file pone.0338189.s002.zip › train/0/p4_385_2.png]

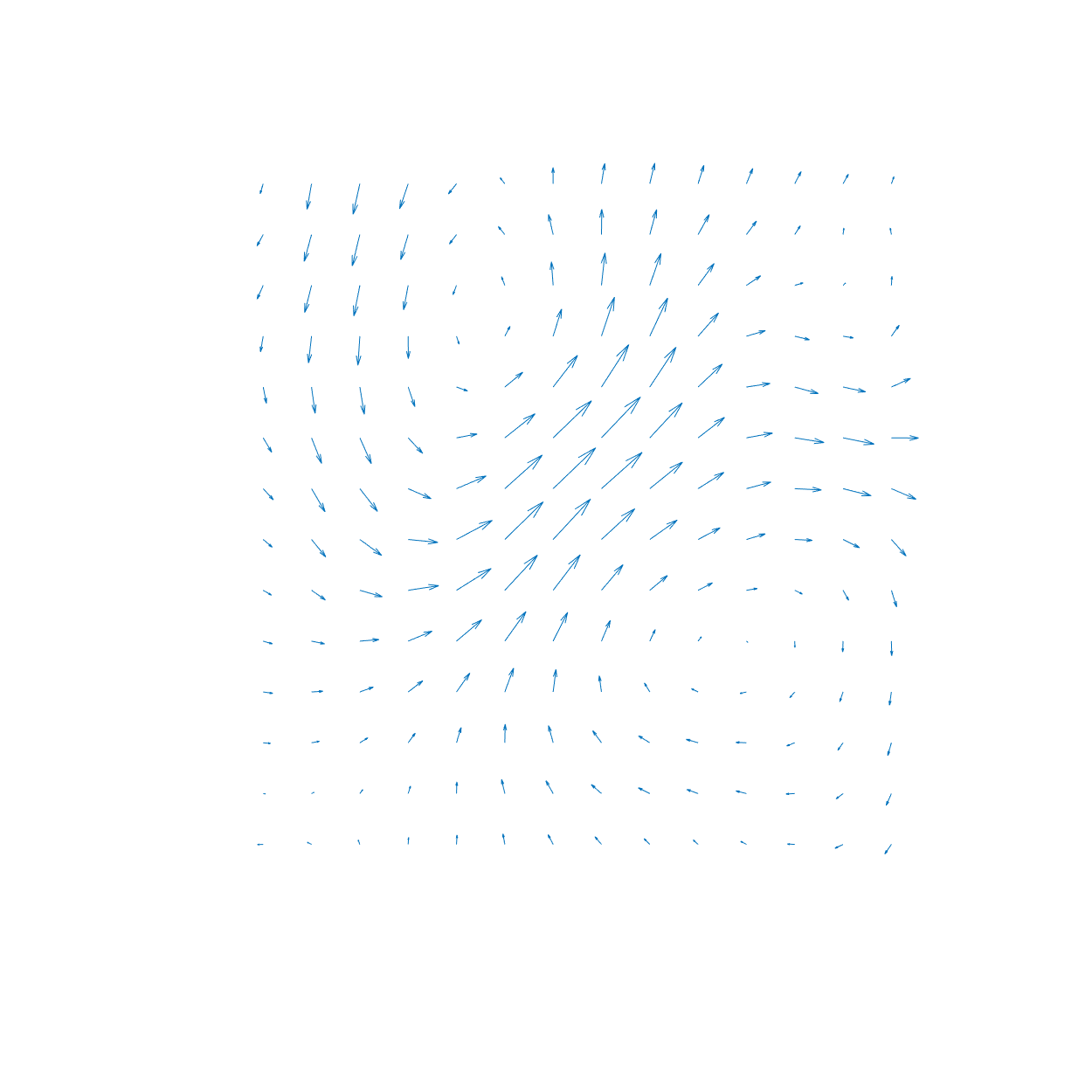

Supplement: S2 MCG raw data 2 — The raw MCG dataset includes categories 0-3 for training and validation. (ZIP) [file pone.0338189.s002.zip › train/0/p4_385_3.png]

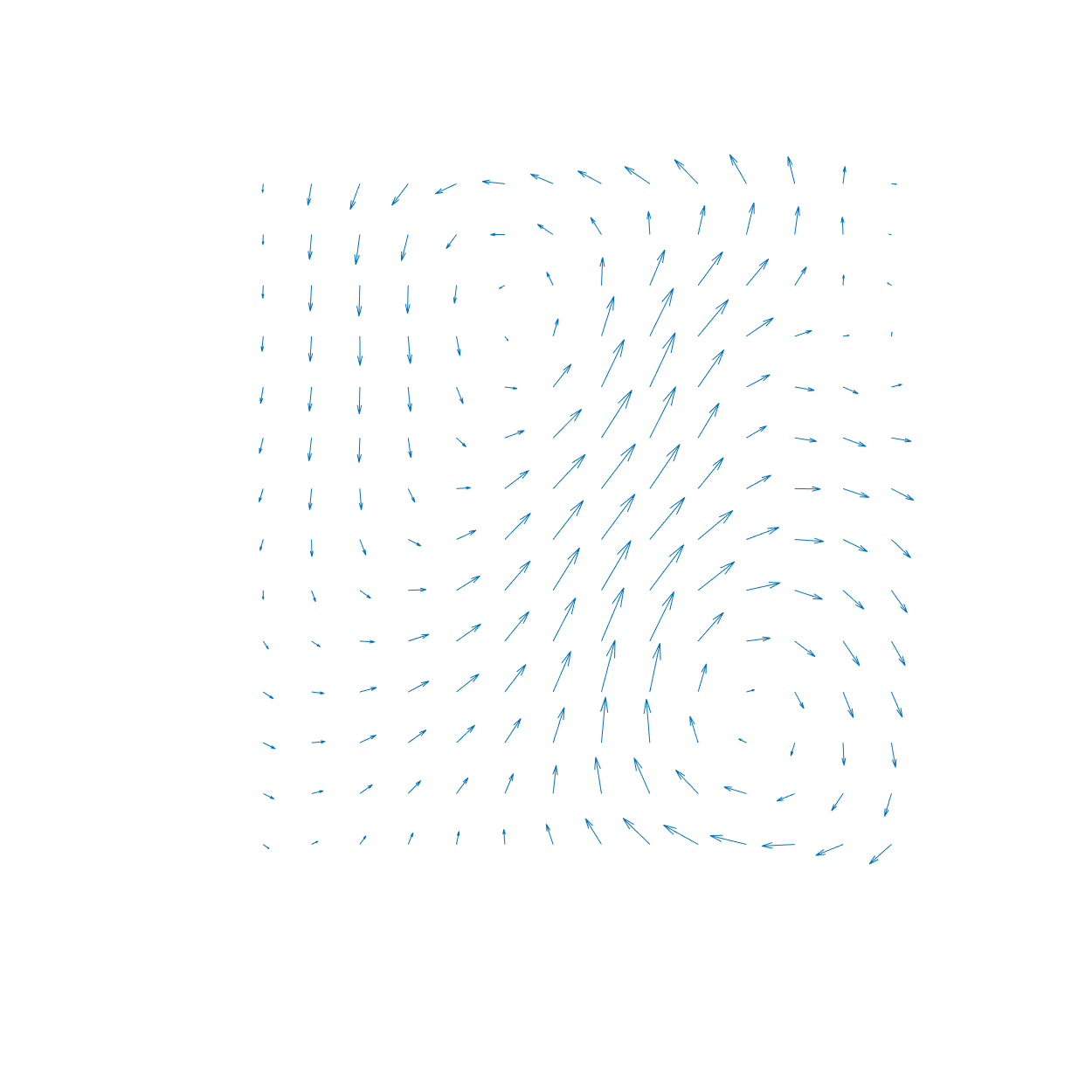

Supplement: S2 MCG raw data 2 — The raw MCG dataset includes categories 0-3 for training and validation. (ZIP) [file pone.0338189.s002.zip › train/0/p5_385_1.png]

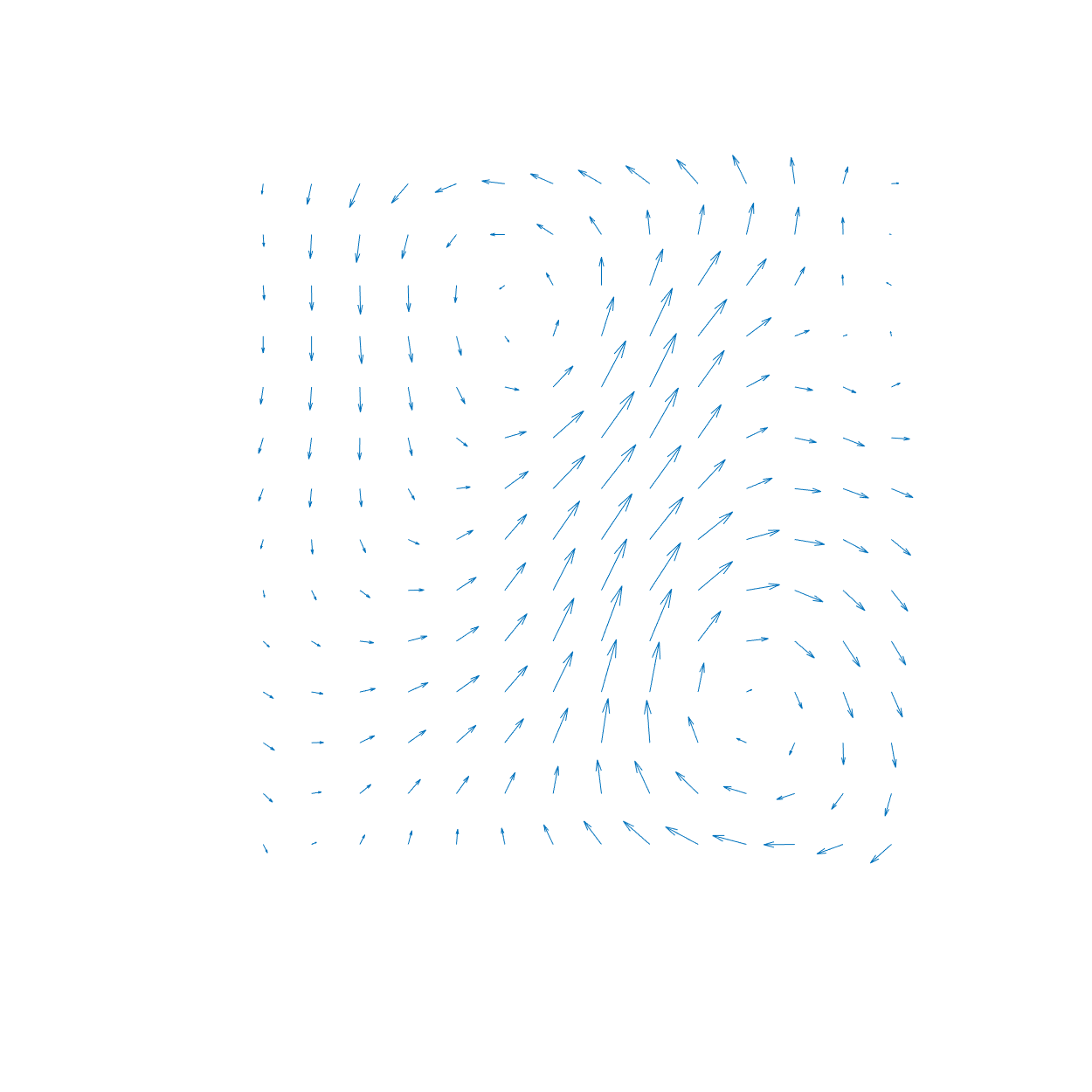

Supplement: S2 MCG raw data 2 — The raw MCG dataset includes categories 0-3 for training and validation. (ZIP) [file pone.0338189.s002.zip › train/0/p5_385_2.png]

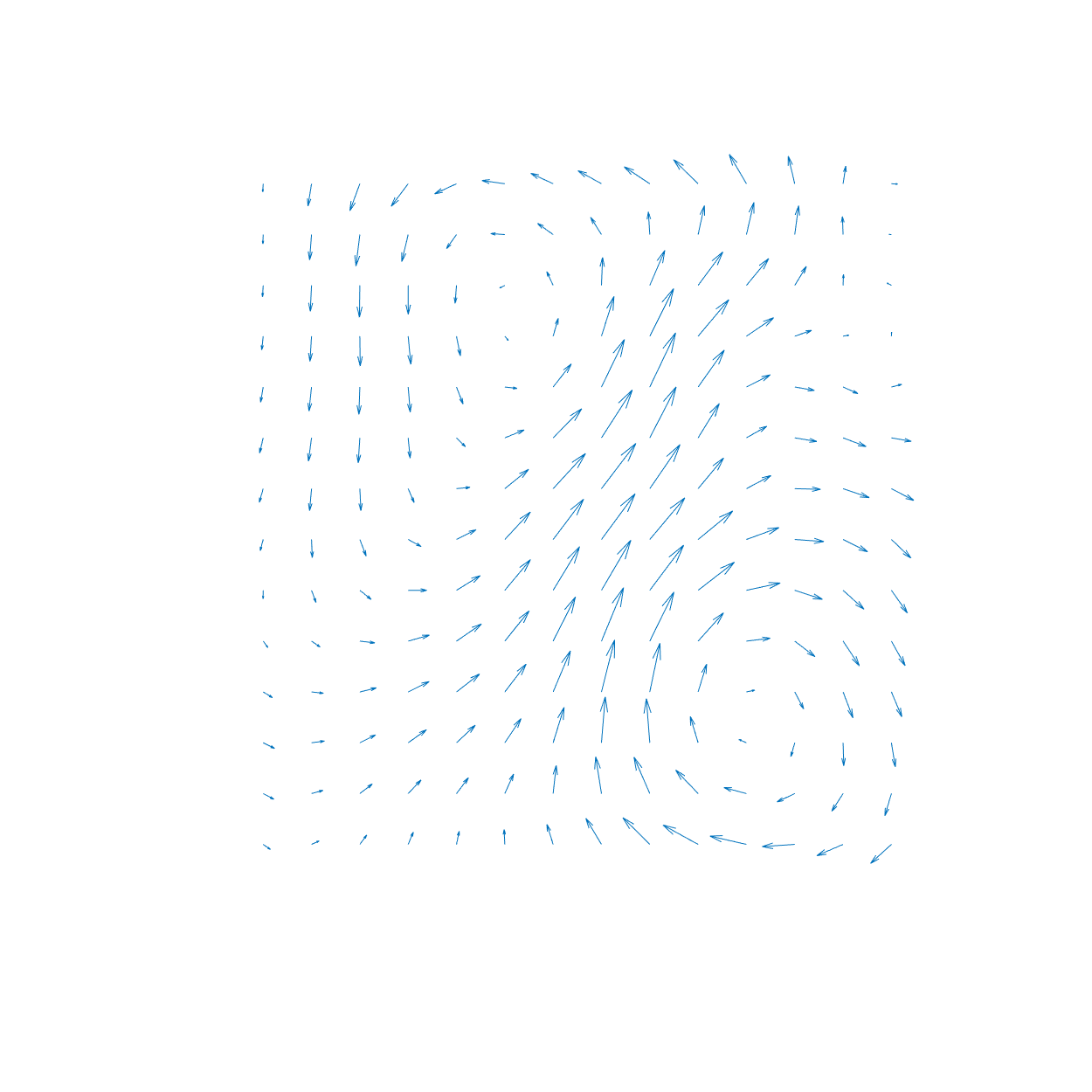

Supplement: S2 MCG raw data 2 — The raw MCG dataset includes categories 0-3 for training and validation. (ZIP) [file pone.0338189.s002.zip › train/0/p5_385_3.png]

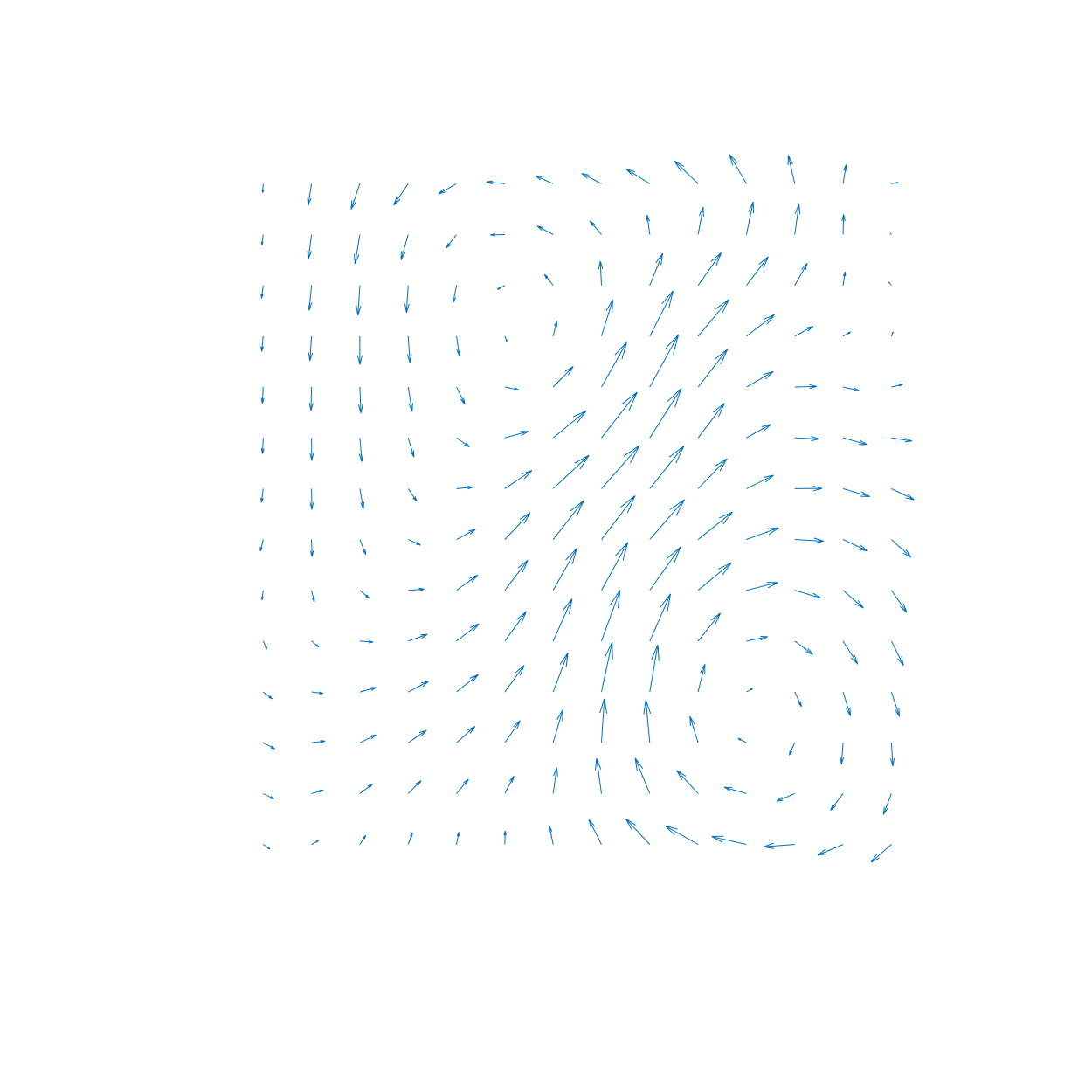

Supplement: S2 MCG raw data 2 — The raw MCG dataset includes categories 0-3 for training and validation. (ZIP) [file pone.0338189.s002.zip › train/0/p5_390_1.png]

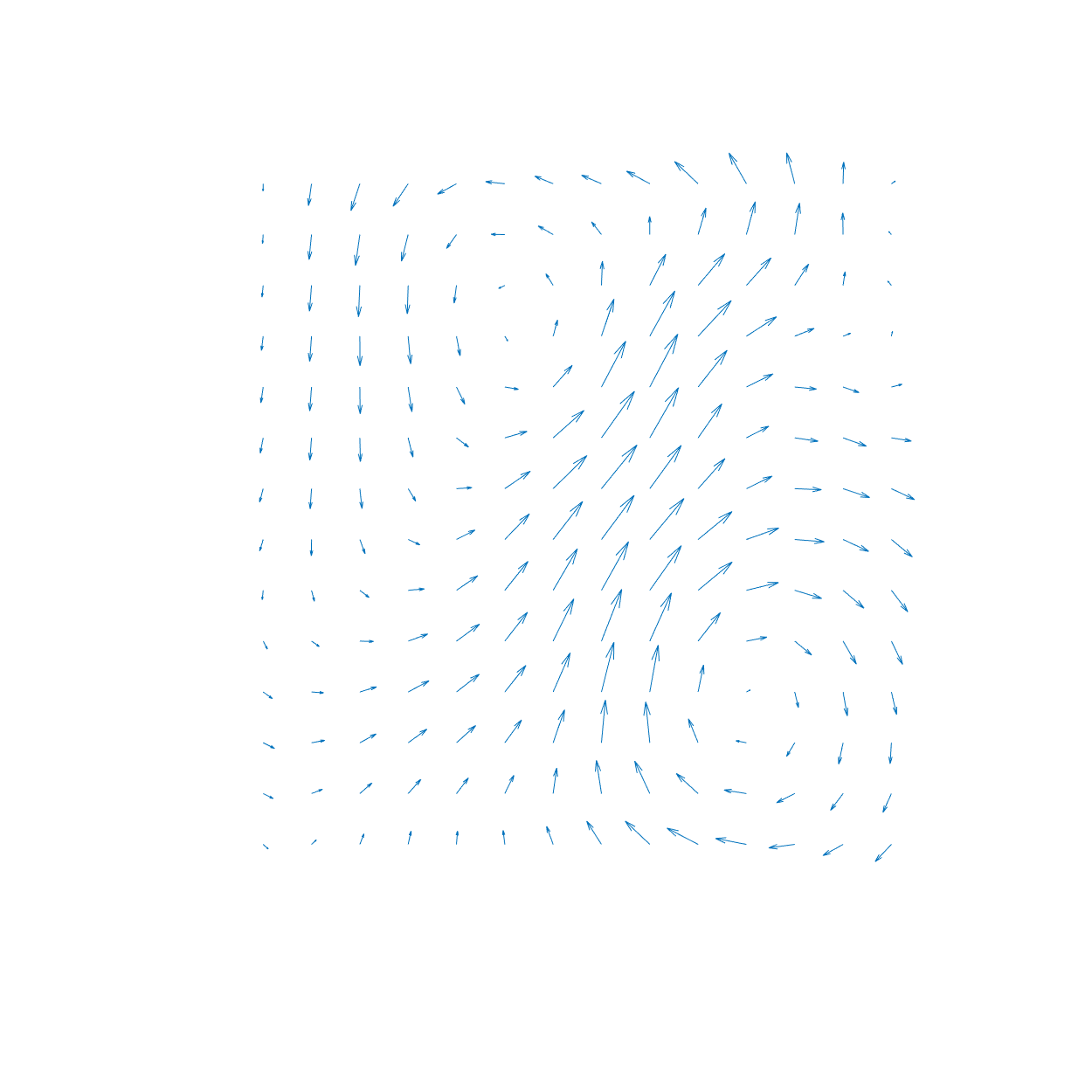

Supplement: S2 MCG raw data 2 — The raw MCG dataset includes categories 0-3 for training and validation. (ZIP) [file pone.0338189.s002.zip › train/0/p5_390_2.png]

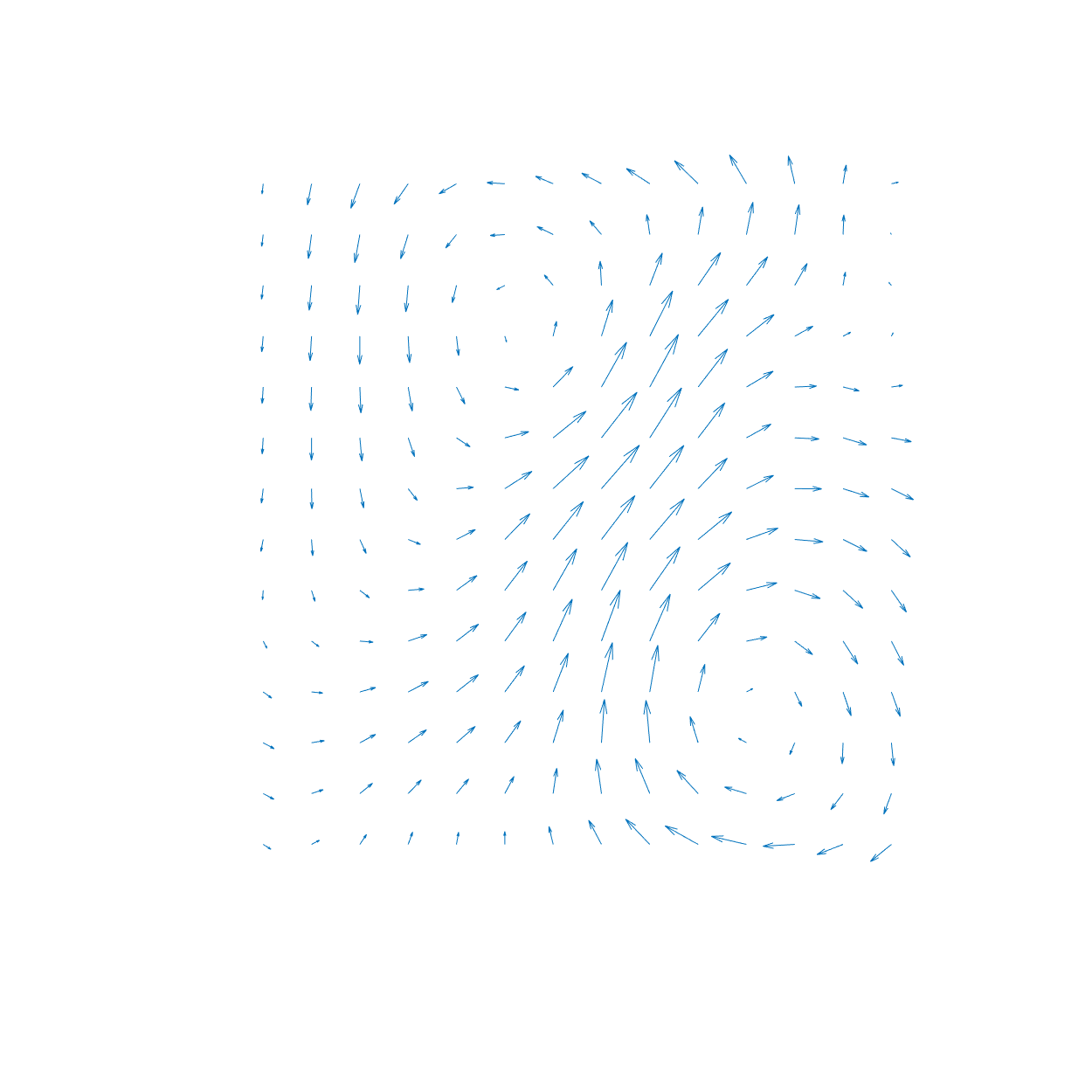

Supplement: S2 MCG raw data 2 — The raw MCG dataset includes categories 0-3 for training and validation. (ZIP) [file pone.0338189.s002.zip › train/0/p5_390_3.png]

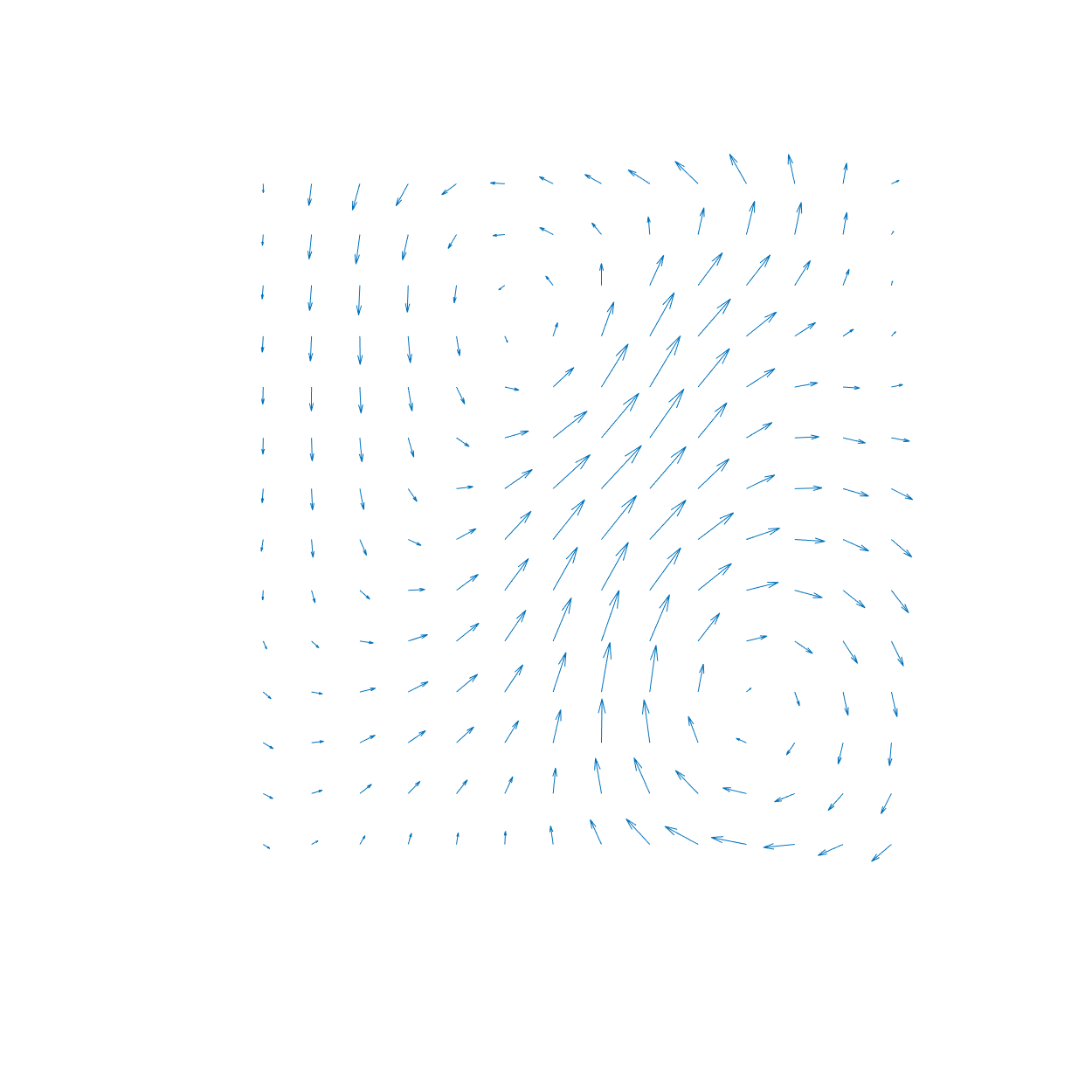

Supplement: S2 MCG raw data 2 — The raw MCG dataset includes categories 0-3 for training and validation. (ZIP) [file pone.0338189.s002.zip › train/0/p5_395_1.png]

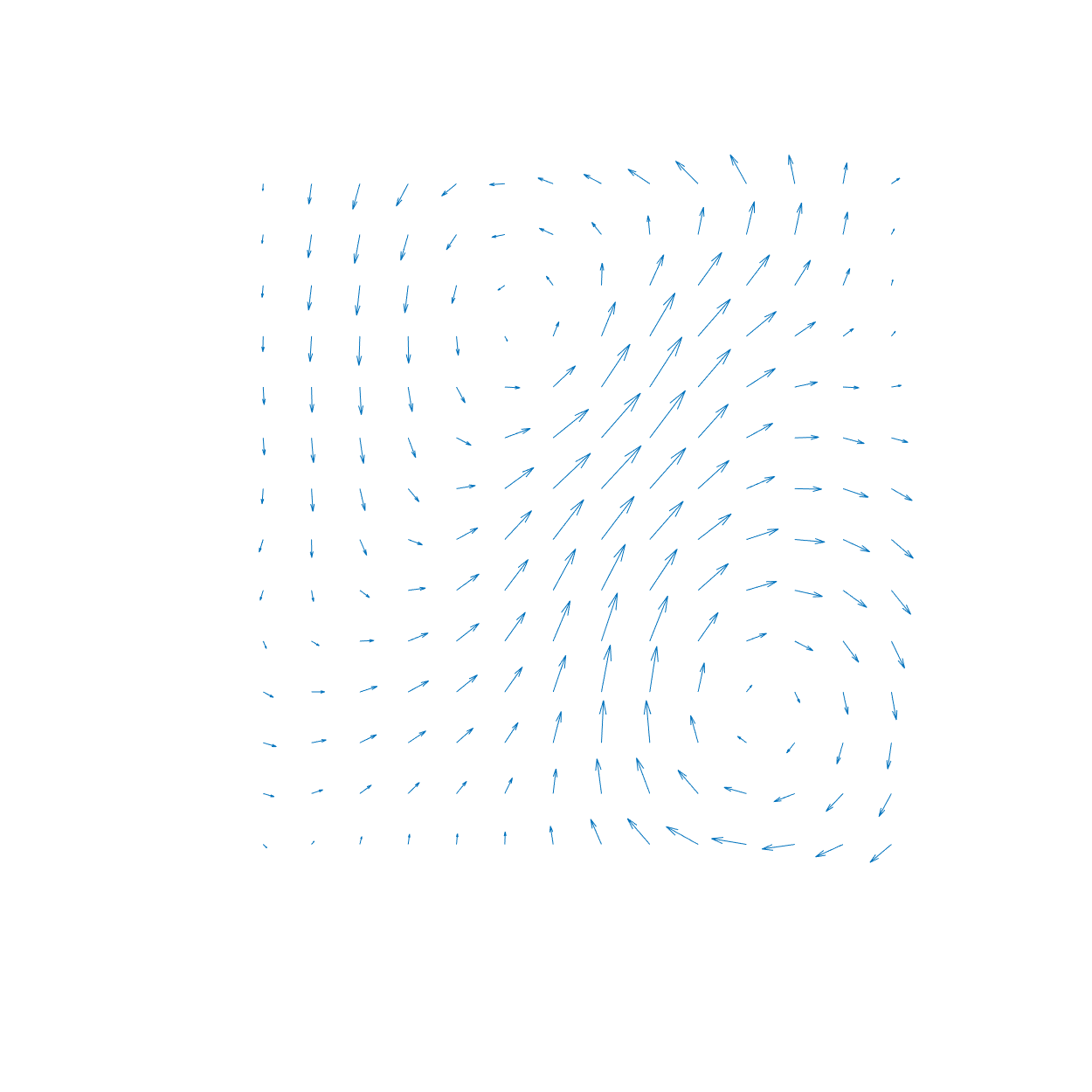

Supplement: S2 MCG raw data 2 — The raw MCG dataset includes categories 0-3 for training and validation. (ZIP) [file pone.0338189.s002.zip › train/0/p5_395_2.png]

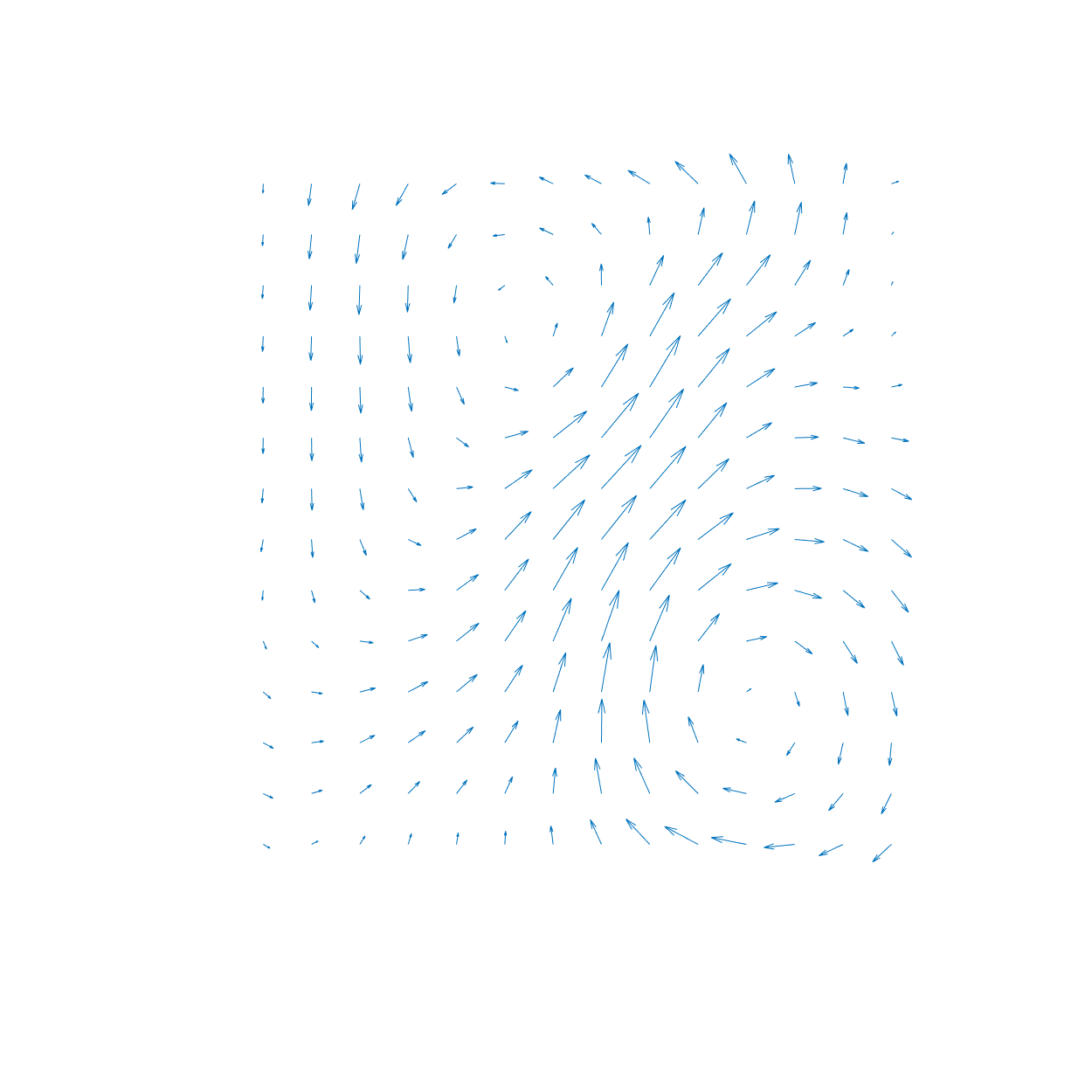

Supplement: S2 MCG raw data 2 — The raw MCG dataset includes categories 0-3 for training and validation. (ZIP) [file pone.0338189.s002.zip › train/0/p5_395_3.png]

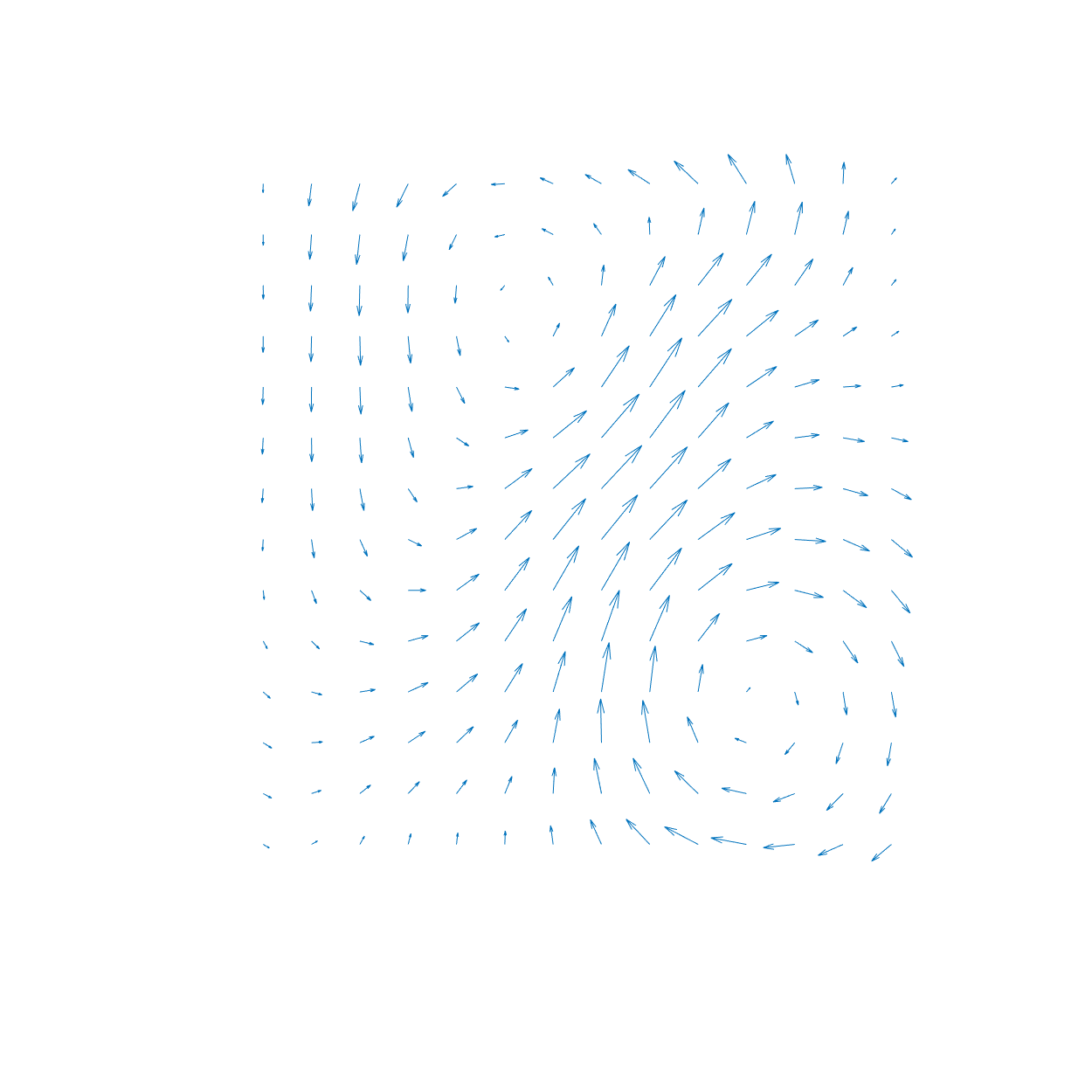

Supplement: S2 MCG raw data 2 — The raw MCG dataset includes categories 0-3 for training and validation. (ZIP) [file pone.0338189.s002.zip › train/0/p5_400_1.png]

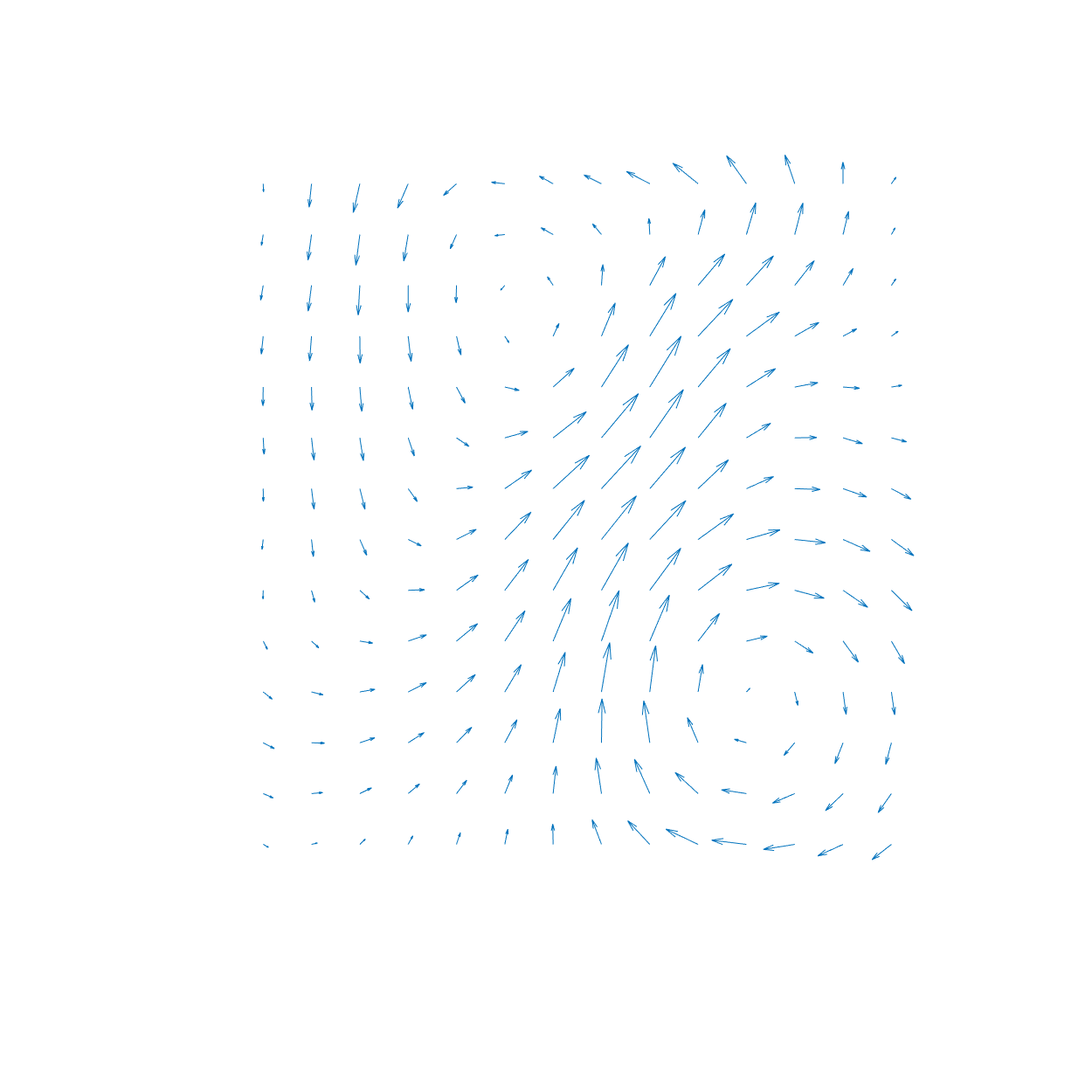

Supplement: S2 MCG raw data 2 — The raw MCG dataset includes categories 0-3 for training and validation. (ZIP) [file pone.0338189.s002.zip › train/0/p5_400_2.png]

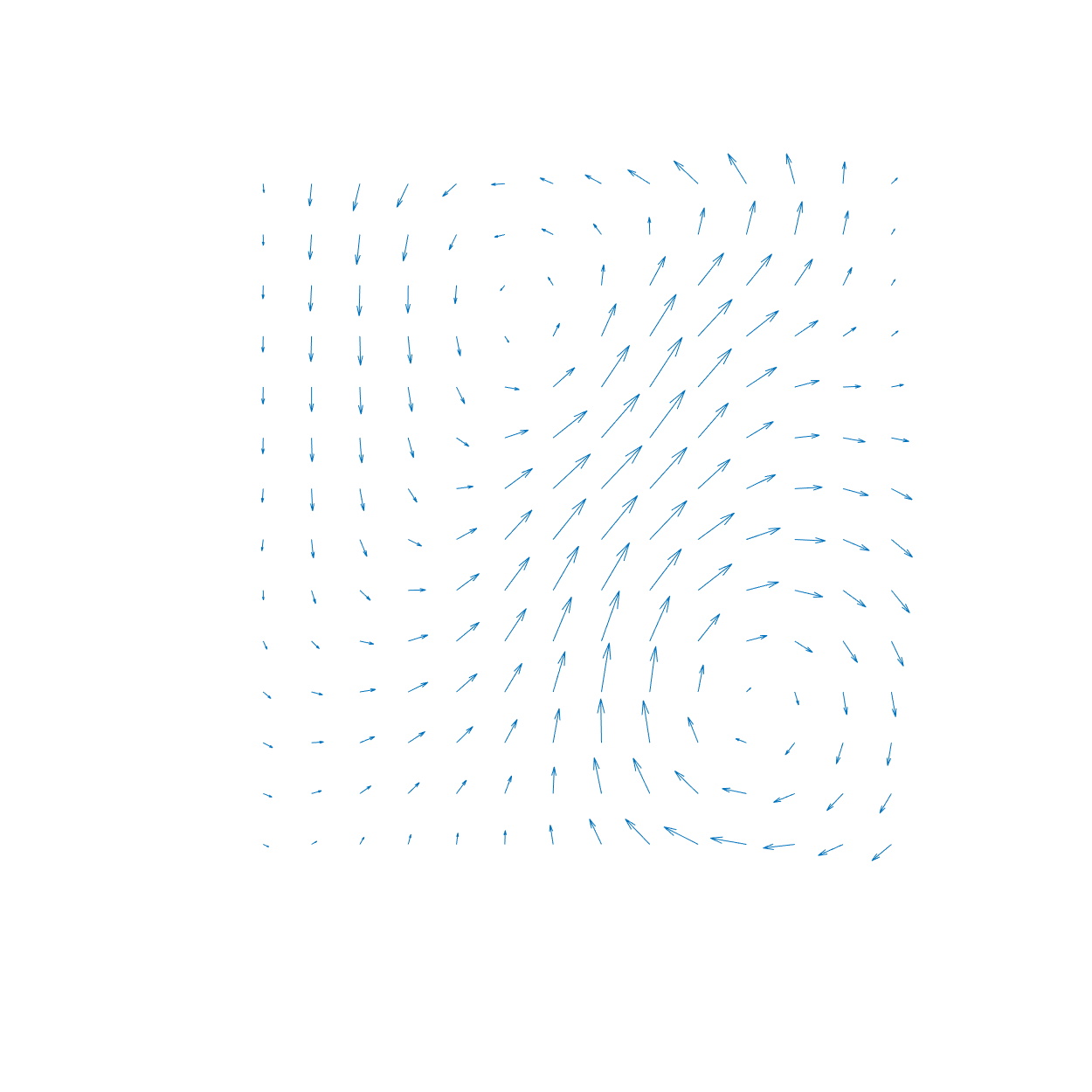

Supplement: S2 MCG raw data 2 — The raw MCG dataset includes categories 0-3 for training and validation. (ZIP) [file pone.0338189.s002.zip › train/0/p5_400_3.png]

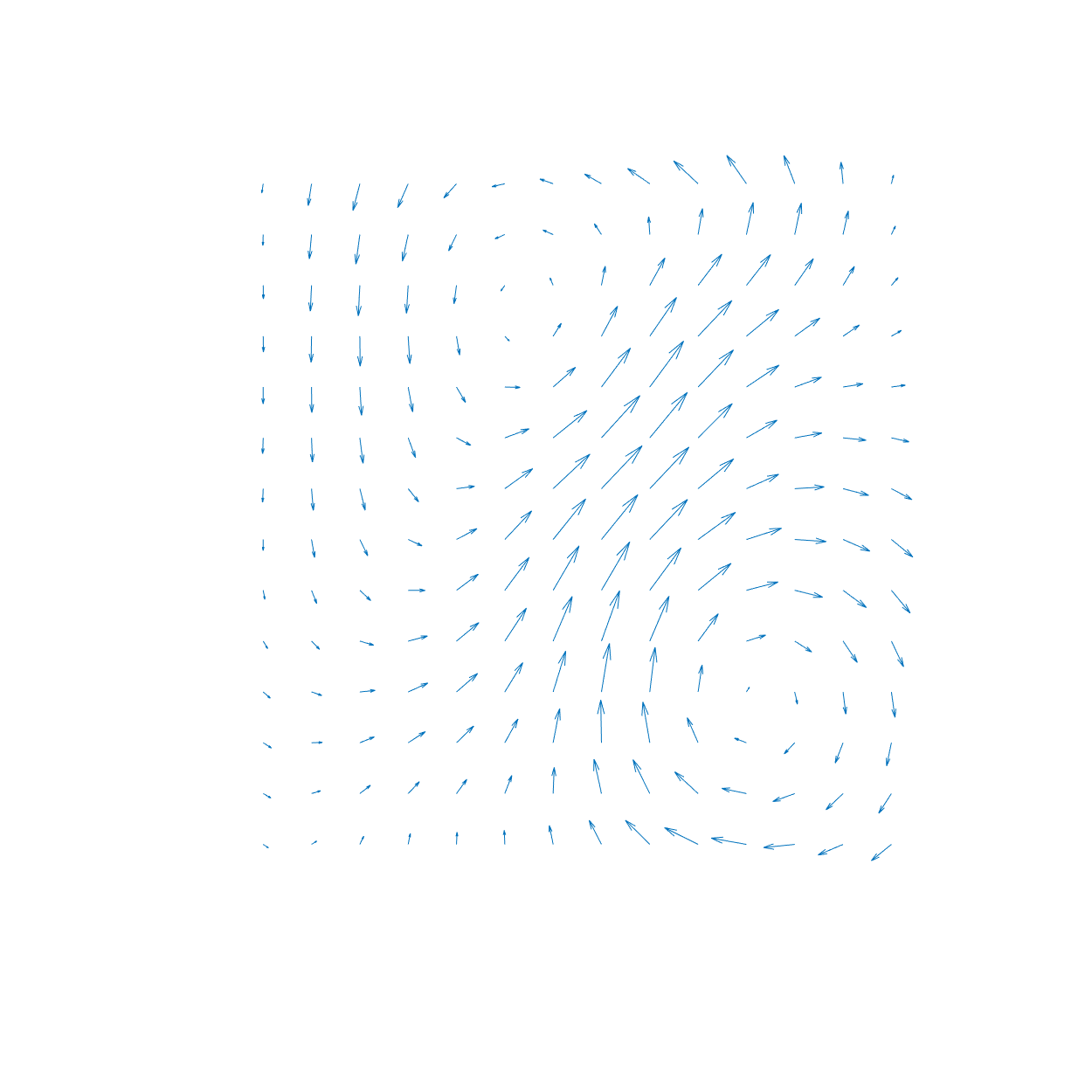

Supplement: S2 MCG raw data 2 — The raw MCG dataset includes categories 0-3 for training and validation. (ZIP) [file pone.0338189.s002.zip › train/0/p5_405_1.png]

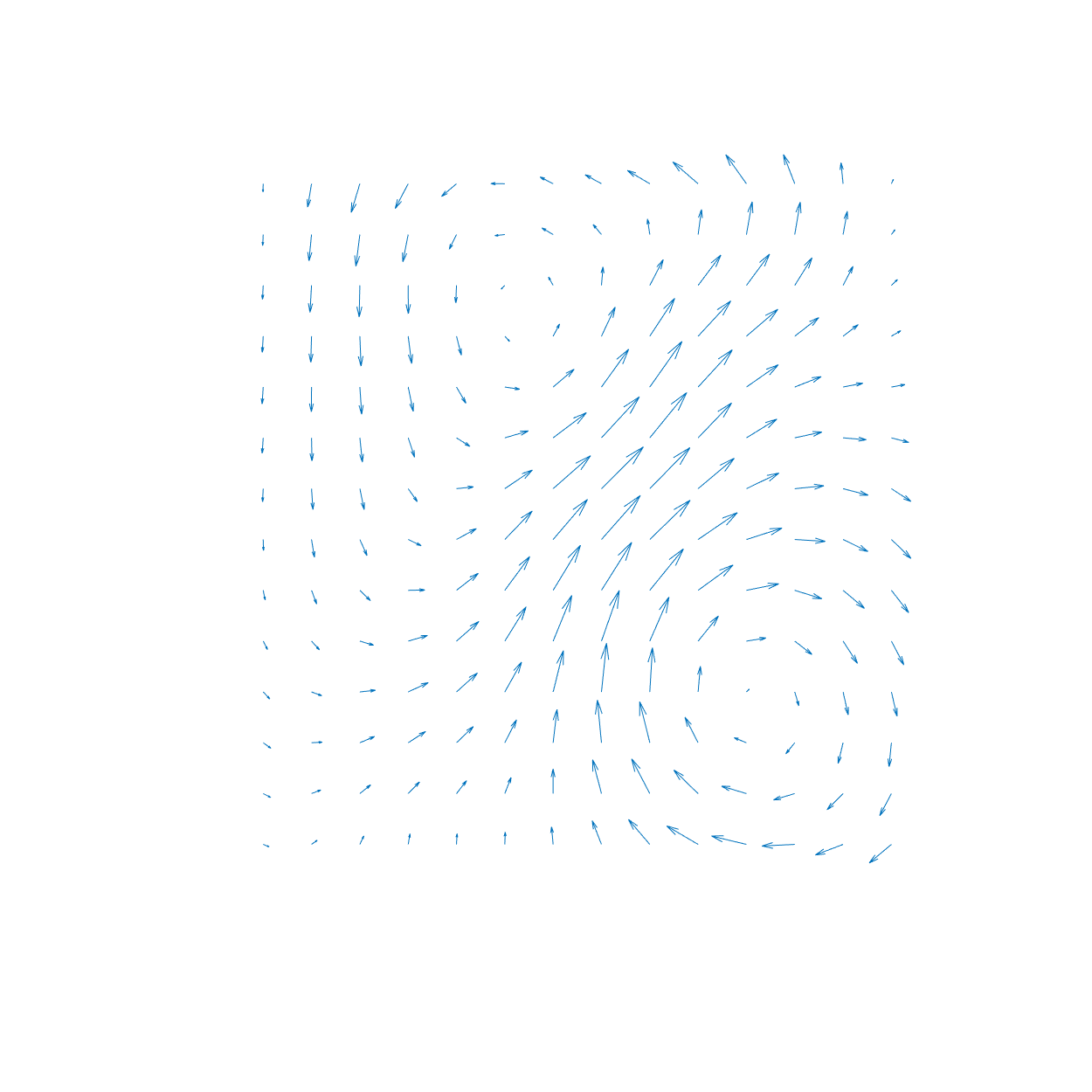

Supplement: S2 MCG raw data 2 — The raw MCG dataset includes categories 0-3 for training and validation. (ZIP) [file pone.0338189.s002.zip › train/0/p5_405_2.png]

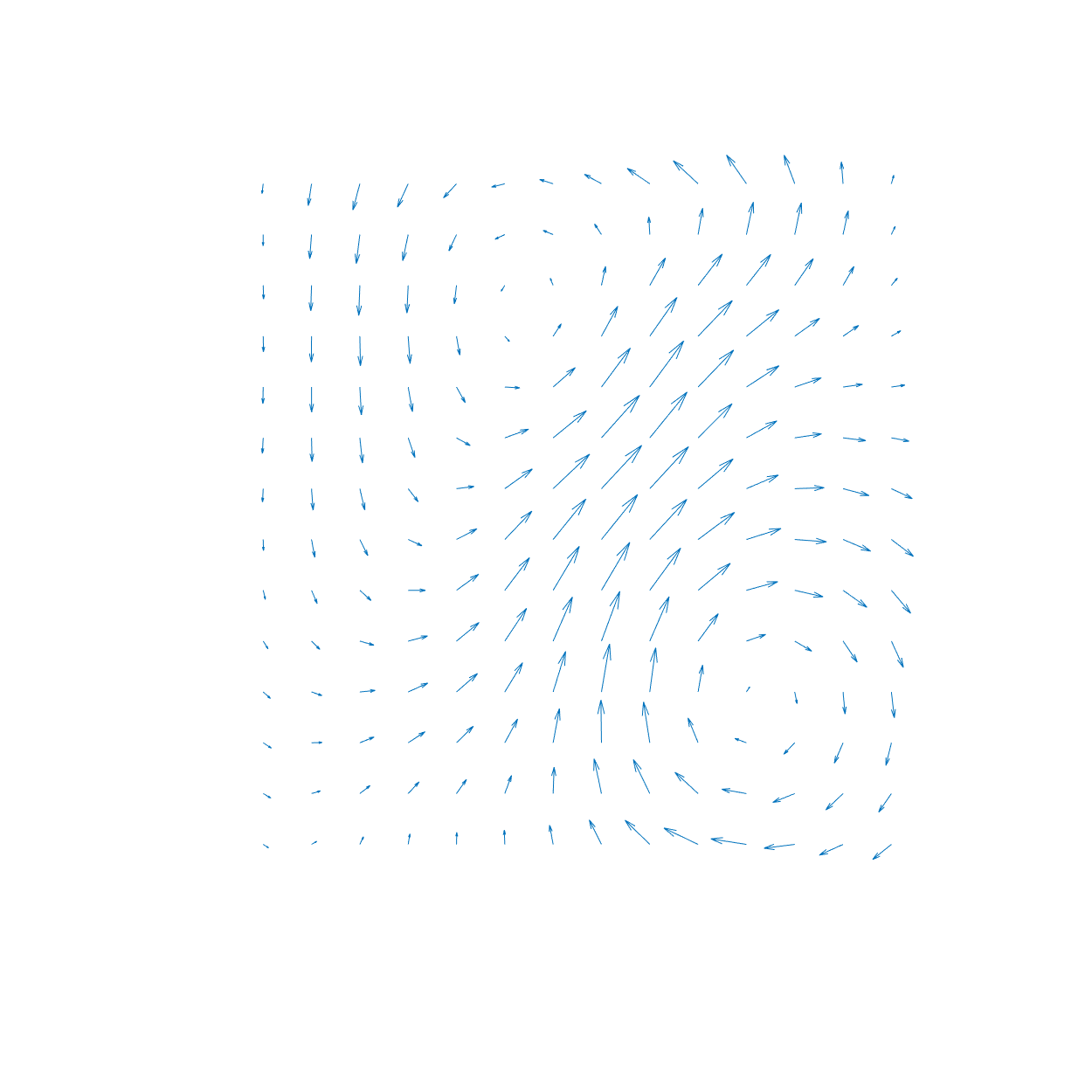

Supplement: S2 MCG raw data 2 — The raw MCG dataset includes categories 0-3 for training and validation. (ZIP) [file pone.0338189.s002.zip › train/0/p5_405_3.png]

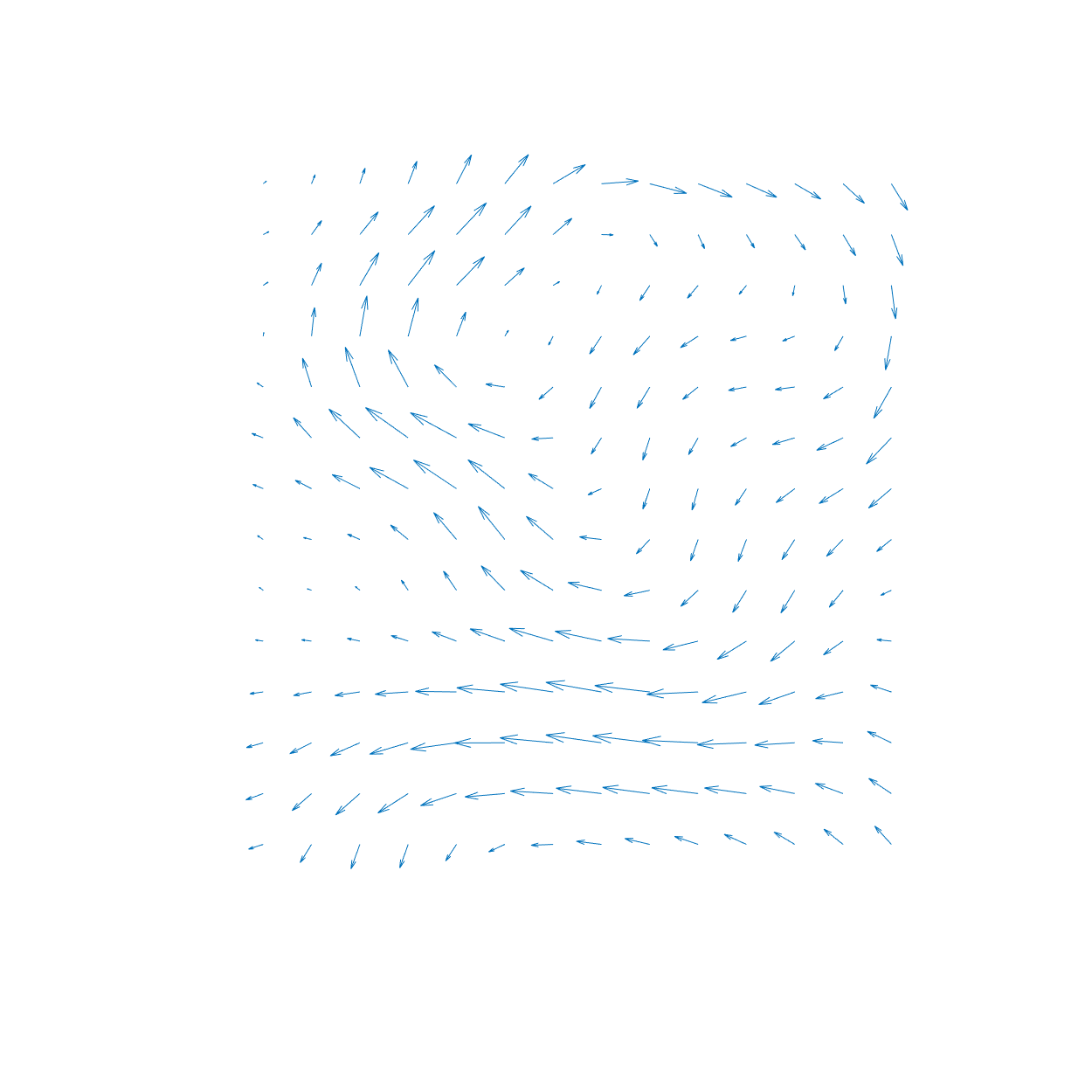

Supplement: S3 MCG raw data 3 — The raw MCG dataset includes category 4 for training and validation. (ZIP) [file pone.0338189.s003.zip › train/4/p10_270_1.png]

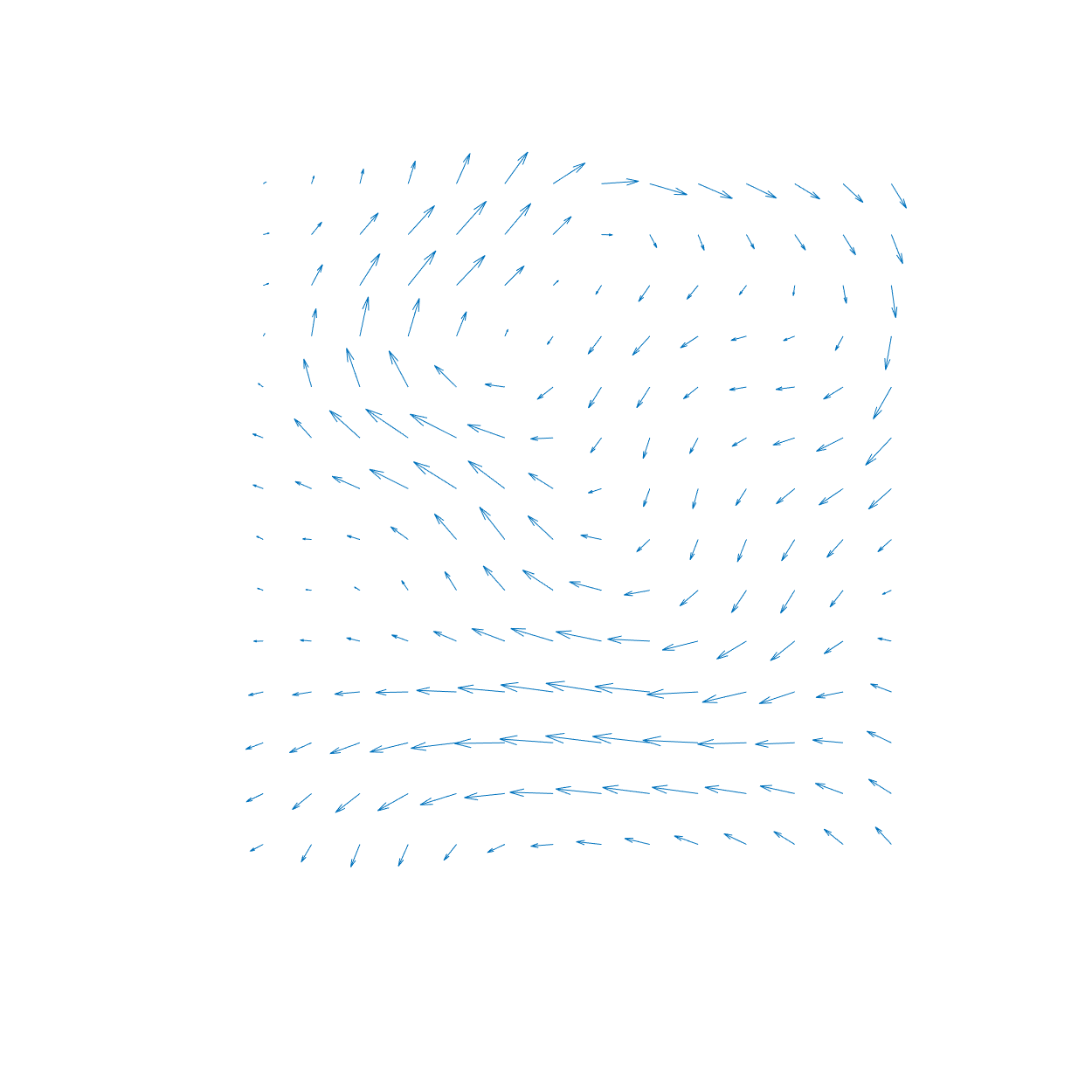

Supplement: S3 MCG raw data 3 — The raw MCG dataset includes category 4 for training and validation. (ZIP) [file pone.0338189.s003.zip › train/4/p10_270_2.png]

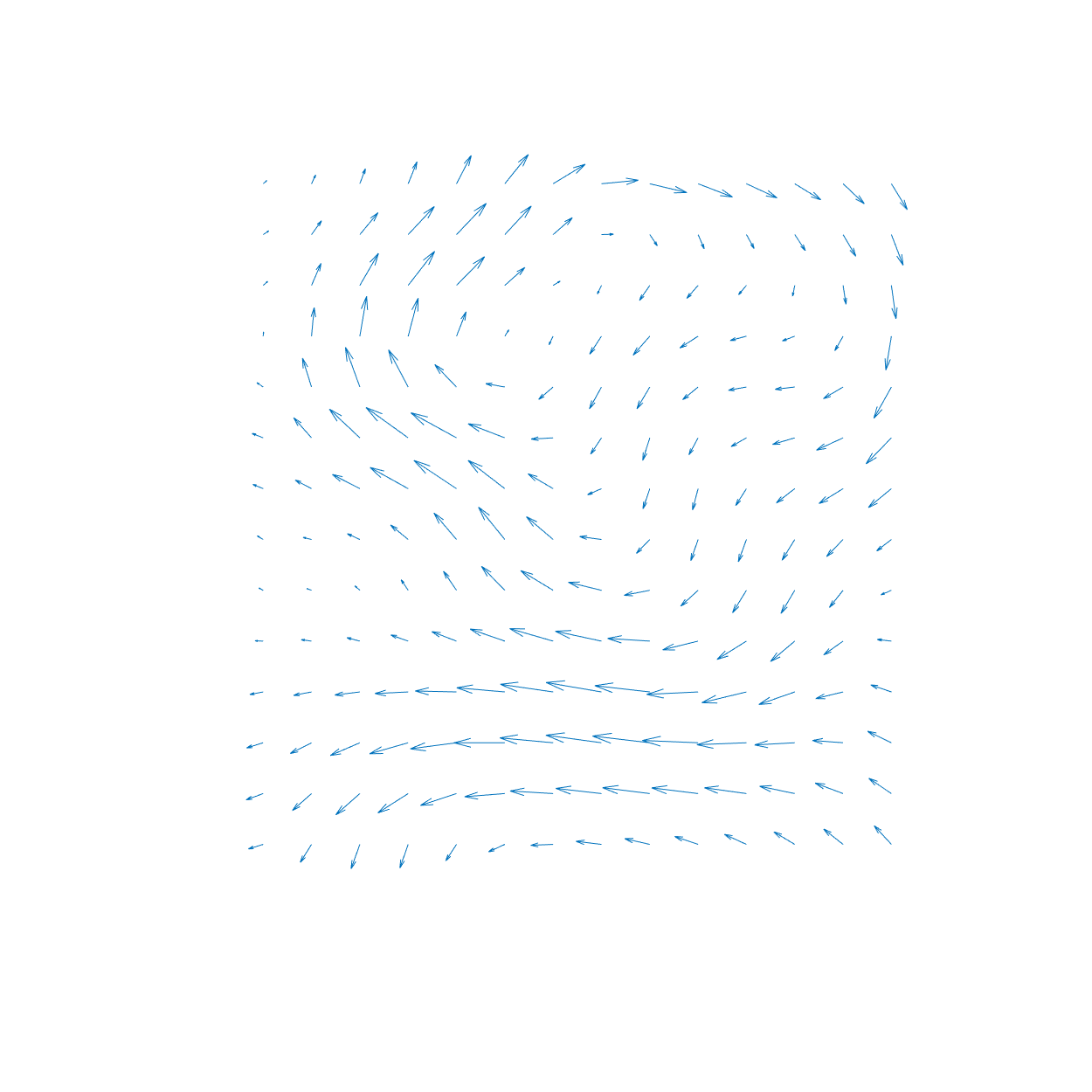

Supplement: S3 MCG raw data 3 — The raw MCG dataset includes category 4 for training and validation. (ZIP) [file pone.0338189.s003.zip › train/4/p10_270_3.png]

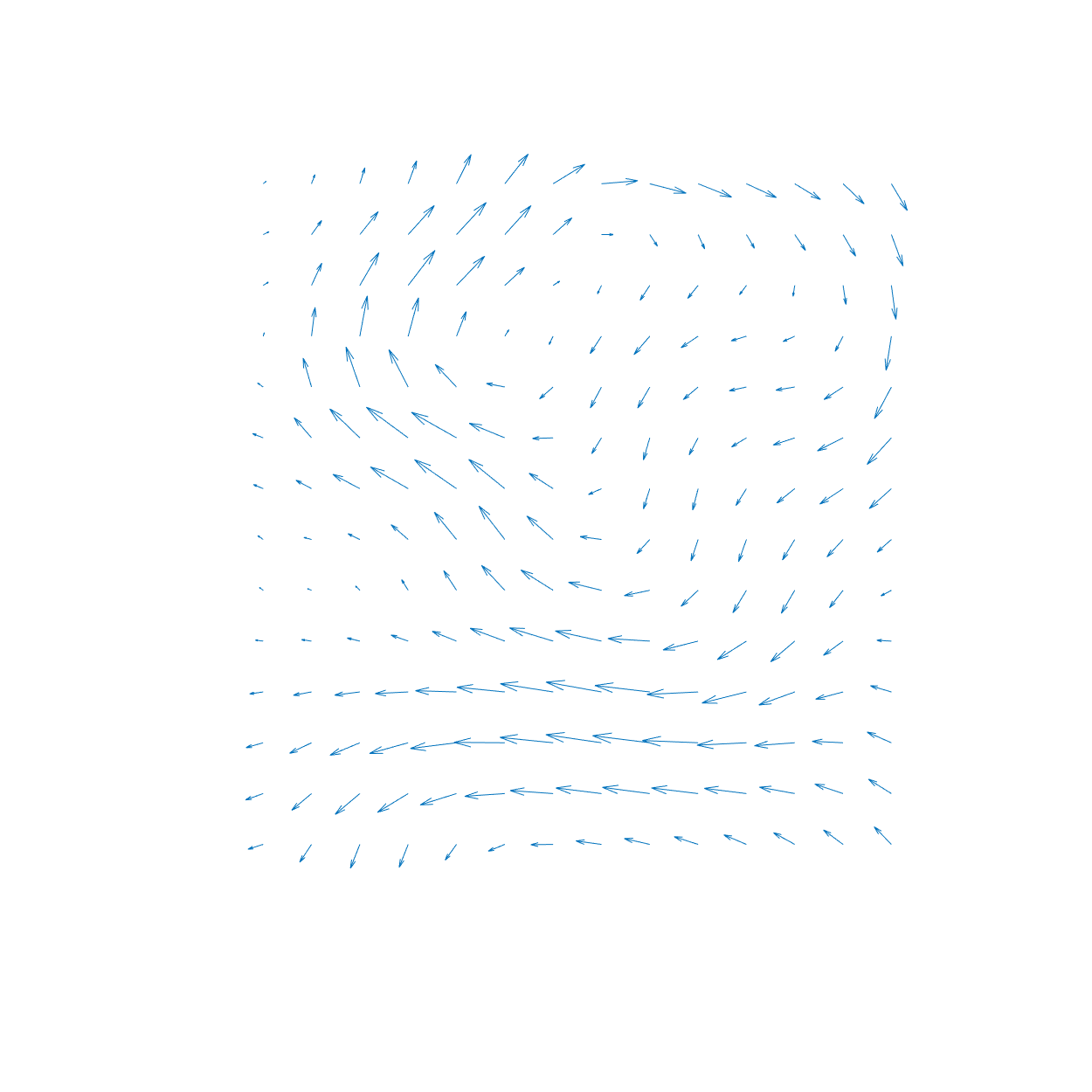

Supplement: S3 MCG raw data 3 — The raw MCG dataset includes category 4 for training and validation. (ZIP) [file pone.0338189.s003.zip › train/4/p10_270_4.png]

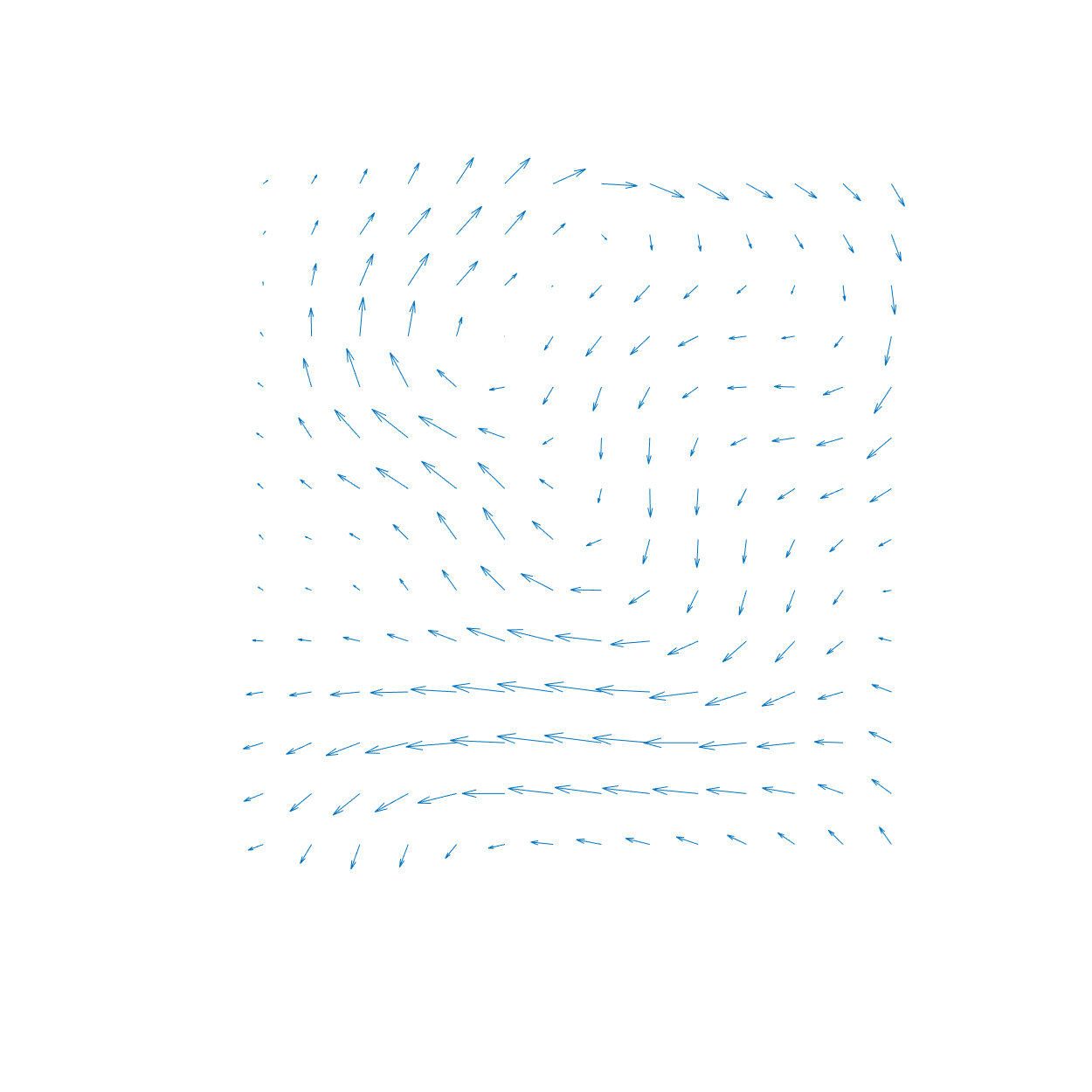

Supplement: S3 MCG raw data 3 — The raw MCG dataset includes category 4 for training and validation. (ZIP) [file pone.0338189.s003.zip › train/4/p10_275_1.png]

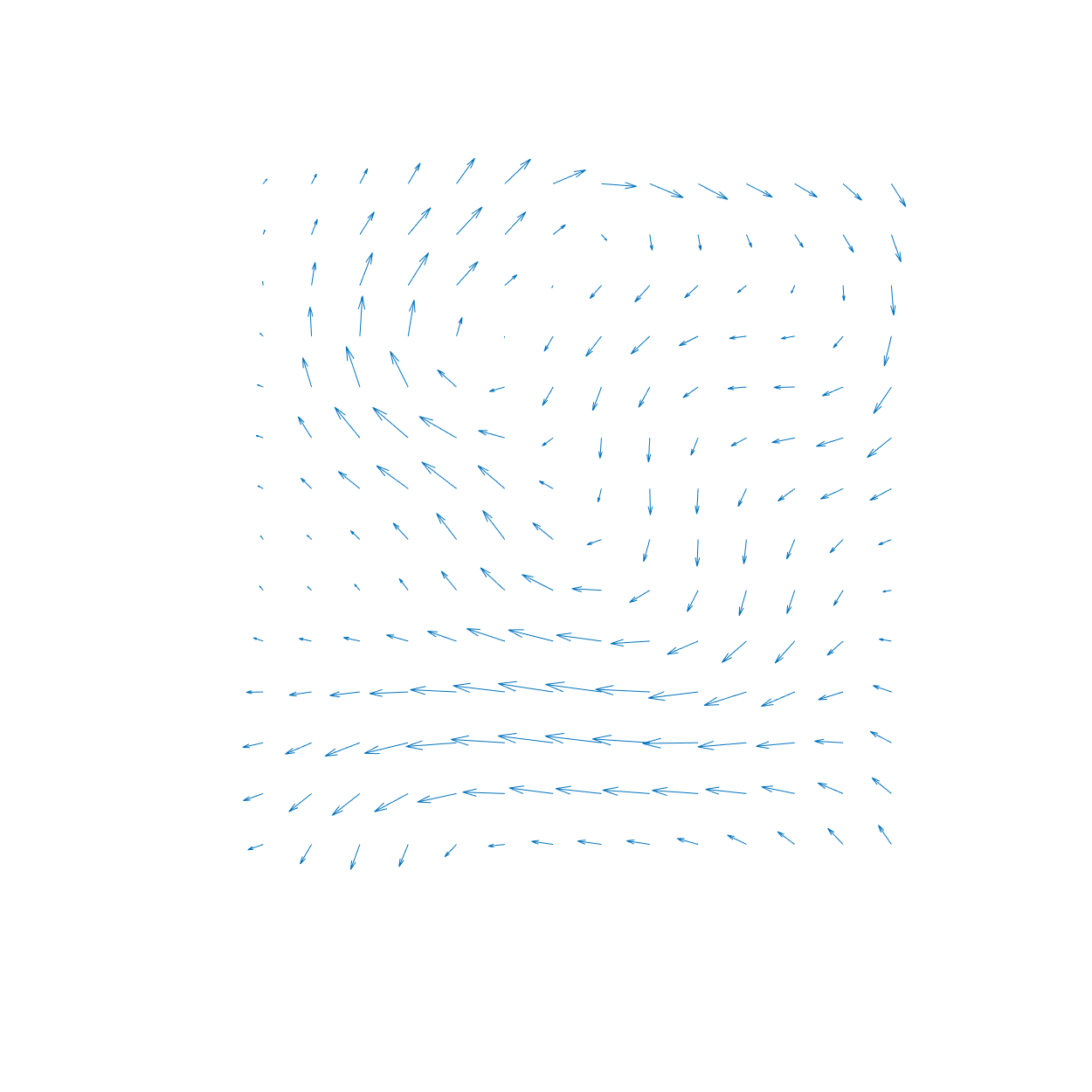

Supplement: S3 MCG raw data 3 — The raw MCG dataset includes category 4 for training and validation. (ZIP) [file pone.0338189.s003.zip › train/4/p10_275_2.png]

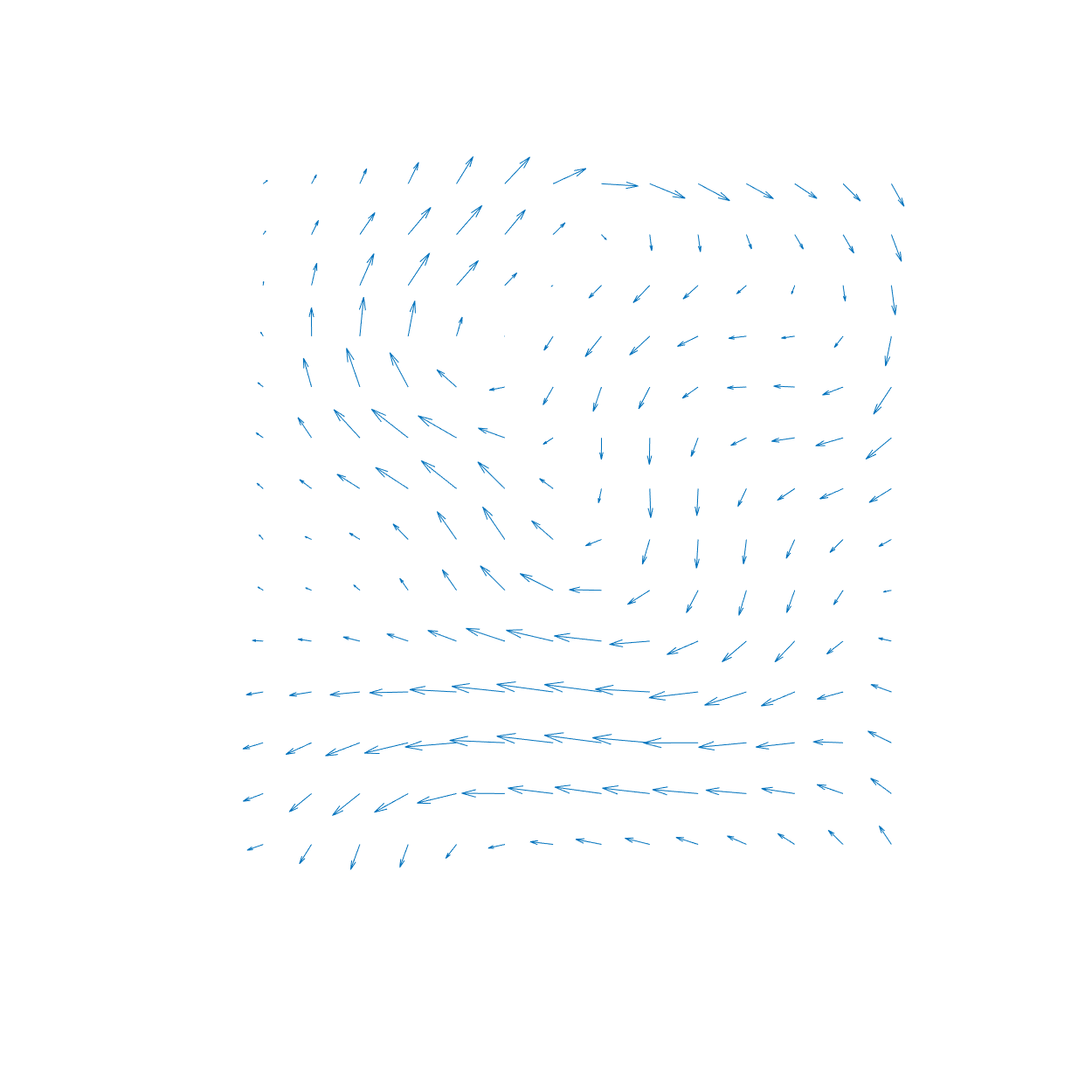

Supplement: S3 MCG raw data 3 — The raw MCG dataset includes category 4 for training and validation. (ZIP) [file pone.0338189.s003.zip › train/4/p10_275_3.png]

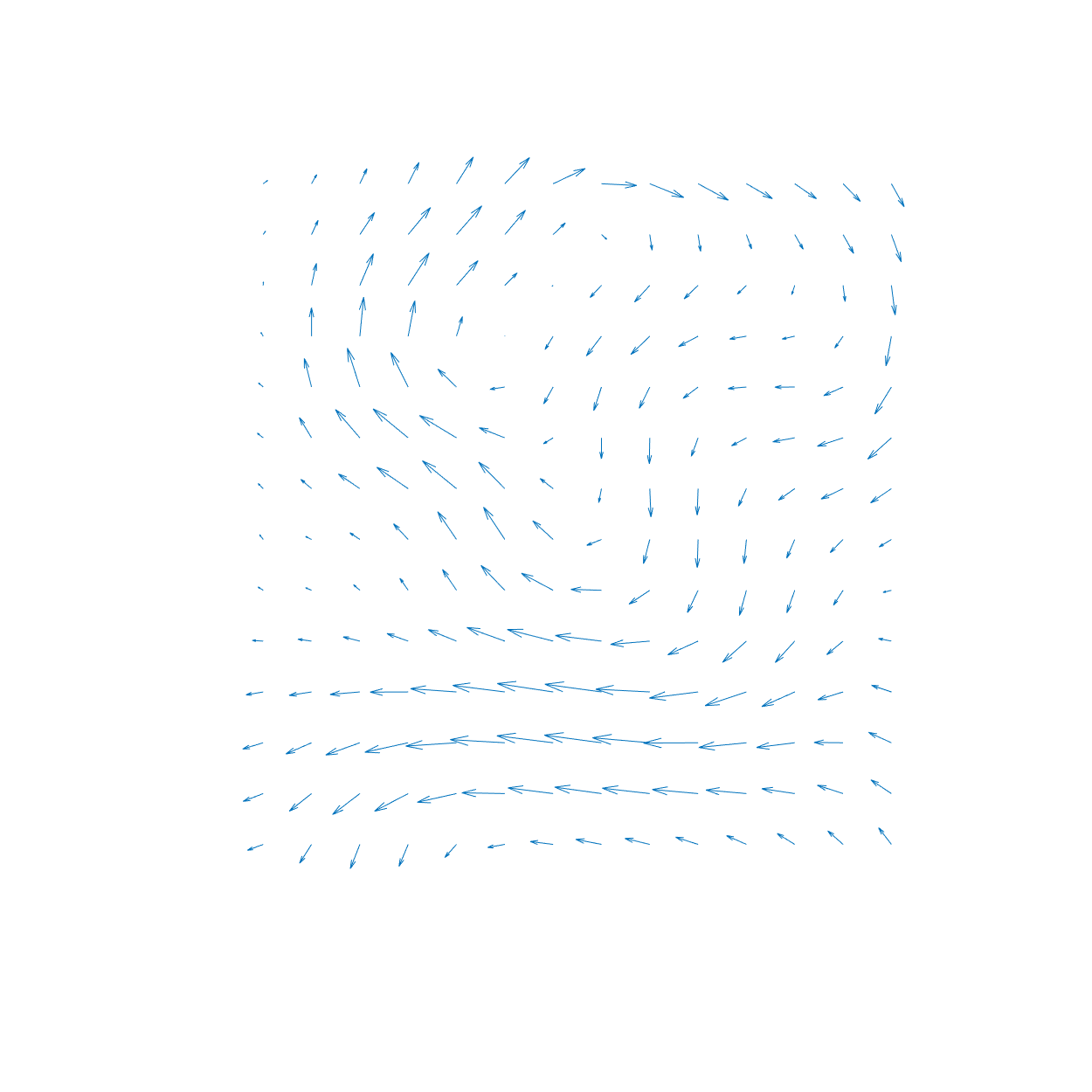

Supplement: S3 MCG raw data 3 — The raw MCG dataset includes category 4 for training and validation. (ZIP) [file pone.0338189.s003.zip › train/4/p10_275_4.png]

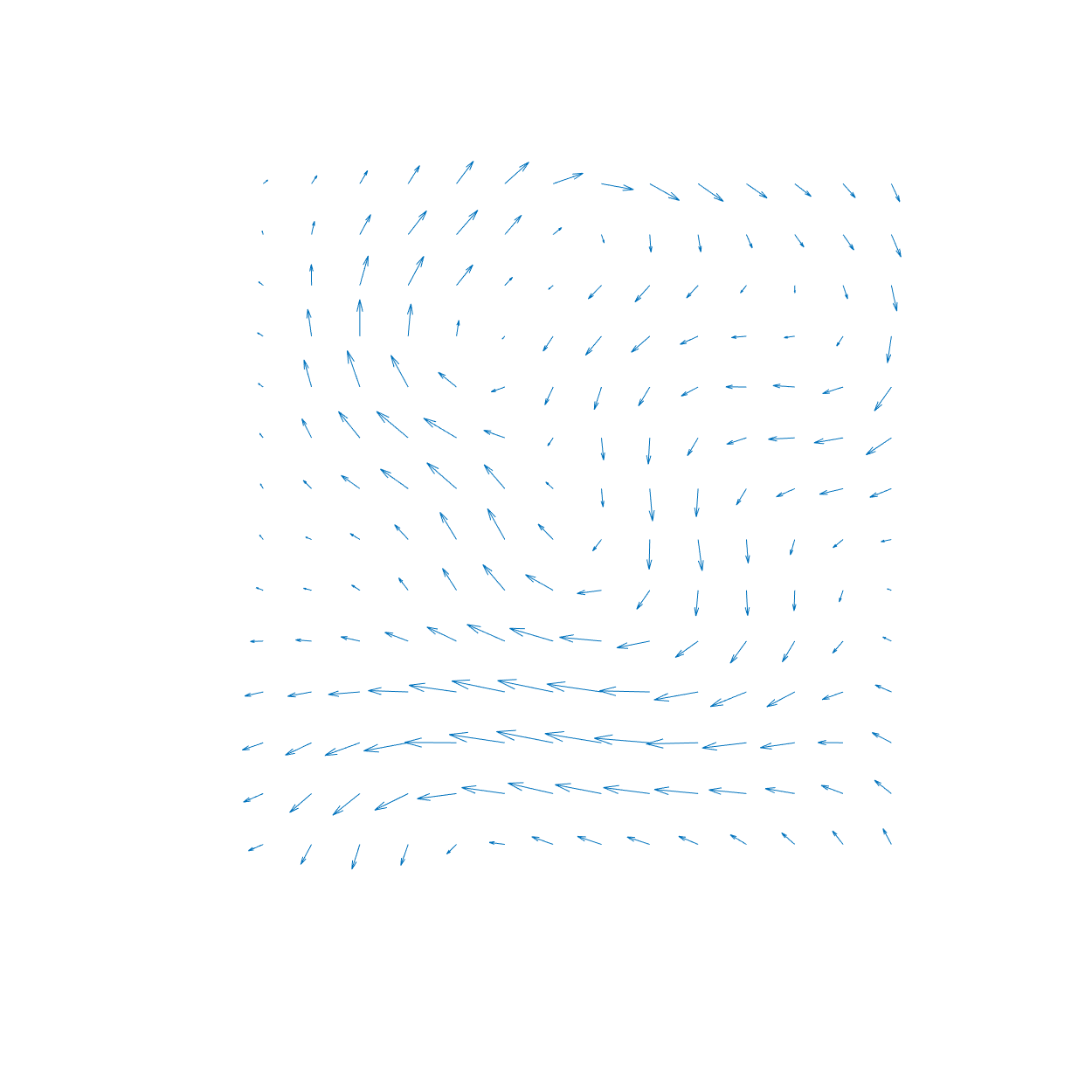

Supplement: S3 MCG raw data 3 — The raw MCG dataset includes category 4 for training and validation. (ZIP) [file pone.0338189.s003.zip › train/4/p10_280_1.png]

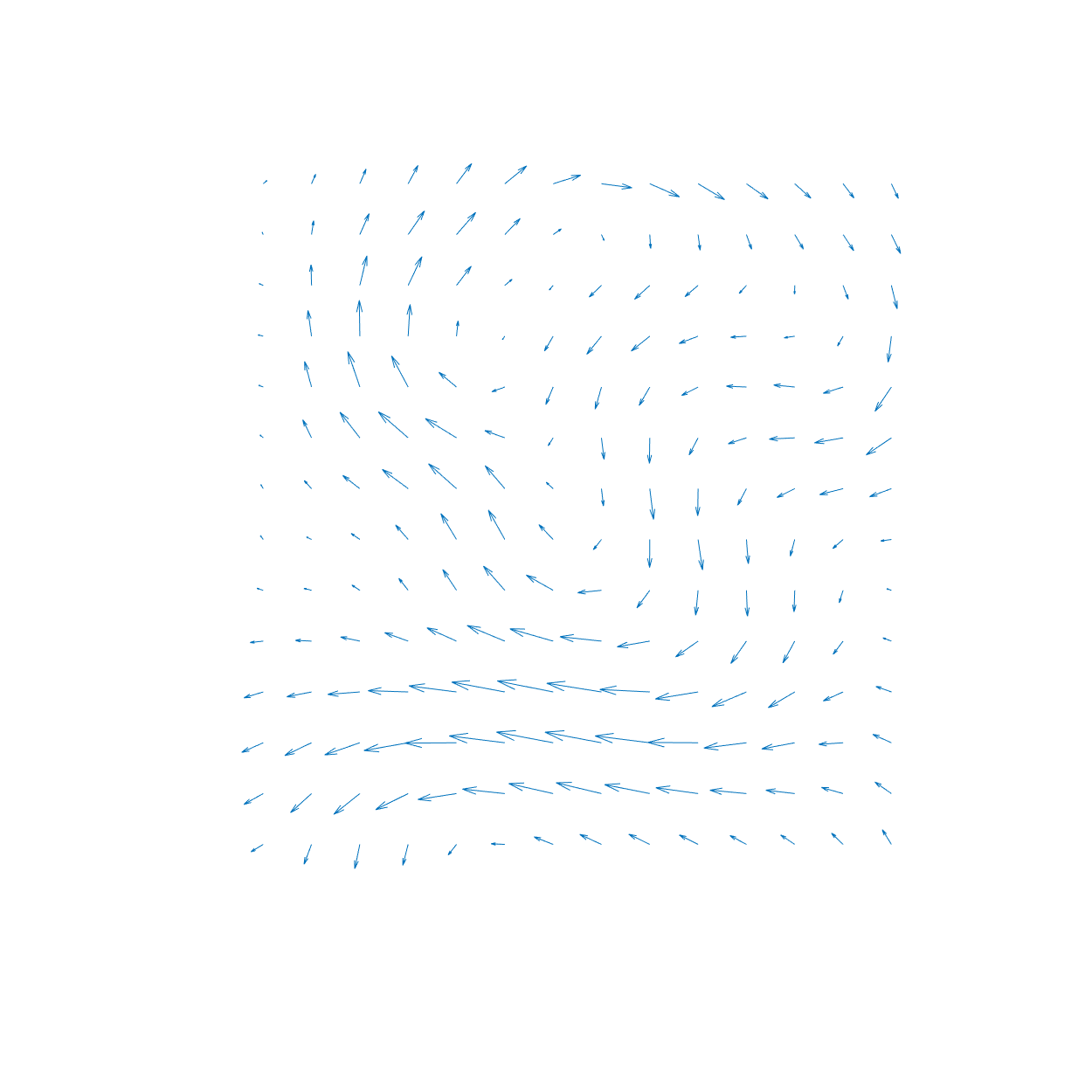

Supplement: S3 MCG raw data 3 — The raw MCG dataset includes category 4 for training and validation. (ZIP) [file pone.0338189.s003.zip › train/4/p10_280_2.png]

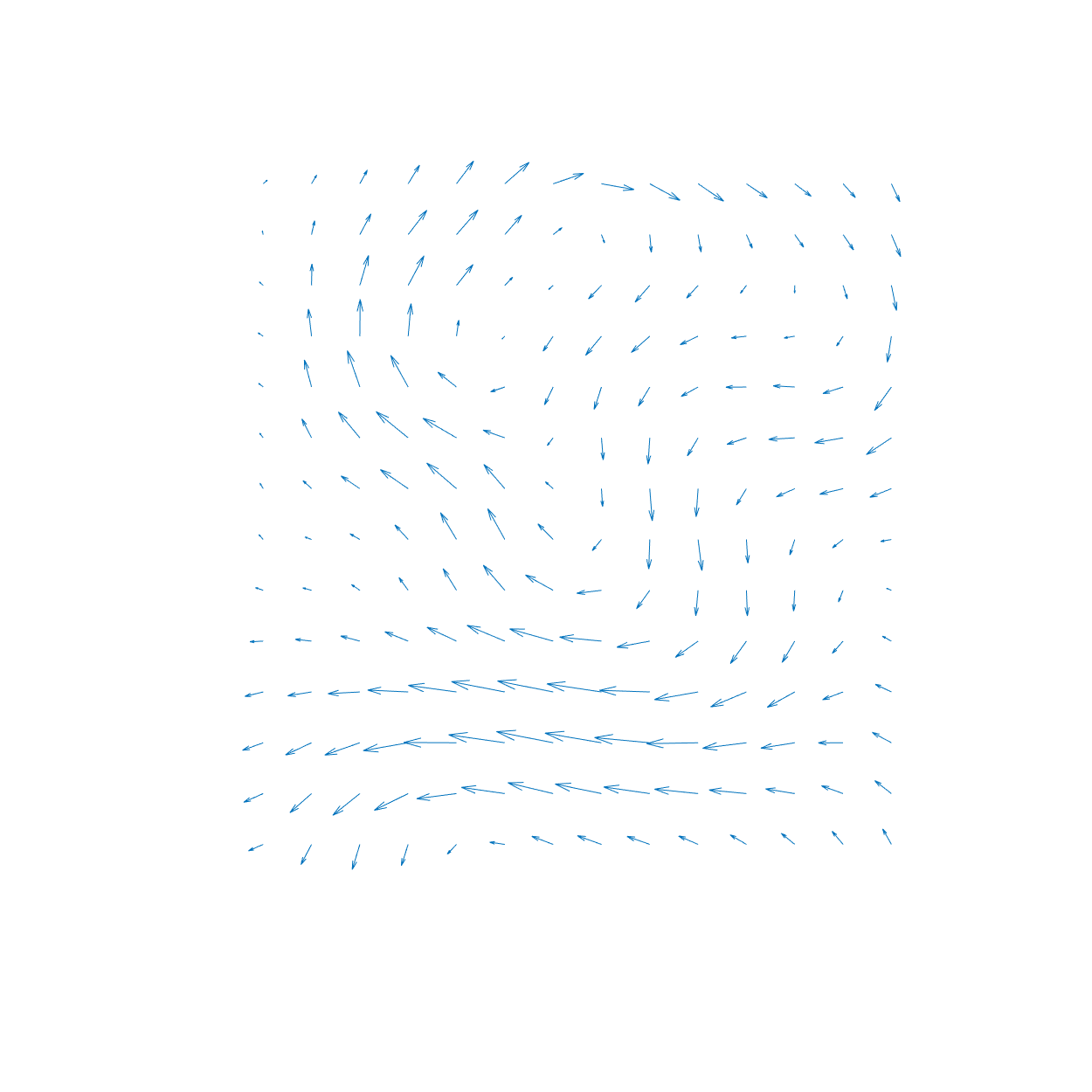

Supplement: S3 MCG raw data 3 — The raw MCG dataset includes category 4 for training and validation. (ZIP) [file pone.0338189.s003.zip › train/4/p10_280_3.png]

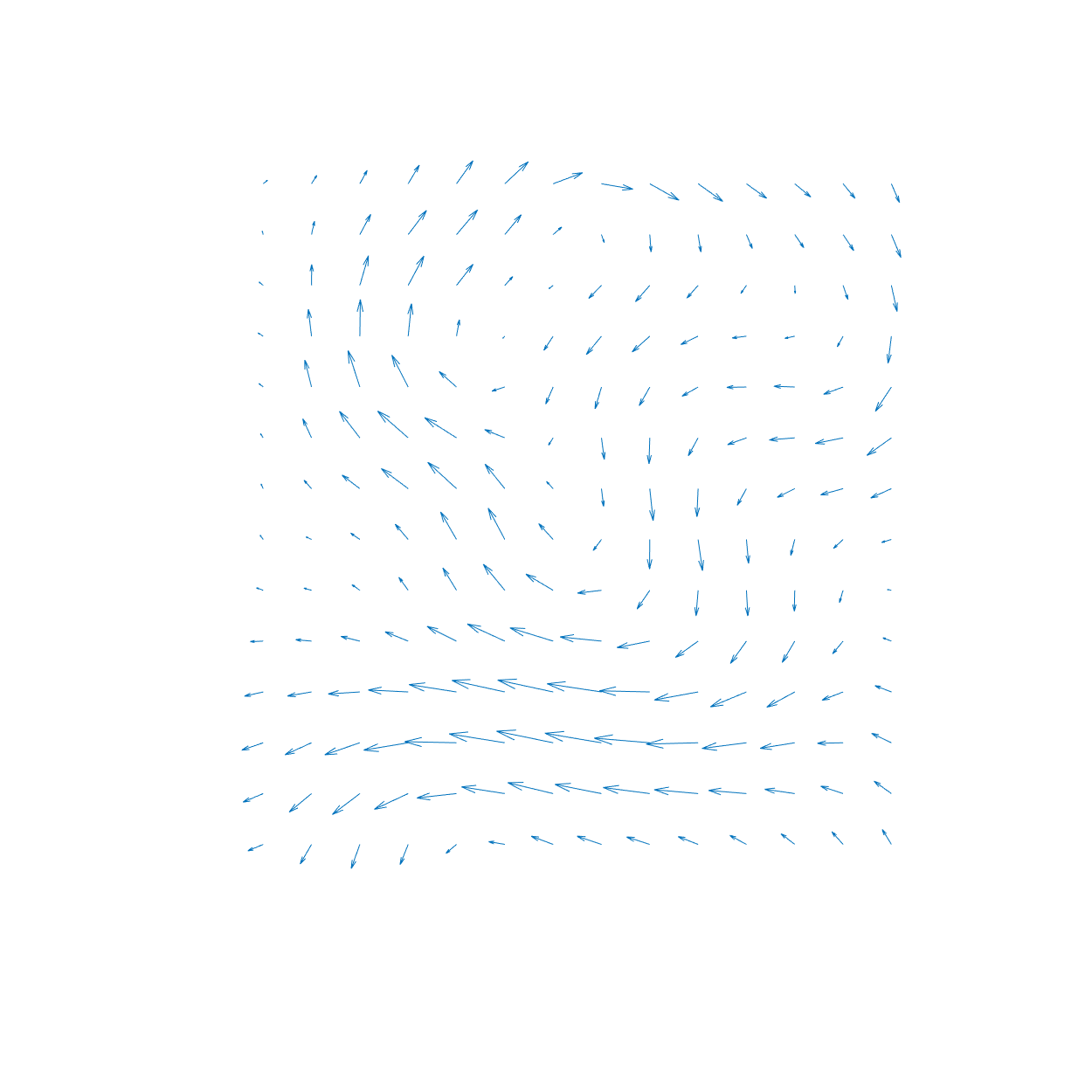

Supplement: S3 MCG raw data 3 — The raw MCG dataset includes category 4 for training and validation. (ZIP) [file pone.0338189.s003.zip › train/4/p10_280_4.png]

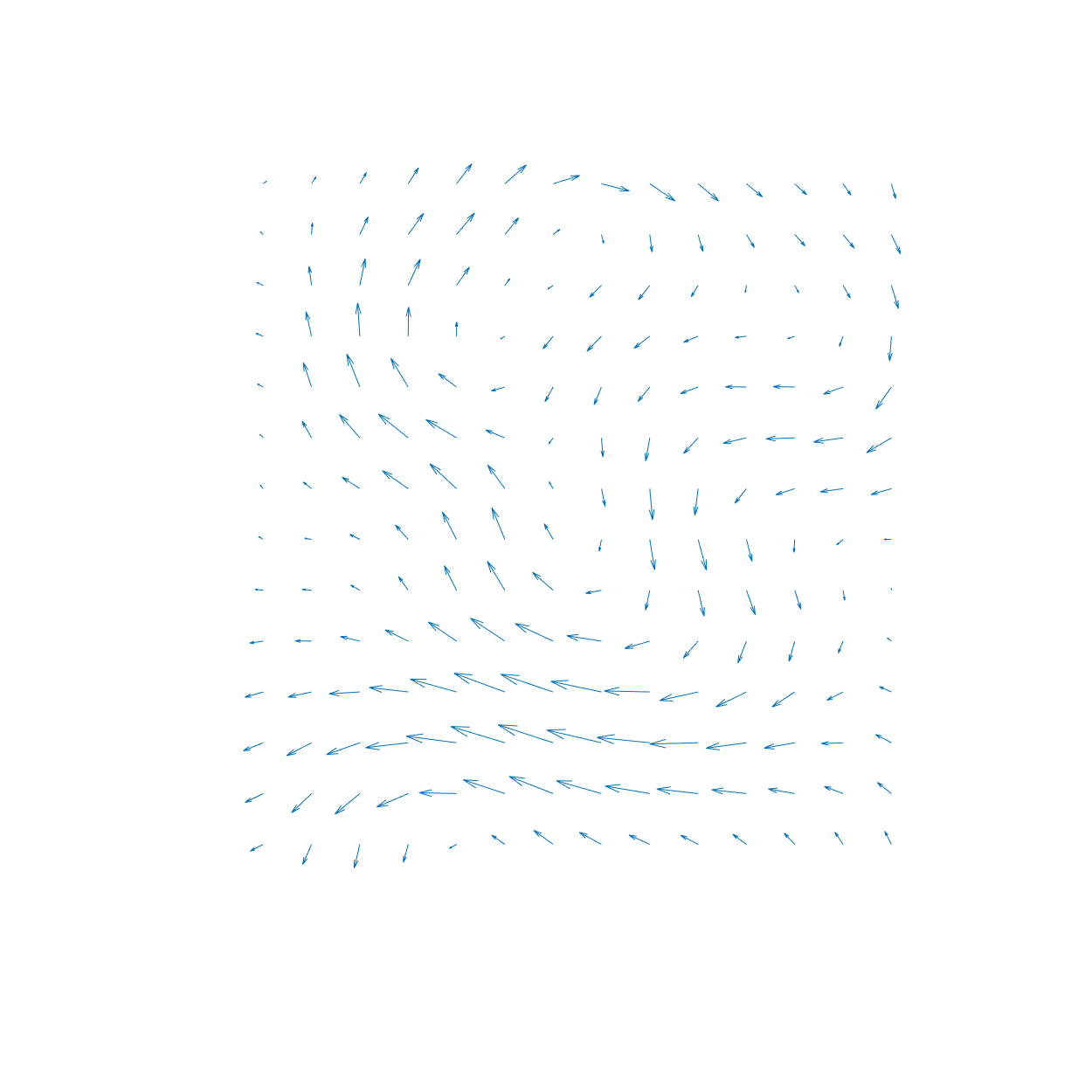

Supplement: S3 MCG raw data 3 — The raw MCG dataset includes category 4 for training and validation. (ZIP) [file pone.0338189.s003.zip › train/4/p10_285_1.png]

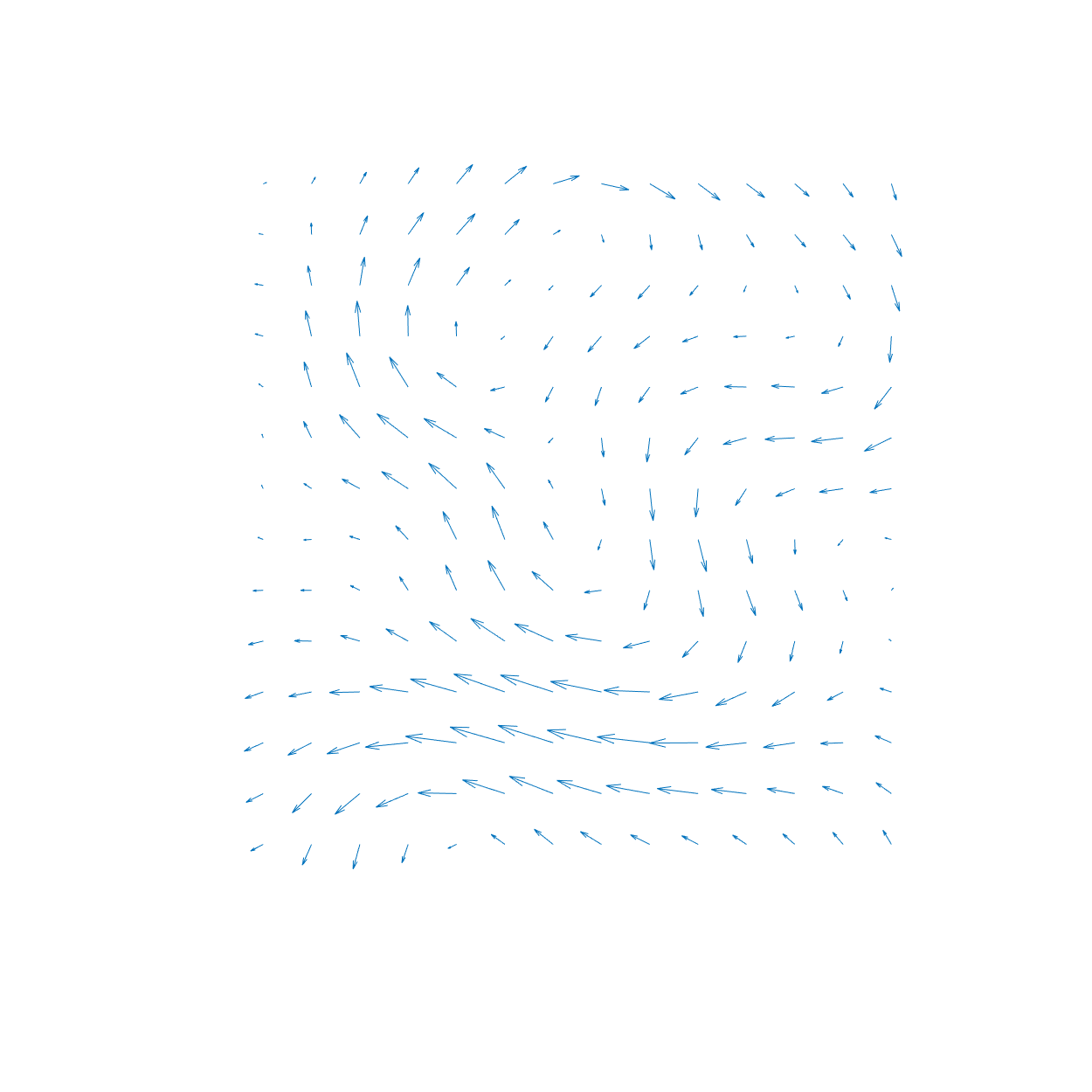

Supplement: S3 MCG raw data 3 — The raw MCG dataset includes category 4 for training and validation. (ZIP) [file pone.0338189.s003.zip › train/4/p10_285_2.png]

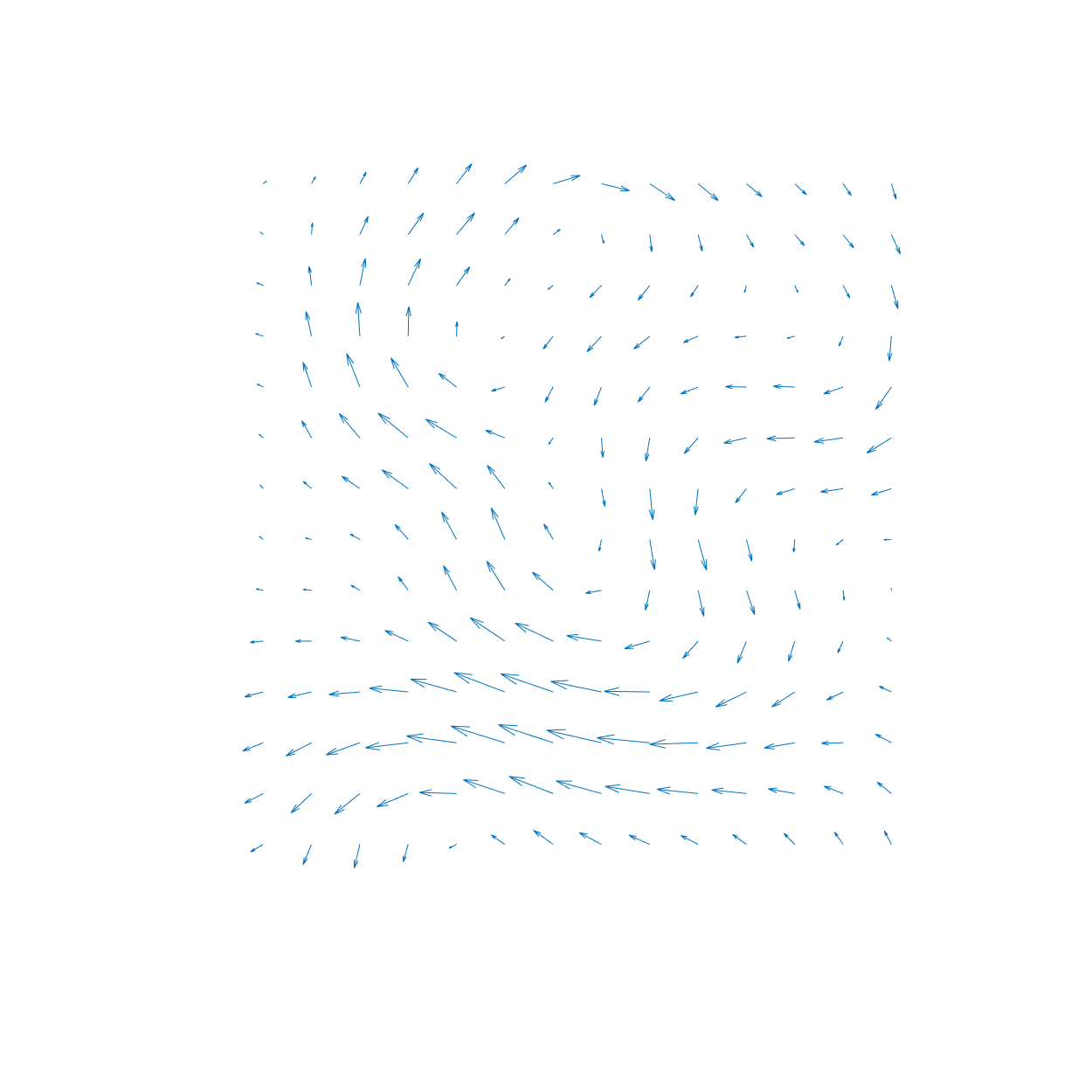

Supplement: S3 MCG raw data 3 — The raw MCG dataset includes category 4 for training and validation. (ZIP) [file pone.0338189.s003.zip › train/4/p10_285_3.png]

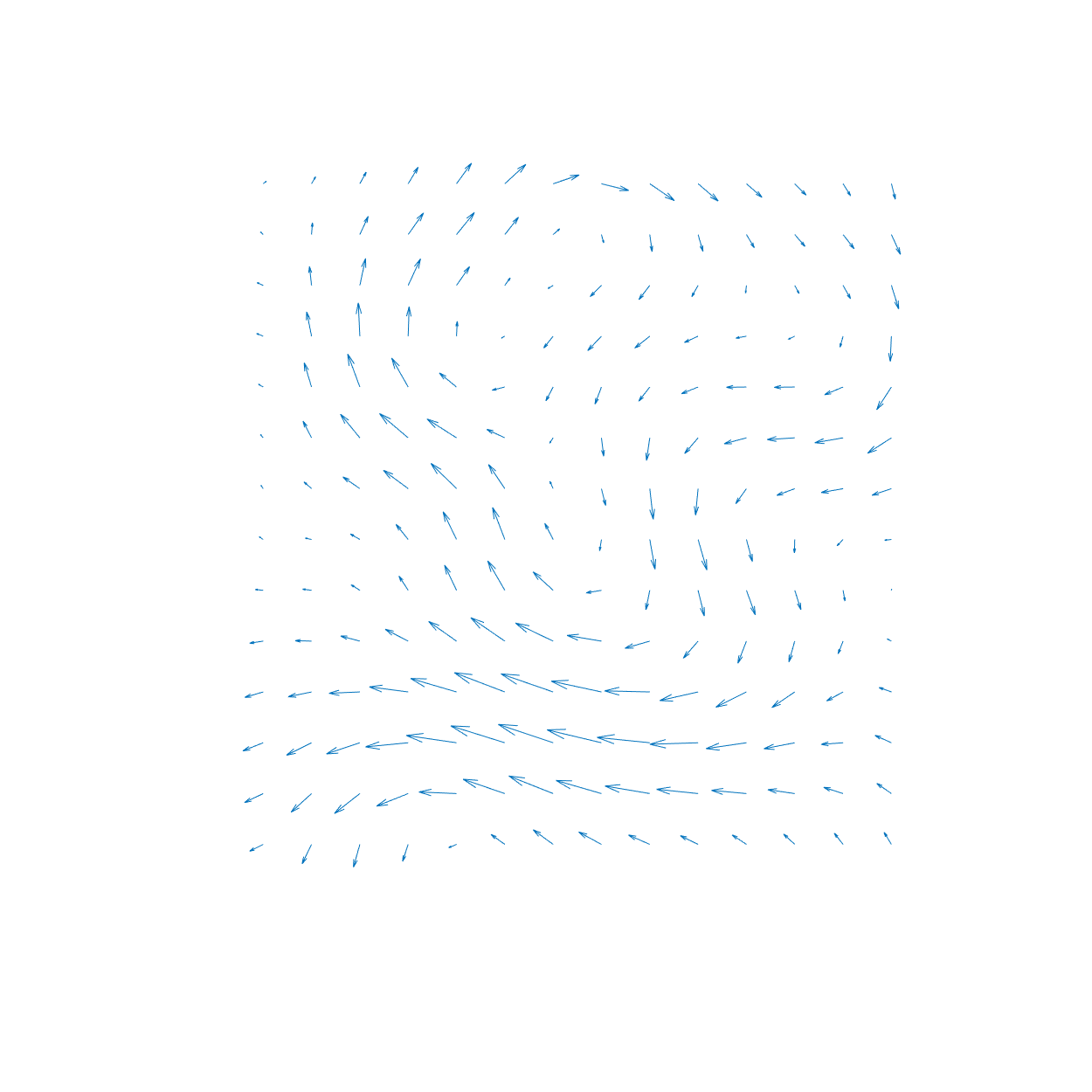

Supplement: S3 MCG raw data 3 — The raw MCG dataset includes category 4 for training and validation. (ZIP) [file pone.0338189.s003.zip › train/4/p10_285_4.png]

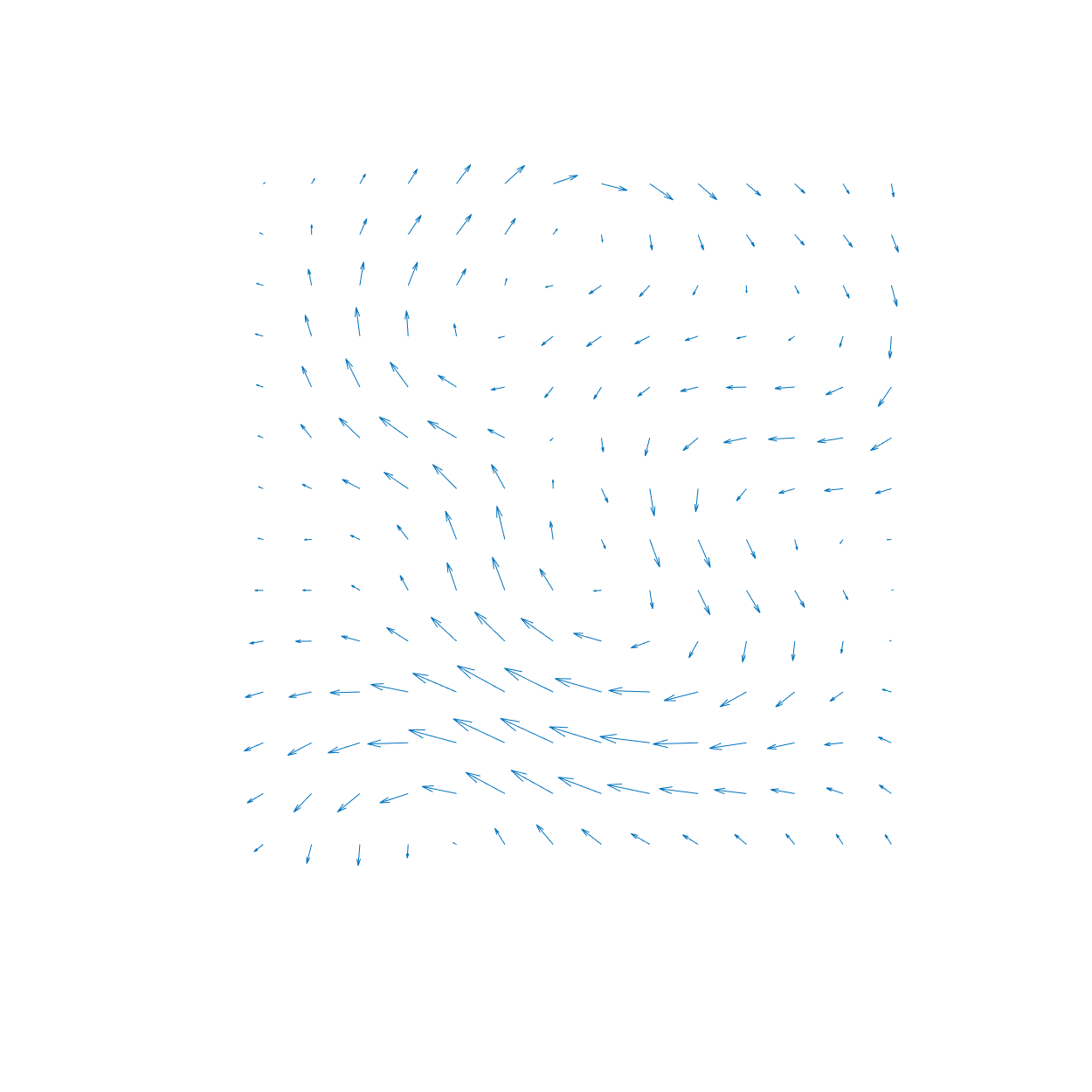

Supplement: S3 MCG raw data 3 — The raw MCG dataset includes category 4 for training and validation. (ZIP) [file pone.0338189.s003.zip › train/4/p10_290_1.png]

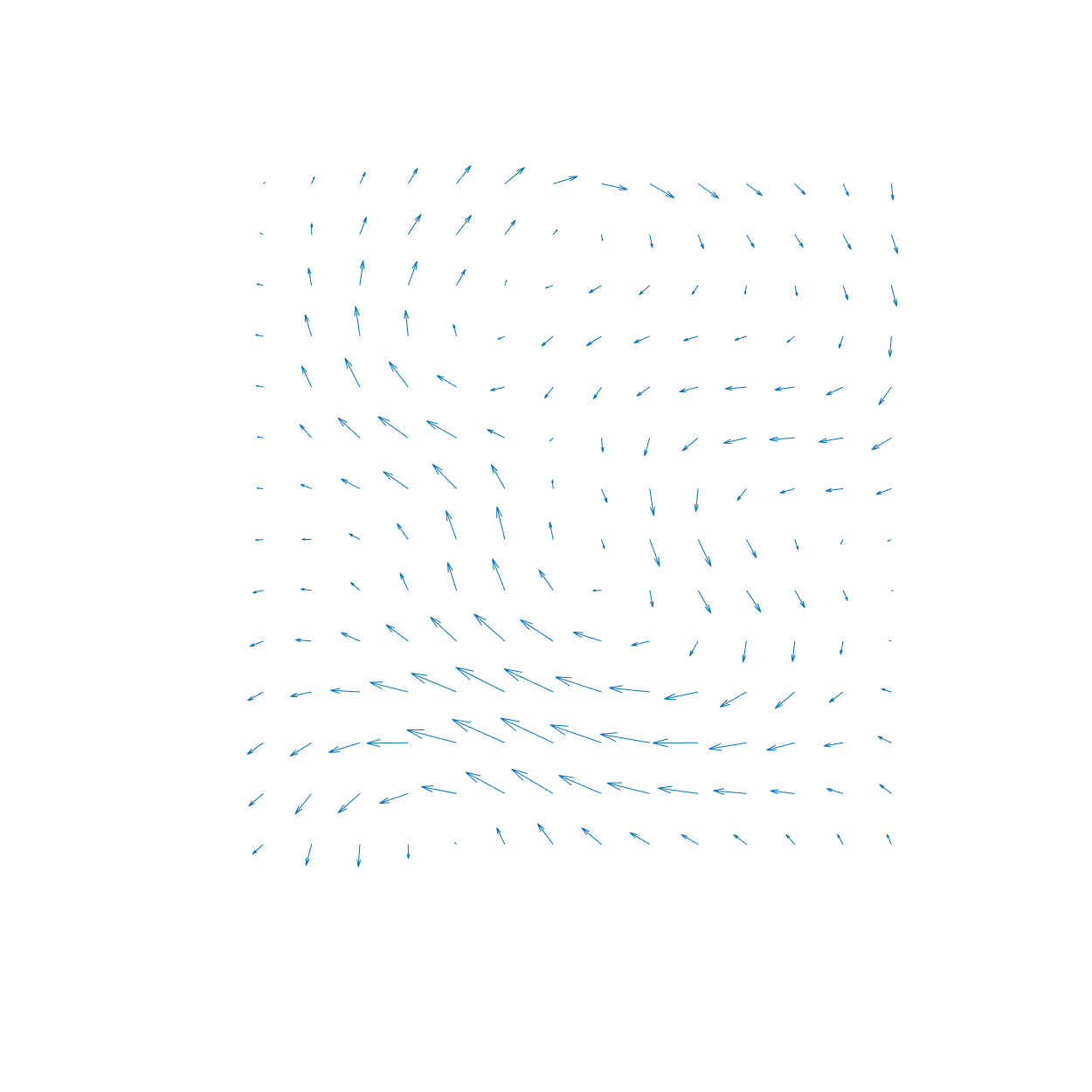

Supplement: S3 MCG raw data 3 — The raw MCG dataset includes category 4 for training and validation. (ZIP) [file pone.0338189.s003.zip › train/4/p10_290_2.png]

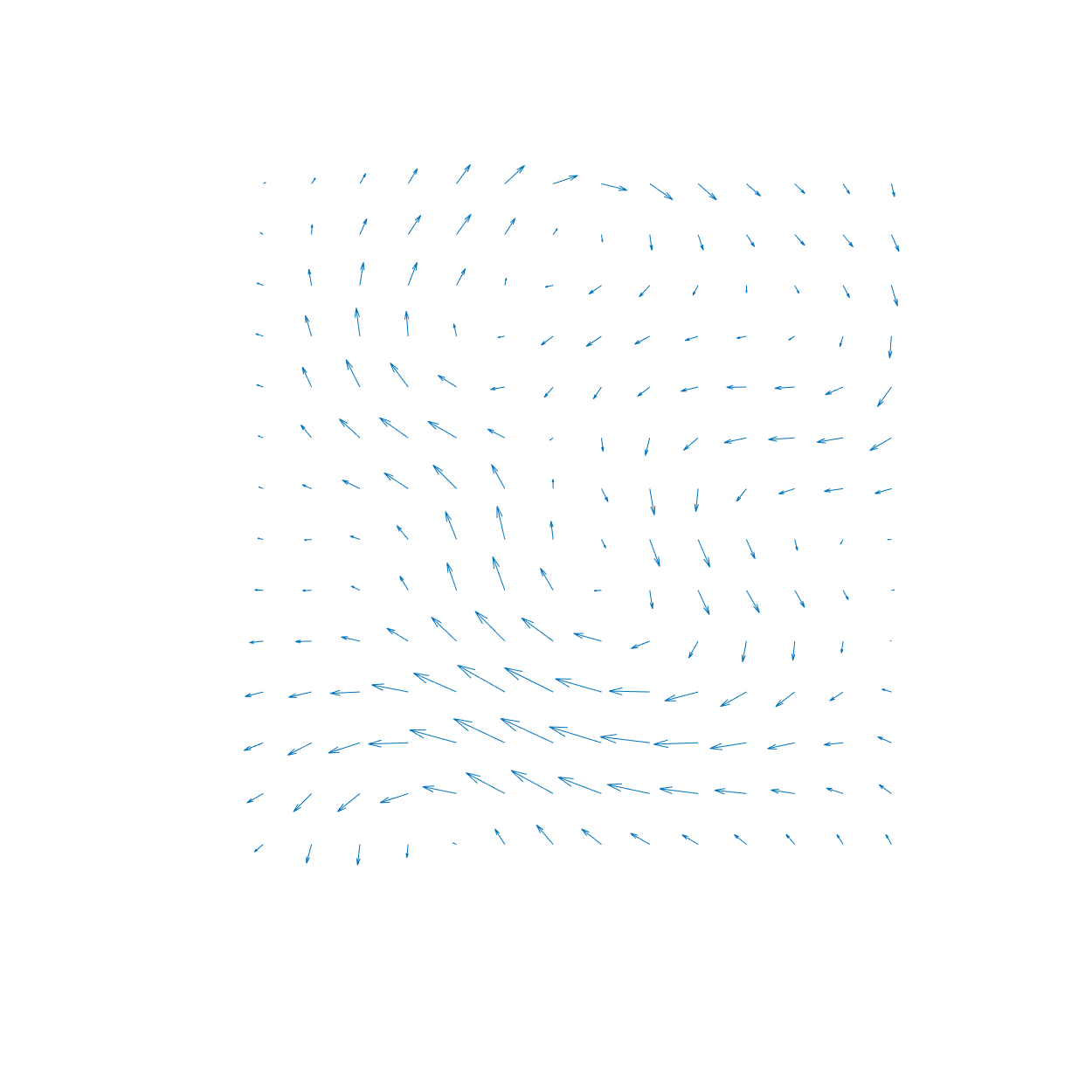

Supplement: S3 MCG raw data 3 — The raw MCG dataset includes category 4 for training and validation. (ZIP) [file pone.0338189.s003.zip › train/4/p10_290_3.png]

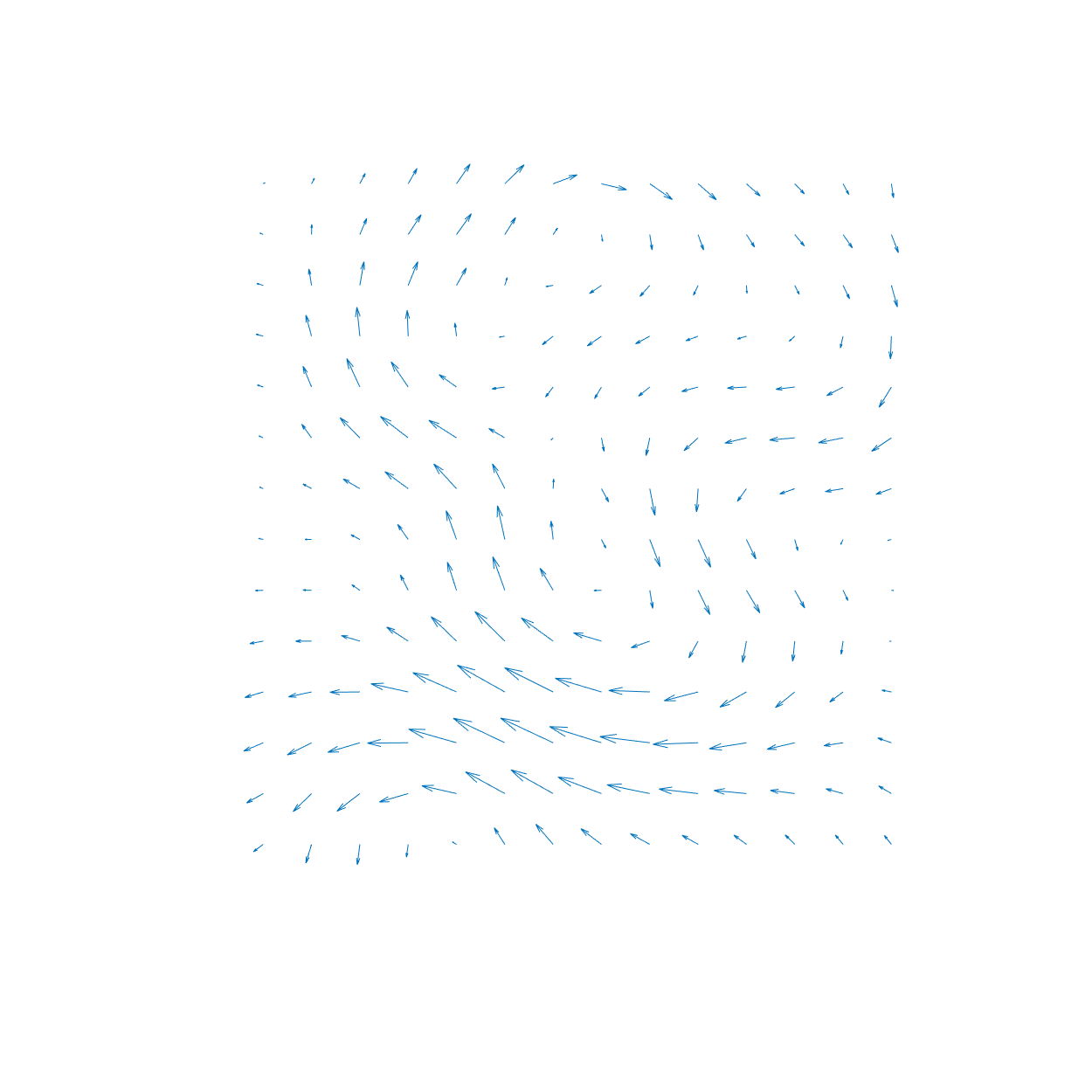

Supplement: S3 MCG raw data 3 — The raw MCG dataset includes category 4 for training and validation. (ZIP) [file pone.0338189.s003.zip › train/4/p10_290_4.png]

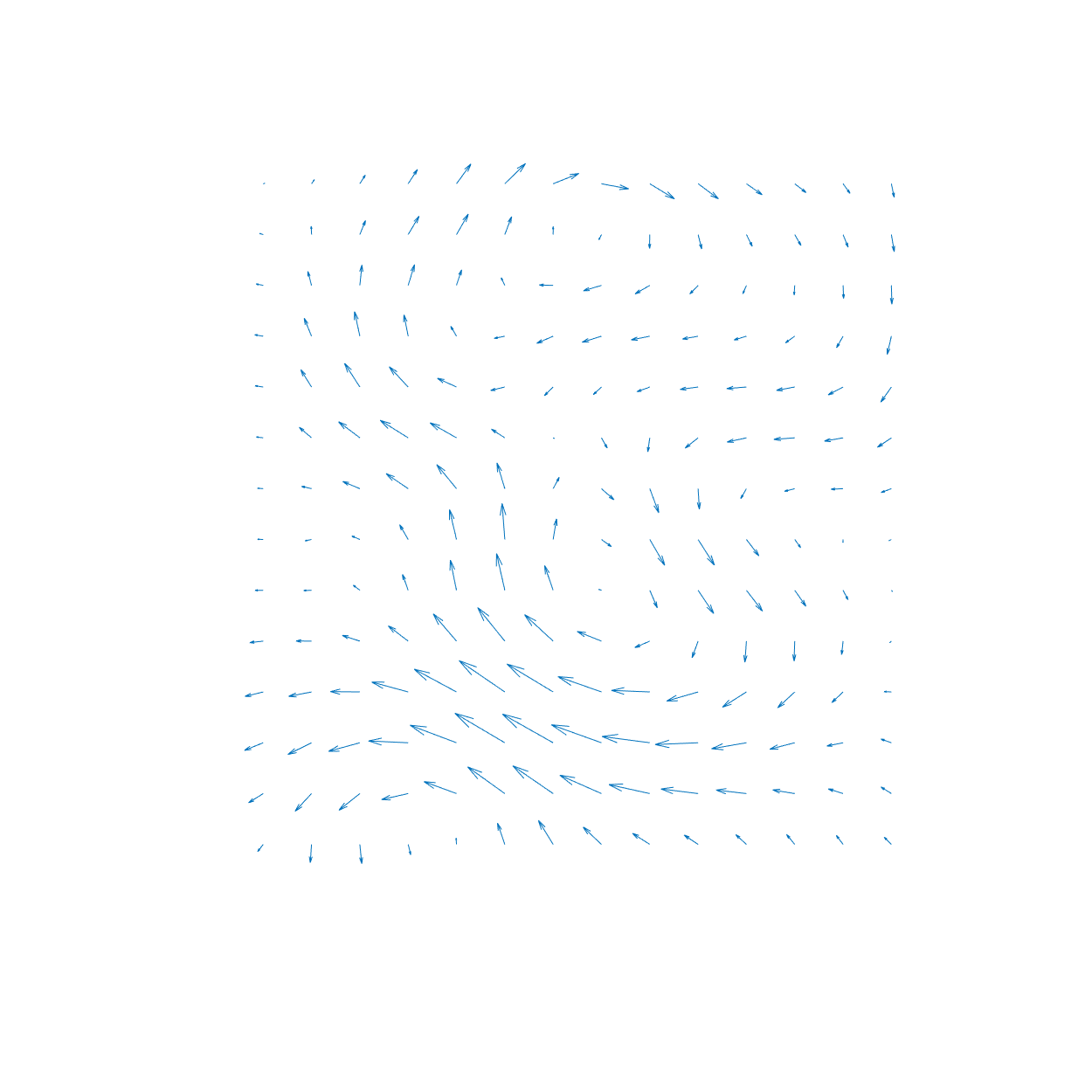

Supplement: S3 MCG raw data 3 — The raw MCG dataset includes category 4 for training and validation. (ZIP) [file pone.0338189.s003.zip › train/4/p10_295_1.png]

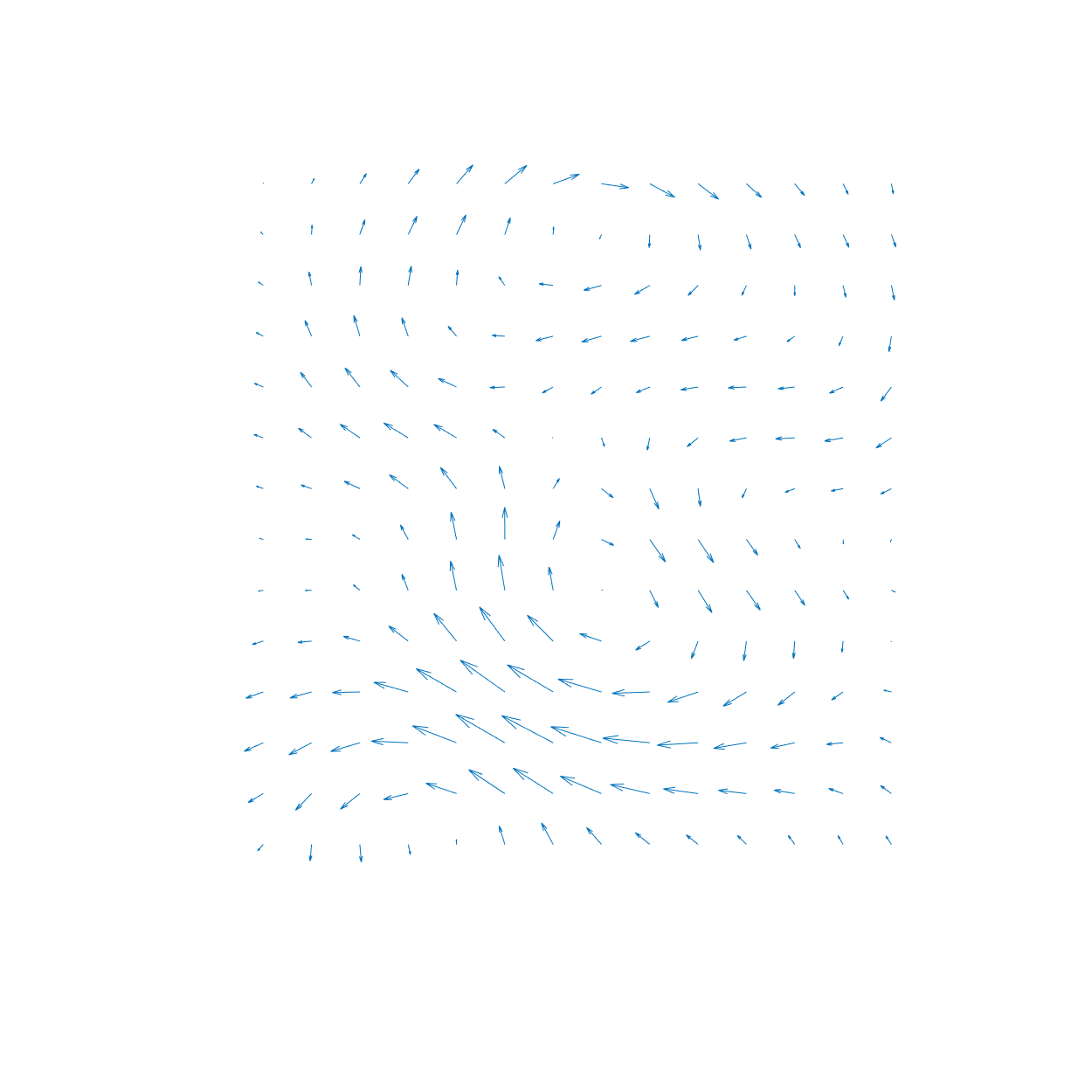

Supplement: S3 MCG raw data 3 — The raw MCG dataset includes category 4 for training and validation. (ZIP) [file pone.0338189.s003.zip › train/4/p10_295_2.png]

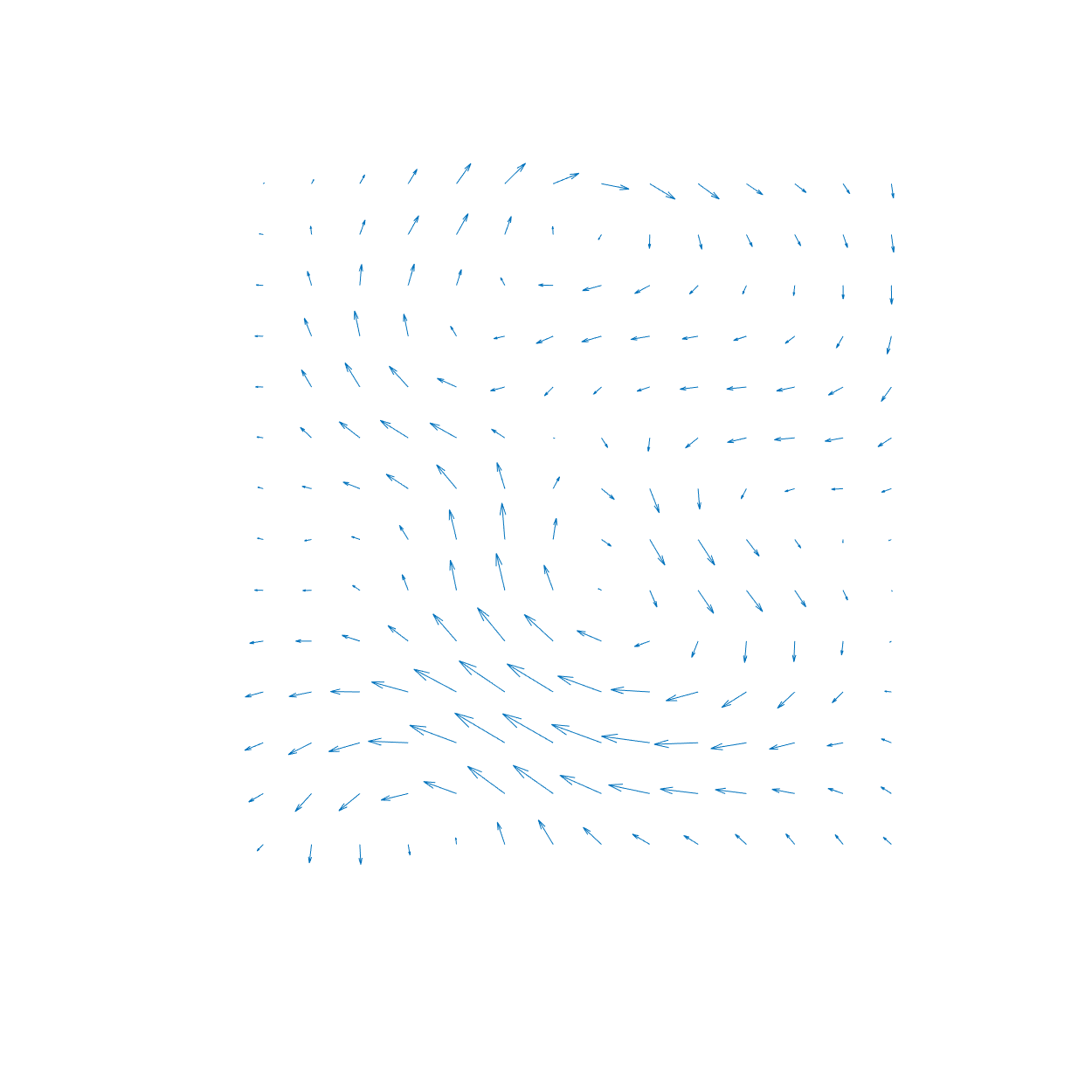

Supplement: S3 MCG raw data 3 — The raw MCG dataset includes category 4 for training and validation. (ZIP) [file pone.0338189.s003.zip › train/4/p10_295_3.png]

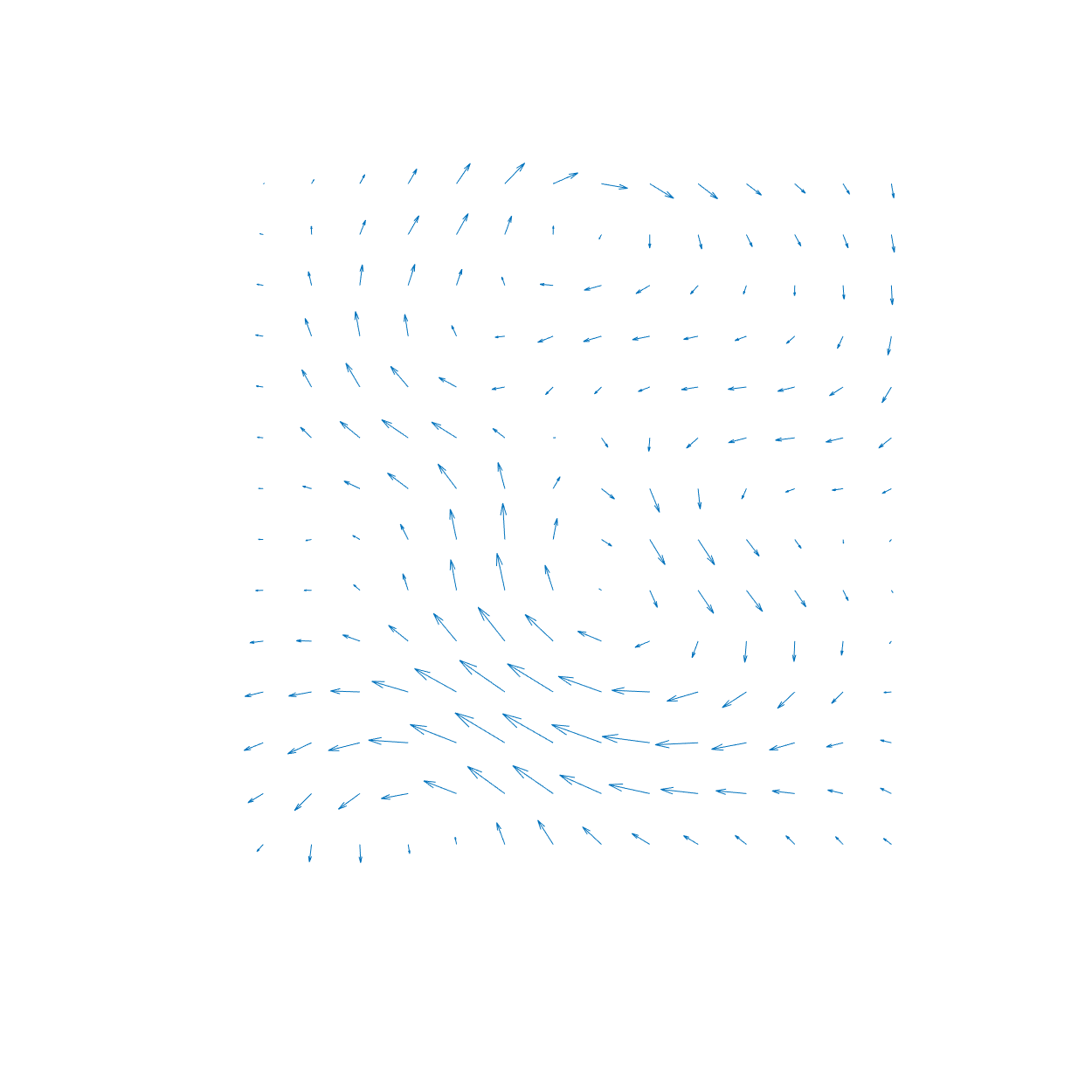

Supplement: S3 MCG raw data 3 — The raw MCG dataset includes category 4 for training and validation. (ZIP) [file pone.0338189.s003.zip › train/4/p10_295_4.png]

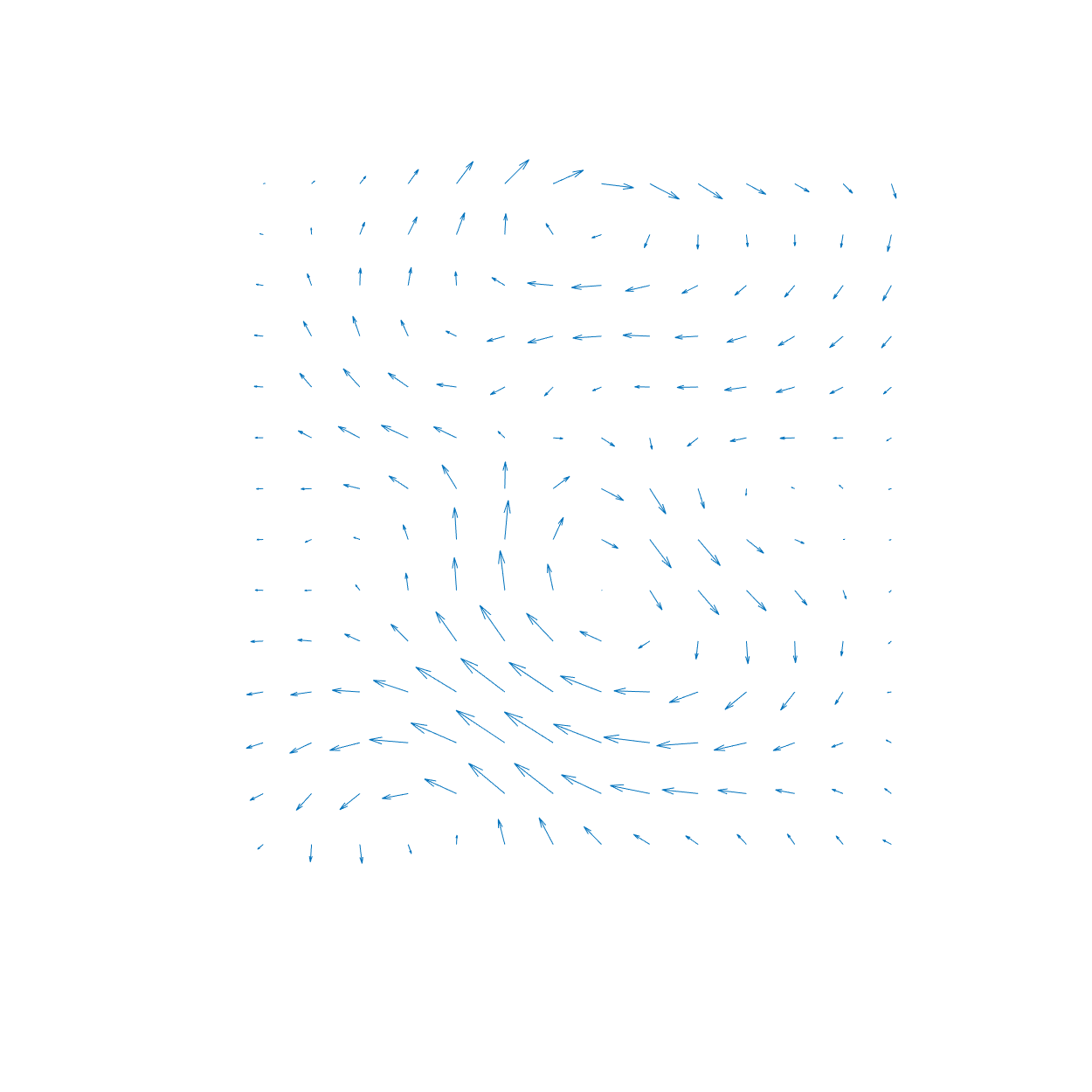

Supplement: S3 MCG raw data 3 — The raw MCG dataset includes category 4 for training and validation. (ZIP) [file pone.0338189.s003.zip › train/4/p10_300_1.png]

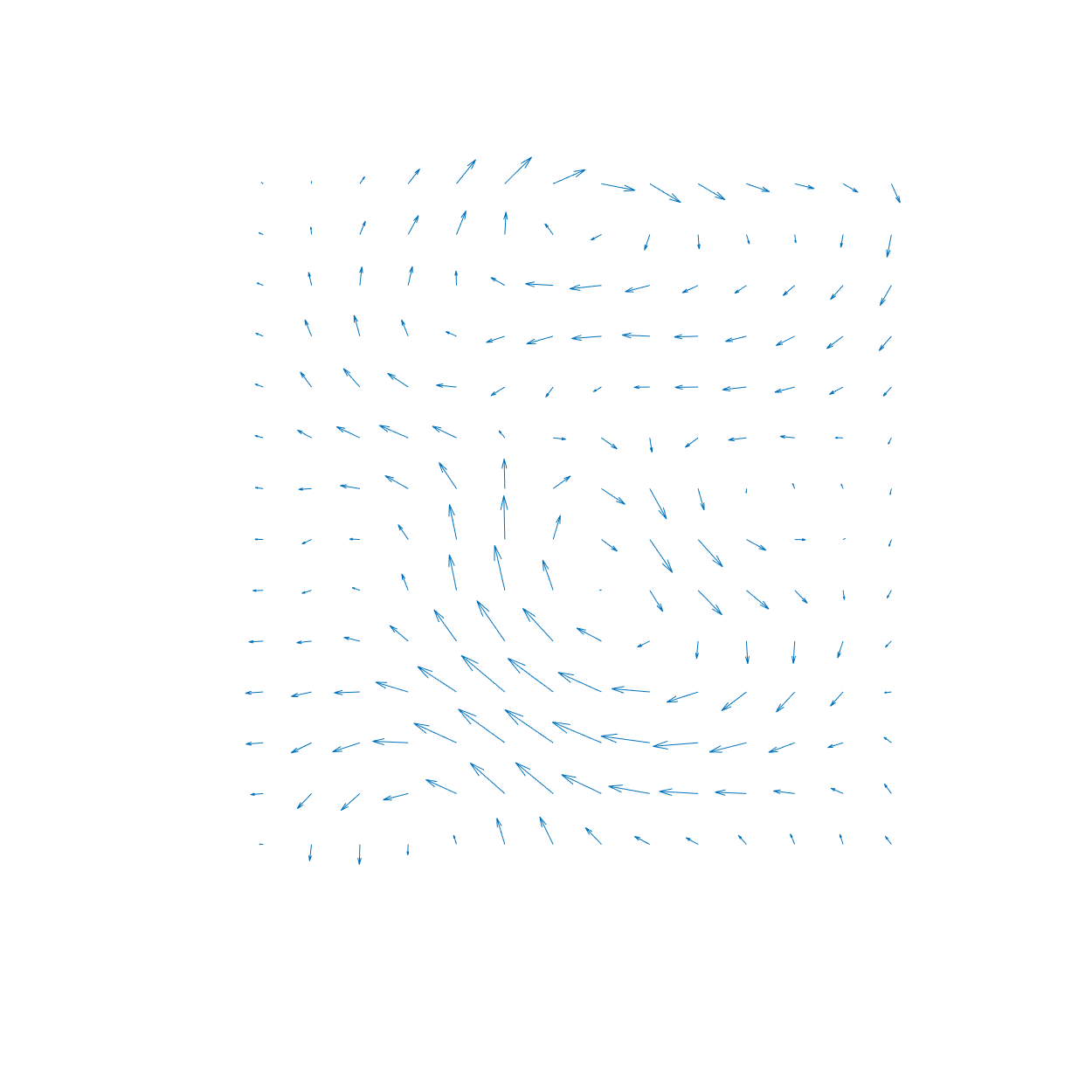

Supplement: S3 MCG raw data 3 — The raw MCG dataset includes category 4 for training and validation. (ZIP) [file pone.0338189.s003.zip › train/4/p10_300_2.png]

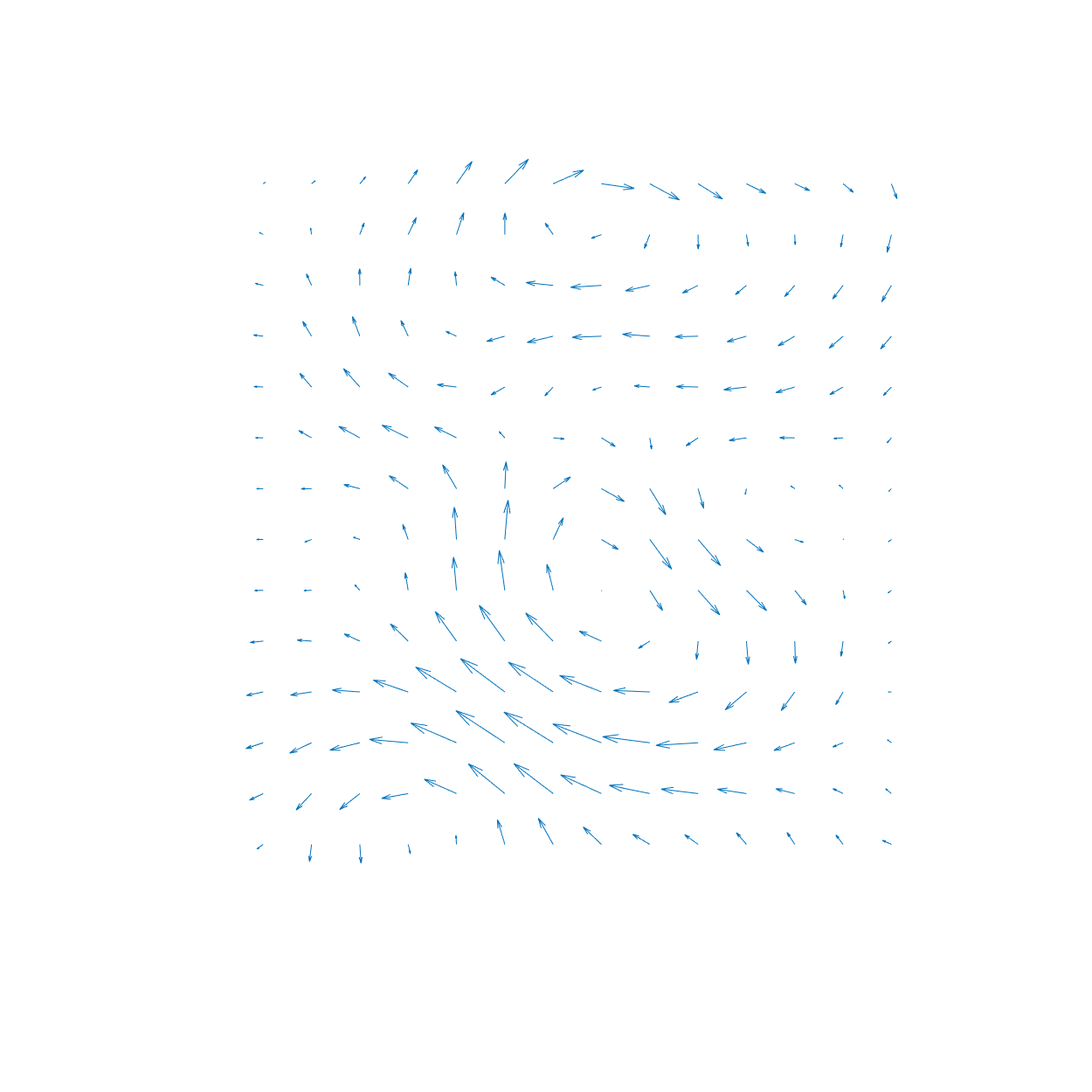

Supplement: S3 MCG raw data 3 — The raw MCG dataset includes category 4 for training and validation. (ZIP) [file pone.0338189.s003.zip › train/4/p10_300_3.png]

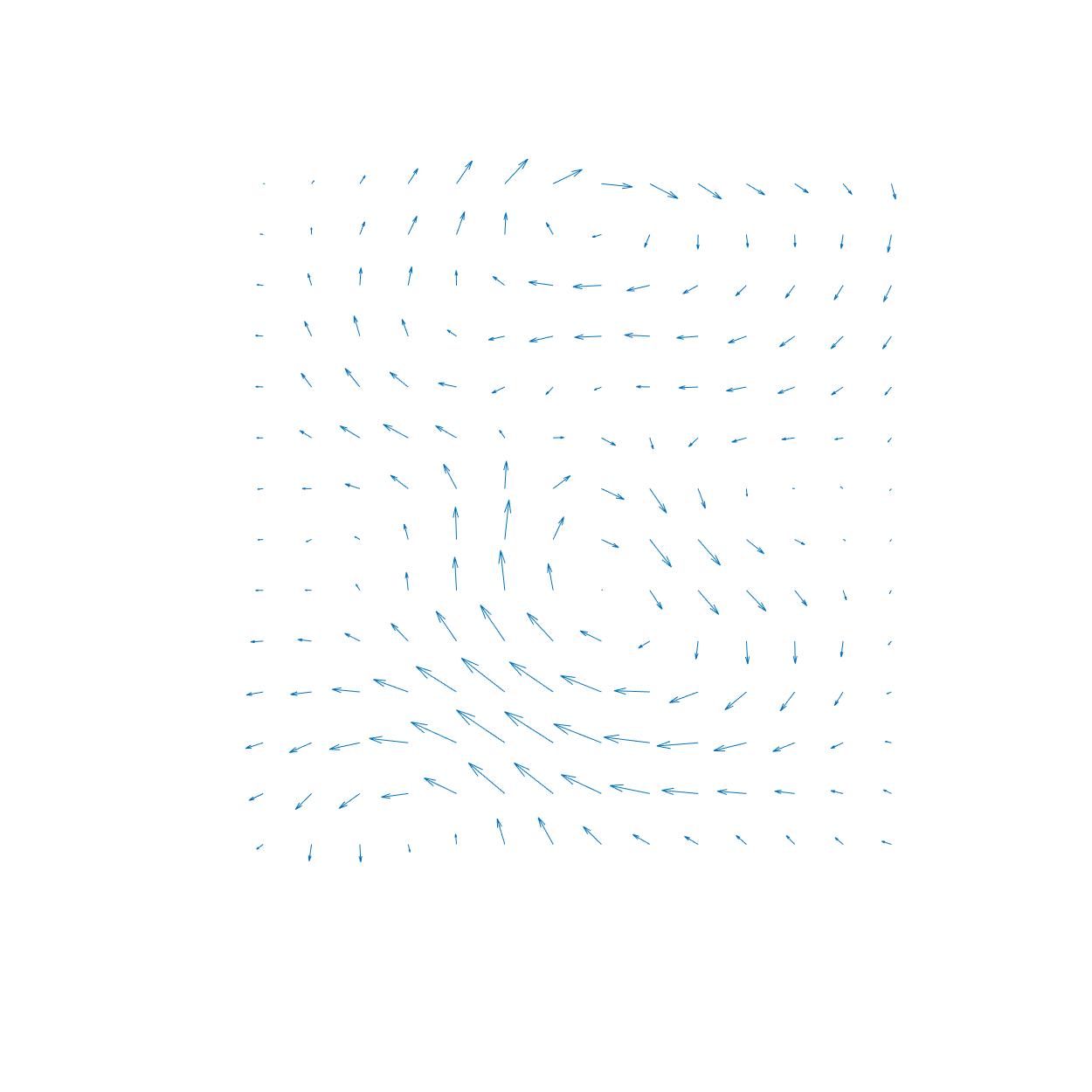

Supplement: S3 MCG raw data 3 — The raw MCG dataset includes category 4 for training and validation. (ZIP) [file pone.0338189.s003.zip › train/4/p10_300_4.png]

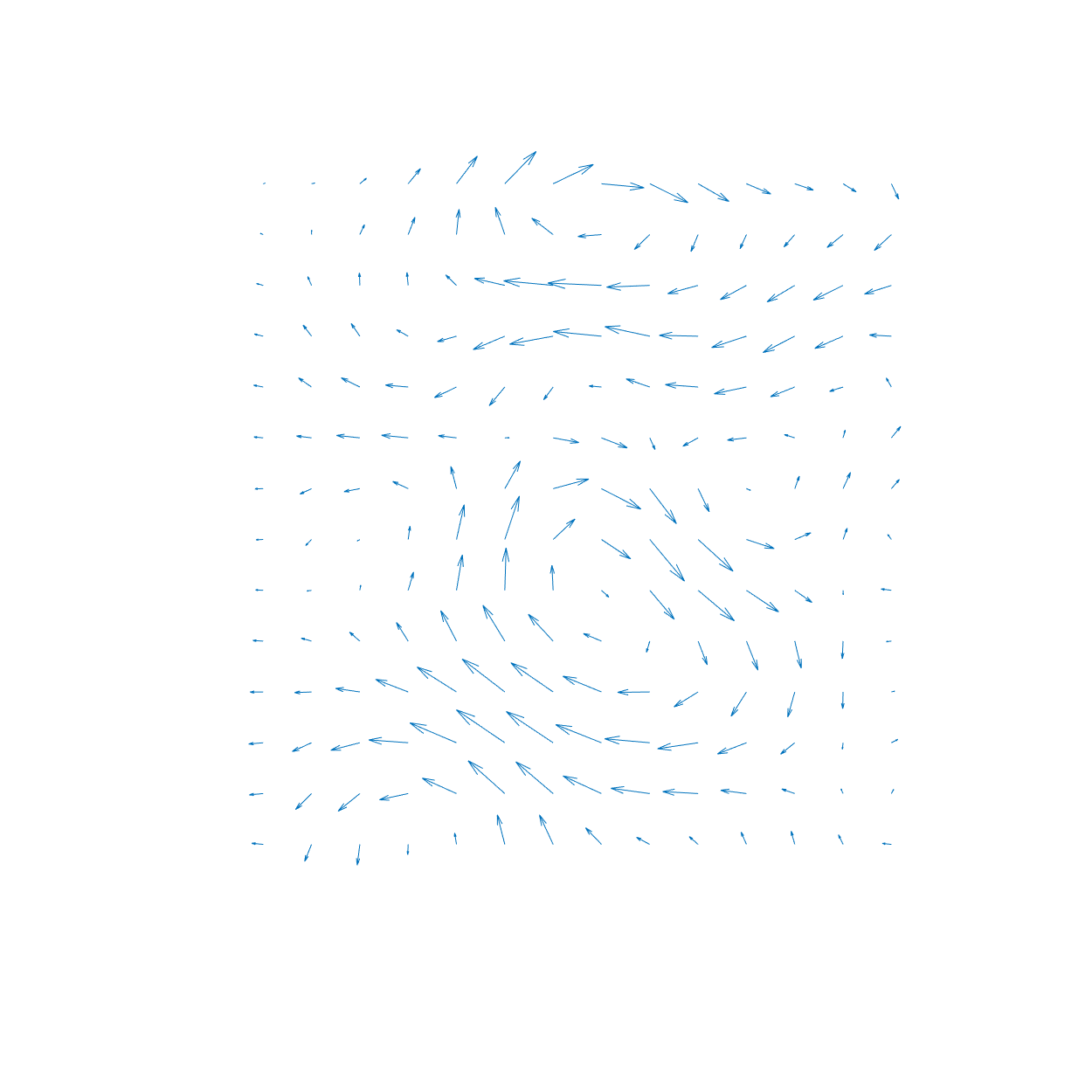

Supplement: S3 MCG raw data 3 — The raw MCG dataset includes category 4 for training and validation. (ZIP) [file pone.0338189.s003.zip › train/4/p10_305_1.png]

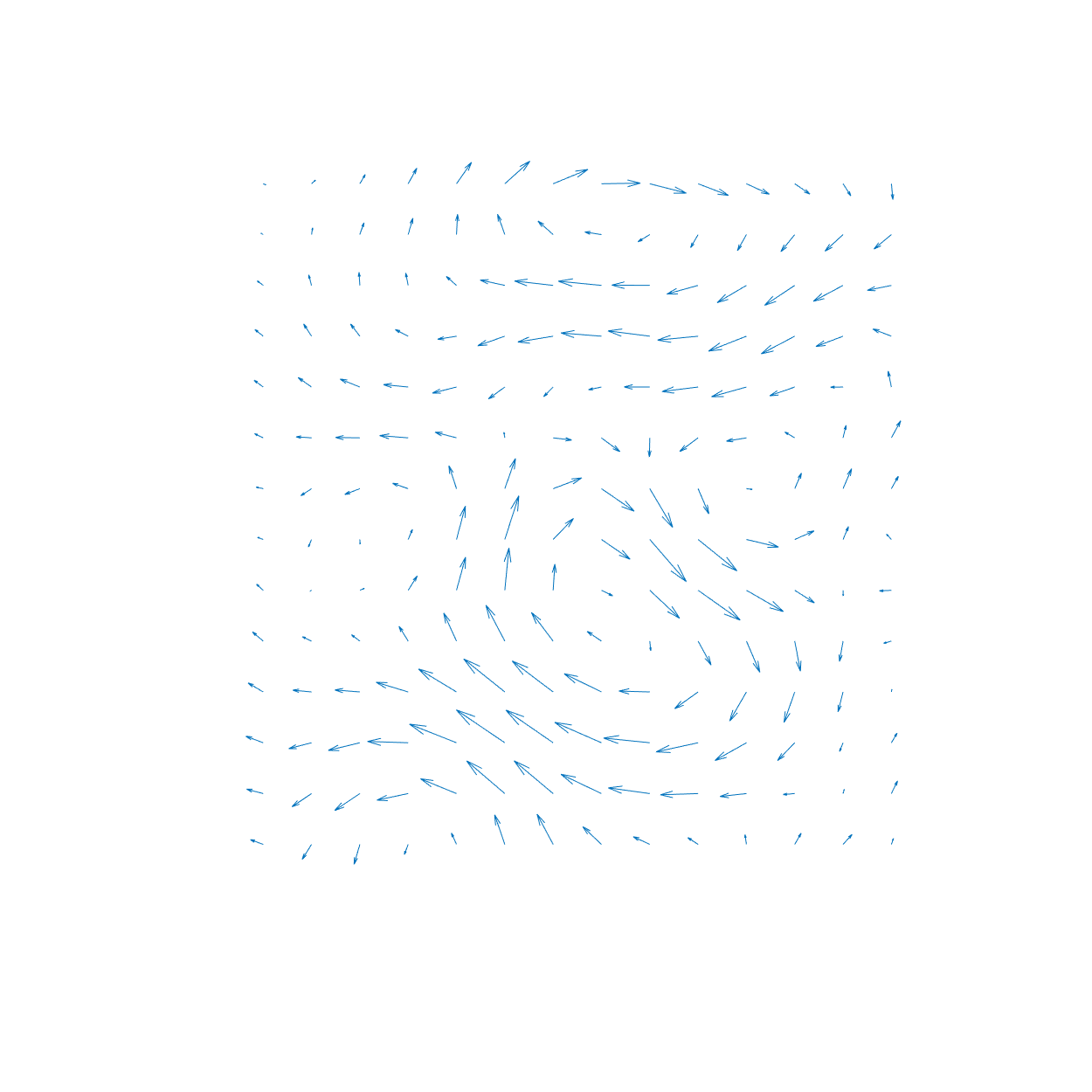

Supplement: S3 MCG raw data 3 — The raw MCG dataset includes category 4 for training and validation. (ZIP) [file pone.0338189.s003.zip › train/4/p10_305_2.png]

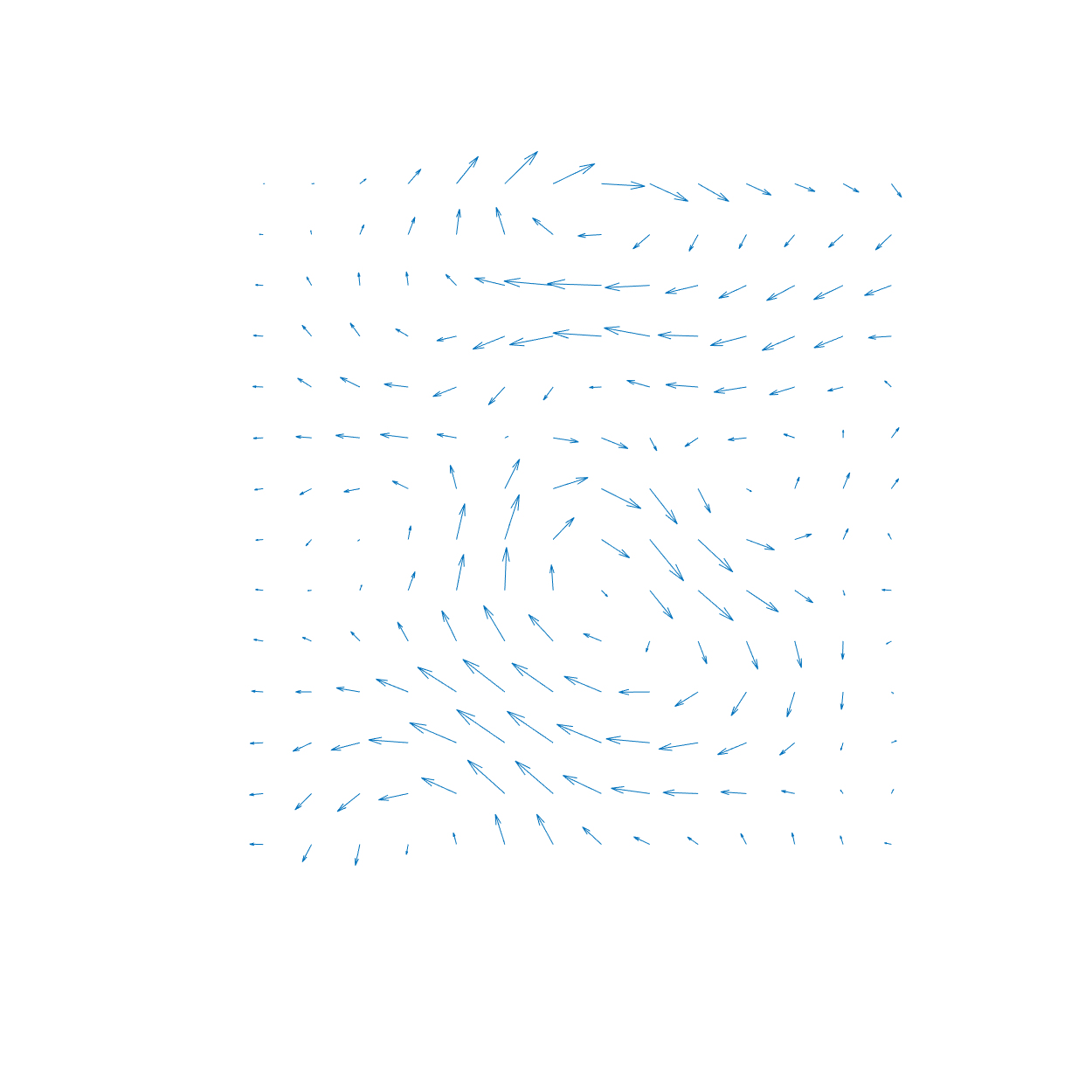

Supplement: S3 MCG raw data 3 — The raw MCG dataset includes category 4 for training and validation. (ZIP) [file pone.0338189.s003.zip › train/4/p10_305_3.png]

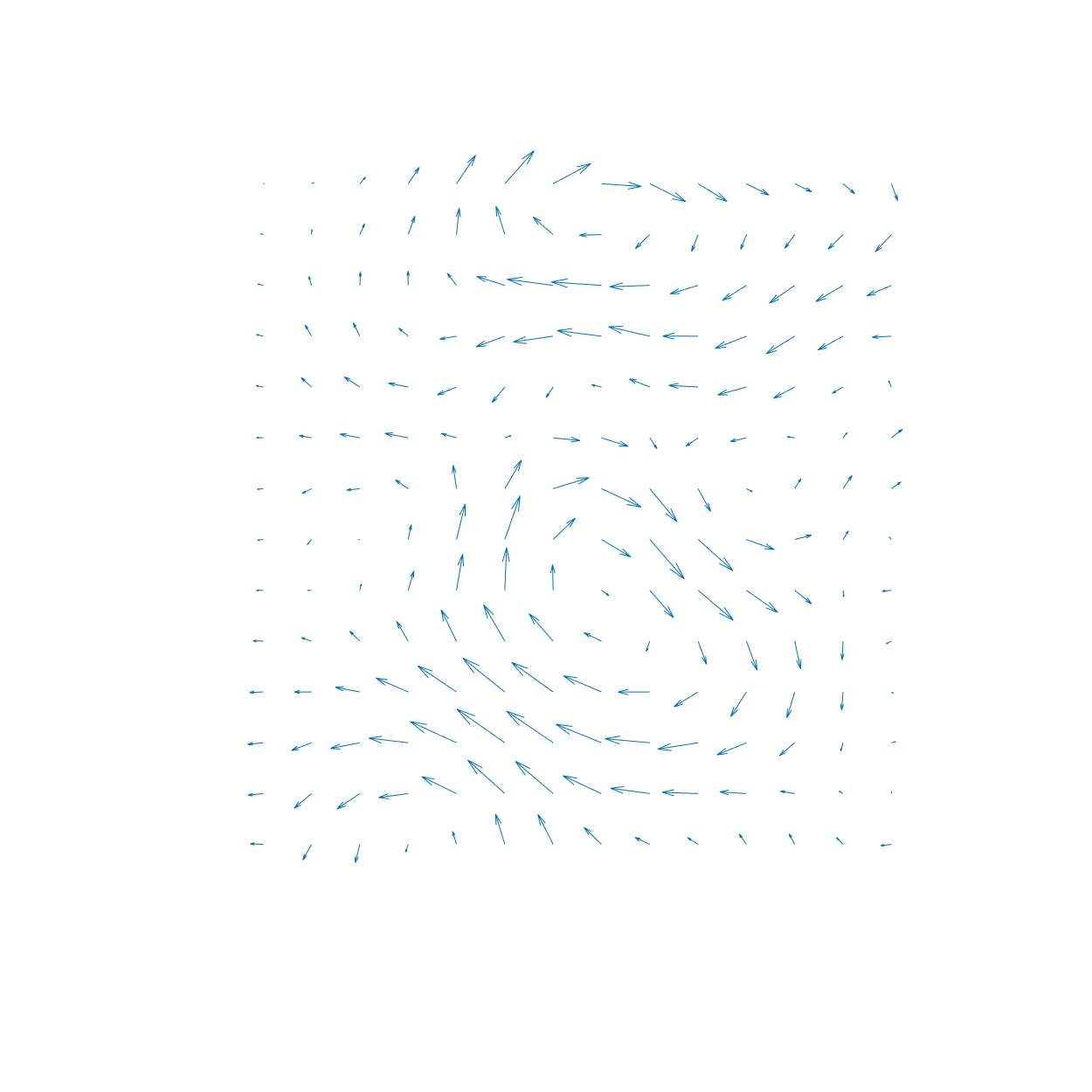

Supplement: S3 MCG raw data 3 — The raw MCG dataset includes category 4 for training and validation. (ZIP) [file pone.0338189.s003.zip › train/4/p10_305_4.png]

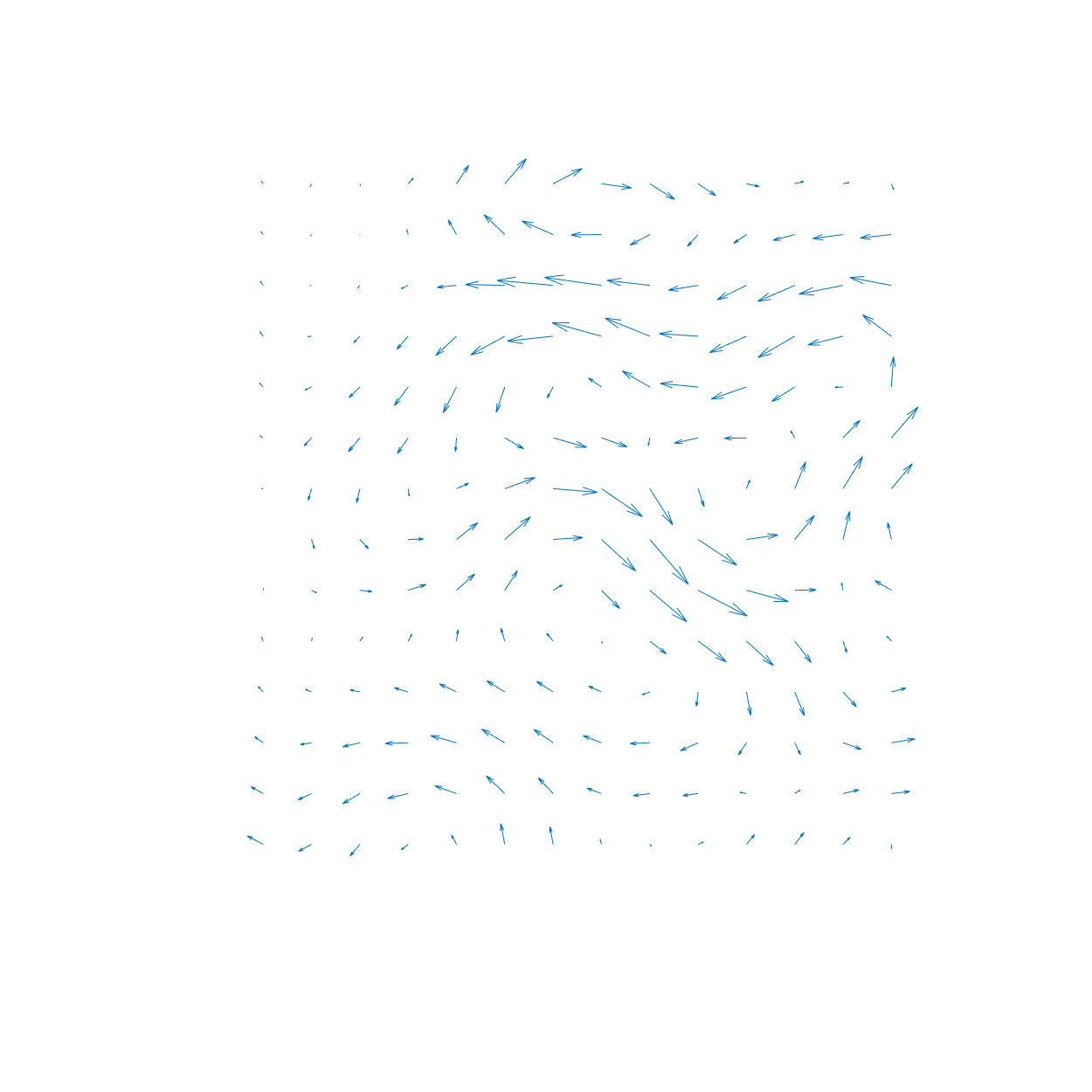

Supplement: S3 MCG raw data 3 — The raw MCG dataset includes category 4 for training and validation. (ZIP) [file pone.0338189.s003.zip › train/4/p10_310_1.png]

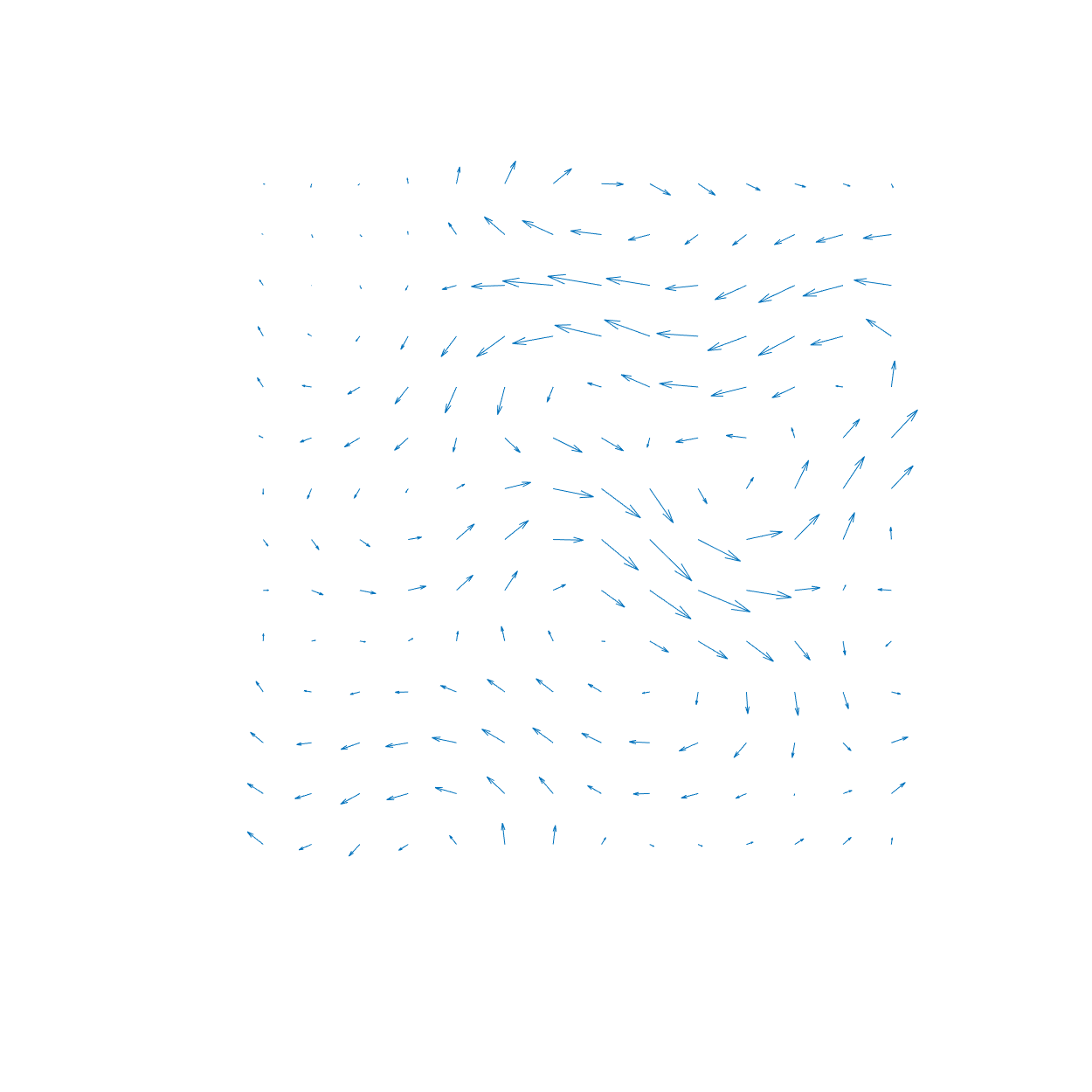

Supplement: S3 MCG raw data 3 — The raw MCG dataset includes category 4 for training and validation. (ZIP) [file pone.0338189.s003.zip › train/4/p10_310_2.png]

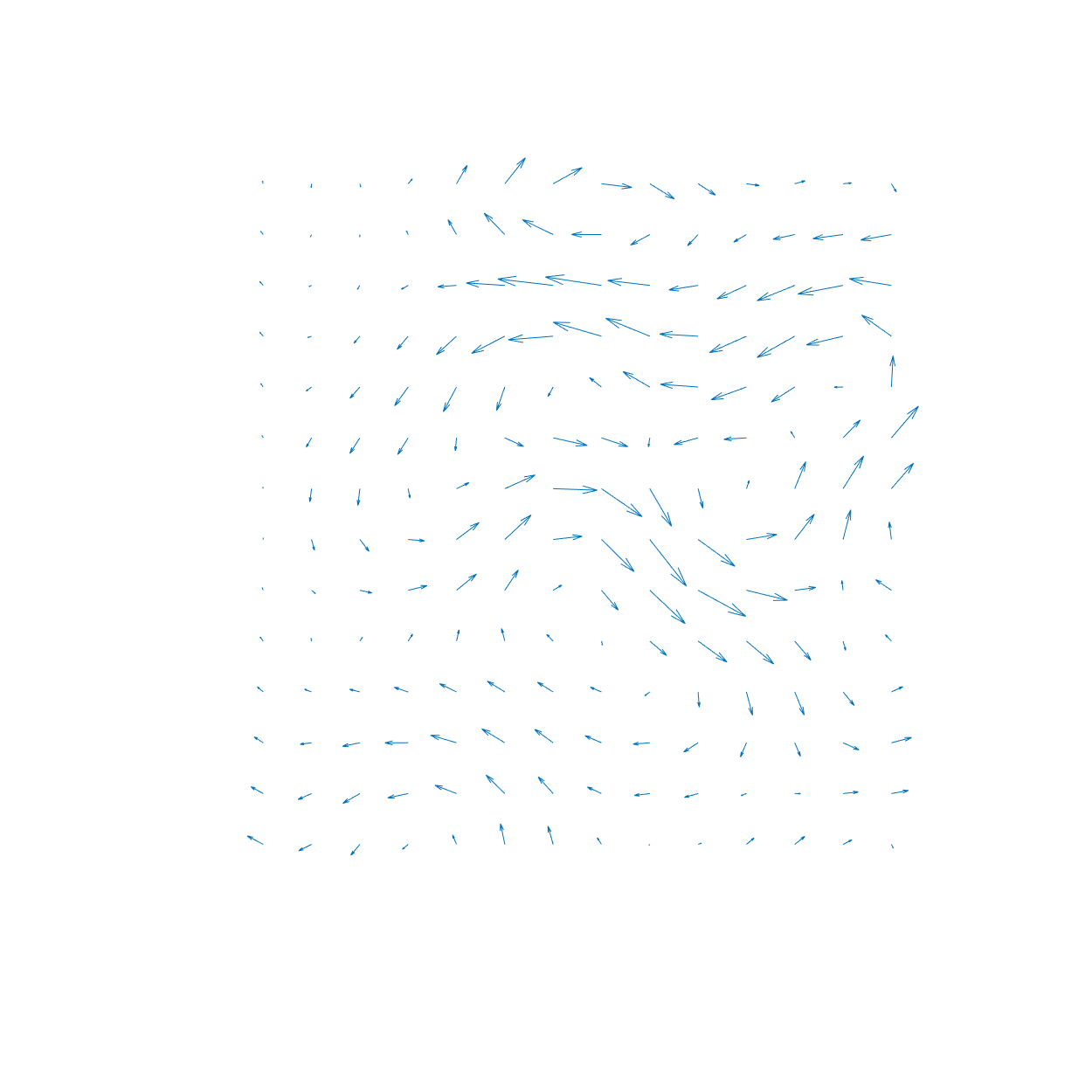

Supplement: S3 MCG raw data 3 — The raw MCG dataset includes category 4 for training and validation. (ZIP) [file pone.0338189.s003.zip › train/4/p10_310_3.png]

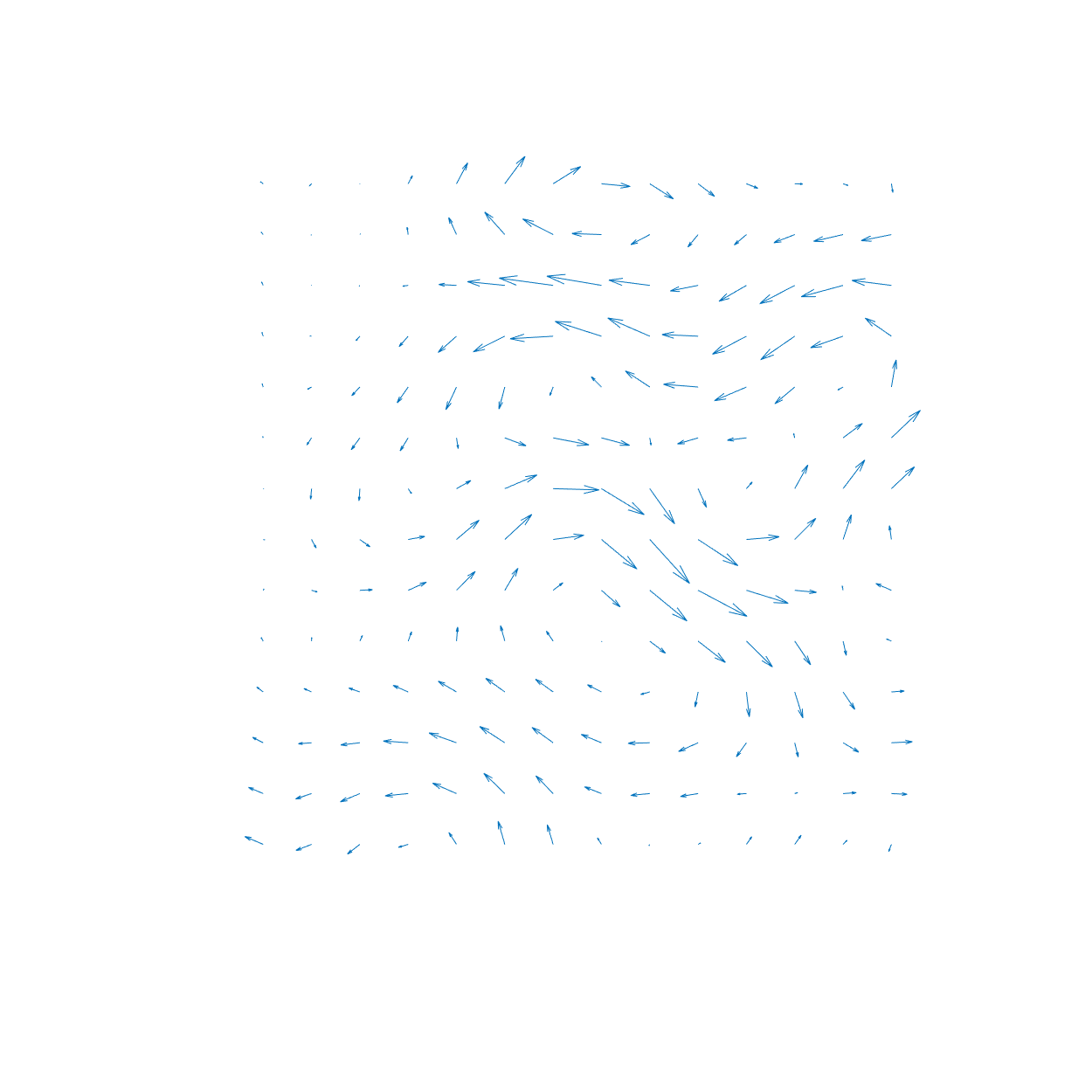

Supplement: S3 MCG raw data 3 — The raw MCG dataset includes category 4 for training and validation. (ZIP) [file pone.0338189.s003.zip › train/4/p10_310_4.png]

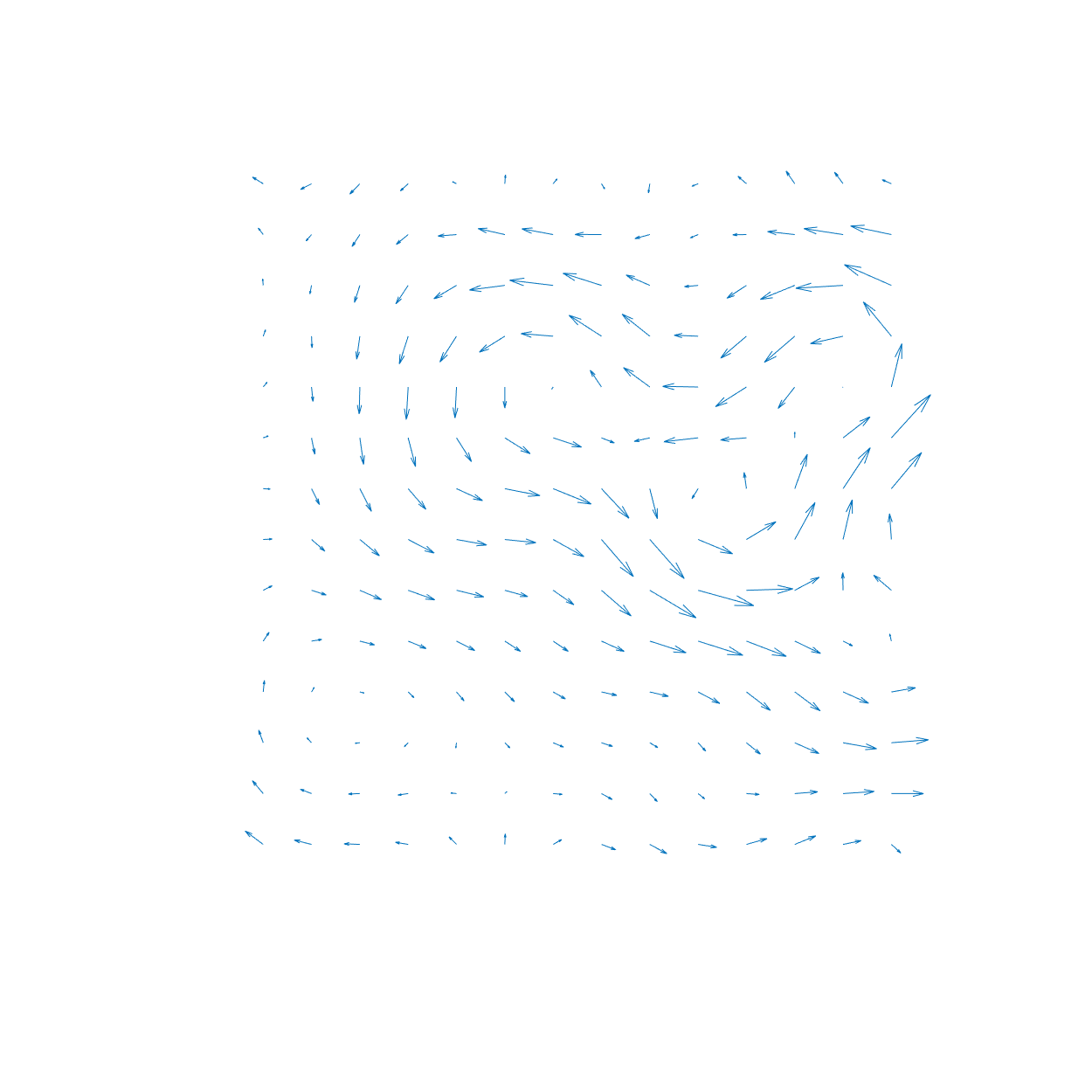

Supplement: S3 MCG raw data 3 — The raw MCG dataset includes category 4 for training and validation. (ZIP) [file pone.0338189.s003.zip › train/4/p10_315_1.png]

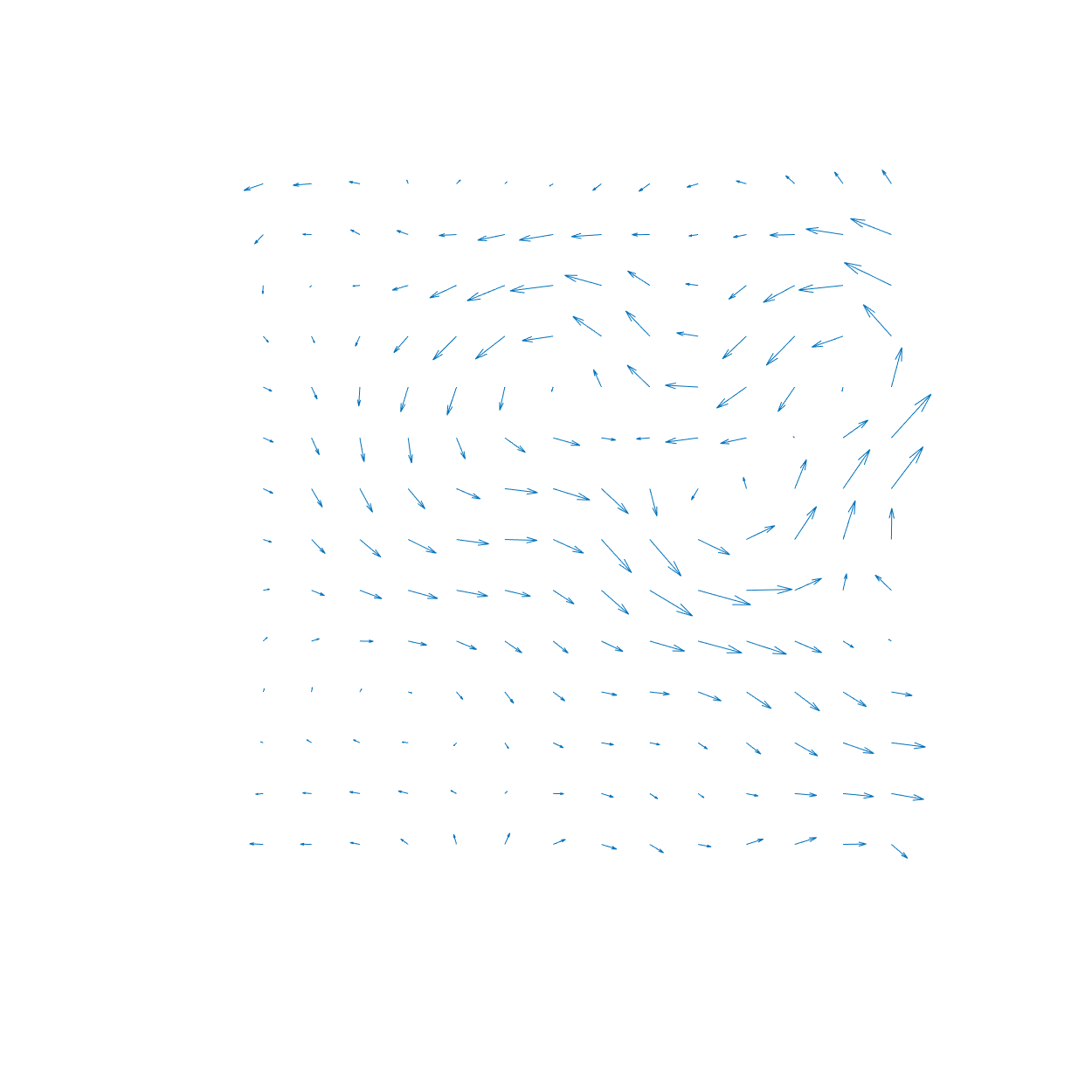

Supplement: S3 MCG raw data 3 — The raw MCG dataset includes category 4 for training and validation. (ZIP) [file pone.0338189.s003.zip › train/4/p10_315_2.png]

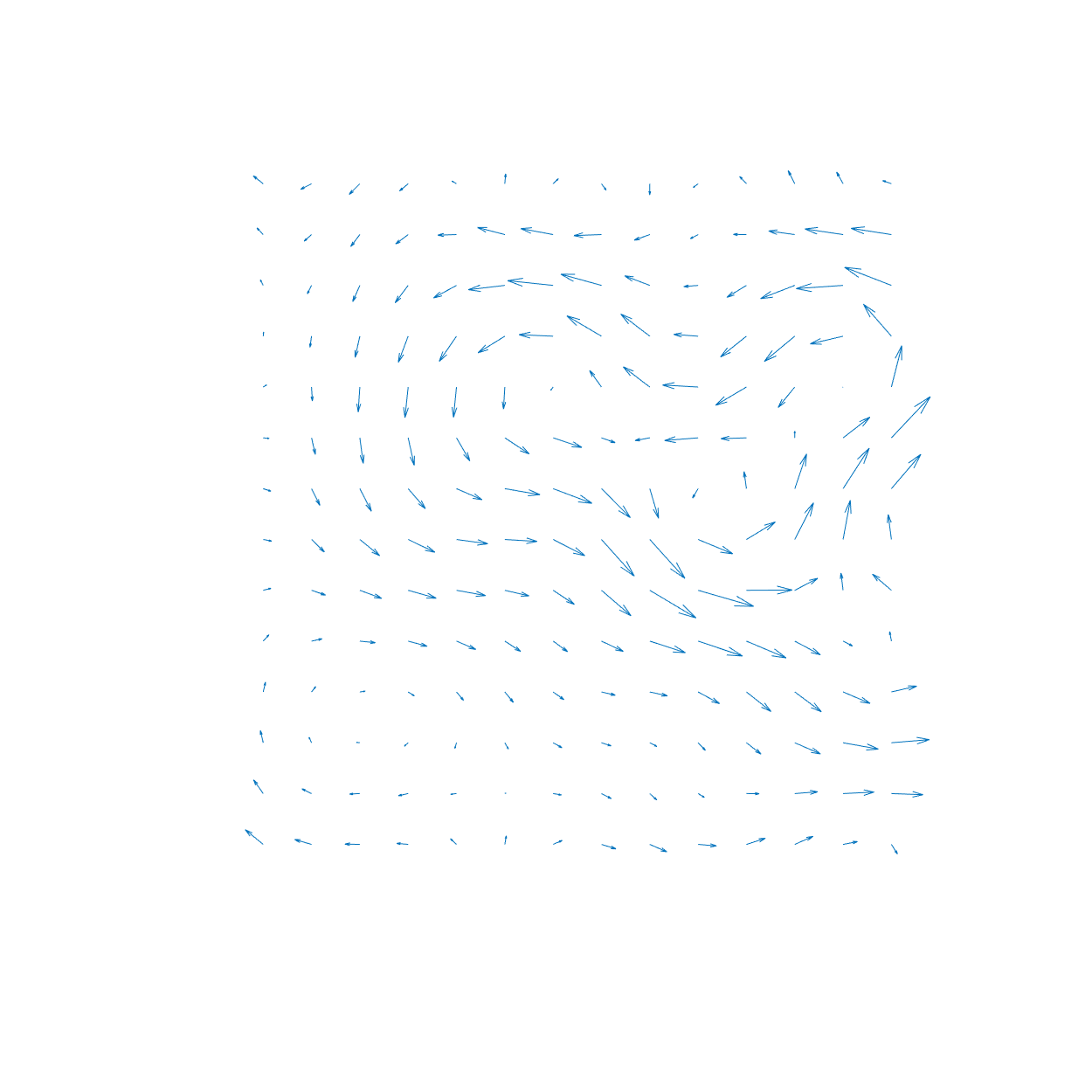

Supplement: S3 MCG raw data 3 — The raw MCG dataset includes category 4 for training and validation. (ZIP) [file pone.0338189.s003.zip › train/4/p10_315_3.png]

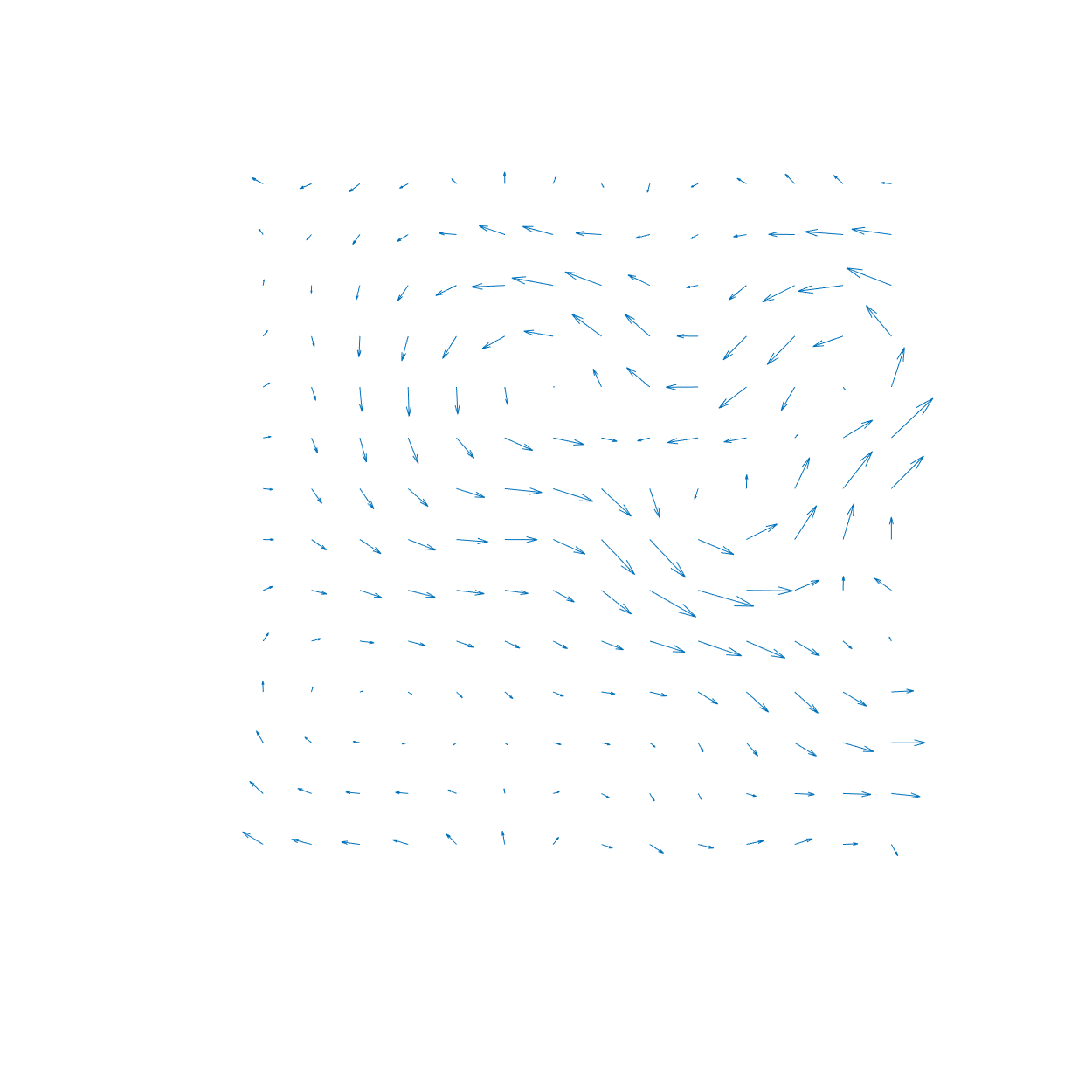

Supplement: S3 MCG raw data 3 — The raw MCG dataset includes category 4 for training and validation. (ZIP) [file pone.0338189.s003.zip › train/4/p10_315_4.png]

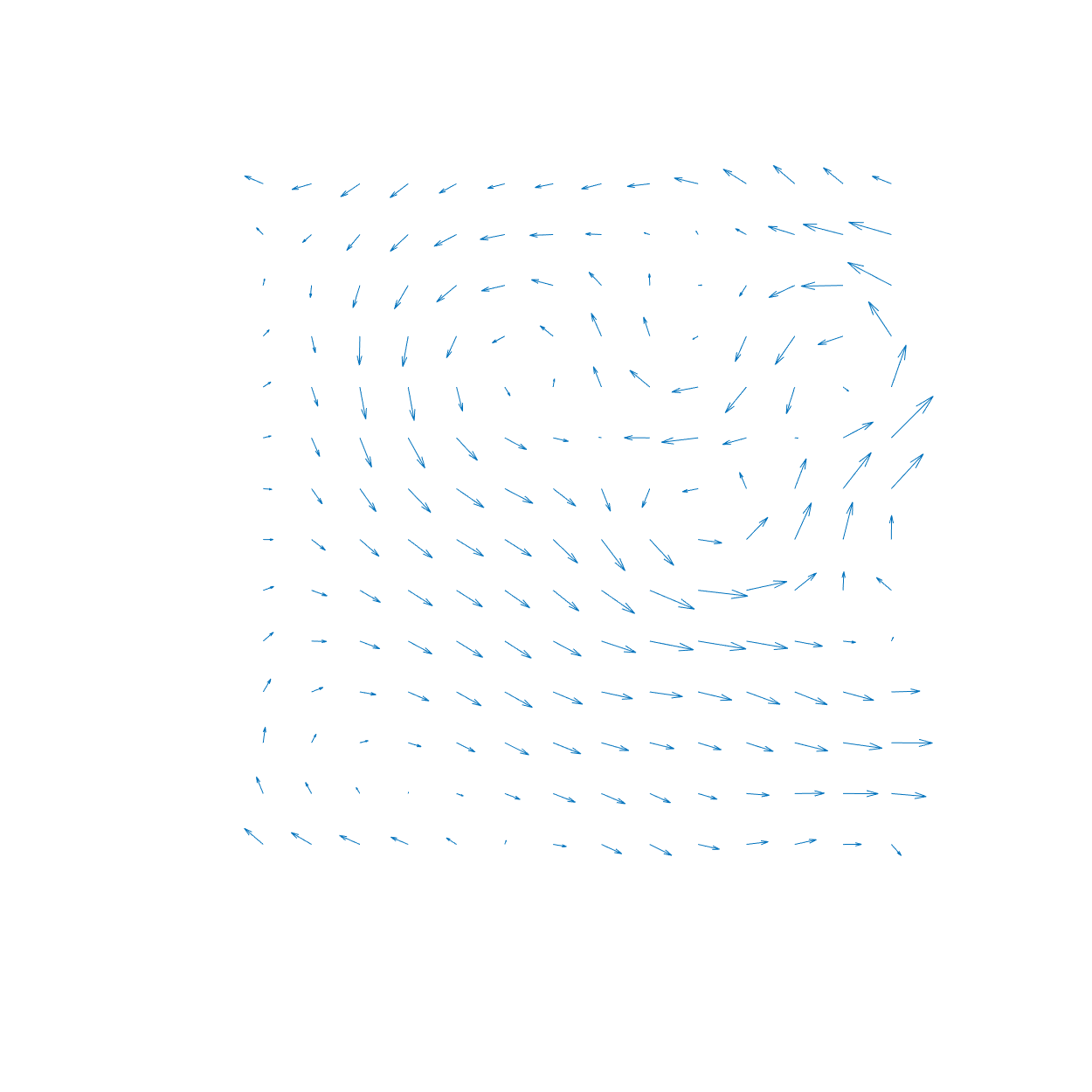

Supplement: S3 MCG raw data 3 — The raw MCG dataset includes category 4 for training and validation. (ZIP) [file pone.0338189.s003.zip › train/4/p10_320_1.png]

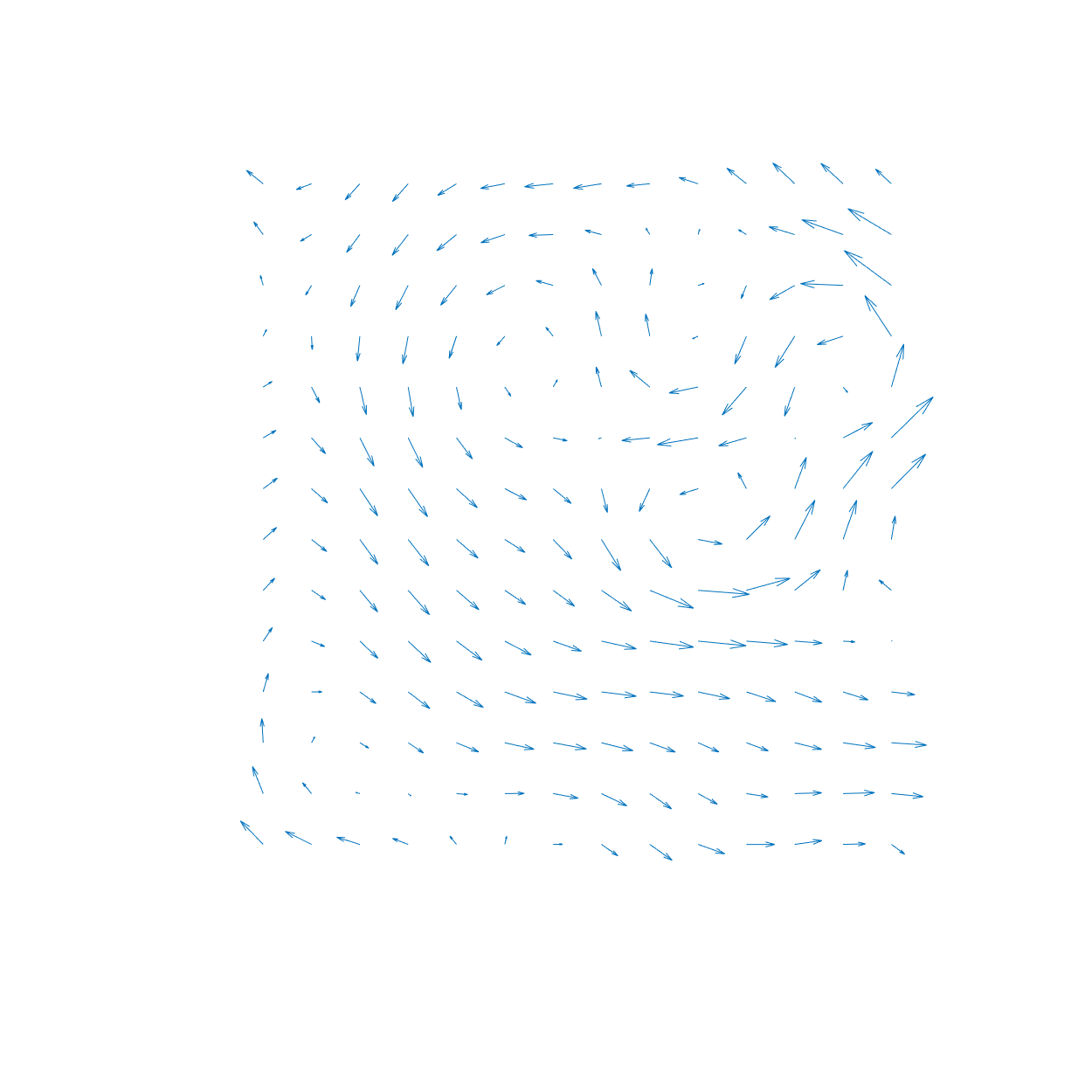

Supplement: S3 MCG raw data 3 — The raw MCG dataset includes category 4 for training and validation. (ZIP) [file pone.0338189.s003.zip › train/4/p10_320_2.png]

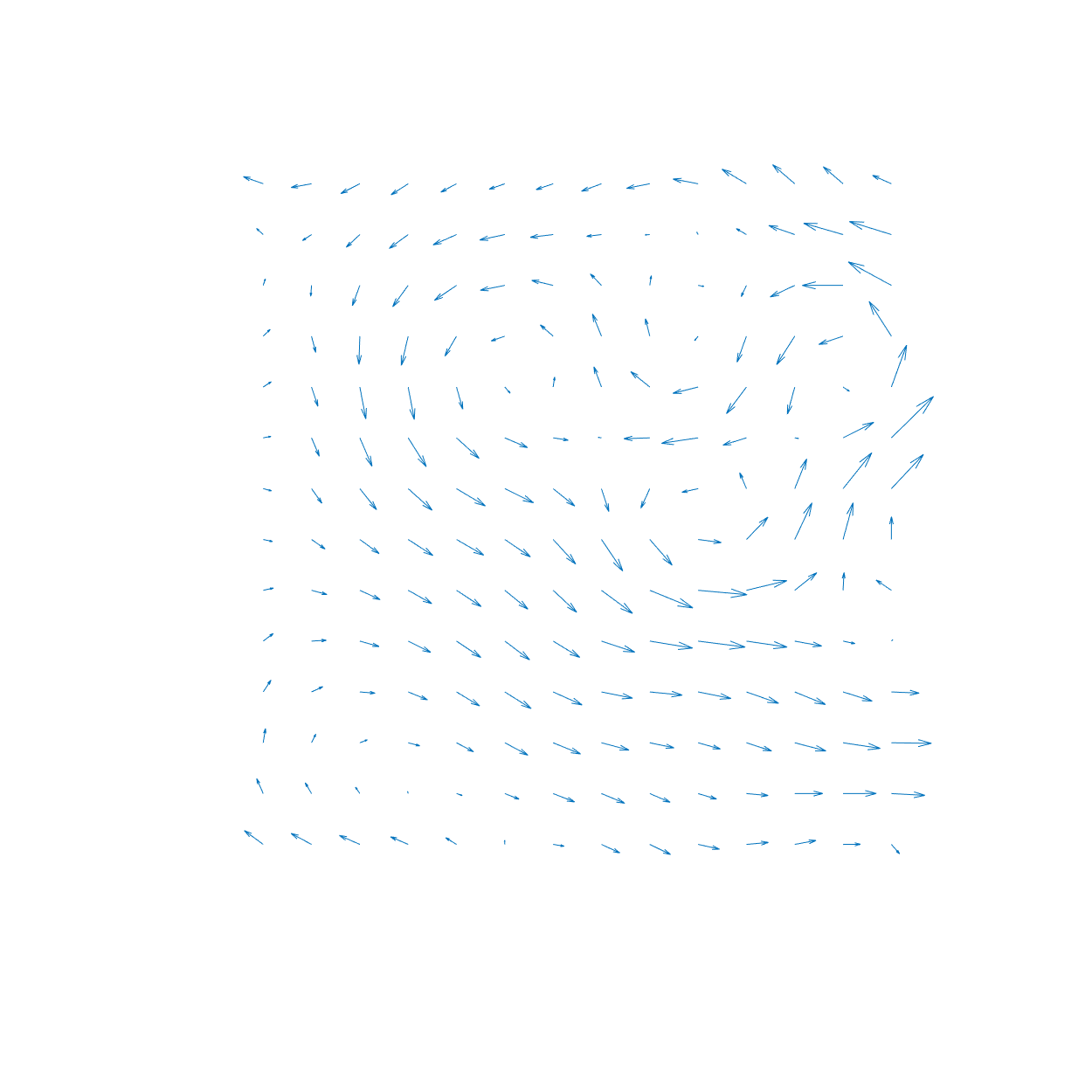

Supplement: S3 MCG raw data 3 — The raw MCG dataset includes category 4 for training and validation. (ZIP) [file pone.0338189.s003.zip › train/4/p10_320_3.png]

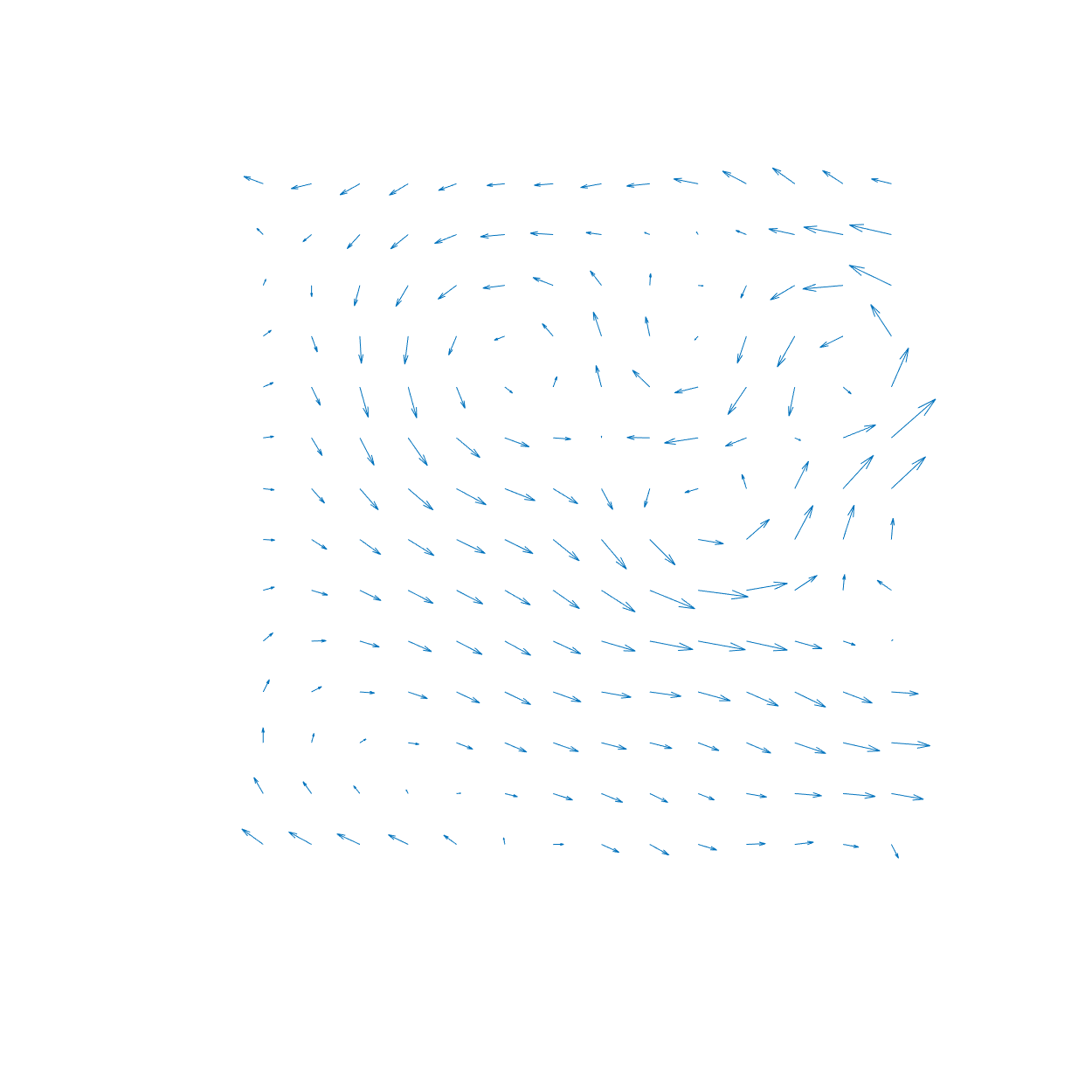

Supplement: S3 MCG raw data 3 — The raw MCG dataset includes category 4 for training and validation. (ZIP) [file pone.0338189.s003.zip › train/4/p10_320_4.png]

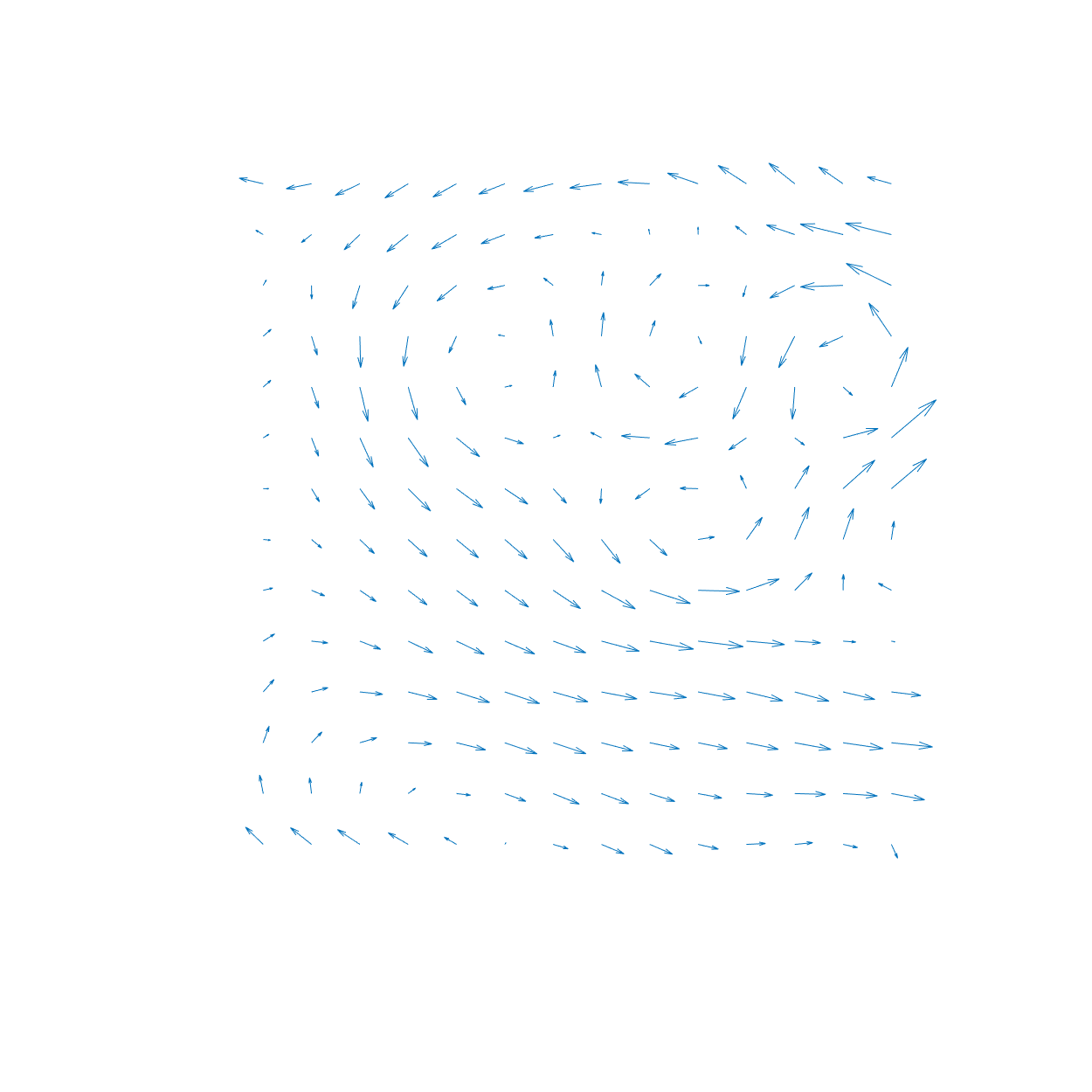

Supplement: S3 MCG raw data 3 — The raw MCG dataset includes category 4 for training and validation. (ZIP) [file pone.0338189.s003.zip › train/4/p10_325_1.png]

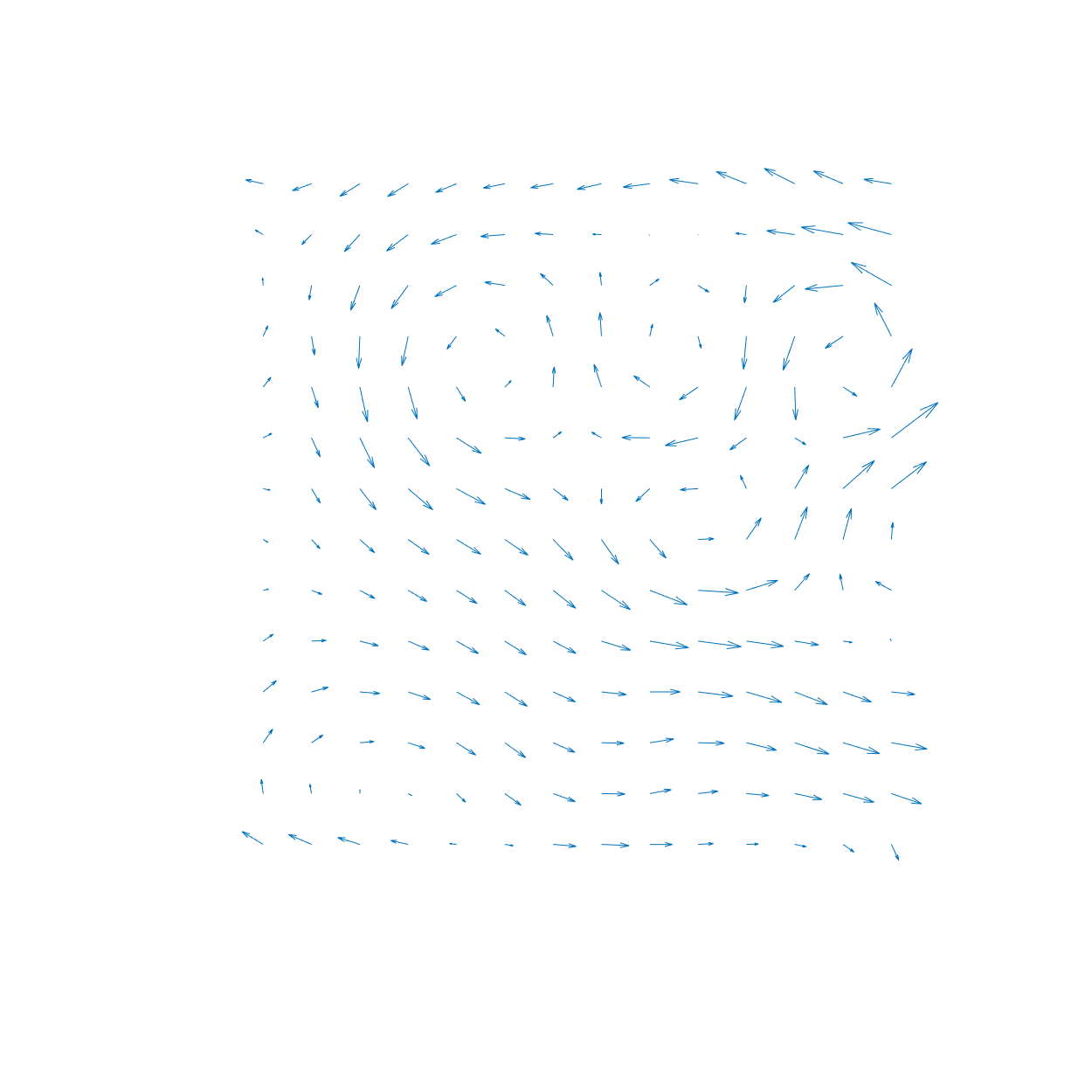

Supplement: S3 MCG raw data 3 — The raw MCG dataset includes category 4 for training and validation. (ZIP) [file pone.0338189.s003.zip › train/4/p10_325_2.png]

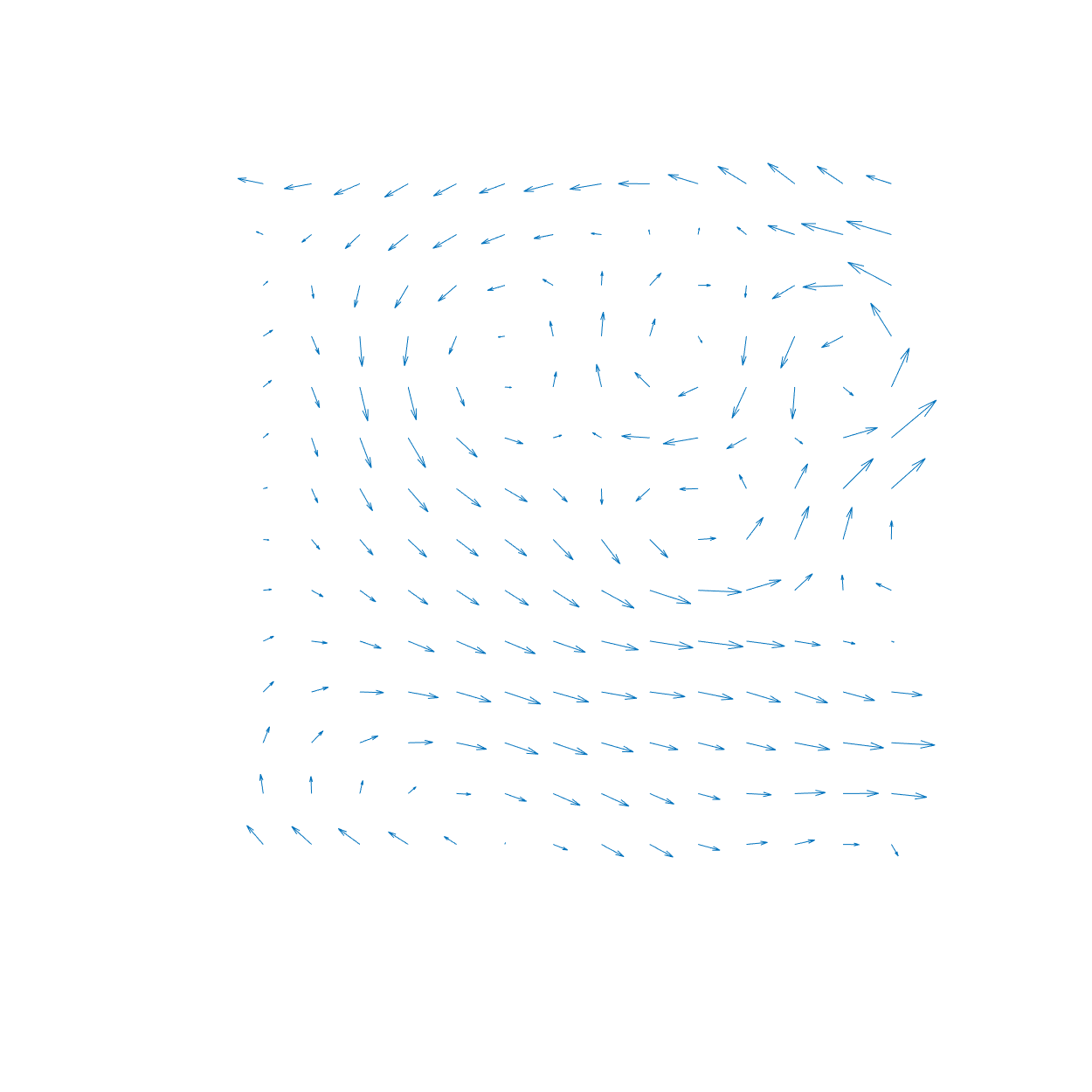

Supplement: S3 MCG raw data 3 — The raw MCG dataset includes category 4 for training and validation. (ZIP) [file pone.0338189.s003.zip › train/4/p10_325_3.png]

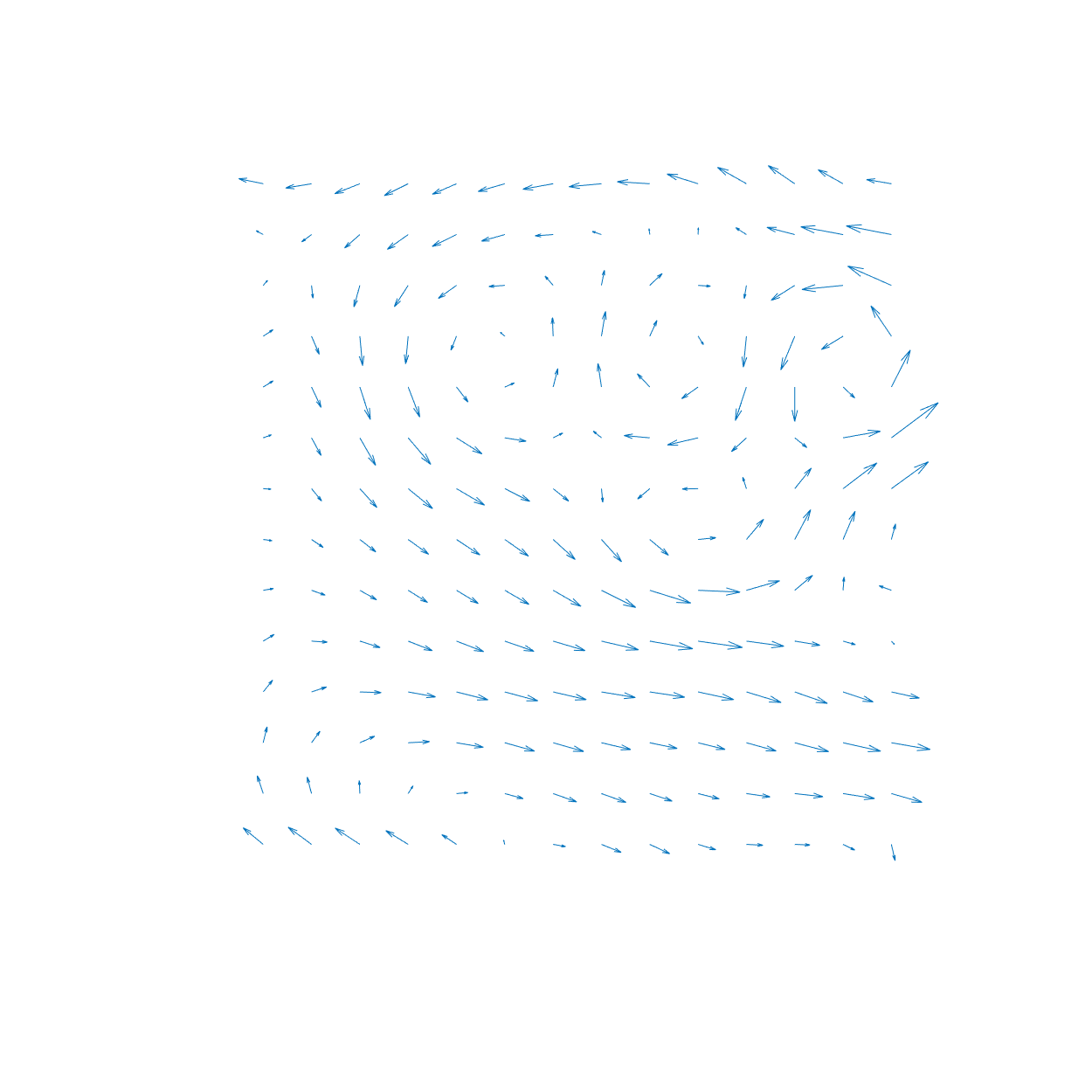

Supplement: S3 MCG raw data 3 — The raw MCG dataset includes category 4 for training and validation. (ZIP) [file pone.0338189.s003.zip › train/4/p10_325_4.png]

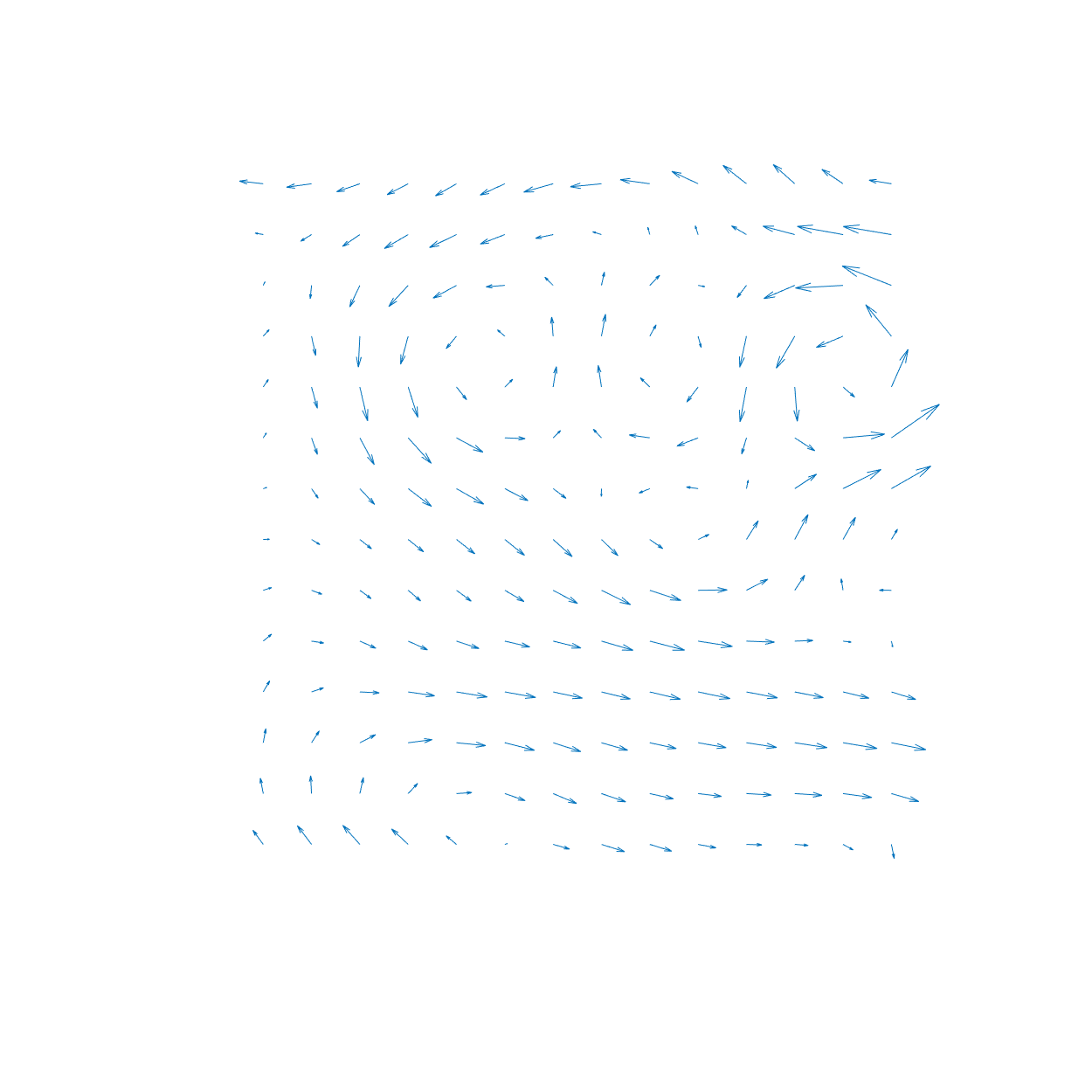

Supplement: S3 MCG raw data 3 — The raw MCG dataset includes category 4 for training and validation. (ZIP) [file pone.0338189.s003.zip › train/4/p10_330_1.png]

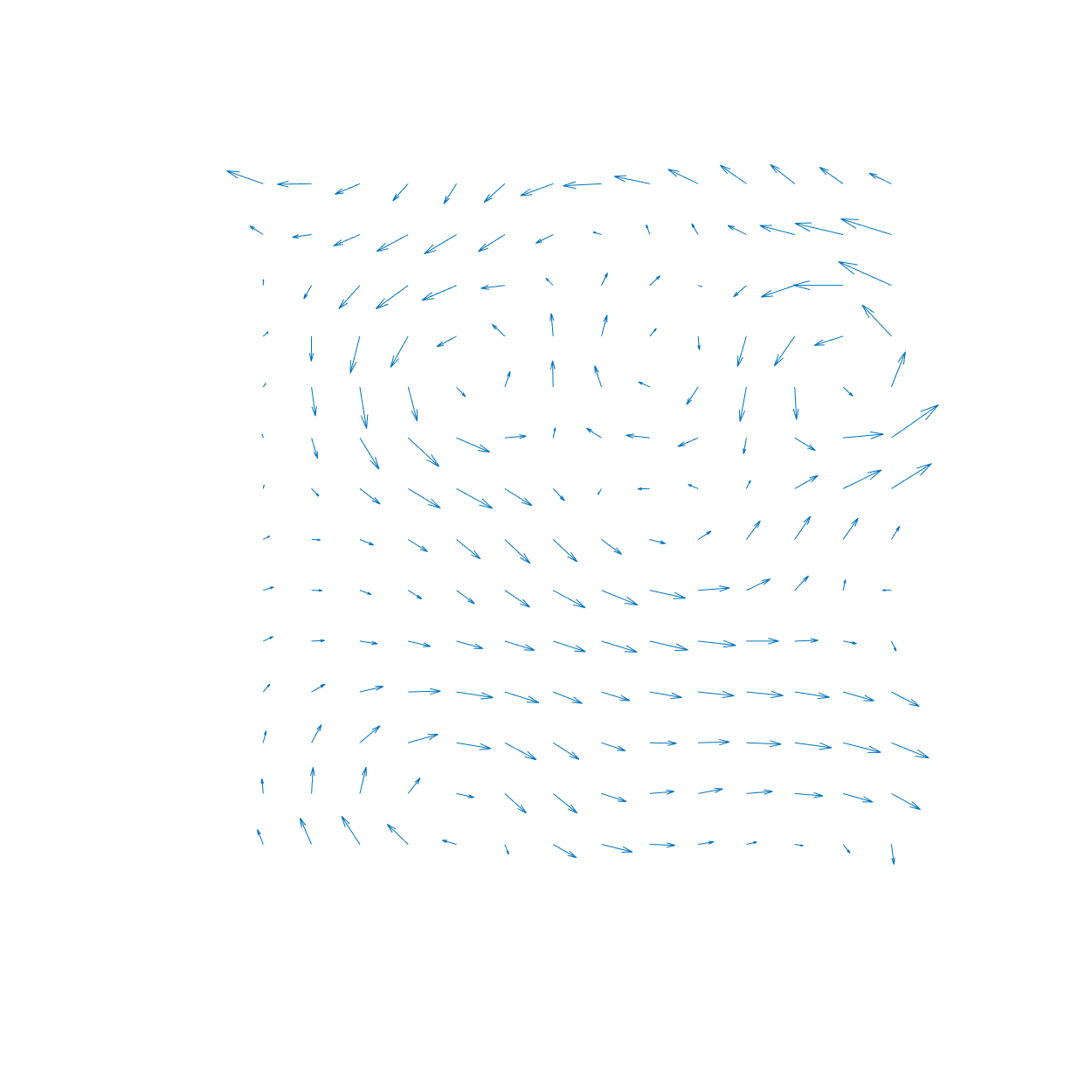

Supplement: S3 MCG raw data 3 — The raw MCG dataset includes category 4 for training and validation. (ZIP) [file pone.0338189.s003.zip › train/4/p10_330_2.png]
