# Supplementary figures and images for: Classification of current density vector map using transformer hybrid residual network (part 3 of 6)
Source: PLoS One. 2025 Dec 16;20(12):e0338189. doi: 10.1371/journal.pone.0338189 (PMC12707687; doi:10.1371/journal.pone.0338189)

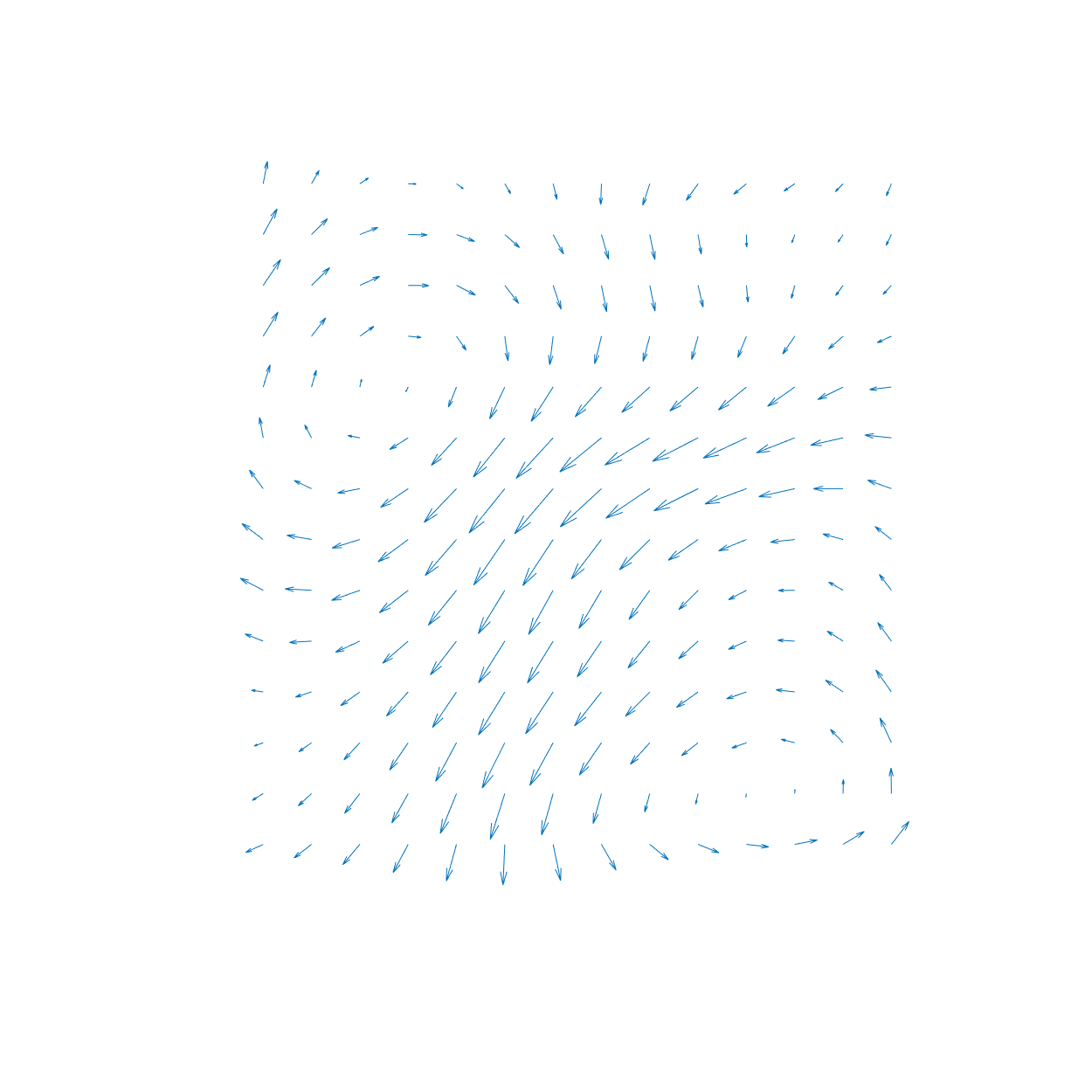

Supplement: S2 MCG raw data 2 — The raw MCG dataset includes categories 0-3 for training and validation. (ZIP) [file pone.0338189.s002.zip › train/0/p11_245_1.png]

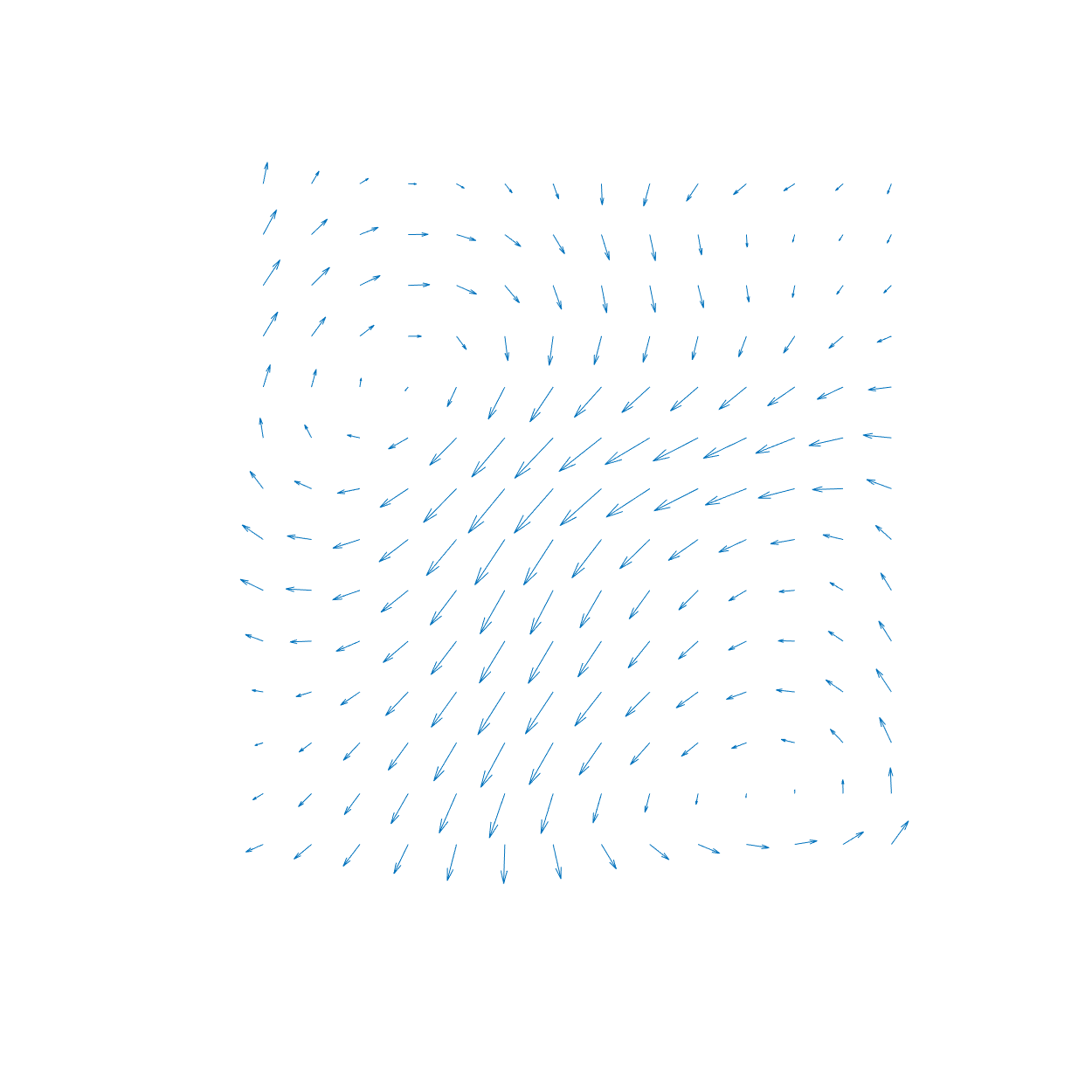

Supplement: S2 MCG raw data 2 — The raw MCG dataset includes categories 0-3 for training and validation. (ZIP) [file pone.0338189.s002.zip › train/0/p11_245_2.png]

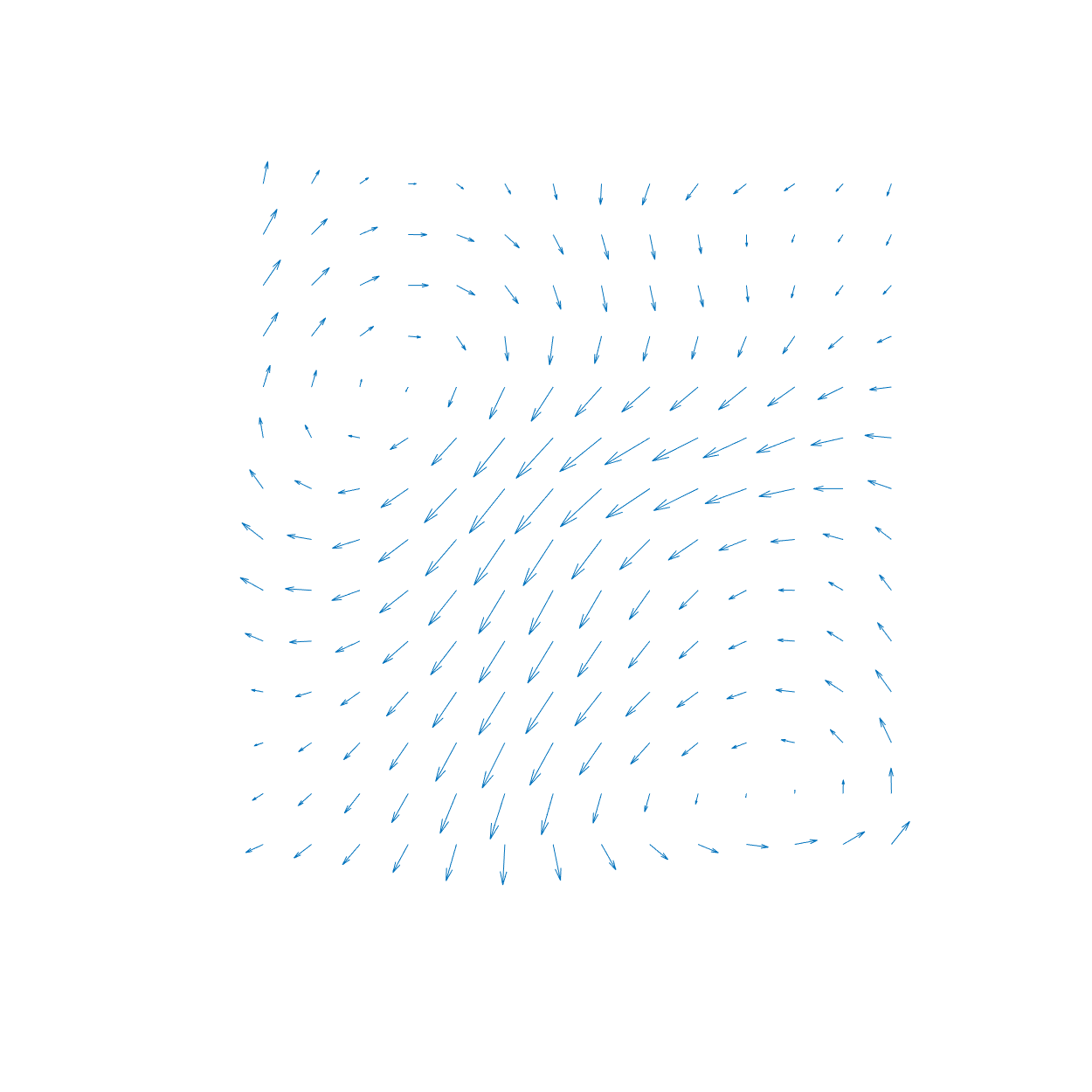

Supplement: S2 MCG raw data 2 — The raw MCG dataset includes categories 0-3 for training and validation. (ZIP) [file pone.0338189.s002.zip › train/0/p11_245_3.png]

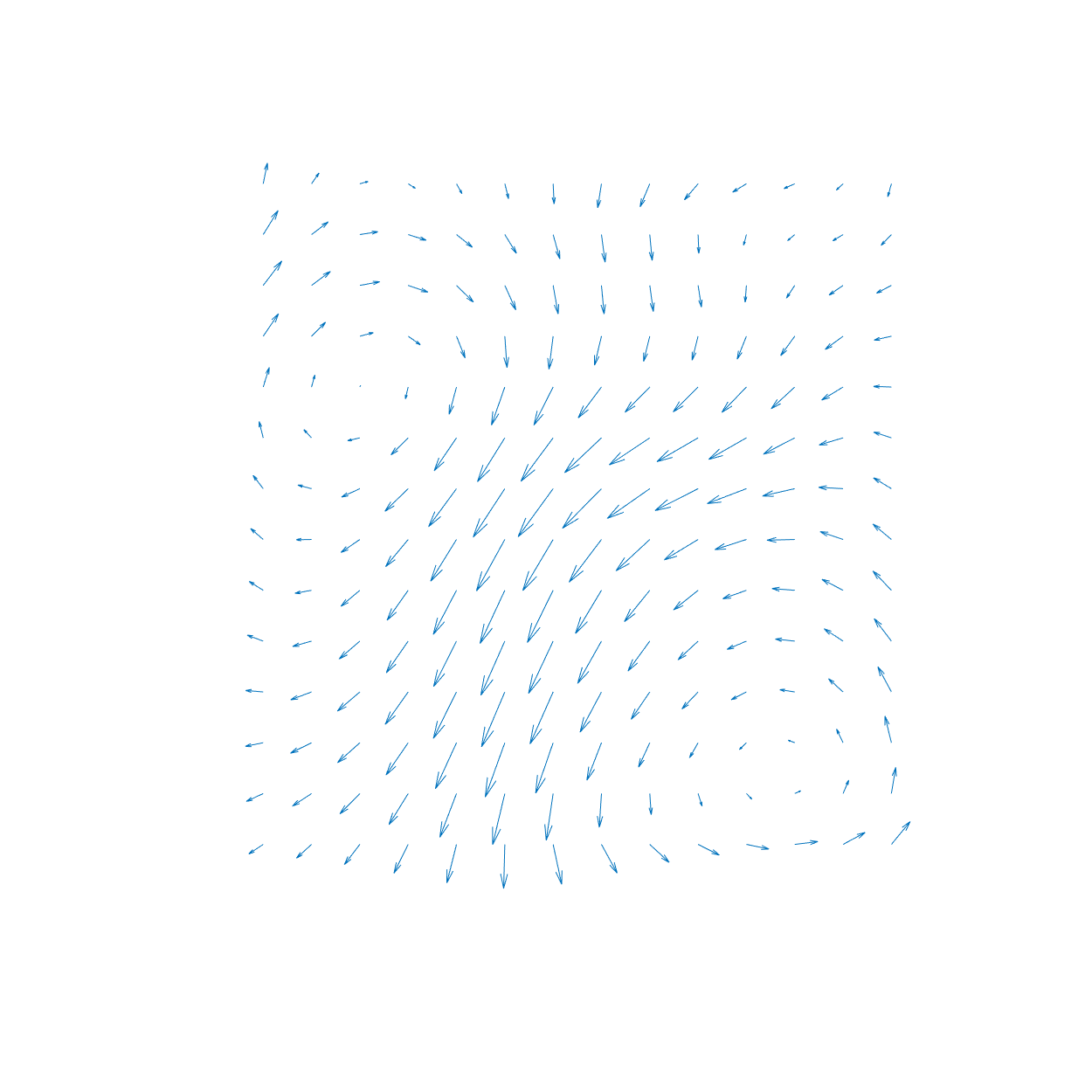

Supplement: S2 MCG raw data 2 — The raw MCG dataset includes categories 0-3 for training and validation. (ZIP) [file pone.0338189.s002.zip › train/0/p11_250_1.png]

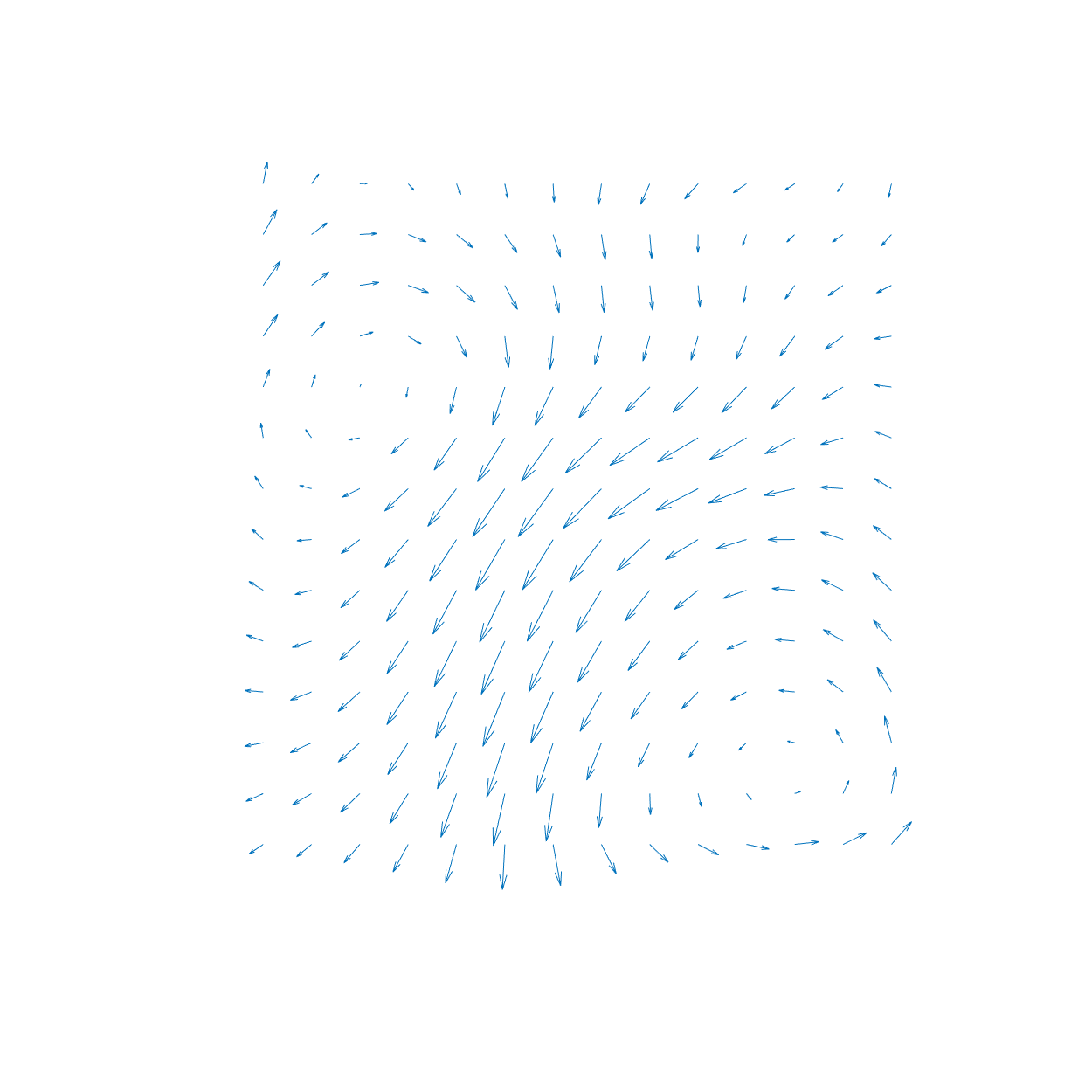

Supplement: S2 MCG raw data 2 — The raw MCG dataset includes categories 0-3 for training and validation. (ZIP) [file pone.0338189.s002.zip › train/0/p11_250_2.png]

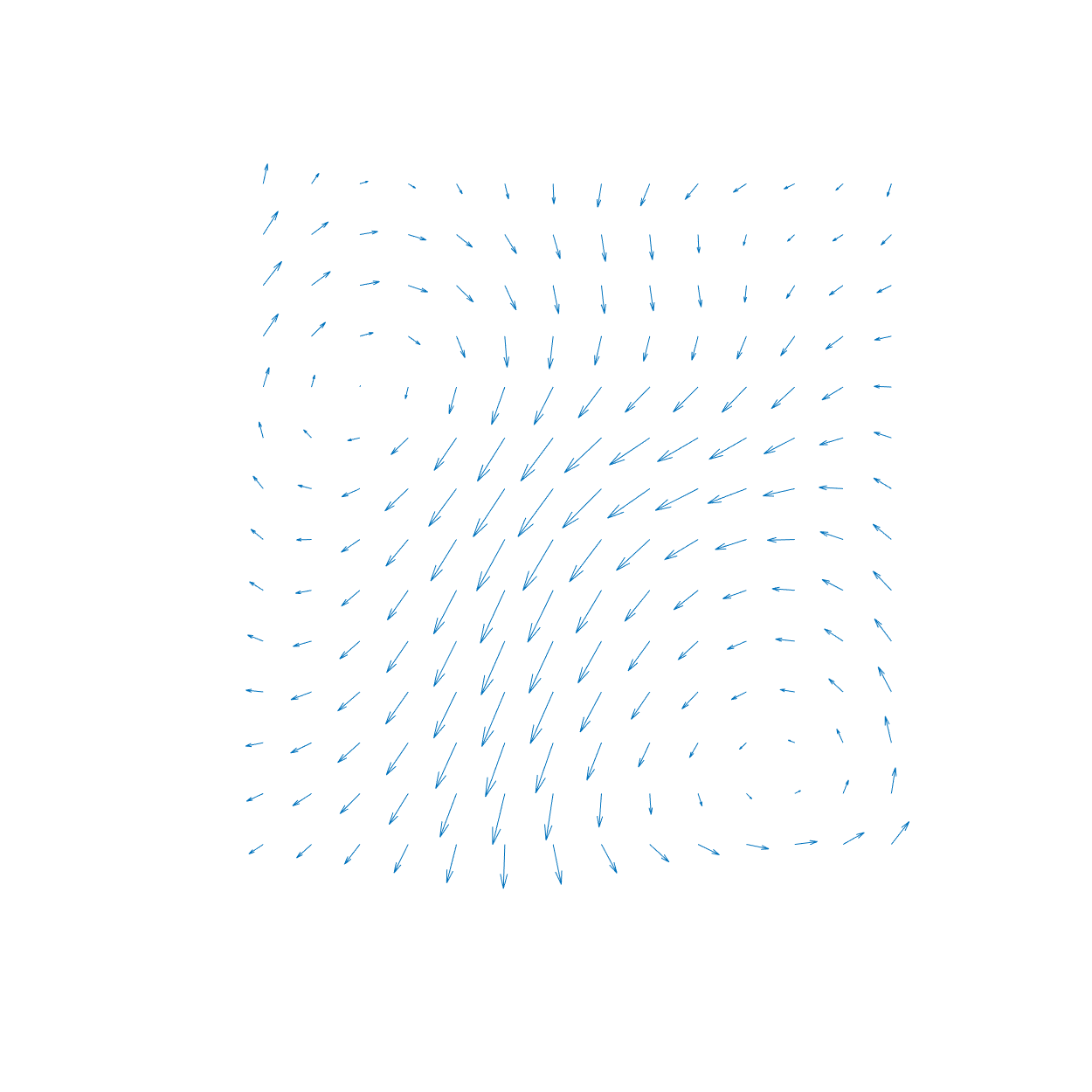

Supplement: S2 MCG raw data 2 — The raw MCG dataset includes categories 0-3 for training and validation. (ZIP) [file pone.0338189.s002.zip › train/0/p11_250_3.png]

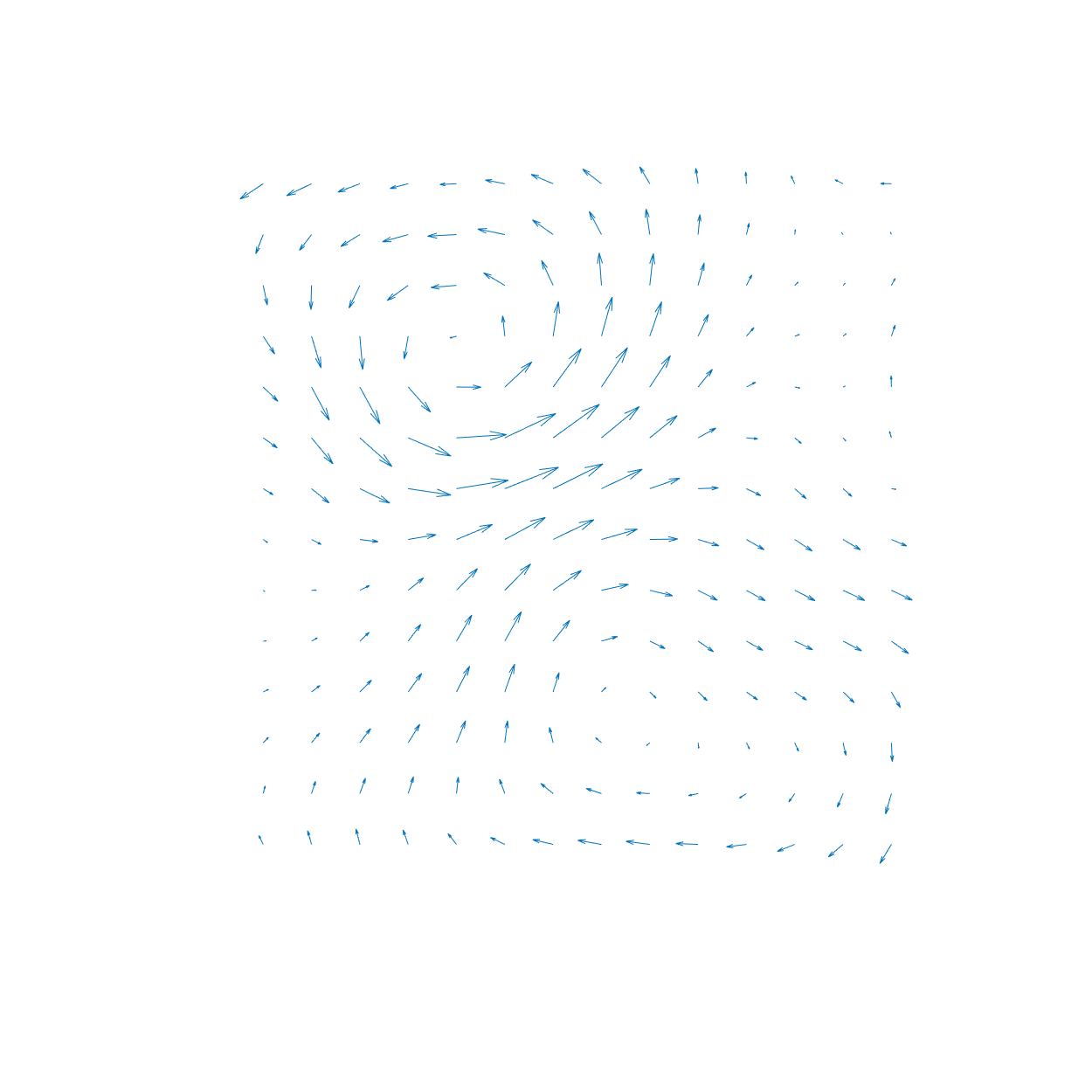

Supplement: S2 MCG raw data 2 — The raw MCG dataset includes categories 0-3 for training and validation. (ZIP) [file pone.0338189.s002.zip › train/0/p2_365_1.png]

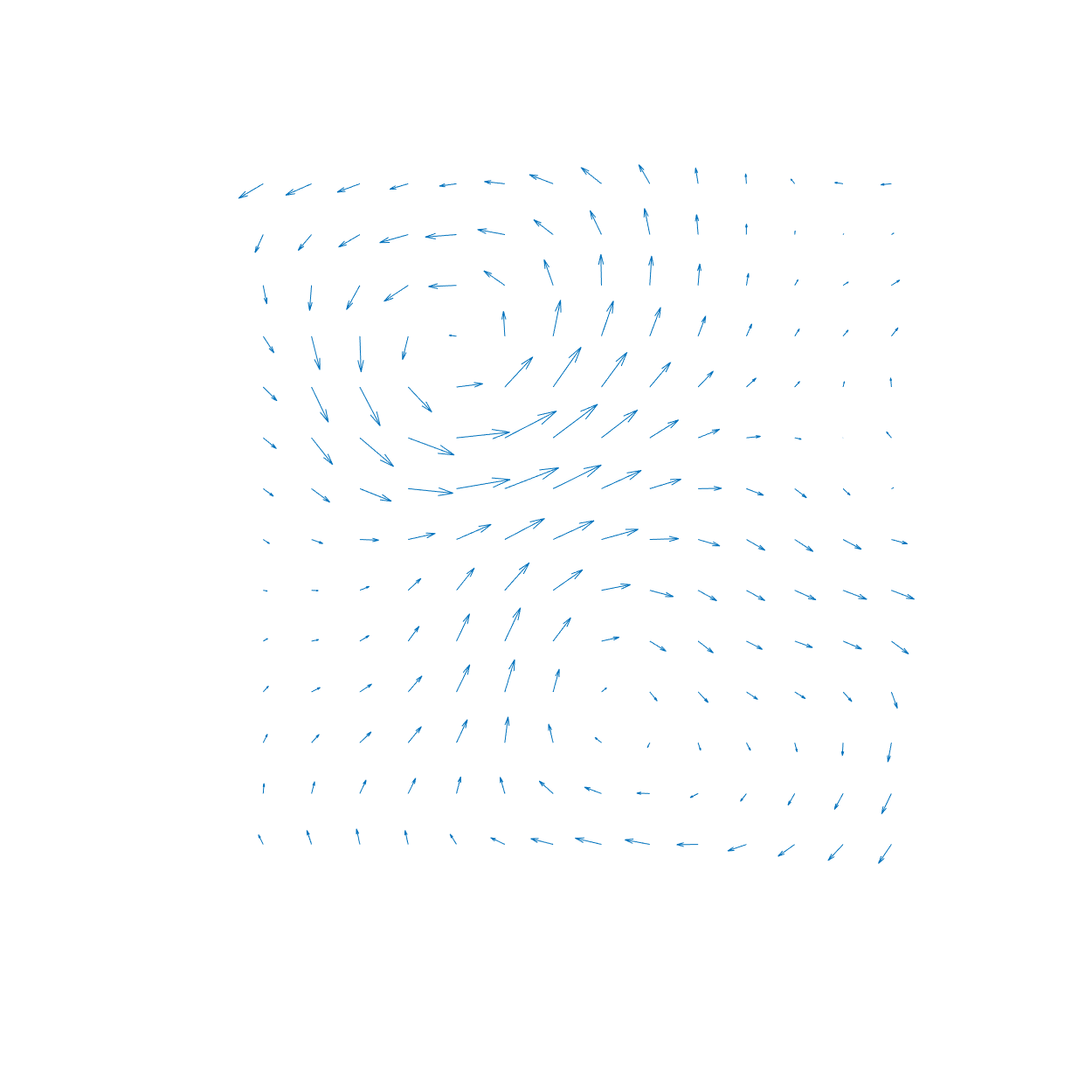

Supplement: S2 MCG raw data 2 — The raw MCG dataset includes categories 0-3 for training and validation. (ZIP) [file pone.0338189.s002.zip › train/0/p2_365_2.png]

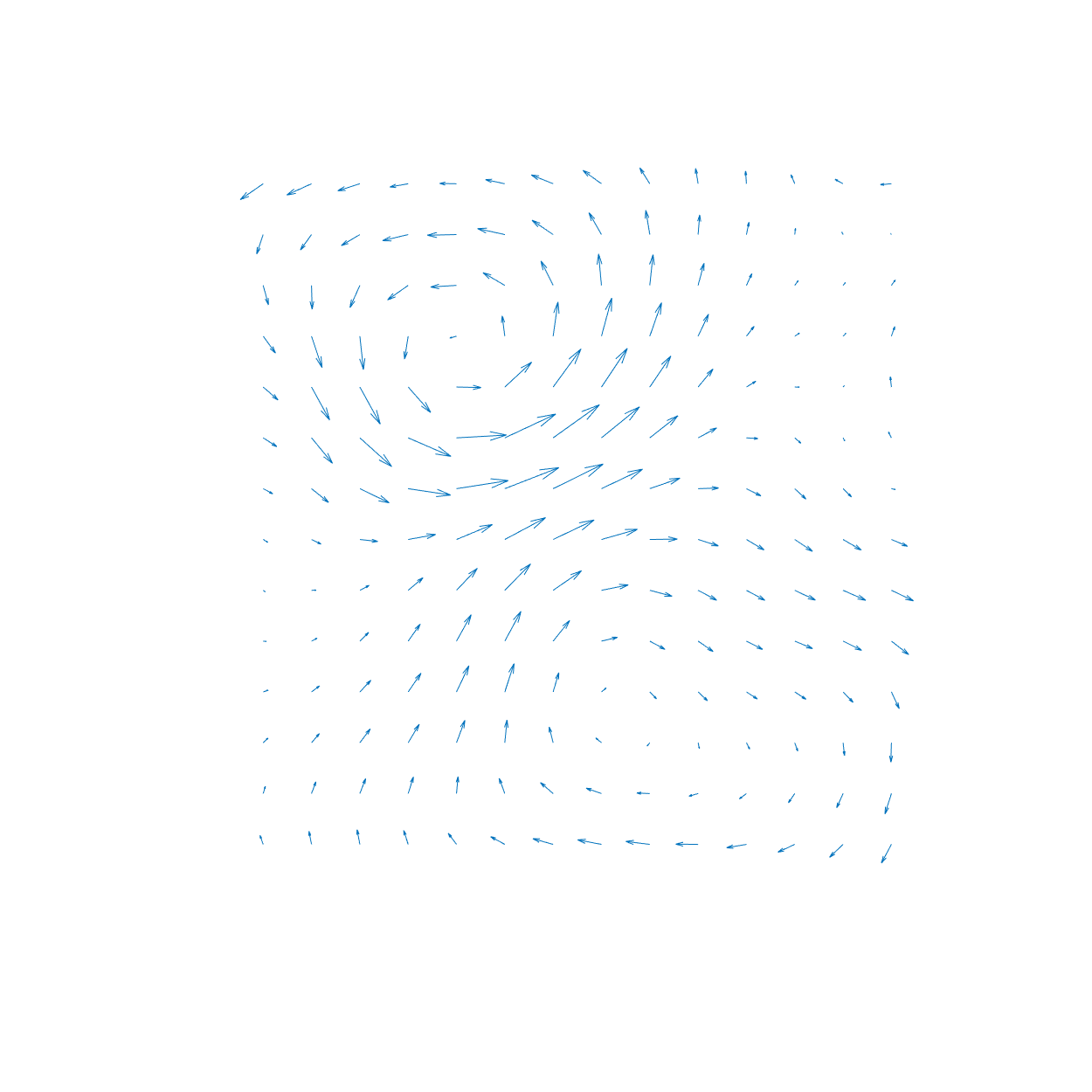

Supplement: S2 MCG raw data 2 — The raw MCG dataset includes categories 0-3 for training and validation. (ZIP) [file pone.0338189.s002.zip › train/0/p2_365_3.png]

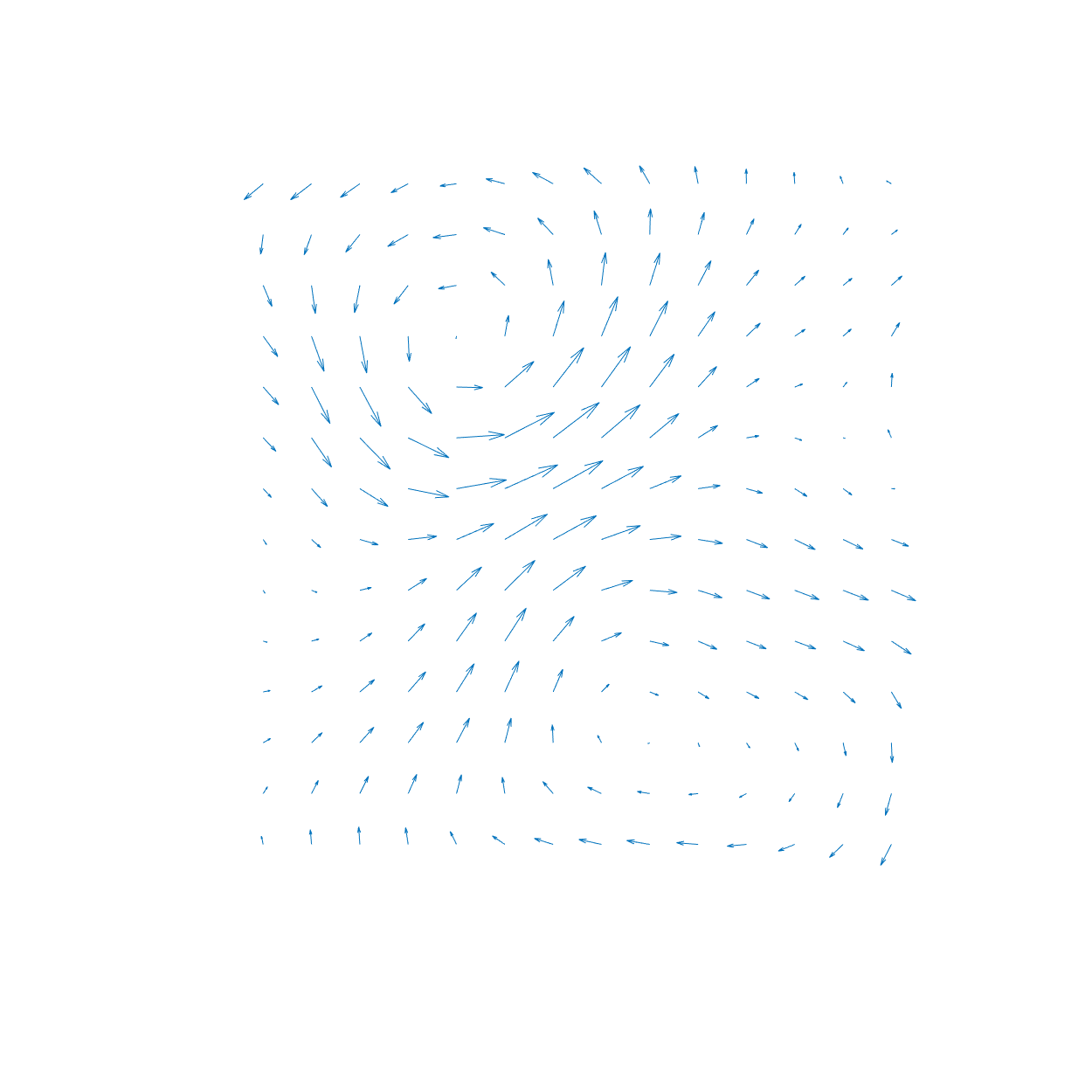

Supplement: S2 MCG raw data 2 — The raw MCG dataset includes categories 0-3 for training and validation. (ZIP) [file pone.0338189.s002.zip › train/0/p2_370_1.png]

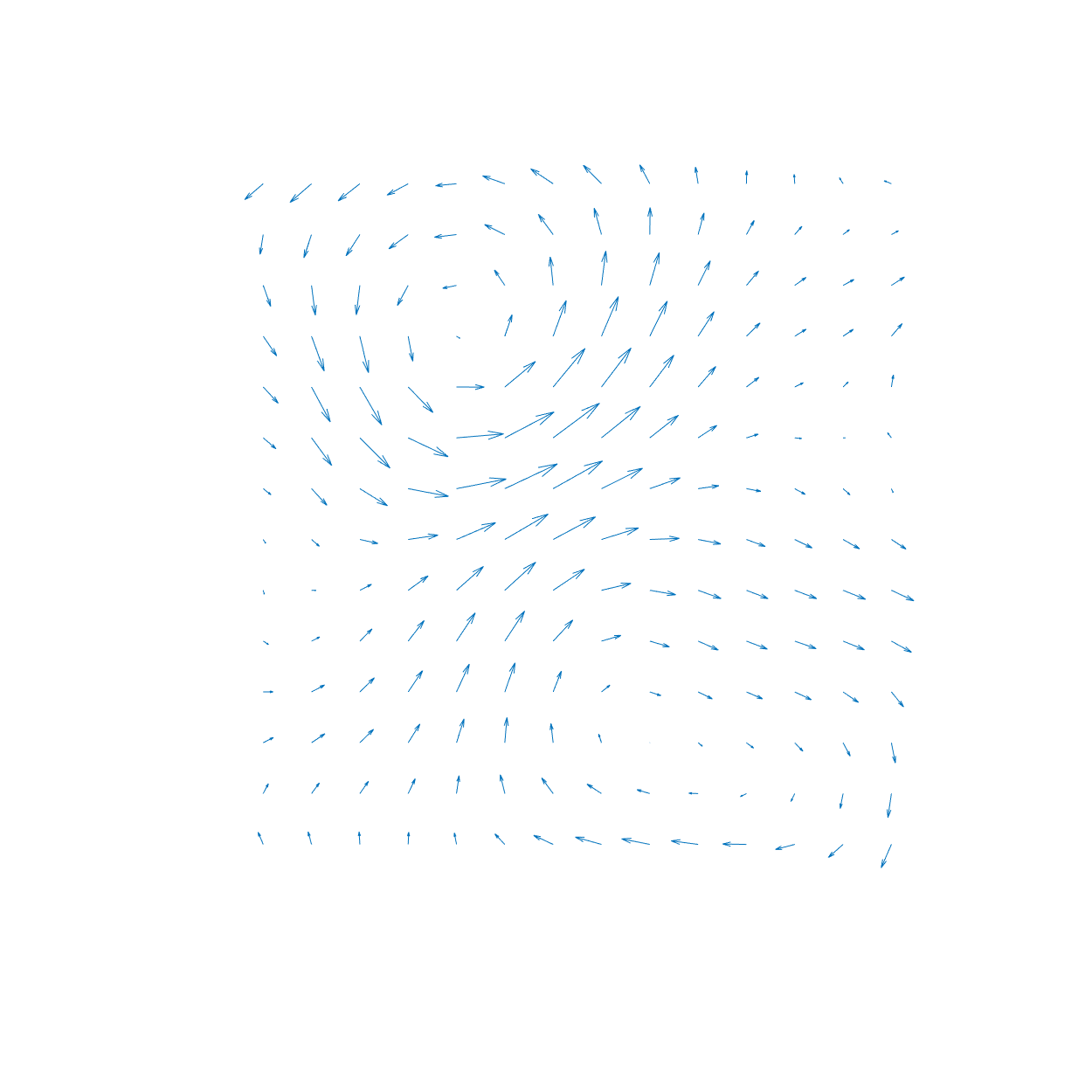

Supplement: S2 MCG raw data 2 — The raw MCG dataset includes categories 0-3 for training and validation. (ZIP) [file pone.0338189.s002.zip › train/0/p2_370_2.png]

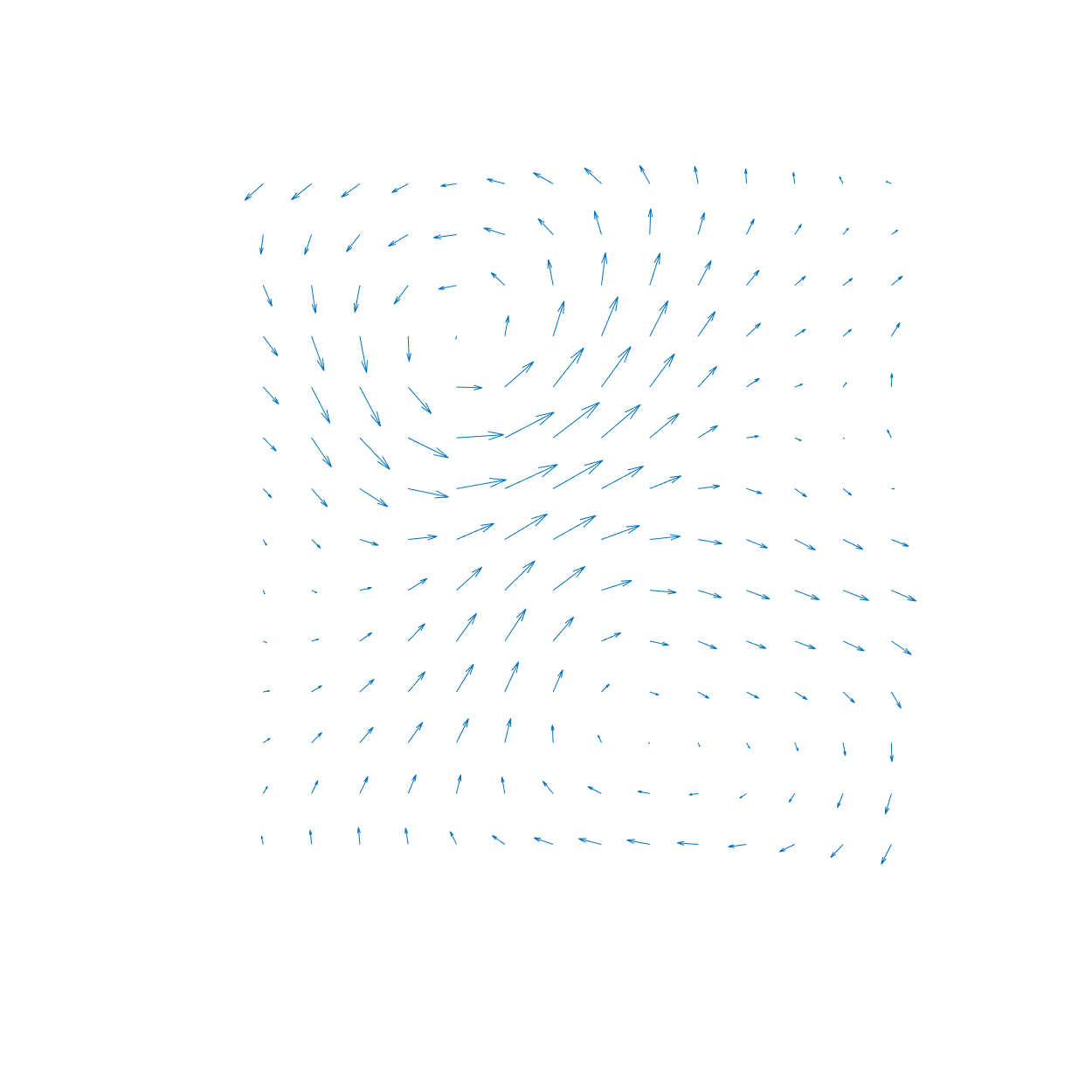

Supplement: S2 MCG raw data 2 — The raw MCG dataset includes categories 0-3 for training and validation. (ZIP) [file pone.0338189.s002.zip › train/0/p2_370_3.png]

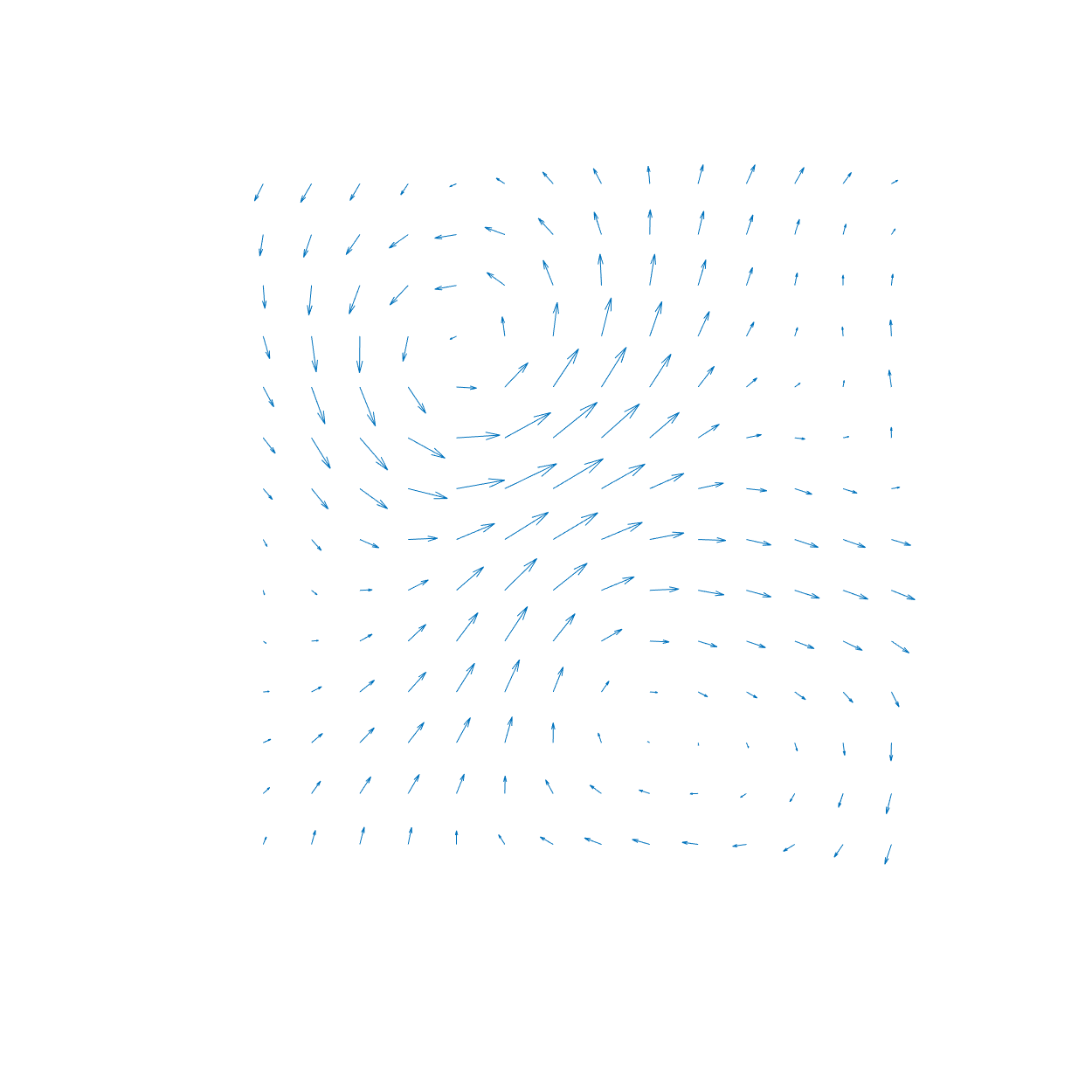

Supplement: S2 MCG raw data 2 — The raw MCG dataset includes categories 0-3 for training and validation. (ZIP) [file pone.0338189.s002.zip › train/0/p2_375_1.png]

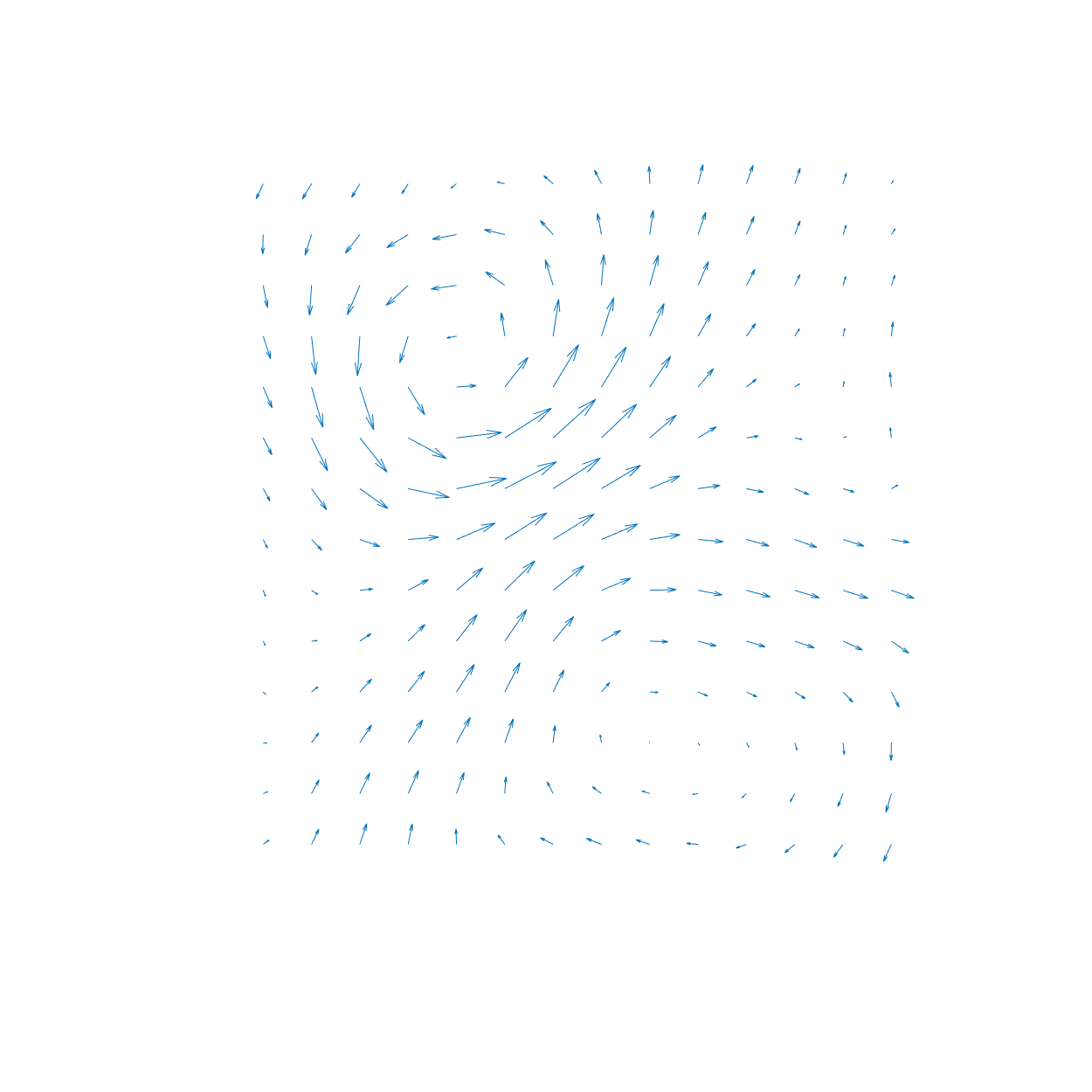

Supplement: S2 MCG raw data 2 — The raw MCG dataset includes categories 0-3 for training and validation. (ZIP) [file pone.0338189.s002.zip › train/0/p2_375_2.png]

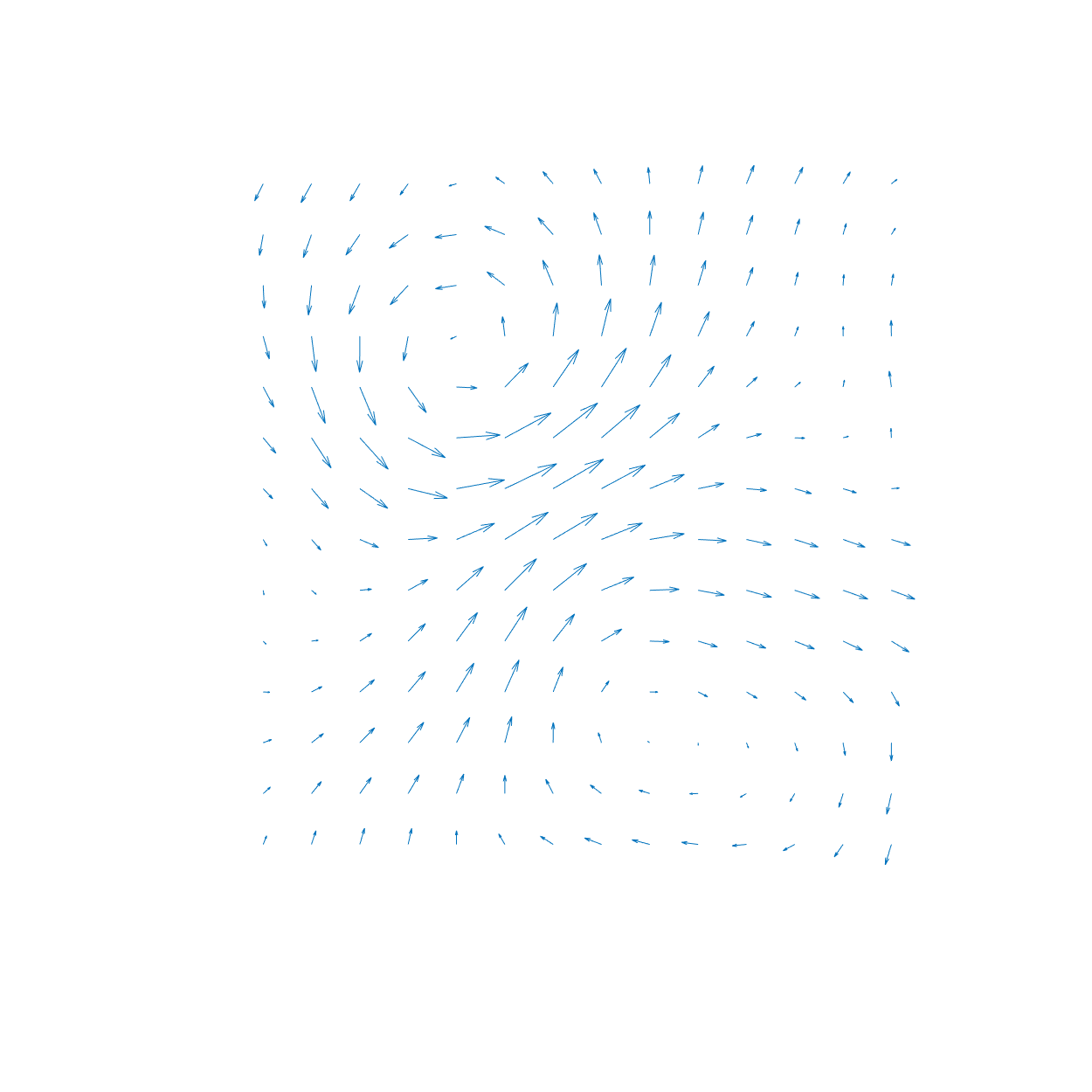

Supplement: S2 MCG raw data 2 — The raw MCG dataset includes categories 0-3 for training and validation. (ZIP) [file pone.0338189.s002.zip › train/0/p2_375_3.png]

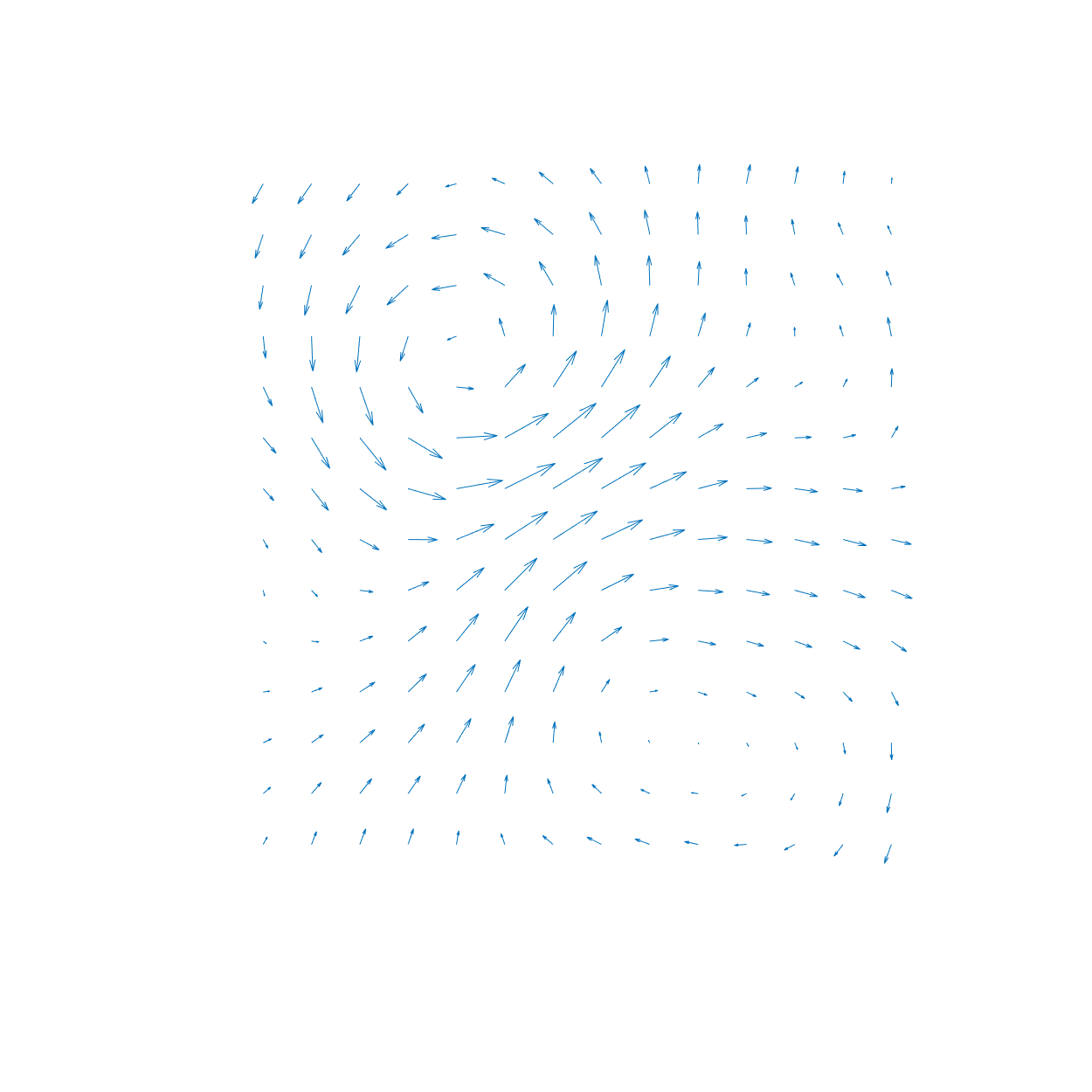

Supplement: S2 MCG raw data 2 — The raw MCG dataset includes categories 0-3 for training and validation. (ZIP) [file pone.0338189.s002.zip › train/0/p2_380_1.png]

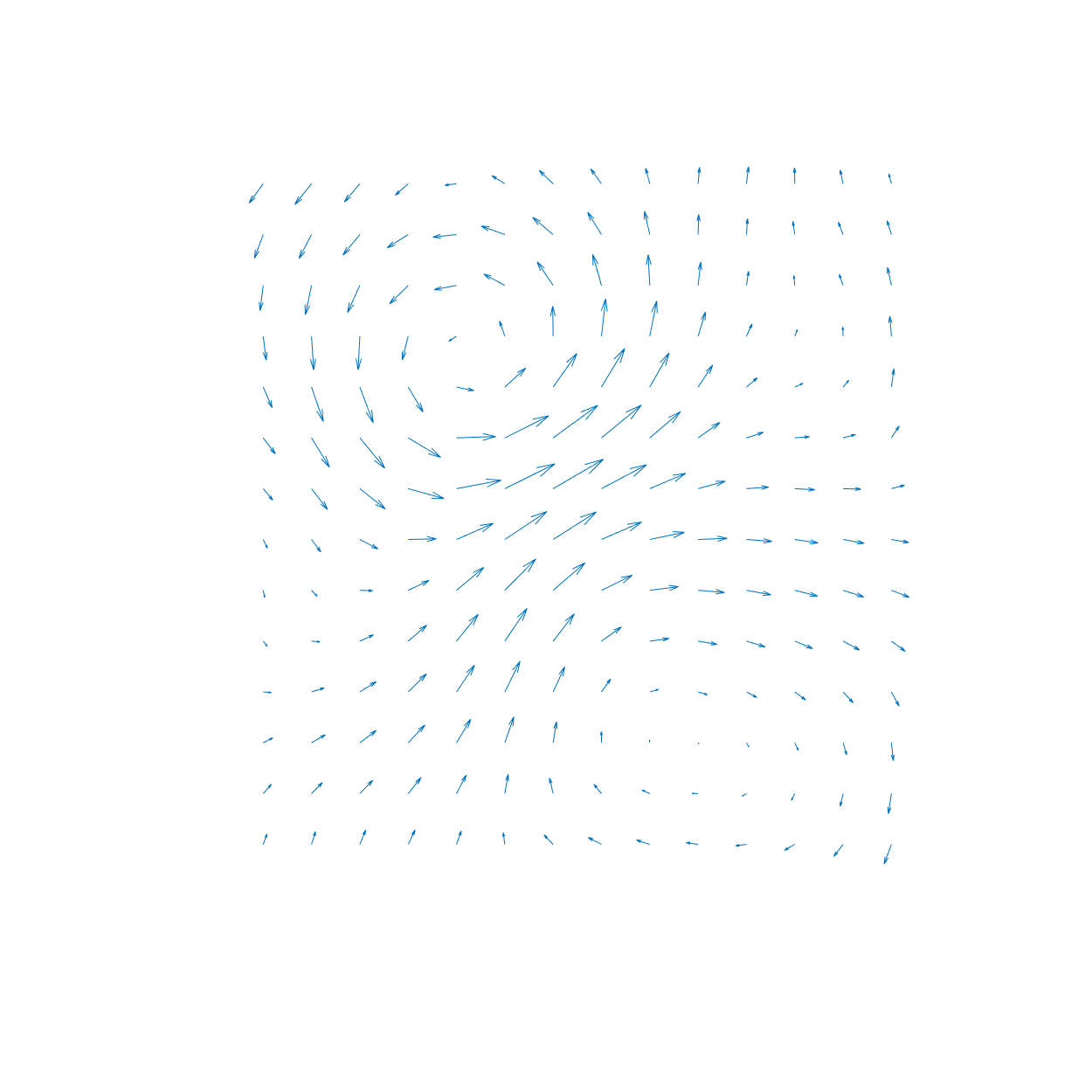

Supplement: S2 MCG raw data 2 — The raw MCG dataset includes categories 0-3 for training and validation. (ZIP) [file pone.0338189.s002.zip › train/0/p2_380_2.png]

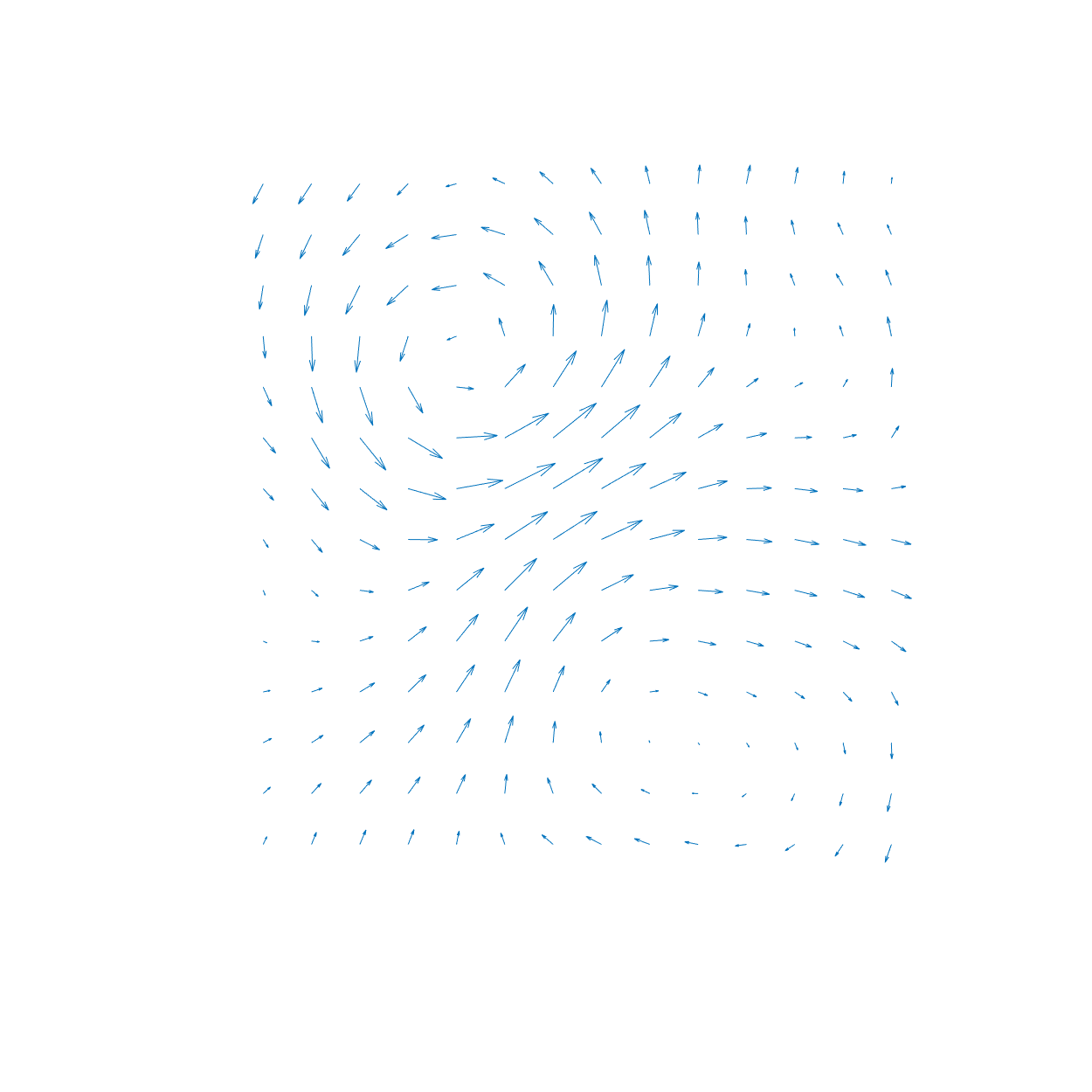

Supplement: S2 MCG raw data 2 — The raw MCG dataset includes categories 0-3 for training and validation. (ZIP) [file pone.0338189.s002.zip › train/0/p2_380_3.png]

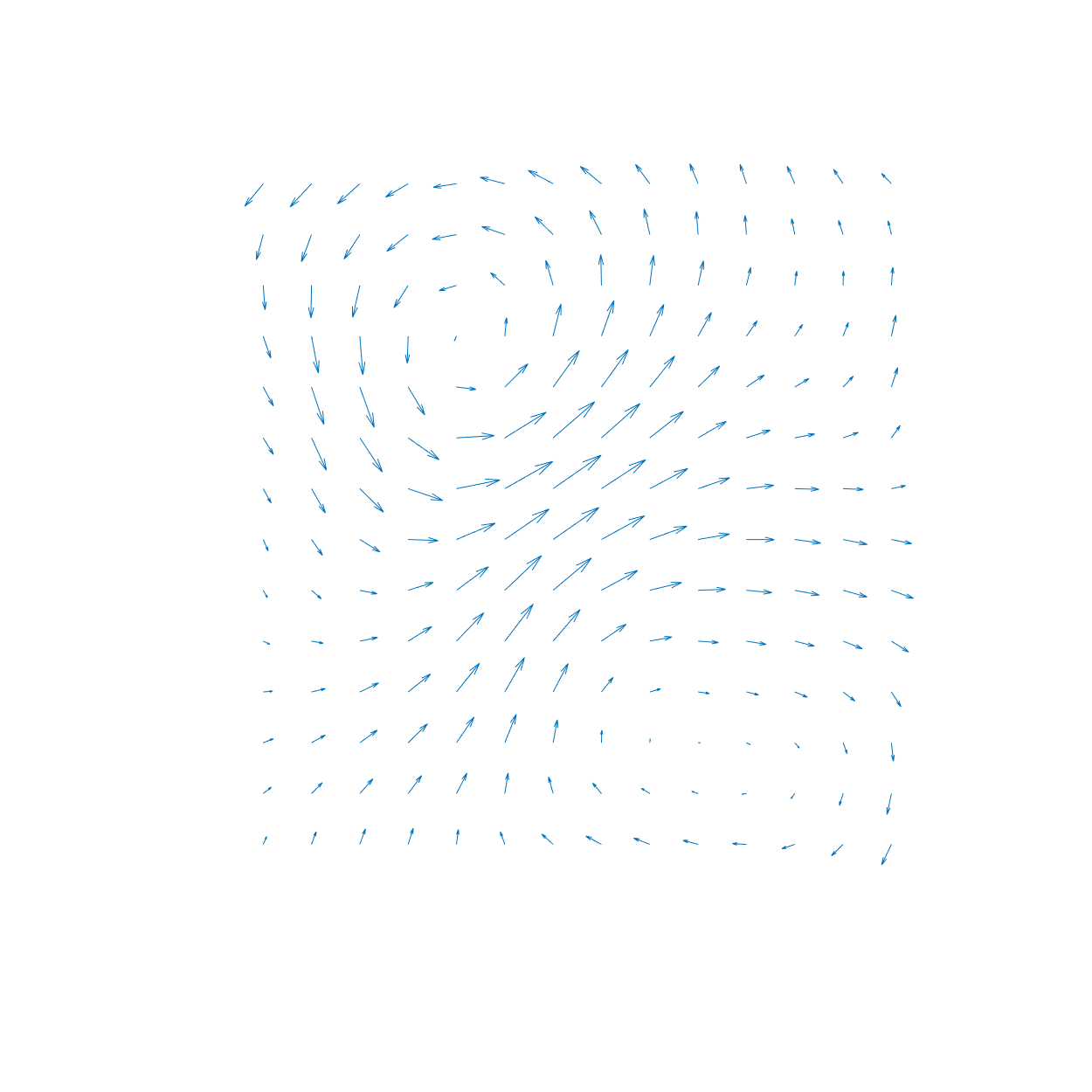

Supplement: S2 MCG raw data 2 — The raw MCG dataset includes categories 0-3 for training and validation. (ZIP) [file pone.0338189.s002.zip › train/0/p2_385_1.png]

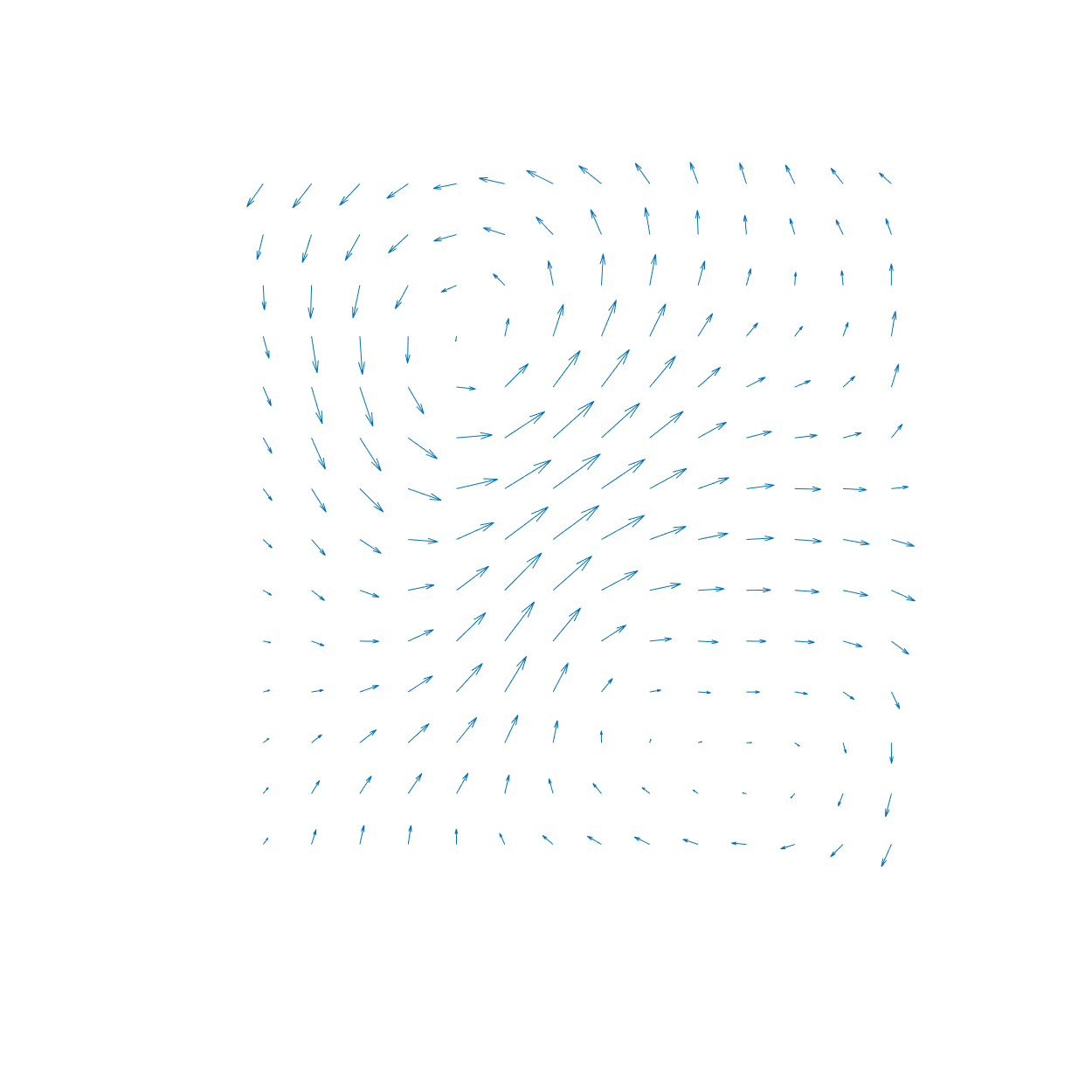

Supplement: S2 MCG raw data 2 — The raw MCG dataset includes categories 0-3 for training and validation. (ZIP) [file pone.0338189.s002.zip › train/0/p2_385_2.png]

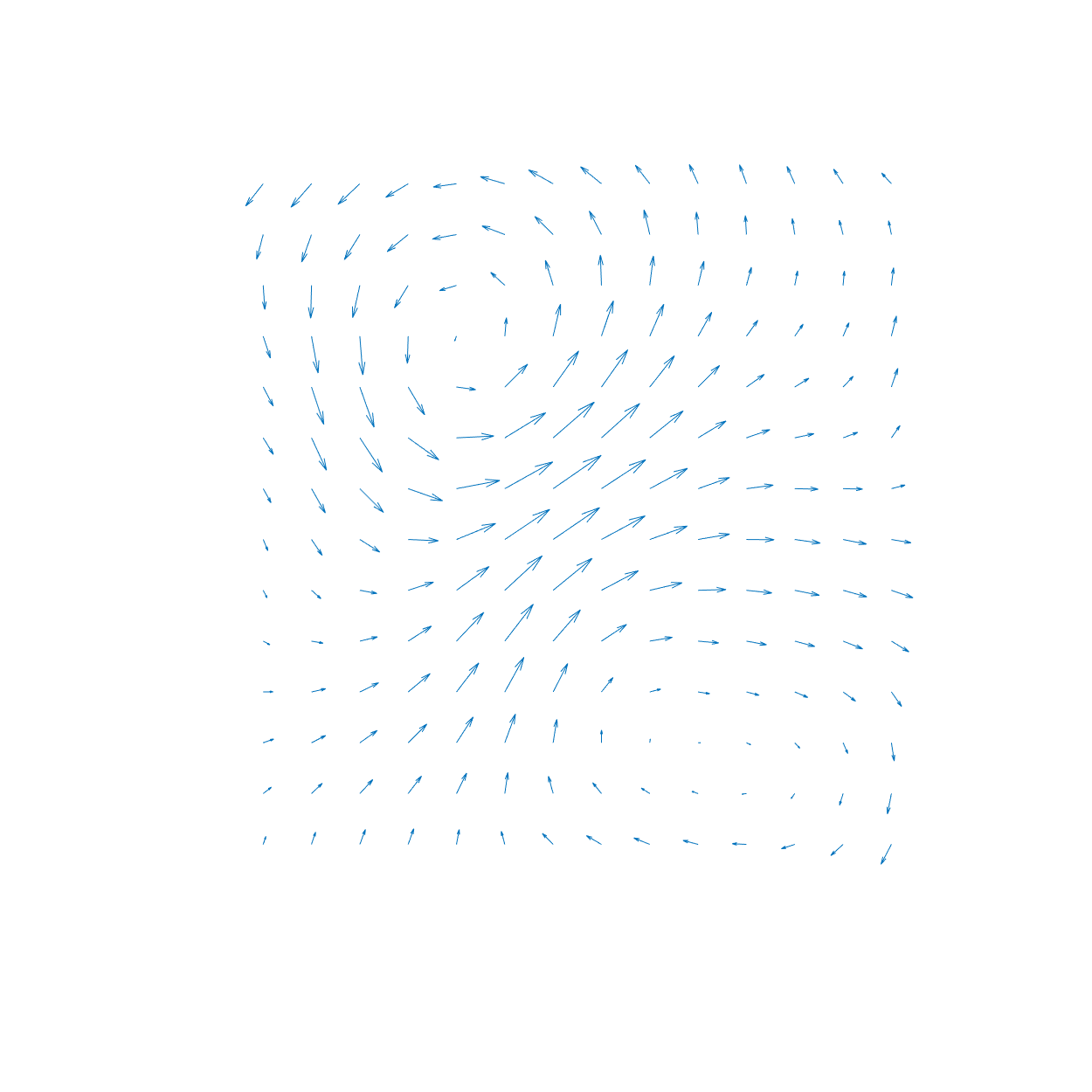

Supplement: S2 MCG raw data 2 — The raw MCG dataset includes categories 0-3 for training and validation. (ZIP) [file pone.0338189.s002.zip › train/0/p2_385_3.png]

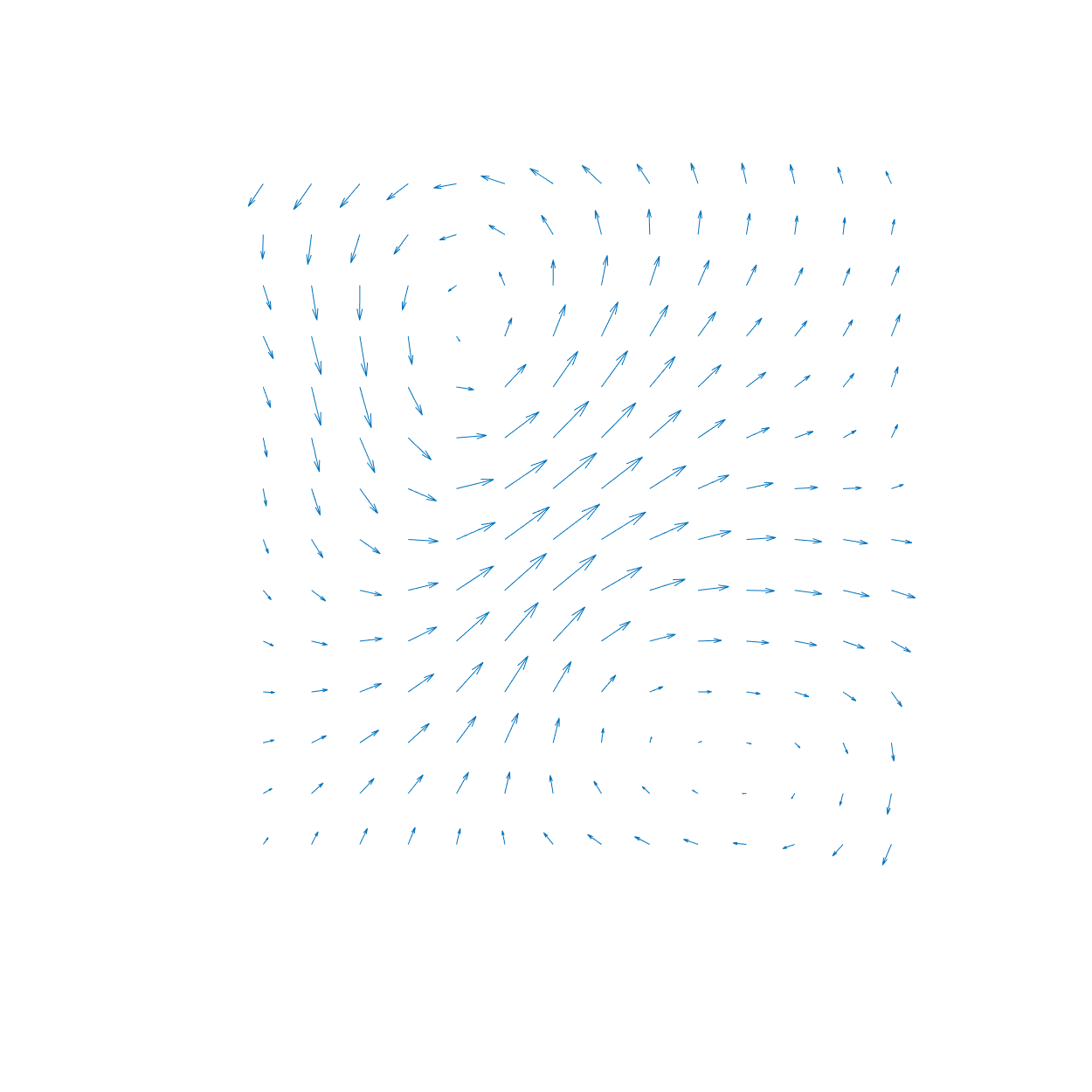

Supplement: S2 MCG raw data 2 — The raw MCG dataset includes categories 0-3 for training and validation. (ZIP) [file pone.0338189.s002.zip › train/0/p2_390_1.png]

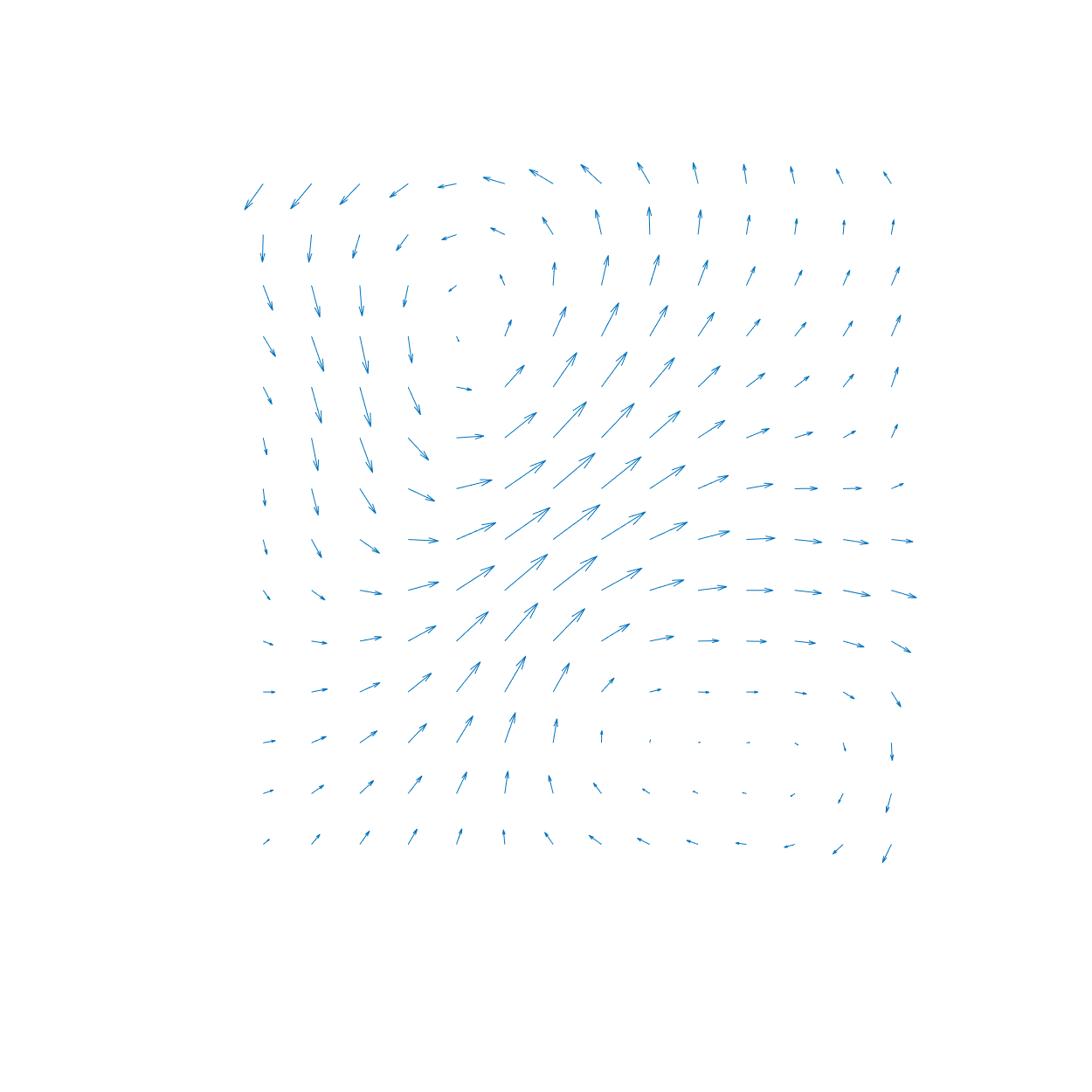

Supplement: S2 MCG raw data 2 — The raw MCG dataset includes categories 0-3 for training and validation. (ZIP) [file pone.0338189.s002.zip › train/0/p2_390_2.png]

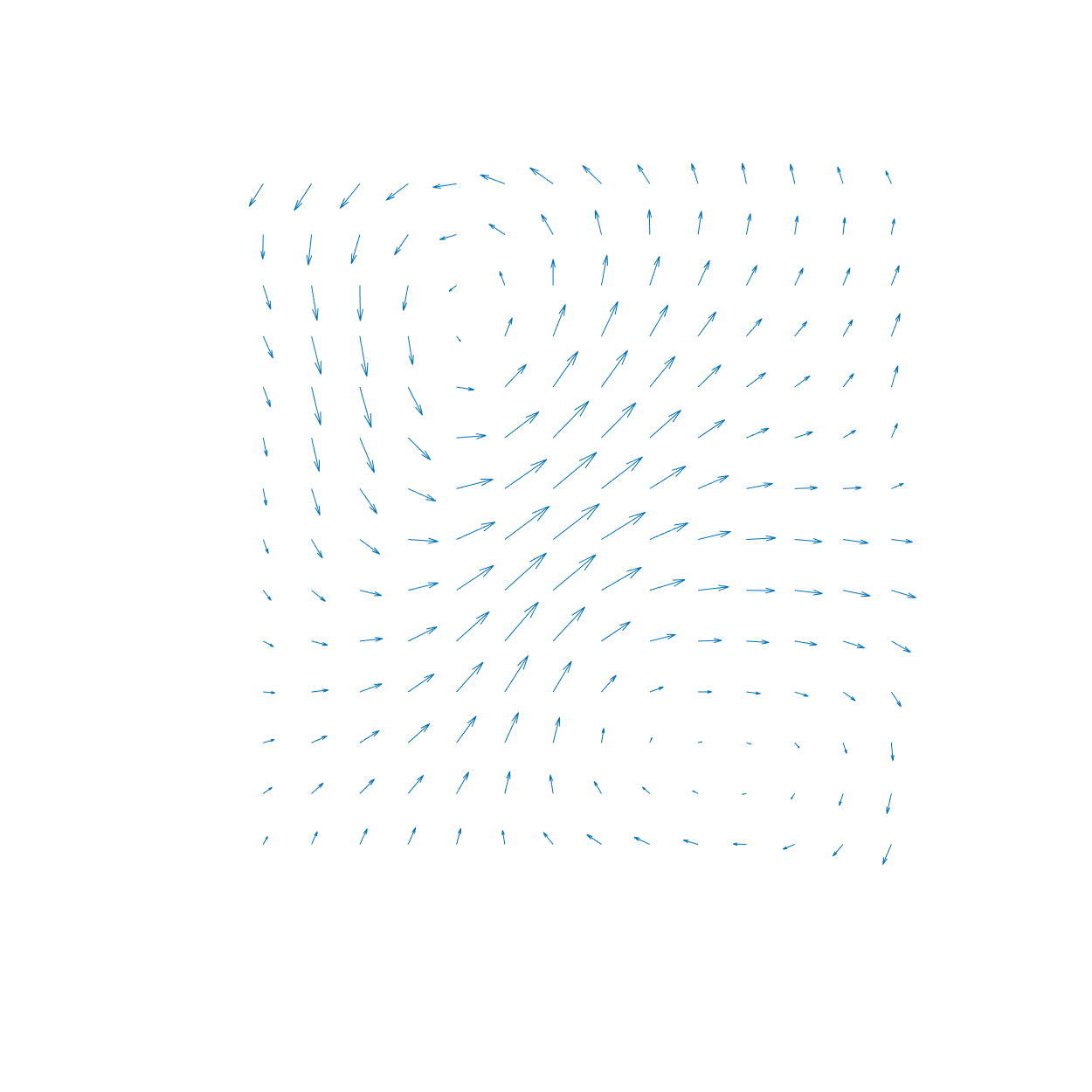

Supplement: S2 MCG raw data 2 — The raw MCG dataset includes categories 0-3 for training and validation. (ZIP) [file pone.0338189.s002.zip › train/0/p2_390_3.png]

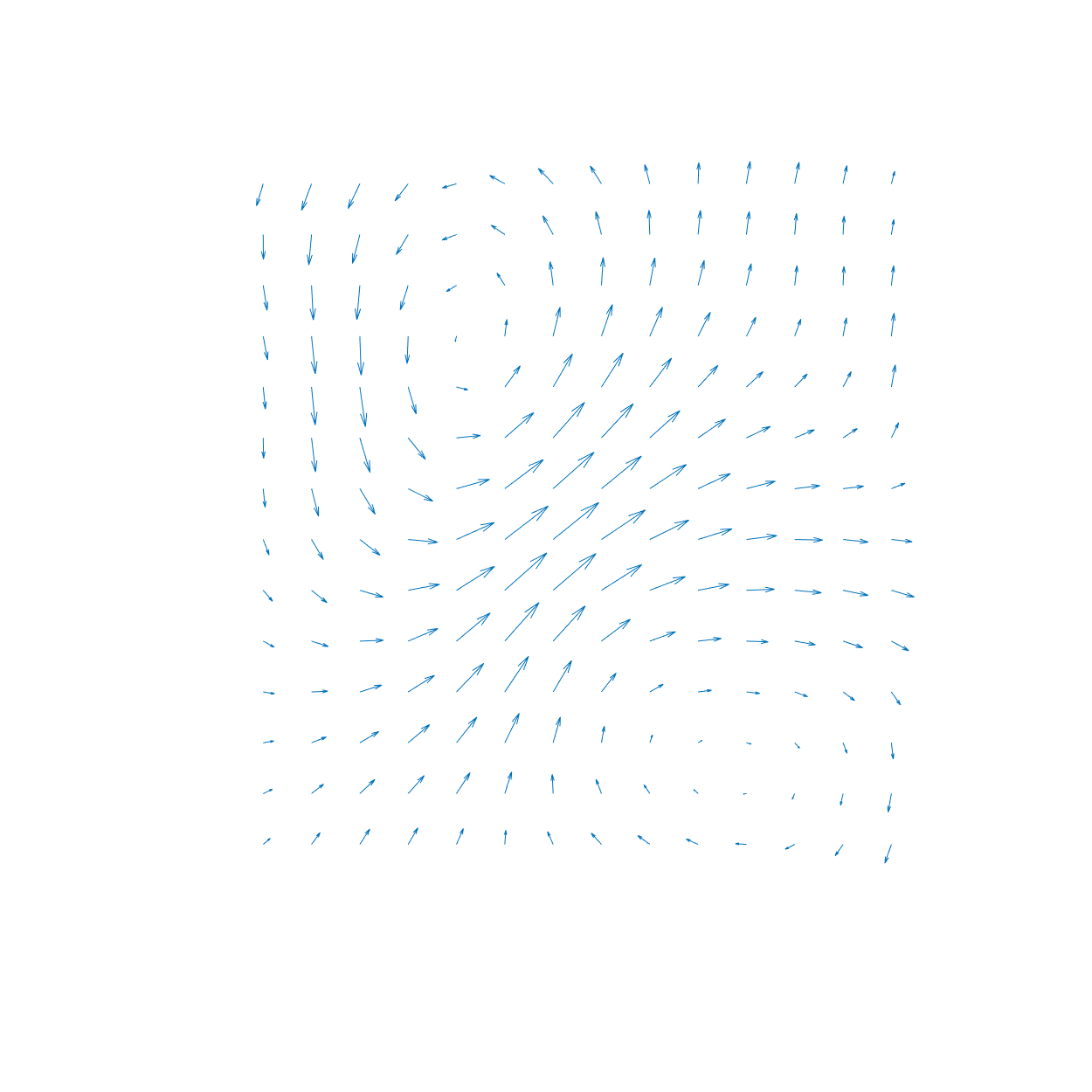

Supplement: S2 MCG raw data 2 — The raw MCG dataset includes categories 0-3 for training and validation. (ZIP) [file pone.0338189.s002.zip › train/0/p2_395_1.png]

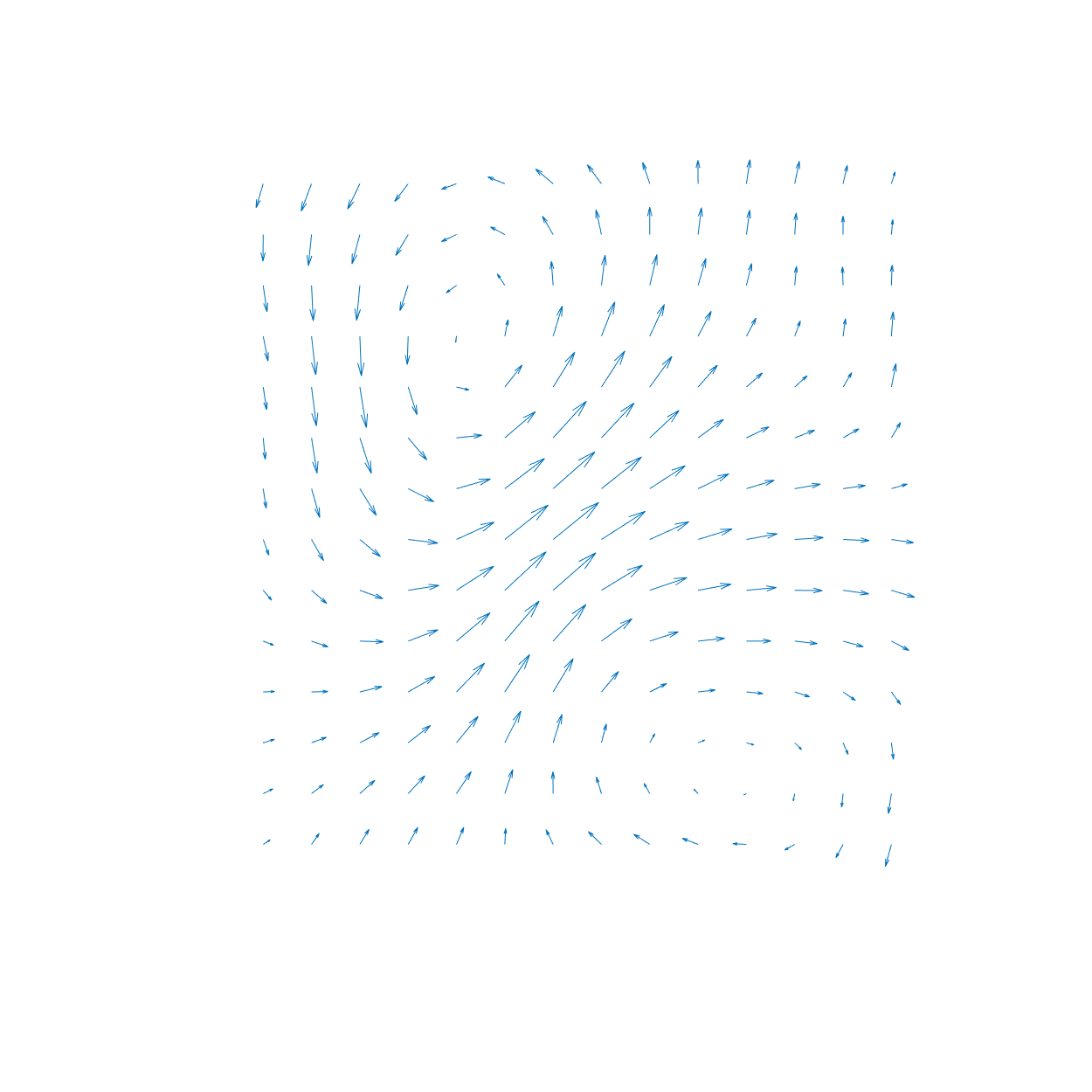

Supplement: S2 MCG raw data 2 — The raw MCG dataset includes categories 0-3 for training and validation. (ZIP) [file pone.0338189.s002.zip › train/0/p2_395_2.png]

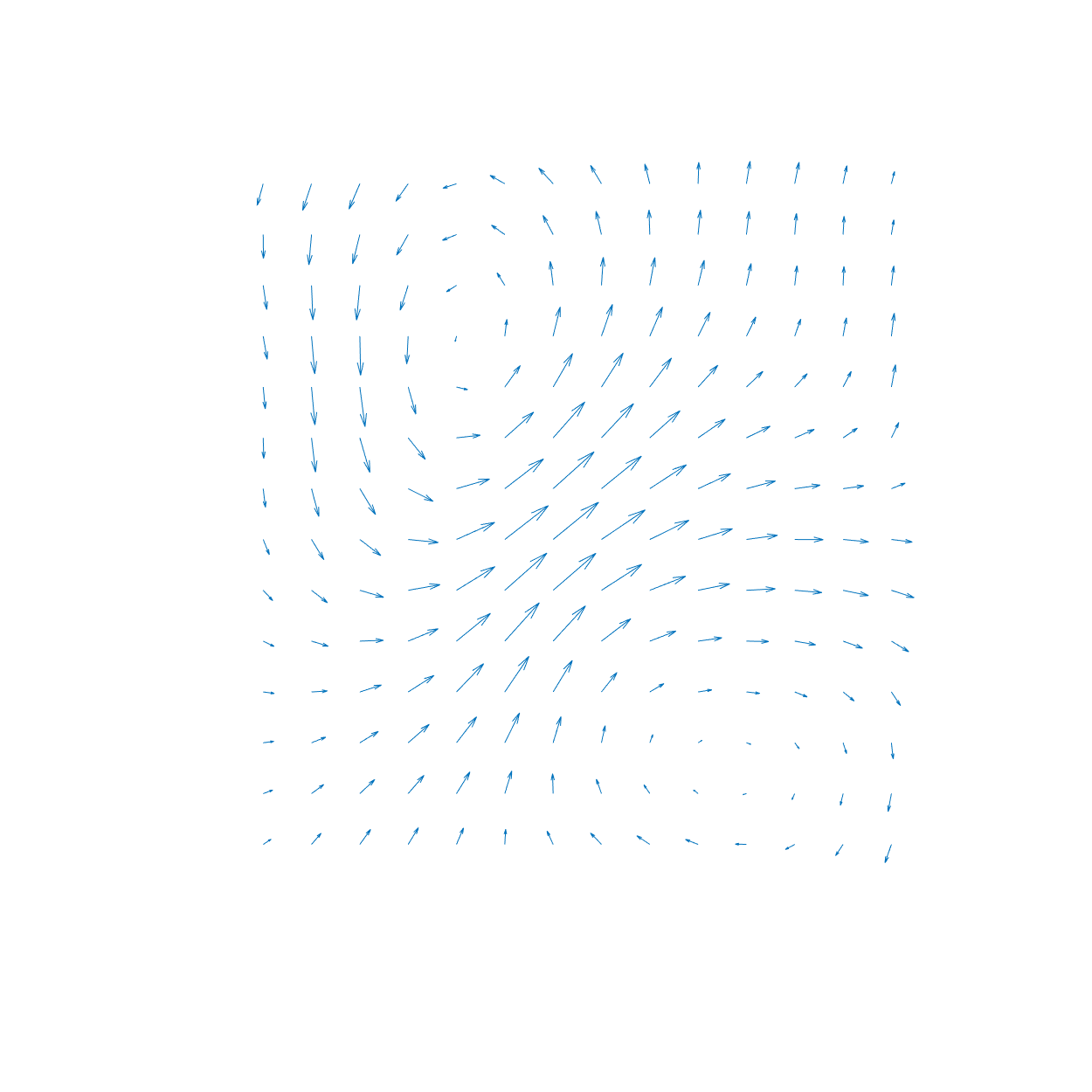

Supplement: S2 MCG raw data 2 — The raw MCG dataset includes categories 0-3 for training and validation. (ZIP) [file pone.0338189.s002.zip › train/0/p2_395_3.png]

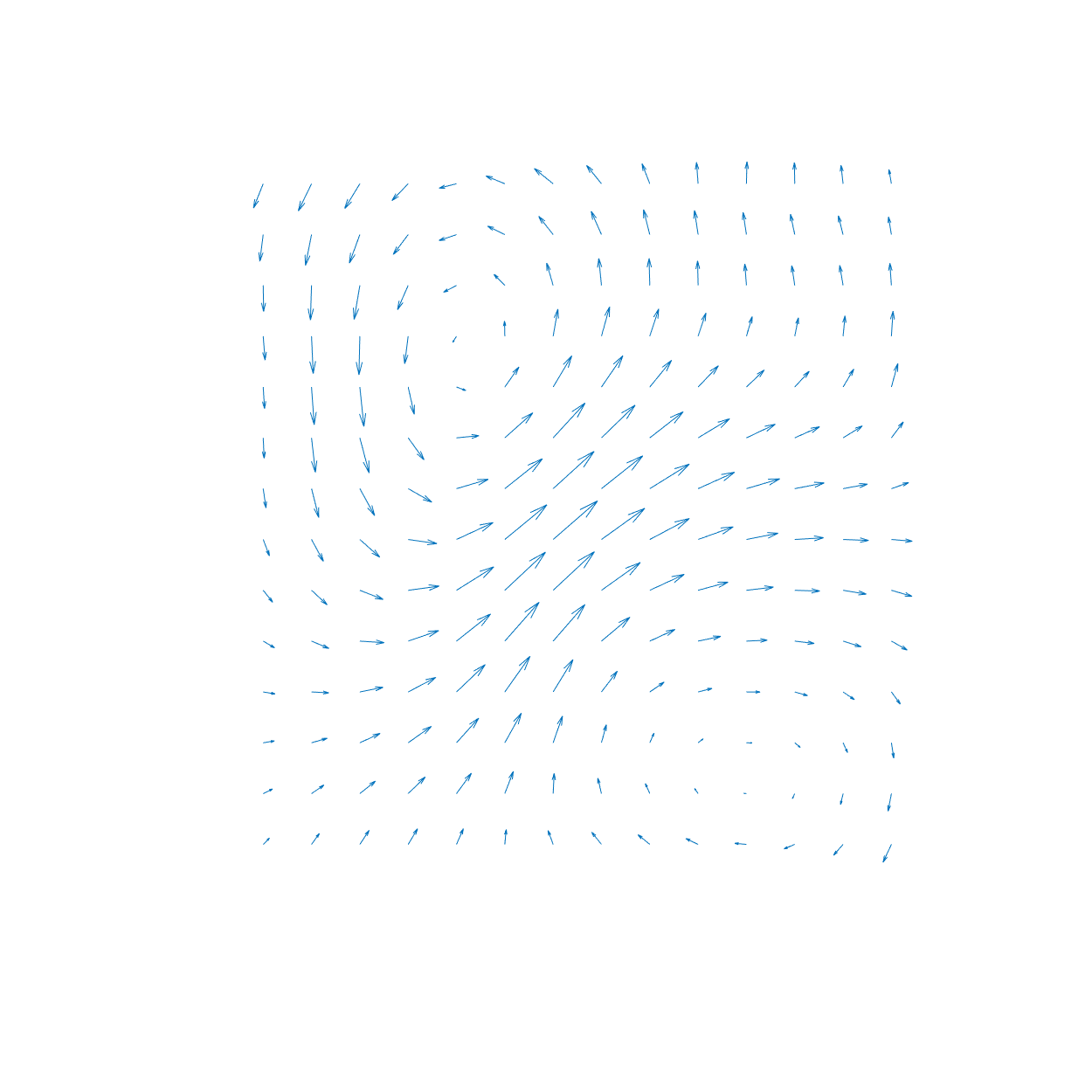

Supplement: S2 MCG raw data 2 — The raw MCG dataset includes categories 0-3 for training and validation. (ZIP) [file pone.0338189.s002.zip › train/0/p2_400_1.png]

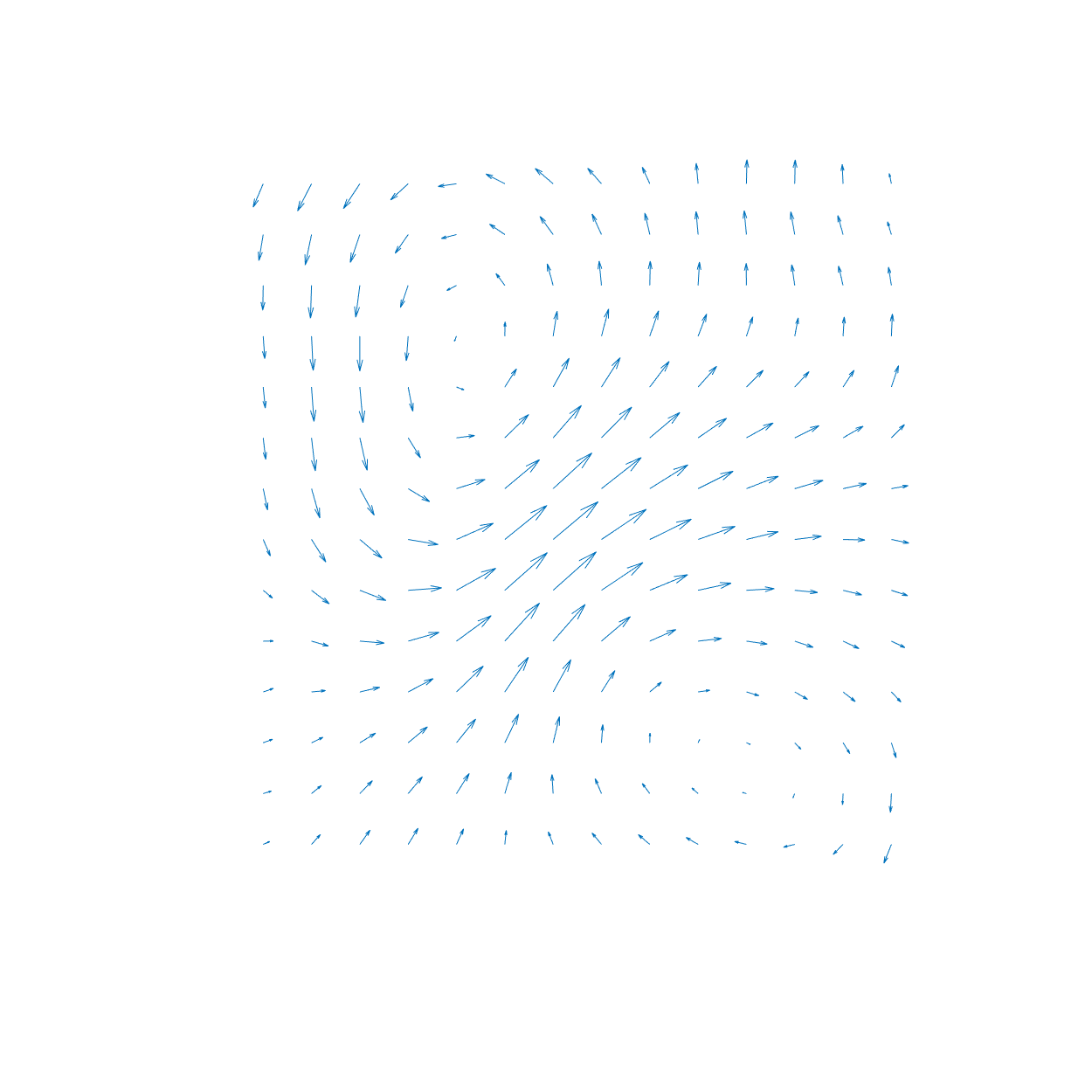

Supplement: S2 MCG raw data 2 — The raw MCG dataset includes categories 0-3 for training and validation. (ZIP) [file pone.0338189.s002.zip › train/0/p2_400_2.png]

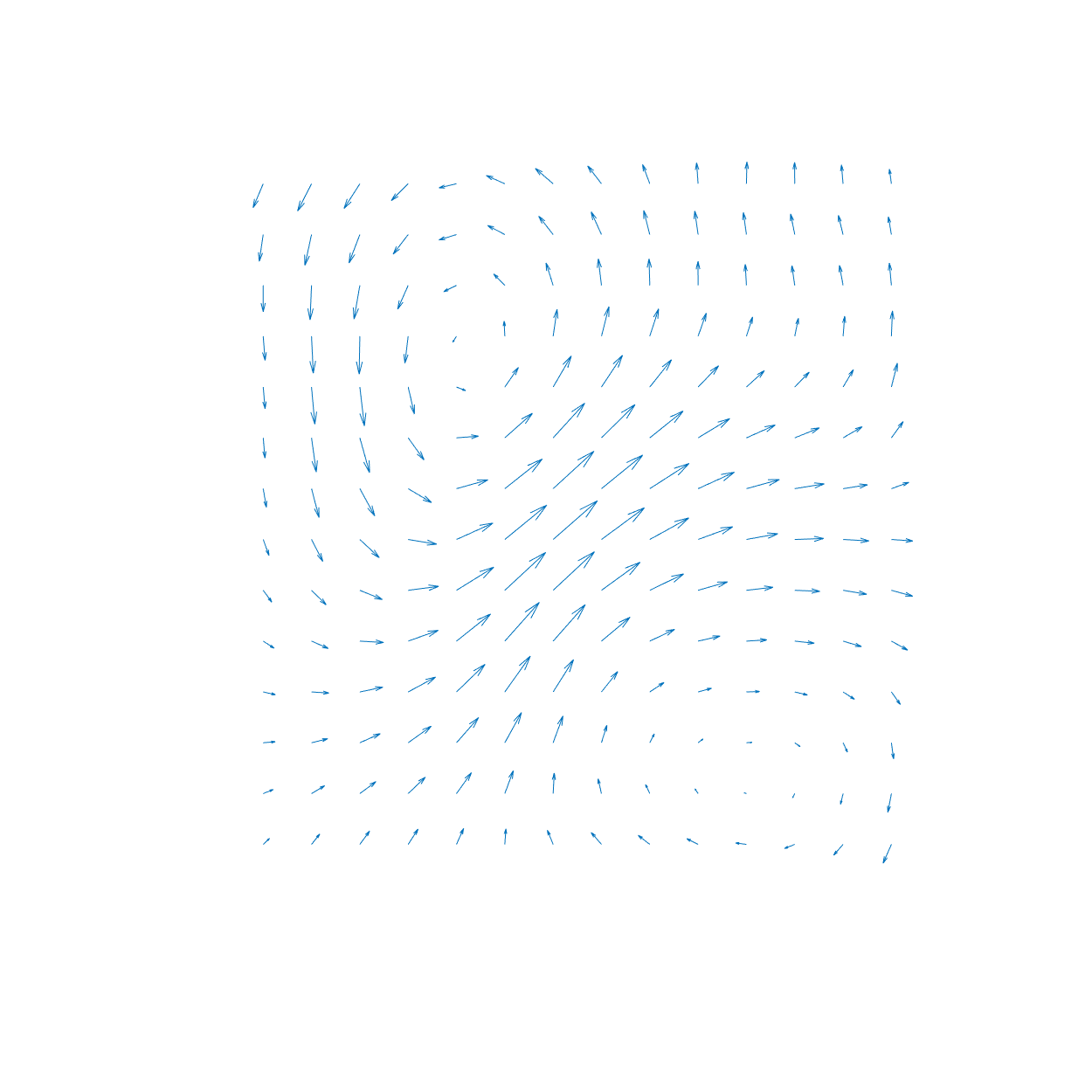

Supplement: S2 MCG raw data 2 — The raw MCG dataset includes categories 0-3 for training and validation. (ZIP) [file pone.0338189.s002.zip › train/0/p2_400_3.png]

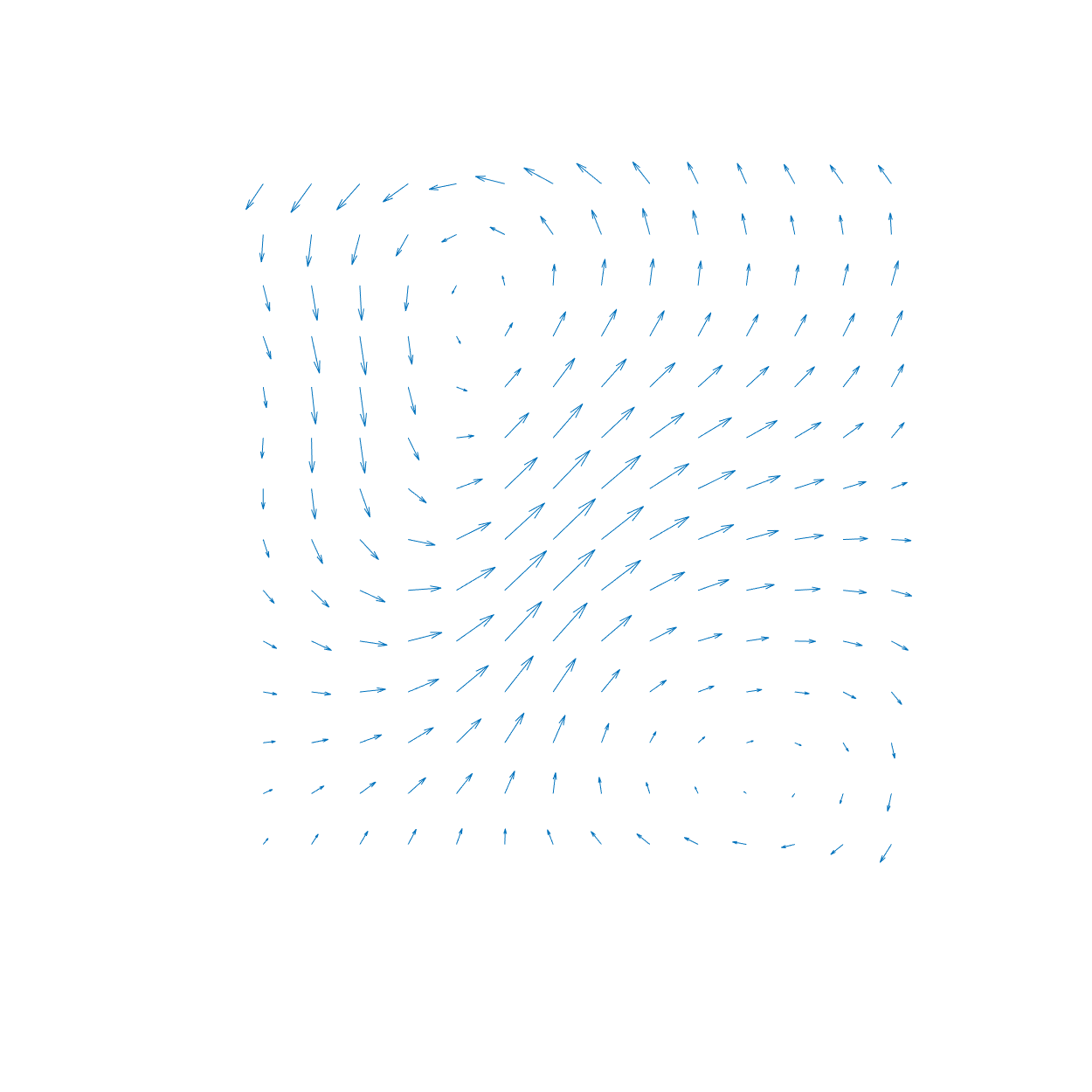

Supplement: S2 MCG raw data 2 — The raw MCG dataset includes categories 0-3 for training and validation. (ZIP) [file pone.0338189.s002.zip › train/0/p2_405_1.png]

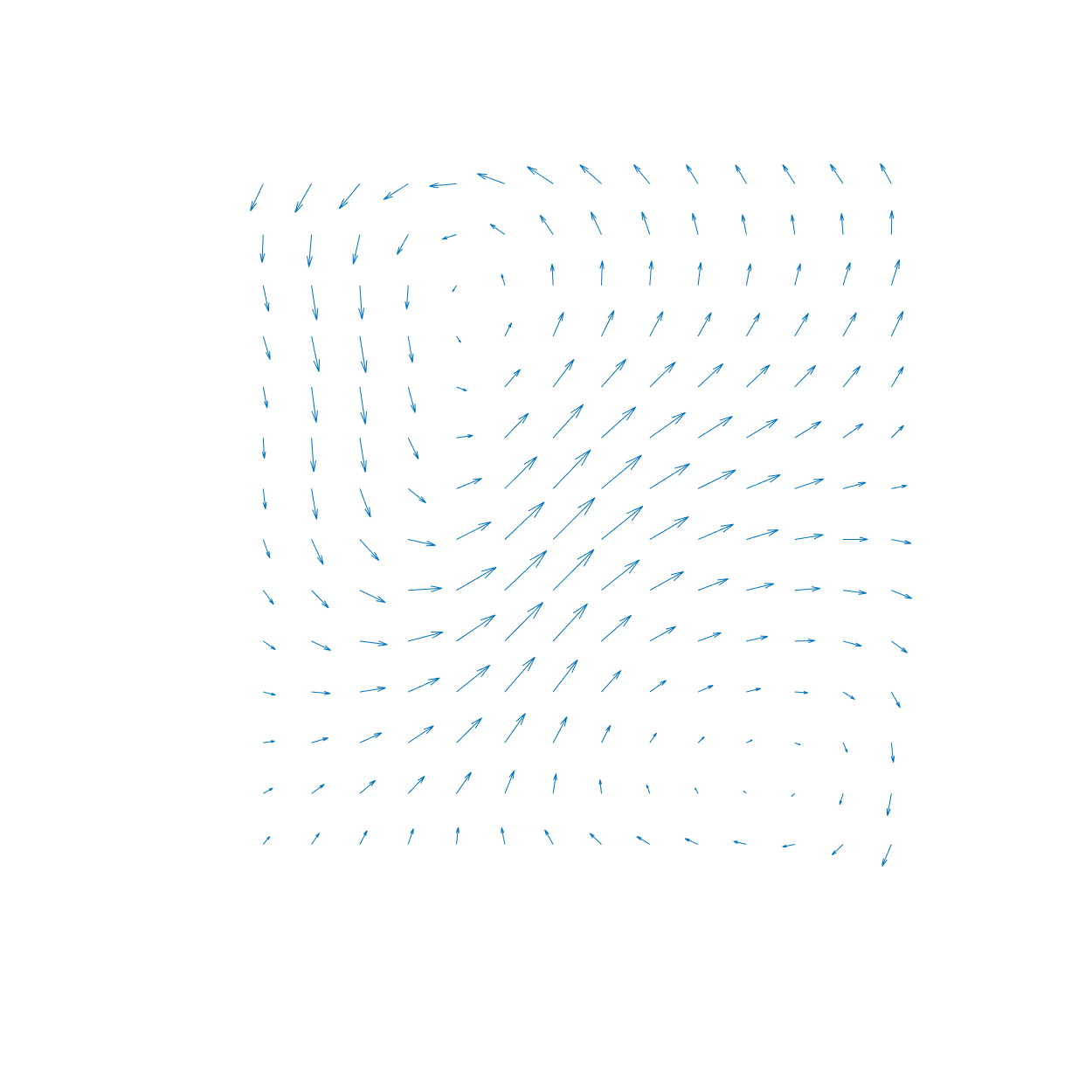

Supplement: S2 MCG raw data 2 — The raw MCG dataset includes categories 0-3 for training and validation. (ZIP) [file pone.0338189.s002.zip › train/0/p2_405_2.png]

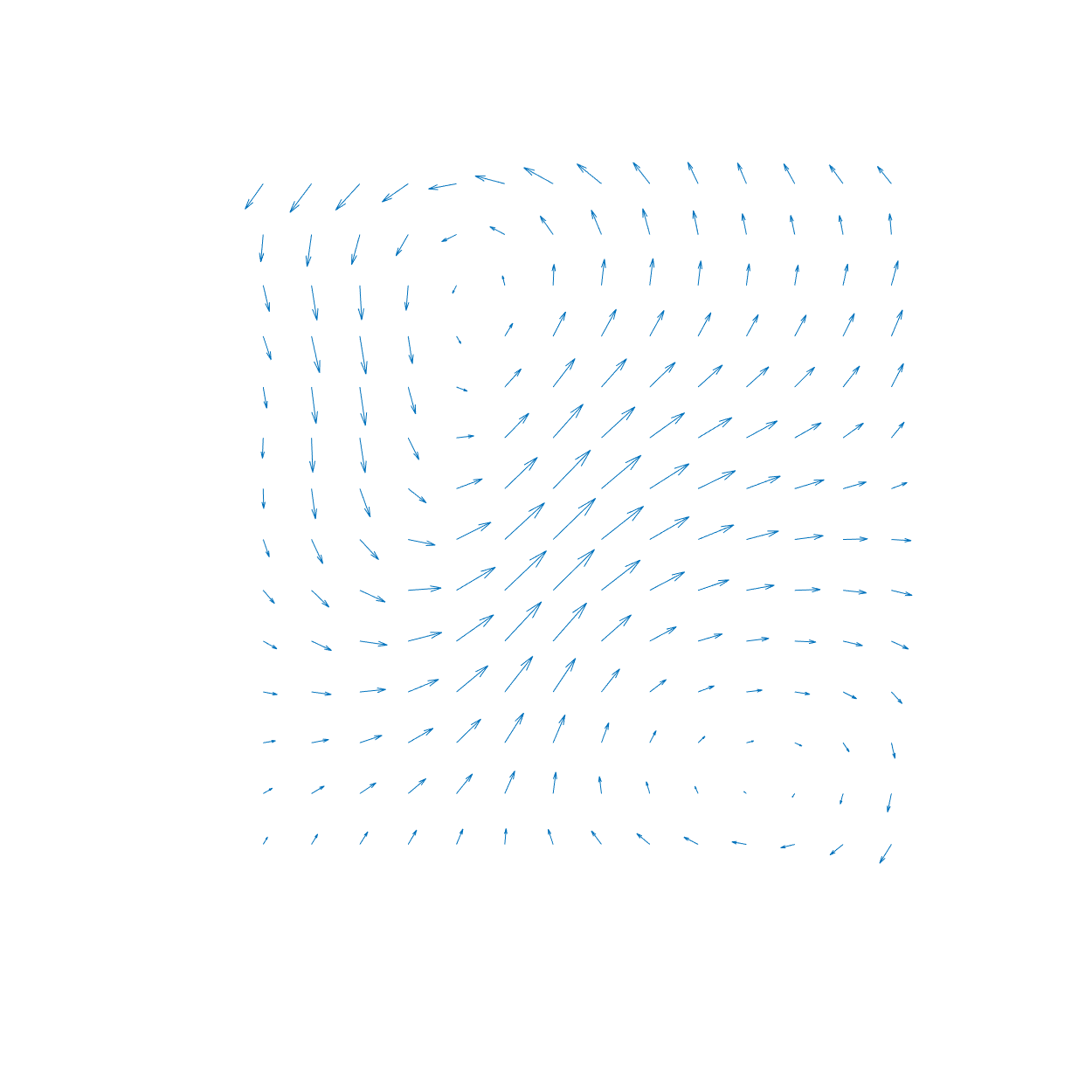

Supplement: S2 MCG raw data 2 — The raw MCG dataset includes categories 0-3 for training and validation. (ZIP) [file pone.0338189.s002.zip › train/0/p2_405_3.png]

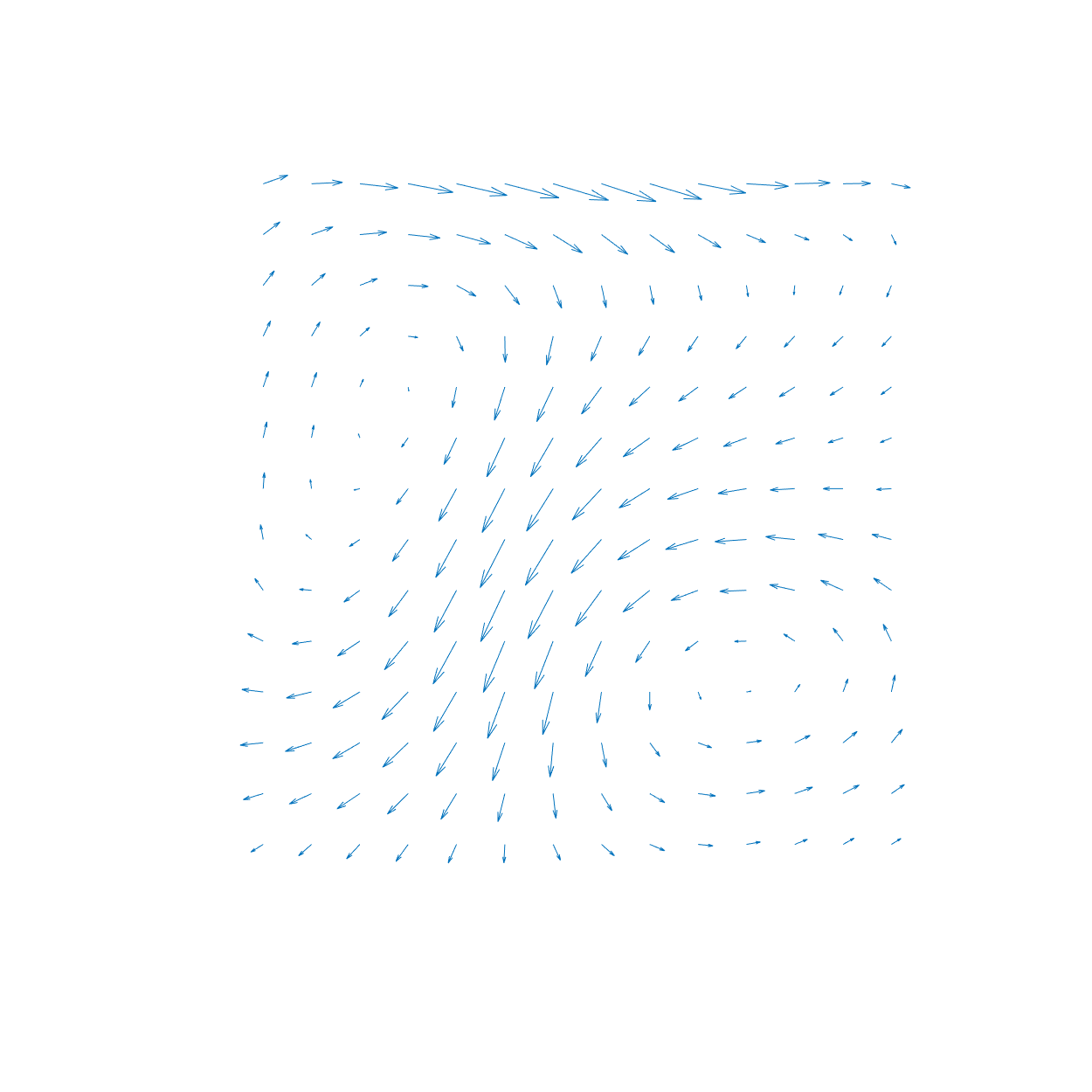

Supplement: S2 MCG raw data 2 — The raw MCG dataset includes categories 0-3 for training and validation. (ZIP) [file pone.0338189.s002.zip › train/0/p3_235_1.png]

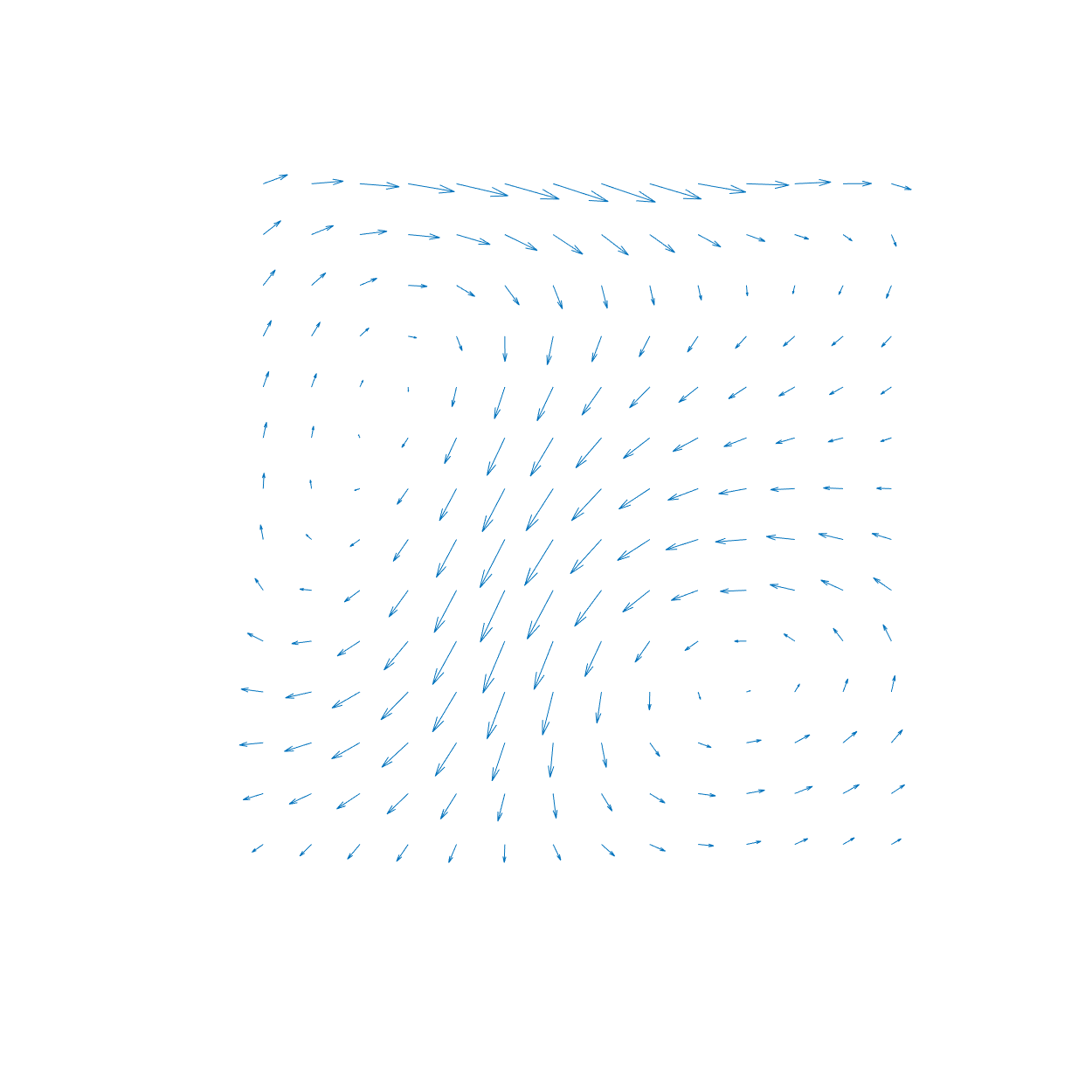

Supplement: S2 MCG raw data 2 — The raw MCG dataset includes categories 0-3 for training and validation. (ZIP) [file pone.0338189.s002.zip › train/0/p3_235_2.png]

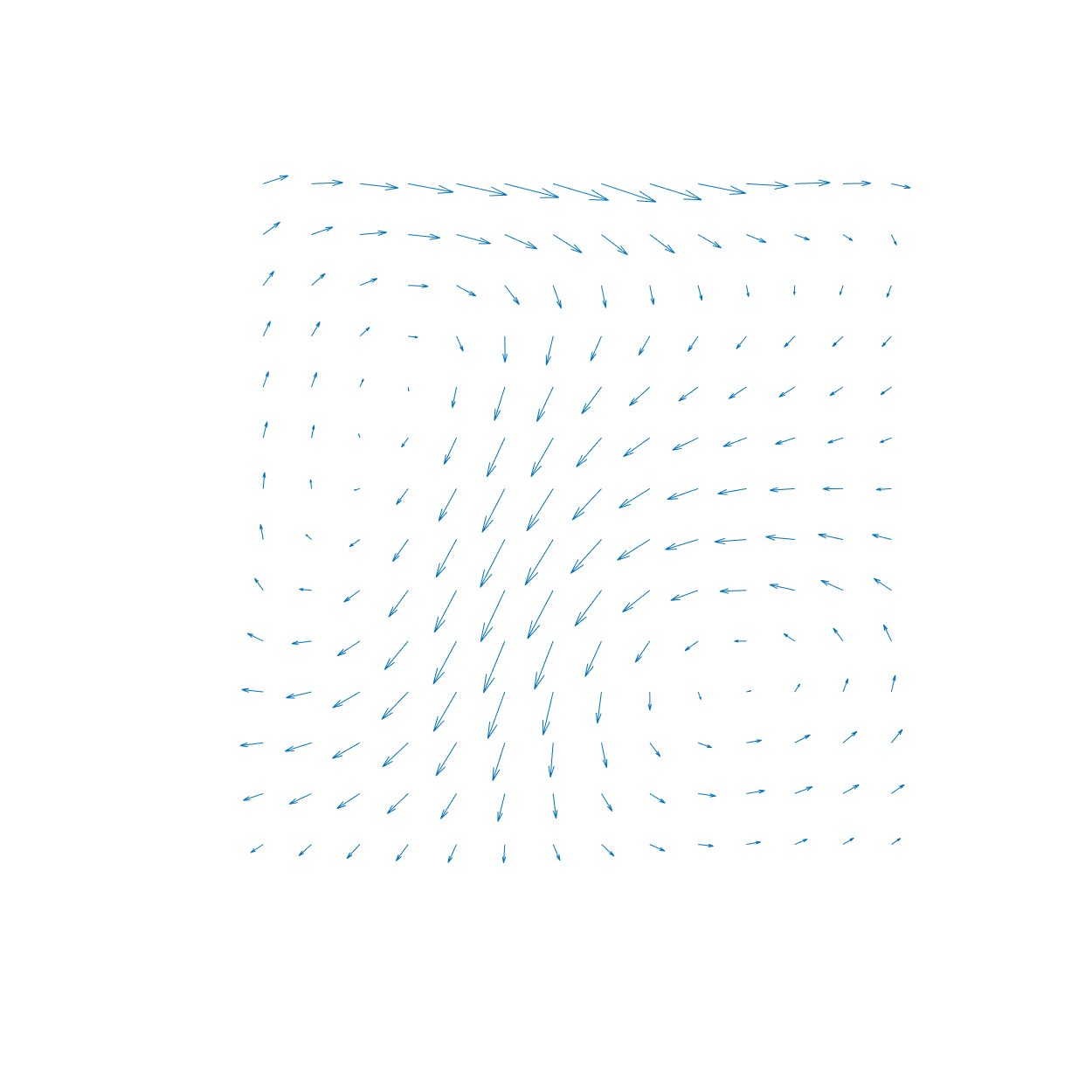

Supplement: S2 MCG raw data 2 — The raw MCG dataset includes categories 0-3 for training and validation. (ZIP) [file pone.0338189.s002.zip › train/0/p3_235_3.png]

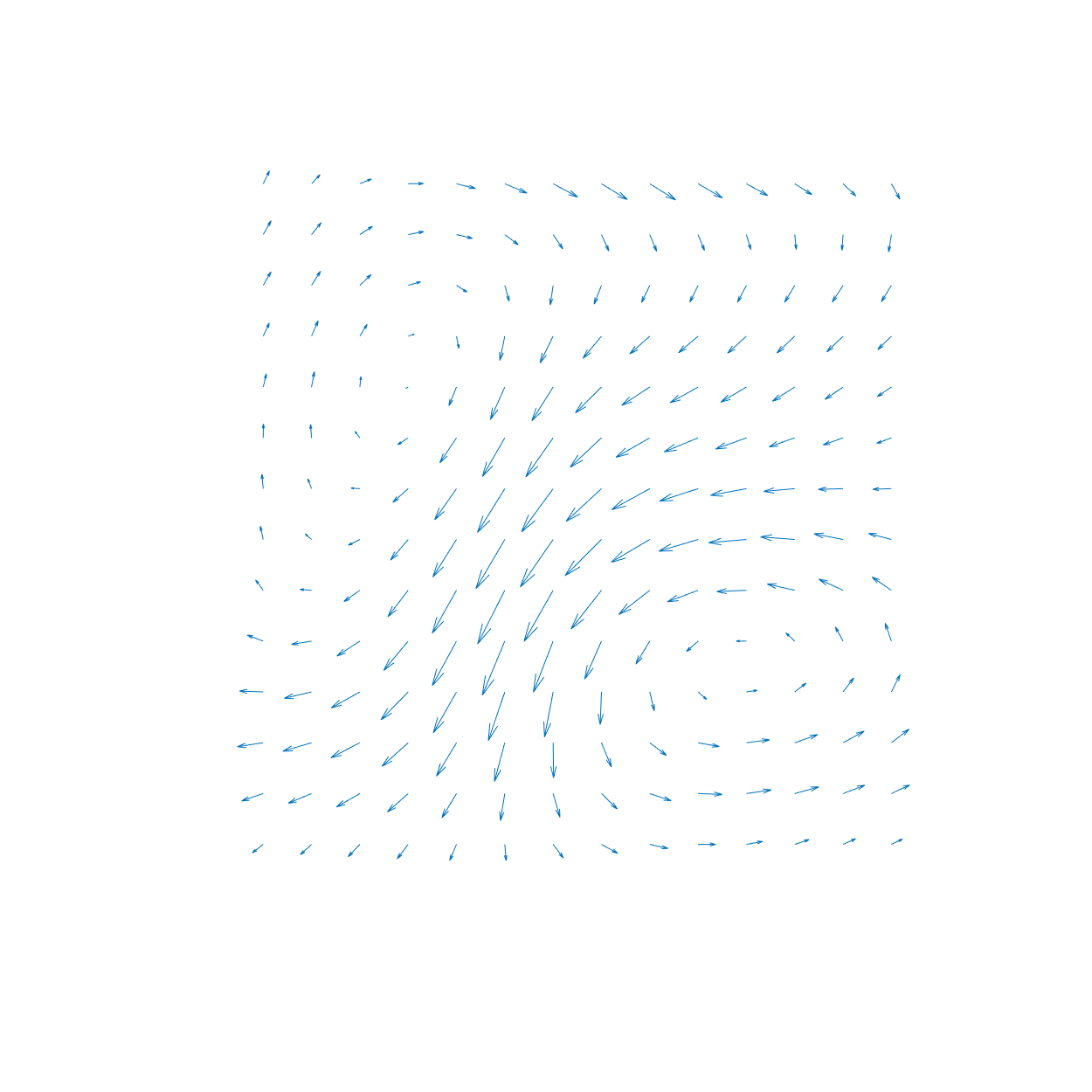

Supplement: S2 MCG raw data 2 — The raw MCG dataset includes categories 0-3 for training and validation. (ZIP) [file pone.0338189.s002.zip › train/0/p3_240_1.png]

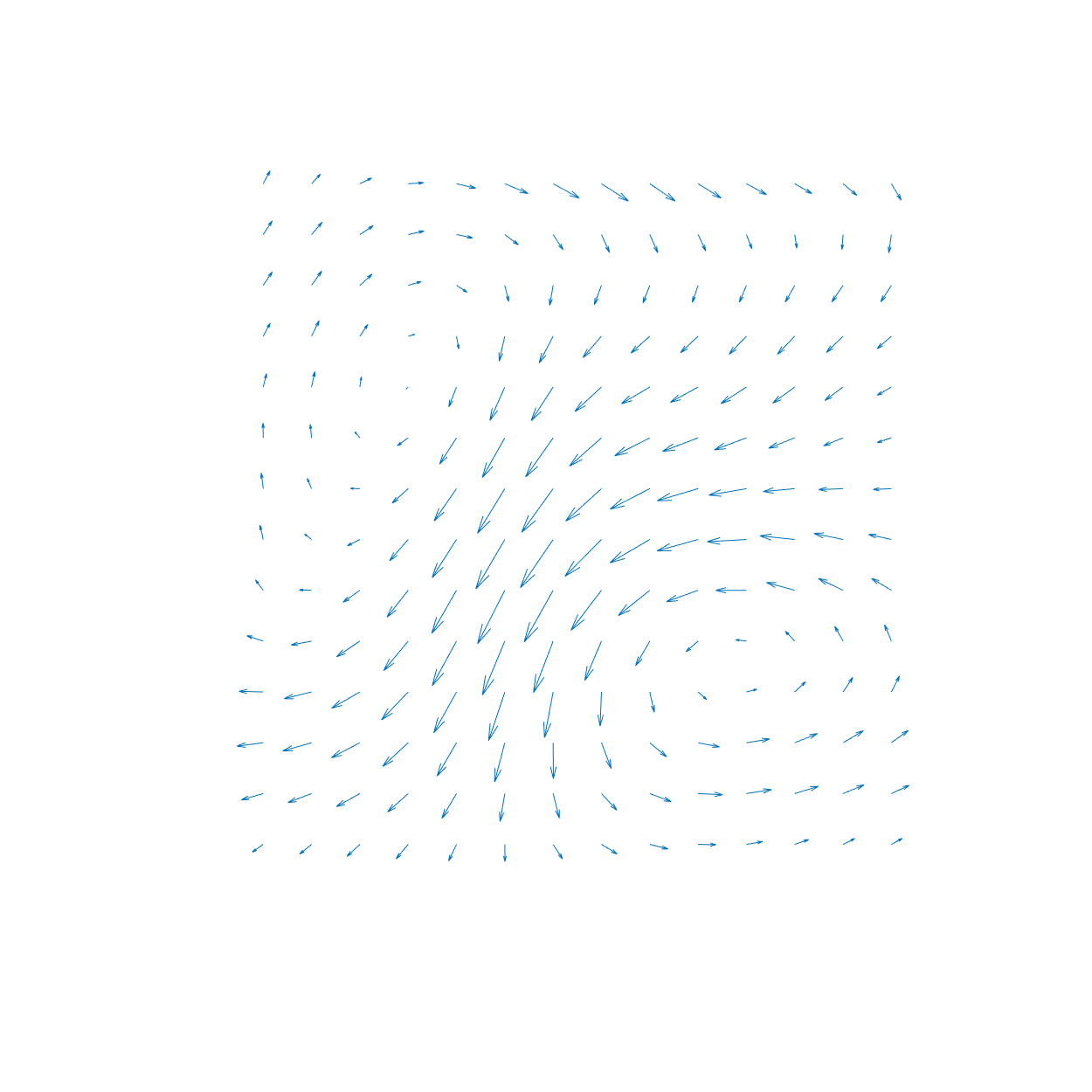

Supplement: S2 MCG raw data 2 — The raw MCG dataset includes categories 0-3 for training and validation. (ZIP) [file pone.0338189.s002.zip › train/0/p3_240_2.png]

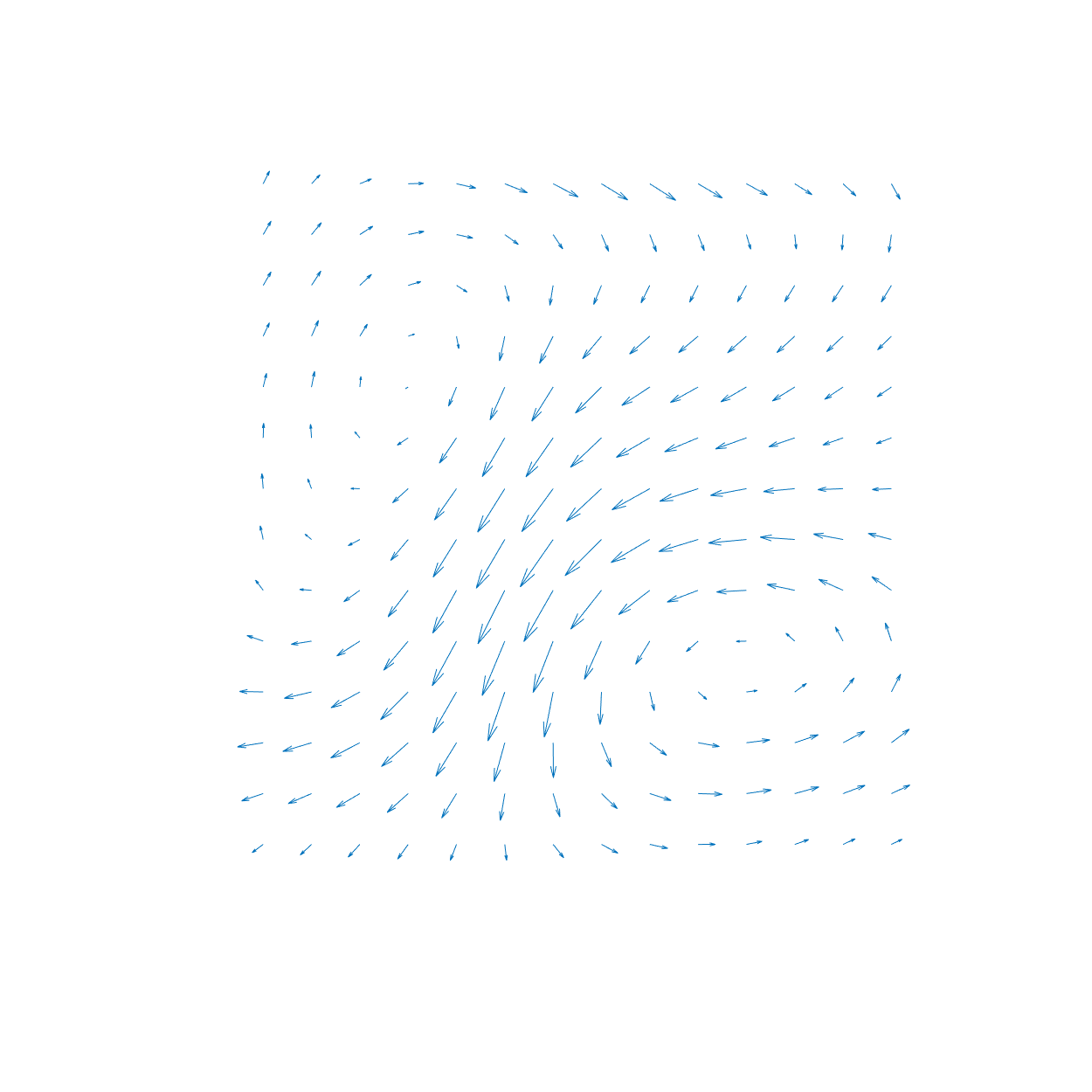

Supplement: S2 MCG raw data 2 — The raw MCG dataset includes categories 0-3 for training and validation. (ZIP) [file pone.0338189.s002.zip › train/0/p3_240_3.png]

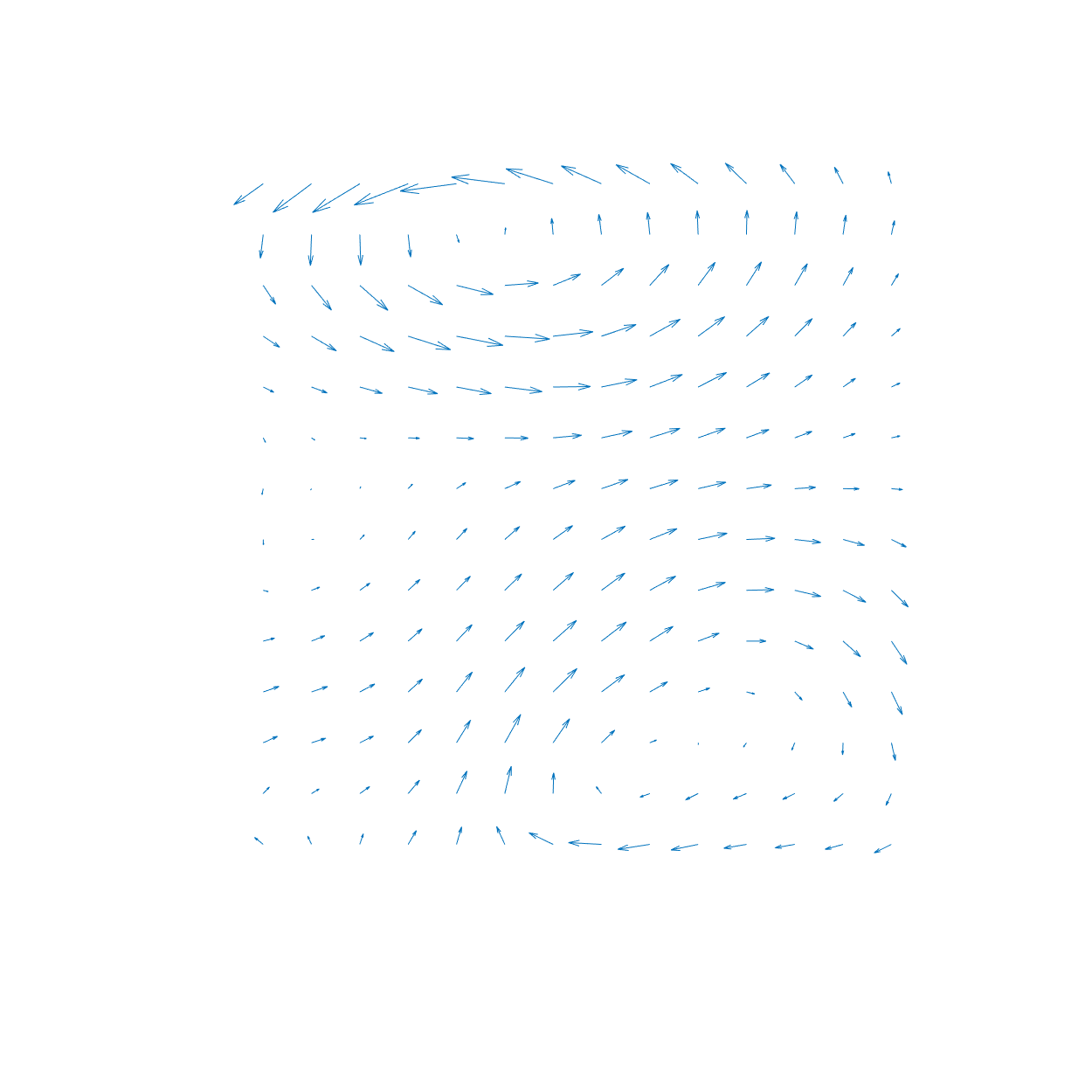

Supplement: S2 MCG raw data 2 — The raw MCG dataset includes categories 0-3 for training and validation. (ZIP) [file pone.0338189.s002.zip › train/0/p3_435_1.png]

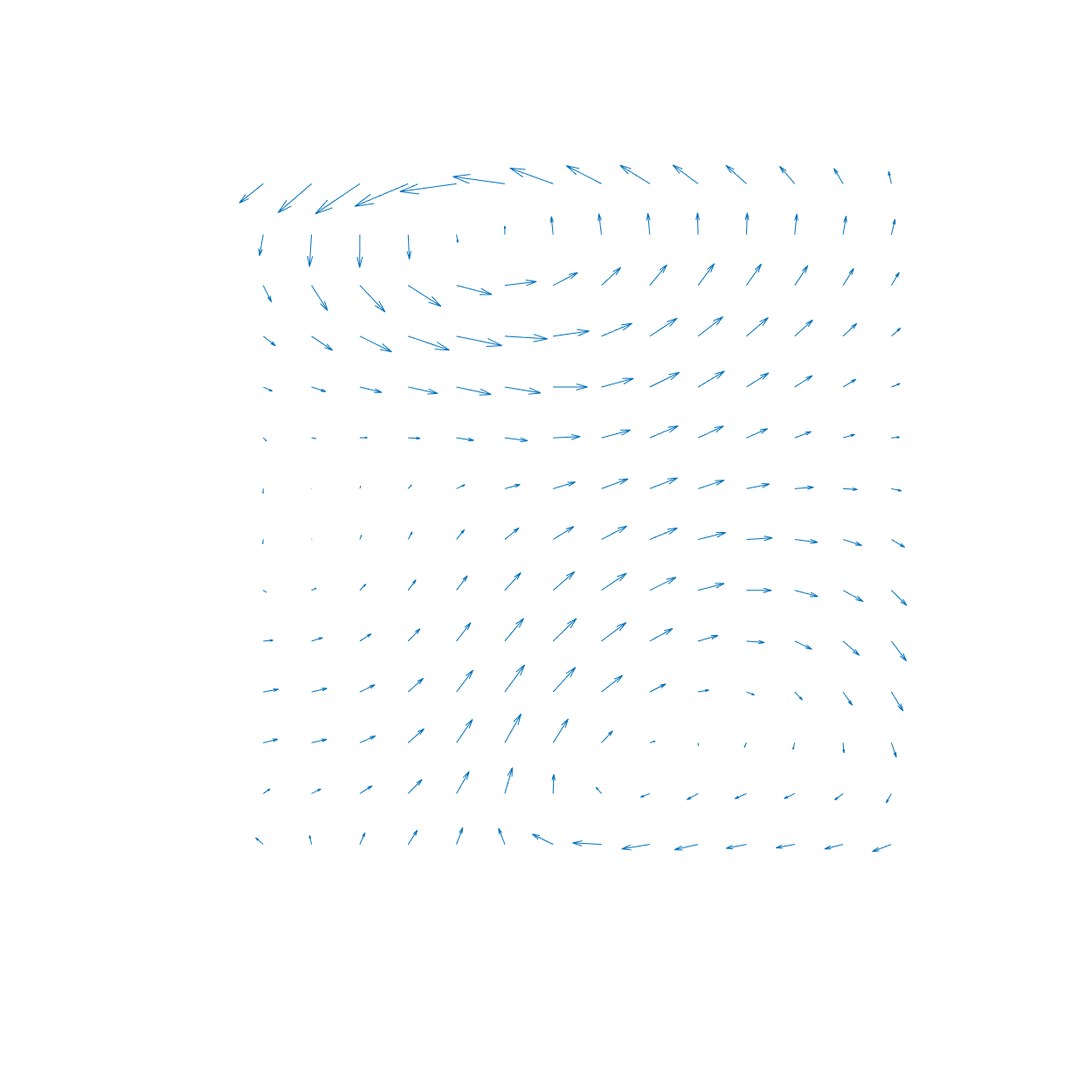

Supplement: S2 MCG raw data 2 — The raw MCG dataset includes categories 0-3 for training and validation. (ZIP) [file pone.0338189.s002.zip › train/0/p3_435_2.png]

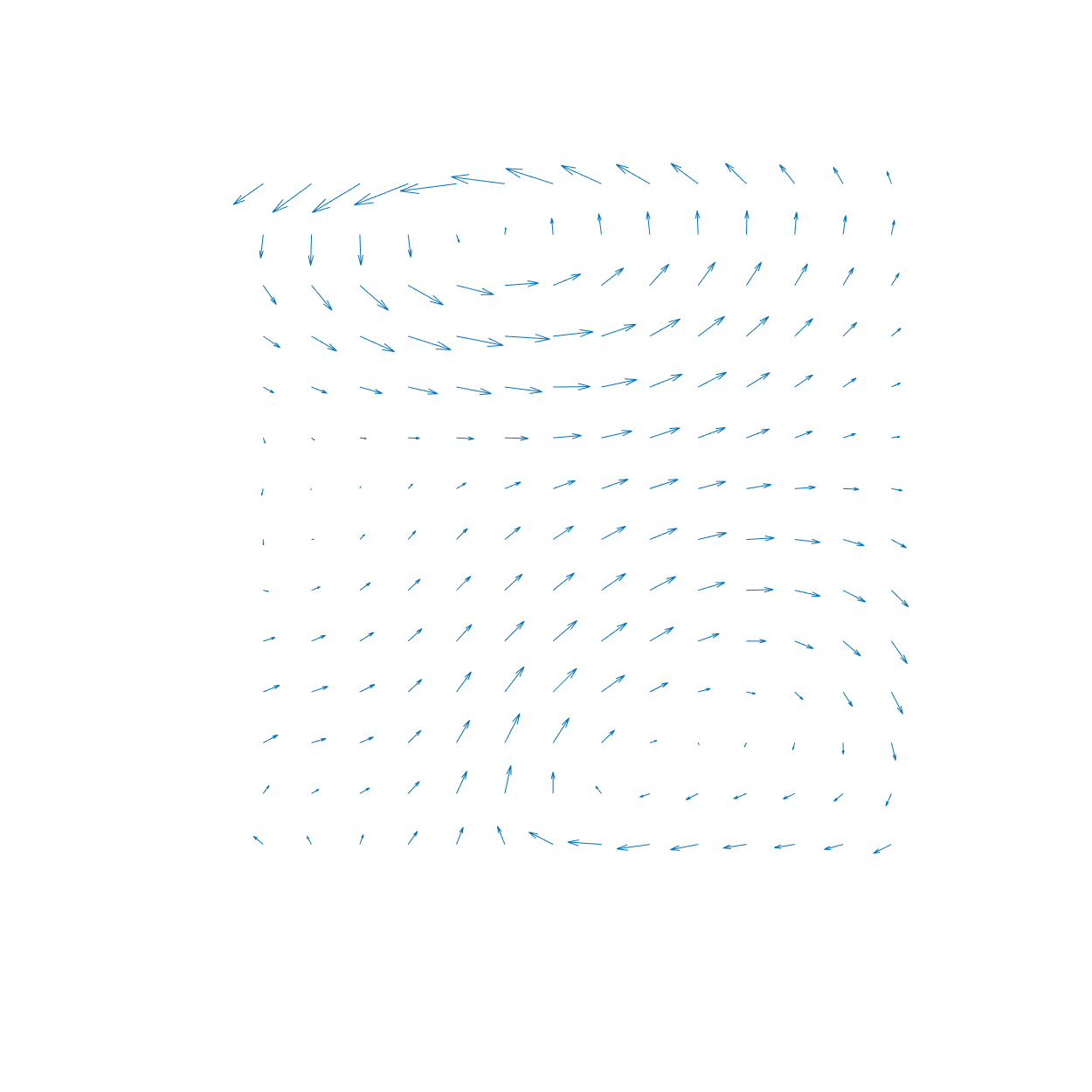

Supplement: S2 MCG raw data 2 — The raw MCG dataset includes categories 0-3 for training and validation. (ZIP) [file pone.0338189.s002.zip › train/0/p3_435_3.png]

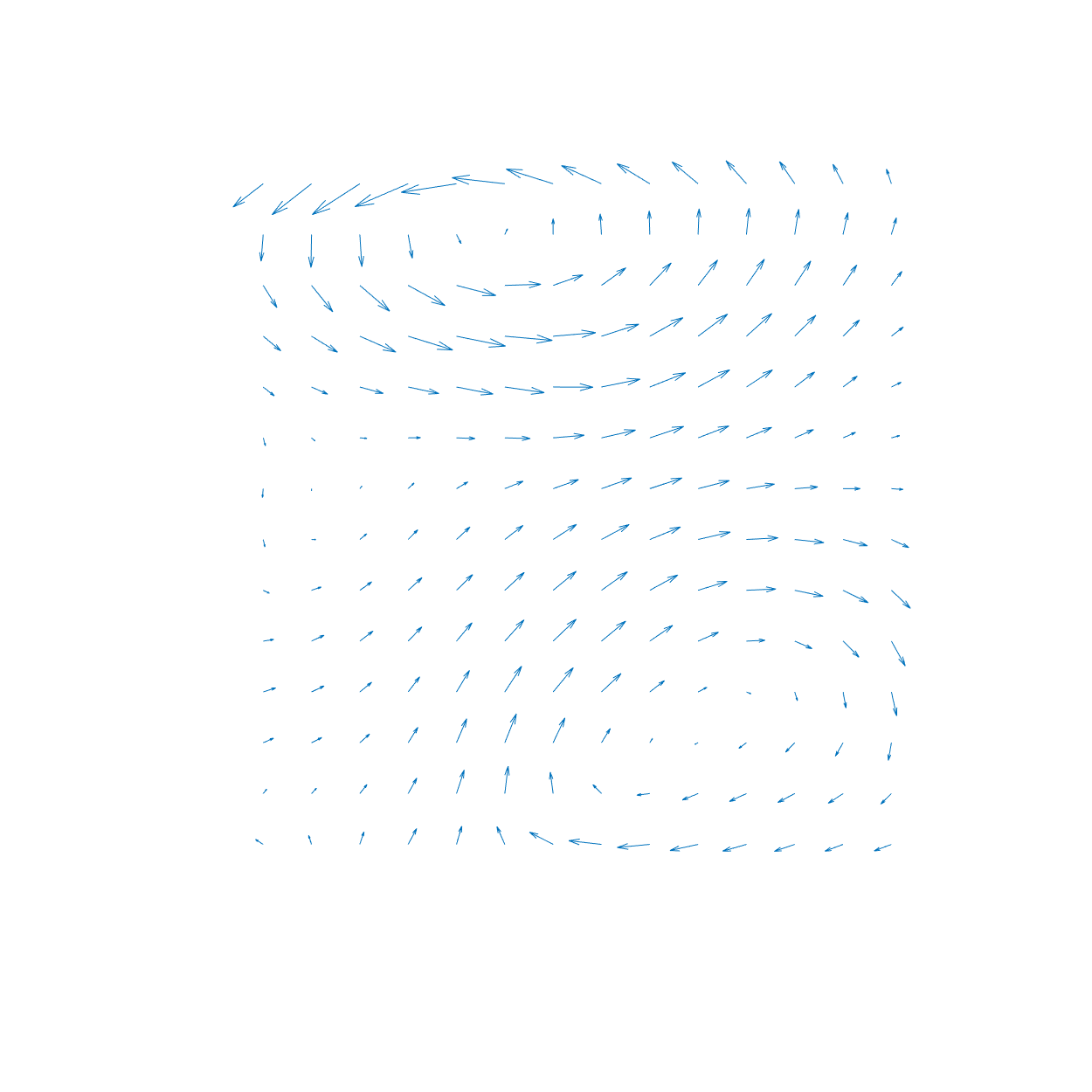

Supplement: S2 MCG raw data 2 — The raw MCG dataset includes categories 0-3 for training and validation. (ZIP) [file pone.0338189.s002.zip › train/0/p3_440_1.png]

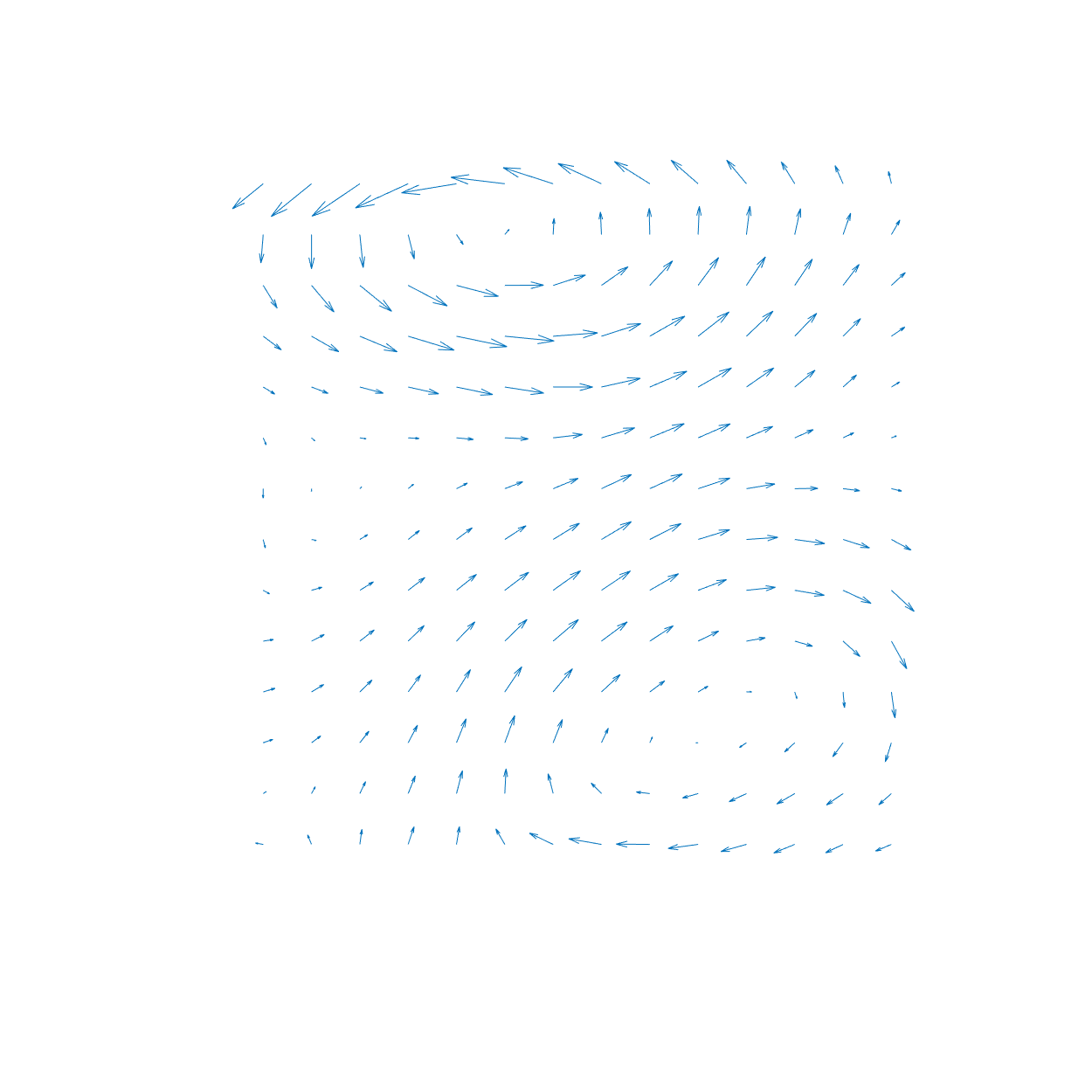

Supplement: S2 MCG raw data 2 — The raw MCG dataset includes categories 0-3 for training and validation. (ZIP) [file pone.0338189.s002.zip › train/0/p3_440_2.png]

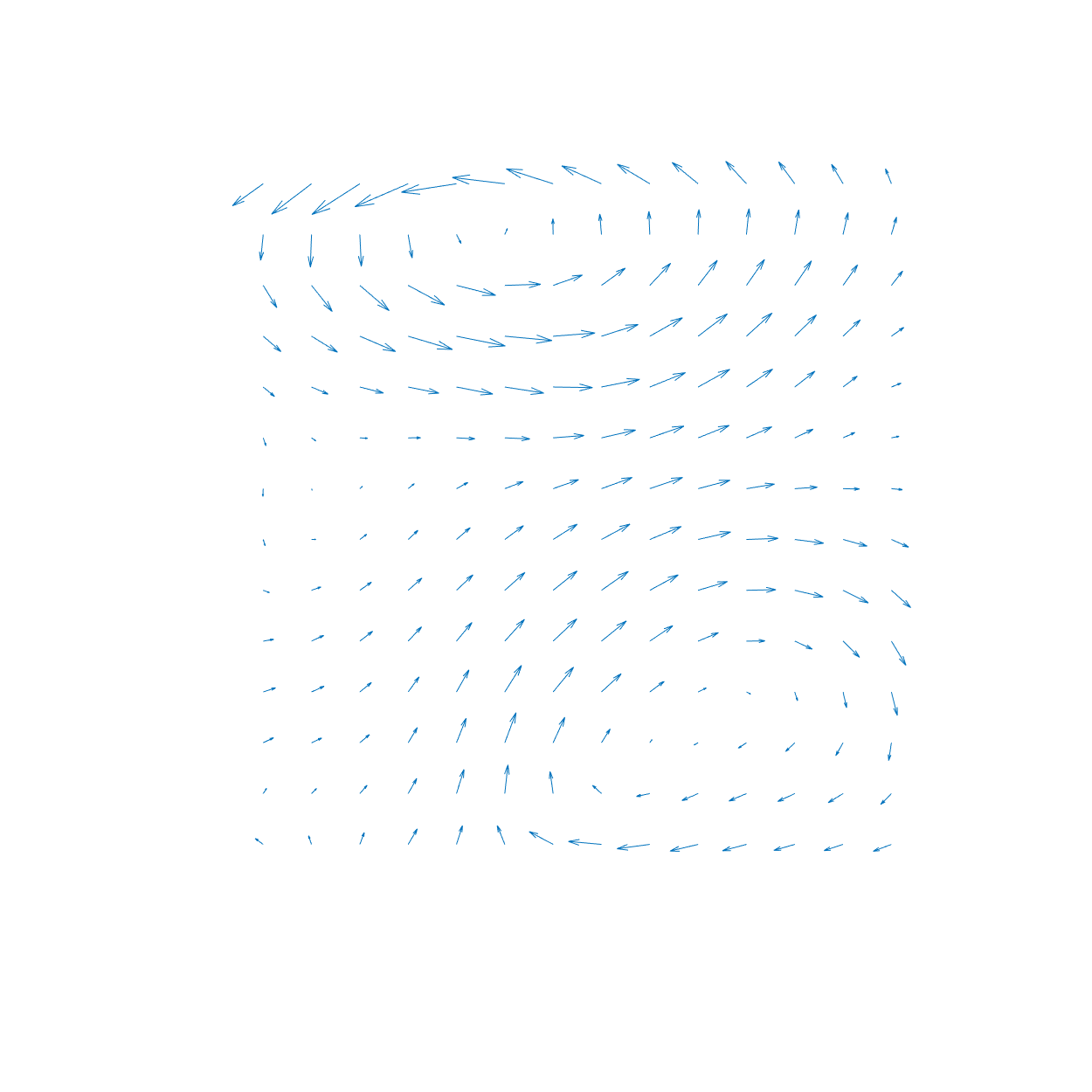

Supplement: S2 MCG raw data 2 — The raw MCG dataset includes categories 0-3 for training and validation. (ZIP) [file pone.0338189.s002.zip › train/0/p3_440_3.png]

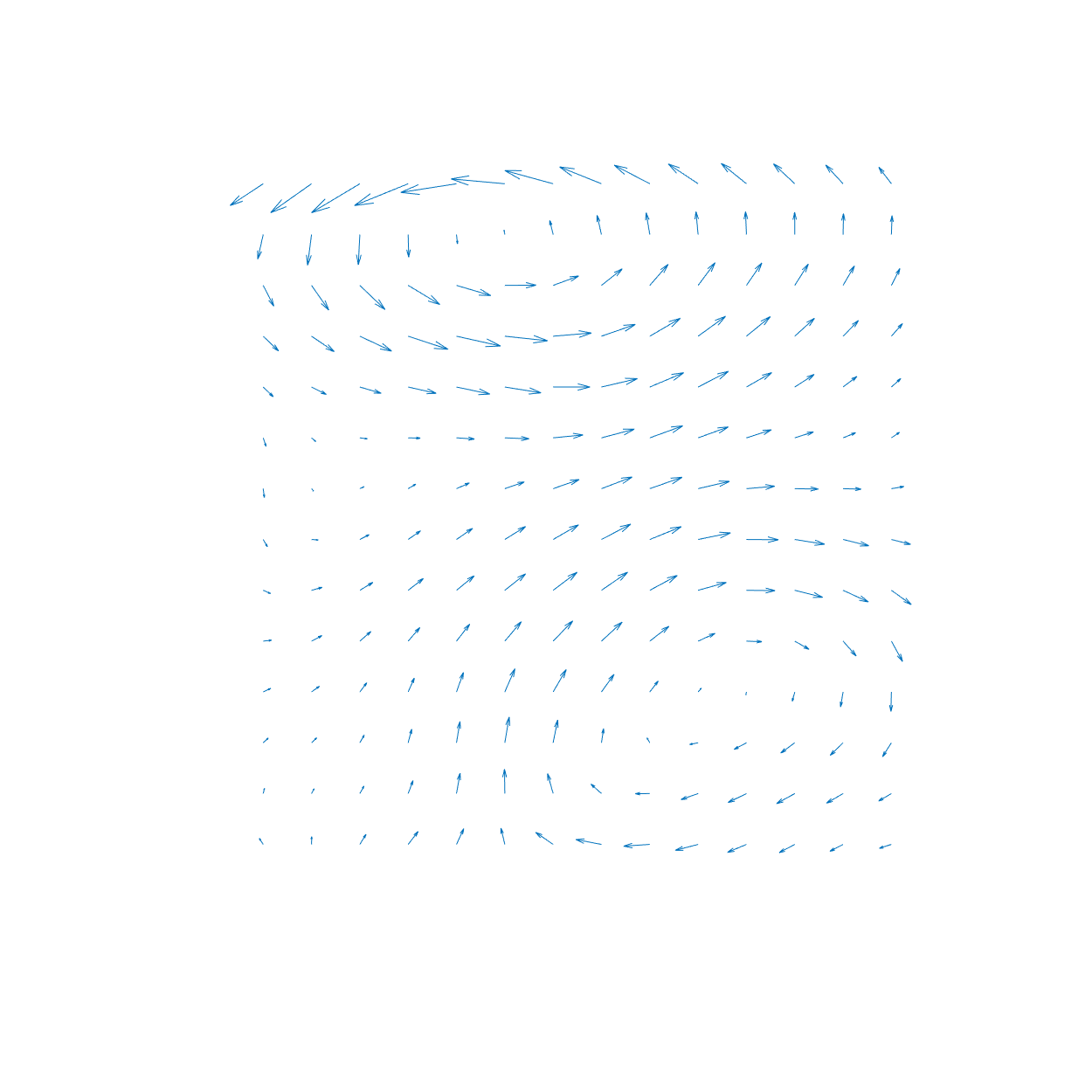

Supplement: S2 MCG raw data 2 — The raw MCG dataset includes categories 0-3 for training and validation. (ZIP) [file pone.0338189.s002.zip › train/0/p3_445_1.png]

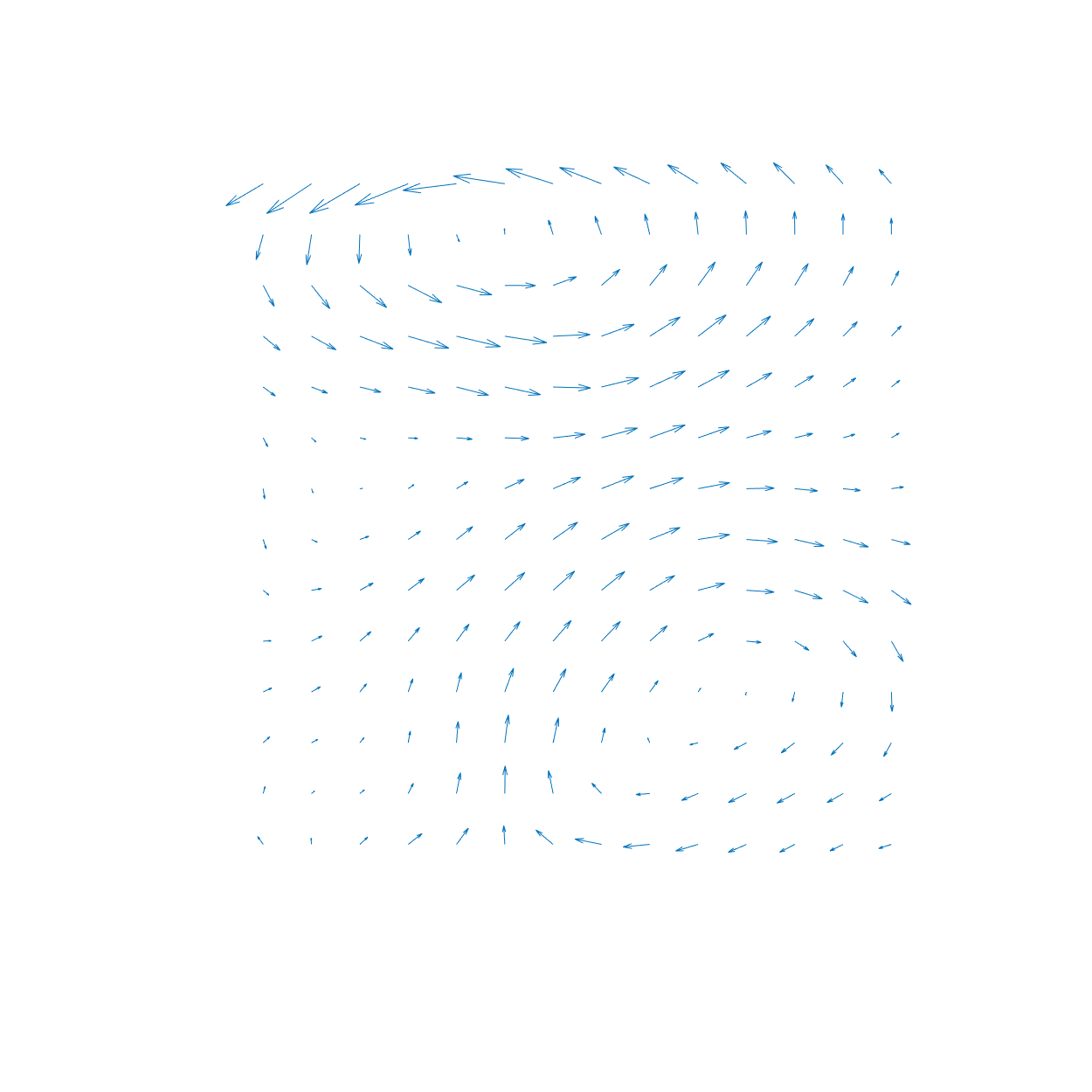

Supplement: S2 MCG raw data 2 — The raw MCG dataset includes categories 0-3 for training and validation. (ZIP) [file pone.0338189.s002.zip › train/0/p3_445_2.png]

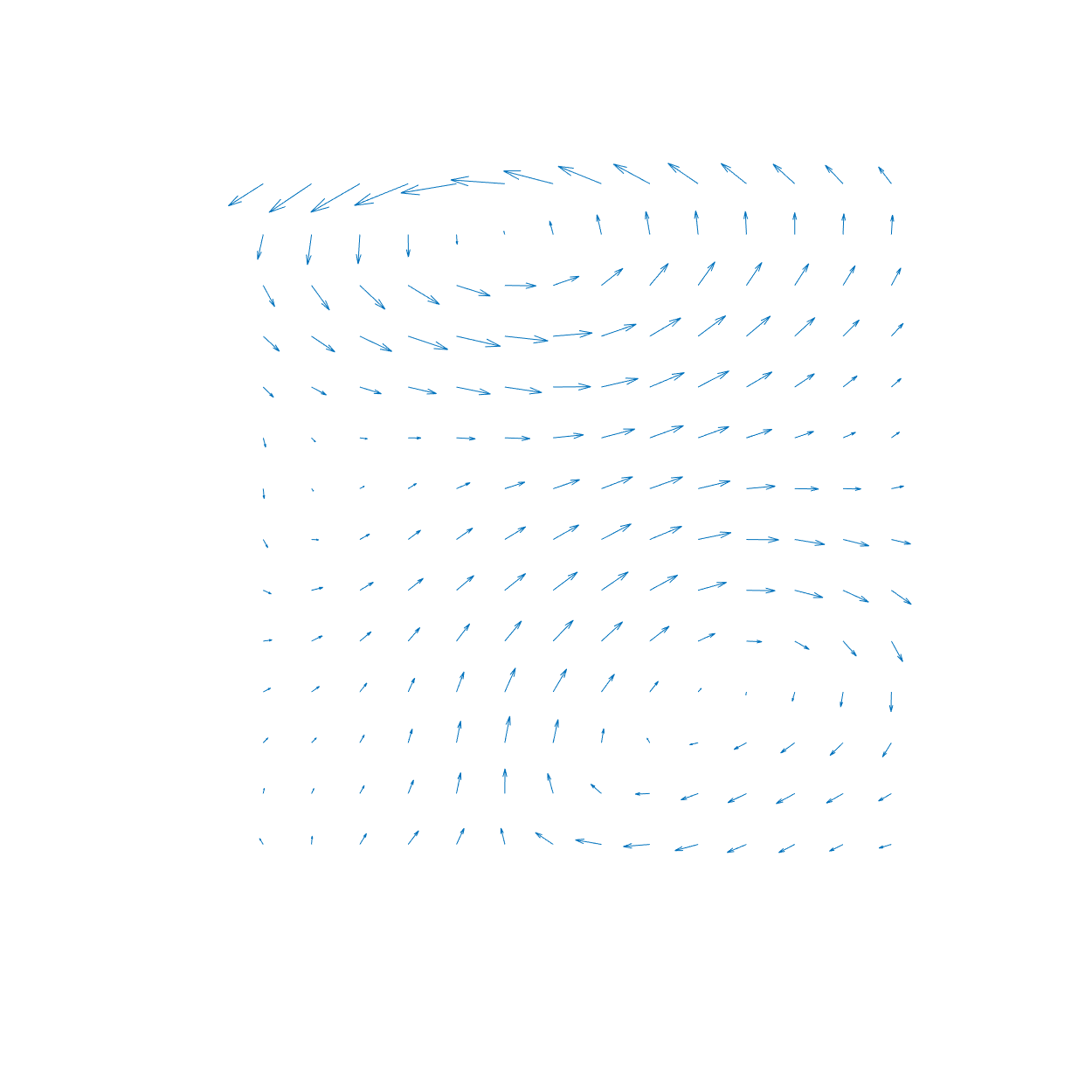

Supplement: S2 MCG raw data 2 — The raw MCG dataset includes categories 0-3 for training and validation. (ZIP) [file pone.0338189.s002.zip › train/0/p3_445_3.png]

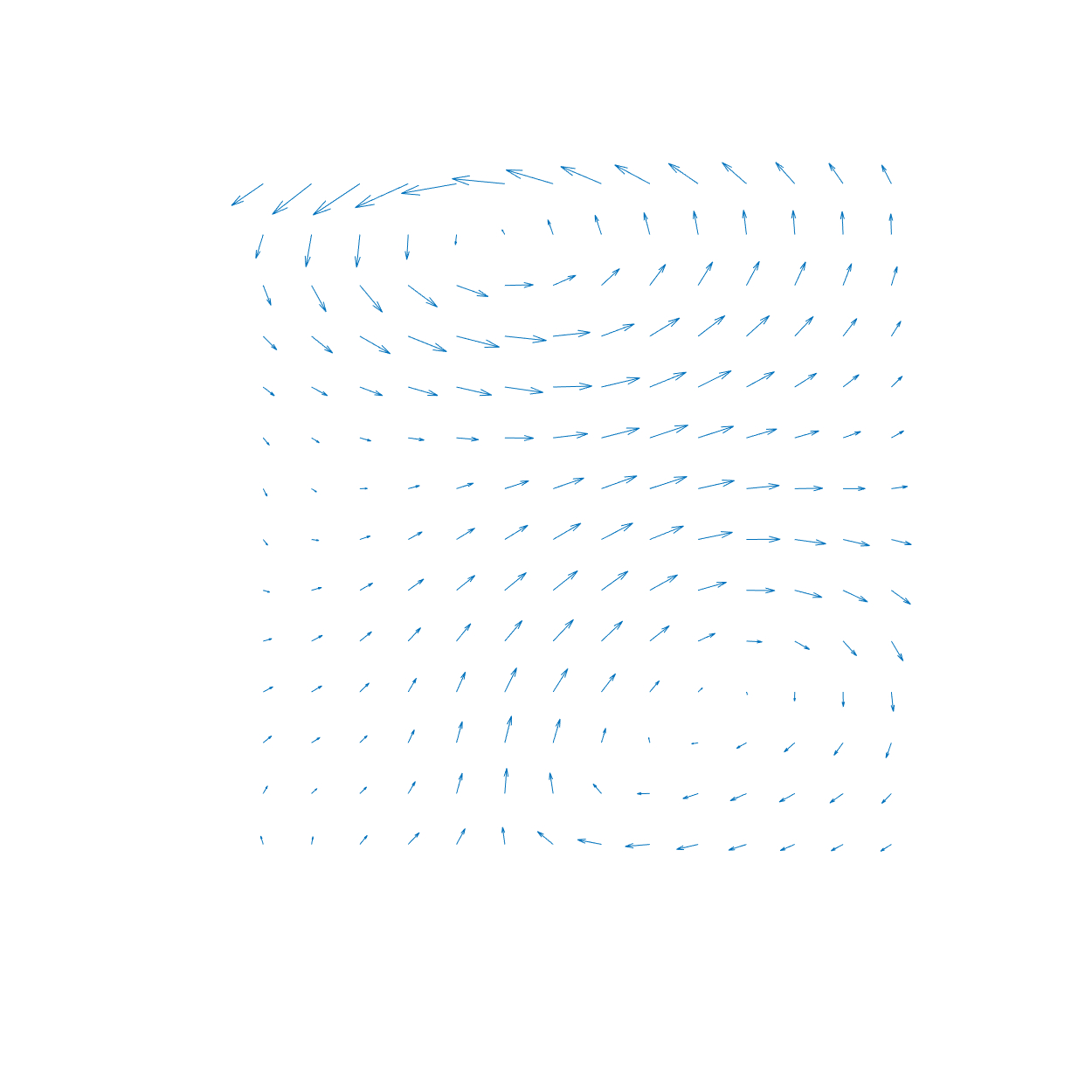

Supplement: S2 MCG raw data 2 — The raw MCG dataset includes categories 0-3 for training and validation. (ZIP) [file pone.0338189.s002.zip › train/0/p3_450_1.png]

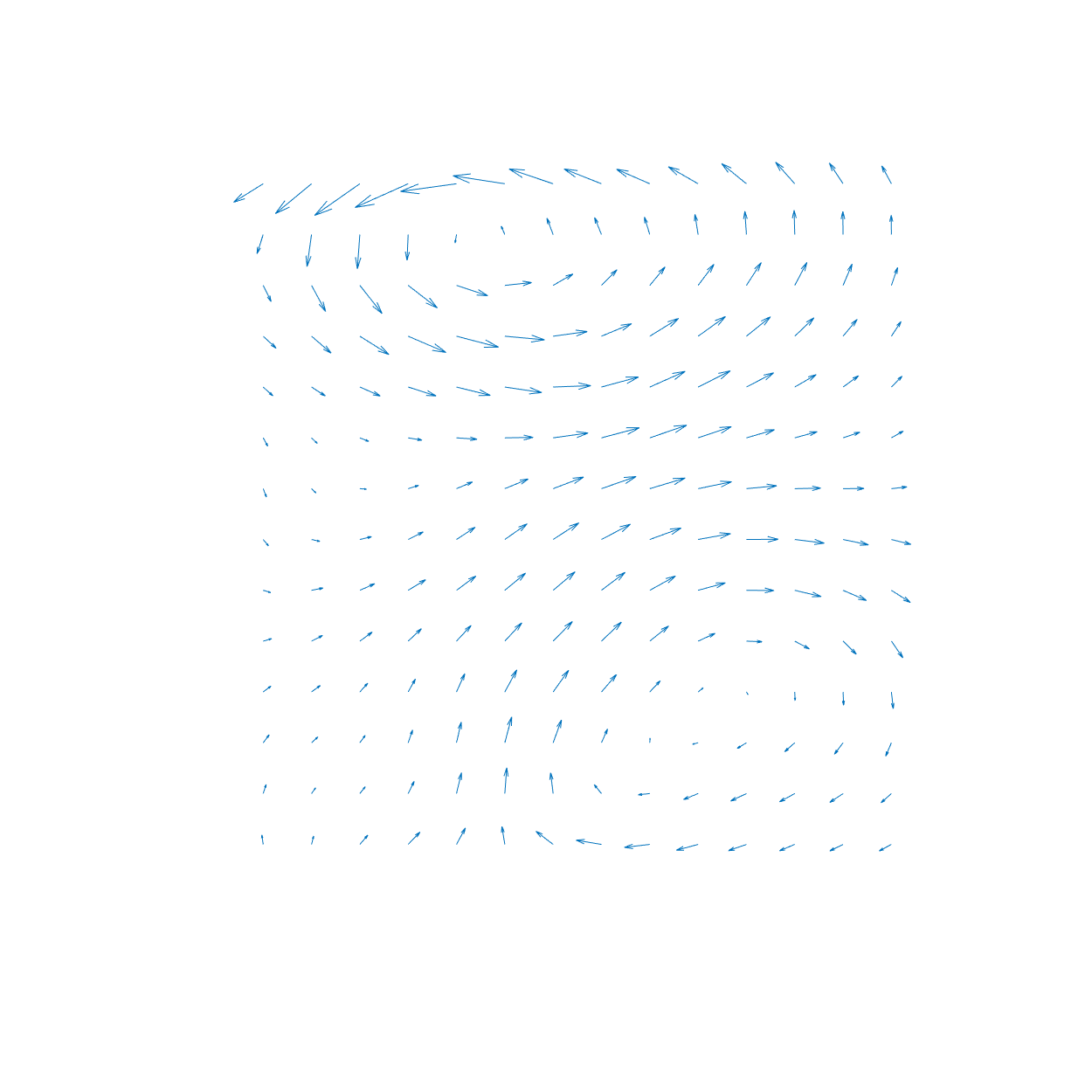

Supplement: S2 MCG raw data 2 — The raw MCG dataset includes categories 0-3 for training and validation. (ZIP) [file pone.0338189.s002.zip › train/0/p3_450_2.png]

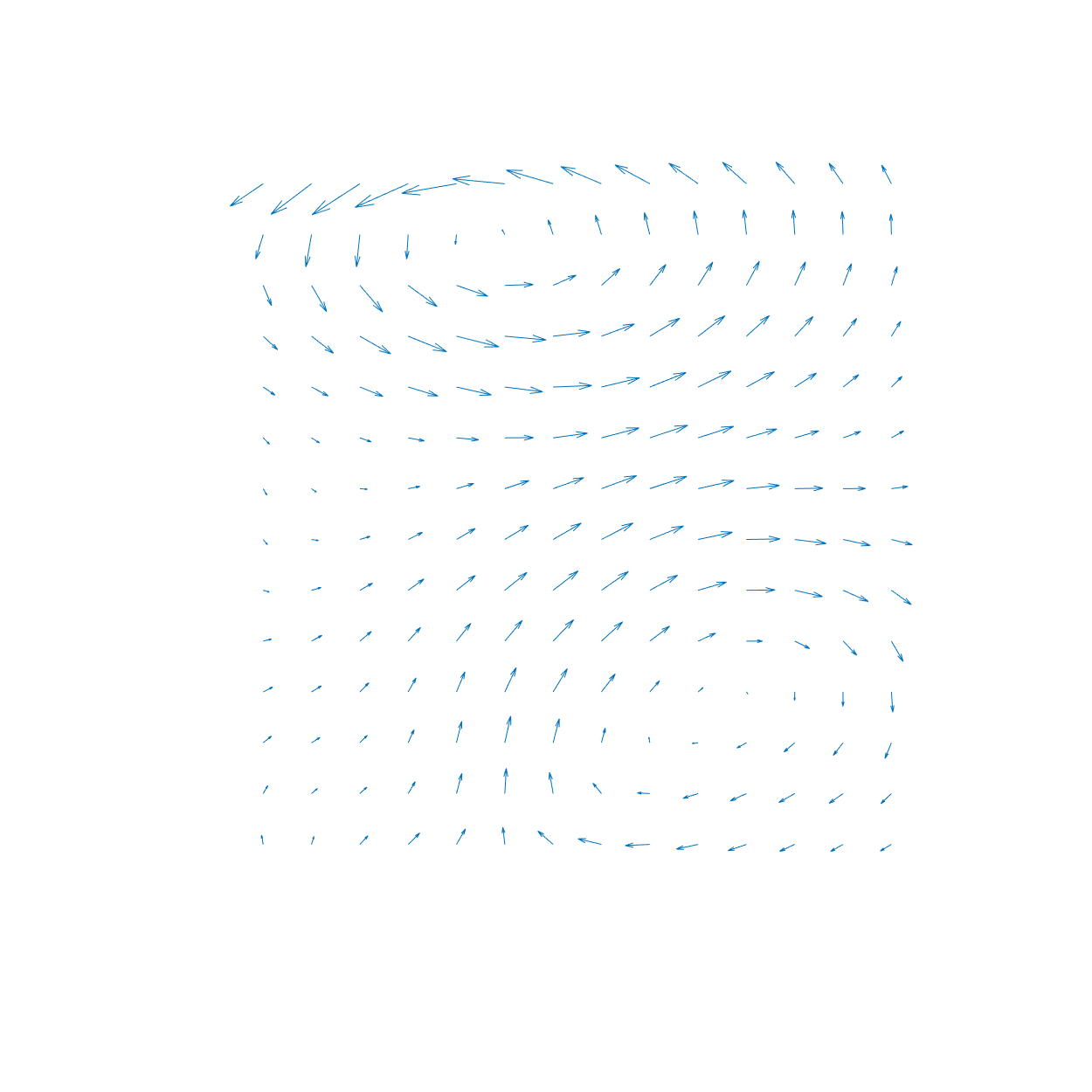

Supplement: S2 MCG raw data 2 — The raw MCG dataset includes categories 0-3 for training and validation. (ZIP) [file pone.0338189.s002.zip › train/0/p3_450_3.png]

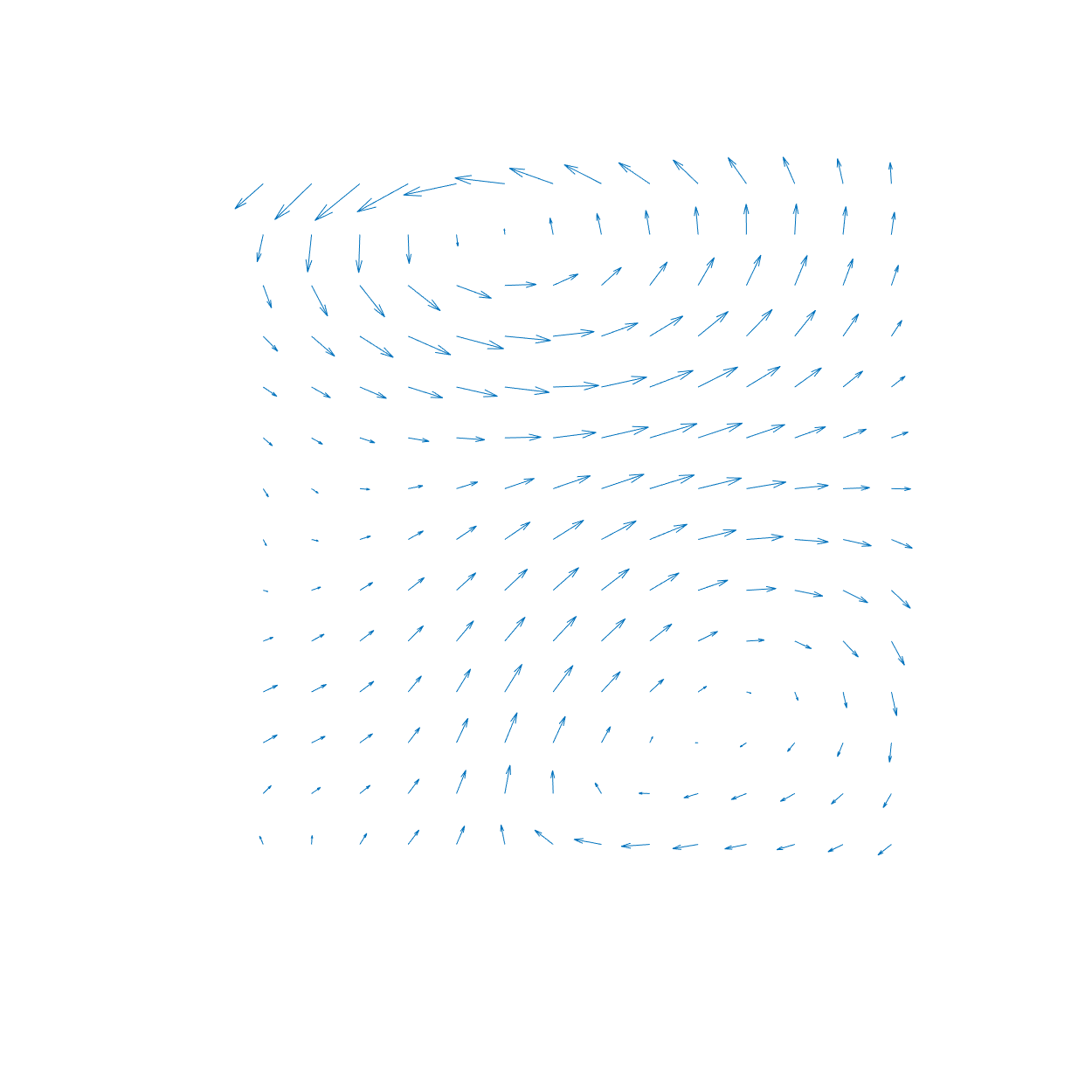

Supplement: S2 MCG raw data 2 — The raw MCG dataset includes categories 0-3 for training and validation. (ZIP) [file pone.0338189.s002.zip › train/0/p3_455_1.png]

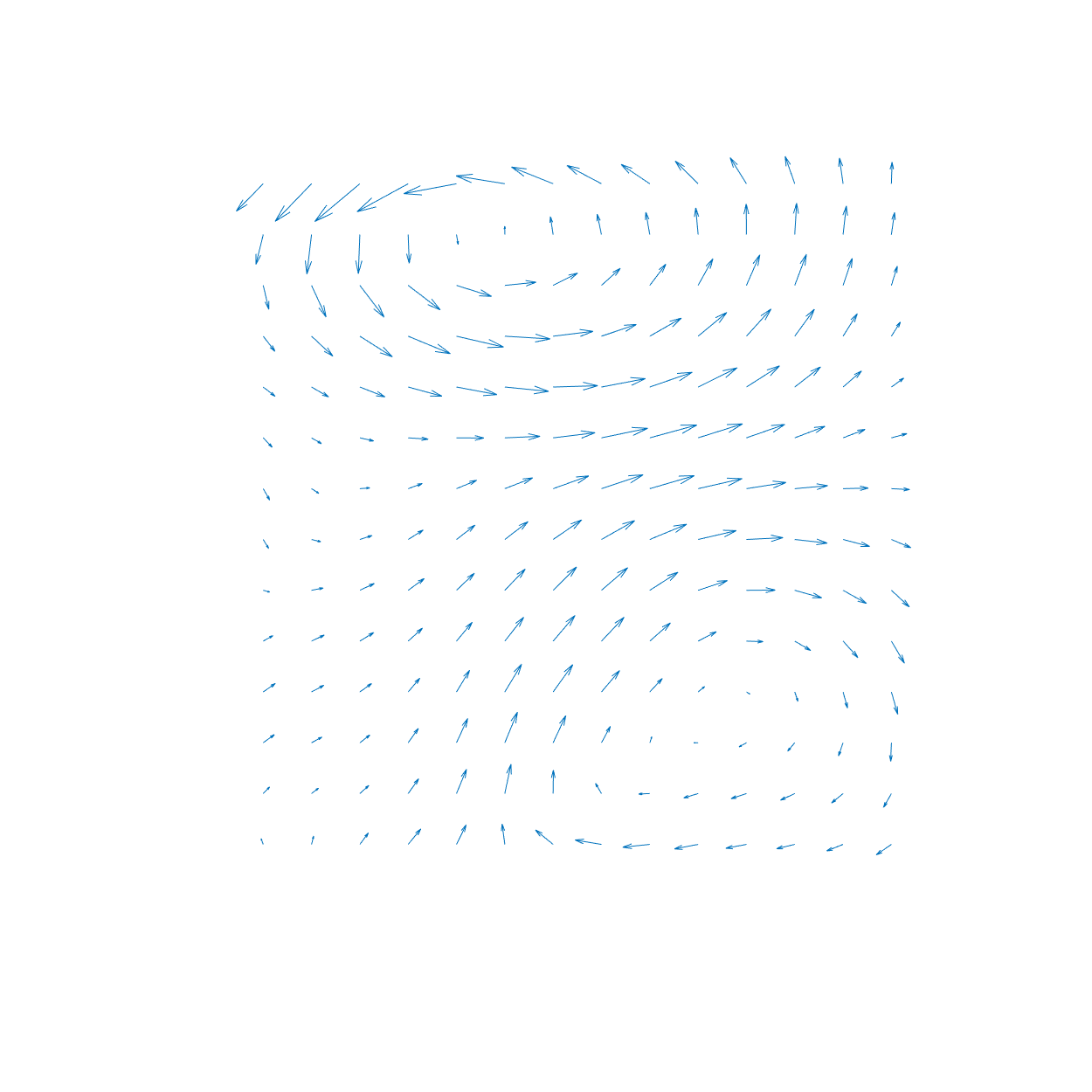

Supplement: S2 MCG raw data 2 — The raw MCG dataset includes categories 0-3 for training and validation. (ZIP) [file pone.0338189.s002.zip › train/0/p3_455_2.png]

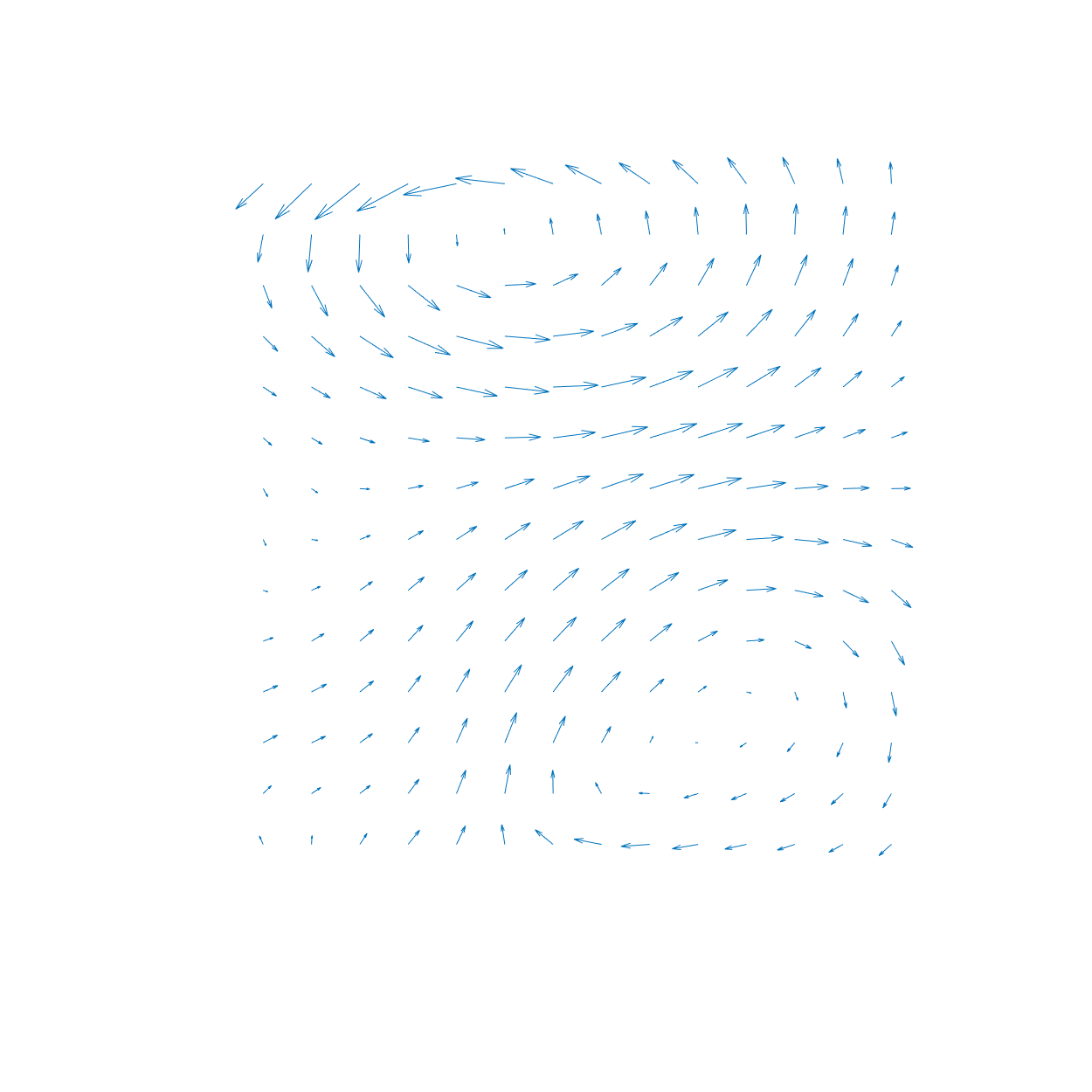

Supplement: S2 MCG raw data 2 — The raw MCG dataset includes categories 0-3 for training and validation. (ZIP) [file pone.0338189.s002.zip › train/0/p3_455_3.png]

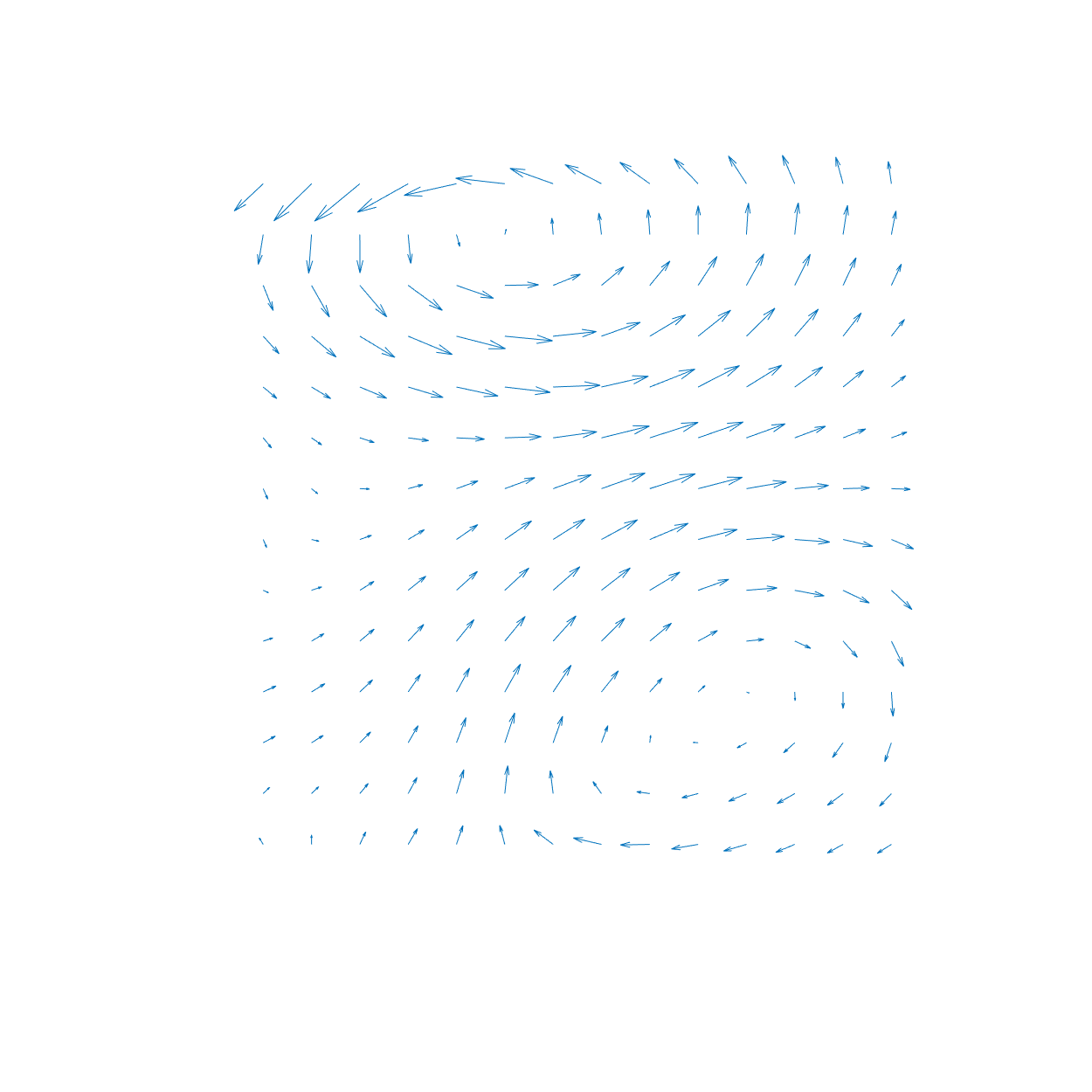

Supplement: S2 MCG raw data 2 — The raw MCG dataset includes categories 0-3 for training and validation. (ZIP) [file pone.0338189.s002.zip › train/0/p3_460_1.png]

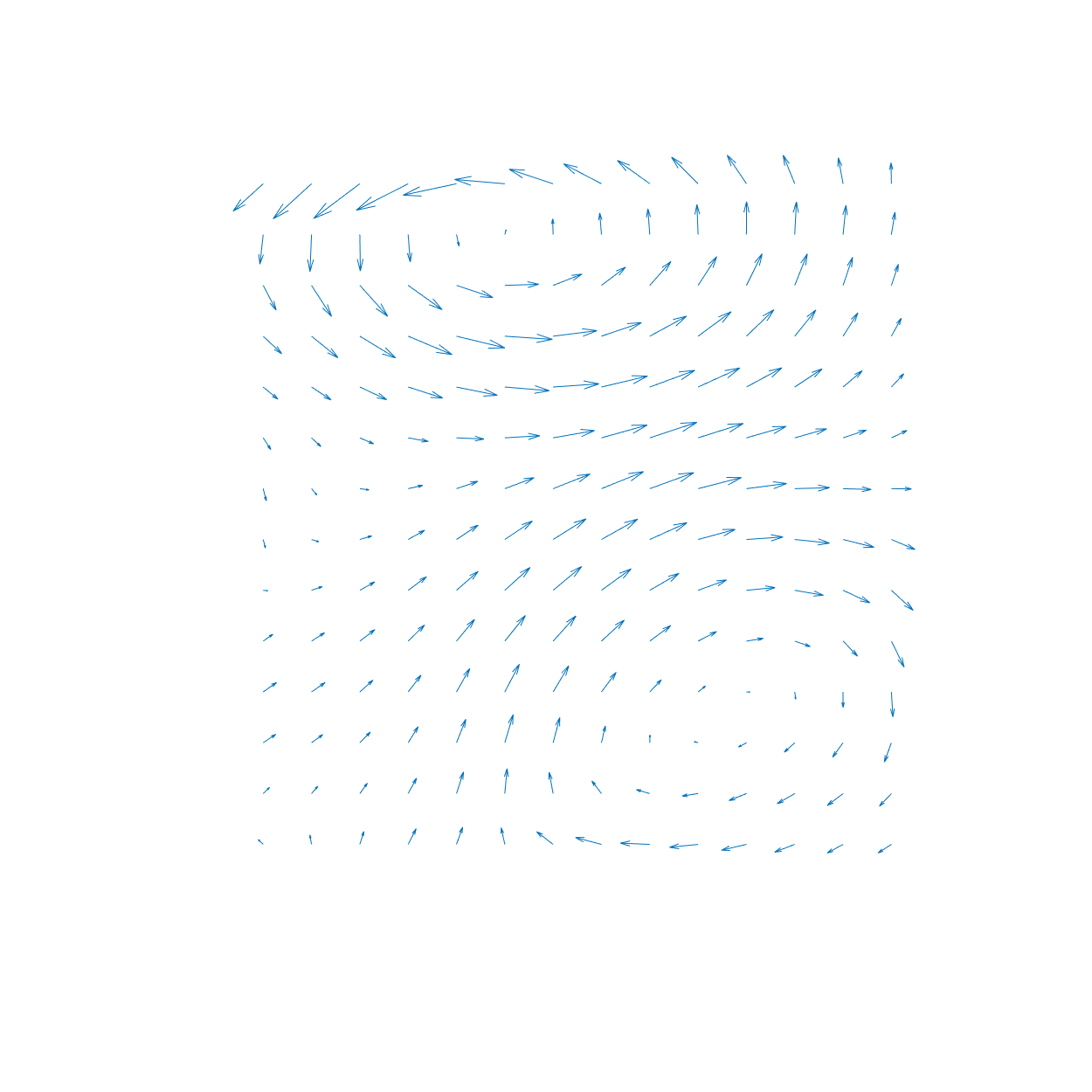

Supplement: S2 MCG raw data 2 — The raw MCG dataset includes categories 0-3 for training and validation. (ZIP) [file pone.0338189.s002.zip › train/0/p3_460_2.png]

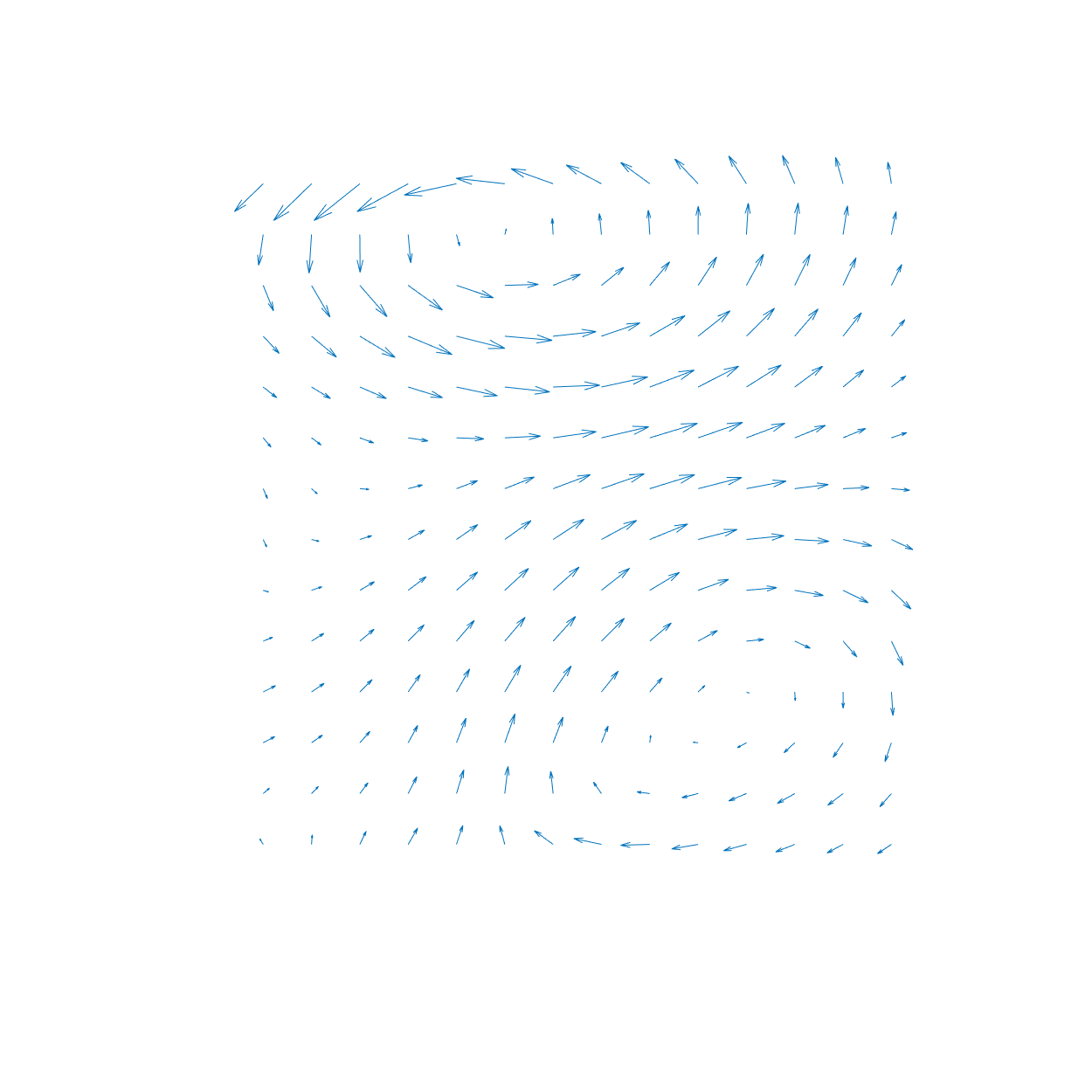

Supplement: S2 MCG raw data 2 — The raw MCG dataset includes categories 0-3 for training and validation. (ZIP) [file pone.0338189.s002.zip › train/0/p3_460_3.png]

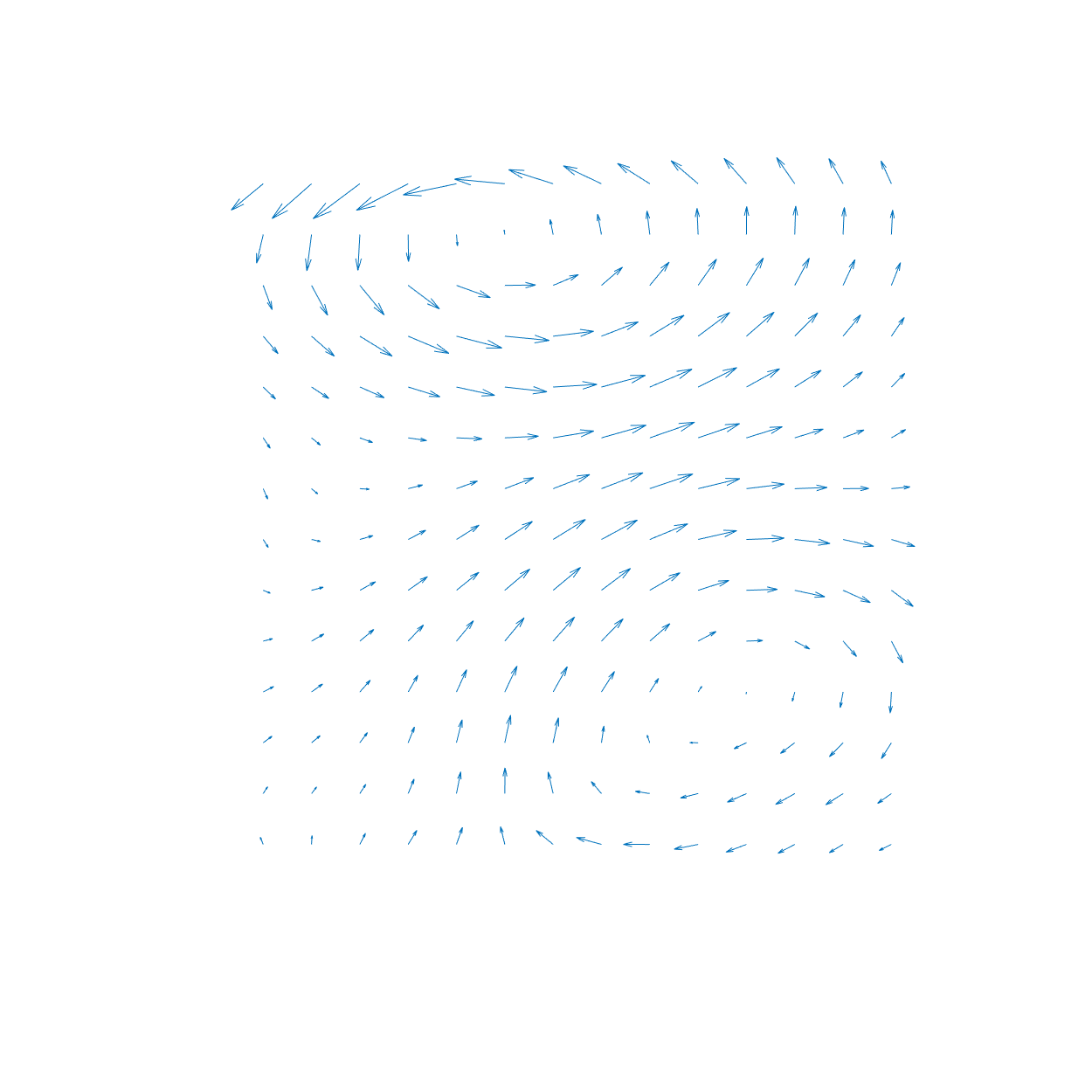

Supplement: S2 MCG raw data 2 — The raw MCG dataset includes categories 0-3 for training and validation. (ZIP) [file pone.0338189.s002.zip › train/0/p3_465_1.png]

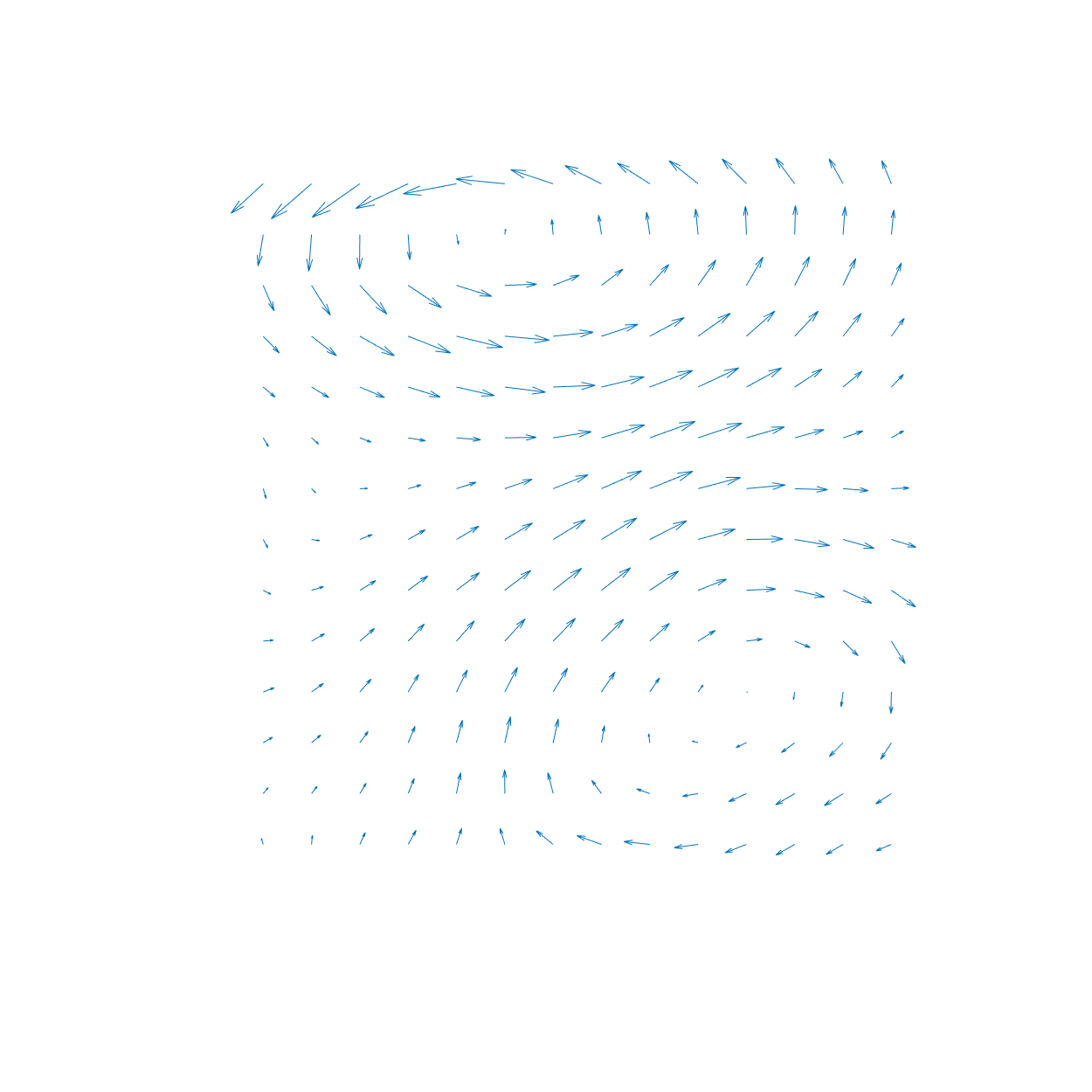

Supplement: S2 MCG raw data 2 — The raw MCG dataset includes categories 0-3 for training and validation. (ZIP) [file pone.0338189.s002.zip › train/0/p3_465_2.png]

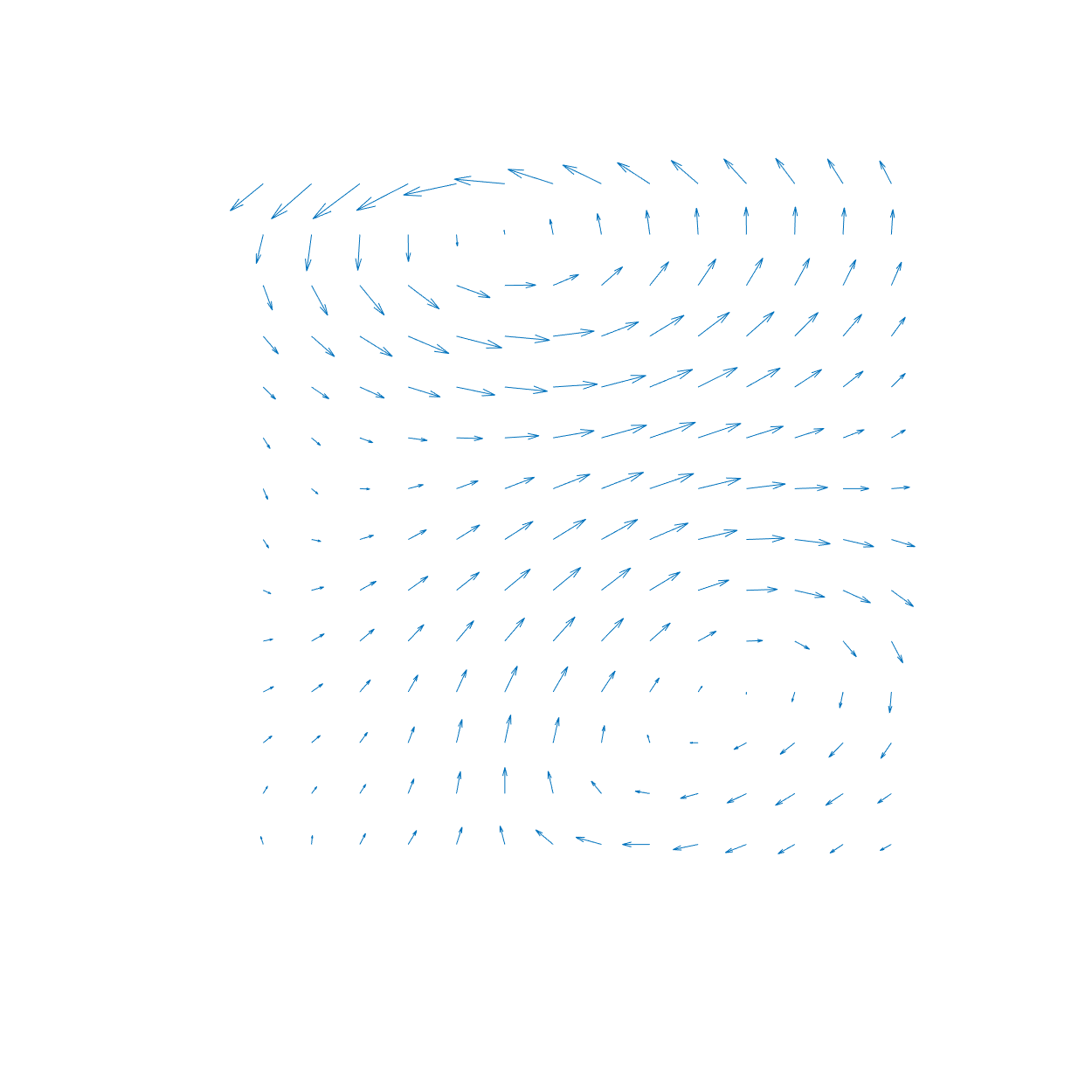

Supplement: S2 MCG raw data 2 — The raw MCG dataset includes categories 0-3 for training and validation. (ZIP) [file pone.0338189.s002.zip › train/0/p3_465_3.png]

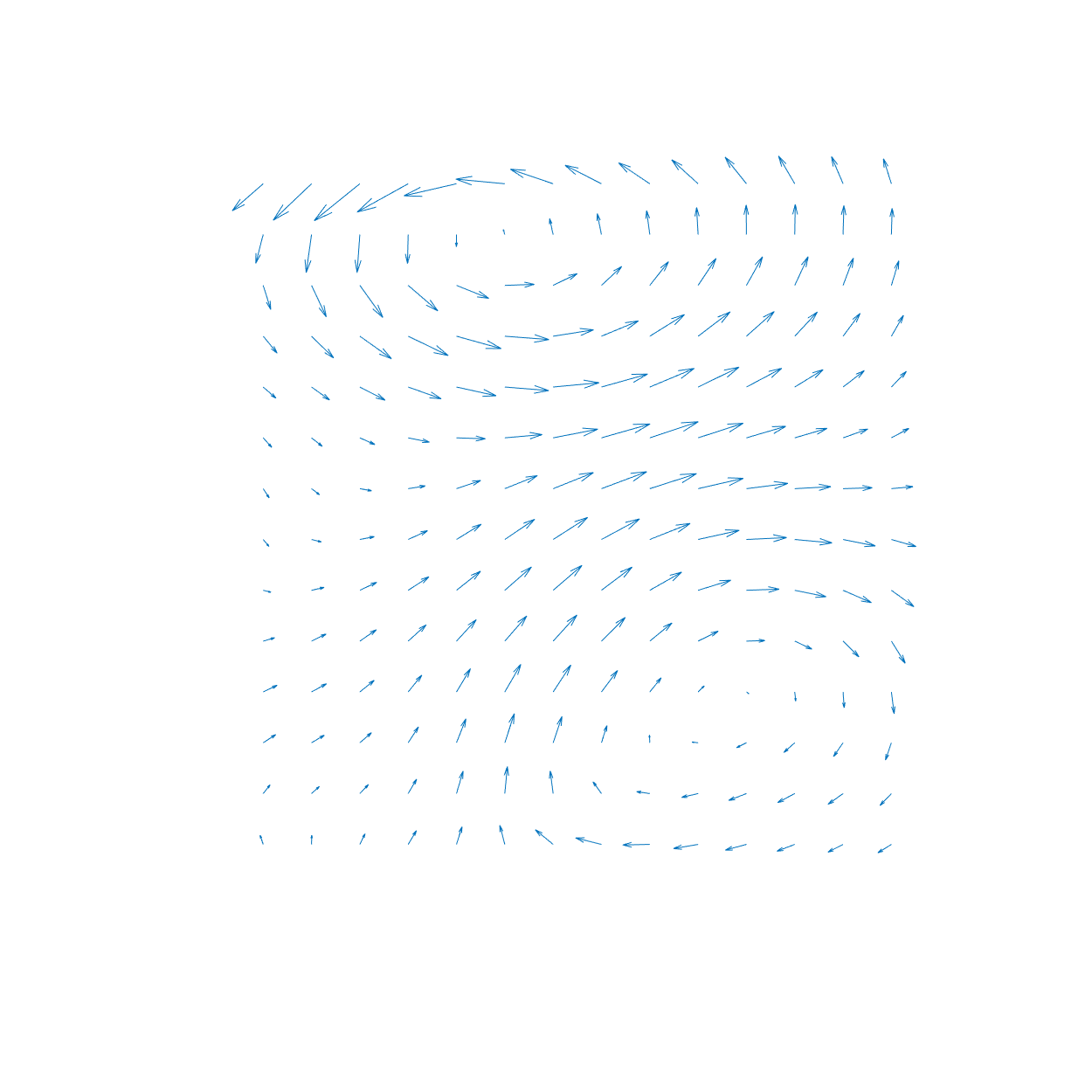

Supplement: S2 MCG raw data 2 — The raw MCG dataset includes categories 0-3 for training and validation. (ZIP) [file pone.0338189.s002.zip › train/0/p3_470_1.png]

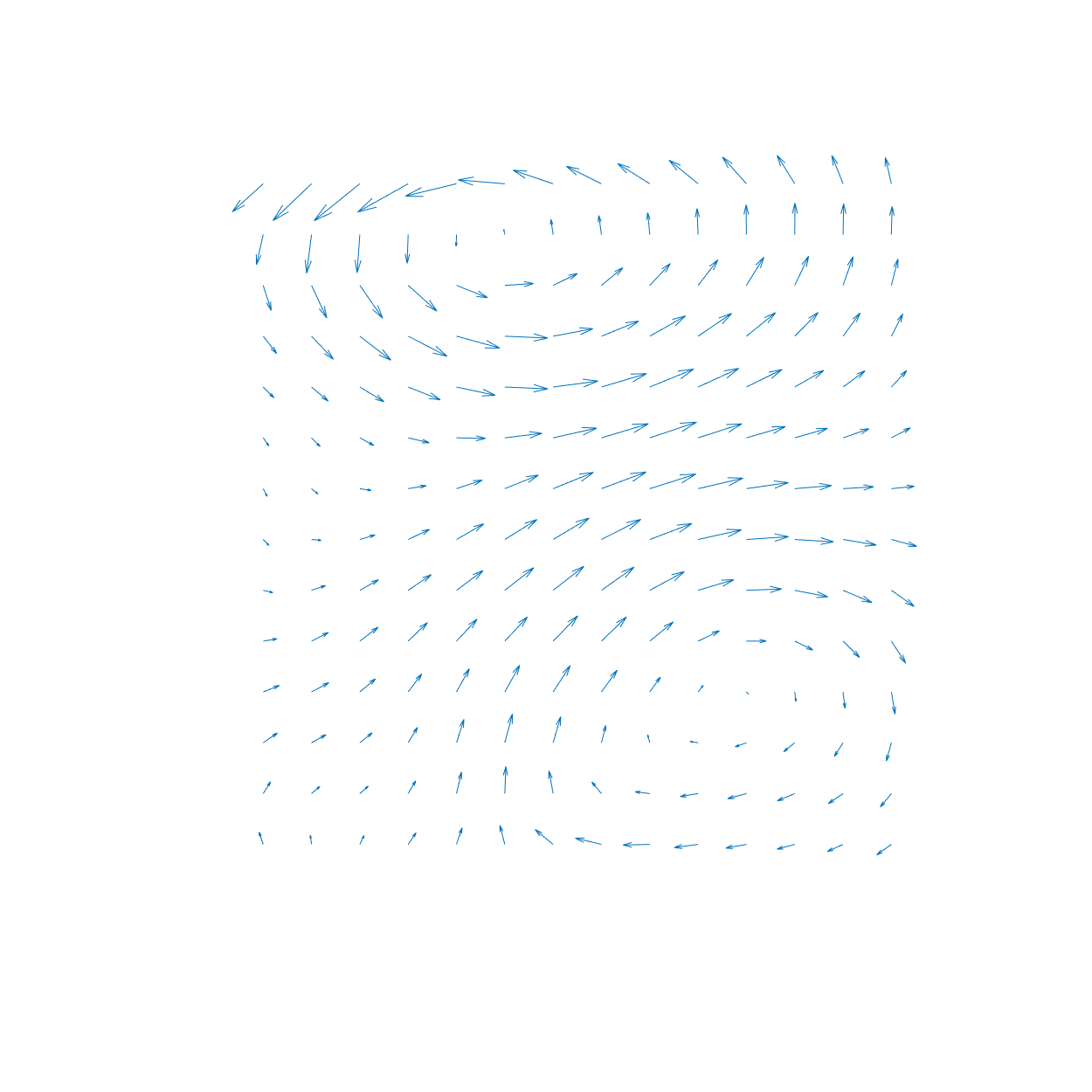

Supplement: S2 MCG raw data 2 — The raw MCG dataset includes categories 0-3 for training and validation. (ZIP) [file pone.0338189.s002.zip › train/0/p3_470_2.png]

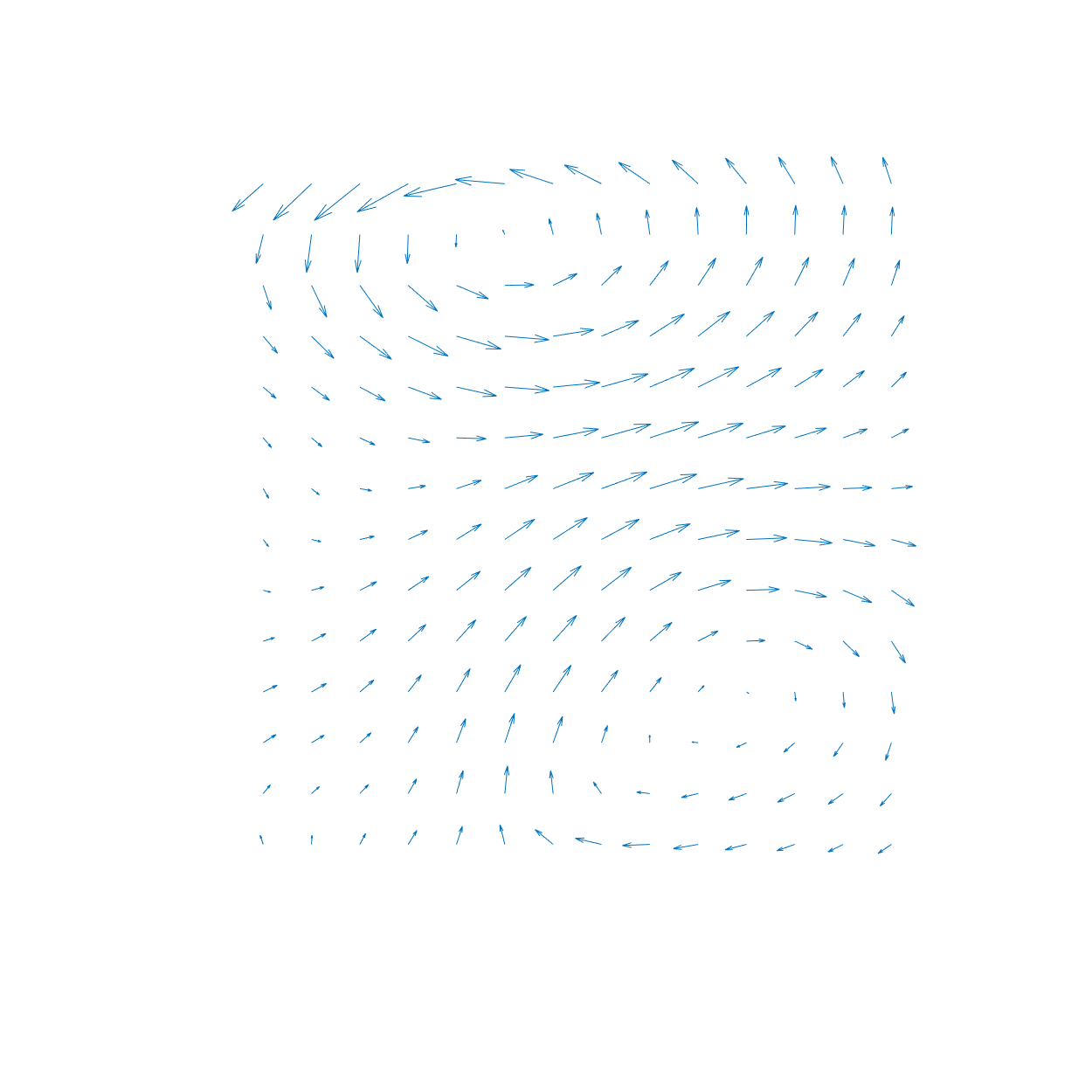

Supplement: S2 MCG raw data 2 — The raw MCG dataset includes categories 0-3 for training and validation. (ZIP) [file pone.0338189.s002.zip › train/0/p3_470_3.png]

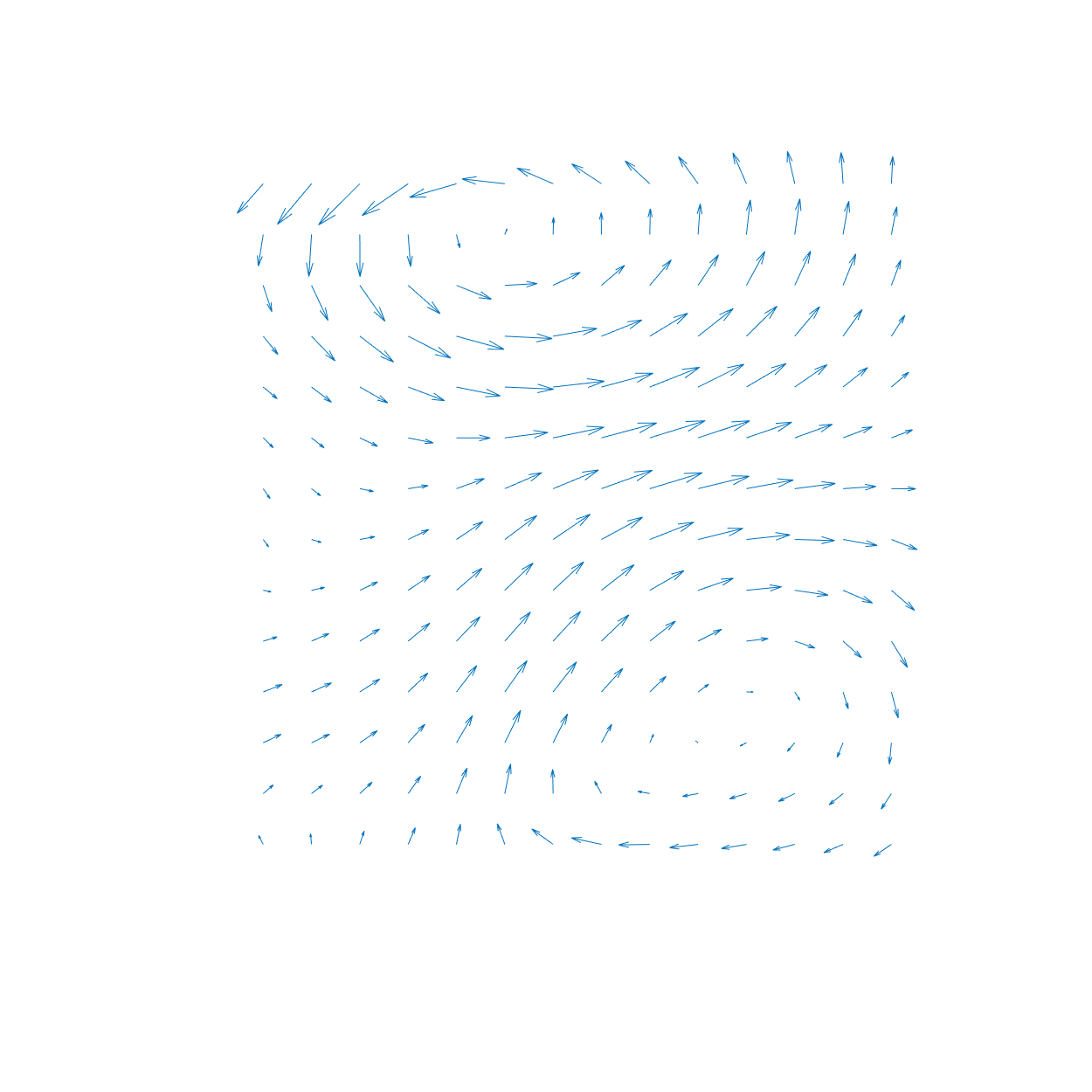

Supplement: S2 MCG raw data 2 — The raw MCG dataset includes categories 0-3 for training and validation. (ZIP) [file pone.0338189.s002.zip › train/0/p3_475_1.png]

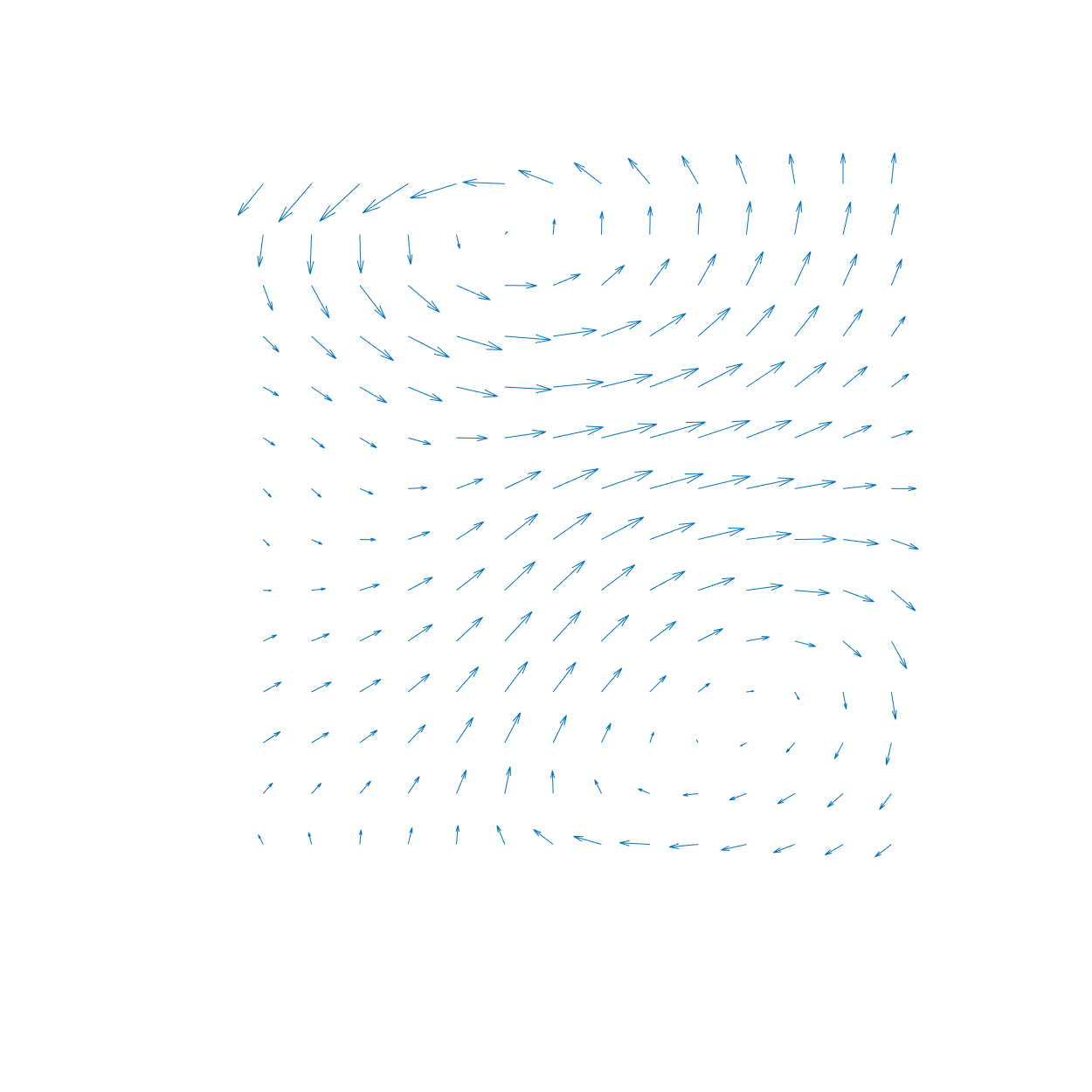

Supplement: S2 MCG raw data 2 — The raw MCG dataset includes categories 0-3 for training and validation. (ZIP) [file pone.0338189.s002.zip › train/0/p3_475_2.png]

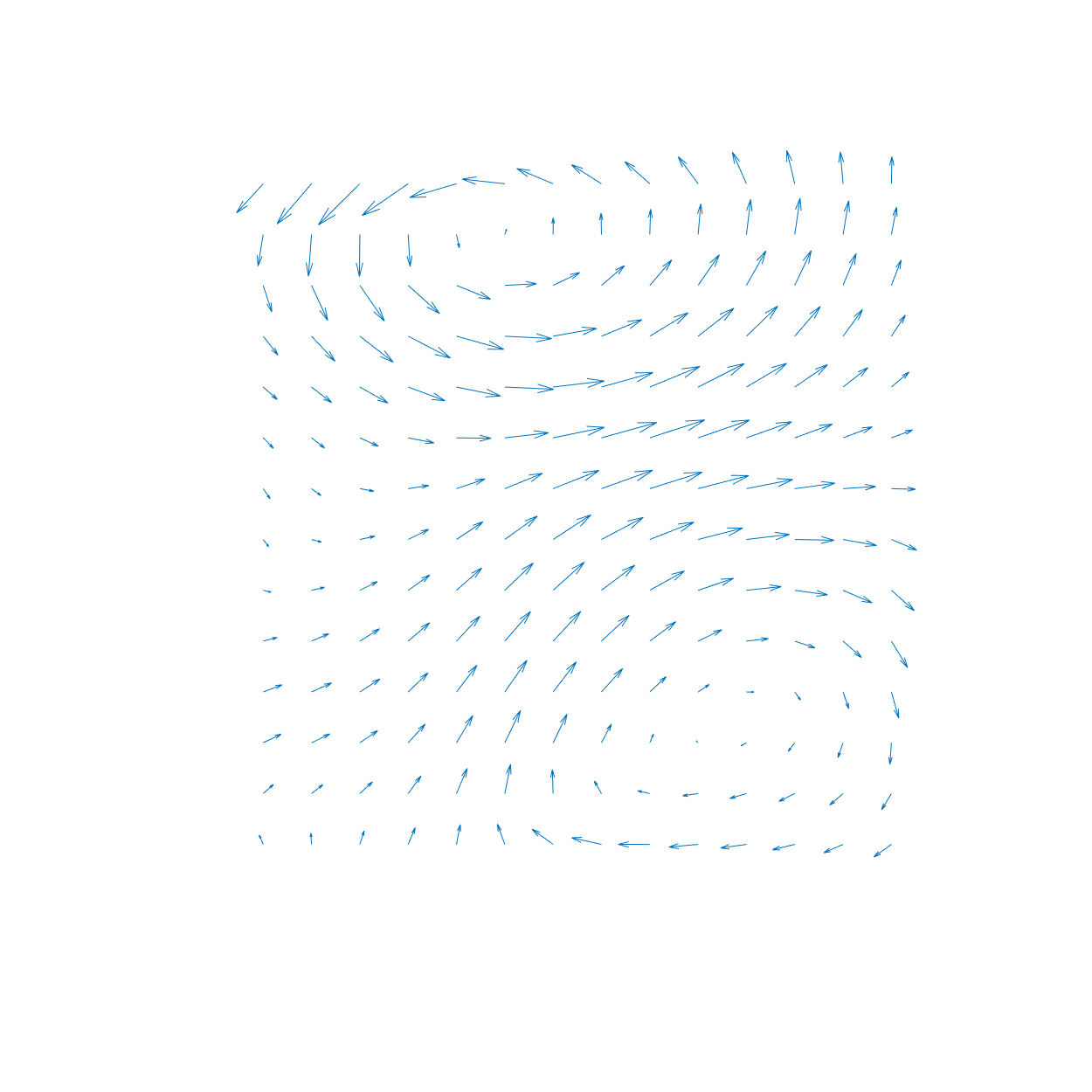

Supplement: S2 MCG raw data 2 — The raw MCG dataset includes categories 0-3 for training and validation. (ZIP) [file pone.0338189.s002.zip › train/0/p3_475_3.png]

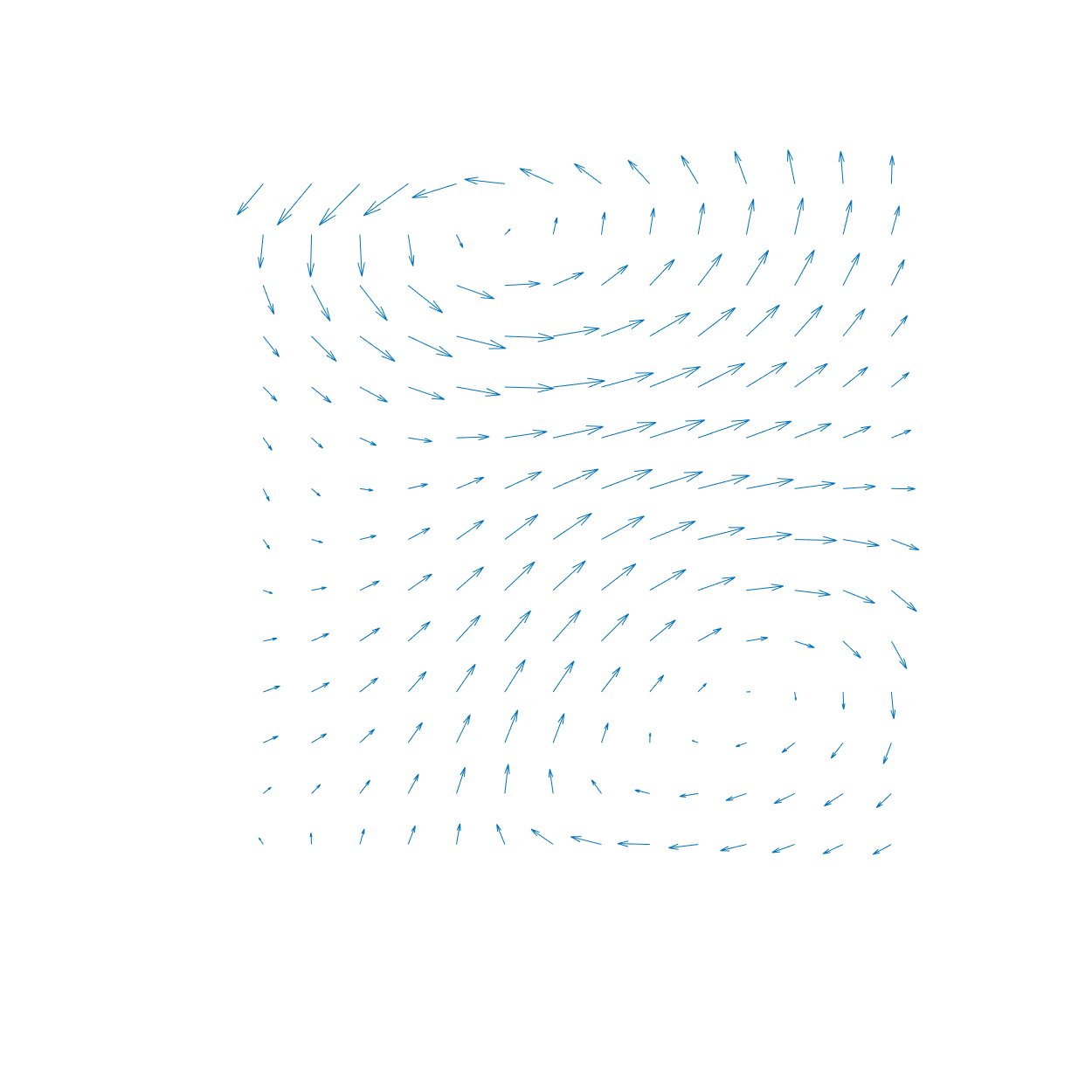

Supplement: S2 MCG raw data 2 — The raw MCG dataset includes categories 0-3 for training and validation. (ZIP) [file pone.0338189.s002.zip › train/0/p3_480_1.png]

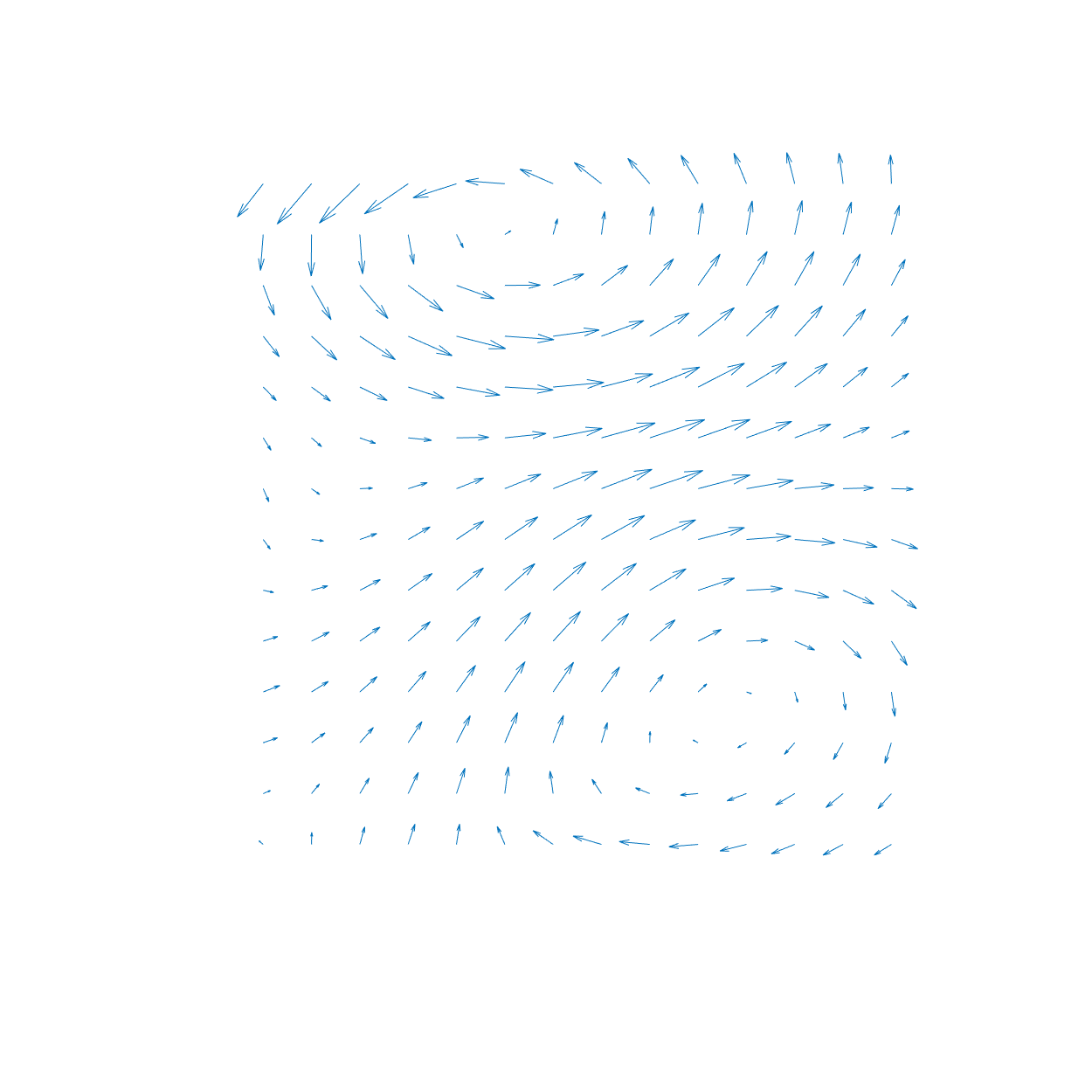

Supplement: S2 MCG raw data 2 — The raw MCG dataset includes categories 0-3 for training and validation. (ZIP) [file pone.0338189.s002.zip › train/0/p3_480_2.png]

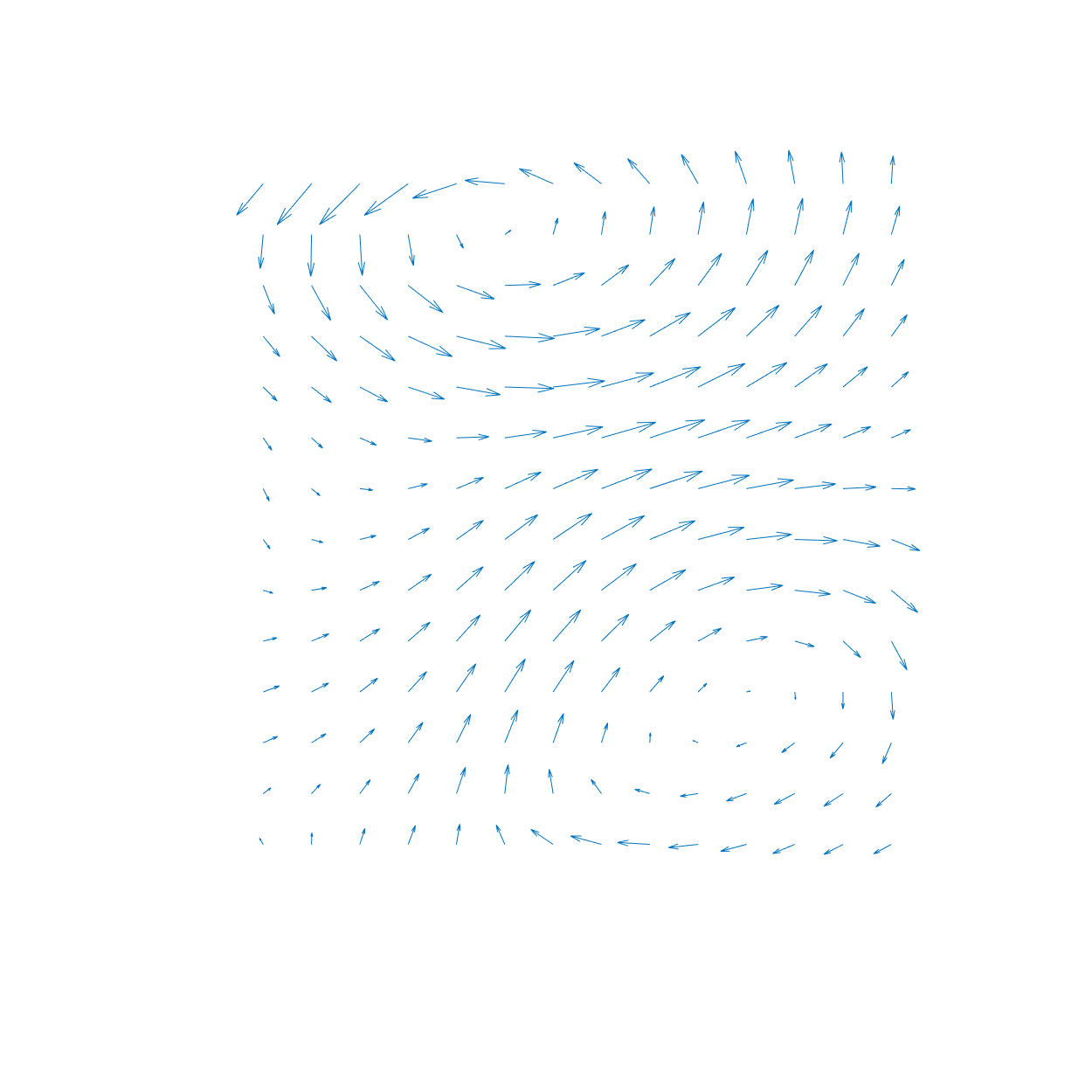

Supplement: S2 MCG raw data 2 — The raw MCG dataset includes categories 0-3 for training and validation. (ZIP) [file pone.0338189.s002.zip › train/0/p3_480_3.png]

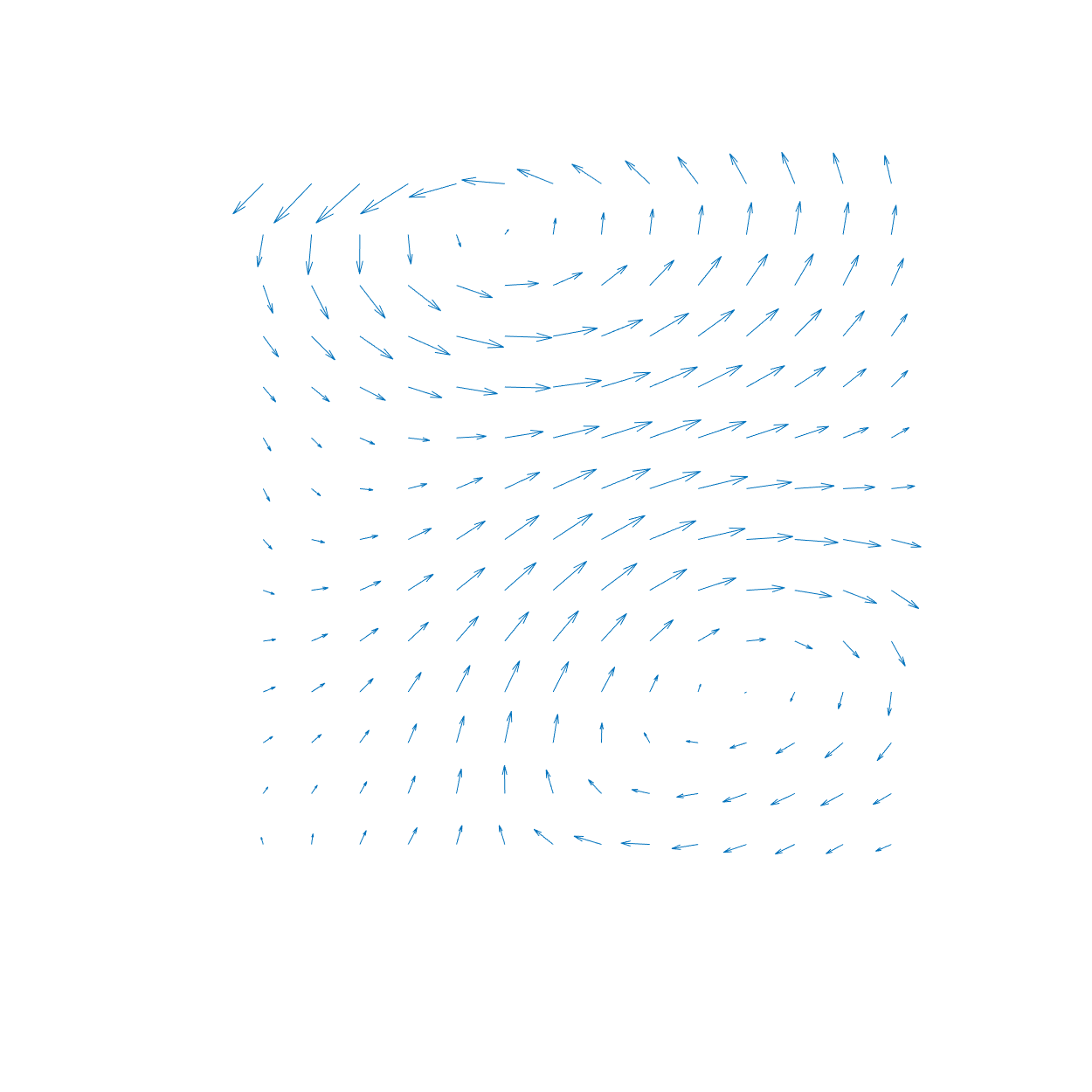

Supplement: S2 MCG raw data 2 — The raw MCG dataset includes categories 0-3 for training and validation. (ZIP) [file pone.0338189.s002.zip › train/0/p3_485_1.png]

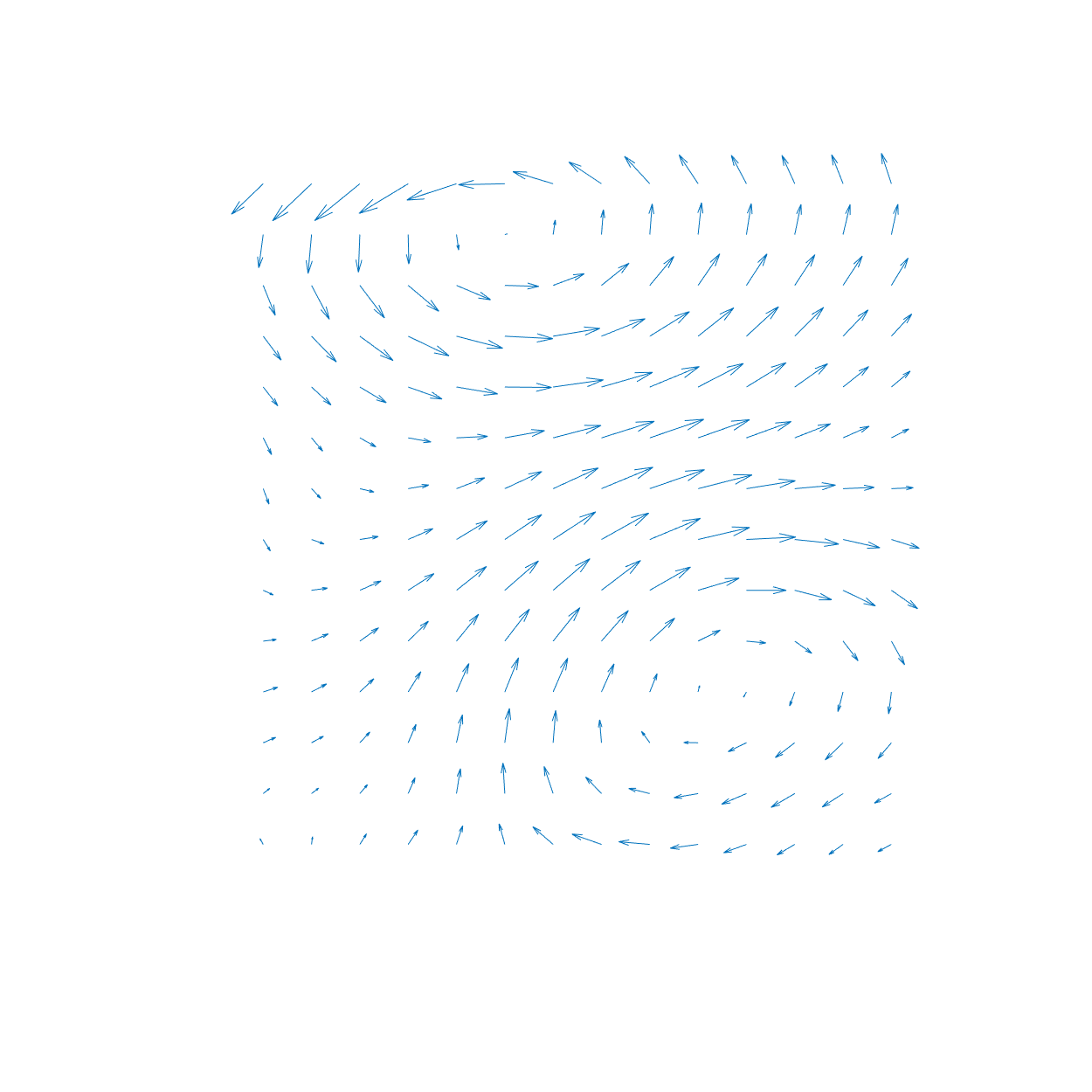

Supplement: S2 MCG raw data 2 — The raw MCG dataset includes categories 0-3 for training and validation. (ZIP) [file pone.0338189.s002.zip › train/0/p3_485_2.png]

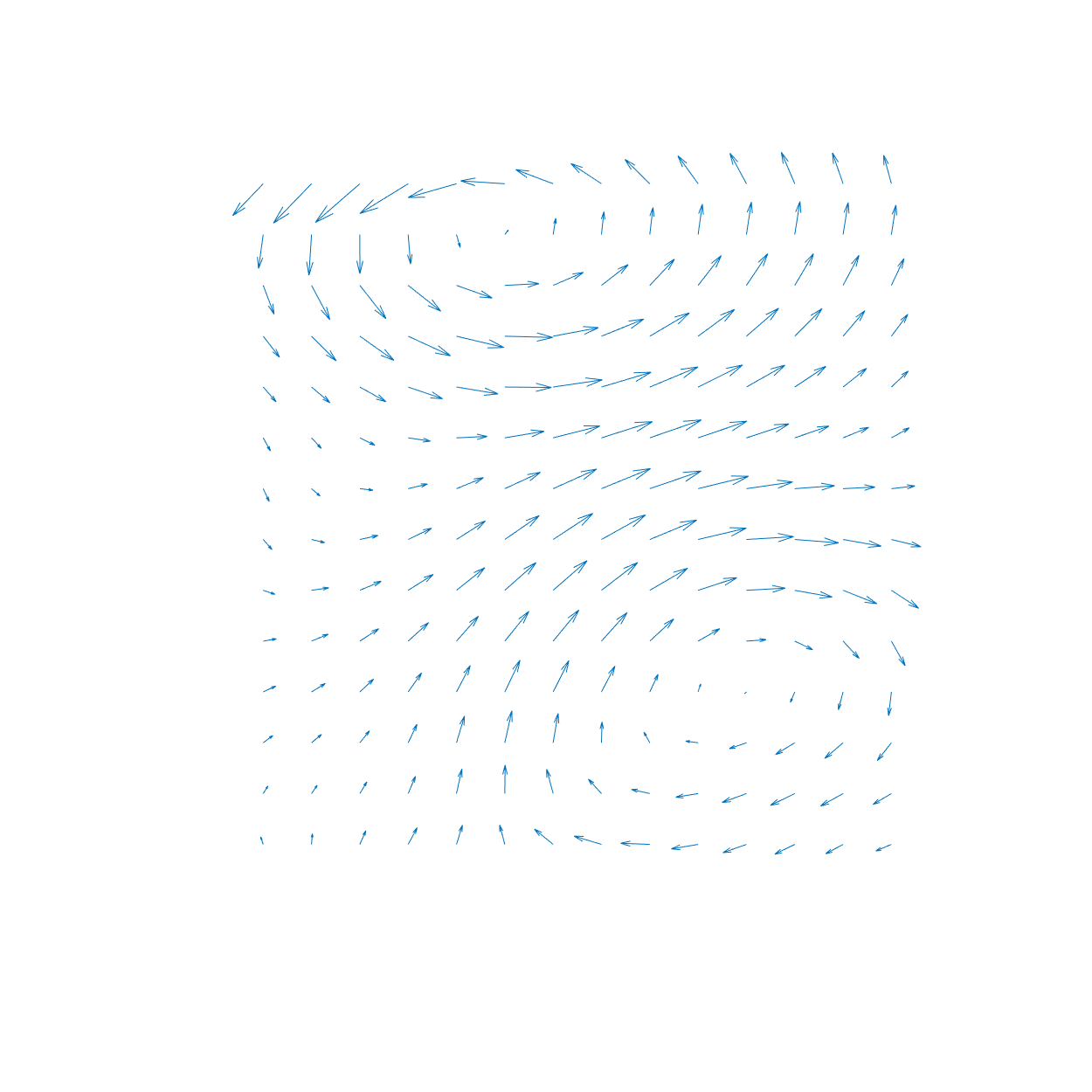

Supplement: S2 MCG raw data 2 — The raw MCG dataset includes categories 0-3 for training and validation. (ZIP) [file pone.0338189.s002.zip › train/0/p3_485_3.png]

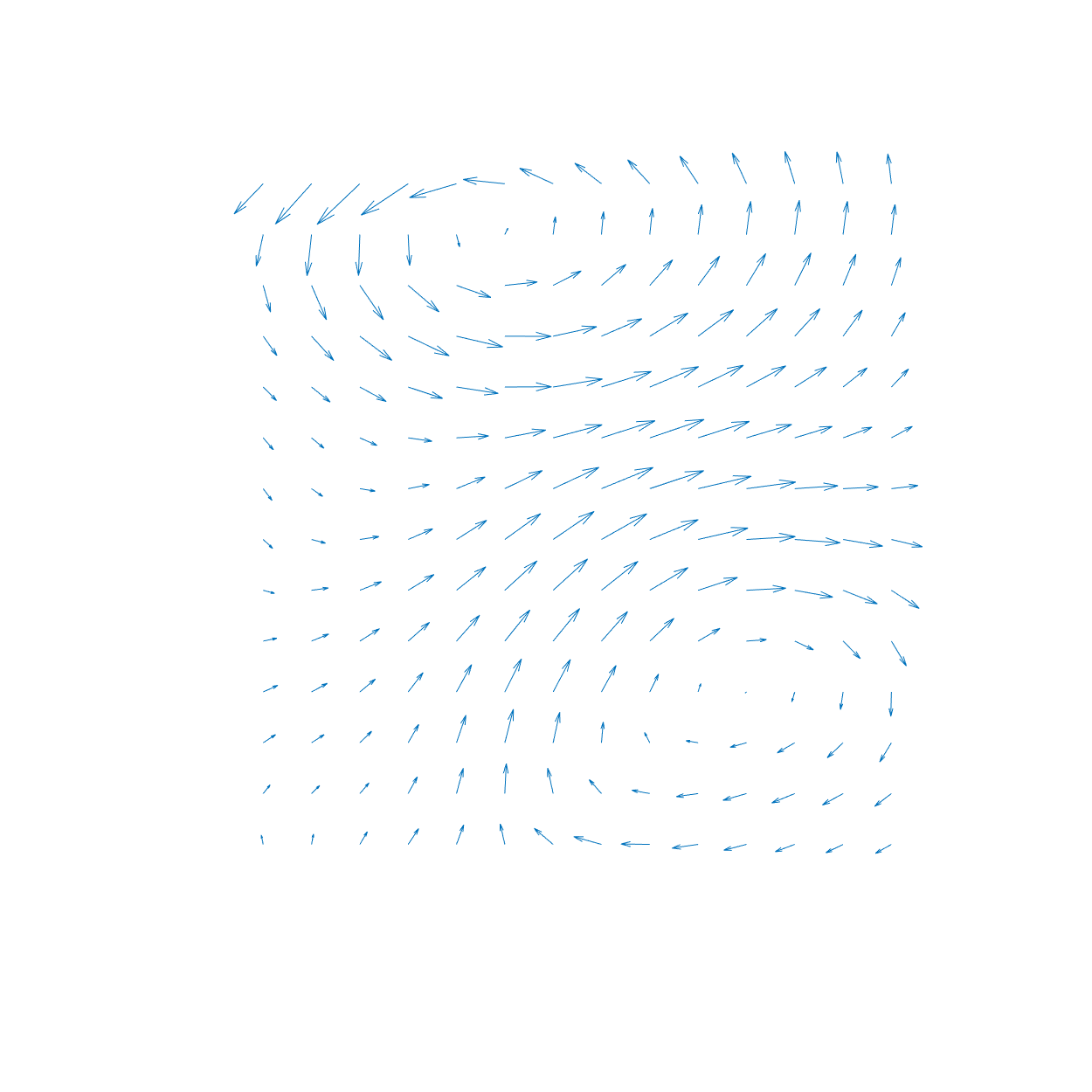

Supplement: S2 MCG raw data 2 — The raw MCG dataset includes categories 0-3 for training and validation. (ZIP) [file pone.0338189.s002.zip › train/0/p3_490_1.png]

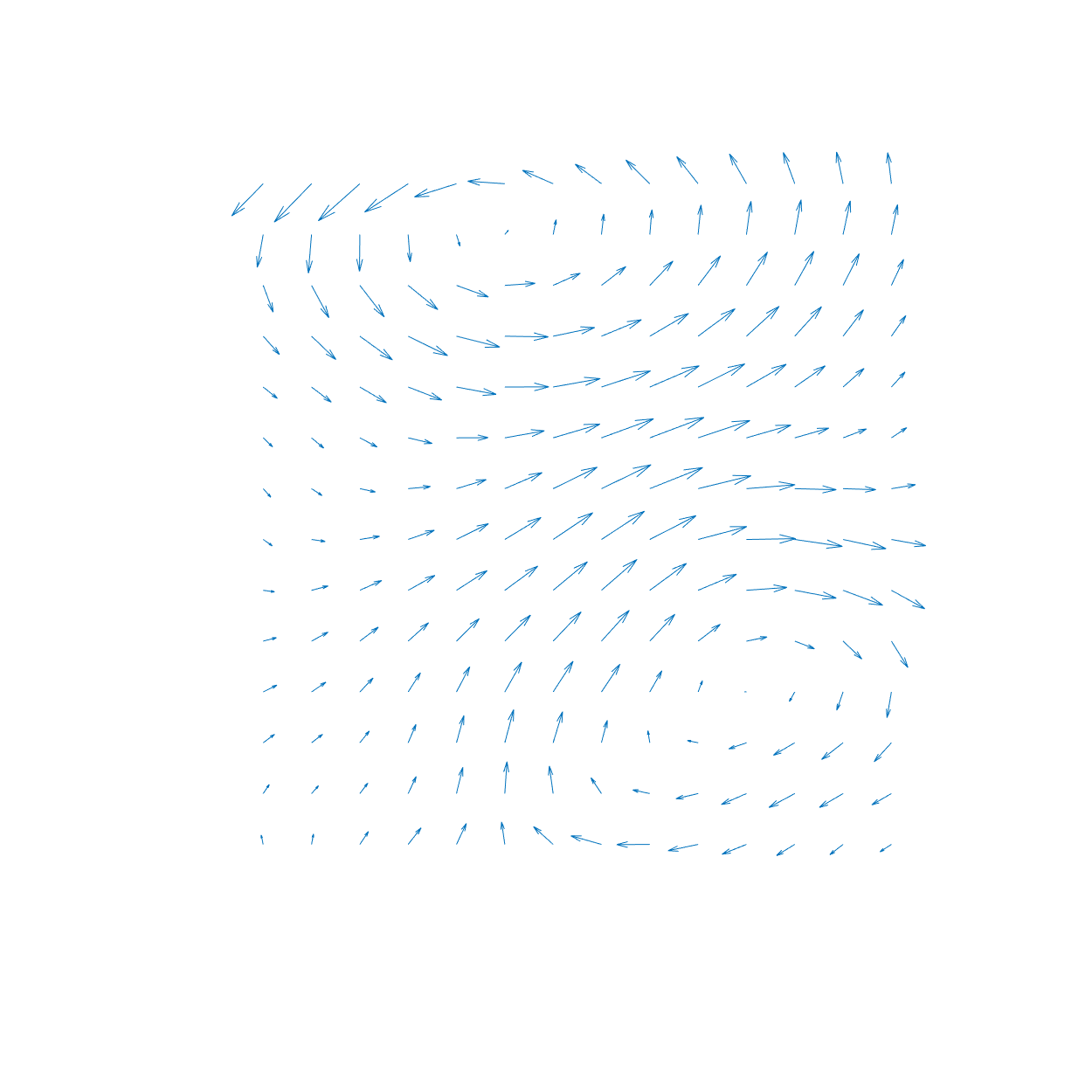

Supplement: S2 MCG raw data 2 — The raw MCG dataset includes categories 0-3 for training and validation. (ZIP) [file pone.0338189.s002.zip › train/0/p3_490_2.png]

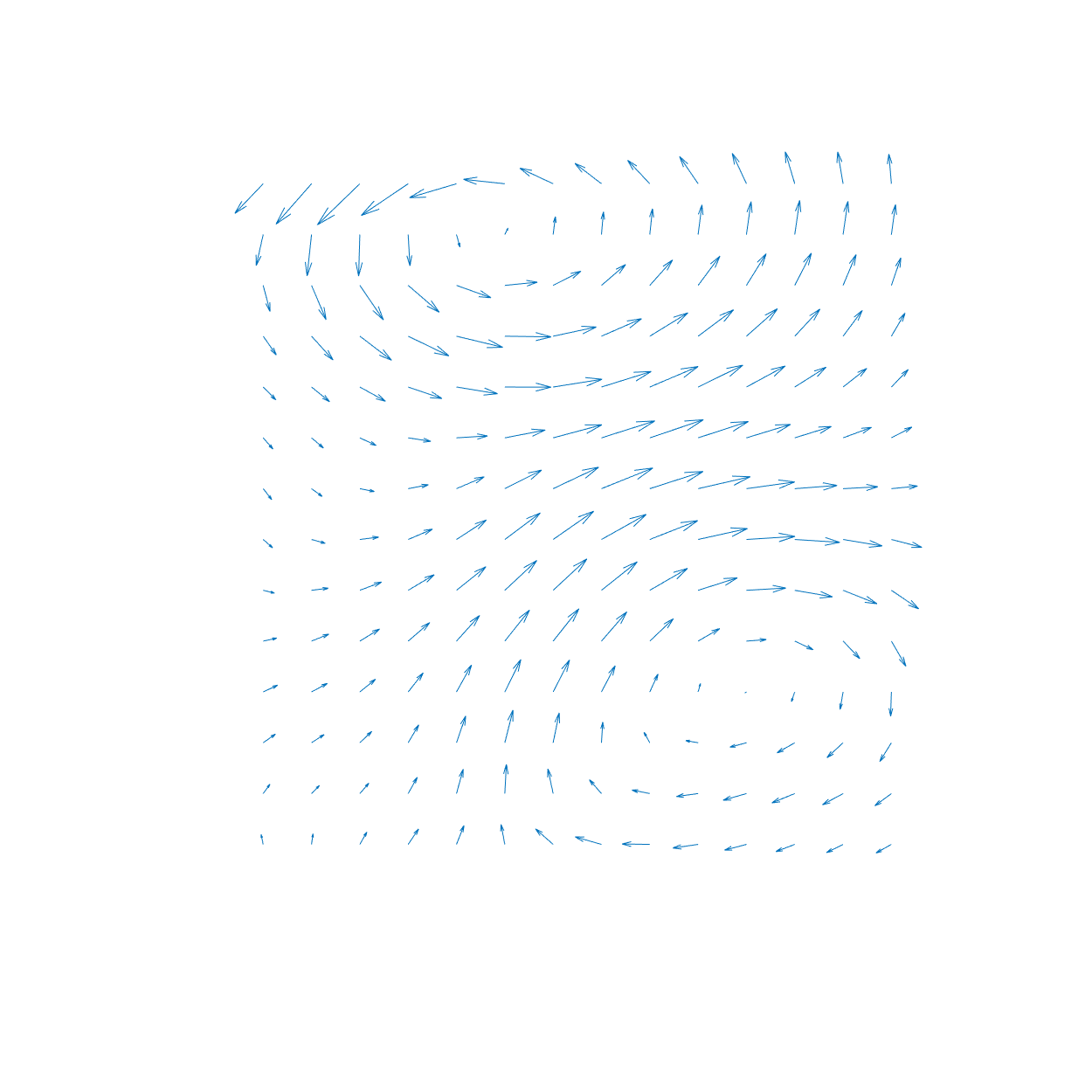

Supplement: S2 MCG raw data 2 — The raw MCG dataset includes categories 0-3 for training and validation. (ZIP) [file pone.0338189.s002.zip › train/0/p3_490_3.png]

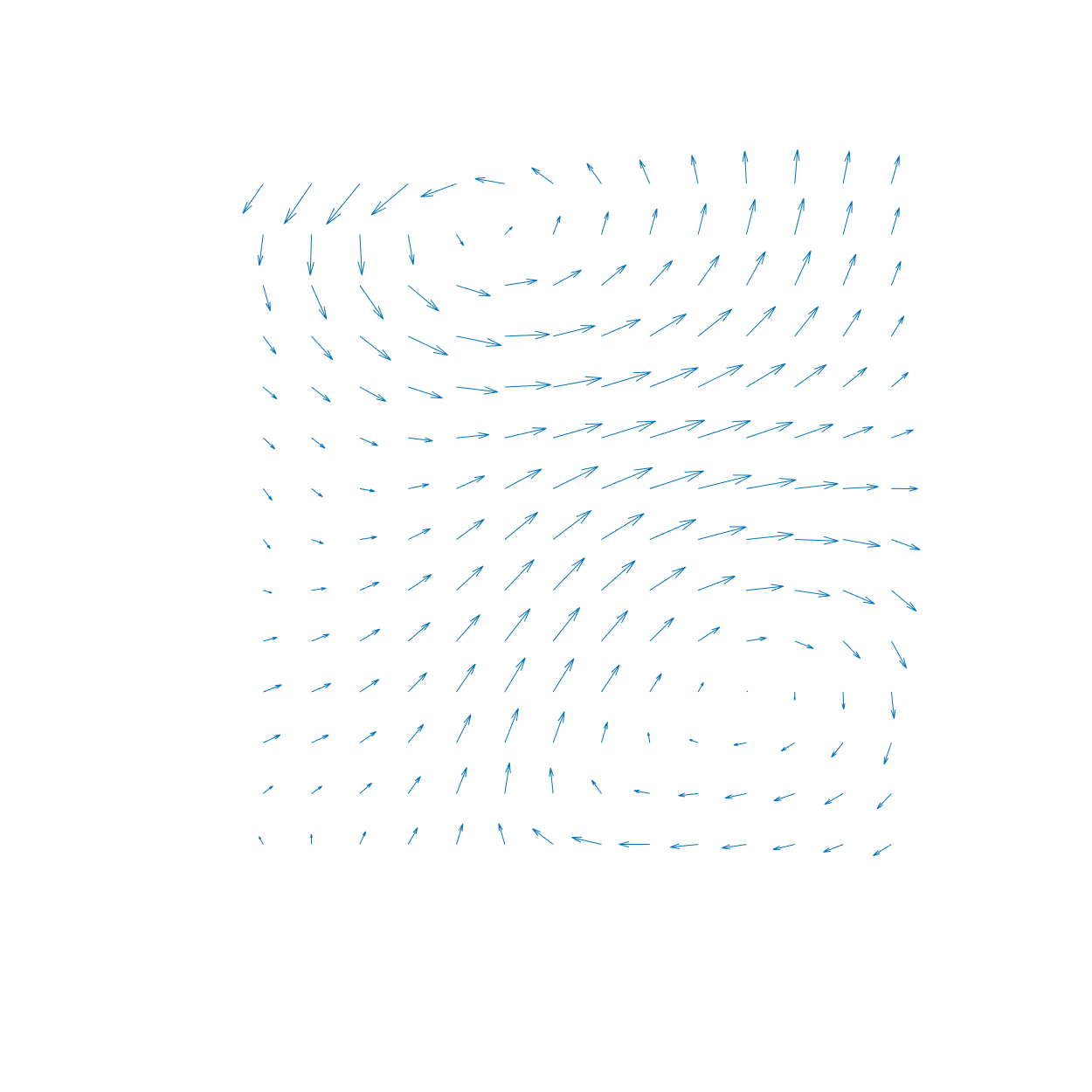

Supplement: S2 MCG raw data 2 — The raw MCG dataset includes categories 0-3 for training and validation. (ZIP) [file pone.0338189.s002.zip › train/0/p3_495_1.png]

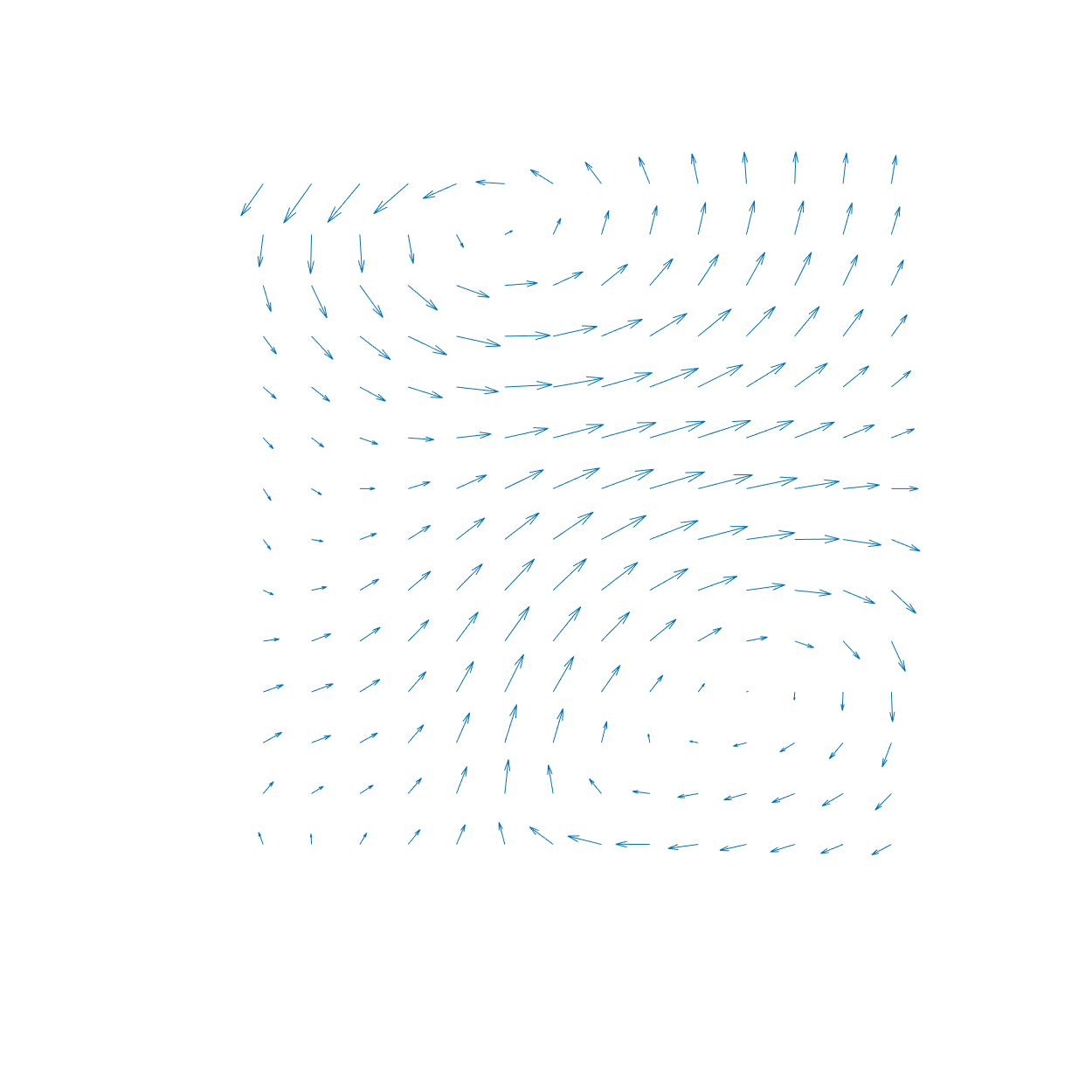

Supplement: S2 MCG raw data 2 — The raw MCG dataset includes categories 0-3 for training and validation. (ZIP) [file pone.0338189.s002.zip › train/0/p3_495_2.png]

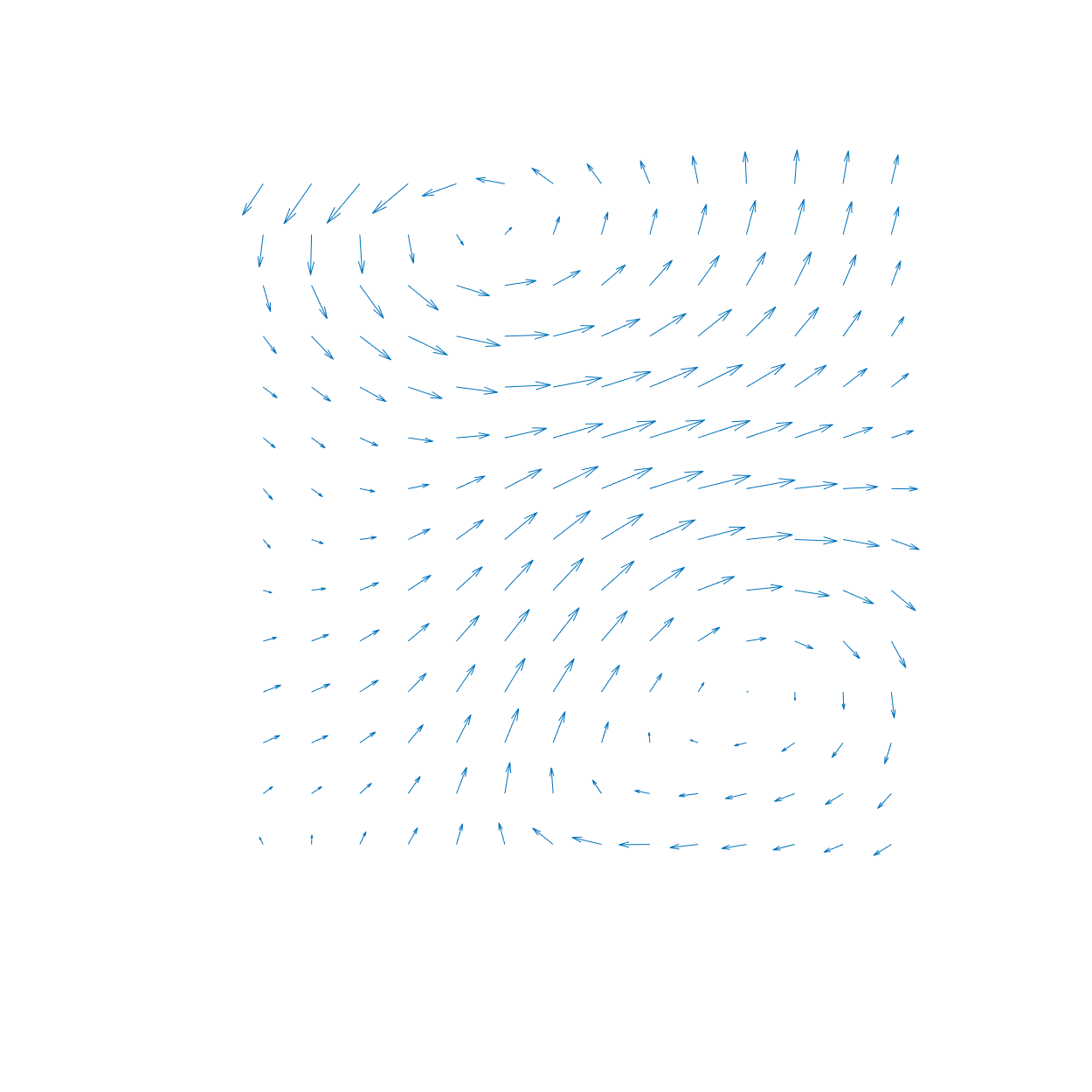

Supplement: S2 MCG raw data 2 — The raw MCG dataset includes categories 0-3 for training and validation. (ZIP) [file pone.0338189.s002.zip › train/0/p3_495_3.png]

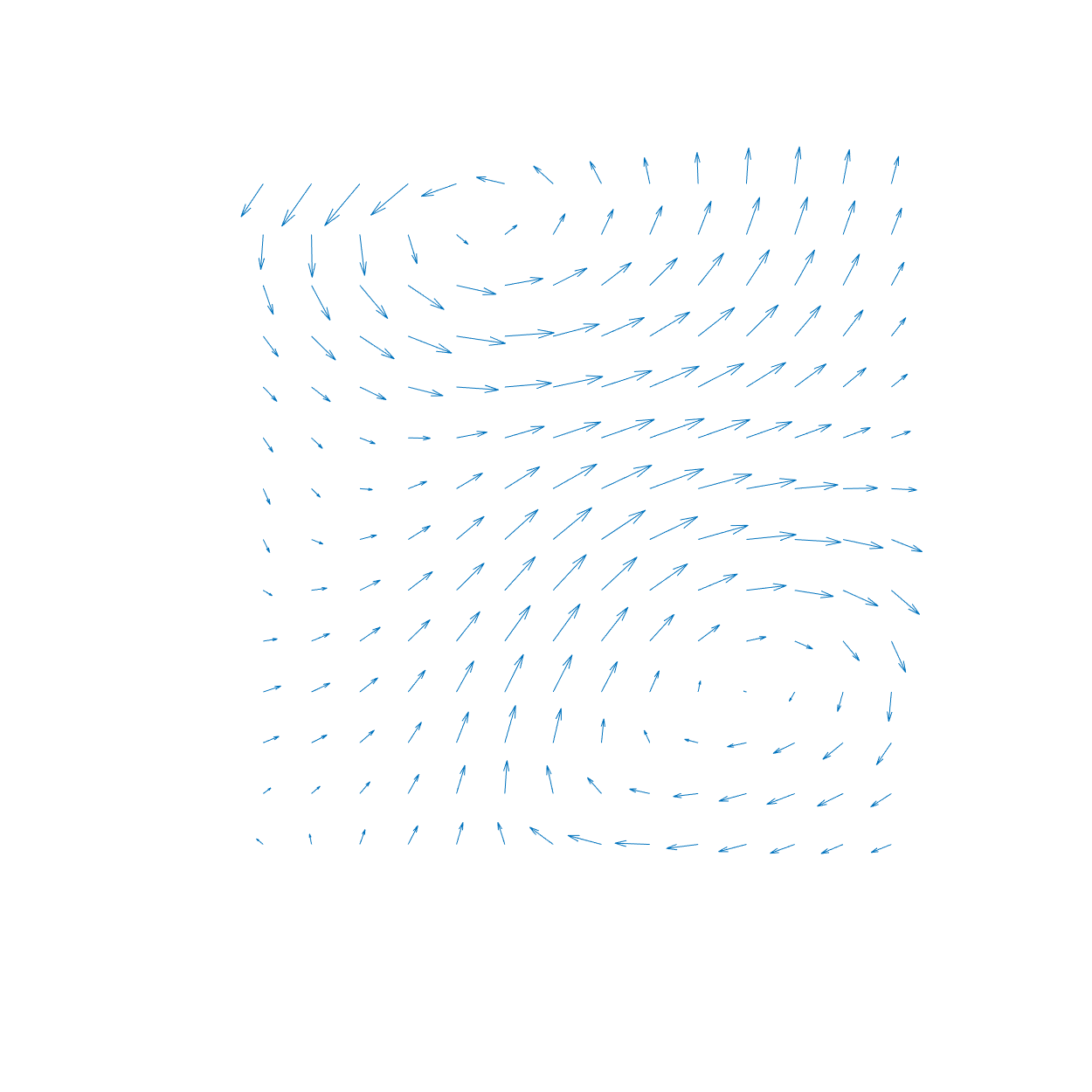

Supplement: S2 MCG raw data 2 — The raw MCG dataset includes categories 0-3 for training and validation. (ZIP) [file pone.0338189.s002.zip › train/0/p3_500_1.png]

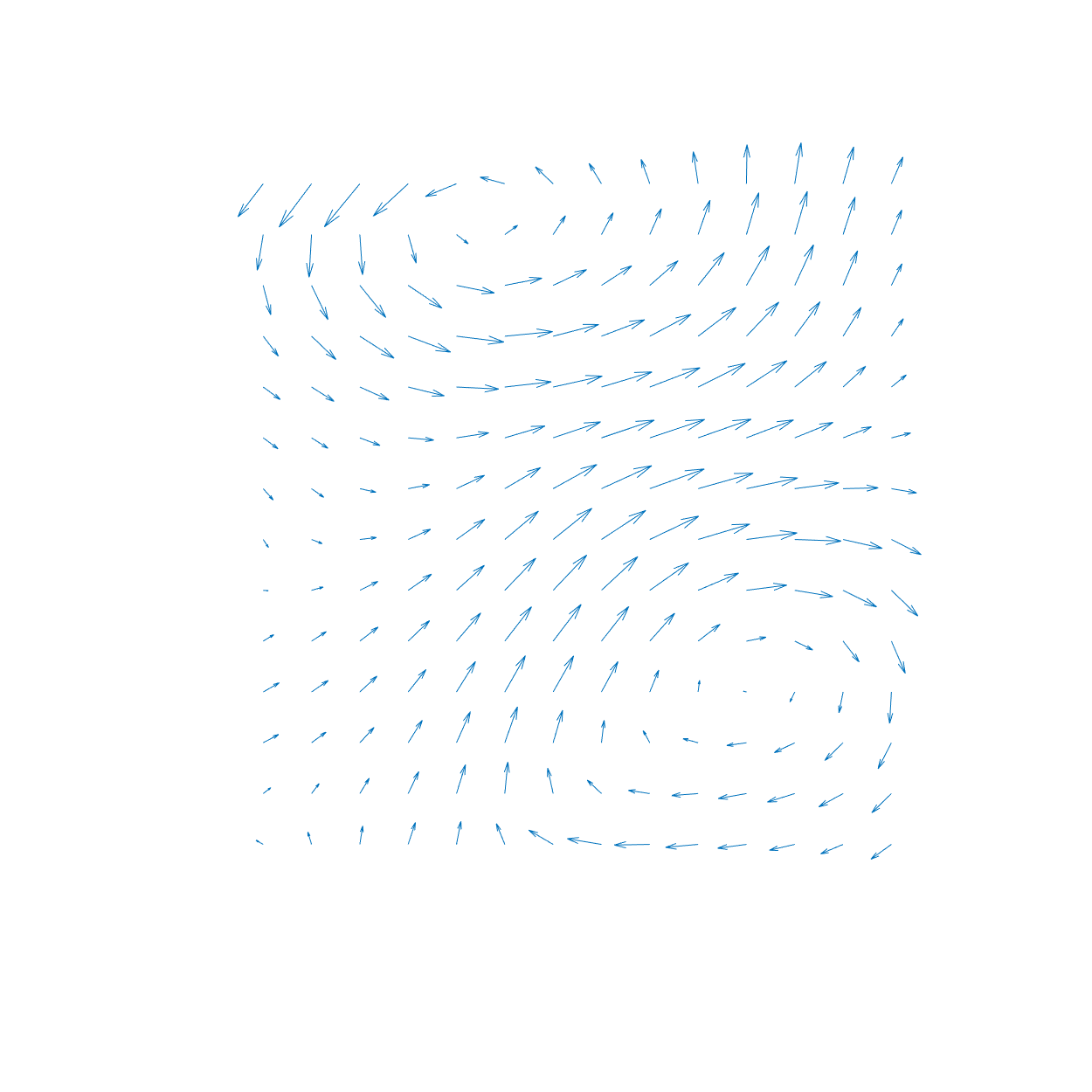

Supplement: S2 MCG raw data 2 — The raw MCG dataset includes categories 0-3 for training and validation. (ZIP) [file pone.0338189.s002.zip › train/0/p3_500_2.png]

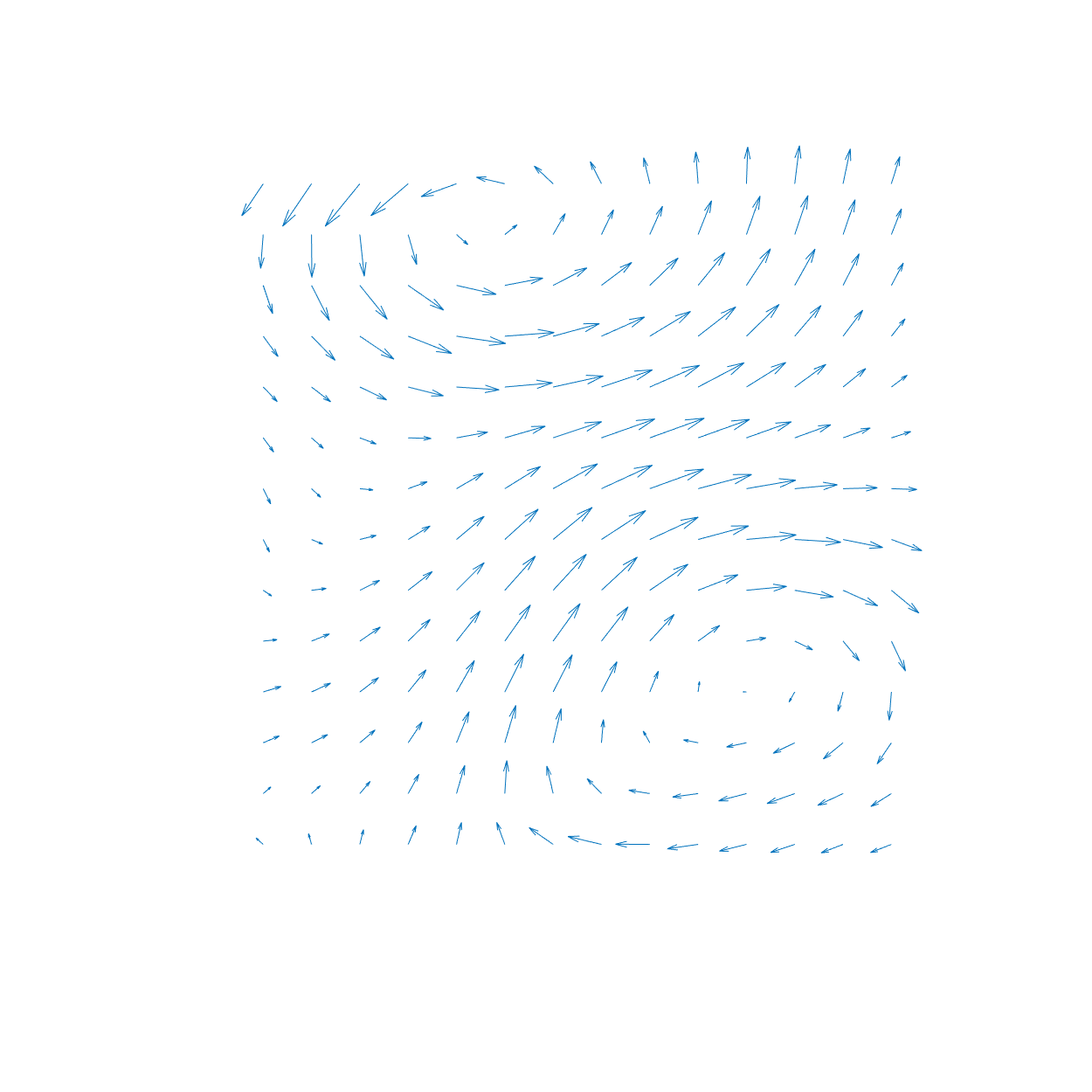

Supplement: S2 MCG raw data 2 — The raw MCG dataset includes categories 0-3 for training and validation. (ZIP) [file pone.0338189.s002.zip › train/0/p3_500_3.png]

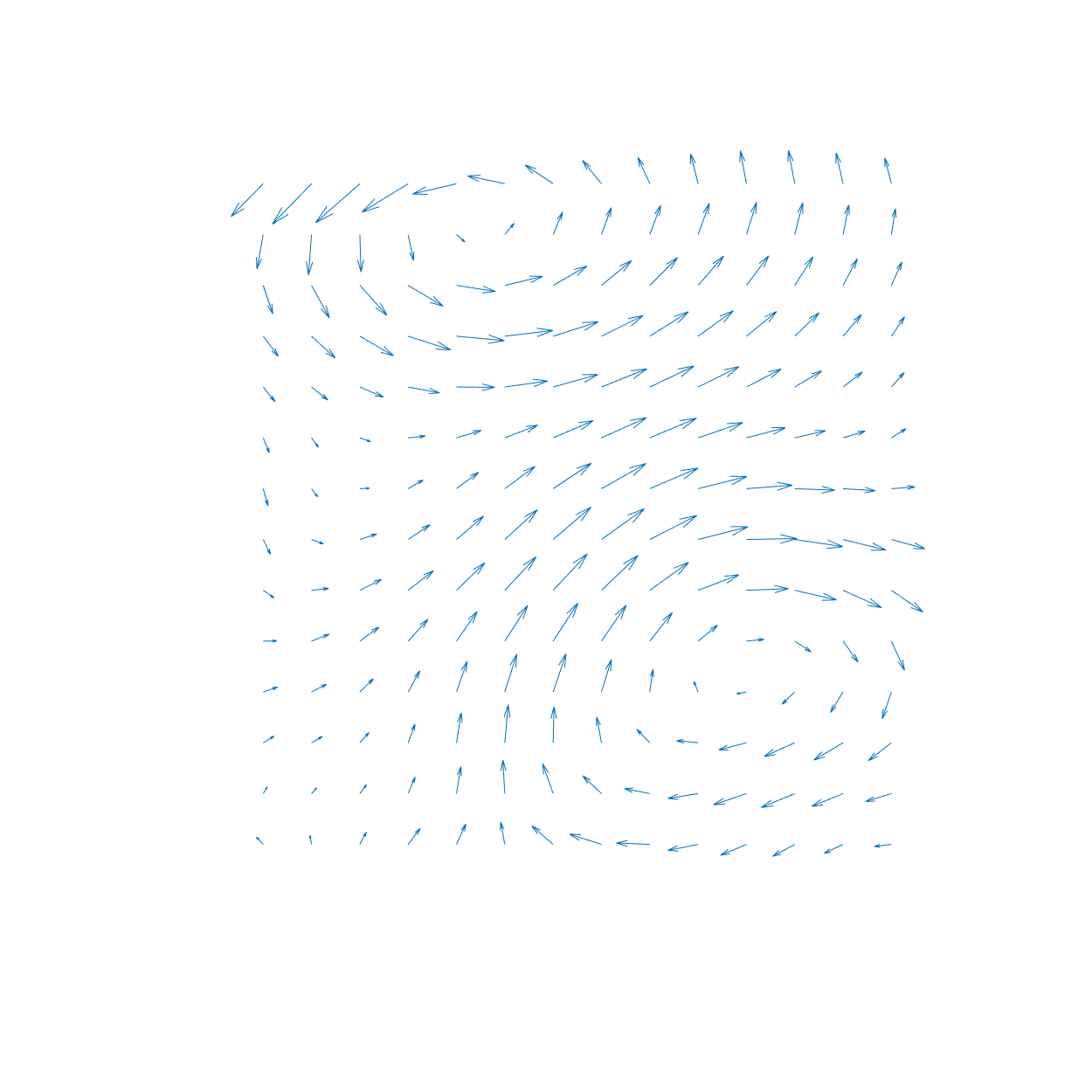

Supplement: S2 MCG raw data 2 — The raw MCG dataset includes categories 0-3 for training and validation. (ZIP) [file pone.0338189.s002.zip › train/0/p3_505_1.png]

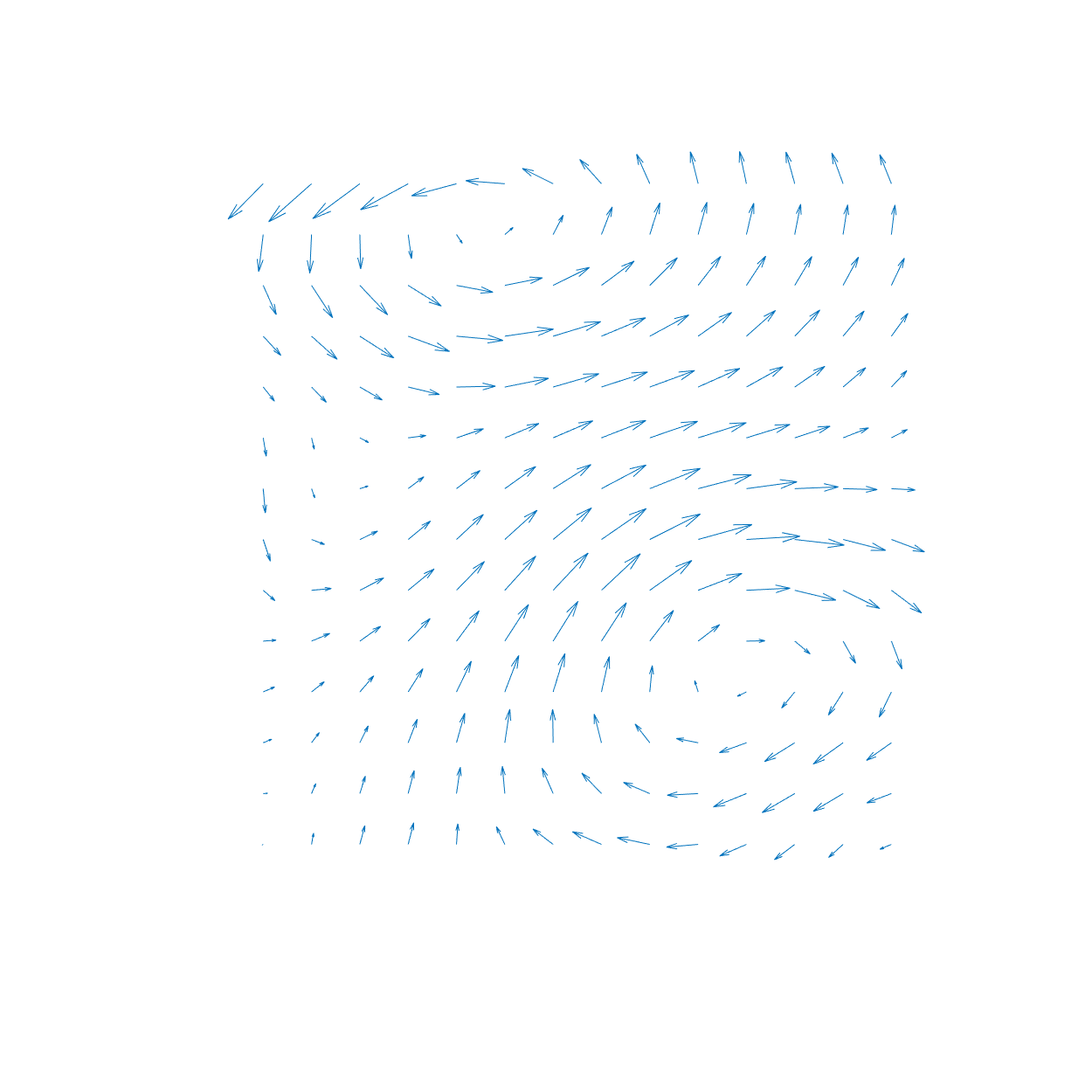

Supplement: S2 MCG raw data 2 — The raw MCG dataset includes categories 0-3 for training and validation. (ZIP) [file pone.0338189.s002.zip › train/0/p3_505_2.png]

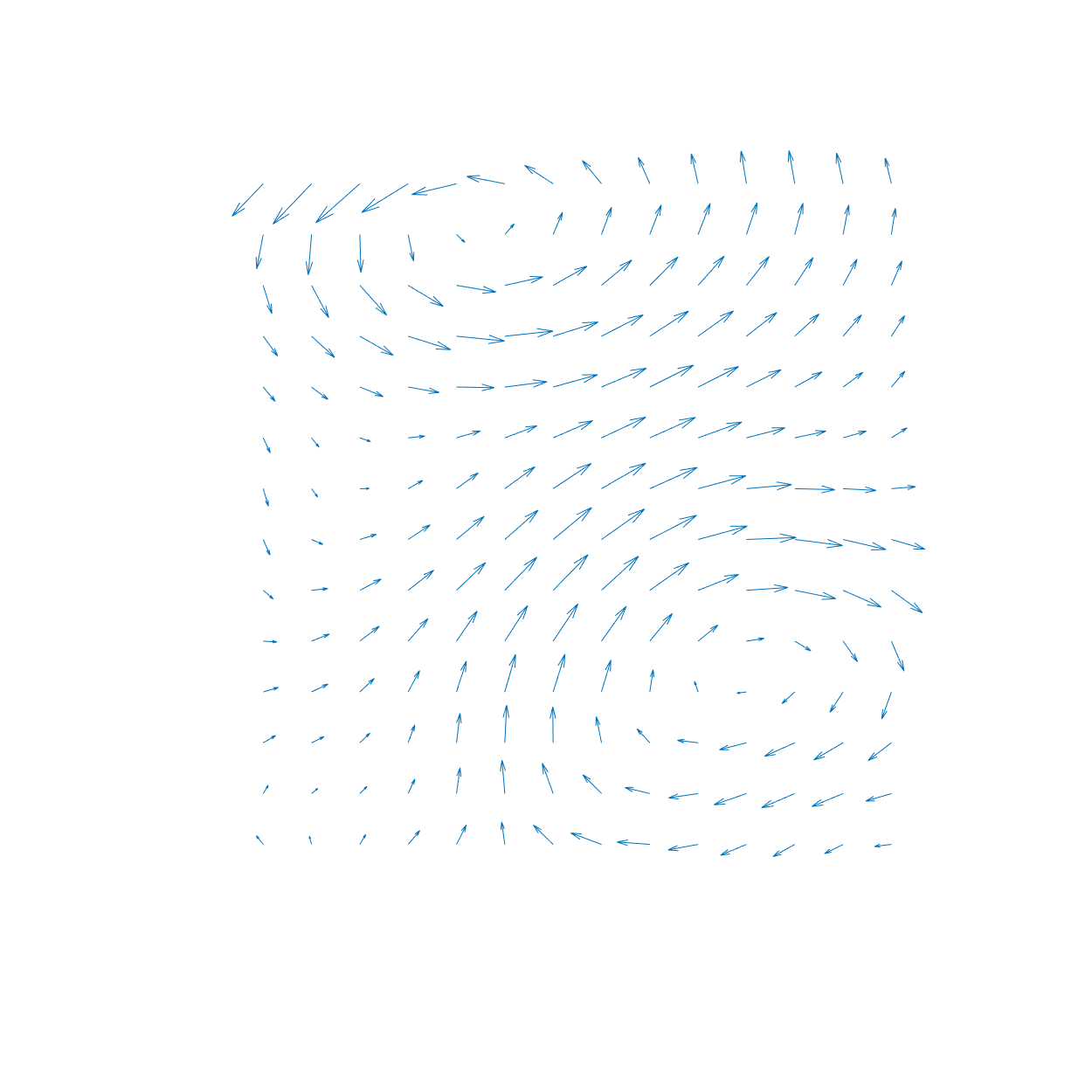

Supplement: S2 MCG raw data 2 — The raw MCG dataset includes categories 0-3 for training and validation. (ZIP) [file pone.0338189.s002.zip › train/0/p3_505_3.png]

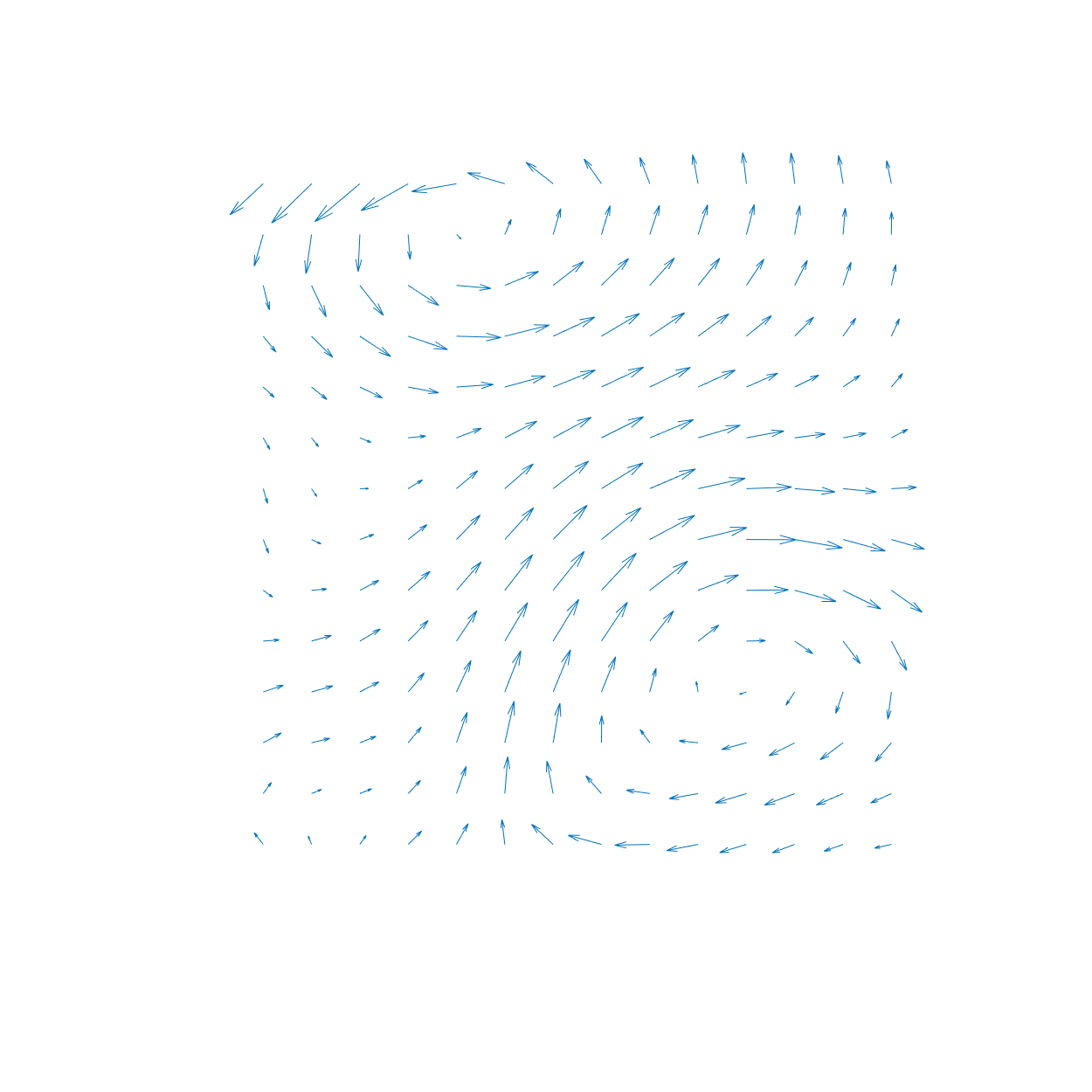

Supplement: S2 MCG raw data 2 — The raw MCG dataset includes categories 0-3 for training and validation. (ZIP) [file pone.0338189.s002.zip › train/0/p3_510_1.png]

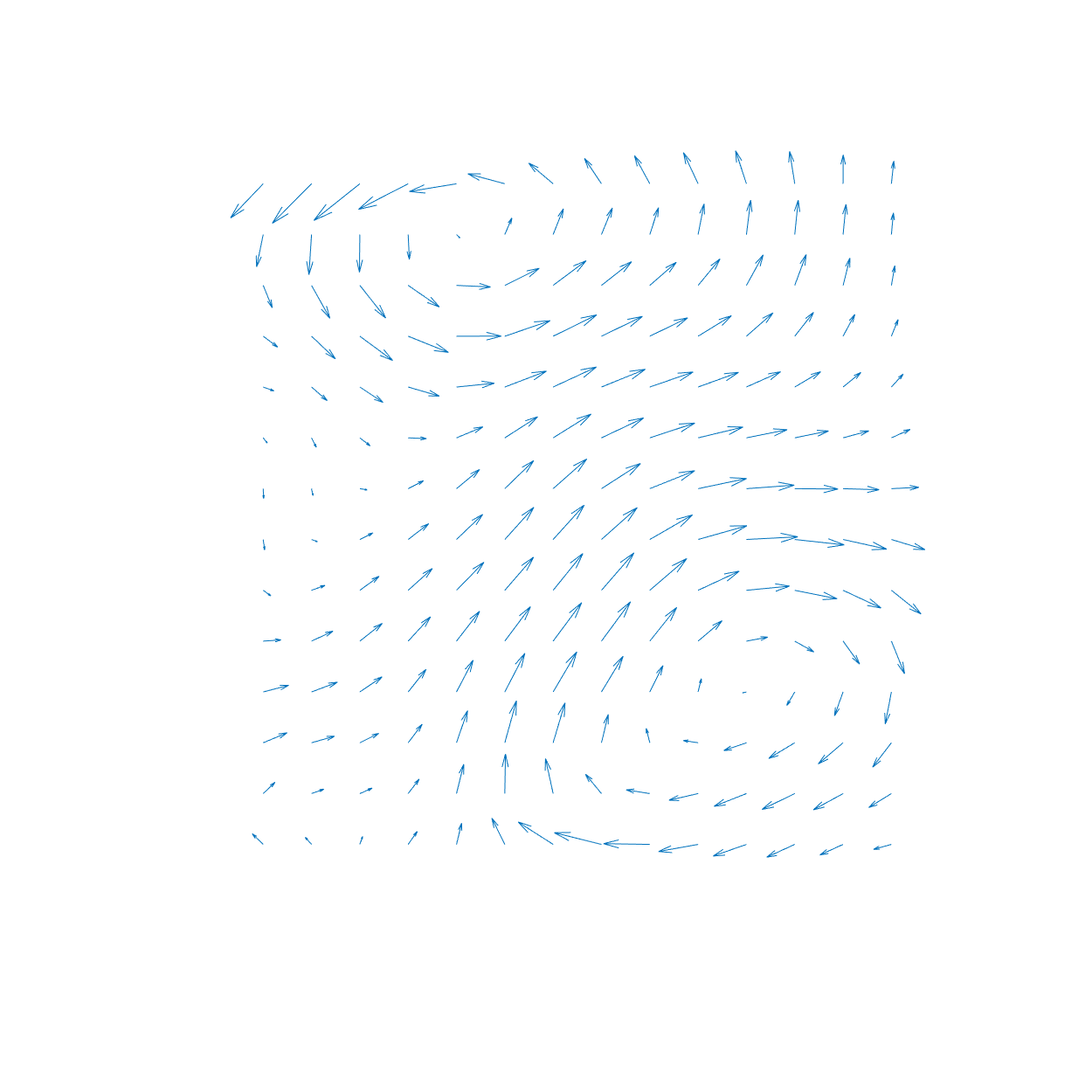

Supplement: S2 MCG raw data 2 — The raw MCG dataset includes categories 0-3 for training and validation. (ZIP) [file pone.0338189.s002.zip › train/0/p3_510_2.png]

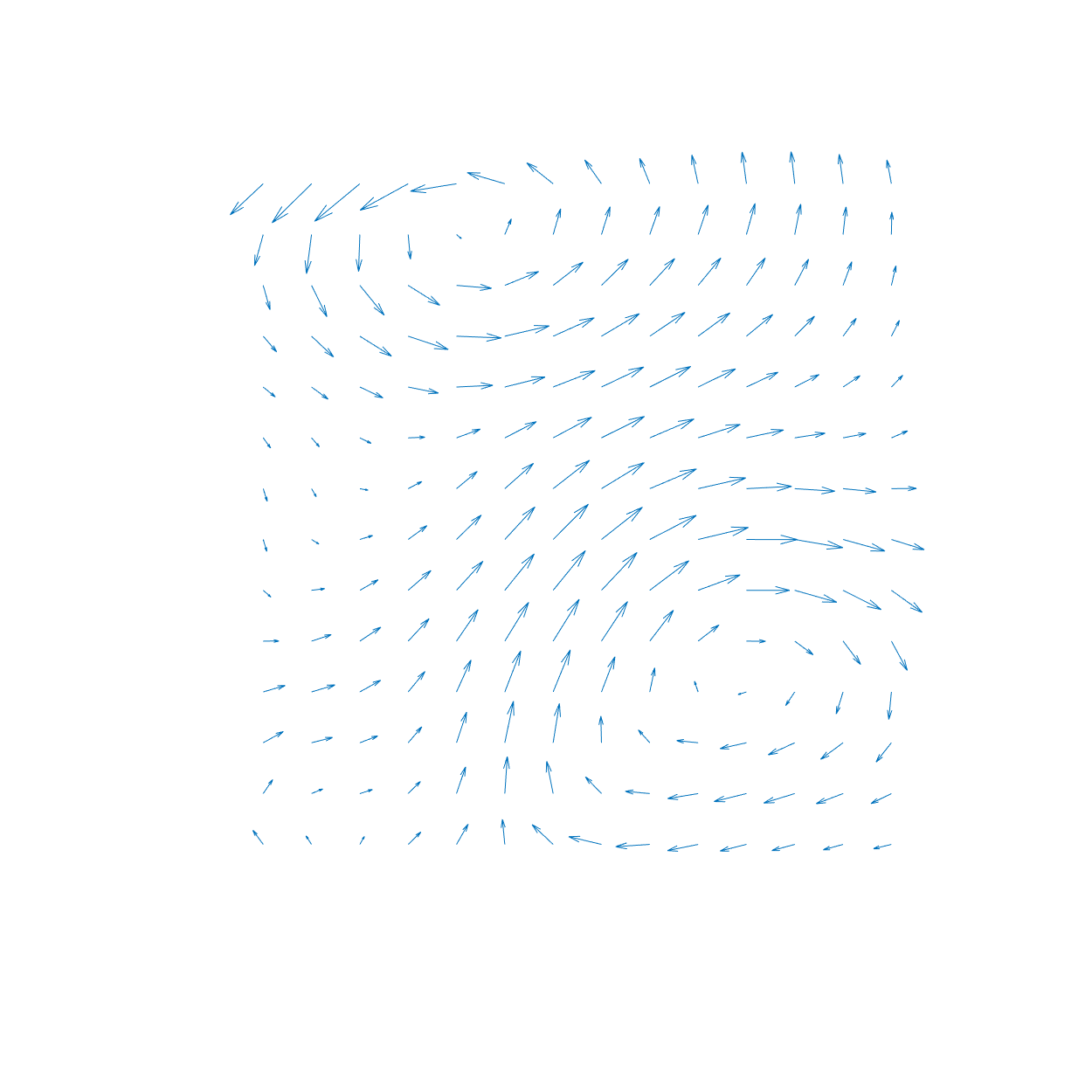

Supplement: S2 MCG raw data 2 — The raw MCG dataset includes categories 0-3 for training and validation. (ZIP) [file pone.0338189.s002.zip › train/0/p3_510_3.png]

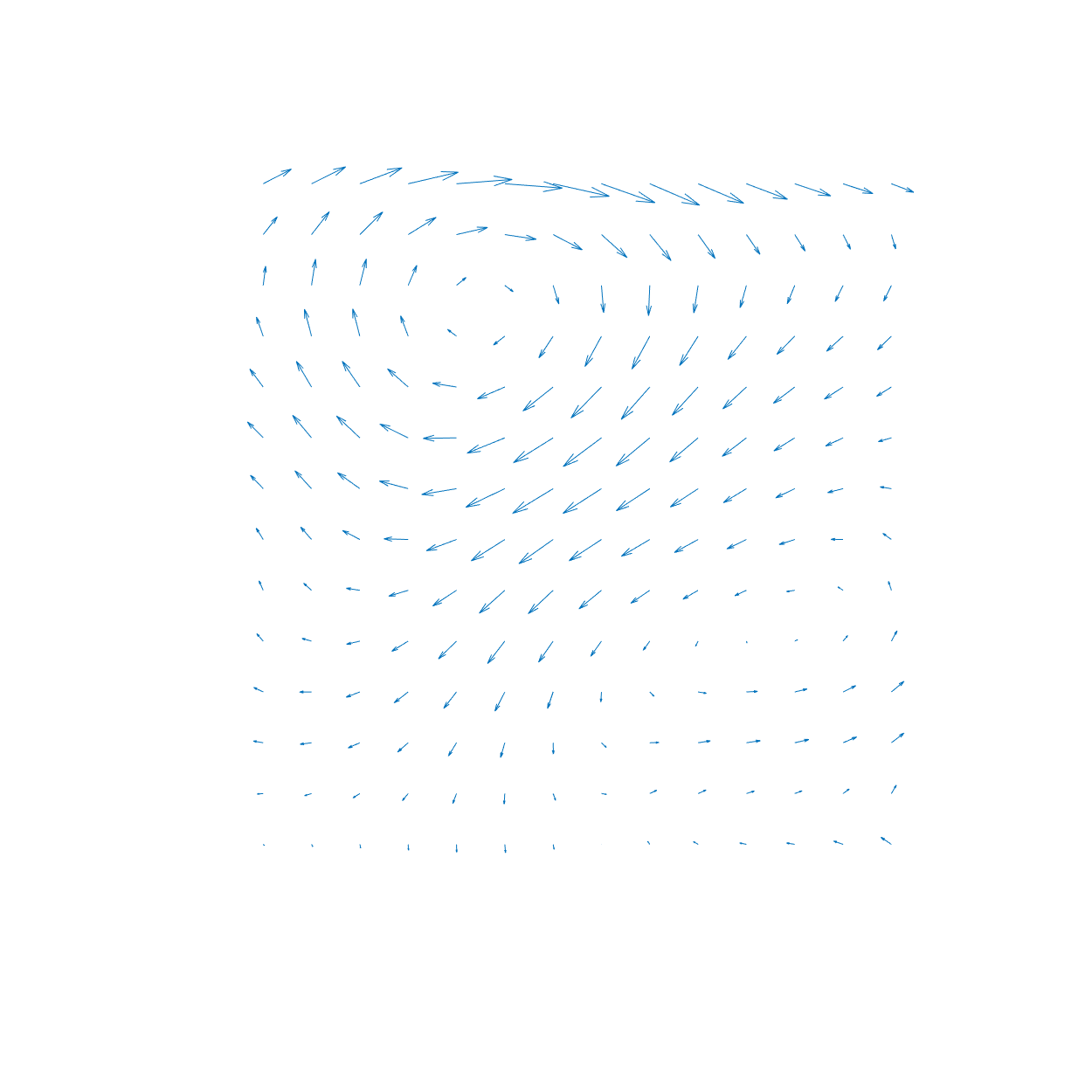

Supplement: S2 MCG raw data 2 — The raw MCG dataset includes categories 0-3 for training and validation. (ZIP) [file pone.0338189.s002.zip › train/0/p4_200_1.png]

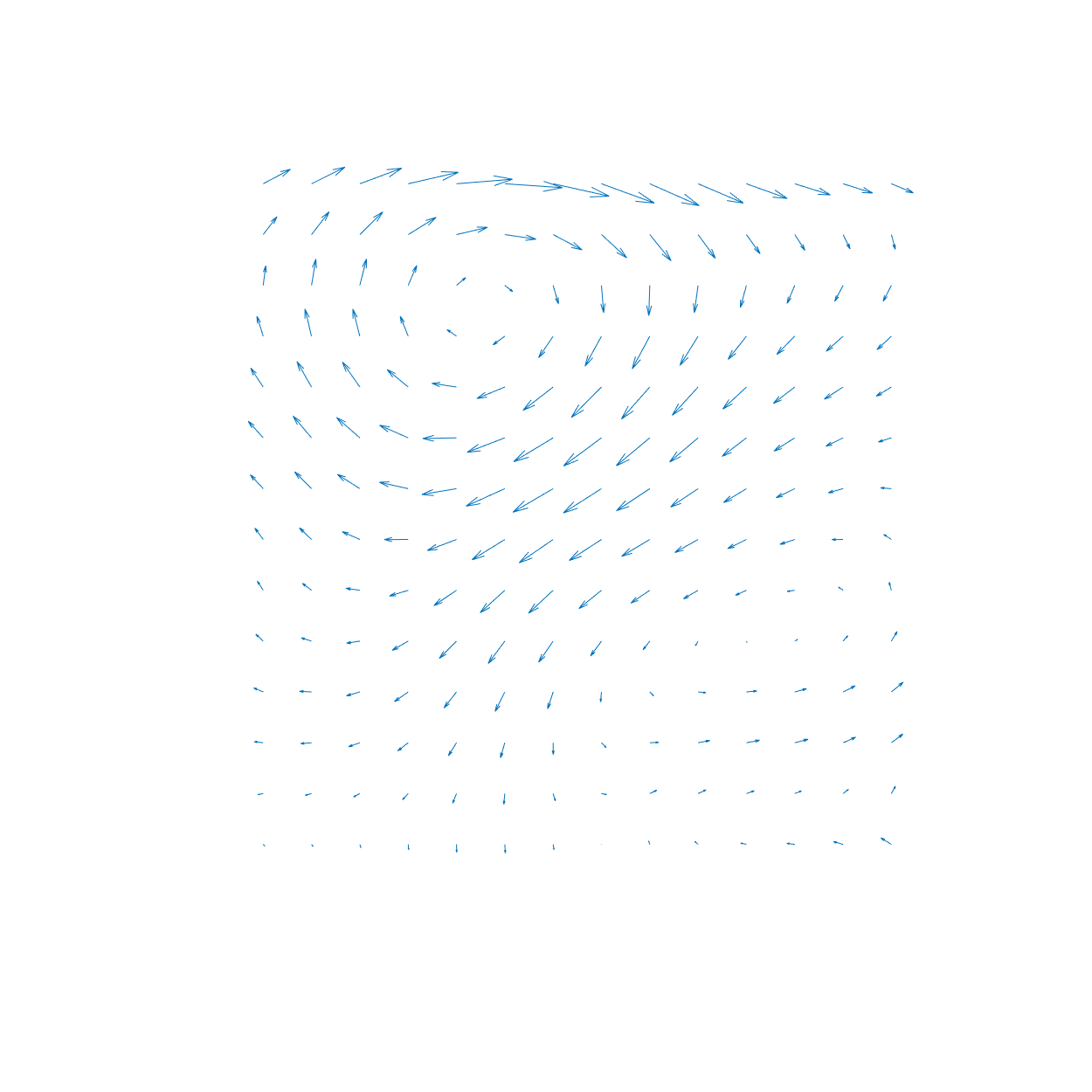

Supplement: S2 MCG raw data 2 — The raw MCG dataset includes categories 0-3 for training and validation. (ZIP) [file pone.0338189.s002.zip › train/0/p4_200_2.png]

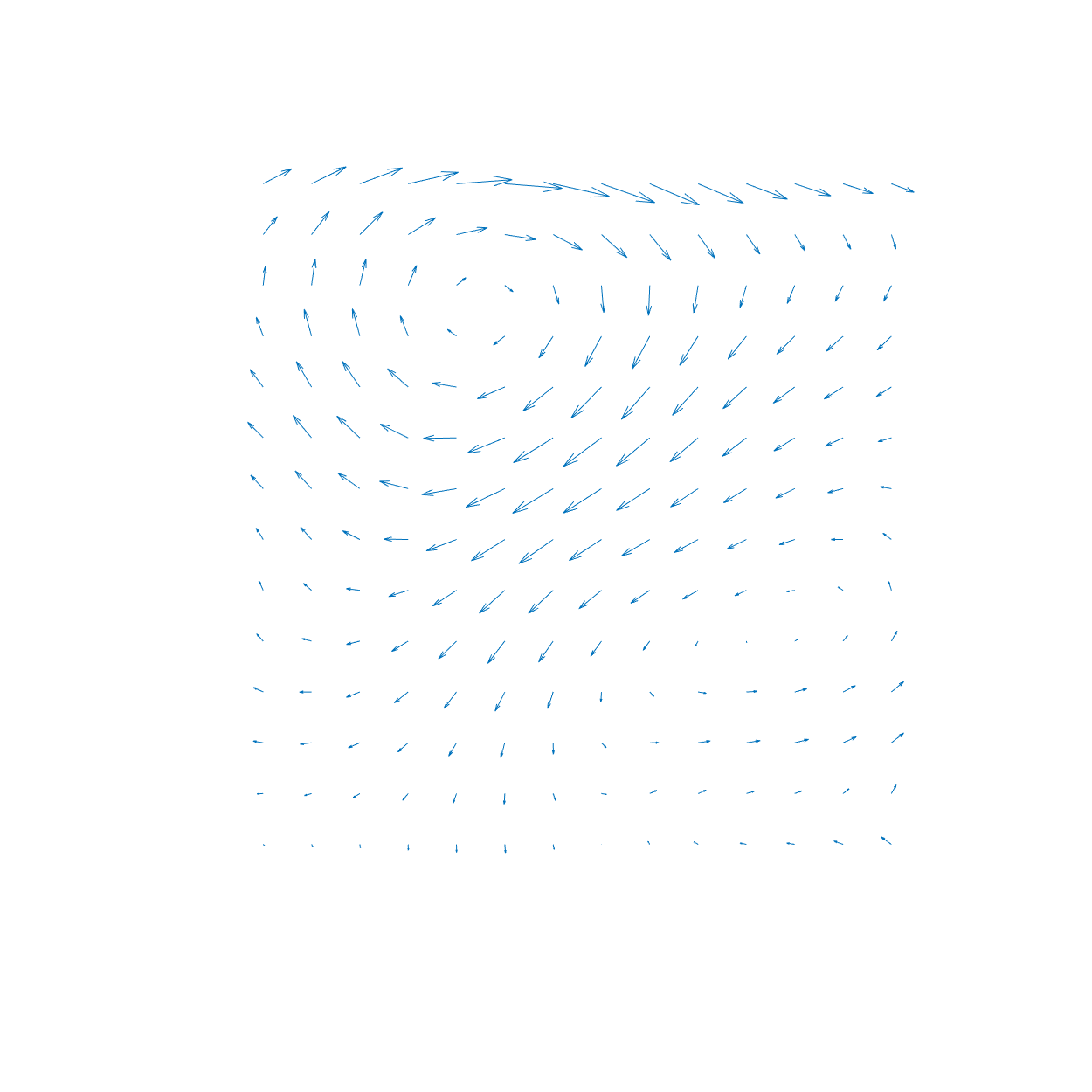

Supplement: S2 MCG raw data 2 — The raw MCG dataset includes categories 0-3 for training and validation. (ZIP) [file pone.0338189.s002.zip › train/0/p4_200_3.png]

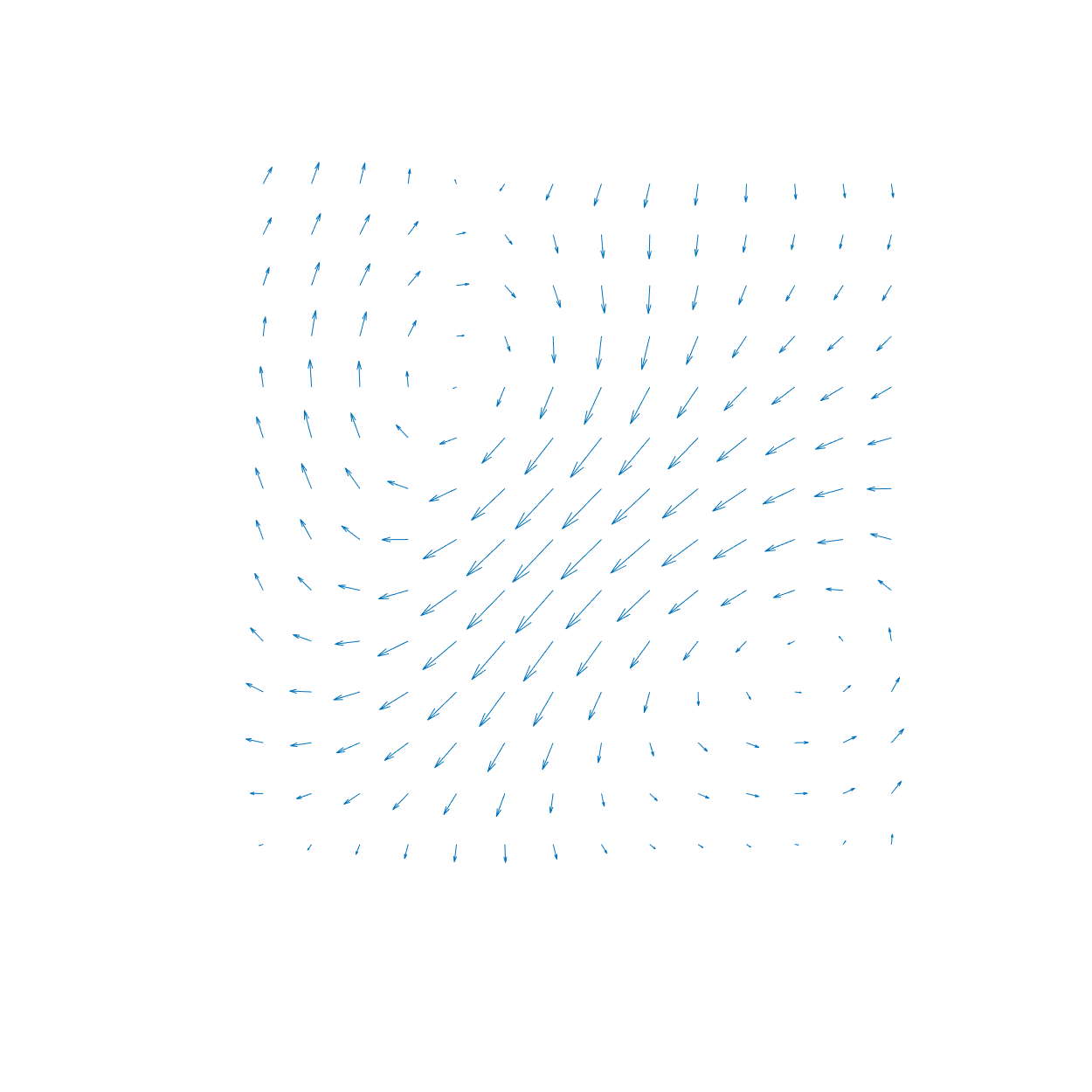

Supplement: S2 MCG raw data 2 — The raw MCG dataset includes categories 0-3 for training and validation. (ZIP) [file pone.0338189.s002.zip › train/0/p4_205_1.png]

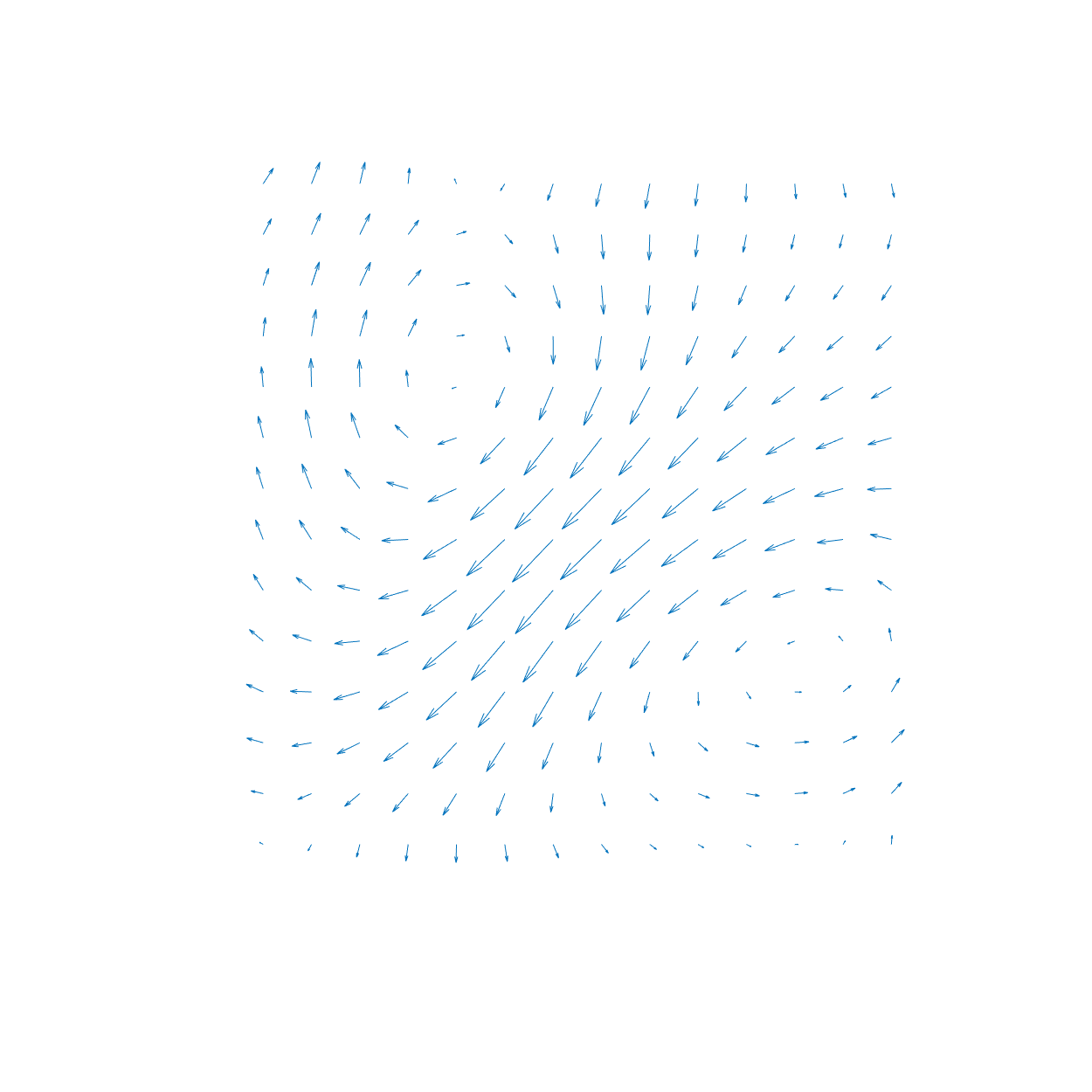

Supplement: S2 MCG raw data 2 — The raw MCG dataset includes categories 0-3 for training and validation. (ZIP) [file pone.0338189.s002.zip › train/0/p4_205_2.png]

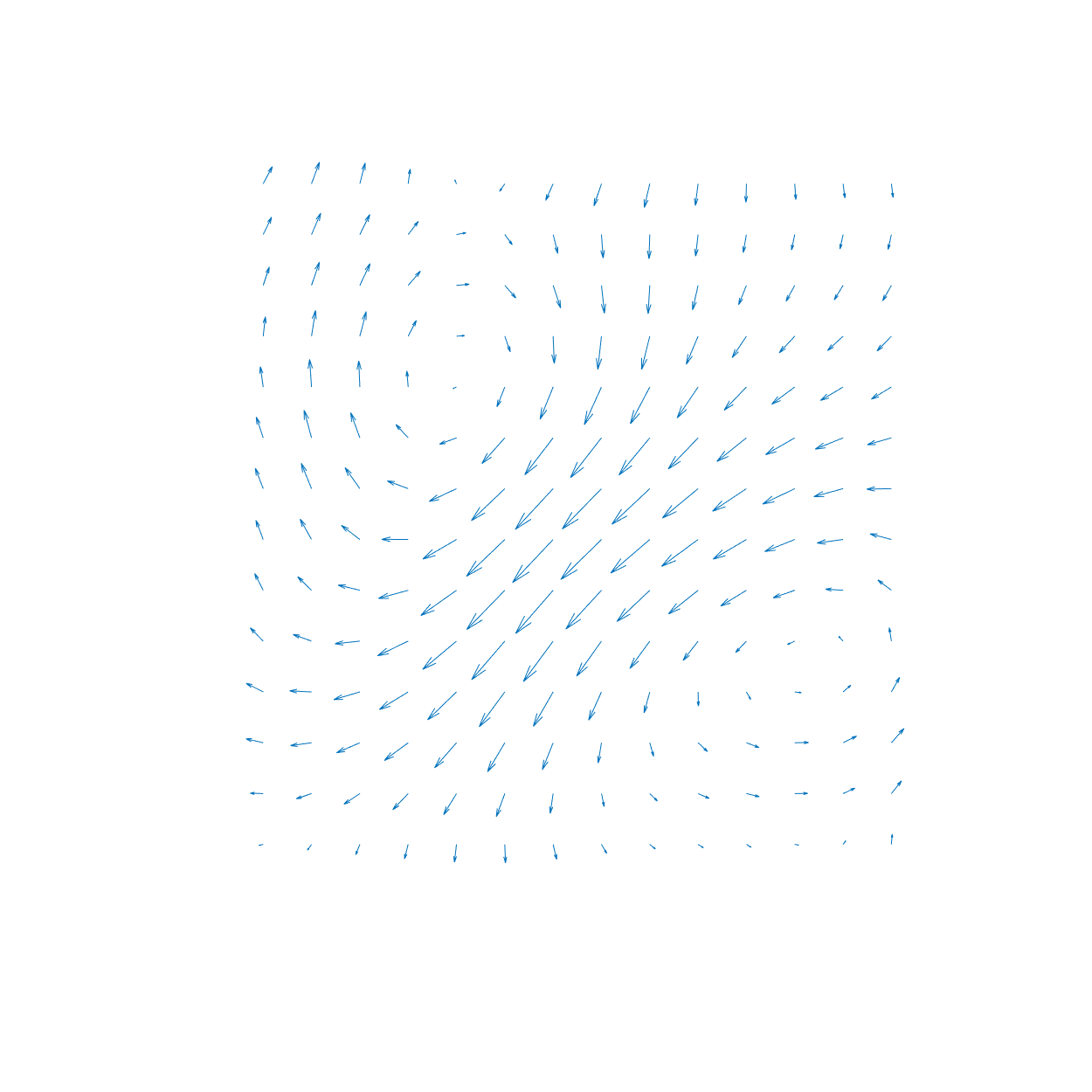

Supplement: S2 MCG raw data 2 — The raw MCG dataset includes categories 0-3 for training and validation. (ZIP) [file pone.0338189.s002.zip › train/0/p4_205_3.png]

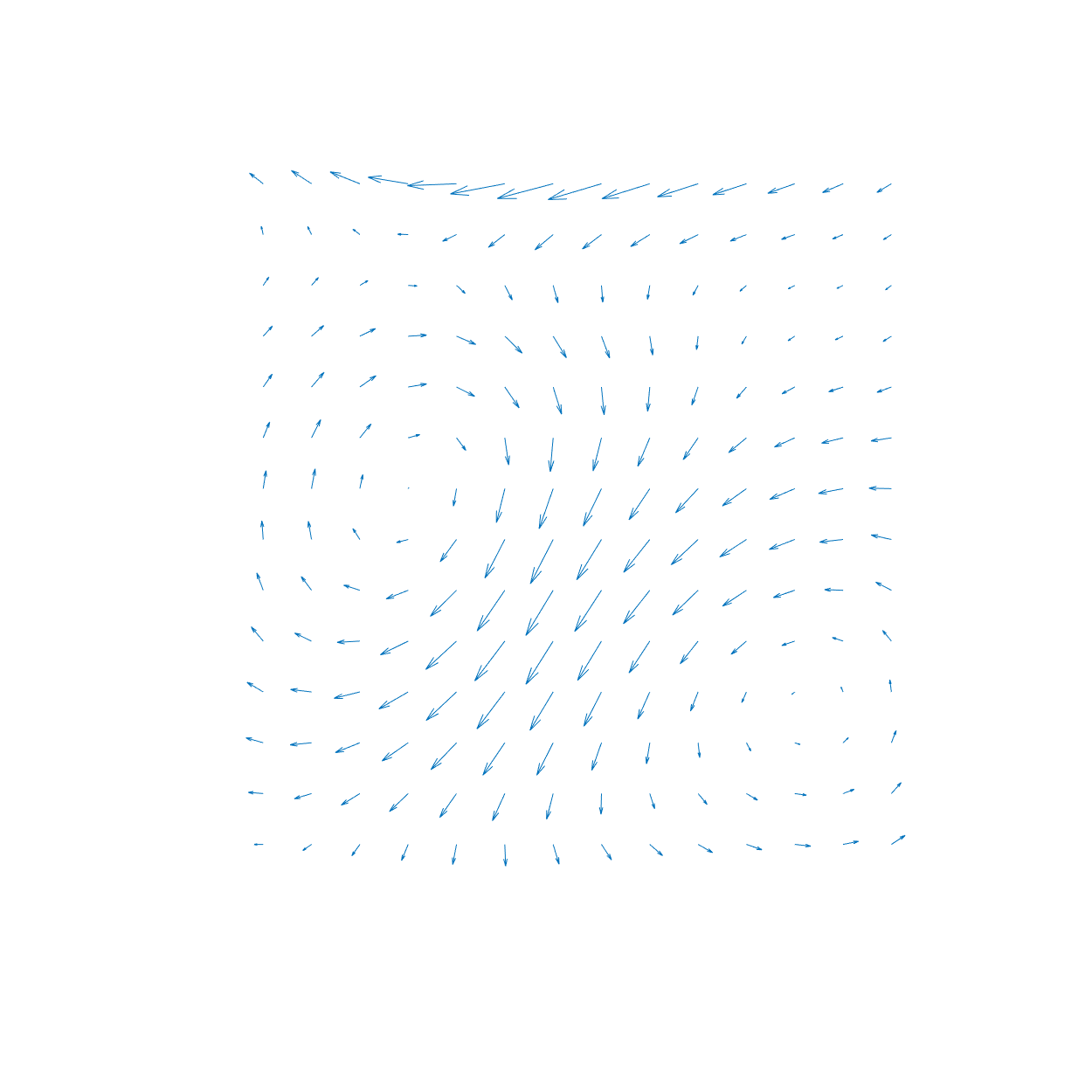

Supplement: S2 MCG raw data 2 — The raw MCG dataset includes categories 0-3 for training and validation. (ZIP) [file pone.0338189.s002.zip › train/0/p4_210_1.png]

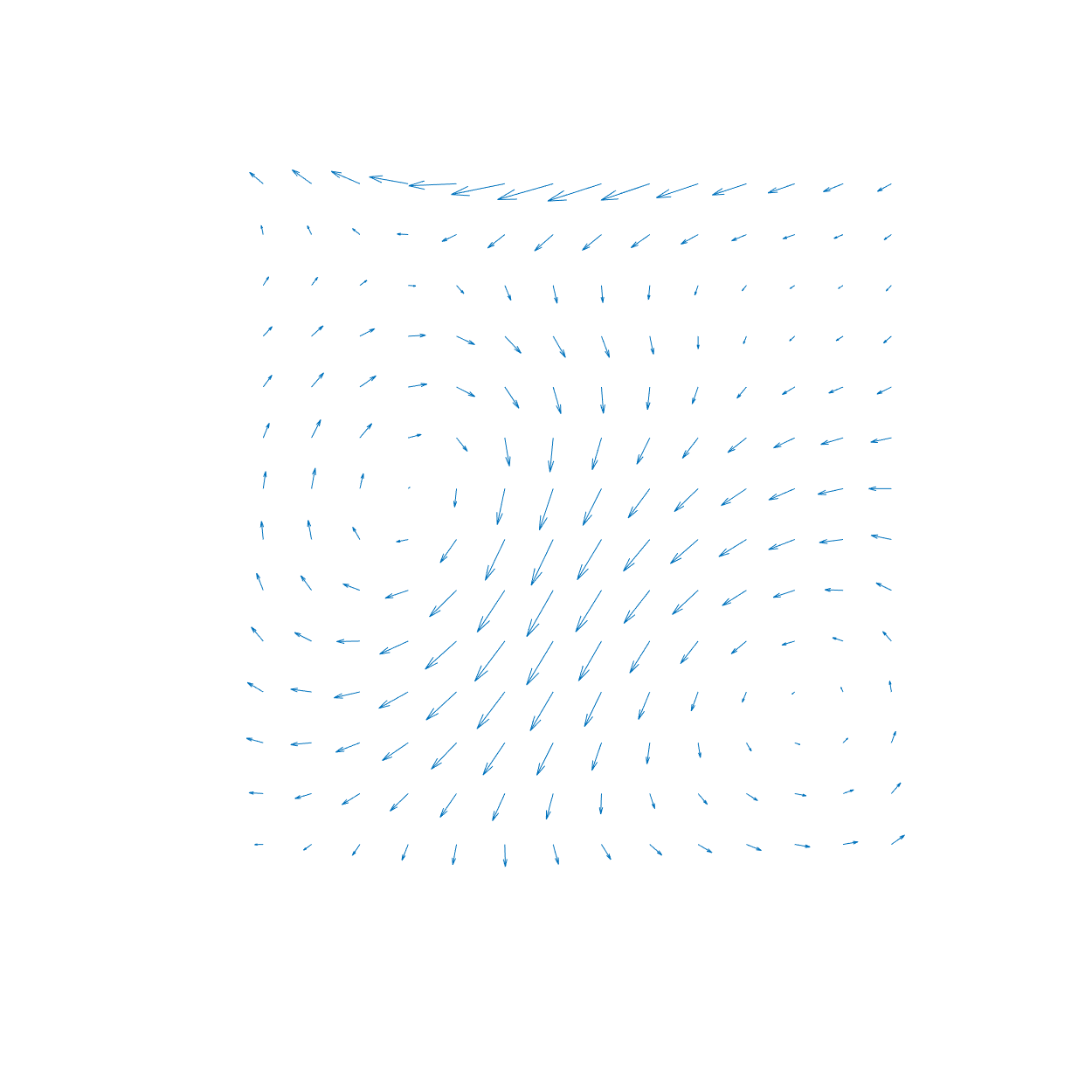

Supplement: S2 MCG raw data 2 — The raw MCG dataset includes categories 0-3 for training and validation. (ZIP) [file pone.0338189.s002.zip › train/0/p4_210_2.png]

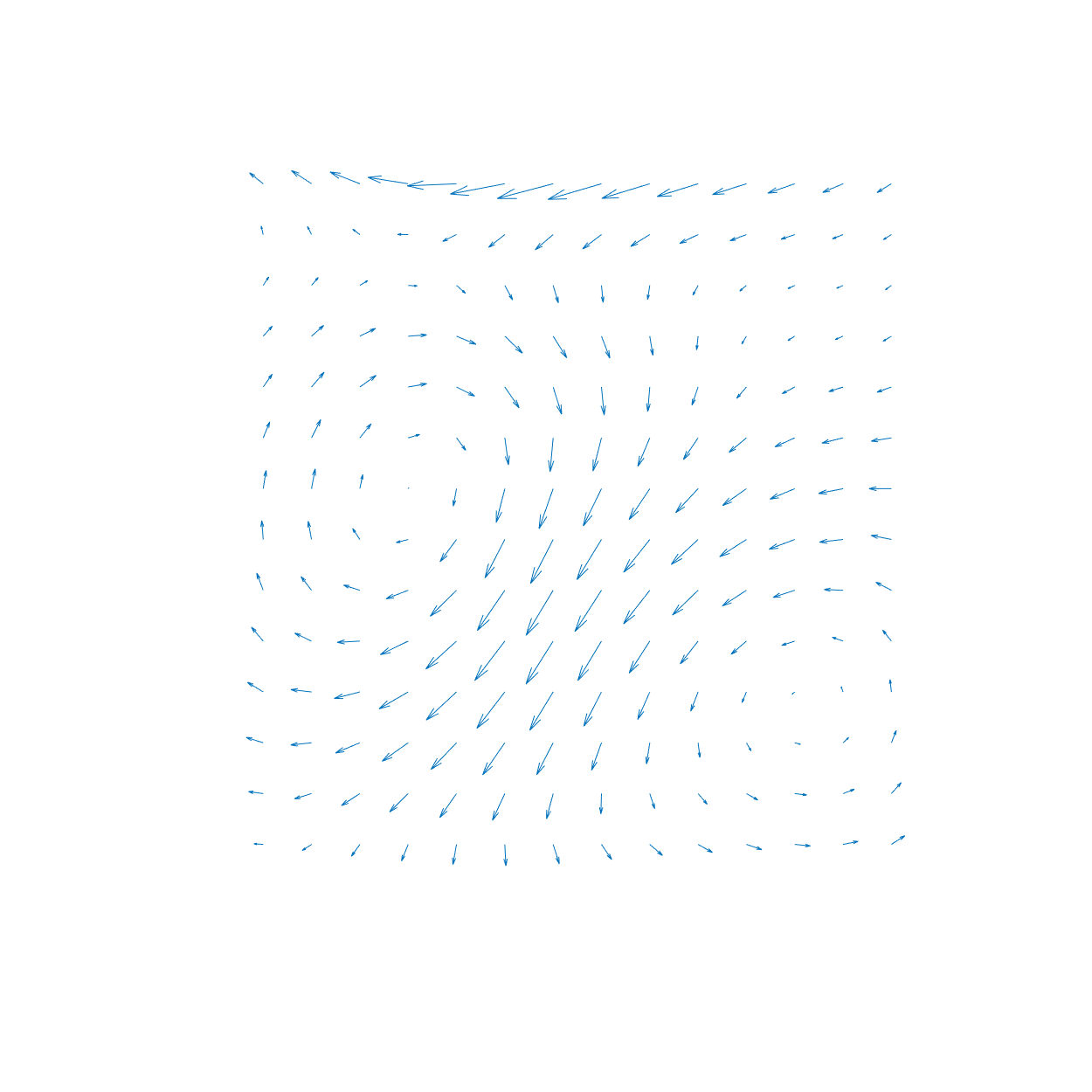

Supplement: S2 MCG raw data 2 — The raw MCG dataset includes categories 0-3 for training and validation. (ZIP) [file pone.0338189.s002.zip › train/0/p4_210_3.png]

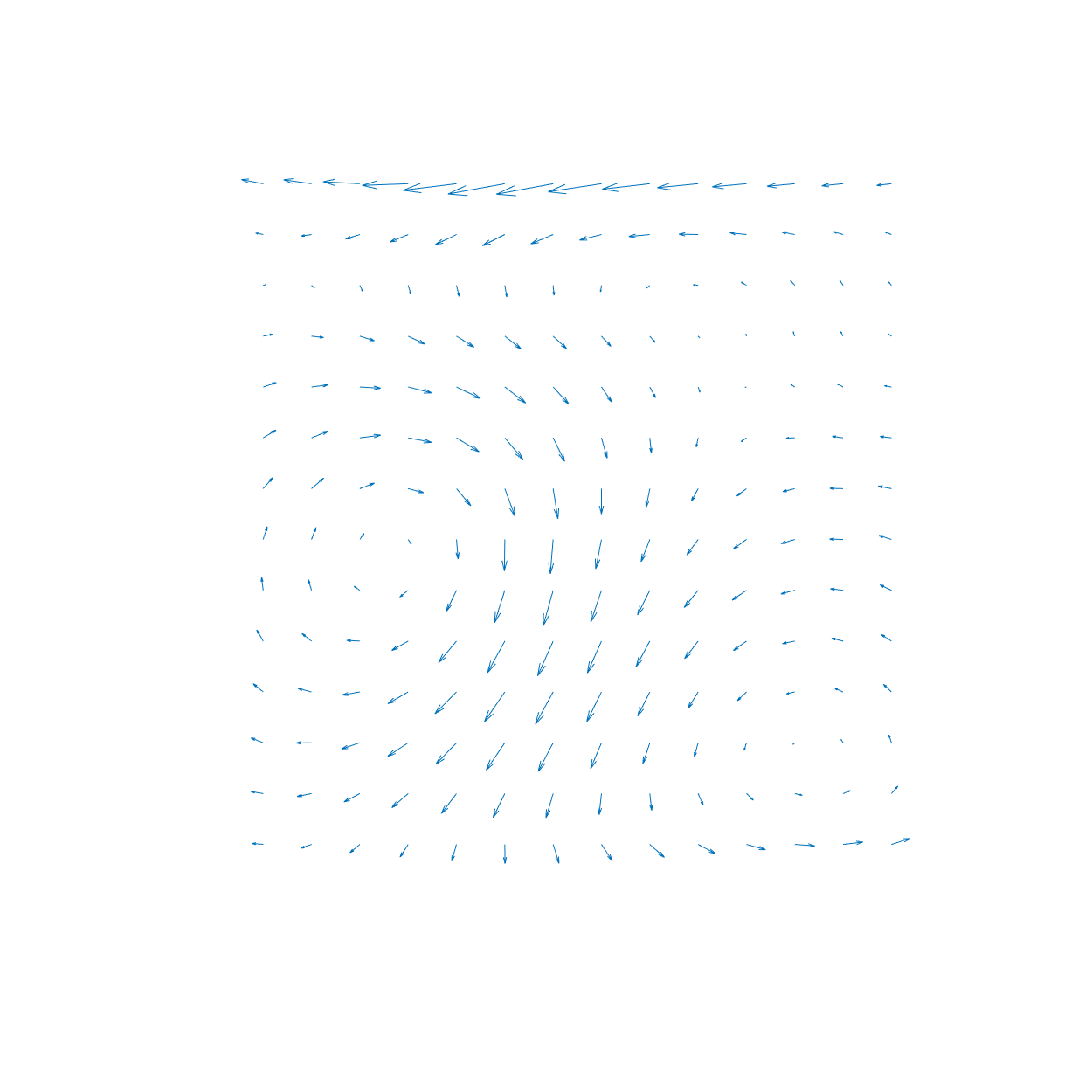

Supplement: S2 MCG raw data 2 — The raw MCG dataset includes categories 0-3 for training and validation. (ZIP) [file pone.0338189.s002.zip › train/0/p4_215_1.png]

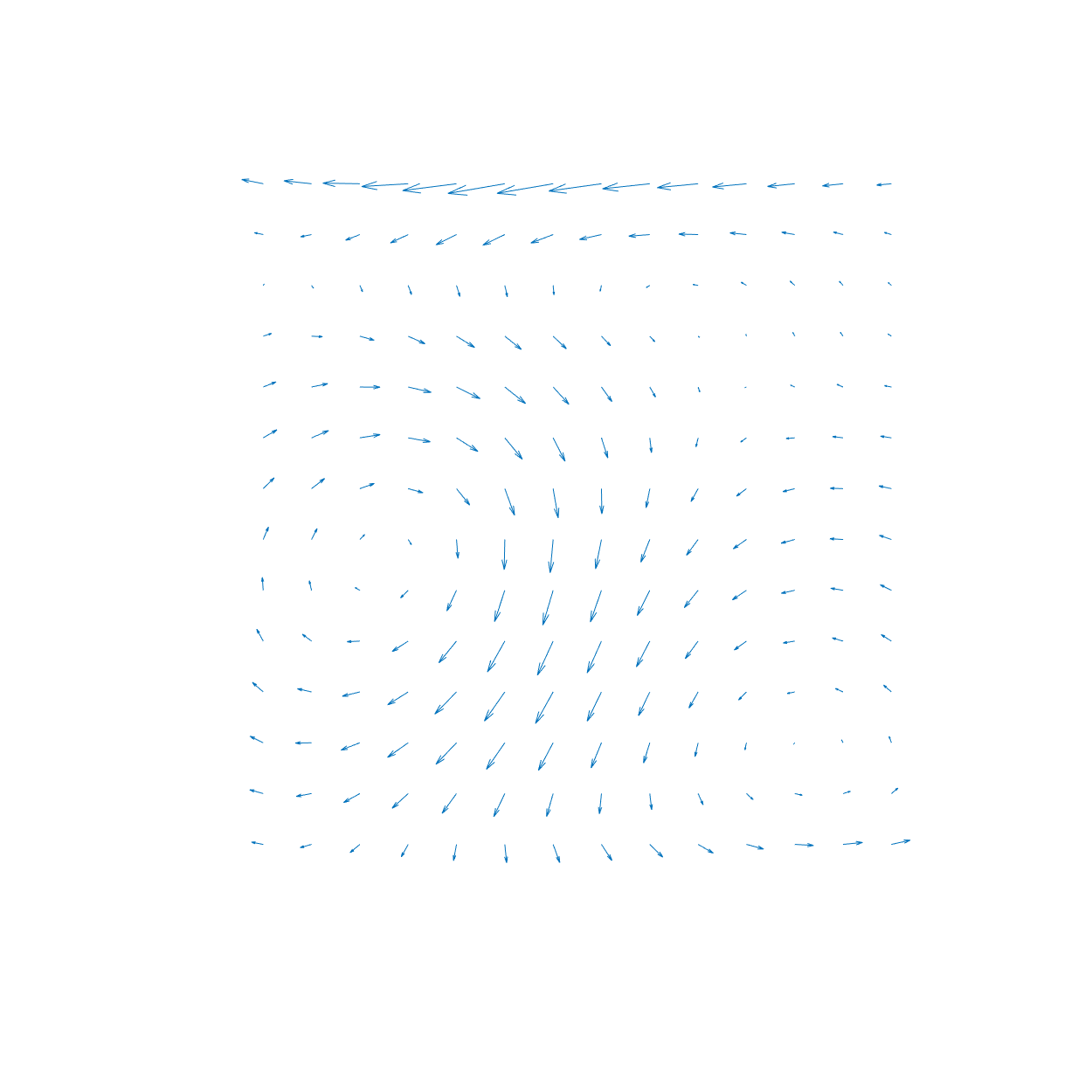

Supplement: S2 MCG raw data 2 — The raw MCG dataset includes categories 0-3 for training and validation. (ZIP) [file pone.0338189.s002.zip › train/0/p4_215_2.png]

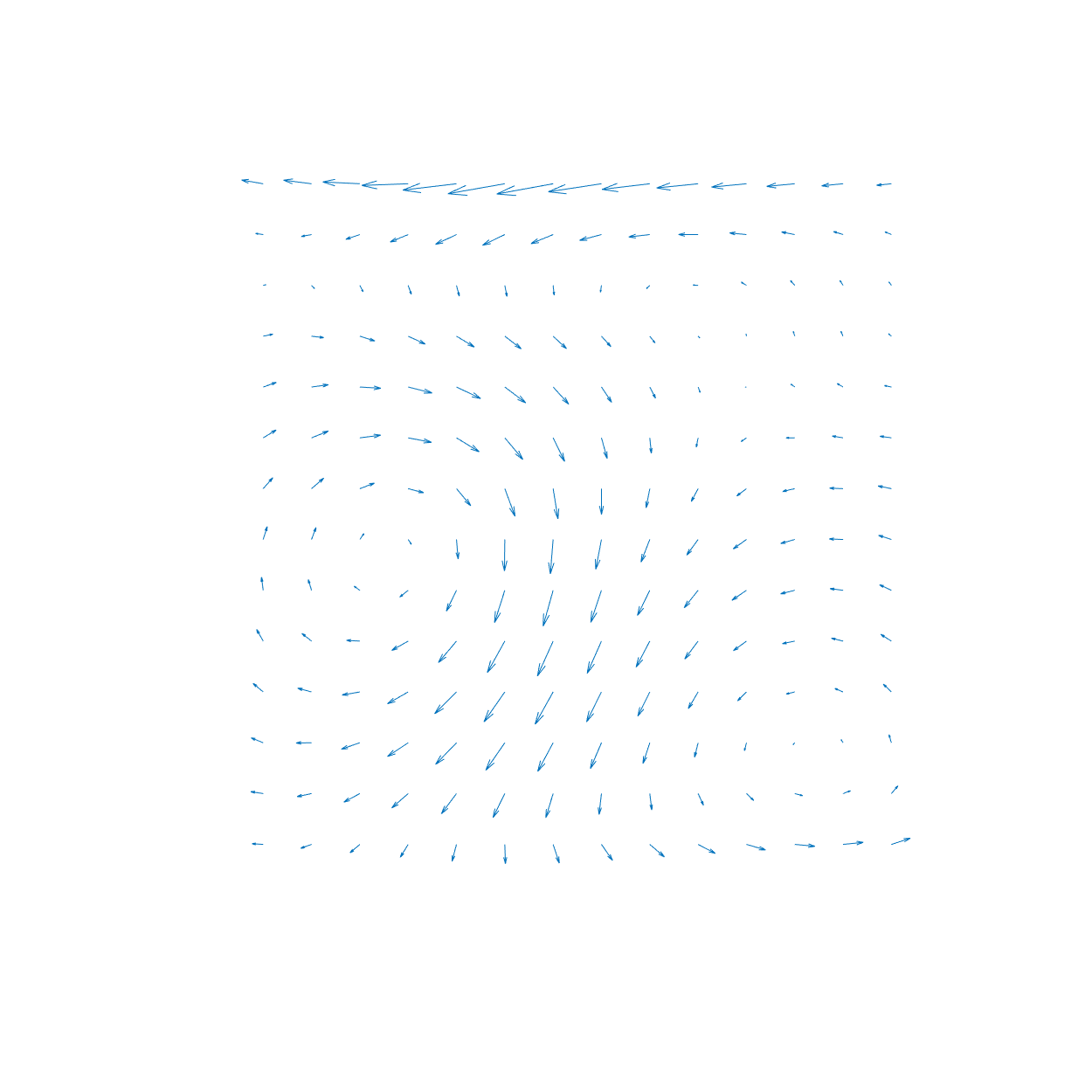

Supplement: S2 MCG raw data 2 — The raw MCG dataset includes categories 0-3 for training and validation. (ZIP) [file pone.0338189.s002.zip › train/0/p4_215_3.png]

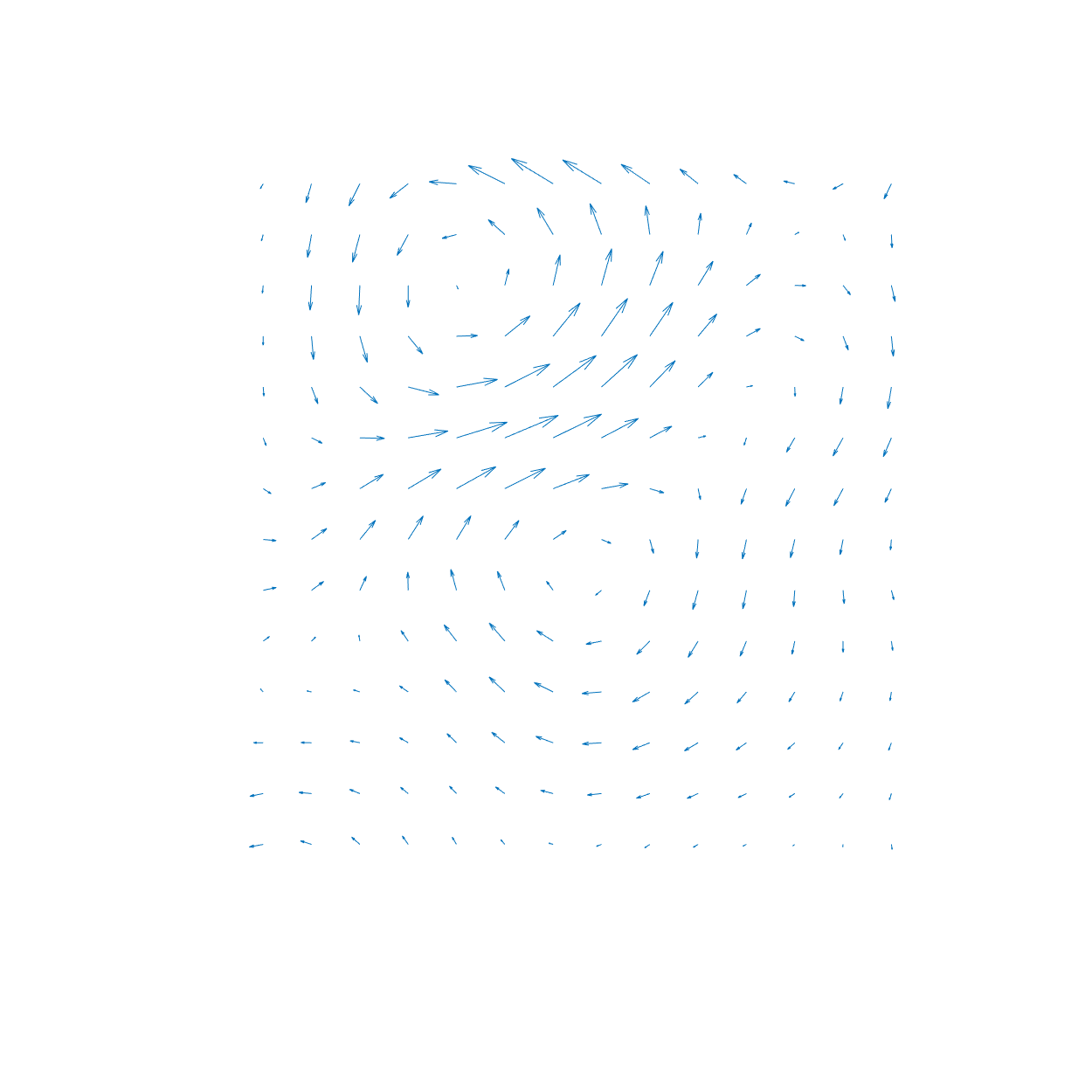

Supplement: S2 MCG raw data 2 — The raw MCG dataset includes categories 0-3 for training and validation. (ZIP) [file pone.0338189.s002.zip › train/0/p4_330_1.png]
