# Supplementary figures and images for: Classification of current density vector map using transformer hybrid residual network (part 6 of 6)
Source: PLoS One. 2025 Dec 16;20(12):e0338189. doi: 10.1371/journal.pone.0338189 (PMC12707687; doi:10.1371/journal.pone.0338189)

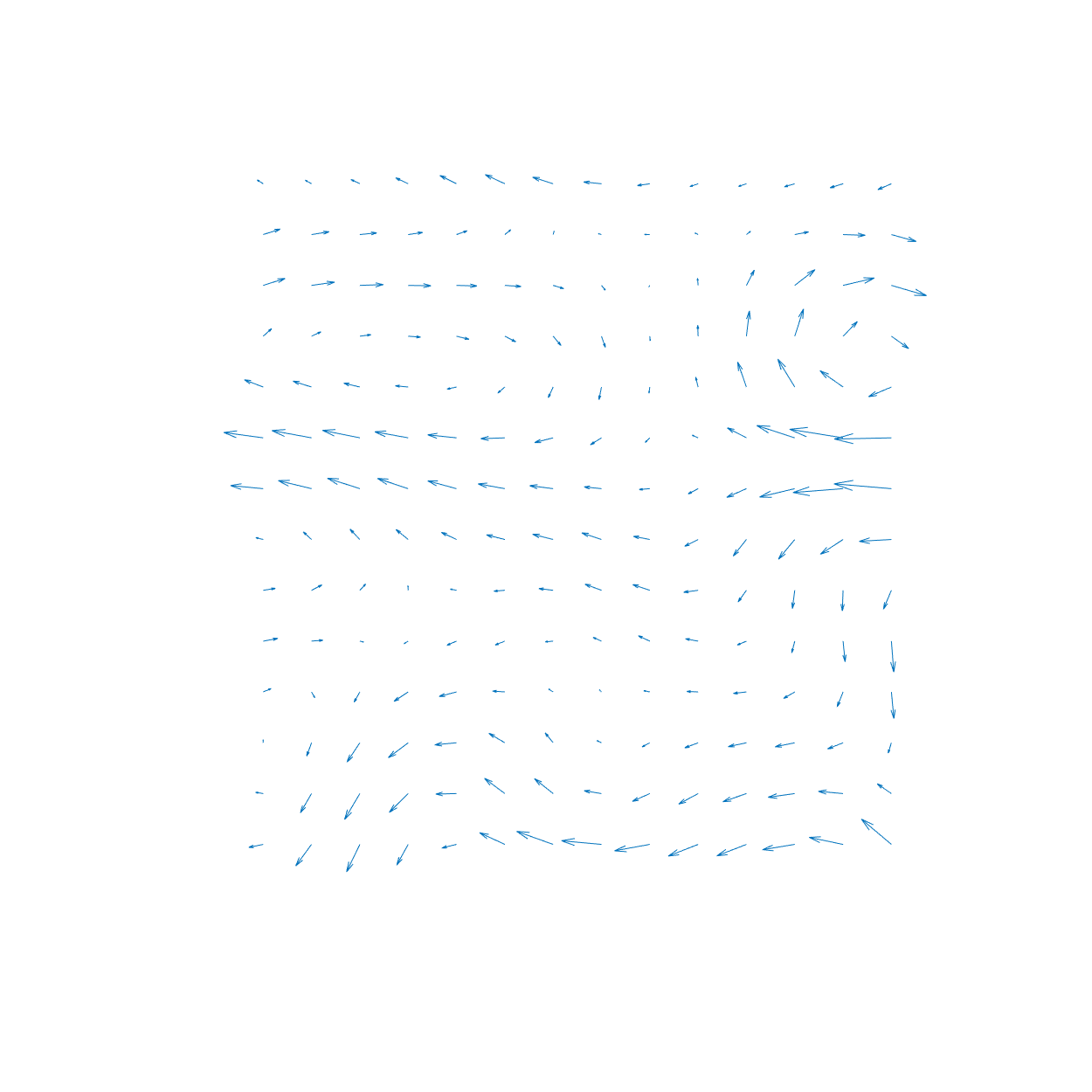

Supplement: S3 MCG raw data 3 — The raw MCG dataset includes category 4 for training and validation. (ZIP) [file pone.0338189.s003.zip › train/4/p10_550_3.png]

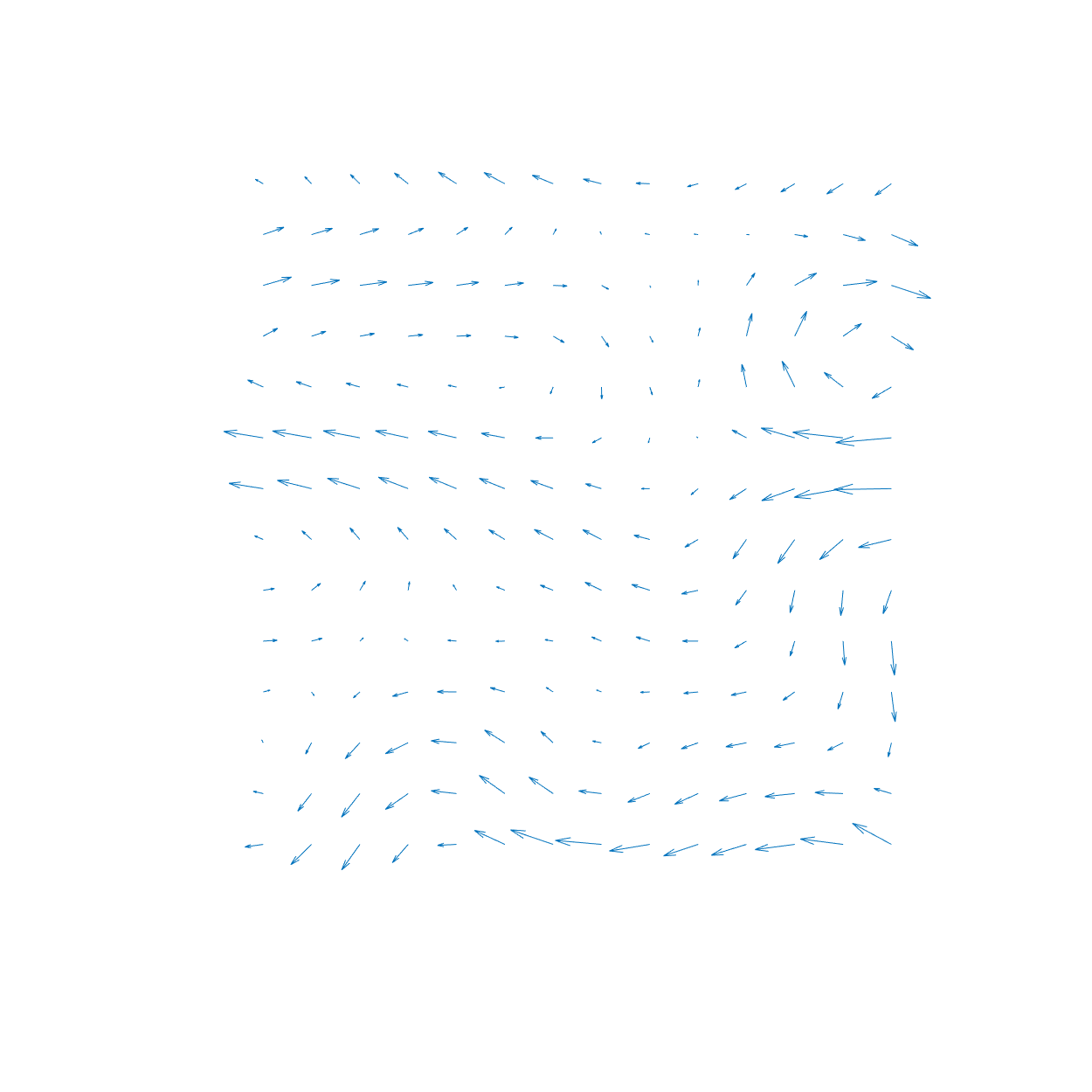

Supplement: S3 MCG raw data 3 — The raw MCG dataset includes category 4 for training and validation. (ZIP) [file pone.0338189.s003.zip › train/4/p10_550_4.png]

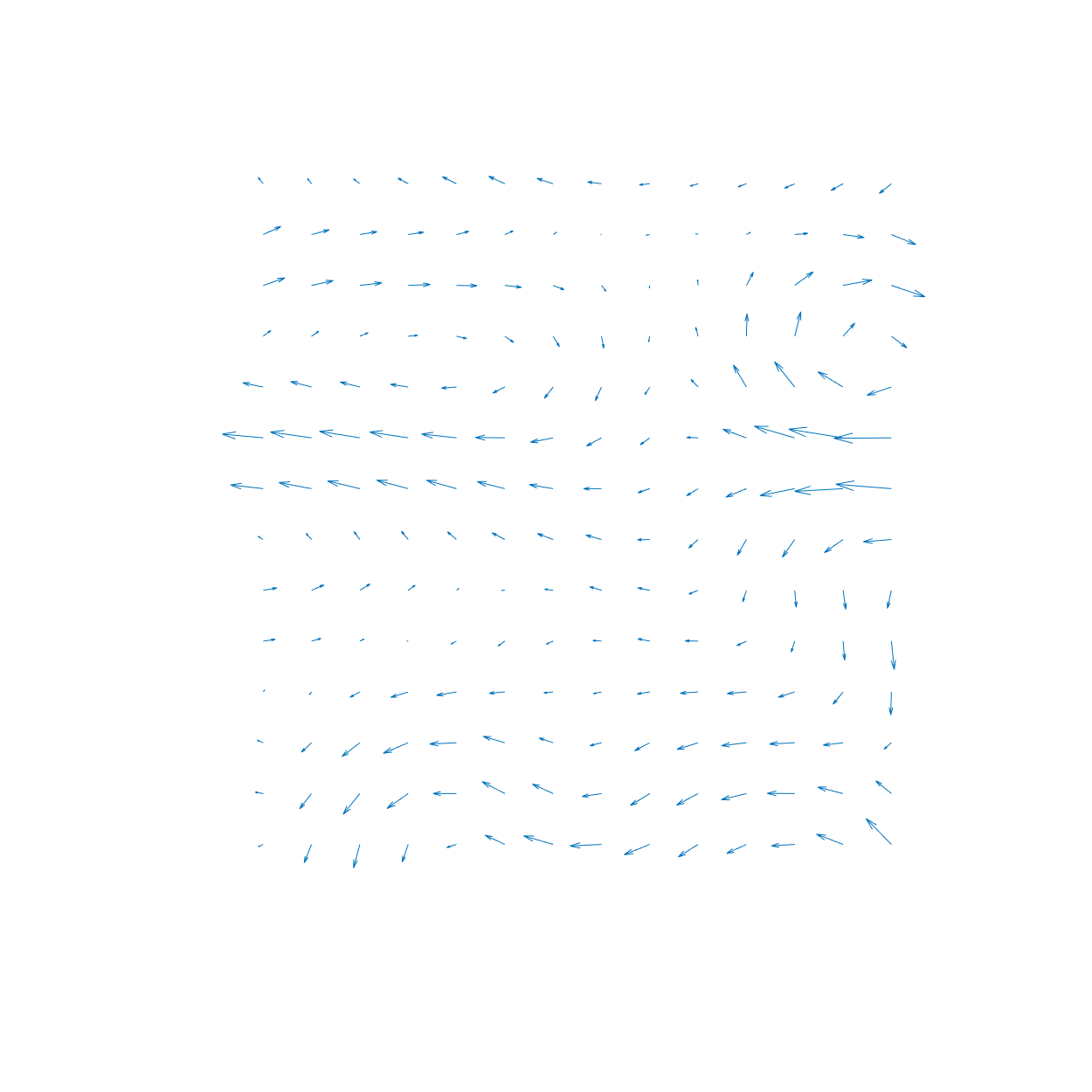

Supplement: S3 MCG raw data 3 — The raw MCG dataset includes category 4 for training and validation. (ZIP) [file pone.0338189.s003.zip › train/4/p10_555_1.png]

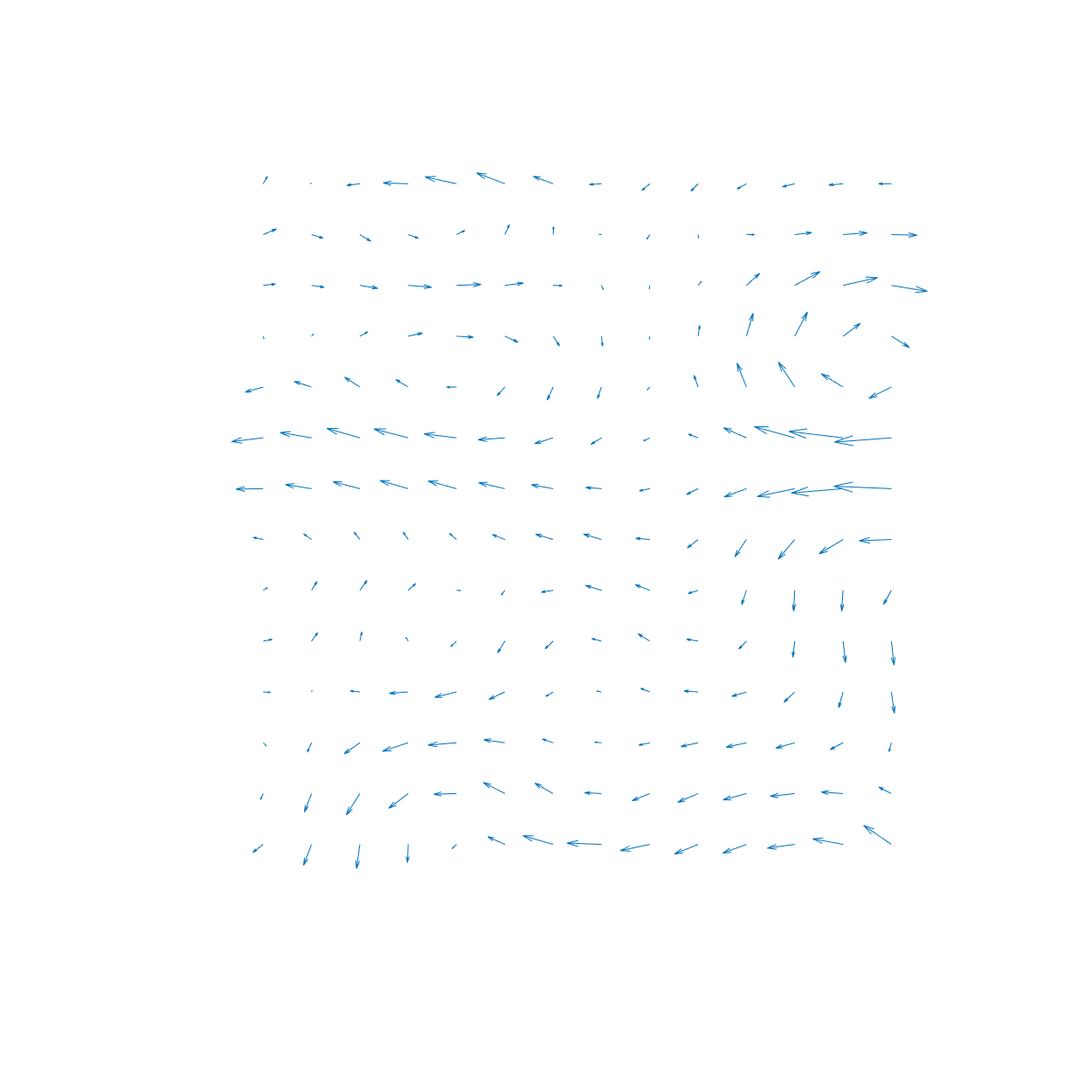

Supplement: S3 MCG raw data 3 — The raw MCG dataset includes category 4 for training and validation. (ZIP) [file pone.0338189.s003.zip › train/4/p10_555_2.png]

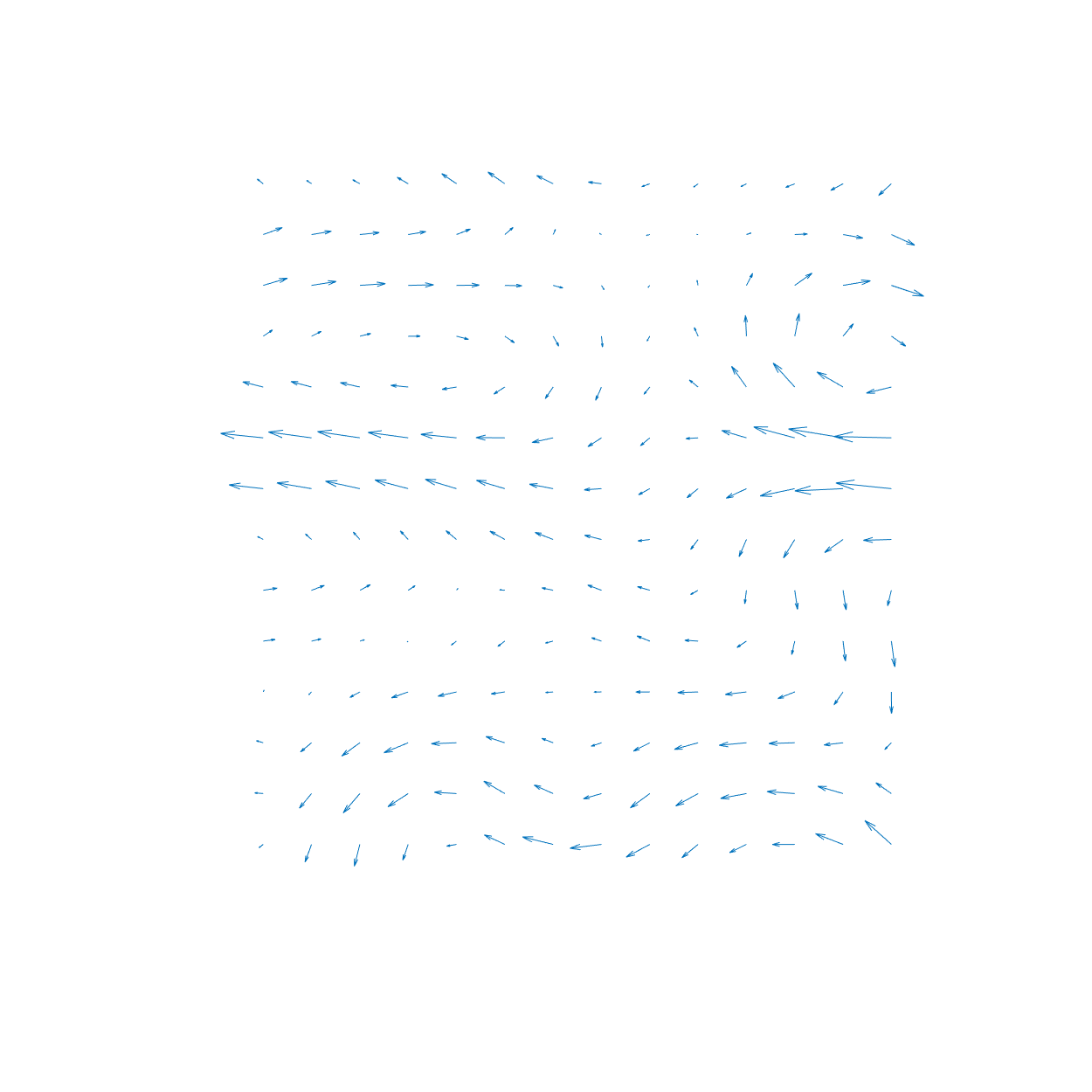

Supplement: S3 MCG raw data 3 — The raw MCG dataset includes category 4 for training and validation. (ZIP) [file pone.0338189.s003.zip › train/4/p10_555_3.png]

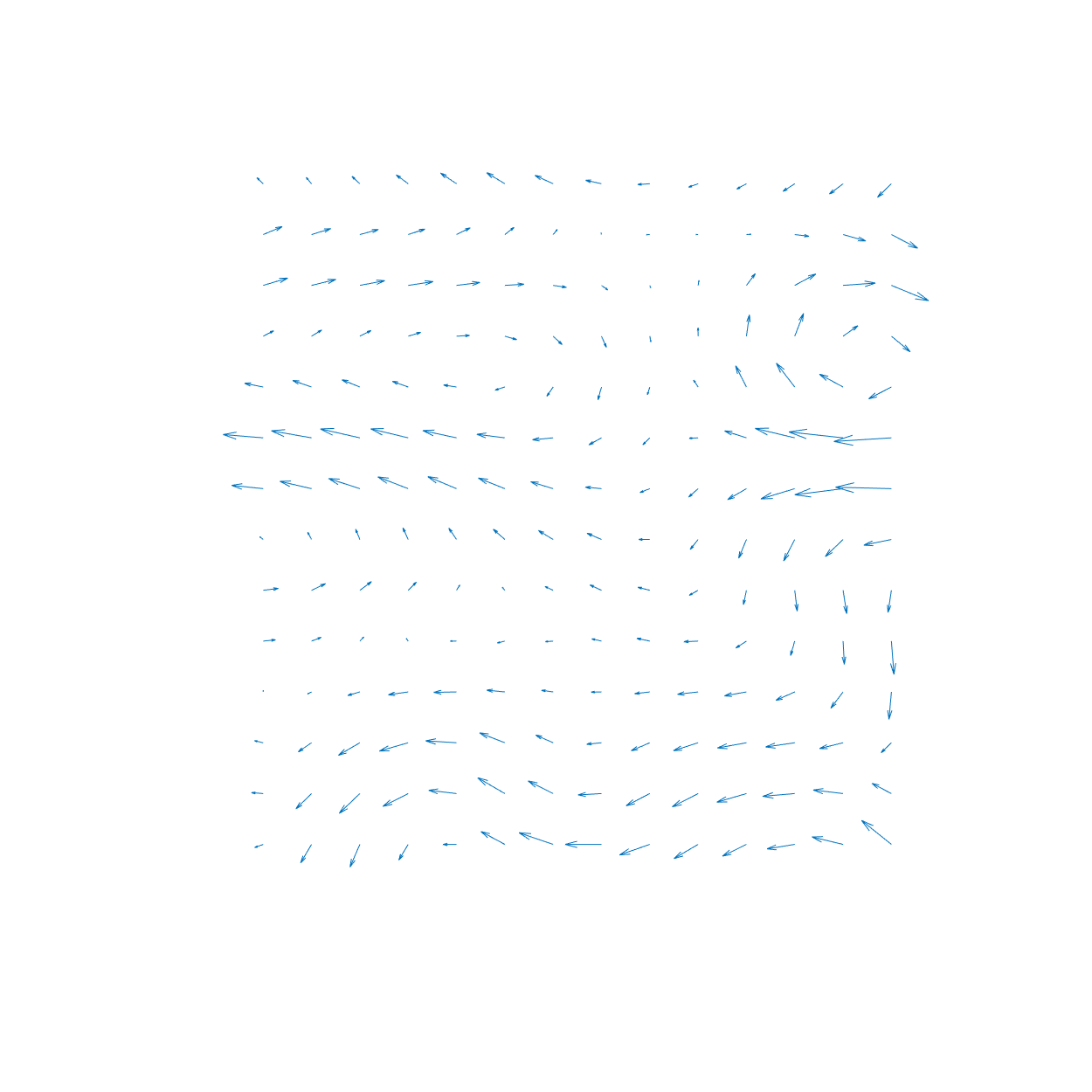

Supplement: S3 MCG raw data 3 — The raw MCG dataset includes category 4 for training and validation. (ZIP) [file pone.0338189.s003.zip › train/4/p10_555_4.png]

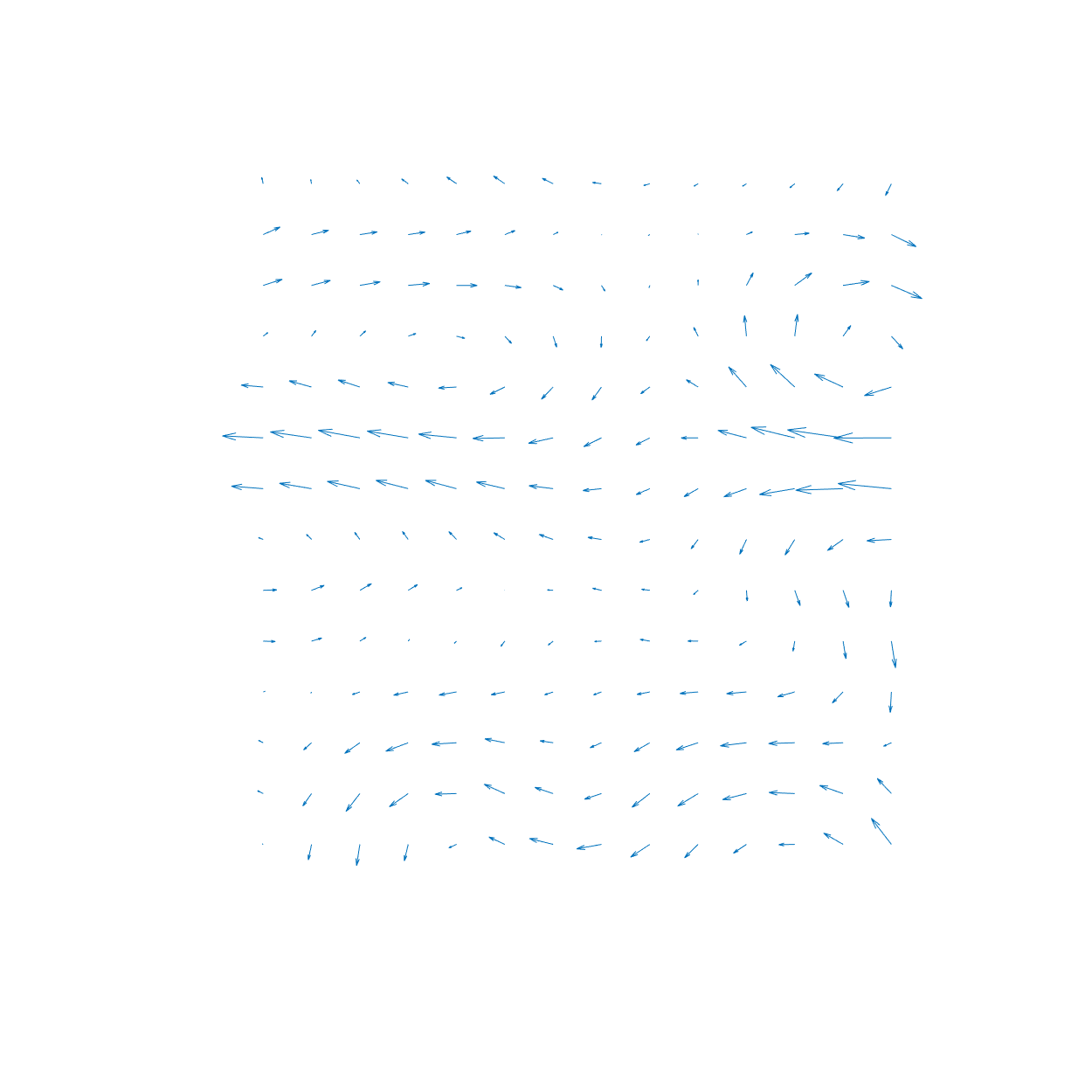

Supplement: S3 MCG raw data 3 — The raw MCG dataset includes category 4 for training and validation. (ZIP) [file pone.0338189.s003.zip › train/4/p10_560_1.png]

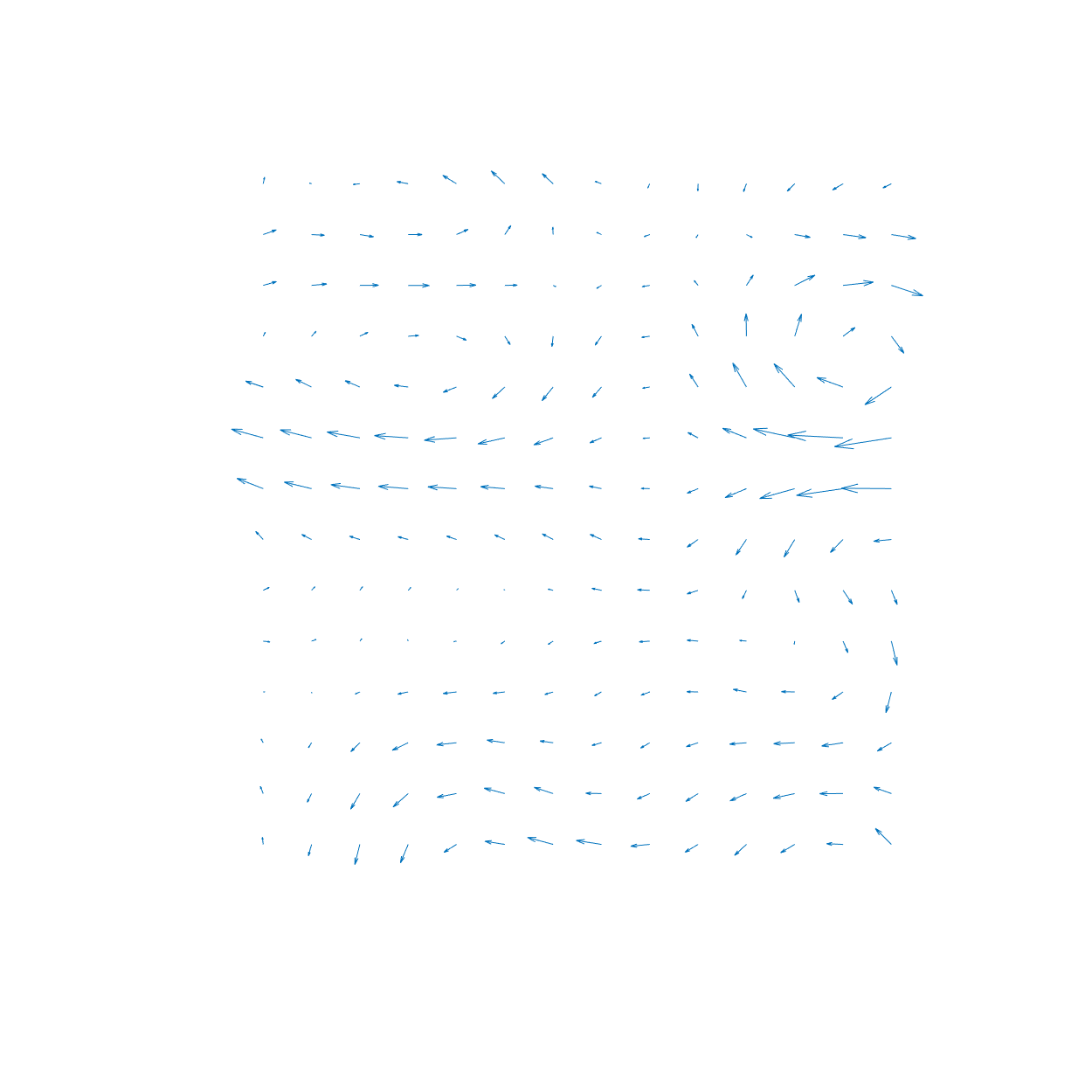

Supplement: S3 MCG raw data 3 — The raw MCG dataset includes category 4 for training and validation. (ZIP) [file pone.0338189.s003.zip › train/4/p10_560_2.png]

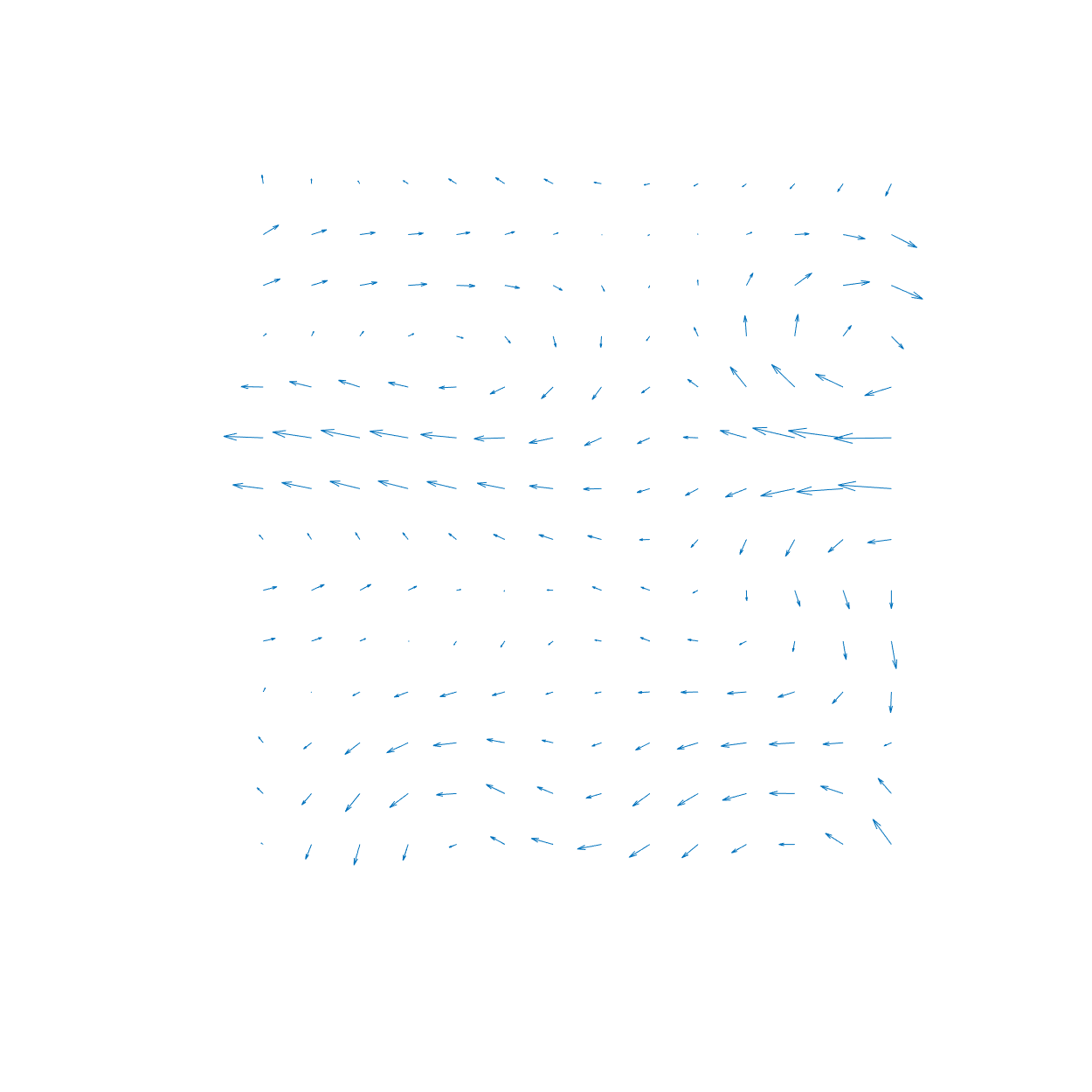

Supplement: S3 MCG raw data 3 — The raw MCG dataset includes category 4 for training and validation. (ZIP) [file pone.0338189.s003.zip › train/4/p10_560_3.png]

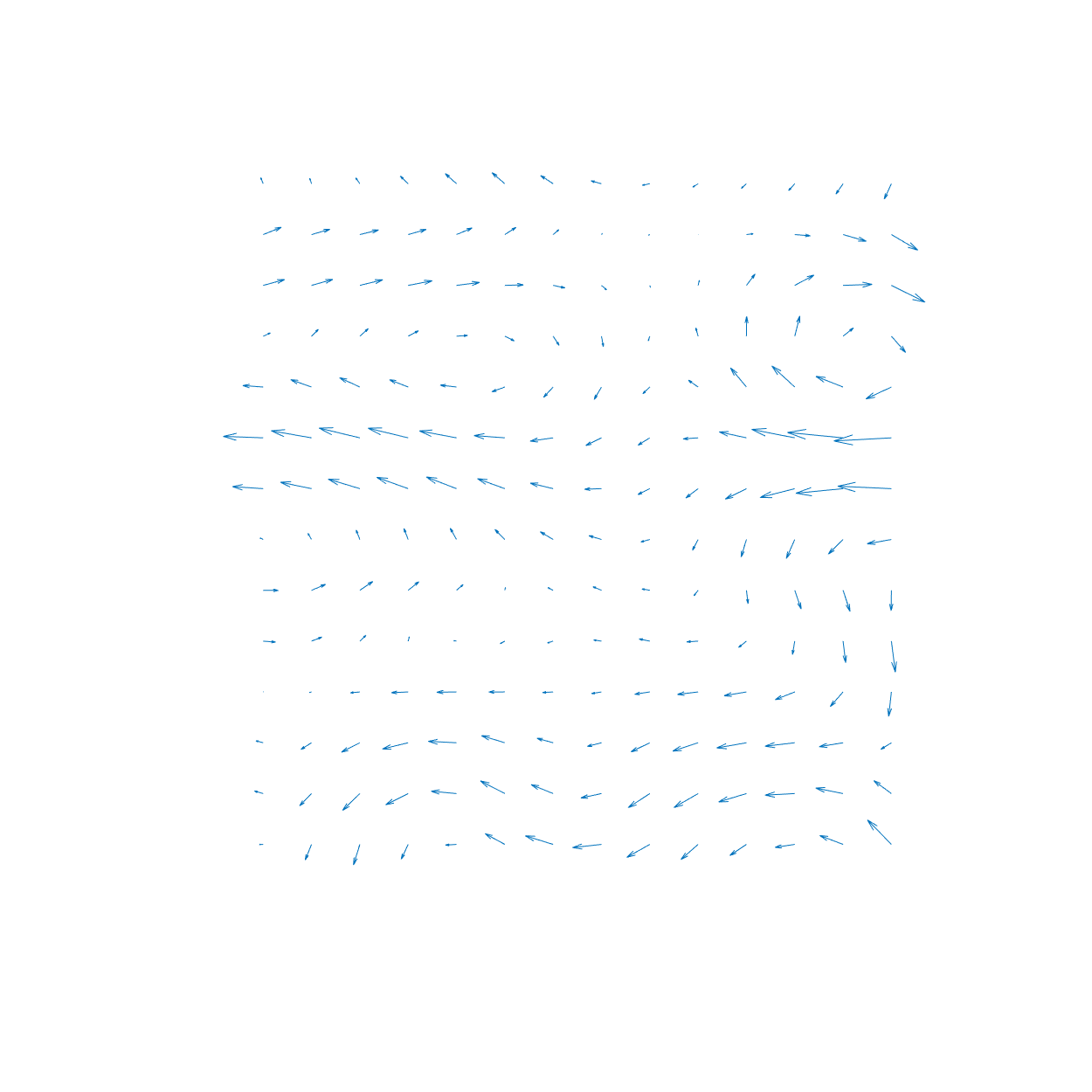

Supplement: S3 MCG raw data 3 — The raw MCG dataset includes category 4 for training and validation. (ZIP) [file pone.0338189.s003.zip › train/4/p10_560_4.png]

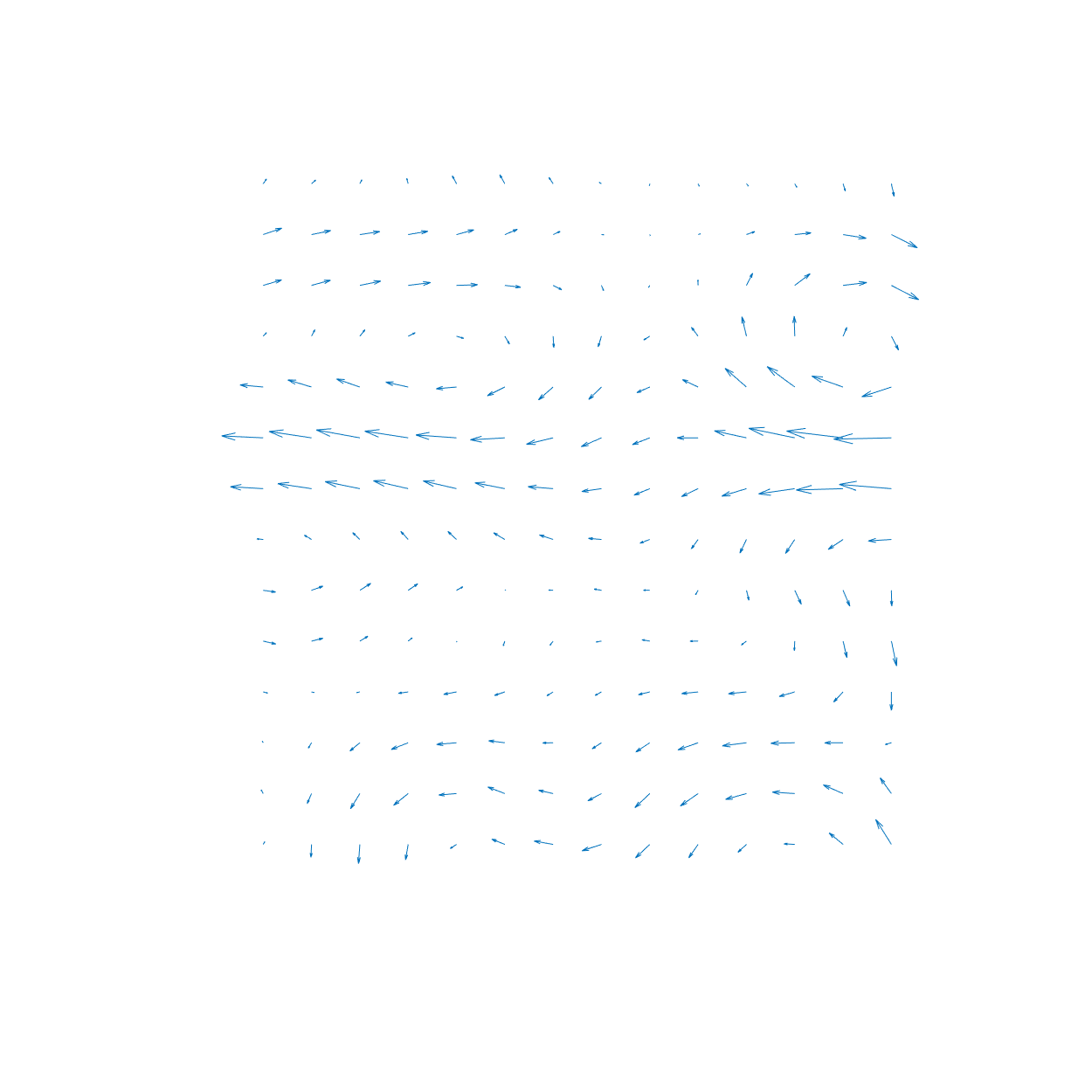

Supplement: S3 MCG raw data 3 — The raw MCG dataset includes category 4 for training and validation. (ZIP) [file pone.0338189.s003.zip › train/4/p10_565_1.png]

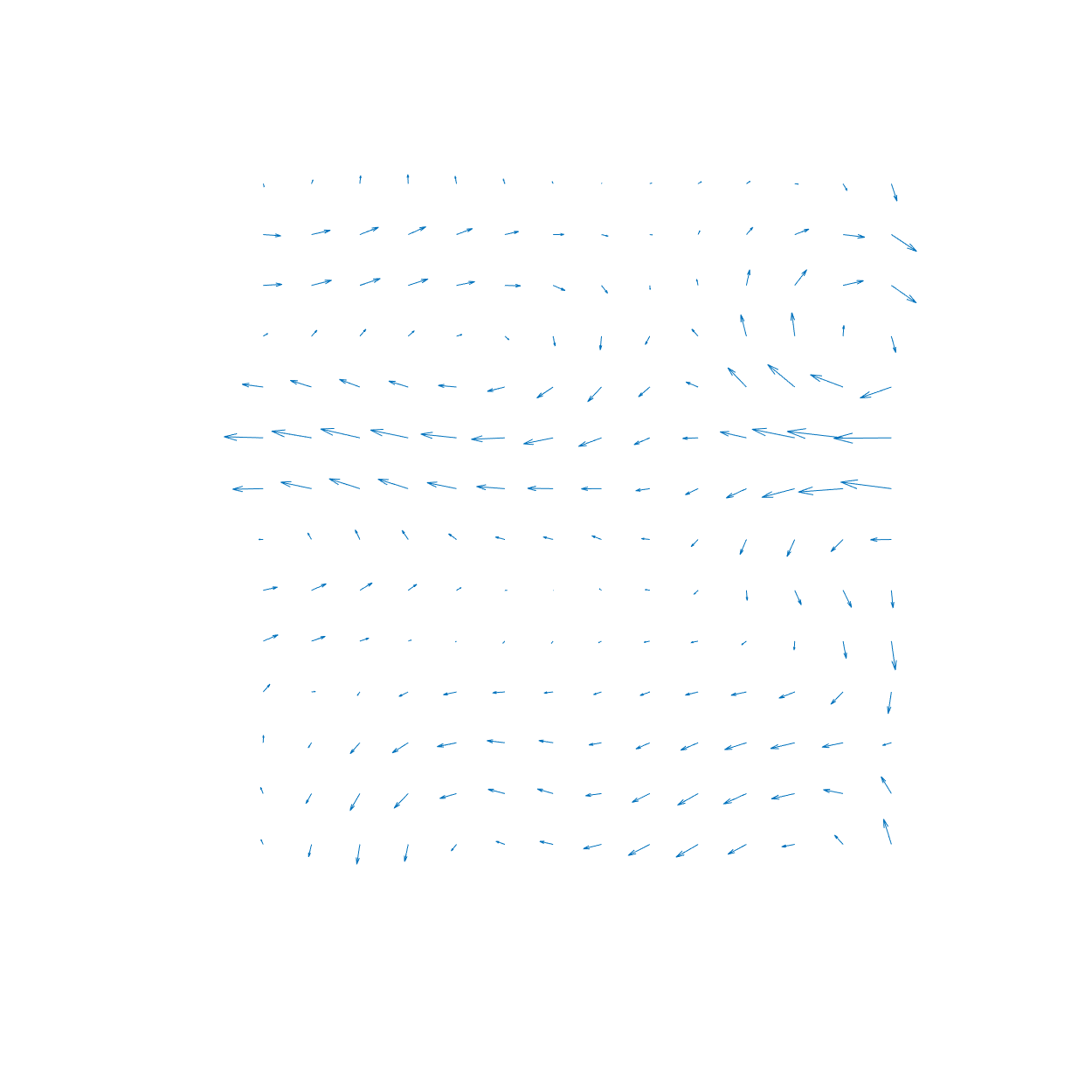

Supplement: S3 MCG raw data 3 — The raw MCG dataset includes category 4 for training and validation. (ZIP) [file pone.0338189.s003.zip › train/4/p10_565_2.png]

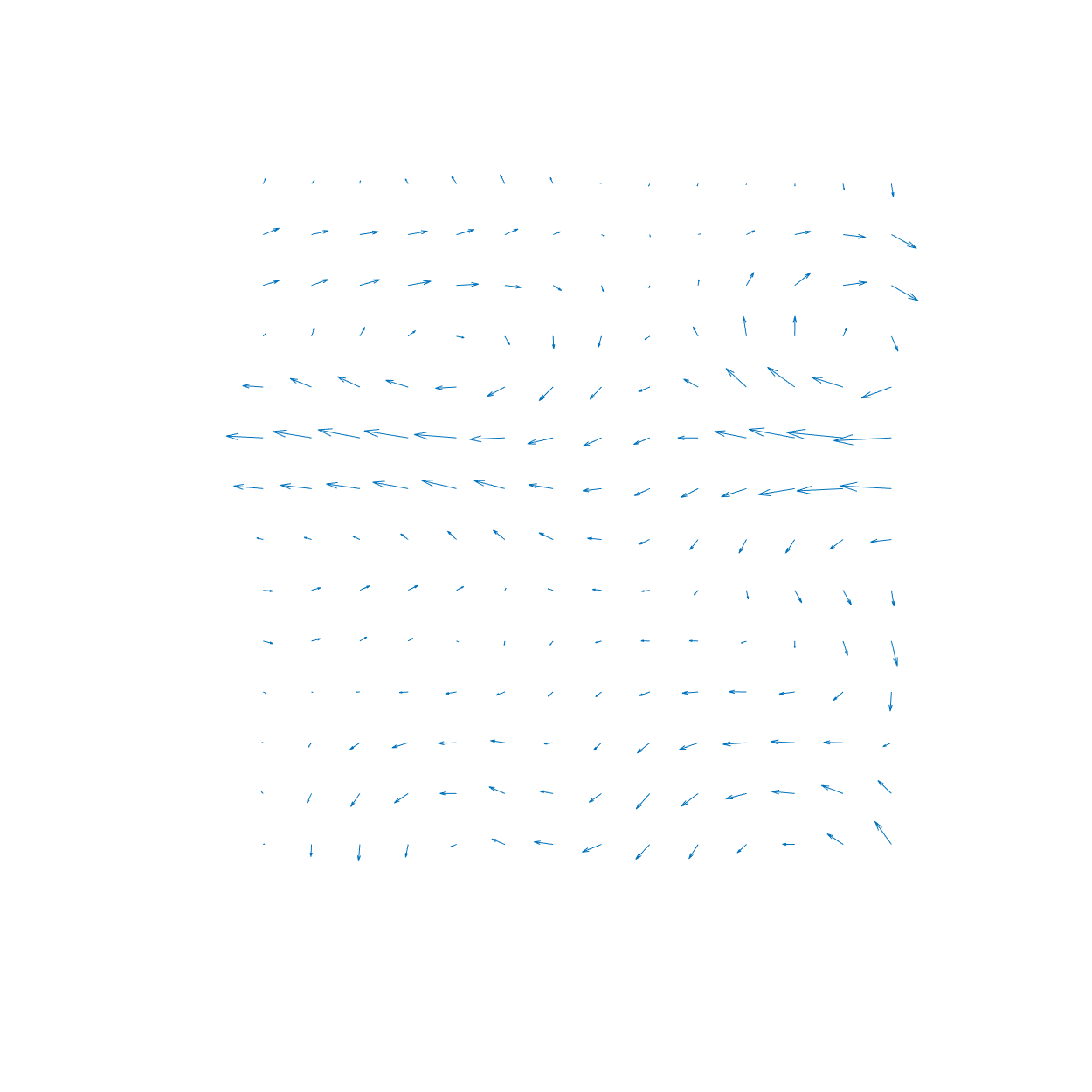

Supplement: S3 MCG raw data 3 — The raw MCG dataset includes category 4 for training and validation. (ZIP) [file pone.0338189.s003.zip › train/4/p10_565_3.png]

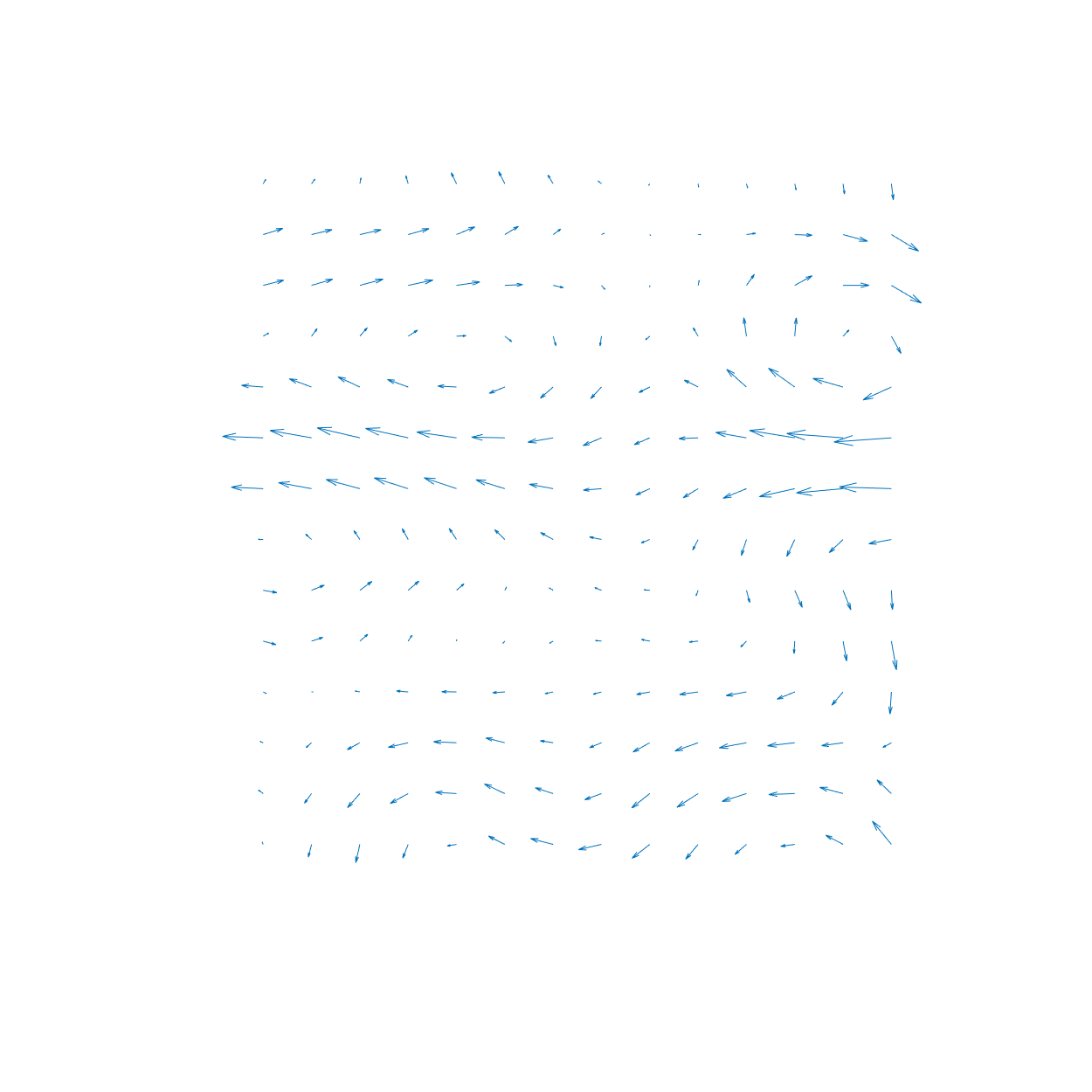

Supplement: S3 MCG raw data 3 — The raw MCG dataset includes category 4 for training and validation. (ZIP) [file pone.0338189.s003.zip › train/4/p10_565_4.png]

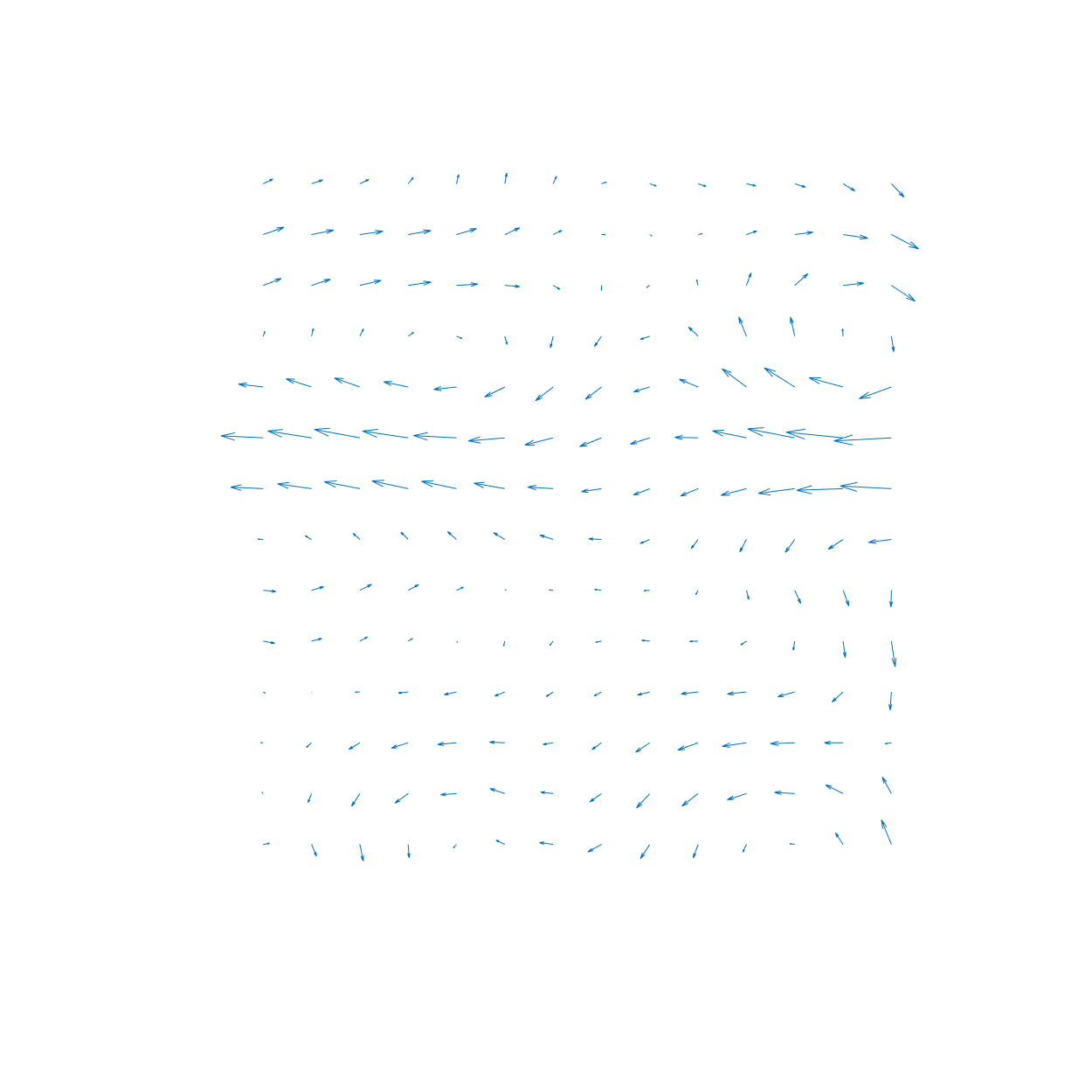

Supplement: S3 MCG raw data 3 — The raw MCG dataset includes category 4 for training and validation. (ZIP) [file pone.0338189.s003.zip › train/4/p10_570_1.png]

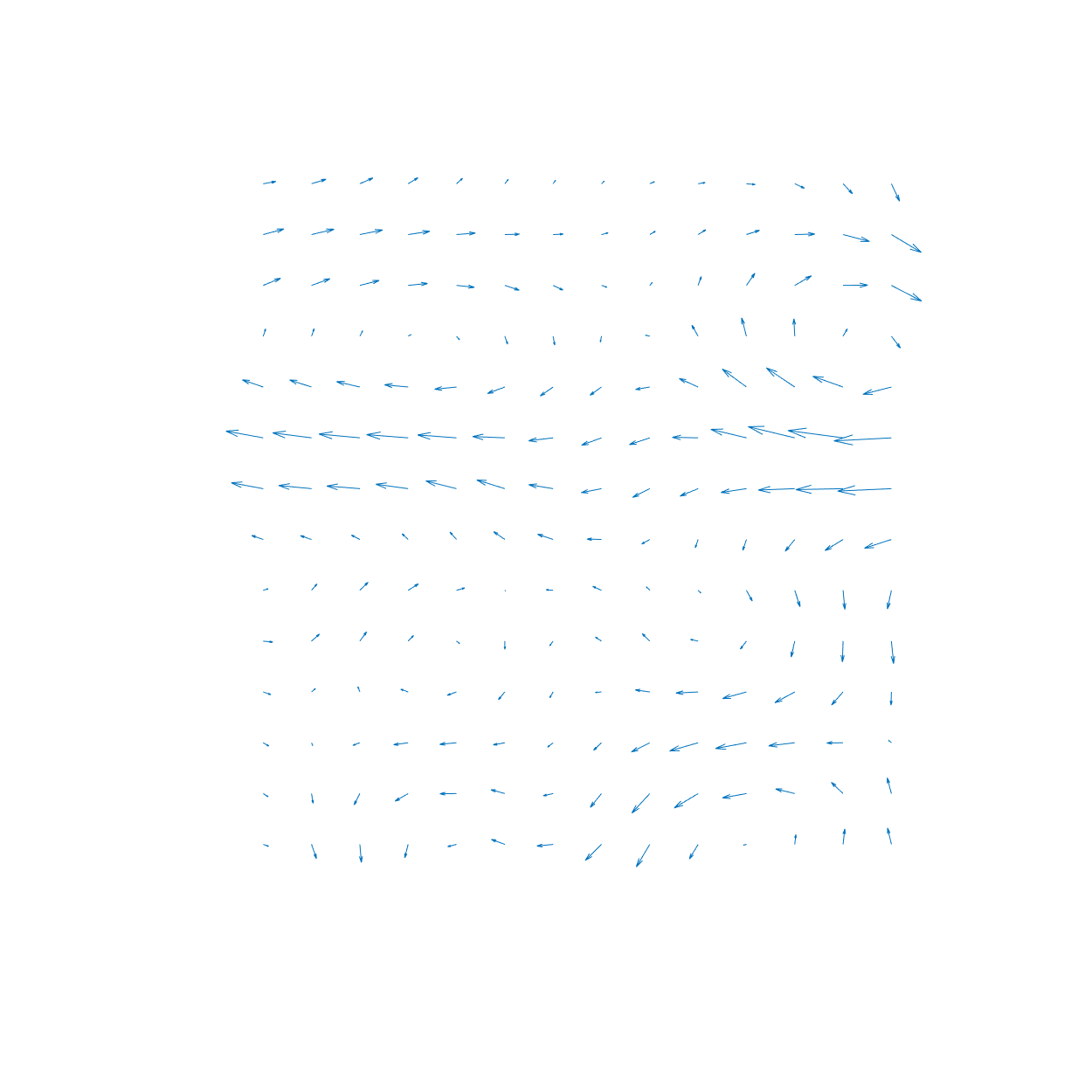

Supplement: S3 MCG raw data 3 — The raw MCG dataset includes category 4 for training and validation. (ZIP) [file pone.0338189.s003.zip › train/4/p10_570_2.png]

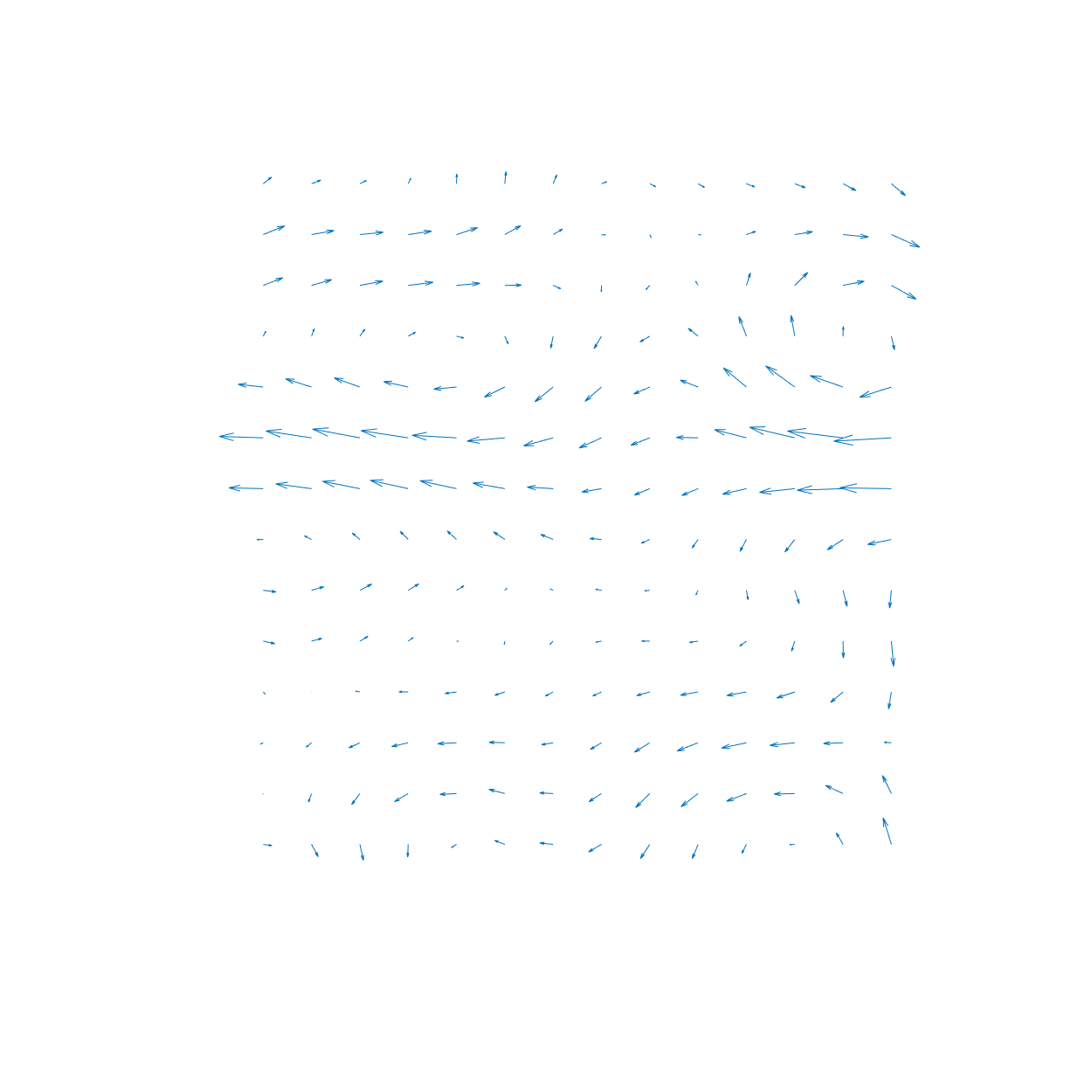

Supplement: S3 MCG raw data 3 — The raw MCG dataset includes category 4 for training and validation. (ZIP) [file pone.0338189.s003.zip › train/4/p10_570_3.png]

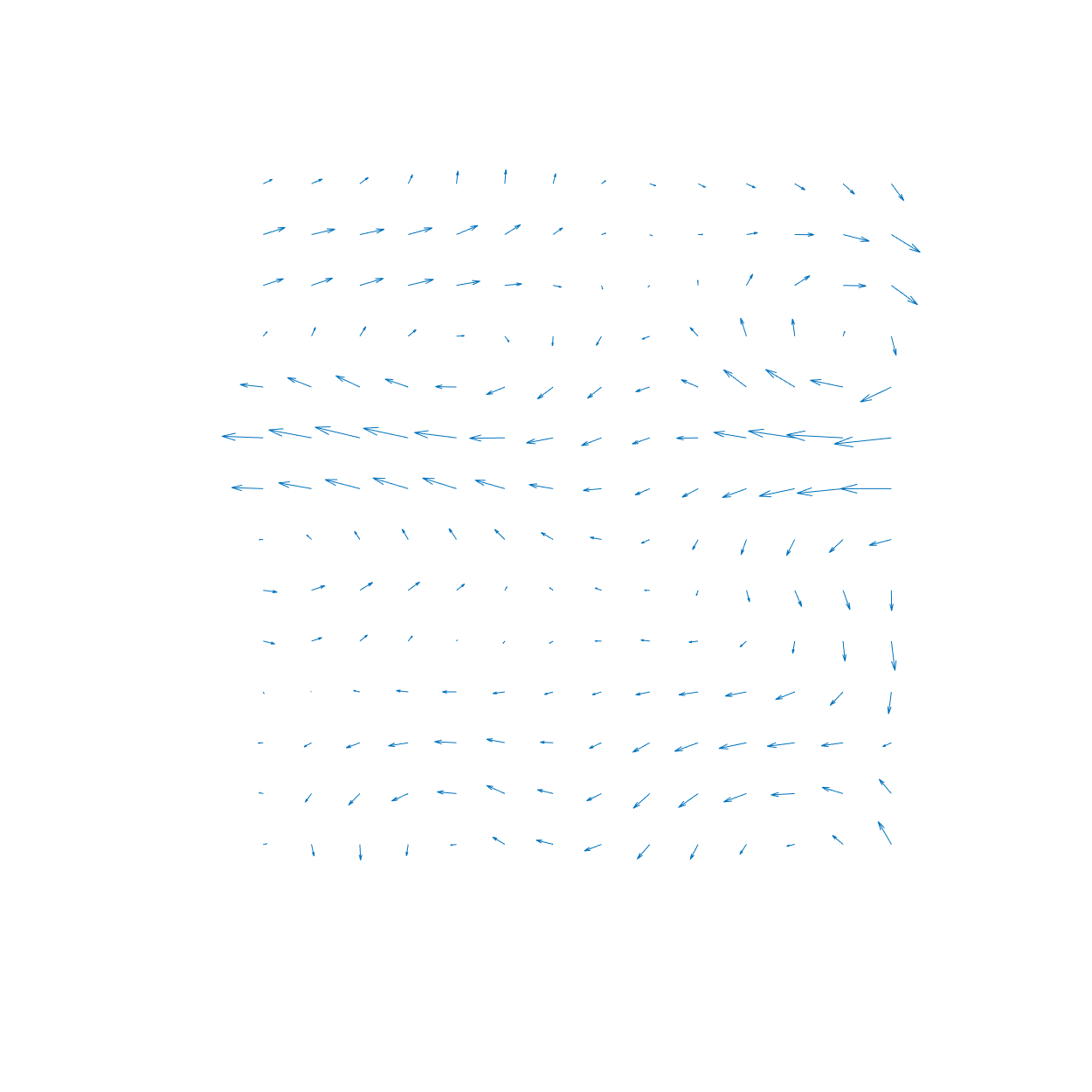

Supplement: S3 MCG raw data 3 — The raw MCG dataset includes category 4 for training and validation. (ZIP) [file pone.0338189.s003.zip › train/4/p10_570_4.png]

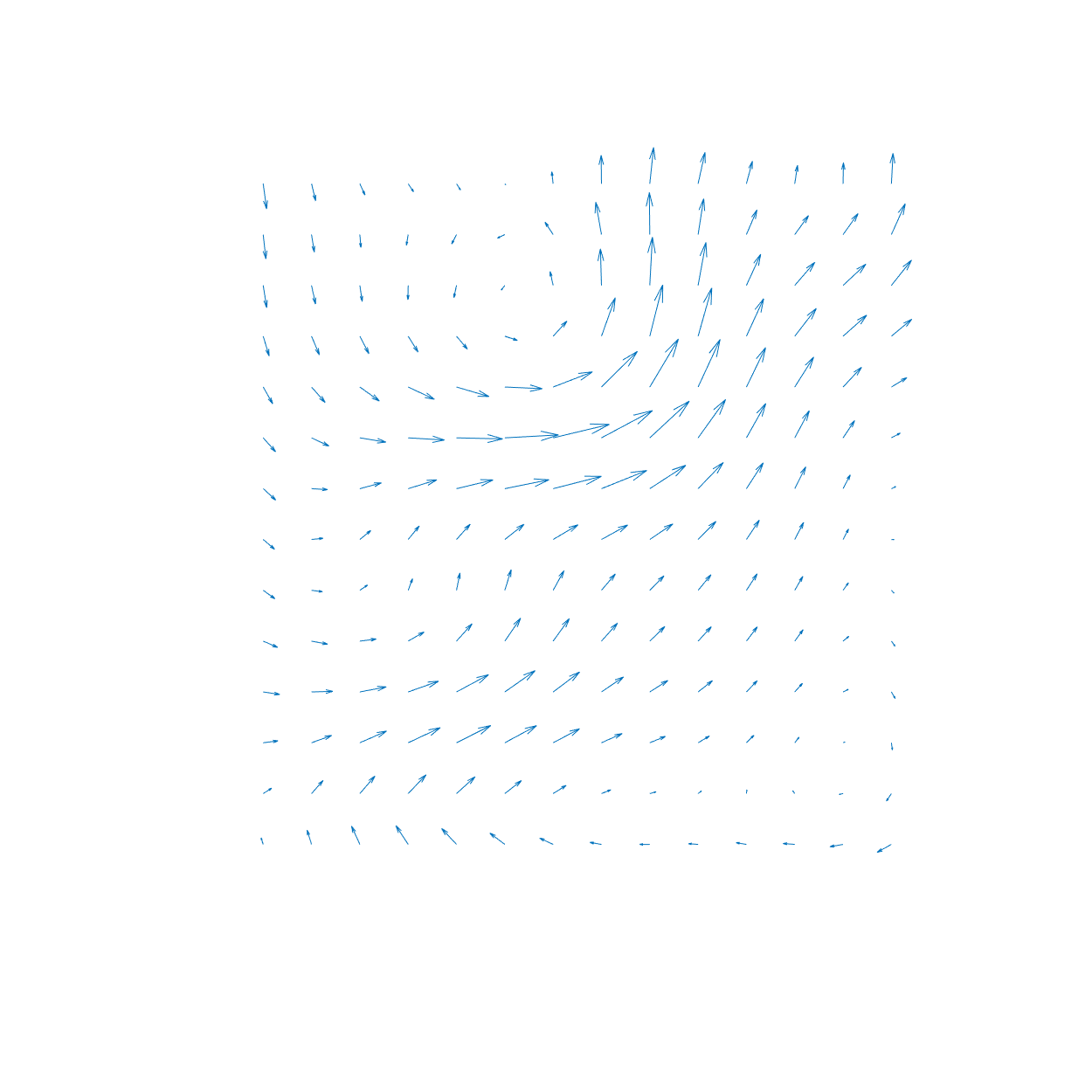

Supplement: S3 MCG raw data 3 — The raw MCG dataset includes category 4 for training and validation. (ZIP) [file pone.0338189.s003.zip › train/4/p11_220_1.png]

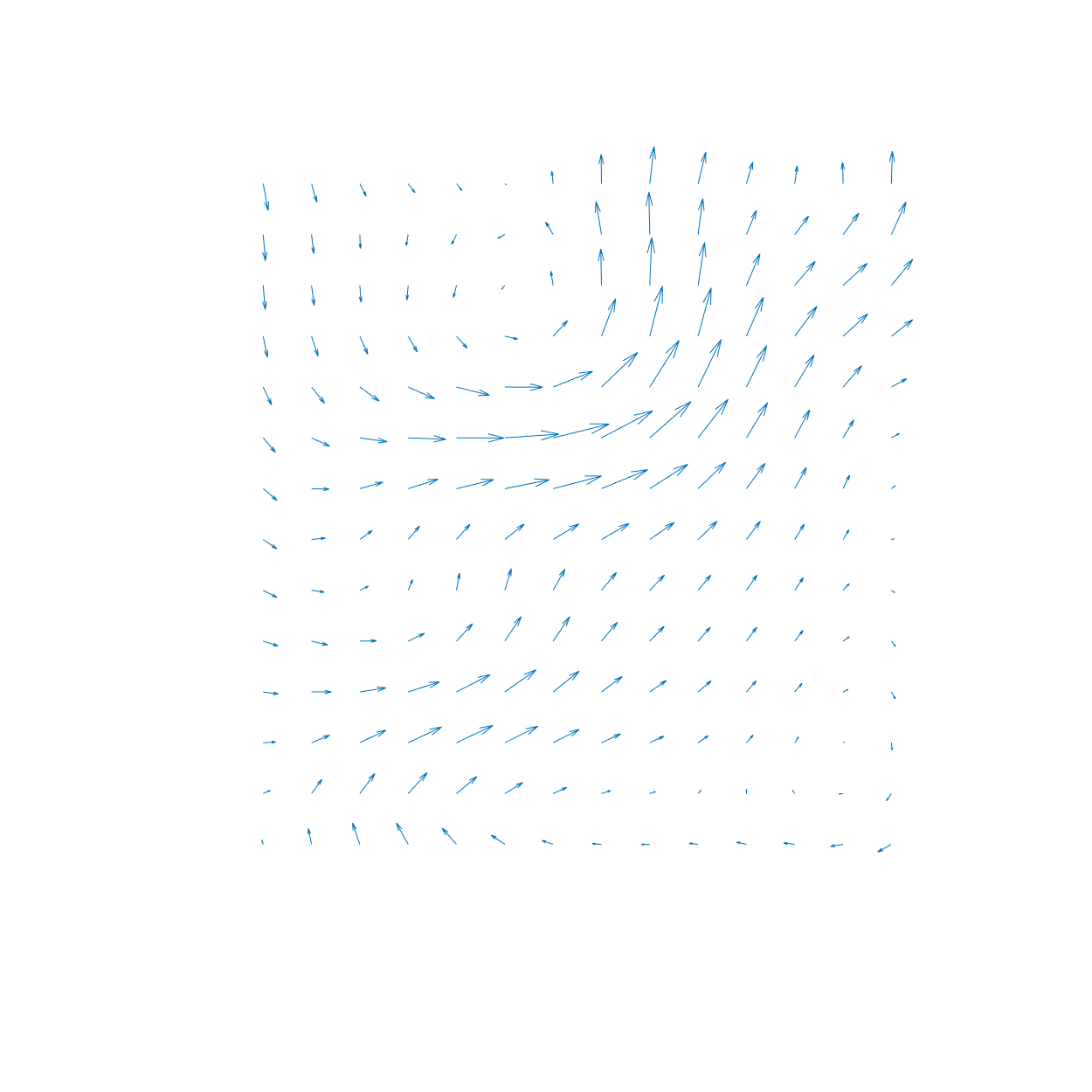

Supplement: S3 MCG raw data 3 — The raw MCG dataset includes category 4 for training and validation. (ZIP) [file pone.0338189.s003.zip › train/4/p11_220_2.png]

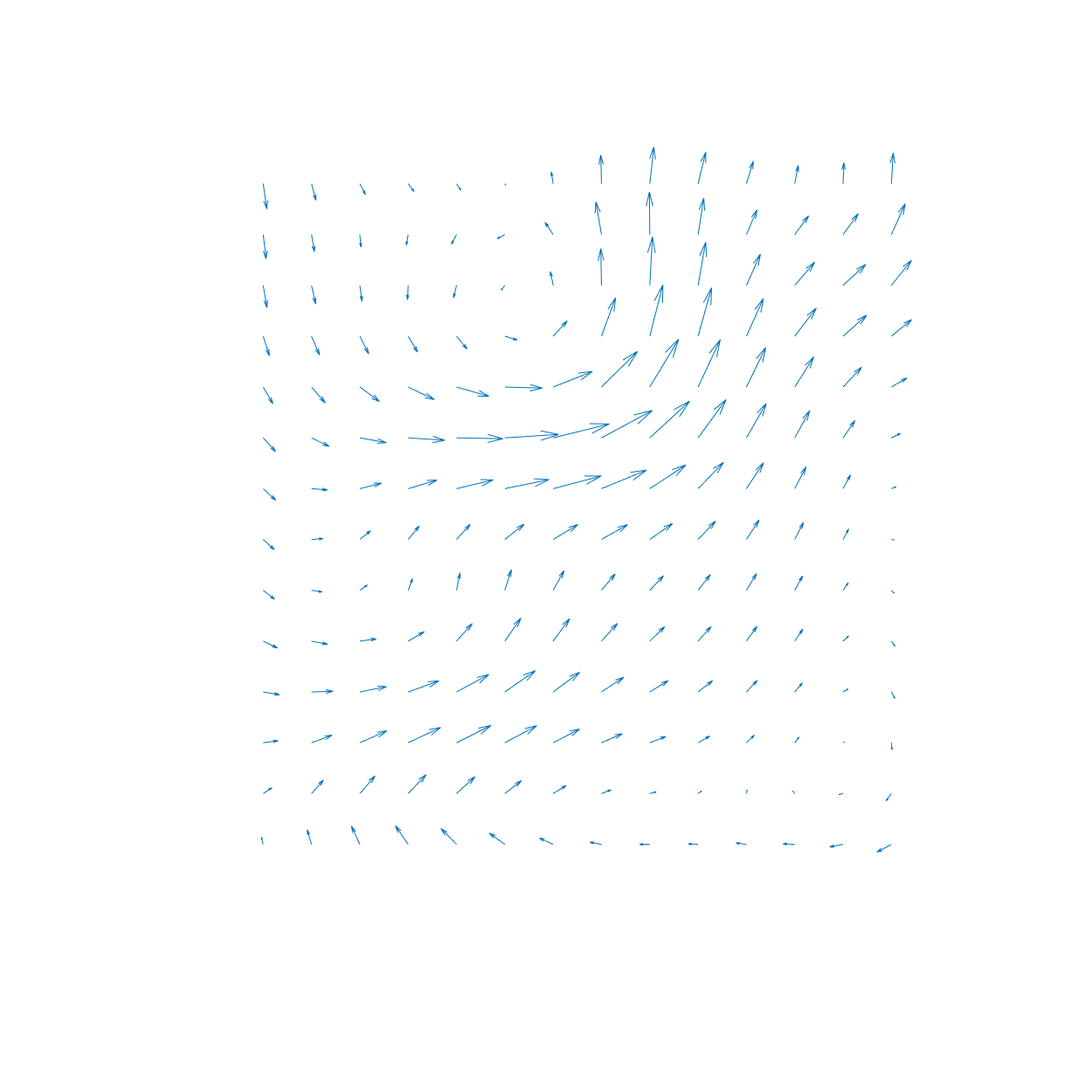

Supplement: S3 MCG raw data 3 — The raw MCG dataset includes category 4 for training and validation. (ZIP) [file pone.0338189.s003.zip › train/4/p11_220_3.png]

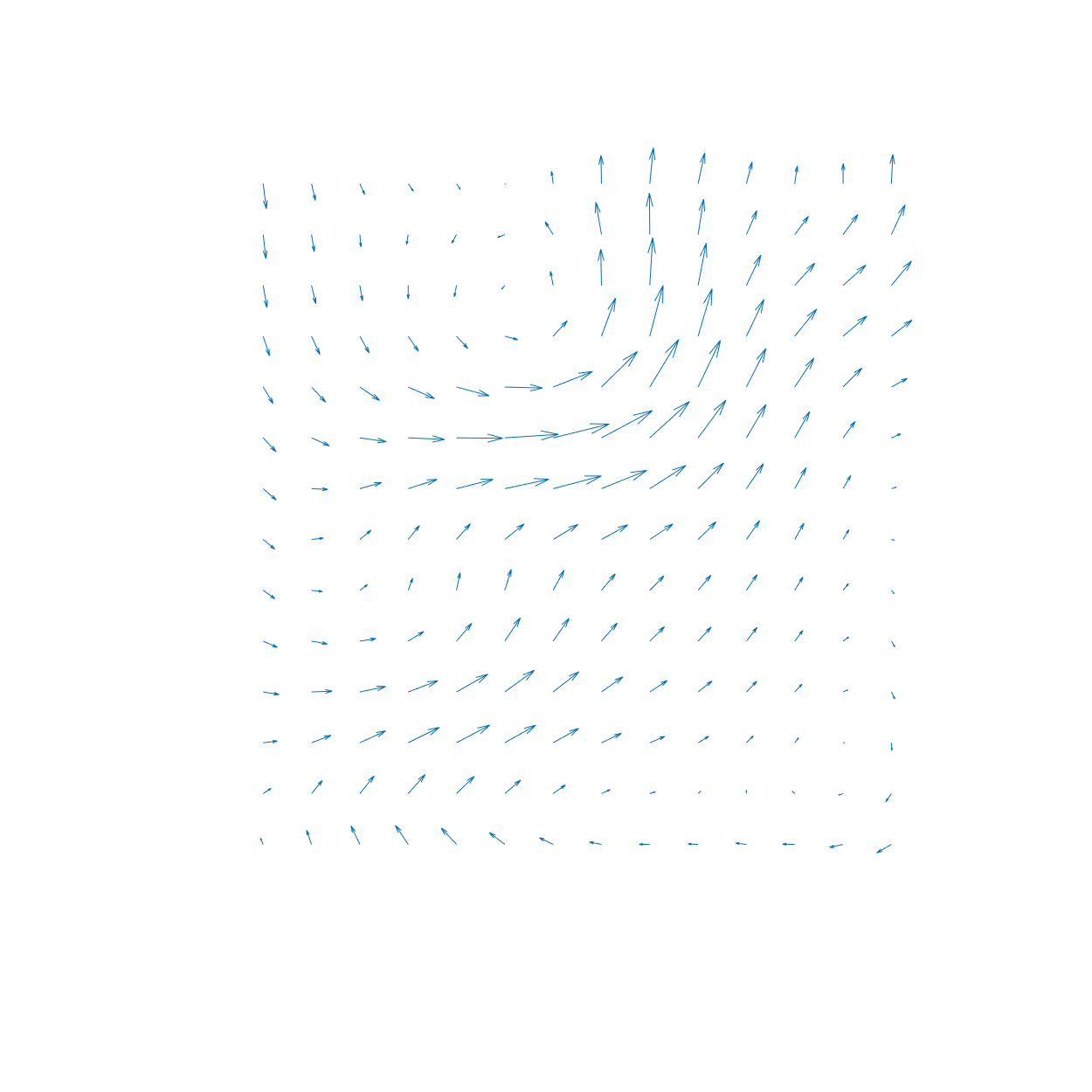

Supplement: S3 MCG raw data 3 — The raw MCG dataset includes category 4 for training and validation. (ZIP) [file pone.0338189.s003.zip › train/4/p11_220_4.png]

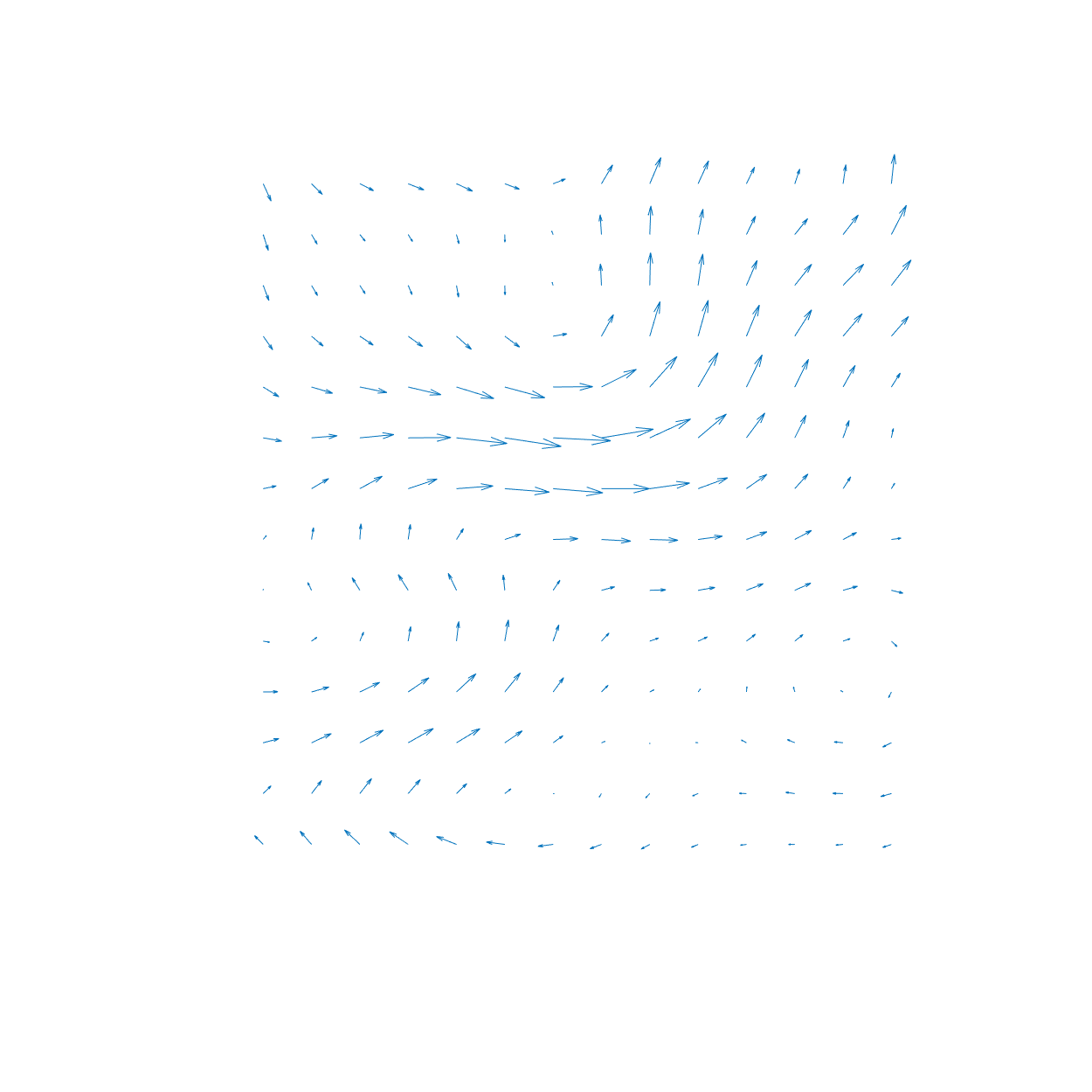

Supplement: S3 MCG raw data 3 — The raw MCG dataset includes category 4 for training and validation. (ZIP) [file pone.0338189.s003.zip › train/4/p11_225_1.png]

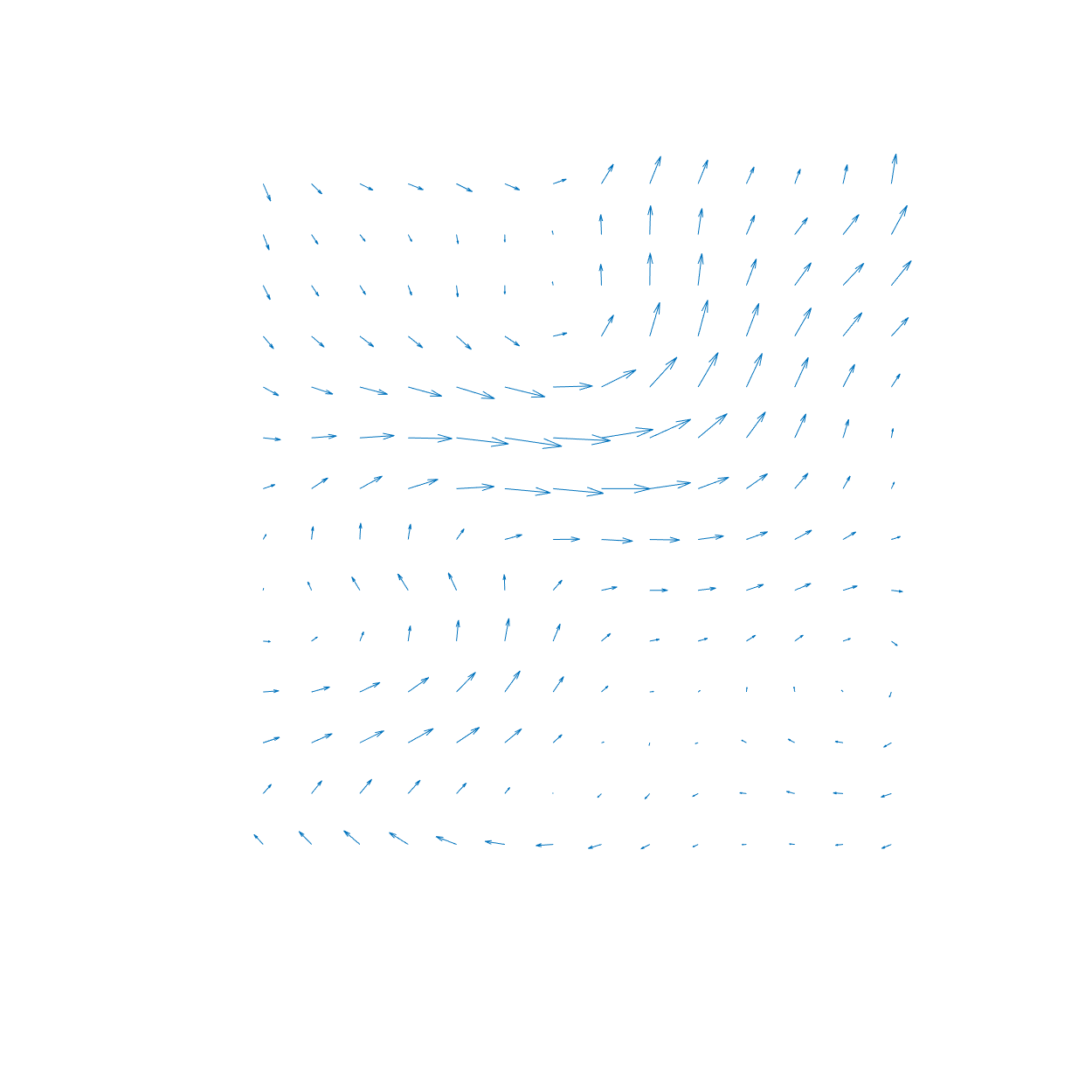

Supplement: S3 MCG raw data 3 — The raw MCG dataset includes category 4 for training and validation. (ZIP) [file pone.0338189.s003.zip › train/4/p11_225_2.png]

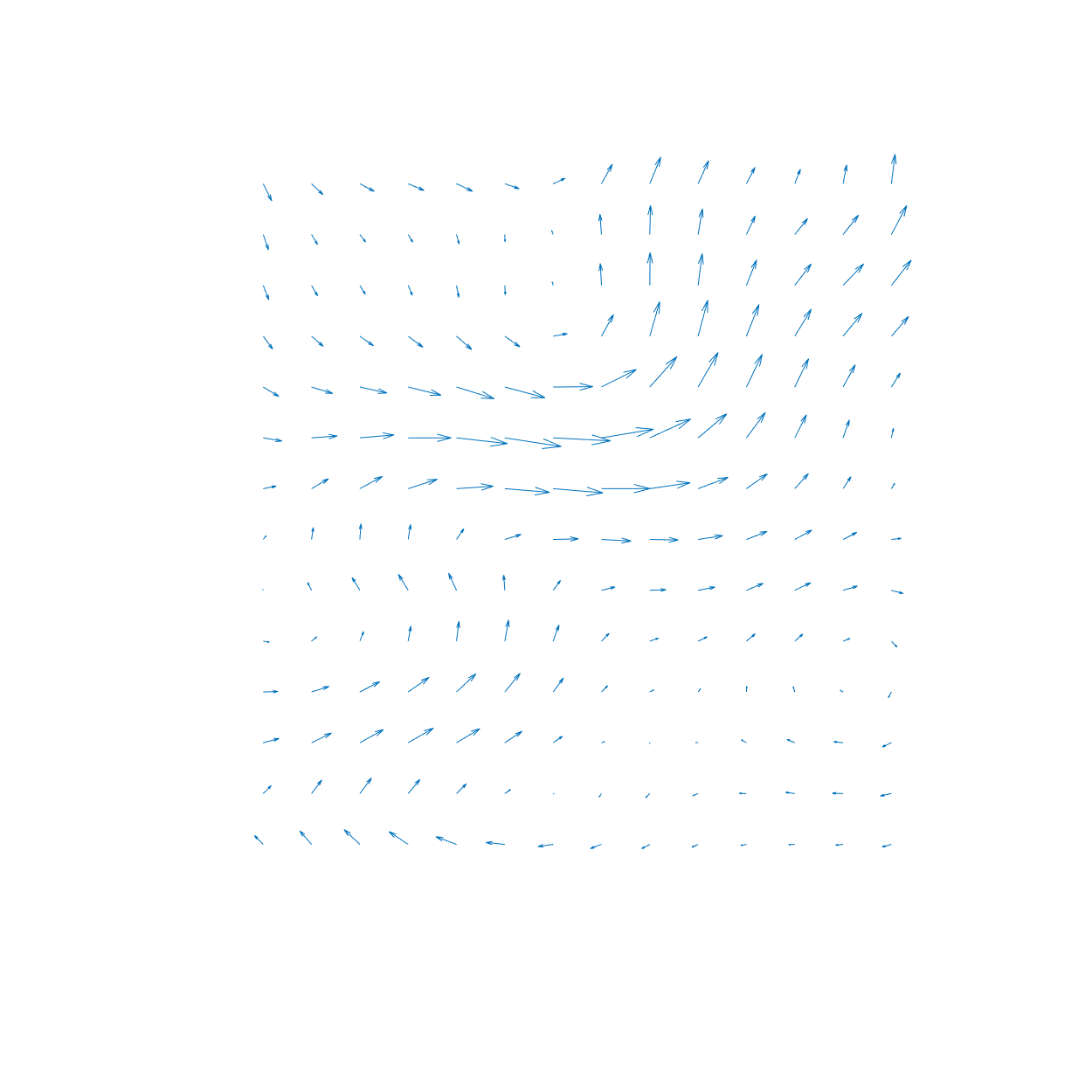

Supplement: S3 MCG raw data 3 — The raw MCG dataset includes category 4 for training and validation. (ZIP) [file pone.0338189.s003.zip › train/4/p11_225_3.png]

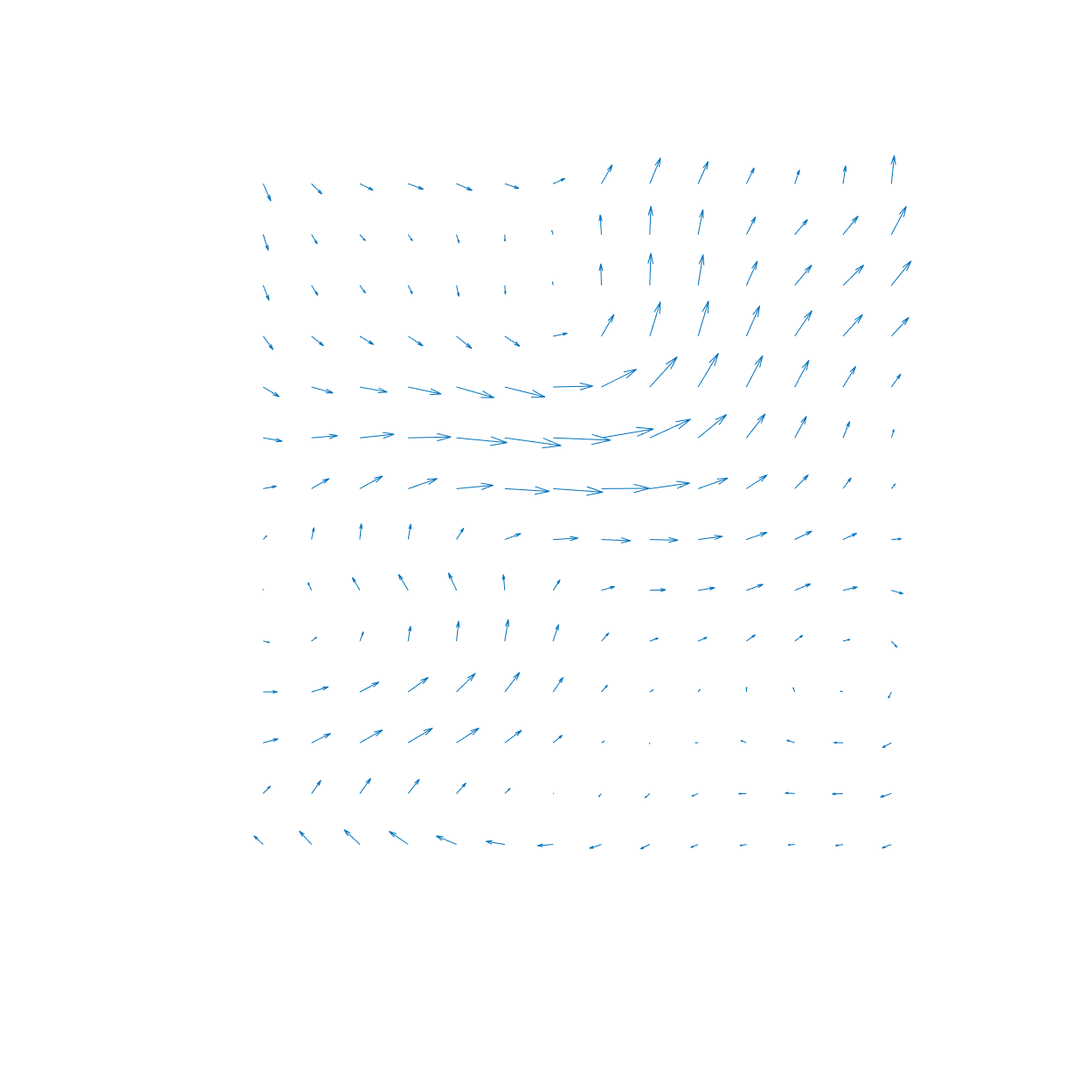

Supplement: S3 MCG raw data 3 — The raw MCG dataset includes category 4 for training and validation. (ZIP) [file pone.0338189.s003.zip › train/4/p11_225_4.png]

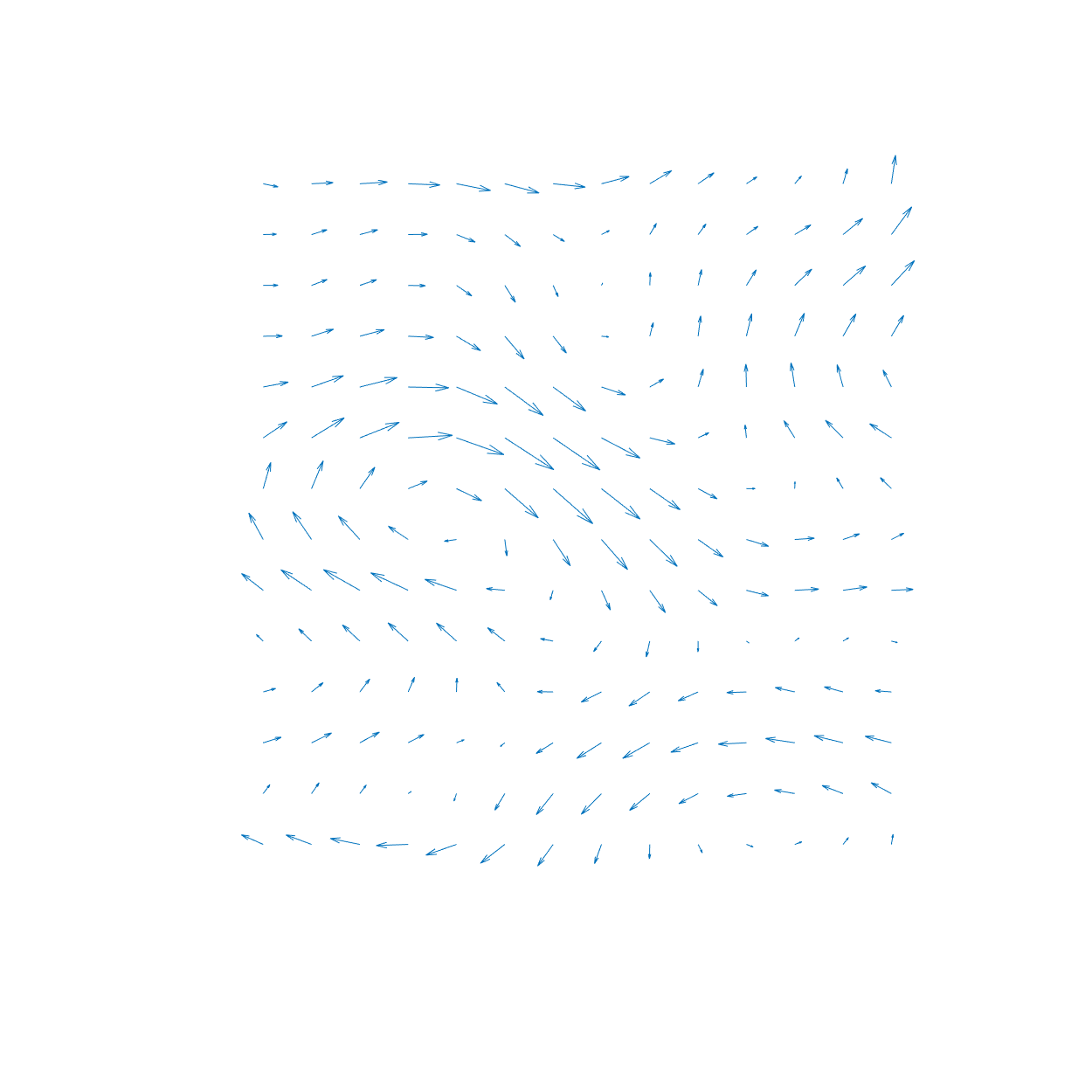

Supplement: S3 MCG raw data 3 — The raw MCG dataset includes category 4 for training and validation. (ZIP) [file pone.0338189.s003.zip › train/4/p11_230_1.png]

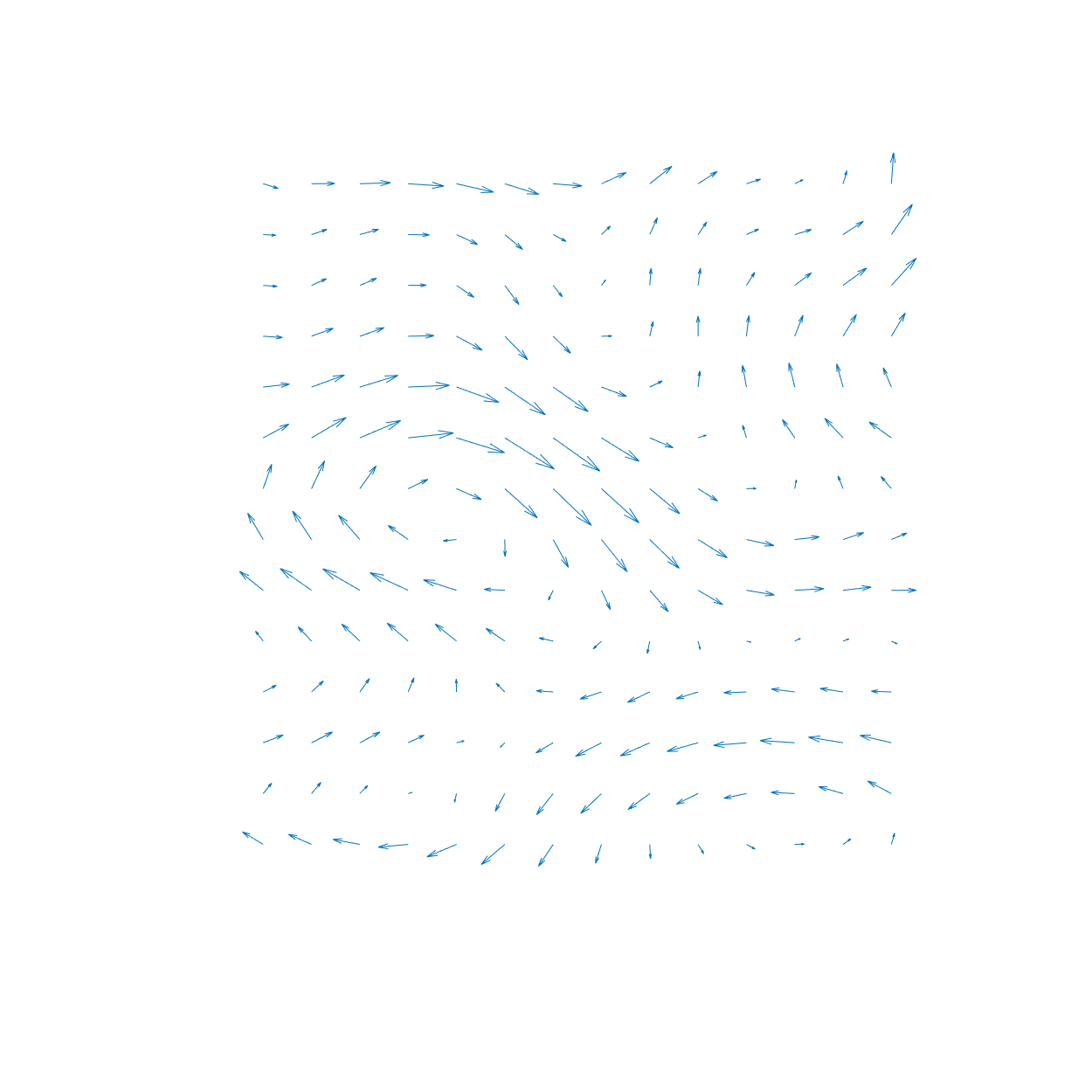

Supplement: S3 MCG raw data 3 — The raw MCG dataset includes category 4 for training and validation. (ZIP) [file pone.0338189.s003.zip › train/4/p11_230_2.png]

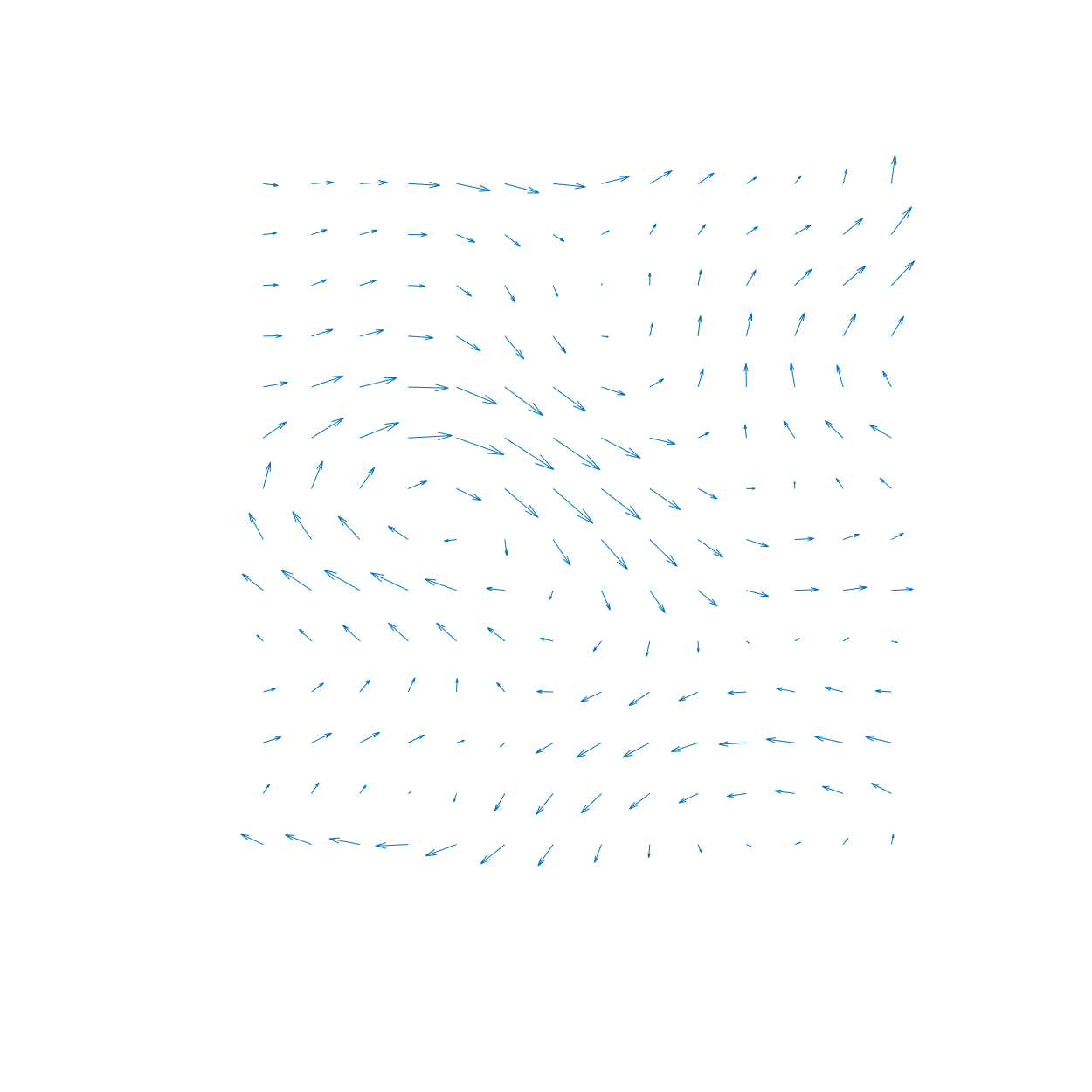

Supplement: S3 MCG raw data 3 — The raw MCG dataset includes category 4 for training and validation. (ZIP) [file pone.0338189.s003.zip › train/4/p11_230_3.png]

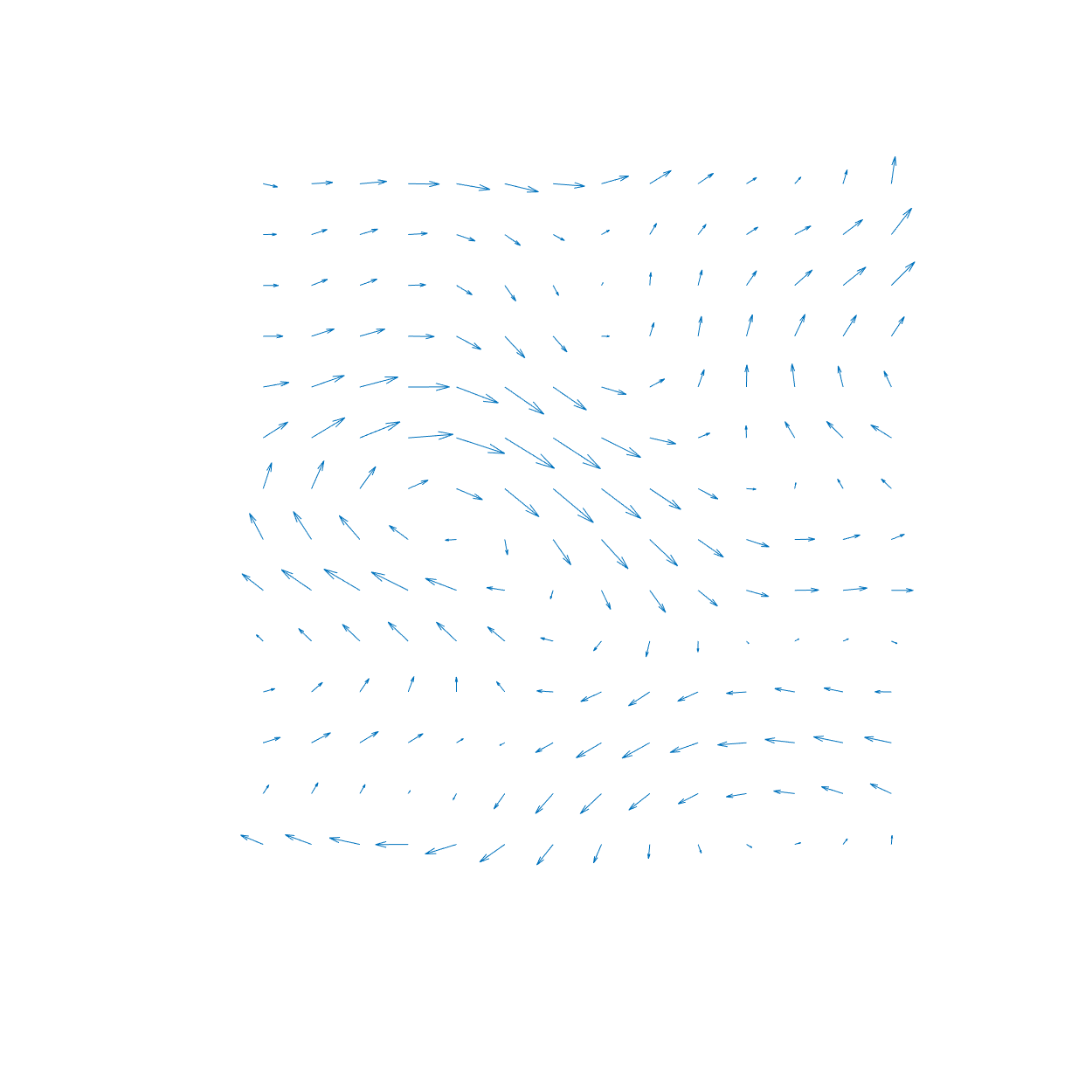

Supplement: S3 MCG raw data 3 — The raw MCG dataset includes category 4 for training and validation. (ZIP) [file pone.0338189.s003.zip › train/4/p11_230_4.png]

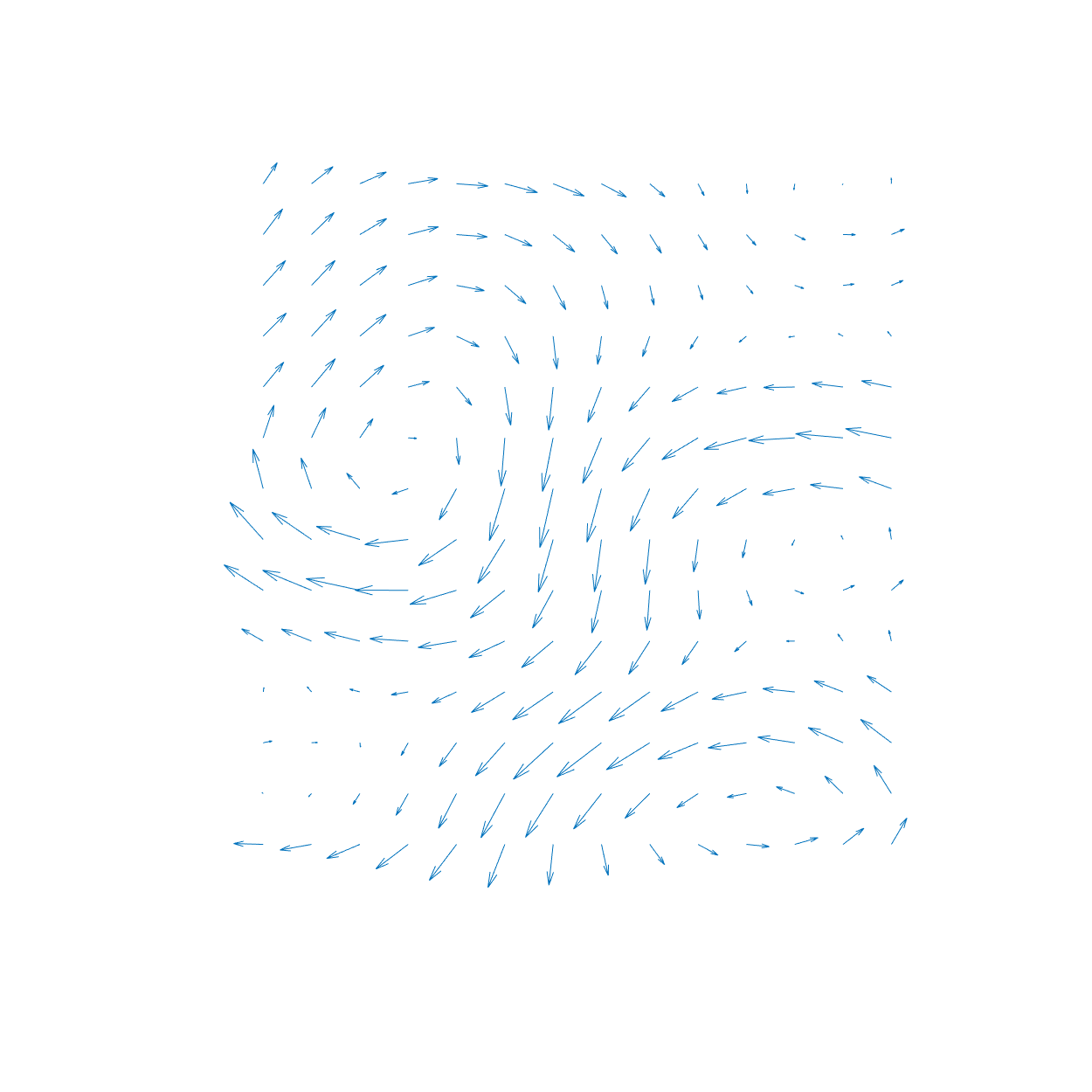

Supplement: S3 MCG raw data 3 — The raw MCG dataset includes category 4 for training and validation. (ZIP) [file pone.0338189.s003.zip › train/4/p11_235_1.png]

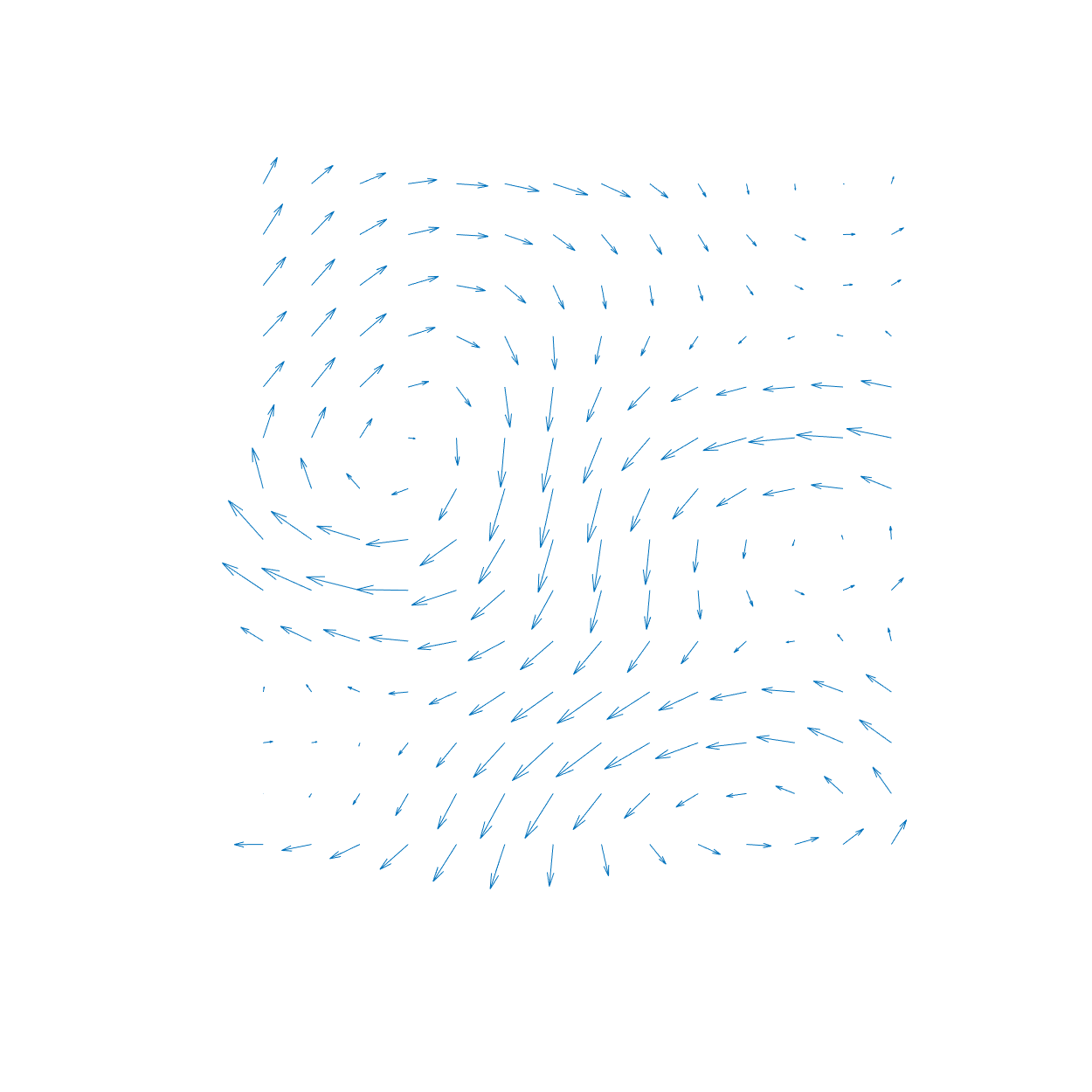

Supplement: S3 MCG raw data 3 — The raw MCG dataset includes category 4 for training and validation. (ZIP) [file pone.0338189.s003.zip › train/4/p11_235_2.png]

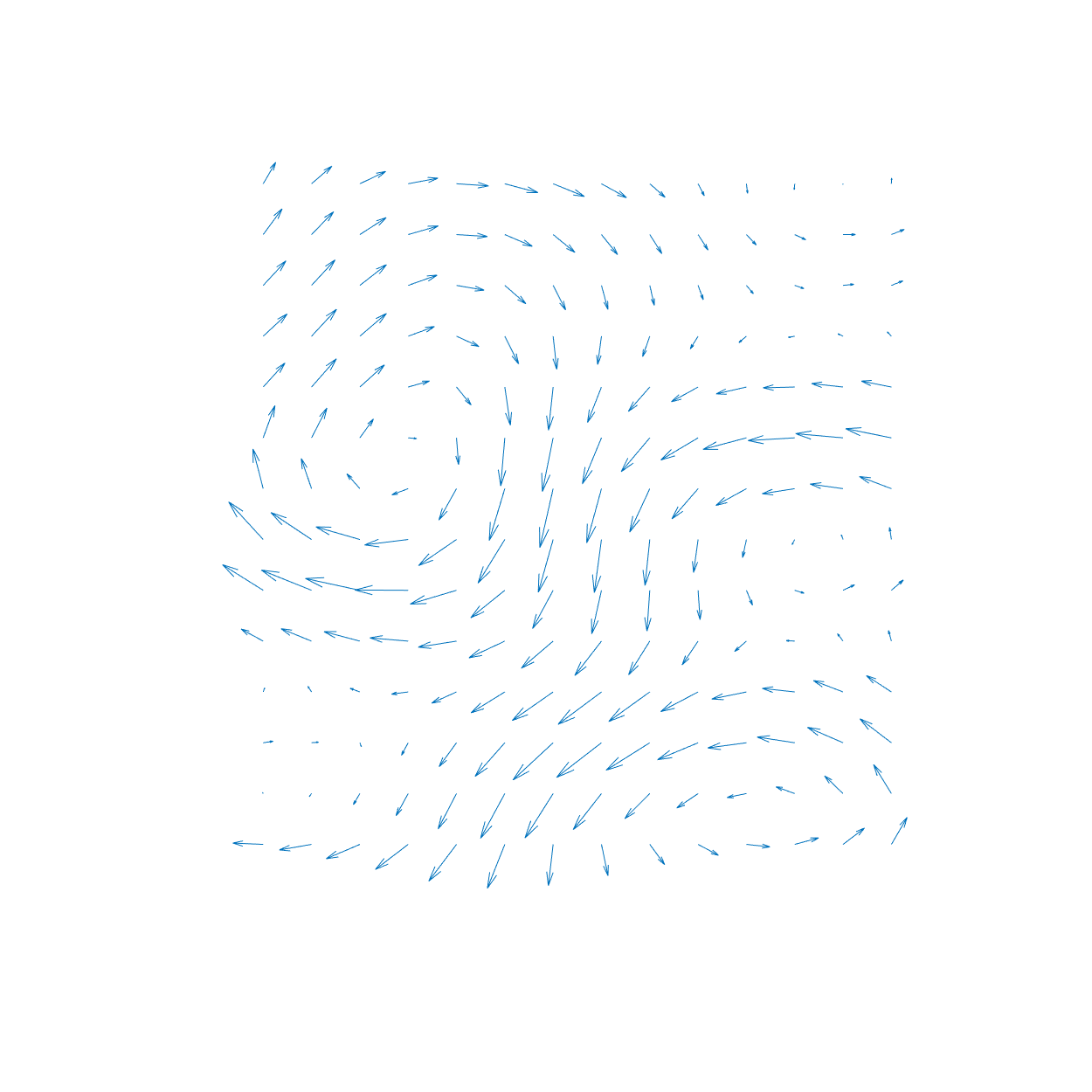

Supplement: S3 MCG raw data 3 — The raw MCG dataset includes category 4 for training and validation. (ZIP) [file pone.0338189.s003.zip › train/4/p11_235_3.png]

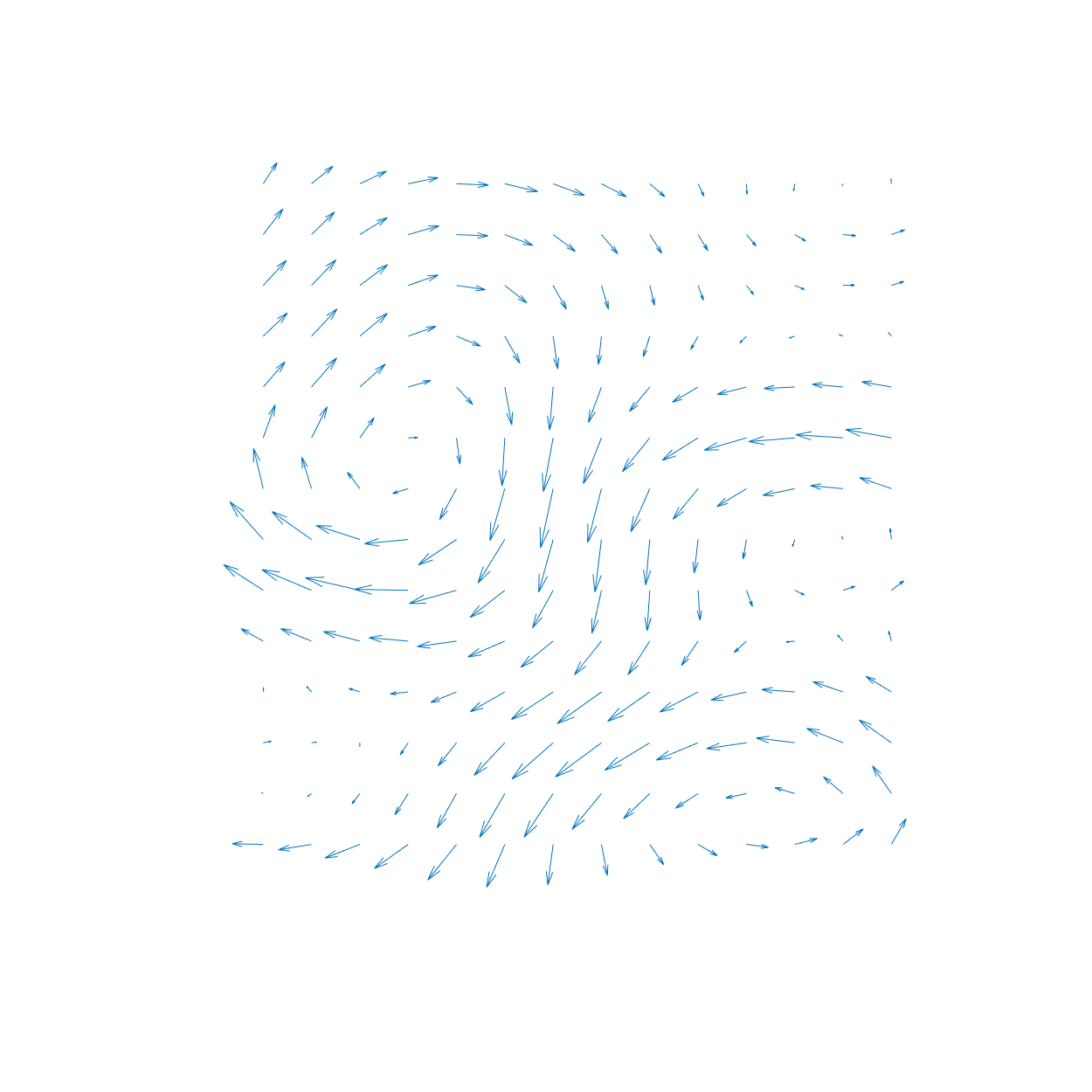

Supplement: S3 MCG raw data 3 — The raw MCG dataset includes category 4 for training and validation. (ZIP) [file pone.0338189.s003.zip › train/4/p11_235_4.png]

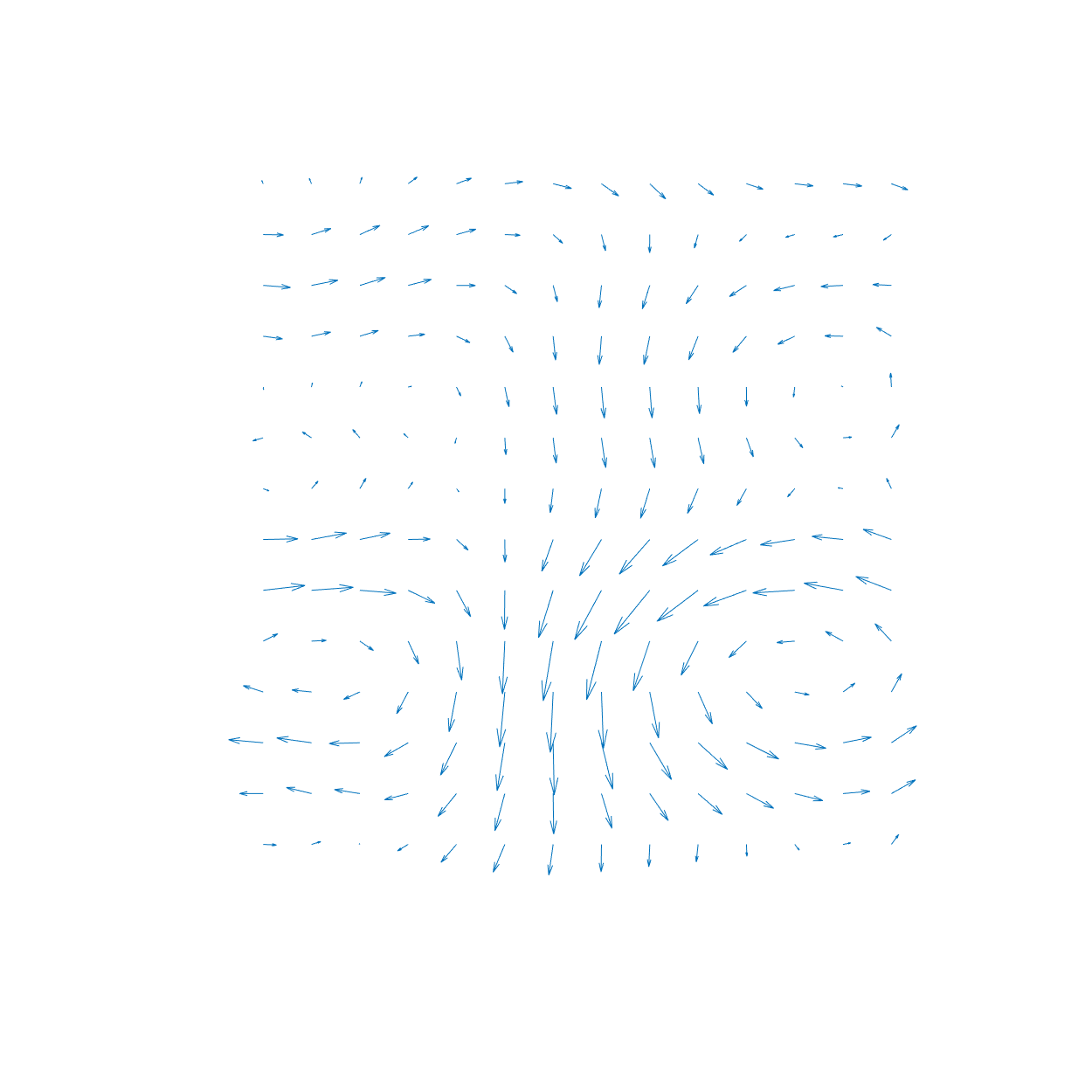

Supplement: S3 MCG raw data 3 — The raw MCG dataset includes category 4 for training and validation. (ZIP) [file pone.0338189.s003.zip › train/4/p11_270_1.png]

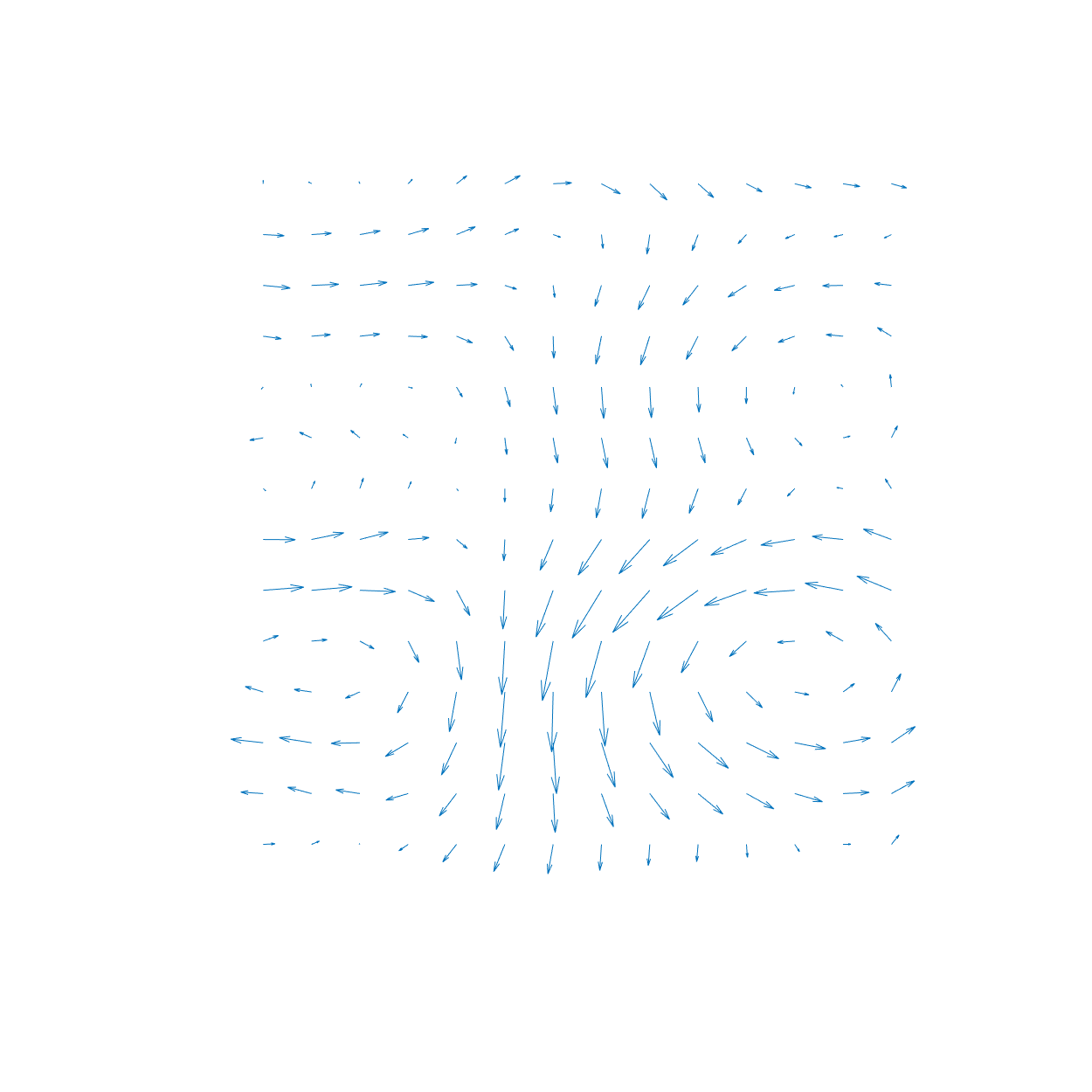

Supplement: S3 MCG raw data 3 — The raw MCG dataset includes category 4 for training and validation. (ZIP) [file pone.0338189.s003.zip › train/4/p11_270_2.png]

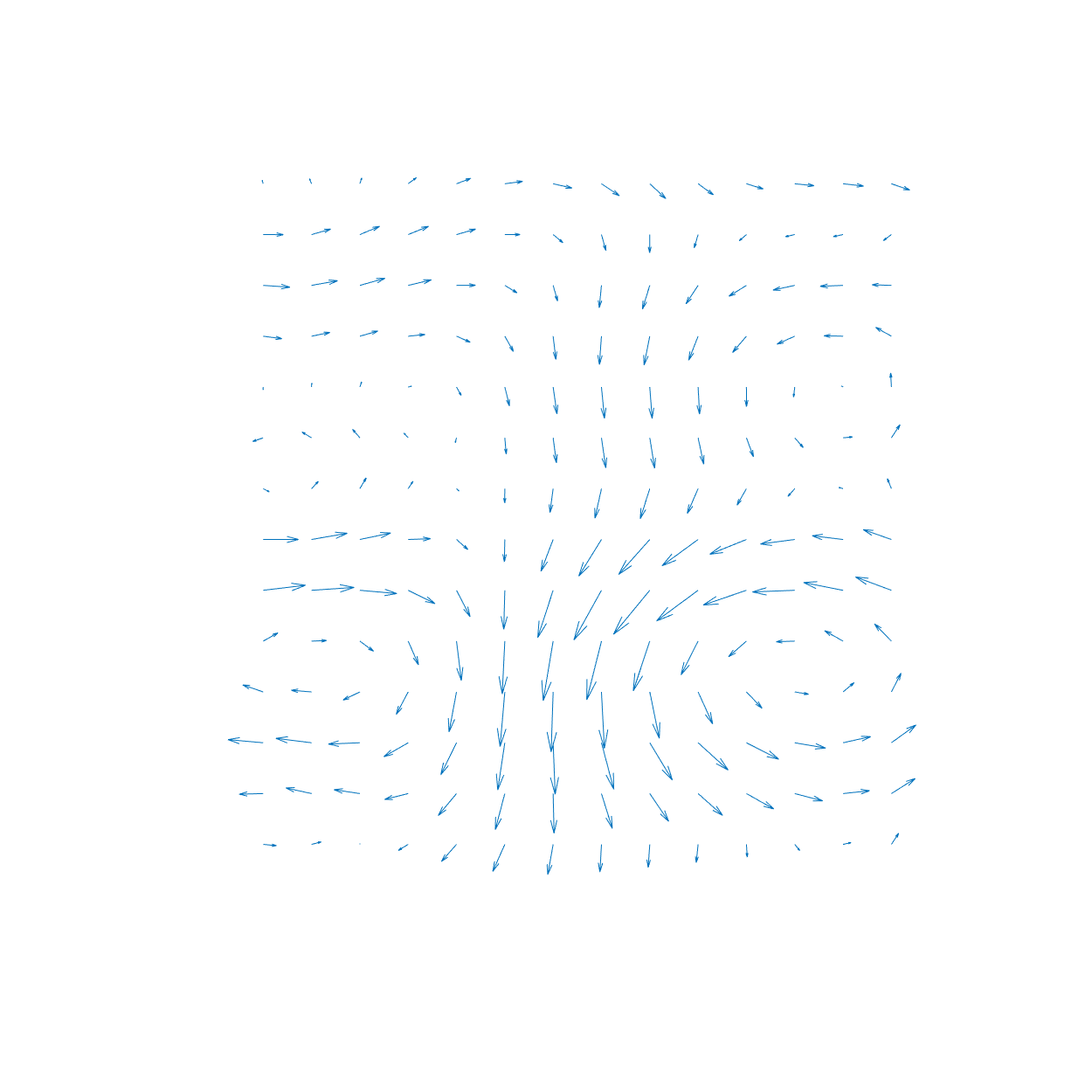

Supplement: S3 MCG raw data 3 — The raw MCG dataset includes category 4 for training and validation. (ZIP) [file pone.0338189.s003.zip › train/4/p11_270_3.png]

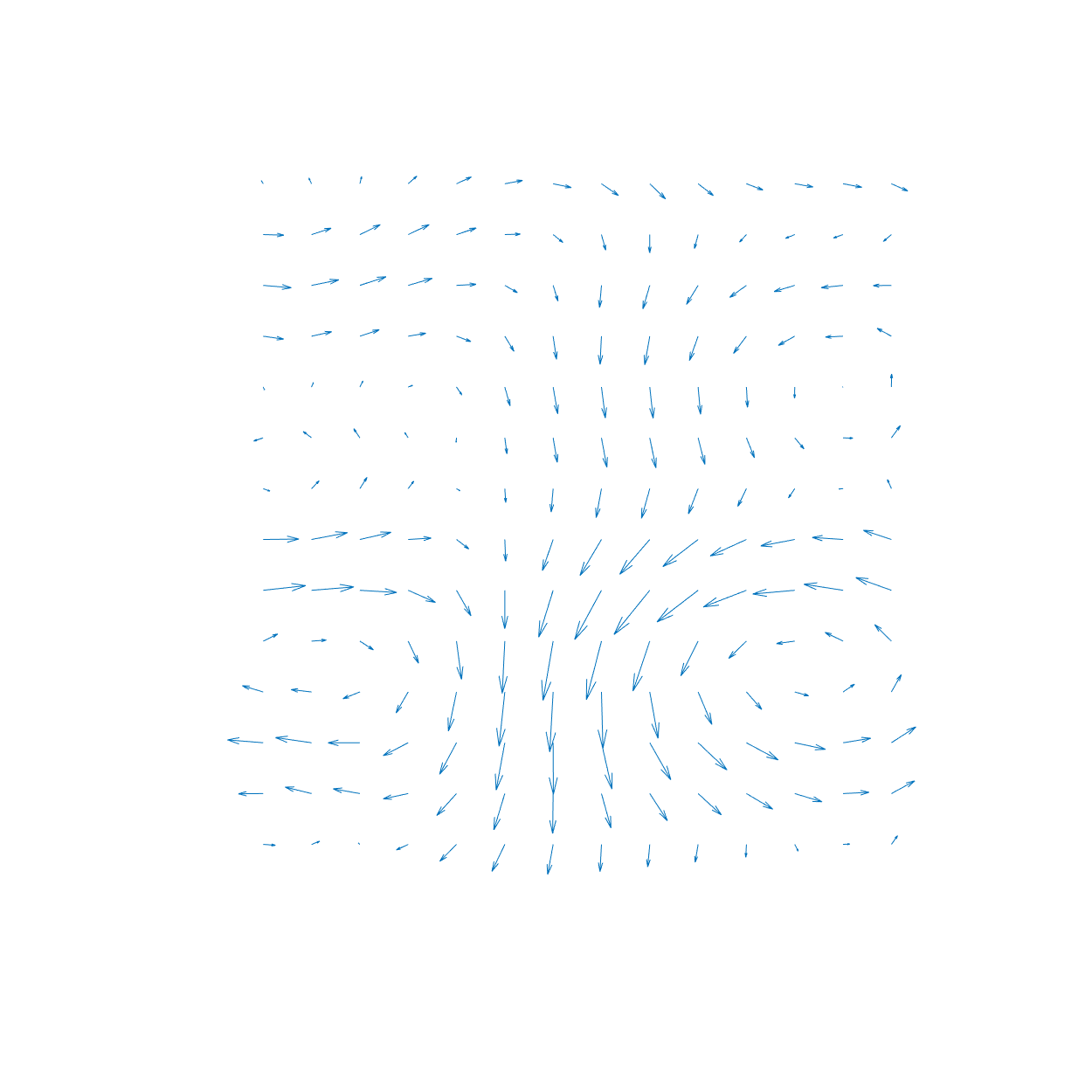

Supplement: S3 MCG raw data 3 — The raw MCG dataset includes category 4 for training and validation. (ZIP) [file pone.0338189.s003.zip › train/4/p11_270_4.png]

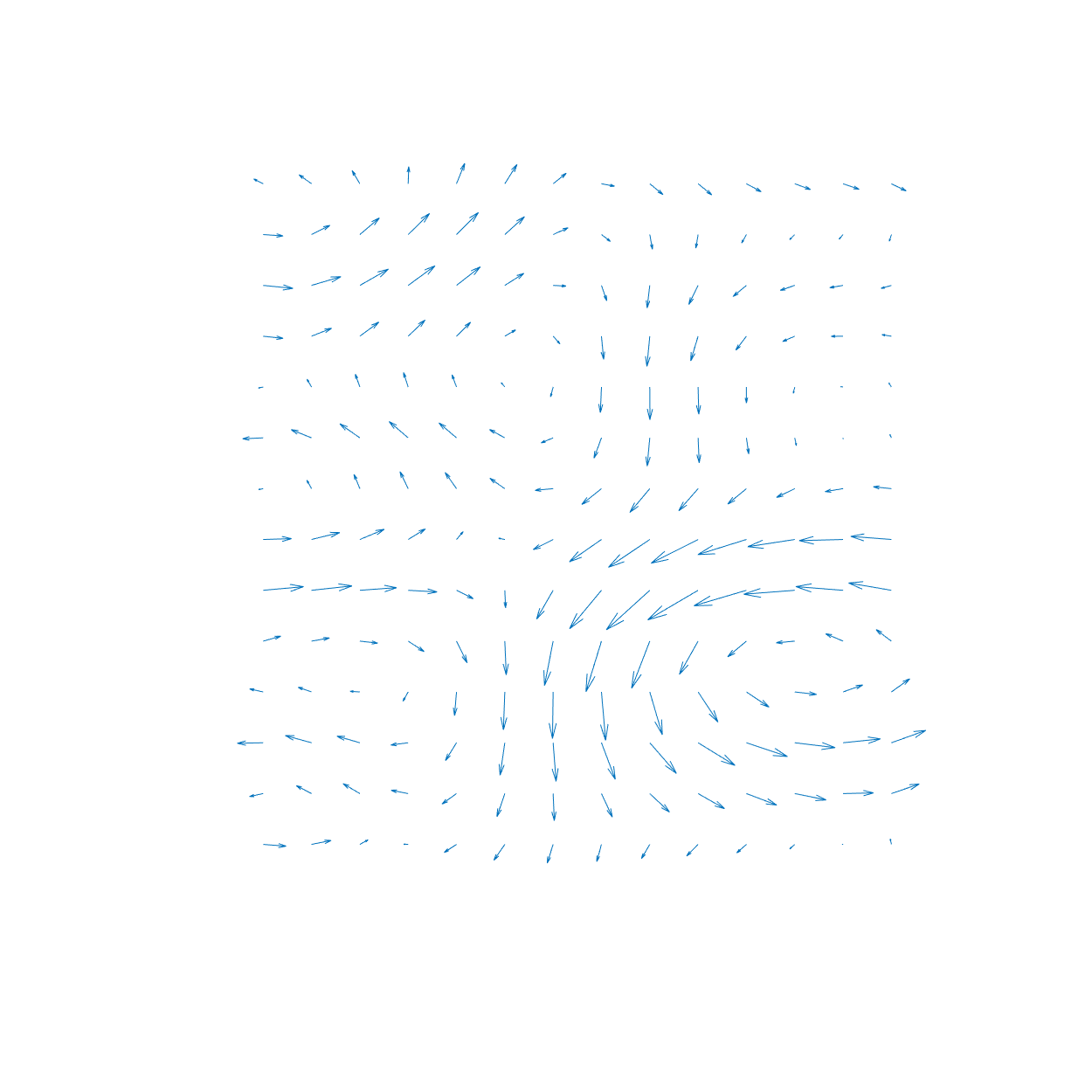

Supplement: S3 MCG raw data 3 — The raw MCG dataset includes category 4 for training and validation. (ZIP) [file pone.0338189.s003.zip › train/4/p11_275_1.png]

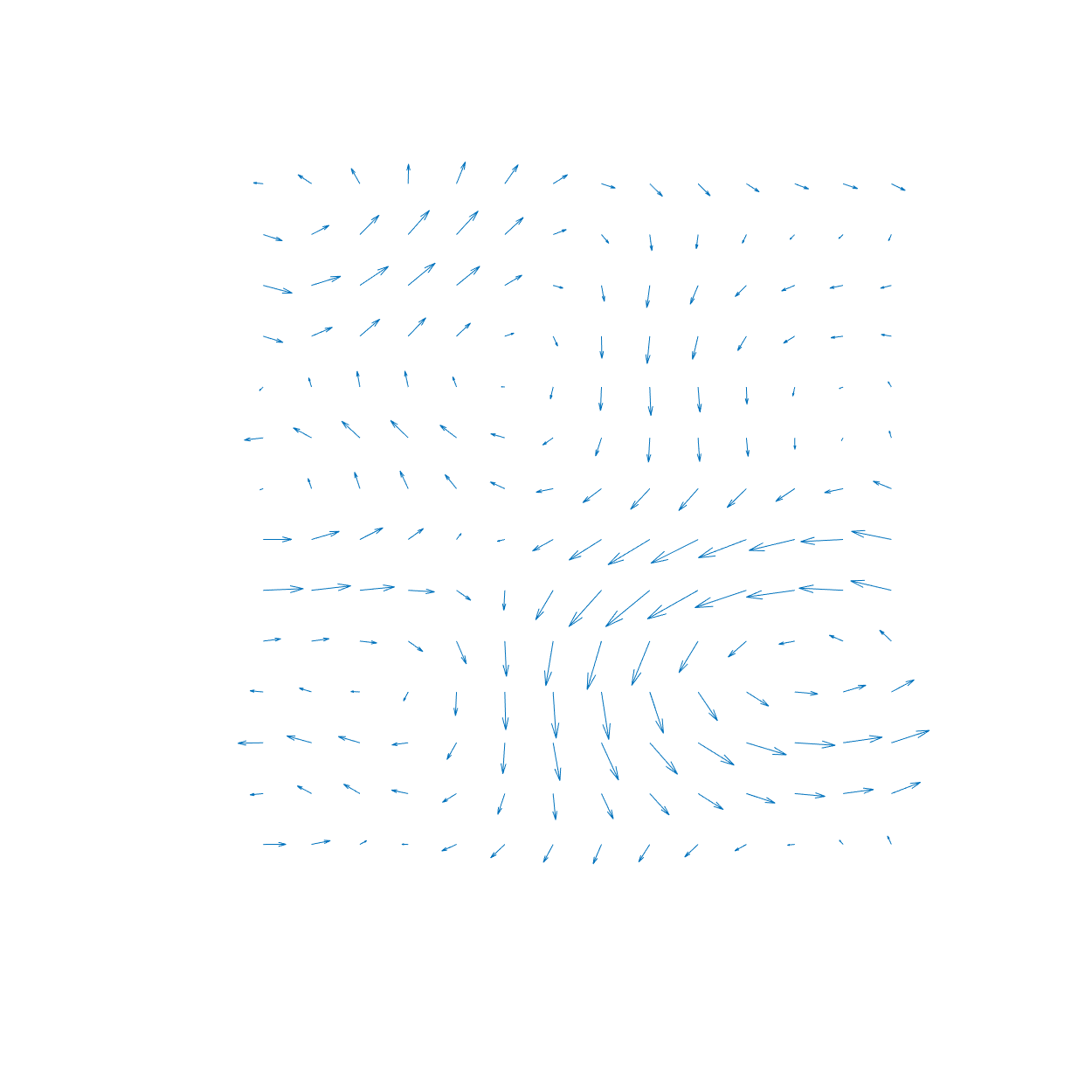

Supplement: S3 MCG raw data 3 — The raw MCG dataset includes category 4 for training and validation. (ZIP) [file pone.0338189.s003.zip › train/4/p11_275_2.png]

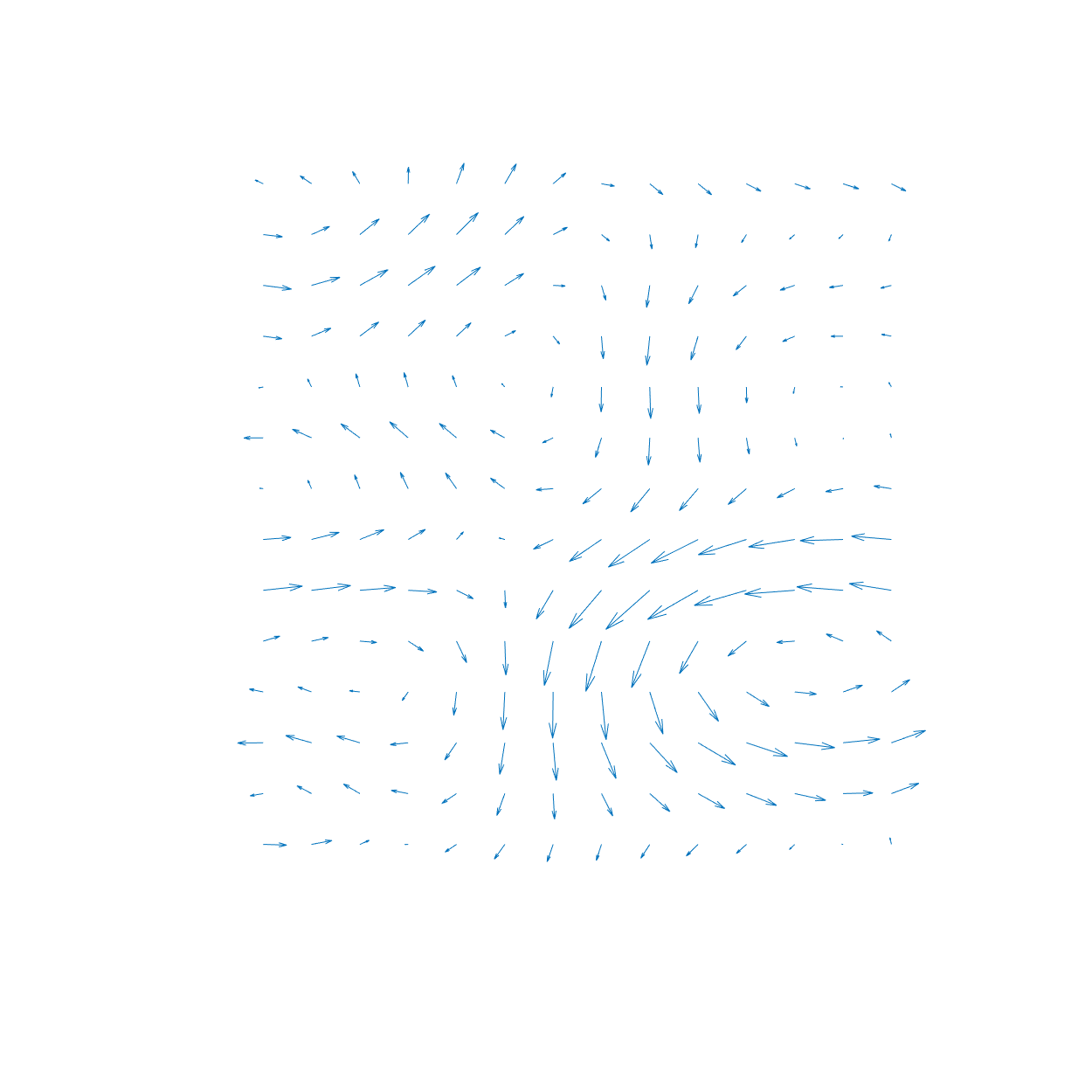

Supplement: S3 MCG raw data 3 — The raw MCG dataset includes category 4 for training and validation. (ZIP) [file pone.0338189.s003.zip › train/4/p11_275_3.png]

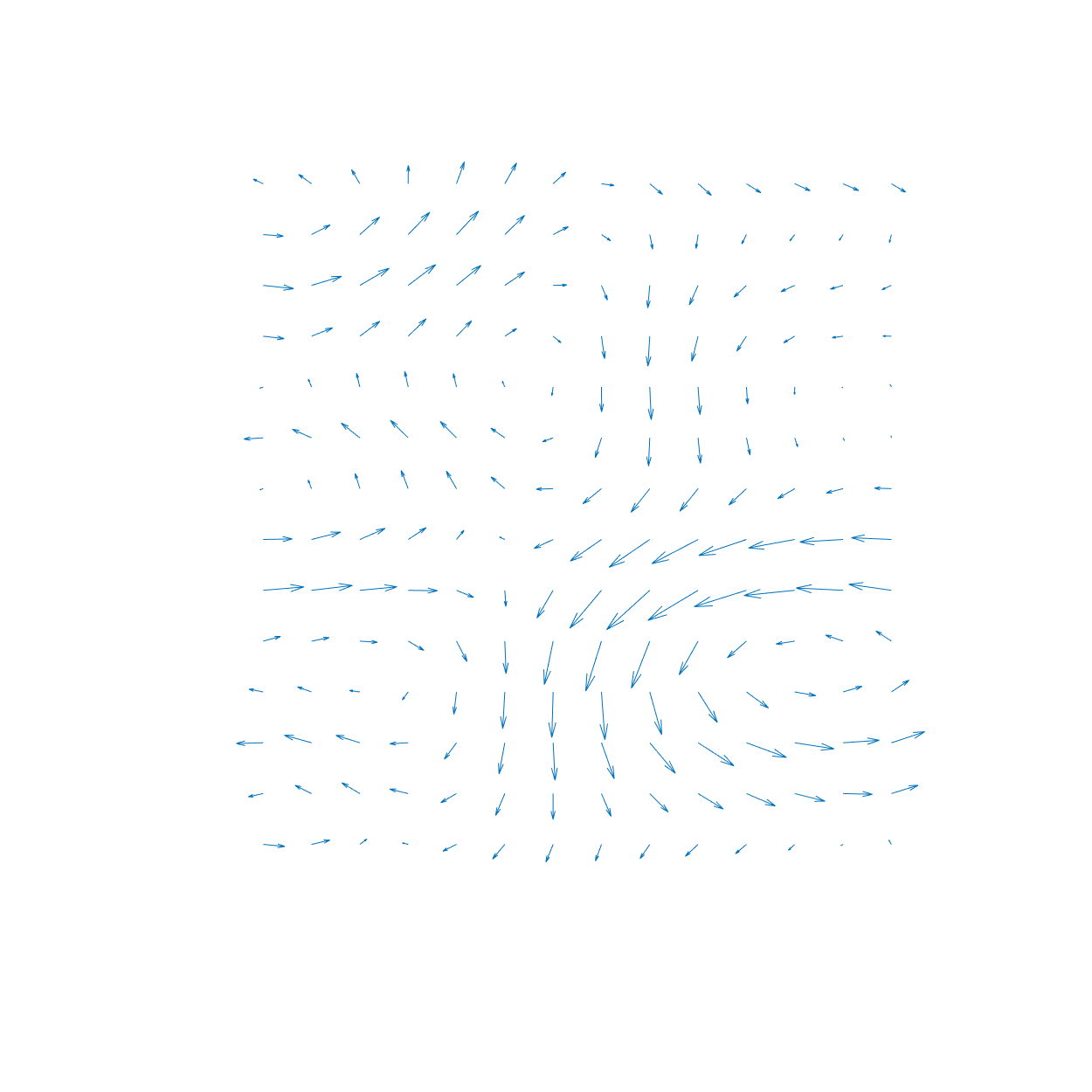

Supplement: S3 MCG raw data 3 — The raw MCG dataset includes category 4 for training and validation. (ZIP) [file pone.0338189.s003.zip › train/4/p11_275_4.png]

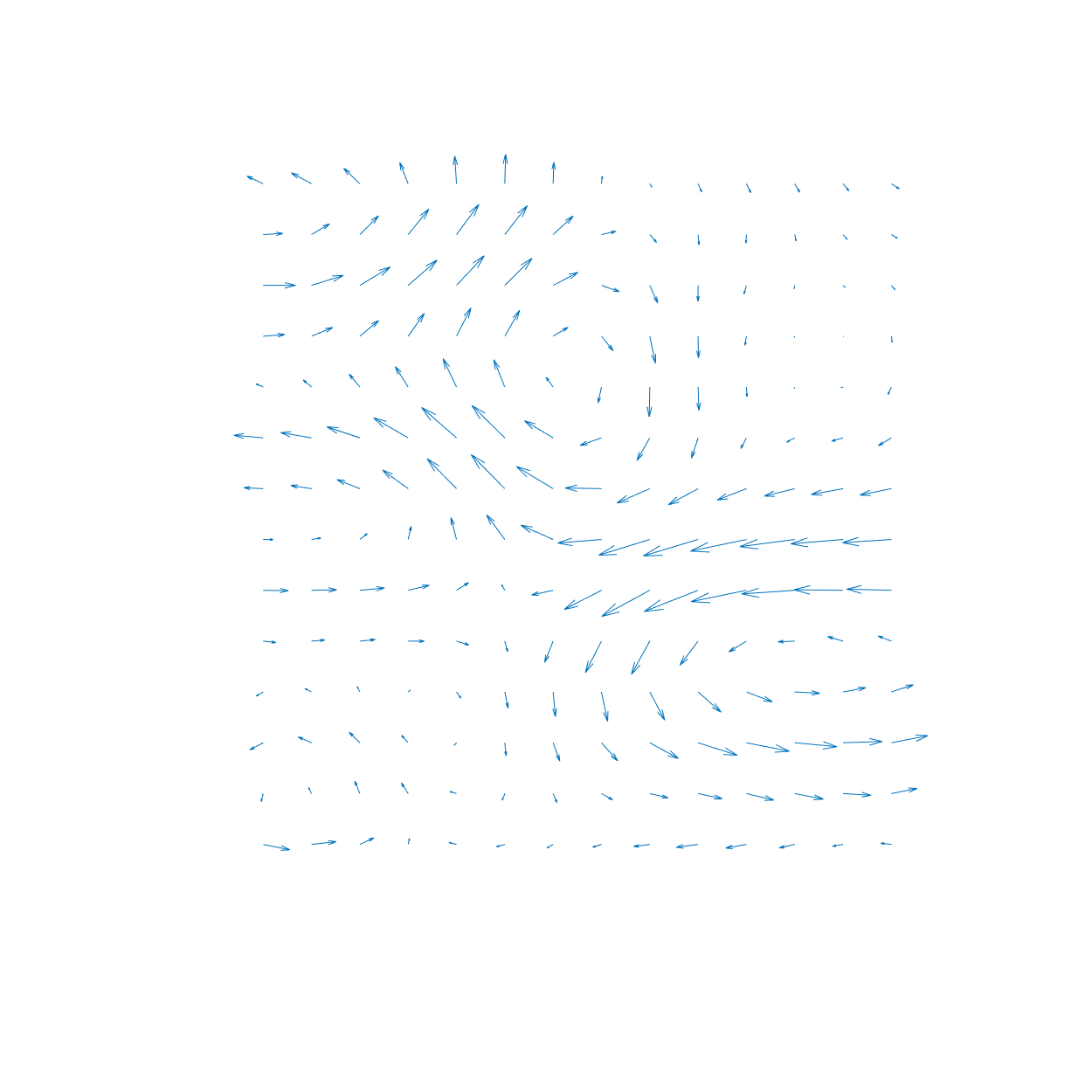

Supplement: S3 MCG raw data 3 — The raw MCG dataset includes category 4 for training and validation. (ZIP) [file pone.0338189.s003.zip › train/4/p11_280_1.png]

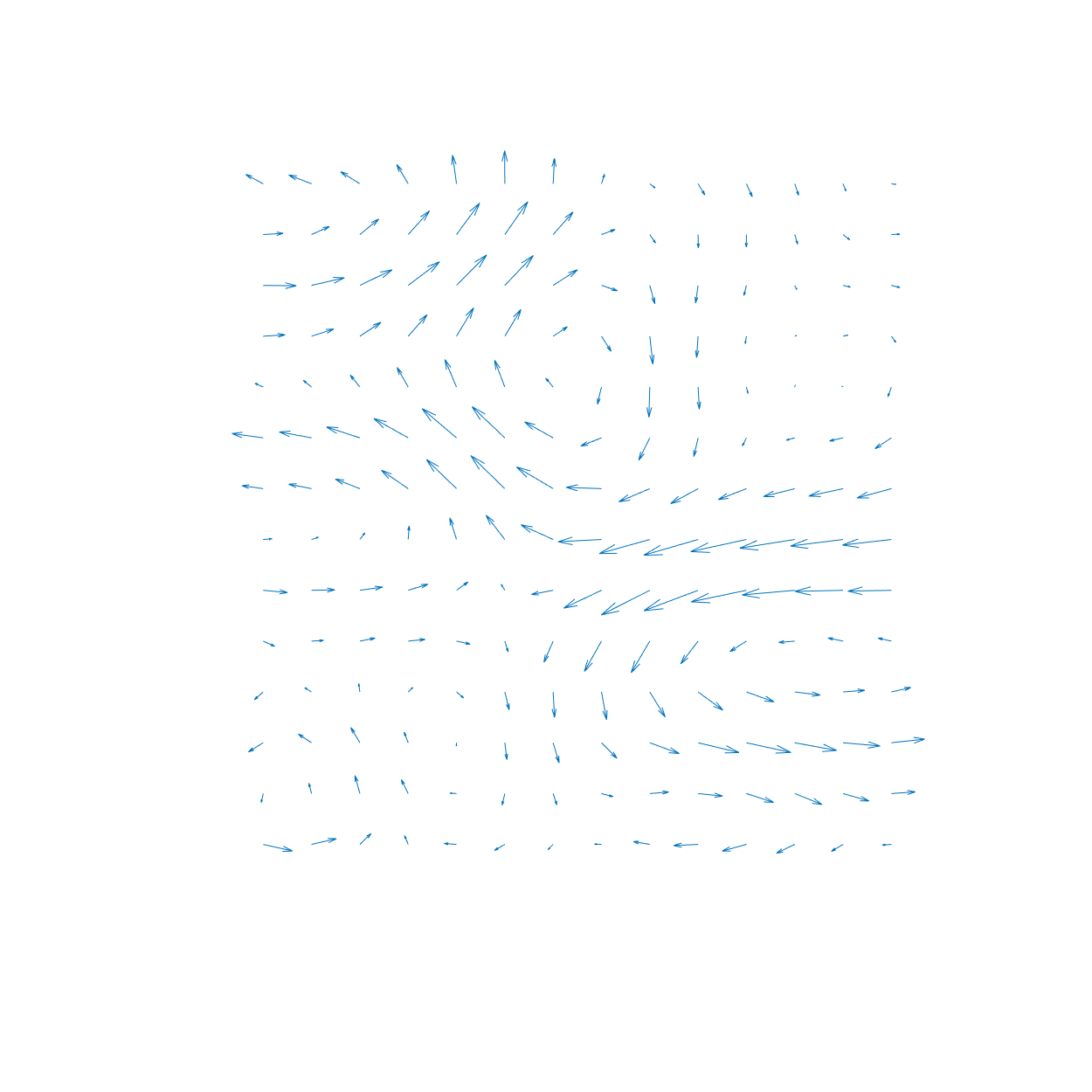

Supplement: S3 MCG raw data 3 — The raw MCG dataset includes category 4 for training and validation. (ZIP) [file pone.0338189.s003.zip › train/4/p11_280_2.png]

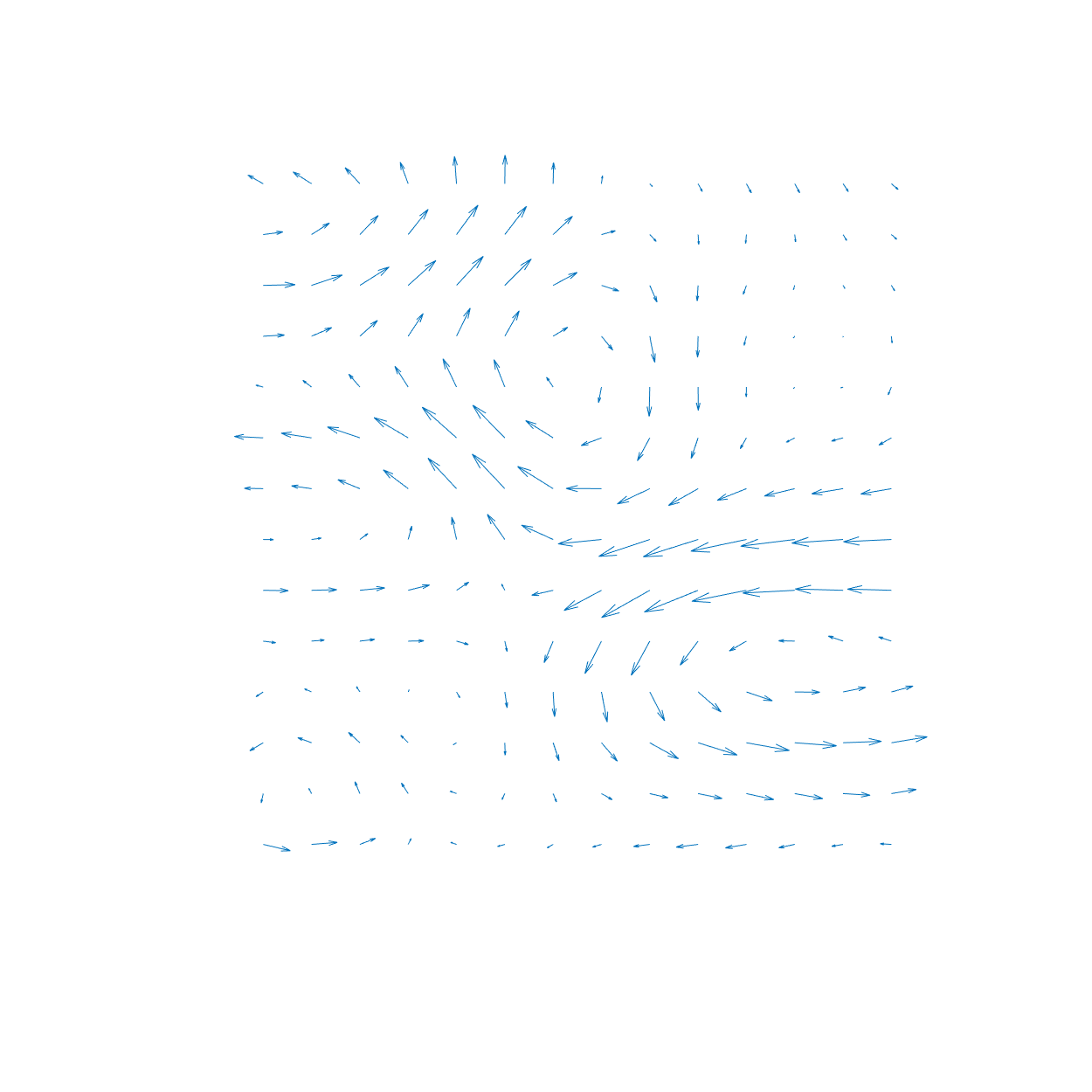

Supplement: S3 MCG raw data 3 — The raw MCG dataset includes category 4 for training and validation. (ZIP) [file pone.0338189.s003.zip › train/4/p11_280_3.png]

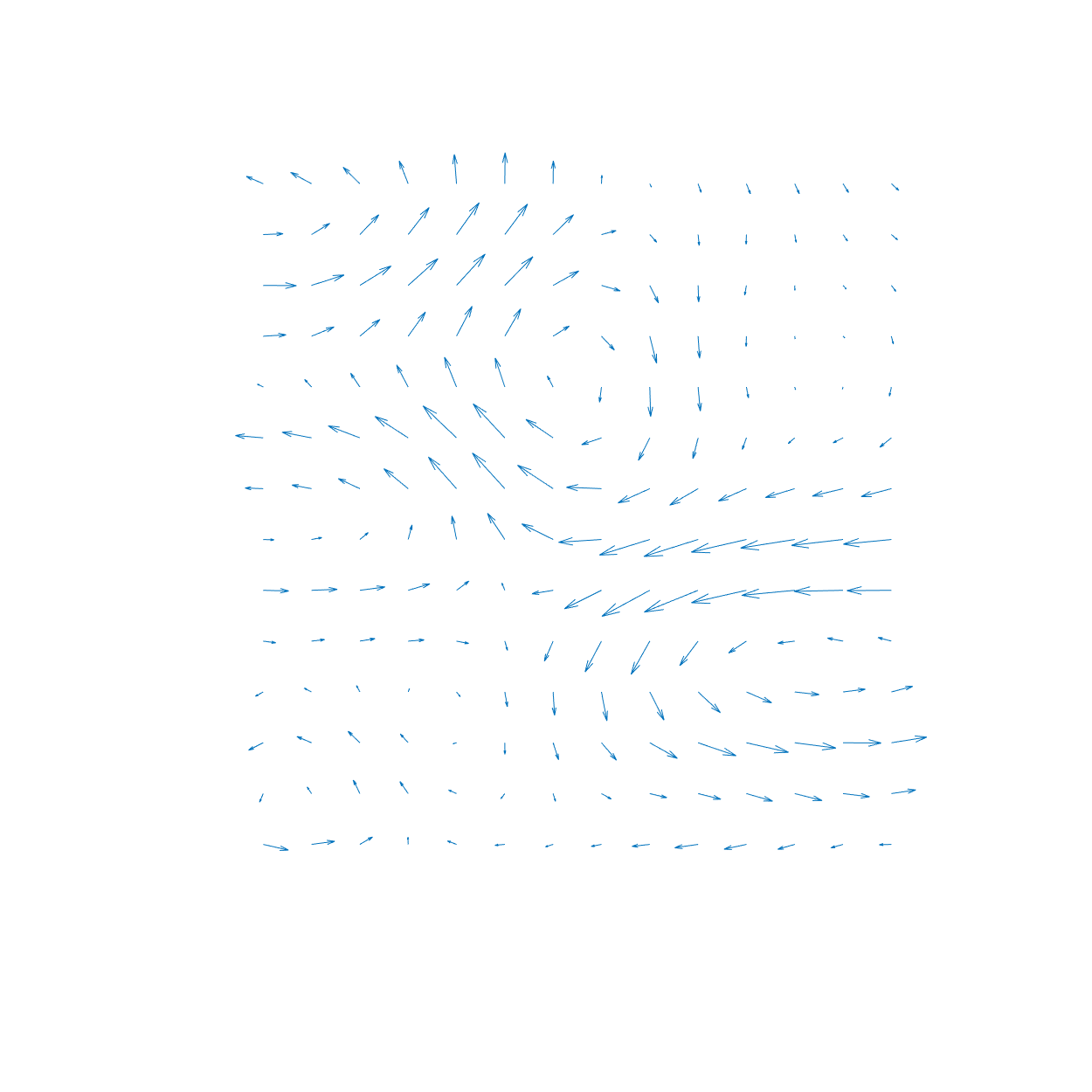

Supplement: S3 MCG raw data 3 — The raw MCG dataset includes category 4 for training and validation. (ZIP) [file pone.0338189.s003.zip › train/4/p11_280_4.png]

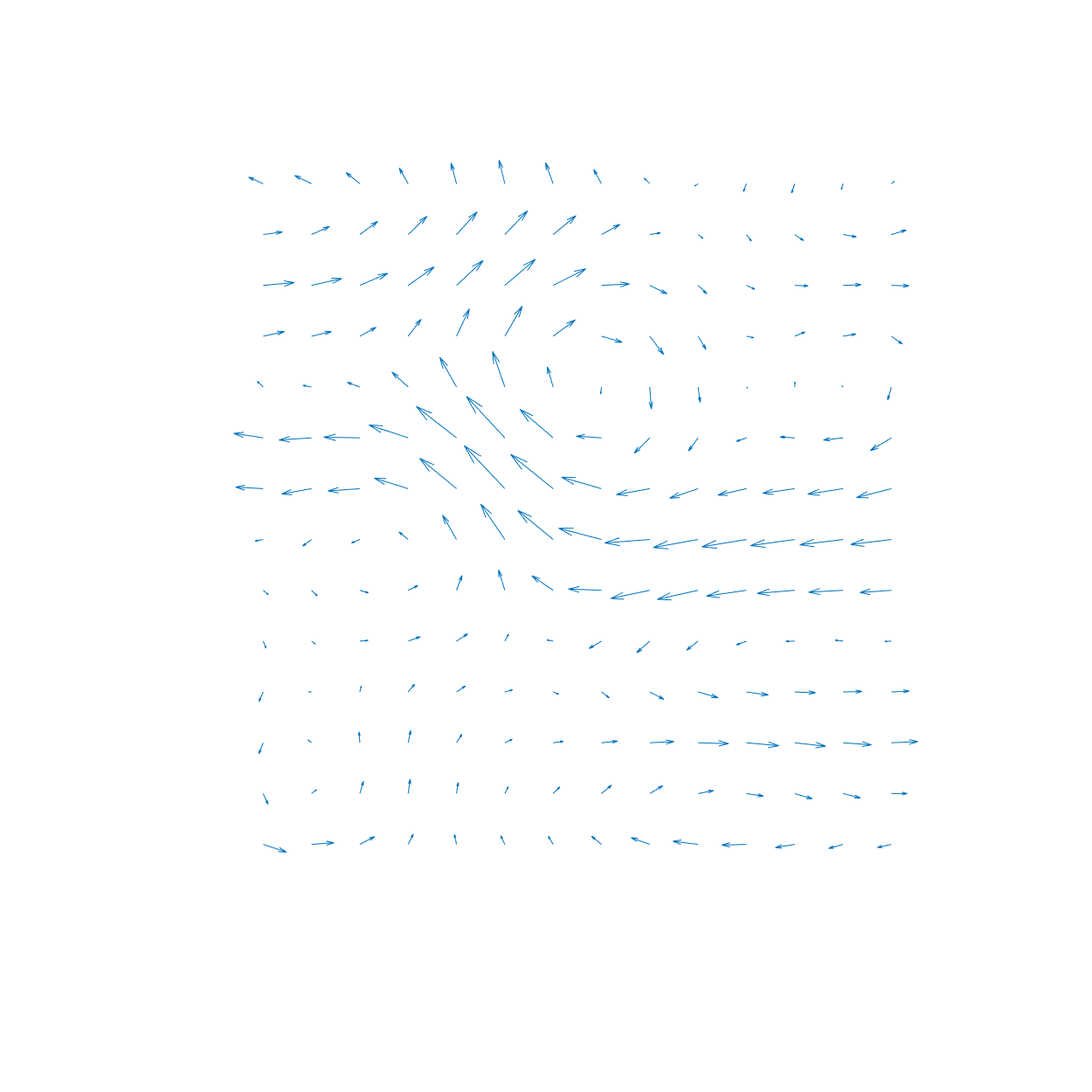

Supplement: S3 MCG raw data 3 — The raw MCG dataset includes category 4 for training and validation. (ZIP) [file pone.0338189.s003.zip › train/4/p11_285_1.png]

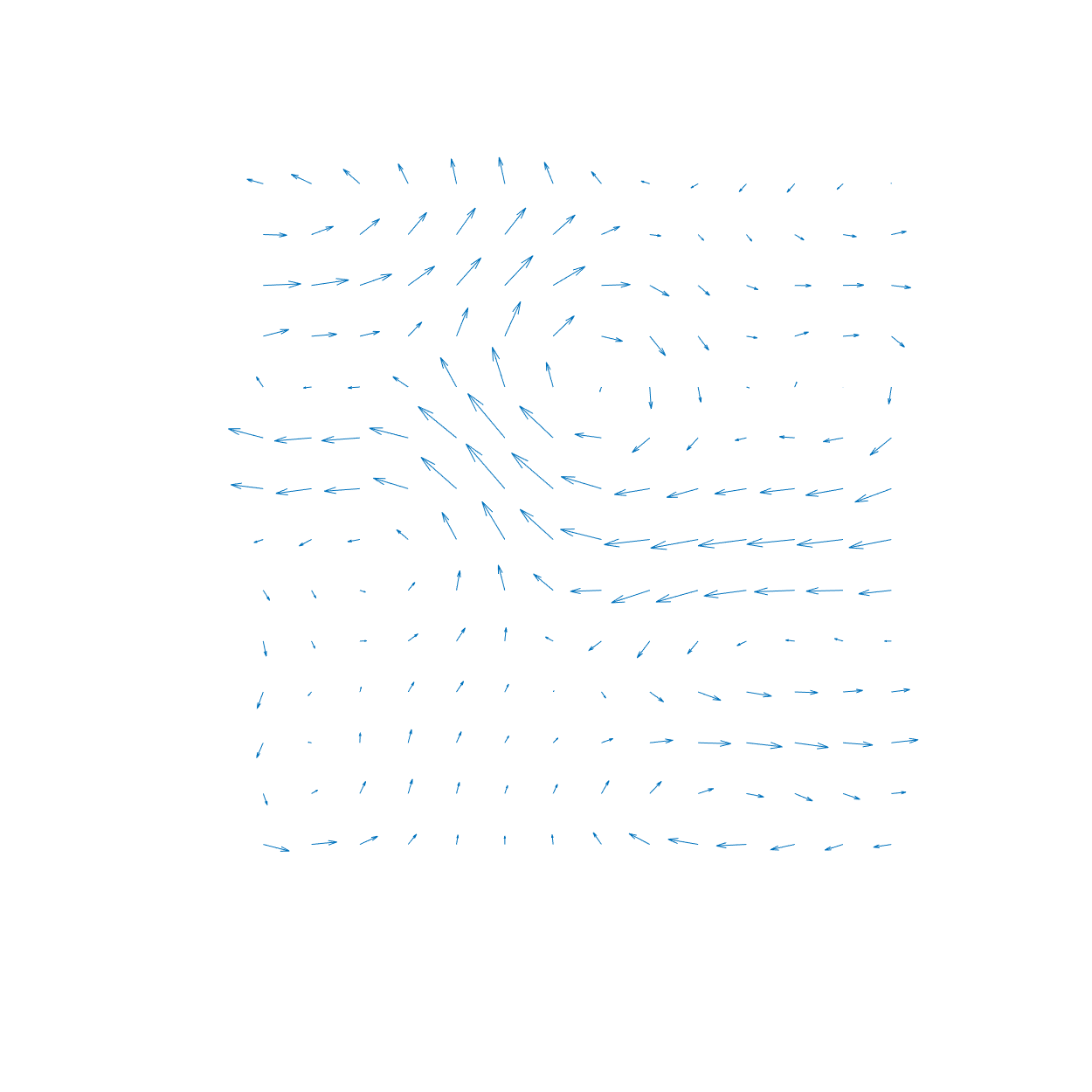

Supplement: S3 MCG raw data 3 — The raw MCG dataset includes category 4 for training and validation. (ZIP) [file pone.0338189.s003.zip › train/4/p11_285_2.png]

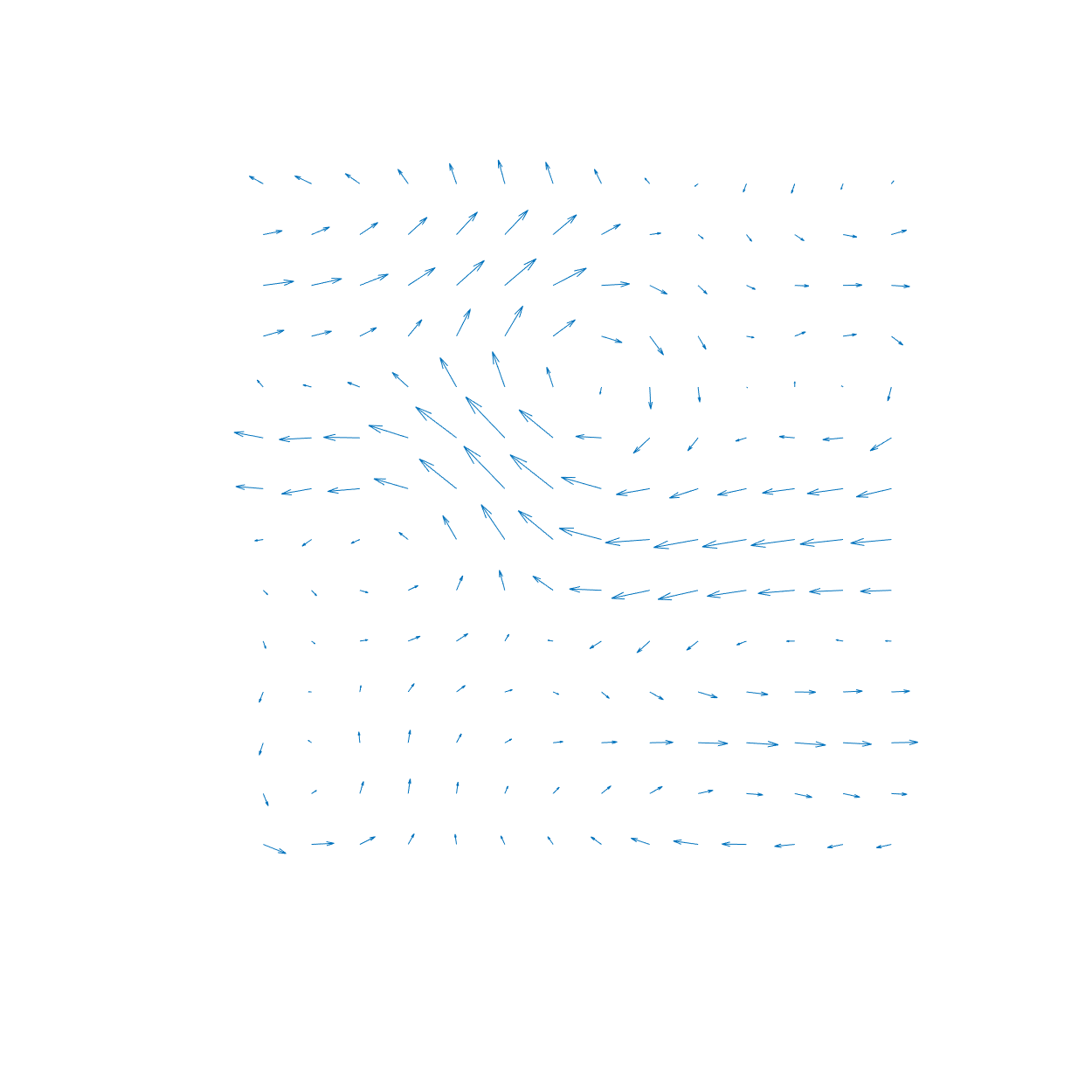

Supplement: S3 MCG raw data 3 — The raw MCG dataset includes category 4 for training and validation. (ZIP) [file pone.0338189.s003.zip › train/4/p11_285_3.png]

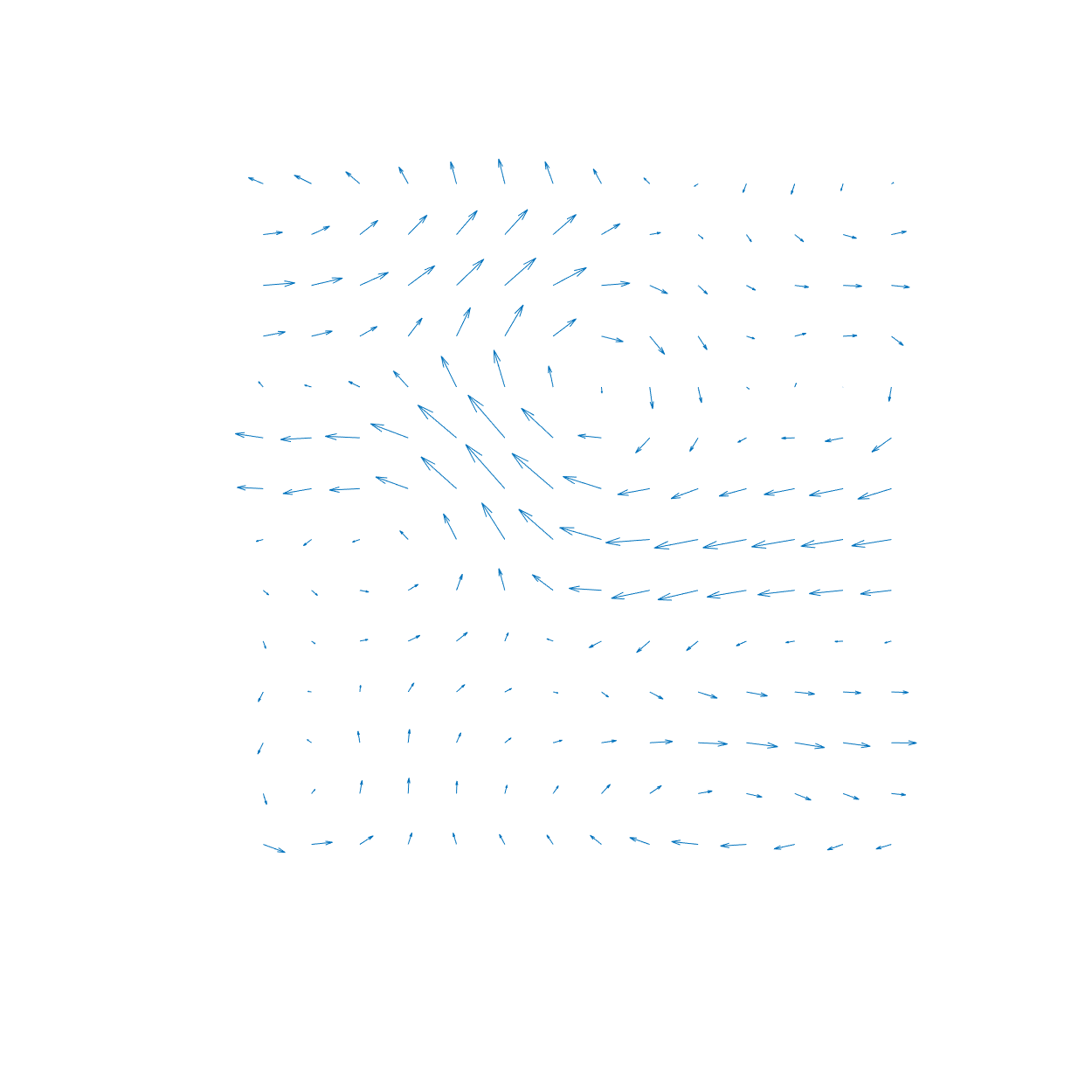

Supplement: S3 MCG raw data 3 — The raw MCG dataset includes category 4 for training and validation. (ZIP) [file pone.0338189.s003.zip › train/4/p11_285_4.png]
