# Supplementary figures and images for: Classification of current density vector map using transformer hybrid residual network (part 5 of 6)
Source: PLoS One. 2025 Dec 16;20(12):e0338189. doi: 10.1371/journal.pone.0338189 (PMC12707687; doi:10.1371/journal.pone.0338189)

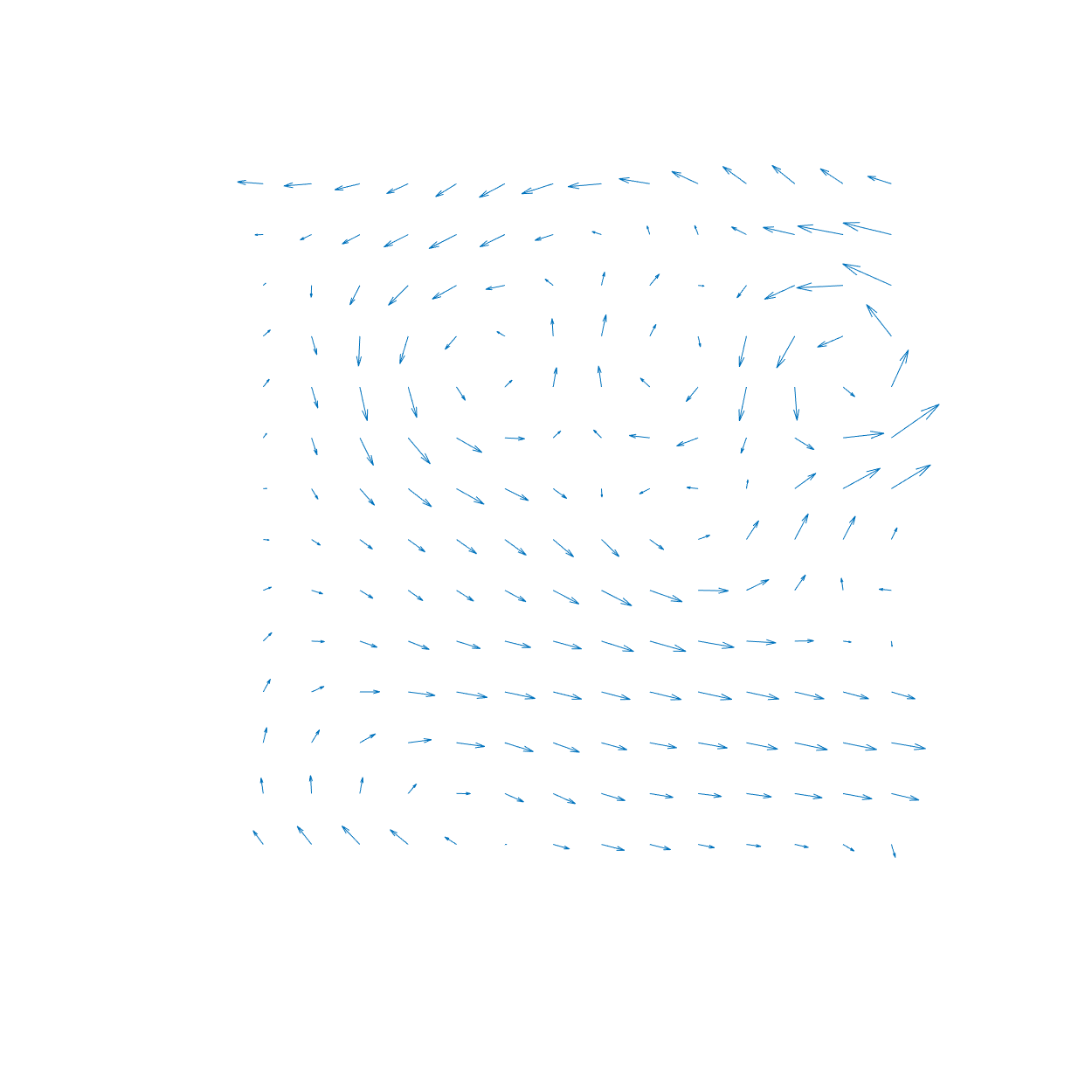

Supplement: S3 MCG raw data 3 — The raw MCG dataset includes category 4 for training and validation. (ZIP) [file pone.0338189.s003.zip › train/4/p10_330_3.png]

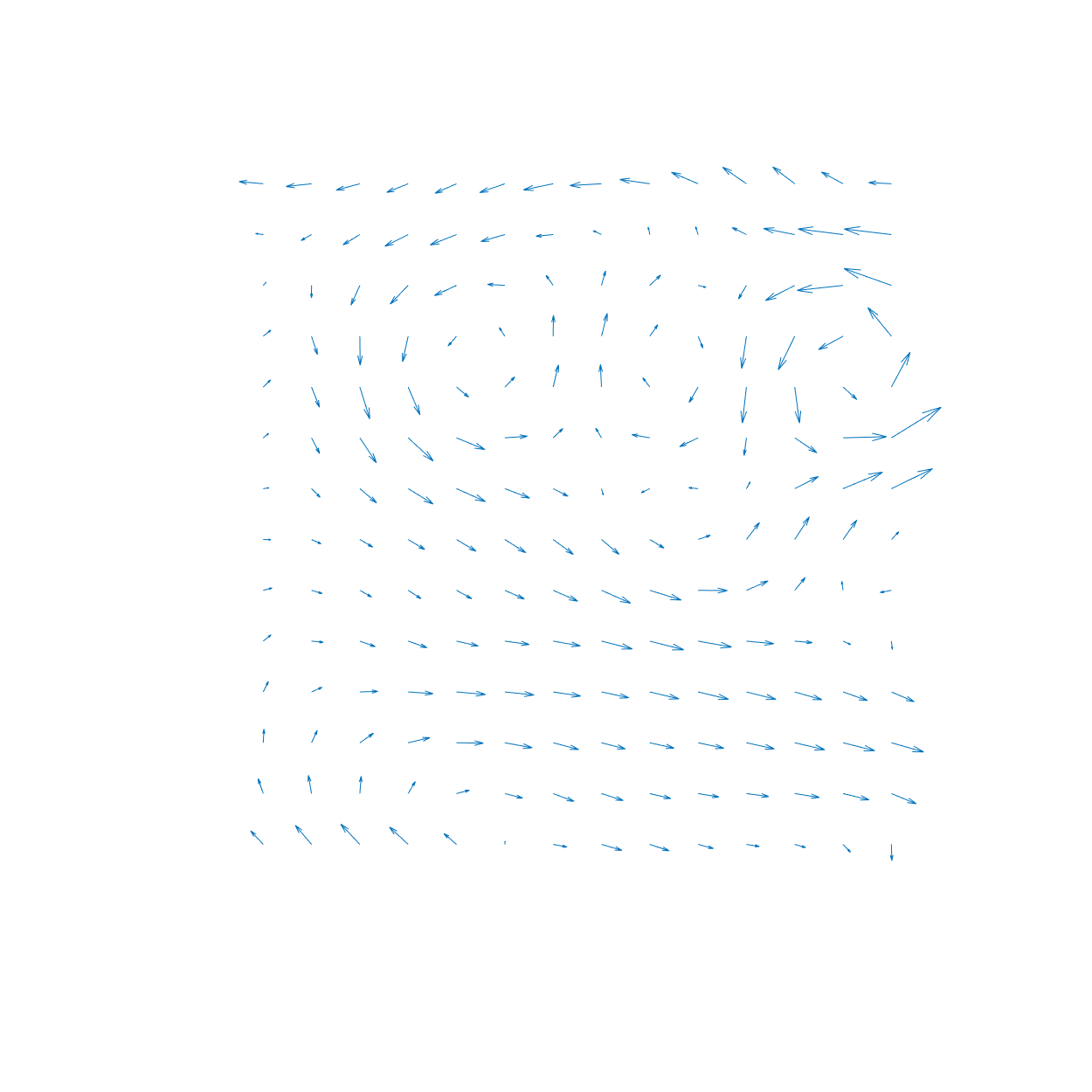

Supplement: S3 MCG raw data 3 — The raw MCG dataset includes category 4 for training and validation. (ZIP) [file pone.0338189.s003.zip › train/4/p10_330_4.png]

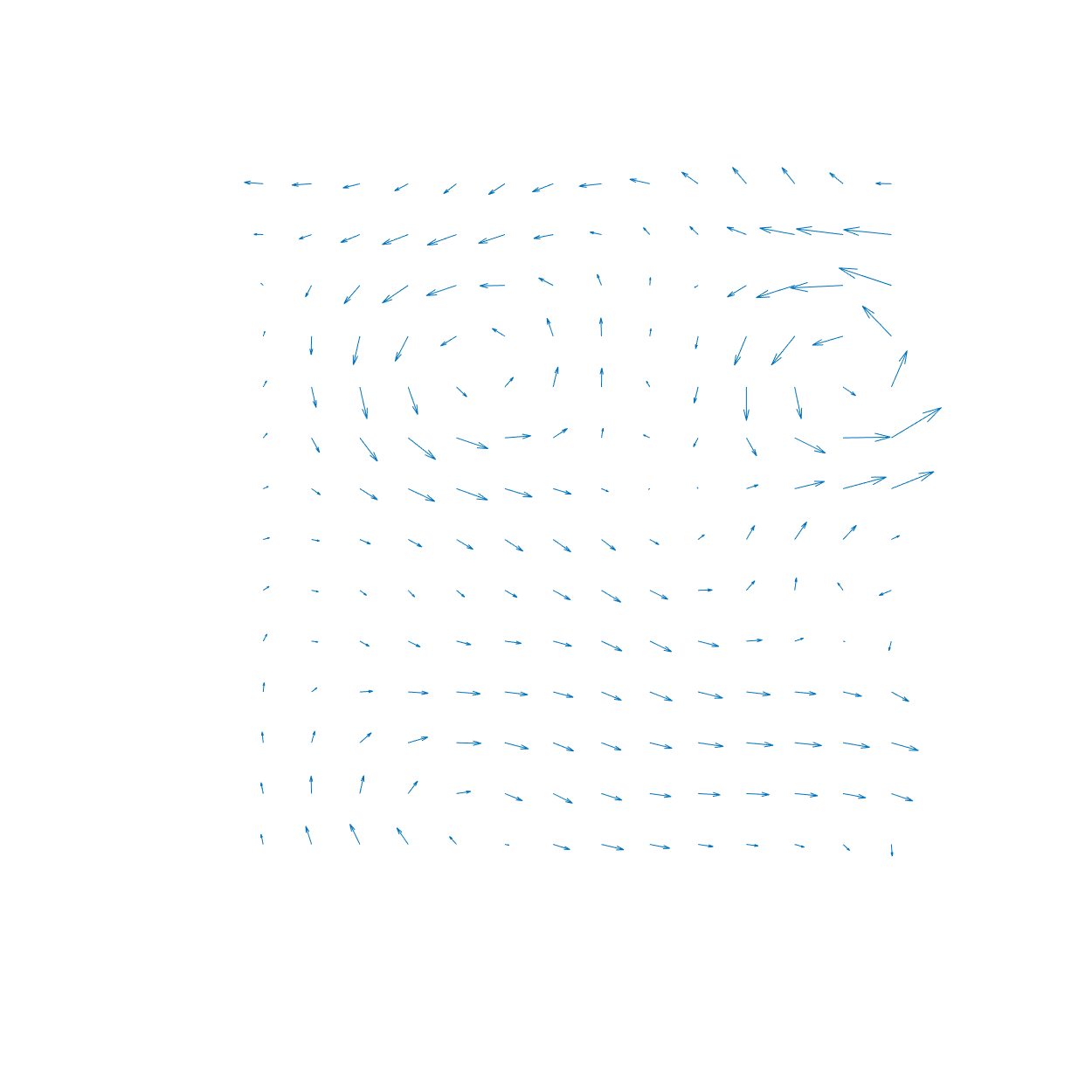

Supplement: S3 MCG raw data 3 — The raw MCG dataset includes category 4 for training and validation. (ZIP) [file pone.0338189.s003.zip › train/4/p10_335_1.png]

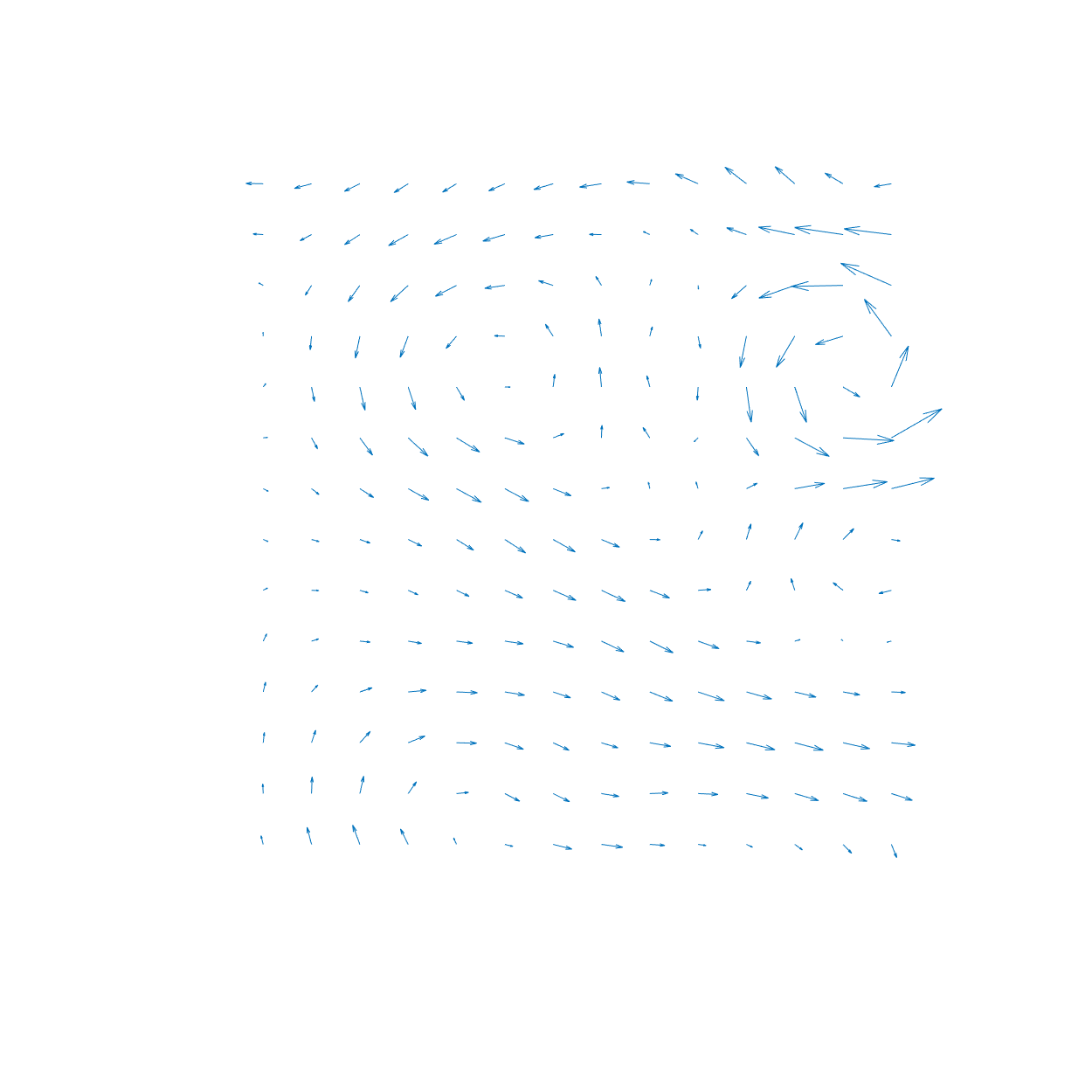

Supplement: S3 MCG raw data 3 — The raw MCG dataset includes category 4 for training and validation. (ZIP) [file pone.0338189.s003.zip › train/4/p10_335_2.png]

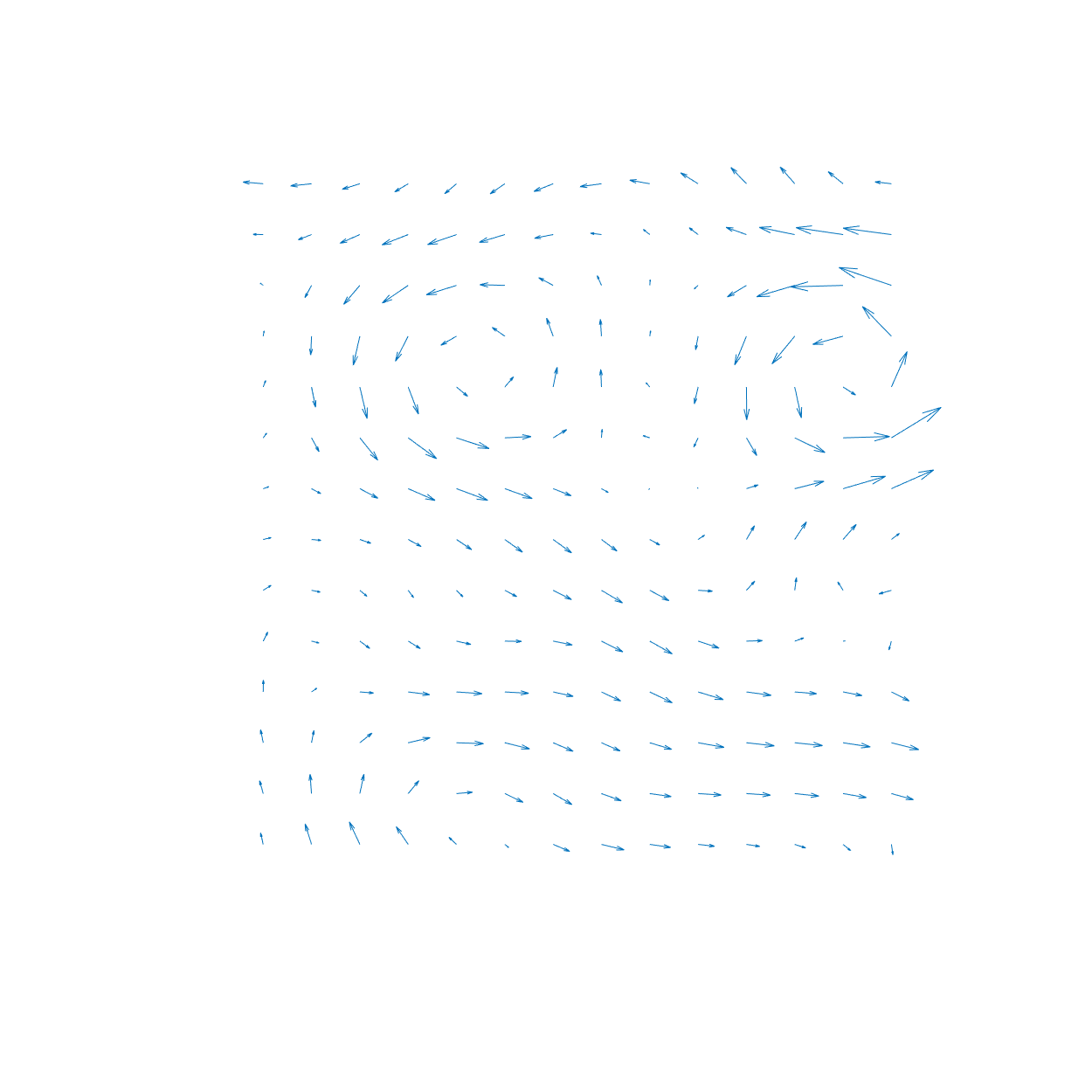

Supplement: S3 MCG raw data 3 — The raw MCG dataset includes category 4 for training and validation. (ZIP) [file pone.0338189.s003.zip › train/4/p10_335_3.png]

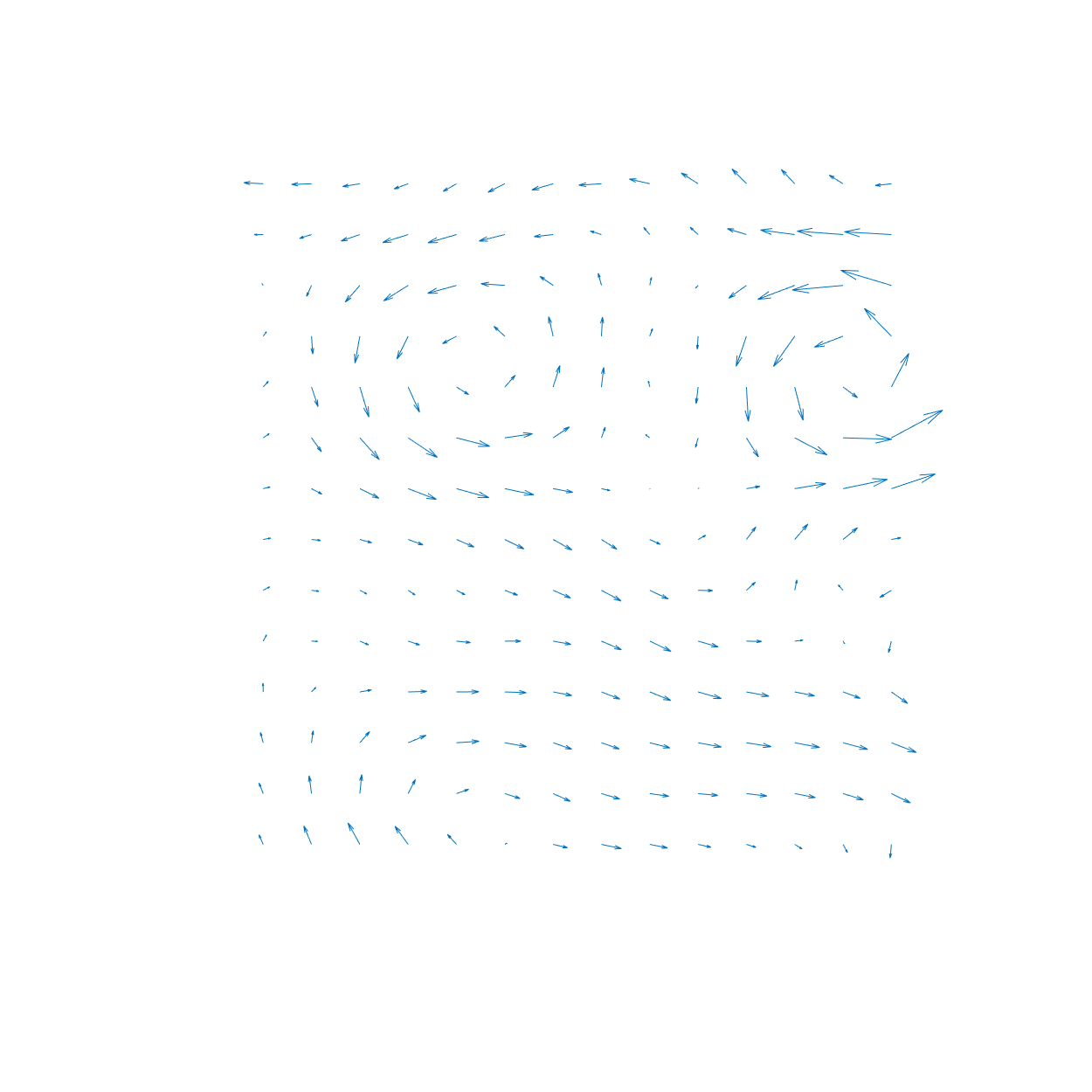

Supplement: S3 MCG raw data 3 — The raw MCG dataset includes category 4 for training and validation. (ZIP) [file pone.0338189.s003.zip › train/4/p10_335_4.png]

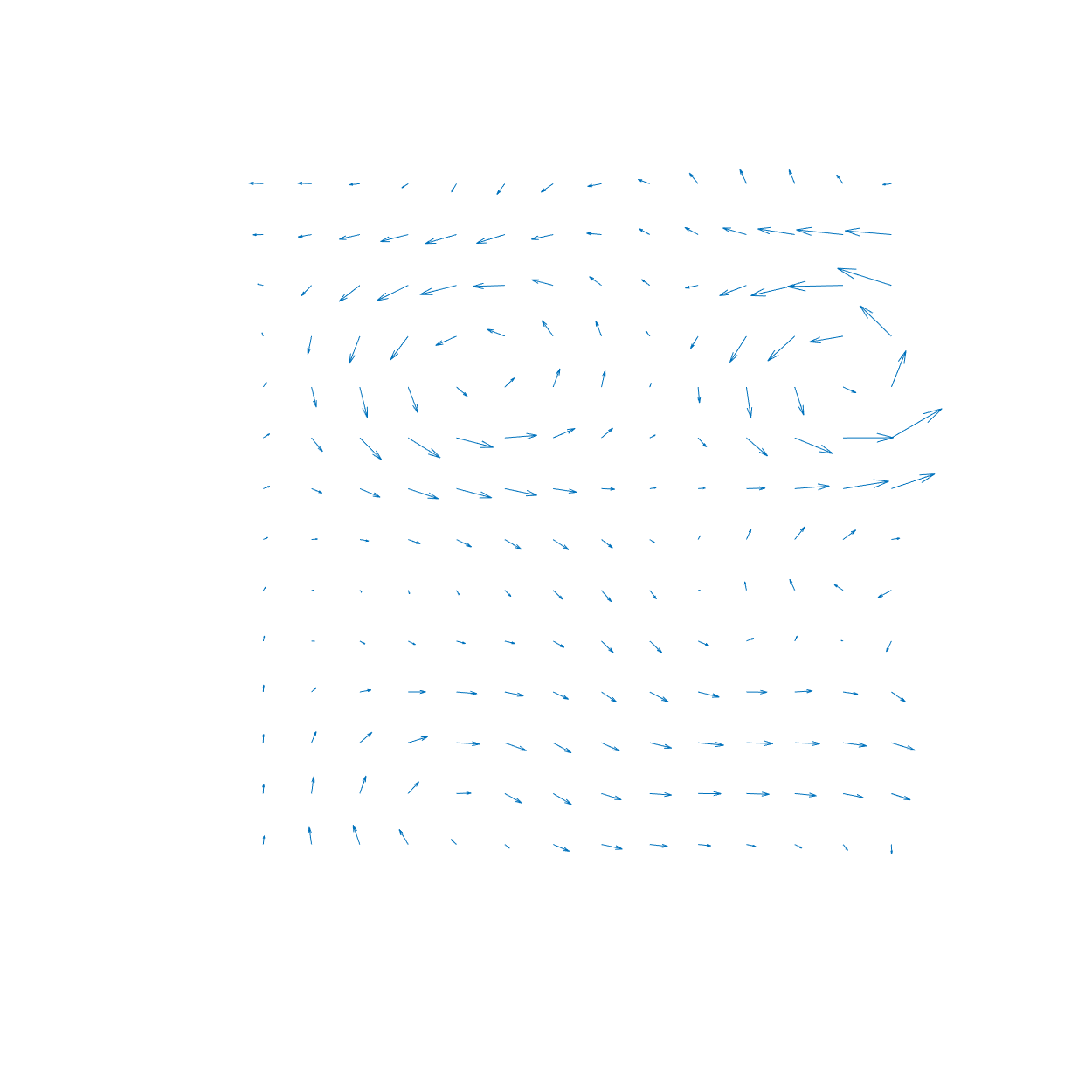

Supplement: S3 MCG raw data 3 — The raw MCG dataset includes category 4 for training and validation. (ZIP) [file pone.0338189.s003.zip › train/4/p10_340_1.png]

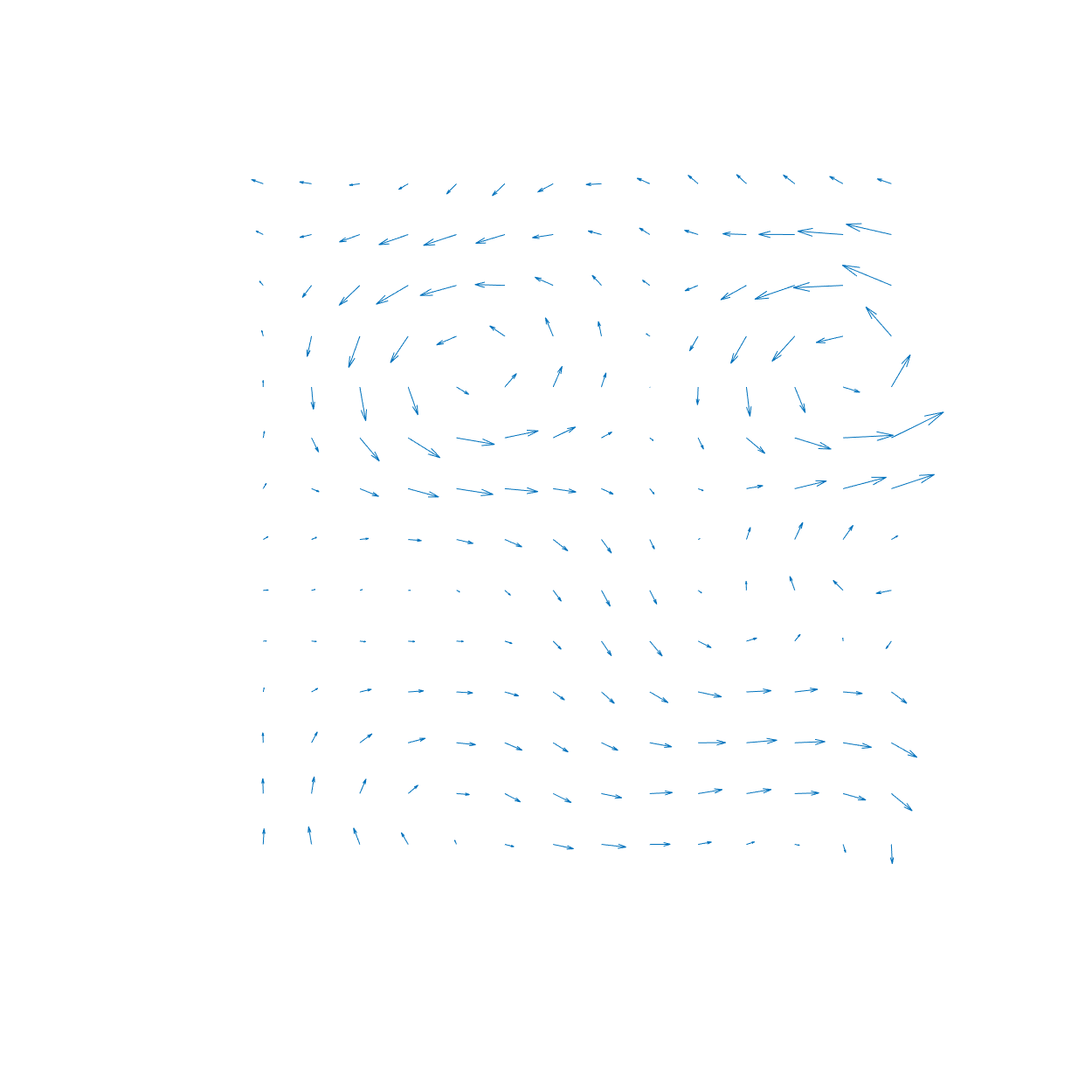

Supplement: S3 MCG raw data 3 — The raw MCG dataset includes category 4 for training and validation. (ZIP) [file pone.0338189.s003.zip › train/4/p10_340_2.png]

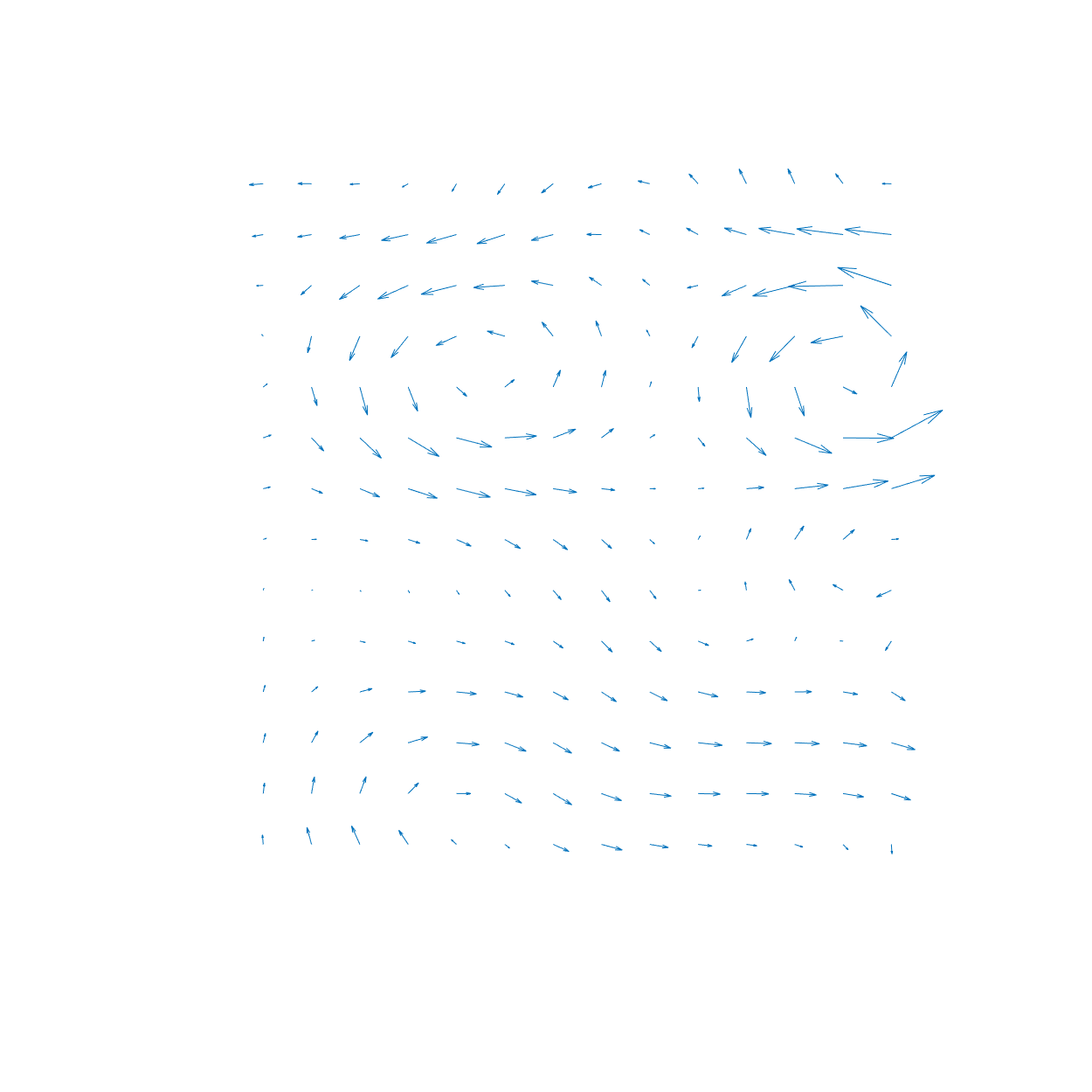

Supplement: S3 MCG raw data 3 — The raw MCG dataset includes category 4 for training and validation. (ZIP) [file pone.0338189.s003.zip › train/4/p10_340_3.png]

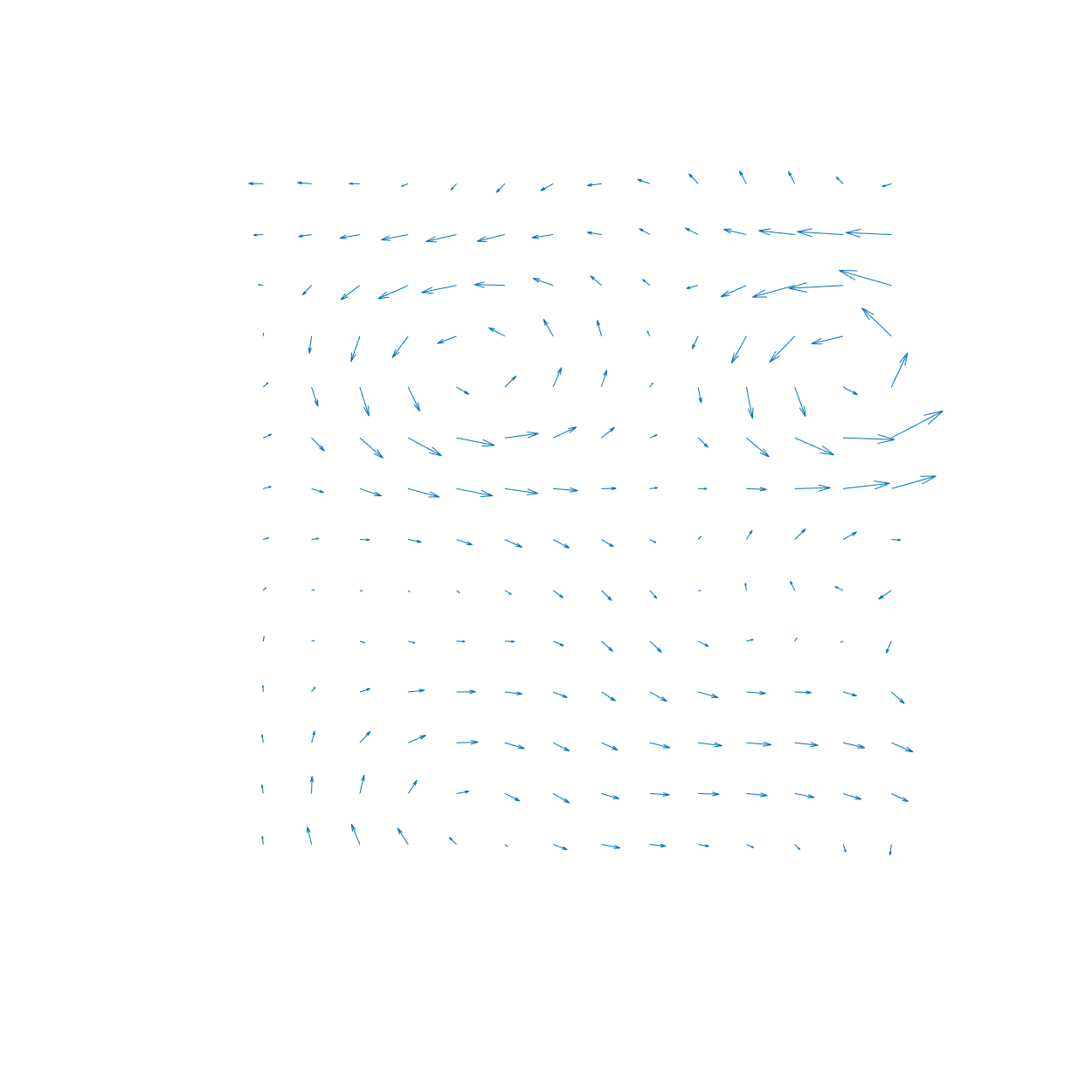

Supplement: S3 MCG raw data 3 — The raw MCG dataset includes category 4 for training and validation. (ZIP) [file pone.0338189.s003.zip › train/4/p10_340_4.png]

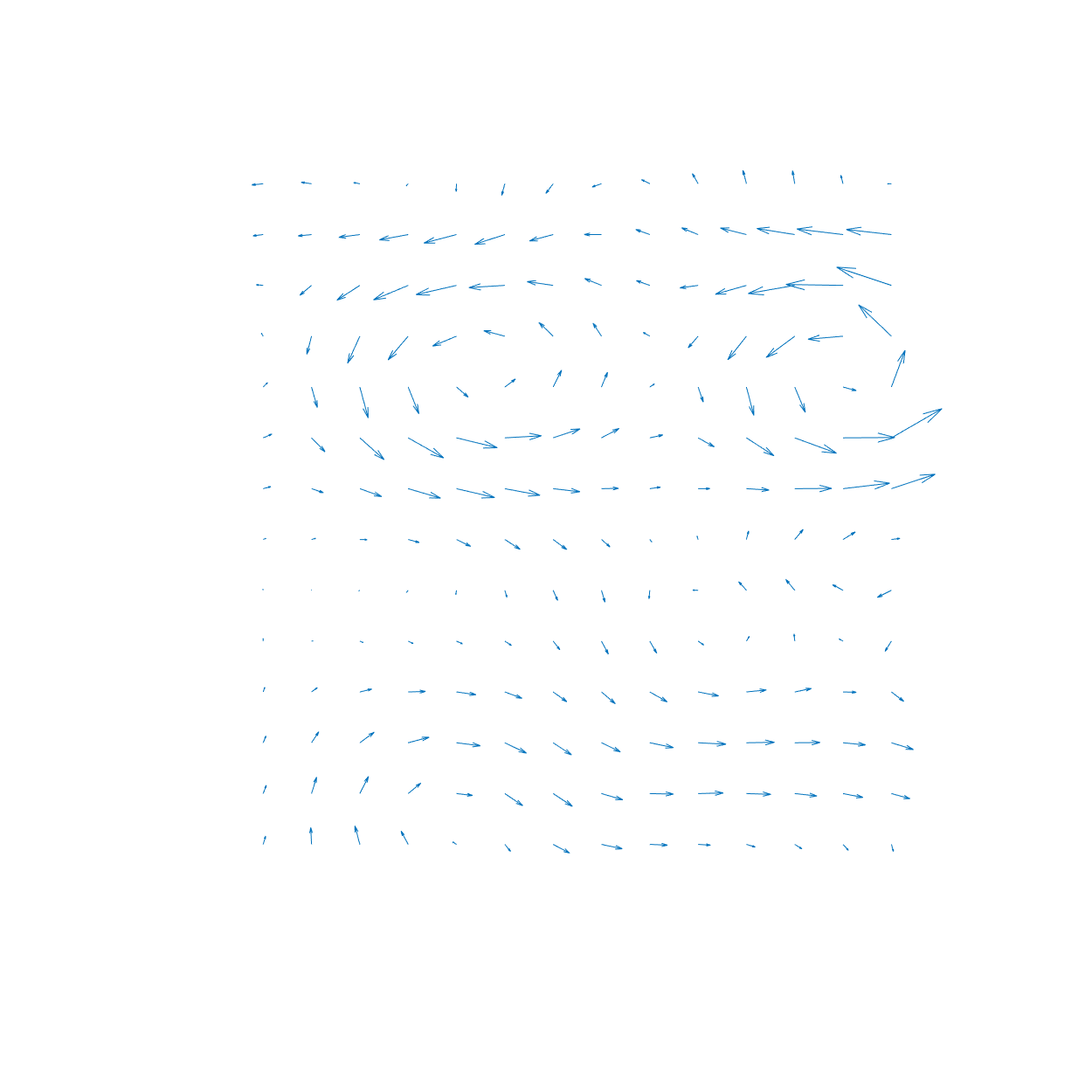

Supplement: S3 MCG raw data 3 — The raw MCG dataset includes category 4 for training and validation. (ZIP) [file pone.0338189.s003.zip › train/4/p10_345_1.png]

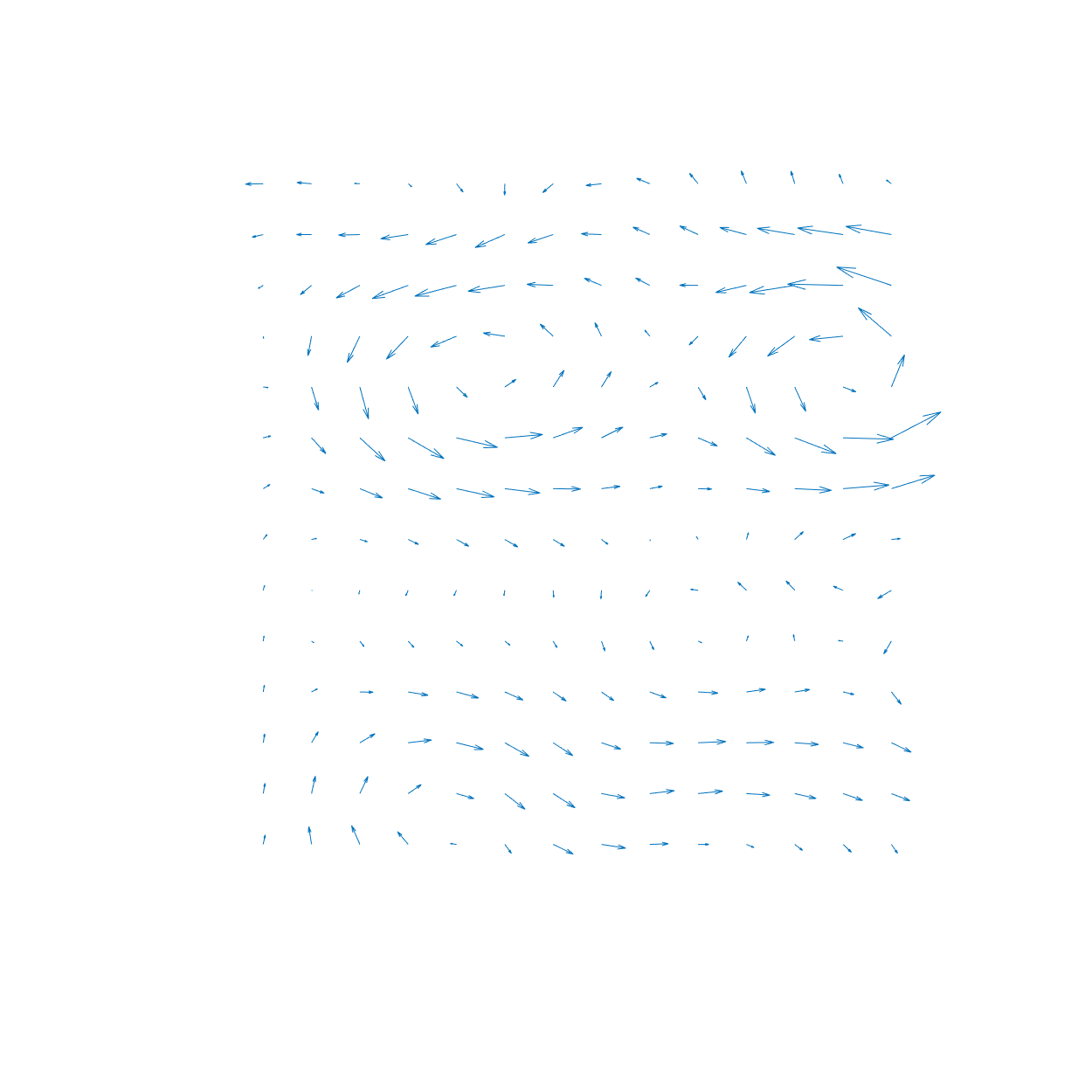

Supplement: S3 MCG raw data 3 — The raw MCG dataset includes category 4 for training and validation. (ZIP) [file pone.0338189.s003.zip › train/4/p10_345_2.png]

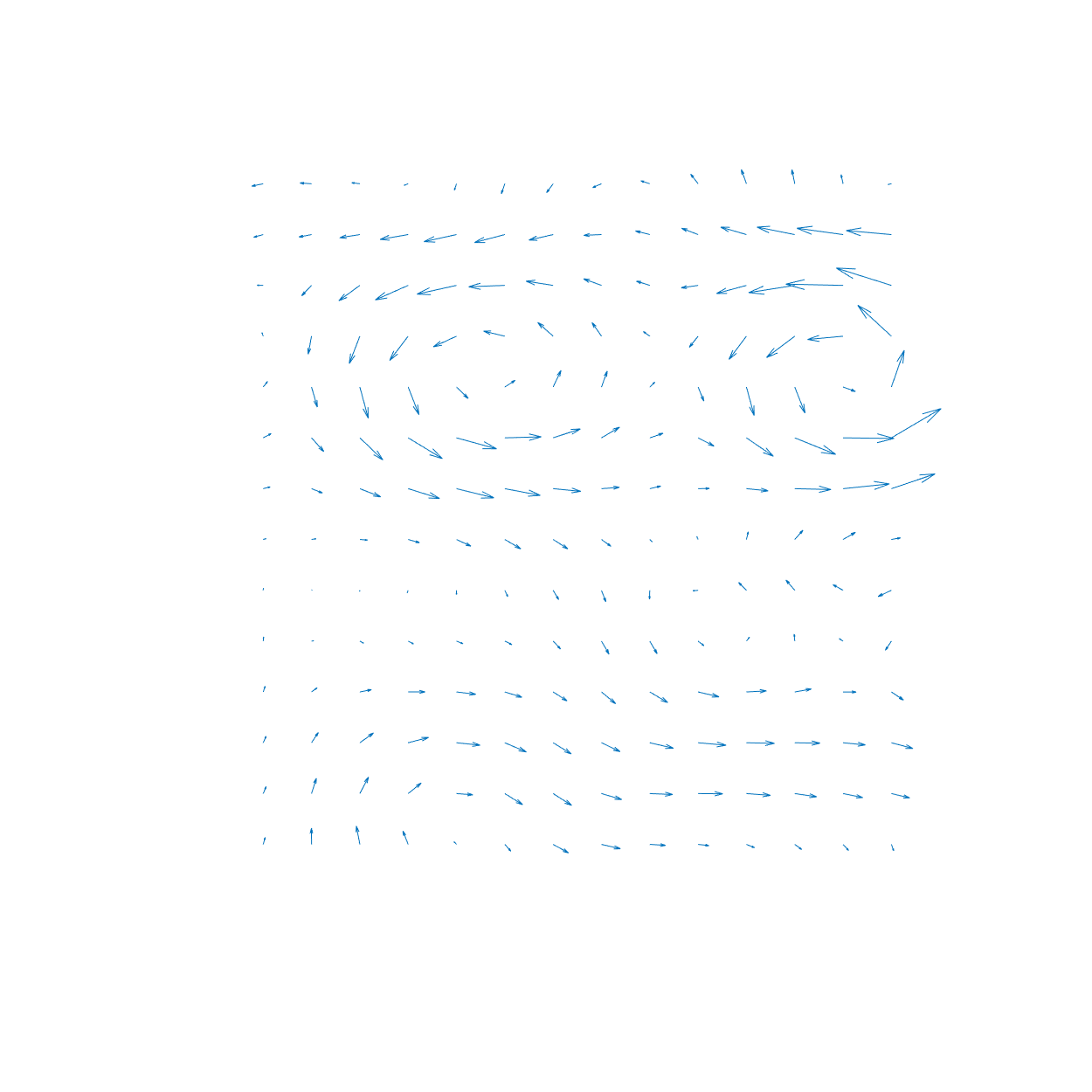

Supplement: S3 MCG raw data 3 — The raw MCG dataset includes category 4 for training and validation. (ZIP) [file pone.0338189.s003.zip › train/4/p10_345_3.png]

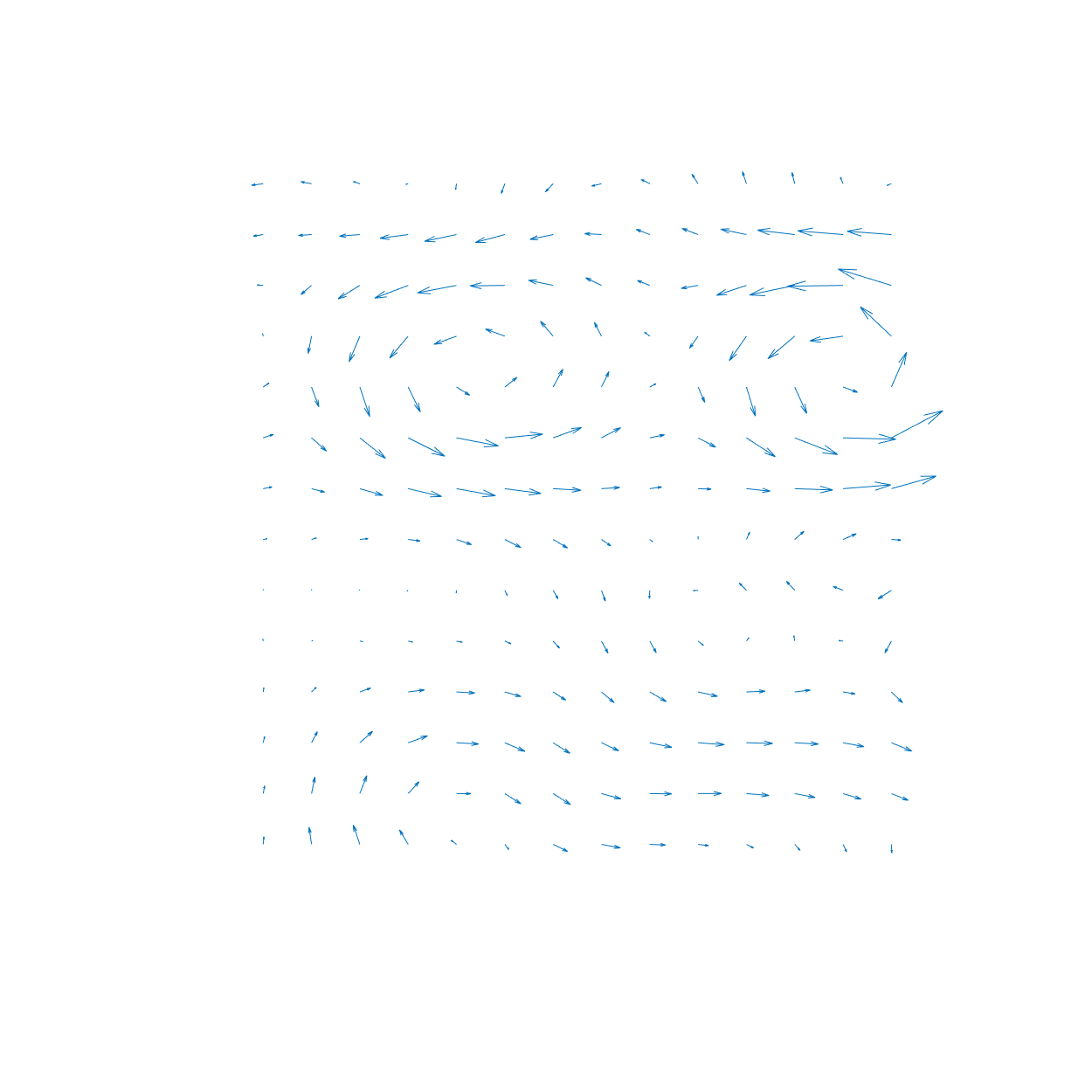

Supplement: S3 MCG raw data 3 — The raw MCG dataset includes category 4 for training and validation. (ZIP) [file pone.0338189.s003.zip › train/4/p10_345_4.png]

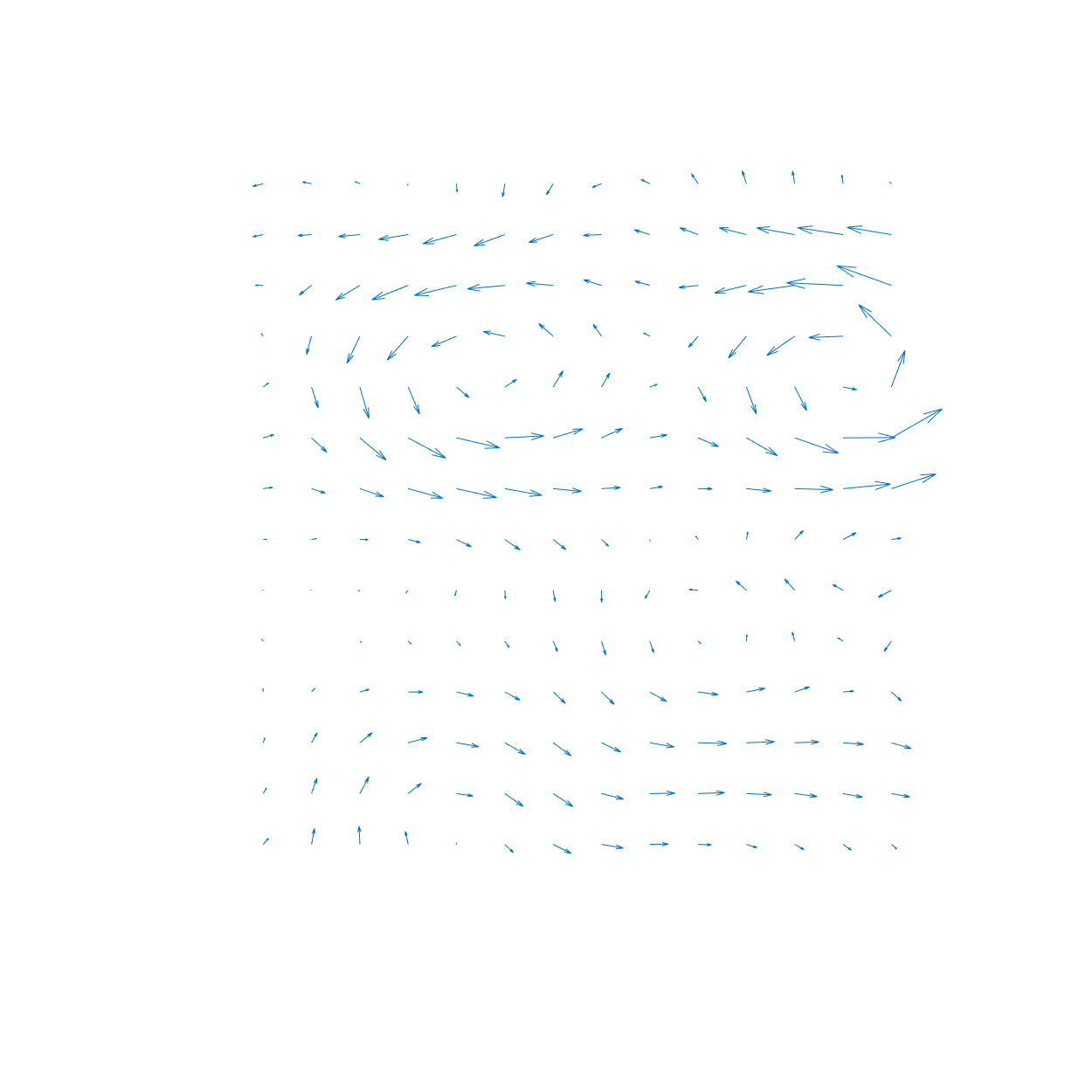

Supplement: S3 MCG raw data 3 — The raw MCG dataset includes category 4 for training and validation. (ZIP) [file pone.0338189.s003.zip › train/4/p10_350_1.png]

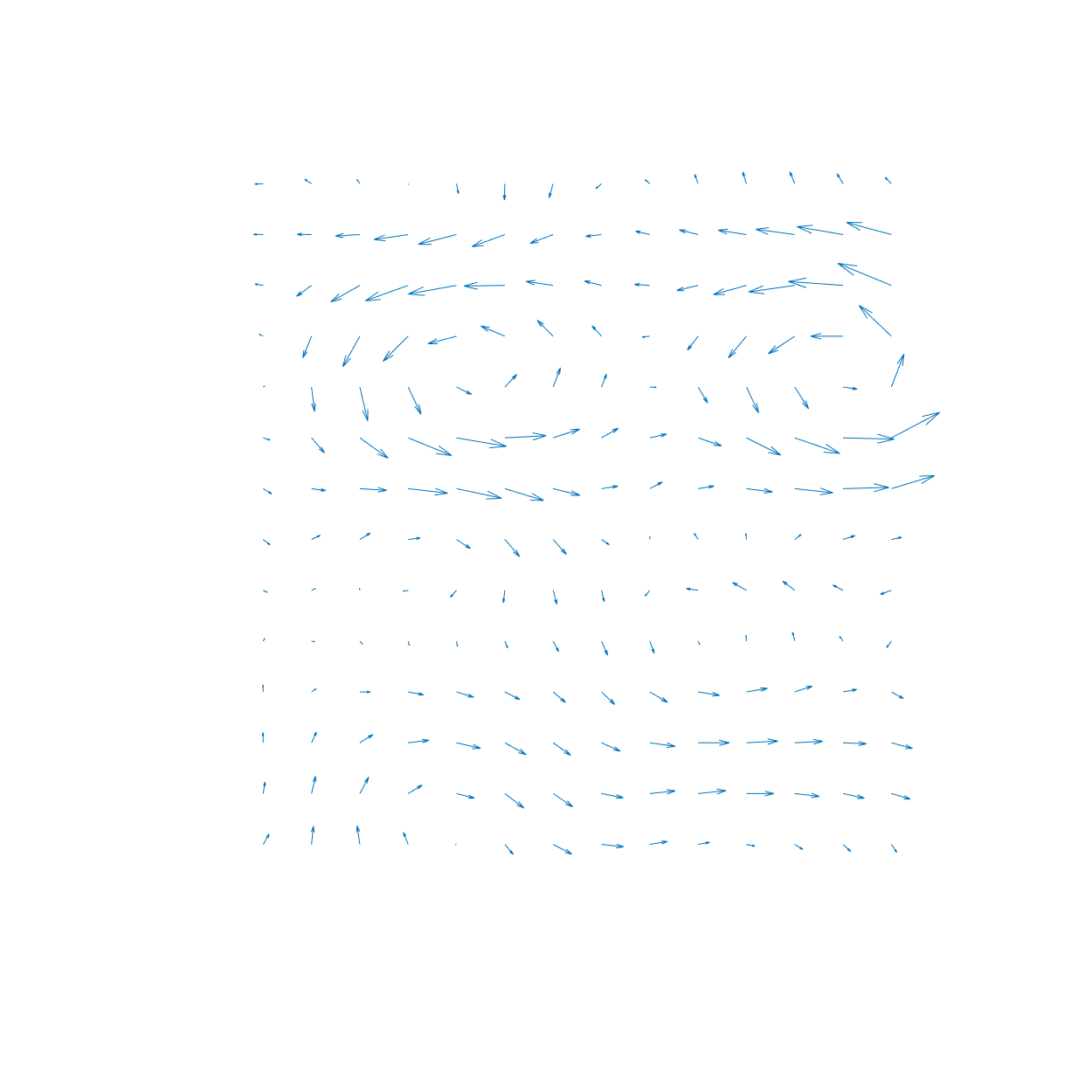

Supplement: S3 MCG raw data 3 — The raw MCG dataset includes category 4 for training and validation. (ZIP) [file pone.0338189.s003.zip › train/4/p10_350_2.png]

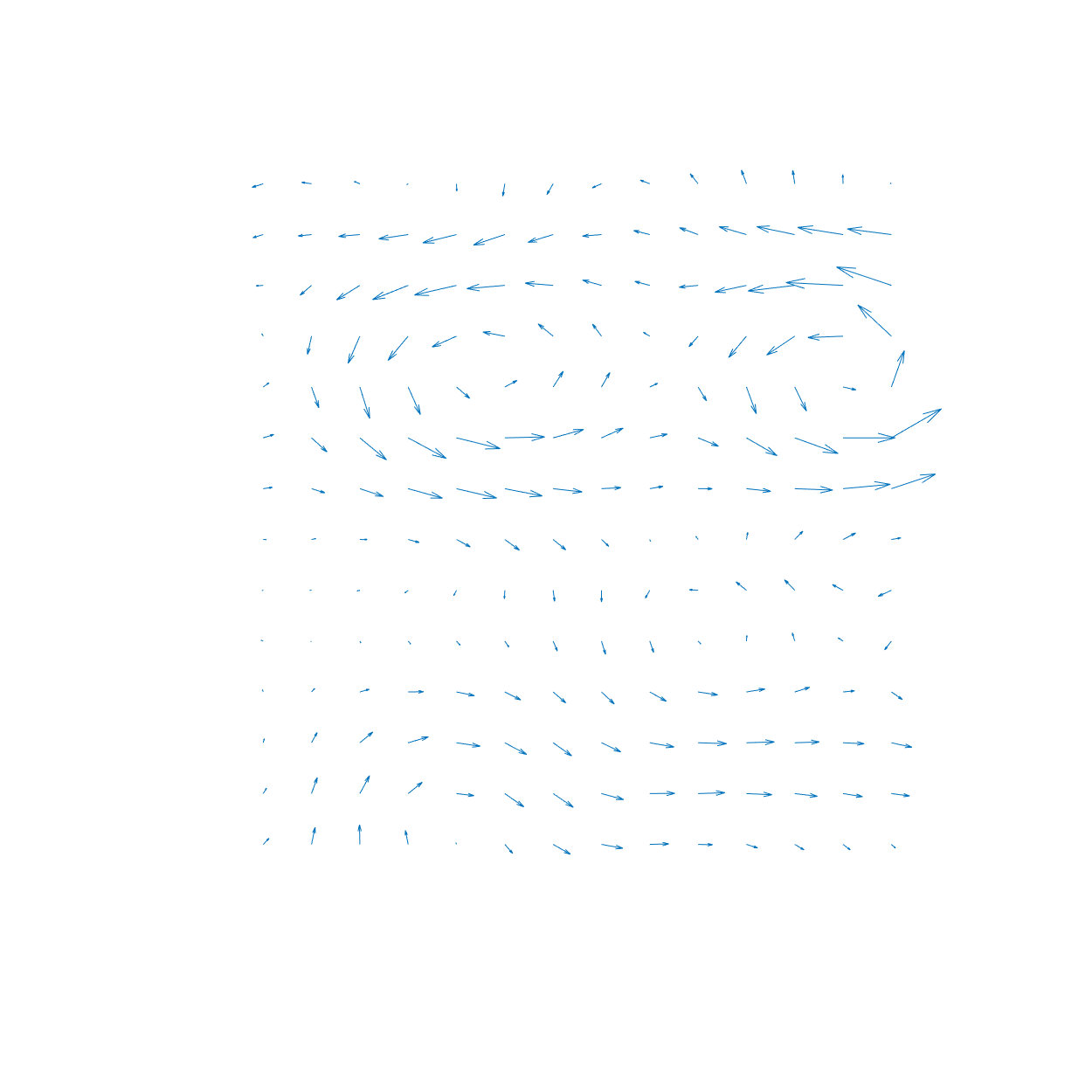

Supplement: S3 MCG raw data 3 — The raw MCG dataset includes category 4 for training and validation. (ZIP) [file pone.0338189.s003.zip › train/4/p10_350_3.png]

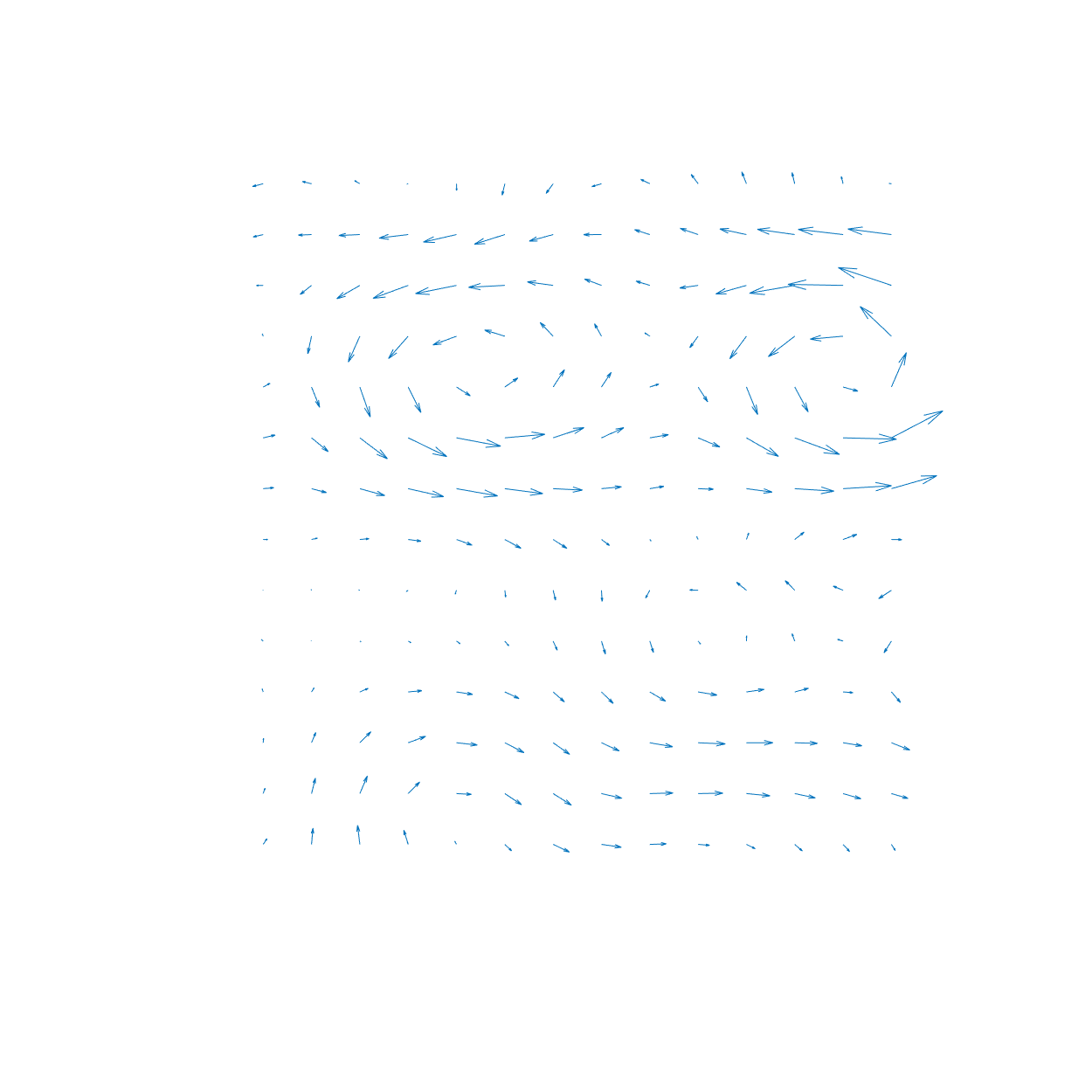

Supplement: S3 MCG raw data 3 — The raw MCG dataset includes category 4 for training and validation. (ZIP) [file pone.0338189.s003.zip › train/4/p10_350_4.png]

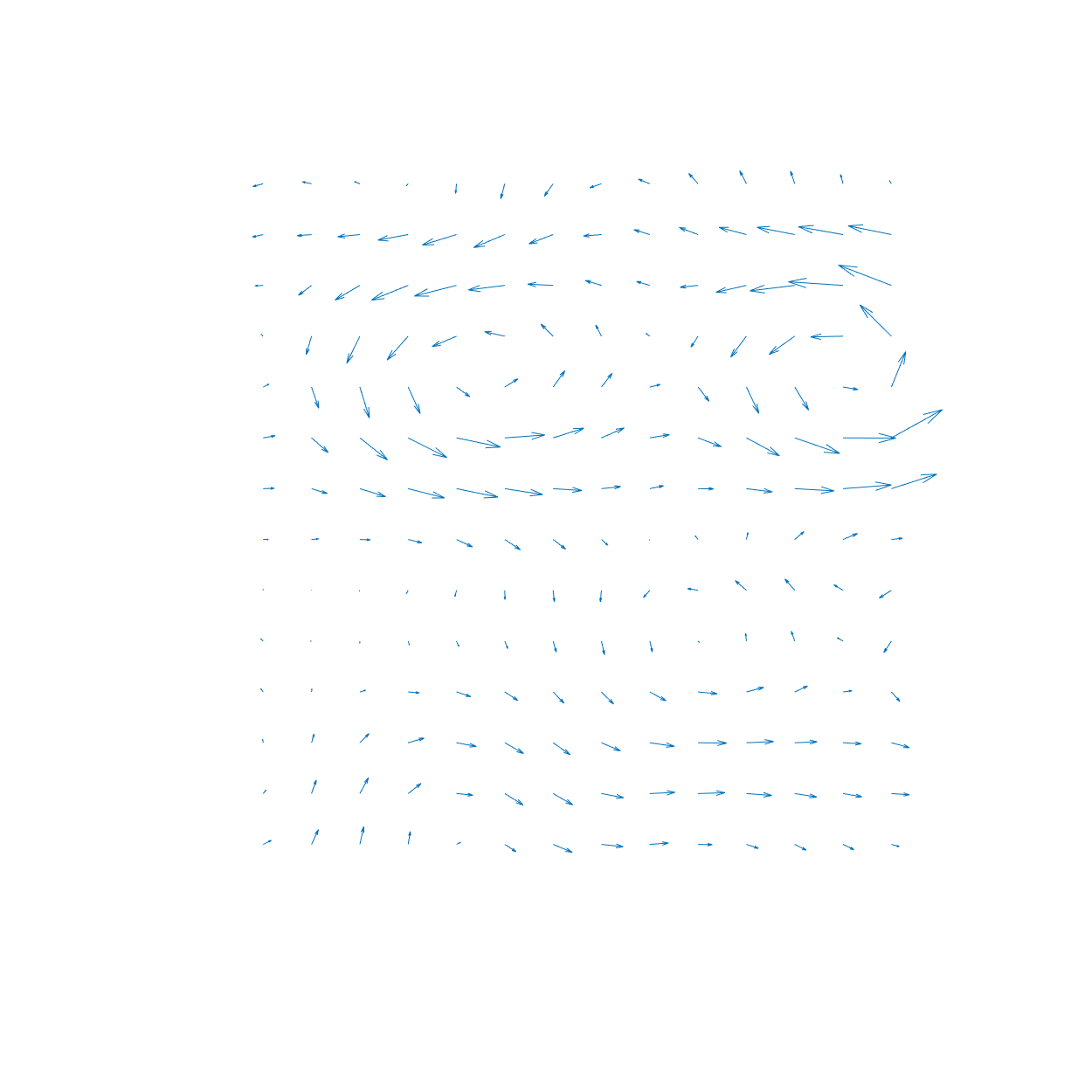

Supplement: S3 MCG raw data 3 — The raw MCG dataset includes category 4 for training and validation. (ZIP) [file pone.0338189.s003.zip › train/4/p10_355_1.png]

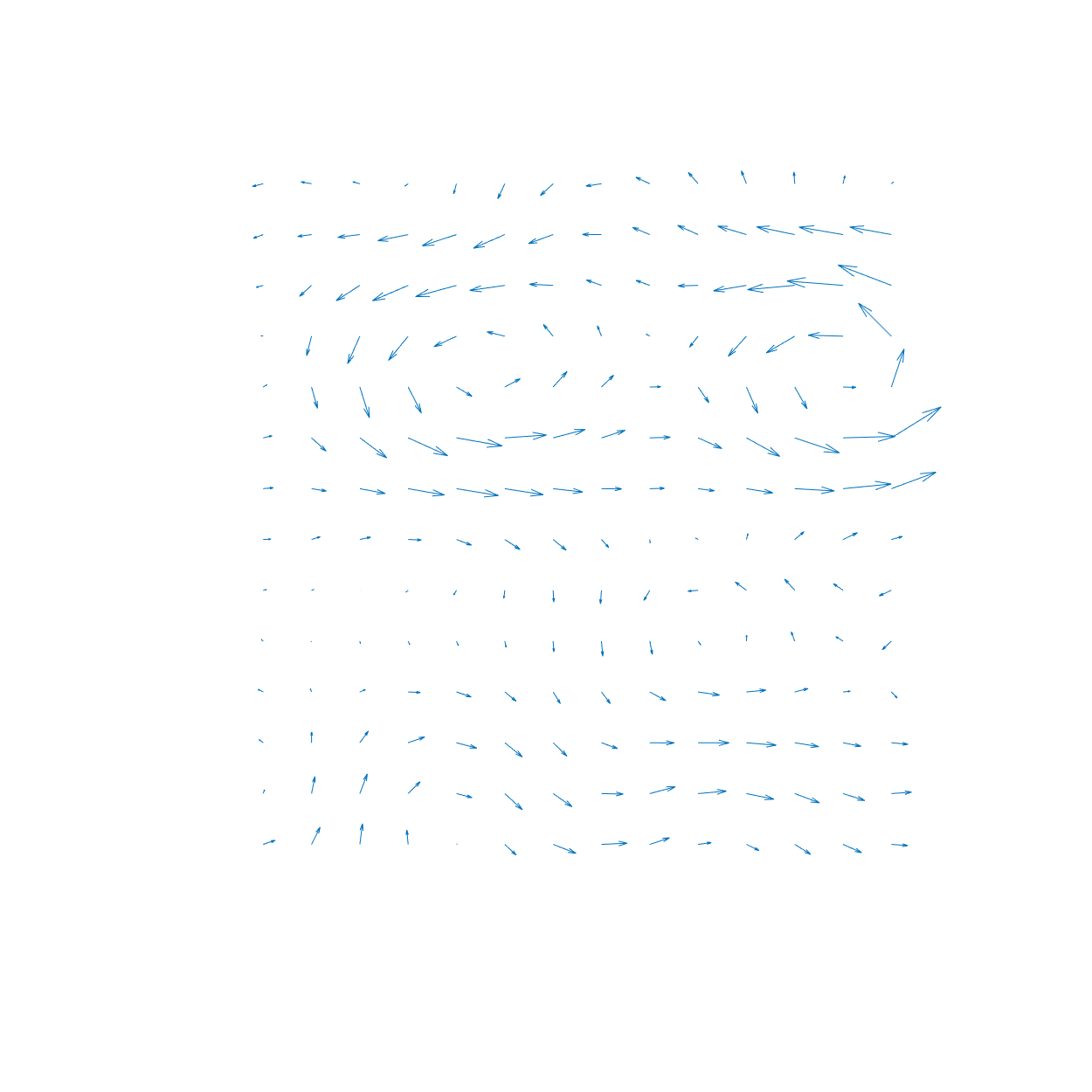

Supplement: S3 MCG raw data 3 — The raw MCG dataset includes category 4 for training and validation. (ZIP) [file pone.0338189.s003.zip › train/4/p10_355_2.png]

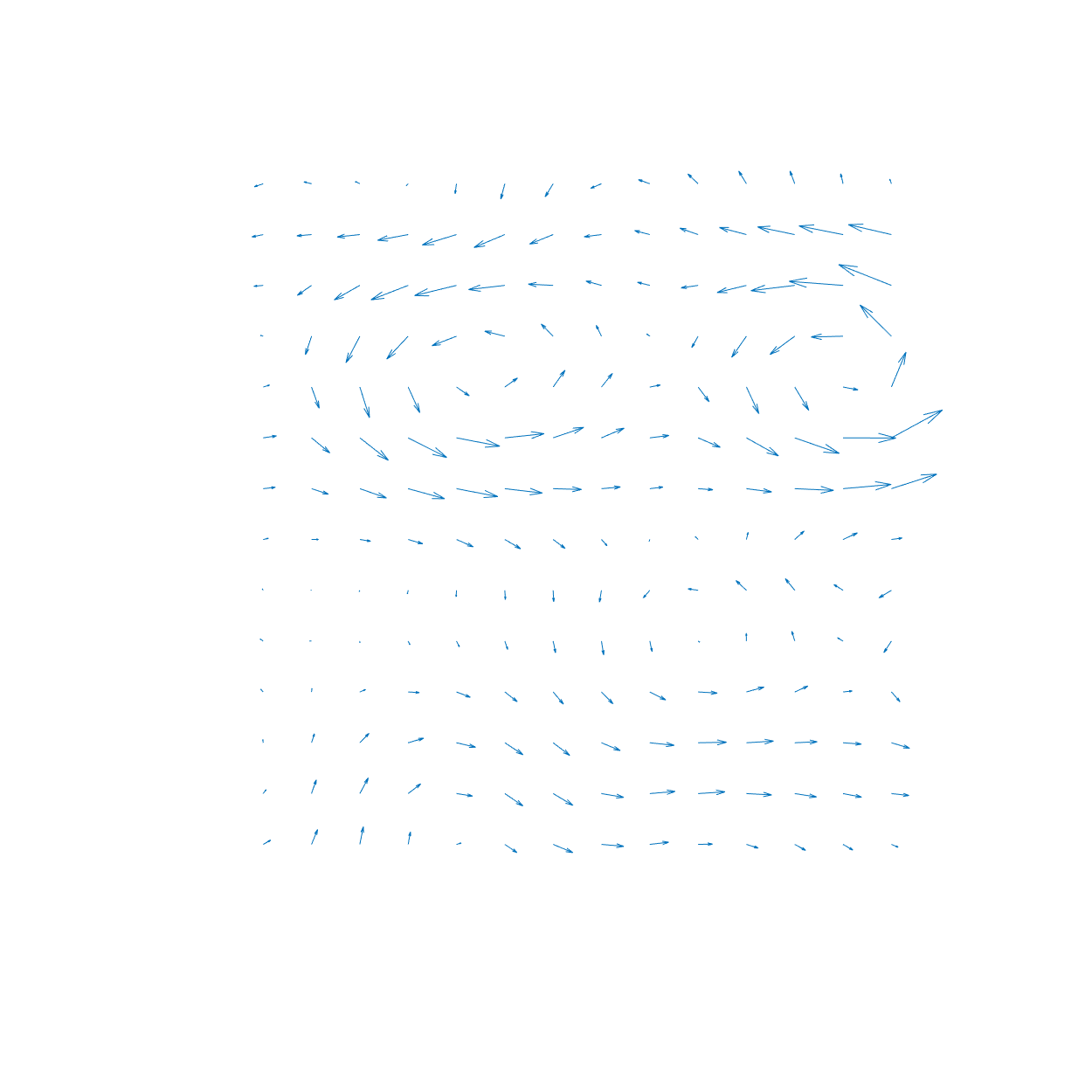

Supplement: S3 MCG raw data 3 — The raw MCG dataset includes category 4 for training and validation. (ZIP) [file pone.0338189.s003.zip › train/4/p10_355_3.png]

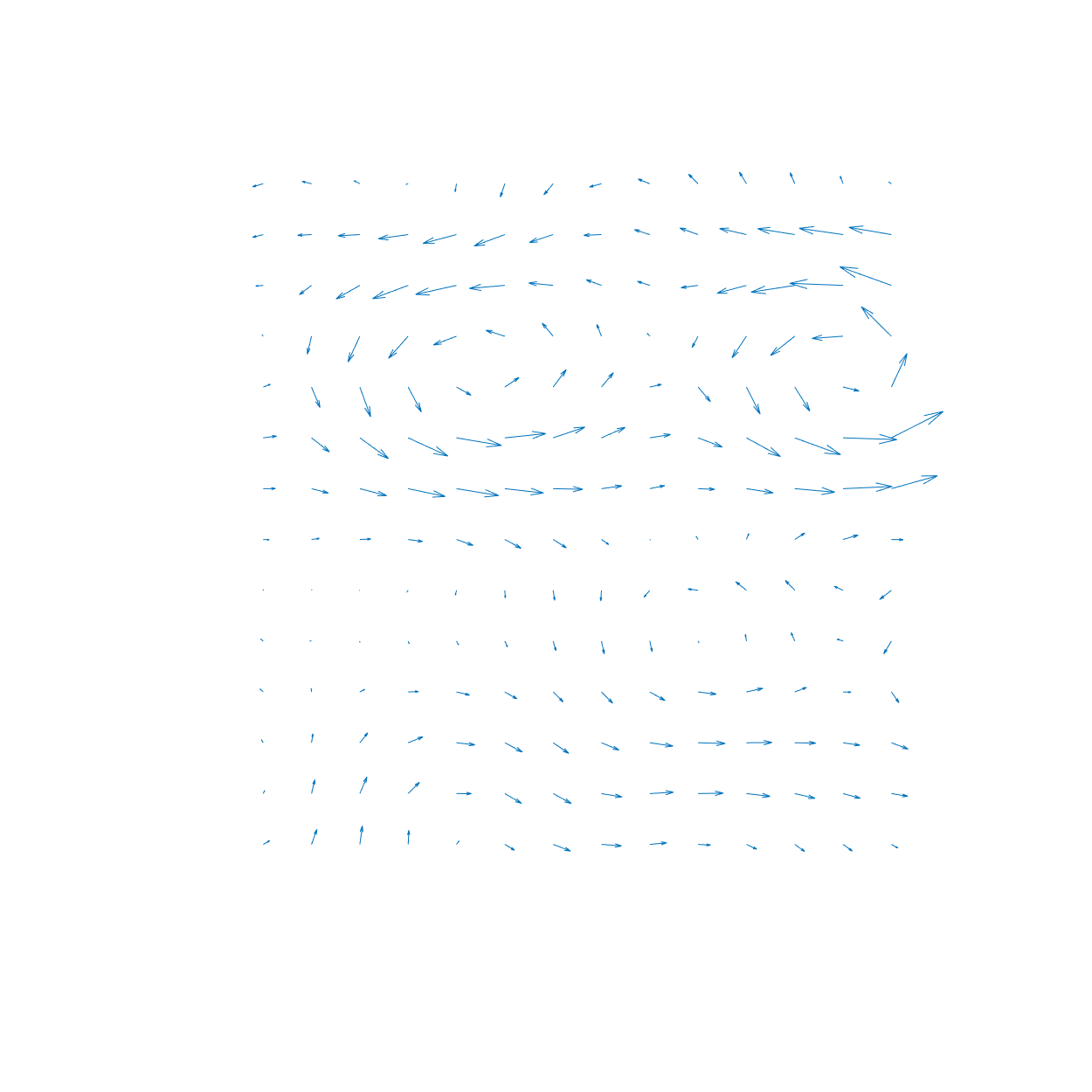

Supplement: S3 MCG raw data 3 — The raw MCG dataset includes category 4 for training and validation. (ZIP) [file pone.0338189.s003.zip › train/4/p10_355_4.png]

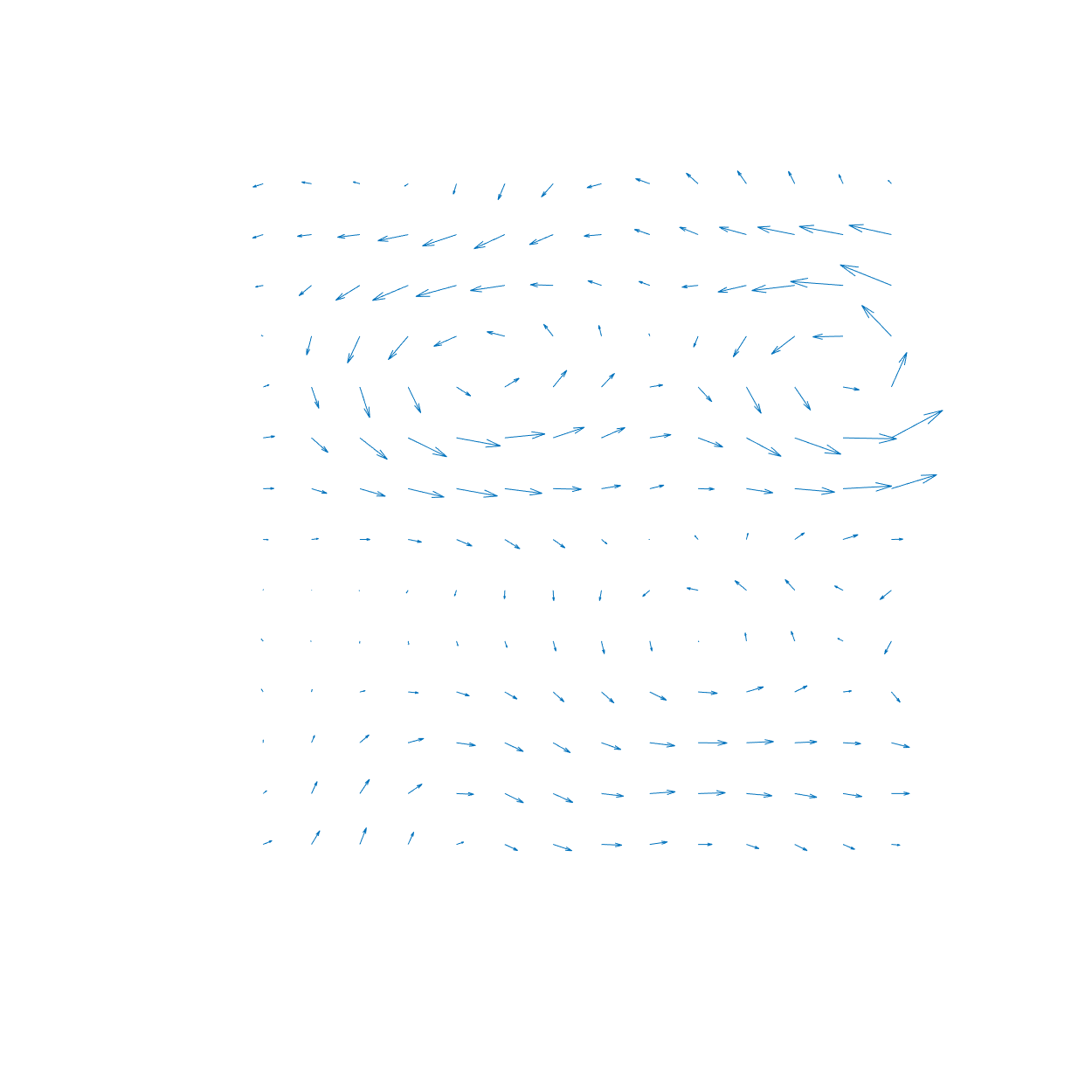

Supplement: S3 MCG raw data 3 — The raw MCG dataset includes category 4 for training and validation. (ZIP) [file pone.0338189.s003.zip › train/4/p10_360_1.png]

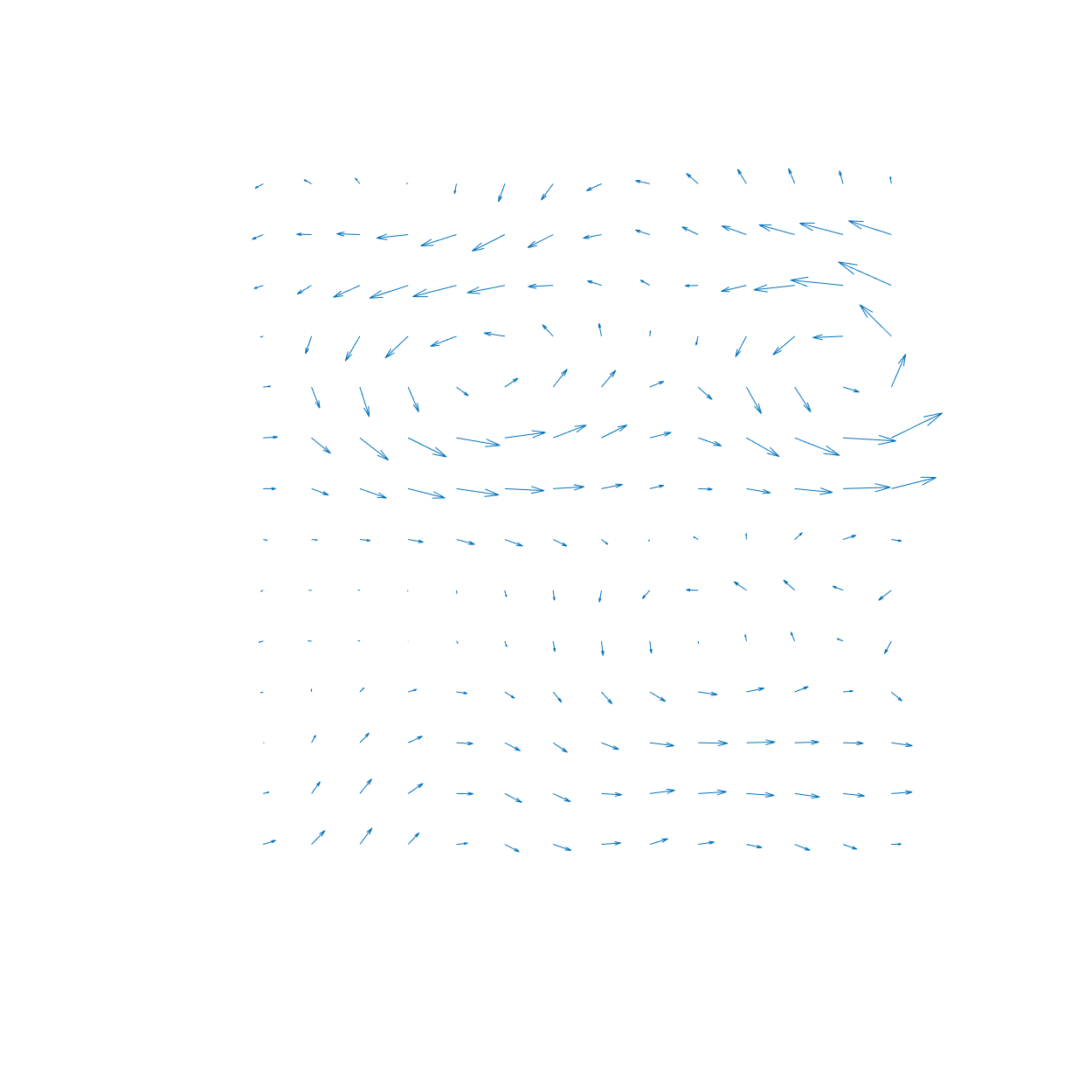

Supplement: S3 MCG raw data 3 — The raw MCG dataset includes category 4 for training and validation. (ZIP) [file pone.0338189.s003.zip › train/4/p10_360_2.png]

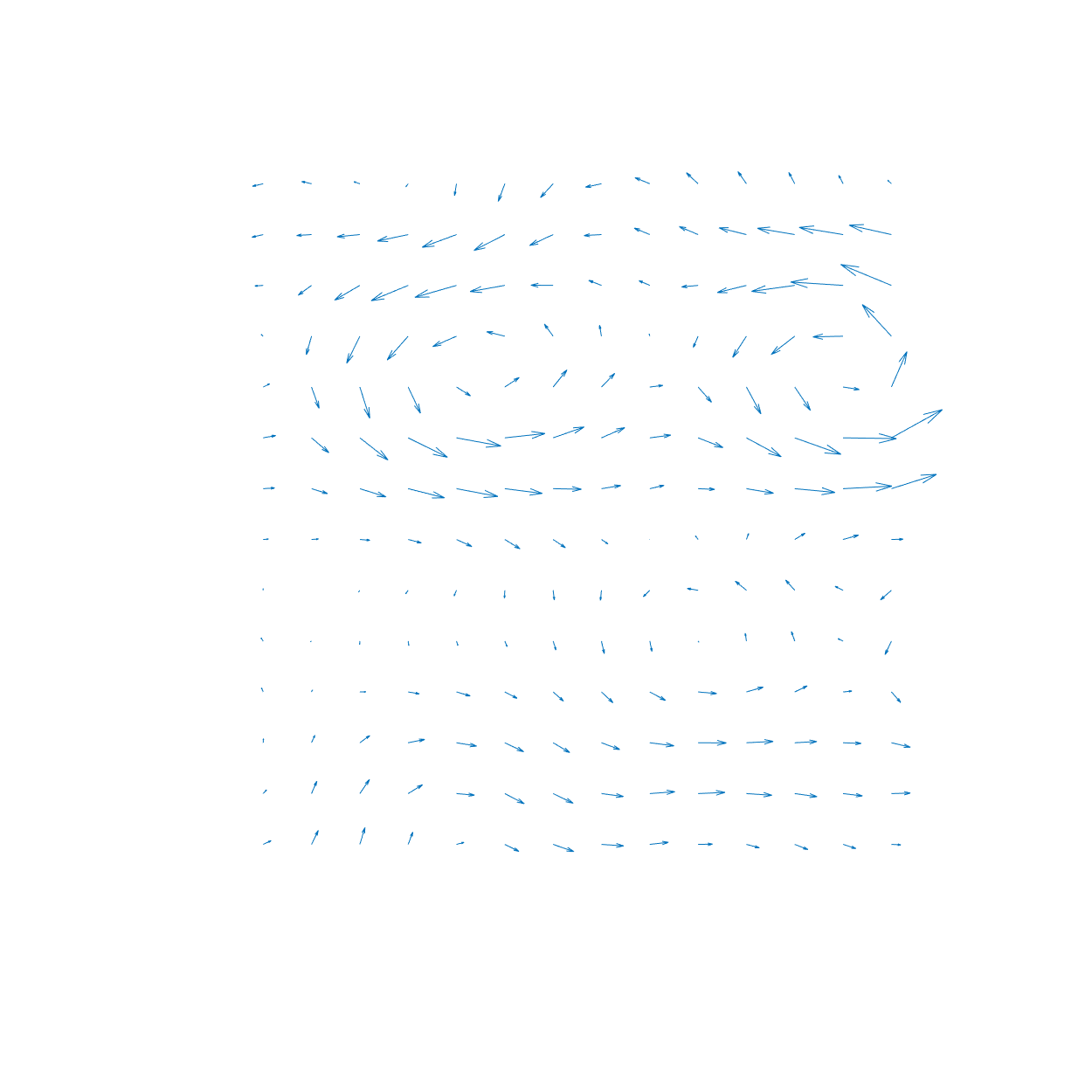

Supplement: S3 MCG raw data 3 — The raw MCG dataset includes category 4 for training and validation. (ZIP) [file pone.0338189.s003.zip › train/4/p10_360_3.png]

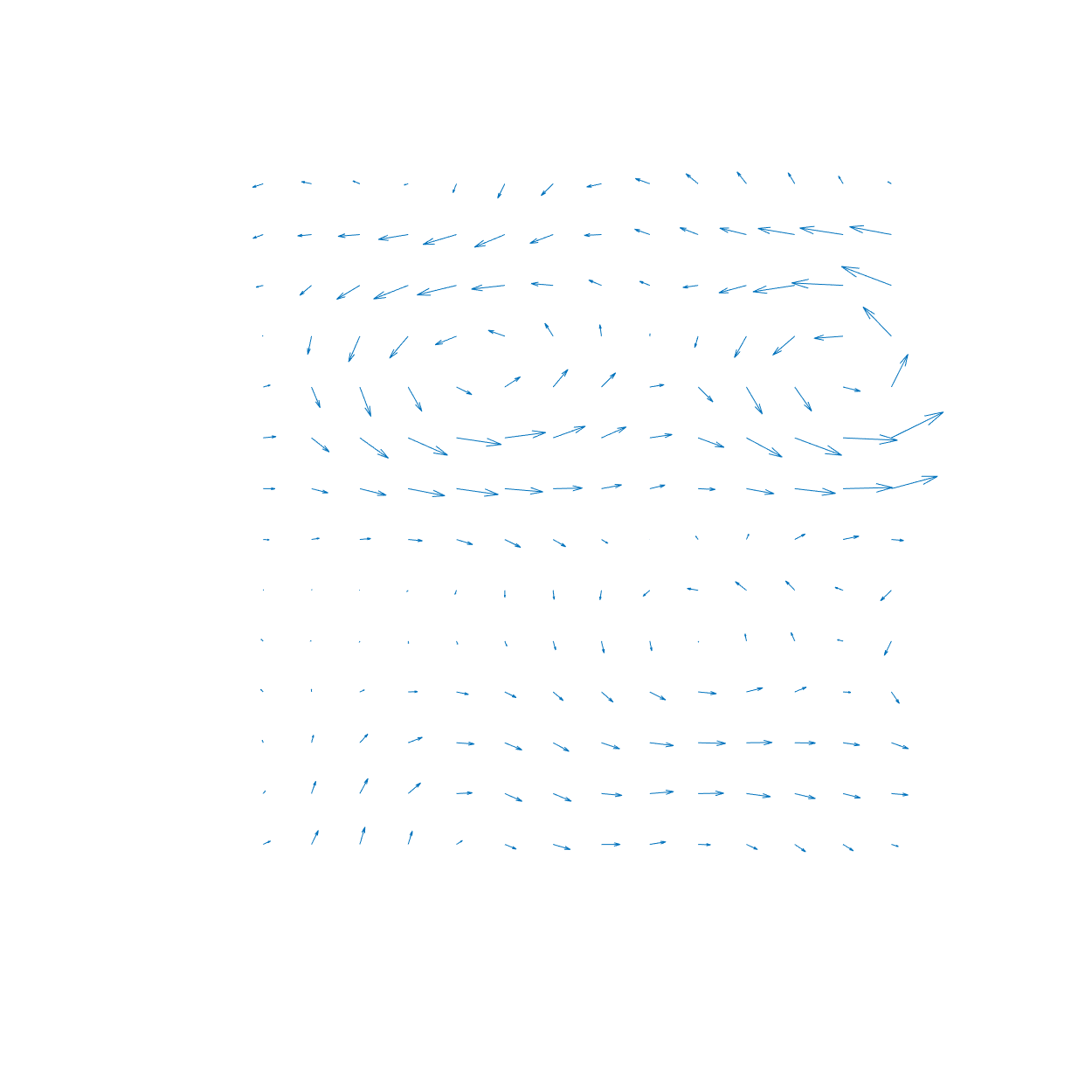

Supplement: S3 MCG raw data 3 — The raw MCG dataset includes category 4 for training and validation. (ZIP) [file pone.0338189.s003.zip › train/4/p10_360_4.png]

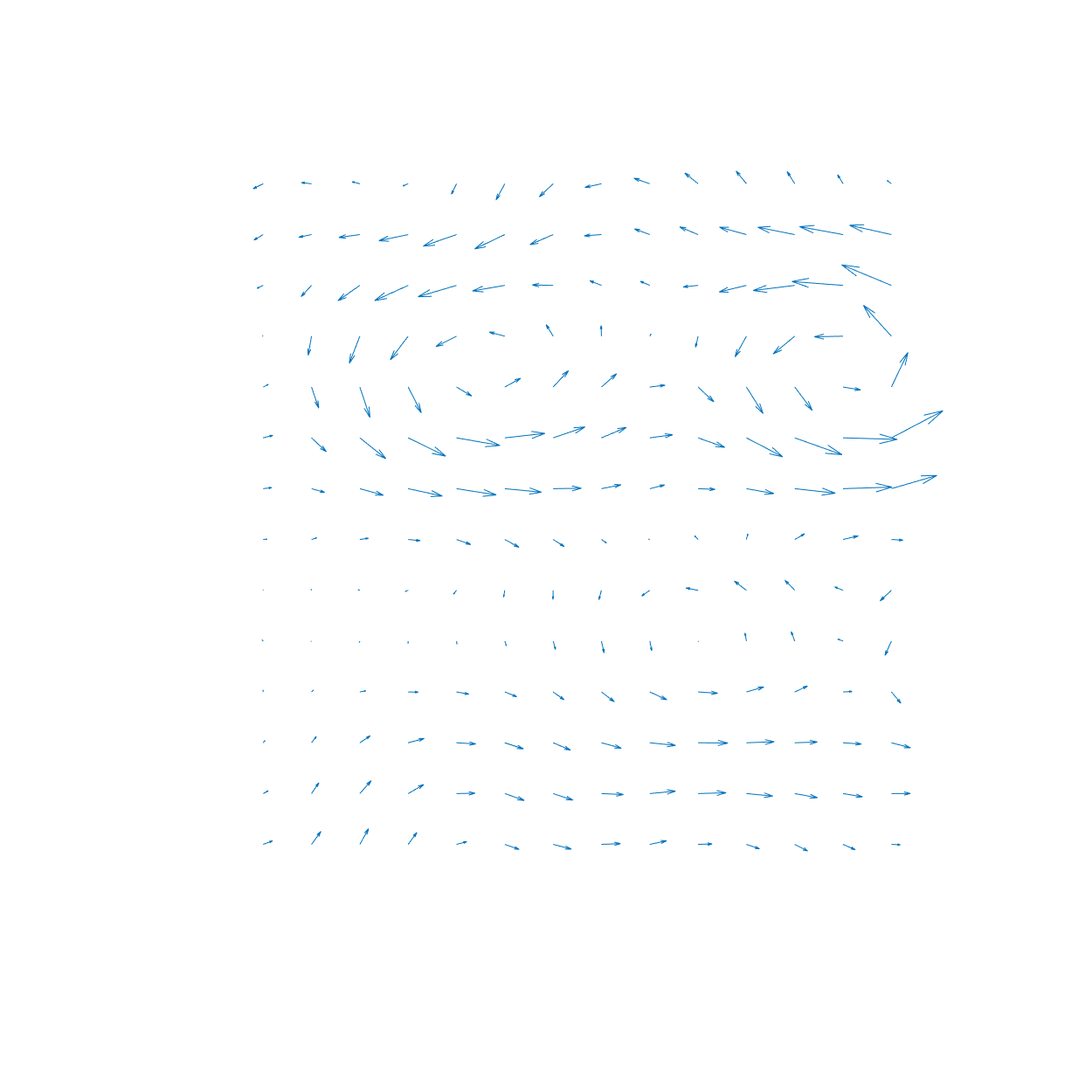

Supplement: S3 MCG raw data 3 — The raw MCG dataset includes category 4 for training and validation. (ZIP) [file pone.0338189.s003.zip › train/4/p10_365_1.png]

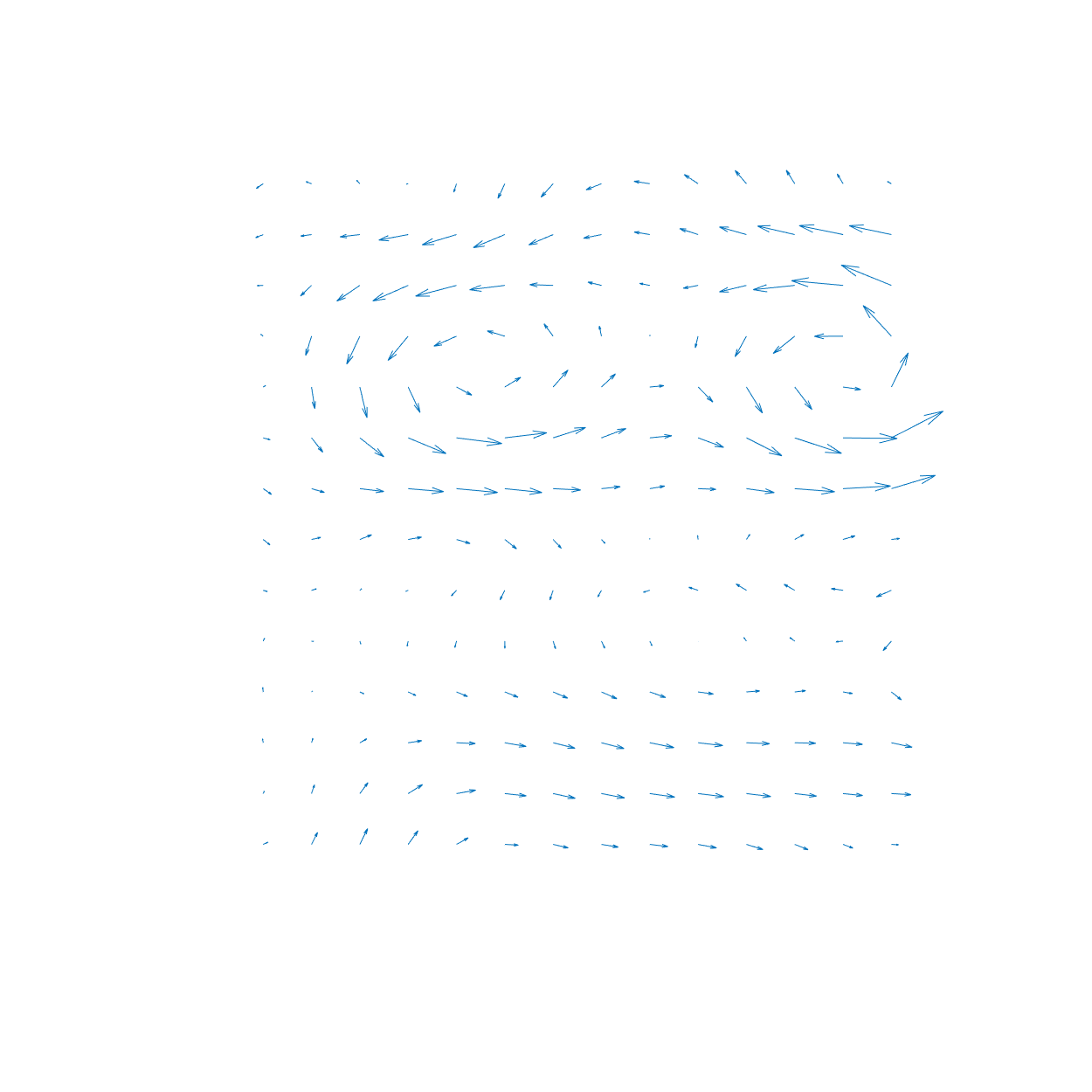

Supplement: S3 MCG raw data 3 — The raw MCG dataset includes category 4 for training and validation. (ZIP) [file pone.0338189.s003.zip › train/4/p10_365_2.png]

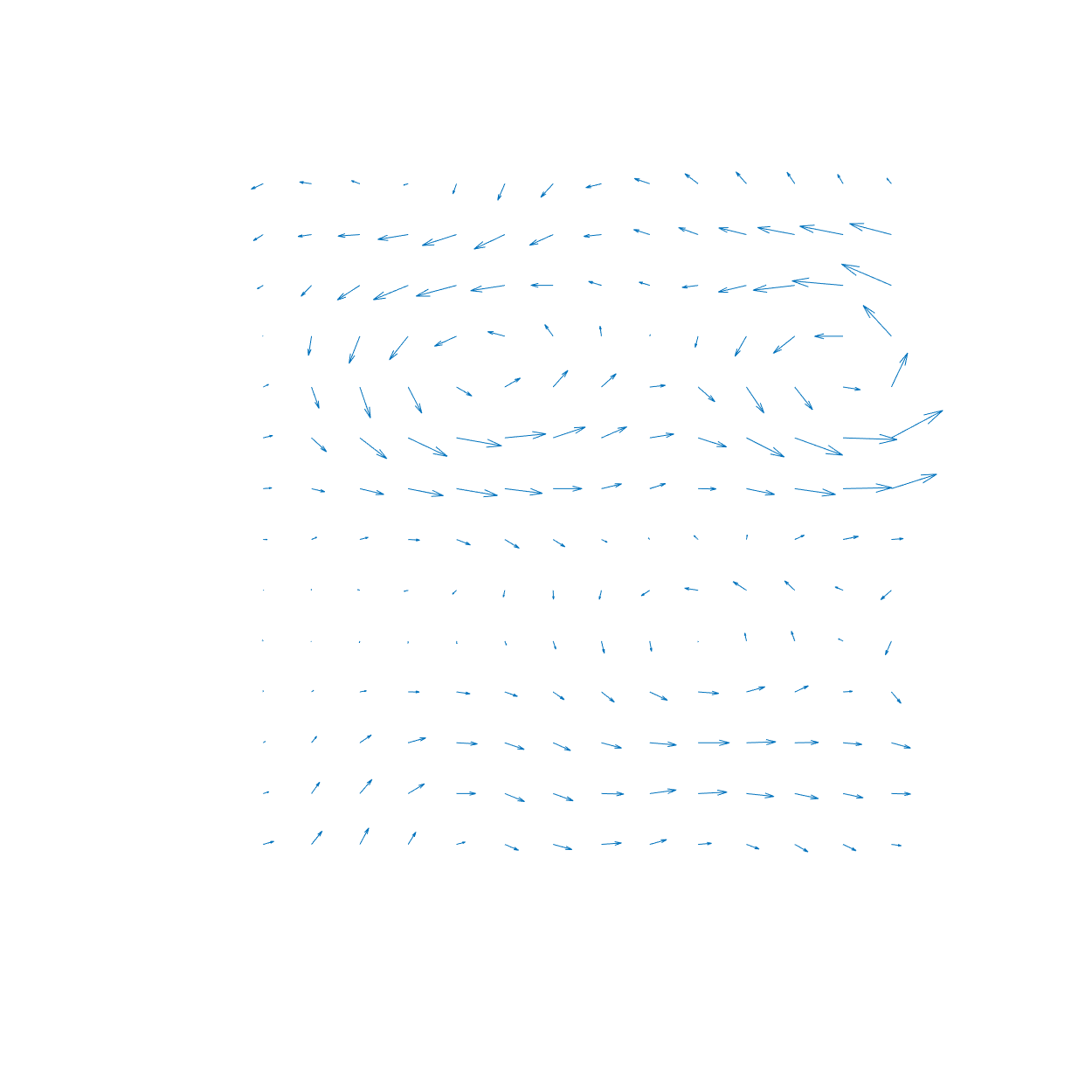

Supplement: S3 MCG raw data 3 — The raw MCG dataset includes category 4 for training and validation. (ZIP) [file pone.0338189.s003.zip › train/4/p10_365_3.png]

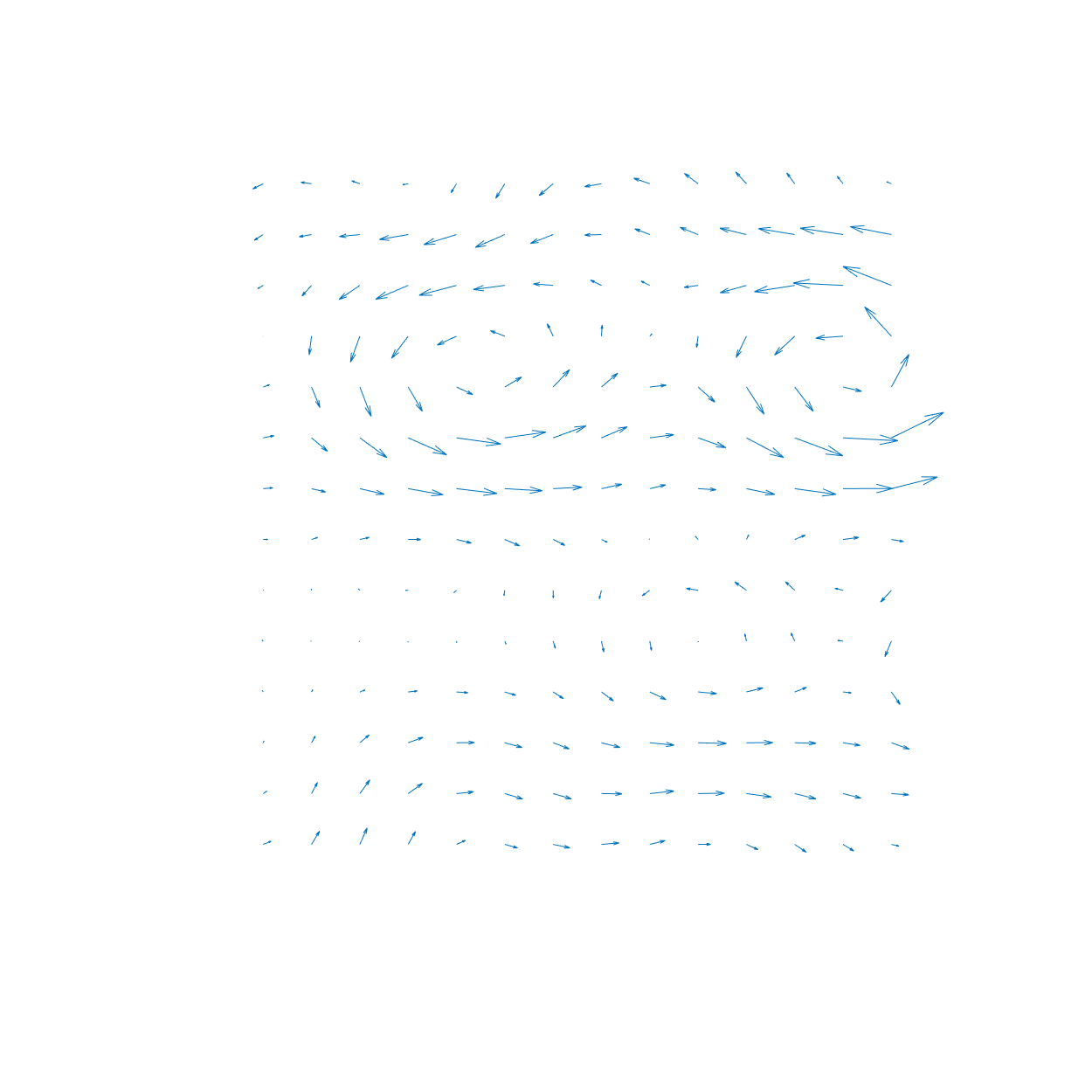

Supplement: S3 MCG raw data 3 — The raw MCG dataset includes category 4 for training and validation. (ZIP) [file pone.0338189.s003.zip › train/4/p10_365_4.png]

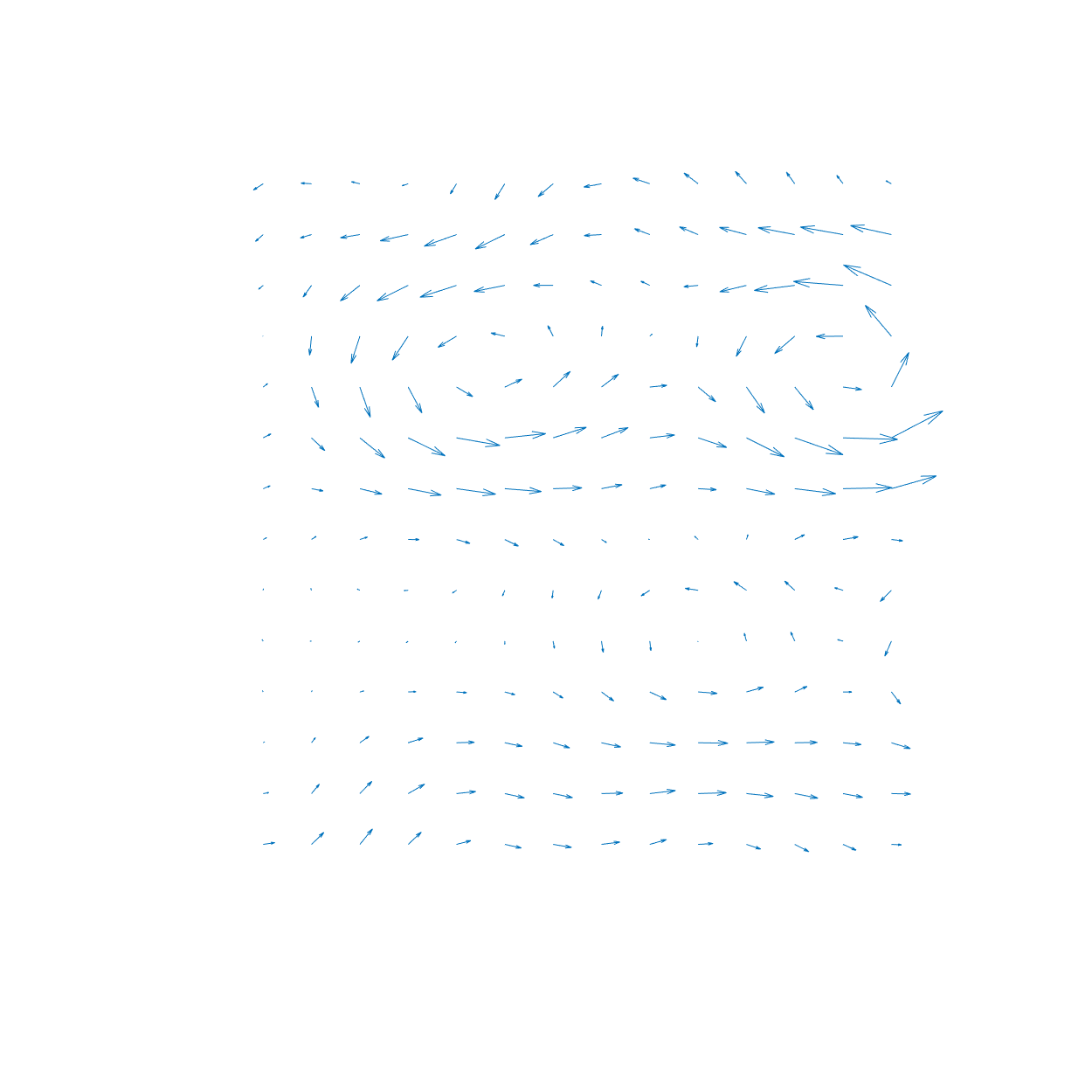

Supplement: S3 MCG raw data 3 — The raw MCG dataset includes category 4 for training and validation. (ZIP) [file pone.0338189.s003.zip › train/4/p10_370_1.png]

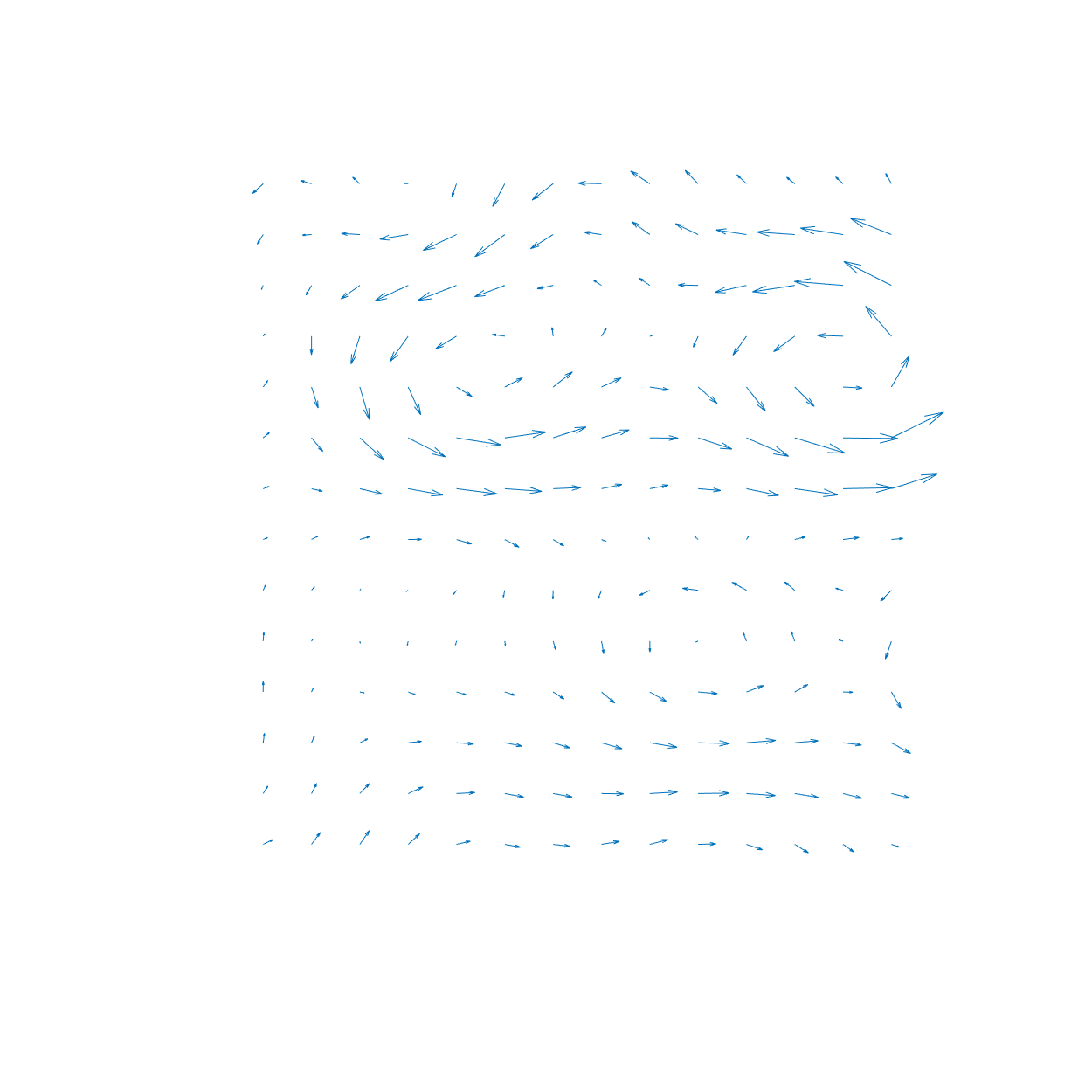

Supplement: S3 MCG raw data 3 — The raw MCG dataset includes category 4 for training and validation. (ZIP) [file pone.0338189.s003.zip › train/4/p10_370_2.png]

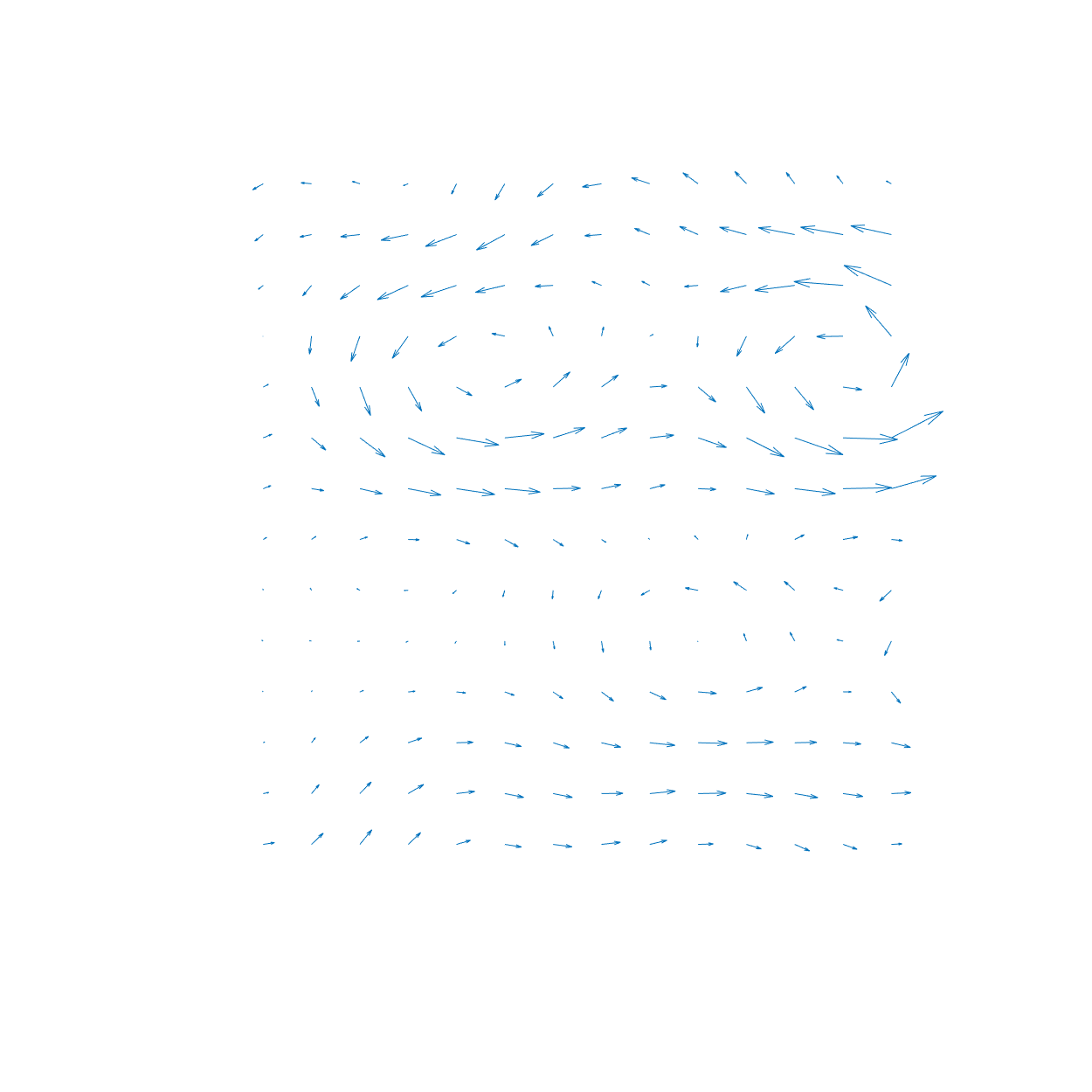

Supplement: S3 MCG raw data 3 — The raw MCG dataset includes category 4 for training and validation. (ZIP) [file pone.0338189.s003.zip › train/4/p10_370_3.png]

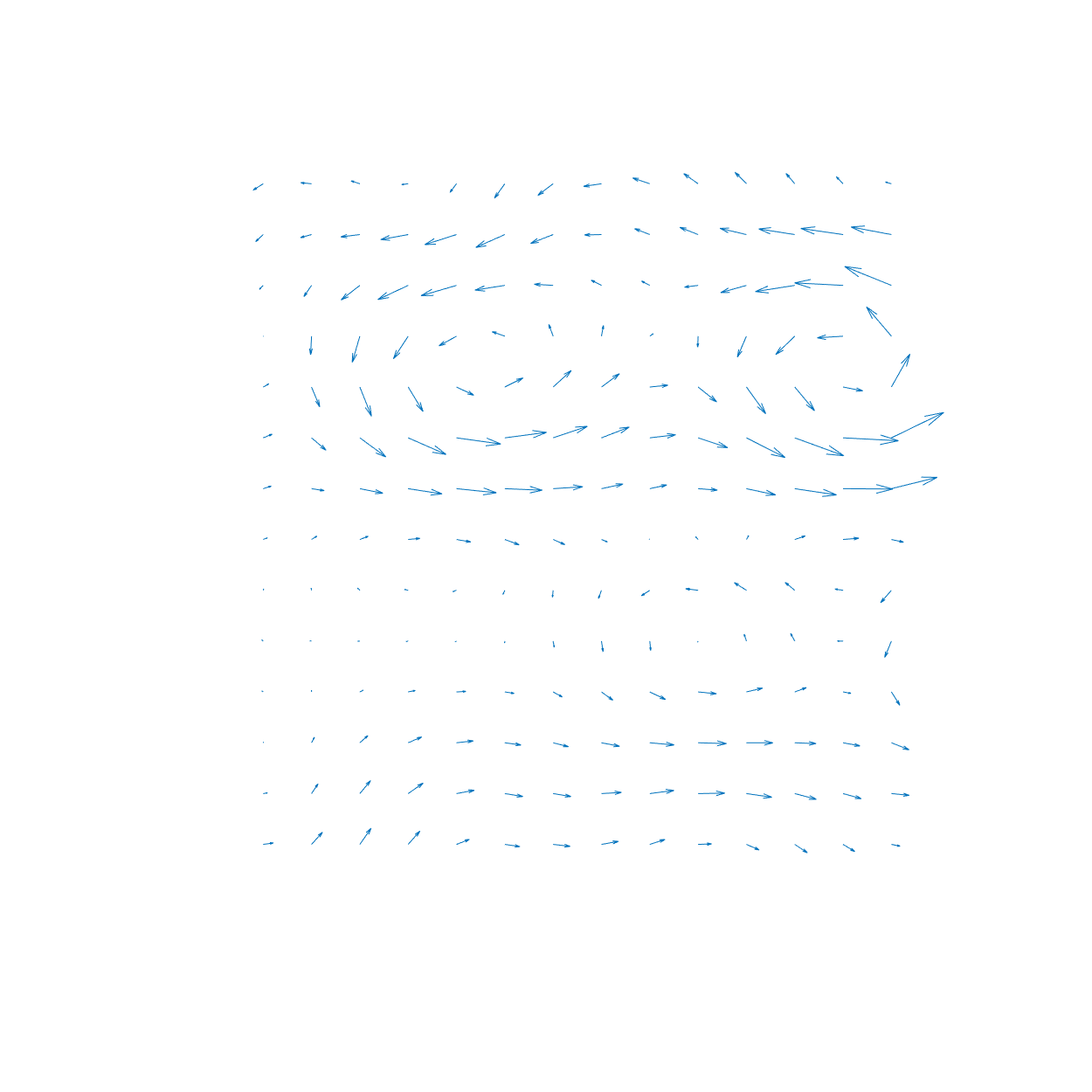

Supplement: S3 MCG raw data 3 — The raw MCG dataset includes category 4 for training and validation. (ZIP) [file pone.0338189.s003.zip › train/4/p10_370_4.png]

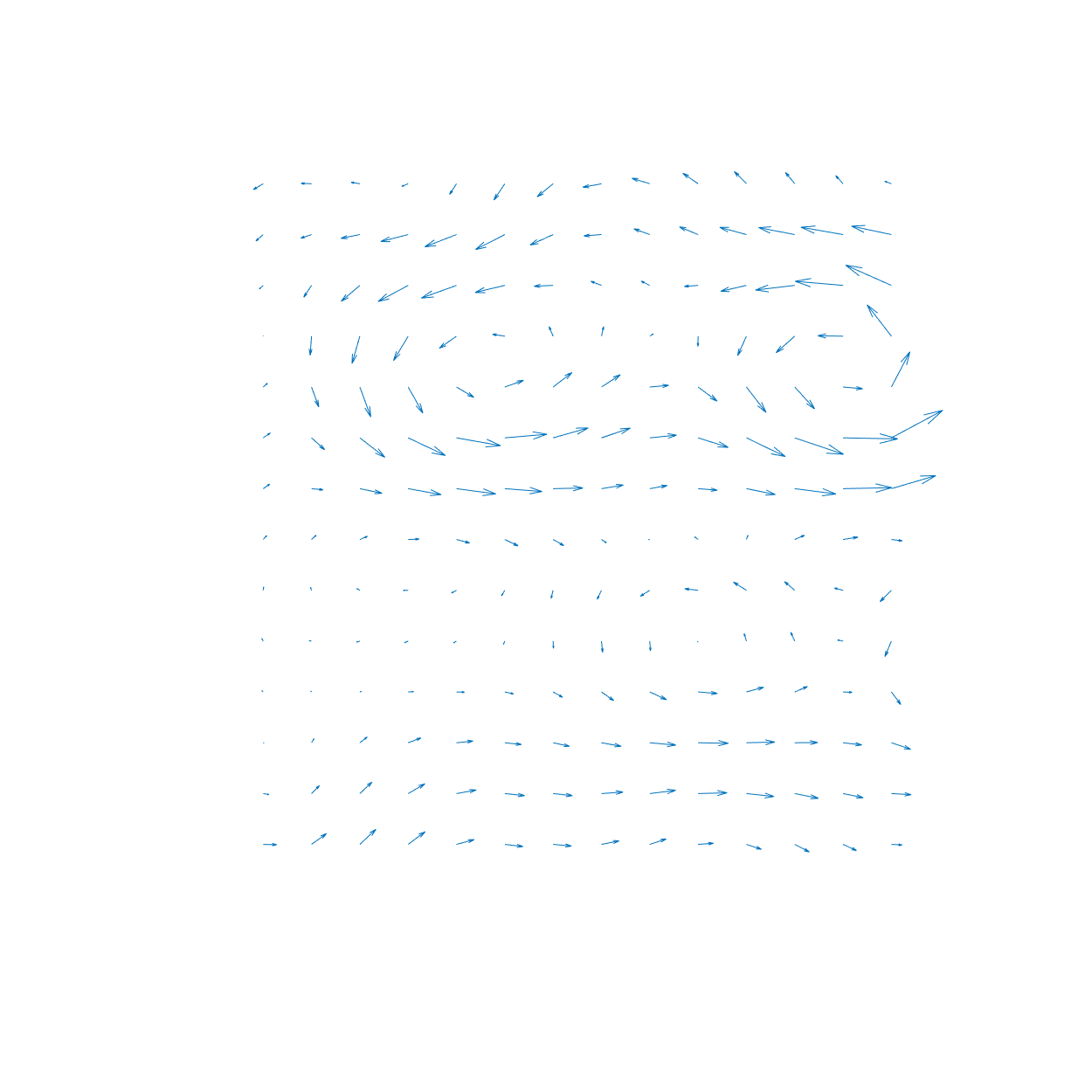

Supplement: S3 MCG raw data 3 — The raw MCG dataset includes category 4 for training and validation. (ZIP) [file pone.0338189.s003.zip › train/4/p10_375_1.png]

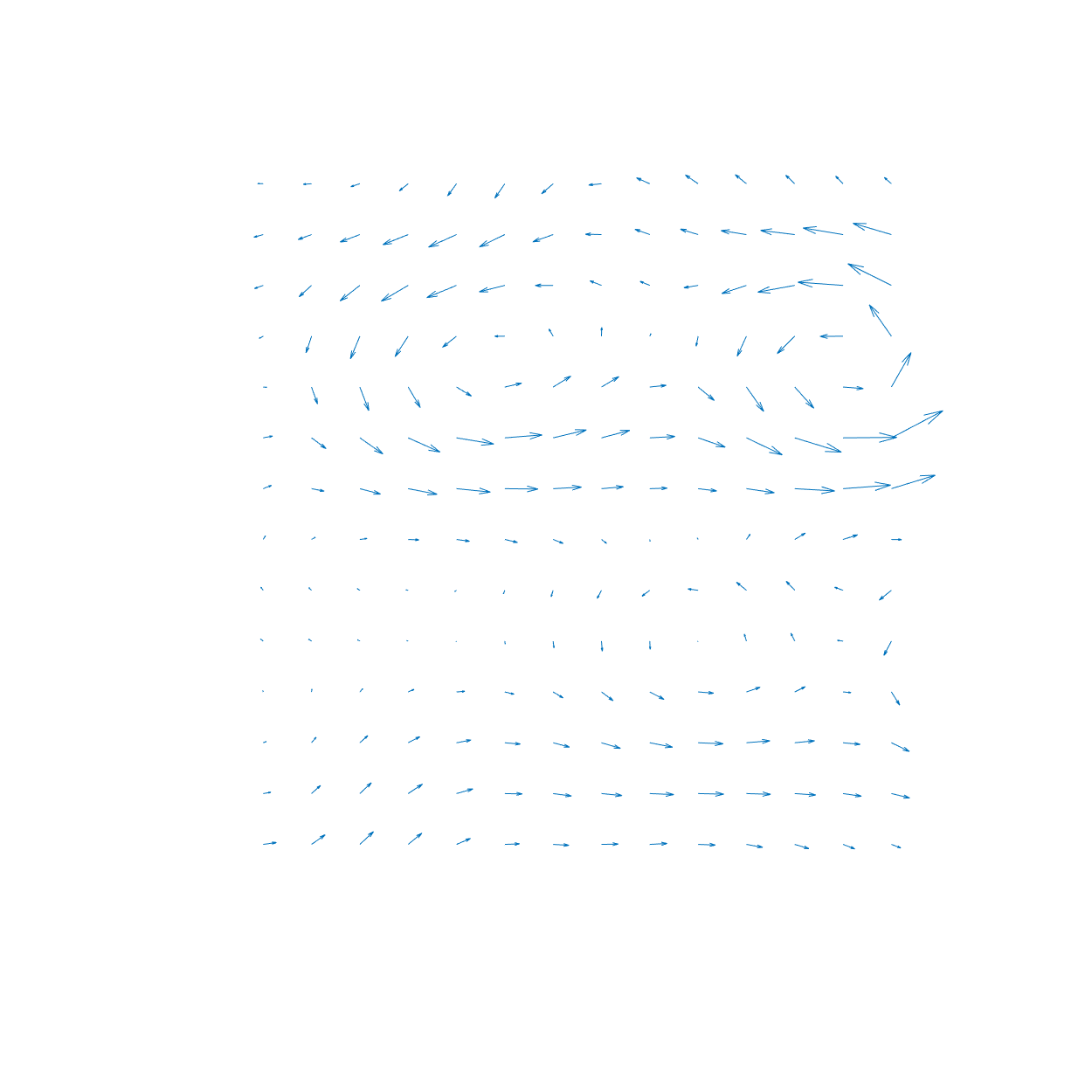

Supplement: S3 MCG raw data 3 — The raw MCG dataset includes category 4 for training and validation. (ZIP) [file pone.0338189.s003.zip › train/4/p10_375_2.png]

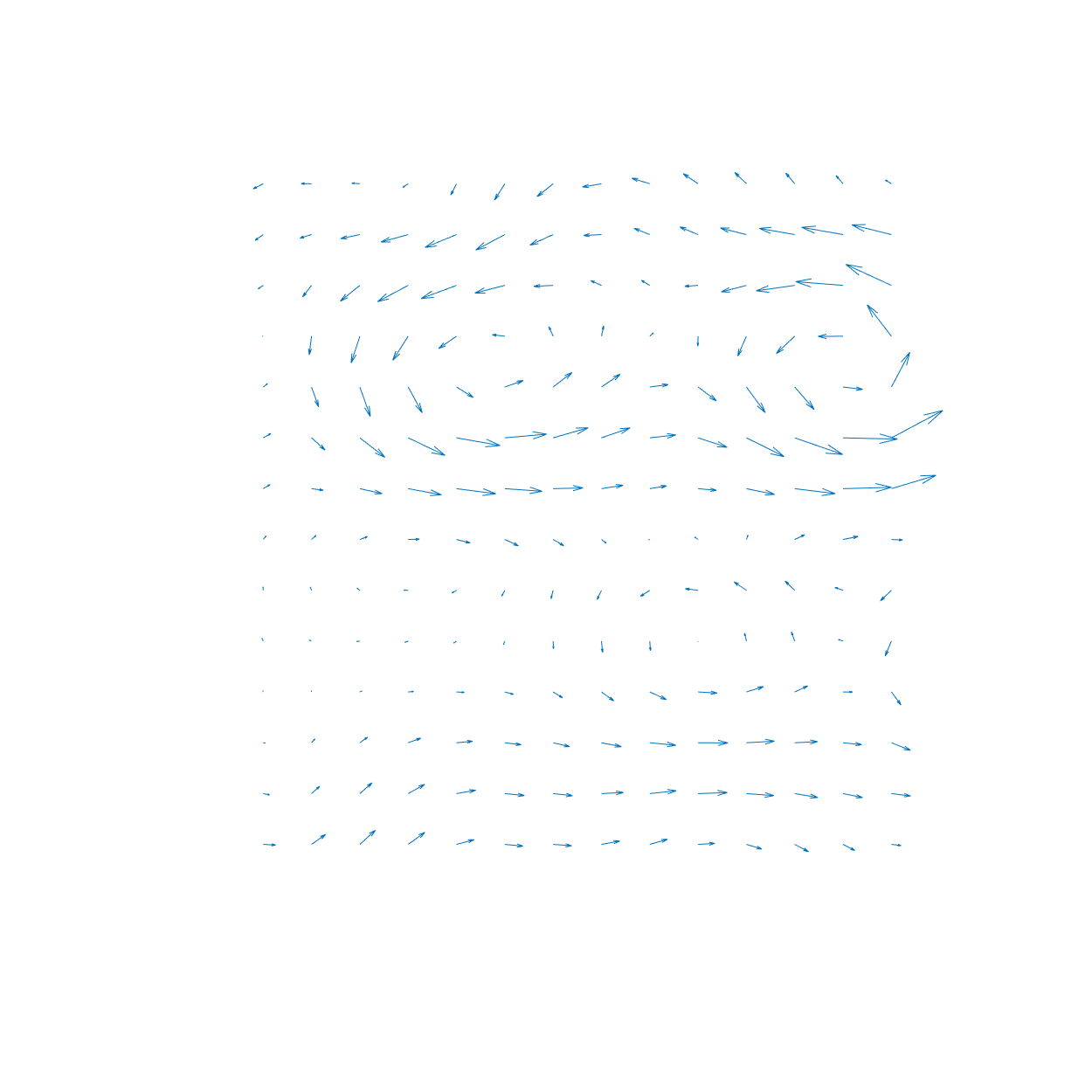

Supplement: S3 MCG raw data 3 — The raw MCG dataset includes category 4 for training and validation. (ZIP) [file pone.0338189.s003.zip › train/4/p10_375_3.png]

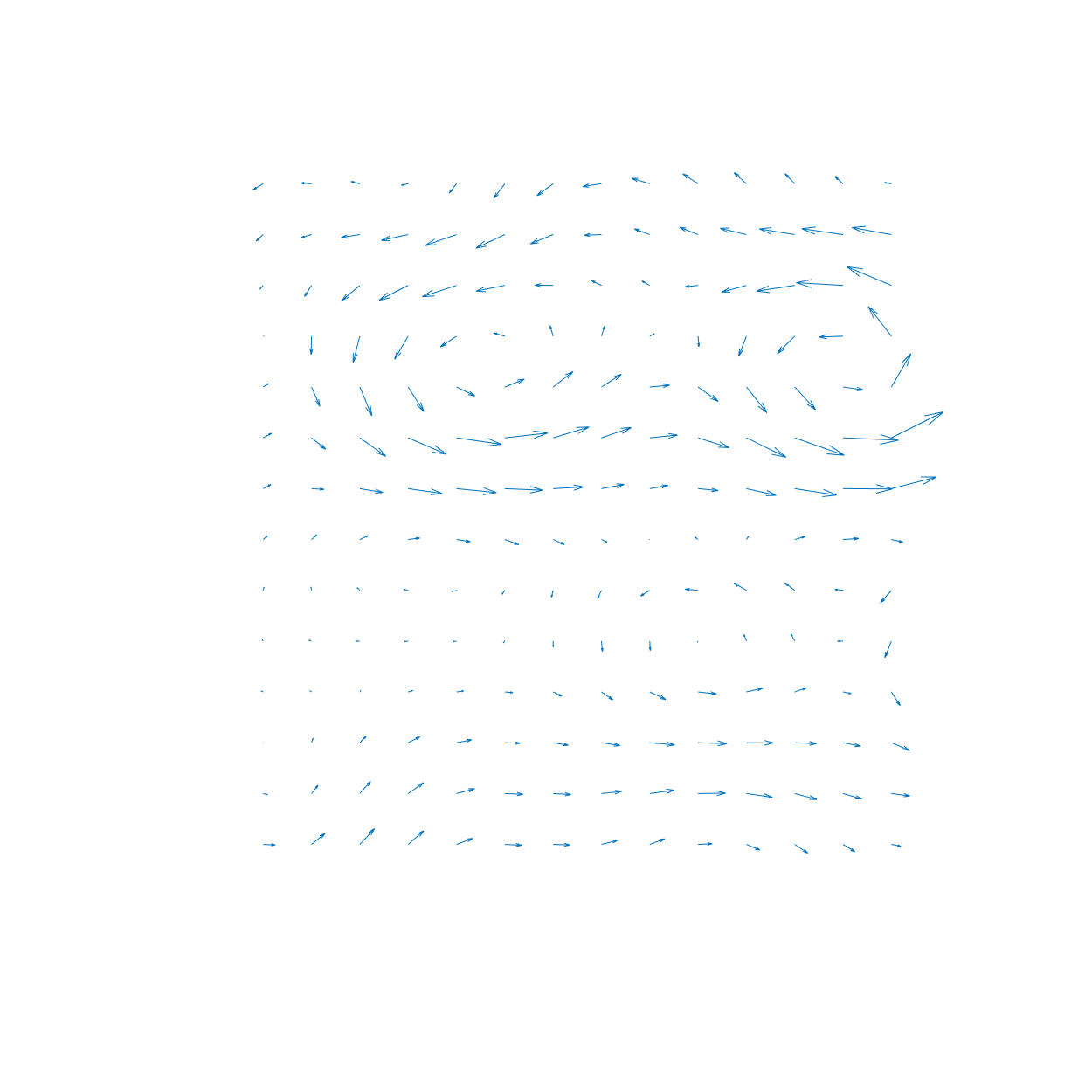

Supplement: S3 MCG raw data 3 — The raw MCG dataset includes category 4 for training and validation. (ZIP) [file pone.0338189.s003.zip › train/4/p10_375_4.png]

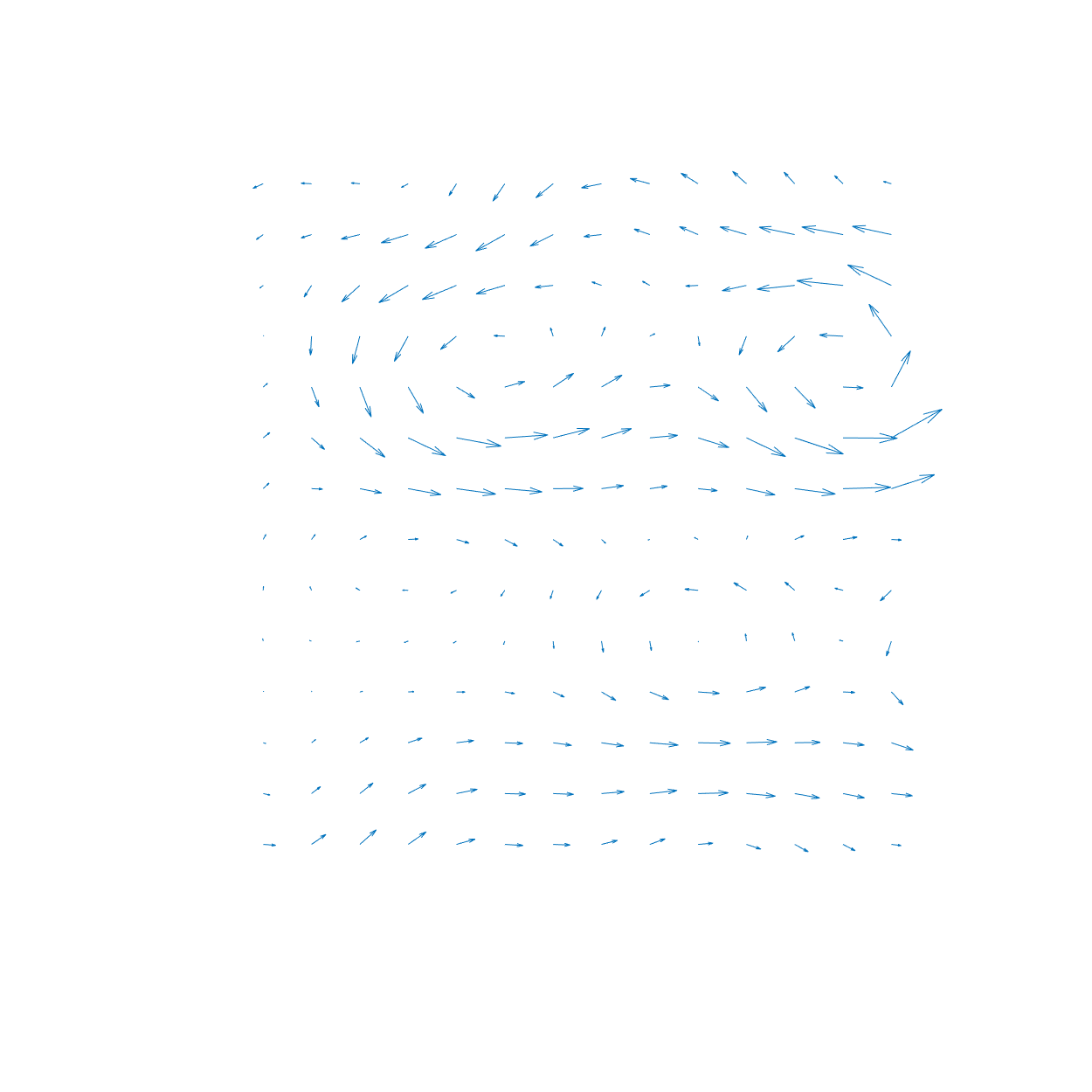

Supplement: S3 MCG raw data 3 — The raw MCG dataset includes category 4 for training and validation. (ZIP) [file pone.0338189.s003.zip › train/4/p10_380_1.png]

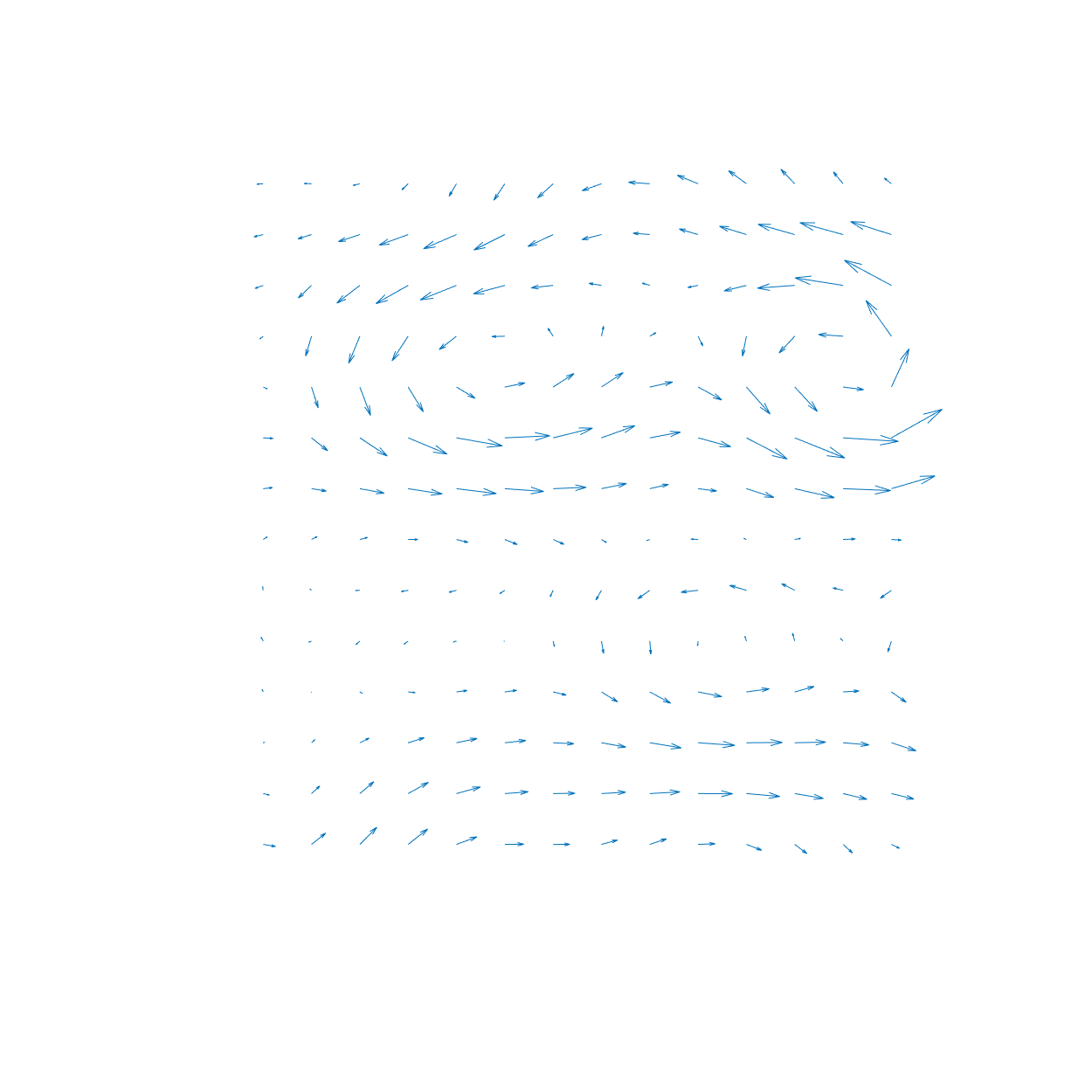

Supplement: S3 MCG raw data 3 — The raw MCG dataset includes category 4 for training and validation. (ZIP) [file pone.0338189.s003.zip › train/4/p10_380_2.png]

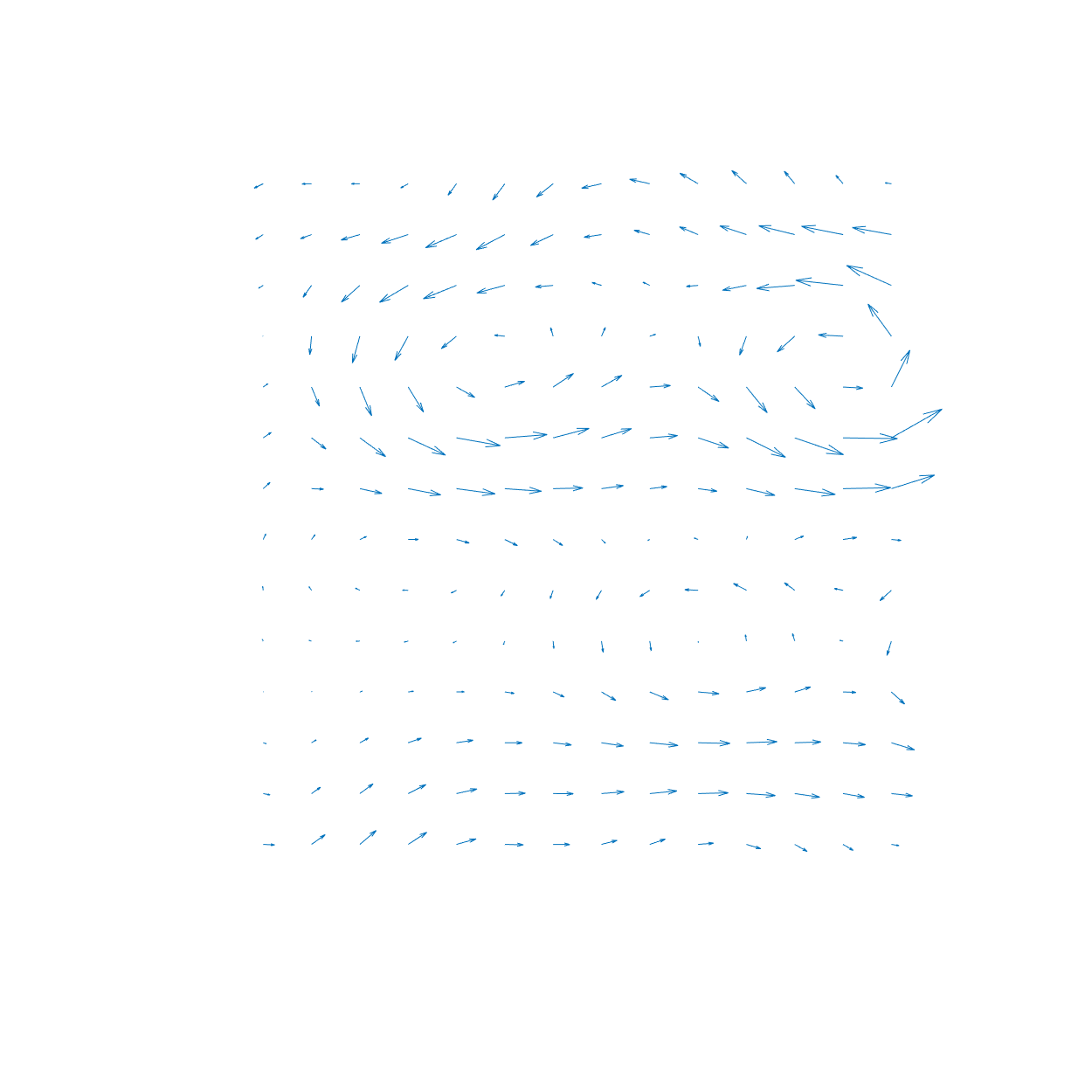

Supplement: S3 MCG raw data 3 — The raw MCG dataset includes category 4 for training and validation. (ZIP) [file pone.0338189.s003.zip › train/4/p10_380_3.png]

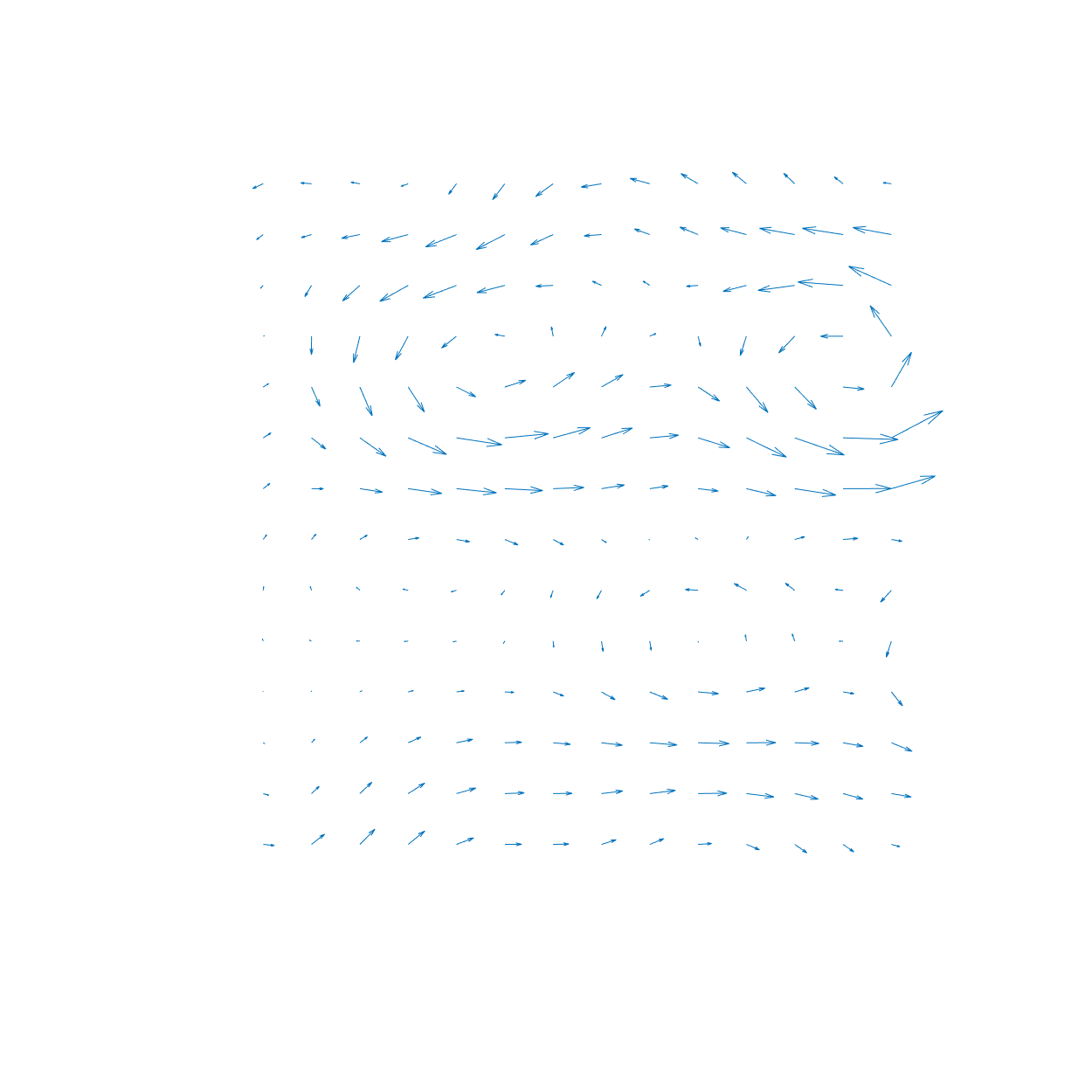

Supplement: S3 MCG raw data 3 — The raw MCG dataset includes category 4 for training and validation. (ZIP) [file pone.0338189.s003.zip › train/4/p10_380_4.png]

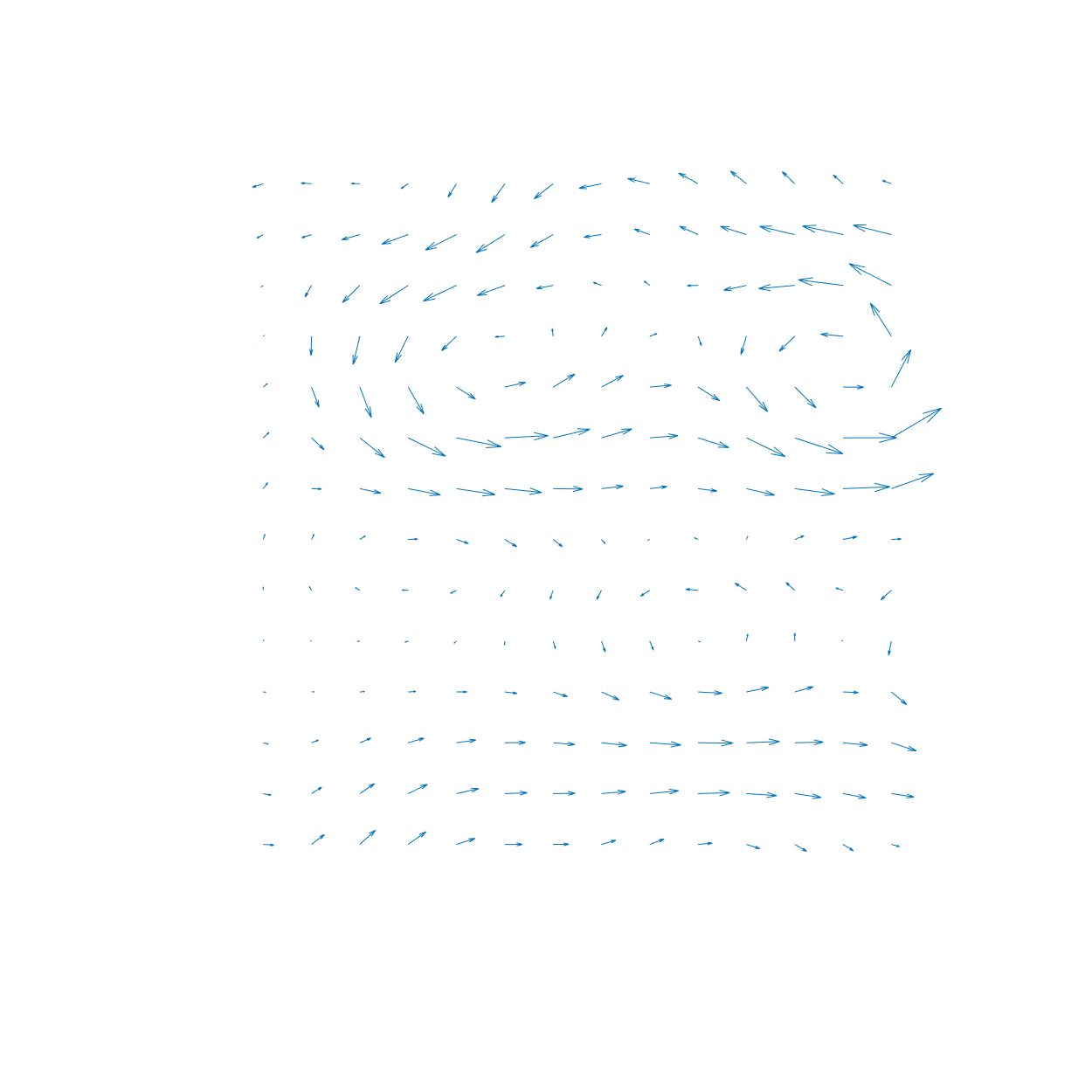

Supplement: S3 MCG raw data 3 — The raw MCG dataset includes category 4 for training and validation. (ZIP) [file pone.0338189.s003.zip › train/4/p10_385_1.png]

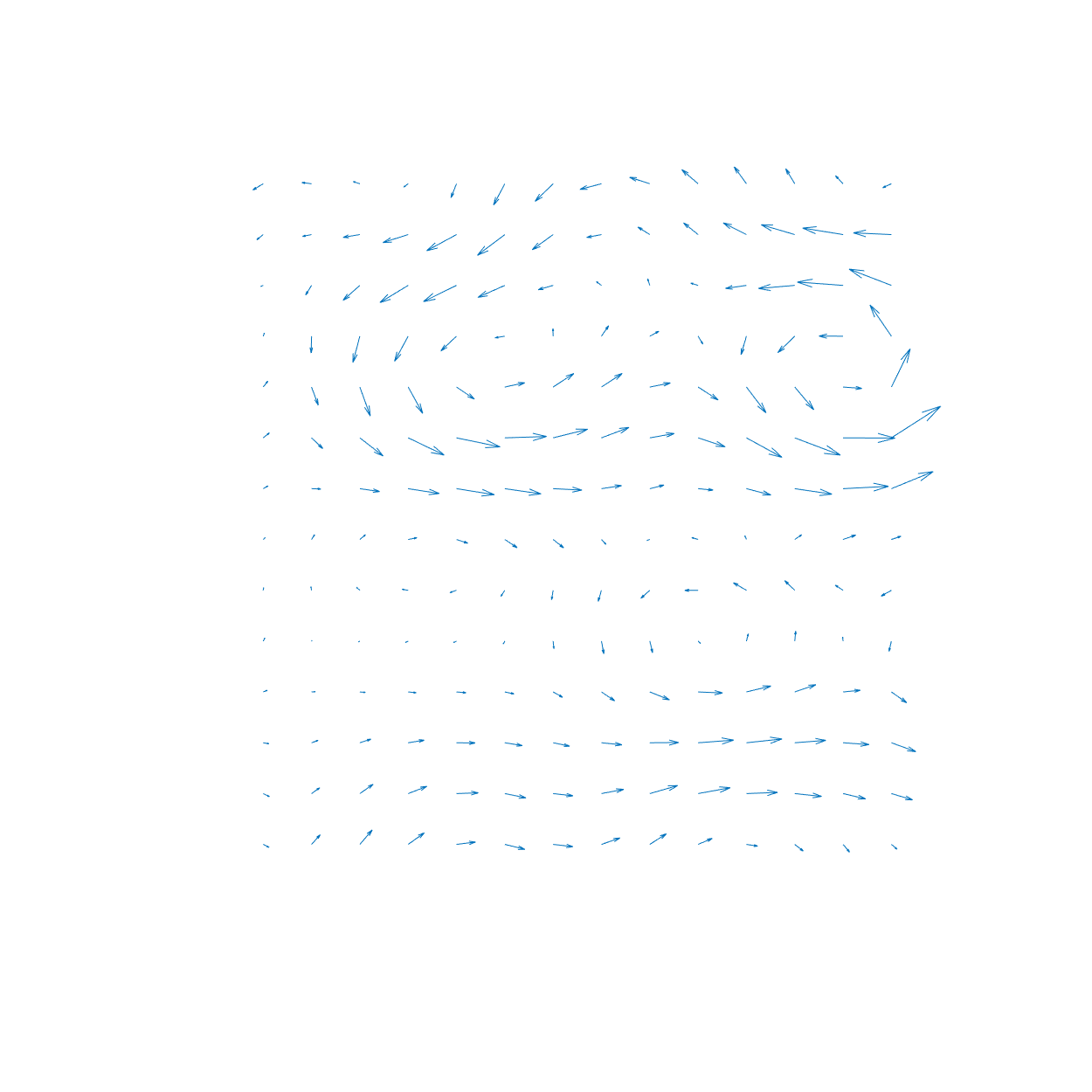

Supplement: S3 MCG raw data 3 — The raw MCG dataset includes category 4 for training and validation. (ZIP) [file pone.0338189.s003.zip › train/4/p10_385_2.png]

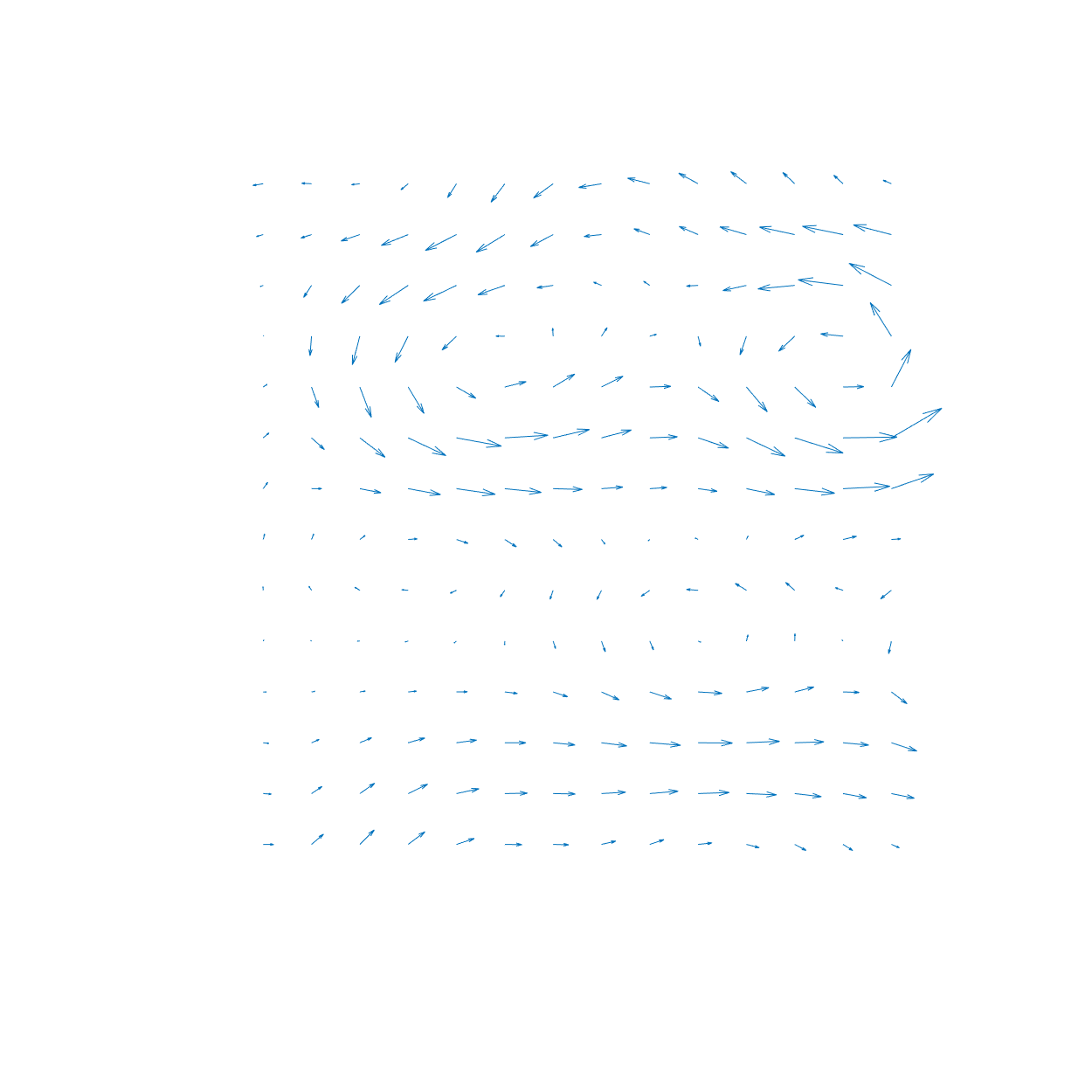

Supplement: S3 MCG raw data 3 — The raw MCG dataset includes category 4 for training and validation. (ZIP) [file pone.0338189.s003.zip › train/4/p10_385_3.png]

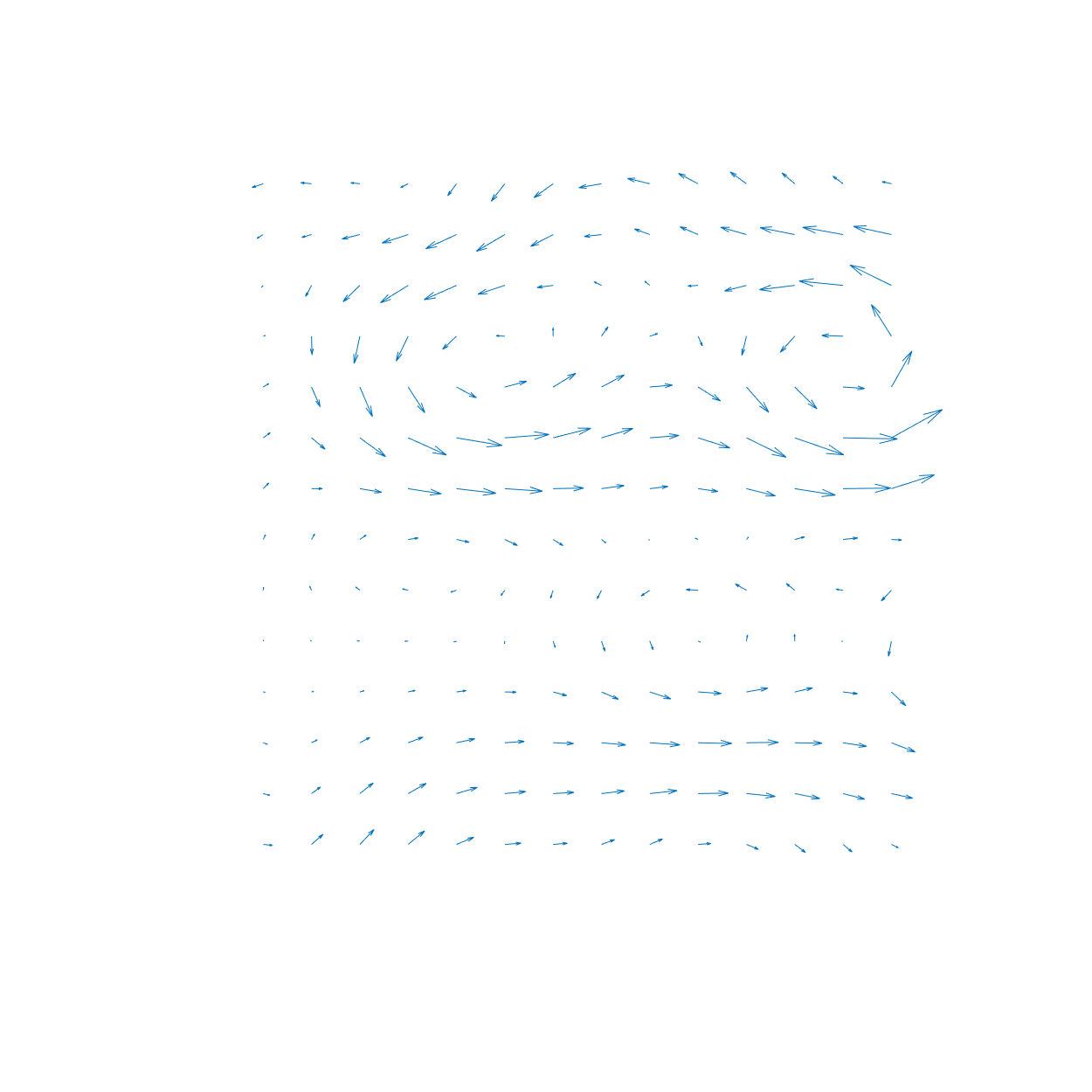

Supplement: S3 MCG raw data 3 — The raw MCG dataset includes category 4 for training and validation. (ZIP) [file pone.0338189.s003.zip › train/4/p10_385_4.png]

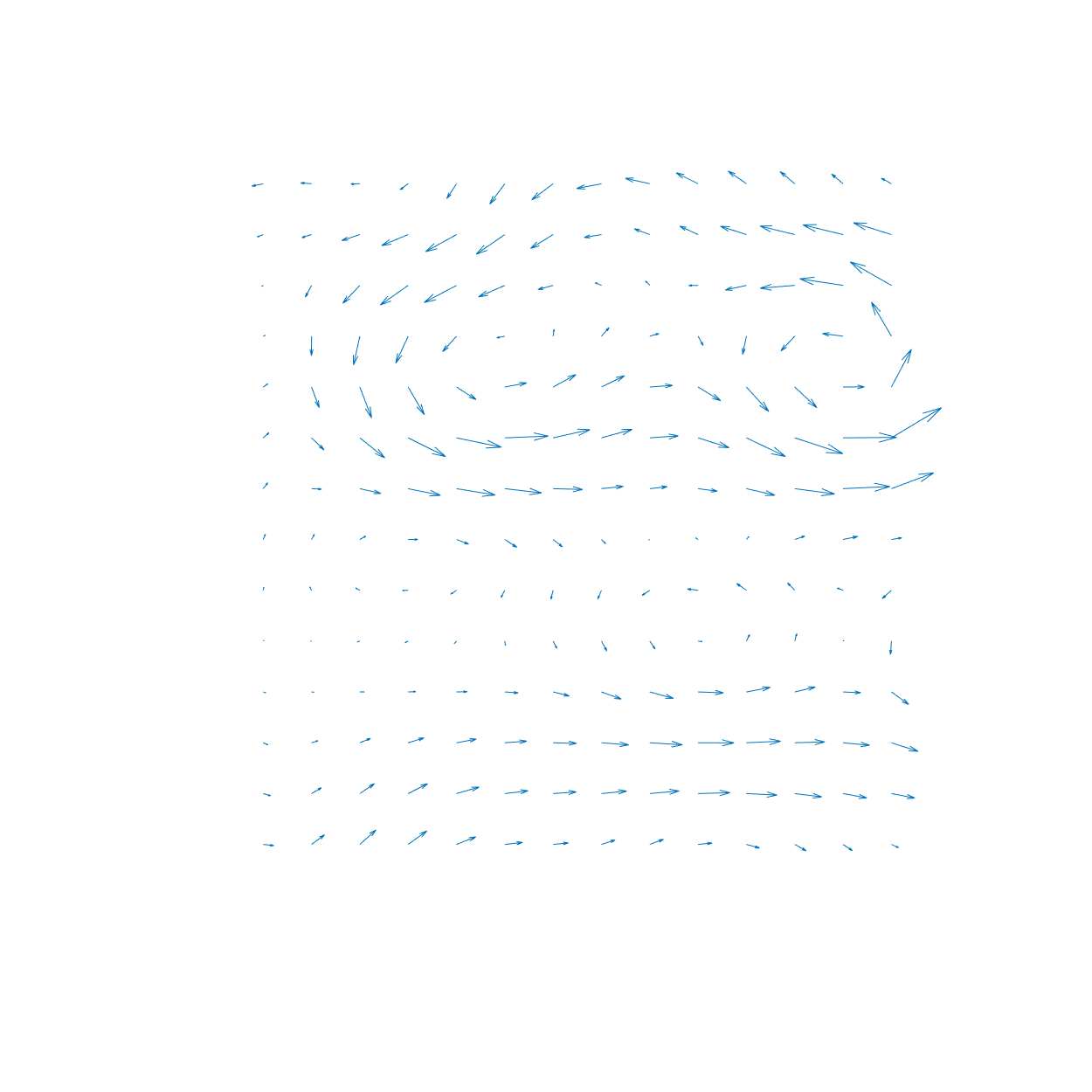

Supplement: S3 MCG raw data 3 — The raw MCG dataset includes category 4 for training and validation. (ZIP) [file pone.0338189.s003.zip › train/4/p10_390_1.png]

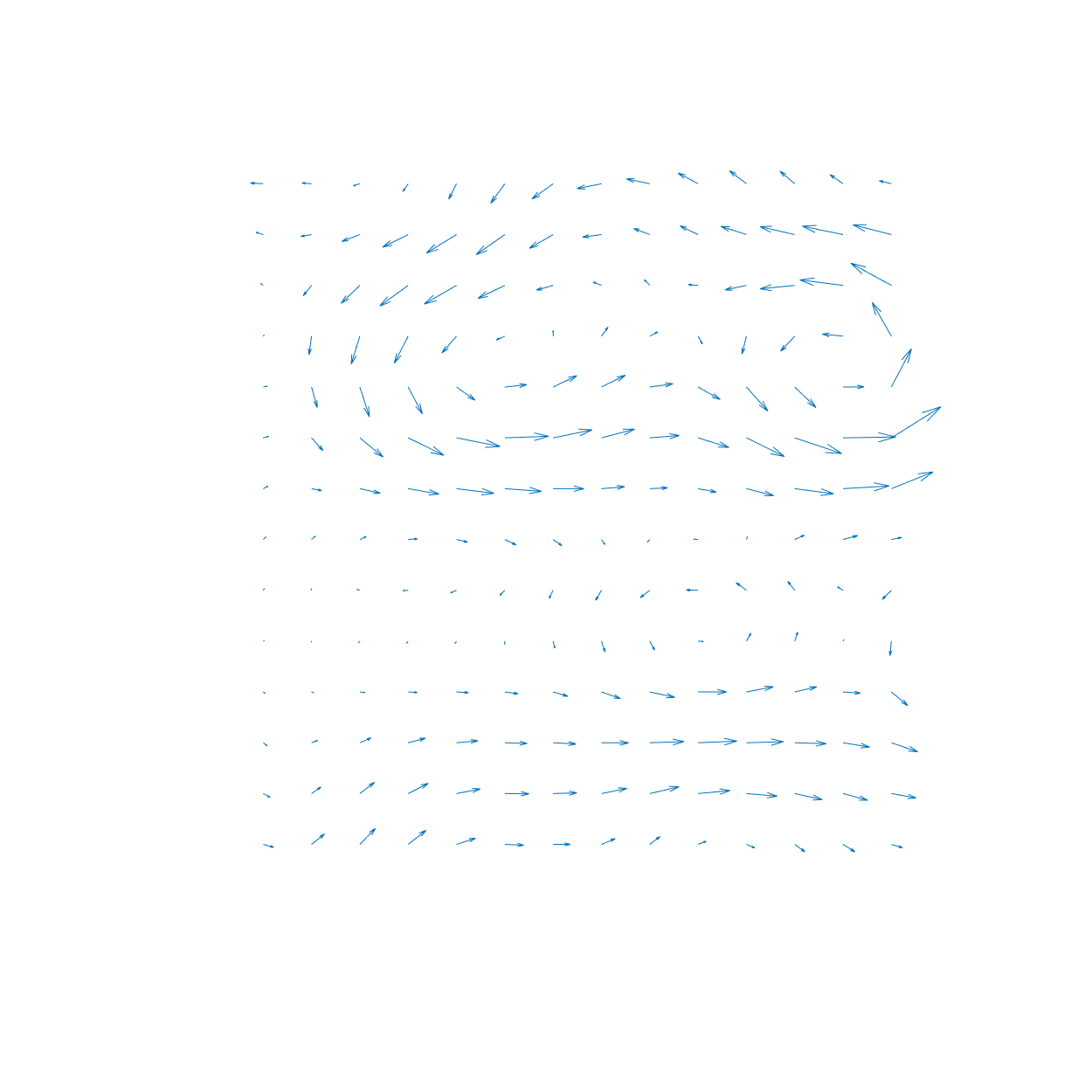

Supplement: S3 MCG raw data 3 — The raw MCG dataset includes category 4 for training and validation. (ZIP) [file pone.0338189.s003.zip › train/4/p10_390_2.png]

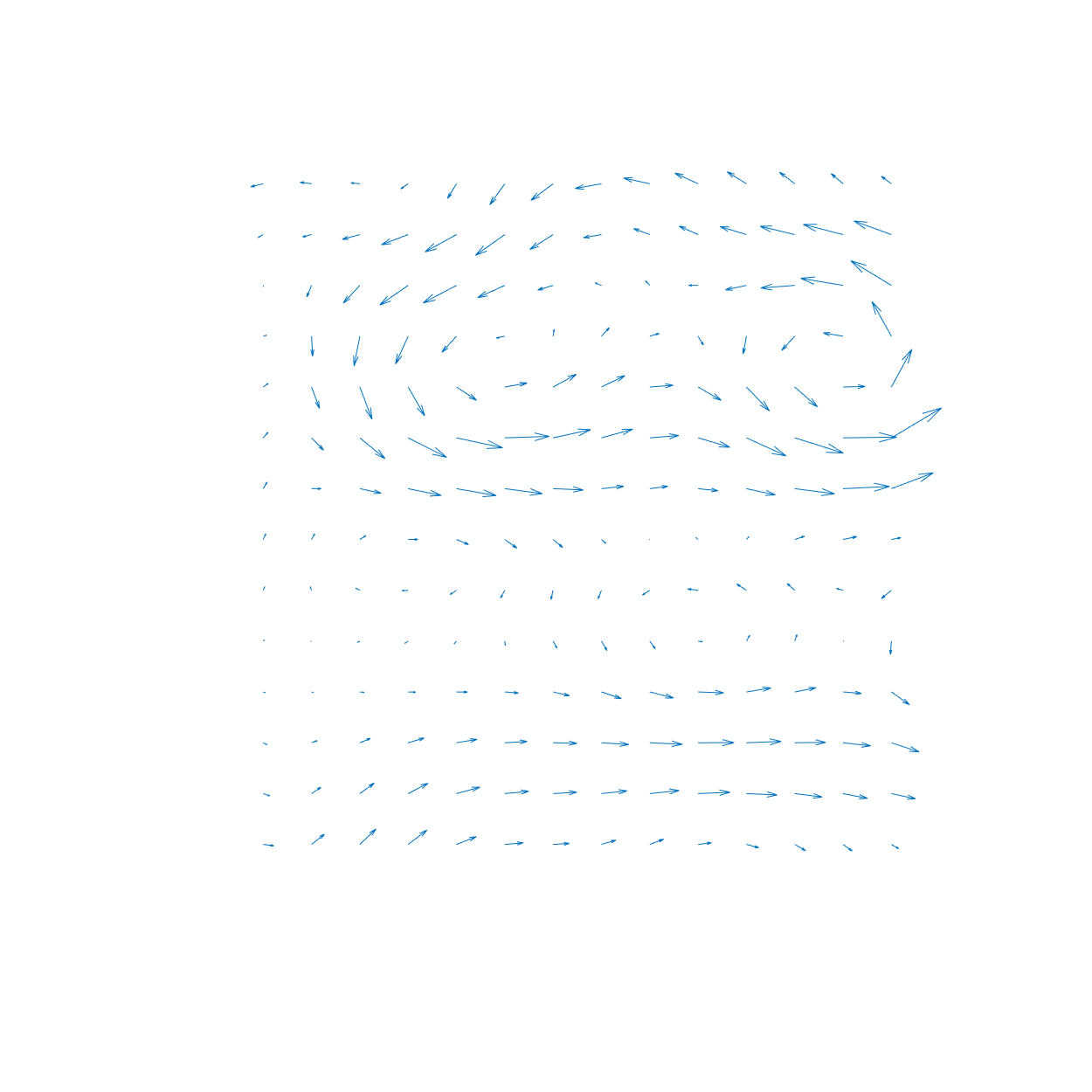

Supplement: S3 MCG raw data 3 — The raw MCG dataset includes category 4 for training and validation. (ZIP) [file pone.0338189.s003.zip › train/4/p10_390_3.png]

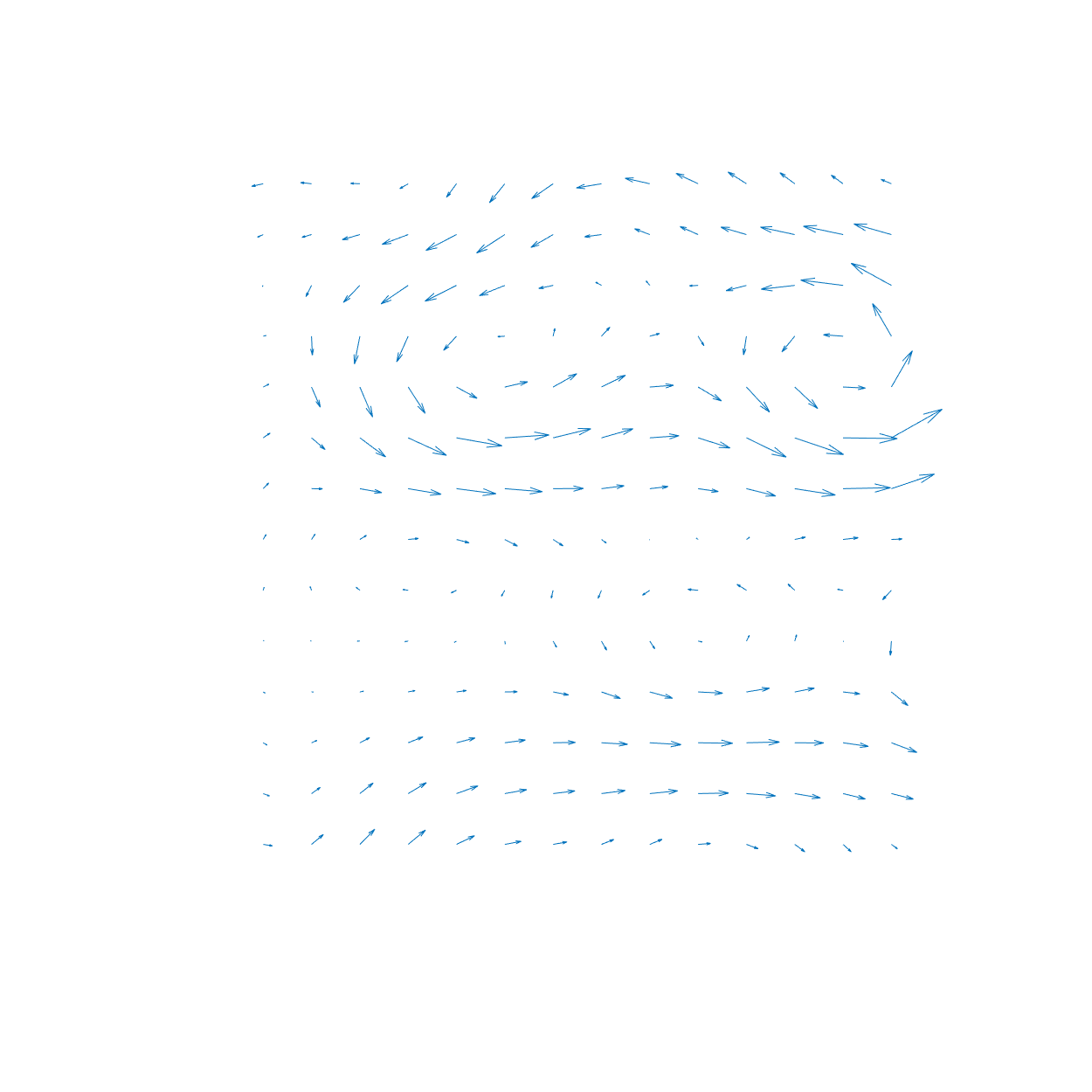

Supplement: S3 MCG raw data 3 — The raw MCG dataset includes category 4 for training and validation. (ZIP) [file pone.0338189.s003.zip › train/4/p10_390_4.png]

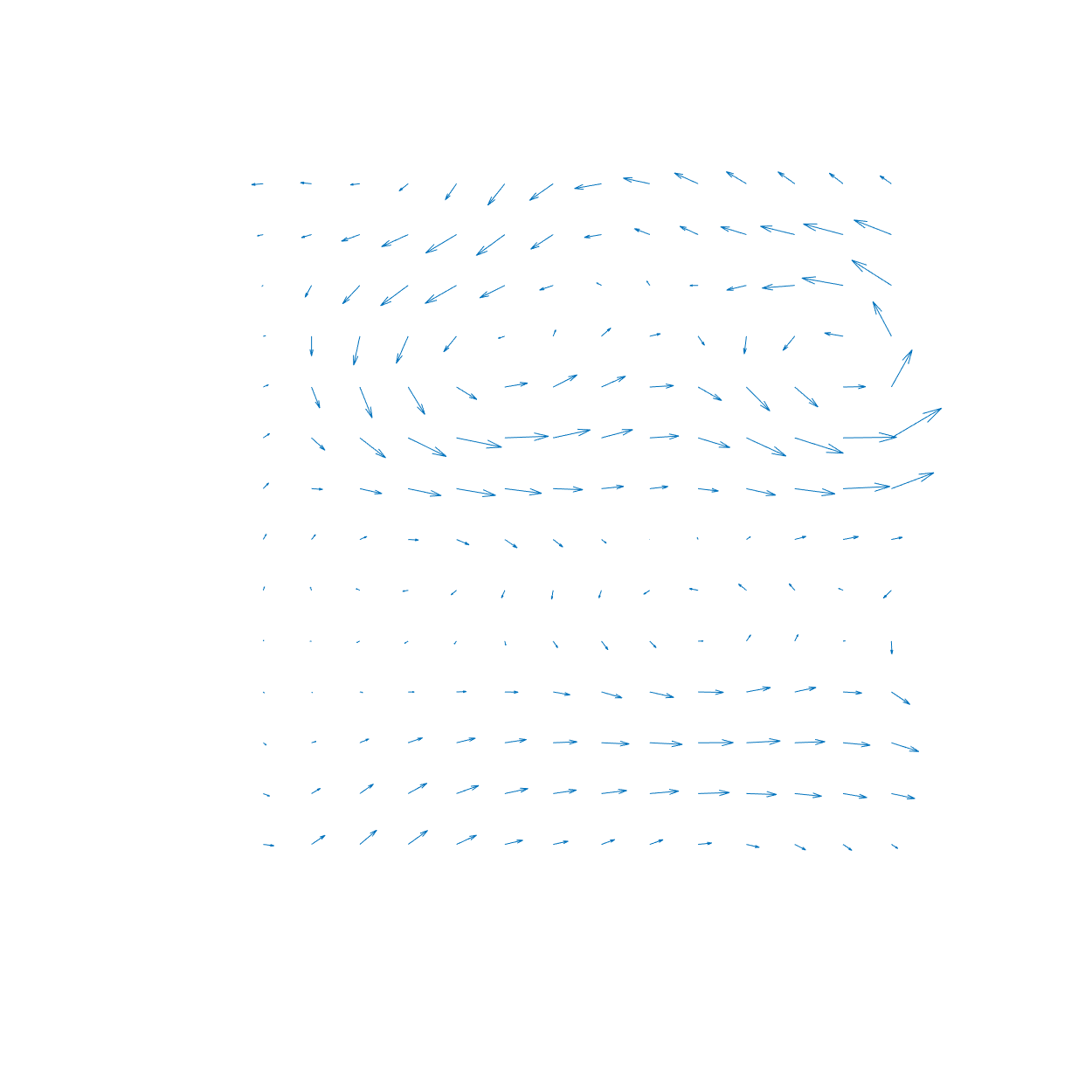

Supplement: S3 MCG raw data 3 — The raw MCG dataset includes category 4 for training and validation. (ZIP) [file pone.0338189.s003.zip › train/4/p10_395_1.png]

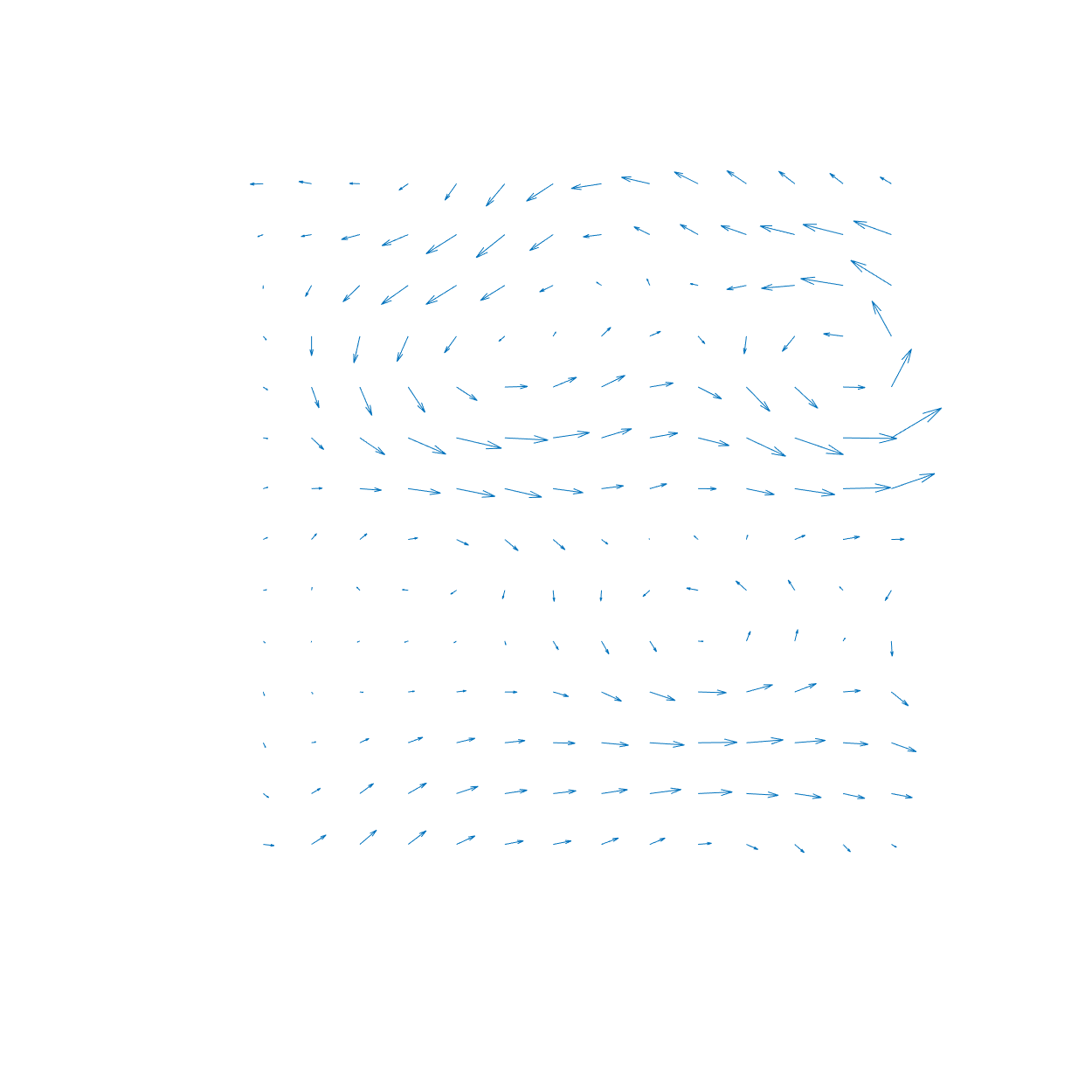

Supplement: S3 MCG raw data 3 — The raw MCG dataset includes category 4 for training and validation. (ZIP) [file pone.0338189.s003.zip › train/4/p10_395_2.png]

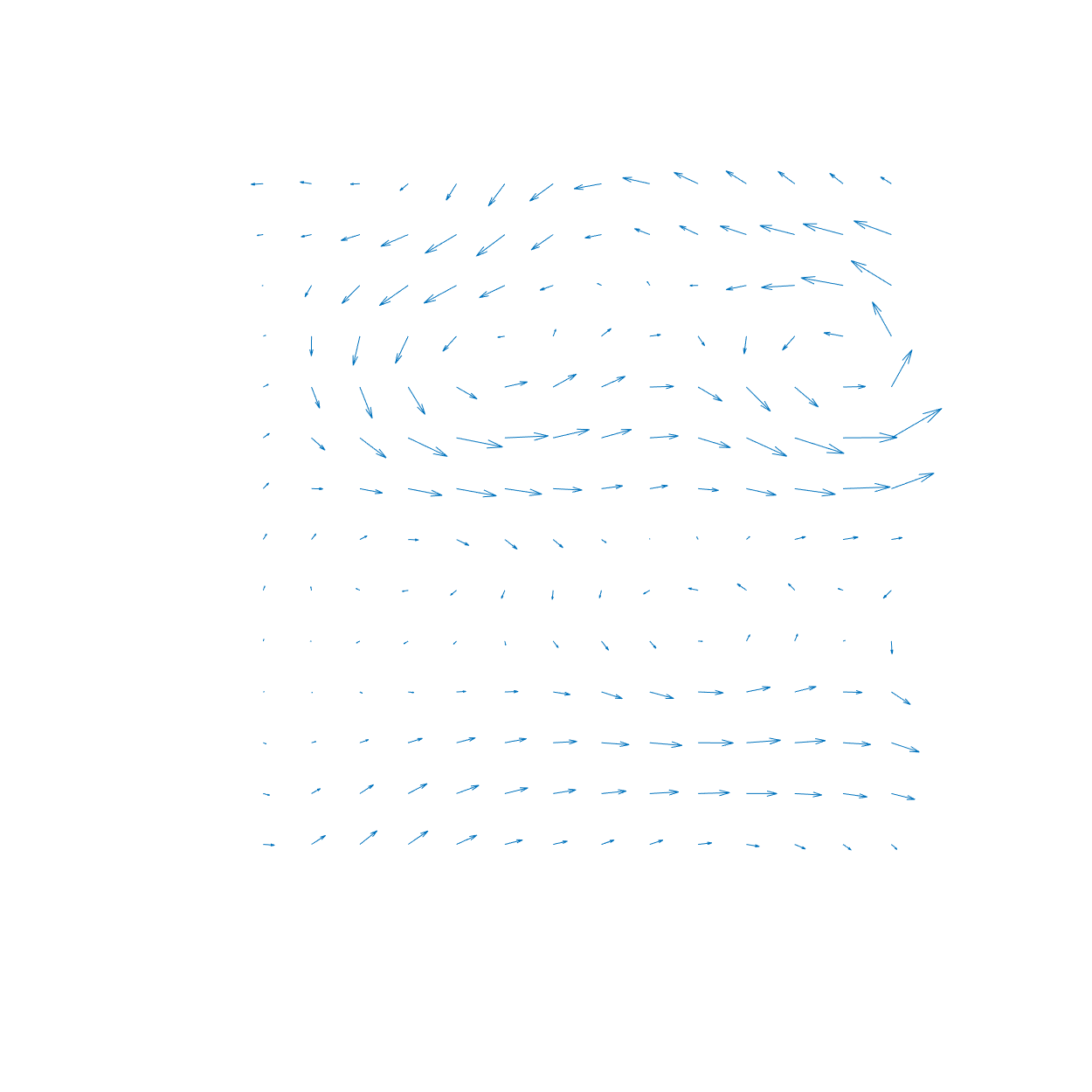

Supplement: S3 MCG raw data 3 — The raw MCG dataset includes category 4 for training and validation. (ZIP) [file pone.0338189.s003.zip › train/4/p10_395_3.png]

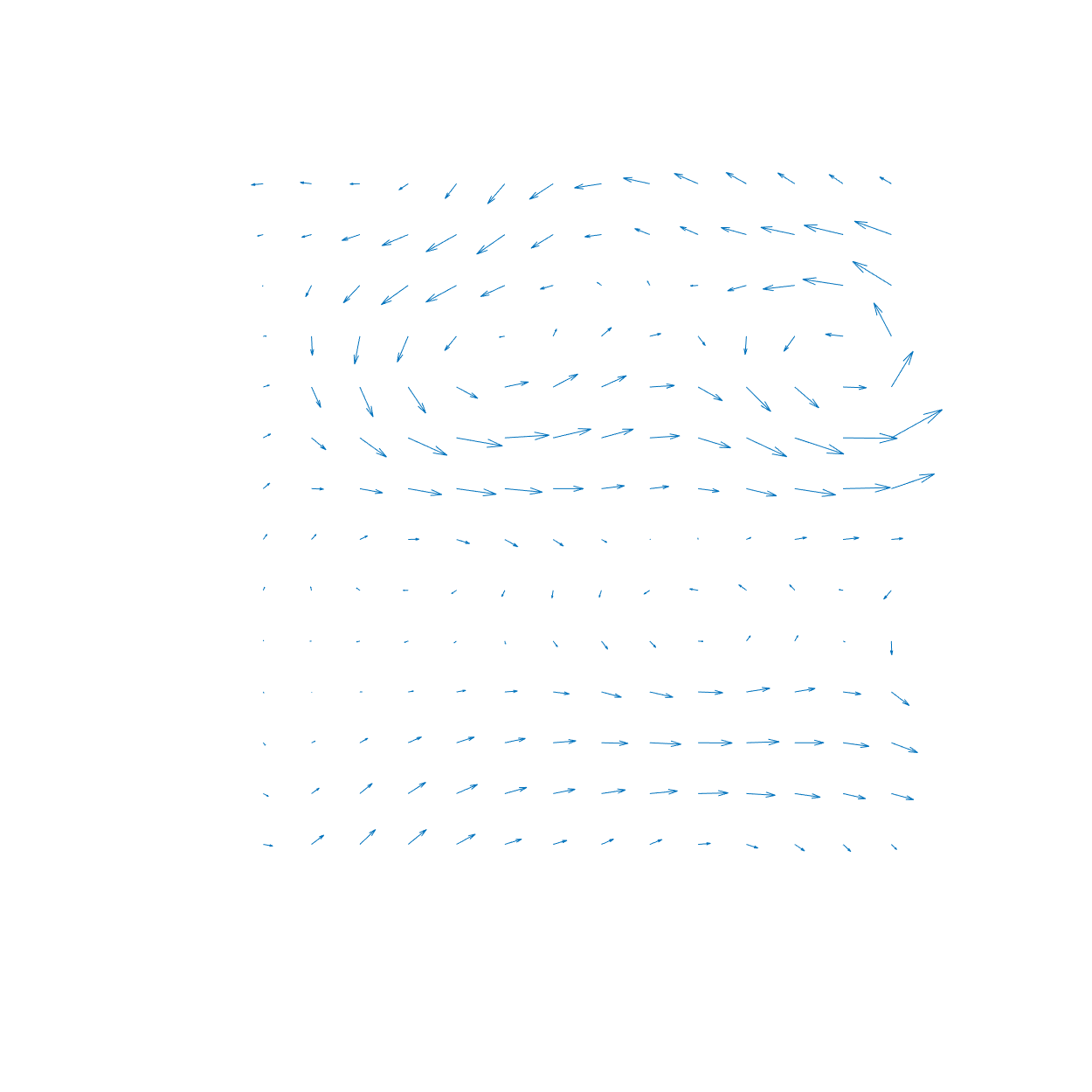

Supplement: S3 MCG raw data 3 — The raw MCG dataset includes category 4 for training and validation. (ZIP) [file pone.0338189.s003.zip › train/4/p10_395_4.png]

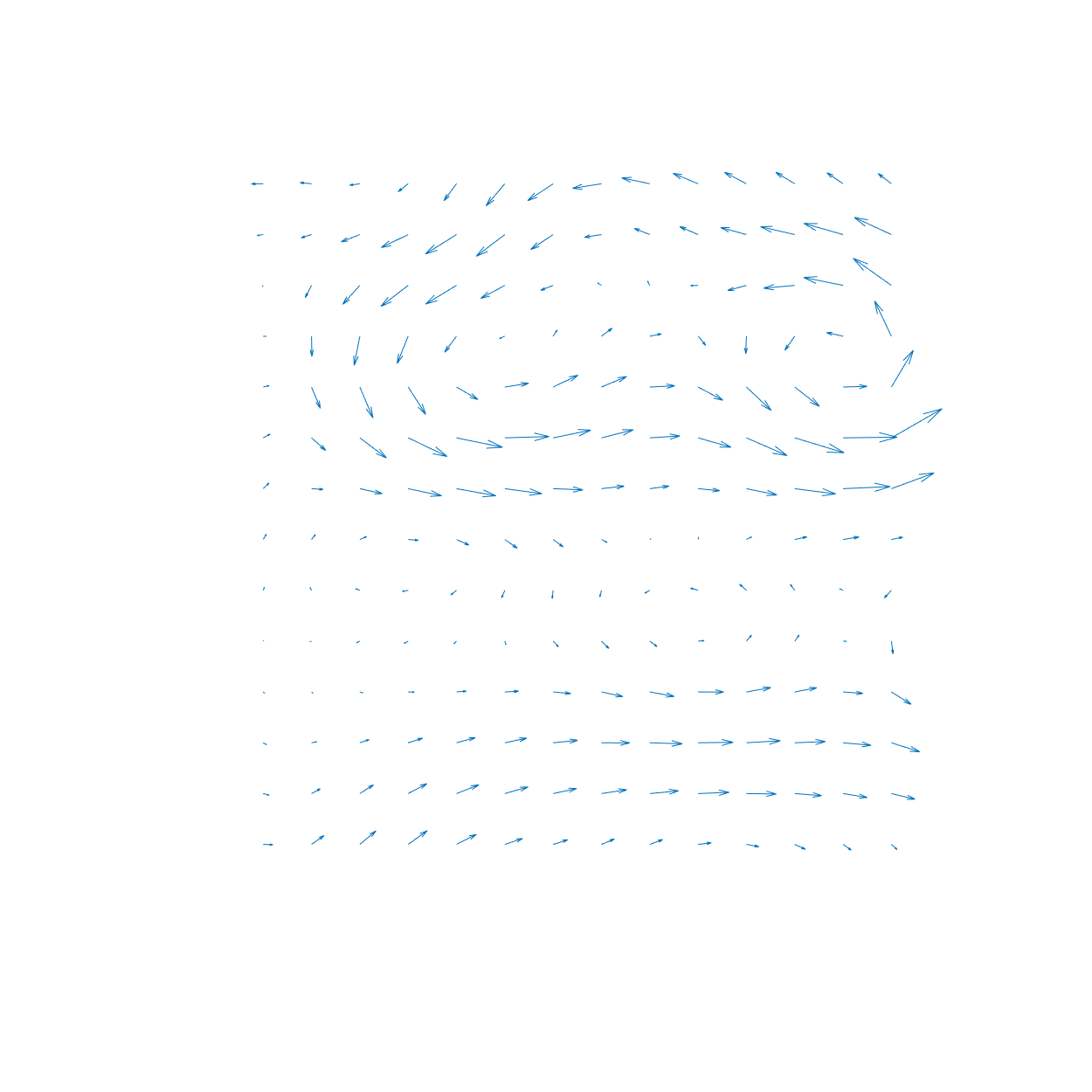

Supplement: S3 MCG raw data 3 — The raw MCG dataset includes category 4 for training and validation. (ZIP) [file pone.0338189.s003.zip › train/4/p10_400_1.png]

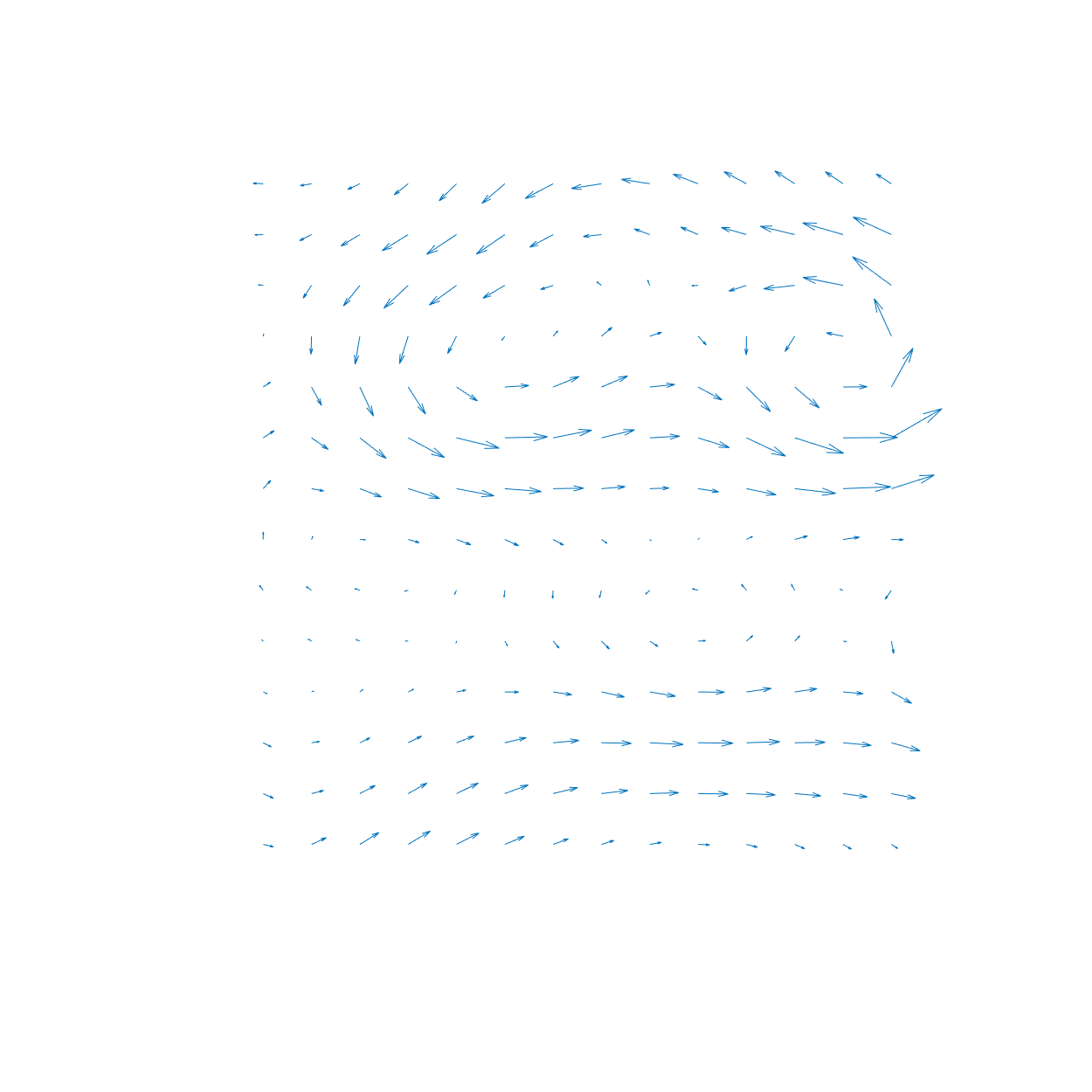

Supplement: S3 MCG raw data 3 — The raw MCG dataset includes category 4 for training and validation. (ZIP) [file pone.0338189.s003.zip › train/4/p10_400_2.png]

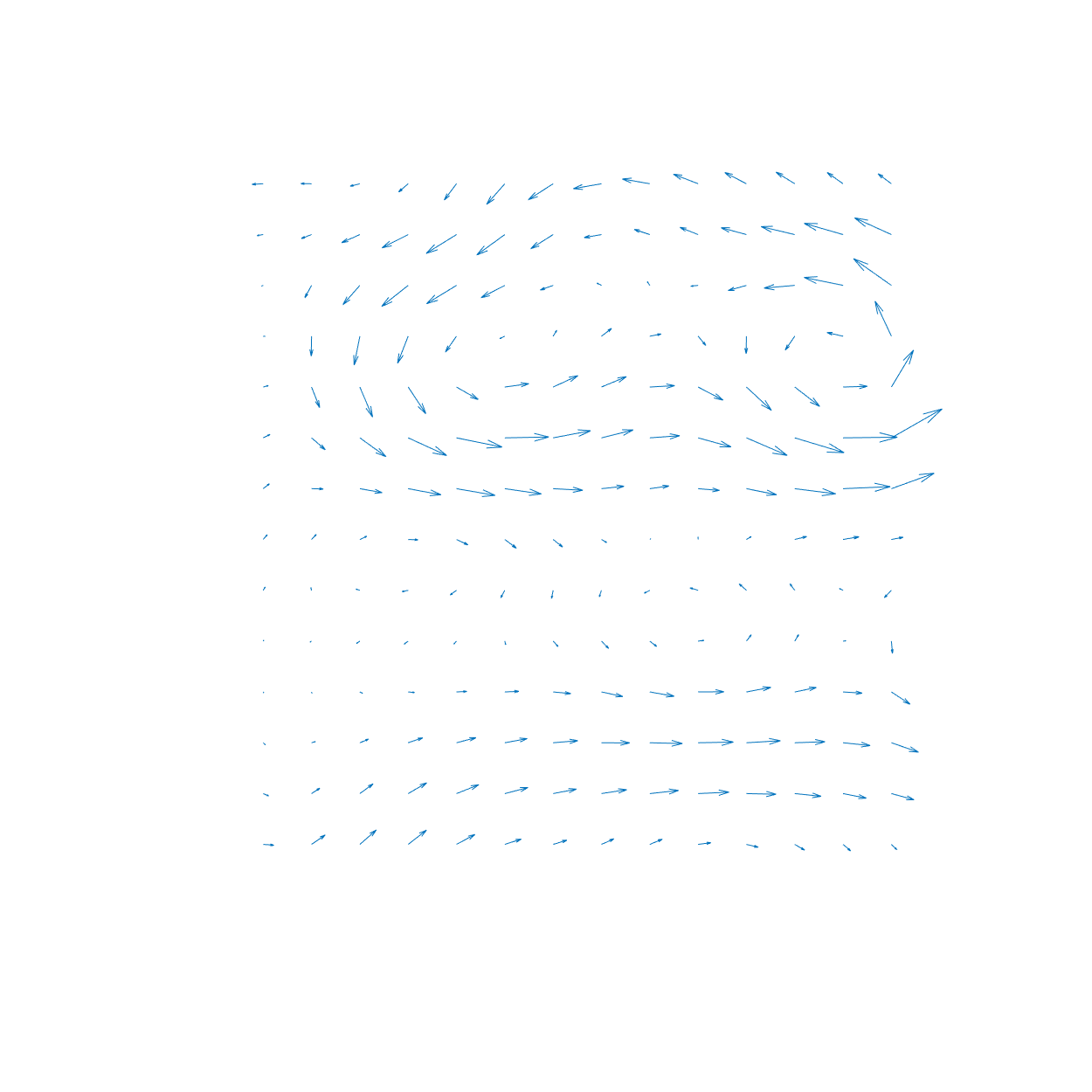

Supplement: S3 MCG raw data 3 — The raw MCG dataset includes category 4 for training and validation. (ZIP) [file pone.0338189.s003.zip › train/4/p10_400_3.png]

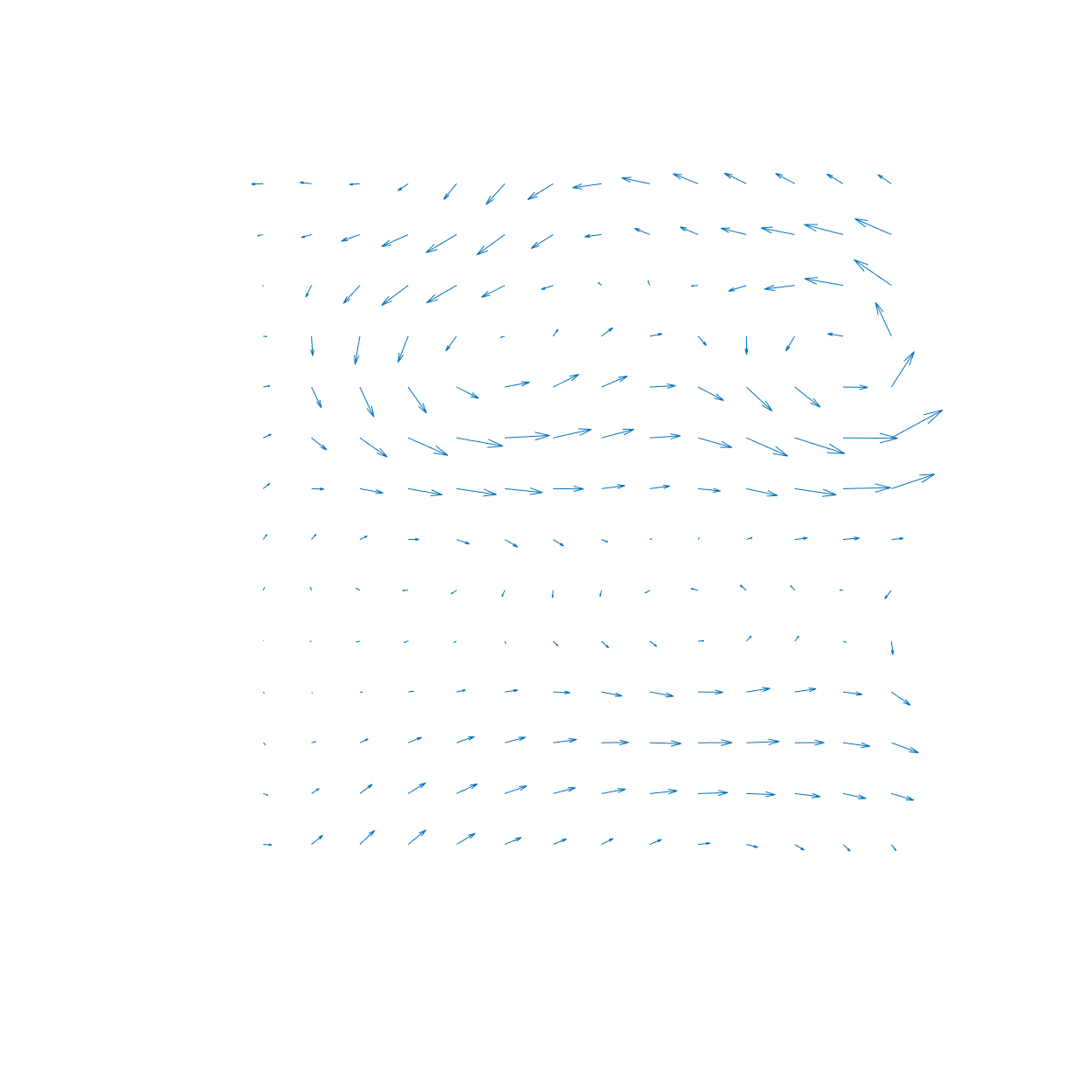

Supplement: S3 MCG raw data 3 — The raw MCG dataset includes category 4 for training and validation. (ZIP) [file pone.0338189.s003.zip › train/4/p10_400_4.png]

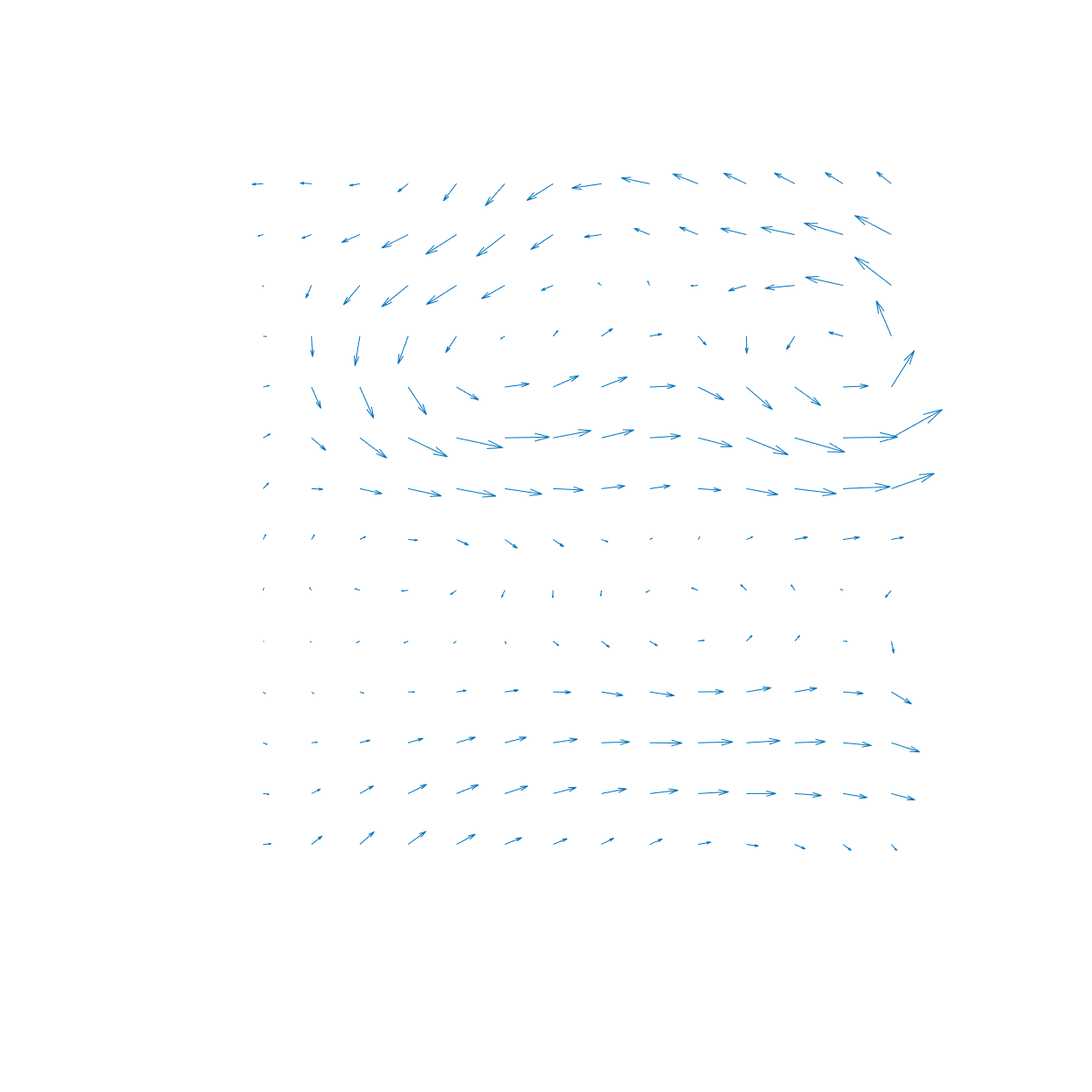

Supplement: S3 MCG raw data 3 — The raw MCG dataset includes category 4 for training and validation. (ZIP) [file pone.0338189.s003.zip › train/4/p10_405_1.png]

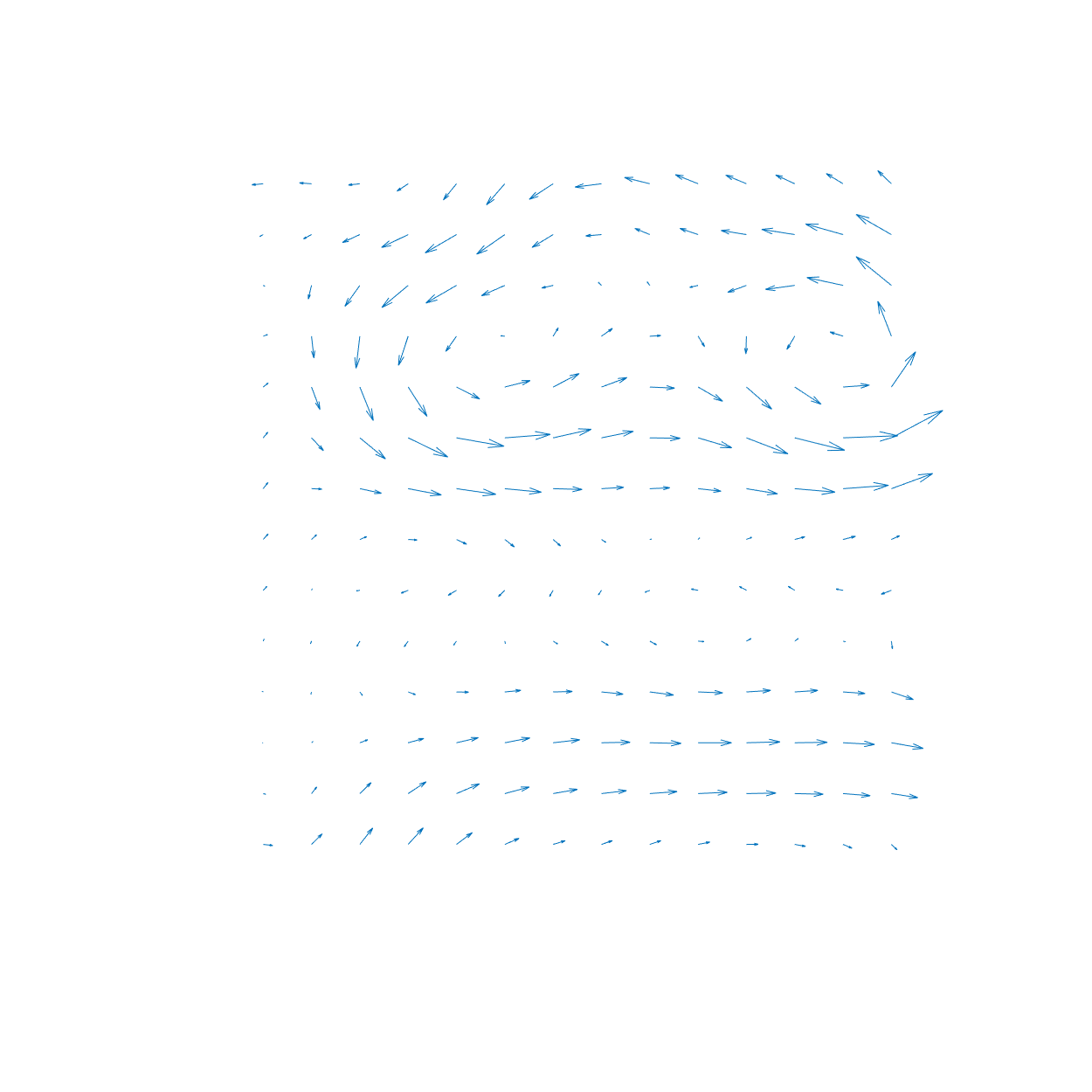

Supplement: S3 MCG raw data 3 — The raw MCG dataset includes category 4 for training and validation. (ZIP) [file pone.0338189.s003.zip › train/4/p10_405_2.png]

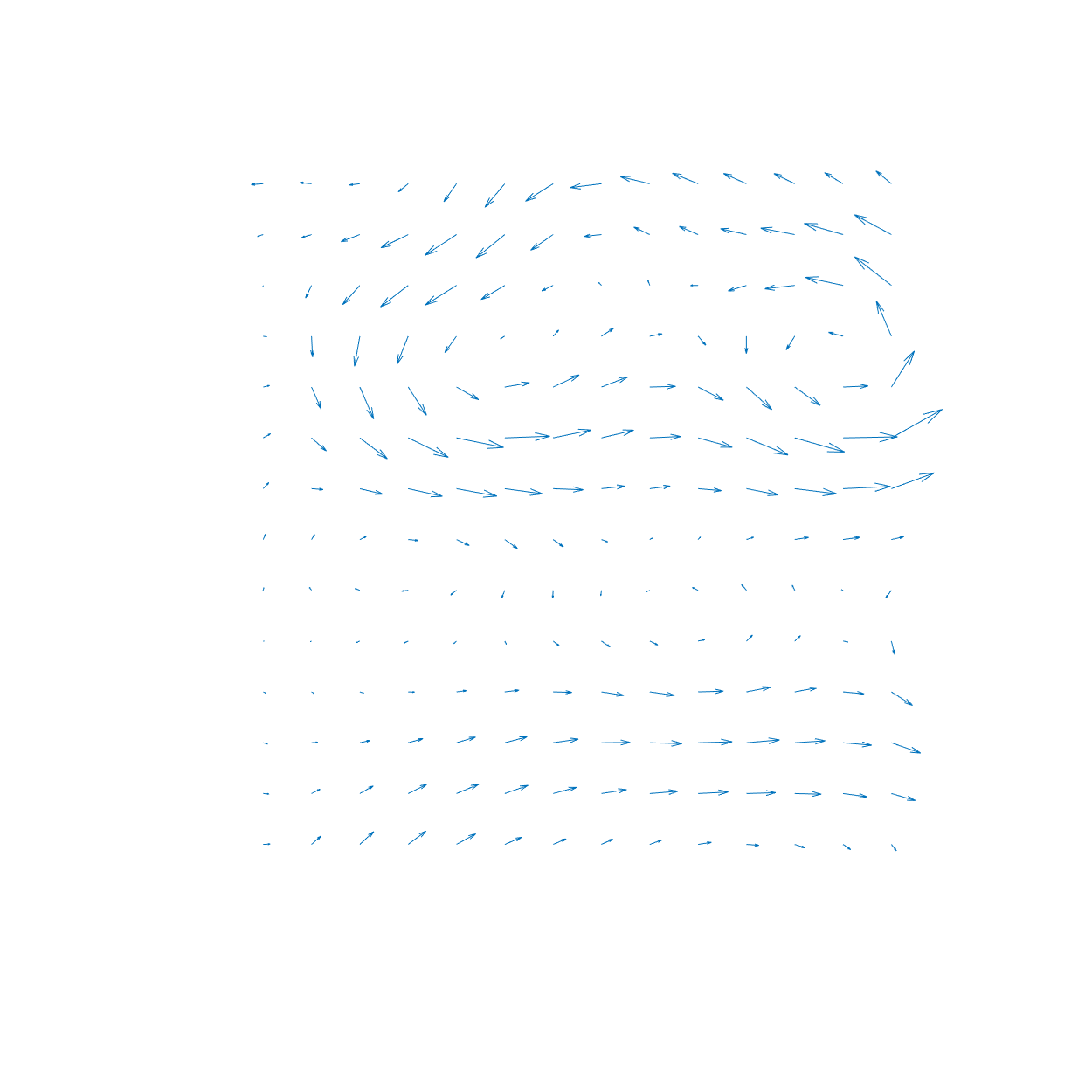

Supplement: S3 MCG raw data 3 — The raw MCG dataset includes category 4 for training and validation. (ZIP) [file pone.0338189.s003.zip › train/4/p10_405_3.png]

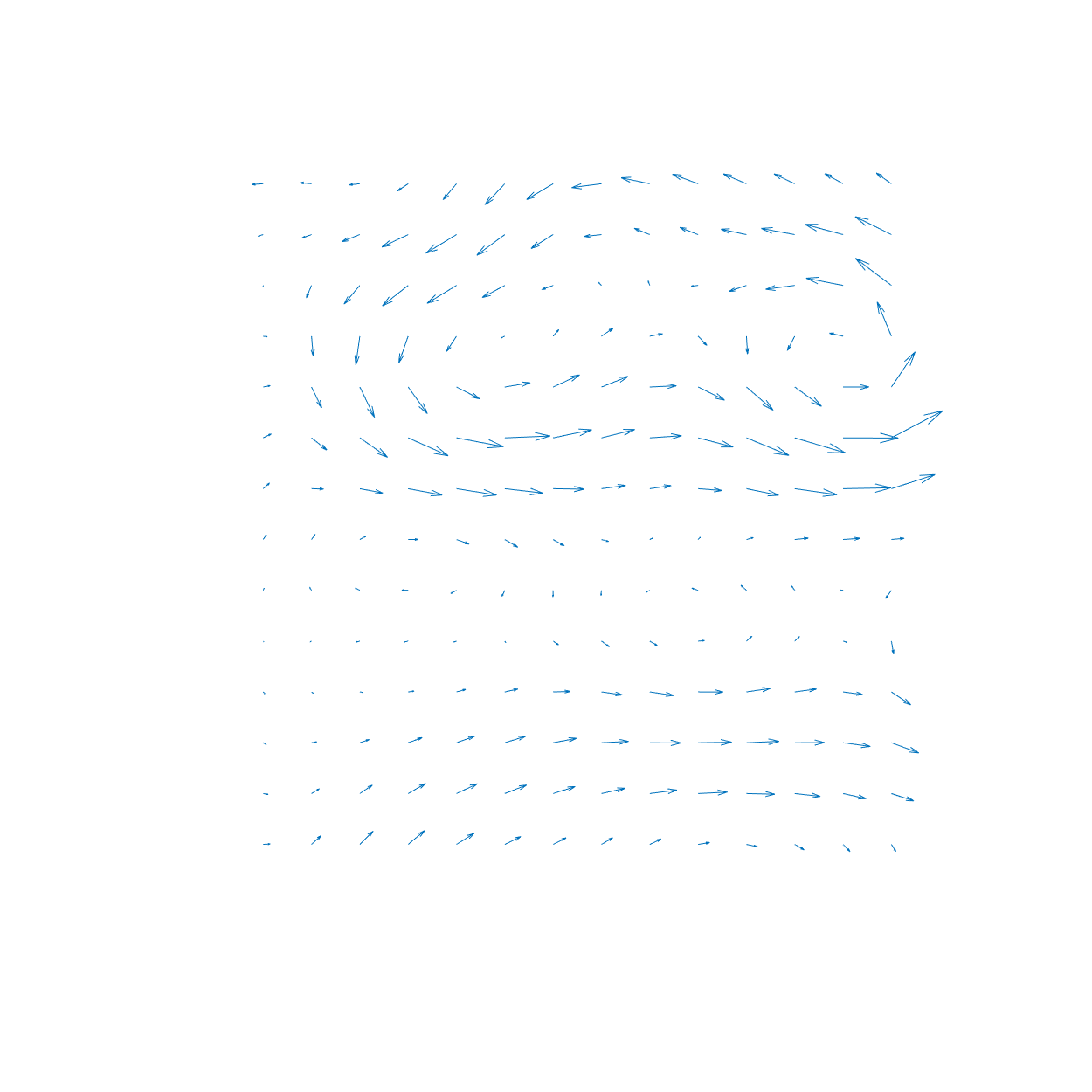

Supplement: S3 MCG raw data 3 — The raw MCG dataset includes category 4 for training and validation. (ZIP) [file pone.0338189.s003.zip › train/4/p10_405_4.png]

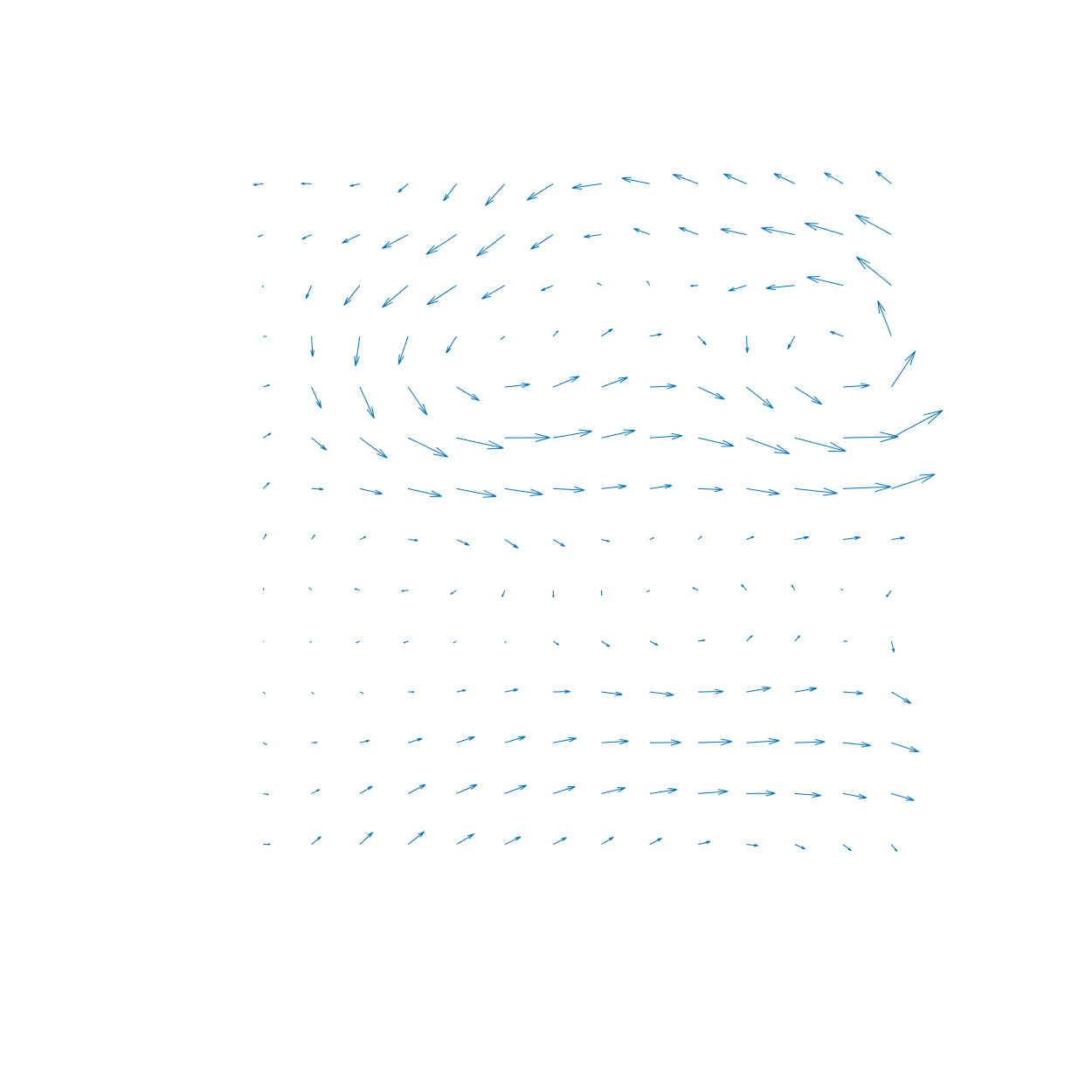

Supplement: S3 MCG raw data 3 — The raw MCG dataset includes category 4 for training and validation. (ZIP) [file pone.0338189.s003.zip › train/4/p10_410_1.png]

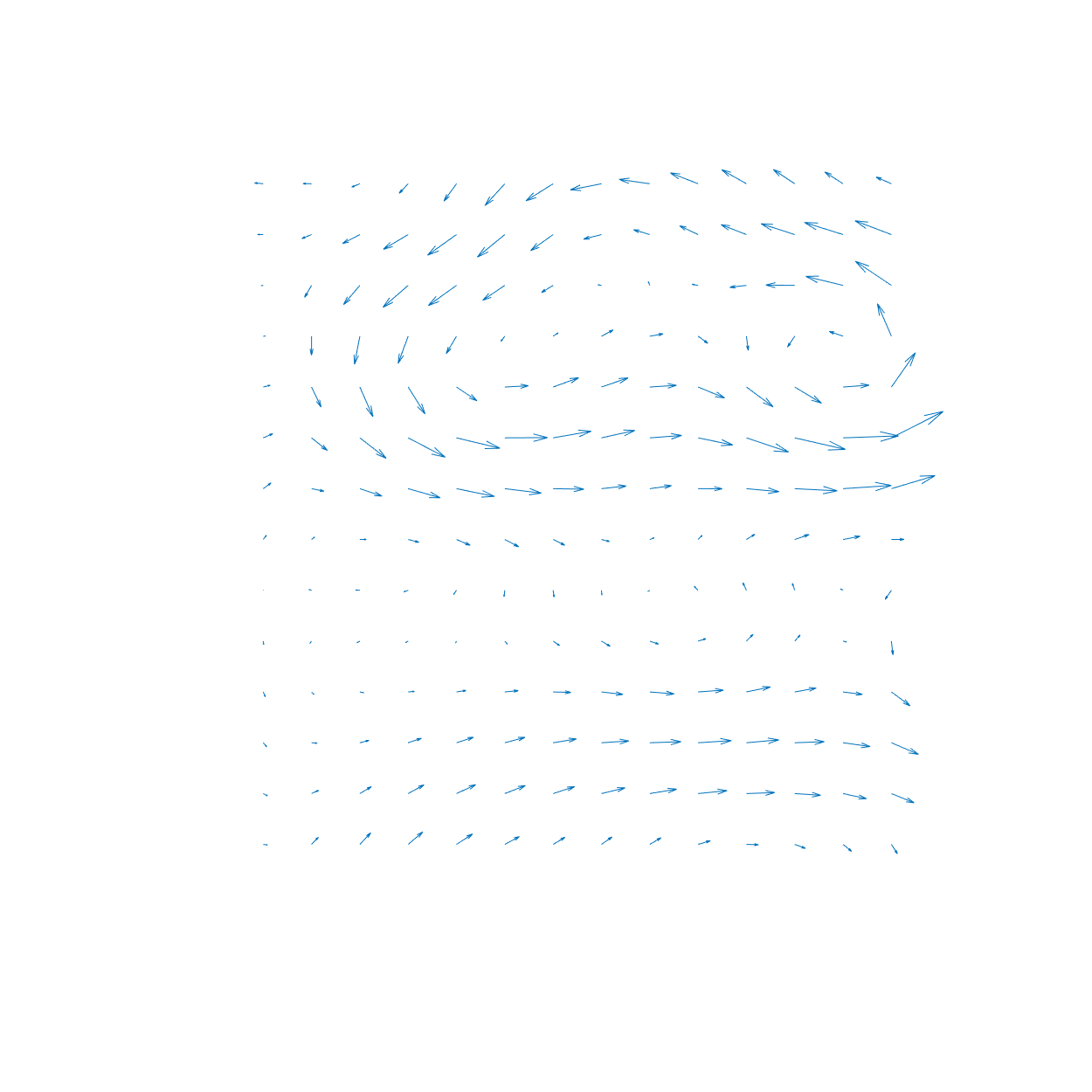

Supplement: S3 MCG raw data 3 — The raw MCG dataset includes category 4 for training and validation. (ZIP) [file pone.0338189.s003.zip › train/4/p10_410_2.png]

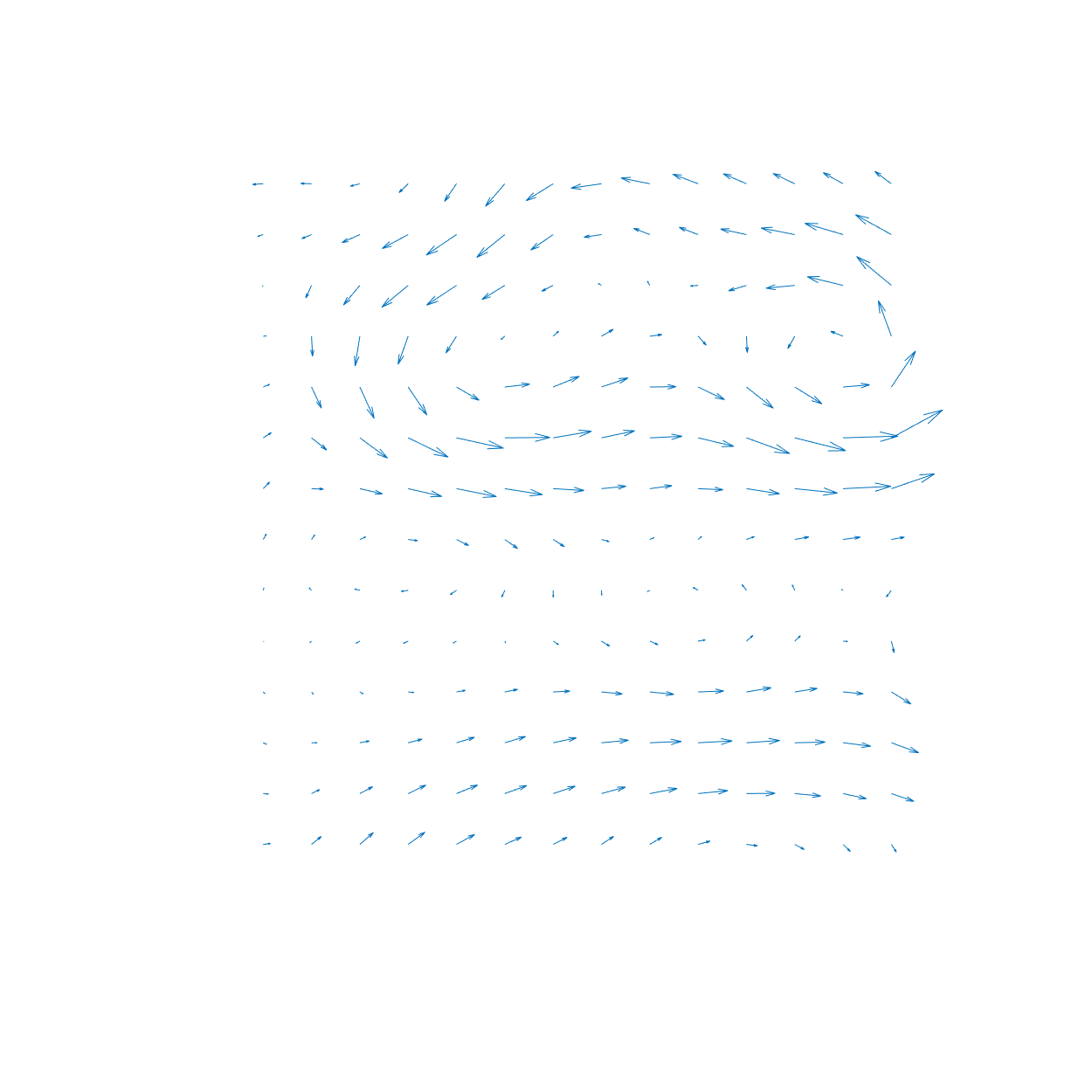

Supplement: S3 MCG raw data 3 — The raw MCG dataset includes category 4 for training and validation. (ZIP) [file pone.0338189.s003.zip › train/4/p10_410_3.png]

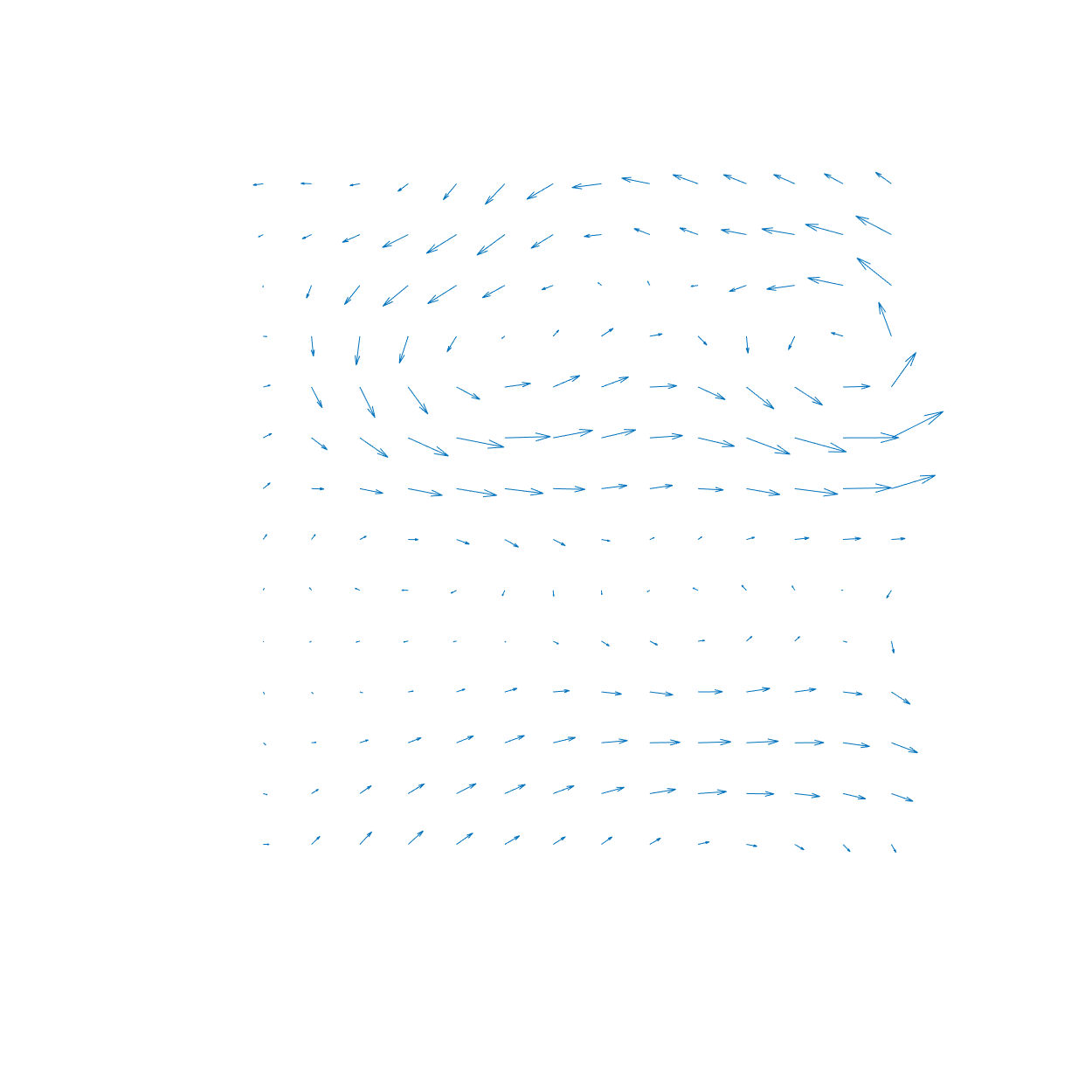

Supplement: S3 MCG raw data 3 — The raw MCG dataset includes category 4 for training and validation. (ZIP) [file pone.0338189.s003.zip › train/4/p10_410_4.png]

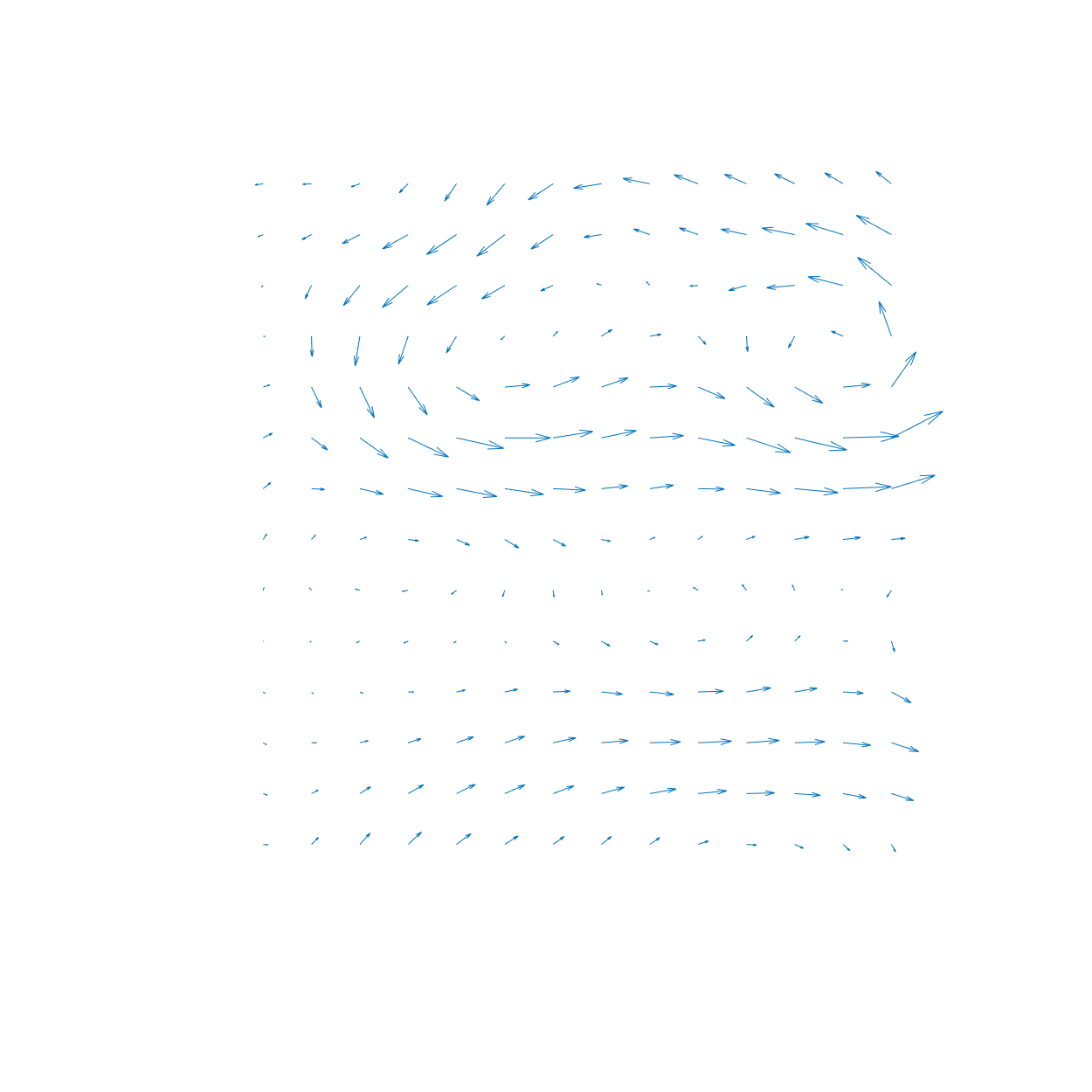

Supplement: S3 MCG raw data 3 — The raw MCG dataset includes category 4 for training and validation. (ZIP) [file pone.0338189.s003.zip › train/4/p10_415_1.png]

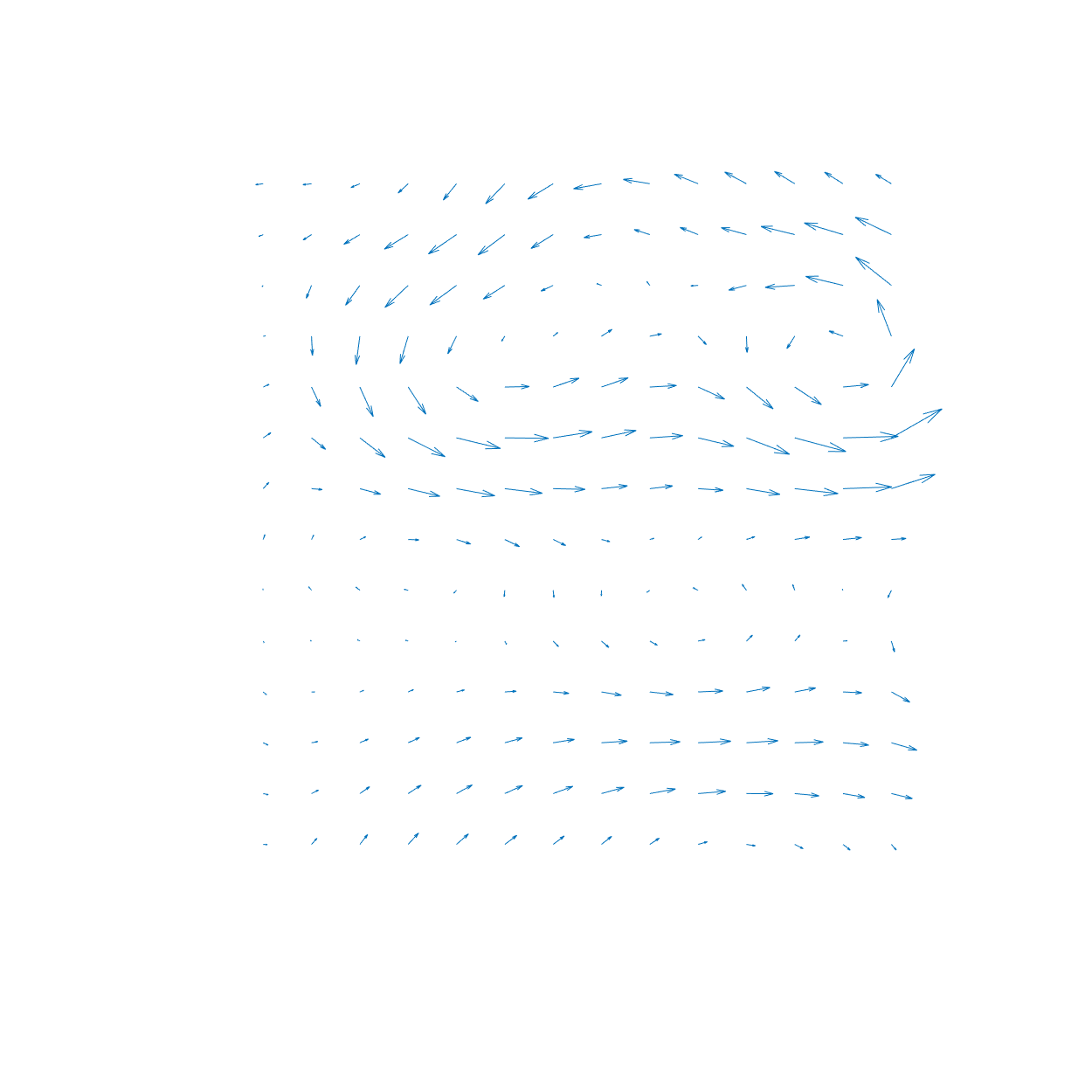

Supplement: S3 MCG raw data 3 — The raw MCG dataset includes category 4 for training and validation. (ZIP) [file pone.0338189.s003.zip › train/4/p10_415_2.png]

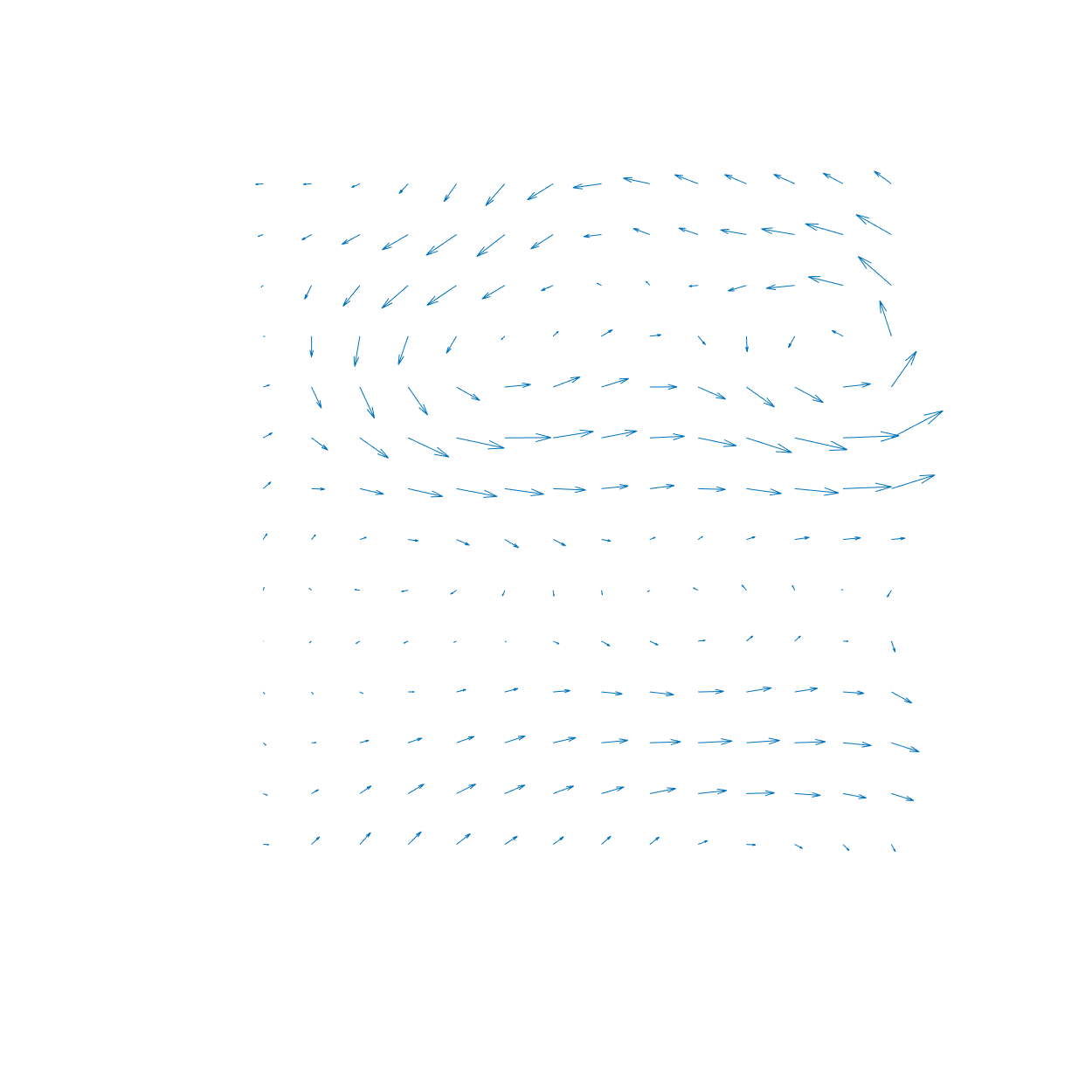

Supplement: S3 MCG raw data 3 — The raw MCG dataset includes category 4 for training and validation. (ZIP) [file pone.0338189.s003.zip › train/4/p10_415_3.png]

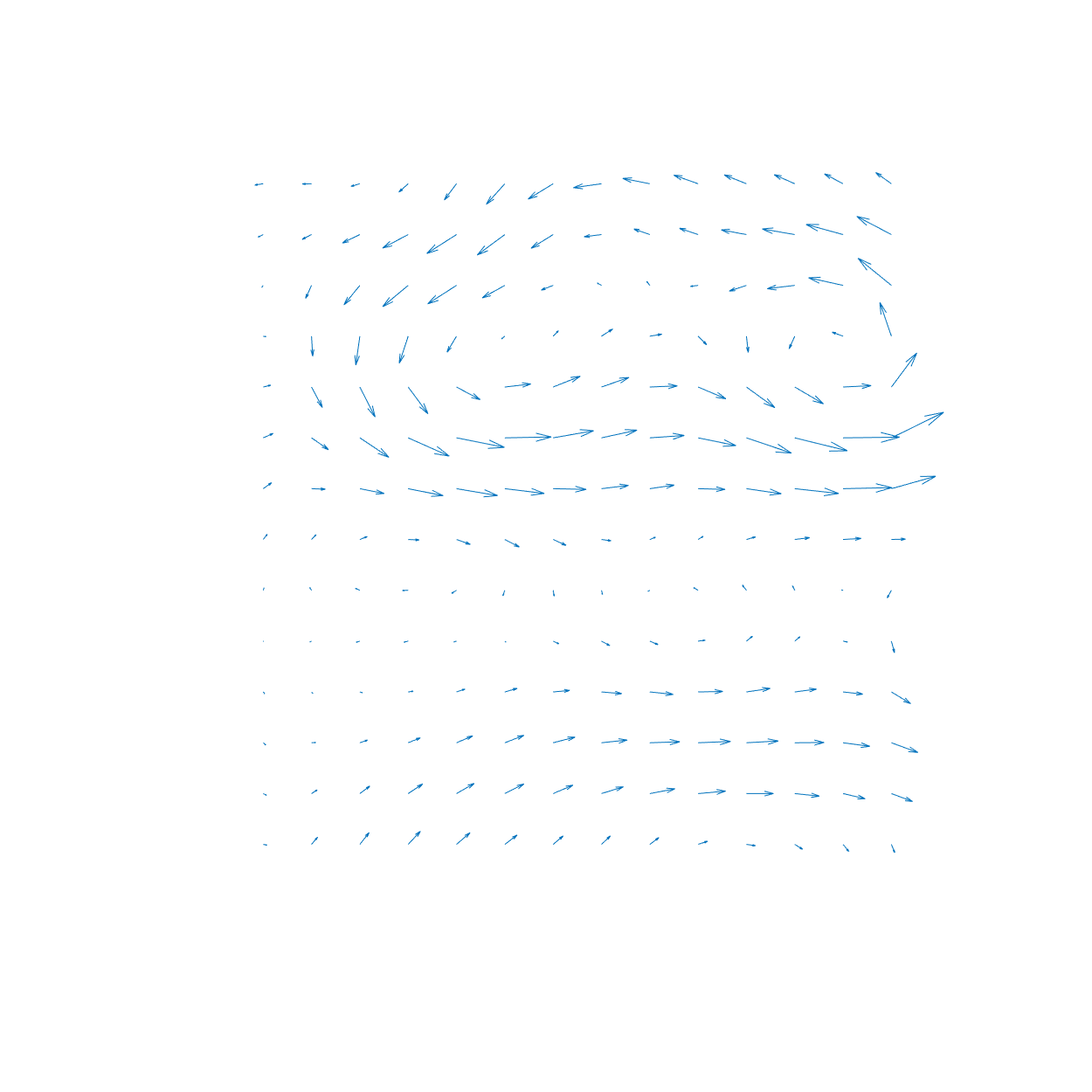

Supplement: S3 MCG raw data 3 — The raw MCG dataset includes category 4 for training and validation. (ZIP) [file pone.0338189.s003.zip › train/4/p10_415_4.png]

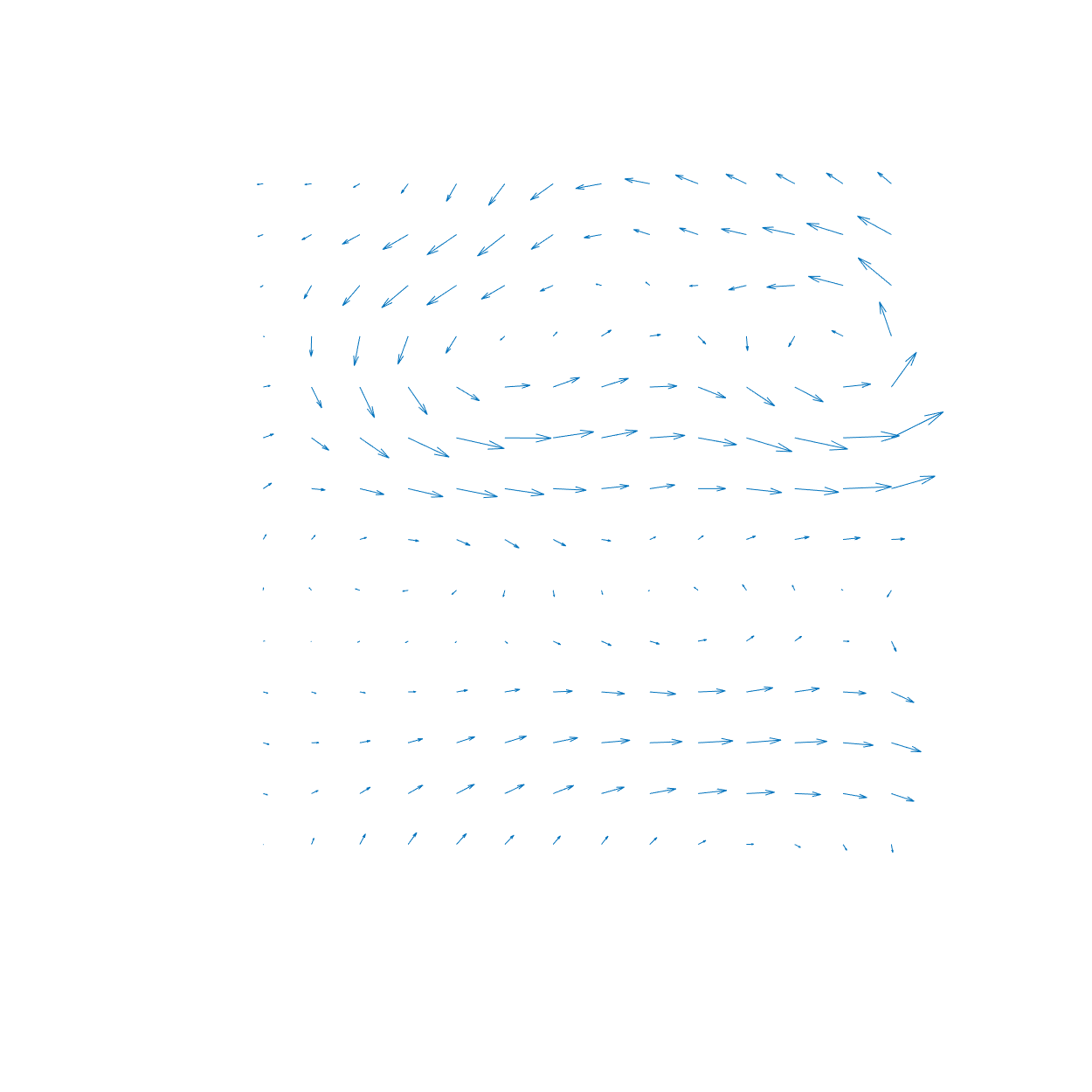

Supplement: S3 MCG raw data 3 — The raw MCG dataset includes category 4 for training and validation. (ZIP) [file pone.0338189.s003.zip › train/4/p10_420_1.png]

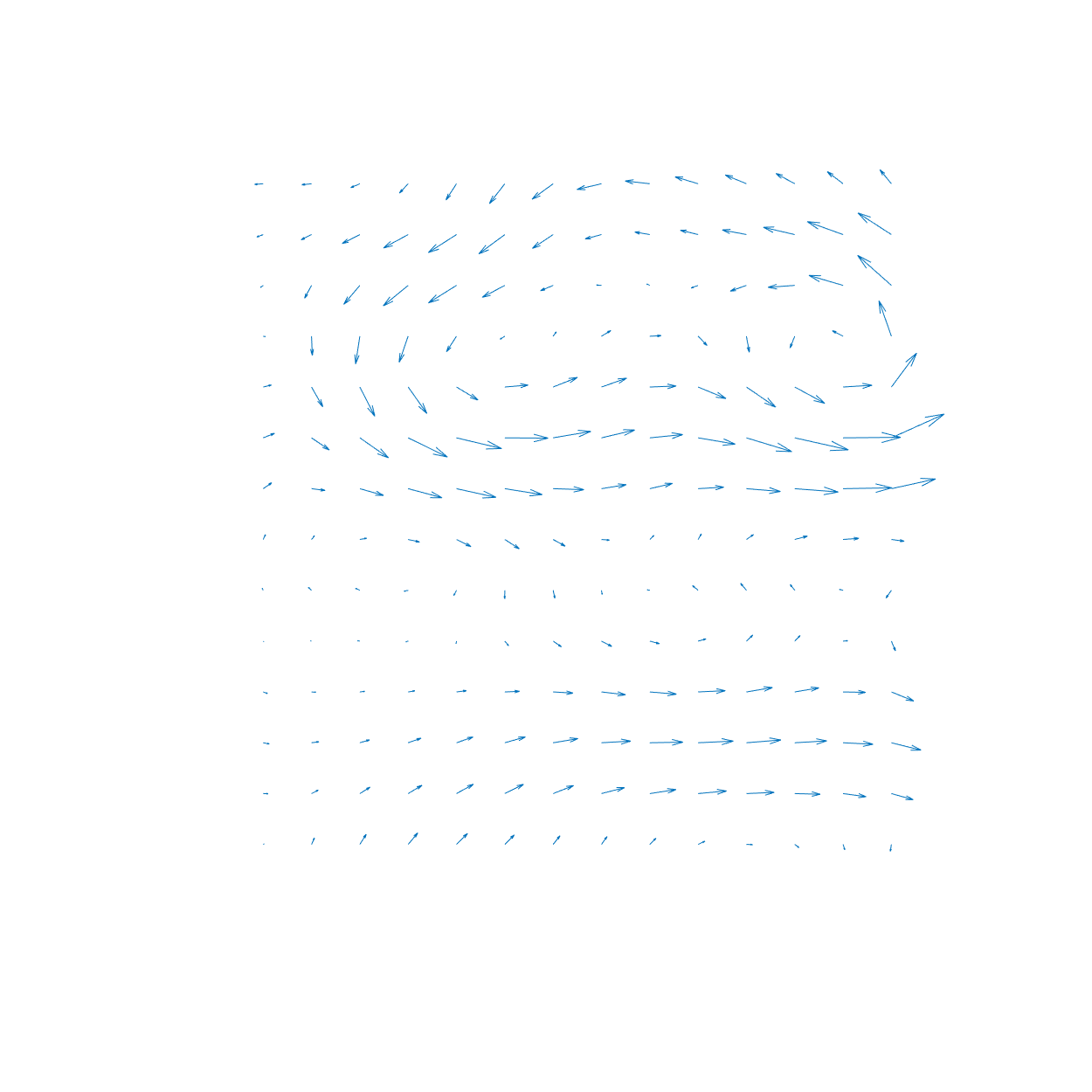

Supplement: S3 MCG raw data 3 — The raw MCG dataset includes category 4 for training and validation. (ZIP) [file pone.0338189.s003.zip › train/4/p10_420_2.png]

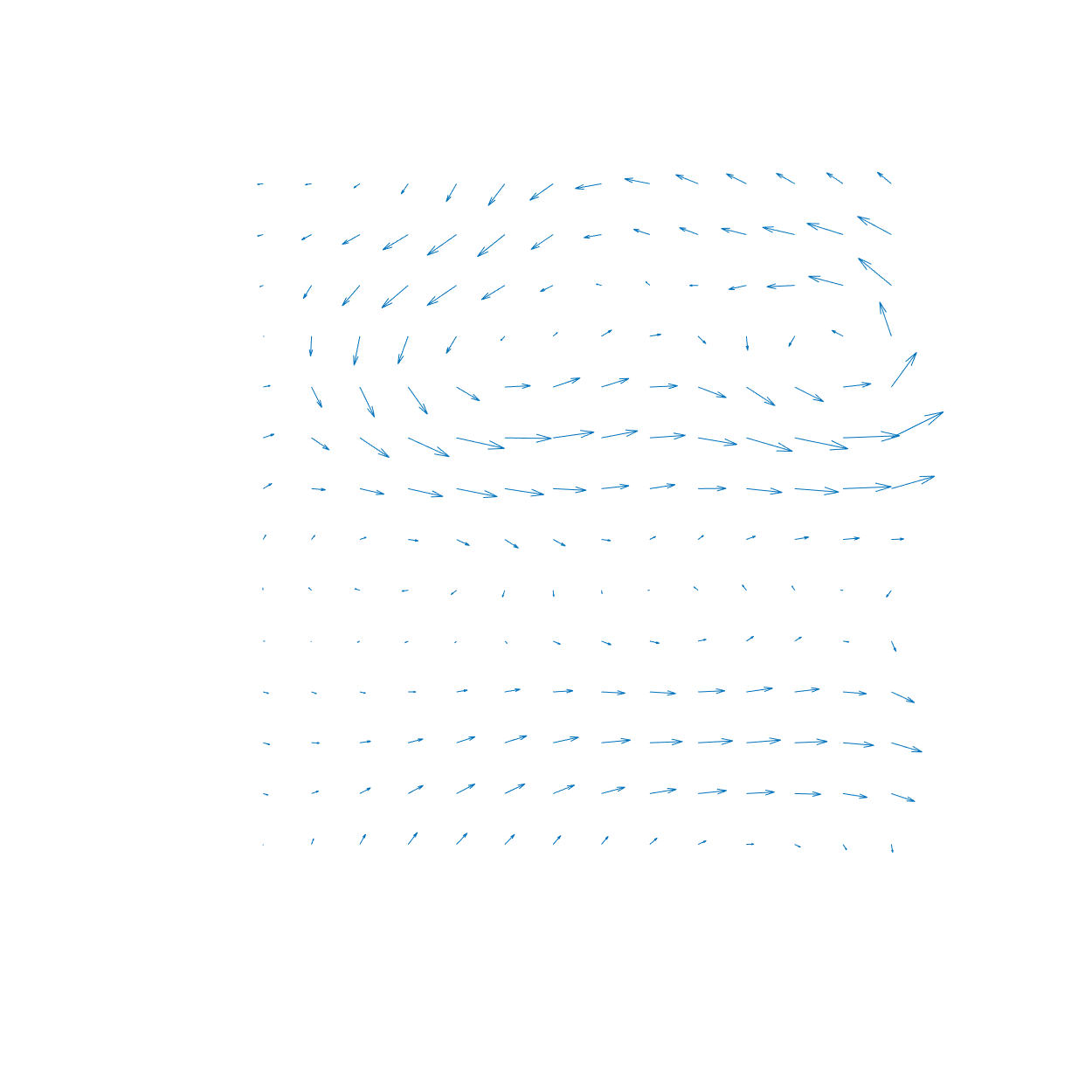

Supplement: S3 MCG raw data 3 — The raw MCG dataset includes category 4 for training and validation. (ZIP) [file pone.0338189.s003.zip › train/4/p10_420_3.png]

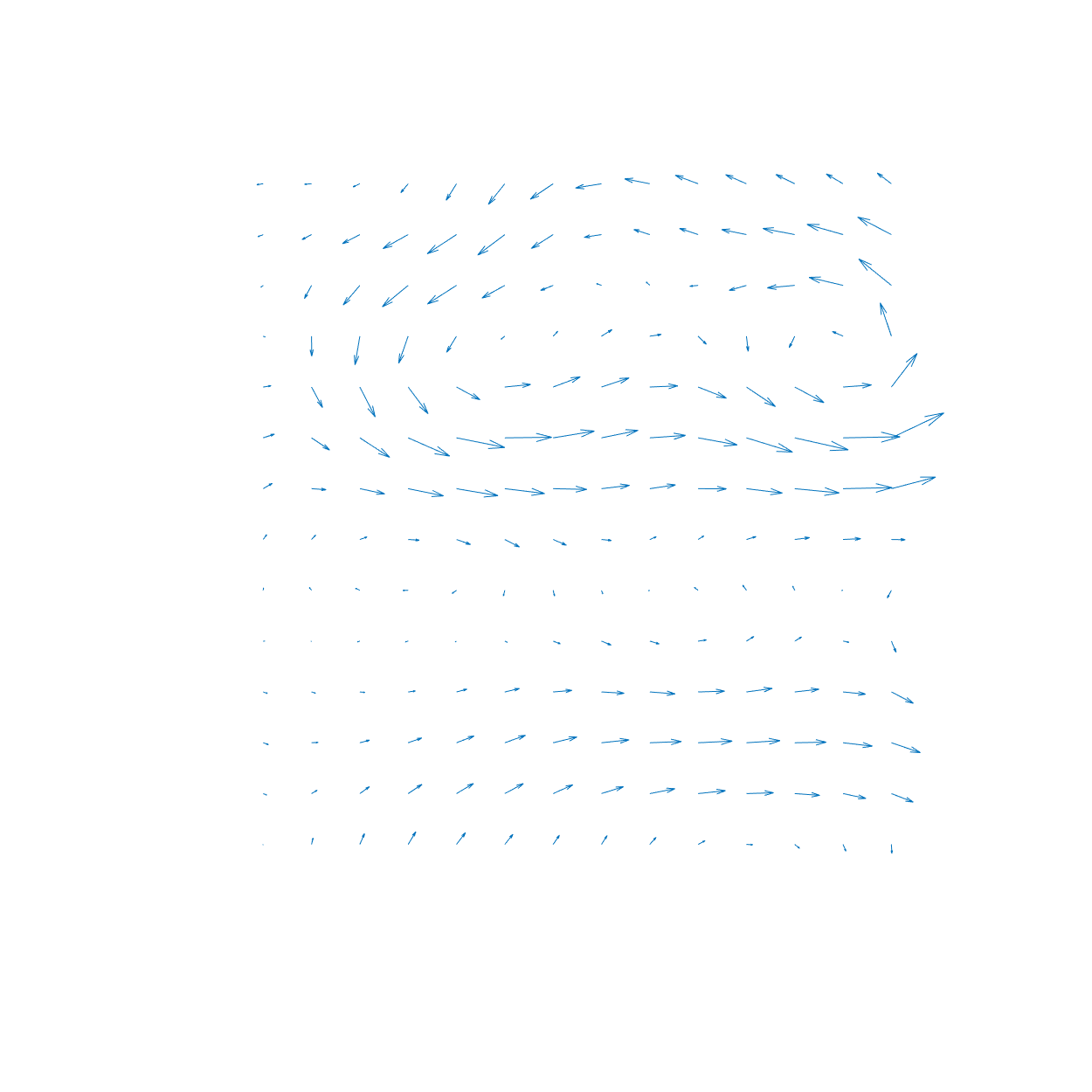

Supplement: S3 MCG raw data 3 — The raw MCG dataset includes category 4 for training and validation. (ZIP) [file pone.0338189.s003.zip › train/4/p10_420_4.png]

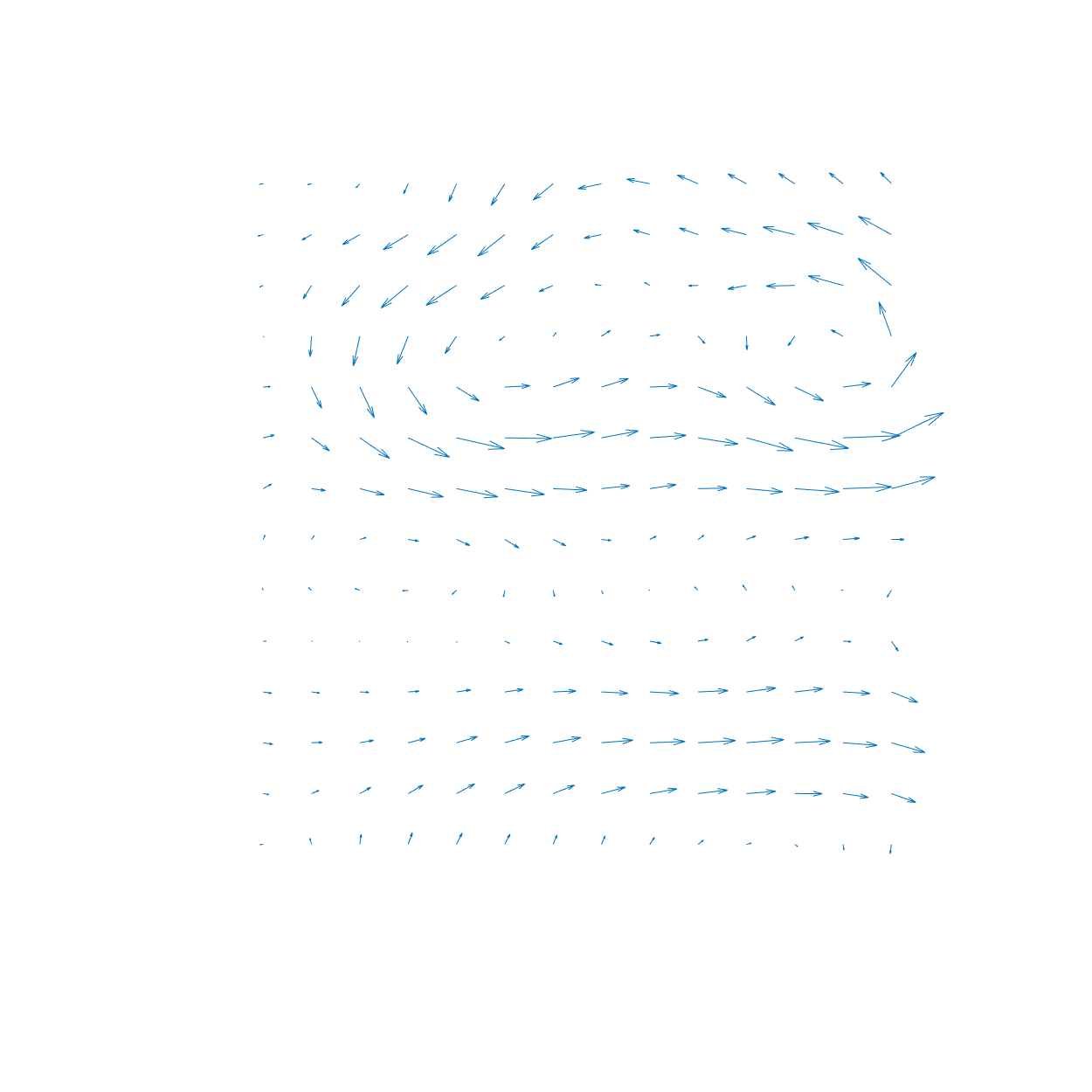

Supplement: S3 MCG raw data 3 — The raw MCG dataset includes category 4 for training and validation. (ZIP) [file pone.0338189.s003.zip › train/4/p10_425_1.png]

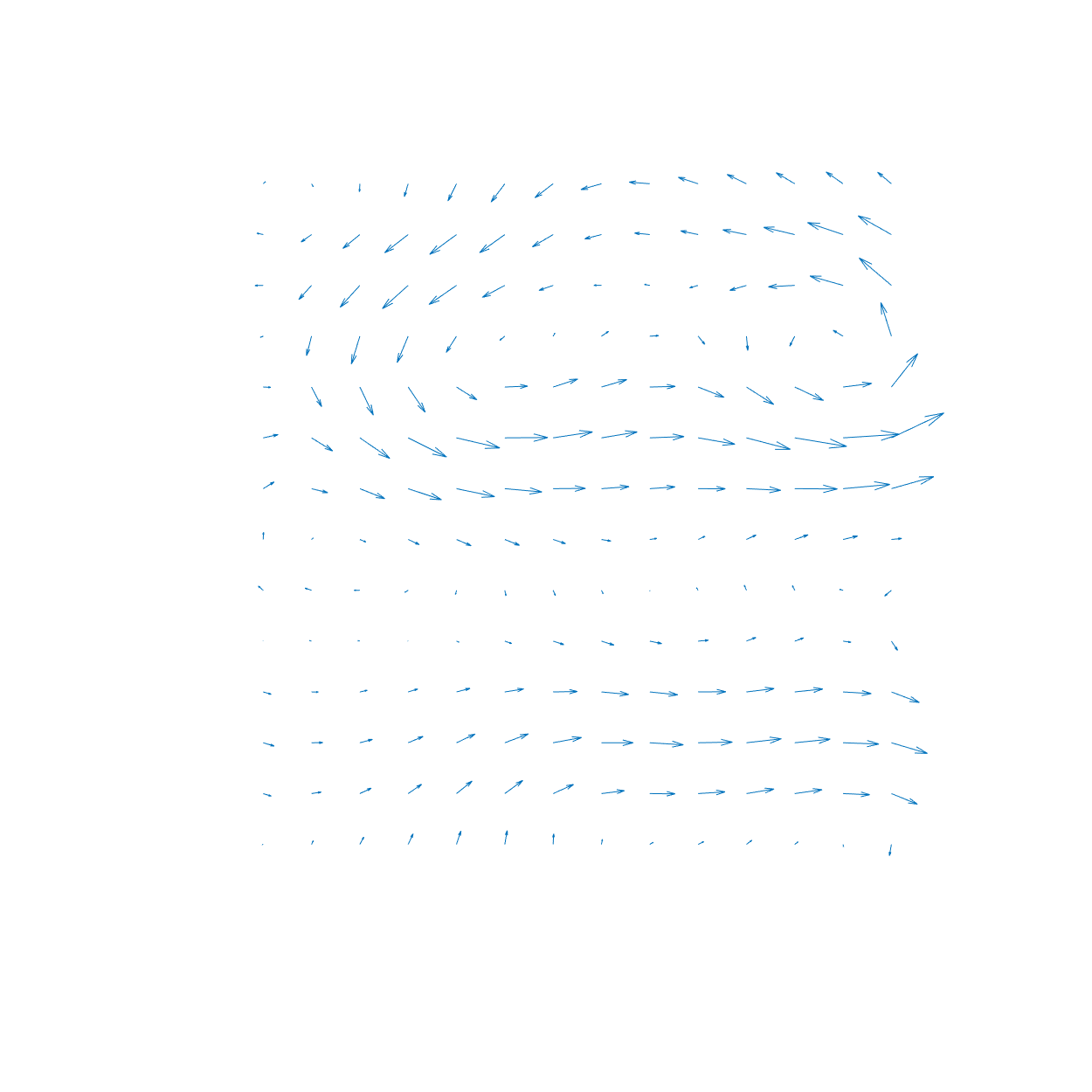

Supplement: S3 MCG raw data 3 — The raw MCG dataset includes category 4 for training and validation. (ZIP) [file pone.0338189.s003.zip › train/4/p10_425_2.png]

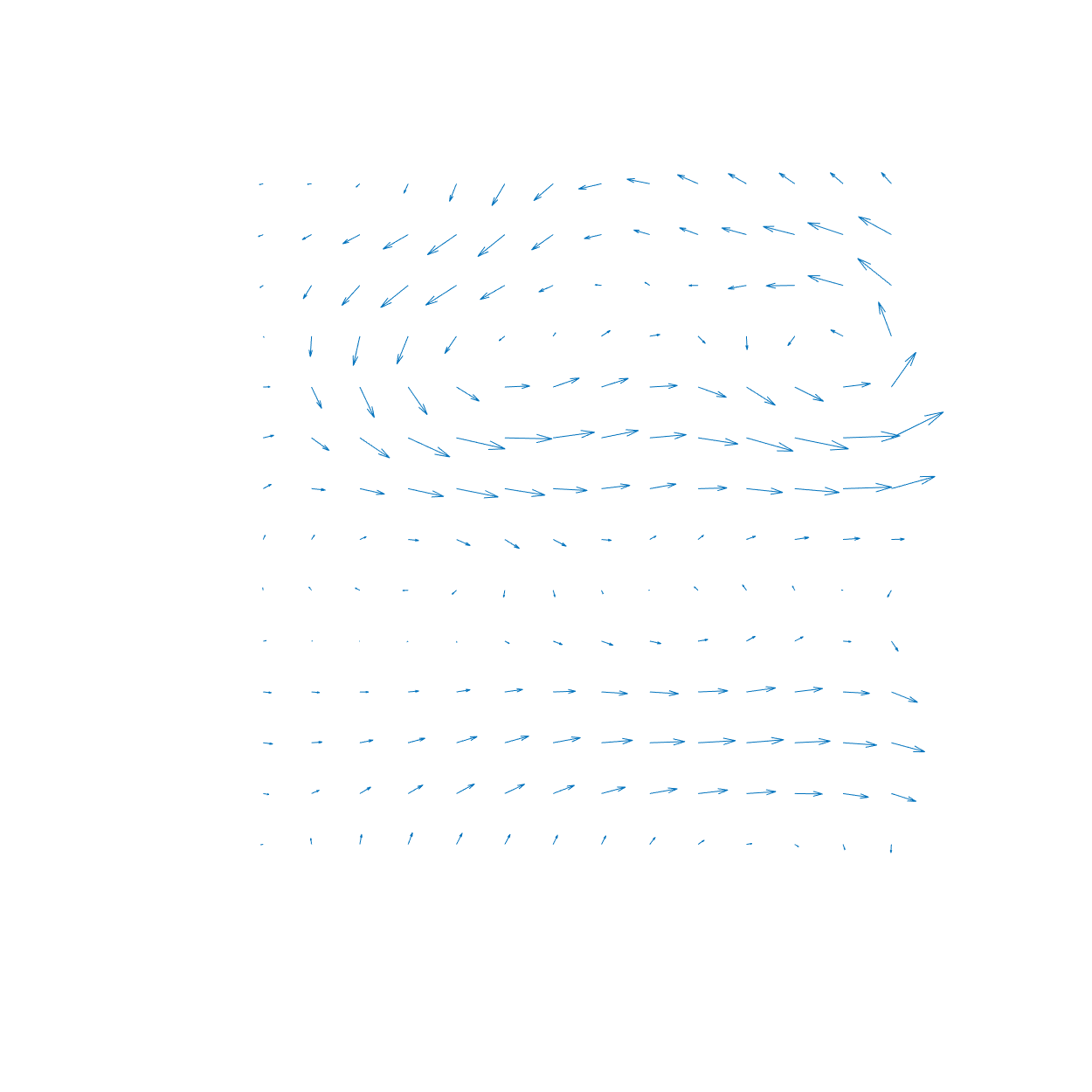

Supplement: S3 MCG raw data 3 — The raw MCG dataset includes category 4 for training and validation. (ZIP) [file pone.0338189.s003.zip › train/4/p10_425_3.png]

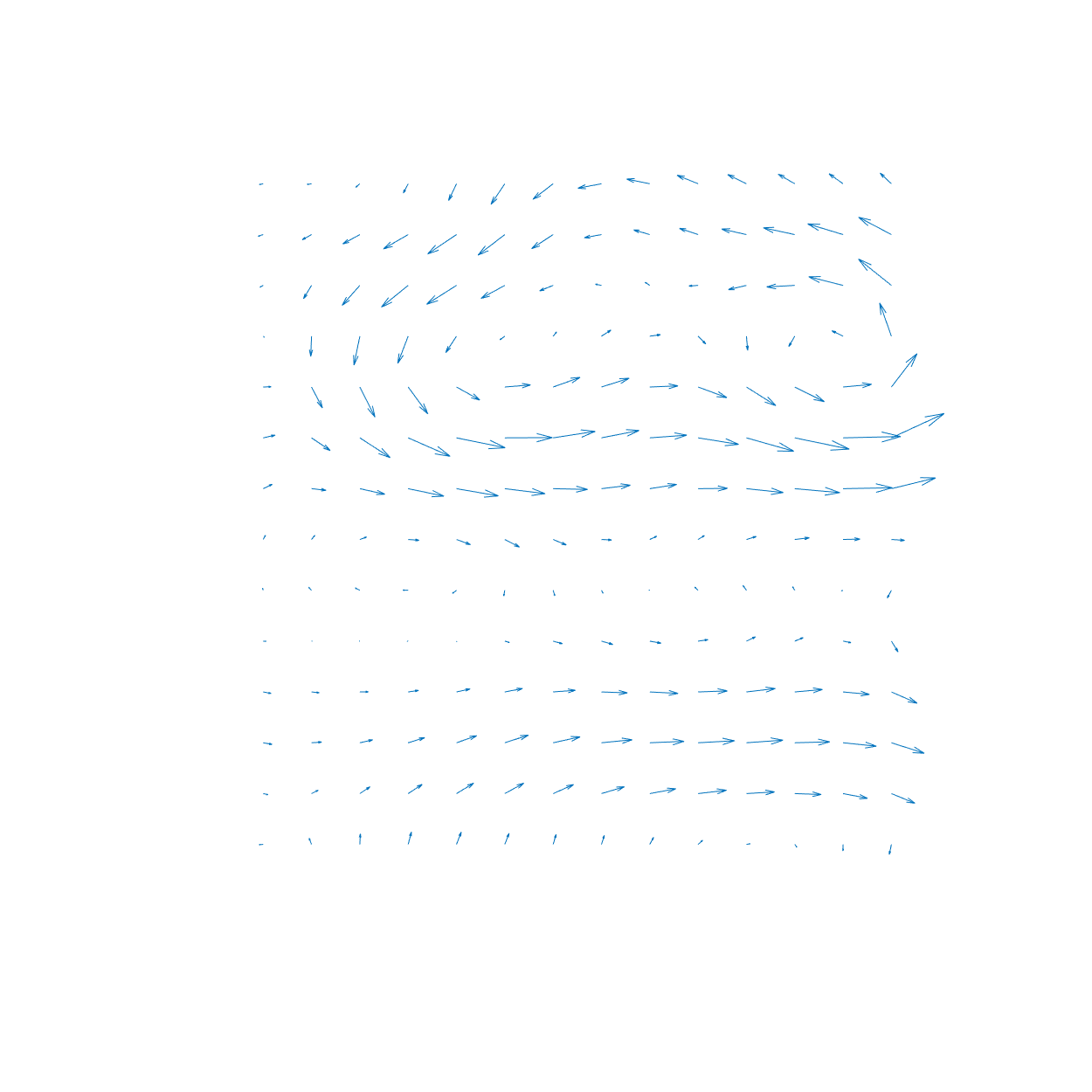

Supplement: S3 MCG raw data 3 — The raw MCG dataset includes category 4 for training and validation. (ZIP) [file pone.0338189.s003.zip › train/4/p10_425_4.png]

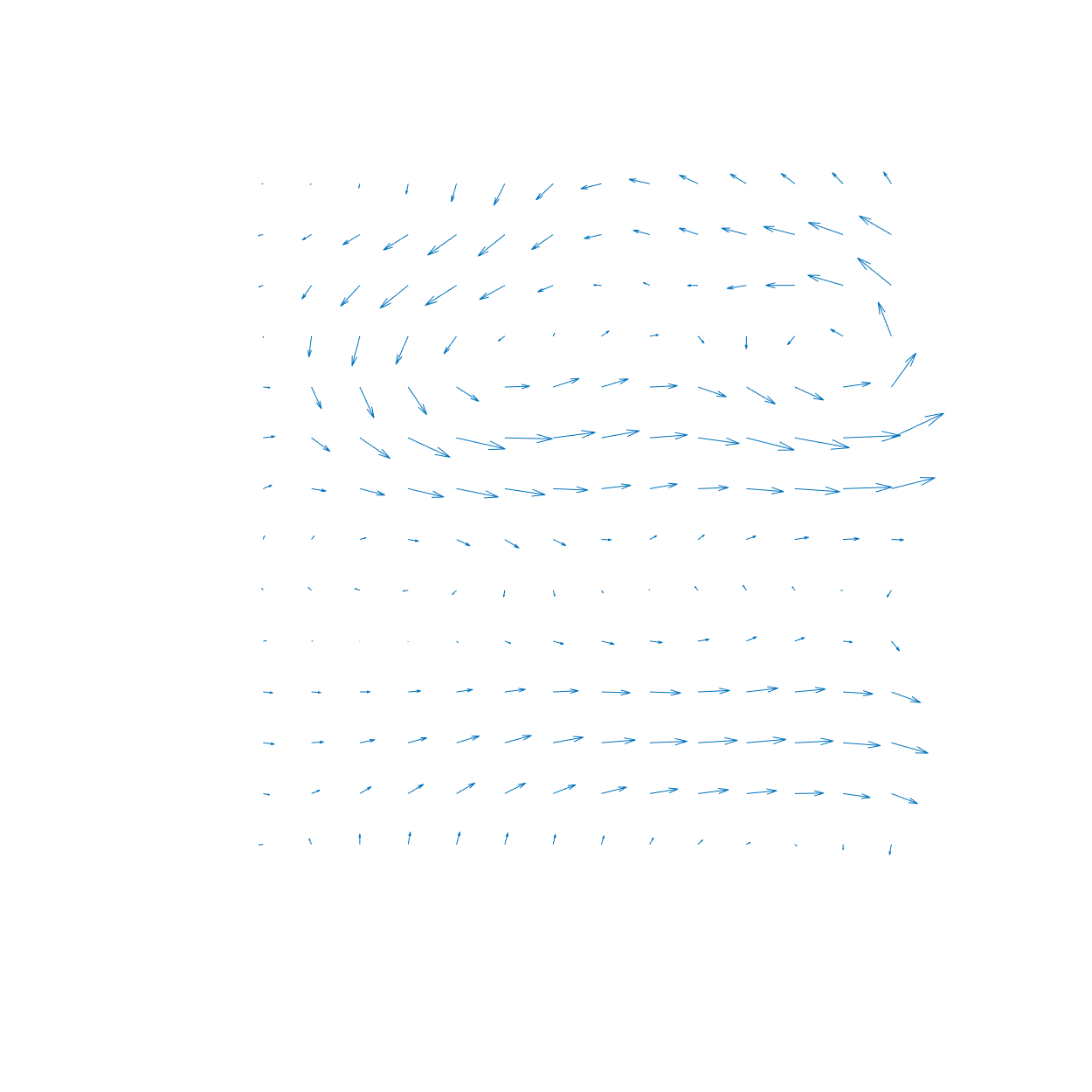

Supplement: S3 MCG raw data 3 — The raw MCG dataset includes category 4 for training and validation. (ZIP) [file pone.0338189.s003.zip › train/4/p10_430_1.png]

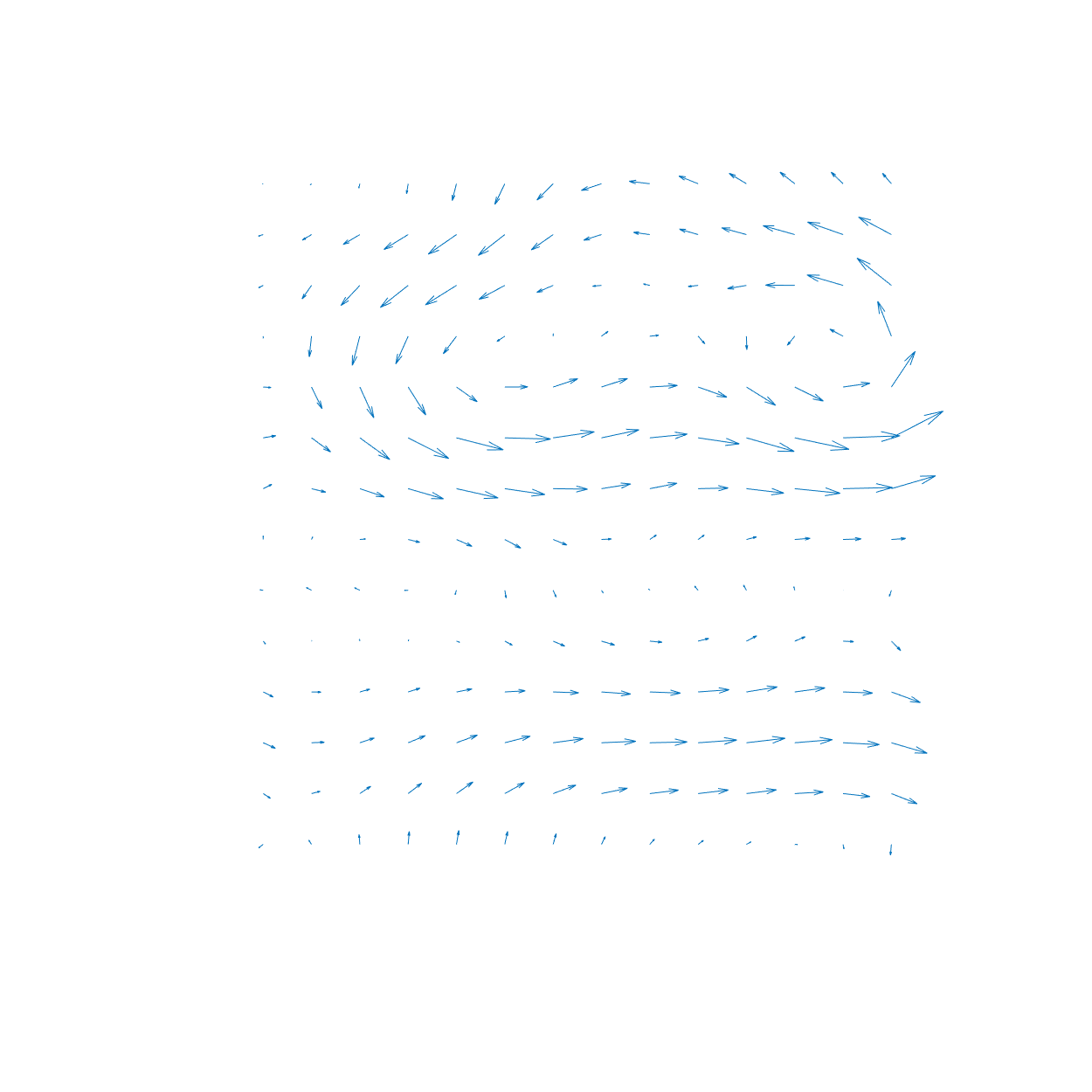

Supplement: S3 MCG raw data 3 — The raw MCG dataset includes category 4 for training and validation. (ZIP) [file pone.0338189.s003.zip › train/4/p10_430_2.png]

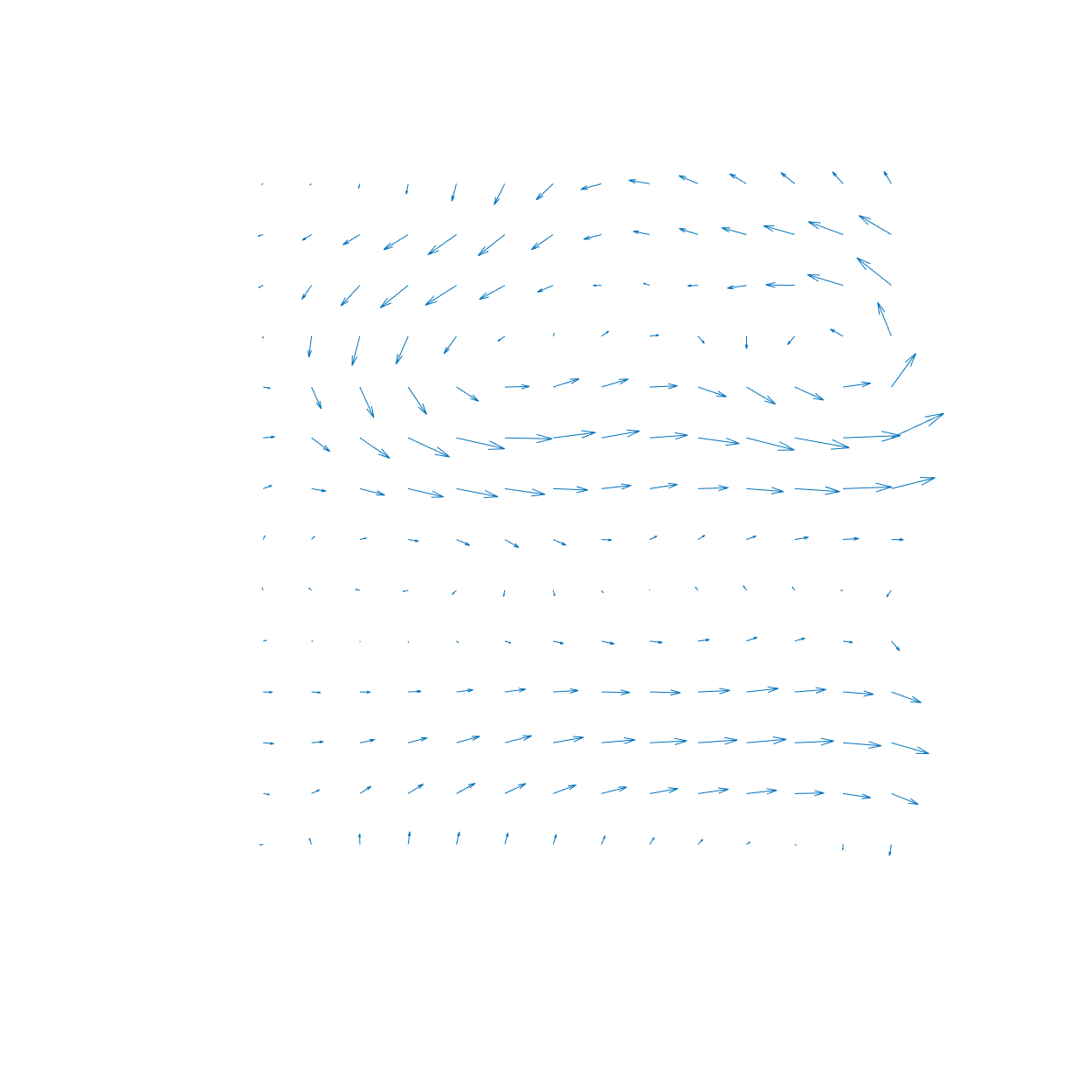

Supplement: S3 MCG raw data 3 — The raw MCG dataset includes category 4 for training and validation. (ZIP) [file pone.0338189.s003.zip › train/4/p10_430_3.png]

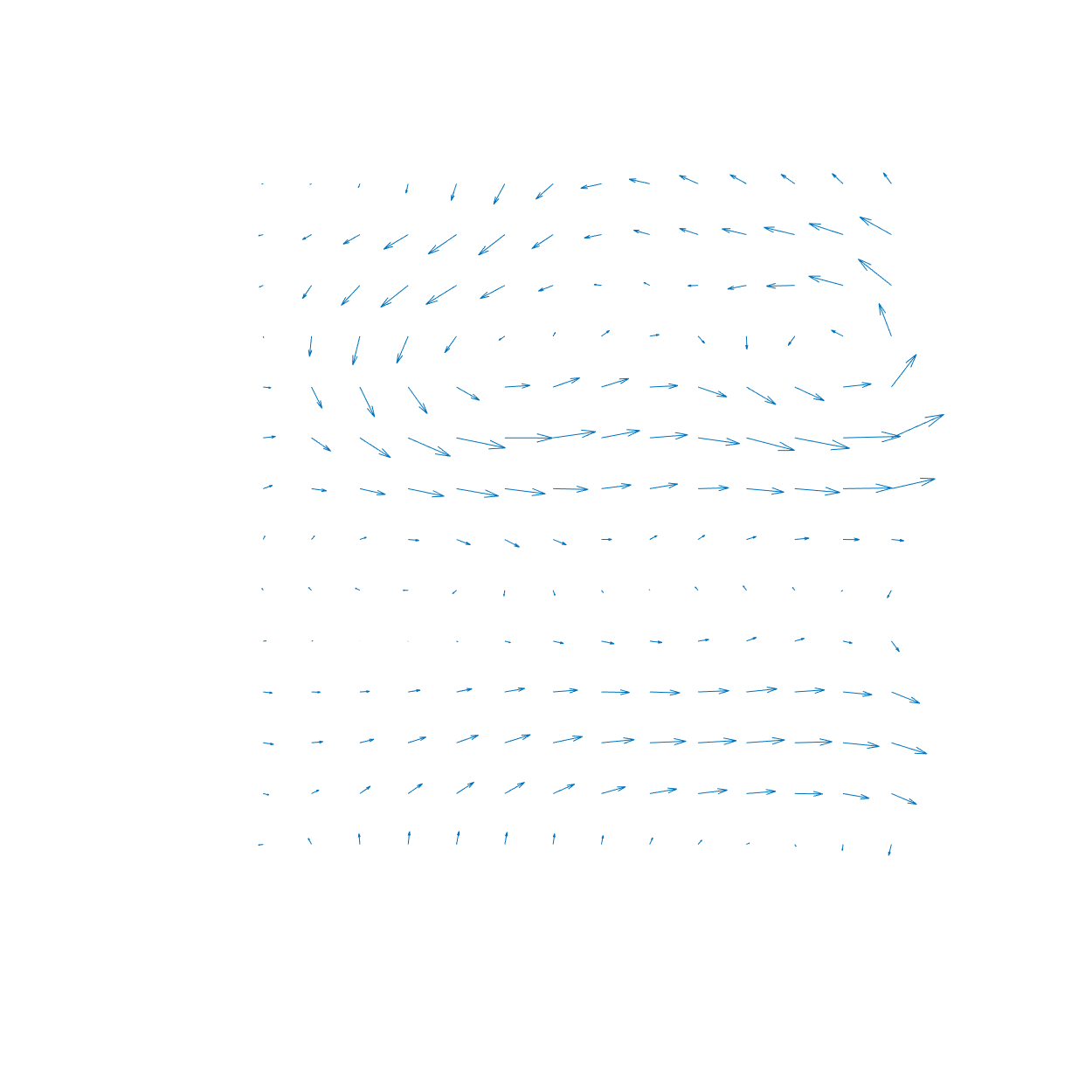

Supplement: S3 MCG raw data 3 — The raw MCG dataset includes category 4 for training and validation. (ZIP) [file pone.0338189.s003.zip › train/4/p10_430_4.png]

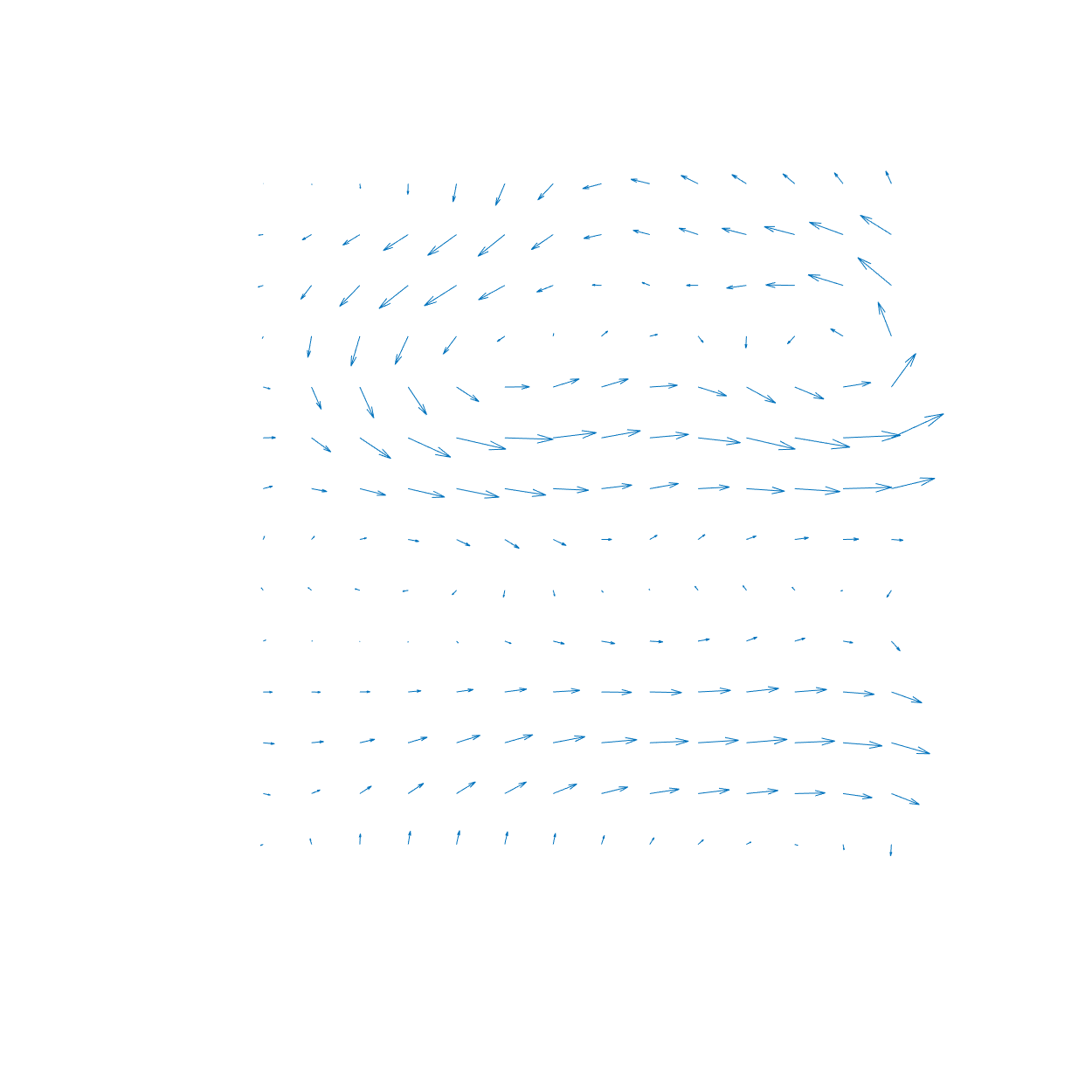

Supplement: S3 MCG raw data 3 — The raw MCG dataset includes category 4 for training and validation. (ZIP) [file pone.0338189.s003.zip › train/4/p10_435_1.png]

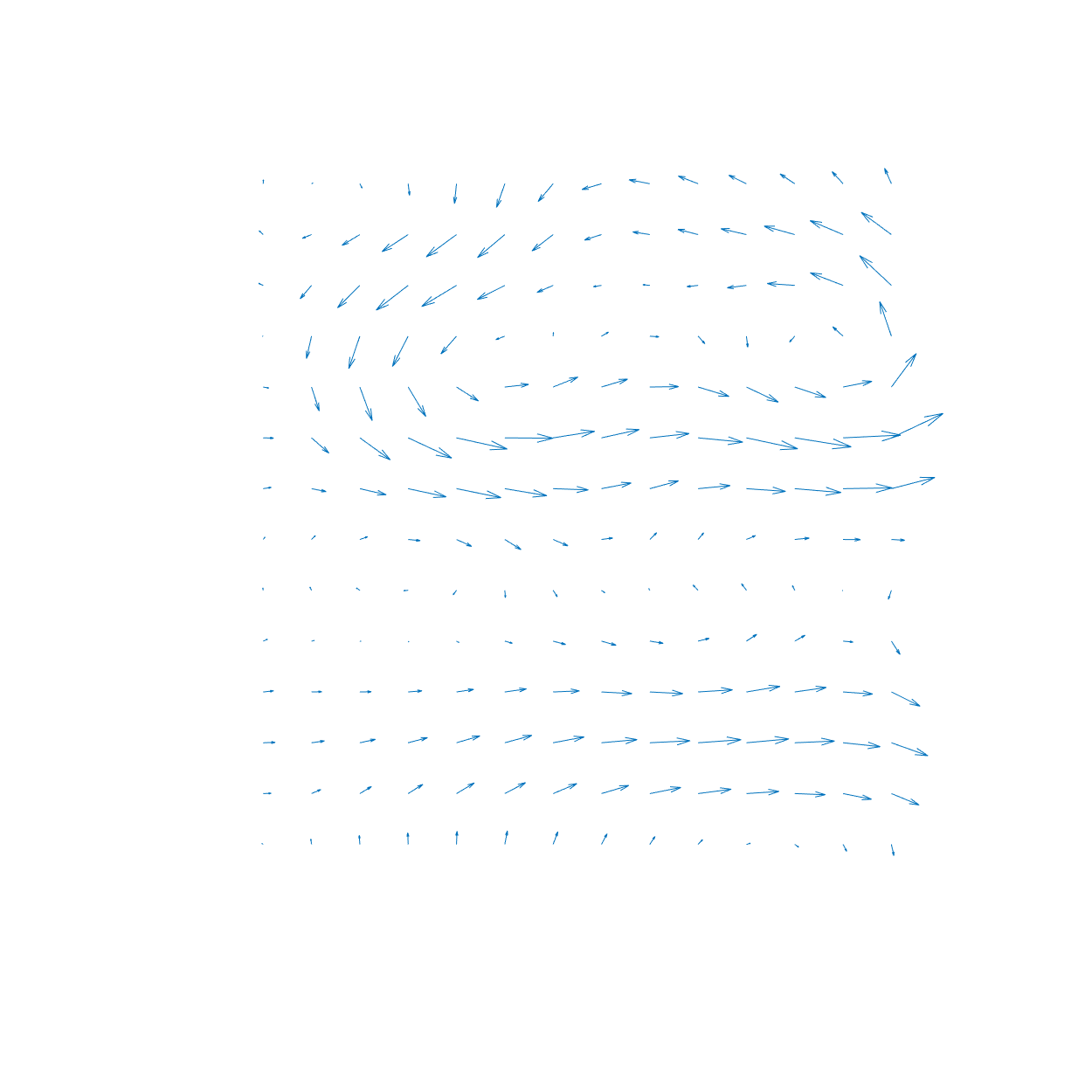

Supplement: S3 MCG raw data 3 — The raw MCG dataset includes category 4 for training and validation. (ZIP) [file pone.0338189.s003.zip › train/4/p10_435_2.png]

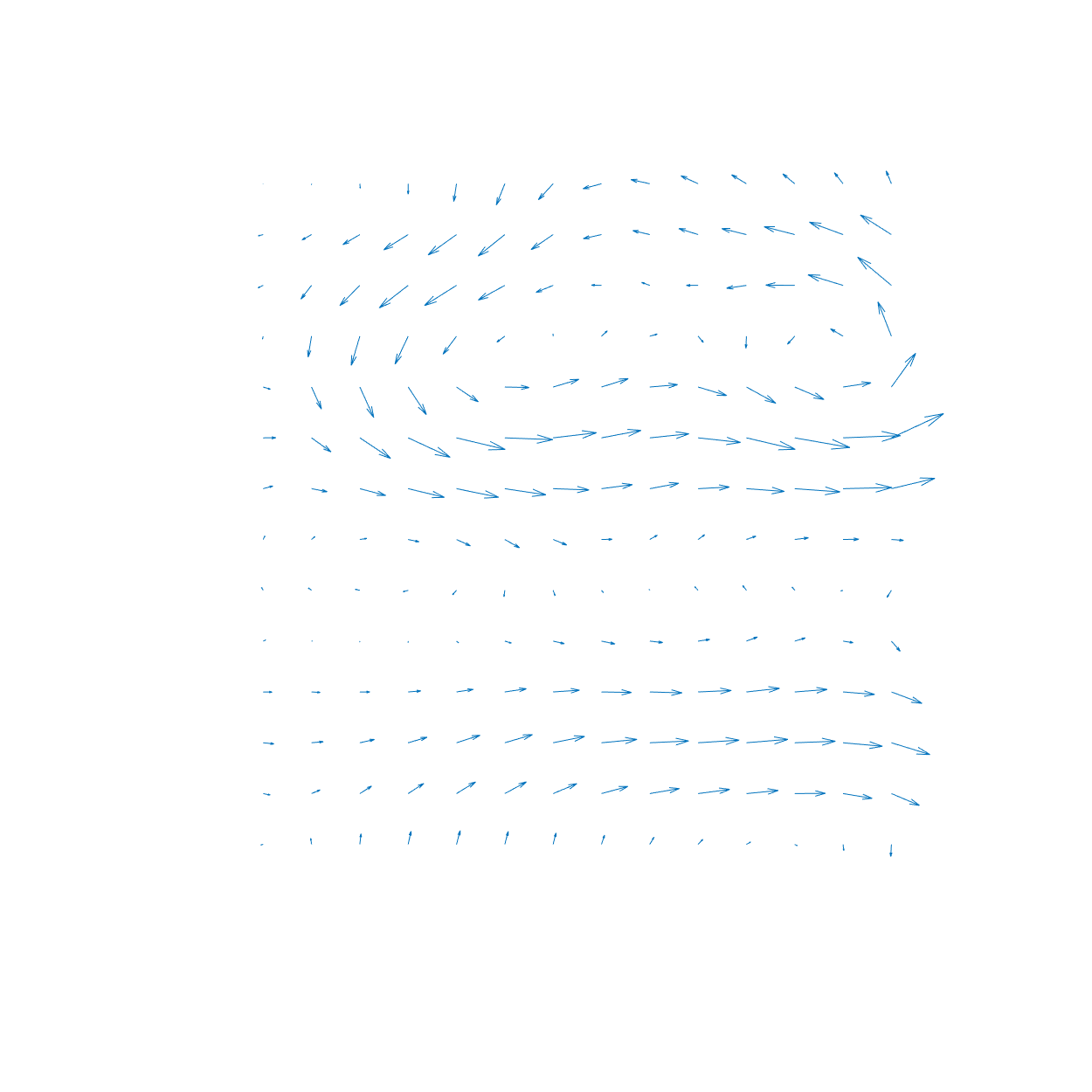

Supplement: S3 MCG raw data 3 — The raw MCG dataset includes category 4 for training and validation. (ZIP) [file pone.0338189.s003.zip › train/4/p10_435_3.png]

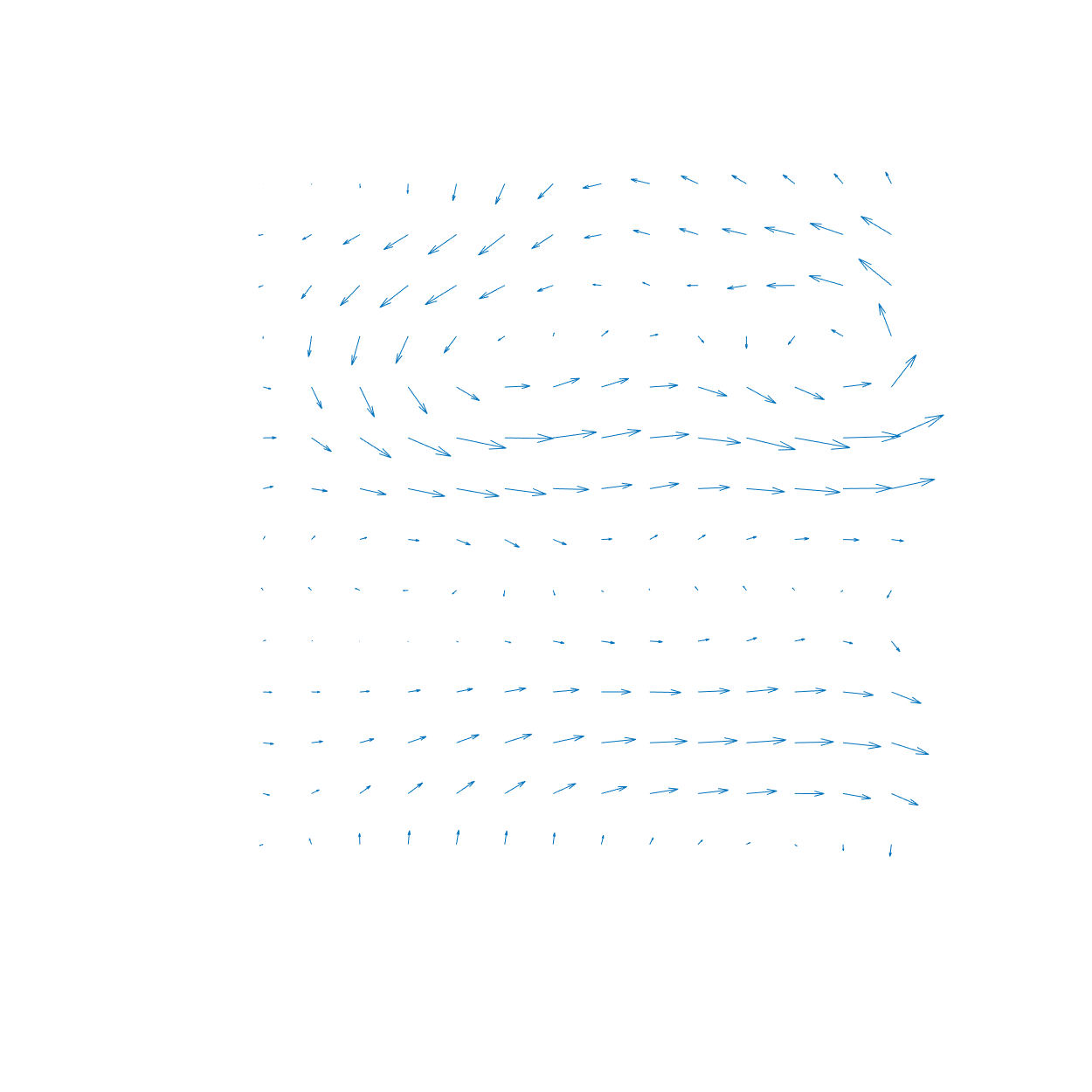

Supplement: S3 MCG raw data 3 — The raw MCG dataset includes category 4 for training and validation. (ZIP) [file pone.0338189.s003.zip › train/4/p10_435_4.png]

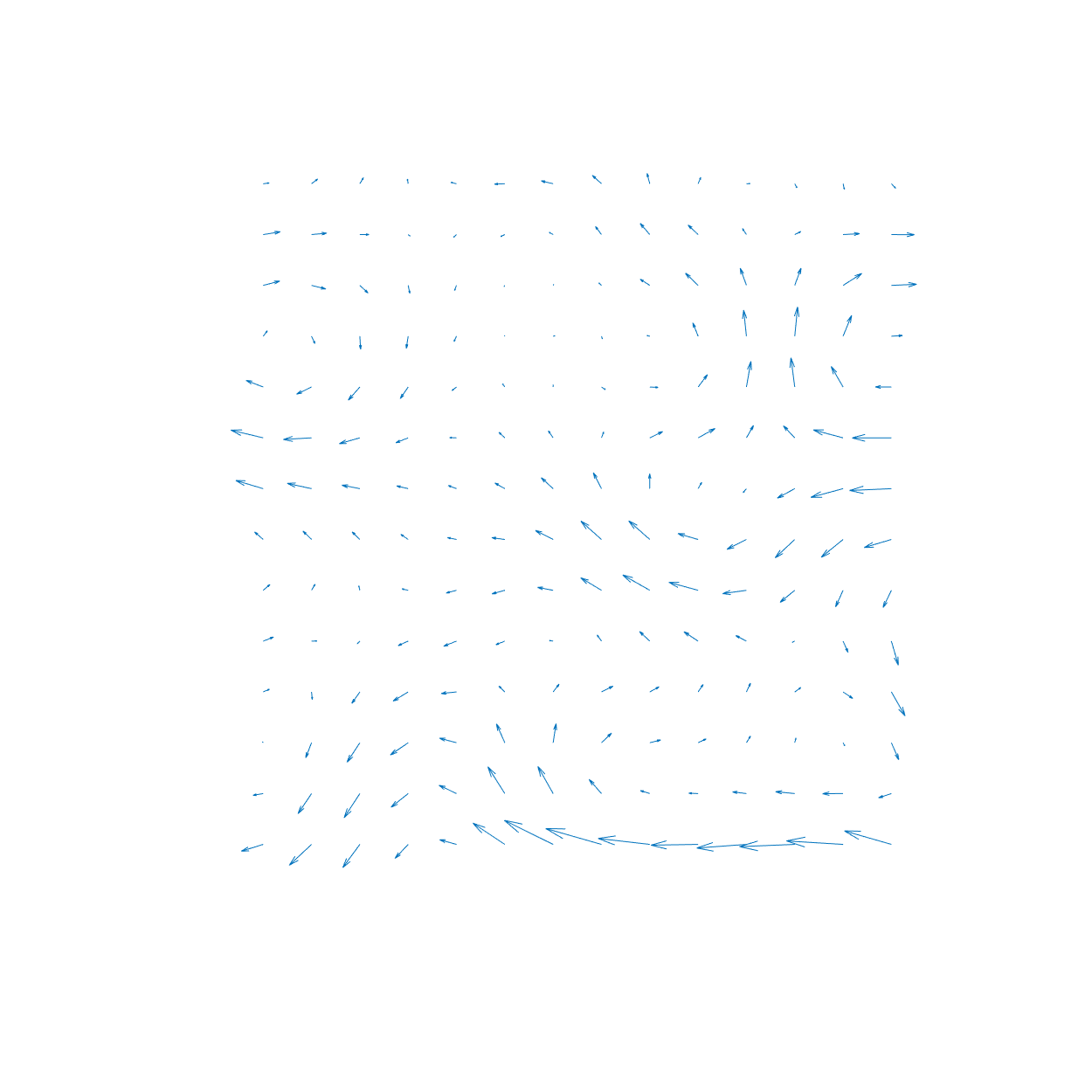

Supplement: S3 MCG raw data 3 — The raw MCG dataset includes category 4 for training and validation. (ZIP) [file pone.0338189.s003.zip › train/4/p10_535_1.png]

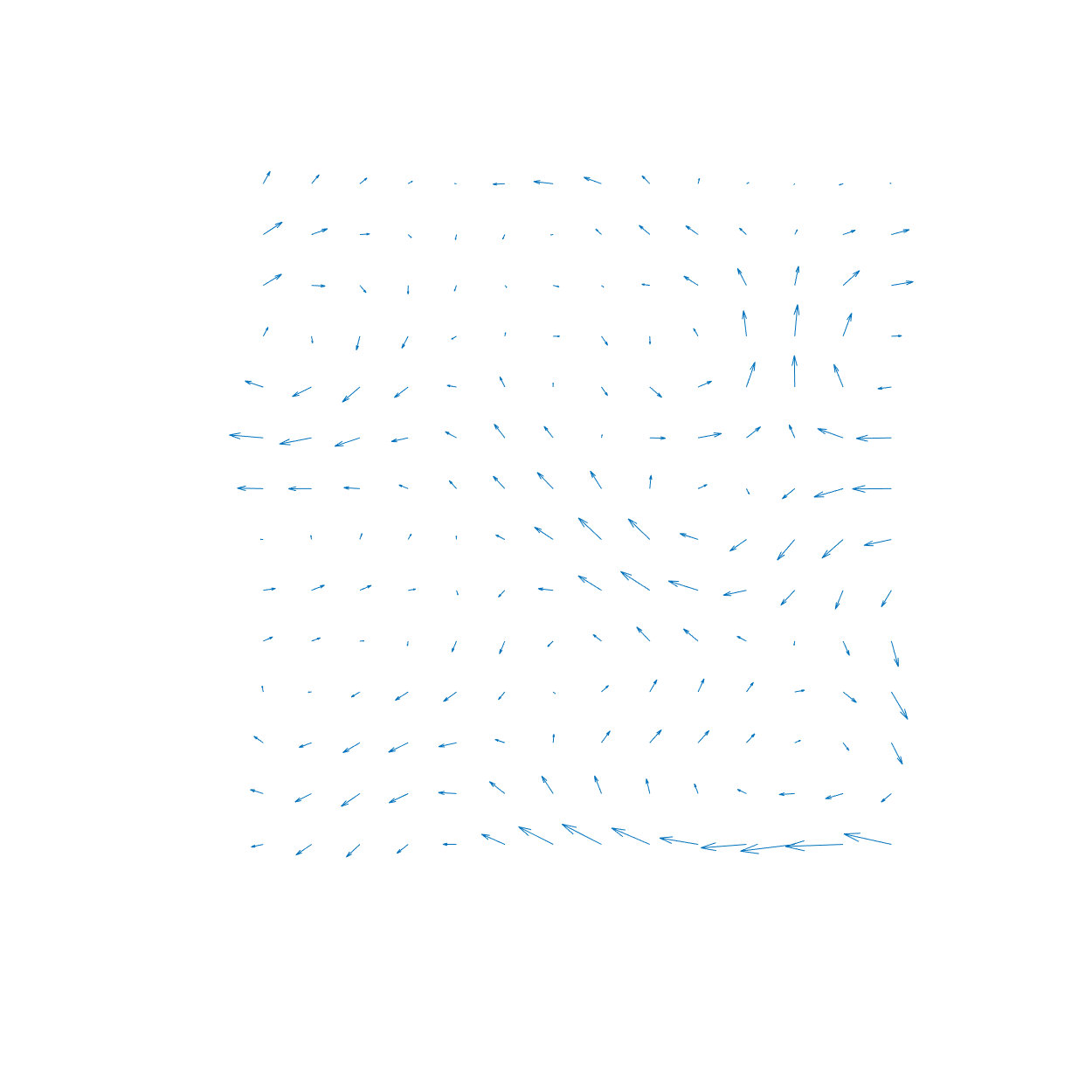

Supplement: S3 MCG raw data 3 — The raw MCG dataset includes category 4 for training and validation. (ZIP) [file pone.0338189.s003.zip › train/4/p10_535_2.png]

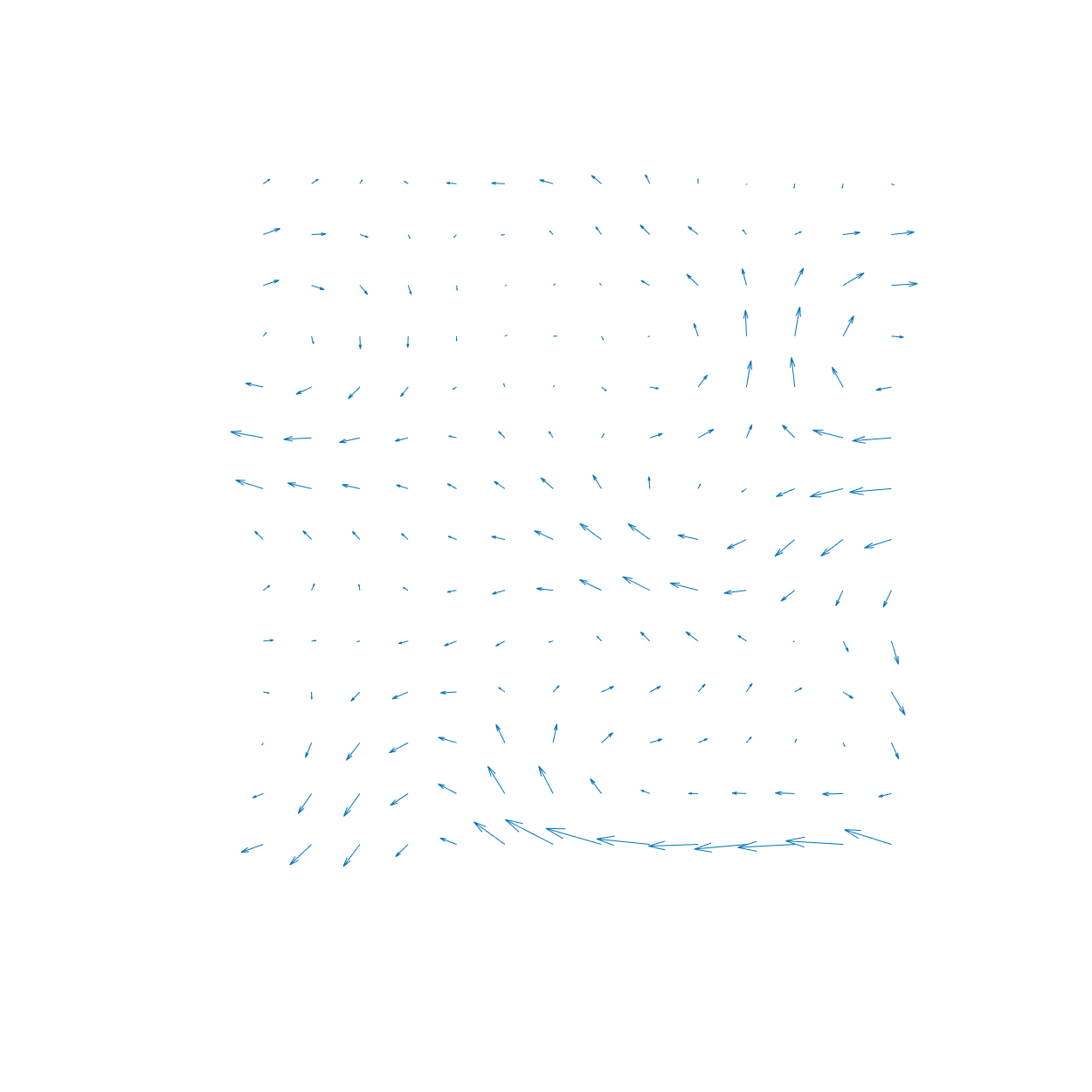

Supplement: S3 MCG raw data 3 — The raw MCG dataset includes category 4 for training and validation. (ZIP) [file pone.0338189.s003.zip › train/4/p10_535_3.png]

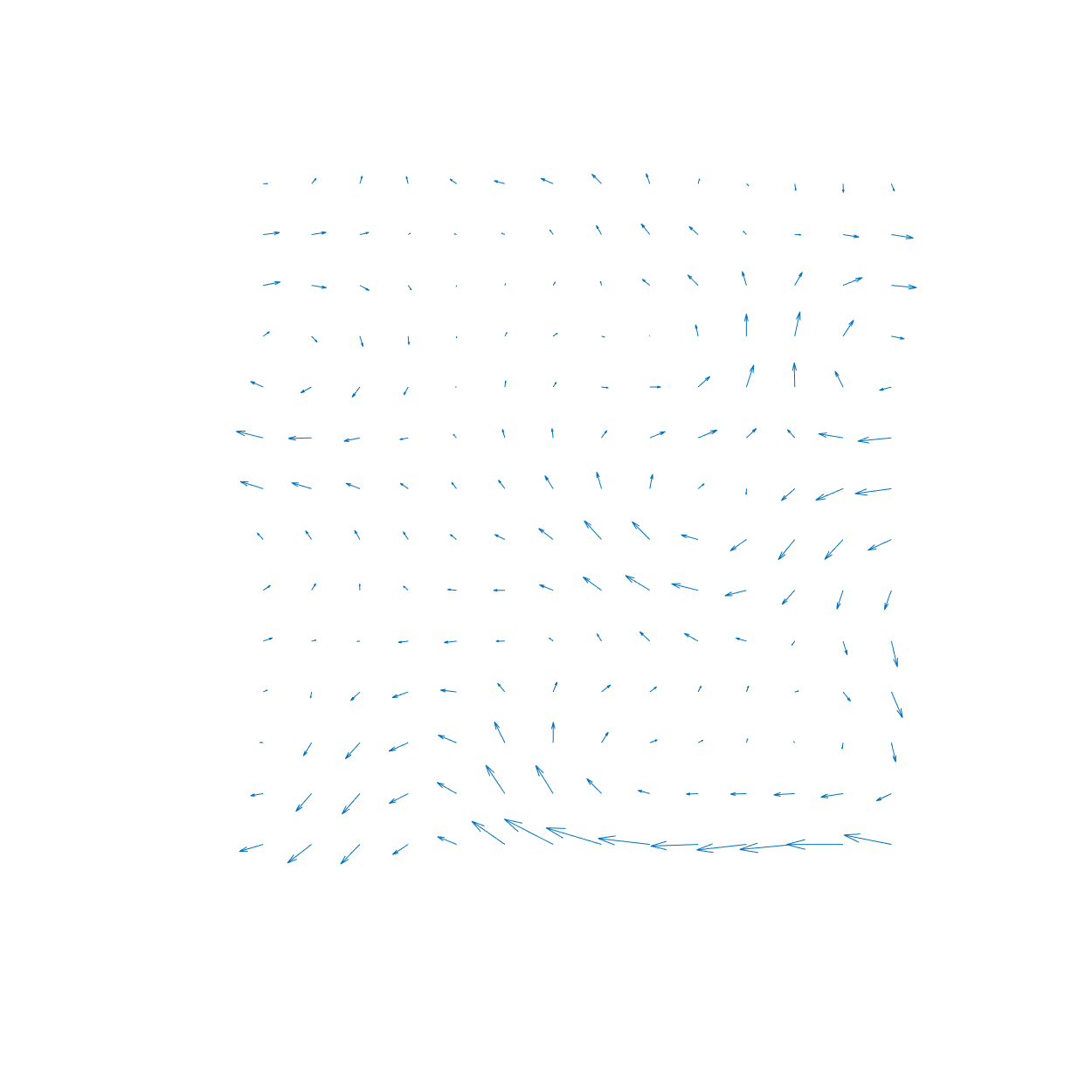

Supplement: S3 MCG raw data 3 — The raw MCG dataset includes category 4 for training and validation. (ZIP) [file pone.0338189.s003.zip › train/4/p10_535_4.png]

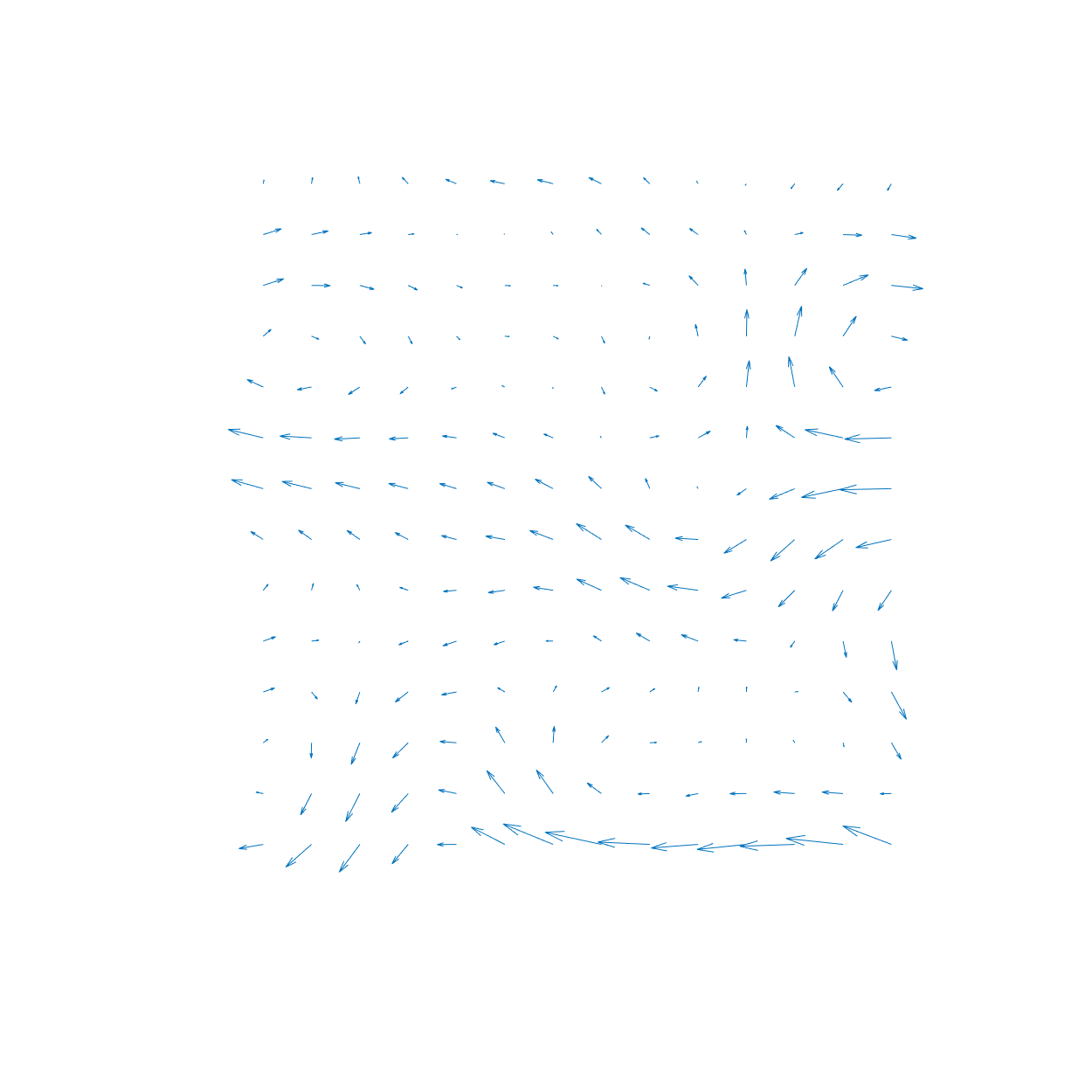

Supplement: S3 MCG raw data 3 — The raw MCG dataset includes category 4 for training and validation. (ZIP) [file pone.0338189.s003.zip › train/4/p10_540_1.png]

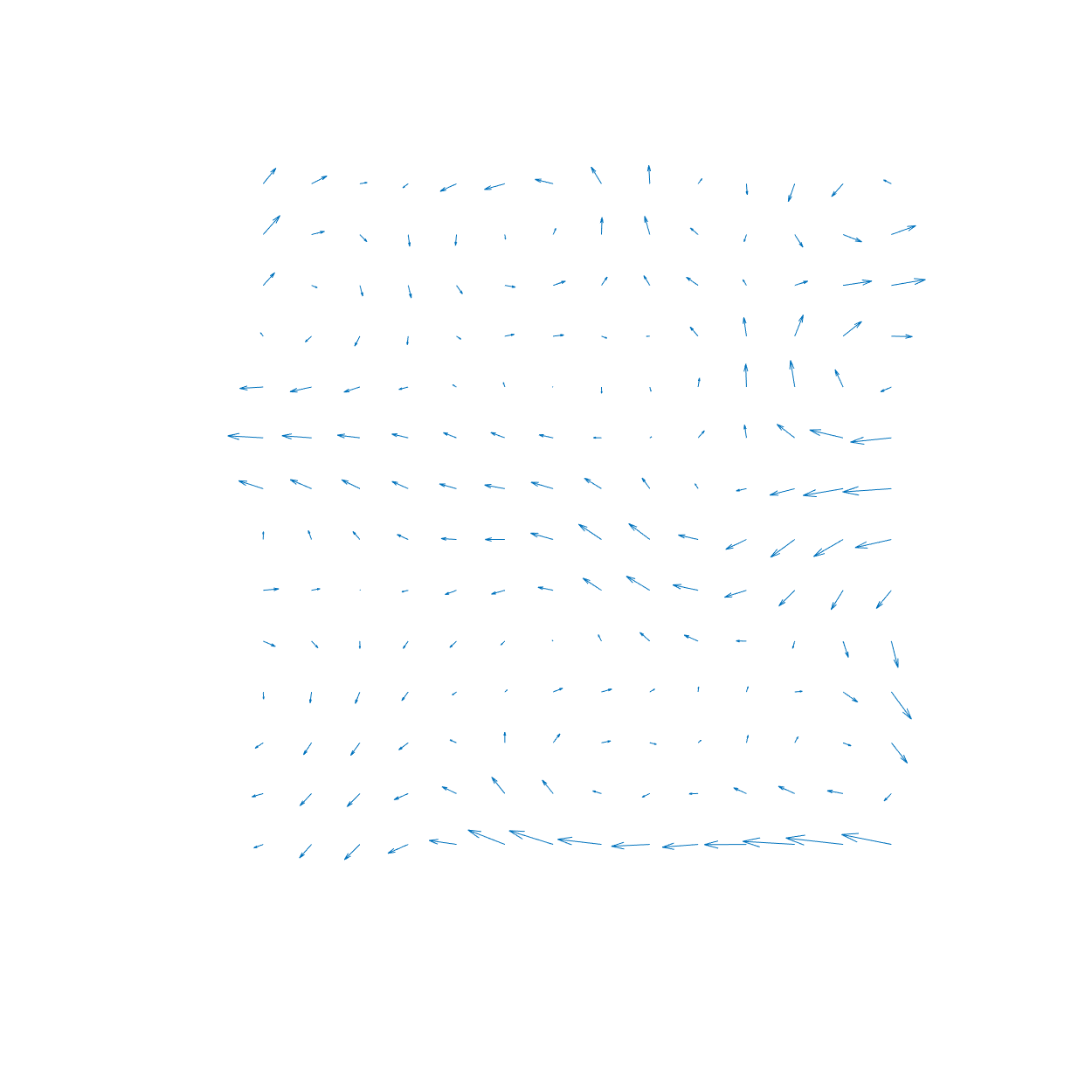

Supplement: S3 MCG raw data 3 — The raw MCG dataset includes category 4 for training and validation. (ZIP) [file pone.0338189.s003.zip › train/4/p10_540_2.png]

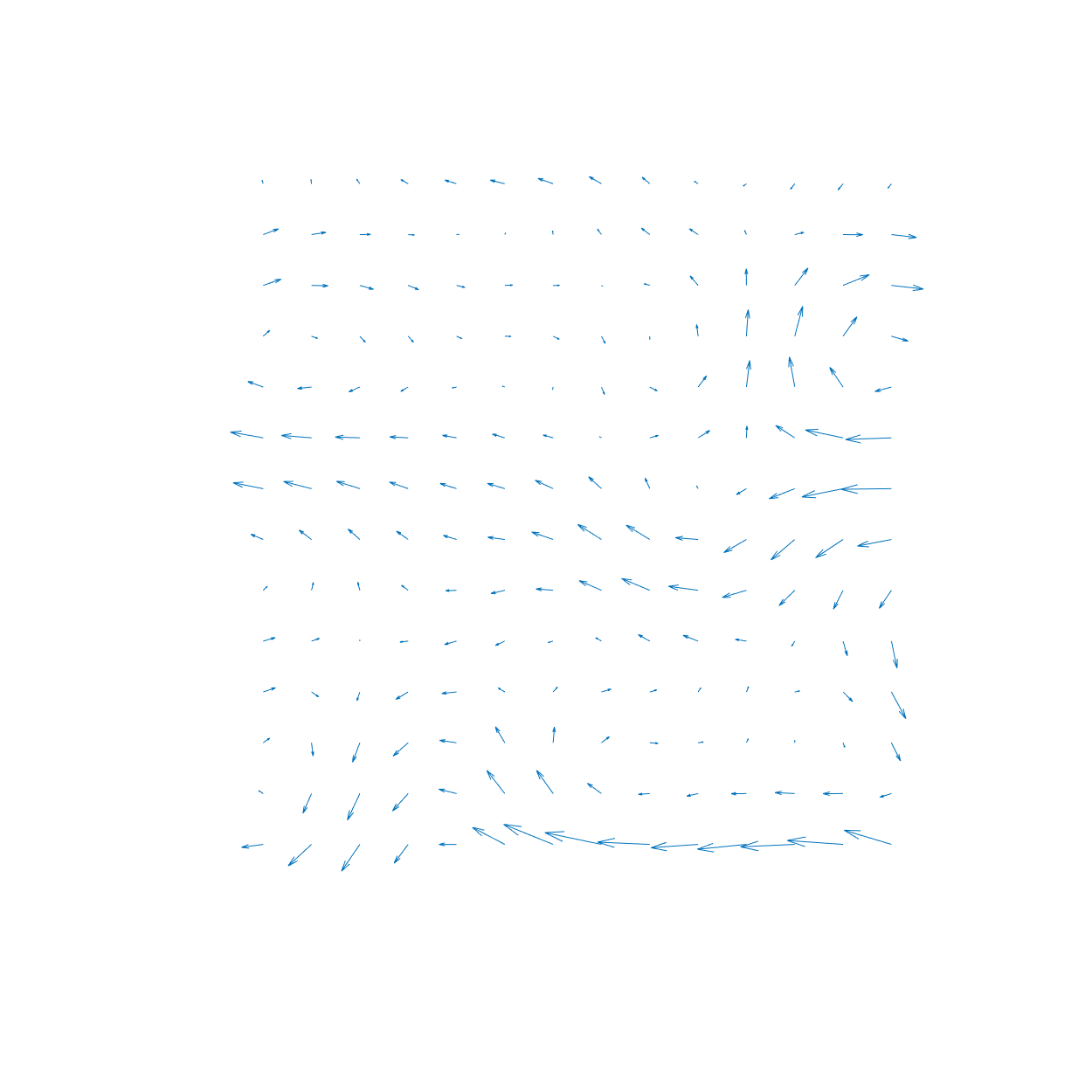

Supplement: S3 MCG raw data 3 — The raw MCG dataset includes category 4 for training and validation. (ZIP) [file pone.0338189.s003.zip › train/4/p10_540_3.png]

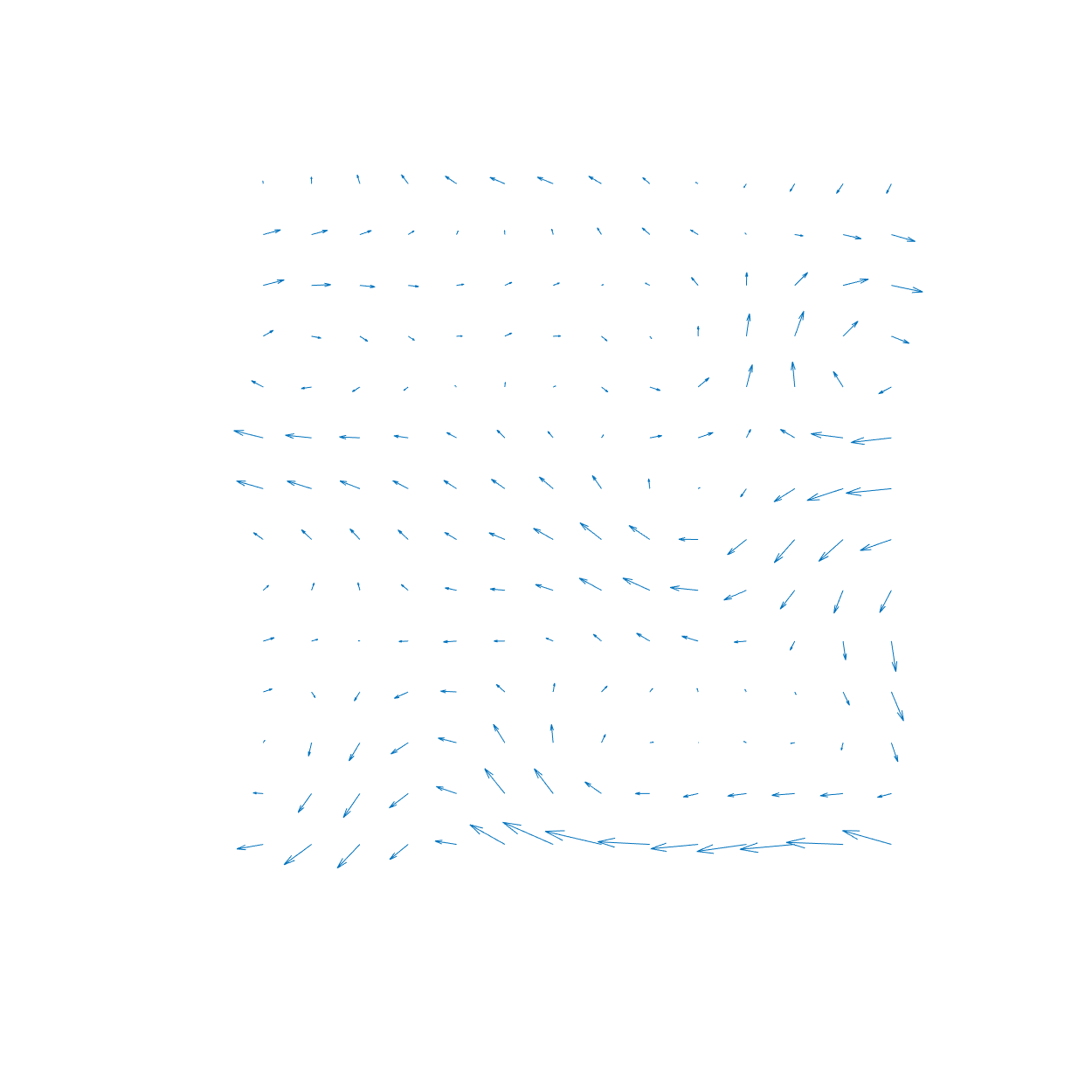

Supplement: S3 MCG raw data 3 — The raw MCG dataset includes category 4 for training and validation. (ZIP) [file pone.0338189.s003.zip › train/4/p10_540_4.png]

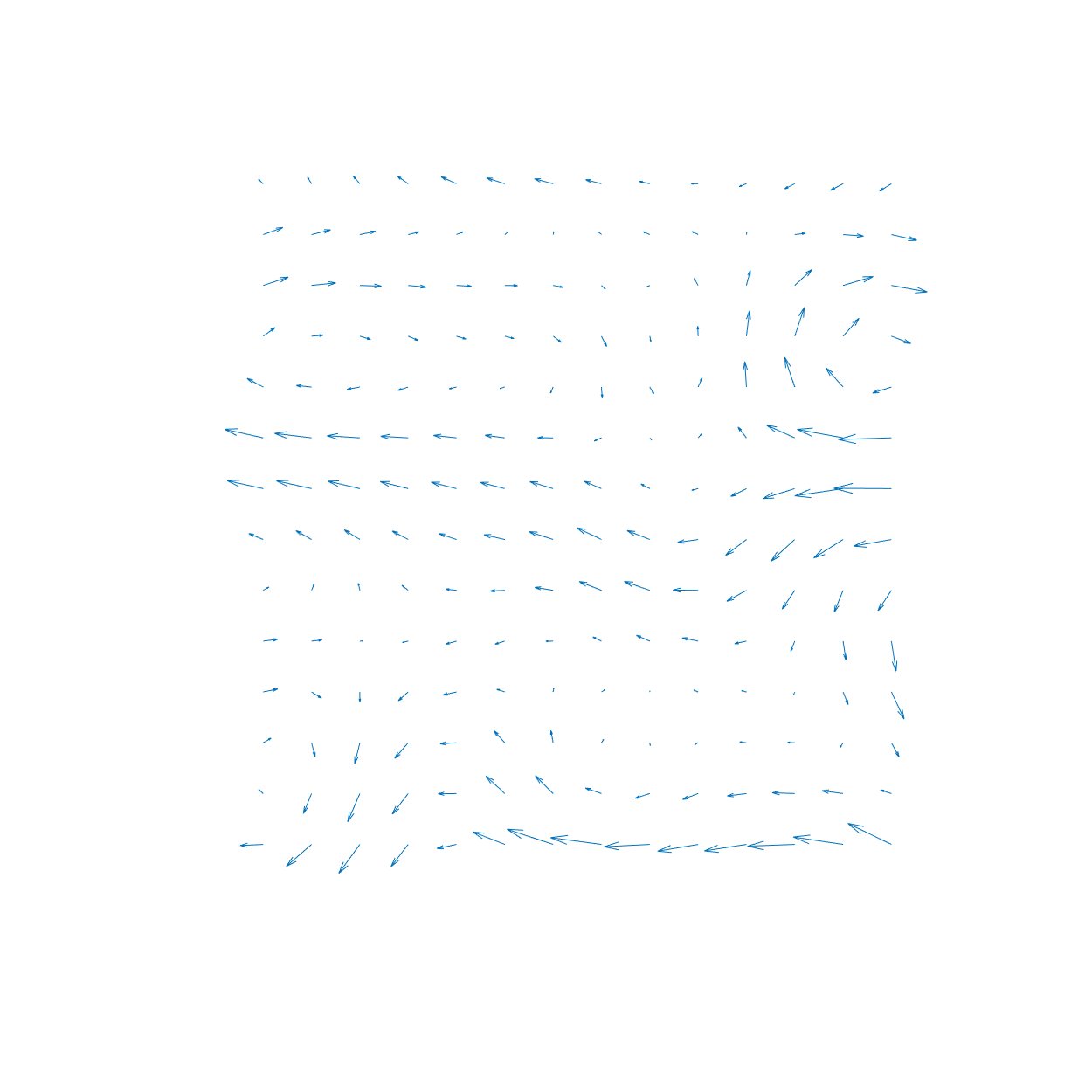

Supplement: S3 MCG raw data 3 — The raw MCG dataset includes category 4 for training and validation. (ZIP) [file pone.0338189.s003.zip › train/4/p10_545_1.png]

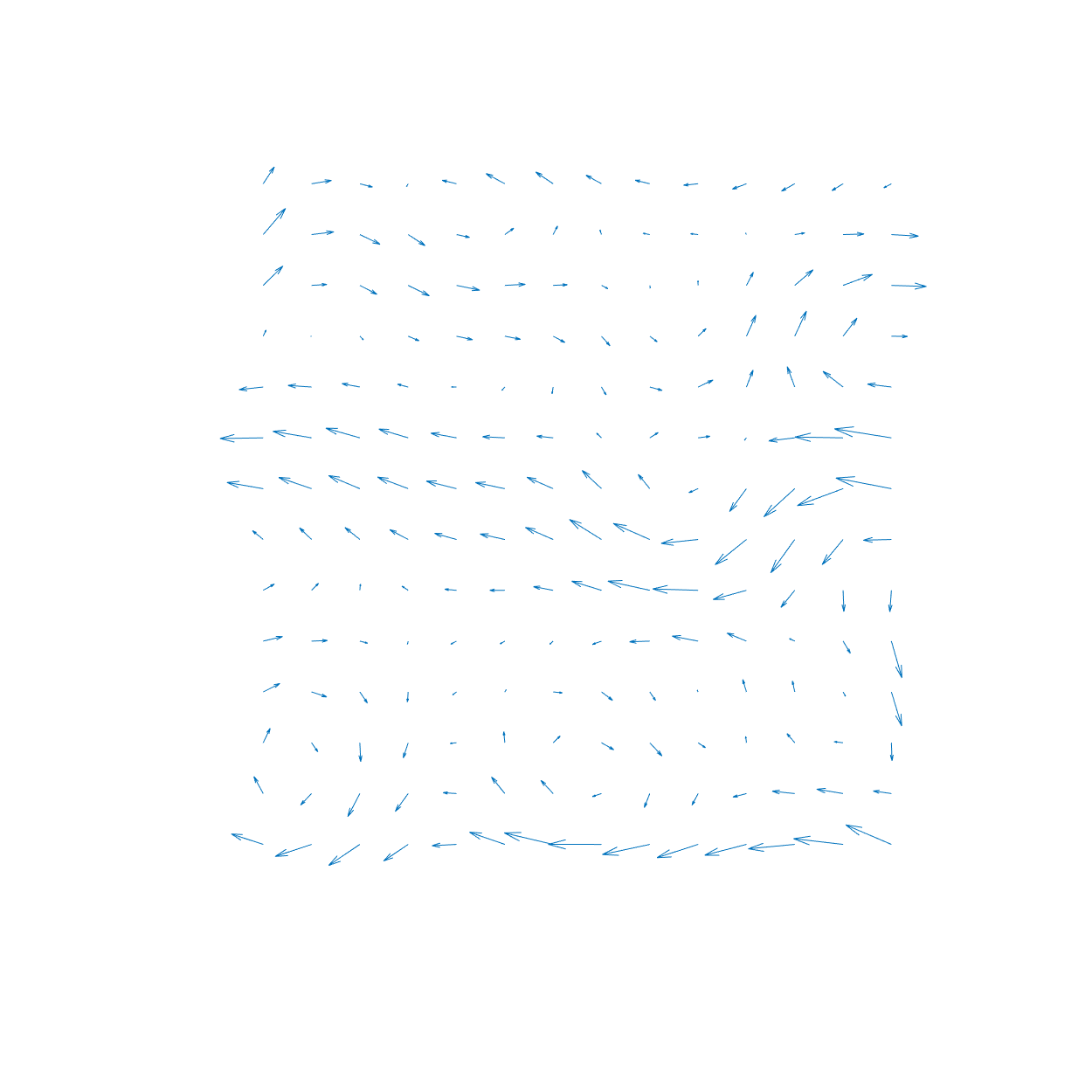

Supplement: S3 MCG raw data 3 — The raw MCG dataset includes category 4 for training and validation. (ZIP) [file pone.0338189.s003.zip › train/4/p10_545_2.png]

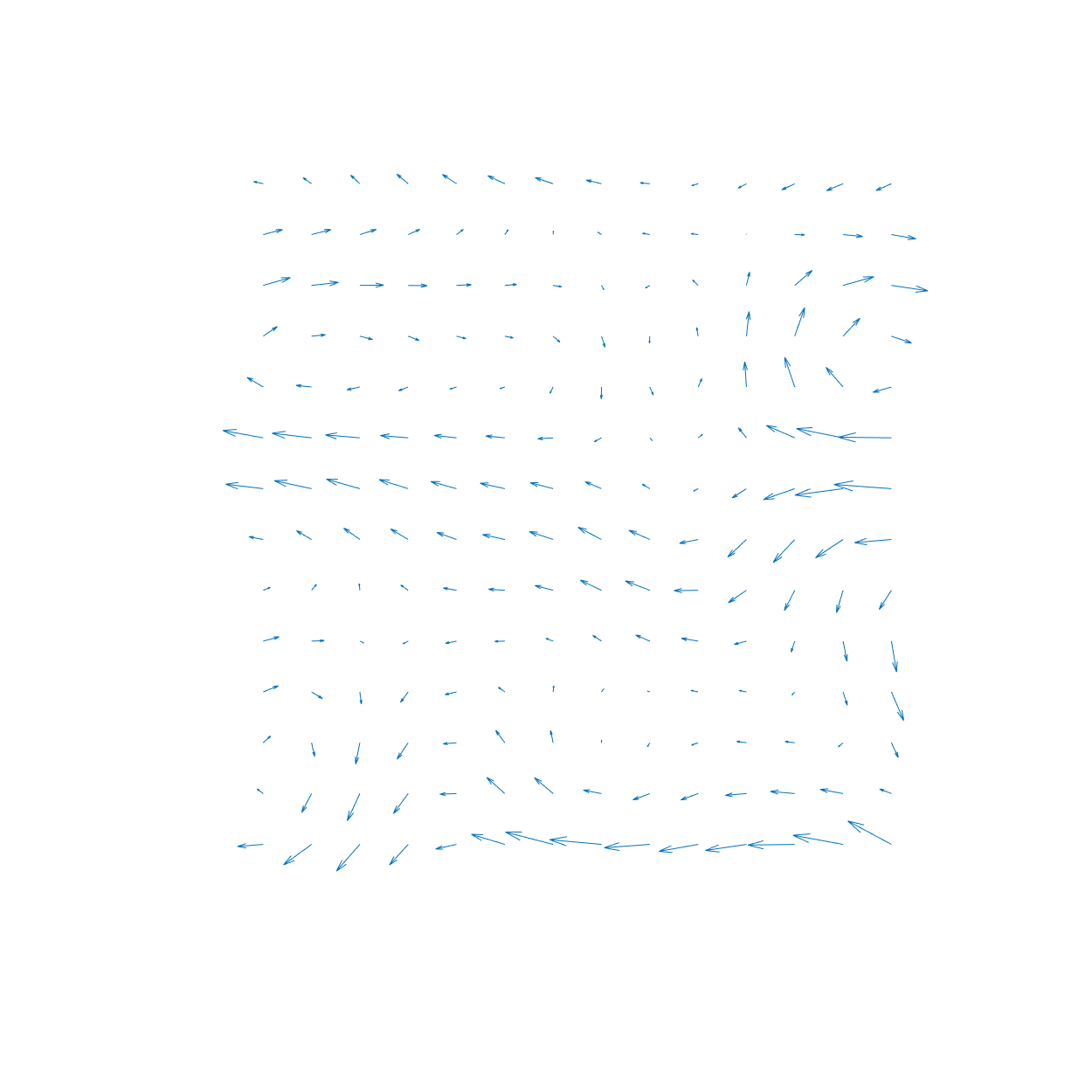

Supplement: S3 MCG raw data 3 — The raw MCG dataset includes category 4 for training and validation. (ZIP) [file pone.0338189.s003.zip › train/4/p10_545_3.png]

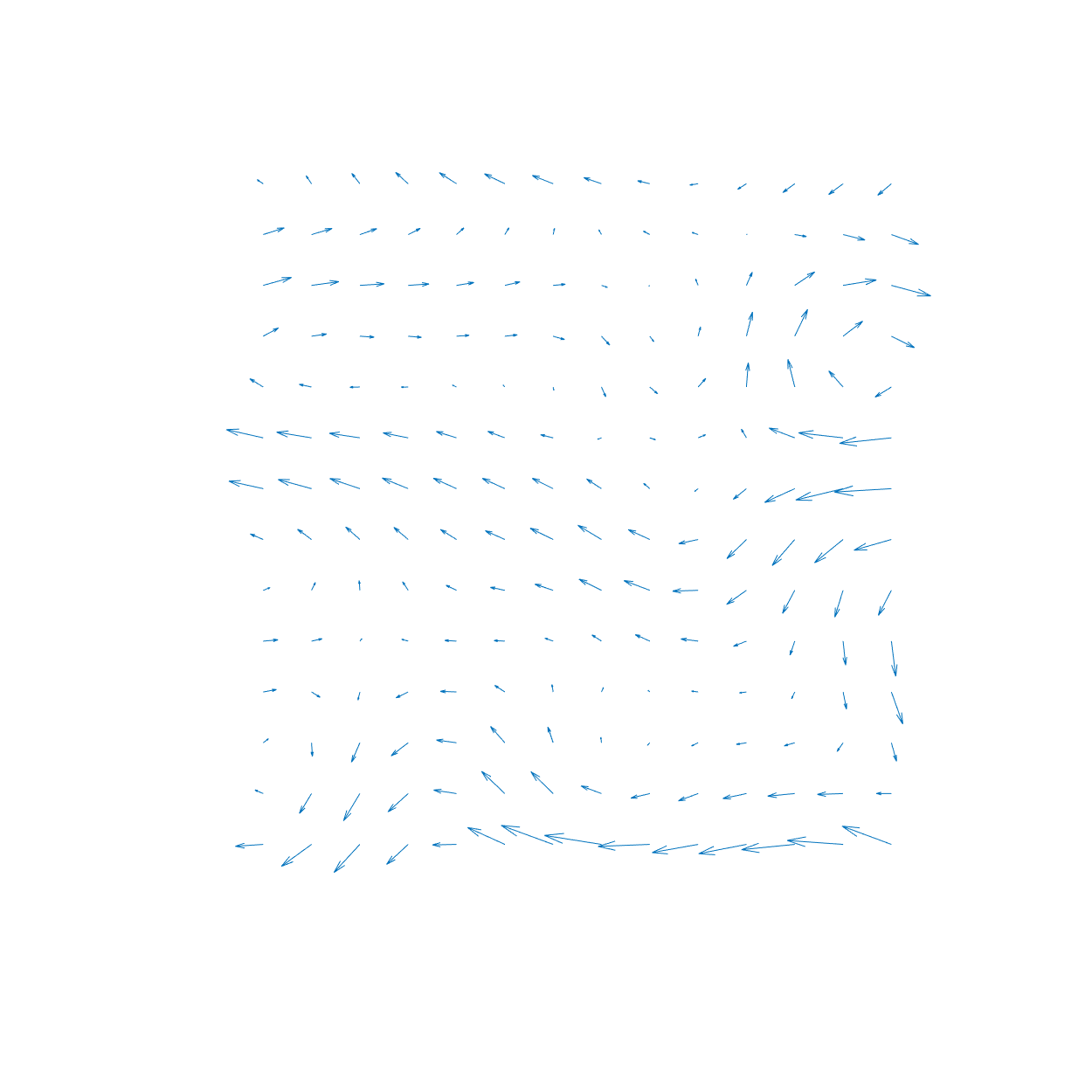

Supplement: S3 MCG raw data 3 — The raw MCG dataset includes category 4 for training and validation. (ZIP) [file pone.0338189.s003.zip › train/4/p10_545_4.png]

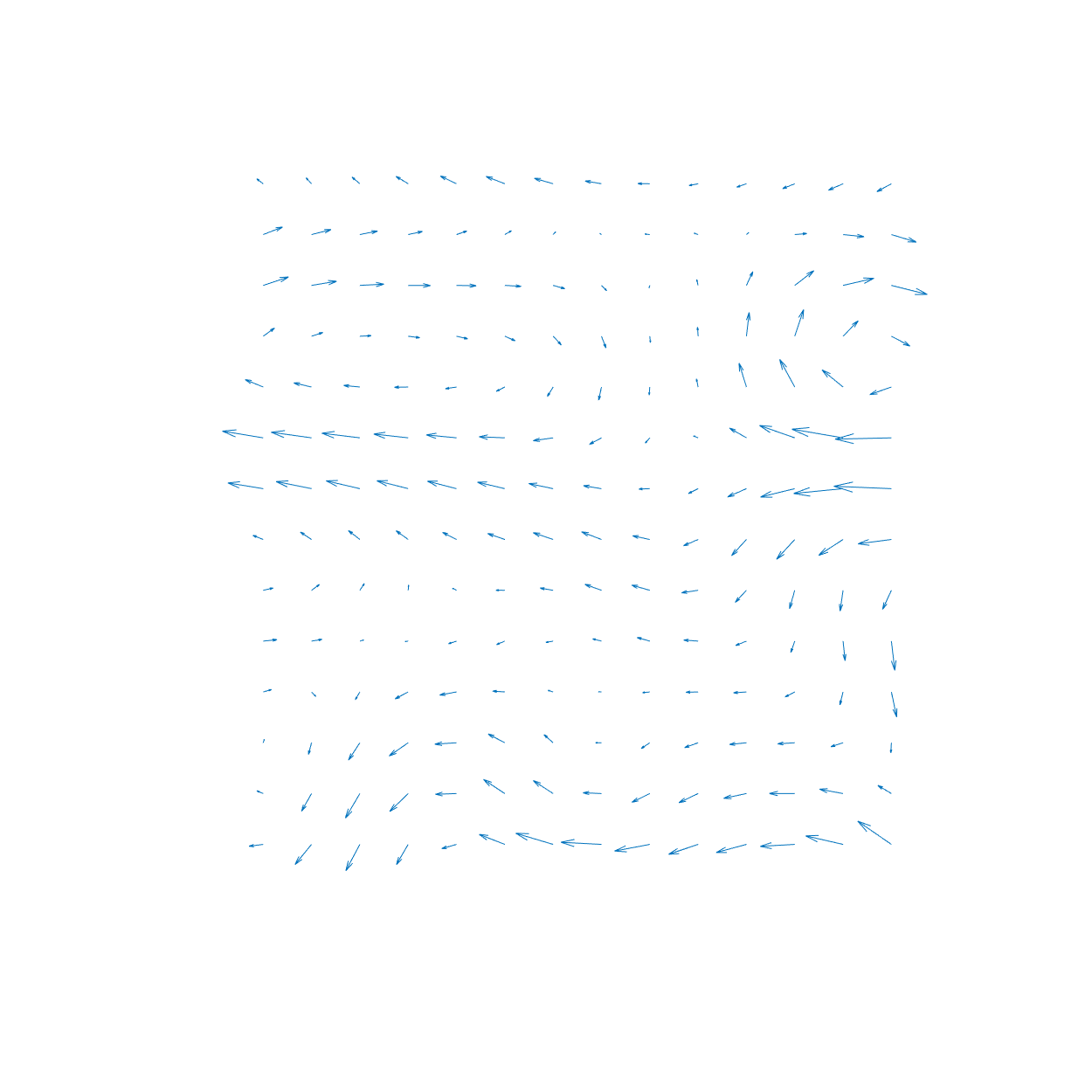

Supplement: S3 MCG raw data 3 — The raw MCG dataset includes category 4 for training and validation. (ZIP) [file pone.0338189.s003.zip › train/4/p10_550_1.png]

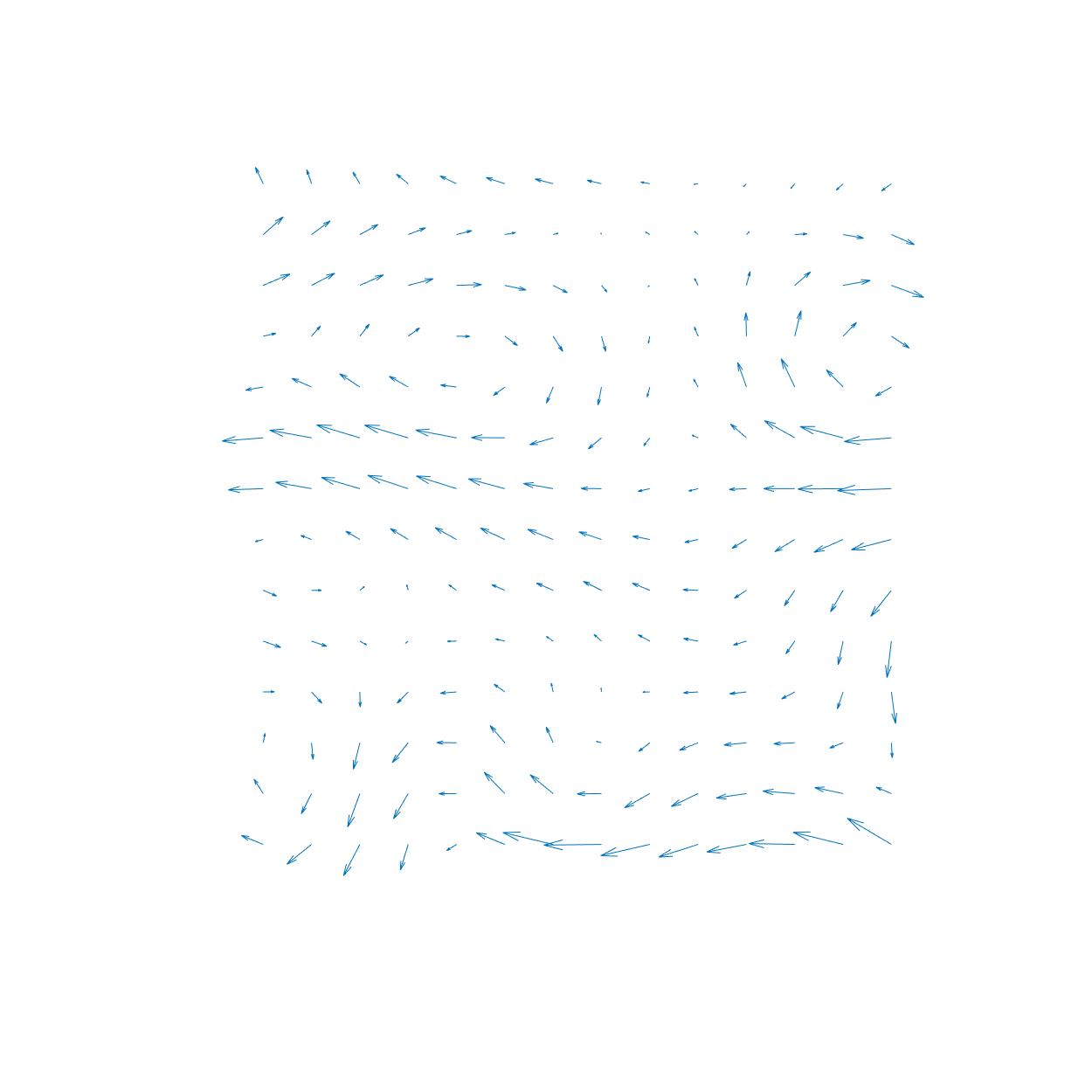

Supplement: S3 MCG raw data 3 — The raw MCG dataset includes category 4 for training and validation. (ZIP) [file pone.0338189.s003.zip › train/4/p10_550_2.png]
