# Supplementary material for: Classification of current density vector map using transformer hybrid residual network
Source: PLoS One. 2025 Dec 16;20(12):e0338189. doi: 10.1371/journal.pone.0338189 (PMC12707687; doi:10.1371/journal.pone.0338189)
Supplement: Attachment — Submitted filename: Response to Reviewers.docx [file pone.0338189.s004.docx]

**Responses**

**Dear Reviewers:**

We would like to thank 2 anonymous reviewers for their insightful suggestions and valuable comments, which helped to improve the manuscript. We have revised our paper, and the related changes to the manuscript are highlight with red text color. And we have responded to the Reviewers' comments point by point as follows:

## Response to the Reviewer 1

**Q1: Several instances in the manuscript show multiple references cited within a single sentence without clear separation. Please ensure each reference is distinctly and correctly formatted.**

**Response:** We sincerely thank the reviewer for the careful observation regarding citation formatting. In response, we have thoroughly reviewed the manuscript and ensured that multiple references cited within a single sentence are clearly separated and correctly formatted according to the journal’s style. All relevant changes have been implemented throughout the text.

**Q2: Avoid using keywords such as “we”, “us” and “you” in the manuscript. Academic writing should maintain a formal and objective tone throughout.**

**Response:** We appreciate the reviewer’s comment on maintaining a formal and objective tone. We have carefully revised the manuscript with the following actions:

1. Replaced first-person expressions with impersonal, objective phrasing.
2. Employed passive voice or process-oriented wording in Methods and Results where appropriate to reduce subjectivity.
3. Removed second-person forms and replaced them with neutral nominal phrases or structured statements when necessary.

These changes have been implemented consistently across the manuscript, in line with the journal’s style requirements.

**Q3:** **The following reference appears to be incomplete or unverifiable:“31. He X, Huang Y, Li S, Su S, Zheng W, Hu Z, Lin Q. Measurement of cardiac magnetic field based on atomic magnetometry. Chinese Journal of Medical Physics, 2017.”Please provide a valid citation with complete bibliographic details or evidence of its availability.**

**Response:** We thank the reviewer for pointing out the incomplete citation. The full bibliographic details and evidence of availability have now been provided, and the reference list has been updated accordingly (with in-text citations synchronized). The DOI is: 10.3969/j.issn.1005-202X.2017.11.016. The manuscript has been revised to ensure compliance with the journal’s reference style.

**Q4: The results claim 100% accuracy, which may appear unrealistic in practical scenarios. Kindly justify this outcome and explain how further research or improvement is still relevant in the context of your proposed model.**

**Response:** We thank the reviewer for the insightful comment on the classification accuracy. We fully agree that the 100% precision and specificity in certain categories require clarification, and we appreciate the opportunity to elaborate on their rationality and subsequent improvement directions.

1. **Rationality of the Result**

- The 97.52% in the manuscript refers to the overall average accuracy, while 100% only applies to the precision and specificity of Category 1 (normal pattern) and Category 2 (mild abnormality) (see Table 2), not the overall accuracy across all categories.
- Clinical features of Category 1 and 2 are significantly distinguishable from other categories (regular current distribution patterns for normal/mildly abnormal cases). Combined with ResNet’s local feature extraction and Transformer’s global dependency modeling (Section 2.4), the model can accurately capture these clear classification boundaries.

2. **Future Research and Improvements**

- Optimize class balance: Addressing the current imbalance (Category 4 with the highest proportion: 2235 samples; Category 0 with the lowest: 290 samples), we will adopt weighted loss functions and stratified oversampling strategies to balance the model’s learning weight for each category.
- Expand dataset scale: We plan to collect more MCG data, which will cover more complex pathological types (e.g., coronary heart disease of varying severity and arrhythmia), to reduce the selection bias caused by the small sample size.

## Response to the Reviewer 2

**Q1: The figures are well-prepared and visually clear. However, captions are missing — each figure currently includes only a title. Figures should be self-contained, with detailed captions that describe all subpanels, symbols, and relevant details to ensure clarity without referring back to the main text.**

**Response:** We sincerely thank the reviewer for this helpful comment. We have carefully revised all figures and added informative captions so that each figure is self-contained.

- **Fig 1. CDVM imaging framework** Processing of raw MCG signals and imaging of the current density vector map
- **Fig 2. Cardiac magneto signal** A single-channel cardiac magneto signal waveform over one cardiac cycle, illustrating the typical morphology with P wave, QRS complex, J point, ST segment, and T wave.
- **Fig 3. Multichannel MCG data** Multichannel MCG data from 49 channels.
- **Fig 4. Generation of current density vector map** Raw 49-channel MCG signals were preprocessed (denoising and baseline correction) to plot the magnetic field map, and the current density vector map was reconstructed over the ST–T segment with a 5ms sampling interval.
- **Fig 5. Data enhancement** Example of an augmented CDVM generated by noise addition, ARIMA prediction, and interpolation.
- **Fig 6. Classification system of CDVM** Category 0–1 denote normal patterns, category 2 indicates mild abnormality, and category 3–4 signify severe abnormalities. Representative examples for each category are shown.
- **Fig 7. Network structure schematic diagram** The model consists of a ResNet-18 backbone for local feature extraction, a Transformer encoder for global context modeling, and classification heads for CDVM classification. Data preprocessing and augmentation are applied prior to training; arrows indicate the forward flow.
- **Fig 8. Network architectures performance comparison** Performance of CNN, MLP, and ResNet18. CNN/MLP val 51.95% (CNN train 51.53%); ResNet18 train 82.82%, val 83.41%.; ResNet18 performs notably better.
- **Fig 9. Hyperparameter optimization results** Bayesian optimization explored 30 configurations across learning rate, weight decay, dropout, model dimension (d\_model), number of layers, and feedforward dimension, with nhead fixed at 8.
- **Fig 10. ResNet18-LSTM vs ResNet18-Transformer Encoder** The proposed model peaks at 94.81% validation accuracy at epoch 26, outperforming the ResNet-LSTM network (92.06%), with steadily decreasing validation loss and early stopping, indicating better temporal pattern modeling by the Transformer and no overfitting.
- **Fig 11. Transfer learning and Transformer encoder components ablation studies results** ResNet: 83.41% val. accuracy; +TL: 92.35%; +TE (no TL): 85.57%; full ResNet‑TL‑TE: 94.81% with the lowest, most stable validation loss (0.155). TL accelerates convergence via better initialization, while TE improves temporal modeling and training stability.
- **Fig 12. Confusion matrix of test set** The ResNet‑TL‑TE model attains an average accuracy of 97.52% on CDVM classification. The matrix visualizes per‑category predictions and errors, revealing near‑perfect diesscrimination for Categories 1–2 and balanced performance across all categories.
- **Table 1. Optimal hyperparameters configuration** The best validation accuracy (92.35%) was obtained with a shallow-but-wide transformer setting.
- **Table 2. Precision, Recall, and Specificity for Each Category** Categories 1–2 reach 100% precision and specificity; Category 0 has 100% recall; other Categories are high overall.
